# Supplementary figures and images for: Neuroblastoma signalling models unveil combination therapies targeting feedback-mediated resistance
Source: PLoS Comput Biol. 2021 Nov 4;17(11):e1009515. doi: 10.1371/journal.pcbi.1009515 (PMC8604339; doi:10.1371/journal.pcbi.1009515)

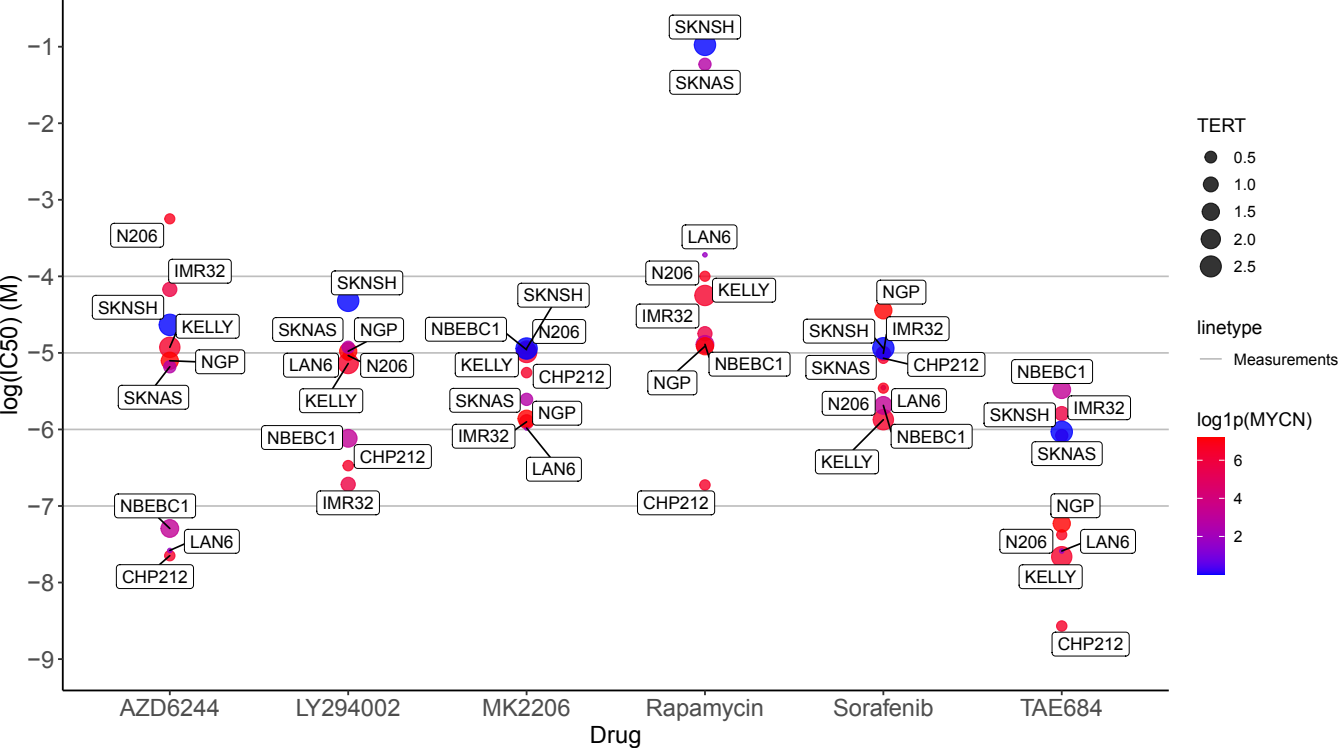

Supplement: S1 Fig — (PDF) [file pcbi.1009515.s001.pdf]

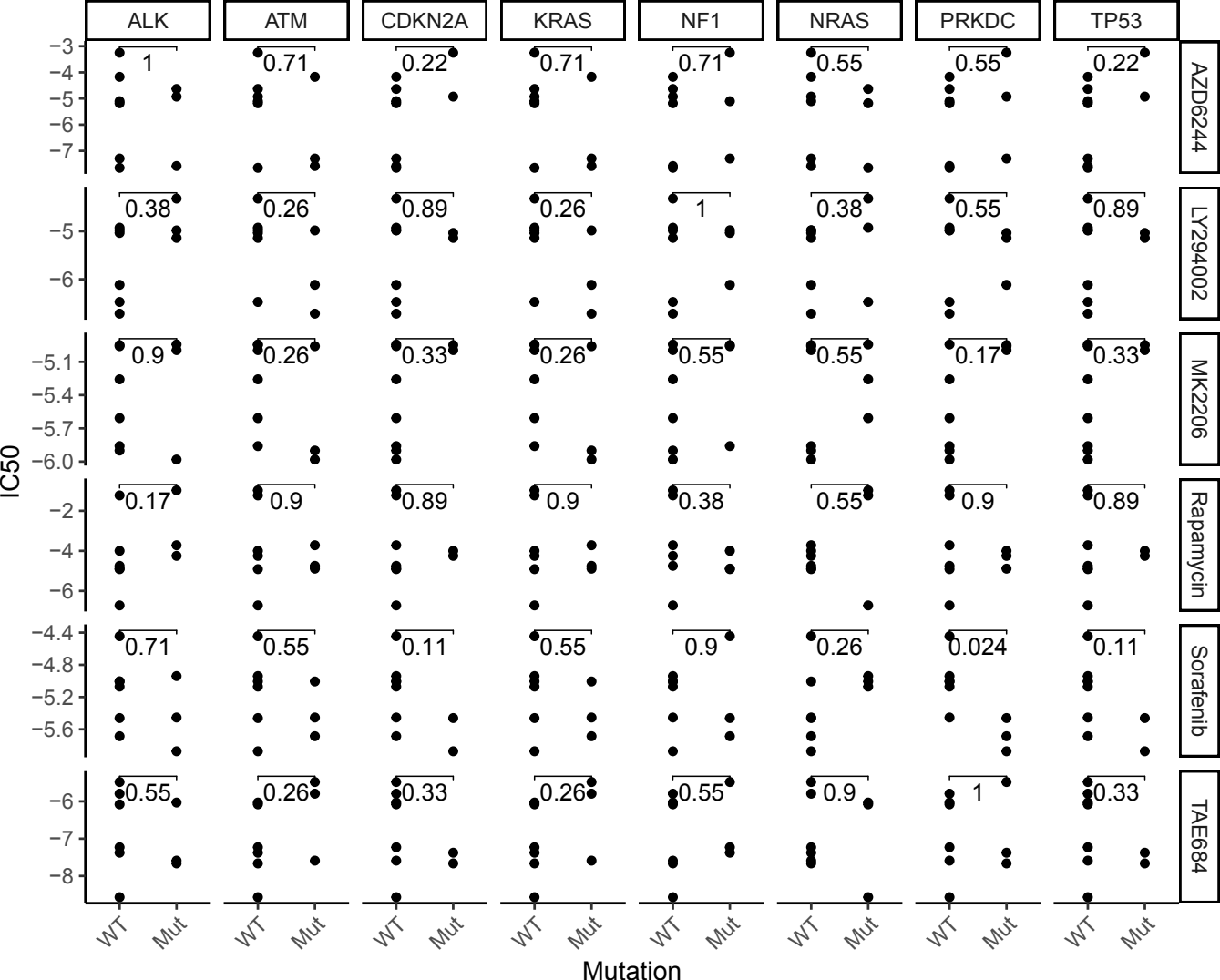

Supplement: S2 Fig — t-test comparison of the IC50 in mutant (Mut) versus wild type (WT) for RAS/P53 and associated genes with mutation frequency between 30% and 70% in our panel. (PDF) [file pcbi.1009515.s002.pdf]

# 1000 most variable genes

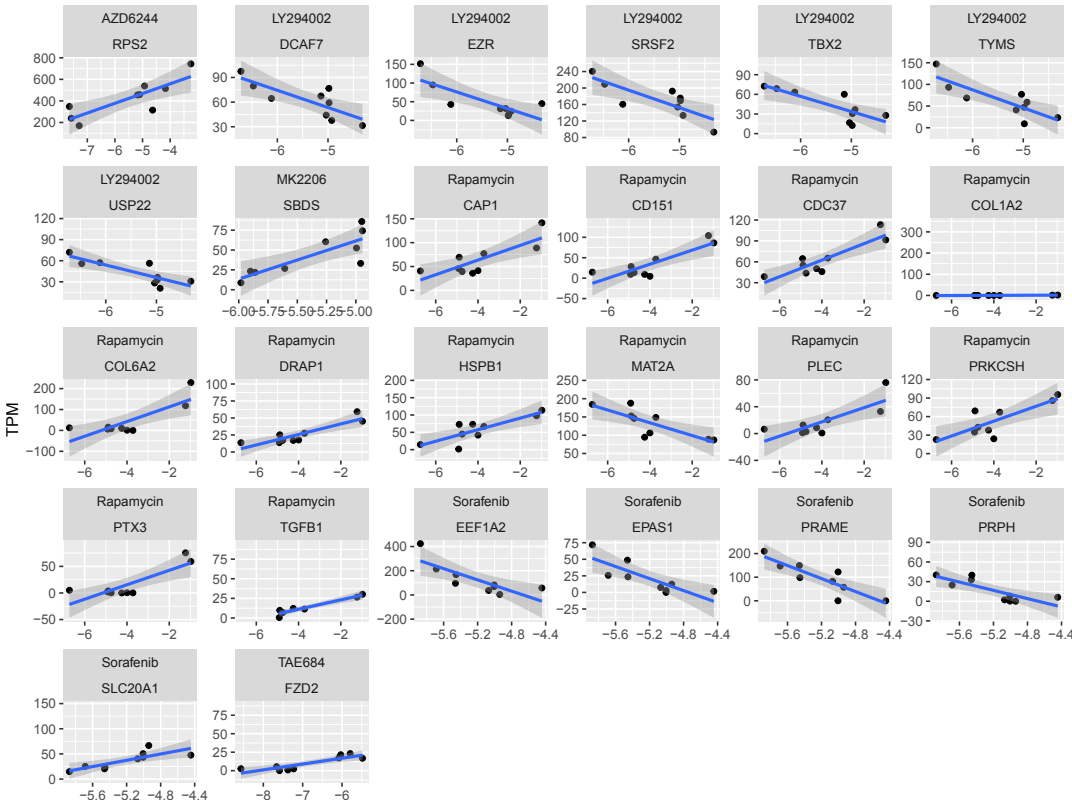

## GO transduction genes

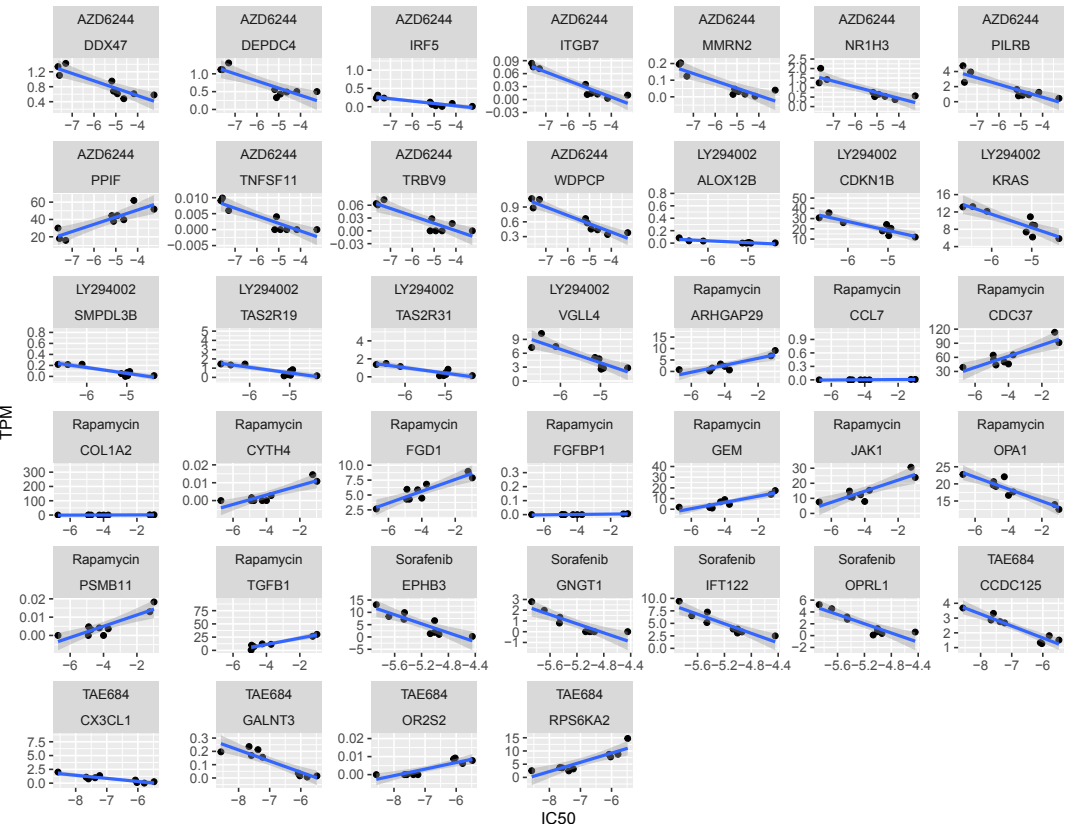

Supplement: S3 Fig — Top correlation between IC50 and mRNA transcript per million for the 1000 most variable genes (top, adjusted p>0.93) and GO signal transduction genes (bottom, adjusted p>0.94). (PDF) [file pcbi.1009515.s003.pdf]

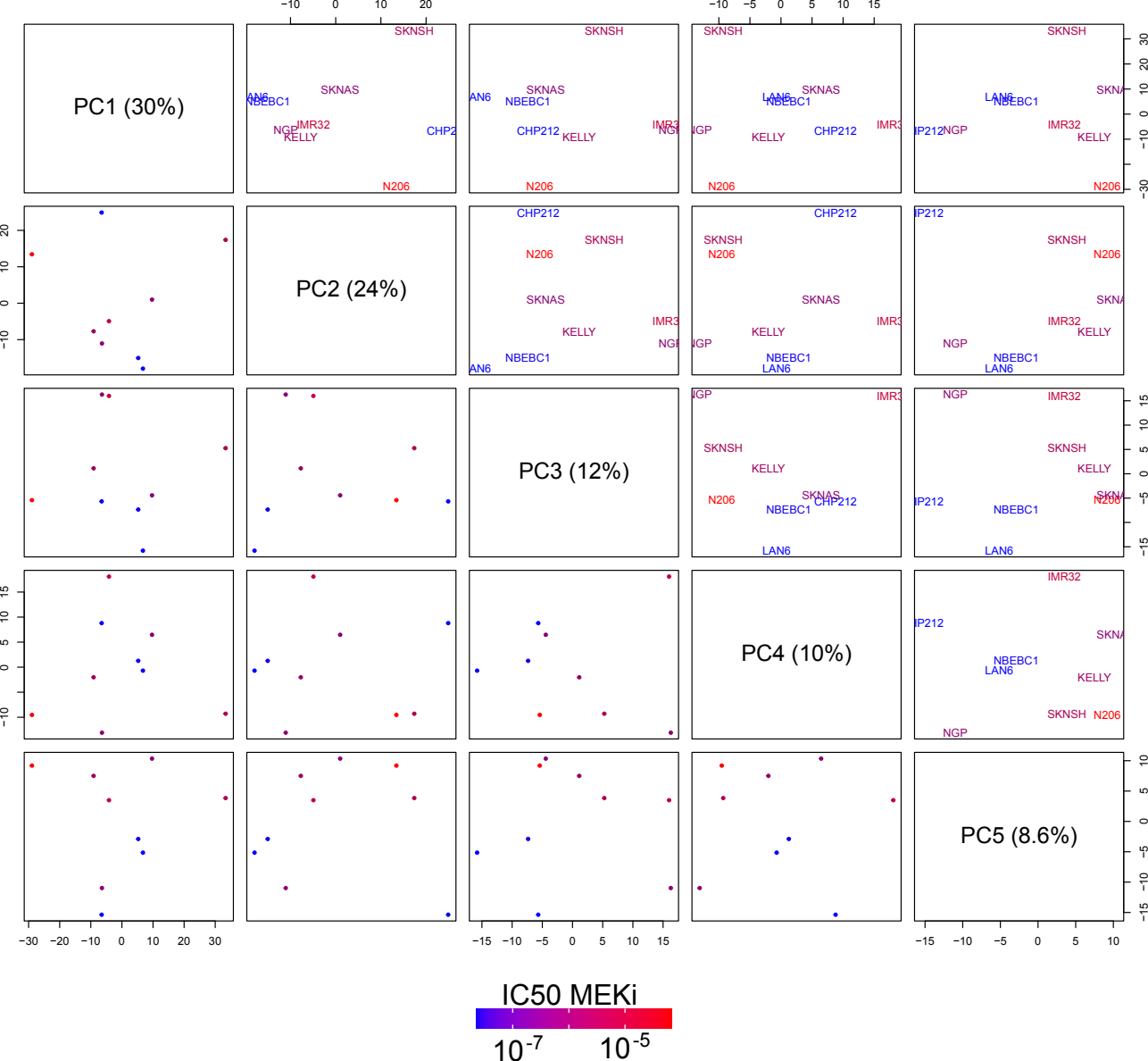

Supplement: S4 Fig — Principal component analysis of the 1000 most variable genes. All components up to the first one explaining less than 10% of the variance are shown. (PDF) [file pcbi.1009515.s004.pdf]

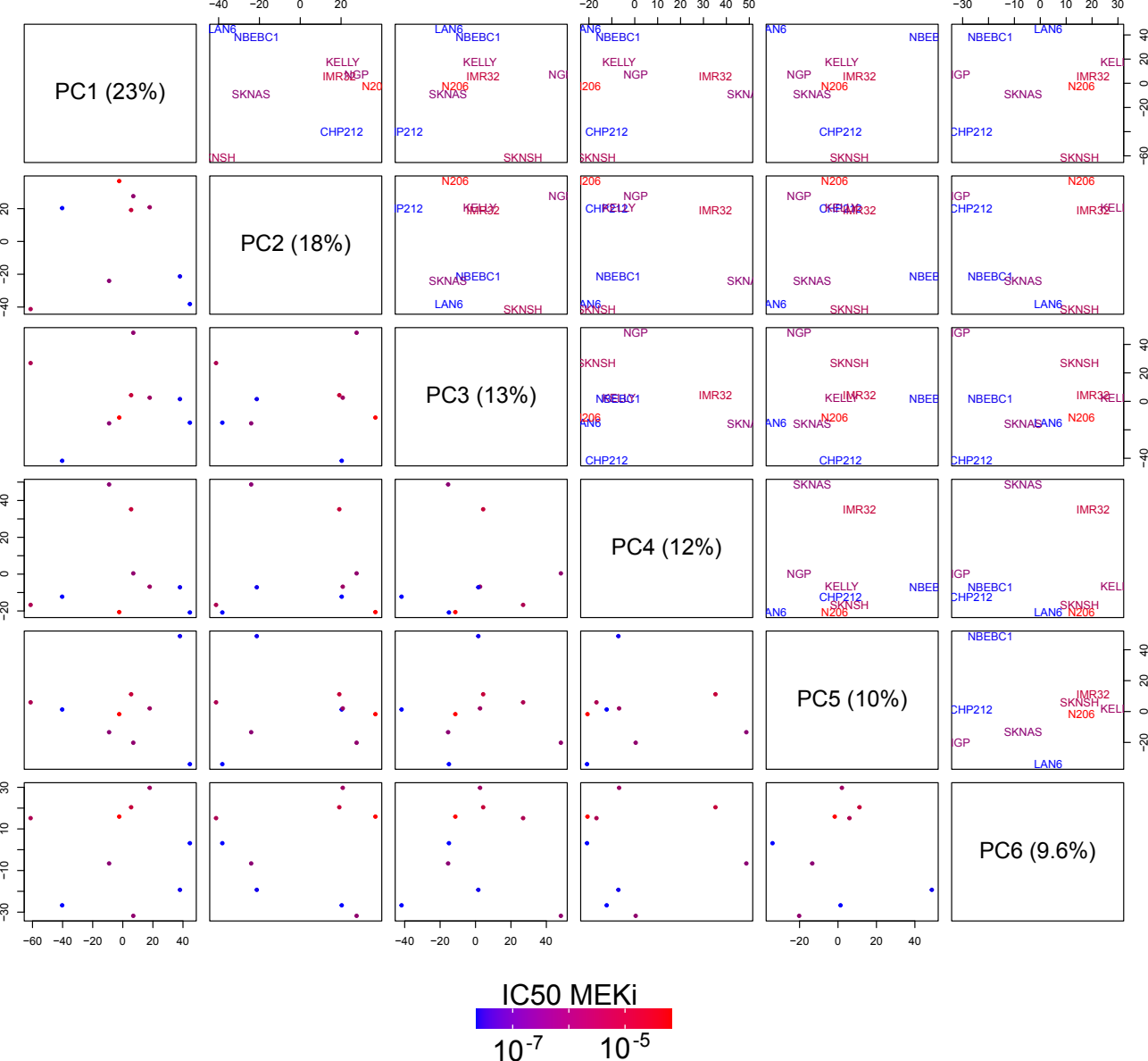

Supplement: S5 Fig — Principal component analysis of the 5262 signal transduction genes. All components up to the first one explaining less than 10% of the variance are shown. (PDF) [file pcbi.1009515.s005.pdf]

AZD6244 IC50 vs NF1 expression, R2= 0.34 p-value= 0.102

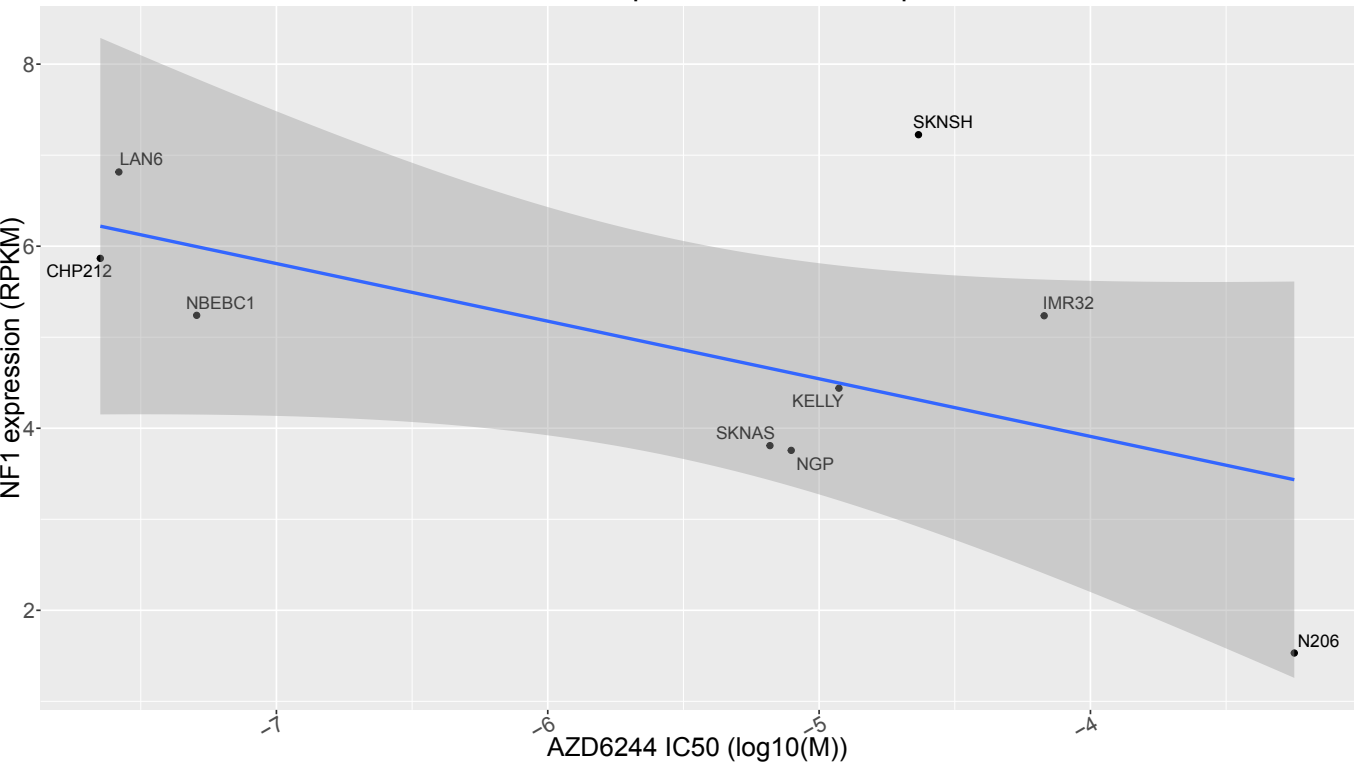

TAE684 IC50 vs ALK expression, R2= 0.0079 p-value= 0.82

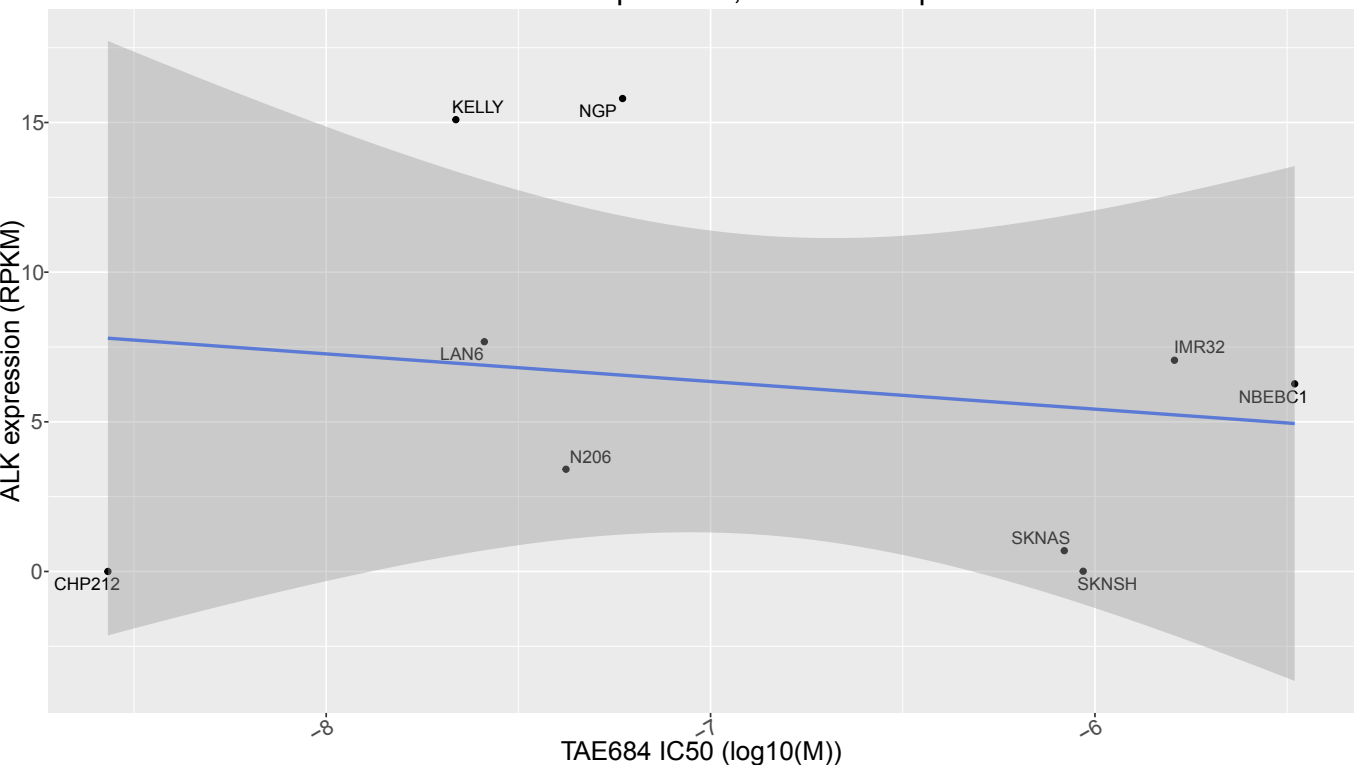

Supplement: S6 Fig — Correlation of NF1 expression with AZD6244 IC50 and ALK expression with TAE684 IC50. (PDF) [file pcbi.1009515.s006.pdf]

Target receptors expression

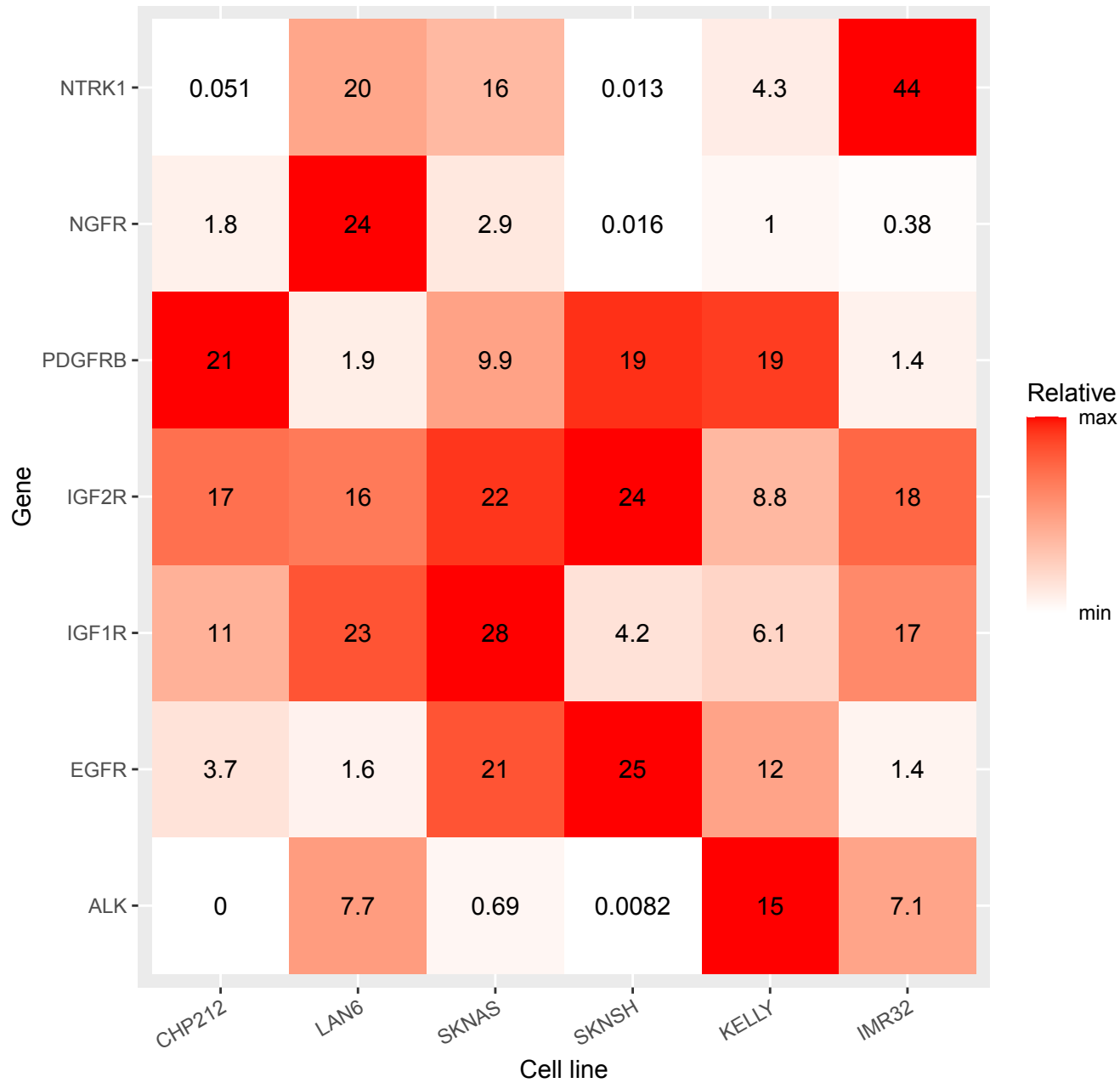

Supplement: S7 Fig — (PDF) [file pcbi.1009515.s007.pdf]

Adaptor &amp; ERBB family expression

Gene

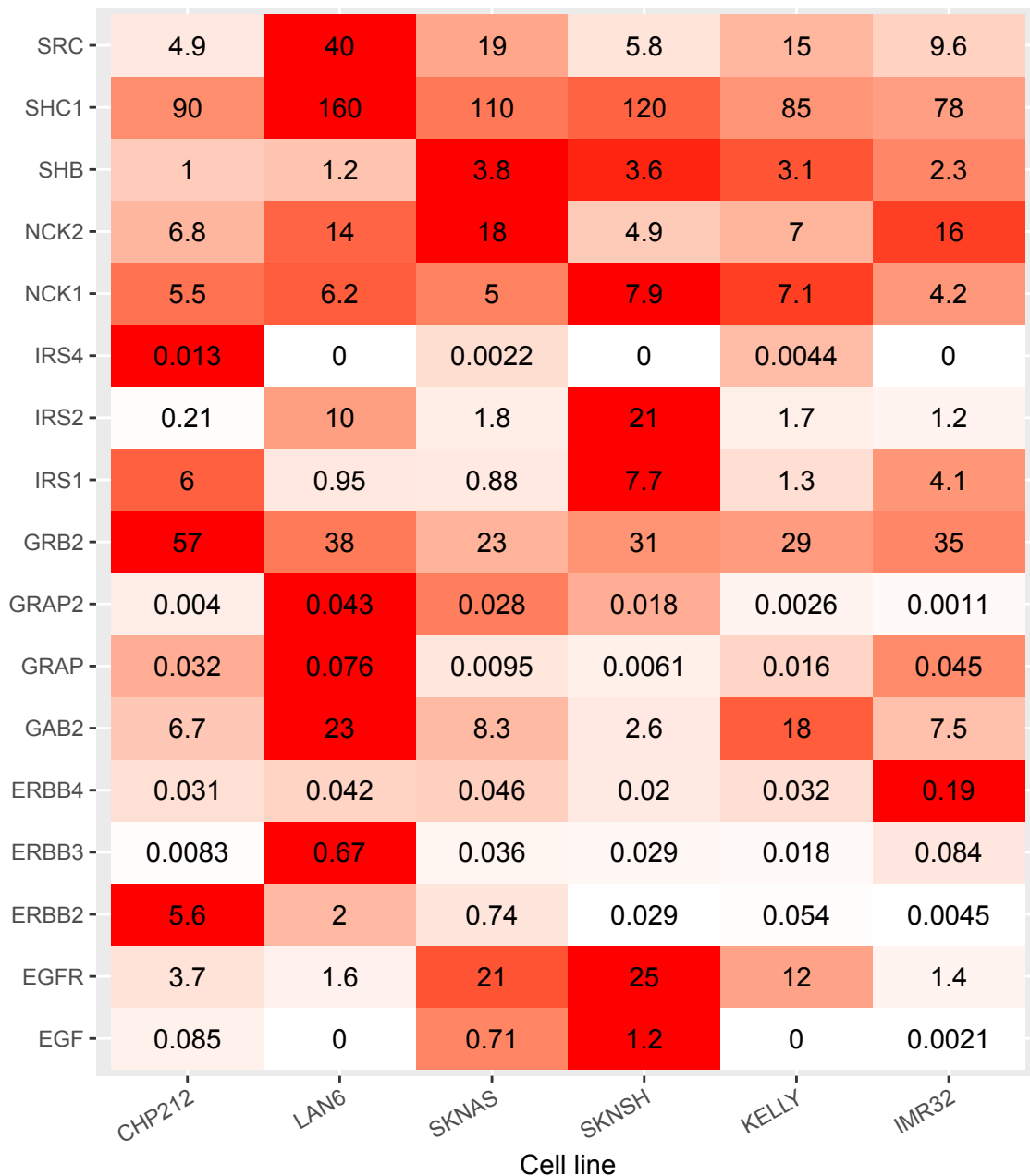

Relative

max

min

Cell line

Supplement: S8 Fig — (PDF) [file pcbi.1009515.s008.pdf]

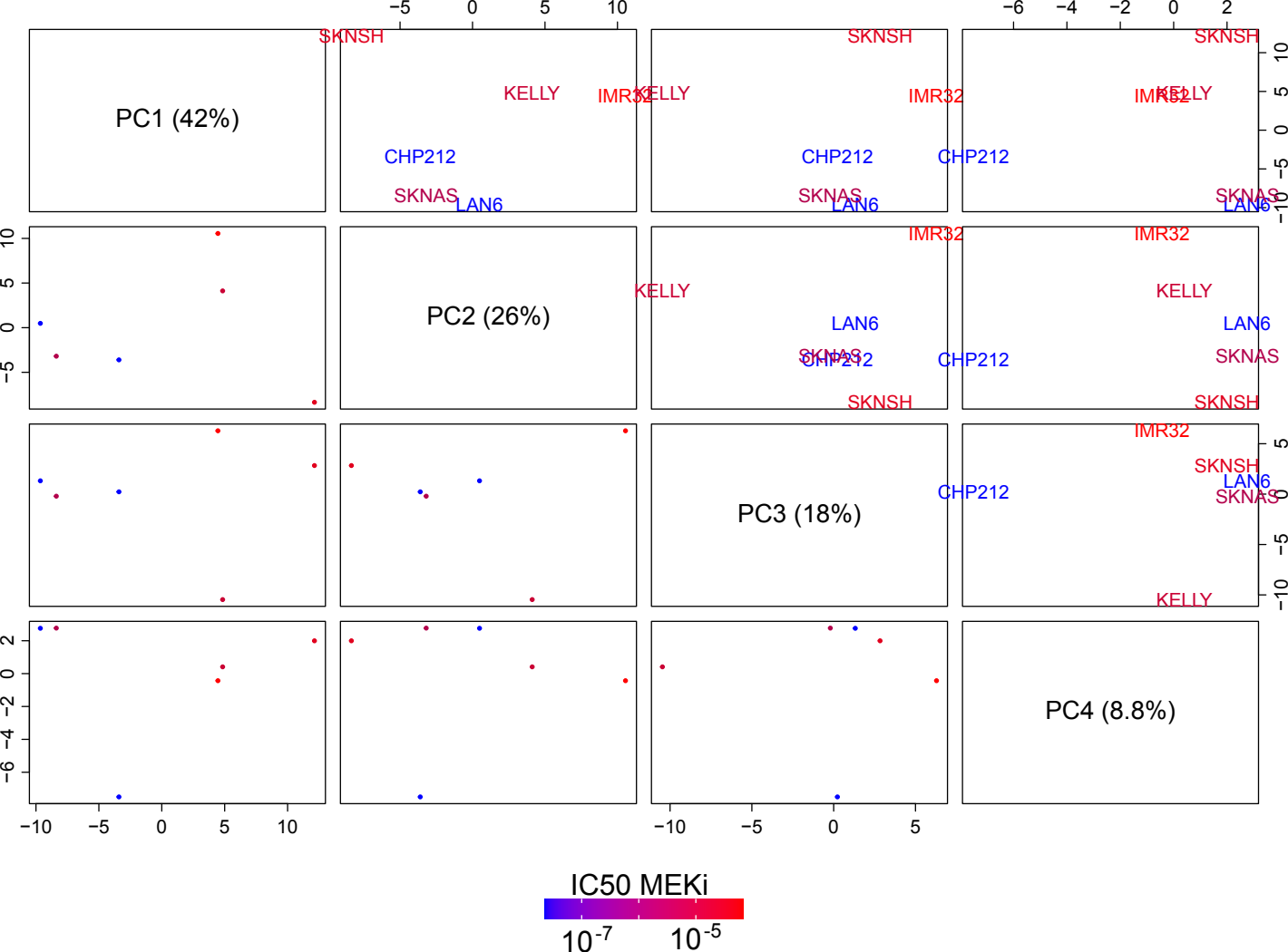

Supplement: S9 Fig — Pair-plot of the principal components from the perturbation in 2. All components up to the first one explaining less than 10% of the variance are shown. (PDF) [file pcbi.1009515.s009.pdf]

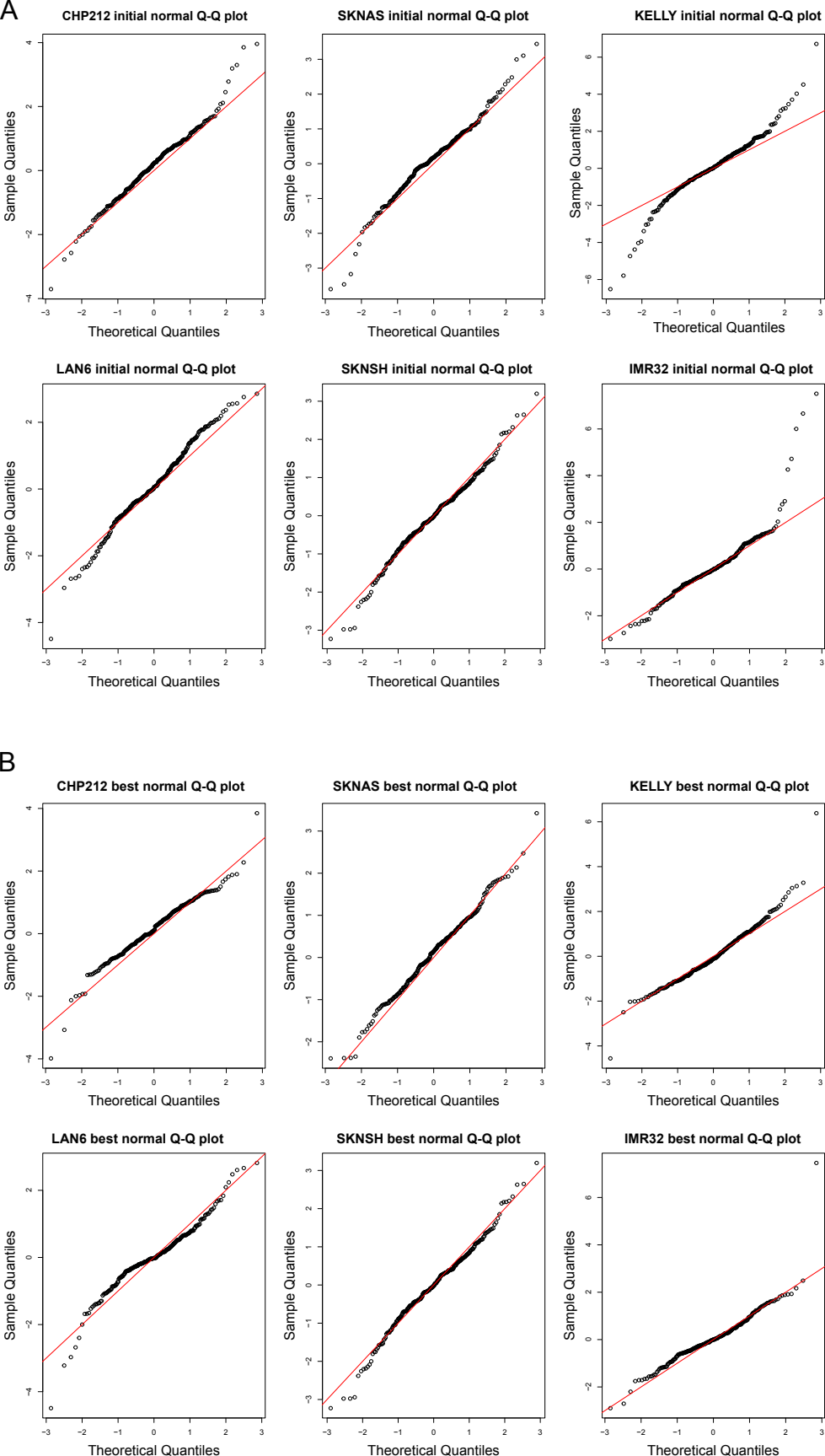

Supplement: S11 Fig — Quantile-quantile plots of the initial models using the (A) literature topology and (B) the final model after extension. (PDF) [file pcbi.1009515.s011.pdf]

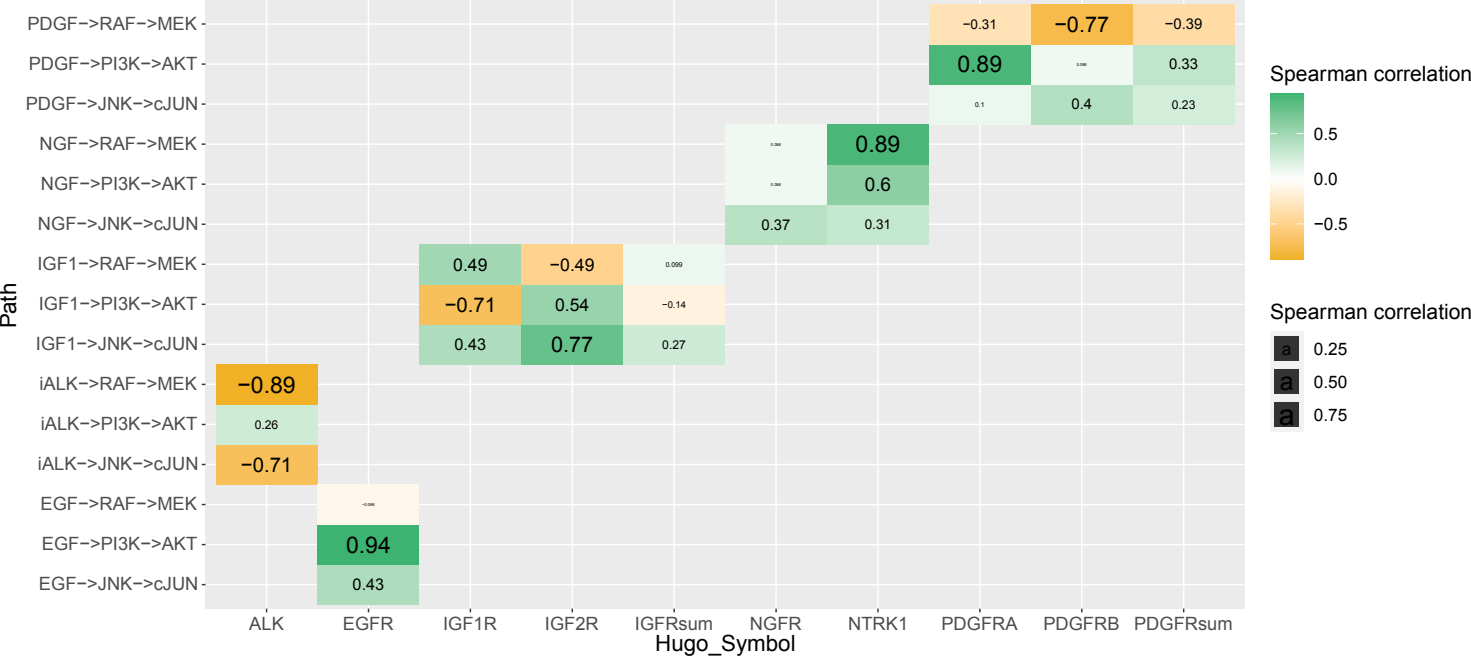

Supplement: S12 Fig — Correlation between the fitted path value from ligands to readouts and the expression of the matching receptor or receptor family. IGFRsum and PDGFRsum are the sum of the isoforms expression for IGFR and PDGFR respectively. (PDF) [file pcbi.1009515.s012.pdf]

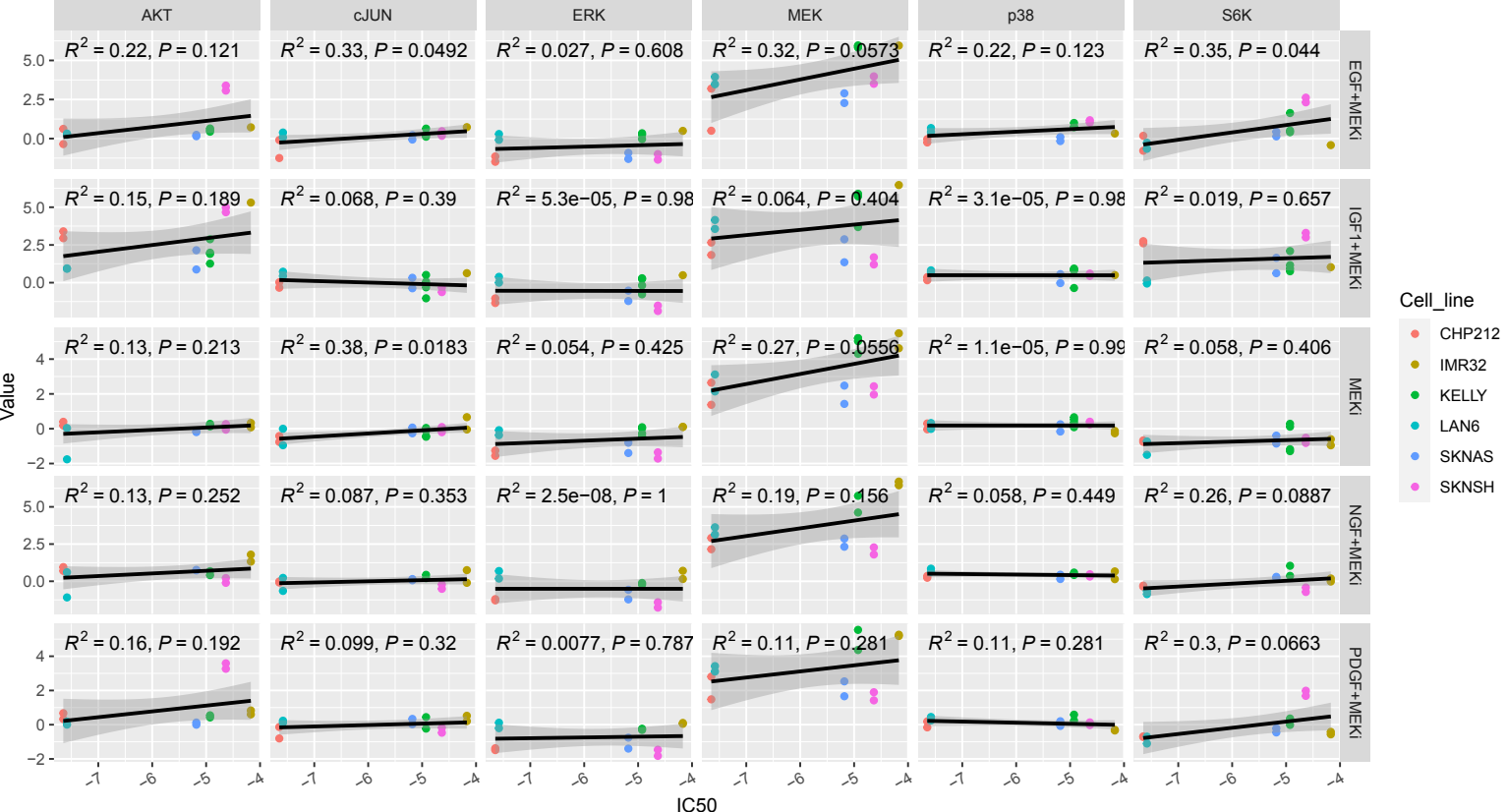

Supplement: S13 Fig — Linear model fit of AZD6244 IC50 response to perturbations including AZD6244. Points are independent replicates, n = 2. (PDF) [file pcbi.1009515.s013.pdf]

Differentially expressed treatments

IMR32

N206

Treatment

TMT signal

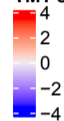

Treatment

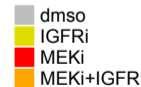

Supplement: S14 Fig — Differentially measured phosphopeptides in IMR32 and N206 after 4h inhibition (FDR < 0.05, n = 3) classified by treatment(s) where the phosphosite is differentially expressed. (PDF) [file pcbi.1009515.s014.pdf]

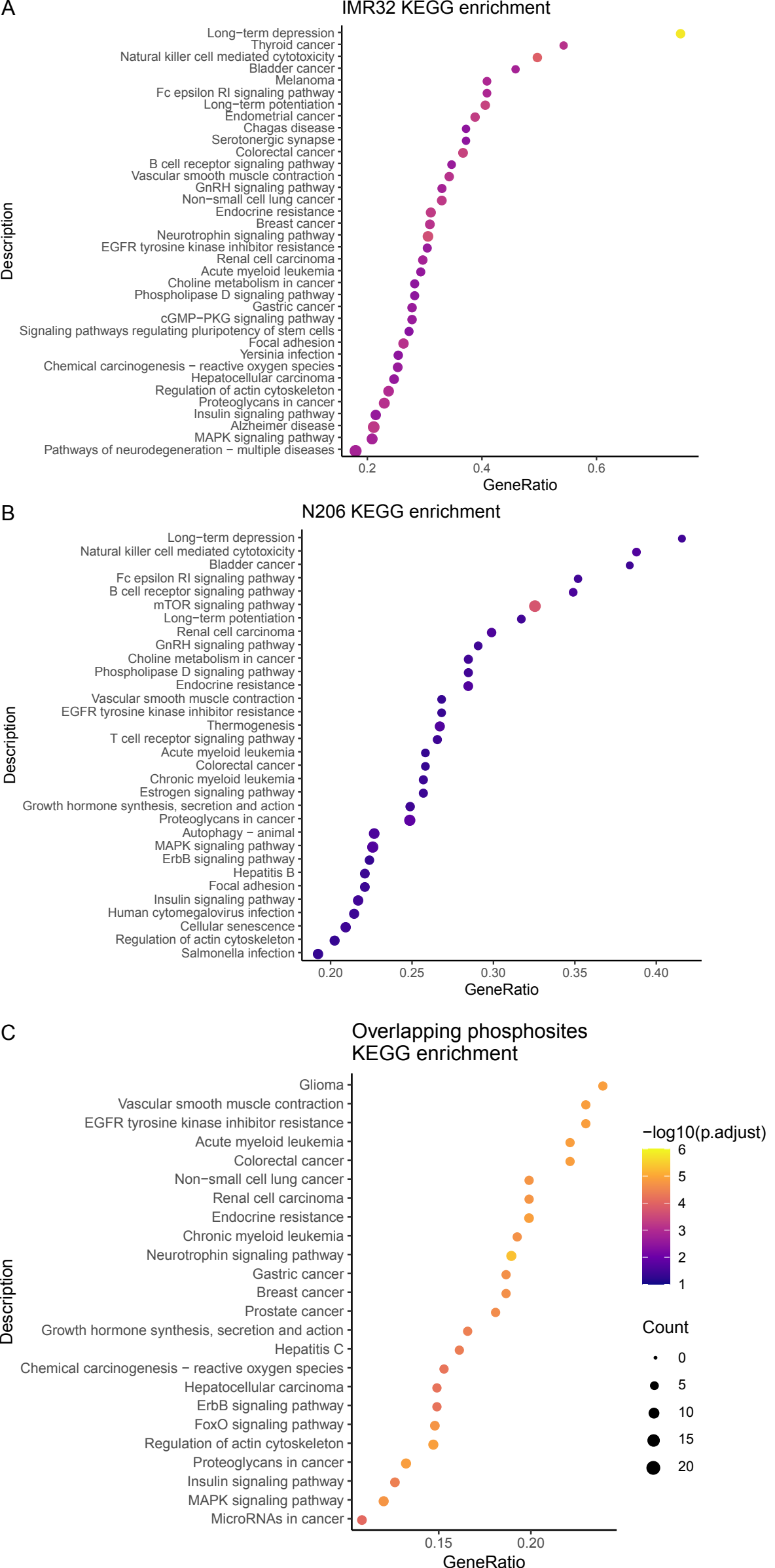

Supplement: S15 Fig — KEGG enrichment of unique genes corresponding to phosphopeptides differentially expressed after MEKi, IGFRi or MEKi+IGFRi treatment in (A) IMR32, (B) N206 or (C) both strictly. Enrichment was computed using the R package enrichKEGG. (PDF) [file pcbi.1009515.s015.pdf]

**A**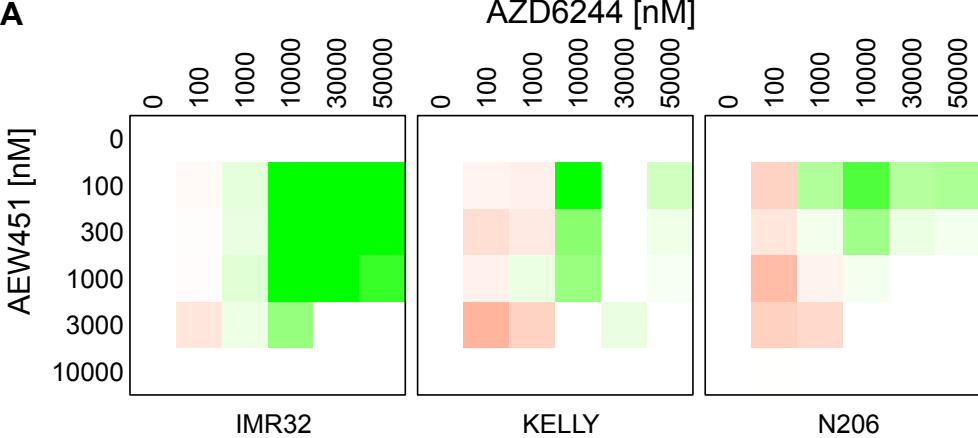

**Loewe  
synergy**

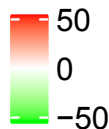**B**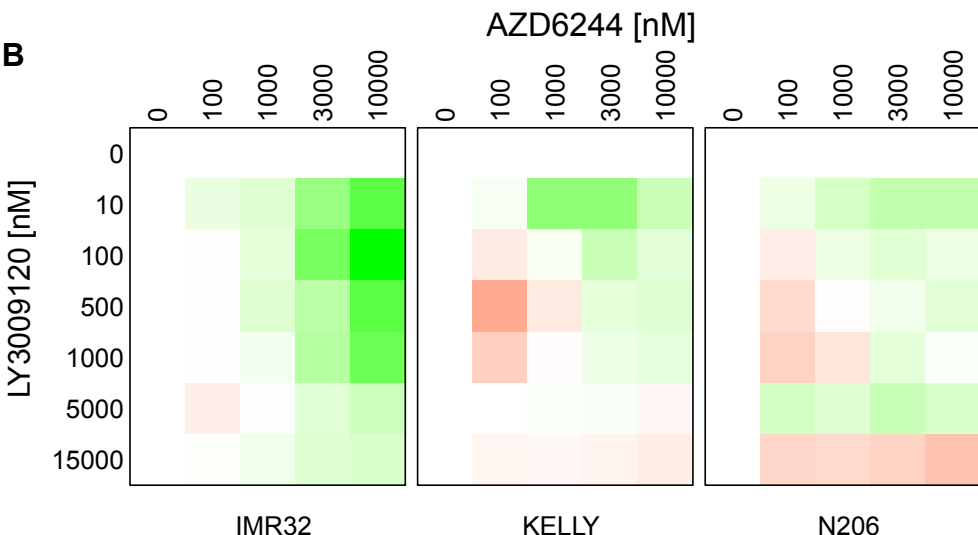

Supplement: S16 Fig — Loewe synergy for the combinations of AZD6244 with (A) AEW541 or (B) LY3009120 shown in 6B. Synergy scores were computed with the R package synergyfinder. Positive scores indicate synergy, negative scores indicate antagonism. (PDF) [file pcbi.1009515.s016.pdf]

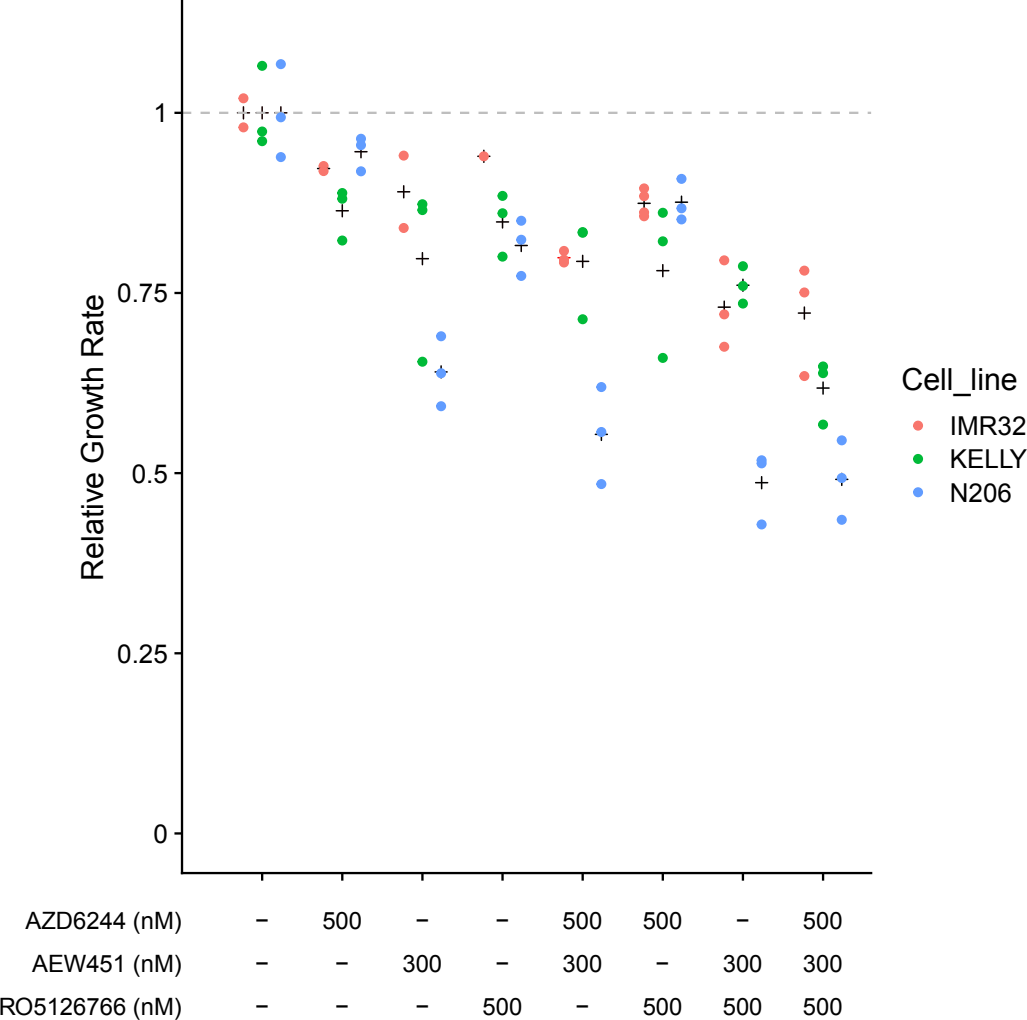

Supplement: S17 Fig — Relative viability of IMR32, KELLY and N206 after treatment with AZD6244, AEW541 and RO5126766 alone or in combination. Confluency was tracked for 72h using the Incucyte Zoom. Growth rate was fitted to the confluency curve and normalised to the average growth rate of the corresponding DMSO controls. black crosses indicate the mean value for each cell line for the corresponding treatment. (PDF) [file pcbi.1009515.s017.pdf]

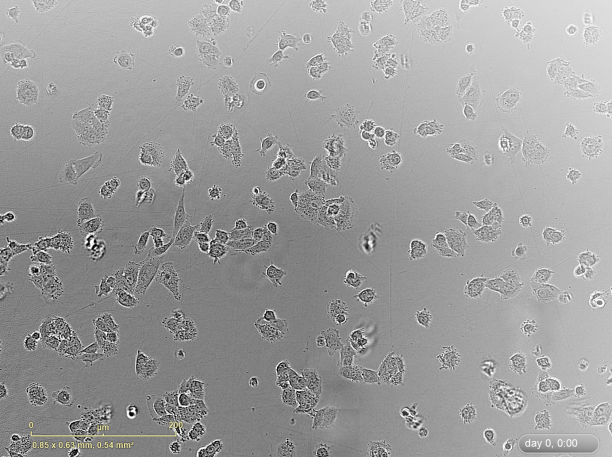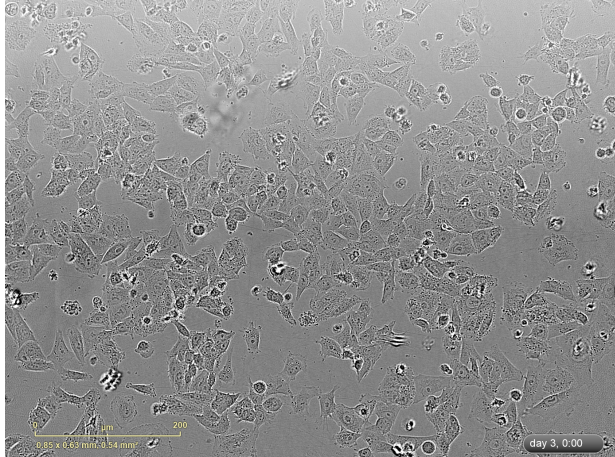

DMSO

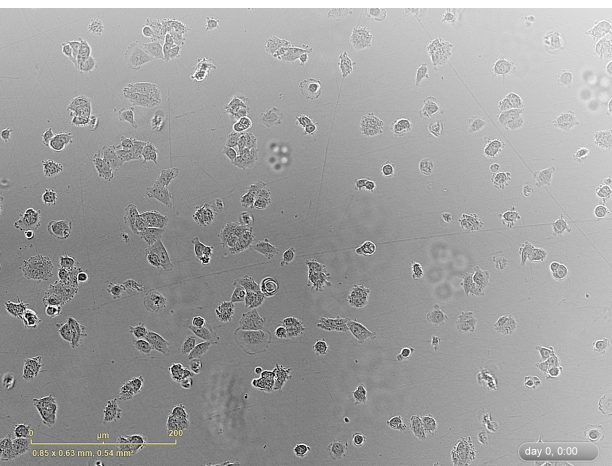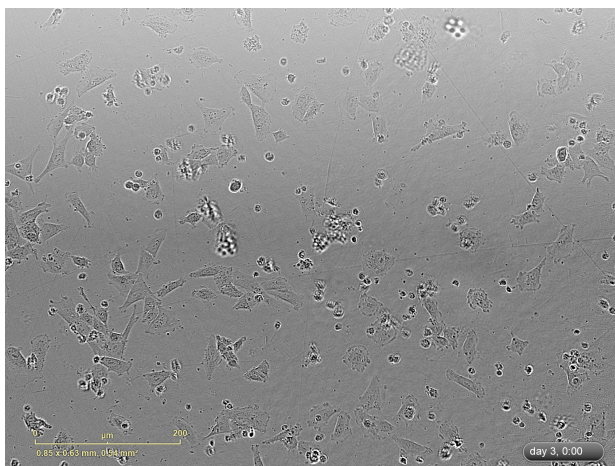

AZD6244 10µM

Supplement: S18 Fig — Incucyte image of the AZD6244-sensitive cell line CHP212 immediately after and 72h after DMSO or AZD6244 treatment. (PDF) [file pcbi.1009515.s018.pdf]

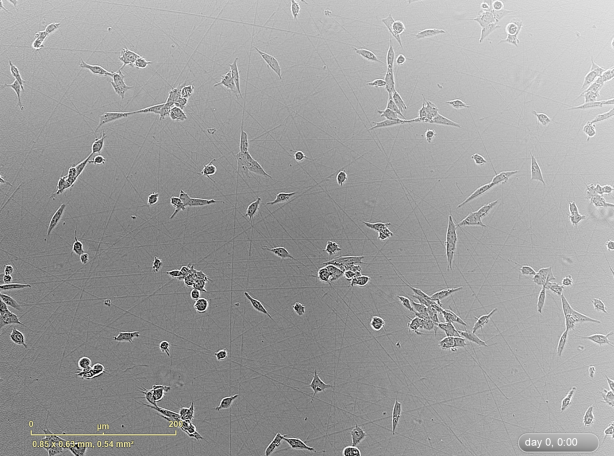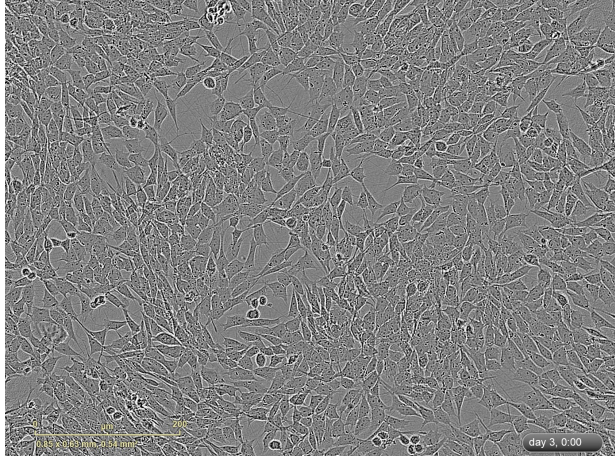

DMSO

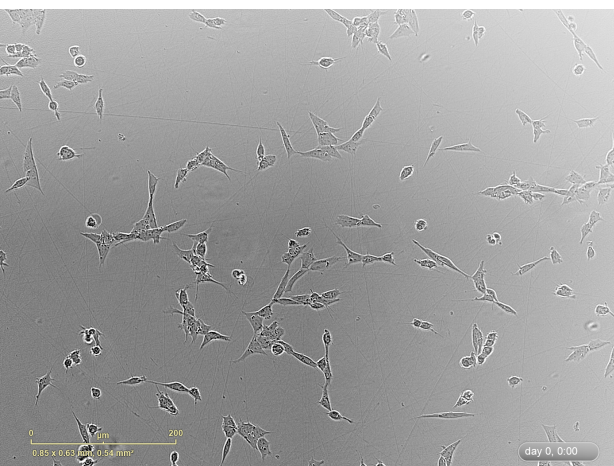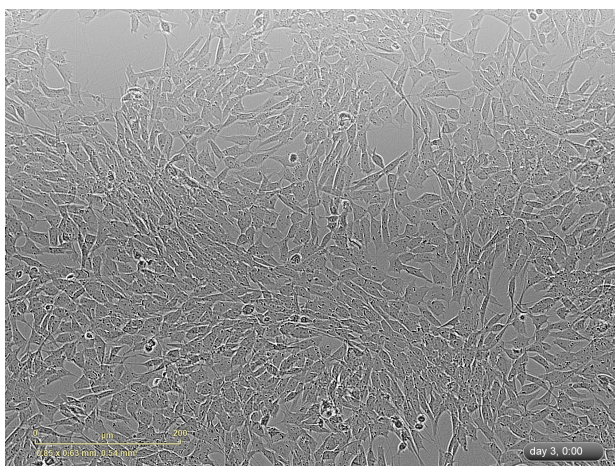

AZD6244 10 $\mu\text{M}$

Supplement: S19 Fig — Incucyte image of the AZD6244-resistant cell line IMR32 immediately after and 72h after DMSO or AZD6244 treatment. (PDF) [file pcbi.1009515.s019.pdf]
