# Supplementary material for: Neuroblastoma signalling models unveil combination therapies targeting feedback-mediated resistance
Source: PLoS Comput Biol. 2021 Nov 4;17(11):e1009515. doi: 10.1371/journal.pcbi.1009515 (PMC8604339; doi:10.1371/journal.pcbi.1009515)
Supplement: S10 Fig — Main loadings in the first 3 principal components of the perturbation data PCA. Colors correspond to the component for which the condition has the highest absolute weight. Table indicates the weight for the top 10 conditions of the first 3 principal components. (PDF) [file pcbi.1009515.s010.pdf]

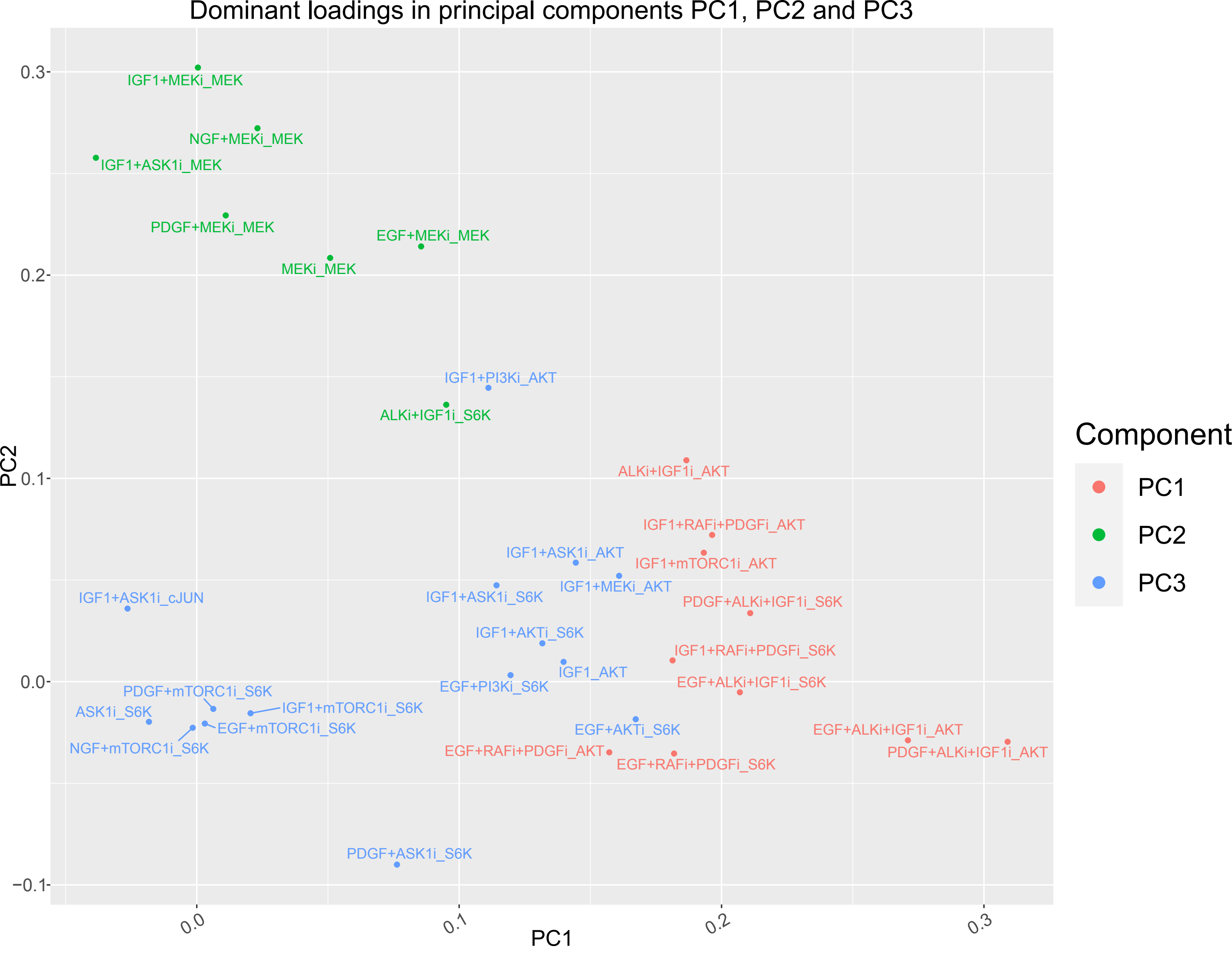

Condition_PC1	PC1	Condition_PC2	PC2	Condition_PC3	PC3
PDGF+ALKi+IGF1i_AKT	0.309	IGF1+MEKi_MEK	0.302	IGF1+ASK1i_MEK	-0.235
EGF+ALKi+IGF1i_AKT	0.271	NGF+MEKi_MEK	0.272	IGF1+PI3Ki_AKT	-0.213
PDGF+ALKi+IGF1i_S6K	0.211	IGF1+ASK1i_MEK	0.258	IGF1+mTORC1i_S6K	-0.210
EGF+ALKi+IGF1i_S6K	0.207	PDGF+MEKi_MEK	0.229	PDGF+ASK1i_S6K	-0.209
IGF1+RAFi+PDGFi_AKT	0.196	EGF+MEKi_MEK	0.214	PDGF+mTORC1i_S6K	-0.208
IGF1+mTORC1i_AKT	0.193	MEKi_MEK	0.208	NGF+mTORC1i_S6K	-0.202
ALKi+IGF1i_AKT	0.187	IGF1+PI3Ki_AKT	0.145	IGF1_AKT	-0.200
EGF+RAFi+PDGFi_S6K	0.182	ALKi+IGF1i_S6K	0.136	EGF+PI3Ki_S6K	0.183
IGF1+RAFi+PDGFi_S6K	0.181	EGF+ASK1i_AKT	-0.118	EGF+AKTi_S6K	0.180
EGF+AKTi_S6K	0.167	IGF1+MEKi_ERK	0.117	IGF1+MEKi_AKT	-0.179
