# Supplementary material for: Neuroblastoma signalling models unveil combination therapies targeting feedback-mediated resistance
Source: PLoS Comput Biol. 2021 Nov 4;17(11):e1009515. doi: 10.1371/journal.pcbi.1009515 (PMC8604339; doi:10.1371/journal.pcbi.1009515)
Supplement: S1 File — (ZIP) [file pcbi.1009515.s022.zip › TNB_characterisation/data_characterisation_TNB.html]

Data characterisation TNB


# Data characterisation TNB

#### Mathurin Dorel

#### 2020-07-13

```
#!/usr/bin/Rscript
```

# Data characterisation TerminateNB

## Loading data

### Load IC50 data

### Mutations from WES data

```
## Warning: 2 parsing failures.
##  row   col               expected actual                             file
## 1036 PHENO no trailing characters     ,0 'WES_TNB_mafs_snvs/BE2C_S10.maf'
## 1310 PHENO no trailing characters     ,0 'WES_TNB_mafs_snvs/BE2C_S10.maf'
```

```
## Warning: 1 parsing failure.
##  row   col               expected actual                               file
## 1660 PHENO no trailing characters     ,0 'WES_TNB_mafs_snvs/NBEBC1_S14.maf'
```

```
## Warning: 1 parsing failure.
##  row   col               expected actual                            file
## 1538 PHENO no trailing characters     ,0 'WES_TNB_mafs_snvs/SHEP_S1.maf'
```

```
## Warning: 1 parsing failure.
##  row   col               expected actual                             file
## 1516 PHENO no trailing characters     ,0 'WES_TNB_mafs_snvs/SKNSH_S7.maf'
```

#### Genes of interest

```
## `summarise()` ungrouping output (override with `.groups` argument)
## `summarise()` ungrouping output (override with `.groups` argument)
```

```
## Joining, by = "Cell_line"
## Joining, by = "Cell_line"
```

| Hugo\_Symbol | Variant\_Classification | Variant\_Type | Cell\_line | Protein\_Change |
| --- | --- | --- | --- | --- |
| ALK | Missense\_Mutation | SNV | CHLA20 | p.R1275Q |
| ALK | Missense\_Mutation | SNV | KELLY | p.F1174L |
| ALK | Missense\_Mutation | SNV | LAN1 | p.F1174L |
| ALK | Missense\_Mutation | SNV | LAN6 | p.D1091N |
| ALK | Missense\_Mutation | SNV | SHEP | p.F1174L |
| ALK | Missense\_Mutation | SNV | SKNSH | p.F1174L |
| ATM | Missense\_Mutation | SNV | CHLA20 | p.S707P |
| ATM | Missense\_Mutation | SNV | IMR32 | p.I1547M |
| ATM | Missense\_Mutation | SNV | KAN | p.H2788L |
| ATM | Missense\_Mutation | SNV | LAN1 | p.S1655P |
| ATM | Missense\_Mutation | SNV | LAN1 | p.L2307F |
| ATM | Missense\_Mutation | SNV | LAN1 | p.D2672E |
| ATM | Missense\_Mutation | SNV | LAN6 | p.S2218R |
| ATM | Missense\_Mutation | SNV | NBEBC1 | p.C1674Y |
| ATM | Missense\_Mutation | SNV | NBEBC1 | p.T1743K |
| ATM | Nonsense\_Mutation | SNV | NBEBC1 | p.R2419\* |
| ATR | Missense\_Mutation | SNV | KAN | p.I2197T |
| ATR | Missense\_Mutation | SNV | SKNAS | p.V2158F |
| ATRX | Missense\_Mutation | SNV | BE2C | p.E929Q |
| ATRX | Missense\_Mutation | SNV | BE2C | p.R907Q |
| ATRX | Missense\_Mutation | SNV | BE2C | p.N755K |
| ATRX | Missense\_Mutation | SNV | CHLA20 | p.E929Q |
| ATRX | Missense\_Mutation | SNV | GIMEN | p.V1181L |
| ATRX | Missense\_Mutation | SNV | GIMEN | p.E929Q |
| ATRX | Missense\_Mutation | SNV | IMR32 | p.E1456G |
| ATRX | Missense\_Mutation | SNV | KAN | p.S2142T |
| ATRX | Missense\_Mutation | SNV | KAN | p.D1852G |
| ATRX | Missense\_Mutation | SNV | KAN | p.E929Q |
| ATRX | Missense\_Mutation | SNV | KAN | p.K96I |
| ATRX | Missense\_Mutation | SNV | KAN | p.K46R |
| ATRX | Missense\_Mutation | SNV | LAN6 | p.E929Q |
| ATRX | Missense\_Mutation | SNV | N206 | p.E929Q |
| ATRX | Missense\_Mutation | SNV | N206 | p.R907Q |
| ATRX | Missense\_Mutation | SNV | N206 | p.N755K |
| ATRX | Missense\_Mutation | SNV | NBEBC1 | p.E2360G |
| ATRX | Missense\_Mutation | SNV | NBEBC1 | p.K2359E |
| ATRX | Missense\_Mutation | SNV | NBEBC1 | p.G2018W |
| ATRX | Missense\_Mutation | SNV | NBEBC1 | p.D2010E |
| ATRX | Missense\_Mutation | SNV | NBEBC1 | p.Q1663R |
| ATRX | Missense\_Mutation | SNV | NBEBC1 | p.E1460K |
| ATRX | Missense\_Mutation | SNV | NBEBC1 | p.E929Q |
| ATRX | Missense\_Mutation | SNV | NBEBC1 | p.N862K |
| ATRX | Missense\_Mutation | SNV | SHEP | p.E929Q |
| ATRX | Missense\_Mutation | SNV | SKNAS | p.E929Q |
| ATRX | Missense\_Mutation | SNV | SKNSH | p.E929Q |
| BRAF | Missense\_Mutation | SNV | IMR32 | p.K507E |
| BRAF | Missense\_Mutation | SNV | KAN | p.P14R |
| CDKN2A | Missense\_Mutation | SNV | KELLY | p.A68T |
| CDKN2A | Frame\_Shift\_Del | DEL | N206 | p.A76Pfs\*70 |
| DEPTOR | Missense\_Mutation | SNV | LAN6 | p.Q239K |
| KRAS | Missense\_Mutation | SNV | IMR32 | p.V181M |
| KRAS | Missense\_Mutation | SNV | LAN6 | p.G12C |
| KRAS | Missense\_Mutation | SNV | NBEBC1 | p.G12D |
| MAPK10 | Missense\_Mutation | SNV | IMR32 | p.I124M |
| MAPK8 | Missense\_Mutation | SNV | KELLY | p.A282D |
| MAPK8 | Missense\_Mutation | SNV | N206 | p.A282D |
| MTOR | Missense\_Mutation | SNV | IMR32 | p.F1888V |
| MTOR | Missense\_Mutation | SNV | IMR32 | p.R1482C |
| MTOR | Nonsense\_Mutation | SNV | NBEBC1 | p.W1633\* |
| NF1 | Frame\_Shift\_Del | DEL | BE2C | NA |
| NF1 | Missense\_Mutation | SNV | LAN1 | p.D1047V |
| NF1 | Frame\_Shift\_Del | DEL | N206 | p.N664\* |
| NF1 | Missense\_Mutation | SNV | NBEBC1 | p.P464Q |
| NF1 | Nonsense\_Mutation | SNV | NGP | p.E1423\* |
| NRAS | Missense\_Mutation | SNV | CHP212 | p.Q61K |
| NRAS | Missense\_Mutation | SNV | SKNAS | p.Q61K |
| NRAS | Missense\_Mutation | SNV | SKNSH | p.Q61K |
| PIK3CA | Missense\_Mutation | SNV | BE2C | p.M697I |
| PRKDC | Missense\_Mutation | SNV | KAN | p.L545F |
| PRKDC | Missense\_Mutation | SNV | KELLY | p.V1452L |
| PRKDC | Missense\_Mutation | SNV | N206 | p.V1452L |
| PRKDC | Missense\_Mutation | SNV | NBEBC1 | p.C2857R |
| RICTOR | Missense\_Mutation | SNV | LAN1 | p.C728Y |
| TP53 | Missense\_Mutation | SNV | BE2C | p.C135F |
| TP53 | Missense\_Mutation | SNV | KELLY | p.P177T |
| TP53 | Nonsense\_Mutation | SNV | LAN1 | p.C182\* |
| TP53 | Missense\_Mutation | SNV | N206 | p.P177T |

```
## $BE2C
## [1] "PIK3CA" "TP53"   "NF1"    "ATRX"   "ATRX"   "ATRX"  
## 
## $CHLA20
## [1] "ALK"  "ATM"  "ATRX"
## 
## $CHP212
## [1] "NRAS"
## 
## $GIMEN
## [1] "ATRX" "ATRX"
## 
## $IMR32
## [1] "MTOR"   "MTOR"   "MAPK10" "BRAF"   "ATM"    "KRAS"   "ATRX"  
## 
## $KAN
## [1] "ATR"   "BRAF"  "PRKDC" "ATM"   "ATRX"  "ATRX"  "ATRX"  "ATRX"  "ATRX" 
## 
## $KELLY
## [1] "ALK"    "PRKDC"  "CDKN2A" "MAPK8"  "TP53"  
## 
## $LAN1
## [1] "ALK"    "RICTOR" "ATM"    "ATM"    "ATM"    "TP53"   "NF1"   
## 
## $LAN6
## [1] "ALK"    "DEPTOR" "ATM"    "KRAS"   "ATRX"  
## 
## $N206
## [1] "PRKDC"  "CDKN2A" "MAPK8"  "TP53"   "NF1"    "ATRX"   "ATRX"   "ATRX"  
## 
## $NBEBC1
##  [1] "MTOR"  "PRKDC" "ATM"   "ATM"   "ATM"   "KRAS"  "NF1"   "ATRX"  "ATRX"  "ATRX"  "ATRX"  "ATRX"  "ATRX"  "ATRX"  "ATRX" 
## 
## $NGP
## [1] "NF1"
## 
## $SHEP
## [1] "ALK"  "ATRX"
## 
## $SKNAS
## [1] "NRAS" "ATR"  "ATRX"
## 
## $SKNSH
## [1] "NRAS" "ALK"  "ATRX"
```

### Load RNAseq data

Generate a matrix to annotate the mutation and perturbation heatmaps with RNAseq data

### Load perturbations data

```
## Warning: `as.tibble()` is deprecated as of tibble 2.0.0.
## Please use `as_tibble()` instead.
## The signature and semantics have changed, see `?as_tibble`.
## This warning is displayed once every 8 hours.
## Call `lifecycle::last_warnings()` to see where this warning was generated.
```

```
## Warning in qt(0.975, n - 1): NaNs produced

## Warning in qt(0.975, n - 1): NaNs produced

## Warning in qt(0.975, n - 1): NaNs produced

## Warning in qt(0.975, n - 1): NaNs produced

## Warning in qt(0.975, n - 1): NaNs produced

## Warning in qt(0.975, n - 1): NaNs produced

## Warning in qt(0.975, n - 1): NaNs produced

## Warning in qt(0.975, n - 1): NaNs produced

## Warning in qt(0.975, n - 1): NaNs produced

## Warning in qt(0.975, n - 1): NaNs produced

## Warning in qt(0.975, n - 1): NaNs produced

## Warning in qt(0.975, n - 1): NaNs produced

## Warning in qt(0.975, n - 1): NaNs produced

## Warning in qt(0.975, n - 1): NaNs produced

## Warning in qt(0.975, n - 1): NaNs produced

## Warning in qt(0.975, n - 1): NaNs produced

## Warning in qt(0.975, n - 1): NaNs produced

## Warning in qt(0.975, n - 1): NaNs produced

## Warning in qt(0.975, n - 1): NaNs produced

## Warning in qt(0.975, n - 1): NaNs produced

## Warning in qt(0.975, n - 1): NaNs produced

## Warning in qt(0.975, n - 1): NaNs produced

## Warning in qt(0.975, n - 1): NaNs produced

## Warning in qt(0.975, n - 1): NaNs produced

## Warning in qt(0.975, n - 1): NaNs produced

## Warning in qt(0.975, n - 1): NaNs produced

## Warning in qt(0.975, n - 1): NaNs produced

## Warning in qt(0.975, n - 1): NaNs produced

## Warning in qt(0.975, n - 1): NaNs produced

## Warning in qt(0.975, n - 1): NaNs produced

## Warning in qt(0.975, n - 1): NaNs produced

## Warning in qt(0.975, n - 1): NaNs produced

## Warning in qt(0.975, n - 1): NaNs produced

## Warning in qt(0.975, n - 1): NaNs produced

## Warning in qt(0.975, n - 1): NaNs produced

## Warning in qt(0.975, n - 1): NaNs produced

## Warning in qt(0.975, n - 1): NaNs produced

## Warning in qt(0.975, n - 1): NaNs produced

## Warning in qt(0.975, n - 1): NaNs produced

## Warning in qt(0.975, n - 1): NaNs produced

## Warning in qt(0.975, n - 1): NaNs produced

## Warning in qt(0.975, n - 1): NaNs produced

## Warning in qt(0.975, n - 1): NaNs produced

## Warning in qt(0.975, n - 1): NaNs produced

## Warning in qt(0.975, n - 1): NaNs produced

## Warning in qt(0.975, n - 1): NaNs produced

## Warning in qt(0.975, n - 1): NaNs produced

## Warning in qt(0.975, n - 1): NaNs produced

## Warning in qt(0.975, n - 1): NaNs produced

## Warning in qt(0.975, n - 1): NaNs produced

## Warning in qt(0.975, n - 1): NaNs produced

## Warning in qt(0.975, n - 1): NaNs produced

## Warning in qt(0.975, n - 1): NaNs produced

## Warning in qt(0.975, n - 1): NaNs produced

## Warning in qt(0.975, n - 1): NaNs produced

## Warning in qt(0.975, n - 1): NaNs produced

## Warning in qt(0.975, n - 1): NaNs produced

## Warning in qt(0.975, n - 1): NaNs produced

## Warning in qt(0.975, n - 1): NaNs produced

## Warning in qt(0.975, n - 1): NaNs produced

## Warning in qt(0.975, n - 1): NaNs produced

## Warning in qt(0.975, n - 1): NaNs produced

## Warning in qt(0.975, n - 1): NaNs produced

## Warning in qt(0.975, n - 1): NaNs produced

## Warning in qt(0.975, n - 1): NaNs produced

## Warning in qt(0.975, n - 1): NaNs produced

## Warning in qt(0.975, n - 1): NaNs produced

## Warning in qt(0.975, n - 1): NaNs produced

## Warning in qt(0.975, n - 1): NaNs produced

## Warning in qt(0.975, n - 1): NaNs produced

## Warning in qt(0.975, n - 1): NaNs produced

## Warning in qt(0.975, n - 1): NaNs produced

## Warning in qt(0.975, n - 1): NaNs produced

## Warning in qt(0.975, n - 1): NaNs produced

## Warning in qt(0.975, n - 1): NaNs produced

## Warning in qt(0.975, n - 1): NaNs produced

## Warning in qt(0.975, n - 1): NaNs produced

## Warning in qt(0.975, n - 1): NaNs produced

## Warning in qt(0.975, n - 1): NaNs produced

## Warning in qt(0.975, n - 1): NaNs produced

## Warning in qt(0.975, n - 1): NaNs produced

## Warning in qt(0.975, n - 1): NaNs produced

## Warning in qt(0.975, n - 1): NaNs produced

## Warning in qt(0.975, n - 1): NaNs produced

## Warning in qt(0.975, n - 1): NaNs produced

## Warning in qt(0.975, n - 1): NaNs produced

## Warning in qt(0.975, n - 1): NaNs produced

## Warning in qt(0.975, n - 1): NaNs produced

## Warning in qt(0.975, n - 1): NaNs produced

## Warning in qt(0.975, n - 1): NaNs produced

## Warning in qt(0.975, n - 1): NaNs produced

## Warning in qt(0.975, n - 1): NaNs produced

## Warning in qt(0.975, n - 1): NaNs produced

## Warning in qt(0.975, n - 1): NaNs produced

## Warning in qt(0.975, n - 1): NaNs produced

## Warning in qt(0.975, n - 1): NaNs produced

## Warning in qt(0.975, n - 1): NaNs produced

## Warning in qt(0.975, n - 1): NaNs produced

## Warning in qt(0.975, n - 1): NaNs produced

## Warning in qt(0.975, n - 1): NaNs produced

## Warning in qt(0.975, n - 1): NaNs produced

## Warning in qt(0.975, n - 1): NaNs produced

## Warning in qt(0.975, n - 1): NaNs produced

## Warning in qt(0.975, n - 1): NaNs produced

## Warning in qt(0.975, n - 1): NaNs produced

## Warning in qt(0.975, n - 1): NaNs produced

## Warning in qt(0.975, n - 1): NaNs produced

## Warning in qt(0.975, n - 1): NaNs produced

## Warning in qt(0.975, n - 1): NaNs produced

## Warning in qt(0.975, n - 1): NaNs produced

## Warning in qt(0.975, n - 1): NaNs produced

## Warning in qt(0.975, n - 1): NaNs produced

## Warning in qt(0.975, n - 1): NaNs produced

## Warning in qt(0.975, n - 1): NaNs produced

## Warning in qt(0.975, n - 1): NaNs produced

## Warning in qt(0.975, n - 1): NaNs produced

## Warning in qt(0.975, n - 1): NaNs produced

## Warning in qt(0.975, n - 1): NaNs produced

## Warning in qt(0.975, n - 1): NaNs produced

## Warning in qt(0.975, n - 1): NaNs produced

## Warning in qt(0.975, n - 1): NaNs produced

## Warning in qt(0.975, n - 1): NaNs produced

## Warning in qt(0.975, n - 1): NaNs produced

## Warning in qt(0.975, n - 1): NaNs produced

## Warning in qt(0.975, n - 1): NaNs produced

## Warning in qt(0.975, n - 1): NaNs produced

## Warning in qt(0.975, n - 1): NaNs produced

## Warning in qt(0.975, n - 1): NaNs produced

## Warning in qt(0.975, n - 1): NaNs produced

## Warning in qt(0.975, n - 1): NaNs produced

## Warning in qt(0.975, n - 1): NaNs produced

## Warning in qt(0.975, n - 1): NaNs produced

## Warning in qt(0.975, n - 1): NaNs produced

## Warning in qt(0.975, n - 1): NaNs produced

## Warning in qt(0.975, n - 1): NaNs produced

## Warning in qt(0.975, n - 1): NaNs produced

## Warning in qt(0.975, n - 1): NaNs produced

## Warning in qt(0.975, n - 1): NaNs produced

## Warning in qt(0.975, n - 1): NaNs produced

## Warning in qt(0.975, n - 1): NaNs produced

## Warning in qt(0.975, n - 1): NaNs produced

## Warning in qt(0.975, n - 1): NaNs produced

## Warning in qt(0.975, n - 1): NaNs produced

## Warning in qt(0.975, n - 1): NaNs produced

## Warning in qt(0.975, n - 1): NaNs produced

## Warning in qt(0.975, n - 1): NaNs produced

## Warning in qt(0.975, n - 1): NaNs produced

## Warning in qt(0.975, n - 1): NaNs produced

## Warning in qt(0.975, n - 1): NaNs produced

## Warning in qt(0.975, n - 1): NaNs produced

## Warning in qt(0.975, n - 1): NaNs produced

## Warning in qt(0.975, n - 1): NaNs produced

## Warning in qt(0.975, n - 1): NaNs produced

## Warning in qt(0.975, n - 1): NaNs produced

## Warning in qt(0.975, n - 1): NaNs produced

## Warning in qt(0.975, n - 1): NaNs produced

## Warning in qt(0.975, n - 1): NaNs produced

## Warning in qt(0.975, n - 1): NaNs produced

## Warning in qt(0.975, n - 1): NaNs produced

## Warning in qt(0.975, n - 1): NaNs produced

## Warning in qt(0.975, n - 1): NaNs produced

## Warning in qt(0.975, n - 1): NaNs produced

## Warning in qt(0.975, n - 1): NaNs produced

## Warning in qt(0.975, n - 1): NaNs produced

## Warning in qt(0.975, n - 1): NaNs produced

## Warning in qt(0.975, n - 1): NaNs produced

## Warning in qt(0.975, n - 1): NaNs produced

## Warning in qt(0.975, n - 1): NaNs produced

## Warning in qt(0.975, n - 1): NaNs produced

## Warning in qt(0.975, n - 1): NaNs produced

## Warning in qt(0.975, n - 1): NaNs produced

## Warning in qt(0.975, n - 1): NaNs produced

## Warning in qt(0.975, n - 1): NaNs produced

## Warning in qt(0.975, n - 1): NaNs produced

## Warning in qt(0.975, n - 1): NaNs produced

## Warning in qt(0.975, n - 1): NaNs produced

## Warning in qt(0.975, n - 1): NaNs produced

## Warning in qt(0.975, n - 1): NaNs produced

## Warning in qt(0.975, n - 1): NaNs produced

## Warning in qt(0.975, n - 1): NaNs produced

## Warning in qt(0.975, n - 1): NaNs produced

## Warning in qt(0.975, n - 1): NaNs produced

## Warning in qt(0.975, n - 1): NaNs produced

## Warning in qt(0.975, n - 1): NaNs produced

## Warning in qt(0.975, n - 1): NaNs produced

## Warning in qt(0.975, n - 1): NaNs produced

## Warning in qt(0.975, n - 1): NaNs produced

## Warning in qt(0.975, n - 1): NaNs produced

## Warning in qt(0.975, n - 1): NaNs produced

## Warning in qt(0.975, n - 1): NaNs produced

## Warning in qt(0.975, n - 1): NaNs produced

## Warning in qt(0.975, n - 1): NaNs produced

## Warning in qt(0.975, n - 1): NaNs produced

## Warning in qt(0.975, n - 1): NaNs produced

## Warning in qt(0.975, n - 1): NaNs produced

## Warning in qt(0.975, n - 1): NaNs produced

## Warning in qt(0.975, n - 1): NaNs produced

## Warning in qt(0.975, n - 1): NaNs produced

## Warning in qt(0.975, n - 1): NaNs produced

## Warning in qt(0.975, n - 1): NaNs produced

## Warning in qt(0.975, n - 1): NaNs produced

## Warning in qt(0.975, n - 1): NaNs produced

## Warning in qt(0.975, n - 1): NaNs produced

## Warning in qt(0.975, n - 1): NaNs produced

## Warning in qt(0.975, n - 1): NaNs produced

## Warning in qt(0.975, n - 1): NaNs produced

## Warning in qt(0.975, n - 1): NaNs produced

## Warning in qt(0.975, n - 1): NaNs produced

## Warning in qt(0.975, n - 1): NaNs produced

## Warning in qt(0.975, n - 1): NaNs produced

## Warning in qt(0.975, n - 1): NaNs produced

## Warning in qt(0.975, n - 1): NaNs produced

## Warning in qt(0.975, n - 1): NaNs produced

## Warning in qt(0.975, n - 1): NaNs produced

## Warning in qt(0.975, n - 1): NaNs produced

## Warning in qt(0.975, n - 1): NaNs produced

## Warning in qt(0.975, n - 1): NaNs produced

## Warning in qt(0.975, n - 1): NaNs produced

## Warning in qt(0.975, n - 1): NaNs produced

## Warning in qt(0.975, n - 1): NaNs produced

## Warning in qt(0.975, n - 1): NaNs produced

## Warning in qt(0.975, n - 1): NaNs produced

## Warning in qt(0.975, n - 1): NaNs produced

## Warning in qt(0.975, n - 1): NaNs produced

## Warning in qt(0.975, n - 1): NaNs produced

## Warning in qt(0.975, n - 1): NaNs produced

## Warning in qt(0.975, n - 1): NaNs produced

## Warning in qt(0.975, n - 1): NaNs produced

## Warning in qt(0.975, n - 1): NaNs produced
```

### Summary of data available {.active}

```
## Warning: The input is a data frame, convert it to the matrix.
```

### Functions to annotate mutation heatmaps

## Plotting

### Transcriptomics

- receptors\_expression\_initial\_line, echo=FALSE, message=FALSE

- selection\_transcriptomics, fig.height=10, echo=FALSE, message=FALSE

| Hugo\_Symbol | CHP212 | N206 | BE2C | CHLA20 | GIMEN | IMR32 | KAN | KELLY | LAN1 | LAN6 | NBEBC1 | NGP | SHEP | SKNAS | SKNSH |
| --- | --- | --- | --- | --- | --- | --- | --- | --- | --- | --- | --- | --- | --- | --- | --- |
| IRS1 | 5.9647247 | 0.7830493 | 2.063062 | 0.6593744 | 7.392069 | 4.145315 | 1.277577 | 1.284026 | 3.449798 | 0.945306 | 0.7837948 | 0.4872439 | 18.839530 | 0.8797608 | 7.674899 |
| GRB2 | 57.1883679 | 28.0846935 | 16.422871 | 34.5556680 | 36.588937 | 34.968902 | 24.381746 | 29.152105 | 47.650442 | 38.349975 | 31.2493178 | 25.7266498 | 38.376339 | 22.7042976 | 31.408764 |
| IRS2 | 0.2104088 | 2.5945039 | 3.123996 | 2.0637034 | 10.499105 | 1.200465 | 8.961400 | 1.729890 | 2.299956 | 10.401861 | 14.3163498 | 6.9526073 | 1.710928 | 1.8288034 | 20.934451 |

### Mutations {.tabset} #### Quality control Most mutated genes

 Check on initially perturbed lines are here.

```
## Warning: Using default `alter_fun` graphics and reset `col`.
```

#### Paper figures

WES oncoplot on signalling genes by type or classification of mutation

 Ackerman classification, exact mutations

### IC50 plots

#### IC50 with cell name

```
## Warning: Removed 1 rows containing missing values (geom_text).
```

```
## Joining, by = "Cell_line"
## Joining, by = "Cell_line"
```

```
## Warning: Ignoring unknown aesthetics: label
```

```
## Warning: Width not defined. Set with `position_dodge(width = ?)`
```

```
## Warning: Removed 1 rows containing missing values (geom_point).
```

```
## Warning: Removed 2 rows containing missing values (geom_point).
```

```
## Warning: Removed 2 rows containing missing values (geom_text).
```

#### IC50 vs selected genes expression

```
## Joining, by = "Cell_line"
## Joining, by = "Cell_line"
```

#### IC50 vs mutations

```
## Warning: `funs()` is deprecated as of dplyr 0.8.0.
## Please use a list of either functions or lambdas: 
## 
##   # Simple named list: 
##   list(mean = mean, median = median)
## 
##   # Auto named with `tibble::lst()`: 
##   tibble::lst(mean, median)
## 
##   # Using lambdas
##   list(~ mean(., trim = .2), ~ median(., na.rm = TRUE))
## This warning is displayed once every 8 hours.
## Call `lifecycle::last_warnings()` to see where this warning was generated.
```

```
## Joining, by = "Cell_line"
## Joining, by = "Cell_line"
```

```
## `geom_smooth()` using formula 'y ~ x'
```

```
## Joining, by = "Cell_line"
```

```
## `geom_smooth()` using formula 'y ~ x'
```

```
## Joining, by = "Cell_line"
```

```
## `geom_smooth()` using formula 'y ~ x'
```

```
## Joining, by = "Cell_line"
```

```
## `geom_smooth()` using method = 'loess' and formula 'y ~ x'
```

```
## Warning in simpleLoess(y, x, w, span, degree = degree, parametric = parametric, : at -0.005
```

```
## Warning in simpleLoess(y, x, w, span, degree = degree, parametric = parametric, : radius 2.5e-05
```

```
## Warning in simpleLoess(y, x, w, span, degree = degree, parametric = parametric, : all data on boundary of neighborhood. make span bigger
```

```
## Warning in simpleLoess(y, x, w, span, degree = degree, parametric = parametric, : pseudoinverse used at -0.005
```

```
## Warning in simpleLoess(y, x, w, span, degree = degree, parametric = parametric, : neighborhood radius 0.005
```

```
## Warning in simpleLoess(y, x, w, span, degree = degree, parametric = parametric, : reciprocal condition number 1
```

```
## Warning in simpleLoess(y, x, w, span, degree = degree, parametric = parametric, : There are other near singularities as well. 1.01
```

```
## Warning in simpleLoess(y, x, w, span, degree = degree, parametric = parametric, : zero-width neighborhood. make span bigger
```

```
## Warning: Computation failed in `stat_smooth()`:
## NA/NaN/Inf in foreign function call (arg 5)
```

```
## Warning in simpleLoess(y, x, w, span, degree = degree, parametric = parametric, : pseudoinverse used at -0.005
```

```
## Warning in simpleLoess(y, x, w, span, degree = degree, parametric = parametric, : neighborhood radius 1.005
```

```
## Warning in simpleLoess(y, x, w, span, degree = degree, parametric = parametric, : reciprocal condition number 0
```

```
## Warning in simpleLoess(y, x, w, span, degree = degree, parametric = parametric, : There are other near singularities as well. 1.01
```

```
## Warning in predLoess(object$y, object$x, newx = if (is.null(newdata)) object$x else if (is.data.frame(newdata)) as.matrix(model.frame(delete.response(terms(object)), : pseudoinverse used at -0.005
```

```
## Warning in predLoess(object$y, object$x, newx = if (is.null(newdata)) object$x else if (is.data.frame(newdata)) as.matrix(model.frame(delete.response(terms(object)), : neighborhood radius 1.005
```

```
## Warning in predLoess(object$y, object$x, newx = if (is.null(newdata)) object$x else if (is.data.frame(newdata)) as.matrix(model.frame(delete.response(terms(object)), : reciprocal condition number 0
```

```
## Warning in predLoess(object$y, object$x, newx = if (is.null(newdata)) object$x else if (is.data.frame(newdata)) as.matrix(model.frame(delete.response(terms(object)), : There are other near
## singularities as well. 1.01
```

```
## Warning in simpleLoess(y, x, w, span, degree = degree, parametric = parametric, : at -0.005
```

```
## Warning in simpleLoess(y, x, w, span, degree = degree, parametric = parametric, : radius 2.5e-05
```

```
## Warning in simpleLoess(y, x, w, span, degree = degree, parametric = parametric, : all data on boundary of neighborhood. make span bigger
```

```
## Warning in simpleLoess(y, x, w, span, degree = degree, parametric = parametric, : pseudoinverse used at -0.005
```

```
## Warning in simpleLoess(y, x, w, span, degree = degree, parametric = parametric, : neighborhood radius 0.005
```

```
## Warning in simpleLoess(y, x, w, span, degree = degree, parametric = parametric, : reciprocal condition number 1
```

```
## Warning in simpleLoess(y, x, w, span, degree = degree, parametric = parametric, : There are other near singularities as well. 1.01
```

```
## Warning in simpleLoess(y, x, w, span, degree = degree, parametric = parametric, : zero-width neighborhood. make span bigger
```

```
## Warning: Computation failed in `stat_smooth()`:
## NA/NaN/Inf in foreign function call (arg 5)
```

```
## Warning in simpleLoess(y, x, w, span, degree = degree, parametric = parametric, : at -0.005
```

```
## Warning in simpleLoess(y, x, w, span, degree = degree, parametric = parametric, : radius 2.5e-05
```

```
## Warning in simpleLoess(y, x, w, span, degree = degree, parametric = parametric, : all data on boundary of neighborhood. make span bigger
```

```
## Warning in simpleLoess(y, x, w, span, degree = degree, parametric = parametric, : pseudoinverse used at -0.005
```

```
## Warning in simpleLoess(y, x, w, span, degree = degree, parametric = parametric, : neighborhood radius 0.005
```

```
## Warning in simpleLoess(y, x, w, span, degree = degree, parametric = parametric, : reciprocal condition number 1
```

```
## Warning in simpleLoess(y, x, w, span, degree = degree, parametric = parametric, : There are other near singularities as well. 1.01
```

```
## Warning in simpleLoess(y, x, w, span, degree = degree, parametric = parametric, : zero-width neighborhood. make span bigger
```

```
## Warning: Computation failed in `stat_smooth()`:
## NA/NaN/Inf in foreign function call (arg 5)
```

```
## Warning in simpleLoess(y, x, w, span, degree = degree, parametric = parametric, : at -0.005
```

```
## Warning in simpleLoess(y, x, w, span, degree = degree, parametric = parametric, : radius 2.5e-05
```

```
## Warning in simpleLoess(y, x, w, span, degree = degree, parametric = parametric, : all data on boundary of neighborhood. make span bigger
```

```
## Warning in simpleLoess(y, x, w, span, degree = degree, parametric = parametric, : pseudoinverse used at -0.005
```

```
## Warning in simpleLoess(y, x, w, span, degree = degree, parametric = parametric, : neighborhood radius 0.005
```

```
## Warning in simpleLoess(y, x, w, span, degree = degree, parametric = parametric, : reciprocal condition number 1
```

```
## Warning in simpleLoess(y, x, w, span, degree = degree, parametric = parametric, : There are other near singularities as well. 1.01
```

```
## Warning in simpleLoess(y, x, w, span, degree = degree, parametric = parametric, : zero-width neighborhood. make span bigger
```

```
## Warning: Computation failed in `stat_smooth()`:
## NA/NaN/Inf in foreign function call (arg 5)
```

```
## Warning in simpleLoess(y, x, w, span, degree = degree, parametric = parametric, : at -0.005
```

```
## Warning in simpleLoess(y, x, w, span, degree = degree, parametric = parametric, : radius 2.5e-05
```

```
## Warning in simpleLoess(y, x, w, span, degree = degree, parametric = parametric, : all data on boundary of neighborhood. make span bigger
```

```
## Warning in simpleLoess(y, x, w, span, degree = degree, parametric = parametric, : pseudoinverse used at -0.005
```

```
## Warning in simpleLoess(y, x, w, span, degree = degree, parametric = parametric, : neighborhood radius 0.005
```

```
## Warning in simpleLoess(y, x, w, span, degree = degree, parametric = parametric, : reciprocal condition number 1
```

```
## Warning in simpleLoess(y, x, w, span, degree = degree, parametric = parametric, : There are other near singularities as well. 1.01
```

```
## Warning in simpleLoess(y, x, w, span, degree = degree, parametric = parametric, : zero-width neighborhood. make span bigger
```

```
## Warning: Computation failed in `stat_smooth()`:
## NA/NaN/Inf in foreign function call (arg 5)
```

```
## Warning in simpleLoess(y, x, w, span, degree = degree, parametric = parametric, : at -0.005
```

```
## Warning in simpleLoess(y, x, w, span, degree = degree, parametric = parametric, : radius 2.5e-05
```

```
## Warning in simpleLoess(y, x, w, span, degree = degree, parametric = parametric, : all data on boundary of neighborhood. make span bigger
```

```
## Warning in simpleLoess(y, x, w, span, degree = degree, parametric = parametric, : pseudoinverse used at -0.005
```

```
## Warning in simpleLoess(y, x, w, span, degree = degree, parametric = parametric, : neighborhood radius 0.005
```

```
## Warning in simpleLoess(y, x, w, span, degree = degree, parametric = parametric, : reciprocal condition number 1
```

```
## Warning in simpleLoess(y, x, w, span, degree = degree, parametric = parametric, : There are other near singularities as well. 1.01
```

```
## Warning in simpleLoess(y, x, w, span, degree = degree, parametric = parametric, : zero-width neighborhood. make span bigger
```

```
## Warning: Computation failed in `stat_smooth()`:
## NA/NaN/Inf in foreign function call (arg 5)
```

```
## Warning in simpleLoess(y, x, w, span, degree = degree, parametric = parametric, : at -0.005
```

```
## Warning in simpleLoess(y, x, w, span, degree = degree, parametric = parametric, : radius 2.5e-05
```

```
## Warning in simpleLoess(y, x, w, span, degree = degree, parametric = parametric, : all data on boundary of neighborhood. make span bigger
```

```
## Warning in simpleLoess(y, x, w, span, degree = degree, parametric = parametric, : pseudoinverse used at -0.005
```

```
## Warning in simpleLoess(y, x, w, span, degree = degree, parametric = parametric, : neighborhood radius 0.005
```

```
## Warning in simpleLoess(y, x, w, span, degree = degree, parametric = parametric, : reciprocal condition number 1
```

```
## Warning in simpleLoess(y, x, w, span, degree = degree, parametric = parametric, : There are other near singularities as well. 1.01
```

```
## Warning in simpleLoess(y, x, w, span, degree = degree, parametric = parametric, : zero-width neighborhood. make span bigger
```

```
## Warning: Computation failed in `stat_smooth()`:
## NA/NaN/Inf in foreign function call (arg 5)
```

```
## Warning in simpleLoess(y, x, w, span, degree = degree, parametric = parametric, : at -0.005
```

```
## Warning in simpleLoess(y, x, w, span, degree = degree, parametric = parametric, : radius 2.5e-05
```

```
## Warning in simpleLoess(y, x, w, span, degree = degree, parametric = parametric, : all data on boundary of neighborhood. make span bigger
```

```
## Warning in simpleLoess(y, x, w, span, degree = degree, parametric = parametric, : pseudoinverse used at -0.005
```

```
## Warning in simpleLoess(y, x, w, span, degree = degree, parametric = parametric, : neighborhood radius 0.005
```

```
## Warning in simpleLoess(y, x, w, span, degree = degree, parametric = parametric, : reciprocal condition number 1
```

```
## Warning in simpleLoess(y, x, w, span, degree = degree, parametric = parametric, : There are other near singularities as well. 1.01
```

```
## Warning in simpleLoess(y, x, w, span, degree = degree, parametric = parametric, : zero-width neighborhood. make span bigger
```

```
## Warning: Computation failed in `stat_smooth()`:
## NA/NaN/Inf in foreign function call (arg 5)
```

```
## Warning in simpleLoess(y, x, w, span, degree = degree, parametric = parametric, : at -0.005
```

```
## Warning in simpleLoess(y, x, w, span, degree = degree, parametric = parametric, : radius 2.5e-05
```

```
## Warning in simpleLoess(y, x, w, span, degree = degree, parametric = parametric, : all data on boundary of neighborhood. make span bigger
```

```
## Warning in simpleLoess(y, x, w, span, degree = degree, parametric = parametric, : pseudoinverse used at -0.005
```

```
## Warning in simpleLoess(y, x, w, span, degree = degree, parametric = parametric, : neighborhood radius 0.005
```

```
## Warning in simpleLoess(y, x, w, span, degree = degree, parametric = parametric, : reciprocal condition number 1
```

```
## Warning in simpleLoess(y, x, w, span, degree = degree, parametric = parametric, : There are other near singularities as well. 1.01
```

```
## Warning in simpleLoess(y, x, w, span, degree = degree, parametric = parametric, : zero-width neighborhood. make span bigger
```

```
## Warning: Computation failed in `stat_smooth()`:
## NA/NaN/Inf in foreign function call (arg 5)
```

```
## Warning in simpleLoess(y, x, w, span, degree = degree, parametric = parametric, : at -0.005
```

```
## Warning in simpleLoess(y, x, w, span, degree = degree, parametric = parametric, : radius 2.5e-05
```

```
## Warning in simpleLoess(y, x, w, span, degree = degree, parametric = parametric, : all data on boundary of neighborhood. make span bigger
```

```
## Warning in simpleLoess(y, x, w, span, degree = degree, parametric = parametric, : pseudoinverse used at -0.005
```

```
## Warning in simpleLoess(y, x, w, span, degree = degree, parametric = parametric, : neighborhood radius 0.005
```

```
## Warning in simpleLoess(y, x, w, span, degree = degree, parametric = parametric, : reciprocal condition number 1
```

```
## Warning in simpleLoess(y, x, w, span, degree = degree, parametric = parametric, : There are other near singularities as well. 1.01
```

```
## Warning in simpleLoess(y, x, w, span, degree = degree, parametric = parametric, : zero-width neighborhood. make span bigger
```

```
## Warning: Computation failed in `stat_smooth()`:
## NA/NaN/Inf in foreign function call (arg 5)
```

```
## Warning in simpleLoess(y, x, w, span, degree = degree, parametric = parametric, : at -0.005
```

```
## Warning in simpleLoess(y, x, w, span, degree = degree, parametric = parametric, : radius 2.5e-05
```

```
## Warning in simpleLoess(y, x, w, span, degree = degree, parametric = parametric, : all data on boundary of neighborhood. make span bigger
```

```
## Warning in simpleLoess(y, x, w, span, degree = degree, parametric = parametric, : pseudoinverse used at -0.005
```

```
## Warning in simpleLoess(y, x, w, span, degree = degree, parametric = parametric, : neighborhood radius 0.005
```

```
## Warning in simpleLoess(y, x, w, span, degree = degree, parametric = parametric, : reciprocal condition number 1
```

```
## Warning in simpleLoess(y, x, w, span, degree = degree, parametric = parametric, : There are other near singularities as well. 1.01
```

```
## Warning in simpleLoess(y, x, w, span, degree = degree, parametric = parametric, : zero-width neighborhood. make span bigger
```

```
## Warning: Computation failed in `stat_smooth()`:
## NA/NaN/Inf in foreign function call (arg 5)
```

```
## Warning in simpleLoess(y, x, w, span, degree = degree, parametric = parametric, : pseudoinverse used at -0.005
```

```
## Warning in simpleLoess(y, x, w, span, degree = degree, parametric = parametric, : neighborhood radius 1.005
```

```
## Warning in simpleLoess(y, x, w, span, degree = degree, parametric = parametric, : reciprocal condition number 0
```

```
## Warning in simpleLoess(y, x, w, span, degree = degree, parametric = parametric, : There are other near singularities as well. 1.01
```

```
## Warning in predLoess(object$y, object$x, newx = if (is.null(newdata)) object$x else if (is.data.frame(newdata)) as.matrix(model.frame(delete.response(terms(object)), : pseudoinverse used at -0.005
```

```
## Warning in predLoess(object$y, object$x, newx = if (is.null(newdata)) object$x else if (is.data.frame(newdata)) as.matrix(model.frame(delete.response(terms(object)), : neighborhood radius 1.005
```

```
## Warning in predLoess(object$y, object$x, newx = if (is.null(newdata)) object$x else if (is.data.frame(newdata)) as.matrix(model.frame(delete.response(terms(object)), : reciprocal condition number 0
```

```
## Warning in predLoess(object$y, object$x, newx = if (is.null(newdata)) object$x else if (is.data.frame(newdata)) as.matrix(model.frame(delete.response(terms(object)), : There are other near
## singularities as well. 1.01
```

```
## Warning in simpleLoess(y, x, w, span, degree = degree, parametric = parametric, : at -0.005
```

```
## Warning in simpleLoess(y, x, w, span, degree = degree, parametric = parametric, : radius 2.5e-05
```

```
## Warning in simpleLoess(y, x, w, span, degree = degree, parametric = parametric, : all data on boundary of neighborhood. make span bigger
```

```
## Warning in simpleLoess(y, x, w, span, degree = degree, parametric = parametric, : pseudoinverse used at -0.005
```

```
## Warning in simpleLoess(y, x, w, span, degree = degree, parametric = parametric, : neighborhood radius 0.005
```

```
## Warning in simpleLoess(y, x, w, span, degree = degree, parametric = parametric, : reciprocal condition number 1
```

```
## Warning in simpleLoess(y, x, w, span, degree = degree, parametric = parametric, : There are other near singularities as well. 1.01
```

```
## Warning in simpleLoess(y, x, w, span, degree = degree, parametric = parametric, : zero-width neighborhood. make span bigger
```

```
## Warning: Computation failed in `stat_smooth()`:
## NA/NaN/Inf in foreign function call (arg 5)
```

```
## Warning in simpleLoess(y, x, w, span, degree = degree, parametric = parametric, : at -0.005
```

```
## Warning in simpleLoess(y, x, w, span, degree = degree, parametric = parametric, : radius 2.5e-05
```

```
## Warning in simpleLoess(y, x, w, span, degree = degree, parametric = parametric, : all data on boundary of neighborhood. make span bigger
```

```
## Warning in simpleLoess(y, x, w, span, degree = degree, parametric = parametric, : pseudoinverse used at -0.005
```

```
## Warning in simpleLoess(y, x, w, span, degree = degree, parametric = parametric, : neighborhood radius 0.005
```

```
## Warning in simpleLoess(y, x, w, span, degree = degree, parametric = parametric, : reciprocal condition number 1
```

```
## Warning in simpleLoess(y, x, w, span, degree = degree, parametric = parametric, : There are other near singularities as well. 1.01
```

```
## Warning in simpleLoess(y, x, w, span, degree = degree, parametric = parametric, : zero-width neighborhood. make span bigger
```

```
## Warning: Computation failed in `stat_smooth()`:
## NA/NaN/Inf in foreign function call (arg 5)
```

```
## Warning in simpleLoess(y, x, w, span, degree = degree, parametric = parametric, : at -0.005
```

```
## Warning in simpleLoess(y, x, w, span, degree = degree, parametric = parametric, : radius 2.5e-05
```

```
## Warning in simpleLoess(y, x, w, span, degree = degree, parametric = parametric, : all data on boundary of neighborhood. make span bigger
```

```
## Warning in simpleLoess(y, x, w, span, degree = degree, parametric = parametric, : pseudoinverse used at -0.005
```

```
## Warning in simpleLoess(y, x, w, span, degree = degree, parametric = parametric, : neighborhood radius 0.005
```

```
## Warning in simpleLoess(y, x, w, span, degree = degree, parametric = parametric, : reciprocal condition number 1
```

```
## Warning in simpleLoess(y, x, w, span, degree = degree, parametric = parametric, : There are other near singularities as well. 1.01
```

```
## Warning in simpleLoess(y, x, w, span, degree = degree, parametric = parametric, : zero-width neighborhood. make span bigger
```

```
## Warning: Computation failed in `stat_smooth()`:
## NA/NaN/Inf in foreign function call (arg 5)
```

```
## Warning in simpleLoess(y, x, w, span, degree = degree, parametric = parametric, : at -0.005
```

```
## Warning in simpleLoess(y, x, w, span, degree = degree, parametric = parametric, : radius 2.5e-05
```

```
## Warning in simpleLoess(y, x, w, span, degree = degree, parametric = parametric, : all data on boundary of neighborhood. make span bigger
```

```
## Warning in simpleLoess(y, x, w, span, degree = degree, parametric = parametric, : pseudoinverse used at -0.005
```

```
## Warning in simpleLoess(y, x, w, span, degree = degree, parametric = parametric, : neighborhood radius 0.005
```

```
## Warning in simpleLoess(y, x, w, span, degree = degree, parametric = parametric, : reciprocal condition number 1
```

```
## Warning in simpleLoess(y, x, w, span, degree = degree, parametric = parametric, : There are other near singularities as well. 1.01
```

```
## Warning in simpleLoess(y, x, w, span, degree = degree, parametric = parametric, : zero-width neighborhood. make span bigger
```

```
## Warning: Computation failed in `stat_smooth()`:
## NA/NaN/Inf in foreign function call (arg 5)
```

```
## Warning in simpleLoess(y, x, w, span, degree = degree, parametric = parametric, : at -0.005
```

```
## Warning in simpleLoess(y, x, w, span, degree = degree, parametric = parametric, : radius 2.5e-05
```

```
## Warning in simpleLoess(y, x, w, span, degree = degree, parametric = parametric, : all data on boundary of neighborhood. make span bigger
```

```
## Warning in simpleLoess(y, x, w, span, degree = degree, parametric = parametric, : pseudoinverse used at -0.005
```

```
## Warning in simpleLoess(y, x, w, span, degree = degree, parametric = parametric, : neighborhood radius 0.005
```

```
## Warning in simpleLoess(y, x, w, span, degree = degree, parametric = parametric, : reciprocal condition number 1
```

```
## Warning in simpleLoess(y, x, w, span, degree = degree, parametric = parametric, : There are other near singularities as well. 1.01
```

```
## Warning in simpleLoess(y, x, w, span, degree = degree, parametric = parametric, : zero-width neighborhood. make span bigger
```

```
## Warning: Computation failed in `stat_smooth()`:
## NA/NaN/Inf in foreign function call (arg 5)
```

```
## Warning in simpleLoess(y, x, w, span, degree = degree, parametric = parametric, : at -0.005
```

```
## Warning in simpleLoess(y, x, w, span, degree = degree, parametric = parametric, : radius 2.5e-05
```

```
## Warning in simpleLoess(y, x, w, span, degree = degree, parametric = parametric, : all data on boundary of neighborhood. make span bigger
```

```
## Warning in simpleLoess(y, x, w, span, degree = degree, parametric = parametric, : pseudoinverse used at -0.005
```

```
## Warning in simpleLoess(y, x, w, span, degree = degree, parametric = parametric, : neighborhood radius 0.005
```

```
## Warning in simpleLoess(y, x, w, span, degree = degree, parametric = parametric, : reciprocal condition number 1
```

```
## Warning in simpleLoess(y, x, w, span, degree = degree, parametric = parametric, : There are other near singularities as well. 1.01
```

```
## Warning in simpleLoess(y, x, w, span, degree = degree, parametric = parametric, : zero-width neighborhood. make span bigger
```

```
## Warning: Computation failed in `stat_smooth()`:
## NA/NaN/Inf in foreign function call (arg 5)
```

```
## Warning in simpleLoess(y, x, w, span, degree = degree, parametric = parametric, : at -0.005
```

```
## Warning in simpleLoess(y, x, w, span, degree = degree, parametric = parametric, : radius 2.5e-05
```

```
## Warning in simpleLoess(y, x, w, span, degree = degree, parametric = parametric, : all data on boundary of neighborhood. make span bigger
```

```
## Warning in simpleLoess(y, x, w, span, degree = degree, parametric = parametric, : pseudoinverse used at -0.005
```

```
## Warning in simpleLoess(y, x, w, span, degree = degree, parametric = parametric, : neighborhood radius 0.005
```

```
## Warning in simpleLoess(y, x, w, span, degree = degree, parametric = parametric, : reciprocal condition number 1
```

```
## Warning in simpleLoess(y, x, w, span, degree = degree, parametric = parametric, : There are other near singularities as well. 1.01
```

```
## Warning in simpleLoess(y, x, w, span, degree = degree, parametric = parametric, : zero-width neighborhood. make span bigger
```

```
## Warning: Computation failed in `stat_smooth()`:
## NA/NaN/Inf in foreign function call (arg 5)
```

```
## Warning in simpleLoess(y, x, w, span, degree = degree, parametric = parametric, : at -0.005
```

```
## Warning in simpleLoess(y, x, w, span, degree = degree, parametric = parametric, : radius 2.5e-05
```

```
## Warning in simpleLoess(y, x, w, span, degree = degree, parametric = parametric, : all data on boundary of neighborhood. make span bigger
```

```
## Warning in simpleLoess(y, x, w, span, degree = degree, parametric = parametric, : pseudoinverse used at -0.005
```

```
## Warning in simpleLoess(y, x, w, span, degree = degree, parametric = parametric, : neighborhood radius 0.005
```

```
## Warning in simpleLoess(y, x, w, span, degree = degree, parametric = parametric, : reciprocal condition number 1
```

```
## Warning in simpleLoess(y, x, w, span, degree = degree, parametric = parametric, : There are other near singularities as well. 1.01
```

```
## Warning in simpleLoess(y, x, w, span, degree = degree, parametric = parametric, : zero-width neighborhood. make span bigger
```

```
## Warning: Computation failed in `stat_smooth()`:
## NA/NaN/Inf in foreign function call (arg 5)
```

```
## Warning in simpleLoess(y, x, w, span, degree = degree, parametric = parametric, : at -0.005
```

```
## Warning in simpleLoess(y, x, w, span, degree = degree, parametric = parametric, : radius 2.5e-05
```

```
## Warning in simpleLoess(y, x, w, span, degree = degree, parametric = parametric, : all data on boundary of neighborhood. make span bigger
```

```
## Warning in simpleLoess(y, x, w, span, degree = degree, parametric = parametric, : pseudoinverse used at -0.005
```

```
## Warning in simpleLoess(y, x, w, span, degree = degree, parametric = parametric, : neighborhood radius 0.005
```

```
## Warning in simpleLoess(y, x, w, span, degree = degree, parametric = parametric, : reciprocal condition number 1
```

```
## Warning in simpleLoess(y, x, w, span, degree = degree, parametric = parametric, : There are other near singularities as well. 1.01
```

```
## Warning in simpleLoess(y, x, w, span, degree = degree, parametric = parametric, : zero-width neighborhood. make span bigger
```

```
## Warning: Computation failed in `stat_smooth()`:
## NA/NaN/Inf in foreign function call (arg 5)
```

```
## Warning in simpleLoess(y, x, w, span, degree = degree, parametric = parametric, : at -0.005
```

```
## Warning in simpleLoess(y, x, w, span, degree = degree, parametric = parametric, : radius 2.5e-05
```

```
## Warning in simpleLoess(y, x, w, span, degree = degree, parametric = parametric, : all data on boundary of neighborhood. make span bigger
```

```
## Warning in simpleLoess(y, x, w, span, degree = degree, parametric = parametric, : pseudoinverse used at -0.005
```

```
## Warning in simpleLoess(y, x, w, span, degree = degree, parametric = parametric, : neighborhood radius 0.005
```

```
## Warning in simpleLoess(y, x, w, span, degree = degree, parametric = parametric, : reciprocal condition number 1
```

```
## Warning in simpleLoess(y, x, w, span, degree = degree, parametric = parametric, : There are other near singularities as well. 1.01
```

```
## Warning in simpleLoess(y, x, w, span, degree = degree, parametric = parametric, : zero-width neighborhood. make span bigger
```

```
## Warning: Computation failed in `stat_smooth()`:
## NA/NaN/Inf in foreign function call (arg 5)
```

```
## Warning in simpleLoess(y, x, w, span, degree = degree, parametric = parametric, : pseudoinverse used at -0.005
```

```
## Warning in simpleLoess(y, x, w, span, degree = degree, parametric = parametric, : neighborhood radius 1.005
```

```
## Warning in simpleLoess(y, x, w, span, degree = degree, parametric = parametric, : reciprocal condition number 0
```

```
## Warning in simpleLoess(y, x, w, span, degree = degree, parametric = parametric, : There are other near singularities as well. 1.01
```

```
## Warning in predLoess(object$y, object$x, newx = if (is.null(newdata)) object$x else if (is.data.frame(newdata)) as.matrix(model.frame(delete.response(terms(object)), : pseudoinverse used at -0.005
```

```
## Warning in predLoess(object$y, object$x, newx = if (is.null(newdata)) object$x else if (is.data.frame(newdata)) as.matrix(model.frame(delete.response(terms(object)), : neighborhood radius 1.005
```

```
## Warning in predLoess(object$y, object$x, newx = if (is.null(newdata)) object$x else if (is.data.frame(newdata)) as.matrix(model.frame(delete.response(terms(object)), : reciprocal condition number 0
```

```
## Warning in predLoess(object$y, object$x, newx = if (is.null(newdata)) object$x else if (is.data.frame(newdata)) as.matrix(model.frame(delete.response(terms(object)), : There are other near
## singularities as well. 1.01
```

```
## Warning in simpleLoess(y, x, w, span, degree = degree, parametric = parametric, : at -0.005
```

```
## Warning in simpleLoess(y, x, w, span, degree = degree, parametric = parametric, : radius 2.5e-05
```

```
## Warning in simpleLoess(y, x, w, span, degree = degree, parametric = parametric, : all data on boundary of neighborhood. make span bigger
```

```
## Warning in simpleLoess(y, x, w, span, degree = degree, parametric = parametric, : pseudoinverse used at -0.005
```

```
## Warning in simpleLoess(y, x, w, span, degree = degree, parametric = parametric, : neighborhood radius 0.005
```

```
## Warning in simpleLoess(y, x, w, span, degree = degree, parametric = parametric, : reciprocal condition number 1
```

```
## Warning in simpleLoess(y, x, w, span, degree = degree, parametric = parametric, : There are other near singularities as well. 1.01
```

```
## Warning in simpleLoess(y, x, w, span, degree = degree, parametric = parametric, : zero-width neighborhood. make span bigger
```

```
## Warning: Computation failed in `stat_smooth()`:
## NA/NaN/Inf in foreign function call (arg 5)
```

```
## Warning in simpleLoess(y, x, w, span, degree = degree, parametric = parametric, : at -0.005
```

```
## Warning in simpleLoess(y, x, w, span, degree = degree, parametric = parametric, : radius 2.5e-05
```

```
## Warning in simpleLoess(y, x, w, span, degree = degree, parametric = parametric, : all data on boundary of neighborhood. make span bigger
```

```
## Warning in simpleLoess(y, x, w, span, degree = degree, parametric = parametric, : pseudoinverse used at -0.005
```

```
## Warning in simpleLoess(y, x, w, span, degree = degree, parametric = parametric, : neighborhood radius 0.005
```

```
## Warning in simpleLoess(y, x, w, span, degree = degree, parametric = parametric, : reciprocal condition number 1
```

```
## Warning in simpleLoess(y, x, w, span, degree = degree, parametric = parametric, : There are other near singularities as well. 1.01
```

```
## Warning in simpleLoess(y, x, w, span, degree = degree, parametric = parametric, : zero-width neighborhood. make span bigger
```

```
## Warning: Computation failed in `stat_smooth()`:
## NA/NaN/Inf in foreign function call (arg 5)
```

```
## Warning in simpleLoess(y, x, w, span, degree = degree, parametric = parametric, : at -0.005
```

```
## Warning in simpleLoess(y, x, w, span, degree = degree, parametric = parametric, : radius 2.5e-05
```

```
## Warning in simpleLoess(y, x, w, span, degree = degree, parametric = parametric, : all data on boundary of neighborhood. make span bigger
```

```
## Warning in simpleLoess(y, x, w, span, degree = degree, parametric = parametric, : pseudoinverse used at -0.005
```

```
## Warning in simpleLoess(y, x, w, span, degree = degree, parametric = parametric, : neighborhood radius 0.005
```

```
## Warning in simpleLoess(y, x, w, span, degree = degree, parametric = parametric, : reciprocal condition number 1
```

```
## Warning in simpleLoess(y, x, w, span, degree = degree, parametric = parametric, : There are other near singularities as well. 1.01
```

```
## Warning in simpleLoess(y, x, w, span, degree = degree, parametric = parametric, : zero-width neighborhood. make span bigger
```

```
## Warning: Computation failed in `stat_smooth()`:
## NA/NaN/Inf in foreign function call (arg 5)
```

```
## Warning in simpleLoess(y, x, w, span, degree = degree, parametric = parametric, : at -0.005
```

```
## Warning in simpleLoess(y, x, w, span, degree = degree, parametric = parametric, : radius 2.5e-05
```

```
## Warning in simpleLoess(y, x, w, span, degree = degree, parametric = parametric, : all data on boundary of neighborhood. make span bigger
```

```
## Warning in simpleLoess(y, x, w, span, degree = degree, parametric = parametric, : pseudoinverse used at -0.005
```

```
## Warning in simpleLoess(y, x, w, span, degree = degree, parametric = parametric, : neighborhood radius 0.005
```

```
## Warning in simpleLoess(y, x, w, span, degree = degree, parametric = parametric, : reciprocal condition number 1
```

```
## Warning in simpleLoess(y, x, w, span, degree = degree, parametric = parametric, : There are other near singularities as well. 1.01
```

```
## Warning in simpleLoess(y, x, w, span, degree = degree, parametric = parametric, : zero-width neighborhood. make span bigger
```

```
## Warning: Computation failed in `stat_smooth()`:
## NA/NaN/Inf in foreign function call (arg 5)
```

```
## Warning in simpleLoess(y, x, w, span, degree = degree, parametric = parametric, : at -0.005
```

```
## Warning in simpleLoess(y, x, w, span, degree = degree, parametric = parametric, : radius 2.5e-05
```

```
## Warning in simpleLoess(y, x, w, span, degree = degree, parametric = parametric, : all data on boundary of neighborhood. make span bigger
```

```
## Warning in simpleLoess(y, x, w, span, degree = degree, parametric = parametric, : pseudoinverse used at -0.005
```

```
## Warning in simpleLoess(y, x, w, span, degree = degree, parametric = parametric, : neighborhood radius 0.005
```

```
## Warning in simpleLoess(y, x, w, span, degree = degree, parametric = parametric, : reciprocal condition number 1
```

```
## Warning in simpleLoess(y, x, w, span, degree = degree, parametric = parametric, : There are other near singularities as well. 1.01
```

```
## Warning in simpleLoess(y, x, w, span, degree = degree, parametric = parametric, : zero-width neighborhood. make span bigger
```

```
## Warning: Computation failed in `stat_smooth()`:
## NA/NaN/Inf in foreign function call (arg 5)
```

```
## Warning in simpleLoess(y, x, w, span, degree = degree, parametric = parametric, : at -0.005
```

```
## Warning in simpleLoess(y, x, w, span, degree = degree, parametric = parametric, : radius 2.5e-05
```

```
## Warning in simpleLoess(y, x, w, span, degree = degree, parametric = parametric, : all data on boundary of neighborhood. make span bigger
```

```
## Warning in simpleLoess(y, x, w, span, degree = degree, parametric = parametric, : pseudoinverse used at -0.005
```

```
## Warning in simpleLoess(y, x, w, span, degree = degree, parametric = parametric, : neighborhood radius 0.005
```

```
## Warning in simpleLoess(y, x, w, span, degree = degree, parametric = parametric, : reciprocal condition number 1
```

```
## Warning in simpleLoess(y, x, w, span, degree = degree, parametric = parametric, : There are other near singularities as well. 1.01
```

```
## Warning in simpleLoess(y, x, w, span, degree = degree, parametric = parametric, : zero-width neighborhood. make span bigger
```

```
## Warning: Computation failed in `stat_smooth()`:
## NA/NaN/Inf in foreign function call (arg 5)
```

```
## Warning in simpleLoess(y, x, w, span, degree = degree, parametric = parametric, : at -0.005
```

```
## Warning in simpleLoess(y, x, w, span, degree = degree, parametric = parametric, : radius 2.5e-05
```

```
## Warning in simpleLoess(y, x, w, span, degree = degree, parametric = parametric, : all data on boundary of neighborhood. make span bigger
```

```
## Warning in simpleLoess(y, x, w, span, degree = degree, parametric = parametric, : pseudoinverse used at -0.005
```

```
## Warning in simpleLoess(y, x, w, span, degree = degree, parametric = parametric, : neighborhood radius 0.005
```

```
## Warning in simpleLoess(y, x, w, span, degree = degree, parametric = parametric, : reciprocal condition number 1
```

```
## Warning in simpleLoess(y, x, w, span, degree = degree, parametric = parametric, : There are other near singularities as well. 1.01
```

```
## Warning in simpleLoess(y, x, w, span, degree = degree, parametric = parametric, : zero-width neighborhood. make span bigger
```

```
## Warning: Computation failed in `stat_smooth()`:
## NA/NaN/Inf in foreign function call (arg 5)
```

```
## Warning in simpleLoess(y, x, w, span, degree = degree, parametric = parametric, : at -0.005
```

```
## Warning in simpleLoess(y, x, w, span, degree = degree, parametric = parametric, : radius 2.5e-05
```

```
## Warning in simpleLoess(y, x, w, span, degree = degree, parametric = parametric, : all data on boundary of neighborhood. make span bigger
```

```
## Warning in simpleLoess(y, x, w, span, degree = degree, parametric = parametric, : pseudoinverse used at -0.005
```

```
## Warning in simpleLoess(y, x, w, span, degree = degree, parametric = parametric, : neighborhood radius 0.005
```

```
## Warning in simpleLoess(y, x, w, span, degree = degree, parametric = parametric, : reciprocal condition number 1
```

```
## Warning in simpleLoess(y, x, w, span, degree = degree, parametric = parametric, : There are other near singularities as well. 1.01
```

```
## Warning in simpleLoess(y, x, w, span, degree = degree, parametric = parametric, : zero-width neighborhood. make span bigger
```

```
## Warning: Computation failed in `stat_smooth()`:
## NA/NaN/Inf in foreign function call (arg 5)
```

```
## Warning in simpleLoess(y, x, w, span, degree = degree, parametric = parametric, : at -0.005
```

```
## Warning in simpleLoess(y, x, w, span, degree = degree, parametric = parametric, : radius 2.5e-05
```

```
## Warning in simpleLoess(y, x, w, span, degree = degree, parametric = parametric, : all data on boundary of neighborhood. make span bigger
```

```
## Warning in simpleLoess(y, x, w, span, degree = degree, parametric = parametric, : pseudoinverse used at -0.005
```

```
## Warning in simpleLoess(y, x, w, span, degree = degree, parametric = parametric, : neighborhood radius 0.005
```

```
## Warning in simpleLoess(y, x, w, span, degree = degree, parametric = parametric, : reciprocal condition number 1
```

```
## Warning in simpleLoess(y, x, w, span, degree = degree, parametric = parametric, : There are other near singularities as well. 1.01
```

```
## Warning in simpleLoess(y, x, w, span, degree = degree, parametric = parametric, : zero-width neighborhood. make span bigger
```

```
## Warning: Computation failed in `stat_smooth()`:
## NA/NaN/Inf in foreign function call (arg 5)
```

```
## Warning in simpleLoess(y, x, w, span, degree = degree, parametric = parametric, : pseudoinverse used at -0.005
```

```
## Warning in simpleLoess(y, x, w, span, degree = degree, parametric = parametric, : neighborhood radius 1.005
```

```
## Warning in simpleLoess(y, x, w, span, degree = degree, parametric = parametric, : reciprocal condition number 0
```

```
## Warning in simpleLoess(y, x, w, span, degree = degree, parametric = parametric, : There are other near singularities as well. 1.01
```

```
## Warning in predLoess(object$y, object$x, newx = if (is.null(newdata)) object$x else if (is.data.frame(newdata)) as.matrix(model.frame(delete.response(terms(object)), : pseudoinverse used at -0.005
```

```
## Warning in predLoess(object$y, object$x, newx = if (is.null(newdata)) object$x else if (is.data.frame(newdata)) as.matrix(model.frame(delete.response(terms(object)), : neighborhood radius 1.005
```

```
## Warning in predLoess(object$y, object$x, newx = if (is.null(newdata)) object$x else if (is.data.frame(newdata)) as.matrix(model.frame(delete.response(terms(object)), : reciprocal condition number 0
```

```
## Warning in predLoess(object$y, object$x, newx = if (is.null(newdata)) object$x else if (is.data.frame(newdata)) as.matrix(model.frame(delete.response(terms(object)), : There are other near
## singularities as well. 1.01
```

```
## Warning in simpleLoess(y, x, w, span, degree = degree, parametric = parametric, : at -0.005
```

```
## Warning in simpleLoess(y, x, w, span, degree = degree, parametric = parametric, : radius 2.5e-05
```

```
## Warning in simpleLoess(y, x, w, span, degree = degree, parametric = parametric, : all data on boundary of neighborhood. make span bigger
```

```
## Warning in simpleLoess(y, x, w, span, degree = degree, parametric = parametric, : pseudoinverse used at -0.005
```

```
## Warning in simpleLoess(y, x, w, span, degree = degree, parametric = parametric, : neighborhood radius 0.005
```

```
## Warning in simpleLoess(y, x, w, span, degree = degree, parametric = parametric, : reciprocal condition number 1
```

```
## Warning in simpleLoess(y, x, w, span, degree = degree, parametric = parametric, : There are other near singularities as well. 1.01
```

```
## Warning in simpleLoess(y, x, w, span, degree = degree, parametric = parametric, : zero-width neighborhood. make span bigger
```

```
## Warning: Computation failed in `stat_smooth()`:
## NA/NaN/Inf in foreign function call (arg 5)
```

```
## Warning in simpleLoess(y, x, w, span, degree = degree, parametric = parametric, : at -0.005
```

```
## Warning in simpleLoess(y, x, w, span, degree = degree, parametric = parametric, : radius 2.5e-05
```

```
## Warning in simpleLoess(y, x, w, span, degree = degree, parametric = parametric, : all data on boundary of neighborhood. make span bigger
```

```
## Warning in simpleLoess(y, x, w, span, degree = degree, parametric = parametric, : pseudoinverse used at -0.005
```

```
## Warning in simpleLoess(y, x, w, span, degree = degree, parametric = parametric, : neighborhood radius 0.005
```

```
## Warning in simpleLoess(y, x, w, span, degree = degree, parametric = parametric, : reciprocal condition number 1
```

```
## Warning in simpleLoess(y, x, w, span, degree = degree, parametric = parametric, : There are other near singularities as well. 1.01
```

```
## Warning in simpleLoess(y, x, w, span, degree = degree, parametric = parametric, : zero-width neighborhood. make span bigger
```

```
## Warning: Computation failed in `stat_smooth()`:
## NA/NaN/Inf in foreign function call (arg 5)
```

```
## Warning in simpleLoess(y, x, w, span, degree = degree, parametric = parametric, : at -0.005
```

```
## Warning in simpleLoess(y, x, w, span, degree = degree, parametric = parametric, : radius 2.5e-05
```

```
## Warning in simpleLoess(y, x, w, span, degree = degree, parametric = parametric, : all data on boundary of neighborhood. make span bigger
```

```
## Warning in simpleLoess(y, x, w, span, degree = degree, parametric = parametric, : pseudoinverse used at -0.005
```

```
## Warning in simpleLoess(y, x, w, span, degree = degree, parametric = parametric, : neighborhood radius 0.005
```

```
## Warning in simpleLoess(y, x, w, span, degree = degree, parametric = parametric, : reciprocal condition number 1
```

```
## Warning in simpleLoess(y, x, w, span, degree = degree, parametric = parametric, : There are other near singularities as well. 1.01
```

```
## Warning in simpleLoess(y, x, w, span, degree = degree, parametric = parametric, : zero-width neighborhood. make span bigger
```

```
## Warning: Computation failed in `stat_smooth()`:
## NA/NaN/Inf in foreign function call (arg 5)
```

```
## Warning in simpleLoess(y, x, w, span, degree = degree, parametric = parametric, : pseudoinverse used at -0.005
```

```
## Warning in simpleLoess(y, x, w, span, degree = degree, parametric = parametric, : neighborhood radius 1.005
```

```
## Warning in simpleLoess(y, x, w, span, degree = degree, parametric = parametric, : reciprocal condition number 0
```

```
## Warning in simpleLoess(y, x, w, span, degree = degree, parametric = parametric, : There are other near singularities as well. 1.01
```

```
## Warning in predLoess(object$y, object$x, newx = if (is.null(newdata)) object$x else if (is.data.frame(newdata)) as.matrix(model.frame(delete.response(terms(object)), : pseudoinverse used at -0.005
```

```
## Warning in predLoess(object$y, object$x, newx = if (is.null(newdata)) object$x else if (is.data.frame(newdata)) as.matrix(model.frame(delete.response(terms(object)), : neighborhood radius 1.005
```

```
## Warning in predLoess(object$y, object$x, newx = if (is.null(newdata)) object$x else if (is.data.frame(newdata)) as.matrix(model.frame(delete.response(terms(object)), : reciprocal condition number 0
```

```
## Warning in predLoess(object$y, object$x, newx = if (is.null(newdata)) object$x else if (is.data.frame(newdata)) as.matrix(model.frame(delete.response(terms(object)), : There are other near
## singularities as well. 1.01
```

```
## Warning in simpleLoess(y, x, w, span, degree = degree, parametric = parametric, : pseudoinverse used at -0.005
```

```
## Warning in simpleLoess(y, x, w, span, degree = degree, parametric = parametric, : neighborhood radius 1.005
```

```
## Warning in simpleLoess(y, x, w, span, degree = degree, parametric = parametric, : reciprocal condition number 0
```

```
## Warning in simpleLoess(y, x, w, span, degree = degree, parametric = parametric, : There are other near singularities as well. 1.01
```

```
## Warning in predLoess(object$y, object$x, newx = if (is.null(newdata)) object$x else if (is.data.frame(newdata)) as.matrix(model.frame(delete.response(terms(object)), : pseudoinverse used at -0.005
```

```
## Warning in predLoess(object$y, object$x, newx = if (is.null(newdata)) object$x else if (is.data.frame(newdata)) as.matrix(model.frame(delete.response(terms(object)), : neighborhood radius 1.005
```

```
## Warning in predLoess(object$y, object$x, newx = if (is.null(newdata)) object$x else if (is.data.frame(newdata)) as.matrix(model.frame(delete.response(terms(object)), : reciprocal condition number 0
```

```
## Warning in predLoess(object$y, object$x, newx = if (is.null(newdata)) object$x else if (is.data.frame(newdata)) as.matrix(model.frame(delete.response(terms(object)), : There are other near
## singularities as well. 1.01
```

```
## Warning in simpleLoess(y, x, w, span, degree = degree, parametric = parametric, : at -0.005
```

```
## Warning in simpleLoess(y, x, w, span, degree = degree, parametric = parametric, : radius 2.5e-05
```

```
## Warning in simpleLoess(y, x, w, span, degree = degree, parametric = parametric, : all data on boundary of neighborhood. make span bigger
```

```
## Warning in simpleLoess(y, x, w, span, degree = degree, parametric = parametric, : pseudoinverse used at -0.005
```

```
## Warning in simpleLoess(y, x, w, span, degree = degree, parametric = parametric, : neighborhood radius 0.005
```

```
## Warning in simpleLoess(y, x, w, span, degree = degree, parametric = parametric, : reciprocal condition number 1
```

```
## Warning in simpleLoess(y, x, w, span, degree = degree, parametric = parametric, : There are other near singularities as well. 1.01
```

```
## Warning in simpleLoess(y, x, w, span, degree = degree, parametric = parametric, : zero-width neighborhood. make span bigger
```

```
## Warning: Computation failed in `stat_smooth()`:
## NA/NaN/Inf in foreign function call (arg 5)
```

```
## Warning in simpleLoess(y, x, w, span, degree = degree, parametric = parametric, : at -0.005
```

```
## Warning in simpleLoess(y, x, w, span, degree = degree, parametric = parametric, : radius 2.5e-05
```

```
## Warning in simpleLoess(y, x, w, span, degree = degree, parametric = parametric, : all data on boundary of neighborhood. make span bigger
```

```
## Warning in simpleLoess(y, x, w, span, degree = degree, parametric = parametric, : pseudoinverse used at -0.005
```

```
## Warning in simpleLoess(y, x, w, span, degree = degree, parametric = parametric, : neighborhood radius 0.005
```

```
## Warning in simpleLoess(y, x, w, span, degree = degree, parametric = parametric, : reciprocal condition number 1
```

```
## Warning in simpleLoess(y, x, w, span, degree = degree, parametric = parametric, : There are other near singularities as well. 1.01
```

```
## Warning in simpleLoess(y, x, w, span, degree = degree, parametric = parametric, : zero-width neighborhood. make span bigger
```

```
## Warning: Computation failed in `stat_smooth()`:
## NA/NaN/Inf in foreign function call (arg 5)
```

```
## Warning in simpleLoess(y, x, w, span, degree = degree, parametric = parametric, : at -0.005
```

```
## Warning in simpleLoess(y, x, w, span, degree = degree, parametric = parametric, : radius 2.5e-05
```

```
## Warning in simpleLoess(y, x, w, span, degree = degree, parametric = parametric, : all data on boundary of neighborhood. make span bigger
```

```
## Warning in simpleLoess(y, x, w, span, degree = degree, parametric = parametric, : pseudoinverse used at -0.005
```

```
## Warning in simpleLoess(y, x, w, span, degree = degree, parametric = parametric, : neighborhood radius 0.005
```

```
## Warning in simpleLoess(y, x, w, span, degree = degree, parametric = parametric, : reciprocal condition number 1
```

```
## Warning in simpleLoess(y, x, w, span, degree = degree, parametric = parametric, : There are other near singularities as well. 1.01
```

```
## Warning in simpleLoess(y, x, w, span, degree = degree, parametric = parametric, : zero-width neighborhood. make span bigger
```

```
## Warning: Computation failed in `stat_smooth()`:
## NA/NaN/Inf in foreign function call (arg 5)
```

```
## Warning in simpleLoess(y, x, w, span, degree = degree, parametric = parametric, : at -0.005
```

```
## Warning in simpleLoess(y, x, w, span, degree = degree, parametric = parametric, : radius 2.5e-05
```

```
## Warning in simpleLoess(y, x, w, span, degree = degree, parametric = parametric, : all data on boundary of neighborhood. make span bigger
```

```
## Warning in simpleLoess(y, x, w, span, degree = degree, parametric = parametric, : pseudoinverse used at -0.005
```

```
## Warning in simpleLoess(y, x, w, span, degree = degree, parametric = parametric, : neighborhood radius 0.005
```

```
## Warning in simpleLoess(y, x, w, span, degree = degree, parametric = parametric, : reciprocal condition number 1
```

```
## Warning in simpleLoess(y, x, w, span, degree = degree, parametric = parametric, : There are other near singularities as well. 1.01
```

```
## Warning in simpleLoess(y, x, w, span, degree = degree, parametric = parametric, : zero-width neighborhood. make span bigger
```

```
## Warning: Computation failed in `stat_smooth()`:
## NA/NaN/Inf in foreign function call (arg 5)
```

```
## Warning in simpleLoess(y, x, w, span, degree = degree, parametric = parametric, : at -0.005
```

```
## Warning in simpleLoess(y, x, w, span, degree = degree, parametric = parametric, : radius 2.5e-05
```

```
## Warning in simpleLoess(y, x, w, span, degree = degree, parametric = parametric, : all data on boundary of neighborhood. make span bigger
```

```
## Warning in simpleLoess(y, x, w, span, degree = degree, parametric = parametric, : pseudoinverse used at -0.005
```

```
## Warning in simpleLoess(y, x, w, span, degree = degree, parametric = parametric, : neighborhood radius 0.005
```

```
## Warning in simpleLoess(y, x, w, span, degree = degree, parametric = parametric, : reciprocal condition number 1
```

```
## Warning in simpleLoess(y, x, w, span, degree = degree, parametric = parametric, : There are other near singularities as well. 1.01
```

```
## Warning in simpleLoess(y, x, w, span, degree = degree, parametric = parametric, : zero-width neighborhood. make span bigger
```

```
## Warning: Computation failed in `stat_smooth()`:
## NA/NaN/Inf in foreign function call (arg 5)
```

```
## Warning in simpleLoess(y, x, w, span, degree = degree, parametric = parametric, : at -0.005
```

```
## Warning in simpleLoess(y, x, w, span, degree = degree, parametric = parametric, : radius 2.5e-05
```

```
## Warning in simpleLoess(y, x, w, span, degree = degree, parametric = parametric, : all data on boundary of neighborhood. make span bigger
```

```
## Warning in simpleLoess(y, x, w, span, degree = degree, parametric = parametric, : pseudoinverse used at -0.005
```

```
## Warning in simpleLoess(y, x, w, span, degree = degree, parametric = parametric, : neighborhood radius 0.005
```

```
## Warning in simpleLoess(y, x, w, span, degree = degree, parametric = parametric, : reciprocal condition number 1
```

```
## Warning in simpleLoess(y, x, w, span, degree = degree, parametric = parametric, : There are other near singularities as well. 1.01
```

```
## Warning in simpleLoess(y, x, w, span, degree = degree, parametric = parametric, : zero-width neighborhood. make span bigger
```

```
## Warning: Computation failed in `stat_smooth()`:
## NA/NaN/Inf in foreign function call (arg 5)
```

```
## Warning in simpleLoess(y, x, w, span, degree = degree, parametric = parametric, : at -0.005
```

```
## Warning in simpleLoess(y, x, w, span, degree = degree, parametric = parametric, : radius 2.5e-05
```

```
## Warning in simpleLoess(y, x, w, span, degree = degree, parametric = parametric, : all data on boundary of neighborhood. make span bigger
```

```
## Warning in simpleLoess(y, x, w, span, degree = degree, parametric = parametric, : pseudoinverse used at -0.005
```

```
## Warning in simpleLoess(y, x, w, span, degree = degree, parametric = parametric, : neighborhood radius 0.005
```

```
## Warning in simpleLoess(y, x, w, span, degree = degree, parametric = parametric, : reciprocal condition number 1
```

```
## Warning in simpleLoess(y, x, w, span, degree = degree, parametric = parametric, : There are other near singularities as well. 1.01
```

```
## Warning in simpleLoess(y, x, w, span, degree = degree, parametric = parametric, : zero-width neighborhood. make span bigger
```

```
## Warning: Computation failed in `stat_smooth()`:
## NA/NaN/Inf in foreign function call (arg 5)
```

```
## Warning in simpleLoess(y, x, w, span, degree = degree, parametric = parametric, : at -0.005
```

```
## Warning in simpleLoess(y, x, w, span, degree = degree, parametric = parametric, : radius 2.5e-05
```

```
## Warning in simpleLoess(y, x, w, span, degree = degree, parametric = parametric, : all data on boundary of neighborhood. make span bigger
```

```
## Warning in simpleLoess(y, x, w, span, degree = degree, parametric = parametric, : pseudoinverse used at -0.005
```

```
## Warning in simpleLoess(y, x, w, span, degree = degree, parametric = parametric, : neighborhood radius 0.005
```

```
## Warning in simpleLoess(y, x, w, span, degree = degree, parametric = parametric, : reciprocal condition number 1
```

```
## Warning in simpleLoess(y, x, w, span, degree = degree, parametric = parametric, : There are other near singularities as well. 1.01
```

```
## Warning in simpleLoess(y, x, w, span, degree = degree, parametric = parametric, : zero-width neighborhood. make span bigger
```

```
## Warning: Computation failed in `stat_smooth()`:
## NA/NaN/Inf in foreign function call (arg 5)
```

```
## Warning in simpleLoess(y, x, w, span, degree = degree, parametric = parametric, : at -0.005
```

```
## Warning in simpleLoess(y, x, w, span, degree = degree, parametric = parametric, : radius 2.5e-05
```

```
## Warning in simpleLoess(y, x, w, span, degree = degree, parametric = parametric, : all data on boundary of neighborhood. make span bigger
```

```
## Warning in simpleLoess(y, x, w, span, degree = degree, parametric = parametric, : pseudoinverse used at -0.005
```

```
## Warning in simpleLoess(y, x, w, span, degree = degree, parametric = parametric, : neighborhood radius 0.005
```

```
## Warning in simpleLoess(y, x, w, span, degree = degree, parametric = parametric, : reciprocal condition number 1
```

```
## Warning in simpleLoess(y, x, w, span, degree = degree, parametric = parametric, : There are other near singularities as well. 1.01
```

```
## Warning in simpleLoess(y, x, w, span, degree = degree, parametric = parametric, : zero-width neighborhood. make span bigger
```

```
## Warning: Computation failed in `stat_smooth()`:
## NA/NaN/Inf in foreign function call (arg 5)
```

```
## Warning in simpleLoess(y, x, w, span, degree = degree, parametric = parametric, : at -0.005
```

```
## Warning in simpleLoess(y, x, w, span, degree = degree, parametric = parametric, : radius 2.5e-05
```

```
## Warning in simpleLoess(y, x, w, span, degree = degree, parametric = parametric, : all data on boundary of neighborhood. make span bigger
```

```
## Warning in simpleLoess(y, x, w, span, degree = degree, parametric = parametric, : pseudoinverse used at -0.005
```

```
## Warning in simpleLoess(y, x, w, span, degree = degree, parametric = parametric, : neighborhood radius 0.005
```

```
## Warning in simpleLoess(y, x, w, span, degree = degree, parametric = parametric, : reciprocal condition number 1
```

```
## Warning in simpleLoess(y, x, w, span, degree = degree, parametric = parametric, : There are other near singularities as well. 1.01
```

```
## Warning in simpleLoess(y, x, w, span, degree = degree, parametric = parametric, : zero-width neighborhood. make span bigger
```

```
## Warning: Computation failed in `stat_smooth()`:
## NA/NaN/Inf in foreign function call (arg 5)
```

```
## Warning in simpleLoess(y, x, w, span, degree = degree, parametric = parametric, : at -0.005
```

```
## Warning in simpleLoess(y, x, w, span, degree = degree, parametric = parametric, : radius 2.5e-05
```

```
## Warning in simpleLoess(y, x, w, span, degree = degree, parametric = parametric, : all data on boundary of neighborhood. make span bigger
```

```
## Warning in simpleLoess(y, x, w, span, degree = degree, parametric = parametric, : pseudoinverse used at -0.005
```

```
## Warning in simpleLoess(y, x, w, span, degree = degree, parametric = parametric, : neighborhood radius 0.005
```

```
## Warning in simpleLoess(y, x, w, span, degree = degree, parametric = parametric, : reciprocal condition number 1
```

```
## Warning in simpleLoess(y, x, w, span, degree = degree, parametric = parametric, : There are other near singularities as well. 1.01
```

```
## Warning in simpleLoess(y, x, w, span, degree = degree, parametric = parametric, : zero-width neighborhood. make span bigger
```

```
## Warning: Computation failed in `stat_smooth()`:
## NA/NaN/Inf in foreign function call (arg 5)
```

```
## Warning in simpleLoess(y, x, w, span, degree = degree, parametric = parametric, : at -0.005
```

```
## Warning in simpleLoess(y, x, w, span, degree = degree, parametric = parametric, : radius 2.5e-05
```

```
## Warning in simpleLoess(y, x, w, span, degree = degree, parametric = parametric, : all data on boundary of neighborhood. make span bigger
```

```
## Warning in simpleLoess(y, x, w, span, degree = degree, parametric = parametric, : pseudoinverse used at -0.005
```

```
## Warning in simpleLoess(y, x, w, span, degree = degree, parametric = parametric, : neighborhood radius 0.005
```

```
## Warning in simpleLoess(y, x, w, span, degree = degree, parametric = parametric, : reciprocal condition number 1
```

```
## Warning in simpleLoess(y, x, w, span, degree = degree, parametric = parametric, : There are other near singularities as well. 1.01
```

```
## Warning in simpleLoess(y, x, w, span, degree = degree, parametric = parametric, : zero-width neighborhood. make span bigger
```

```
## Warning: Computation failed in `stat_smooth()`:
## NA/NaN/Inf in foreign function call (arg 5)
```

```
## Warning in simpleLoess(y, x, w, span, degree = degree, parametric = parametric, : at -0.005
```

```
## Warning in simpleLoess(y, x, w, span, degree = degree, parametric = parametric, : radius 2.5e-05
```

```
## Warning in simpleLoess(y, x, w, span, degree = degree, parametric = parametric, : all data on boundary of neighborhood. make span bigger
```

```
## Warning in simpleLoess(y, x, w, span, degree = degree, parametric = parametric, : pseudoinverse used at -0.005
```

```
## Warning in simpleLoess(y, x, w, span, degree = degree, parametric = parametric, : neighborhood radius 0.005
```

```
## Warning in simpleLoess(y, x, w, span, degree = degree, parametric = parametric, : reciprocal condition number 1
```

```
## Warning in simpleLoess(y, x, w, span, degree = degree, parametric = parametric, : There are other near singularities as well. 1.01
```

```
## Warning in simpleLoess(y, x, w, span, degree = degree, parametric = parametric, : zero-width neighborhood. make span bigger
```

```
## Warning: Computation failed in `stat_smooth()`:
## NA/NaN/Inf in foreign function call (arg 5)
```

```
## Warning in simpleLoess(y, x, w, span, degree = degree, parametric = parametric, : at -0.005
```

```
## Warning in simpleLoess(y, x, w, span, degree = degree, parametric = parametric, : radius 2.5e-05
```

```
## Warning in simpleLoess(y, x, w, span, degree = degree, parametric = parametric, : all data on boundary of neighborhood. make span bigger
```

```
## Warning in simpleLoess(y, x, w, span, degree = degree, parametric = parametric, : pseudoinverse used at -0.005
```

```
## Warning in simpleLoess(y, x, w, span, degree = degree, parametric = parametric, : neighborhood radius 0.005
```

```
## Warning in simpleLoess(y, x, w, span, degree = degree, parametric = parametric, : reciprocal condition number 1
```

```
## Warning in simpleLoess(y, x, w, span, degree = degree, parametric = parametric, : There are other near singularities as well. 1.01
```

```
## Warning in simpleLoess(y, x, w, span, degree = degree, parametric = parametric, : zero-width neighborhood. make span bigger
```

```
## Warning: Computation failed in `stat_smooth()`:
## NA/NaN/Inf in foreign function call (arg 5)
```

```
## Warning in simpleLoess(y, x, w, span, degree = degree, parametric = parametric, : at -0.005
```

```
## Warning in simpleLoess(y, x, w, span, degree = degree, parametric = parametric, : radius 2.5e-05
```

```
## Warning in simpleLoess(y, x, w, span, degree = degree, parametric = parametric, : all data on boundary of neighborhood. make span bigger
```

```
## Warning in simpleLoess(y, x, w, span, degree = degree, parametric = parametric, : pseudoinverse used at -0.005
```

```
## Warning in simpleLoess(y, x, w, span, degree = degree, parametric = parametric, : neighborhood radius 0.005
```

```
## Warning in simpleLoess(y, x, w, span, degree = degree, parametric = parametric, : reciprocal condition number 1
```

```
## Warning in simpleLoess(y, x, w, span, degree = degree, parametric = parametric, : There are other near singularities as well. 1.01
```

```
## Warning in simpleLoess(y, x, w, span, degree = degree, parametric = parametric, : zero-width neighborhood. make span bigger
```

```
## Warning: Computation failed in `stat_smooth()`:
## NA/NaN/Inf in foreign function call (arg 5)
```

```
## Warning in simpleLoess(y, x, w, span, degree = degree, parametric = parametric, : pseudoinverse used at -0.005
```

```
## Warning in simpleLoess(y, x, w, span, degree = degree, parametric = parametric, : neighborhood radius 1.005
```

```
## Warning in simpleLoess(y, x, w, span, degree = degree, parametric = parametric, : reciprocal condition number 0
```

```
## Warning in simpleLoess(y, x, w, span, degree = degree, parametric = parametric, : There are other near singularities as well. 1.01
```

```
## Warning in predLoess(object$y, object$x, newx = if (is.null(newdata)) object$x else if (is.data.frame(newdata)) as.matrix(model.frame(delete.response(terms(object)), : pseudoinverse used at -0.005
```

```
## Warning in predLoess(object$y, object$x, newx = if (is.null(newdata)) object$x else if (is.data.frame(newdata)) as.matrix(model.frame(delete.response(terms(object)), : neighborhood radius 1.005
```

```
## Warning in predLoess(object$y, object$x, newx = if (is.null(newdata)) object$x else if (is.data.frame(newdata)) as.matrix(model.frame(delete.response(terms(object)), : reciprocal condition number 0
```

```
## Warning in predLoess(object$y, object$x, newx = if (is.null(newdata)) object$x else if (is.data.frame(newdata)) as.matrix(model.frame(delete.response(terms(object)), : There are other near
## singularities as well. 1.01
```

```
## Warning in simpleLoess(y, x, w, span, degree = degree, parametric = parametric, : at -0.005
```

```
## Warning in simpleLoess(y, x, w, span, degree = degree, parametric = parametric, : radius 2.5e-05
```

```
## Warning in simpleLoess(y, x, w, span, degree = degree, parametric = parametric, : all data on boundary of neighborhood. make span bigger
```

```
## Warning in simpleLoess(y, x, w, span, degree = degree, parametric = parametric, : pseudoinverse used at -0.005
```

```
## Warning in simpleLoess(y, x, w, span, degree = degree, parametric = parametric, : neighborhood radius 0.005
```

```
## Warning in simpleLoess(y, x, w, span, degree = degree, parametric = parametric, : reciprocal condition number 1
```

```
## Warning in simpleLoess(y, x, w, span, degree = degree, parametric = parametric, : There are other near singularities as well. 1.01
```

```
## Warning in simpleLoess(y, x, w, span, degree = degree, parametric = parametric, : zero-width neighborhood. make span bigger
```

```
## Warning: Computation failed in `stat_smooth()`:
## NA/NaN/Inf in foreign function call (arg 5)
```

```
## Warning in simpleLoess(y, x, w, span, degree = degree, parametric = parametric, : at -0.005
```

```
## Warning in simpleLoess(y, x, w, span, degree = degree, parametric = parametric, : radius 2.5e-05
```

```
## Warning in simpleLoess(y, x, w, span, degree = degree, parametric = parametric, : all data on boundary of neighborhood. make span bigger
```

```
## Warning in simpleLoess(y, x, w, span, degree = degree, parametric = parametric, : pseudoinverse used at -0.005
```

```
## Warning in simpleLoess(y, x, w, span, degree = degree, parametric = parametric, : neighborhood radius 0.005
```

```
## Warning in simpleLoess(y, x, w, span, degree = degree, parametric = parametric, : reciprocal condition number 1
```

```
## Warning in simpleLoess(y, x, w, span, degree = degree, parametric = parametric, : There are other near singularities as well. 1.01
```

```
## Warning in simpleLoess(y, x, w, span, degree = degree, parametric = parametric, : zero-width neighborhood. make span bigger
```

```
## Warning: Computation failed in `stat_smooth()`:
## NA/NaN/Inf in foreign function call (arg 5)
```

```
## Warning in simpleLoess(y, x, w, span, degree = degree, parametric = parametric, : pseudoinverse used at -0.005
```

```
## Warning in simpleLoess(y, x, w, span, degree = degree, parametric = parametric, : neighborhood radius 1.005
```

```
## Warning in simpleLoess(y, x, w, span, degree = degree, parametric = parametric, : reciprocal condition number 0
```

```
## Warning in simpleLoess(y, x, w, span, degree = degree, parametric = parametric, : There are other near singularities as well. 1.01
```

```
## Warning in predLoess(object$y, object$x, newx = if (is.null(newdata)) object$x else if (is.data.frame(newdata)) as.matrix(model.frame(delete.response(terms(object)), : pseudoinverse used at -0.005
```

```
## Warning in predLoess(object$y, object$x, newx = if (is.null(newdata)) object$x else if (is.data.frame(newdata)) as.matrix(model.frame(delete.response(terms(object)), : neighborhood radius 1.005
```

```
## Warning in predLoess(object$y, object$x, newx = if (is.null(newdata)) object$x else if (is.data.frame(newdata)) as.matrix(model.frame(delete.response(terms(object)), : reciprocal condition number 0
```

```
## Warning in predLoess(object$y, object$x, newx = if (is.null(newdata)) object$x else if (is.data.frame(newdata)) as.matrix(model.frame(delete.response(terms(object)), : There are other near
## singularities as well. 1.01
```

```
## Warning in simpleLoess(y, x, w, span, degree = degree, parametric = parametric, : at -0.005
```

```
## Warning in simpleLoess(y, x, w, span, degree = degree, parametric = parametric, : radius 2.5e-05
```

```
## Warning in simpleLoess(y, x, w, span, degree = degree, parametric = parametric, : all data on boundary of neighborhood. make span bigger
```

```
## Warning in simpleLoess(y, x, w, span, degree = degree, parametric = parametric, : pseudoinverse used at -0.005
```

```
## Warning in simpleLoess(y, x, w, span, degree = degree, parametric = parametric, : neighborhood radius 0.005
```

```
## Warning in simpleLoess(y, x, w, span, degree = degree, parametric = parametric, : reciprocal condition number 1
```

```
## Warning in simpleLoess(y, x, w, span, degree = degree, parametric = parametric, : There are other near singularities as well. 1.01
```

```
## Warning in simpleLoess(y, x, w, span, degree = degree, parametric = parametric, : zero-width neighborhood. make span bigger
```

```
## Warning: Computation failed in `stat_smooth()`:
## NA/NaN/Inf in foreign function call (arg 5)
```

```
## Warning in simpleLoess(y, x, w, span, degree = degree, parametric = parametric, : pseudoinverse used at -0.005
```

```
## Warning in simpleLoess(y, x, w, span, degree = degree, parametric = parametric, : neighborhood radius 1.005
```

```
## Warning in simpleLoess(y, x, w, span, degree = degree, parametric = parametric, : reciprocal condition number 0
```

```
## Warning in simpleLoess(y, x, w, span, degree = degree, parametric = parametric, : There are other near singularities as well. 1.01
```

```
## Warning in predLoess(object$y, object$x, newx = if (is.null(newdata)) object$x else if (is.data.frame(newdata)) as.matrix(model.frame(delete.response(terms(object)), : pseudoinverse used at -0.005
```

```
## Warning in predLoess(object$y, object$x, newx = if (is.null(newdata)) object$x else if (is.data.frame(newdata)) as.matrix(model.frame(delete.response(terms(object)), : neighborhood radius 1.005
```

```
## Warning in predLoess(object$y, object$x, newx = if (is.null(newdata)) object$x else if (is.data.frame(newdata)) as.matrix(model.frame(delete.response(terms(object)), : reciprocal condition number 0
```

```
## Warning in predLoess(object$y, object$x, newx = if (is.null(newdata)) object$x else if (is.data.frame(newdata)) as.matrix(model.frame(delete.response(terms(object)), : There are other near
## singularities as well. 1.01
```

```
## Warning in simpleLoess(y, x, w, span, degree = degree, parametric = parametric, : pseudoinverse used at -0.005
```

```
## Warning in simpleLoess(y, x, w, span, degree = degree, parametric = parametric, : neighborhood radius 1.005
```

```
## Warning in simpleLoess(y, x, w, span, degree = degree, parametric = parametric, : reciprocal condition number 0
```

```
## Warning in simpleLoess(y, x, w, span, degree = degree, parametric = parametric, : There are other near singularities as well. 1.01
```

```
## Warning in predLoess(object$y, object$x, newx = if (is.null(newdata)) object$x else if (is.data.frame(newdata)) as.matrix(model.frame(delete.response(terms(object)), : pseudoinverse used at -0.005
```

```
## Warning in predLoess(object$y, object$x, newx = if (is.null(newdata)) object$x else if (is.data.frame(newdata)) as.matrix(model.frame(delete.response(terms(object)), : neighborhood radius 1.005
```

```
## Warning in predLoess(object$y, object$x, newx = if (is.null(newdata)) object$x else if (is.data.frame(newdata)) as.matrix(model.frame(delete.response(terms(object)), : reciprocal condition number 0
```

```
## Warning in predLoess(object$y, object$x, newx = if (is.null(newdata)) object$x else if (is.data.frame(newdata)) as.matrix(model.frame(delete.response(terms(object)), : There are other near
## singularities as well. 1.01
```

```
## Warning in simpleLoess(y, x, w, span, degree = degree, parametric = parametric, : at -0.005
```

```
## Warning in simpleLoess(y, x, w, span, degree = degree, parametric = parametric, : radius 2.5e-05
```

```
## Warning in simpleLoess(y, x, w, span, degree = degree, parametric = parametric, : all data on boundary of neighborhood. make span bigger
```

```
## Warning in simpleLoess(y, x, w, span, degree = degree, parametric = parametric, : pseudoinverse used at -0.005
```

```
## Warning in simpleLoess(y, x, w, span, degree = degree, parametric = parametric, : neighborhood radius 0.005
```

```
## Warning in simpleLoess(y, x, w, span, degree = degree, parametric = parametric, : reciprocal condition number 1
```

```
## Warning in simpleLoess(y, x, w, span, degree = degree, parametric = parametric, : There are other near singularities as well. 1.01
```

```
## Warning in simpleLoess(y, x, w, span, degree = degree, parametric = parametric, : zero-width neighborhood. make span bigger
```

```
## Warning: Computation failed in `stat_smooth()`:
## NA/NaN/Inf in foreign function call (arg 5)
```

```
## Warning in simpleLoess(y, x, w, span, degree = degree, parametric = parametric, : at -0.005
```

```
## Warning in simpleLoess(y, x, w, span, degree = degree, parametric = parametric, : radius 2.5e-05
```

```
## Warning in simpleLoess(y, x, w, span, degree = degree, parametric = parametric, : all data on boundary of neighborhood. make span bigger
```

```
## Warning in simpleLoess(y, x, w, span, degree = degree, parametric = parametric, : pseudoinverse used at -0.005
```

```
## Warning in simpleLoess(y, x, w, span, degree = degree, parametric = parametric, : neighborhood radius 0.005
```

```
## Warning in simpleLoess(y, x, w, span, degree = degree, parametric = parametric, : reciprocal condition number 1
```

```
## Warning in simpleLoess(y, x, w, span, degree = degree, parametric = parametric, : There are other near singularities as well. 1.01
```

```
## Warning in simpleLoess(y, x, w, span, degree = degree, parametric = parametric, : zero-width neighborhood. make span bigger
```

```
## Warning: Computation failed in `stat_smooth()`:
## NA/NaN/Inf in foreign function call (arg 5)
```

```
## Warning in simpleLoess(y, x, w, span, degree = degree, parametric = parametric, : at -0.005
```

```
## Warning in simpleLoess(y, x, w, span, degree = degree, parametric = parametric, : radius 2.5e-05
```

```
## Warning in simpleLoess(y, x, w, span, degree = degree, parametric = parametric, : all data on boundary of neighborhood. make span bigger
```

```
## Warning in simpleLoess(y, x, w, span, degree = degree, parametric = parametric, : pseudoinverse used at -0.005
```

```
## Warning in simpleLoess(y, x, w, span, degree = degree, parametric = parametric, : neighborhood radius 0.005
```

```
## Warning in simpleLoess(y, x, w, span, degree = degree, parametric = parametric, : reciprocal condition number 1
```

```
## Warning in simpleLoess(y, x, w, span, degree = degree, parametric = parametric, : There are other near singularities as well. 1.01
```

```
## Warning in simpleLoess(y, x, w, span, degree = degree, parametric = parametric, : zero-width neighborhood. make span bigger
```

```
## Warning: Computation failed in `stat_smooth()`:
## NA/NaN/Inf in foreign function call (arg 5)
```

```
## Warning in simpleLoess(y, x, w, span, degree = degree, parametric = parametric, : at -0.005
```

```
## Warning in simpleLoess(y, x, w, span, degree = degree, parametric = parametric, : radius 2.5e-05
```

```
## Warning in simpleLoess(y, x, w, span, degree = degree, parametric = parametric, : all data on boundary of neighborhood. make span bigger
```

```
## Warning in simpleLoess(y, x, w, span, degree = degree, parametric = parametric, : pseudoinverse used at -0.005
```

```
## Warning in simpleLoess(y, x, w, span, degree = degree, parametric = parametric, : neighborhood radius 0.005
```

```
## Warning in simpleLoess(y, x, w, span, degree = degree, parametric = parametric, : reciprocal condition number 1
```

```
## Warning in simpleLoess(y, x, w, span, degree = degree, parametric = parametric, : There are other near singularities as well. 1.01
```

```
## Warning in simpleLoess(y, x, w, span, degree = degree, parametric = parametric, : zero-width neighborhood. make span bigger
```

```
## Warning: Computation failed in `stat_smooth()`:
## NA/NaN/Inf in foreign function call (arg 5)
```

```
## Warning in simpleLoess(y, x, w, span, degree = degree, parametric = parametric, : at -0.005
```

```
## Warning in simpleLoess(y, x, w, span, degree = degree, parametric = parametric, : radius 2.5e-05
```

```
## Warning in simpleLoess(y, x, w, span, degree = degree, parametric = parametric, : all data on boundary of neighborhood. make span bigger
```

```
## Warning in simpleLoess(y, x, w, span, degree = degree, parametric = parametric, : pseudoinverse used at -0.005
```

```
## Warning in simpleLoess(y, x, w, span, degree = degree, parametric = parametric, : neighborhood radius 0.005
```

```
## Warning in simpleLoess(y, x, w, span, degree = degree, parametric = parametric, : reciprocal condition number 1
```

```
## Warning in simpleLoess(y, x, w, span, degree = degree, parametric = parametric, : There are other near singularities as well. 1.01
```

```
## Warning in simpleLoess(y, x, w, span, degree = degree, parametric = parametric, : zero-width neighborhood. make span bigger
```

```
## Warning: Computation failed in `stat_smooth()`:
## NA/NaN/Inf in foreign function call (arg 5)
```

```
## Warning in simpleLoess(y, x, w, span, degree = degree, parametric = parametric, : at -0.005
```

```
## Warning in simpleLoess(y, x, w, span, degree = degree, parametric = parametric, : radius 2.5e-05
```

```
## Warning in simpleLoess(y, x, w, span, degree = degree, parametric = parametric, : all data on boundary of neighborhood. make span bigger
```

```
## Warning in simpleLoess(y, x, w, span, degree = degree, parametric = parametric, : pseudoinverse used at -0.005
```

```
## Warning in simpleLoess(y, x, w, span, degree = degree, parametric = parametric, : neighborhood radius 0.005
```

```
## Warning in simpleLoess(y, x, w, span, degree = degree, parametric = parametric, : reciprocal condition number 1
```

```
## Warning in simpleLoess(y, x, w, span, degree = degree, parametric = parametric, : There are other near singularities as well. 1.01
```

```
## Warning in simpleLoess(y, x, w, span, degree = degree, parametric = parametric, : zero-width neighborhood. make span bigger
```

```
## Warning: Computation failed in `stat_smooth()`:
## NA/NaN/Inf in foreign function call (arg 5)
```

```
## Warning in simpleLoess(y, x, w, span, degree = degree, parametric = parametric, : at -0.005
```

```
## Warning in simpleLoess(y, x, w, span, degree = degree, parametric = parametric, : radius 2.5e-05
```

```
## Warning in simpleLoess(y, x, w, span, degree = degree, parametric = parametric, : all data on boundary of neighborhood. make span bigger
```

```
## Warning in simpleLoess(y, x, w, span, degree = degree, parametric = parametric, : pseudoinverse used at -0.005
```

```
## Warning in simpleLoess(y, x, w, span, degree = degree, parametric = parametric, : neighborhood radius 0.005
```

```
## Warning in simpleLoess(y, x, w, span, degree = degree, parametric = parametric, : reciprocal condition number 1
```

```
## Warning in simpleLoess(y, x, w, span, degree = degree, parametric = parametric, : There are other near singularities as well. 1.01
```

```
## Warning in simpleLoess(y, x, w, span, degree = degree, parametric = parametric, : zero-width neighborhood. make span bigger
```

```
## Warning: Computation failed in `stat_smooth()`:
## NA/NaN/Inf in foreign function call (arg 5)
```

```
## Warning in simpleLoess(y, x, w, span, degree = degree, parametric = parametric, : at -0.005
```

```
## Warning in simpleLoess(y, x, w, span, degree = degree, parametric = parametric, : radius 2.5e-05
```

```
## Warning in simpleLoess(y, x, w, span, degree = degree, parametric = parametric, : all data on boundary of neighborhood. make span bigger
```

```
## Warning in simpleLoess(y, x, w, span, degree = degree, parametric = parametric, : pseudoinverse used at -0.005
```

```
## Warning in simpleLoess(y, x, w, span, degree = degree, parametric = parametric, : neighborhood radius 0.005
```

```
## Warning in simpleLoess(y, x, w, span, degree = degree, parametric = parametric, : reciprocal condition number 1
```

```
## Warning in simpleLoess(y, x, w, span, degree = degree, parametric = parametric, : There are other near singularities as well. 1.01
```

```
## Warning in simpleLoess(y, x, w, span, degree = degree, parametric = parametric, : zero-width neighborhood. make span bigger
```

```
## Warning: Computation failed in `stat_smooth()`:
## NA/NaN/Inf in foreign function call (arg 5)
```

```
## Warning in simpleLoess(y, x, w, span, degree = degree, parametric = parametric, : at -0.005
```

```
## Warning in simpleLoess(y, x, w, span, degree = degree, parametric = parametric, : radius 2.5e-05
```

```
## Warning in simpleLoess(y, x, w, span, degree = degree, parametric = parametric, : all data on boundary of neighborhood. make span bigger
```

```
## Warning in simpleLoess(y, x, w, span, degree = degree, parametric = parametric, : pseudoinverse used at -0.005
```

```
## Warning in simpleLoess(y, x, w, span, degree = degree, parametric = parametric, : neighborhood radius 0.005
```

```
## Warning in simpleLoess(y, x, w, span, degree = degree, parametric = parametric, : reciprocal condition number 1
```

```
## Warning in simpleLoess(y, x, w, span, degree = degree, parametric = parametric, : There are other near singularities as well. 1.01
```

```
## Warning in simpleLoess(y, x, w, span, degree = degree, parametric = parametric, : zero-width neighborhood. make span bigger
```

```
## Warning: Computation failed in `stat_smooth()`:
## NA/NaN/Inf in foreign function call (arg 5)
```

```
## Warning in simpleLoess(y, x, w, span, degree = degree, parametric = parametric, : at -0.005
```

```
## Warning in simpleLoess(y, x, w, span, degree = degree, parametric = parametric, : radius 2.5e-05
```

```
## Warning in simpleLoess(y, x, w, span, degree = degree, parametric = parametric, : all data on boundary of neighborhood. make span bigger
```

```
## Warning in simpleLoess(y, x, w, span, degree = degree, parametric = parametric, : pseudoinverse used at -0.005
```

```
## Warning in simpleLoess(y, x, w, span, degree = degree, parametric = parametric, : neighborhood radius 0.005
```

```
## Warning in simpleLoess(y, x, w, span, degree = degree, parametric = parametric, : reciprocal condition number 1
```

```
## Warning in simpleLoess(y, x, w, span, degree = degree, parametric = parametric, : There are other near singularities as well. 1.01
```

```
## Warning in simpleLoess(y, x, w, span, degree = degree, parametric = parametric, : zero-width neighborhood. make span bigger
```

```
## Warning: Computation failed in `stat_smooth()`:
## NA/NaN/Inf in foreign function call (arg 5)
```

```
## Warning in simpleLoess(y, x, w, span, degree = degree, parametric = parametric, : at -0.005
```

```
## Warning in simpleLoess(y, x, w, span, degree = degree, parametric = parametric, : radius 2.5e-05
```

```
## Warning in simpleLoess(y, x, w, span, degree = degree, parametric = parametric, : all data on boundary of neighborhood. make span bigger
```

```
## Warning in simpleLoess(y, x, w, span, degree = degree, parametric = parametric, : pseudoinverse used at -0.005
```

```
## Warning in simpleLoess(y, x, w, span, degree = degree, parametric = parametric, : neighborhood radius 0.005
```

```
## Warning in simpleLoess(y, x, w, span, degree = degree, parametric = parametric, : reciprocal condition number 1
```

```
## Warning in simpleLoess(y, x, w, span, degree = degree, parametric = parametric, : There are other near singularities as well. 1.01
```

```
## Warning in simpleLoess(y, x, w, span, degree = degree, parametric = parametric, : zero-width neighborhood. make span bigger
```

```
## Warning: Computation failed in `stat_smooth()`:
## NA/NaN/Inf in foreign function call (arg 5)
```

```
## Warning in simpleLoess(y, x, w, span, degree = degree, parametric = parametric, : at -0.005
```

```
## Warning in simpleLoess(y, x, w, span, degree = degree, parametric = parametric, : radius 2.5e-05
```

```
## Warning in simpleLoess(y, x, w, span, degree = degree, parametric = parametric, : all data on boundary of neighborhood. make span bigger
```

```
## Warning in simpleLoess(y, x, w, span, degree = degree, parametric = parametric, : pseudoinverse used at -0.005
```

```
## Warning in simpleLoess(y, x, w, span, degree = degree, parametric = parametric, : neighborhood radius 0.005
```

```
## Warning in simpleLoess(y, x, w, span, degree = degree, parametric = parametric, : reciprocal condition number 1
```

```
## Warning in simpleLoess(y, x, w, span, degree = degree, parametric = parametric, : There are other near singularities as well. 1.01
```

```
## Warning in simpleLoess(y, x, w, span, degree = degree, parametric = parametric, : zero-width neighborhood. make span bigger
```

```
## Warning: Computation failed in `stat_smooth()`:
## NA/NaN/Inf in foreign function call (arg 5)
```

```
## Warning in simpleLoess(y, x, w, span, degree = degree, parametric = parametric, : at -0.005
```

```
## Warning in simpleLoess(y, x, w, span, degree = degree, parametric = parametric, : radius 2.5e-05
```

```
## Warning in simpleLoess(y, x, w, span, degree = degree, parametric = parametric, : all data on boundary of neighborhood. make span bigger
```

```
## Warning in simpleLoess(y, x, w, span, degree = degree, parametric = parametric, : pseudoinverse used at -0.005
```

```
## Warning in simpleLoess(y, x, w, span, degree = degree, parametric = parametric, : neighborhood radius 0.005
```

```
## Warning in simpleLoess(y, x, w, span, degree = degree, parametric = parametric, : reciprocal condition number 1
```

```
## Warning in simpleLoess(y, x, w, span, degree = degree, parametric = parametric, : There are other near singularities as well. 1.01
```

```
## Warning in simpleLoess(y, x, w, span, degree = degree, parametric = parametric, : zero-width neighborhood. make span bigger
```

```
## Warning: Computation failed in `stat_smooth()`:
## NA/NaN/Inf in foreign function call (arg 5)
```

```
## Warning in simpleLoess(y, x, w, span, degree = degree, parametric = parametric, : at -0.005
```

```
## Warning in simpleLoess(y, x, w, span, degree = degree, parametric = parametric, : radius 2.5e-05
```

```
## Warning in simpleLoess(y, x, w, span, degree = degree, parametric = parametric, : all data on boundary of neighborhood. make span bigger
```

```
## Warning in simpleLoess(y, x, w, span, degree = degree, parametric = parametric, : pseudoinverse used at -0.005
```

```
## Warning in simpleLoess(y, x, w, span, degree = degree, parametric = parametric, : neighborhood radius 0.005
```

```
## Warning in simpleLoess(y, x, w, span, degree = degree, parametric = parametric, : reciprocal condition number 1
```

```
## Warning in simpleLoess(y, x, w, span, degree = degree, parametric = parametric, : There are other near singularities as well. 1.01
```

```
## Warning in simpleLoess(y, x, w, span, degree = degree, parametric = parametric, : zero-width neighborhood. make span bigger
```

```
## Warning: Computation failed in `stat_smooth()`:
## NA/NaN/Inf in foreign function call (arg 5)
```

```
## Warning in simpleLoess(y, x, w, span, degree = degree, parametric = parametric, : at -0.005
```

```
## Warning in simpleLoess(y, x, w, span, degree = degree, parametric = parametric, : radius 2.5e-05
```

```
## Warning in simpleLoess(y, x, w, span, degree = degree, parametric = parametric, : all data on boundary of neighborhood. make span bigger
```

```
## Warning in simpleLoess(y, x, w, span, degree = degree, parametric = parametric, : pseudoinverse used at -0.005
```

```
## Warning in simpleLoess(y, x, w, span, degree = degree, parametric = parametric, : neighborhood radius 0.005
```

```
## Warning in simpleLoess(y, x, w, span, degree = degree, parametric = parametric, : reciprocal condition number 1
```

```
## Warning in simpleLoess(y, x, w, span, degree = degree, parametric = parametric, : There are other near singularities as well. 1.01
```

```
## Warning in simpleLoess(y, x, w, span, degree = degree, parametric = parametric, : zero-width neighborhood. make span bigger
```

```
## Warning: Computation failed in `stat_smooth()`:
## NA/NaN/Inf in foreign function call (arg 5)
```

```
## Warning in simpleLoess(y, x, w, span, degree = degree, parametric = parametric, : at -0.005
```

```
## Warning in simpleLoess(y, x, w, span, degree = degree, parametric = parametric, : radius 2.5e-05
```

```
## Warning in simpleLoess(y, x, w, span, degree = degree, parametric = parametric, : all data on boundary of neighborhood. make span bigger
```

```
## Warning in simpleLoess(y, x, w, span, degree = degree, parametric = parametric, : pseudoinverse used at -0.005
```

```
## Warning in simpleLoess(y, x, w, span, degree = degree, parametric = parametric, : neighborhood radius 0.005
```

```
## Warning in simpleLoess(y, x, w, span, degree = degree, parametric = parametric, : reciprocal condition number 1
```

```
## Warning in simpleLoess(y, x, w, span, degree = degree, parametric = parametric, : There are other near singularities as well. 1.01
```

```
## Warning in simpleLoess(y, x, w, span, degree = degree, parametric = parametric, : zero-width neighborhood. make span bigger
```

```
## Warning: Computation failed in `stat_smooth()`:
## NA/NaN/Inf in foreign function call (arg 5)
```

```
## Warning in simpleLoess(y, x, w, span, degree = degree, parametric = parametric, : at -0.005
```

```
## Warning in simpleLoess(y, x, w, span, degree = degree, parametric = parametric, : radius 2.5e-05
```

```
## Warning in simpleLoess(y, x, w, span, degree = degree, parametric = parametric, : all data on boundary of neighborhood. make span bigger
```

```
## Warning in simpleLoess(y, x, w, span, degree = degree, parametric = parametric, : pseudoinverse used at -0.005
```

```
## Warning in simpleLoess(y, x, w, span, degree = degree, parametric = parametric, : neighborhood radius 0.005
```

```
## Warning in simpleLoess(y, x, w, span, degree = degree, parametric = parametric, : reciprocal condition number 1
```

```
## Warning in simpleLoess(y, x, w, span, degree = degree, parametric = parametric, : There are other near singularities as well. 1.01
```

```
## Warning in simpleLoess(y, x, w, span, degree = degree, parametric = parametric, : zero-width neighborhood. make span bigger
```

```
## Warning: Computation failed in `stat_smooth()`:
## NA/NaN/Inf in foreign function call (arg 5)
```

```
## Warning in simpleLoess(y, x, w, span, degree = degree, parametric = parametric, : at -0.005
```

```
## Warning in simpleLoess(y, x, w, span, degree = degree, parametric = parametric, : radius 2.5e-05
```

```
## Warning in simpleLoess(y, x, w, span, degree = degree, parametric = parametric, : all data on boundary of neighborhood. make span bigger
```

```
## Warning in simpleLoess(y, x, w, span, degree = degree, parametric = parametric, : pseudoinverse used at -0.005
```

```
## Warning in simpleLoess(y, x, w, span, degree = degree, parametric = parametric, : neighborhood radius 0.005
```

```
## Warning in simpleLoess(y, x, w, span, degree = degree, parametric = parametric, : reciprocal condition number 1
```

```
## Warning in simpleLoess(y, x, w, span, degree = degree, parametric = parametric, : There are other near singularities as well. 1.01
```

```
## Warning in simpleLoess(y, x, w, span, degree = degree, parametric = parametric, : zero-width neighborhood. make span bigger
```

```
## Warning: Computation failed in `stat_smooth()`:
## NA/NaN/Inf in foreign function call (arg 5)
```

```
## Warning in simpleLoess(y, x, w, span, degree = degree, parametric = parametric, : at -0.005
```

```
## Warning in simpleLoess(y, x, w, span, degree = degree, parametric = parametric, : radius 2.5e-05
```

```
## Warning in simpleLoess(y, x, w, span, degree = degree, parametric = parametric, : all data on boundary of neighborhood. make span bigger
```

```
## Warning in simpleLoess(y, x, w, span, degree = degree, parametric = parametric, : pseudoinverse used at -0.005
```

```
## Warning in simpleLoess(y, x, w, span, degree = degree, parametric = parametric, : neighborhood radius 0.005
```

```
## Warning in simpleLoess(y, x, w, span, degree = degree, parametric = parametric, : reciprocal condition number 1
```

```
## Warning in simpleLoess(y, x, w, span, degree = degree, parametric = parametric, : There are other near singularities as well. 1.01
```

```
## Warning in simpleLoess(y, x, w, span, degree = degree, parametric = parametric, : zero-width neighborhood. make span bigger
```

```
## Warning: Computation failed in `stat_smooth()`:
## NA/NaN/Inf in foreign function call (arg 5)
```

```
## Warning in simpleLoess(y, x, w, span, degree = degree, parametric = parametric, : pseudoinverse used at -0.005
```

```
## Warning in simpleLoess(y, x, w, span, degree = degree, parametric = parametric, : neighborhood radius 1.005
```

```
## Warning in simpleLoess(y, x, w, span, degree = degree, parametric = parametric, : reciprocal condition number 0
```

```
## Warning in simpleLoess(y, x, w, span, degree = degree, parametric = parametric, : There are other near singularities as well. 1.01
```

```
## Warning in predLoess(object$y, object$x, newx = if (is.null(newdata)) object$x else if (is.data.frame(newdata)) as.matrix(model.frame(delete.response(terms(object)), : pseudoinverse used at -0.005
```

```
## Warning in predLoess(object$y, object$x, newx = if (is.null(newdata)) object$x else if (is.data.frame(newdata)) as.matrix(model.frame(delete.response(terms(object)), : neighborhood radius 1.005
```

```
## Warning in predLoess(object$y, object$x, newx = if (is.null(newdata)) object$x else if (is.data.frame(newdata)) as.matrix(model.frame(delete.response(terms(object)), : reciprocal condition number 0
```

```
## Warning in predLoess(object$y, object$x, newx = if (is.null(newdata)) object$x else if (is.data.frame(newdata)) as.matrix(model.frame(delete.response(terms(object)), : There are other near
## singularities as well. 1.01
```

```
## Warning in simpleLoess(y, x, w, span, degree = degree, parametric = parametric, : at -0.005
```

```
## Warning in simpleLoess(y, x, w, span, degree = degree, parametric = parametric, : radius 2.5e-05
```

```
## Warning in simpleLoess(y, x, w, span, degree = degree, parametric = parametric, : all data on boundary of neighborhood. make span bigger
```

```
## Warning in simpleLoess(y, x, w, span, degree = degree, parametric = parametric, : pseudoinverse used at -0.005
```

```
## Warning in simpleLoess(y, x, w, span, degree = degree, parametric = parametric, : neighborhood radius 0.005
```

```
## Warning in simpleLoess(y, x, w, span, degree = degree, parametric = parametric, : reciprocal condition number 1
```

```
## Warning in simpleLoess(y, x, w, span, degree = degree, parametric = parametric, : There are other near singularities as well. 1.01
```

```
## Warning in simpleLoess(y, x, w, span, degree = degree, parametric = parametric, : zero-width neighborhood. make span bigger
```

```
## Warning: Computation failed in `stat_smooth()`:
## NA/NaN/Inf in foreign function call (arg 5)
```

```
## Warning in simpleLoess(y, x, w, span, degree = degree, parametric = parametric, : at -0.005
```

```
## Warning in simpleLoess(y, x, w, span, degree = degree, parametric = parametric, : radius 2.5e-05
```

```
## Warning in simpleLoess(y, x, w, span, degree = degree, parametric = parametric, : all data on boundary of neighborhood. make span bigger
```

```
## Warning in simpleLoess(y, x, w, span, degree = degree, parametric = parametric, : pseudoinverse used at -0.005
```

```
## Warning in simpleLoess(y, x, w, span, degree = degree, parametric = parametric, : neighborhood radius 0.005
```

```
## Warning in simpleLoess(y, x, w, span, degree = degree, parametric = parametric, : reciprocal condition number 1
```

```
## Warning in simpleLoess(y, x, w, span, degree = degree, parametric = parametric, : There are other near singularities as well. 1.01
```

```
## Warning in simpleLoess(y, x, w, span, degree = degree, parametric = parametric, : zero-width neighborhood. make span bigger
```

```
## Warning: Computation failed in `stat_smooth()`:
## NA/NaN/Inf in foreign function call (arg 5)
```

```
## Warning in simpleLoess(y, x, w, span, degree = degree, parametric = parametric, : at -0.005
```

```
## Warning in simpleLoess(y, x, w, span, degree = degree, parametric = parametric, : radius 2.5e-05
```

```
## Warning in simpleLoess(y, x, w, span, degree = degree, parametric = parametric, : all data on boundary of neighborhood. make span bigger
```

```
## Warning in simpleLoess(y, x, w, span, degree = degree, parametric = parametric, : pseudoinverse used at -0.005
```

```
## Warning in simpleLoess(y, x, w, span, degree = degree, parametric = parametric, : neighborhood radius 0.005
```

```
## Warning in simpleLoess(y, x, w, span, degree = degree, parametric = parametric, : reciprocal condition number 1
```

```
## Warning in simpleLoess(y, x, w, span, degree = degree, parametric = parametric, : There are other near singularities as well. 1.01
```

```
## Warning in simpleLoess(y, x, w, span, degree = degree, parametric = parametric, : zero-width neighborhood. make span bigger
```

```
## Warning: Computation failed in `stat_smooth()`:
## NA/NaN/Inf in foreign function call (arg 5)
```

```
## Warning in simpleLoess(y, x, w, span, degree = degree, parametric = parametric, : at -0.005
```

```
## Warning in simpleLoess(y, x, w, span, degree = degree, parametric = parametric, : radius 2.5e-05
```

```
## Warning in simpleLoess(y, x, w, span, degree = degree, parametric = parametric, : all data on boundary of neighborhood. make span bigger
```

```
## Warning in simpleLoess(y, x, w, span, degree = degree, parametric = parametric, : pseudoinverse used at -0.005
```

```
## Warning in simpleLoess(y, x, w, span, degree = degree, parametric = parametric, : neighborhood radius 0.005
```

```
## Warning in simpleLoess(y, x, w, span, degree = degree, parametric = parametric, : reciprocal condition number 1
```

```
## Warning in simpleLoess(y, x, w, span, degree = degree, parametric = parametric, : There are other near singularities as well. 1.01
```

```
## Warning in simpleLoess(y, x, w, span, degree = degree, parametric = parametric, : zero-width neighborhood. make span bigger
```

```
## Warning: Computation failed in `stat_smooth()`:
## NA/NaN/Inf in foreign function call (arg 5)
```

```
## Warning in simpleLoess(y, x, w, span, degree = degree, parametric = parametric, : at -0.005
```

```
## Warning in simpleLoess(y, x, w, span, degree = degree, parametric = parametric, : radius 2.5e-05
```

```
## Warning in simpleLoess(y, x, w, span, degree = degree, parametric = parametric, : all data on boundary of neighborhood. make span bigger
```

```
## Warning in simpleLoess(y, x, w, span, degree = degree, parametric = parametric, : pseudoinverse used at -0.005
```

```
## Warning in simpleLoess(y, x, w, span, degree = degree, parametric = parametric, : neighborhood radius 0.005
```

```
## Warning in simpleLoess(y, x, w, span, degree = degree, parametric = parametric, : reciprocal condition number 1
```

```
## Warning in simpleLoess(y, x, w, span, degree = degree, parametric = parametric, : There are other near singularities as well. 1.01
```

```
## Warning in simpleLoess(y, x, w, span, degree = degree, parametric = parametric, : zero-width neighborhood. make span bigger
```

```
## Warning: Computation failed in `stat_smooth()`:
## NA/NaN/Inf in foreign function call (arg 5)
```

```
## Warning in simpleLoess(y, x, w, span, degree = degree, parametric = parametric, : at -0.005
```

```
## Warning in simpleLoess(y, x, w, span, degree = degree, parametric = parametric, : radius 2.5e-05
```

```
## Warning in simpleLoess(y, x, w, span, degree = degree, parametric = parametric, : all data on boundary of neighborhood. make span bigger
```

```
## Warning in simpleLoess(y, x, w, span, degree = degree, parametric = parametric, : pseudoinverse used at -0.005
```

```
## Warning in simpleLoess(y, x, w, span, degree = degree, parametric = parametric, : neighborhood radius 0.005
```

```
## Warning in simpleLoess(y, x, w, span, degree = degree, parametric = parametric, : reciprocal condition number 1
```

```
## Warning in simpleLoess(y, x, w, span, degree = degree, parametric = parametric, : There are other near singularities as well. 1.01
```

```
## Warning in simpleLoess(y, x, w, span, degree = degree, parametric = parametric, : zero-width neighborhood. make span bigger
```

```
## Warning: Computation failed in `stat_smooth()`:
## NA/NaN/Inf in foreign function call (arg 5)
```

```
## Warning in simpleLoess(y, x, w, span, degree = degree, parametric = parametric, : pseudoinverse used at -0.005
```

```
## Warning in simpleLoess(y, x, w, span, degree = degree, parametric = parametric, : neighborhood radius 1.005
```

```
## Warning in simpleLoess(y, x, w, span, degree = degree, parametric = parametric, : reciprocal condition number 0
```

```
## Warning in simpleLoess(y, x, w, span, degree = degree, parametric = parametric, : There are other near singularities as well. 1.01
```

```
## Warning in predLoess(object$y, object$x, newx = if (is.null(newdata)) object$x else if (is.data.frame(newdata)) as.matrix(model.frame(delete.response(terms(object)), : pseudoinverse used at -0.005
```

```
## Warning in predLoess(object$y, object$x, newx = if (is.null(newdata)) object$x else if (is.data.frame(newdata)) as.matrix(model.frame(delete.response(terms(object)), : neighborhood radius 1.005
```

```
## Warning in predLoess(object$y, object$x, newx = if (is.null(newdata)) object$x else if (is.data.frame(newdata)) as.matrix(model.frame(delete.response(terms(object)), : reciprocal condition number 0
```

```
## Warning in predLoess(object$y, object$x, newx = if (is.null(newdata)) object$x else if (is.data.frame(newdata)) as.matrix(model.frame(delete.response(terms(object)), : There are other near
## singularities as well. 1.01
```

```
## Warning in simpleLoess(y, x, w, span, degree = degree, parametric = parametric, : at -0.005
```

```
## Warning in simpleLoess(y, x, w, span, degree = degree, parametric = parametric, : radius 2.5e-05
```

```
## Warning in simpleLoess(y, x, w, span, degree = degree, parametric = parametric, : all data on boundary of neighborhood. make span bigger
```

```
## Warning in simpleLoess(y, x, w, span, degree = degree, parametric = parametric, : pseudoinverse used at -0.005
```

```
## Warning in simpleLoess(y, x, w, span, degree = degree, parametric = parametric, : neighborhood radius 0.005
```

```
## Warning in simpleLoess(y, x, w, span, degree = degree, parametric = parametric, : reciprocal condition number 1
```

```
## Warning in simpleLoess(y, x, w, span, degree = degree, parametric = parametric, : There are other near singularities as well. 1.01
```

```
## Warning in simpleLoess(y, x, w, span, degree = degree, parametric = parametric, : zero-width neighborhood. make span bigger
```

```
## Warning: Computation failed in `stat_smooth()`:
## NA/NaN/Inf in foreign function call (arg 5)
```

```
## Warning in simpleLoess(y, x, w, span, degree = degree, parametric = parametric, : at -0.005
```

```
## Warning in simpleLoess(y, x, w, span, degree = degree, parametric = parametric, : radius 2.5e-05
```

```
## Warning in simpleLoess(y, x, w, span, degree = degree, parametric = parametric, : all data on boundary of neighborhood. make span bigger
```

```
## Warning in simpleLoess(y, x, w, span, degree = degree, parametric = parametric, : pseudoinverse used at -0.005
```

```
## Warning in simpleLoess(y, x, w, span, degree = degree, parametric = parametric, : neighborhood radius 0.005
```

```
## Warning in simpleLoess(y, x, w, span, degree = degree, parametric = parametric, : reciprocal condition number 1
```

```
## Warning in simpleLoess(y, x, w, span, degree = degree, parametric = parametric, : There are other near singularities as well. 1.01
```

```
## Warning in simpleLoess(y, x, w, span, degree = degree, parametric = parametric, : zero-width neighborhood. make span bigger
```

```
## Warning: Computation failed in `stat_smooth()`:
## NA/NaN/Inf in foreign function call (arg 5)
```

```
## Warning in simpleLoess(y, x, w, span, degree = degree, parametric = parametric, : at -0.005
```

```
## Warning in simpleLoess(y, x, w, span, degree = degree, parametric = parametric, : radius 2.5e-05
```

```
## Warning in simpleLoess(y, x, w, span, degree = degree, parametric = parametric, : all data on boundary of neighborhood. make span bigger
```

```
## Warning in simpleLoess(y, x, w, span, degree = degree, parametric = parametric, : pseudoinverse used at -0.005
```

```
## Warning in simpleLoess(y, x, w, span, degree = degree, parametric = parametric, : neighborhood radius 0.005
```

```
## Warning in simpleLoess(y, x, w, span, degree = degree, parametric = parametric, : reciprocal condition number 1
```

```
## Warning in simpleLoess(y, x, w, span, degree = degree, parametric = parametric, : There are other near singularities as well. 1.01
```

```
## Warning in simpleLoess(y, x, w, span, degree = degree, parametric = parametric, : zero-width neighborhood. make span bigger
```

```
## Warning: Computation failed in `stat_smooth()`:
## NA/NaN/Inf in foreign function call (arg 5)
```

```
## Warning in simpleLoess(y, x, w, span, degree = degree, parametric = parametric, : pseudoinverse used at -0.005
```

```
## Warning in simpleLoess(y, x, w, span, degree = degree, parametric = parametric, : neighborhood radius 1.005
```

```
## Warning in simpleLoess(y, x, w, span, degree = degree, parametric = parametric, : reciprocal condition number 0
```

```
## Warning in simpleLoess(y, x, w, span, degree = degree, parametric = parametric, : There are other near singularities as well. 1.01
```

```
## Warning in predLoess(object$y, object$x, newx = if (is.null(newdata)) object$x else if (is.data.frame(newdata)) as.matrix(model.frame(delete.response(terms(object)), : pseudoinverse used at -0.005
```

```
## Warning in predLoess(object$y, object$x, newx = if (is.null(newdata)) object$x else if (is.data.frame(newdata)) as.matrix(model.frame(delete.response(terms(object)), : neighborhood radius 1.005
```

```
## Warning in predLoess(object$y, object$x, newx = if (is.null(newdata)) object$x else if (is.data.frame(newdata)) as.matrix(model.frame(delete.response(terms(object)), : reciprocal condition number 0
```

```
## Warning in predLoess(object$y, object$x, newx = if (is.null(newdata)) object$x else if (is.data.frame(newdata)) as.matrix(model.frame(delete.response(terms(object)), : There are other near
## singularities as well. 1.01
```

```
## Warning in simpleLoess(y, x, w, span, degree = degree, parametric = parametric, : at -0.005
```

```
## Warning in simpleLoess(y, x, w, span, degree = degree, parametric = parametric, : radius 2.5e-05
```

```
## Warning in simpleLoess(y, x, w, span, degree = degree, parametric = parametric, : all data on boundary of neighborhood. make span bigger
```

```
## Warning in simpleLoess(y, x, w, span, degree = degree, parametric = parametric, : pseudoinverse used at -0.005
```

```
## Warning in simpleLoess(y, x, w, span, degree = degree, parametric = parametric, : neighborhood radius 0.005
```

```
## Warning in simpleLoess(y, x, w, span, degree = degree, parametric = parametric, : reciprocal condition number 1
```

```
## Warning in simpleLoess(y, x, w, span, degree = degree, parametric = parametric, : There are other near singularities as well. 1.01
```

```
## Warning in simpleLoess(y, x, w, span, degree = degree, parametric = parametric, : zero-width neighborhood. make span bigger
```

```
## Warning: Computation failed in `stat_smooth()`:
## NA/NaN/Inf in foreign function call (arg 5)
```

```
## Warning in simpleLoess(y, x, w, span, degree = degree, parametric = parametric, : at -0.005
```

```
## Warning in simpleLoess(y, x, w, span, degree = degree, parametric = parametric, : radius 2.5e-05
```

```
## Warning in simpleLoess(y, x, w, span, degree = degree, parametric = parametric, : all data on boundary of neighborhood. make span bigger
```

```
## Warning in simpleLoess(y, x, w, span, degree = degree, parametric = parametric, : pseudoinverse used at -0.005
```

```
## Warning in simpleLoess(y, x, w, span, degree = degree, parametric = parametric, : neighborhood radius 0.005
```

```
## Warning in simpleLoess(y, x, w, span, degree = degree, parametric = parametric, : reciprocal condition number 1
```

```
## Warning in simpleLoess(y, x, w, span, degree = degree, parametric = parametric, : There are other near singularities as well. 1.01
```

```
## Warning in simpleLoess(y, x, w, span, degree = degree, parametric = parametric, : zero-width neighborhood. make span bigger
```

```
## Warning: Computation failed in `stat_smooth()`:
## NA/NaN/Inf in foreign function call (arg 5)
```

```
## Warning in simpleLoess(y, x, w, span, degree = degree, parametric = parametric, : pseudoinverse used at -0.005
```

```
## Warning in simpleLoess(y, x, w, span, degree = degree, parametric = parametric, : neighborhood radius 1.005
```

```
## Warning in simpleLoess(y, x, w, span, degree = degree, parametric = parametric, : reciprocal condition number 0
```

```
## Warning in simpleLoess(y, x, w, span, degree = degree, parametric = parametric, : There are other near singularities as well. 1.01
```

```
## Warning in predLoess(object$y, object$x, newx = if (is.null(newdata)) object$x else if (is.data.frame(newdata)) as.matrix(model.frame(delete.response(terms(object)), : pseudoinverse used at -0.005
```

```
## Warning in predLoess(object$y, object$x, newx = if (is.null(newdata)) object$x else if (is.data.frame(newdata)) as.matrix(model.frame(delete.response(terms(object)), : neighborhood radius 1.005
```

```
## Warning in predLoess(object$y, object$x, newx = if (is.null(newdata)) object$x else if (is.data.frame(newdata)) as.matrix(model.frame(delete.response(terms(object)), : reciprocal condition number 0
```

```
## Warning in predLoess(object$y, object$x, newx = if (is.null(newdata)) object$x else if (is.data.frame(newdata)) as.matrix(model.frame(delete.response(terms(object)), : There are other near
## singularities as well. 1.01
```

```
## Warning in simpleLoess(y, x, w, span, degree = degree, parametric = parametric, : at -0.005
```

```
## Warning in simpleLoess(y, x, w, span, degree = degree, parametric = parametric, : radius 2.5e-05
```

```
## Warning in simpleLoess(y, x, w, span, degree = degree, parametric = parametric, : all data on boundary of neighborhood. make span bigger
```

```
## Warning in simpleLoess(y, x, w, span, degree = degree, parametric = parametric, : pseudoinverse used at -0.005
```

```
## Warning in simpleLoess(y, x, w, span, degree = degree, parametric = parametric, : neighborhood radius 0.005
```

```
## Warning in simpleLoess(y, x, w, span, degree = degree, parametric = parametric, : reciprocal condition number 1
```

```
## Warning in simpleLoess(y, x, w, span, degree = degree, parametric = parametric, : There are other near singularities as well. 1.01
```

```
## Warning in simpleLoess(y, x, w, span, degree = degree, parametric = parametric, : zero-width neighborhood. make span bigger
```

```
## Warning: Computation failed in `stat_smooth()`:
## NA/NaN/Inf in foreign function call (arg 5)
```

```
## Warning in simpleLoess(y, x, w, span, degree = degree, parametric = parametric, : pseudoinverse used at -0.005
```

```
## Warning in simpleLoess(y, x, w, span, degree = degree, parametric = parametric, : neighborhood radius 1.005
```

```
## Warning in simpleLoess(y, x, w, span, degree = degree, parametric = parametric, : reciprocal condition number 0
```

```
## Warning in simpleLoess(y, x, w, span, degree = degree, parametric = parametric, : There are other near singularities as well. 1.01
```

```
## Warning in predLoess(object$y, object$x, newx = if (is.null(newdata)) object$x else if (is.data.frame(newdata)) as.matrix(model.frame(delete.response(terms(object)), : pseudoinverse used at -0.005
```

```
## Warning in predLoess(object$y, object$x, newx = if (is.null(newdata)) object$x else if (is.data.frame(newdata)) as.matrix(model.frame(delete.response(terms(object)), : neighborhood radius 1.005
```

```
## Warning in predLoess(object$y, object$x, newx = if (is.null(newdata)) object$x else if (is.data.frame(newdata)) as.matrix(model.frame(delete.response(terms(object)), : reciprocal condition number 0
```

```
## Warning in predLoess(object$y, object$x, newx = if (is.null(newdata)) object$x else if (is.data.frame(newdata)) as.matrix(model.frame(delete.response(terms(object)), : There are other near
## singularities as well. 1.01
```

```
## Warning in simpleLoess(y, x, w, span, degree = degree, parametric = parametric, : at -0.005
```

```
## Warning in simpleLoess(y, x, w, span, degree = degree, parametric = parametric, : radius 2.5e-05
```

```
## Warning in simpleLoess(y, x, w, span, degree = degree, parametric = parametric, : all data on boundary of neighborhood. make span bigger
```

```
## Warning in simpleLoess(y, x, w, span, degree = degree, parametric = parametric, : pseudoinverse used at -0.005
```

```
## Warning in simpleLoess(y, x, w, span, degree = degree, parametric = parametric, : neighborhood radius 0.005
```

```
## Warning in simpleLoess(y, x, w, span, degree = degree, parametric = parametric, : reciprocal condition number 1
```

```
## Warning in simpleLoess(y, x, w, span, degree = degree, parametric = parametric, : There are other near singularities as well. 1.01
```

```
## Warning in simpleLoess(y, x, w, span, degree = degree, parametric = parametric, : zero-width neighborhood. make span bigger
```

```
## Warning: Computation failed in `stat_smooth()`:
## NA/NaN/Inf in foreign function call (arg 5)
```

```
## Warning in simpleLoess(y, x, w, span, degree = degree, parametric = parametric, : at -0.005
```

```
## Warning in simpleLoess(y, x, w, span, degree = degree, parametric = parametric, : radius 2.5e-05
```

```
## Warning in simpleLoess(y, x, w, span, degree = degree, parametric = parametric, : all data on boundary of neighborhood. make span bigger
```

```
## Warning in simpleLoess(y, x, w, span, degree = degree, parametric = parametric, : pseudoinverse used at -0.005
```

```
## Warning in simpleLoess(y, x, w, span, degree = degree, parametric = parametric, : neighborhood radius 0.005
```

```
## Warning in simpleLoess(y, x, w, span, degree = degree, parametric = parametric, : reciprocal condition number 1
```

```
## Warning in simpleLoess(y, x, w, span, degree = degree, parametric = parametric, : There are other near singularities as well. 1.01
```

```
## Warning in simpleLoess(y, x, w, span, degree = degree, parametric = parametric, : zero-width neighborhood. make span bigger
```

```
## Warning: Computation failed in `stat_smooth()`:
## NA/NaN/Inf in foreign function call (arg 5)
```

```
## Warning in simpleLoess(y, x, w, span, degree = degree, parametric = parametric, : at -0.005
```

```
## Warning in simpleLoess(y, x, w, span, degree = degree, parametric = parametric, : radius 2.5e-05
```

```
## Warning in simpleLoess(y, x, w, span, degree = degree, parametric = parametric, : all data on boundary of neighborhood. make span bigger
```

```
## Warning in simpleLoess(y, x, w, span, degree = degree, parametric = parametric, : pseudoinverse used at -0.005
```

```
## Warning in simpleLoess(y, x, w, span, degree = degree, parametric = parametric, : neighborhood radius 0.005
```

```
## Warning in simpleLoess(y, x, w, span, degree = degree, parametric = parametric, : reciprocal condition number 1
```

```
## Warning in simpleLoess(y, x, w, span, degree = degree, parametric = parametric, : There are other near singularities as well. 1.01
```

```
## Warning in simpleLoess(y, x, w, span, degree = degree, parametric = parametric, : zero-width neighborhood. make span bigger
```

```
## Warning: Computation failed in `stat_smooth()`:
## NA/NaN/Inf in foreign function call (arg 5)
```

```
## Warning in simpleLoess(y, x, w, span, degree = degree, parametric = parametric, : at -0.005
```

```
## Warning in simpleLoess(y, x, w, span, degree = degree, parametric = parametric, : radius 2.5e-05
```

```
## Warning in simpleLoess(y, x, w, span, degree = degree, parametric = parametric, : all data on boundary of neighborhood. make span bigger
```

```
## Warning in simpleLoess(y, x, w, span, degree = degree, parametric = parametric, : pseudoinverse used at -0.005
```

```
## Warning in simpleLoess(y, x, w, span, degree = degree, parametric = parametric, : neighborhood radius 0.005
```

```
## Warning in simpleLoess(y, x, w, span, degree = degree, parametric = parametric, : reciprocal condition number 1
```

```
## Warning in simpleLoess(y, x, w, span, degree = degree, parametric = parametric, : There are other near singularities as well. 1.01
```

```
## Warning in simpleLoess(y, x, w, span, degree = degree, parametric = parametric, : zero-width neighborhood. make span bigger
```

```
## Warning: Computation failed in `stat_smooth()`:
## NA/NaN/Inf in foreign function call (arg 5)
```

```
## Warning in simpleLoess(y, x, w, span, degree = degree, parametric = parametric, : pseudoinverse used at -0.005
```

```
## Warning in simpleLoess(y, x, w, span, degree = degree, parametric = parametric, : neighborhood radius 1.005
```

```
## Warning in simpleLoess(y, x, w, span, degree = degree, parametric = parametric, : reciprocal condition number 0
```

```
## Warning in simpleLoess(y, x, w, span, degree = degree, parametric = parametric, : There are other near singularities as well. 1.01
```

```
## Warning in predLoess(object$y, object$x, newx = if (is.null(newdata)) object$x else if (is.data.frame(newdata)) as.matrix(model.frame(delete.response(terms(object)), : pseudoinverse used at -0.005
```

```
## Warning in predLoess(object$y, object$x, newx = if (is.null(newdata)) object$x else if (is.data.frame(newdata)) as.matrix(model.frame(delete.response(terms(object)), : neighborhood radius 1.005
```

```
## Warning in predLoess(object$y, object$x, newx = if (is.null(newdata)) object$x else if (is.data.frame(newdata)) as.matrix(model.frame(delete.response(terms(object)), : reciprocal condition number 0
```

```
## Warning in predLoess(object$y, object$x, newx = if (is.null(newdata)) object$x else if (is.data.frame(newdata)) as.matrix(model.frame(delete.response(terms(object)), : There are other near
## singularities as well. 1.01
```

```
## Warning in simpleLoess(y, x, w, span, degree = degree, parametric = parametric, : at -0.005
```

```
## Warning in simpleLoess(y, x, w, span, degree = degree, parametric = parametric, : radius 2.5e-05
```

```
## Warning in simpleLoess(y, x, w, span, degree = degree, parametric = parametric, : all data on boundary of neighborhood. make span bigger
```

```
## Warning in simpleLoess(y, x, w, span, degree = degree, parametric = parametric, : pseudoinverse used at -0.005
```

```
## Warning in simpleLoess(y, x, w, span, degree = degree, parametric = parametric, : neighborhood radius 0.005
```

```
## Warning in simpleLoess(y, x, w, span, degree = degree, parametric = parametric, : reciprocal condition number 1
```

```
## Warning in simpleLoess(y, x, w, span, degree = degree, parametric = parametric, : There are other near singularities as well. 1.01
```

```
## Warning in simpleLoess(y, x, w, span, degree = degree, parametric = parametric, : zero-width neighborhood. make span bigger
```

```
## Warning: Computation failed in `stat_smooth()`:
## NA/NaN/Inf in foreign function call (arg 5)
```

```
## Warning in simpleLoess(y, x, w, span, degree = degree, parametric = parametric, : at -0.005
```

```
## Warning in simpleLoess(y, x, w, span, degree = degree, parametric = parametric, : radius 2.5e-05
```

```
## Warning in simpleLoess(y, x, w, span, degree = degree, parametric = parametric, : all data on boundary of neighborhood. make span bigger
```

```
## Warning in simpleLoess(y, x, w, span, degree = degree, parametric = parametric, : pseudoinverse used at -0.005
```

```
## Warning in simpleLoess(y, x, w, span, degree = degree, parametric = parametric, : neighborhood radius 0.005
```

```
## Warning in simpleLoess(y, x, w, span, degree = degree, parametric = parametric, : reciprocal condition number 1
```

```
## Warning in simpleLoess(y, x, w, span, degree = degree, parametric = parametric, : There are other near singularities as well. 1.01
```

```
## Warning in simpleLoess(y, x, w, span, degree = degree, parametric = parametric, : zero-width neighborhood. make span bigger
```

```
## Warning: Computation failed in `stat_smooth()`:
## NA/NaN/Inf in foreign function call (arg 5)
```

```
## Warning in simpleLoess(y, x, w, span, degree = degree, parametric = parametric, : at -0.005
```

```
## Warning in simpleLoess(y, x, w, span, degree = degree, parametric = parametric, : radius 2.5e-05
```

```
## Warning in simpleLoess(y, x, w, span, degree = degree, parametric = parametric, : all data on boundary of neighborhood. make span bigger
```

```
## Warning in simpleLoess(y, x, w, span, degree = degree, parametric = parametric, : pseudoinverse used at -0.005
```

```
## Warning in simpleLoess(y, x, w, span, degree = degree, parametric = parametric, : neighborhood radius 0.005
```

```
## Warning in simpleLoess(y, x, w, span, degree = degree, parametric = parametric, : reciprocal condition number 1
```

```
## Warning in simpleLoess(y, x, w, span, degree = degree, parametric = parametric, : There are other near singularities as well. 1.01
```

```
## Warning in simpleLoess(y, x, w, span, degree = degree, parametric = parametric, : zero-width neighborhood. make span bigger
```

```
## Warning: Computation failed in `stat_smooth()`:
## NA/NaN/Inf in foreign function call (arg 5)
```

```
## Warning in simpleLoess(y, x, w, span, degree = degree, parametric = parametric, : at -0.005
```

```
## Warning in simpleLoess(y, x, w, span, degree = degree, parametric = parametric, : radius 2.5e-05
```

```
## Warning in simpleLoess(y, x, w, span, degree = degree, parametric = parametric, : all data on boundary of neighborhood. make span bigger
```

```
## Warning in simpleLoess(y, x, w, span, degree = degree, parametric = parametric, : pseudoinverse used at -0.005
```

```
## Warning in simpleLoess(y, x, w, span, degree = degree, parametric = parametric, : neighborhood radius 0.005
```

```
## Warning in simpleLoess(y, x, w, span, degree = degree, parametric = parametric, : reciprocal condition number 1
```

```
## Warning in simpleLoess(y, x, w, span, degree = degree, parametric = parametric, : There are other near singularities as well. 1.01
```

```
## Warning in simpleLoess(y, x, w, span, degree = degree, parametric = parametric, : zero-width neighborhood. make span bigger
```

```
## Warning: Computation failed in `stat_smooth()`:
## NA/NaN/Inf in foreign function call (arg 5)
```

```
## Warning in simpleLoess(y, x, w, span, degree = degree, parametric = parametric, : at -0.005
```

```
## Warning in simpleLoess(y, x, w, span, degree = degree, parametric = parametric, : radius 2.5e-05
```

```
## Warning in simpleLoess(y, x, w, span, degree = degree, parametric = parametric, : all data on boundary of neighborhood. make span bigger
```

```
## Warning in simpleLoess(y, x, w, span, degree = degree, parametric = parametric, : pseudoinverse used at -0.005
```

```
## Warning in simpleLoess(y, x, w, span, degree = degree, parametric = parametric, : neighborhood radius 0.005
```

```
## Warning in simpleLoess(y, x, w, span, degree = degree, parametric = parametric, : reciprocal condition number 1
```

```
## Warning in simpleLoess(y, x, w, span, degree = degree, parametric = parametric, : There are other near singularities as well. 1.01
```

```
## Warning in simpleLoess(y, x, w, span, degree = degree, parametric = parametric, : zero-width neighborhood. make span bigger
```

```
## Warning: Computation failed in `stat_smooth()`:
## NA/NaN/Inf in foreign function call (arg 5)
```

```
## Warning in simpleLoess(y, x, w, span, degree = degree, parametric = parametric, : at -0.005
```

```
## Warning in simpleLoess(y, x, w, span, degree = degree, parametric = parametric, : radius 2.5e-05
```

```
## Warning in simpleLoess(y, x, w, span, degree = degree, parametric = parametric, : all data on boundary of neighborhood. make span bigger
```

```
## Warning in simpleLoess(y, x, w, span, degree = degree, parametric = parametric, : pseudoinverse used at -0.005
```

```
## Warning in simpleLoess(y, x, w, span, degree = degree, parametric = parametric, : neighborhood radius 0.005
```

```
## Warning in simpleLoess(y, x, w, span, degree = degree, parametric = parametric, : reciprocal condition number 1
```

```
## Warning in simpleLoess(y, x, w, span, degree = degree, parametric = parametric, : There are other near singularities as well. 1.01
```

```
## Warning in simpleLoess(y, x, w, span, degree = degree, parametric = parametric, : zero-width neighborhood. make span bigger
```

```
## Warning: Computation failed in `stat_smooth()`:
## NA/NaN/Inf in foreign function call (arg 5)
```

```
## Warning in simpleLoess(y, x, w, span, degree = degree, parametric = parametric, : at -0.005
```

```
## Warning in simpleLoess(y, x, w, span, degree = degree, parametric = parametric, : radius 2.5e-05
```

```
## Warning in simpleLoess(y, x, w, span, degree = degree, parametric = parametric, : all data on boundary of neighborhood. make span bigger
```

```
## Warning in simpleLoess(y, x, w, span, degree = degree, parametric = parametric, : pseudoinverse used at -0.005
```

```
## Warning in simpleLoess(y, x, w, span, degree = degree, parametric = parametric, : neighborhood radius 0.005
```

```
## Warning in simpleLoess(y, x, w, span, degree = degree, parametric = parametric, : reciprocal condition number 1
```

```
## Warning in simpleLoess(y, x, w, span, degree = degree, parametric = parametric, : There are other near singularities as well. 1.01
```

```
## Warning in simpleLoess(y, x, w, span, degree = degree, parametric = parametric, : zero-width neighborhood. make span bigger
```

```
## Warning: Computation failed in `stat_smooth()`:
## NA/NaN/Inf in foreign function call (arg 5)
```

```
## Warning in simpleLoess(y, x, w, span, degree = degree, parametric = parametric, : at -0.005
```

```
## Warning in simpleLoess(y, x, w, span, degree = degree, parametric = parametric, : radius 2.5e-05
```

```
## Warning in simpleLoess(y, x, w, span, degree = degree, parametric = parametric, : all data on boundary of neighborhood. make span bigger
```

```
## Warning in simpleLoess(y, x, w, span, degree = degree, parametric = parametric, : pseudoinverse used at -0.005
```

```
## Warning in simpleLoess(y, x, w, span, degree = degree, parametric = parametric, : neighborhood radius 0.005
```

```
## Warning in simpleLoess(y, x, w, span, degree = degree, parametric = parametric, : reciprocal condition number 1
```

```
## Warning in simpleLoess(y, x, w, span, degree = degree, parametric = parametric, : There are other near singularities as well. 1.01
```

```
## Warning in simpleLoess(y, x, w, span, degree = degree, parametric = parametric, : zero-width neighborhood. make span bigger
```

```
## Warning: Computation failed in `stat_smooth()`:
## NA/NaN/Inf in foreign function call (arg 5)
```

```
## Warning in simpleLoess(y, x, w, span, degree = degree, parametric = parametric, : at -0.005
```

```
## Warning in simpleLoess(y, x, w, span, degree = degree, parametric = parametric, : radius 2.5e-05
```

```
## Warning in simpleLoess(y, x, w, span, degree = degree, parametric = parametric, : all data on boundary of neighborhood. make span bigger
```

```
## Warning in simpleLoess(y, x, w, span, degree = degree, parametric = parametric, : pseudoinverse used at -0.005
```

```
## Warning in simpleLoess(y, x, w, span, degree = degree, parametric = parametric, : neighborhood radius 0.005
```

```
## Warning in simpleLoess(y, x, w, span, degree = degree, parametric = parametric, : reciprocal condition number 1
```

```
## Warning in simpleLoess(y, x, w, span, degree = degree, parametric = parametric, : There are other near singularities as well. 1.01
```

```
## Warning in simpleLoess(y, x, w, span, degree = degree, parametric = parametric, : zero-width neighborhood. make span bigger
```

```
## Warning: Computation failed in `stat_smooth()`:
## NA/NaN/Inf in foreign function call (arg 5)
```

```
## Warning in simpleLoess(y, x, w, span, degree = degree, parametric = parametric, : at -0.005
```

```
## Warning in simpleLoess(y, x, w, span, degree = degree, parametric = parametric, : radius 2.5e-05
```

```
## Warning in simpleLoess(y, x, w, span, degree = degree, parametric = parametric, : all data on boundary of neighborhood. make span bigger
```

```
## Warning in simpleLoess(y, x, w, span, degree = degree, parametric = parametric, : pseudoinverse used at -0.005
```

```
## Warning in simpleLoess(y, x, w, span, degree = degree, parametric = parametric, : neighborhood radius 0.005
```

```
## Warning in simpleLoess(y, x, w, span, degree = degree, parametric = parametric, : reciprocal condition number 1
```

```
## Warning in simpleLoess(y, x, w, span, degree = degree, parametric = parametric, : There are other near singularities as well. 1.01
```

```
## Warning in simpleLoess(y, x, w, span, degree = degree, parametric = parametric, : zero-width neighborhood. make span bigger
```

```
## Warning: Computation failed in `stat_smooth()`:
## NA/NaN/Inf in foreign function call (arg 5)
```

```
## Warning in simpleLoess(y, x, w, span, degree = degree, parametric = parametric, : at -0.005
```

```
## Warning in simpleLoess(y, x, w, span, degree = degree, parametric = parametric, : radius 2.5e-05
```

```
## Warning in simpleLoess(y, x, w, span, degree = degree, parametric = parametric, : all data on boundary of neighborhood. make span bigger
```

```
## Warning in simpleLoess(y, x, w, span, degree = degree, parametric = parametric, : pseudoinverse used at -0.005
```

```
## Warning in simpleLoess(y, x, w, span, degree = degree, parametric = parametric, : neighborhood radius 0.005
```

```
## Warning in simpleLoess(y, x, w, span, degree = degree, parametric = parametric, : reciprocal condition number 1
```

```
## Warning in simpleLoess(y, x, w, span, degree = degree, parametric = parametric, : There are other near singularities as well. 1.01
```

```
## Warning in simpleLoess(y, x, w, span, degree = degree, parametric = parametric, : zero-width neighborhood. make span bigger
```

```
## Warning: Computation failed in `stat_smooth()`:
## NA/NaN/Inf in foreign function call (arg 5)
```

```
## Warning in simpleLoess(y, x, w, span, degree = degree, parametric = parametric, : pseudoinverse used at -0.005
```

```
## Warning in simpleLoess(y, x, w, span, degree = degree, parametric = parametric, : neighborhood radius 1.005
```

```
## Warning in simpleLoess(y, x, w, span, degree = degree, parametric = parametric, : reciprocal condition number 0
```

```
## Warning in simpleLoess(y, x, w, span, degree = degree, parametric = parametric, : There are other near singularities as well. 1.01
```

```
## Warning in predLoess(object$y, object$x, newx = if (is.null(newdata)) object$x else if (is.data.frame(newdata)) as.matrix(model.frame(delete.response(terms(object)), : pseudoinverse used at -0.005
```

```
## Warning in predLoess(object$y, object$x, newx = if (is.null(newdata)) object$x else if (is.data.frame(newdata)) as.matrix(model.frame(delete.response(terms(object)), : neighborhood radius 1.005
```

```
## Warning in predLoess(object$y, object$x, newx = if (is.null(newdata)) object$x else if (is.data.frame(newdata)) as.matrix(model.frame(delete.response(terms(object)), : reciprocal condition number 0
```

```
## Warning in predLoess(object$y, object$x, newx = if (is.null(newdata)) object$x else if (is.data.frame(newdata)) as.matrix(model.frame(delete.response(terms(object)), : There are other near
## singularities as well. 1.01
```

```
## Warning in simpleLoess(y, x, w, span, degree = degree, parametric = parametric, : at -0.005
```

```
## Warning in simpleLoess(y, x, w, span, degree = degree, parametric = parametric, : radius 2.5e-05
```

```
## Warning in simpleLoess(y, x, w, span, degree = degree, parametric = parametric, : all data on boundary of neighborhood. make span bigger
```

```
## Warning in simpleLoess(y, x, w, span, degree = degree, parametric = parametric, : pseudoinverse used at -0.005
```

```
## Warning in simpleLoess(y, x, w, span, degree = degree, parametric = parametric, : neighborhood radius 0.005
```

```
## Warning in simpleLoess(y, x, w, span, degree = degree, parametric = parametric, : reciprocal condition number 1
```

```
## Warning in simpleLoess(y, x, w, span, degree = degree, parametric = parametric, : There are other near singularities as well. 1.01
```

```
## Warning in simpleLoess(y, x, w, span, degree = degree, parametric = parametric, : zero-width neighborhood. make span bigger
```

```
## Warning: Computation failed in `stat_smooth()`:
## NA/NaN/Inf in foreign function call (arg 5)
```

```
## Warning in simpleLoess(y, x, w, span, degree = degree, parametric = parametric, : at -0.005
```

```
## Warning in simpleLoess(y, x, w, span, degree = degree, parametric = parametric, : radius 2.5e-05
```

```
## Warning in simpleLoess(y, x, w, span, degree = degree, parametric = parametric, : all data on boundary of neighborhood. make span bigger
```

```
## Warning in simpleLoess(y, x, w, span, degree = degree, parametric = parametric, : pseudoinverse used at -0.005
```

```
## Warning in simpleLoess(y, x, w, span, degree = degree, parametric = parametric, : neighborhood radius 0.005
```

```
## Warning in simpleLoess(y, x, w, span, degree = degree, parametric = parametric, : reciprocal condition number 1
```

```
## Warning in simpleLoess(y, x, w, span, degree = degree, parametric = parametric, : There are other near singularities as well. 1.01
```

```
## Warning in simpleLoess(y, x, w, span, degree = degree, parametric = parametric, : zero-width neighborhood. make span bigger
```

```
## Warning: Computation failed in `stat_smooth()`:
## NA/NaN/Inf in foreign function call (arg 5)
```

```
## Warning in simpleLoess(y, x, w, span, degree = degree, parametric = parametric, : pseudoinverse used at -0.005
```

```
## Warning in simpleLoess(y, x, w, span, degree = degree, parametric = parametric, : neighborhood radius 1.005
```

```
## Warning in simpleLoess(y, x, w, span, degree = degree, parametric = parametric, : reciprocal condition number 0
```

```
## Warning in simpleLoess(y, x, w, span, degree = degree, parametric = parametric, : There are other near singularities as well. 1.01
```

```
## Warning in predLoess(object$y, object$x, newx = if (is.null(newdata)) object$x else if (is.data.frame(newdata)) as.matrix(model.frame(delete.response(terms(object)), : pseudoinverse used at -0.005
```

```
## Warning in predLoess(object$y, object$x, newx = if (is.null(newdata)) object$x else if (is.data.frame(newdata)) as.matrix(model.frame(delete.response(terms(object)), : neighborhood radius 1.005
```

```
## Warning in predLoess(object$y, object$x, newx = if (is.null(newdata)) object$x else if (is.data.frame(newdata)) as.matrix(model.frame(delete.response(terms(object)), : reciprocal condition number 0
```

```
## Warning in predLoess(object$y, object$x, newx = if (is.null(newdata)) object$x else if (is.data.frame(newdata)) as.matrix(model.frame(delete.response(terms(object)), : There are other near
## singularities as well. 1.01
```

```
## Warning in simpleLoess(y, x, w, span, degree = degree, parametric = parametric, : at -0.005
```

```
## Warning in simpleLoess(y, x, w, span, degree = degree, parametric = parametric, : radius 2.5e-05
```

```
## Warning in simpleLoess(y, x, w, span, degree = degree, parametric = parametric, : all data on boundary of neighborhood. make span bigger
```

```
## Warning in simpleLoess(y, x, w, span, degree = degree, parametric = parametric, : pseudoinverse used at -0.005
```

```
## Warning in simpleLoess(y, x, w, span, degree = degree, parametric = parametric, : neighborhood radius 0.005
```

```
## Warning in simpleLoess(y, x, w, span, degree = degree, parametric = parametric, : reciprocal condition number 1
```

```
## Warning in simpleLoess(y, x, w, span, degree = degree, parametric = parametric, : There are other near singularities as well. 1.01
```

```
## Warning in simpleLoess(y, x, w, span, degree = degree, parametric = parametric, : zero-width neighborhood. make span bigger
```

```
## Warning: Computation failed in `stat_smooth()`:
## NA/NaN/Inf in foreign function call (arg 5)
```

```
## Warning in simpleLoess(y, x, w, span, degree = degree, parametric = parametric, : at -0.005
```

```
## Warning in simpleLoess(y, x, w, span, degree = degree, parametric = parametric, : radius 2.5e-05
```

```
## Warning in simpleLoess(y, x, w, span, degree = degree, parametric = parametric, : all data on boundary of neighborhood. make span bigger
```

```
## Warning in simpleLoess(y, x, w, span, degree = degree, parametric = parametric, : pseudoinverse used at -0.005
```

```
## Warning in simpleLoess(y, x, w, span, degree = degree, parametric = parametric, : neighborhood radius 0.005
```

```
## Warning in simpleLoess(y, x, w, span, degree = degree, parametric = parametric, : reciprocal condition number 1
```

```
## Warning in simpleLoess(y, x, w, span, degree = degree, parametric = parametric, : There are other near singularities as well. 1.01
```

```
## Warning in simpleLoess(y, x, w, span, degree = degree, parametric = parametric, : zero-width neighborhood. make span bigger
```

```
## Warning: Computation failed in `stat_smooth()`:
## NA/NaN/Inf in foreign function call (arg 5)
```

```
## Warning in simpleLoess(y, x, w, span, degree = degree, parametric = parametric, : at -0.005
```

```
## Warning in simpleLoess(y, x, w, span, degree = degree, parametric = parametric, : radius 2.5e-05
```

```
## Warning in simpleLoess(y, x, w, span, degree = degree, parametric = parametric, : all data on boundary of neighborhood. make span bigger
```

```
## Warning in simpleLoess(y, x, w, span, degree = degree, parametric = parametric, : pseudoinverse used at -0.005
```

```
## Warning in simpleLoess(y, x, w, span, degree = degree, parametric = parametric, : neighborhood radius 0.005
```

```
## Warning in simpleLoess(y, x, w, span, degree = degree, parametric = parametric, : reciprocal condition number 1
```

```
## Warning in simpleLoess(y, x, w, span, degree = degree, parametric = parametric, : There are other near singularities as well. 1.01
```

```
## Warning in simpleLoess(y, x, w, span, degree = degree, parametric = parametric, : zero-width neighborhood. make span bigger
```

```
## Warning: Computation failed in `stat_smooth()`:
## NA/NaN/Inf in foreign function call (arg 5)
```

```
## Warning in simpleLoess(y, x, w, span, degree = degree, parametric = parametric, : pseudoinverse used at -0.005
```

```
## Warning in simpleLoess(y, x, w, span, degree = degree, parametric = parametric, : neighborhood radius 1.005
```

```
## Warning in simpleLoess(y, x, w, span, degree = degree, parametric = parametric, : reciprocal condition number 0
```

```
## Warning in simpleLoess(y, x, w, span, degree = degree, parametric = parametric, : There are other near singularities as well. 1.01
```

```
## Warning in predLoess(object$y, object$x, newx = if (is.null(newdata)) object$x else if (is.data.frame(newdata)) as.matrix(model.frame(delete.response(terms(object)), : pseudoinverse used at -0.005
```

```
## Warning in predLoess(object$y, object$x, newx = if (is.null(newdata)) object$x else if (is.data.frame(newdata)) as.matrix(model.frame(delete.response(terms(object)), : neighborhood radius 1.005
```

```
## Warning in predLoess(object$y, object$x, newx = if (is.null(newdata)) object$x else if (is.data.frame(newdata)) as.matrix(model.frame(delete.response(terms(object)), : reciprocal condition number 0
```

```
## Warning in predLoess(object$y, object$x, newx = if (is.null(newdata)) object$x else if (is.data.frame(newdata)) as.matrix(model.frame(delete.response(terms(object)), : There are other near
## singularities as well. 1.01
```

```
## Warning in simpleLoess(y, x, w, span, degree = degree, parametric = parametric, : at -0.005
```

```
## Warning in simpleLoess(y, x, w, span, degree = degree, parametric = parametric, : radius 2.5e-05
```

```
## Warning in simpleLoess(y, x, w, span, degree = degree, parametric = parametric, : all data on boundary of neighborhood. make span bigger
```

```
## Warning in simpleLoess(y, x, w, span, degree = degree, parametric = parametric, : pseudoinverse used at -0.005
```

```
## Warning in simpleLoess(y, x, w, span, degree = degree, parametric = parametric, : neighborhood radius 0.005
```

```
## Warning in simpleLoess(y, x, w, span, degree = degree, parametric = parametric, : reciprocal condition number 1
```

```
## Warning in simpleLoess(y, x, w, span, degree = degree, parametric = parametric, : There are other near singularities as well. 1.01
```

```
## Warning in simpleLoess(y, x, w, span, degree = degree, parametric = parametric, : zero-width neighborhood. make span bigger
```

```
## Warning: Computation failed in `stat_smooth()`:
## NA/NaN/Inf in foreign function call (arg 5)
```

```
## Warning in simpleLoess(y, x, w, span, degree = degree, parametric = parametric, : at -0.005
```

```
## Warning in simpleLoess(y, x, w, span, degree = degree, parametric = parametric, : radius 2.5e-05
```

```
## Warning in simpleLoess(y, x, w, span, degree = degree, parametric = parametric, : all data on boundary of neighborhood. make span bigger
```

```
## Warning in simpleLoess(y, x, w, span, degree = degree, parametric = parametric, : pseudoinverse used at -0.005
```

```
## Warning in simpleLoess(y, x, w, span, degree = degree, parametric = parametric, : neighborhood radius 0.005
```

```
## Warning in simpleLoess(y, x, w, span, degree = degree, parametric = parametric, : reciprocal condition number 1
```

```
## Warning in simpleLoess(y, x, w, span, degree = degree, parametric = parametric, : There are other near singularities as well. 1.01
```

```
## Warning in simpleLoess(y, x, w, span, degree = degree, parametric = parametric, : zero-width neighborhood. make span bigger
```

```
## Warning: Computation failed in `stat_smooth()`:
## NA/NaN/Inf in foreign function call (arg 5)
```

```
## Warning in simpleLoess(y, x, w, span, degree = degree, parametric = parametric, : at -0.005
```

```
## Warning in simpleLoess(y, x, w, span, degree = degree, parametric = parametric, : radius 2.5e-05
```

```
## Warning in simpleLoess(y, x, w, span, degree = degree, parametric = parametric, : all data on boundary of neighborhood. make span bigger
```

```
## Warning in simpleLoess(y, x, w, span, degree = degree, parametric = parametric, : pseudoinverse used at -0.005
```

```
## Warning in simpleLoess(y, x, w, span, degree = degree, parametric = parametric, : neighborhood radius 0.005
```

```
## Warning in simpleLoess(y, x, w, span, degree = degree, parametric = parametric, : reciprocal condition number 1
```

```
## Warning in simpleLoess(y, x, w, span, degree = degree, parametric = parametric, : There are other near singularities as well. 1.01
```

```
## Warning in simpleLoess(y, x, w, span, degree = degree, parametric = parametric, : zero-width neighborhood. make span bigger
```

```
## Warning: Computation failed in `stat_smooth()`:
## NA/NaN/Inf in foreign function call (arg 5)
```

```
## Warning in simpleLoess(y, x, w, span, degree = degree, parametric = parametric, : at -0.005
```

```
## Warning in simpleLoess(y, x, w, span, degree = degree, parametric = parametric, : radius 2.5e-05
```

```
## Warning in simpleLoess(y, x, w, span, degree = degree, parametric = parametric, : all data on boundary of neighborhood. make span bigger
```

```
## Warning in simpleLoess(y, x, w, span, degree = degree, parametric = parametric, : pseudoinverse used at -0.005
```

```
## Warning in simpleLoess(y, x, w, span, degree = degree, parametric = parametric, : neighborhood radius 0.005
```

```
## Warning in simpleLoess(y, x, w, span, degree = degree, parametric = parametric, : reciprocal condition number 1
```

```
## Warning in simpleLoess(y, x, w, span, degree = degree, parametric = parametric, : There are other near singularities as well. 1.01
```

```
## Warning in simpleLoess(y, x, w, span, degree = degree, parametric = parametric, : zero-width neighborhood. make span bigger
```

```
## Warning: Computation failed in `stat_smooth()`:
## NA/NaN/Inf in foreign function call (arg 5)
```

```
## Warning in simpleLoess(y, x, w, span, degree = degree, parametric = parametric, : at -0.005
```

```
## Warning in simpleLoess(y, x, w, span, degree = degree, parametric = parametric, : radius 2.5e-05
```

```
## Warning in simpleLoess(y, x, w, span, degree = degree, parametric = parametric, : all data on boundary of neighborhood. make span bigger
```

```
## Warning in simpleLoess(y, x, w, span, degree = degree, parametric = parametric, : pseudoinverse used at -0.005
```

```
## Warning in simpleLoess(y, x, w, span, degree = degree, parametric = parametric, : neighborhood radius 0.005
```

```
## Warning in simpleLoess(y, x, w, span, degree = degree, parametric = parametric, : reciprocal condition number 1
```

```
## Warning in simpleLoess(y, x, w, span, degree = degree, parametric = parametric, : There are other near singularities as well. 1.01
```

```
## Warning in simpleLoess(y, x, w, span, degree = degree, parametric = parametric, : zero-width neighborhood. make span bigger
```

```
## Warning: Computation failed in `stat_smooth()`:
## NA/NaN/Inf in foreign function call (arg 5)
```

```
## Warning in simpleLoess(y, x, w, span, degree = degree, parametric = parametric, : at -0.005
```

```
## Warning in simpleLoess(y, x, w, span, degree = degree, parametric = parametric, : radius 2.5e-05
```

```
## Warning in simpleLoess(y, x, w, span, degree = degree, parametric = parametric, : all data on boundary of neighborhood. make span bigger
```

```
## Warning in simpleLoess(y, x, w, span, degree = degree, parametric = parametric, : pseudoinverse used at -0.005
```

```
## Warning in simpleLoess(y, x, w, span, degree = degree, parametric = parametric, : neighborhood radius 0.005
```

```
## Warning in simpleLoess(y, x, w, span, degree = degree, parametric = parametric, : reciprocal condition number 1
```

```
## Warning in simpleLoess(y, x, w, span, degree = degree, parametric = parametric, : There are other near singularities as well. 1.01
```

```
## Warning in simpleLoess(y, x, w, span, degree = degree, parametric = parametric, : zero-width neighborhood. make span bigger
```

```
## Warning: Computation failed in `stat_smooth()`:
## NA/NaN/Inf in foreign function call (arg 5)
```

```
## Warning in simpleLoess(y, x, w, span, degree = degree, parametric = parametric, : at -0.005
```

```
## Warning in simpleLoess(y, x, w, span, degree = degree, parametric = parametric, : radius 2.5e-05
```

```
## Warning in simpleLoess(y, x, w, span, degree = degree, parametric = parametric, : all data on boundary of neighborhood. make span bigger
```

```
## Warning in simpleLoess(y, x, w, span, degree = degree, parametric = parametric, : pseudoinverse used at -0.005
```

```
## Warning in simpleLoess(y, x, w, span, degree = degree, parametric = parametric, : neighborhood radius 0.005
```

```
## Warning in simpleLoess(y, x, w, span, degree = degree, parametric = parametric, : reciprocal condition number 1
```

```
## Warning in simpleLoess(y, x, w, span, degree = degree, parametric = parametric, : There are other near singularities as well. 1.01
```

```
## Warning in simpleLoess(y, x, w, span, degree = degree, parametric = parametric, : zero-width neighborhood. make span bigger
```

```
## Warning: Computation failed in `stat_smooth()`:
## NA/NaN/Inf in foreign function call (arg 5)
```

```
## Warning in simpleLoess(y, x, w, span, degree = degree, parametric = parametric, : at -0.005
```

```
## Warning in simpleLoess(y, x, w, span, degree = degree, parametric = parametric, : radius 2.5e-05
```

```
## Warning in simpleLoess(y, x, w, span, degree = degree, parametric = parametric, : all data on boundary of neighborhood. make span bigger
```

```
## Warning in simpleLoess(y, x, w, span, degree = degree, parametric = parametric, : pseudoinverse used at -0.005
```

```
## Warning in simpleLoess(y, x, w, span, degree = degree, parametric = parametric, : neighborhood radius 0.005
```

```
## Warning in simpleLoess(y, x, w, span, degree = degree, parametric = parametric, : reciprocal condition number 1
```

```
## Warning in simpleLoess(y, x, w, span, degree = degree, parametric = parametric, : There are other near singularities as well. 1.01
```

```
## Warning in simpleLoess(y, x, w, span, degree = degree, parametric = parametric, : zero-width neighborhood. make span bigger
```

```
## Warning: Computation failed in `stat_smooth()`:
## NA/NaN/Inf in foreign function call (arg 5)
```

```
## Warning in simpleLoess(y, x, w, span, degree = degree, parametric = parametric, : at -0.005
```

```
## Warning in simpleLoess(y, x, w, span, degree = degree, parametric = parametric, : radius 2.5e-05
```

```
## Warning in simpleLoess(y, x, w, span, degree = degree, parametric = parametric, : all data on boundary of neighborhood. make span bigger
```

```
## Warning in simpleLoess(y, x, w, span, degree = degree, parametric = parametric, : pseudoinverse used at -0.005
```

```
## Warning in simpleLoess(y, x, w, span, degree = degree, parametric = parametric, : neighborhood radius 0.005
```

```
## Warning in simpleLoess(y, x, w, span, degree = degree, parametric = parametric, : reciprocal condition number 1
```

```
## Warning in simpleLoess(y, x, w, span, degree = degree, parametric = parametric, : There are other near singularities as well. 1.01
```

```
## Warning in simpleLoess(y, x, w, span, degree = degree, parametric = parametric, : zero-width neighborhood. make span bigger
```

```
## Warning: Computation failed in `stat_smooth()`:
## NA/NaN/Inf in foreign function call (arg 5)
```

```
## Warning in simpleLoess(y, x, w, span, degree = degree, parametric = parametric, : at -0.005
```

```
## Warning in simpleLoess(y, x, w, span, degree = degree, parametric = parametric, : radius 2.5e-05
```

```
## Warning in simpleLoess(y, x, w, span, degree = degree, parametric = parametric, : all data on boundary of neighborhood. make span bigger
```

```
## Warning in simpleLoess(y, x, w, span, degree = degree, parametric = parametric, : pseudoinverse used at -0.005
```

```
## Warning in simpleLoess(y, x, w, span, degree = degree, parametric = parametric, : neighborhood radius 0.005
```

```
## Warning in simpleLoess(y, x, w, span, degree = degree, parametric = parametric, : reciprocal condition number 1
```

```
## Warning in simpleLoess(y, x, w, span, degree = degree, parametric = parametric, : There are other near singularities as well. 1.01
```

```
## Warning in simpleLoess(y, x, w, span, degree = degree, parametric = parametric, : zero-width neighborhood. make span bigger
```

```
## Warning: Computation failed in `stat_smooth()`:
## NA/NaN/Inf in foreign function call (arg 5)
```

```
## Warning in simpleLoess(y, x, w, span, degree = degree, parametric = parametric, : at -0.005
```

```
## Warning in simpleLoess(y, x, w, span, degree = degree, parametric = parametric, : radius 2.5e-05
```

```
## Warning in simpleLoess(y, x, w, span, degree = degree, parametric = parametric, : all data on boundary of neighborhood. make span bigger
```

```
## Warning in simpleLoess(y, x, w, span, degree = degree, parametric = parametric, : pseudoinverse used at -0.005
```

```
## Warning in simpleLoess(y, x, w, span, degree = degree, parametric = parametric, : neighborhood radius 0.005
```

```
## Warning in simpleLoess(y, x, w, span, degree = degree, parametric = parametric, : reciprocal condition number 1
```

```
## Warning in simpleLoess(y, x, w, span, degree = degree, parametric = parametric, : There are other near singularities as well. 1.01
```

```
## Warning in simpleLoess(y, x, w, span, degree = degree, parametric = parametric, : zero-width neighborhood. make span bigger
```

```
## Warning: Computation failed in `stat_smooth()`:
## NA/NaN/Inf in foreign function call (arg 5)
```

```
## Warning in simpleLoess(y, x, w, span, degree = degree, parametric = parametric, : at -0.005
```

```
## Warning in simpleLoess(y, x, w, span, degree = degree, parametric = parametric, : radius 2.5e-05
```

```
## Warning in simpleLoess(y, x, w, span, degree = degree, parametric = parametric, : all data on boundary of neighborhood. make span bigger
```

```
## Warning in simpleLoess(y, x, w, span, degree = degree, parametric = parametric, : pseudoinverse used at -0.005
```

```
## Warning in simpleLoess(y, x, w, span, degree = degree, parametric = parametric, : neighborhood radius 0.005
```

```
## Warning in simpleLoess(y, x, w, span, degree = degree, parametric = parametric, : reciprocal condition number 1
```

```
## Warning in simpleLoess(y, x, w, span, degree = degree, parametric = parametric, : There are other near singularities as well. 1.01
```

```
## Warning in simpleLoess(y, x, w, span, degree = degree, parametric = parametric, : zero-width neighborhood. make span bigger
```

```
## Warning: Computation failed in `stat_smooth()`:
## NA/NaN/Inf in foreign function call (arg 5)
```

```
## Warning in simpleLoess(y, x, w, span, degree = degree, parametric = parametric, : at -0.005
```

```
## Warning in simpleLoess(y, x, w, span, degree = degree, parametric = parametric, : radius 2.5e-05
```

```
## Warning in simpleLoess(y, x, w, span, degree = degree, parametric = parametric, : all data on boundary of neighborhood. make span bigger
```

```
## Warning in simpleLoess(y, x, w, span, degree = degree, parametric = parametric, : pseudoinverse used at -0.005
```

```
## Warning in simpleLoess(y, x, w, span, degree = degree, parametric = parametric, : neighborhood radius 0.005
```

```
## Warning in simpleLoess(y, x, w, span, degree = degree, parametric = parametric, : reciprocal condition number 1
```

```
## Warning in simpleLoess(y, x, w, span, degree = degree, parametric = parametric, : There are other near singularities as well. 1.01
```

```
## Warning in simpleLoess(y, x, w, span, degree = degree, parametric = parametric, : zero-width neighborhood. make span bigger
```

```
## Warning: Computation failed in `stat_smooth()`:
## NA/NaN/Inf in foreign function call (arg 5)
```

```
## Warning in simpleLoess(y, x, w, span, degree = degree, parametric = parametric, : at -0.005
```

```
## Warning in simpleLoess(y, x, w, span, degree = degree, parametric = parametric, : radius 2.5e-05
```

```
## Warning in simpleLoess(y, x, w, span, degree = degree, parametric = parametric, : all data on boundary of neighborhood. make span bigger
```

```
## Warning in simpleLoess(y, x, w, span, degree = degree, parametric = parametric, : pseudoinverse used at -0.005
```

```
## Warning in simpleLoess(y, x, w, span, degree = degree, parametric = parametric, : neighborhood radius 0.005
```

```
## Warning in simpleLoess(y, x, w, span, degree = degree, parametric = parametric, : reciprocal condition number 1
```

```
## Warning in simpleLoess(y, x, w, span, degree = degree, parametric = parametric, : There are other near singularities as well. 1.01
```

```
## Warning in simpleLoess(y, x, w, span, degree = degree, parametric = parametric, : zero-width neighborhood. make span bigger
```

```
## Warning: Computation failed in `stat_smooth()`:
## NA/NaN/Inf in foreign function call (arg 5)
```

```
## Warning in simpleLoess(y, x, w, span, degree = degree, parametric = parametric, : at -0.005
```

```
## Warning in simpleLoess(y, x, w, span, degree = degree, parametric = parametric, : radius 2.5e-05
```

```
## Warning in simpleLoess(y, x, w, span, degree = degree, parametric = parametric, : all data on boundary of neighborhood. make span bigger
```

```
## Warning in simpleLoess(y, x, w, span, degree = degree, parametric = parametric, : pseudoinverse used at -0.005
```

```
## Warning in simpleLoess(y, x, w, span, degree = degree, parametric = parametric, : neighborhood radius 0.005
```

```
## Warning in simpleLoess(y, x, w, span, degree = degree, parametric = parametric, : reciprocal condition number 1
```

```
## Warning in simpleLoess(y, x, w, span, degree = degree, parametric = parametric, : There are other near singularities as well. 1.01
```

```
## Warning in simpleLoess(y, x, w, span, degree = degree, parametric = parametric, : zero-width neighborhood. make span bigger
```

```
## Warning: Computation failed in `stat_smooth()`:
## NA/NaN/Inf in foreign function call (arg 5)
```

```
## Warning in simpleLoess(y, x, w, span, degree = degree, parametric = parametric, : at -0.005
```

```
## Warning in simpleLoess(y, x, w, span, degree = degree, parametric = parametric, : radius 2.5e-05
```

```
## Warning in simpleLoess(y, x, w, span, degree = degree, parametric = parametric, : all data on boundary of neighborhood. make span bigger
```

```
## Warning in simpleLoess(y, x, w, span, degree = degree, parametric = parametric, : pseudoinverse used at -0.005
```

```
## Warning in simpleLoess(y, x, w, span, degree = degree, parametric = parametric, : neighborhood radius 0.005
```

```
## Warning in simpleLoess(y, x, w, span, degree = degree, parametric = parametric, : reciprocal condition number 1
```

```
## Warning in simpleLoess(y, x, w, span, degree = degree, parametric = parametric, : There are other near singularities as well. 1.01
```

```
## Warning in simpleLoess(y, x, w, span, degree = degree, parametric = parametric, : zero-width neighborhood. make span bigger
```

```
## Warning: Computation failed in `stat_smooth()`:
## NA/NaN/Inf in foreign function call (arg 5)
```

```
## Warning in simpleLoess(y, x, w, span, degree = degree, parametric = parametric, : at -0.005
```

```
## Warning in simpleLoess(y, x, w, span, degree = degree, parametric = parametric, : radius 2.5e-05
```

```
## Warning in simpleLoess(y, x, w, span, degree = degree, parametric = parametric, : all data on boundary of neighborhood. make span bigger
```

```
## Warning in simpleLoess(y, x, w, span, degree = degree, parametric = parametric, : pseudoinverse used at -0.005
```

```
## Warning in simpleLoess(y, x, w, span, degree = degree, parametric = parametric, : neighborhood radius 0.005
```

```
## Warning in simpleLoess(y, x, w, span, degree = degree, parametric = parametric, : reciprocal condition number 1
```

```
## Warning in simpleLoess(y, x, w, span, degree = degree, parametric = parametric, : There are other near singularities as well. 1.01
```

```
## Warning in simpleLoess(y, x, w, span, degree = degree, parametric = parametric, : zero-width neighborhood. make span bigger
```

```
## Warning: Computation failed in `stat_smooth()`:
## NA/NaN/Inf in foreign function call (arg 5)
```

```
## Warning in simpleLoess(y, x, w, span, degree = degree, parametric = parametric, : at -0.005
```

```
## Warning in simpleLoess(y, x, w, span, degree = degree, parametric = parametric, : radius 2.5e-05
```

```
## Warning in simpleLoess(y, x, w, span, degree = degree, parametric = parametric, : all data on boundary of neighborhood. make span bigger
```

```
## Warning in simpleLoess(y, x, w, span, degree = degree, parametric = parametric, : pseudoinverse used at -0.005
```

```
## Warning in simpleLoess(y, x, w, span, degree = degree, parametric = parametric, : neighborhood radius 0.005
```

```
## Warning in simpleLoess(y, x, w, span, degree = degree, parametric = parametric, : reciprocal condition number 1
```

```
## Warning in simpleLoess(y, x, w, span, degree = degree, parametric = parametric, : There are other near singularities as well. 1.01
```

```
## Warning in simpleLoess(y, x, w, span, degree = degree, parametric = parametric, : zero-width neighborhood. make span bigger
```

```
## Warning: Computation failed in `stat_smooth()`:
## NA/NaN/Inf in foreign function call (arg 5)
```

```
## Warning in simpleLoess(y, x, w, span, degree = degree, parametric = parametric, : at -0.005
```

```
## Warning in simpleLoess(y, x, w, span, degree = degree, parametric = parametric, : radius 2.5e-05
```

```
## Warning in simpleLoess(y, x, w, span, degree = degree, parametric = parametric, : all data on boundary of neighborhood. make span bigger
```

```
## Warning in simpleLoess(y, x, w, span, degree = degree, parametric = parametric, : pseudoinverse used at -0.005
```

```
## Warning in simpleLoess(y, x, w, span, degree = degree, parametric = parametric, : neighborhood radius 0.005
```

```
## Warning in simpleLoess(y, x, w, span, degree = degree, parametric = parametric, : reciprocal condition number 1
```

```
## Warning in simpleLoess(y, x, w, span, degree = degree, parametric = parametric, : There are other near singularities as well. 1.01
```

```
## Warning in simpleLoess(y, x, w, span, degree = degree, parametric = parametric, : zero-width neighborhood. make span bigger
```

```
## Warning: Computation failed in `stat_smooth()`:
## NA/NaN/Inf in foreign function call (arg 5)
```

```
## Warning in simpleLoess(y, x, w, span, degree = degree, parametric = parametric, : pseudoinverse used at -0.005
```

```
## Warning in simpleLoess(y, x, w, span, degree = degree, parametric = parametric, : neighborhood radius 1.005
```

```
## Warning in simpleLoess(y, x, w, span, degree = degree, parametric = parametric, : reciprocal condition number 0
```

```
## Warning in simpleLoess(y, x, w, span, degree = degree, parametric = parametric, : There are other near singularities as well. 1.01
```

```
## Warning in predLoess(object$y, object$x, newx = if (is.null(newdata)) object$x else if (is.data.frame(newdata)) as.matrix(model.frame(delete.response(terms(object)), : pseudoinverse used at -0.005
```

```
## Warning in predLoess(object$y, object$x, newx = if (is.null(newdata)) object$x else if (is.data.frame(newdata)) as.matrix(model.frame(delete.response(terms(object)), : neighborhood radius 1.005
```

```
## Warning in predLoess(object$y, object$x, newx = if (is.null(newdata)) object$x else if (is.data.frame(newdata)) as.matrix(model.frame(delete.response(terms(object)), : reciprocal condition number 0
```

```
## Warning in predLoess(object$y, object$x, newx = if (is.null(newdata)) object$x else if (is.data.frame(newdata)) as.matrix(model.frame(delete.response(terms(object)), : There are other near
## singularities as well. 1.01
```

```
## Warning in simpleLoess(y, x, w, span, degree = degree, parametric = parametric, : at -0.005
```

```
## Warning in simpleLoess(y, x, w, span, degree = degree, parametric = parametric, : radius 2.5e-05
```

```
## Warning in simpleLoess(y, x, w, span, degree = degree, parametric = parametric, : all data on boundary of neighborhood. make span bigger
```

```
## Warning in simpleLoess(y, x, w, span, degree = degree, parametric = parametric, : pseudoinverse used at -0.005
```

```
## Warning in simpleLoess(y, x, w, span, degree = degree, parametric = parametric, : neighborhood radius 0.005
```

```
## Warning in simpleLoess(y, x, w, span, degree = degree, parametric = parametric, : reciprocal condition number 1
```

```
## Warning in simpleLoess(y, x, w, span, degree = degree, parametric = parametric, : There are other near singularities as well. 1.01
```

```
## Warning in simpleLoess(y, x, w, span, degree = degree, parametric = parametric, : zero-width neighborhood. make span bigger
```

```
## Warning: Computation failed in `stat_smooth()`:
## NA/NaN/Inf in foreign function call (arg 5)
```

```
## Warning in simpleLoess(y, x, w, span, degree = degree, parametric = parametric, : at -0.005
```

```
## Warning in simpleLoess(y, x, w, span, degree = degree, parametric = parametric, : radius 2.5e-05
```

```
## Warning in simpleLoess(y, x, w, span, degree = degree, parametric = parametric, : all data on boundary of neighborhood. make span bigger
```

```
## Warning in simpleLoess(y, x, w, span, degree = degree, parametric = parametric, : pseudoinverse used at -0.005
```

```
## Warning in simpleLoess(y, x, w, span, degree = degree, parametric = parametric, : neighborhood radius 0.005
```

```
## Warning in simpleLoess(y, x, w, span, degree = degree, parametric = parametric, : reciprocal condition number 1
```

```
## Warning in simpleLoess(y, x, w, span, degree = degree, parametric = parametric, : There are other near singularities as well. 1.01
```

```
## Warning in simpleLoess(y, x, w, span, degree = degree, parametric = parametric, : zero-width neighborhood. make span bigger
```

```
## Warning: Computation failed in `stat_smooth()`:
## NA/NaN/Inf in foreign function call (arg 5)
```

```
## Warning in simpleLoess(y, x, w, span, degree = degree, parametric = parametric, : at -0.005
```

```
## Warning in simpleLoess(y, x, w, span, degree = degree, parametric = parametric, : radius 2.5e-05
```

```
## Warning in simpleLoess(y, x, w, span, degree = degree, parametric = parametric, : all data on boundary of neighborhood. make span bigger
```

```
## Warning in simpleLoess(y, x, w, span, degree = degree, parametric = parametric, : pseudoinverse used at -0.005
```

```
## Warning in simpleLoess(y, x, w, span, degree = degree, parametric = parametric, : neighborhood radius 0.005
```

```
## Warning in simpleLoess(y, x, w, span, degree = degree, parametric = parametric, : reciprocal condition number 1
```

```
## Warning in simpleLoess(y, x, w, span, degree = degree, parametric = parametric, : There are other near singularities as well. 1.01
```

```
## Warning in simpleLoess(y, x, w, span, degree = degree, parametric = parametric, : zero-width neighborhood. make span bigger
```

```
## Warning: Computation failed in `stat_smooth()`:
## NA/NaN/Inf in foreign function call (arg 5)
```

```
## Warning in simpleLoess(y, x, w, span, degree = degree, parametric = parametric, : at -0.005
```

```
## Warning in simpleLoess(y, x, w, span, degree = degree, parametric = parametric, : radius 2.5e-05
```

```
## Warning in simpleLoess(y, x, w, span, degree = degree, parametric = parametric, : all data on boundary of neighborhood. make span bigger
```

```
## Warning in simpleLoess(y, x, w, span, degree = degree, parametric = parametric, : pseudoinverse used at -0.005
```

```
## Warning in simpleLoess(y, x, w, span, degree = degree, parametric = parametric, : neighborhood radius 0.005
```

```
## Warning in simpleLoess(y, x, w, span, degree = degree, parametric = parametric, : reciprocal condition number 1
```

```
## Warning in simpleLoess(y, x, w, span, degree = degree, parametric = parametric, : There are other near singularities as well. 1.01
```

```
## Warning in simpleLoess(y, x, w, span, degree = degree, parametric = parametric, : zero-width neighborhood. make span bigger
```

```
## Warning: Computation failed in `stat_smooth()`:
## NA/NaN/Inf in foreign function call (arg 5)
```

```
## Warning in simpleLoess(y, x, w, span, degree = degree, parametric = parametric, : at -0.005
```

```
## Warning in simpleLoess(y, x, w, span, degree = degree, parametric = parametric, : radius 2.5e-05
```

```
## Warning in simpleLoess(y, x, w, span, degree = degree, parametric = parametric, : all data on boundary of neighborhood. make span bigger
```

```
## Warning in simpleLoess(y, x, w, span, degree = degree, parametric = parametric, : pseudoinverse used at -0.005
```

```
## Warning in simpleLoess(y, x, w, span, degree = degree, parametric = parametric, : neighborhood radius 0.005
```

```
## Warning in simpleLoess(y, x, w, span, degree = degree, parametric = parametric, : reciprocal condition number 1
```

```
## Warning in simpleLoess(y, x, w, span, degree = degree, parametric = parametric, : There are other near singularities as well. 1.01
```

```
## Warning in simpleLoess(y, x, w, span, degree = degree, parametric = parametric, : zero-width neighborhood. make span bigger
```

```
## Warning: Computation failed in `stat_smooth()`:
## NA/NaN/Inf in foreign function call (arg 5)
```

```
## Warning in simpleLoess(y, x, w, span, degree = degree, parametric = parametric, : at -0.005
```

```
## Warning in simpleLoess(y, x, w, span, degree = degree, parametric = parametric, : radius 2.5e-05
```

```
## Warning in simpleLoess(y, x, w, span, degree = degree, parametric = parametric, : all data on boundary of neighborhood. make span bigger
```

```
## Warning in simpleLoess(y, x, w, span, degree = degree, parametric = parametric, : pseudoinverse used at -0.005
```

```
## Warning in simpleLoess(y, x, w, span, degree = degree, parametric = parametric, : neighborhood radius 0.005
```

```
## Warning in simpleLoess(y, x, w, span, degree = degree, parametric = parametric, : reciprocal condition number 1
```

```
## Warning in simpleLoess(y, x, w, span, degree = degree, parametric = parametric, : There are other near singularities as well. 1.01
```

```
## Warning in simpleLoess(y, x, w, span, degree = degree, parametric = parametric, : zero-width neighborhood. make span bigger
```

```
## Warning: Computation failed in `stat_smooth()`:
## NA/NaN/Inf in foreign function call (arg 5)
```

```
## Warning in simpleLoess(y, x, w, span, degree = degree, parametric = parametric, : at -0.005
```

```
## Warning in simpleLoess(y, x, w, span, degree = degree, parametric = parametric, : radius 2.5e-05
```

```
## Warning in simpleLoess(y, x, w, span, degree = degree, parametric = parametric, : all data on boundary of neighborhood. make span bigger
```

```
## Warning in simpleLoess(y, x, w, span, degree = degree, parametric = parametric, : pseudoinverse used at -0.005
```

```
## Warning in simpleLoess(y, x, w, span, degree = degree, parametric = parametric, : neighborhood radius 0.005
```

```
## Warning in simpleLoess(y, x, w, span, degree = degree, parametric = parametric, : reciprocal condition number 1
```

```
## Warning in simpleLoess(y, x, w, span, degree = degree, parametric = parametric, : There are other near singularities as well. 1.01
```

```
## Warning in simpleLoess(y, x, w, span, degree = degree, parametric = parametric, : zero-width neighborhood. make span bigger
```

```
## Warning: Computation failed in `stat_smooth()`:
## NA/NaN/Inf in foreign function call (arg 5)
```

```
## Warning in simpleLoess(y, x, w, span, degree = degree, parametric = parametric, : at -0.005
```

```
## Warning in simpleLoess(y, x, w, span, degree = degree, parametric = parametric, : radius 2.5e-05
```

```
## Warning in simpleLoess(y, x, w, span, degree = degree, parametric = parametric, : all data on boundary of neighborhood. make span bigger
```

```
## Warning in simpleLoess(y, x, w, span, degree = degree, parametric = parametric, : pseudoinverse used at -0.005
```

```
## Warning in simpleLoess(y, x, w, span, degree = degree, parametric = parametric, : neighborhood radius 0.005
```

```
## Warning in simpleLoess(y, x, w, span, degree = degree, parametric = parametric, : reciprocal condition number 1
```

```
## Warning in simpleLoess(y, x, w, span, degree = degree, parametric = parametric, : There are other near singularities as well. 1.01
```

```
## Warning in simpleLoess(y, x, w, span, degree = degree, parametric = parametric, : zero-width neighborhood. make span bigger
```

```
## Warning: Computation failed in `stat_smooth()`:
## NA/NaN/Inf in foreign function call (arg 5)
```

```
## Warning in simpleLoess(y, x, w, span, degree = degree, parametric = parametric, : at -0.005
```

```
## Warning in simpleLoess(y, x, w, span, degree = degree, parametric = parametric, : radius 2.5e-05
```

```
## Warning in simpleLoess(y, x, w, span, degree = degree, parametric = parametric, : all data on boundary of neighborhood. make span bigger
```

```
## Warning in simpleLoess(y, x, w, span, degree = degree, parametric = parametric, : pseudoinverse used at -0.005
```

```
## Warning in simpleLoess(y, x, w, span, degree = degree, parametric = parametric, : neighborhood radius 0.005
```

```
## Warning in simpleLoess(y, x, w, span, degree = degree, parametric = parametric, : reciprocal condition number 1
```

```
## Warning in simpleLoess(y, x, w, span, degree = degree, parametric = parametric, : There are other near singularities as well. 1.01
```

```
## Warning in simpleLoess(y, x, w, span, degree = degree, parametric = parametric, : zero-width neighborhood. make span bigger
```

```
## Warning: Computation failed in `stat_smooth()`:
## NA/NaN/Inf in foreign function call (arg 5)
```

```
## Warning in simpleLoess(y, x, w, span, degree = degree, parametric = parametric, : at -0.005
```

```
## Warning in simpleLoess(y, x, w, span, degree = degree, parametric = parametric, : radius 2.5e-05
```

```
## Warning in simpleLoess(y, x, w, span, degree = degree, parametric = parametric, : all data on boundary of neighborhood. make span bigger
```

```
## Warning in simpleLoess(y, x, w, span, degree = degree, parametric = parametric, : pseudoinverse used at -0.005
```

```
## Warning in simpleLoess(y, x, w, span, degree = degree, parametric = parametric, : neighborhood radius 0.005
```

```
## Warning in simpleLoess(y, x, w, span, degree = degree, parametric = parametric, : reciprocal condition number 1
```

```
## Warning in simpleLoess(y, x, w, span, degree = degree, parametric = parametric, : There are other near singularities as well. 1.01
```

```
## Warning in simpleLoess(y, x, w, span, degree = degree, parametric = parametric, : zero-width neighborhood. make span bigger
```

```
## Warning: Computation failed in `stat_smooth()`:
## NA/NaN/Inf in foreign function call (arg 5)
```

```
## Warning in simpleLoess(y, x, w, span, degree = degree, parametric = parametric, : at -0.005
```

```
## Warning in simpleLoess(y, x, w, span, degree = degree, parametric = parametric, : radius 2.5e-05
```

```
## Warning in simpleLoess(y, x, w, span, degree = degree, parametric = parametric, : all data on boundary of neighborhood. make span bigger
```

```
## Warning in simpleLoess(y, x, w, span, degree = degree, parametric = parametric, : pseudoinverse used at -0.005
```

```
## Warning in simpleLoess(y, x, w, span, degree = degree, parametric = parametric, : neighborhood radius 0.005
```

```
## Warning in simpleLoess(y, x, w, span, degree = degree, parametric = parametric, : reciprocal condition number 1
```

```
## Warning in simpleLoess(y, x, w, span, degree = degree, parametric = parametric, : There are other near singularities as well. 1.01
```

```
## Warning in simpleLoess(y, x, w, span, degree = degree, parametric = parametric, : zero-width neighborhood. make span bigger
```

```
## Warning: Computation failed in `stat_smooth()`:
## NA/NaN/Inf in foreign function call (arg 5)
```

```
## Warning in simpleLoess(y, x, w, span, degree = degree, parametric = parametric, : at -0.005
```

```
## Warning in simpleLoess(y, x, w, span, degree = degree, parametric = parametric, : radius 2.5e-05
```

```
## Warning in simpleLoess(y, x, w, span, degree = degree, parametric = parametric, : all data on boundary of neighborhood. make span bigger
```

```
## Warning in simpleLoess(y, x, w, span, degree = degree, parametric = parametric, : pseudoinverse used at -0.005
```

```
## Warning in simpleLoess(y, x, w, span, degree = degree, parametric = parametric, : neighborhood radius 0.005
```

```
## Warning in simpleLoess(y, x, w, span, degree = degree, parametric = parametric, : reciprocal condition number 1
```

```
## Warning in simpleLoess(y, x, w, span, degree = degree, parametric = parametric, : There are other near singularities as well. 1.01
```

```
## Warning in simpleLoess(y, x, w, span, degree = degree, parametric = parametric, : zero-width neighborhood. make span bigger
```

```
## Warning: Computation failed in `stat_smooth()`:
## NA/NaN/Inf in foreign function call (arg 5)
```

```
## Warning in simpleLoess(y, x, w, span, degree = degree, parametric = parametric, : at -0.005
```

```
## Warning in simpleLoess(y, x, w, span, degree = degree, parametric = parametric, : radius 2.5e-05
```

```
## Warning in simpleLoess(y, x, w, span, degree = degree, parametric = parametric, : all data on boundary of neighborhood. make span bigger
```

```
## Warning in simpleLoess(y, x, w, span, degree = degree, parametric = parametric, : pseudoinverse used at -0.005
```

```
## Warning in simpleLoess(y, x, w, span, degree = degree, parametric = parametric, : neighborhood radius 0.005
```

```
## Warning in simpleLoess(y, x, w, span, degree = degree, parametric = parametric, : reciprocal condition number 1
```

```
## Warning in simpleLoess(y, x, w, span, degree = degree, parametric = parametric, : There are other near singularities as well. 1.01
```

```
## Warning in simpleLoess(y, x, w, span, degree = degree, parametric = parametric, : zero-width neighborhood. make span bigger
```

```
## Warning: Computation failed in `stat_smooth()`:
## NA/NaN/Inf in foreign function call (arg 5)
```

```
## Warning in simpleLoess(y, x, w, span, degree = degree, parametric = parametric, : at -0.005
```

```
## Warning in simpleLoess(y, x, w, span, degree = degree, parametric = parametric, : radius 2.5e-05
```

```
## Warning in simpleLoess(y, x, w, span, degree = degree, parametric = parametric, : all data on boundary of neighborhood. make span bigger
```

```
## Warning in simpleLoess(y, x, w, span, degree = degree, parametric = parametric, : pseudoinverse used at -0.005
```

```
## Warning in simpleLoess(y, x, w, span, degree = degree, parametric = parametric, : neighborhood radius 0.005
```

```
## Warning in simpleLoess(y, x, w, span, degree = degree, parametric = parametric, : reciprocal condition number 1
```

```
## Warning in simpleLoess(y, x, w, span, degree = degree, parametric = parametric, : There are other near singularities as well. 1.01
```

```
## Warning in simpleLoess(y, x, w, span, degree = degree, parametric = parametric, : zero-width neighborhood. make span bigger
```

```
## Warning: Computation failed in `stat_smooth()`:
## NA/NaN/Inf in foreign function call (arg 5)
```

```
## Warning in simpleLoess(y, x, w, span, degree = degree, parametric = parametric, : pseudoinverse used at -0.005
```

```
## Warning in simpleLoess(y, x, w, span, degree = degree, parametric = parametric, : neighborhood radius 1.005
```

```
## Warning in simpleLoess(y, x, w, span, degree = degree, parametric = parametric, : reciprocal condition number 0
```

```
## Warning in simpleLoess(y, x, w, span, degree = degree, parametric = parametric, : There are other near singularities as well. 1.01
```

```
## Warning in predLoess(object$y, object$x, newx = if (is.null(newdata)) object$x else if (is.data.frame(newdata)) as.matrix(model.frame(delete.response(terms(object)), : pseudoinverse used at -0.005
```

```
## Warning in predLoess(object$y, object$x, newx = if (is.null(newdata)) object$x else if (is.data.frame(newdata)) as.matrix(model.frame(delete.response(terms(object)), : neighborhood radius 1.005
```

```
## Warning in predLoess(object$y, object$x, newx = if (is.null(newdata)) object$x else if (is.data.frame(newdata)) as.matrix(model.frame(delete.response(terms(object)), : reciprocal condition number 0
```

```
## Warning in predLoess(object$y, object$x, newx = if (is.null(newdata)) object$x else if (is.data.frame(newdata)) as.matrix(model.frame(delete.response(terms(object)), : There are other near
## singularities as well. 1.01
```

```
## Warning in simpleLoess(y, x, w, span, degree = degree, parametric = parametric, : at -0.005
```

```
## Warning in simpleLoess(y, x, w, span, degree = degree, parametric = parametric, : radius 2.5e-05
```

```
## Warning in simpleLoess(y, x, w, span, degree = degree, parametric = parametric, : all data on boundary of neighborhood. make span bigger
```

```
## Warning in simpleLoess(y, x, w, span, degree = degree, parametric = parametric, : pseudoinverse used at -0.005
```

```
## Warning in simpleLoess(y, x, w, span, degree = degree, parametric = parametric, : neighborhood radius 0.005
```

```
## Warning in simpleLoess(y, x, w, span, degree = degree, parametric = parametric, : reciprocal condition number 1
```

```
## Warning in simpleLoess(y, x, w, span, degree = degree, parametric = parametric, : There are other near singularities as well. 1.01
```

```
## Warning in simpleLoess(y, x, w, span, degree = degree, parametric = parametric, : zero-width neighborhood. make span bigger
```

```
## Warning: Computation failed in `stat_smooth()`:
## NA/NaN/Inf in foreign function call (arg 5)
```

```
## Warning in simpleLoess(y, x, w, span, degree = degree, parametric = parametric, : pseudoinverse used at -0.005
```

```
## Warning in simpleLoess(y, x, w, span, degree = degree, parametric = parametric, : neighborhood radius 1.005
```

```
## Warning in simpleLoess(y, x, w, span, degree = degree, parametric = parametric, : reciprocal condition number 0
```

```
## Warning in simpleLoess(y, x, w, span, degree = degree, parametric = parametric, : There are other near singularities as well. 1.01
```

```
## Warning in predLoess(object$y, object$x, newx = if (is.null(newdata)) object$x else if (is.data.frame(newdata)) as.matrix(model.frame(delete.response(terms(object)), : pseudoinverse used at -0.005
```

```
## Warning in predLoess(object$y, object$x, newx = if (is.null(newdata)) object$x else if (is.data.frame(newdata)) as.matrix(model.frame(delete.response(terms(object)), : neighborhood radius 1.005
```

```
## Warning in predLoess(object$y, object$x, newx = if (is.null(newdata)) object$x else if (is.data.frame(newdata)) as.matrix(model.frame(delete.response(terms(object)), : reciprocal condition number 0
```

```
## Warning in predLoess(object$y, object$x, newx = if (is.null(newdata)) object$x else if (is.data.frame(newdata)) as.matrix(model.frame(delete.response(terms(object)), : There are other near
## singularities as well. 1.01
```

```
## Warning in simpleLoess(y, x, w, span, degree = degree, parametric = parametric, : at -0.005
```

```
## Warning in simpleLoess(y, x, w, span, degree = degree, parametric = parametric, : radius 2.5e-05
```

```
## Warning in simpleLoess(y, x, w, span, degree = degree, parametric = parametric, : all data on boundary of neighborhood. make span bigger
```

```
## Warning in simpleLoess(y, x, w, span, degree = degree, parametric = parametric, : pseudoinverse used at -0.005
```

```
## Warning in simpleLoess(y, x, w, span, degree = degree, parametric = parametric, : neighborhood radius 0.005
```

```
## Warning in simpleLoess(y, x, w, span, degree = degree, parametric = parametric, : reciprocal condition number 1
```

```
## Warning in simpleLoess(y, x, w, span, degree = degree, parametric = parametric, : There are other near singularities as well. 1.01
```

```
## Warning in simpleLoess(y, x, w, span, degree = degree, parametric = parametric, : zero-width neighborhood. make span bigger
```

```
## Warning: Computation failed in `stat_smooth()`:
## NA/NaN/Inf in foreign function call (arg 5)
```

```
## Warning in simpleLoess(y, x, w, span, degree = degree, parametric = parametric, : at -0.005
```

```
## Warning in simpleLoess(y, x, w, span, degree = degree, parametric = parametric, : radius 2.5e-05
```

```
## Warning in simpleLoess(y, x, w, span, degree = degree, parametric = parametric, : all data on boundary of neighborhood. make span bigger
```

```
## Warning in simpleLoess(y, x, w, span, degree = degree, parametric = parametric, : pseudoinverse used at -0.005
```

```
## Warning in simpleLoess(y, x, w, span, degree = degree, parametric = parametric, : neighborhood radius 0.005
```

```
## Warning in simpleLoess(y, x, w, span, degree = degree, parametric = parametric, : reciprocal condition number 1
```

```
## Warning in simpleLoess(y, x, w, span, degree = degree, parametric = parametric, : There are other near singularities as well. 1.01
```

```
## Warning in simpleLoess(y, x, w, span, degree = degree, parametric = parametric, : zero-width neighborhood. make span bigger
```

```
## Warning: Computation failed in `stat_smooth()`:
## NA/NaN/Inf in foreign function call (arg 5)
```

```
## Warning in simpleLoess(y, x, w, span, degree = degree, parametric = parametric, : at -0.005
```

```
## Warning in simpleLoess(y, x, w, span, degree = degree, parametric = parametric, : radius 2.5e-05
```

```
## Warning in simpleLoess(y, x, w, span, degree = degree, parametric = parametric, : all data on boundary of neighborhood. make span bigger
```

```
## Warning in simpleLoess(y, x, w, span, degree = degree, parametric = parametric, : pseudoinverse used at -0.005
```

```
## Warning in simpleLoess(y, x, w, span, degree = degree, parametric = parametric, : neighborhood radius 0.005
```

```
## Warning in simpleLoess(y, x, w, span, degree = degree, parametric = parametric, : reciprocal condition number 1
```

```
## Warning in simpleLoess(y, x, w, span, degree = degree, parametric = parametric, : There are other near singularities as well. 1.01
```

```
## Warning in simpleLoess(y, x, w, span, degree = degree, parametric = parametric, : zero-width neighborhood. make span bigger
```

```
## Warning: Computation failed in `stat_smooth()`:
## NA/NaN/Inf in foreign function call (arg 5)
```

```
## Warning in simpleLoess(y, x, w, span, degree = degree, parametric = parametric, : at -0.005
```

```
## Warning in simpleLoess(y, x, w, span, degree = degree, parametric = parametric, : radius 2.5e-05
```

```
## Warning in simpleLoess(y, x, w, span, degree = degree, parametric = parametric, : all data on boundary of neighborhood. make span bigger
```

```
## Warning in simpleLoess(y, x, w, span, degree = degree, parametric = parametric, : pseudoinverse used at -0.005
```

```
## Warning in simpleLoess(y, x, w, span, degree = degree, parametric = parametric, : neighborhood radius 0.005
```

```
## Warning in simpleLoess(y, x, w, span, degree = degree, parametric = parametric, : reciprocal condition number 1
```

```
## Warning in simpleLoess(y, x, w, span, degree = degree, parametric = parametric, : There are other near singularities as well. 1.01
```

```
## Warning in simpleLoess(y, x, w, span, degree = degree, parametric = parametric, : zero-width neighborhood. make span bigger
```

```
## Warning: Computation failed in `stat_smooth()`:
## NA/NaN/Inf in foreign function call (arg 5)
```

```
## Warning in simpleLoess(y, x, w, span, degree = degree, parametric = parametric, : at -0.005
```

```
## Warning in simpleLoess(y, x, w, span, degree = degree, parametric = parametric, : radius 2.5e-05
```

```
## Warning in simpleLoess(y, x, w, span, degree = degree, parametric = parametric, : all data on boundary of neighborhood. make span bigger
```

```
## Warning in simpleLoess(y, x, w, span, degree = degree, parametric = parametric, : pseudoinverse used at -0.005
```

```
## Warning in simpleLoess(y, x, w, span, degree = degree, parametric = parametric, : neighborhood radius 0.005
```

```
## Warning in simpleLoess(y, x, w, span, degree = degree, parametric = parametric, : reciprocal condition number 1
```

```
## Warning in simpleLoess(y, x, w, span, degree = degree, parametric = parametric, : There are other near singularities as well. 1.01
```

```
## Warning in simpleLoess(y, x, w, span, degree = degree, parametric = parametric, : zero-width neighborhood. make span bigger
```

```
## Warning: Computation failed in `stat_smooth()`:
## NA/NaN/Inf in foreign function call (arg 5)
```

```
## Warning in simpleLoess(y, x, w, span, degree = degree, parametric = parametric, : at -0.005
```

```
## Warning in simpleLoess(y, x, w, span, degree = degree, parametric = parametric, : radius 2.5e-05
```

```
## Warning in simpleLoess(y, x, w, span, degree = degree, parametric = parametric, : all data on boundary of neighborhood. make span bigger
```

```
## Warning in simpleLoess(y, x, w, span, degree = degree, parametric = parametric, : pseudoinverse used at -0.005
```

```
## Warning in simpleLoess(y, x, w, span, degree = degree, parametric = parametric, : neighborhood radius 0.005
```

```
## Warning in simpleLoess(y, x, w, span, degree = degree, parametric = parametric, : reciprocal condition number 1
```

```
## Warning in simpleLoess(y, x, w, span, degree = degree, parametric = parametric, : There are other near singularities as well. 1.01
```

```
## Warning in simpleLoess(y, x, w, span, degree = degree, parametric = parametric, : zero-width neighborhood. make span bigger
```

```
## Warning: Computation failed in `stat_smooth()`:
## NA/NaN/Inf in foreign function call (arg 5)
```

```
## Warning in simpleLoess(y, x, w, span, degree = degree, parametric = parametric, : at -0.005
```

```
## Warning in simpleLoess(y, x, w, span, degree = degree, parametric = parametric, : radius 2.5e-05
```

```
## Warning in simpleLoess(y, x, w, span, degree = degree, parametric = parametric, : all data on boundary of neighborhood. make span bigger
```

```
## Warning in simpleLoess(y, x, w, span, degree = degree, parametric = parametric, : pseudoinverse used at -0.005
```

```
## Warning in simpleLoess(y, x, w, span, degree = degree, parametric = parametric, : neighborhood radius 0.005
```

```
## Warning in simpleLoess(y, x, w, span, degree = degree, parametric = parametric, : reciprocal condition number 1
```

```
## Warning in simpleLoess(y, x, w, span, degree = degree, parametric = parametric, : There are other near singularities as well. 1.01
```

```
## Warning in simpleLoess(y, x, w, span, degree = degree, parametric = parametric, : zero-width neighborhood. make span bigger
```

```
## Warning: Computation failed in `stat_smooth()`:
## NA/NaN/Inf in foreign function call (arg 5)
```

```
## Warning in simpleLoess(y, x, w, span, degree = degree, parametric = parametric, : at -0.005
```

```
## Warning in simpleLoess(y, x, w, span, degree = degree, parametric = parametric, : radius 2.5e-05
```

```
## Warning in simpleLoess(y, x, w, span, degree = degree, parametric = parametric, : all data on boundary of neighborhood. make span bigger
```

```
## Warning in simpleLoess(y, x, w, span, degree = degree, parametric = parametric, : pseudoinverse used at -0.005
```

```
## Warning in simpleLoess(y, x, w, span, degree = degree, parametric = parametric, : neighborhood radius 0.005
```

```
## Warning in simpleLoess(y, x, w, span, degree = degree, parametric = parametric, : reciprocal condition number 1
```

```
## Warning in simpleLoess(y, x, w, span, degree = degree, parametric = parametric, : There are other near singularities as well. 1.01
```

```
## Warning in simpleLoess(y, x, w, span, degree = degree, parametric = parametric, : zero-width neighborhood. make span bigger
```

```
## Warning: Computation failed in `stat_smooth()`:
## NA/NaN/Inf in foreign function call (arg 5)
```

```
## Warning in simpleLoess(y, x, w, span, degree = degree, parametric = parametric, : at -0.005
```

```
## Warning in simpleLoess(y, x, w, span, degree = degree, parametric = parametric, : radius 2.5e-05
```

```
## Warning in simpleLoess(y, x, w, span, degree = degree, parametric = parametric, : all data on boundary of neighborhood. make span bigger
```

```
## Warning in simpleLoess(y, x, w, span, degree = degree, parametric = parametric, : pseudoinverse used at -0.005
```

```
## Warning in simpleLoess(y, x, w, span, degree = degree, parametric = parametric, : neighborhood radius 0.005
```

```
## Warning in simpleLoess(y, x, w, span, degree = degree, parametric = parametric, : reciprocal condition number 1
```

```
## Warning in simpleLoess(y, x, w, span, degree = degree, parametric = parametric, : There are other near singularities as well. 1.01
```

```
## Warning in simpleLoess(y, x, w, span, degree = degree, parametric = parametric, : zero-width neighborhood. make span bigger
```

```
## Warning: Computation failed in `stat_smooth()`:
## NA/NaN/Inf in foreign function call (arg 5)
```

```
## Warning in simpleLoess(y, x, w, span, degree = degree, parametric = parametric, : at -0.005
```

```
## Warning in simpleLoess(y, x, w, span, degree = degree, parametric = parametric, : radius 2.5e-05
```

```
## Warning in simpleLoess(y, x, w, span, degree = degree, parametric = parametric, : all data on boundary of neighborhood. make span bigger
```

```
## Warning in simpleLoess(y, x, w, span, degree = degree, parametric = parametric, : pseudoinverse used at -0.005
```

```
## Warning in simpleLoess(y, x, w, span, degree = degree, parametric = parametric, : neighborhood radius 0.005
```

```
## Warning in simpleLoess(y, x, w, span, degree = degree, parametric = parametric, : reciprocal condition number 1
```

```
## Warning in simpleLoess(y, x, w, span, degree = degree, parametric = parametric, : There are other near singularities as well. 1.01
```

```
## Warning in simpleLoess(y, x, w, span, degree = degree, parametric = parametric, : zero-width neighborhood. make span bigger
```

```
## Warning: Computation failed in `stat_smooth()`:
## NA/NaN/Inf in foreign function call (arg 5)
```

```
## Warning in simpleLoess(y, x, w, span, degree = degree, parametric = parametric, : at -0.005
```

```
## Warning in simpleLoess(y, x, w, span, degree = degree, parametric = parametric, : radius 2.5e-05
```

```
## Warning in simpleLoess(y, x, w, span, degree = degree, parametric = parametric, : all data on boundary of neighborhood. make span bigger
```

```
## Warning in simpleLoess(y, x, w, span, degree = degree, parametric = parametric, : pseudoinverse used at -0.005
```

```
## Warning in simpleLoess(y, x, w, span, degree = degree, parametric = parametric, : neighborhood radius 0.005
```

```
## Warning in simpleLoess(y, x, w, span, degree = degree, parametric = parametric, : reciprocal condition number 1
```

```
## Warning in simpleLoess(y, x, w, span, degree = degree, parametric = parametric, : There are other near singularities as well. 1.01
```

```
## Warning in simpleLoess(y, x, w, span, degree = degree, parametric = parametric, : zero-width neighborhood. make span bigger
```

```
## Warning: Computation failed in `stat_smooth()`:
## NA/NaN/Inf in foreign function call (arg 5)
```

```
## Warning in simpleLoess(y, x, w, span, degree = degree, parametric = parametric, : at -0.005
```

```
## Warning in simpleLoess(y, x, w, span, degree = degree, parametric = parametric, : radius 2.5e-05
```

```
## Warning in simpleLoess(y, x, w, span, degree = degree, parametric = parametric, : all data on boundary of neighborhood. make span bigger
```

```
## Warning in simpleLoess(y, x, w, span, degree = degree, parametric = parametric, : pseudoinverse used at -0.005
```

```
## Warning in simpleLoess(y, x, w, span, degree = degree, parametric = parametric, : neighborhood radius 0.005
```

```
## Warning in simpleLoess(y, x, w, span, degree = degree, parametric = parametric, : reciprocal condition number 1
```

```
## Warning in simpleLoess(y, x, w, span, degree = degree, parametric = parametric, : There are other near singularities as well. 1.01
```

```
## Warning in simpleLoess(y, x, w, span, degree = degree, parametric = parametric, : zero-width neighborhood. make span bigger
```

```
## Warning: Computation failed in `stat_smooth()`:
## NA/NaN/Inf in foreign function call (arg 5)
```

```
## Warning in simpleLoess(y, x, w, span, degree = degree, parametric = parametric, : at -0.005
```

```
## Warning in simpleLoess(y, x, w, span, degree = degree, parametric = parametric, : radius 2.5e-05
```

```
## Warning in simpleLoess(y, x, w, span, degree = degree, parametric = parametric, : all data on boundary of neighborhood. make span bigger
```

```
## Warning in simpleLoess(y, x, w, span, degree = degree, parametric = parametric, : pseudoinverse used at -0.005
```

```
## Warning in simpleLoess(y, x, w, span, degree = degree, parametric = parametric, : neighborhood radius 0.005
```

```
## Warning in simpleLoess(y, x, w, span, degree = degree, parametric = parametric, : reciprocal condition number 1
```

```
## Warning in simpleLoess(y, x, w, span, degree = degree, parametric = parametric, : There are other near singularities as well. 1.01
```

```
## Warning in simpleLoess(y, x, w, span, degree = degree, parametric = parametric, : zero-width neighborhood. make span bigger
```

```
## Warning: Computation failed in `stat_smooth()`:
## NA/NaN/Inf in foreign function call (arg 5)
```

```
## Warning in simpleLoess(y, x, w, span, degree = degree, parametric = parametric, : at -0.005
```

```
## Warning in simpleLoess(y, x, w, span, degree = degree, parametric = parametric, : radius 2.5e-05
```

```
## Warning in simpleLoess(y, x, w, span, degree = degree, parametric = parametric, : all data on boundary of neighborhood. make span bigger
```

```
## Warning in simpleLoess(y, x, w, span, degree = degree, parametric = parametric, : pseudoinverse used at -0.005
```

```
## Warning in simpleLoess(y, x, w, span, degree = degree, parametric = parametric, : neighborhood radius 0.005
```

```
## Warning in simpleLoess(y, x, w, span, degree = degree, parametric = parametric, : reciprocal condition number 1
```

```
## Warning in simpleLoess(y, x, w, span, degree = degree, parametric = parametric, : There are other near singularities as well. 1.01
```

```
## Warning in simpleLoess(y, x, w, span, degree = degree, parametric = parametric, : zero-width neighborhood. make span bigger
```

```
## Warning: Computation failed in `stat_smooth()`:
## NA/NaN/Inf in foreign function call (arg 5)
```

```
## Warning in simpleLoess(y, x, w, span, degree = degree, parametric = parametric, : at -0.005
```

```
## Warning in simpleLoess(y, x, w, span, degree = degree, parametric = parametric, : radius 2.5e-05
```

```
## Warning in simpleLoess(y, x, w, span, degree = degree, parametric = parametric, : all data on boundary of neighborhood. make span bigger
```

```
## Warning in simpleLoess(y, x, w, span, degree = degree, parametric = parametric, : pseudoinverse used at -0.005
```

```
## Warning in simpleLoess(y, x, w, span, degree = degree, parametric = parametric, : neighborhood radius 0.005
```

```
## Warning in simpleLoess(y, x, w, span, degree = degree, parametric = parametric, : reciprocal condition number 1
```

```
## Warning in simpleLoess(y, x, w, span, degree = degree, parametric = parametric, : There are other near singularities as well. 1.01
```

```
## Warning in simpleLoess(y, x, w, span, degree = degree, parametric = parametric, : zero-width neighborhood. make span bigger
```

```
## Warning: Computation failed in `stat_smooth()`:
## NA/NaN/Inf in foreign function call (arg 5)
```

```
## Warning in simpleLoess(y, x, w, span, degree = degree, parametric = parametric, : at -0.005
```

```
## Warning in simpleLoess(y, x, w, span, degree = degree, parametric = parametric, : radius 2.5e-05
```

```
## Warning in simpleLoess(y, x, w, span, degree = degree, parametric = parametric, : all data on boundary of neighborhood. make span bigger
```

```
## Warning in simpleLoess(y, x, w, span, degree = degree, parametric = parametric, : pseudoinverse used at -0.005
```

```
## Warning in simpleLoess(y, x, w, span, degree = degree, parametric = parametric, : neighborhood radius 0.005
```

```
## Warning in simpleLoess(y, x, w, span, degree = degree, parametric = parametric, : reciprocal condition number 1
```

```
## Warning in simpleLoess(y, x, w, span, degree = degree, parametric = parametric, : There are other near singularities as well. 1.01
```

```
## Warning in simpleLoess(y, x, w, span, degree = degree, parametric = parametric, : zero-width neighborhood. make span bigger
```

```
## Warning: Computation failed in `stat_smooth()`:
## NA/NaN/Inf in foreign function call (arg 5)
```

```
## Warning in simpleLoess(y, x, w, span, degree = degree, parametric = parametric, : pseudoinverse used at -0.005
```

```
## Warning in simpleLoess(y, x, w, span, degree = degree, parametric = parametric, : neighborhood radius 1.005
```

```
## Warning in simpleLoess(y, x, w, span, degree = degree, parametric = parametric, : reciprocal condition number 0
```

```
## Warning in simpleLoess(y, x, w, span, degree = degree, parametric = parametric, : There are other near singularities as well. 1.01
```

```
## Warning in predLoess(object$y, object$x, newx = if (is.null(newdata)) object$x else if (is.data.frame(newdata)) as.matrix(model.frame(delete.response(terms(object)), : pseudoinverse used at -0.005
```

```
## Warning in predLoess(object$y, object$x, newx = if (is.null(newdata)) object$x else if (is.data.frame(newdata)) as.matrix(model.frame(delete.response(terms(object)), : neighborhood radius 1.005
```

```
## Warning in predLoess(object$y, object$x, newx = if (is.null(newdata)) object$x else if (is.data.frame(newdata)) as.matrix(model.frame(delete.response(terms(object)), : reciprocal condition number 0
```

```
## Warning in predLoess(object$y, object$x, newx = if (is.null(newdata)) object$x else if (is.data.frame(newdata)) as.matrix(model.frame(delete.response(terms(object)), : There are other near
## singularities as well. 1.01
```

```
## Warning in simpleLoess(y, x, w, span, degree = degree, parametric = parametric, : at -0.005
```

```
## Warning in simpleLoess(y, x, w, span, degree = degree, parametric = parametric, : radius 2.5e-05
```

```
## Warning in simpleLoess(y, x, w, span, degree = degree, parametric = parametric, : all data on boundary of neighborhood. make span bigger
```

```
## Warning in simpleLoess(y, x, w, span, degree = degree, parametric = parametric, : pseudoinverse used at -0.005
```

```
## Warning in simpleLoess(y, x, w, span, degree = degree, parametric = parametric, : neighborhood radius 0.005
```

```
## Warning in simpleLoess(y, x, w, span, degree = degree, parametric = parametric, : reciprocal condition number 1
```

```
## Warning in simpleLoess(y, x, w, span, degree = degree, parametric = parametric, : There are other near singularities as well. 1.01
```

```
## Warning in simpleLoess(y, x, w, span, degree = degree, parametric = parametric, : zero-width neighborhood. make span bigger
```

```
## Warning: Computation failed in `stat_smooth()`:
## NA/NaN/Inf in foreign function call (arg 5)
```

```
## Warning in simpleLoess(y, x, w, span, degree = degree, parametric = parametric, : at -0.005
```

```
## Warning in simpleLoess(y, x, w, span, degree = degree, parametric = parametric, : radius 2.5e-05
```

```
## Warning in simpleLoess(y, x, w, span, degree = degree, parametric = parametric, : all data on boundary of neighborhood. make span bigger
```

```
## Warning in simpleLoess(y, x, w, span, degree = degree, parametric = parametric, : pseudoinverse used at -0.005
```

```
## Warning in simpleLoess(y, x, w, span, degree = degree, parametric = parametric, : neighborhood radius 0.005
```

```
## Warning in simpleLoess(y, x, w, span, degree = degree, parametric = parametric, : reciprocal condition number 1
```

```
## Warning in simpleLoess(y, x, w, span, degree = degree, parametric = parametric, : There are other near singularities as well. 1.01
```

```
## Warning in simpleLoess(y, x, w, span, degree = degree, parametric = parametric, : zero-width neighborhood. make span bigger
```

```
## Warning: Computation failed in `stat_smooth()`:
## NA/NaN/Inf in foreign function call (arg 5)
```

```
## Warning in simpleLoess(y, x, w, span, degree = degree, parametric = parametric, : at -0.005
```

```
## Warning in simpleLoess(y, x, w, span, degree = degree, parametric = parametric, : radius 2.5e-05
```

```
## Warning in simpleLoess(y, x, w, span, degree = degree, parametric = parametric, : all data on boundary of neighborhood. make span bigger
```

```
## Warning in simpleLoess(y, x, w, span, degree = degree, parametric = parametric, : pseudoinverse used at -0.005
```

```
## Warning in simpleLoess(y, x, w, span, degree = degree, parametric = parametric, : neighborhood radius 0.005
```

```
## Warning in simpleLoess(y, x, w, span, degree = degree, parametric = parametric, : reciprocal condition number 1
```

```
## Warning in simpleLoess(y, x, w, span, degree = degree, parametric = parametric, : There are other near singularities as well. 1.01
```

```
## Warning in simpleLoess(y, x, w, span, degree = degree, parametric = parametric, : zero-width neighborhood. make span bigger
```

```
## Warning: Computation failed in `stat_smooth()`:
## NA/NaN/Inf in foreign function call (arg 5)
```

```
## Warning in simpleLoess(y, x, w, span, degree = degree, parametric = parametric, : at -0.005
```

```
## Warning in simpleLoess(y, x, w, span, degree = degree, parametric = parametric, : radius 2.5e-05
```

```
## Warning in simpleLoess(y, x, w, span, degree = degree, parametric = parametric, : all data on boundary of neighborhood. make span bigger
```

```
## Warning in simpleLoess(y, x, w, span, degree = degree, parametric = parametric, : pseudoinverse used at -0.005
```

```
## Warning in simpleLoess(y, x, w, span, degree = degree, parametric = parametric, : neighborhood radius 0.005
```

```
## Warning in simpleLoess(y, x, w, span, degree = degree, parametric = parametric, : reciprocal condition number 1
```

```
## Warning in simpleLoess(y, x, w, span, degree = degree, parametric = parametric, : There are other near singularities as well. 1.01
```

```
## Warning in simpleLoess(y, x, w, span, degree = degree, parametric = parametric, : zero-width neighborhood. make span bigger
```

```
## Warning: Computation failed in `stat_smooth()`:
## NA/NaN/Inf in foreign function call (arg 5)
```

```
## Warning in simpleLoess(y, x, w, span, degree = degree, parametric = parametric, : at -0.005
```

```
## Warning in simpleLoess(y, x, w, span, degree = degree, parametric = parametric, : radius 2.5e-05
```

```
## Warning in simpleLoess(y, x, w, span, degree = degree, parametric = parametric, : all data on boundary of neighborhood. make span bigger
```

```
## Warning in simpleLoess(y, x, w, span, degree = degree, parametric = parametric, : pseudoinverse used at -0.005
```

```
## Warning in simpleLoess(y, x, w, span, degree = degree, parametric = parametric, : neighborhood radius 0.005
```

```
## Warning in simpleLoess(y, x, w, span, degree = degree, parametric = parametric, : reciprocal condition number 1
```

```
## Warning in simpleLoess(y, x, w, span, degree = degree, parametric = parametric, : There are other near singularities as well. 1.01
```

```
## Warning in simpleLoess(y, x, w, span, degree = degree, parametric = parametric, : zero-width neighborhood. make span bigger
```

```
## Warning: Computation failed in `stat_smooth()`:
## NA/NaN/Inf in foreign function call (arg 5)
```

```
## Warning in simpleLoess(y, x, w, span, degree = degree, parametric = parametric, : pseudoinverse used at -0.005
```

```
## Warning in simpleLoess(y, x, w, span, degree = degree, parametric = parametric, : neighborhood radius 1.005
```

```
## Warning in simpleLoess(y, x, w, span, degree = degree, parametric = parametric, : reciprocal condition number 0
```

```
## Warning in simpleLoess(y, x, w, span, degree = degree, parametric = parametric, : There are other near singularities as well. 1.01
```

```
## Warning in predLoess(object$y, object$x, newx = if (is.null(newdata)) object$x else if (is.data.frame(newdata)) as.matrix(model.frame(delete.response(terms(object)), : pseudoinverse used at -0.005
```

```
## Warning in predLoess(object$y, object$x, newx = if (is.null(newdata)) object$x else if (is.data.frame(newdata)) as.matrix(model.frame(delete.response(terms(object)), : neighborhood radius 1.005
```

```
## Warning in predLoess(object$y, object$x, newx = if (is.null(newdata)) object$x else if (is.data.frame(newdata)) as.matrix(model.frame(delete.response(terms(object)), : reciprocal condition number 0
```

```
## Warning in predLoess(object$y, object$x, newx = if (is.null(newdata)) object$x else if (is.data.frame(newdata)) as.matrix(model.frame(delete.response(terms(object)), : There are other near
## singularities as well. 1.01
```

```
## Warning in simpleLoess(y, x, w, span, degree = degree, parametric = parametric, : at -0.005
```

```
## Warning in simpleLoess(y, x, w, span, degree = degree, parametric = parametric, : radius 2.5e-05
```

```
## Warning in simpleLoess(y, x, w, span, degree = degree, parametric = parametric, : all data on boundary of neighborhood. make span bigger
```

```
## Warning in simpleLoess(y, x, w, span, degree = degree, parametric = parametric, : pseudoinverse used at -0.005
```

```
## Warning in simpleLoess(y, x, w, span, degree = degree, parametric = parametric, : neighborhood radius 0.005
```

```
## Warning in simpleLoess(y, x, w, span, degree = degree, parametric = parametric, : reciprocal condition number 1
```

```
## Warning in simpleLoess(y, x, w, span, degree = degree, parametric = parametric, : There are other near singularities as well. 1.01
```

```
## Warning in simpleLoess(y, x, w, span, degree = degree, parametric = parametric, : zero-width neighborhood. make span bigger
```

```
## Warning: Computation failed in `stat_smooth()`:
## NA/NaN/Inf in foreign function call (arg 5)
```

```
## Warning in simpleLoess(y, x, w, span, degree = degree, parametric = parametric, : at -0.005
```

```
## Warning in simpleLoess(y, x, w, span, degree = degree, parametric = parametric, : radius 2.5e-05
```

```
## Warning in simpleLoess(y, x, w, span, degree = degree, parametric = parametric, : all data on boundary of neighborhood. make span bigger
```

```
## Warning in simpleLoess(y, x, w, span, degree = degree, parametric = parametric, : pseudoinverse used at -0.005
```

```
## Warning in simpleLoess(y, x, w, span, degree = degree, parametric = parametric, : neighborhood radius 0.005
```

```
## Warning in simpleLoess(y, x, w, span, degree = degree, parametric = parametric, : reciprocal condition number 1
```

```
## Warning in simpleLoess(y, x, w, span, degree = degree, parametric = parametric, : There are other near singularities as well. 1.01
```

```
## Warning in simpleLoess(y, x, w, span, degree = degree, parametric = parametric, : zero-width neighborhood. make span bigger
```

```
## Warning: Computation failed in `stat_smooth()`:
## NA/NaN/Inf in foreign function call (arg 5)
```

```
## Warning in simpleLoess(y, x, w, span, degree = degree, parametric = parametric, : at -0.005
```

```
## Warning in simpleLoess(y, x, w, span, degree = degree, parametric = parametric, : radius 2.5e-05
```

```
## Warning in simpleLoess(y, x, w, span, degree = degree, parametric = parametric, : all data on boundary of neighborhood. make span bigger
```

```
## Warning in simpleLoess(y, x, w, span, degree = degree, parametric = parametric, : pseudoinverse used at -0.005
```

```
## Warning in simpleLoess(y, x, w, span, degree = degree, parametric = parametric, : neighborhood radius 0.005
```

```
## Warning in simpleLoess(y, x, w, span, degree = degree, parametric = parametric, : reciprocal condition number 1
```

```
## Warning in simpleLoess(y, x, w, span, degree = degree, parametric = parametric, : There are other near singularities as well. 1.01
```

```
## Warning in simpleLoess(y, x, w, span, degree = degree, parametric = parametric, : zero-width neighborhood. make span bigger
```

```
## Warning: Computation failed in `stat_smooth()`:
## NA/NaN/Inf in foreign function call (arg 5)
```

```
## Warning in simpleLoess(y, x, w, span, degree = degree, parametric = parametric, : at -0.005
```

```
## Warning in simpleLoess(y, x, w, span, degree = degree, parametric = parametric, : radius 2.5e-05
```

```
## Warning in simpleLoess(y, x, w, span, degree = degree, parametric = parametric, : all data on boundary of neighborhood. make span bigger
```

```
## Warning in simpleLoess(y, x, w, span, degree = degree, parametric = parametric, : pseudoinverse used at -0.005
```

```
## Warning in simpleLoess(y, x, w, span, degree = degree, parametric = parametric, : neighborhood radius 0.005
```

```
## Warning in simpleLoess(y, x, w, span, degree = degree, parametric = parametric, : reciprocal condition number 1
```

```
## Warning in simpleLoess(y, x, w, span, degree = degree, parametric = parametric, : There are other near singularities as well. 1.01
```

```
## Warning in simpleLoess(y, x, w, span, degree = degree, parametric = parametric, : zero-width neighborhood. make span bigger
```

```
## Warning: Computation failed in `stat_smooth()`:
## NA/NaN/Inf in foreign function call (arg 5)
```

```
## Warning in simpleLoess(y, x, w, span, degree = degree, parametric = parametric, : at -0.005
```

```
## Warning in simpleLoess(y, x, w, span, degree = degree, parametric = parametric, : radius 2.5e-05
```

```
## Warning in simpleLoess(y, x, w, span, degree = degree, parametric = parametric, : all data on boundary of neighborhood. make span bigger
```

```
## Warning in simpleLoess(y, x, w, span, degree = degree, parametric = parametric, : pseudoinverse used at -0.005
```

```
## Warning in simpleLoess(y, x, w, span, degree = degree, parametric = parametric, : neighborhood radius 0.005
```

```
## Warning in simpleLoess(y, x, w, span, degree = degree, parametric = parametric, : reciprocal condition number 1
```

```
## Warning in simpleLoess(y, x, w, span, degree = degree, parametric = parametric, : There are other near singularities as well. 1.01
```

```
## Warning in simpleLoess(y, x, w, span, degree = degree, parametric = parametric, : zero-width neighborhood. make span bigger
```

```
## Warning: Computation failed in `stat_smooth()`:
## NA/NaN/Inf in foreign function call (arg 5)
```

```
## Warning in simpleLoess(y, x, w, span, degree = degree, parametric = parametric, : at -0.005
```

```
## Warning in simpleLoess(y, x, w, span, degree = degree, parametric = parametric, : radius 2.5e-05
```

```
## Warning in simpleLoess(y, x, w, span, degree = degree, parametric = parametric, : all data on boundary of neighborhood. make span bigger
```

```
## Warning in simpleLoess(y, x, w, span, degree = degree, parametric = parametric, : pseudoinverse used at -0.005
```

```
## Warning in simpleLoess(y, x, w, span, degree = degree, parametric = parametric, : neighborhood radius 0.005
```

```
## Warning in simpleLoess(y, x, w, span, degree = degree, parametric = parametric, : reciprocal condition number 1
```

```
## Warning in simpleLoess(y, x, w, span, degree = degree, parametric = parametric, : There are other near singularities as well. 1.01
```

```
## Warning in simpleLoess(y, x, w, span, degree = degree, parametric = parametric, : zero-width neighborhood. make span bigger
```

```
## Warning: Computation failed in `stat_smooth()`:
## NA/NaN/Inf in foreign function call (arg 5)
```

```
## Warning in simpleLoess(y, x, w, span, degree = degree, parametric = parametric, : at -0.005
```

```
## Warning in simpleLoess(y, x, w, span, degree = degree, parametric = parametric, : radius 2.5e-05
```

```
## Warning in simpleLoess(y, x, w, span, degree = degree, parametric = parametric, : all data on boundary of neighborhood. make span bigger
```

```
## Warning in simpleLoess(y, x, w, span, degree = degree, parametric = parametric, : pseudoinverse used at -0.005
```

```
## Warning in simpleLoess(y, x, w, span, degree = degree, parametric = parametric, : neighborhood radius 0.005
```

```
## Warning in simpleLoess(y, x, w, span, degree = degree, parametric = parametric, : reciprocal condition number 1
```

```
## Warning in simpleLoess(y, x, w, span, degree = degree, parametric = parametric, : There are other near singularities as well. 1.01
```

```
## Warning in simpleLoess(y, x, w, span, degree = degree, parametric = parametric, : zero-width neighborhood. make span bigger
```

```
## Warning: Computation failed in `stat_smooth()`:
## NA/NaN/Inf in foreign function call (arg 5)
```

```
## Warning in simpleLoess(y, x, w, span, degree = degree, parametric = parametric, : at -0.005
```

```
## Warning in simpleLoess(y, x, w, span, degree = degree, parametric = parametric, : radius 2.5e-05
```

```
## Warning in simpleLoess(y, x, w, span, degree = degree, parametric = parametric, : all data on boundary of neighborhood. make span bigger
```

```
## Warning in simpleLoess(y, x, w, span, degree = degree, parametric = parametric, : pseudoinverse used at -0.005
```

```
## Warning in simpleLoess(y, x, w, span, degree = degree, parametric = parametric, : neighborhood radius 0.005
```

```
## Warning in simpleLoess(y, x, w, span, degree = degree, parametric = parametric, : reciprocal condition number 1
```

```
## Warning in simpleLoess(y, x, w, span, degree = degree, parametric = parametric, : There are other near singularities as well. 1.01
```

```
## Warning in simpleLoess(y, x, w, span, degree = degree, parametric = parametric, : zero-width neighborhood. make span bigger
```

```
## Warning: Computation failed in `stat_smooth()`:
## NA/NaN/Inf in foreign function call (arg 5)
```

```
## Warning in simpleLoess(y, x, w, span, degree = degree, parametric = parametric, : at -0.005
```

```
## Warning in simpleLoess(y, x, w, span, degree = degree, parametric = parametric, : radius 2.5e-05
```

```
## Warning in simpleLoess(y, x, w, span, degree = degree, parametric = parametric, : all data on boundary of neighborhood. make span bigger
```

```
## Warning in simpleLoess(y, x, w, span, degree = degree, parametric = parametric, : pseudoinverse used at -0.005
```

```
## Warning in simpleLoess(y, x, w, span, degree = degree, parametric = parametric, : neighborhood radius 0.005
```

```
## Warning in simpleLoess(y, x, w, span, degree = degree, parametric = parametric, : reciprocal condition number 1
```

```
## Warning in simpleLoess(y, x, w, span, degree = degree, parametric = parametric, : There are other near singularities as well. 1.01
```

```
## Warning in simpleLoess(y, x, w, span, degree = degree, parametric = parametric, : zero-width neighborhood. make span bigger
```

```
## Warning: Computation failed in `stat_smooth()`:
## NA/NaN/Inf in foreign function call (arg 5)
```

```
## Warning in simpleLoess(y, x, w, span, degree = degree, parametric = parametric, : at -0.005
```

```
## Warning in simpleLoess(y, x, w, span, degree = degree, parametric = parametric, : radius 2.5e-05
```

```
## Warning in simpleLoess(y, x, w, span, degree = degree, parametric = parametric, : all data on boundary of neighborhood. make span bigger
```

```
## Warning in simpleLoess(y, x, w, span, degree = degree, parametric = parametric, : pseudoinverse used at -0.005
```

```
## Warning in simpleLoess(y, x, w, span, degree = degree, parametric = parametric, : neighborhood radius 0.005
```

```
## Warning in simpleLoess(y, x, w, span, degree = degree, parametric = parametric, : reciprocal condition number 1
```

```
## Warning in simpleLoess(y, x, w, span, degree = degree, parametric = parametric, : There are other near singularities as well. 1.01
```

```
## Warning in simpleLoess(y, x, w, span, degree = degree, parametric = parametric, : zero-width neighborhood. make span bigger
```

```
## Warning: Computation failed in `stat_smooth()`:
## NA/NaN/Inf in foreign function call (arg 5)
```

```
## Warning in simpleLoess(y, x, w, span, degree = degree, parametric = parametric, : at -0.005
```

```
## Warning in simpleLoess(y, x, w, span, degree = degree, parametric = parametric, : radius 2.5e-05
```

```
## Warning in simpleLoess(y, x, w, span, degree = degree, parametric = parametric, : all data on boundary of neighborhood. make span bigger
```

```
## Warning in simpleLoess(y, x, w, span, degree = degree, parametric = parametric, : pseudoinverse used at -0.005
```

```
## Warning in simpleLoess(y, x, w, span, degree = degree, parametric = parametric, : neighborhood radius 0.005
```

```
## Warning in simpleLoess(y, x, w, span, degree = degree, parametric = parametric, : reciprocal condition number 1
```

```
## Warning in simpleLoess(y, x, w, span, degree = degree, parametric = parametric, : There are other near singularities as well. 1.01
```

```
## Warning in simpleLoess(y, x, w, span, degree = degree, parametric = parametric, : zero-width neighborhood. make span bigger
```

```
## Warning: Computation failed in `stat_smooth()`:
## NA/NaN/Inf in foreign function call (arg 5)
```

```
## Warning in simpleLoess(y, x, w, span, degree = degree, parametric = parametric, : at -0.005
```

```
## Warning in simpleLoess(y, x, w, span, degree = degree, parametric = parametric, : radius 2.5e-05
```

```
## Warning in simpleLoess(y, x, w, span, degree = degree, parametric = parametric, : all data on boundary of neighborhood. make span bigger
```

```
## Warning in simpleLoess(y, x, w, span, degree = degree, parametric = parametric, : pseudoinverse used at -0.005
```

```
## Warning in simpleLoess(y, x, w, span, degree = degree, parametric = parametric, : neighborhood radius 0.005
```

```
## Warning in simpleLoess(y, x, w, span, degree = degree, parametric = parametric, : reciprocal condition number 1
```

```
## Warning in simpleLoess(y, x, w, span, degree = degree, parametric = parametric, : There are other near singularities as well. 1.01
```

```
## Warning in simpleLoess(y, x, w, span, degree = degree, parametric = parametric, : zero-width neighborhood. make span bigger
```

```
## Warning: Computation failed in `stat_smooth()`:
## NA/NaN/Inf in foreign function call (arg 5)
```

```
## Warning in simpleLoess(y, x, w, span, degree = degree, parametric = parametric, : at -0.005
```

```
## Warning in simpleLoess(y, x, w, span, degree = degree, parametric = parametric, : radius 2.5e-05
```

```
## Warning in simpleLoess(y, x, w, span, degree = degree, parametric = parametric, : all data on boundary of neighborhood. make span bigger
```

```
## Warning in simpleLoess(y, x, w, span, degree = degree, parametric = parametric, : pseudoinverse used at -0.005
```

```
## Warning in simpleLoess(y, x, w, span, degree = degree, parametric = parametric, : neighborhood radius 0.005
```

```
## Warning in simpleLoess(y, x, w, span, degree = degree, parametric = parametric, : reciprocal condition number 1
```

```
## Warning in simpleLoess(y, x, w, span, degree = degree, parametric = parametric, : There are other near singularities as well. 1.01
```

```
## Warning in simpleLoess(y, x, w, span, degree = degree, parametric = parametric, : zero-width neighborhood. make span bigger
```

```
## Warning: Computation failed in `stat_smooth()`:
## NA/NaN/Inf in foreign function call (arg 5)
```

```
## Warning in simpleLoess(y, x, w, span, degree = degree, parametric = parametric, : at -0.005
```

```
## Warning in simpleLoess(y, x, w, span, degree = degree, parametric = parametric, : radius 2.5e-05
```

```
## Warning in simpleLoess(y, x, w, span, degree = degree, parametric = parametric, : all data on boundary of neighborhood. make span bigger
```

```
## Warning in simpleLoess(y, x, w, span, degree = degree, parametric = parametric, : pseudoinverse used at -0.005
```

```
## Warning in simpleLoess(y, x, w, span, degree = degree, parametric = parametric, : neighborhood radius 0.005
```

```
## Warning in simpleLoess(y, x, w, span, degree = degree, parametric = parametric, : reciprocal condition number 1
```

```
## Warning in simpleLoess(y, x, w, span, degree = degree, parametric = parametric, : There are other near singularities as well. 1.01
```

```
## Warning in simpleLoess(y, x, w, span, degree = degree, parametric = parametric, : zero-width neighborhood. make span bigger
```

```
## Warning: Computation failed in `stat_smooth()`:
## NA/NaN/Inf in foreign function call (arg 5)
```

```
## Warning in simpleLoess(y, x, w, span, degree = degree, parametric = parametric, : at -0.005
```

```
## Warning in simpleLoess(y, x, w, span, degree = degree, parametric = parametric, : radius 2.5e-05
```

```
## Warning in simpleLoess(y, x, w, span, degree = degree, parametric = parametric, : all data on boundary of neighborhood. make span bigger
```

```
## Warning in simpleLoess(y, x, w, span, degree = degree, parametric = parametric, : pseudoinverse used at -0.005
```

```
## Warning in simpleLoess(y, x, w, span, degree = degree, parametric = parametric, : neighborhood radius 0.005
```

```
## Warning in simpleLoess(y, x, w, span, degree = degree, parametric = parametric, : reciprocal condition number 1
```

```
## Warning in simpleLoess(y, x, w, span, degree = degree, parametric = parametric, : There are other near singularities as well. 1.01
```

```
## Warning in simpleLoess(y, x, w, span, degree = degree, parametric = parametric, : zero-width neighborhood. make span bigger
```

```
## Warning: Computation failed in `stat_smooth()`:
## NA/NaN/Inf in foreign function call (arg 5)
```

```
## Warning in simpleLoess(y, x, w, span, degree = degree, parametric = parametric, : at -0.005
```

```
## Warning in simpleLoess(y, x, w, span, degree = degree, parametric = parametric, : radius 2.5e-05
```

```
## Warning in simpleLoess(y, x, w, span, degree = degree, parametric = parametric, : all data on boundary of neighborhood. make span bigger
```

```
## Warning in simpleLoess(y, x, w, span, degree = degree, parametric = parametric, : pseudoinverse used at -0.005
```

```
## Warning in simpleLoess(y, x, w, span, degree = degree, parametric = parametric, : neighborhood radius 0.005
```

```
## Warning in simpleLoess(y, x, w, span, degree = degree, parametric = parametric, : reciprocal condition number 1
```

```
## Warning in simpleLoess(y, x, w, span, degree = degree, parametric = parametric, : There are other near singularities as well. 1.01
```

```
## Warning in simpleLoess(y, x, w, span, degree = degree, parametric = parametric, : zero-width neighborhood. make span bigger
```

```
## Warning: Computation failed in `stat_smooth()`:
## NA/NaN/Inf in foreign function call (arg 5)
```

```
## Warning in simpleLoess(y, x, w, span, degree = degree, parametric = parametric, : at -0.005
```

```
## Warning in simpleLoess(y, x, w, span, degree = degree, parametric = parametric, : radius 2.5e-05
```

```
## Warning in simpleLoess(y, x, w, span, degree = degree, parametric = parametric, : all data on boundary of neighborhood. make span bigger
```

```
## Warning in simpleLoess(y, x, w, span, degree = degree, parametric = parametric, : pseudoinverse used at -0.005
```

```
## Warning in simpleLoess(y, x, w, span, degree = degree, parametric = parametric, : neighborhood radius 0.005
```

```
## Warning in simpleLoess(y, x, w, span, degree = degree, parametric = parametric, : reciprocal condition number 1
```

```
## Warning in simpleLoess(y, x, w, span, degree = degree, parametric = parametric, : There are other near singularities as well. 1.01
```

```
## Warning in simpleLoess(y, x, w, span, degree = degree, parametric = parametric, : zero-width neighborhood. make span bigger
```

```
## Warning: Computation failed in `stat_smooth()`:
## NA/NaN/Inf in foreign function call (arg 5)
```

```
## Warning in simpleLoess(y, x, w, span, degree = degree, parametric = parametric, : at -0.005
```

```
## Warning in simpleLoess(y, x, w, span, degree = degree, parametric = parametric, : radius 2.5e-05
```

```
## Warning in simpleLoess(y, x, w, span, degree = degree, parametric = parametric, : all data on boundary of neighborhood. make span bigger
```

```
## Warning in simpleLoess(y, x, w, span, degree = degree, parametric = parametric, : pseudoinverse used at -0.005
```

```
## Warning in simpleLoess(y, x, w, span, degree = degree, parametric = parametric, : neighborhood radius 0.005
```

```
## Warning in simpleLoess(y, x, w, span, degree = degree, parametric = parametric, : reciprocal condition number 1
```

```
## Warning in simpleLoess(y, x, w, span, degree = degree, parametric = parametric, : There are other near singularities as well. 1.01
```

```
## Warning in simpleLoess(y, x, w, span, degree = degree, parametric = parametric, : zero-width neighborhood. make span bigger
```

```
## Warning: Computation failed in `stat_smooth()`:
## NA/NaN/Inf in foreign function call (arg 5)
```

```
## Warning in simpleLoess(y, x, w, span, degree = degree, parametric = parametric, : at -0.005
```

```
## Warning in simpleLoess(y, x, w, span, degree = degree, parametric = parametric, : radius 2.5e-05
```

```
## Warning in simpleLoess(y, x, w, span, degree = degree, parametric = parametric, : all data on boundary of neighborhood. make span bigger
```

```
## Warning in simpleLoess(y, x, w, span, degree = degree, parametric = parametric, : pseudoinverse used at -0.005
```

```
## Warning in simpleLoess(y, x, w, span, degree = degree, parametric = parametric, : neighborhood radius 0.005
```

```
## Warning in simpleLoess(y, x, w, span, degree = degree, parametric = parametric, : reciprocal condition number 1
```

```
## Warning in simpleLoess(y, x, w, span, degree = degree, parametric = parametric, : There are other near singularities as well. 1.01
```

```
## Warning in simpleLoess(y, x, w, span, degree = degree, parametric = parametric, : zero-width neighborhood. make span bigger
```

```
## Warning: Computation failed in `stat_smooth()`:
## NA/NaN/Inf in foreign function call (arg 5)
```

```
## Warning in simpleLoess(y, x, w, span, degree = degree, parametric = parametric, : at -0.005
```

```
## Warning in simpleLoess(y, x, w, span, degree = degree, parametric = parametric, : radius 2.5e-05
```

```
## Warning in simpleLoess(y, x, w, span, degree = degree, parametric = parametric, : all data on boundary of neighborhood. make span bigger
```

```
## Warning in simpleLoess(y, x, w, span, degree = degree, parametric = parametric, : pseudoinverse used at -0.005
```

```
## Warning in simpleLoess(y, x, w, span, degree = degree, parametric = parametric, : neighborhood radius 0.005
```

```
## Warning in simpleLoess(y, x, w, span, degree = degree, parametric = parametric, : reciprocal condition number 1
```

```
## Warning in simpleLoess(y, x, w, span, degree = degree, parametric = parametric, : There are other near singularities as well. 1.01
```

```
## Warning in simpleLoess(y, x, w, span, degree = degree, parametric = parametric, : zero-width neighborhood. make span bigger
```

```
## Warning: Computation failed in `stat_smooth()`:
## NA/NaN/Inf in foreign function call (arg 5)
```

```
## Warning in simpleLoess(y, x, w, span, degree = degree, parametric = parametric, : at -0.005
```

```
## Warning in simpleLoess(y, x, w, span, degree = degree, parametric = parametric, : radius 2.5e-05
```

```
## Warning in simpleLoess(y, x, w, span, degree = degree, parametric = parametric, : all data on boundary of neighborhood. make span bigger
```

```
## Warning in simpleLoess(y, x, w, span, degree = degree, parametric = parametric, : pseudoinverse used at -0.005
```

```
## Warning in simpleLoess(y, x, w, span, degree = degree, parametric = parametric, : neighborhood radius 0.005
```

```
## Warning in simpleLoess(y, x, w, span, degree = degree, parametric = parametric, : reciprocal condition number 1
```

```
## Warning in simpleLoess(y, x, w, span, degree = degree, parametric = parametric, : There are other near singularities as well. 1.01
```

```
## Warning in simpleLoess(y, x, w, span, degree = degree, parametric = parametric, : zero-width neighborhood. make span bigger
```

```
## Warning: Computation failed in `stat_smooth()`:
## NA/NaN/Inf in foreign function call (arg 5)
```

```
## Warning in simpleLoess(y, x, w, span, degree = degree, parametric = parametric, : at -0.005
```

```
## Warning in simpleLoess(y, x, w, span, degree = degree, parametric = parametric, : radius 2.5e-05
```

```
## Warning in simpleLoess(y, x, w, span, degree = degree, parametric = parametric, : all data on boundary of neighborhood. make span bigger
```

```
## Warning in simpleLoess(y, x, w, span, degree = degree, parametric = parametric, : pseudoinverse used at -0.005
```

```
## Warning in simpleLoess(y, x, w, span, degree = degree, parametric = parametric, : neighborhood radius 0.005
```

```
## Warning in simpleLoess(y, x, w, span, degree = degree, parametric = parametric, : reciprocal condition number 1
```

```
## Warning in simpleLoess(y, x, w, span, degree = degree, parametric = parametric, : There are other near singularities as well. 1.01
```

```
## Warning in simpleLoess(y, x, w, span, degree = degree, parametric = parametric, : zero-width neighborhood. make span bigger
```

```
## Warning: Computation failed in `stat_smooth()`:
## NA/NaN/Inf in foreign function call (arg 5)
```

```
## Warning in simpleLoess(y, x, w, span, degree = degree, parametric = parametric, : at -0.005
```

```
## Warning in simpleLoess(y, x, w, span, degree = degree, parametric = parametric, : radius 2.5e-05
```

```
## Warning in simpleLoess(y, x, w, span, degree = degree, parametric = parametric, : all data on boundary of neighborhood. make span bigger
```

```
## Warning in simpleLoess(y, x, w, span, degree = degree, parametric = parametric, : pseudoinverse used at -0.005
```

```
## Warning in simpleLoess(y, x, w, span, degree = degree, parametric = parametric, : neighborhood radius 0.005
```

```
## Warning in simpleLoess(y, x, w, span, degree = degree, parametric = parametric, : reciprocal condition number 1
```

```
## Warning in simpleLoess(y, x, w, span, degree = degree, parametric = parametric, : There are other near singularities as well. 1.01
```

```
## Warning in simpleLoess(y, x, w, span, degree = degree, parametric = parametric, : zero-width neighborhood. make span bigger
```

```
## Warning: Computation failed in `stat_smooth()`:
## NA/NaN/Inf in foreign function call (arg 5)
```

```
## Warning in simpleLoess(y, x, w, span, degree = degree, parametric = parametric, : at -0.005
```

```
## Warning in simpleLoess(y, x, w, span, degree = degree, parametric = parametric, : radius 2.5e-05
```

```
## Warning in simpleLoess(y, x, w, span, degree = degree, parametric = parametric, : all data on boundary of neighborhood. make span bigger
```

```
## Warning in simpleLoess(y, x, w, span, degree = degree, parametric = parametric, : pseudoinverse used at -0.005
```

```
## Warning in simpleLoess(y, x, w, span, degree = degree, parametric = parametric, : neighborhood radius 0.005
```

```
## Warning in simpleLoess(y, x, w, span, degree = degree, parametric = parametric, : reciprocal condition number 1
```

```
## Warning in simpleLoess(y, x, w, span, degree = degree, parametric = parametric, : There are other near singularities as well. 1.01
```

```
## Warning in simpleLoess(y, x, w, span, degree = degree, parametric = parametric, : zero-width neighborhood. make span bigger
```

```
## Warning: Computation failed in `stat_smooth()`:
## NA/NaN/Inf in foreign function call (arg 5)
```

```
## Warning in simpleLoess(y, x, w, span, degree = degree, parametric = parametric, : at -0.005
```

```
## Warning in simpleLoess(y, x, w, span, degree = degree, parametric = parametric, : radius 2.5e-05
```

```
## Warning in simpleLoess(y, x, w, span, degree = degree, parametric = parametric, : all data on boundary of neighborhood. make span bigger
```

```
## Warning in simpleLoess(y, x, w, span, degree = degree, parametric = parametric, : pseudoinverse used at -0.005
```

```
## Warning in simpleLoess(y, x, w, span, degree = degree, parametric = parametric, : neighborhood radius 0.005
```

```
## Warning in simpleLoess(y, x, w, span, degree = degree, parametric = parametric, : reciprocal condition number 1
```

```
## Warning in simpleLoess(y, x, w, span, degree = degree, parametric = parametric, : There are other near singularities as well. 1.01
```

```
## Warning in simpleLoess(y, x, w, span, degree = degree, parametric = parametric, : zero-width neighborhood. make span bigger
```

```
## Warning: Computation failed in `stat_smooth()`:
## NA/NaN/Inf in foreign function call (arg 5)
```

```
## Warning in simpleLoess(y, x, w, span, degree = degree, parametric = parametric, : at -0.005
```

```
## Warning in simpleLoess(y, x, w, span, degree = degree, parametric = parametric, : radius 2.5e-05
```

```
## Warning in simpleLoess(y, x, w, span, degree = degree, parametric = parametric, : all data on boundary of neighborhood. make span bigger
```

```
## Warning in simpleLoess(y, x, w, span, degree = degree, parametric = parametric, : pseudoinverse used at -0.005
```

```
## Warning in simpleLoess(y, x, w, span, degree = degree, parametric = parametric, : neighborhood radius 0.005
```

```
## Warning in simpleLoess(y, x, w, span, degree = degree, parametric = parametric, : reciprocal condition number 1
```

```
## Warning in simpleLoess(y, x, w, span, degree = degree, parametric = parametric, : There are other near singularities as well. 1.01
```

```
## Warning in simpleLoess(y, x, w, span, degree = degree, parametric = parametric, : zero-width neighborhood. make span bigger
```

```
## Warning: Computation failed in `stat_smooth()`:
## NA/NaN/Inf in foreign function call (arg 5)
```

```
## Warning in simpleLoess(y, x, w, span, degree = degree, parametric = parametric, : at -0.005
```

```
## Warning in simpleLoess(y, x, w, span, degree = degree, parametric = parametric, : radius 2.5e-05
```

```
## Warning in simpleLoess(y, x, w, span, degree = degree, parametric = parametric, : all data on boundary of neighborhood. make span bigger
```

```
## Warning in simpleLoess(y, x, w, span, degree = degree, parametric = parametric, : pseudoinverse used at -0.005
```

```
## Warning in simpleLoess(y, x, w, span, degree = degree, parametric = parametric, : neighborhood radius 0.005
```

```
## Warning in simpleLoess(y, x, w, span, degree = degree, parametric = parametric, : reciprocal condition number 1
```

```
## Warning in simpleLoess(y, x, w, span, degree = degree, parametric = parametric, : There are other near singularities as well. 1.01
```

```
## Warning in simpleLoess(y, x, w, span, degree = degree, parametric = parametric, : zero-width neighborhood. make span bigger
```

```
## Warning: Computation failed in `stat_smooth()`:
## NA/NaN/Inf in foreign function call (arg 5)
```

```
## Warning in simpleLoess(y, x, w, span, degree = degree, parametric = parametric, : at -0.005
```

```
## Warning in simpleLoess(y, x, w, span, degree = degree, parametric = parametric, : radius 2.5e-05
```

```
## Warning in simpleLoess(y, x, w, span, degree = degree, parametric = parametric, : all data on boundary of neighborhood. make span bigger
```

```
## Warning in simpleLoess(y, x, w, span, degree = degree, parametric = parametric, : pseudoinverse used at -0.005
```

```
## Warning in simpleLoess(y, x, w, span, degree = degree, parametric = parametric, : neighborhood radius 0.005
```

```
## Warning in simpleLoess(y, x, w, span, degree = degree, parametric = parametric, : reciprocal condition number 1
```

```
## Warning in simpleLoess(y, x, w, span, degree = degree, parametric = parametric, : There are other near singularities as well. 1.01
```

```
## Warning in simpleLoess(y, x, w, span, degree = degree, parametric = parametric, : zero-width neighborhood. make span bigger
```

```
## Warning: Computation failed in `stat_smooth()`:
## NA/NaN/Inf in foreign function call (arg 5)
```

```
## Warning in simpleLoess(y, x, w, span, degree = degree, parametric = parametric, : at -0.005
```

```
## Warning in simpleLoess(y, x, w, span, degree = degree, parametric = parametric, : radius 2.5e-05
```

```
## Warning in simpleLoess(y, x, w, span, degree = degree, parametric = parametric, : all data on boundary of neighborhood. make span bigger
```

```
## Warning in simpleLoess(y, x, w, span, degree = degree, parametric = parametric, : pseudoinverse used at -0.005
```

```
## Warning in simpleLoess(y, x, w, span, degree = degree, parametric = parametric, : neighborhood radius 0.005
```

```
## Warning in simpleLoess(y, x, w, span, degree = degree, parametric = parametric, : reciprocal condition number 1
```

```
## Warning in simpleLoess(y, x, w, span, degree = degree, parametric = parametric, : There are other near singularities as well. 1.01
```

```
## Warning in simpleLoess(y, x, w, span, degree = degree, parametric = parametric, : zero-width neighborhood. make span bigger
```

```
## Warning: Computation failed in `stat_smooth()`:
## NA/NaN/Inf in foreign function call (arg 5)
```

```
## Warning in simpleLoess(y, x, w, span, degree = degree, parametric = parametric, : at -0.005
```

```
## Warning in simpleLoess(y, x, w, span, degree = degree, parametric = parametric, : radius 2.5e-05
```

```
## Warning in simpleLoess(y, x, w, span, degree = degree, parametric = parametric, : all data on boundary of neighborhood. make span bigger
```

```
## Warning in simpleLoess(y, x, w, span, degree = degree, parametric = parametric, : pseudoinverse used at -0.005
```

```
## Warning in simpleLoess(y, x, w, span, degree = degree, parametric = parametric, : neighborhood radius 0.005
```

```
## Warning in simpleLoess(y, x, w, span, degree = degree, parametric = parametric, : reciprocal condition number 1
```

```
## Warning in simpleLoess(y, x, w, span, degree = degree, parametric = parametric, : There are other near singularities as well. 1.01
```

```
## Warning in simpleLoess(y, x, w, span, degree = degree, parametric = parametric, : zero-width neighborhood. make span bigger
```

```
## Warning: Computation failed in `stat_smooth()`:
## NA/NaN/Inf in foreign function call (arg 5)
```

```
## Warning in simpleLoess(y, x, w, span, degree = degree, parametric = parametric, : at -0.005
```

```
## Warning in simpleLoess(y, x, w, span, degree = degree, parametric = parametric, : radius 2.5e-05
```

```
## Warning in simpleLoess(y, x, w, span, degree = degree, parametric = parametric, : all data on boundary of neighborhood. make span bigger
```

```
## Warning in simpleLoess(y, x, w, span, degree = degree, parametric = parametric, : pseudoinverse used at -0.005
```

```
## Warning in simpleLoess(y, x, w, span, degree = degree, parametric = parametric, : neighborhood radius 0.005
```

```
## Warning in simpleLoess(y, x, w, span, degree = degree, parametric = parametric, : reciprocal condition number 1
```

```
## Warning in simpleLoess(y, x, w, span, degree = degree, parametric = parametric, : There are other near singularities as well. 1.01
```

```
## Warning in simpleLoess(y, x, w, span, degree = degree, parametric = parametric, : zero-width neighborhood. make span bigger
```

```
## Warning: Computation failed in `stat_smooth()`:
## NA/NaN/Inf in foreign function call (arg 5)
```

```
## Warning in simpleLoess(y, x, w, span, degree = degree, parametric = parametric, : at -0.005
```

```
## Warning in simpleLoess(y, x, w, span, degree = degree, parametric = parametric, : radius 2.5e-05
```

```
## Warning in simpleLoess(y, x, w, span, degree = degree, parametric = parametric, : all data on boundary of neighborhood. make span bigger
```

```
## Warning in simpleLoess(y, x, w, span, degree = degree, parametric = parametric, : pseudoinverse used at -0.005
```

```
## Warning in simpleLoess(y, x, w, span, degree = degree, parametric = parametric, : neighborhood radius 0.005
```

```
## Warning in simpleLoess(y, x, w, span, degree = degree, parametric = parametric, : reciprocal condition number 1
```

```
## Warning in simpleLoess(y, x, w, span, degree = degree, parametric = parametric, : There are other near singularities as well. 1.01
```

```
## Warning in simpleLoess(y, x, w, span, degree = degree, parametric = parametric, : zero-width neighborhood. make span bigger
```

```
## Warning: Computation failed in `stat_smooth()`:
## NA/NaN/Inf in foreign function call (arg 5)
```

```
## Warning in simpleLoess(y, x, w, span, degree = degree, parametric = parametric, : at -0.005
```

```
## Warning in simpleLoess(y, x, w, span, degree = degree, parametric = parametric, : radius 2.5e-05
```

```
## Warning in simpleLoess(y, x, w, span, degree = degree, parametric = parametric, : all data on boundary of neighborhood. make span bigger
```

```
## Warning in simpleLoess(y, x, w, span, degree = degree, parametric = parametric, : pseudoinverse used at -0.005
```

```
## Warning in simpleLoess(y, x, w, span, degree = degree, parametric = parametric, : neighborhood radius 0.005
```

```
## Warning in simpleLoess(y, x, w, span, degree = degree, parametric = parametric, : reciprocal condition number 1
```

```
## Warning in simpleLoess(y, x, w, span, degree = degree, parametric = parametric, : There are other near singularities as well. 1.01
```

```
## Warning in simpleLoess(y, x, w, span, degree = degree, parametric = parametric, : zero-width neighborhood. make span bigger
```

```
## Warning: Computation failed in `stat_smooth()`:
## NA/NaN/Inf in foreign function call (arg 5)
```

```
## Warning in simpleLoess(y, x, w, span, degree = degree, parametric = parametric, : at -0.005
```

```
## Warning in simpleLoess(y, x, w, span, degree = degree, parametric = parametric, : radius 2.5e-05
```

```
## Warning in simpleLoess(y, x, w, span, degree = degree, parametric = parametric, : all data on boundary of neighborhood. make span bigger
```

```
## Warning in simpleLoess(y, x, w, span, degree = degree, parametric = parametric, : pseudoinverse used at -0.005
```

```
## Warning in simpleLoess(y, x, w, span, degree = degree, parametric = parametric, : neighborhood radius 0.005
```

```
## Warning in simpleLoess(y, x, w, span, degree = degree, parametric = parametric, : reciprocal condition number 1
```

```
## Warning in simpleLoess(y, x, w, span, degree = degree, parametric = parametric, : There are other near singularities as well. 1.01
```

```
## Warning in simpleLoess(y, x, w, span, degree = degree, parametric = parametric, : zero-width neighborhood. make span bigger
```

```
## Warning: Computation failed in `stat_smooth()`:
## NA/NaN/Inf in foreign function call (arg 5)
```

```
## Warning in simpleLoess(y, x, w, span, degree = degree, parametric = parametric, : at -0.005
```

```
## Warning in simpleLoess(y, x, w, span, degree = degree, parametric = parametric, : radius 2.5e-05
```

```
## Warning in simpleLoess(y, x, w, span, degree = degree, parametric = parametric, : all data on boundary of neighborhood. make span bigger
```

```
## Warning in simpleLoess(y, x, w, span, degree = degree, parametric = parametric, : pseudoinverse used at -0.005
```

```
## Warning in simpleLoess(y, x, w, span, degree = degree, parametric = parametric, : neighborhood radius 0.005
```

```
## Warning in simpleLoess(y, x, w, span, degree = degree, parametric = parametric, : reciprocal condition number 1
```

```
## Warning in simpleLoess(y, x, w, span, degree = degree, parametric = parametric, : There are other near singularities as well. 1.01
```

```
## Warning in simpleLoess(y, x, w, span, degree = degree, parametric = parametric, : zero-width neighborhood. make span bigger
```

```
## Warning: Computation failed in `stat_smooth()`:
## NA/NaN/Inf in foreign function call (arg 5)
```

```
## Warning in simpleLoess(y, x, w, span, degree = degree, parametric = parametric, : at -0.005
```

```
## Warning in simpleLoess(y, x, w, span, degree = degree, parametric = parametric, : radius 2.5e-05
```

```
## Warning in simpleLoess(y, x, w, span, degree = degree, parametric = parametric, : all data on boundary of neighborhood. make span bigger
```

```
## Warning in simpleLoess(y, x, w, span, degree = degree, parametric = parametric, : pseudoinverse used at -0.005
```

```
## Warning in simpleLoess(y, x, w, span, degree = degree, parametric = parametric, : neighborhood radius 0.005
```

```
## Warning in simpleLoess(y, x, w, span, degree = degree, parametric = parametric, : reciprocal condition number 1
```

```
## Warning in simpleLoess(y, x, w, span, degree = degree, parametric = parametric, : There are other near singularities as well. 1.01
```

```
## Warning in simpleLoess(y, x, w, span, degree = degree, parametric = parametric, : zero-width neighborhood. make span bigger
```

```
## Warning: Computation failed in `stat_smooth()`:
## NA/NaN/Inf in foreign function call (arg 5)
```

```
## Warning in simpleLoess(y, x, w, span, degree = degree, parametric = parametric, : at -0.005
```

```
## Warning in simpleLoess(y, x, w, span, degree = degree, parametric = parametric, : radius 2.5e-05
```

```
## Warning in simpleLoess(y, x, w, span, degree = degree, parametric = parametric, : all data on boundary of neighborhood. make span bigger
```

```
## Warning in simpleLoess(y, x, w, span, degree = degree, parametric = parametric, : pseudoinverse used at -0.005
```

```
## Warning in simpleLoess(y, x, w, span, degree = degree, parametric = parametric, : neighborhood radius 0.005
```

```
## Warning in simpleLoess(y, x, w, span, degree = degree, parametric = parametric, : reciprocal condition number 1
```

```
## Warning in simpleLoess(y, x, w, span, degree = degree, parametric = parametric, : There are other near singularities as well. 1.01
```

```
## Warning in simpleLoess(y, x, w, span, degree = degree, parametric = parametric, : zero-width neighborhood. make span bigger
```

```
## Warning: Computation failed in `stat_smooth()`:
## NA/NaN/Inf in foreign function call (arg 5)
```

```
## Warning in simpleLoess(y, x, w, span, degree = degree, parametric = parametric, : at -0.005
```

```
## Warning in simpleLoess(y, x, w, span, degree = degree, parametric = parametric, : radius 2.5e-05
```

```
## Warning in simpleLoess(y, x, w, span, degree = degree, parametric = parametric, : all data on boundary of neighborhood. make span bigger
```

```
## Warning in simpleLoess(y, x, w, span, degree = degree, parametric = parametric, : pseudoinverse used at -0.005
```

```
## Warning in simpleLoess(y, x, w, span, degree = degree, parametric = parametric, : neighborhood radius 0.005
```

```
## Warning in simpleLoess(y, x, w, span, degree = degree, parametric = parametric, : reciprocal condition number 1
```

```
## Warning in simpleLoess(y, x, w, span, degree = degree, parametric = parametric, : There are other near singularities as well. 1.01
```

```
## Warning in simpleLoess(y, x, w, span, degree = degree, parametric = parametric, : zero-width neighborhood. make span bigger
```

```
## Warning: Computation failed in `stat_smooth()`:
## NA/NaN/Inf in foreign function call (arg 5)
```

```
## Warning in simpleLoess(y, x, w, span, degree = degree, parametric = parametric, : at -0.005
```

```
## Warning in simpleLoess(y, x, w, span, degree = degree, parametric = parametric, : radius 2.5e-05
```

```
## Warning in simpleLoess(y, x, w, span, degree = degree, parametric = parametric, : all data on boundary of neighborhood. make span bigger
```

```
## Warning in simpleLoess(y, x, w, span, degree = degree, parametric = parametric, : pseudoinverse used at -0.005
```

```
## Warning in simpleLoess(y, x, w, span, degree = degree, parametric = parametric, : neighborhood radius 0.005
```

```
## Warning in simpleLoess(y, x, w, span, degree = degree, parametric = parametric, : reciprocal condition number 1
```

```
## Warning in simpleLoess(y, x, w, span, degree = degree, parametric = parametric, : There are other near singularities as well. 1.01
```

```
## Warning in simpleLoess(y, x, w, span, degree = degree, parametric = parametric, : zero-width neighborhood. make span bigger
```

```
## Warning: Computation failed in `stat_smooth()`:
## NA/NaN/Inf in foreign function call (arg 5)
```

```
## Warning in simpleLoess(y, x, w, span, degree = degree, parametric = parametric, : at -0.005
```

```
## Warning in simpleLoess(y, x, w, span, degree = degree, parametric = parametric, : radius 2.5e-05
```

```
## Warning in simpleLoess(y, x, w, span, degree = degree, parametric = parametric, : all data on boundary of neighborhood. make span bigger
```

```
## Warning in simpleLoess(y, x, w, span, degree = degree, parametric = parametric, : pseudoinverse used at -0.005
```

```
## Warning in simpleLoess(y, x, w, span, degree = degree, parametric = parametric, : neighborhood radius 0.005
```

```
## Warning in simpleLoess(y, x, w, span, degree = degree, parametric = parametric, : reciprocal condition number 1
```

```
## Warning in simpleLoess(y, x, w, span, degree = degree, parametric = parametric, : There are other near singularities as well. 1.01
```

```
## Warning in simpleLoess(y, x, w, span, degree = degree, parametric = parametric, : zero-width neighborhood. make span bigger
```

```
## Warning: Computation failed in `stat_smooth()`:
## NA/NaN/Inf in foreign function call (arg 5)
```

```
## Warning in simpleLoess(y, x, w, span, degree = degree, parametric = parametric, : at -0.005
```

```
## Warning in simpleLoess(y, x, w, span, degree = degree, parametric = parametric, : radius 2.5e-05
```

```
## Warning in simpleLoess(y, x, w, span, degree = degree, parametric = parametric, : all data on boundary of neighborhood. make span bigger
```

```
## Warning in simpleLoess(y, x, w, span, degree = degree, parametric = parametric, : pseudoinverse used at -0.005
```

```
## Warning in simpleLoess(y, x, w, span, degree = degree, parametric = parametric, : neighborhood radius 0.005
```

```
## Warning in simpleLoess(y, x, w, span, degree = degree, parametric = parametric, : reciprocal condition number 1
```

```
## Warning in simpleLoess(y, x, w, span, degree = degree, parametric = parametric, : There are other near singularities as well. 1.01
```

```
## Warning in simpleLoess(y, x, w, span, degree = degree, parametric = parametric, : zero-width neighborhood. make span bigger
```

```
## Warning: Computation failed in `stat_smooth()`:
## NA/NaN/Inf in foreign function call (arg 5)
```

```
## Warning in simpleLoess(y, x, w, span, degree = degree, parametric = parametric, : at -0.005
```

```
## Warning in simpleLoess(y, x, w, span, degree = degree, parametric = parametric, : radius 2.5e-05
```

```
## Warning in simpleLoess(y, x, w, span, degree = degree, parametric = parametric, : all data on boundary of neighborhood. make span bigger
```

```
## Warning in simpleLoess(y, x, w, span, degree = degree, parametric = parametric, : pseudoinverse used at -0.005
```

```
## Warning in simpleLoess(y, x, w, span, degree = degree, parametric = parametric, : neighborhood radius 0.005
```

```
## Warning in simpleLoess(y, x, w, span, degree = degree, parametric = parametric, : reciprocal condition number 1
```

```
## Warning in simpleLoess(y, x, w, span, degree = degree, parametric = parametric, : There are other near singularities as well. 1.01
```

```
## Warning in simpleLoess(y, x, w, span, degree = degree, parametric = parametric, : zero-width neighborhood. make span bigger
```

```
## Warning: Computation failed in `stat_smooth()`:
## NA/NaN/Inf in foreign function call (arg 5)
```

```
## Warning in simpleLoess(y, x, w, span, degree = degree, parametric = parametric, : at -0.005
```

```
## Warning in simpleLoess(y, x, w, span, degree = degree, parametric = parametric, : radius 2.5e-05
```

```
## Warning in simpleLoess(y, x, w, span, degree = degree, parametric = parametric, : all data on boundary of neighborhood. make span bigger
```

```
## Warning in simpleLoess(y, x, w, span, degree = degree, parametric = parametric, : pseudoinverse used at -0.005
```

```
## Warning in simpleLoess(y, x, w, span, degree = degree, parametric = parametric, : neighborhood radius 0.005
```

```
## Warning in simpleLoess(y, x, w, span, degree = degree, parametric = parametric, : reciprocal condition number 1
```

```
## Warning in simpleLoess(y, x, w, span, degree = degree, parametric = parametric, : There are other near singularities as well. 1.01
```

```
## Warning in simpleLoess(y, x, w, span, degree = degree, parametric = parametric, : zero-width neighborhood. make span bigger
```

```
## Warning: Computation failed in `stat_smooth()`:
## NA/NaN/Inf in foreign function call (arg 5)
```

```
## Warning in simpleLoess(y, x, w, span, degree = degree, parametric = parametric, : at -0.005
```

```
## Warning in simpleLoess(y, x, w, span, degree = degree, parametric = parametric, : radius 2.5e-05
```

```
## Warning in simpleLoess(y, x, w, span, degree = degree, parametric = parametric, : all data on boundary of neighborhood. make span bigger
```

```
## Warning in simpleLoess(y, x, w, span, degree = degree, parametric = parametric, : pseudoinverse used at -0.005
```

```
## Warning in simpleLoess(y, x, w, span, degree = degree, parametric = parametric, : neighborhood radius 0.005
```

```
## Warning in simpleLoess(y, x, w, span, degree = degree, parametric = parametric, : reciprocal condition number 1
```

```
## Warning in simpleLoess(y, x, w, span, degree = degree, parametric = parametric, : There are other near singularities as well. 1.01
```

```
## Warning in simpleLoess(y, x, w, span, degree = degree, parametric = parametric, : zero-width neighborhood. make span bigger
```

```
## Warning: Computation failed in `stat_smooth()`:
## NA/NaN/Inf in foreign function call (arg 5)
```

```
## Warning in simpleLoess(y, x, w, span, degree = degree, parametric = parametric, : pseudoinverse used at -0.005
```

```
## Warning in simpleLoess(y, x, w, span, degree = degree, parametric = parametric, : neighborhood radius 1.005
```

```
## Warning in simpleLoess(y, x, w, span, degree = degree, parametric = parametric, : reciprocal condition number 0
```

```
## Warning in simpleLoess(y, x, w, span, degree = degree, parametric = parametric, : There are other near singularities as well. 1.01
```

```
## Warning in predLoess(object$y, object$x, newx = if (is.null(newdata)) object$x else if (is.data.frame(newdata)) as.matrix(model.frame(delete.response(terms(object)), : pseudoinverse used at -0.005
```

```
## Warning in predLoess(object$y, object$x, newx = if (is.null(newdata)) object$x else if (is.data.frame(newdata)) as.matrix(model.frame(delete.response(terms(object)), : neighborhood radius 1.005
```

```
## Warning in predLoess(object$y, object$x, newx = if (is.null(newdata)) object$x else if (is.data.frame(newdata)) as.matrix(model.frame(delete.response(terms(object)), : reciprocal condition number 0
```

```
## Warning in predLoess(object$y, object$x, newx = if (is.null(newdata)) object$x else if (is.data.frame(newdata)) as.matrix(model.frame(delete.response(terms(object)), : There are other near
## singularities as well. 1.01
```

```
## Warning in simpleLoess(y, x, w, span, degree = degree, parametric = parametric, : pseudoinverse used at -0.005
```

```
## Warning in simpleLoess(y, x, w, span, degree = degree, parametric = parametric, : neighborhood radius 1.005
```

```
## Warning in simpleLoess(y, x, w, span, degree = degree, parametric = parametric, : reciprocal condition number 0
```

```
## Warning in simpleLoess(y, x, w, span, degree = degree, parametric = parametric, : There are other near singularities as well. 1.01
```

```
## Warning in predLoess(object$y, object$x, newx = if (is.null(newdata)) object$x else if (is.data.frame(newdata)) as.matrix(model.frame(delete.response(terms(object)), : pseudoinverse used at -0.005
```

```
## Warning in predLoess(object$y, object$x, newx = if (is.null(newdata)) object$x else if (is.data.frame(newdata)) as.matrix(model.frame(delete.response(terms(object)), : neighborhood radius 1.005
```

```
## Warning in predLoess(object$y, object$x, newx = if (is.null(newdata)) object$x else if (is.data.frame(newdata)) as.matrix(model.frame(delete.response(terms(object)), : reciprocal condition number 0
```

```
## Warning in predLoess(object$y, object$x, newx = if (is.null(newdata)) object$x else if (is.data.frame(newdata)) as.matrix(model.frame(delete.response(terms(object)), : There are other near
## singularities as well. 1.01
```

```
## Warning in simpleLoess(y, x, w, span, degree = degree, parametric = parametric, : at -0.005
```

```
## Warning in simpleLoess(y, x, w, span, degree = degree, parametric = parametric, : radius 2.5e-05
```

```
## Warning in simpleLoess(y, x, w, span, degree = degree, parametric = parametric, : all data on boundary of neighborhood. make span bigger
```

```
## Warning in simpleLoess(y, x, w, span, degree = degree, parametric = parametric, : pseudoinverse used at -0.005
```

```
## Warning in simpleLoess(y, x, w, span, degree = degree, parametric = parametric, : neighborhood radius 0.005
```

```
## Warning in simpleLoess(y, x, w, span, degree = degree, parametric = parametric, : reciprocal condition number 1
```

```
## Warning in simpleLoess(y, x, w, span, degree = degree, parametric = parametric, : There are other near singularities as well. 1.01
```

```
## Warning in simpleLoess(y, x, w, span, degree = degree, parametric = parametric, : zero-width neighborhood. make span bigger
```

```
## Warning: Computation failed in `stat_smooth()`:
## NA/NaN/Inf in foreign function call (arg 5)
```

```
## Warning in simpleLoess(y, x, w, span, degree = degree, parametric = parametric, : at -0.005
```

```
## Warning in simpleLoess(y, x, w, span, degree = degree, parametric = parametric, : radius 2.5e-05
```

```
## Warning in simpleLoess(y, x, w, span, degree = degree, parametric = parametric, : all data on boundary of neighborhood. make span bigger
```

```
## Warning in simpleLoess(y, x, w, span, degree = degree, parametric = parametric, : pseudoinverse used at -0.005
```

```
## Warning in simpleLoess(y, x, w, span, degree = degree, parametric = parametric, : neighborhood radius 0.005
```

```
## Warning in simpleLoess(y, x, w, span, degree = degree, parametric = parametric, : reciprocal condition number 1
```

```
## Warning in simpleLoess(y, x, w, span, degree = degree, parametric = parametric, : There are other near singularities as well. 1.01
```

```
## Warning in simpleLoess(y, x, w, span, degree = degree, parametric = parametric, : zero-width neighborhood. make span bigger
```

```
## Warning: Computation failed in `stat_smooth()`:
## NA/NaN/Inf in foreign function call (arg 5)
```

```
## Warning in simpleLoess(y, x, w, span, degree = degree, parametric = parametric, : at -0.005
```

```
## Warning in simpleLoess(y, x, w, span, degree = degree, parametric = parametric, : radius 2.5e-05
```

```
## Warning in simpleLoess(y, x, w, span, degree = degree, parametric = parametric, : all data on boundary of neighborhood. make span bigger
```

```
## Warning in simpleLoess(y, x, w, span, degree = degree, parametric = parametric, : pseudoinverse used at -0.005
```

```
## Warning in simpleLoess(y, x, w, span, degree = degree, parametric = parametric, : neighborhood radius 0.005
```

```
## Warning in simpleLoess(y, x, w, span, degree = degree, parametric = parametric, : reciprocal condition number 1
```

```
## Warning in simpleLoess(y, x, w, span, degree = degree, parametric = parametric, : There are other near singularities as well. 1.01
```

```
## Warning in simpleLoess(y, x, w, span, degree = degree, parametric = parametric, : zero-width neighborhood. make span bigger
```

```
## Warning: Computation failed in `stat_smooth()`:
## NA/NaN/Inf in foreign function call (arg 5)
```

```
## Warning in simpleLoess(y, x, w, span, degree = degree, parametric = parametric, : at -0.005
```

```
## Warning in simpleLoess(y, x, w, span, degree = degree, parametric = parametric, : radius 2.5e-05
```

```
## Warning in simpleLoess(y, x, w, span, degree = degree, parametric = parametric, : all data on boundary of neighborhood. make span bigger
```

```
## Warning in simpleLoess(y, x, w, span, degree = degree, parametric = parametric, : pseudoinverse used at -0.005
```

```
## Warning in simpleLoess(y, x, w, span, degree = degree, parametric = parametric, : neighborhood radius 0.005
```

```
## Warning in simpleLoess(y, x, w, span, degree = degree, parametric = parametric, : reciprocal condition number 1
```

```
## Warning in simpleLoess(y, x, w, span, degree = degree, parametric = parametric, : There are other near singularities as well. 1.01
```

```
## Warning in simpleLoess(y, x, w, span, degree = degree, parametric = parametric, : zero-width neighborhood. make span bigger
```

```
## Warning: Computation failed in `stat_smooth()`:
## NA/NaN/Inf in foreign function call (arg 5)
```

```
## Warning in simpleLoess(y, x, w, span, degree = degree, parametric = parametric, : at -0.005
```

```
## Warning in simpleLoess(y, x, w, span, degree = degree, parametric = parametric, : radius 2.5e-05
```

```
## Warning in simpleLoess(y, x, w, span, degree = degree, parametric = parametric, : all data on boundary of neighborhood. make span bigger
```

```
## Warning in simpleLoess(y, x, w, span, degree = degree, parametric = parametric, : pseudoinverse used at -0.005
```

```
## Warning in simpleLoess(y, x, w, span, degree = degree, parametric = parametric, : neighborhood radius 0.005
```

```
## Warning in simpleLoess(y, x, w, span, degree = degree, parametric = parametric, : reciprocal condition number 1
```

```
## Warning in simpleLoess(y, x, w, span, degree = degree, parametric = parametric, : There are other near singularities as well. 1.01
```

```
## Warning in simpleLoess(y, x, w, span, degree = degree, parametric = parametric, : zero-width neighborhood. make span bigger
```

```
## Warning: Computation failed in `stat_smooth()`:
## NA/NaN/Inf in foreign function call (arg 5)
```

```
## Warning in simpleLoess(y, x, w, span, degree = degree, parametric = parametric, : at -0.005
```

```
## Warning in simpleLoess(y, x, w, span, degree = degree, parametric = parametric, : radius 2.5e-05
```

```
## Warning in simpleLoess(y, x, w, span, degree = degree, parametric = parametric, : all data on boundary of neighborhood. make span bigger
```

```
## Warning in simpleLoess(y, x, w, span, degree = degree, parametric = parametric, : pseudoinverse used at -0.005
```

```
## Warning in simpleLoess(y, x, w, span, degree = degree, parametric = parametric, : neighborhood radius 0.005
```

```
## Warning in simpleLoess(y, x, w, span, degree = degree, parametric = parametric, : reciprocal condition number 1
```

```
## Warning in simpleLoess(y, x, w, span, degree = degree, parametric = parametric, : There are other near singularities as well. 1.01
```

```
## Warning in simpleLoess(y, x, w, span, degree = degree, parametric = parametric, : zero-width neighborhood. make span bigger
```

```
## Warning: Computation failed in `stat_smooth()`:
## NA/NaN/Inf in foreign function call (arg 5)
```

```
## Warning in simpleLoess(y, x, w, span, degree = degree, parametric = parametric, : at -0.005
```

```
## Warning in simpleLoess(y, x, w, span, degree = degree, parametric = parametric, : radius 2.5e-05
```

```
## Warning in simpleLoess(y, x, w, span, degree = degree, parametric = parametric, : all data on boundary of neighborhood. make span bigger
```

```
## Warning in simpleLoess(y, x, w, span, degree = degree, parametric = parametric, : pseudoinverse used at -0.005
```

```
## Warning in simpleLoess(y, x, w, span, degree = degree, parametric = parametric, : neighborhood radius 0.005
```

```
## Warning in simpleLoess(y, x, w, span, degree = degree, parametric = parametric, : reciprocal condition number 1
```

```
## Warning in simpleLoess(y, x, w, span, degree = degree, parametric = parametric, : There are other near singularities as well. 1.01
```

```
## Warning in simpleLoess(y, x, w, span, degree = degree, parametric = parametric, : zero-width neighborhood. make span bigger
```

```
## Warning: Computation failed in `stat_smooth()`:
## NA/NaN/Inf in foreign function call (arg 5)
```

```
## Warning in simpleLoess(y, x, w, span, degree = degree, parametric = parametric, : pseudoinverse used at -0.005
```

```
## Warning in simpleLoess(y, x, w, span, degree = degree, parametric = parametric, : neighborhood radius 1.005
```

```
## Warning in simpleLoess(y, x, w, span, degree = degree, parametric = parametric, : reciprocal condition number 0
```

```
## Warning in simpleLoess(y, x, w, span, degree = degree, parametric = parametric, : There are other near singularities as well. 1.01
```

```
## Warning in predLoess(object$y, object$x, newx = if (is.null(newdata)) object$x else if (is.data.frame(newdata)) as.matrix(model.frame(delete.response(terms(object)), : pseudoinverse used at -0.005
```

```
## Warning in predLoess(object$y, object$x, newx = if (is.null(newdata)) object$x else if (is.data.frame(newdata)) as.matrix(model.frame(delete.response(terms(object)), : neighborhood radius 1.005
```

```
## Warning in predLoess(object$y, object$x, newx = if (is.null(newdata)) object$x else if (is.data.frame(newdata)) as.matrix(model.frame(delete.response(terms(object)), : reciprocal condition number 0
```

```
## Warning in predLoess(object$y, object$x, newx = if (is.null(newdata)) object$x else if (is.data.frame(newdata)) as.matrix(model.frame(delete.response(terms(object)), : There are other near
## singularities as well. 1.01
```

```
## Warning in simpleLoess(y, x, w, span, degree = degree, parametric = parametric, : pseudoinverse used at -0.005
```

```
## Warning in simpleLoess(y, x, w, span, degree = degree, parametric = parametric, : neighborhood radius 1.005
```

```
## Warning in simpleLoess(y, x, w, span, degree = degree, parametric = parametric, : reciprocal condition number 0
```

```
## Warning in simpleLoess(y, x, w, span, degree = degree, parametric = parametric, : There are other near singularities as well. 1.01
```

```
## Warning in predLoess(object$y, object$x, newx = if (is.null(newdata)) object$x else if (is.data.frame(newdata)) as.matrix(model.frame(delete.response(terms(object)), : pseudoinverse used at -0.005
```

```
## Warning in predLoess(object$y, object$x, newx = if (is.null(newdata)) object$x else if (is.data.frame(newdata)) as.matrix(model.frame(delete.response(terms(object)), : neighborhood radius 1.005
```

```
## Warning in predLoess(object$y, object$x, newx = if (is.null(newdata)) object$x else if (is.data.frame(newdata)) as.matrix(model.frame(delete.response(terms(object)), : reciprocal condition number 0
```

```
## Warning in predLoess(object$y, object$x, newx = if (is.null(newdata)) object$x else if (is.data.frame(newdata)) as.matrix(model.frame(delete.response(terms(object)), : There are other near
## singularities as well. 1.01
```

```
## Warning in simpleLoess(y, x, w, span, degree = degree, parametric = parametric, : at -0.005
```

```
## Warning in simpleLoess(y, x, w, span, degree = degree, parametric = parametric, : radius 2.5e-05
```

```
## Warning in simpleLoess(y, x, w, span, degree = degree, parametric = parametric, : all data on boundary of neighborhood. make span bigger
```

```
## Warning in simpleLoess(y, x, w, span, degree = degree, parametric = parametric, : pseudoinverse used at -0.005
```

```
## Warning in simpleLoess(y, x, w, span, degree = degree, parametric = parametric, : neighborhood radius 0.005
```

```
## Warning in simpleLoess(y, x, w, span, degree = degree, parametric = parametric, : reciprocal condition number 1
```

```
## Warning in simpleLoess(y, x, w, span, degree = degree, parametric = parametric, : There are other near singularities as well. 1.01
```

```
## Warning in simpleLoess(y, x, w, span, degree = degree, parametric = parametric, : zero-width neighborhood. make span bigger
```

```
## Warning: Computation failed in `stat_smooth()`:
## NA/NaN/Inf in foreign function call (arg 5)
```

```
## Warning in simpleLoess(y, x, w, span, degree = degree, parametric = parametric, : at -0.005
```

```
## Warning in simpleLoess(y, x, w, span, degree = degree, parametric = parametric, : radius 2.5e-05
```

```
## Warning in simpleLoess(y, x, w, span, degree = degree, parametric = parametric, : all data on boundary of neighborhood. make span bigger
```

```
## Warning in simpleLoess(y, x, w, span, degree = degree, parametric = parametric, : pseudoinverse used at -0.005
```

```
## Warning in simpleLoess(y, x, w, span, degree = degree, parametric = parametric, : neighborhood radius 0.005
```

```
## Warning in simpleLoess(y, x, w, span, degree = degree, parametric = parametric, : reciprocal condition number 1
```

```
## Warning in simpleLoess(y, x, w, span, degree = degree, parametric = parametric, : There are other near singularities as well. 1.01
```

```
## Warning in simpleLoess(y, x, w, span, degree = degree, parametric = parametric, : zero-width neighborhood. make span bigger
```

```
## Warning: Computation failed in `stat_smooth()`:
## NA/NaN/Inf in foreign function call (arg 5)
```

```
## Warning in simpleLoess(y, x, w, span, degree = degree, parametric = parametric, : at -0.005
```

```
## Warning in simpleLoess(y, x, w, span, degree = degree, parametric = parametric, : radius 2.5e-05
```

```
## Warning in simpleLoess(y, x, w, span, degree = degree, parametric = parametric, : all data on boundary of neighborhood. make span bigger
```

```
## Warning in simpleLoess(y, x, w, span, degree = degree, parametric = parametric, : pseudoinverse used at -0.005
```

```
## Warning in simpleLoess(y, x, w, span, degree = degree, parametric = parametric, : neighborhood radius 0.005
```

```
## Warning in simpleLoess(y, x, w, span, degree = degree, parametric = parametric, : reciprocal condition number 1
```

```
## Warning in simpleLoess(y, x, w, span, degree = degree, parametric = parametric, : There are other near singularities as well. 1.01
```

```
## Warning in simpleLoess(y, x, w, span, degree = degree, parametric = parametric, : zero-width neighborhood. make span bigger
```

```
## Warning: Computation failed in `stat_smooth()`:
## NA/NaN/Inf in foreign function call (arg 5)
```

```
## Warning in simpleLoess(y, x, w, span, degree = degree, parametric = parametric, : at -0.005
```

```
## Warning in simpleLoess(y, x, w, span, degree = degree, parametric = parametric, : radius 2.5e-05
```

```
## Warning in simpleLoess(y, x, w, span, degree = degree, parametric = parametric, : all data on boundary of neighborhood. make span bigger
```

```
## Warning in simpleLoess(y, x, w, span, degree = degree, parametric = parametric, : pseudoinverse used at -0.005
```

```
## Warning in simpleLoess(y, x, w, span, degree = degree, parametric = parametric, : neighborhood radius 0.005
```

```
## Warning in simpleLoess(y, x, w, span, degree = degree, parametric = parametric, : reciprocal condition number 1
```

```
## Warning in simpleLoess(y, x, w, span, degree = degree, parametric = parametric, : There are other near singularities as well. 1.01
```

```
## Warning in simpleLoess(y, x, w, span, degree = degree, parametric = parametric, : zero-width neighborhood. make span bigger
```

```
## Warning: Computation failed in `stat_smooth()`:
## NA/NaN/Inf in foreign function call (arg 5)
```

```
## Warning in simpleLoess(y, x, w, span, degree = degree, parametric = parametric, : at -0.005
```

```
## Warning in simpleLoess(y, x, w, span, degree = degree, parametric = parametric, : radius 2.5e-05
```

```
## Warning in simpleLoess(y, x, w, span, degree = degree, parametric = parametric, : all data on boundary of neighborhood. make span bigger
```

```
## Warning in simpleLoess(y, x, w, span, degree = degree, parametric = parametric, : pseudoinverse used at -0.005
```

```
## Warning in simpleLoess(y, x, w, span, degree = degree, parametric = parametric, : neighborhood radius 0.005
```

```
## Warning in simpleLoess(y, x, w, span, degree = degree, parametric = parametric, : reciprocal condition number 1
```

```
## Warning in simpleLoess(y, x, w, span, degree = degree, parametric = parametric, : There are other near singularities as well. 1.01
```

```
## Warning in simpleLoess(y, x, w, span, degree = degree, parametric = parametric, : zero-width neighborhood. make span bigger
```

```
## Warning: Computation failed in `stat_smooth()`:
## NA/NaN/Inf in foreign function call (arg 5)
```

```
## Warning in simpleLoess(y, x, w, span, degree = degree, parametric = parametric, : at -0.005
```

```
## Warning in simpleLoess(y, x, w, span, degree = degree, parametric = parametric, : radius 2.5e-05
```

```
## Warning in simpleLoess(y, x, w, span, degree = degree, parametric = parametric, : all data on boundary of neighborhood. make span bigger
```

```
## Warning in simpleLoess(y, x, w, span, degree = degree, parametric = parametric, : pseudoinverse used at -0.005
```

```
## Warning in simpleLoess(y, x, w, span, degree = degree, parametric = parametric, : neighborhood radius 0.005
```

```
## Warning in simpleLoess(y, x, w, span, degree = degree, parametric = parametric, : reciprocal condition number 1
```

```
## Warning in simpleLoess(y, x, w, span, degree = degree, parametric = parametric, : There are other near singularities as well. 1.01
```

```
## Warning in simpleLoess(y, x, w, span, degree = degree, parametric = parametric, : zero-width neighborhood. make span bigger
```

```
## Warning: Computation failed in `stat_smooth()`:
## NA/NaN/Inf in foreign function call (arg 5)
```

```
## Warning in simpleLoess(y, x, w, span, degree = degree, parametric = parametric, : at -0.005
```

```
## Warning in simpleLoess(y, x, w, span, degree = degree, parametric = parametric, : radius 2.5e-05
```

```
## Warning in simpleLoess(y, x, w, span, degree = degree, parametric = parametric, : all data on boundary of neighborhood. make span bigger
```

```
## Warning in simpleLoess(y, x, w, span, degree = degree, parametric = parametric, : pseudoinverse used at -0.005
```

```
## Warning in simpleLoess(y, x, w, span, degree = degree, parametric = parametric, : neighborhood radius 0.005
```

```
## Warning in simpleLoess(y, x, w, span, degree = degree, parametric = parametric, : reciprocal condition number 1
```

```
## Warning in simpleLoess(y, x, w, span, degree = degree, parametric = parametric, : There are other near singularities as well. 1.01
```

```
## Warning in simpleLoess(y, x, w, span, degree = degree, parametric = parametric, : zero-width neighborhood. make span bigger
```

```
## Warning: Computation failed in `stat_smooth()`:
## NA/NaN/Inf in foreign function call (arg 5)
```

```
## Warning in simpleLoess(y, x, w, span, degree = degree, parametric = parametric, : at -0.005
```

```
## Warning in simpleLoess(y, x, w, span, degree = degree, parametric = parametric, : radius 2.5e-05
```

```
## Warning in simpleLoess(y, x, w, span, degree = degree, parametric = parametric, : all data on boundary of neighborhood. make span bigger
```

```
## Warning in simpleLoess(y, x, w, span, degree = degree, parametric = parametric, : pseudoinverse used at -0.005
```

```
## Warning in simpleLoess(y, x, w, span, degree = degree, parametric = parametric, : neighborhood radius 0.005
```

```
## Warning in simpleLoess(y, x, w, span, degree = degree, parametric = parametric, : reciprocal condition number 1
```

```
## Warning in simpleLoess(y, x, w, span, degree = degree, parametric = parametric, : There are other near singularities as well. 1.01
```

```
## Warning in simpleLoess(y, x, w, span, degree = degree, parametric = parametric, : zero-width neighborhood. make span bigger
```

```
## Warning: Computation failed in `stat_smooth()`:
## NA/NaN/Inf in foreign function call (arg 5)
```

```
## Warning in simpleLoess(y, x, w, span, degree = degree, parametric = parametric, : at -0.005
```

```
## Warning in simpleLoess(y, x, w, span, degree = degree, parametric = parametric, : radius 2.5e-05
```

```
## Warning in simpleLoess(y, x, w, span, degree = degree, parametric = parametric, : all data on boundary of neighborhood. make span bigger
```

```
## Warning in simpleLoess(y, x, w, span, degree = degree, parametric = parametric, : pseudoinverse used at -0.005
```

```
## Warning in simpleLoess(y, x, w, span, degree = degree, parametric = parametric, : neighborhood radius 0.005
```

```
## Warning in simpleLoess(y, x, w, span, degree = degree, parametric = parametric, : reciprocal condition number 1
```

```
## Warning in simpleLoess(y, x, w, span, degree = degree, parametric = parametric, : There are other near singularities as well. 1.01
```

```
## Warning in simpleLoess(y, x, w, span, degree = degree, parametric = parametric, : zero-width neighborhood. make span bigger
```

```
## Warning: Computation failed in `stat_smooth()`:
## NA/NaN/Inf in foreign function call (arg 5)
```

```
## Warning in simpleLoess(y, x, w, span, degree = degree, parametric = parametric, : pseudoinverse used at -0.005
```

```
## Warning in simpleLoess(y, x, w, span, degree = degree, parametric = parametric, : neighborhood radius 1.005
```

```
## Warning in simpleLoess(y, x, w, span, degree = degree, parametric = parametric, : reciprocal condition number 0
```

```
## Warning in simpleLoess(y, x, w, span, degree = degree, parametric = parametric, : There are other near singularities as well. 1.01
```

```
## Warning in predLoess(object$y, object$x, newx = if (is.null(newdata)) object$x else if (is.data.frame(newdata)) as.matrix(model.frame(delete.response(terms(object)), : pseudoinverse used at -0.005
```

```
## Warning in predLoess(object$y, object$x, newx = if (is.null(newdata)) object$x else if (is.data.frame(newdata)) as.matrix(model.frame(delete.response(terms(object)), : neighborhood radius 1.005
```

```
## Warning in predLoess(object$y, object$x, newx = if (is.null(newdata)) object$x else if (is.data.frame(newdata)) as.matrix(model.frame(delete.response(terms(object)), : reciprocal condition number 0
```

```
## Warning in predLoess(object$y, object$x, newx = if (is.null(newdata)) object$x else if (is.data.frame(newdata)) as.matrix(model.frame(delete.response(terms(object)), : There are other near
## singularities as well. 1.01
```

```
## Warning in simpleLoess(y, x, w, span, degree = degree, parametric = parametric, : at -0.005
```

```
## Warning in simpleLoess(y, x, w, span, degree = degree, parametric = parametric, : radius 2.5e-05
```

```
## Warning in simpleLoess(y, x, w, span, degree = degree, parametric = parametric, : all data on boundary of neighborhood. make span bigger
```

```
## Warning in simpleLoess(y, x, w, span, degree = degree, parametric = parametric, : pseudoinverse used at -0.005
```

```
## Warning in simpleLoess(y, x, w, span, degree = degree, parametric = parametric, : neighborhood radius 0.005
```

```
## Warning in simpleLoess(y, x, w, span, degree = degree, parametric = parametric, : reciprocal condition number 1
```

```
## Warning in simpleLoess(y, x, w, span, degree = degree, parametric = parametric, : There are other near singularities as well. 1.01
```

```
## Warning in simpleLoess(y, x, w, span, degree = degree, parametric = parametric, : zero-width neighborhood. make span bigger
```

```
## Warning: Computation failed in `stat_smooth()`:
## NA/NaN/Inf in foreign function call (arg 5)
```

```
## Warning in simpleLoess(y, x, w, span, degree = degree, parametric = parametric, : at -0.005
```

```
## Warning in simpleLoess(y, x, w, span, degree = degree, parametric = parametric, : radius 2.5e-05
```

```
## Warning in simpleLoess(y, x, w, span, degree = degree, parametric = parametric, : all data on boundary of neighborhood. make span bigger
```

```
## Warning in simpleLoess(y, x, w, span, degree = degree, parametric = parametric, : pseudoinverse used at -0.005
```

```
## Warning in simpleLoess(y, x, w, span, degree = degree, parametric = parametric, : neighborhood radius 0.005
```

```
## Warning in simpleLoess(y, x, w, span, degree = degree, parametric = parametric, : reciprocal condition number 1
```

```
## Warning in simpleLoess(y, x, w, span, degree = degree, parametric = parametric, : There are other near singularities as well. 1.01
```

```
## Warning in simpleLoess(y, x, w, span, degree = degree, parametric = parametric, : zero-width neighborhood. make span bigger
```

```
## Warning: Computation failed in `stat_smooth()`:
## NA/NaN/Inf in foreign function call (arg 5)
```

```
## Warning in simpleLoess(y, x, w, span, degree = degree, parametric = parametric, : at -0.005
```

```
## Warning in simpleLoess(y, x, w, span, degree = degree, parametric = parametric, : radius 2.5e-05
```

```
## Warning in simpleLoess(y, x, w, span, degree = degree, parametric = parametric, : all data on boundary of neighborhood. make span bigger
```

```
## Warning in simpleLoess(y, x, w, span, degree = degree, parametric = parametric, : pseudoinverse used at -0.005
```

```
## Warning in simpleLoess(y, x, w, span, degree = degree, parametric = parametric, : neighborhood radius 0.005
```

```
## Warning in simpleLoess(y, x, w, span, degree = degree, parametric = parametric, : reciprocal condition number 1
```

```
## Warning in simpleLoess(y, x, w, span, degree = degree, parametric = parametric, : There are other near singularities as well. 1.01
```

```
## Warning in simpleLoess(y, x, w, span, degree = degree, parametric = parametric, : zero-width neighborhood. make span bigger
```

```
## Warning: Computation failed in `stat_smooth()`:
## NA/NaN/Inf in foreign function call (arg 5)
```

```
## Warning in simpleLoess(y, x, w, span, degree = degree, parametric = parametric, : at -0.005
```

```
## Warning in simpleLoess(y, x, w, span, degree = degree, parametric = parametric, : radius 2.5e-05
```

```
## Warning in simpleLoess(y, x, w, span, degree = degree, parametric = parametric, : all data on boundary of neighborhood. make span bigger
```

```
## Warning in simpleLoess(y, x, w, span, degree = degree, parametric = parametric, : pseudoinverse used at -0.005
```

```
## Warning in simpleLoess(y, x, w, span, degree = degree, parametric = parametric, : neighborhood radius 0.005
```

```
## Warning in simpleLoess(y, x, w, span, degree = degree, parametric = parametric, : reciprocal condition number 1
```

```
## Warning in simpleLoess(y, x, w, span, degree = degree, parametric = parametric, : There are other near singularities as well. 1.01
```

```
## Warning in simpleLoess(y, x, w, span, degree = degree, parametric = parametric, : zero-width neighborhood. make span bigger
```

```
## Warning: Computation failed in `stat_smooth()`:
## NA/NaN/Inf in foreign function call (arg 5)
```

```
## Warning in simpleLoess(y, x, w, span, degree = degree, parametric = parametric, : at -0.005
```

```
## Warning in simpleLoess(y, x, w, span, degree = degree, parametric = parametric, : radius 2.5e-05
```

```
## Warning in simpleLoess(y, x, w, span, degree = degree, parametric = parametric, : all data on boundary of neighborhood. make span bigger
```

```
## Warning in simpleLoess(y, x, w, span, degree = degree, parametric = parametric, : pseudoinverse used at -0.005
```

```
## Warning in simpleLoess(y, x, w, span, degree = degree, parametric = parametric, : neighborhood radius 0.005
```

```
## Warning in simpleLoess(y, x, w, span, degree = degree, parametric = parametric, : reciprocal condition number 1
```

```
## Warning in simpleLoess(y, x, w, span, degree = degree, parametric = parametric, : There are other near singularities as well. 1.01
```

```
## Warning in simpleLoess(y, x, w, span, degree = degree, parametric = parametric, : zero-width neighborhood. make span bigger
```

```
## Warning: Computation failed in `stat_smooth()`:
## NA/NaN/Inf in foreign function call (arg 5)
```

```
## Warning in simpleLoess(y, x, w, span, degree = degree, parametric = parametric, : pseudoinverse used at -0.005
```

```
## Warning in simpleLoess(y, x, w, span, degree = degree, parametric = parametric, : neighborhood radius 1.005
```

```
## Warning in simpleLoess(y, x, w, span, degree = degree, parametric = parametric, : reciprocal condition number 0
```

```
## Warning in simpleLoess(y, x, w, span, degree = degree, parametric = parametric, : There are other near singularities as well. 1.01
```

```
## Warning in predLoess(object$y, object$x, newx = if (is.null(newdata)) object$x else if (is.data.frame(newdata)) as.matrix(model.frame(delete.response(terms(object)), : pseudoinverse used at -0.005
```

```
## Warning in predLoess(object$y, object$x, newx = if (is.null(newdata)) object$x else if (is.data.frame(newdata)) as.matrix(model.frame(delete.response(terms(object)), : neighborhood radius 1.005
```

```
## Warning in predLoess(object$y, object$x, newx = if (is.null(newdata)) object$x else if (is.data.frame(newdata)) as.matrix(model.frame(delete.response(terms(object)), : reciprocal condition number 0
```

```
## Warning in predLoess(object$y, object$x, newx = if (is.null(newdata)) object$x else if (is.data.frame(newdata)) as.matrix(model.frame(delete.response(terms(object)), : There are other near
## singularities as well. 1.01
```

```
## Warning in simpleLoess(y, x, w, span, degree = degree, parametric = parametric, : at -0.005
```

```
## Warning in simpleLoess(y, x, w, span, degree = degree, parametric = parametric, : radius 2.5e-05
```

```
## Warning in simpleLoess(y, x, w, span, degree = degree, parametric = parametric, : all data on boundary of neighborhood. make span bigger
```

```
## Warning in simpleLoess(y, x, w, span, degree = degree, parametric = parametric, : pseudoinverse used at -0.005
```

```
## Warning in simpleLoess(y, x, w, span, degree = degree, parametric = parametric, : neighborhood radius 0.005
```

```
## Warning in simpleLoess(y, x, w, span, degree = degree, parametric = parametric, : reciprocal condition number 1
```

```
## Warning in simpleLoess(y, x, w, span, degree = degree, parametric = parametric, : There are other near singularities as well. 1.01
```

```
## Warning in simpleLoess(y, x, w, span, degree = degree, parametric = parametric, : zero-width neighborhood. make span bigger
```

```
## Warning: Computation failed in `stat_smooth()`:
## NA/NaN/Inf in foreign function call (arg 5)
```

```
## Warning in simpleLoess(y, x, w, span, degree = degree, parametric = parametric, : at -0.005
```

```
## Warning in simpleLoess(y, x, w, span, degree = degree, parametric = parametric, : radius 2.5e-05
```

```
## Warning in simpleLoess(y, x, w, span, degree = degree, parametric = parametric, : all data on boundary of neighborhood. make span bigger
```

```
## Warning in simpleLoess(y, x, w, span, degree = degree, parametric = parametric, : pseudoinverse used at -0.005
```

```
## Warning in simpleLoess(y, x, w, span, degree = degree, parametric = parametric, : neighborhood radius 0.005
```

```
## Warning in simpleLoess(y, x, w, span, degree = degree, parametric = parametric, : reciprocal condition number 1
```

```
## Warning in simpleLoess(y, x, w, span, degree = degree, parametric = parametric, : There are other near singularities as well. 1.01
```

```
## Warning in simpleLoess(y, x, w, span, degree = degree, parametric = parametric, : zero-width neighborhood. make span bigger
```

```
## Warning: Computation failed in `stat_smooth()`:
## NA/NaN/Inf in foreign function call (arg 5)
```

```
## Warning in simpleLoess(y, x, w, span, degree = degree, parametric = parametric, : at -0.005
```

```
## Warning in simpleLoess(y, x, w, span, degree = degree, parametric = parametric, : radius 2.5e-05
```

```
## Warning in simpleLoess(y, x, w, span, degree = degree, parametric = parametric, : all data on boundary of neighborhood. make span bigger
```

```
## Warning in simpleLoess(y, x, w, span, degree = degree, parametric = parametric, : pseudoinverse used at -0.005
```

```
## Warning in simpleLoess(y, x, w, span, degree = degree, parametric = parametric, : neighborhood radius 0.005
```

```
## Warning in simpleLoess(y, x, w, span, degree = degree, parametric = parametric, : reciprocal condition number 1
```

```
## Warning in simpleLoess(y, x, w, span, degree = degree, parametric = parametric, : There are other near singularities as well. 1.01
```

```
## Warning in simpleLoess(y, x, w, span, degree = degree, parametric = parametric, : zero-width neighborhood. make span bigger
```

```
## Warning: Computation failed in `stat_smooth()`:
## NA/NaN/Inf in foreign function call (arg 5)
```

```
## Warning in simpleLoess(y, x, w, span, degree = degree, parametric = parametric, : at -0.005
```

```
## Warning in simpleLoess(y, x, w, span, degree = degree, parametric = parametric, : radius 2.5e-05
```

```
## Warning in simpleLoess(y, x, w, span, degree = degree, parametric = parametric, : all data on boundary of neighborhood. make span bigger
```

```
## Warning in simpleLoess(y, x, w, span, degree = degree, parametric = parametric, : pseudoinverse used at -0.005
```

```
## Warning in simpleLoess(y, x, w, span, degree = degree, parametric = parametric, : neighborhood radius 0.005
```

```
## Warning in simpleLoess(y, x, w, span, degree = degree, parametric = parametric, : reciprocal condition number 1
```

```
## Warning in simpleLoess(y, x, w, span, degree = degree, parametric = parametric, : There are other near singularities as well. 1.01
```

```
## Warning in simpleLoess(y, x, w, span, degree = degree, parametric = parametric, : zero-width neighborhood. make span bigger
```

```
## Warning: Computation failed in `stat_smooth()`:
## NA/NaN/Inf in foreign function call (arg 5)
```

```
## Warning in simpleLoess(y, x, w, span, degree = degree, parametric = parametric, : at -0.005
```

```
## Warning in simpleLoess(y, x, w, span, degree = degree, parametric = parametric, : radius 2.5e-05
```

```
## Warning in simpleLoess(y, x, w, span, degree = degree, parametric = parametric, : all data on boundary of neighborhood. make span bigger
```

```
## Warning in simpleLoess(y, x, w, span, degree = degree, parametric = parametric, : pseudoinverse used at -0.005
```

```
## Warning in simpleLoess(y, x, w, span, degree = degree, parametric = parametric, : neighborhood radius 0.005
```

```
## Warning in simpleLoess(y, x, w, span, degree = degree, parametric = parametric, : reciprocal condition number 1
```

```
## Warning in simpleLoess(y, x, w, span, degree = degree, parametric = parametric, : There are other near singularities as well. 1.01
```

```
## Warning in simpleLoess(y, x, w, span, degree = degree, parametric = parametric, : zero-width neighborhood. make span bigger
```

```
## Warning: Computation failed in `stat_smooth()`:
## NA/NaN/Inf in foreign function call (arg 5)
```

```
## Warning in simpleLoess(y, x, w, span, degree = degree, parametric = parametric, : at -0.005
```

```
## Warning in simpleLoess(y, x, w, span, degree = degree, parametric = parametric, : radius 2.5e-05
```

```
## Warning in simpleLoess(y, x, w, span, degree = degree, parametric = parametric, : all data on boundary of neighborhood. make span bigger
```

```
## Warning in simpleLoess(y, x, w, span, degree = degree, parametric = parametric, : pseudoinverse used at -0.005
```

```
## Warning in simpleLoess(y, x, w, span, degree = degree, parametric = parametric, : neighborhood radius 0.005
```

```
## Warning in simpleLoess(y, x, w, span, degree = degree, parametric = parametric, : reciprocal condition number 1
```

```
## Warning in simpleLoess(y, x, w, span, degree = degree, parametric = parametric, : There are other near singularities as well. 1.01
```

```
## Warning in simpleLoess(y, x, w, span, degree = degree, parametric = parametric, : zero-width neighborhood. make span bigger
```

```
## Warning: Computation failed in `stat_smooth()`:
## NA/NaN/Inf in foreign function call (arg 5)
```

```
## Warning in simpleLoess(y, x, w, span, degree = degree, parametric = parametric, : at -0.005
```

```
## Warning in simpleLoess(y, x, w, span, degree = degree, parametric = parametric, : radius 2.5e-05
```

```
## Warning in simpleLoess(y, x, w, span, degree = degree, parametric = parametric, : all data on boundary of neighborhood. make span bigger
```

```
## Warning in simpleLoess(y, x, w, span, degree = degree, parametric = parametric, : pseudoinverse used at -0.005
```

```
## Warning in simpleLoess(y, x, w, span, degree = degree, parametric = parametric, : neighborhood radius 0.005
```

```
## Warning in simpleLoess(y, x, w, span, degree = degree, parametric = parametric, : reciprocal condition number 1
```

```
## Warning in simpleLoess(y, x, w, span, degree = degree, parametric = parametric, : There are other near singularities as well. 1.01
```

```
## Warning in simpleLoess(y, x, w, span, degree = degree, parametric = parametric, : zero-width neighborhood. make span bigger
```

```
## Warning: Computation failed in `stat_smooth()`:
## NA/NaN/Inf in foreign function call (arg 5)
```

```
## Warning in simpleLoess(y, x, w, span, degree = degree, parametric = parametric, : pseudoinverse used at -0.005
```

```
## Warning in simpleLoess(y, x, w, span, degree = degree, parametric = parametric, : neighborhood radius 1.005
```

```
## Warning in simpleLoess(y, x, w, span, degree = degree, parametric = parametric, : reciprocal condition number 0
```

```
## Warning in simpleLoess(y, x, w, span, degree = degree, parametric = parametric, : There are other near singularities as well. 1.01
```

```
## Warning in predLoess(object$y, object$x, newx = if (is.null(newdata)) object$x else if (is.data.frame(newdata)) as.matrix(model.frame(delete.response(terms(object)), : pseudoinverse used at -0.005
```

```
## Warning in predLoess(object$y, object$x, newx = if (is.null(newdata)) object$x else if (is.data.frame(newdata)) as.matrix(model.frame(delete.response(terms(object)), : neighborhood radius 1.005
```

```
## Warning in predLoess(object$y, object$x, newx = if (is.null(newdata)) object$x else if (is.data.frame(newdata)) as.matrix(model.frame(delete.response(terms(object)), : reciprocal condition number 0
```

```
## Warning in predLoess(object$y, object$x, newx = if (is.null(newdata)) object$x else if (is.data.frame(newdata)) as.matrix(model.frame(delete.response(terms(object)), : There are other near
## singularities as well. 1.01
```

```
## Warning in simpleLoess(y, x, w, span, degree = degree, parametric = parametric, : at -0.005
```

```
## Warning in simpleLoess(y, x, w, span, degree = degree, parametric = parametric, : radius 2.5e-05
```

```
## Warning in simpleLoess(y, x, w, span, degree = degree, parametric = parametric, : all data on boundary of neighborhood. make span bigger
```

```
## Warning in simpleLoess(y, x, w, span, degree = degree, parametric = parametric, : pseudoinverse used at -0.005
```

```
## Warning in simpleLoess(y, x, w, span, degree = degree, parametric = parametric, : neighborhood radius 0.005
```

```
## Warning in simpleLoess(y, x, w, span, degree = degree, parametric = parametric, : reciprocal condition number 1
```

```
## Warning in simpleLoess(y, x, w, span, degree = degree, parametric = parametric, : There are other near singularities as well. 1.01
```

```
## Warning in simpleLoess(y, x, w, span, degree = degree, parametric = parametric, : zero-width neighborhood. make span bigger
```

```
## Warning: Computation failed in `stat_smooth()`:
## NA/NaN/Inf in foreign function call (arg 5)
```

```
## Warning in simpleLoess(y, x, w, span, degree = degree, parametric = parametric, : at -0.005
```

```
## Warning in simpleLoess(y, x, w, span, degree = degree, parametric = parametric, : radius 2.5e-05
```

```
## Warning in simpleLoess(y, x, w, span, degree = degree, parametric = parametric, : all data on boundary of neighborhood. make span bigger
```

```
## Warning in simpleLoess(y, x, w, span, degree = degree, parametric = parametric, : pseudoinverse used at -0.005
```

```
## Warning in simpleLoess(y, x, w, span, degree = degree, parametric = parametric, : neighborhood radius 0.005
```

```
## Warning in simpleLoess(y, x, w, span, degree = degree, parametric = parametric, : reciprocal condition number 1
```

```
## Warning in simpleLoess(y, x, w, span, degree = degree, parametric = parametric, : There are other near singularities as well. 1.01
```

```
## Warning in simpleLoess(y, x, w, span, degree = degree, parametric = parametric, : zero-width neighborhood. make span bigger
```

```
## Warning: Computation failed in `stat_smooth()`:
## NA/NaN/Inf in foreign function call (arg 5)
```

```
## Warning in simpleLoess(y, x, w, span, degree = degree, parametric = parametric, : at -0.005
```

```
## Warning in simpleLoess(y, x, w, span, degree = degree, parametric = parametric, : radius 2.5e-05
```

```
## Warning in simpleLoess(y, x, w, span, degree = degree, parametric = parametric, : all data on boundary of neighborhood. make span bigger
```

```
## Warning in simpleLoess(y, x, w, span, degree = degree, parametric = parametric, : pseudoinverse used at -0.005
```

```
## Warning in simpleLoess(y, x, w, span, degree = degree, parametric = parametric, : neighborhood radius 0.005
```

```
## Warning in simpleLoess(y, x, w, span, degree = degree, parametric = parametric, : reciprocal condition number 1
```

```
## Warning in simpleLoess(y, x, w, span, degree = degree, parametric = parametric, : There are other near singularities as well. 1.01
```

```
## Warning in simpleLoess(y, x, w, span, degree = degree, parametric = parametric, : zero-width neighborhood. make span bigger
```

```
## Warning: Computation failed in `stat_smooth()`:
## NA/NaN/Inf in foreign function call (arg 5)
```

```
## Warning in simpleLoess(y, x, w, span, degree = degree, parametric = parametric, : at -0.005
```

```
## Warning in simpleLoess(y, x, w, span, degree = degree, parametric = parametric, : radius 2.5e-05
```

```
## Warning in simpleLoess(y, x, w, span, degree = degree, parametric = parametric, : all data on boundary of neighborhood. make span bigger
```

```
## Warning in simpleLoess(y, x, w, span, degree = degree, parametric = parametric, : pseudoinverse used at -0.005
```

```
## Warning in simpleLoess(y, x, w, span, degree = degree, parametric = parametric, : neighborhood radius 0.005
```

```
## Warning in simpleLoess(y, x, w, span, degree = degree, parametric = parametric, : reciprocal condition number 1
```

```
## Warning in simpleLoess(y, x, w, span, degree = degree, parametric = parametric, : There are other near singularities as well. 1.01
```

```
## Warning in simpleLoess(y, x, w, span, degree = degree, parametric = parametric, : zero-width neighborhood. make span bigger
```

```
## Warning: Computation failed in `stat_smooth()`:
## NA/NaN/Inf in foreign function call (arg 5)
```

```
## Warning in simpleLoess(y, x, w, span, degree = degree, parametric = parametric, : pseudoinverse used at -0.005
```

```
## Warning in simpleLoess(y, x, w, span, degree = degree, parametric = parametric, : neighborhood radius 1.005
```

```
## Warning in simpleLoess(y, x, w, span, degree = degree, parametric = parametric, : reciprocal condition number 0
```

```
## Warning in simpleLoess(y, x, w, span, degree = degree, parametric = parametric, : There are other near singularities as well. 1.01
```

```
## Warning in predLoess(object$y, object$x, newx = if (is.null(newdata)) object$x else if (is.data.frame(newdata)) as.matrix(model.frame(delete.response(terms(object)), : pseudoinverse used at -0.005
```

```
## Warning in predLoess(object$y, object$x, newx = if (is.null(newdata)) object$x else if (is.data.frame(newdata)) as.matrix(model.frame(delete.response(terms(object)), : neighborhood radius 1.005
```

```
## Warning in predLoess(object$y, object$x, newx = if (is.null(newdata)) object$x else if (is.data.frame(newdata)) as.matrix(model.frame(delete.response(terms(object)), : reciprocal condition number 0
```

```
## Warning in predLoess(object$y, object$x, newx = if (is.null(newdata)) object$x else if (is.data.frame(newdata)) as.matrix(model.frame(delete.response(terms(object)), : There are other near
## singularities as well. 1.01
```

```
## Warning in simpleLoess(y, x, w, span, degree = degree, parametric = parametric, : at -0.005
```

```
## Warning in simpleLoess(y, x, w, span, degree = degree, parametric = parametric, : radius 2.5e-05
```

```
## Warning in simpleLoess(y, x, w, span, degree = degree, parametric = parametric, : all data on boundary of neighborhood. make span bigger
```

```
## Warning in simpleLoess(y, x, w, span, degree = degree, parametric = parametric, : pseudoinverse used at -0.005
```

```
## Warning in simpleLoess(y, x, w, span, degree = degree, parametric = parametric, : neighborhood radius 0.005
```

```
## Warning in simpleLoess(y, x, w, span, degree = degree, parametric = parametric, : reciprocal condition number 1
```

```
## Warning in simpleLoess(y, x, w, span, degree = degree, parametric = parametric, : There are other near singularities as well. 1.01
```

```
## Warning in simpleLoess(y, x, w, span, degree = degree, parametric = parametric, : zero-width neighborhood. make span bigger
```

```
## Warning: Computation failed in `stat_smooth()`:
## NA/NaN/Inf in foreign function call (arg 5)
```

```
## Warning in simpleLoess(y, x, w, span, degree = degree, parametric = parametric, : at -0.005
```

```
## Warning in simpleLoess(y, x, w, span, degree = degree, parametric = parametric, : radius 2.5e-05
```

```
## Warning in simpleLoess(y, x, w, span, degree = degree, parametric = parametric, : all data on boundary of neighborhood. make span bigger
```

```
## Warning in simpleLoess(y, x, w, span, degree = degree, parametric = parametric, : pseudoinverse used at -0.005
```

```
## Warning in simpleLoess(y, x, w, span, degree = degree, parametric = parametric, : neighborhood radius 0.005
```

```
## Warning in simpleLoess(y, x, w, span, degree = degree, parametric = parametric, : reciprocal condition number 1
```

```
## Warning in simpleLoess(y, x, w, span, degree = degree, parametric = parametric, : There are other near singularities as well. 1.01
```

```
## Warning in simpleLoess(y, x, w, span, degree = degree, parametric = parametric, : zero-width neighborhood. make span bigger
```

```
## Warning: Computation failed in `stat_smooth()`:
## NA/NaN/Inf in foreign function call (arg 5)
```

```
## Warning in simpleLoess(y, x, w, span, degree = degree, parametric = parametric, : at -0.005
```

```
## Warning in simpleLoess(y, x, w, span, degree = degree, parametric = parametric, : radius 2.5e-05
```

```
## Warning in simpleLoess(y, x, w, span, degree = degree, parametric = parametric, : all data on boundary of neighborhood. make span bigger
```

```
## Warning in simpleLoess(y, x, w, span, degree = degree, parametric = parametric, : pseudoinverse used at -0.005
```

```
## Warning in simpleLoess(y, x, w, span, degree = degree, parametric = parametric, : neighborhood radius 0.005
```

```
## Warning in simpleLoess(y, x, w, span, degree = degree, parametric = parametric, : reciprocal condition number 1
```

```
## Warning in simpleLoess(y, x, w, span, degree = degree, parametric = parametric, : There are other near singularities as well. 1.01
```

```
## Warning in simpleLoess(y, x, w, span, degree = degree, parametric = parametric, : zero-width neighborhood. make span bigger
```

```
## Warning: Computation failed in `stat_smooth()`:
## NA/NaN/Inf in foreign function call (arg 5)
```

```
## Warning in simpleLoess(y, x, w, span, degree = degree, parametric = parametric, : pseudoinverse used at -0.005
```

```
## Warning in simpleLoess(y, x, w, span, degree = degree, parametric = parametric, : neighborhood radius 1.005
```

```
## Warning in simpleLoess(y, x, w, span, degree = degree, parametric = parametric, : reciprocal condition number 0
```

```
## Warning in simpleLoess(y, x, w, span, degree = degree, parametric = parametric, : There are other near singularities as well. 1.01
```

```
## Warning in predLoess(object$y, object$x, newx = if (is.null(newdata)) object$x else if (is.data.frame(newdata)) as.matrix(model.frame(delete.response(terms(object)), : pseudoinverse used at -0.005
```

```
## Warning in predLoess(object$y, object$x, newx = if (is.null(newdata)) object$x else if (is.data.frame(newdata)) as.matrix(model.frame(delete.response(terms(object)), : neighborhood radius 1.005
```

```
## Warning in predLoess(object$y, object$x, newx = if (is.null(newdata)) object$x else if (is.data.frame(newdata)) as.matrix(model.frame(delete.response(terms(object)), : reciprocal condition number 0
```

```
## Warning in predLoess(object$y, object$x, newx = if (is.null(newdata)) object$x else if (is.data.frame(newdata)) as.matrix(model.frame(delete.response(terms(object)), : There are other near
## singularities as well. 1.01
```

```
## Warning in simpleLoess(y, x, w, span, degree = degree, parametric = parametric, : at -0.005
```

```
## Warning in simpleLoess(y, x, w, span, degree = degree, parametric = parametric, : radius 2.5e-05
```

```
## Warning in simpleLoess(y, x, w, span, degree = degree, parametric = parametric, : all data on boundary of neighborhood. make span bigger
```

```
## Warning in simpleLoess(y, x, w, span, degree = degree, parametric = parametric, : pseudoinverse used at -0.005
```

```
## Warning in simpleLoess(y, x, w, span, degree = degree, parametric = parametric, : neighborhood radius 0.005
```

```
## Warning in simpleLoess(y, x, w, span, degree = degree, parametric = parametric, : reciprocal condition number 1
```

```
## Warning in simpleLoess(y, x, w, span, degree = degree, parametric = parametric, : There are other near singularities as well. 1.01
```

```
## Warning in simpleLoess(y, x, w, span, degree = degree, parametric = parametric, : zero-width neighborhood. make span bigger
```

```
## Warning: Computation failed in `stat_smooth()`:
## NA/NaN/Inf in foreign function call (arg 5)
```

```
## Warning in simpleLoess(y, x, w, span, degree = degree, parametric = parametric, : at -0.005
```

```
## Warning in simpleLoess(y, x, w, span, degree = degree, parametric = parametric, : radius 2.5e-05
```

```
## Warning in simpleLoess(y, x, w, span, degree = degree, parametric = parametric, : all data on boundary of neighborhood. make span bigger
```

```
## Warning in simpleLoess(y, x, w, span, degree = degree, parametric = parametric, : pseudoinverse used at -0.005
```

```
## Warning in simpleLoess(y, x, w, span, degree = degree, parametric = parametric, : neighborhood radius 0.005
```

```
## Warning in simpleLoess(y, x, w, span, degree = degree, parametric = parametric, : reciprocal condition number 1
```

```
## Warning in simpleLoess(y, x, w, span, degree = degree, parametric = parametric, : There are other near singularities as well. 1.01
```

```
## Warning in simpleLoess(y, x, w, span, degree = degree, parametric = parametric, : zero-width neighborhood. make span bigger
```

```
## Warning: Computation failed in `stat_smooth()`:
## NA/NaN/Inf in foreign function call (arg 5)
```

```
## Warning in simpleLoess(y, x, w, span, degree = degree, parametric = parametric, : pseudoinverse used at -0.005
```

```
## Warning in simpleLoess(y, x, w, span, degree = degree, parametric = parametric, : neighborhood radius 1.005
```

```
## Warning in simpleLoess(y, x, w, span, degree = degree, parametric = parametric, : reciprocal condition number 0
```

```
## Warning in simpleLoess(y, x, w, span, degree = degree, parametric = parametric, : There are other near singularities as well. 1.01
```

```
## Warning in predLoess(object$y, object$x, newx = if (is.null(newdata)) object$x else if (is.data.frame(newdata)) as.matrix(model.frame(delete.response(terms(object)), : pseudoinverse used at -0.005
```

```
## Warning in predLoess(object$y, object$x, newx = if (is.null(newdata)) object$x else if (is.data.frame(newdata)) as.matrix(model.frame(delete.response(terms(object)), : neighborhood radius 1.005
```

```
## Warning in predLoess(object$y, object$x, newx = if (is.null(newdata)) object$x else if (is.data.frame(newdata)) as.matrix(model.frame(delete.response(terms(object)), : reciprocal condition number 0
```

```
## Warning in predLoess(object$y, object$x, newx = if (is.null(newdata)) object$x else if (is.data.frame(newdata)) as.matrix(model.frame(delete.response(terms(object)), : There are other near
## singularities as well. 1.01
```

```
## Warning in simpleLoess(y, x, w, span, degree = degree, parametric = parametric, : pseudoinverse used at -0.005
```

```
## Warning in simpleLoess(y, x, w, span, degree = degree, parametric = parametric, : neighborhood radius 1.005
```

```
## Warning in simpleLoess(y, x, w, span, degree = degree, parametric = parametric, : reciprocal condition number 0
```

```
## Warning in simpleLoess(y, x, w, span, degree = degree, parametric = parametric, : There are other near singularities as well. 1.01
```

```
## Warning in predLoess(object$y, object$x, newx = if (is.null(newdata)) object$x else if (is.data.frame(newdata)) as.matrix(model.frame(delete.response(terms(object)), : pseudoinverse used at -0.005
```

```
## Warning in predLoess(object$y, object$x, newx = if (is.null(newdata)) object$x else if (is.data.frame(newdata)) as.matrix(model.frame(delete.response(terms(object)), : neighborhood radius 1.005
```

```
## Warning in predLoess(object$y, object$x, newx = if (is.null(newdata)) object$x else if (is.data.frame(newdata)) as.matrix(model.frame(delete.response(terms(object)), : reciprocal condition number 0
```

```
## Warning in predLoess(object$y, object$x, newx = if (is.null(newdata)) object$x else if (is.data.frame(newdata)) as.matrix(model.frame(delete.response(terms(object)), : There are other near
## singularities as well. 1.01
```

```
## Warning in simpleLoess(y, x, w, span, degree = degree, parametric = parametric, : at -0.005
```

```
## Warning in simpleLoess(y, x, w, span, degree = degree, parametric = parametric, : radius 2.5e-05
```

```
## Warning in simpleLoess(y, x, w, span, degree = degree, parametric = parametric, : all data on boundary of neighborhood. make span bigger
```

```
## Warning in simpleLoess(y, x, w, span, degree = degree, parametric = parametric, : pseudoinverse used at -0.005
```

```
## Warning in simpleLoess(y, x, w, span, degree = degree, parametric = parametric, : neighborhood radius 0.005
```

```
## Warning in simpleLoess(y, x, w, span, degree = degree, parametric = parametric, : reciprocal condition number 1
```

```
## Warning in simpleLoess(y, x, w, span, degree = degree, parametric = parametric, : There are other near singularities as well. 1.01
```

```
## Warning in simpleLoess(y, x, w, span, degree = degree, parametric = parametric, : zero-width neighborhood. make span bigger
```

```
## Warning: Computation failed in `stat_smooth()`:
## NA/NaN/Inf in foreign function call (arg 5)
```

```
## Warning in simpleLoess(y, x, w, span, degree = degree, parametric = parametric, : at -0.005
```

```
## Warning in simpleLoess(y, x, w, span, degree = degree, parametric = parametric, : radius 2.5e-05
```

```
## Warning in simpleLoess(y, x, w, span, degree = degree, parametric = parametric, : all data on boundary of neighborhood. make span bigger
```

```
## Warning in simpleLoess(y, x, w, span, degree = degree, parametric = parametric, : pseudoinverse used at -0.005
```

```
## Warning in simpleLoess(y, x, w, span, degree = degree, parametric = parametric, : neighborhood radius 0.005
```

```
## Warning in simpleLoess(y, x, w, span, degree = degree, parametric = parametric, : reciprocal condition number 1
```

```
## Warning in simpleLoess(y, x, w, span, degree = degree, parametric = parametric, : There are other near singularities as well. 1.01
```

```
## Warning in simpleLoess(y, x, w, span, degree = degree, parametric = parametric, : zero-width neighborhood. make span bigger
```

```
## Warning: Computation failed in `stat_smooth()`:
## NA/NaN/Inf in foreign function call (arg 5)
```

```
## Warning in simpleLoess(y, x, w, span, degree = degree, parametric = parametric, : at -0.005
```

```
## Warning in simpleLoess(y, x, w, span, degree = degree, parametric = parametric, : radius 2.5e-05
```

```
## Warning in simpleLoess(y, x, w, span, degree = degree, parametric = parametric, : all data on boundary of neighborhood. make span bigger
```

```
## Warning in simpleLoess(y, x, w, span, degree = degree, parametric = parametric, : pseudoinverse used at -0.005
```

```
## Warning in simpleLoess(y, x, w, span, degree = degree, parametric = parametric, : neighborhood radius 0.005
```

```
## Warning in simpleLoess(y, x, w, span, degree = degree, parametric = parametric, : reciprocal condition number 1
```

```
## Warning in simpleLoess(y, x, w, span, degree = degree, parametric = parametric, : There are other near singularities as well. 1.01
```

```
## Warning in simpleLoess(y, x, w, span, degree = degree, parametric = parametric, : zero-width neighborhood. make span bigger
```

```
## Warning: Computation failed in `stat_smooth()`:
## NA/NaN/Inf in foreign function call (arg 5)
```

```
## Warning in simpleLoess(y, x, w, span, degree = degree, parametric = parametric, : at -0.005
```

```
## Warning in simpleLoess(y, x, w, span, degree = degree, parametric = parametric, : radius 2.5e-05
```

```
## Warning in simpleLoess(y, x, w, span, degree = degree, parametric = parametric, : all data on boundary of neighborhood. make span bigger
```

```
## Warning in simpleLoess(y, x, w, span, degree = degree, parametric = parametric, : pseudoinverse used at -0.005
```

```
## Warning in simpleLoess(y, x, w, span, degree = degree, parametric = parametric, : neighborhood radius 0.005
```

```
## Warning in simpleLoess(y, x, w, span, degree = degree, parametric = parametric, : reciprocal condition number 1
```

```
## Warning in simpleLoess(y, x, w, span, degree = degree, parametric = parametric, : There are other near singularities as well. 1.01
```

```
## Warning in simpleLoess(y, x, w, span, degree = degree, parametric = parametric, : zero-width neighborhood. make span bigger
```

```
## Warning: Computation failed in `stat_smooth()`:
## NA/NaN/Inf in foreign function call (arg 5)
```

```
## Warning in simpleLoess(y, x, w, span, degree = degree, parametric = parametric, : at -0.005
```

```
## Warning in simpleLoess(y, x, w, span, degree = degree, parametric = parametric, : radius 2.5e-05
```

```
## Warning in simpleLoess(y, x, w, span, degree = degree, parametric = parametric, : all data on boundary of neighborhood. make span bigger
```

```
## Warning in simpleLoess(y, x, w, span, degree = degree, parametric = parametric, : pseudoinverse used at -0.005
```

```
## Warning in simpleLoess(y, x, w, span, degree = degree, parametric = parametric, : neighborhood radius 0.005
```

```
## Warning in simpleLoess(y, x, w, span, degree = degree, parametric = parametric, : reciprocal condition number 1
```

```
## Warning in simpleLoess(y, x, w, span, degree = degree, parametric = parametric, : There are other near singularities as well. 1.01
```

```
## Warning in simpleLoess(y, x, w, span, degree = degree, parametric = parametric, : zero-width neighborhood. make span bigger
```

```
## Warning: Computation failed in `stat_smooth()`:
## NA/NaN/Inf in foreign function call (arg 5)
```

```
## Warning in simpleLoess(y, x, w, span, degree = degree, parametric = parametric, : at -0.005
```

```
## Warning in simpleLoess(y, x, w, span, degree = degree, parametric = parametric, : radius 2.5e-05
```

```
## Warning in simpleLoess(y, x, w, span, degree = degree, parametric = parametric, : all data on boundary of neighborhood. make span bigger
```

```
## Warning in simpleLoess(y, x, w, span, degree = degree, parametric = parametric, : pseudoinverse used at -0.005
```

```
## Warning in simpleLoess(y, x, w, span, degree = degree, parametric = parametric, : neighborhood radius 0.005
```

```
## Warning in simpleLoess(y, x, w, span, degree = degree, parametric = parametric, : reciprocal condition number 1
```

```
## Warning in simpleLoess(y, x, w, span, degree = degree, parametric = parametric, : There are other near singularities as well. 1.01
```

```
## Warning in simpleLoess(y, x, w, span, degree = degree, parametric = parametric, : zero-width neighborhood. make span bigger
```

```
## Warning: Computation failed in `stat_smooth()`:
## NA/NaN/Inf in foreign function call (arg 5)
```

```
## Warning in simpleLoess(y, x, w, span, degree = degree, parametric = parametric, : at -0.005
```

```
## Warning in simpleLoess(y, x, w, span, degree = degree, parametric = parametric, : radius 2.5e-05
```

```
## Warning in simpleLoess(y, x, w, span, degree = degree, parametric = parametric, : all data on boundary of neighborhood. make span bigger
```

```
## Warning in simpleLoess(y, x, w, span, degree = degree, parametric = parametric, : pseudoinverse used at -0.005
```

```
## Warning in simpleLoess(y, x, w, span, degree = degree, parametric = parametric, : neighborhood radius 0.005
```

```
## Warning in simpleLoess(y, x, w, span, degree = degree, parametric = parametric, : reciprocal condition number 1
```

```
## Warning in simpleLoess(y, x, w, span, degree = degree, parametric = parametric, : There are other near singularities as well. 1.01
```

```
## Warning in simpleLoess(y, x, w, span, degree = degree, parametric = parametric, : zero-width neighborhood. make span bigger
```

```
## Warning: Computation failed in `stat_smooth()`:
## NA/NaN/Inf in foreign function call (arg 5)
```

```
## Warning in simpleLoess(y, x, w, span, degree = degree, parametric = parametric, : at -0.005
```

```
## Warning in simpleLoess(y, x, w, span, degree = degree, parametric = parametric, : radius 2.5e-05
```

```
## Warning in simpleLoess(y, x, w, span, degree = degree, parametric = parametric, : all data on boundary of neighborhood. make span bigger
```

```
## Warning in simpleLoess(y, x, w, span, degree = degree, parametric = parametric, : pseudoinverse used at -0.005
```

```
## Warning in simpleLoess(y, x, w, span, degree = degree, parametric = parametric, : neighborhood radius 0.005
```

```
## Warning in simpleLoess(y, x, w, span, degree = degree, parametric = parametric, : reciprocal condition number 1
```

```
## Warning in simpleLoess(y, x, w, span, degree = degree, parametric = parametric, : There are other near singularities as well. 1.01
```

```
## Warning in simpleLoess(y, x, w, span, degree = degree, parametric = parametric, : zero-width neighborhood. make span bigger
```

```
## Warning: Computation failed in `stat_smooth()`:
## NA/NaN/Inf in foreign function call (arg 5)
```

```
## Warning in simpleLoess(y, x, w, span, degree = degree, parametric = parametric, : at -0.005
```

```
## Warning in simpleLoess(y, x, w, span, degree = degree, parametric = parametric, : radius 2.5e-05
```

```
## Warning in simpleLoess(y, x, w, span, degree = degree, parametric = parametric, : all data on boundary of neighborhood. make span bigger
```

```
## Warning in simpleLoess(y, x, w, span, degree = degree, parametric = parametric, : pseudoinverse used at -0.005
```

```
## Warning in simpleLoess(y, x, w, span, degree = degree, parametric = parametric, : neighborhood radius 0.005
```

```
## Warning in simpleLoess(y, x, w, span, degree = degree, parametric = parametric, : reciprocal condition number 1
```

```
## Warning in simpleLoess(y, x, w, span, degree = degree, parametric = parametric, : There are other near singularities as well. 1.01
```

```
## Warning in simpleLoess(y, x, w, span, degree = degree, parametric = parametric, : zero-width neighborhood. make span bigger
```

```
## Warning: Computation failed in `stat_smooth()`:
## NA/NaN/Inf in foreign function call (arg 5)
```

```
## Warning in simpleLoess(y, x, w, span, degree = degree, parametric = parametric, : at -0.005
```

```
## Warning in simpleLoess(y, x, w, span, degree = degree, parametric = parametric, : radius 2.5e-05
```

```
## Warning in simpleLoess(y, x, w, span, degree = degree, parametric = parametric, : all data on boundary of neighborhood. make span bigger
```

```
## Warning in simpleLoess(y, x, w, span, degree = degree, parametric = parametric, : pseudoinverse used at -0.005
```

```
## Warning in simpleLoess(y, x, w, span, degree = degree, parametric = parametric, : neighborhood radius 0.005
```

```
## Warning in simpleLoess(y, x, w, span, degree = degree, parametric = parametric, : reciprocal condition number 1
```

```
## Warning in simpleLoess(y, x, w, span, degree = degree, parametric = parametric, : There are other near singularities as well. 1.01
```

```
## Warning in simpleLoess(y, x, w, span, degree = degree, parametric = parametric, : zero-width neighborhood. make span bigger
```

```
## Warning: Computation failed in `stat_smooth()`:
## NA/NaN/Inf in foreign function call (arg 5)
```

```
## Warning in simpleLoess(y, x, w, span, degree = degree, parametric = parametric, : pseudoinverse used at -0.005
```

```
## Warning in simpleLoess(y, x, w, span, degree = degree, parametric = parametric, : neighborhood radius 1.005
```

```
## Warning in simpleLoess(y, x, w, span, degree = degree, parametric = parametric, : reciprocal condition number 0
```

```
## Warning in simpleLoess(y, x, w, span, degree = degree, parametric = parametric, : There are other near singularities as well. 1.01
```

```
## Warning in predLoess(object$y, object$x, newx = if (is.null(newdata)) object$x else if (is.data.frame(newdata)) as.matrix(model.frame(delete.response(terms(object)), : pseudoinverse used at -0.005
```

```
## Warning in predLoess(object$y, object$x, newx = if (is.null(newdata)) object$x else if (is.data.frame(newdata)) as.matrix(model.frame(delete.response(terms(object)), : neighborhood radius 1.005
```

```
## Warning in predLoess(object$y, object$x, newx = if (is.null(newdata)) object$x else if (is.data.frame(newdata)) as.matrix(model.frame(delete.response(terms(object)), : reciprocal condition number 0
```

```
## Warning in predLoess(object$y, object$x, newx = if (is.null(newdata)) object$x else if (is.data.frame(newdata)) as.matrix(model.frame(delete.response(terms(object)), : There are other near
## singularities as well. 1.01
```

```
## Warning in simpleLoess(y, x, w, span, degree = degree, parametric = parametric, : at -0.005
```

```
## Warning in simpleLoess(y, x, w, span, degree = degree, parametric = parametric, : radius 2.5e-05
```

```
## Warning in simpleLoess(y, x, w, span, degree = degree, parametric = parametric, : all data on boundary of neighborhood. make span bigger
```

```
## Warning in simpleLoess(y, x, w, span, degree = degree, parametric = parametric, : pseudoinverse used at -0.005
```

```
## Warning in simpleLoess(y, x, w, span, degree = degree, parametric = parametric, : neighborhood radius 0.005
```

```
## Warning in simpleLoess(y, x, w, span, degree = degree, parametric = parametric, : reciprocal condition number 1
```

```
## Warning in simpleLoess(y, x, w, span, degree = degree, parametric = parametric, : There are other near singularities as well. 1.01
```

```
## Warning in simpleLoess(y, x, w, span, degree = degree, parametric = parametric, : zero-width neighborhood. make span bigger
```

```
## Warning: Computation failed in `stat_smooth()`:
## NA/NaN/Inf in foreign function call (arg 5)
```

```
## Warning in simpleLoess(y, x, w, span, degree = degree, parametric = parametric, : at -0.005
```

```
## Warning in simpleLoess(y, x, w, span, degree = degree, parametric = parametric, : radius 2.5e-05
```

```
## Warning in simpleLoess(y, x, w, span, degree = degree, parametric = parametric, : all data on boundary of neighborhood. make span bigger
```

```
## Warning in simpleLoess(y, x, w, span, degree = degree, parametric = parametric, : pseudoinverse used at -0.005
```

```
## Warning in simpleLoess(y, x, w, span, degree = degree, parametric = parametric, : neighborhood radius 0.005
```

```
## Warning in simpleLoess(y, x, w, span, degree = degree, parametric = parametric, : reciprocal condition number 1
```

```
## Warning in simpleLoess(y, x, w, span, degree = degree, parametric = parametric, : There are other near singularities as well. 1.01
```

```
## Warning in simpleLoess(y, x, w, span, degree = degree, parametric = parametric, : zero-width neighborhood. make span bigger
```

```
## Warning: Computation failed in `stat_smooth()`:
## NA/NaN/Inf in foreign function call (arg 5)
```

```
## Warning in simpleLoess(y, x, w, span, degree = degree, parametric = parametric, : at -0.005
```

```
## Warning in simpleLoess(y, x, w, span, degree = degree, parametric = parametric, : radius 2.5e-05
```

```
## Warning in simpleLoess(y, x, w, span, degree = degree, parametric = parametric, : all data on boundary of neighborhood. make span bigger
```

```
## Warning in simpleLoess(y, x, w, span, degree = degree, parametric = parametric, : pseudoinverse used at -0.005
```

```
## Warning in simpleLoess(y, x, w, span, degree = degree, parametric = parametric, : neighborhood radius 0.005
```

```
## Warning in simpleLoess(y, x, w, span, degree = degree, parametric = parametric, : reciprocal condition number 1
```

```
## Warning in simpleLoess(y, x, w, span, degree = degree, parametric = parametric, : There are other near singularities as well. 1.01
```

```
## Warning in simpleLoess(y, x, w, span, degree = degree, parametric = parametric, : zero-width neighborhood. make span bigger
```

```
## Warning: Computation failed in `stat_smooth()`:
## NA/NaN/Inf in foreign function call (arg 5)
```

```
## Warning in simpleLoess(y, x, w, span, degree = degree, parametric = parametric, : at -0.005
```

```
## Warning in simpleLoess(y, x, w, span, degree = degree, parametric = parametric, : radius 2.5e-05
```

```
## Warning in simpleLoess(y, x, w, span, degree = degree, parametric = parametric, : all data on boundary of neighborhood. make span bigger
```

```
## Warning in simpleLoess(y, x, w, span, degree = degree, parametric = parametric, : pseudoinverse used at -0.005
```

```
## Warning in simpleLoess(y, x, w, span, degree = degree, parametric = parametric, : neighborhood radius 0.005
```

```
## Warning in simpleLoess(y, x, w, span, degree = degree, parametric = parametric, : reciprocal condition number 1
```

```
## Warning in simpleLoess(y, x, w, span, degree = degree, parametric = parametric, : There are other near singularities as well. 1.01
```

```
## Warning in simpleLoess(y, x, w, span, degree = degree, parametric = parametric, : zero-width neighborhood. make span bigger
```

```
## Warning: Computation failed in `stat_smooth()`:
## NA/NaN/Inf in foreign function call (arg 5)
```

```
## Warning in simpleLoess(y, x, w, span, degree = degree, parametric = parametric, : pseudoinverse used at -0.005
```

```
## Warning in simpleLoess(y, x, w, span, degree = degree, parametric = parametric, : neighborhood radius 1.005
```

```
## Warning in simpleLoess(y, x, w, span, degree = degree, parametric = parametric, : reciprocal condition number 0
```

```
## Warning in simpleLoess(y, x, w, span, degree = degree, parametric = parametric, : There are other near singularities as well. 1.01
```

```
## Warning in predLoess(object$y, object$x, newx = if (is.null(newdata)) object$x else if (is.data.frame(newdata)) as.matrix(model.frame(delete.response(terms(object)), : pseudoinverse used at -0.005
```

```
## Warning in predLoess(object$y, object$x, newx = if (is.null(newdata)) object$x else if (is.data.frame(newdata)) as.matrix(model.frame(delete.response(terms(object)), : neighborhood radius 1.005
```

```
## Warning in predLoess(object$y, object$x, newx = if (is.null(newdata)) object$x else if (is.data.frame(newdata)) as.matrix(model.frame(delete.response(terms(object)), : reciprocal condition number 0
```

```
## Warning in predLoess(object$y, object$x, newx = if (is.null(newdata)) object$x else if (is.data.frame(newdata)) as.matrix(model.frame(delete.response(terms(object)), : There are other near
## singularities as well. 1.01
```

```
## Warning in simpleLoess(y, x, w, span, degree = degree, parametric = parametric, : at -0.005
```

```
## Warning in simpleLoess(y, x, w, span, degree = degree, parametric = parametric, : radius 2.5e-05
```

```
## Warning in simpleLoess(y, x, w, span, degree = degree, parametric = parametric, : all data on boundary of neighborhood. make span bigger
```

```
## Warning in simpleLoess(y, x, w, span, degree = degree, parametric = parametric, : pseudoinverse used at -0.005
```

```
## Warning in simpleLoess(y, x, w, span, degree = degree, parametric = parametric, : neighborhood radius 0.005
```

```
## Warning in simpleLoess(y, x, w, span, degree = degree, parametric = parametric, : reciprocal condition number 1
```

```
## Warning in simpleLoess(y, x, w, span, degree = degree, parametric = parametric, : There are other near singularities as well. 1.01
```

```
## Warning in simpleLoess(y, x, w, span, degree = degree, parametric = parametric, : zero-width neighborhood. make span bigger
```

```
## Warning: Computation failed in `stat_smooth()`:
## NA/NaN/Inf in foreign function call (arg 5)
```

```
## Warning in simpleLoess(y, x, w, span, degree = degree, parametric = parametric, : at -0.005
```

```
## Warning in simpleLoess(y, x, w, span, degree = degree, parametric = parametric, : radius 2.5e-05
```

```
## Warning in simpleLoess(y, x, w, span, degree = degree, parametric = parametric, : all data on boundary of neighborhood. make span bigger
```

```
## Warning in simpleLoess(y, x, w, span, degree = degree, parametric = parametric, : pseudoinverse used at -0.005
```

```
## Warning in simpleLoess(y, x, w, span, degree = degree, parametric = parametric, : neighborhood radius 0.005
```

```
## Warning in simpleLoess(y, x, w, span, degree = degree, parametric = parametric, : reciprocal condition number 1
```

```
## Warning in simpleLoess(y, x, w, span, degree = degree, parametric = parametric, : There are other near singularities as well. 1.01
```

```
## Warning in simpleLoess(y, x, w, span, degree = degree, parametric = parametric, : zero-width neighborhood. make span bigger
```

```
## Warning: Computation failed in `stat_smooth()`:
## NA/NaN/Inf in foreign function call (arg 5)
```

```
## Warning in simpleLoess(y, x, w, span, degree = degree, parametric = parametric, : at -0.005
```

```
## Warning in simpleLoess(y, x, w, span, degree = degree, parametric = parametric, : radius 2.5e-05
```

```
## Warning in simpleLoess(y, x, w, span, degree = degree, parametric = parametric, : all data on boundary of neighborhood. make span bigger
```

```
## Warning in simpleLoess(y, x, w, span, degree = degree, parametric = parametric, : pseudoinverse used at -0.005
```

```
## Warning in simpleLoess(y, x, w, span, degree = degree, parametric = parametric, : neighborhood radius 0.005
```

```
## Warning in simpleLoess(y, x, w, span, degree = degree, parametric = parametric, : reciprocal condition number 1
```

```
## Warning in simpleLoess(y, x, w, span, degree = degree, parametric = parametric, : There are other near singularities as well. 1.01
```

```
## Warning in simpleLoess(y, x, w, span, degree = degree, parametric = parametric, : zero-width neighborhood. make span bigger
```

```
## Warning: Computation failed in `stat_smooth()`:
## NA/NaN/Inf in foreign function call (arg 5)
```

```
## Warning in simpleLoess(y, x, w, span, degree = degree, parametric = parametric, : at -0.005
```

```
## Warning in simpleLoess(y, x, w, span, degree = degree, parametric = parametric, : radius 2.5e-05
```

```
## Warning in simpleLoess(y, x, w, span, degree = degree, parametric = parametric, : all data on boundary of neighborhood. make span bigger
```

```
## Warning in simpleLoess(y, x, w, span, degree = degree, parametric = parametric, : pseudoinverse used at -0.005
```

```
## Warning in simpleLoess(y, x, w, span, degree = degree, parametric = parametric, : neighborhood radius 0.005
```

```
## Warning in simpleLoess(y, x, w, span, degree = degree, parametric = parametric, : reciprocal condition number 1
```

```
## Warning in simpleLoess(y, x, w, span, degree = degree, parametric = parametric, : There are other near singularities as well. 1.01
```

```
## Warning in simpleLoess(y, x, w, span, degree = degree, parametric = parametric, : zero-width neighborhood. make span bigger
```

```
## Warning: Computation failed in `stat_smooth()`:
## NA/NaN/Inf in foreign function call (arg 5)
```

```
## Warning in simpleLoess(y, x, w, span, degree = degree, parametric = parametric, : at -0.005
```

```
## Warning in simpleLoess(y, x, w, span, degree = degree, parametric = parametric, : radius 2.5e-05
```

```
## Warning in simpleLoess(y, x, w, span, degree = degree, parametric = parametric, : all data on boundary of neighborhood. make span bigger
```

```
## Warning in simpleLoess(y, x, w, span, degree = degree, parametric = parametric, : pseudoinverse used at -0.005
```

```
## Warning in simpleLoess(y, x, w, span, degree = degree, parametric = parametric, : neighborhood radius 0.005
```

```
## Warning in simpleLoess(y, x, w, span, degree = degree, parametric = parametric, : reciprocal condition number 1
```

```
## Warning in simpleLoess(y, x, w, span, degree = degree, parametric = parametric, : There are other near singularities as well. 1.01
```

```
## Warning in simpleLoess(y, x, w, span, degree = degree, parametric = parametric, : zero-width neighborhood. make span bigger
```

```
## Warning: Computation failed in `stat_smooth()`:
## NA/NaN/Inf in foreign function call (arg 5)
```

```
## Warning in simpleLoess(y, x, w, span, degree = degree, parametric = parametric, : at -0.005
```

```
## Warning in simpleLoess(y, x, w, span, degree = degree, parametric = parametric, : radius 2.5e-05
```

```
## Warning in simpleLoess(y, x, w, span, degree = degree, parametric = parametric, : all data on boundary of neighborhood. make span bigger
```

```
## Warning in simpleLoess(y, x, w, span, degree = degree, parametric = parametric, : pseudoinverse used at -0.005
```

```
## Warning in simpleLoess(y, x, w, span, degree = degree, parametric = parametric, : neighborhood radius 0.005
```

```
## Warning in simpleLoess(y, x, w, span, degree = degree, parametric = parametric, : reciprocal condition number 1
```

```
## Warning in simpleLoess(y, x, w, span, degree = degree, parametric = parametric, : There are other near singularities as well. 1.01
```

```
## Warning in simpleLoess(y, x, w, span, degree = degree, parametric = parametric, : zero-width neighborhood. make span bigger
```

```
## Warning: Computation failed in `stat_smooth()`:
## NA/NaN/Inf in foreign function call (arg 5)
```

```
## Warning in simpleLoess(y, x, w, span, degree = degree, parametric = parametric, : at -0.005
```

```
## Warning in simpleLoess(y, x, w, span, degree = degree, parametric = parametric, : radius 2.5e-05
```

```
## Warning in simpleLoess(y, x, w, span, degree = degree, parametric = parametric, : all data on boundary of neighborhood. make span bigger
```

```
## Warning in simpleLoess(y, x, w, span, degree = degree, parametric = parametric, : pseudoinverse used at -0.005
```

```
## Warning in simpleLoess(y, x, w, span, degree = degree, parametric = parametric, : neighborhood radius 0.005
```

```
## Warning in simpleLoess(y, x, w, span, degree = degree, parametric = parametric, : reciprocal condition number 1
```

```
## Warning in simpleLoess(y, x, w, span, degree = degree, parametric = parametric, : There are other near singularities as well. 1.01
```

```
## Warning in simpleLoess(y, x, w, span, degree = degree, parametric = parametric, : zero-width neighborhood. make span bigger
```

```
## Warning: Computation failed in `stat_smooth()`:
## NA/NaN/Inf in foreign function call (arg 5)
```

```
## Warning in simpleLoess(y, x, w, span, degree = degree, parametric = parametric, : pseudoinverse used at -0.005
```

```
## Warning in simpleLoess(y, x, w, span, degree = degree, parametric = parametric, : neighborhood radius 1.005
```

```
## Warning in simpleLoess(y, x, w, span, degree = degree, parametric = parametric, : reciprocal condition number 0
```

```
## Warning in simpleLoess(y, x, w, span, degree = degree, parametric = parametric, : There are other near singularities as well. 1.01
```

```
## Warning in predLoess(object$y, object$x, newx = if (is.null(newdata)) object$x else if (is.data.frame(newdata)) as.matrix(model.frame(delete.response(terms(object)), : pseudoinverse used at -0.005
```

```
## Warning in predLoess(object$y, object$x, newx = if (is.null(newdata)) object$x else if (is.data.frame(newdata)) as.matrix(model.frame(delete.response(terms(object)), : neighborhood radius 1.005
```

```
## Warning in predLoess(object$y, object$x, newx = if (is.null(newdata)) object$x else if (is.data.frame(newdata)) as.matrix(model.frame(delete.response(terms(object)), : reciprocal condition number 0
```

```
## Warning in predLoess(object$y, object$x, newx = if (is.null(newdata)) object$x else if (is.data.frame(newdata)) as.matrix(model.frame(delete.response(terms(object)), : There are other near
## singularities as well. 1.01
```

```
## Warning in simpleLoess(y, x, w, span, degree = degree, parametric = parametric, : at -0.005
```

```
## Warning in simpleLoess(y, x, w, span, degree = degree, parametric = parametric, : radius 2.5e-05
```

```
## Warning in simpleLoess(y, x, w, span, degree = degree, parametric = parametric, : all data on boundary of neighborhood. make span bigger
```

```
## Warning in simpleLoess(y, x, w, span, degree = degree, parametric = parametric, : pseudoinverse used at -0.005
```

```
## Warning in simpleLoess(y, x, w, span, degree = degree, parametric = parametric, : neighborhood radius 0.005
```

```
## Warning in simpleLoess(y, x, w, span, degree = degree, parametric = parametric, : reciprocal condition number 1
```

```
## Warning in simpleLoess(y, x, w, span, degree = degree, parametric = parametric, : There are other near singularities as well. 1.01
```

```
## Warning in simpleLoess(y, x, w, span, degree = degree, parametric = parametric, : zero-width neighborhood. make span bigger
```

```
## Warning: Computation failed in `stat_smooth()`:
## NA/NaN/Inf in foreign function call (arg 5)
```

```
## Warning in simpleLoess(y, x, w, span, degree = degree, parametric = parametric, : at -0.005
```

```
## Warning in simpleLoess(y, x, w, span, degree = degree, parametric = parametric, : radius 2.5e-05
```

```
## Warning in simpleLoess(y, x, w, span, degree = degree, parametric = parametric, : all data on boundary of neighborhood. make span bigger
```

```
## Warning in simpleLoess(y, x, w, span, degree = degree, parametric = parametric, : pseudoinverse used at -0.005
```

```
## Warning in simpleLoess(y, x, w, span, degree = degree, parametric = parametric, : neighborhood radius 0.005
```

```
## Warning in simpleLoess(y, x, w, span, degree = degree, parametric = parametric, : reciprocal condition number 1
```

```
## Warning in simpleLoess(y, x, w, span, degree = degree, parametric = parametric, : There are other near singularities as well. 1.01
```

```
## Warning in simpleLoess(y, x, w, span, degree = degree, parametric = parametric, : zero-width neighborhood. make span bigger
```

```
## Warning: Computation failed in `stat_smooth()`:
## NA/NaN/Inf in foreign function call (arg 5)
```

```
## Warning in simpleLoess(y, x, w, span, degree = degree, parametric = parametric, : at -0.005
```

```
## Warning in simpleLoess(y, x, w, span, degree = degree, parametric = parametric, : radius 2.5e-05
```

```
## Warning in simpleLoess(y, x, w, span, degree = degree, parametric = parametric, : all data on boundary of neighborhood. make span bigger
```

```
## Warning in simpleLoess(y, x, w, span, degree = degree, parametric = parametric, : pseudoinverse used at -0.005
```

```
## Warning in simpleLoess(y, x, w, span, degree = degree, parametric = parametric, : neighborhood radius 0.005
```

```
## Warning in simpleLoess(y, x, w, span, degree = degree, parametric = parametric, : reciprocal condition number 1
```

```
## Warning in simpleLoess(y, x, w, span, degree = degree, parametric = parametric, : There are other near singularities as well. 1.01
```

```
## Warning in simpleLoess(y, x, w, span, degree = degree, parametric = parametric, : zero-width neighborhood. make span bigger
```

```
## Warning: Computation failed in `stat_smooth()`:
## NA/NaN/Inf in foreign function call (arg 5)
```

```
## Warning in simpleLoess(y, x, w, span, degree = degree, parametric = parametric, : at -0.005
```

```
## Warning in simpleLoess(y, x, w, span, degree = degree, parametric = parametric, : radius 2.5e-05
```

```
## Warning in simpleLoess(y, x, w, span, degree = degree, parametric = parametric, : all data on boundary of neighborhood. make span bigger
```

```
## Warning in simpleLoess(y, x, w, span, degree = degree, parametric = parametric, : pseudoinverse used at -0.005
```

```
## Warning in simpleLoess(y, x, w, span, degree = degree, parametric = parametric, : neighborhood radius 0.005
```

```
## Warning in simpleLoess(y, x, w, span, degree = degree, parametric = parametric, : reciprocal condition number 1
```

```
## Warning in simpleLoess(y, x, w, span, degree = degree, parametric = parametric, : There are other near singularities as well. 1.01
```

```
## Warning in simpleLoess(y, x, w, span, degree = degree, parametric = parametric, : zero-width neighborhood. make span bigger
```

```
## Warning: Computation failed in `stat_smooth()`:
## NA/NaN/Inf in foreign function call (arg 5)
```

```
## Warning in simpleLoess(y, x, w, span, degree = degree, parametric = parametric, : pseudoinverse used at -0.005
```

```
## Warning in simpleLoess(y, x, w, span, degree = degree, parametric = parametric, : neighborhood radius 1.005
```

```
## Warning in simpleLoess(y, x, w, span, degree = degree, parametric = parametric, : reciprocal condition number 0
```

```
## Warning in simpleLoess(y, x, w, span, degree = degree, parametric = parametric, : There are other near singularities as well. 1.01
```

```
## Warning in predLoess(object$y, object$x, newx = if (is.null(newdata)) object$x else if (is.data.frame(newdata)) as.matrix(model.frame(delete.response(terms(object)), : pseudoinverse used at -0.005
```

```
## Warning in predLoess(object$y, object$x, newx = if (is.null(newdata)) object$x else if (is.data.frame(newdata)) as.matrix(model.frame(delete.response(terms(object)), : neighborhood radius 1.005
```

```
## Warning in predLoess(object$y, object$x, newx = if (is.null(newdata)) object$x else if (is.data.frame(newdata)) as.matrix(model.frame(delete.response(terms(object)), : reciprocal condition number 0
```

```
## Warning in predLoess(object$y, object$x, newx = if (is.null(newdata)) object$x else if (is.data.frame(newdata)) as.matrix(model.frame(delete.response(terms(object)), : There are other near
## singularities as well. 1.01
```

```
## Warning in simpleLoess(y, x, w, span, degree = degree, parametric = parametric, : at -0.005
```

```
## Warning in simpleLoess(y, x, w, span, degree = degree, parametric = parametric, : radius 2.5e-05
```

```
## Warning in simpleLoess(y, x, w, span, degree = degree, parametric = parametric, : all data on boundary of neighborhood. make span bigger
```

```
## Warning in simpleLoess(y, x, w, span, degree = degree, parametric = parametric, : pseudoinverse used at -0.005
```

```
## Warning in simpleLoess(y, x, w, span, degree = degree, parametric = parametric, : neighborhood radius 0.005
```

```
## Warning in simpleLoess(y, x, w, span, degree = degree, parametric = parametric, : reciprocal condition number 1
```

```
## Warning in simpleLoess(y, x, w, span, degree = degree, parametric = parametric, : There are other near singularities as well. 1.01
```

```
## Warning in simpleLoess(y, x, w, span, degree = degree, parametric = parametric, : zero-width neighborhood. make span bigger
```

```
## Warning: Computation failed in `stat_smooth()`:
## NA/NaN/Inf in foreign function call (arg 5)
```

```
## Warning in simpleLoess(y, x, w, span, degree = degree, parametric = parametric, : at -0.005
```

```
## Warning in simpleLoess(y, x, w, span, degree = degree, parametric = parametric, : radius 2.5e-05
```

```
## Warning in simpleLoess(y, x, w, span, degree = degree, parametric = parametric, : all data on boundary of neighborhood. make span bigger
```

```
## Warning in simpleLoess(y, x, w, span, degree = degree, parametric = parametric, : pseudoinverse used at -0.005
```

```
## Warning in simpleLoess(y, x, w, span, degree = degree, parametric = parametric, : neighborhood radius 0.005
```

```
## Warning in simpleLoess(y, x, w, span, degree = degree, parametric = parametric, : reciprocal condition number 1
```

```
## Warning in simpleLoess(y, x, w, span, degree = degree, parametric = parametric, : There are other near singularities as well. 1.01
```

```
## Warning in simpleLoess(y, x, w, span, degree = degree, parametric = parametric, : zero-width neighborhood. make span bigger
```

```
## Warning: Computation failed in `stat_smooth()`:
## NA/NaN/Inf in foreign function call (arg 5)
```

```
## Warning in simpleLoess(y, x, w, span, degree = degree, parametric = parametric, : at -0.005
```

```
## Warning in simpleLoess(y, x, w, span, degree = degree, parametric = parametric, : radius 2.5e-05
```

```
## Warning in simpleLoess(y, x, w, span, degree = degree, parametric = parametric, : all data on boundary of neighborhood. make span bigger
```

```
## Warning in simpleLoess(y, x, w, span, degree = degree, parametric = parametric, : pseudoinverse used at -0.005
```

```
## Warning in simpleLoess(y, x, w, span, degree = degree, parametric = parametric, : neighborhood radius 0.005
```

```
## Warning in simpleLoess(y, x, w, span, degree = degree, parametric = parametric, : reciprocal condition number 1
```

```
## Warning in simpleLoess(y, x, w, span, degree = degree, parametric = parametric, : There are other near singularities as well. 1.01
```

```
## Warning in simpleLoess(y, x, w, span, degree = degree, parametric = parametric, : zero-width neighborhood. make span bigger
```

```
## Warning: Computation failed in `stat_smooth()`:
## NA/NaN/Inf in foreign function call (arg 5)
```

```
## Warning in simpleLoess(y, x, w, span, degree = degree, parametric = parametric, : at -0.005
```

```
## Warning in simpleLoess(y, x, w, span, degree = degree, parametric = parametric, : radius 2.5e-05
```

```
## Warning in simpleLoess(y, x, w, span, degree = degree, parametric = parametric, : all data on boundary of neighborhood. make span bigger
```

```
## Warning in simpleLoess(y, x, w, span, degree = degree, parametric = parametric, : pseudoinverse used at -0.005
```

```
## Warning in simpleLoess(y, x, w, span, degree = degree, parametric = parametric, : neighborhood radius 0.005
```

```
## Warning in simpleLoess(y, x, w, span, degree = degree, parametric = parametric, : reciprocal condition number 1
```

```
## Warning in simpleLoess(y, x, w, span, degree = degree, parametric = parametric, : There are other near singularities as well. 1.01
```

```
## Warning in simpleLoess(y, x, w, span, degree = degree, parametric = parametric, : zero-width neighborhood. make span bigger
```

```
## Warning: Computation failed in `stat_smooth()`:
## NA/NaN/Inf in foreign function call (arg 5)
```

```
## Warning in simpleLoess(y, x, w, span, degree = degree, parametric = parametric, : at -0.005
```

```
## Warning in simpleLoess(y, x, w, span, degree = degree, parametric = parametric, : radius 2.5e-05
```

```
## Warning in simpleLoess(y, x, w, span, degree = degree, parametric = parametric, : all data on boundary of neighborhood. make span bigger
```

```
## Warning in simpleLoess(y, x, w, span, degree = degree, parametric = parametric, : pseudoinverse used at -0.005
```

```
## Warning in simpleLoess(y, x, w, span, degree = degree, parametric = parametric, : neighborhood radius 0.005
```

```
## Warning in simpleLoess(y, x, w, span, degree = degree, parametric = parametric, : reciprocal condition number 1
```

```
## Warning in simpleLoess(y, x, w, span, degree = degree, parametric = parametric, : There are other near singularities as well. 1.01
```

```
## Warning in simpleLoess(y, x, w, span, degree = degree, parametric = parametric, : zero-width neighborhood. make span bigger
```

```
## Warning: Computation failed in `stat_smooth()`:
## NA/NaN/Inf in foreign function call (arg 5)
```

```
## Warning in simpleLoess(y, x, w, span, degree = degree, parametric = parametric, : at -0.005
```

```
## Warning in simpleLoess(y, x, w, span, degree = degree, parametric = parametric, : radius 2.5e-05
```

```
## Warning in simpleLoess(y, x, w, span, degree = degree, parametric = parametric, : all data on boundary of neighborhood. make span bigger
```

```
## Warning in simpleLoess(y, x, w, span, degree = degree, parametric = parametric, : pseudoinverse used at -0.005
```

```
## Warning in simpleLoess(y, x, w, span, degree = degree, parametric = parametric, : neighborhood radius 0.005
```

```
## Warning in simpleLoess(y, x, w, span, degree = degree, parametric = parametric, : reciprocal condition number 1
```

```
## Warning in simpleLoess(y, x, w, span, degree = degree, parametric = parametric, : There are other near singularities as well. 1.01
```

```
## Warning in simpleLoess(y, x, w, span, degree = degree, parametric = parametric, : zero-width neighborhood. make span bigger
```

```
## Warning: Computation failed in `stat_smooth()`:
## NA/NaN/Inf in foreign function call (arg 5)
```

```
## Warning in simpleLoess(y, x, w, span, degree = degree, parametric = parametric, : at -0.005
```

```
## Warning in simpleLoess(y, x, w, span, degree = degree, parametric = parametric, : radius 2.5e-05
```

```
## Warning in simpleLoess(y, x, w, span, degree = degree, parametric = parametric, : all data on boundary of neighborhood. make span bigger
```

```
## Warning in simpleLoess(y, x, w, span, degree = degree, parametric = parametric, : pseudoinverse used at -0.005
```

```
## Warning in simpleLoess(y, x, w, span, degree = degree, parametric = parametric, : neighborhood radius 0.005
```

```
## Warning in simpleLoess(y, x, w, span, degree = degree, parametric = parametric, : reciprocal condition number 1
```

```
## Warning in simpleLoess(y, x, w, span, degree = degree, parametric = parametric, : There are other near singularities as well. 1.01
```

```
## Warning in simpleLoess(y, x, w, span, degree = degree, parametric = parametric, : zero-width neighborhood. make span bigger
```

```
## Warning: Computation failed in `stat_smooth()`:
## NA/NaN/Inf in foreign function call (arg 5)
```

```
## Warning in simpleLoess(y, x, w, span, degree = degree, parametric = parametric, : at -0.005
```

```
## Warning in simpleLoess(y, x, w, span, degree = degree, parametric = parametric, : radius 2.5e-05
```

```
## Warning in simpleLoess(y, x, w, span, degree = degree, parametric = parametric, : all data on boundary of neighborhood. make span bigger
```

```
## Warning in simpleLoess(y, x, w, span, degree = degree, parametric = parametric, : pseudoinverse used at -0.005
```

```
## Warning in simpleLoess(y, x, w, span, degree = degree, parametric = parametric, : neighborhood radius 0.005
```

```
## Warning in simpleLoess(y, x, w, span, degree = degree, parametric = parametric, : reciprocal condition number 1
```

```
## Warning in simpleLoess(y, x, w, span, degree = degree, parametric = parametric, : There are other near singularities as well. 1.01
```

```
## Warning in simpleLoess(y, x, w, span, degree = degree, parametric = parametric, : zero-width neighborhood. make span bigger
```

```
## Warning: Computation failed in `stat_smooth()`:
## NA/NaN/Inf in foreign function call (arg 5)
```

```
## Warning in simpleLoess(y, x, w, span, degree = degree, parametric = parametric, : at -0.005
```

```
## Warning in simpleLoess(y, x, w, span, degree = degree, parametric = parametric, : radius 2.5e-05
```

```
## Warning in simpleLoess(y, x, w, span, degree = degree, parametric = parametric, : all data on boundary of neighborhood. make span bigger
```

```
## Warning in simpleLoess(y, x, w, span, degree = degree, parametric = parametric, : pseudoinverse used at -0.005
```

```
## Warning in simpleLoess(y, x, w, span, degree = degree, parametric = parametric, : neighborhood radius 0.005
```

```
## Warning in simpleLoess(y, x, w, span, degree = degree, parametric = parametric, : reciprocal condition number 1
```

```
## Warning in simpleLoess(y, x, w, span, degree = degree, parametric = parametric, : There are other near singularities as well. 1.01
```

```
## Warning in simpleLoess(y, x, w, span, degree = degree, parametric = parametric, : zero-width neighborhood. make span bigger
```

```
## Warning: Computation failed in `stat_smooth()`:
## NA/NaN/Inf in foreign function call (arg 5)
```

```
## Warning in simpleLoess(y, x, w, span, degree = degree, parametric = parametric, : at -0.005
```

```
## Warning in simpleLoess(y, x, w, span, degree = degree, parametric = parametric, : radius 2.5e-05
```

```
## Warning in simpleLoess(y, x, w, span, degree = degree, parametric = parametric, : all data on boundary of neighborhood. make span bigger
```

```
## Warning in simpleLoess(y, x, w, span, degree = degree, parametric = parametric, : pseudoinverse used at -0.005
```

```
## Warning in simpleLoess(y, x, w, span, degree = degree, parametric = parametric, : neighborhood radius 0.005
```

```
## Warning in simpleLoess(y, x, w, span, degree = degree, parametric = parametric, : reciprocal condition number 1
```

```
## Warning in simpleLoess(y, x, w, span, degree = degree, parametric = parametric, : There are other near singularities as well. 1.01
```

```
## Warning in simpleLoess(y, x, w, span, degree = degree, parametric = parametric, : zero-width neighborhood. make span bigger
```

```
## Warning: Computation failed in `stat_smooth()`:
## NA/NaN/Inf in foreign function call (arg 5)
```

```
## Warning in simpleLoess(y, x, w, span, degree = degree, parametric = parametric, : at -0.005
```

```
## Warning in simpleLoess(y, x, w, span, degree = degree, parametric = parametric, : radius 2.5e-05
```

```
## Warning in simpleLoess(y, x, w, span, degree = degree, parametric = parametric, : all data on boundary of neighborhood. make span bigger
```

```
## Warning in simpleLoess(y, x, w, span, degree = degree, parametric = parametric, : pseudoinverse used at -0.005
```

```
## Warning in simpleLoess(y, x, w, span, degree = degree, parametric = parametric, : neighborhood radius 0.005
```

```
## Warning in simpleLoess(y, x, w, span, degree = degree, parametric = parametric, : reciprocal condition number 1
```

```
## Warning in simpleLoess(y, x, w, span, degree = degree, parametric = parametric, : There are other near singularities as well. 1.01
```

```
## Warning in simpleLoess(y, x, w, span, degree = degree, parametric = parametric, : zero-width neighborhood. make span bigger
```

```
## Warning: Computation failed in `stat_smooth()`:
## NA/NaN/Inf in foreign function call (arg 5)
```

```
## Warning in simpleLoess(y, x, w, span, degree = degree, parametric = parametric, : at -0.005
```

```
## Warning in simpleLoess(y, x, w, span, degree = degree, parametric = parametric, : radius 2.5e-05
```

```
## Warning in simpleLoess(y, x, w, span, degree = degree, parametric = parametric, : all data on boundary of neighborhood. make span bigger
```

```
## Warning in simpleLoess(y, x, w, span, degree = degree, parametric = parametric, : pseudoinverse used at -0.005
```

```
## Warning in simpleLoess(y, x, w, span, degree = degree, parametric = parametric, : neighborhood radius 0.005
```

```
## Warning in simpleLoess(y, x, w, span, degree = degree, parametric = parametric, : reciprocal condition number 1
```

```
## Warning in simpleLoess(y, x, w, span, degree = degree, parametric = parametric, : There are other near singularities as well. 1.01
```

```
## Warning in simpleLoess(y, x, w, span, degree = degree, parametric = parametric, : zero-width neighborhood. make span bigger
```

```
## Warning: Computation failed in `stat_smooth()`:
## NA/NaN/Inf in foreign function call (arg 5)
```

```
## Warning in simpleLoess(y, x, w, span, degree = degree, parametric = parametric, : at -0.005
```

```
## Warning in simpleLoess(y, x, w, span, degree = degree, parametric = parametric, : radius 2.5e-05
```

```
## Warning in simpleLoess(y, x, w, span, degree = degree, parametric = parametric, : all data on boundary of neighborhood. make span bigger
```

```
## Warning in simpleLoess(y, x, w, span, degree = degree, parametric = parametric, : pseudoinverse used at -0.005
```

```
## Warning in simpleLoess(y, x, w, span, degree = degree, parametric = parametric, : neighborhood radius 0.005
```

```
## Warning in simpleLoess(y, x, w, span, degree = degree, parametric = parametric, : reciprocal condition number 1
```

```
## Warning in simpleLoess(y, x, w, span, degree = degree, parametric = parametric, : There are other near singularities as well. 1.01
```

```
## Warning in simpleLoess(y, x, w, span, degree = degree, parametric = parametric, : zero-width neighborhood. make span bigger
```

```
## Warning: Computation failed in `stat_smooth()`:
## NA/NaN/Inf in foreign function call (arg 5)
```

```
## Warning in simpleLoess(y, x, w, span, degree = degree, parametric = parametric, : at -0.005
```

```
## Warning in simpleLoess(y, x, w, span, degree = degree, parametric = parametric, : radius 2.5e-05
```

```
## Warning in simpleLoess(y, x, w, span, degree = degree, parametric = parametric, : all data on boundary of neighborhood. make span bigger
```

```
## Warning in simpleLoess(y, x, w, span, degree = degree, parametric = parametric, : pseudoinverse used at -0.005
```

```
## Warning in simpleLoess(y, x, w, span, degree = degree, parametric = parametric, : neighborhood radius 0.005
```

```
## Warning in simpleLoess(y, x, w, span, degree = degree, parametric = parametric, : reciprocal condition number 1
```

```
## Warning in simpleLoess(y, x, w, span, degree = degree, parametric = parametric, : There are other near singularities as well. 1.01
```

```
## Warning in simpleLoess(y, x, w, span, degree = degree, parametric = parametric, : zero-width neighborhood. make span bigger
```

```
## Warning: Computation failed in `stat_smooth()`:
## NA/NaN/Inf in foreign function call (arg 5)
```

```
## Warning in simpleLoess(y, x, w, span, degree = degree, parametric = parametric, : at -0.005
```

```
## Warning in simpleLoess(y, x, w, span, degree = degree, parametric = parametric, : radius 2.5e-05
```

```
## Warning in simpleLoess(y, x, w, span, degree = degree, parametric = parametric, : all data on boundary of neighborhood. make span bigger
```

```
## Warning in simpleLoess(y, x, w, span, degree = degree, parametric = parametric, : pseudoinverse used at -0.005
```

```
## Warning in simpleLoess(y, x, w, span, degree = degree, parametric = parametric, : neighborhood radius 0.005
```

```
## Warning in simpleLoess(y, x, w, span, degree = degree, parametric = parametric, : reciprocal condition number 1
```

```
## Warning in simpleLoess(y, x, w, span, degree = degree, parametric = parametric, : There are other near singularities as well. 1.01
```

```
## Warning in simpleLoess(y, x, w, span, degree = degree, parametric = parametric, : zero-width neighborhood. make span bigger
```

```
## Warning: Computation failed in `stat_smooth()`:
## NA/NaN/Inf in foreign function call (arg 5)
```

```
## Warning in simpleLoess(y, x, w, span, degree = degree, parametric = parametric, : at -0.005
```

```
## Warning in simpleLoess(y, x, w, span, degree = degree, parametric = parametric, : radius 2.5e-05
```

```
## Warning in simpleLoess(y, x, w, span, degree = degree, parametric = parametric, : all data on boundary of neighborhood. make span bigger
```

```
## Warning in simpleLoess(y, x, w, span, degree = degree, parametric = parametric, : pseudoinverse used at -0.005
```

```
## Warning in simpleLoess(y, x, w, span, degree = degree, parametric = parametric, : neighborhood radius 0.005
```

```
## Warning in simpleLoess(y, x, w, span, degree = degree, parametric = parametric, : reciprocal condition number 1
```

```
## Warning in simpleLoess(y, x, w, span, degree = degree, parametric = parametric, : There are other near singularities as well. 1.01
```

```
## Warning in simpleLoess(y, x, w, span, degree = degree, parametric = parametric, : zero-width neighborhood. make span bigger
```

```
## Warning: Computation failed in `stat_smooth()`:
## NA/NaN/Inf in foreign function call (arg 5)
```

```
## Warning in simpleLoess(y, x, w, span, degree = degree, parametric = parametric, : at -0.005
```

```
## Warning in simpleLoess(y, x, w, span, degree = degree, parametric = parametric, : radius 2.5e-05
```

```
## Warning in simpleLoess(y, x, w, span, degree = degree, parametric = parametric, : all data on boundary of neighborhood. make span bigger
```

```
## Warning in simpleLoess(y, x, w, span, degree = degree, parametric = parametric, : pseudoinverse used at -0.005
```

```
## Warning in simpleLoess(y, x, w, span, degree = degree, parametric = parametric, : neighborhood radius 0.005
```

```
## Warning in simpleLoess(y, x, w, span, degree = degree, parametric = parametric, : reciprocal condition number 1
```

```
## Warning in simpleLoess(y, x, w, span, degree = degree, parametric = parametric, : There are other near singularities as well. 1.01
```

```
## Warning in simpleLoess(y, x, w, span, degree = degree, parametric = parametric, : zero-width neighborhood. make span bigger
```

```
## Warning: Computation failed in `stat_smooth()`:
## NA/NaN/Inf in foreign function call (arg 5)
```

```
## Warning in simpleLoess(y, x, w, span, degree = degree, parametric = parametric, : at -0.005
```

```
## Warning in simpleLoess(y, x, w, span, degree = degree, parametric = parametric, : radius 2.5e-05
```

```
## Warning in simpleLoess(y, x, w, span, degree = degree, parametric = parametric, : all data on boundary of neighborhood. make span bigger
```

```
## Warning in simpleLoess(y, x, w, span, degree = degree, parametric = parametric, : pseudoinverse used at -0.005
```

```
## Warning in simpleLoess(y, x, w, span, degree = degree, parametric = parametric, : neighborhood radius 0.005
```

```
## Warning in simpleLoess(y, x, w, span, degree = degree, parametric = parametric, : reciprocal condition number 1
```

```
## Warning in simpleLoess(y, x, w, span, degree = degree, parametric = parametric, : There are other near singularities as well. 1.01
```

```
## Warning in simpleLoess(y, x, w, span, degree = degree, parametric = parametric, : zero-width neighborhood. make span bigger
```

```
## Warning: Computation failed in `stat_smooth()`:
## NA/NaN/Inf in foreign function call (arg 5)
```

```
## Warning in simpleLoess(y, x, w, span, degree = degree, parametric = parametric, : pseudoinverse used at -0.005
```

```
## Warning in simpleLoess(y, x, w, span, degree = degree, parametric = parametric, : neighborhood radius 1.005
```

```
## Warning in simpleLoess(y, x, w, span, degree = degree, parametric = parametric, : reciprocal condition number 0
```

```
## Warning in simpleLoess(y, x, w, span, degree = degree, parametric = parametric, : There are other near singularities as well. 1.01
```

```
## Warning in predLoess(object$y, object$x, newx = if (is.null(newdata)) object$x else if (is.data.frame(newdata)) as.matrix(model.frame(delete.response(terms(object)), : pseudoinverse used at -0.005
```

```
## Warning in predLoess(object$y, object$x, newx = if (is.null(newdata)) object$x else if (is.data.frame(newdata)) as.matrix(model.frame(delete.response(terms(object)), : neighborhood radius 1.005
```

```
## Warning in predLoess(object$y, object$x, newx = if (is.null(newdata)) object$x else if (is.data.frame(newdata)) as.matrix(model.frame(delete.response(terms(object)), : reciprocal condition number 0
```

```
## Warning in predLoess(object$y, object$x, newx = if (is.null(newdata)) object$x else if (is.data.frame(newdata)) as.matrix(model.frame(delete.response(terms(object)), : There are other near
## singularities as well. 1.01
```

```
## Warning in simpleLoess(y, x, w, span, degree = degree, parametric = parametric, : at -0.005
```

```
## Warning in simpleLoess(y, x, w, span, degree = degree, parametric = parametric, : radius 2.5e-05
```

```
## Warning in simpleLoess(y, x, w, span, degree = degree, parametric = parametric, : all data on boundary of neighborhood. make span bigger
```

```
## Warning in simpleLoess(y, x, w, span, degree = degree, parametric = parametric, : pseudoinverse used at -0.005
```

```
## Warning in simpleLoess(y, x, w, span, degree = degree, parametric = parametric, : neighborhood radius 0.005
```

```
## Warning in simpleLoess(y, x, w, span, degree = degree, parametric = parametric, : reciprocal condition number 1
```

```
## Warning in simpleLoess(y, x, w, span, degree = degree, parametric = parametric, : There are other near singularities as well. 1.01
```

```
## Warning in simpleLoess(y, x, w, span, degree = degree, parametric = parametric, : zero-width neighborhood. make span bigger
```

```
## Warning: Computation failed in `stat_smooth()`:
## NA/NaN/Inf in foreign function call (arg 5)
```

```
## Warning in simpleLoess(y, x, w, span, degree = degree, parametric = parametric, : at -0.005
```

```
## Warning in simpleLoess(y, x, w, span, degree = degree, parametric = parametric, : radius 2.5e-05
```

```
## Warning in simpleLoess(y, x, w, span, degree = degree, parametric = parametric, : all data on boundary of neighborhood. make span bigger
```

```
## Warning in simpleLoess(y, x, w, span, degree = degree, parametric = parametric, : pseudoinverse used at -0.005
```

```
## Warning in simpleLoess(y, x, w, span, degree = degree, parametric = parametric, : neighborhood radius 0.005
```

```
## Warning in simpleLoess(y, x, w, span, degree = degree, parametric = parametric, : reciprocal condition number 1
```

```
## Warning in simpleLoess(y, x, w, span, degree = degree, parametric = parametric, : There are other near singularities as well. 1.01
```

```
## Warning in simpleLoess(y, x, w, span, degree = degree, parametric = parametric, : zero-width neighborhood. make span bigger
```

```
## Warning: Computation failed in `stat_smooth()`:
## NA/NaN/Inf in foreign function call (arg 5)
```

```
## Warning in simpleLoess(y, x, w, span, degree = degree, parametric = parametric, : at -0.005
```

```
## Warning in simpleLoess(y, x, w, span, degree = degree, parametric = parametric, : radius 2.5e-05
```

```
## Warning in simpleLoess(y, x, w, span, degree = degree, parametric = parametric, : all data on boundary of neighborhood. make span bigger
```

```
## Warning in simpleLoess(y, x, w, span, degree = degree, parametric = parametric, : pseudoinverse used at -0.005
```

```
## Warning in simpleLoess(y, x, w, span, degree = degree, parametric = parametric, : neighborhood radius 0.005
```

```
## Warning in simpleLoess(y, x, w, span, degree = degree, parametric = parametric, : reciprocal condition number 1
```

```
## Warning in simpleLoess(y, x, w, span, degree = degree, parametric = parametric, : There are other near singularities as well. 1.01
```

```
## Warning in simpleLoess(y, x, w, span, degree = degree, parametric = parametric, : zero-width neighborhood. make span bigger
```

```
## Warning: Computation failed in `stat_smooth()`:
## NA/NaN/Inf in foreign function call (arg 5)
```

```
## Warning in simpleLoess(y, x, w, span, degree = degree, parametric = parametric, : at -0.005
```

```
## Warning in simpleLoess(y, x, w, span, degree = degree, parametric = parametric, : radius 2.5e-05
```

```
## Warning in simpleLoess(y, x, w, span, degree = degree, parametric = parametric, : all data on boundary of neighborhood. make span bigger
```

```
## Warning in simpleLoess(y, x, w, span, degree = degree, parametric = parametric, : pseudoinverse used at -0.005
```

```
## Warning in simpleLoess(y, x, w, span, degree = degree, parametric = parametric, : neighborhood radius 0.005
```

```
## Warning in simpleLoess(y, x, w, span, degree = degree, parametric = parametric, : reciprocal condition number 1
```

```
## Warning in simpleLoess(y, x, w, span, degree = degree, parametric = parametric, : There are other near singularities as well. 1.01
```

```
## Warning in simpleLoess(y, x, w, span, degree = degree, parametric = parametric, : zero-width neighborhood. make span bigger
```

```
## Warning: Computation failed in `stat_smooth()`:
## NA/NaN/Inf in foreign function call (arg 5)
```

```
## Warning in simpleLoess(y, x, w, span, degree = degree, parametric = parametric, : pseudoinverse used at -0.005
```

```
## Warning in simpleLoess(y, x, w, span, degree = degree, parametric = parametric, : neighborhood radius 1.005
```

```
## Warning in simpleLoess(y, x, w, span, degree = degree, parametric = parametric, : reciprocal condition number 0
```

```
## Warning in simpleLoess(y, x, w, span, degree = degree, parametric = parametric, : There are other near singularities as well. 1.01
```

```
## Warning in predLoess(object$y, object$x, newx = if (is.null(newdata)) object$x else if (is.data.frame(newdata)) as.matrix(model.frame(delete.response(terms(object)), : pseudoinverse used at -0.005
```

```
## Warning in predLoess(object$y, object$x, newx = if (is.null(newdata)) object$x else if (is.data.frame(newdata)) as.matrix(model.frame(delete.response(terms(object)), : neighborhood radius 1.005
```

```
## Warning in predLoess(object$y, object$x, newx = if (is.null(newdata)) object$x else if (is.data.frame(newdata)) as.matrix(model.frame(delete.response(terms(object)), : reciprocal condition number 0
```

```
## Warning in predLoess(object$y, object$x, newx = if (is.null(newdata)) object$x else if (is.data.frame(newdata)) as.matrix(model.frame(delete.response(terms(object)), : There are other near
## singularities as well. 1.01
```

```
## Warning in simpleLoess(y, x, w, span, degree = degree, parametric = parametric, : at -0.005
```

```
## Warning in simpleLoess(y, x, w, span, degree = degree, parametric = parametric, : radius 2.5e-05
```

```
## Warning in simpleLoess(y, x, w, span, degree = degree, parametric = parametric, : all data on boundary of neighborhood. make span bigger
```

```
## Warning in simpleLoess(y, x, w, span, degree = degree, parametric = parametric, : pseudoinverse used at -0.005
```

```
## Warning in simpleLoess(y, x, w, span, degree = degree, parametric = parametric, : neighborhood radius 0.005
```

```
## Warning in simpleLoess(y, x, w, span, degree = degree, parametric = parametric, : reciprocal condition number 1
```

```
## Warning in simpleLoess(y, x, w, span, degree = degree, parametric = parametric, : There are other near singularities as well. 1.01
```

```
## Warning in simpleLoess(y, x, w, span, degree = degree, parametric = parametric, : zero-width neighborhood. make span bigger
```

```
## Warning: Computation failed in `stat_smooth()`:
## NA/NaN/Inf in foreign function call (arg 5)
```

```
## Warning in simpleLoess(y, x, w, span, degree = degree, parametric = parametric, : at -0.005
```

```
## Warning in simpleLoess(y, x, w, span, degree = degree, parametric = parametric, : radius 2.5e-05
```

```
## Warning in simpleLoess(y, x, w, span, degree = degree, parametric = parametric, : all data on boundary of neighborhood. make span bigger
```

```
## Warning in simpleLoess(y, x, w, span, degree = degree, parametric = parametric, : pseudoinverse used at -0.005
```

```
## Warning in simpleLoess(y, x, w, span, degree = degree, parametric = parametric, : neighborhood radius 0.005
```

```
## Warning in simpleLoess(y, x, w, span, degree = degree, parametric = parametric, : reciprocal condition number 1
```

```
## Warning in simpleLoess(y, x, w, span, degree = degree, parametric = parametric, : There are other near singularities as well. 1.01
```

```
## Warning in simpleLoess(y, x, w, span, degree = degree, parametric = parametric, : zero-width neighborhood. make span bigger
```

```
## Warning: Computation failed in `stat_smooth()`:
## NA/NaN/Inf in foreign function call (arg 5)
```

```
## Warning in simpleLoess(y, x, w, span, degree = degree, parametric = parametric, : at -0.005
```

```
## Warning in simpleLoess(y, x, w, span, degree = degree, parametric = parametric, : radius 2.5e-05
```

```
## Warning in simpleLoess(y, x, w, span, degree = degree, parametric = parametric, : all data on boundary of neighborhood. make span bigger
```

```
## Warning in simpleLoess(y, x, w, span, degree = degree, parametric = parametric, : pseudoinverse used at -0.005
```

```
## Warning in simpleLoess(y, x, w, span, degree = degree, parametric = parametric, : neighborhood radius 0.005
```

```
## Warning in simpleLoess(y, x, w, span, degree = degree, parametric = parametric, : reciprocal condition number 1
```

```
## Warning in simpleLoess(y, x, w, span, degree = degree, parametric = parametric, : There are other near singularities as well. 1.01
```

```
## Warning in simpleLoess(y, x, w, span, degree = degree, parametric = parametric, : zero-width neighborhood. make span bigger
```

```
## Warning: Computation failed in `stat_smooth()`:
## NA/NaN/Inf in foreign function call (arg 5)
```

```
## Warning in simpleLoess(y, x, w, span, degree = degree, parametric = parametric, : at -0.005
```

```
## Warning in simpleLoess(y, x, w, span, degree = degree, parametric = parametric, : radius 2.5e-05
```

```
## Warning in simpleLoess(y, x, w, span, degree = degree, parametric = parametric, : all data on boundary of neighborhood. make span bigger
```

```
## Warning in simpleLoess(y, x, w, span, degree = degree, parametric = parametric, : pseudoinverse used at -0.005
```

```
## Warning in simpleLoess(y, x, w, span, degree = degree, parametric = parametric, : neighborhood radius 0.005
```

```
## Warning in simpleLoess(y, x, w, span, degree = degree, parametric = parametric, : reciprocal condition number 1
```

```
## Warning in simpleLoess(y, x, w, span, degree = degree, parametric = parametric, : There are other near singularities as well. 1.01
```

```
## Warning in simpleLoess(y, x, w, span, degree = degree, parametric = parametric, : zero-width neighborhood. make span bigger
```

```
## Warning: Computation failed in `stat_smooth()`:
## NA/NaN/Inf in foreign function call (arg 5)
```

```
## Warning in simpleLoess(y, x, w, span, degree = degree, parametric = parametric, : at -0.005
```

```
## Warning in simpleLoess(y, x, w, span, degree = degree, parametric = parametric, : radius 2.5e-05
```

```
## Warning in simpleLoess(y, x, w, span, degree = degree, parametric = parametric, : all data on boundary of neighborhood. make span bigger
```

```
## Warning in simpleLoess(y, x, w, span, degree = degree, parametric = parametric, : pseudoinverse used at -0.005
```

```
## Warning in simpleLoess(y, x, w, span, degree = degree, parametric = parametric, : neighborhood radius 0.005
```

```
## Warning in simpleLoess(y, x, w, span, degree = degree, parametric = parametric, : reciprocal condition number 1
```

```
## Warning in simpleLoess(y, x, w, span, degree = degree, parametric = parametric, : There are other near singularities as well. 1.01
```

```
## Warning in simpleLoess(y, x, w, span, degree = degree, parametric = parametric, : zero-width neighborhood. make span bigger
```

```
## Warning: Computation failed in `stat_smooth()`:
## NA/NaN/Inf in foreign function call (arg 5)
```

```
## Warning in simpleLoess(y, x, w, span, degree = degree, parametric = parametric, : at -0.005
```

```
## Warning in simpleLoess(y, x, w, span, degree = degree, parametric = parametric, : radius 2.5e-05
```

```
## Warning in simpleLoess(y, x, w, span, degree = degree, parametric = parametric, : all data on boundary of neighborhood. make span bigger
```

```
## Warning in simpleLoess(y, x, w, span, degree = degree, parametric = parametric, : pseudoinverse used at -0.005
```

```
## Warning in simpleLoess(y, x, w, span, degree = degree, parametric = parametric, : neighborhood radius 0.005
```

```
## Warning in simpleLoess(y, x, w, span, degree = degree, parametric = parametric, : reciprocal condition number 1
```

```
## Warning in simpleLoess(y, x, w, span, degree = degree, parametric = parametric, : There are other near singularities as well. 1.01
```

```
## Warning in simpleLoess(y, x, w, span, degree = degree, parametric = parametric, : zero-width neighborhood. make span bigger
```

```
## Warning: Computation failed in `stat_smooth()`:
## NA/NaN/Inf in foreign function call (arg 5)
```

```
## Warning in simpleLoess(y, x, w, span, degree = degree, parametric = parametric, : at -0.005
```

```
## Warning in simpleLoess(y, x, w, span, degree = degree, parametric = parametric, : radius 2.5e-05
```

```
## Warning in simpleLoess(y, x, w, span, degree = degree, parametric = parametric, : all data on boundary of neighborhood. make span bigger
```

```
## Warning in simpleLoess(y, x, w, span, degree = degree, parametric = parametric, : pseudoinverse used at -0.005
```

```
## Warning in simpleLoess(y, x, w, span, degree = degree, parametric = parametric, : neighborhood radius 0.005
```

```
## Warning in simpleLoess(y, x, w, span, degree = degree, parametric = parametric, : reciprocal condition number 1
```

```
## Warning in simpleLoess(y, x, w, span, degree = degree, parametric = parametric, : There are other near singularities as well. 1.01
```

```
## Warning in simpleLoess(y, x, w, span, degree = degree, parametric = parametric, : zero-width neighborhood. make span bigger
```

```
## Warning: Computation failed in `stat_smooth()`:
## NA/NaN/Inf in foreign function call (arg 5)
```

```
## Warning in simpleLoess(y, x, w, span, degree = degree, parametric = parametric, : at -0.005
```

```
## Warning in simpleLoess(y, x, w, span, degree = degree, parametric = parametric, : radius 2.5e-05
```

```
## Warning in simpleLoess(y, x, w, span, degree = degree, parametric = parametric, : all data on boundary of neighborhood. make span bigger
```

```
## Warning in simpleLoess(y, x, w, span, degree = degree, parametric = parametric, : pseudoinverse used at -0.005
```

```
## Warning in simpleLoess(y, x, w, span, degree = degree, parametric = parametric, : neighborhood radius 0.005
```

```
## Warning in simpleLoess(y, x, w, span, degree = degree, parametric = parametric, : reciprocal condition number 1
```

```
## Warning in simpleLoess(y, x, w, span, degree = degree, parametric = parametric, : There are other near singularities as well. 1.01
```

```
## Warning in simpleLoess(y, x, w, span, degree = degree, parametric = parametric, : zero-width neighborhood. make span bigger
```

```
## Warning: Computation failed in `stat_smooth()`:
## NA/NaN/Inf in foreign function call (arg 5)
```

```
## Warning in simpleLoess(y, x, w, span, degree = degree, parametric = parametric, : at -0.005
```

```
## Warning in simpleLoess(y, x, w, span, degree = degree, parametric = parametric, : radius 2.5e-05
```

```
## Warning in simpleLoess(y, x, w, span, degree = degree, parametric = parametric, : all data on boundary of neighborhood. make span bigger
```

```
## Warning in simpleLoess(y, x, w, span, degree = degree, parametric = parametric, : pseudoinverse used at -0.005
```

```
## Warning in simpleLoess(y, x, w, span, degree = degree, parametric = parametric, : neighborhood radius 0.005
```

```
## Warning in simpleLoess(y, x, w, span, degree = degree, parametric = parametric, : reciprocal condition number 1
```

```
## Warning in simpleLoess(y, x, w, span, degree = degree, parametric = parametric, : There are other near singularities as well. 1.01
```

```
## Warning in simpleLoess(y, x, w, span, degree = degree, parametric = parametric, : zero-width neighborhood. make span bigger
```

```
## Warning: Computation failed in `stat_smooth()`:
## NA/NaN/Inf in foreign function call (arg 5)
```

```
## Warning in simpleLoess(y, x, w, span, degree = degree, parametric = parametric, : at -0.005
```

```
## Warning in simpleLoess(y, x, w, span, degree = degree, parametric = parametric, : radius 2.5e-05
```

```
## Warning in simpleLoess(y, x, w, span, degree = degree, parametric = parametric, : all data on boundary of neighborhood. make span bigger
```

```
## Warning in simpleLoess(y, x, w, span, degree = degree, parametric = parametric, : pseudoinverse used at -0.005
```

```
## Warning in simpleLoess(y, x, w, span, degree = degree, parametric = parametric, : neighborhood radius 0.005
```

```
## Warning in simpleLoess(y, x, w, span, degree = degree, parametric = parametric, : reciprocal condition number 1
```

```
## Warning in simpleLoess(y, x, w, span, degree = degree, parametric = parametric, : There are other near singularities as well. 1.01
```

```
## Warning in simpleLoess(y, x, w, span, degree = degree, parametric = parametric, : zero-width neighborhood. make span bigger
```

```
## Warning: Computation failed in `stat_smooth()`:
## NA/NaN/Inf in foreign function call (arg 5)
```

```
## Warning in simpleLoess(y, x, w, span, degree = degree, parametric = parametric, : at -0.005
```

```
## Warning in simpleLoess(y, x, w, span, degree = degree, parametric = parametric, : radius 2.5e-05
```

```
## Warning in simpleLoess(y, x, w, span, degree = degree, parametric = parametric, : all data on boundary of neighborhood. make span bigger
```

```
## Warning in simpleLoess(y, x, w, span, degree = degree, parametric = parametric, : pseudoinverse used at -0.005
```

```
## Warning in simpleLoess(y, x, w, span, degree = degree, parametric = parametric, : neighborhood radius 0.005
```

```
## Warning in simpleLoess(y, x, w, span, degree = degree, parametric = parametric, : reciprocal condition number 1
```

```
## Warning in simpleLoess(y, x, w, span, degree = degree, parametric = parametric, : There are other near singularities as well. 1.01
```

```
## Warning in simpleLoess(y, x, w, span, degree = degree, parametric = parametric, : zero-width neighborhood. make span bigger
```

```
## Warning: Computation failed in `stat_smooth()`:
## NA/NaN/Inf in foreign function call (arg 5)
```

```
## Warning in simpleLoess(y, x, w, span, degree = degree, parametric = parametric, : at -0.005
```

```
## Warning in simpleLoess(y, x, w, span, degree = degree, parametric = parametric, : radius 2.5e-05
```

```
## Warning in simpleLoess(y, x, w, span, degree = degree, parametric = parametric, : all data on boundary of neighborhood. make span bigger
```

```
## Warning in simpleLoess(y, x, w, span, degree = degree, parametric = parametric, : pseudoinverse used at -0.005
```

```
## Warning in simpleLoess(y, x, w, span, degree = degree, parametric = parametric, : neighborhood radius 0.005
```

```
## Warning in simpleLoess(y, x, w, span, degree = degree, parametric = parametric, : reciprocal condition number 1
```

```
## Warning in simpleLoess(y, x, w, span, degree = degree, parametric = parametric, : There are other near singularities as well. 1.01
```

```
## Warning in simpleLoess(y, x, w, span, degree = degree, parametric = parametric, : zero-width neighborhood. make span bigger
```

```
## Warning: Computation failed in `stat_smooth()`:
## NA/NaN/Inf in foreign function call (arg 5)
```

```
## Warning in simpleLoess(y, x, w, span, degree = degree, parametric = parametric, : at -0.005
```

```
## Warning in simpleLoess(y, x, w, span, degree = degree, parametric = parametric, : radius 2.5e-05
```

```
## Warning in simpleLoess(y, x, w, span, degree = degree, parametric = parametric, : all data on boundary of neighborhood. make span bigger
```

```
## Warning in simpleLoess(y, x, w, span, degree = degree, parametric = parametric, : pseudoinverse used at -0.005
```

```
## Warning in simpleLoess(y, x, w, span, degree = degree, parametric = parametric, : neighborhood radius 0.005
```

```
## Warning in simpleLoess(y, x, w, span, degree = degree, parametric = parametric, : reciprocal condition number 1
```

```
## Warning in simpleLoess(y, x, w, span, degree = degree, parametric = parametric, : There are other near singularities as well. 1.01
```

```
## Warning in simpleLoess(y, x, w, span, degree = degree, parametric = parametric, : zero-width neighborhood. make span bigger
```

```
## Warning: Computation failed in `stat_smooth()`:
## NA/NaN/Inf in foreign function call (arg 5)
```

```
## Warning in simpleLoess(y, x, w, span, degree = degree, parametric = parametric, : at -0.005
```

```
## Warning in simpleLoess(y, x, w, span, degree = degree, parametric = parametric, : radius 2.5e-05
```

```
## Warning in simpleLoess(y, x, w, span, degree = degree, parametric = parametric, : all data on boundary of neighborhood. make span bigger
```

```
## Warning in simpleLoess(y, x, w, span, degree = degree, parametric = parametric, : pseudoinverse used at -0.005
```

```
## Warning in simpleLoess(y, x, w, span, degree = degree, parametric = parametric, : neighborhood radius 0.005
```

```
## Warning in simpleLoess(y, x, w, span, degree = degree, parametric = parametric, : reciprocal condition number 1
```

```
## Warning in simpleLoess(y, x, w, span, degree = degree, parametric = parametric, : There are other near singularities as well. 1.01
```

```
## Warning in simpleLoess(y, x, w, span, degree = degree, parametric = parametric, : zero-width neighborhood. make span bigger
```

```
## Warning: Computation failed in `stat_smooth()`:
## NA/NaN/Inf in foreign function call (arg 5)
```

```
## Warning in simpleLoess(y, x, w, span, degree = degree, parametric = parametric, : at -0.005
```

```
## Warning in simpleLoess(y, x, w, span, degree = degree, parametric = parametric, : radius 2.5e-05
```

```
## Warning in simpleLoess(y, x, w, span, degree = degree, parametric = parametric, : all data on boundary of neighborhood. make span bigger
```

```
## Warning in simpleLoess(y, x, w, span, degree = degree, parametric = parametric, : pseudoinverse used at -0.005
```

```
## Warning in simpleLoess(y, x, w, span, degree = degree, parametric = parametric, : neighborhood radius 0.005
```

```
## Warning in simpleLoess(y, x, w, span, degree = degree, parametric = parametric, : reciprocal condition number 1
```

```
## Warning in simpleLoess(y, x, w, span, degree = degree, parametric = parametric, : There are other near singularities as well. 1.01
```

```
## Warning in simpleLoess(y, x, w, span, degree = degree, parametric = parametric, : zero-width neighborhood. make span bigger
```

```
## Warning: Computation failed in `stat_smooth()`:
## NA/NaN/Inf in foreign function call (arg 5)
```

```
## Warning in simpleLoess(y, x, w, span, degree = degree, parametric = parametric, : at -0.005
```

```
## Warning in simpleLoess(y, x, w, span, degree = degree, parametric = parametric, : radius 2.5e-05
```

```
## Warning in simpleLoess(y, x, w, span, degree = degree, parametric = parametric, : all data on boundary of neighborhood. make span bigger
```

```
## Warning in simpleLoess(y, x, w, span, degree = degree, parametric = parametric, : pseudoinverse used at -0.005
```

```
## Warning in simpleLoess(y, x, w, span, degree = degree, parametric = parametric, : neighborhood radius 0.005
```

```
## Warning in simpleLoess(y, x, w, span, degree = degree, parametric = parametric, : reciprocal condition number 1
```

```
## Warning in simpleLoess(y, x, w, span, degree = degree, parametric = parametric, : There are other near singularities as well. 1.01
```

```
## Warning in simpleLoess(y, x, w, span, degree = degree, parametric = parametric, : zero-width neighborhood. make span bigger
```

```
## Warning: Computation failed in `stat_smooth()`:
## NA/NaN/Inf in foreign function call (arg 5)
```

```
## Warning in simpleLoess(y, x, w, span, degree = degree, parametric = parametric, : at -0.005
```

```
## Warning in simpleLoess(y, x, w, span, degree = degree, parametric = parametric, : radius 2.5e-05
```

```
## Warning in simpleLoess(y, x, w, span, degree = degree, parametric = parametric, : all data on boundary of neighborhood. make span bigger
```

```
## Warning in simpleLoess(y, x, w, span, degree = degree, parametric = parametric, : pseudoinverse used at -0.005
```

```
## Warning in simpleLoess(y, x, w, span, degree = degree, parametric = parametric, : neighborhood radius 0.005
```

```
## Warning in simpleLoess(y, x, w, span, degree = degree, parametric = parametric, : reciprocal condition number 1
```

```
## Warning in simpleLoess(y, x, w, span, degree = degree, parametric = parametric, : There are other near singularities as well. 1.01
```

```
## Warning in simpleLoess(y, x, w, span, degree = degree, parametric = parametric, : zero-width neighborhood. make span bigger
```

```
## Warning: Computation failed in `stat_smooth()`:
## NA/NaN/Inf in foreign function call (arg 5)
```

```
## Warning in simpleLoess(y, x, w, span, degree = degree, parametric = parametric, : at -0.005
```

```
## Warning in simpleLoess(y, x, w, span, degree = degree, parametric = parametric, : radius 2.5e-05
```

```
## Warning in simpleLoess(y, x, w, span, degree = degree, parametric = parametric, : all data on boundary of neighborhood. make span bigger
```

```
## Warning in simpleLoess(y, x, w, span, degree = degree, parametric = parametric, : pseudoinverse used at -0.005
```

```
## Warning in simpleLoess(y, x, w, span, degree = degree, parametric = parametric, : neighborhood radius 0.005
```

```
## Warning in simpleLoess(y, x, w, span, degree = degree, parametric = parametric, : reciprocal condition number 1
```

```
## Warning in simpleLoess(y, x, w, span, degree = degree, parametric = parametric, : There are other near singularities as well. 1.01
```

```
## Warning in simpleLoess(y, x, w, span, degree = degree, parametric = parametric, : zero-width neighborhood. make span bigger
```

```
## Warning: Computation failed in `stat_smooth()`:
## NA/NaN/Inf in foreign function call (arg 5)
```

```
## Warning in simpleLoess(y, x, w, span, degree = degree, parametric = parametric, : at -0.005
```

```
## Warning in simpleLoess(y, x, w, span, degree = degree, parametric = parametric, : radius 2.5e-05
```

```
## Warning in simpleLoess(y, x, w, span, degree = degree, parametric = parametric, : all data on boundary of neighborhood. make span bigger
```

```
## Warning in simpleLoess(y, x, w, span, degree = degree, parametric = parametric, : pseudoinverse used at -0.005
```

```
## Warning in simpleLoess(y, x, w, span, degree = degree, parametric = parametric, : neighborhood radius 0.005
```

```
## Warning in simpleLoess(y, x, w, span, degree = degree, parametric = parametric, : reciprocal condition number 1
```

```
## Warning in simpleLoess(y, x, w, span, degree = degree, parametric = parametric, : There are other near singularities as well. 1.01
```

```
## Warning in simpleLoess(y, x, w, span, degree = degree, parametric = parametric, : zero-width neighborhood. make span bigger
```

```
## Warning: Computation failed in `stat_smooth()`:
## NA/NaN/Inf in foreign function call (arg 5)
```

```
## Warning in simpleLoess(y, x, w, span, degree = degree, parametric = parametric, : at -0.005
```

```
## Warning in simpleLoess(y, x, w, span, degree = degree, parametric = parametric, : radius 2.5e-05
```

```
## Warning in simpleLoess(y, x, w, span, degree = degree, parametric = parametric, : all data on boundary of neighborhood. make span bigger
```

```
## Warning in simpleLoess(y, x, w, span, degree = degree, parametric = parametric, : pseudoinverse used at -0.005
```

```
## Warning in simpleLoess(y, x, w, span, degree = degree, parametric = parametric, : neighborhood radius 0.005
```

```
## Warning in simpleLoess(y, x, w, span, degree = degree, parametric = parametric, : reciprocal condition number 1
```

```
## Warning in simpleLoess(y, x, w, span, degree = degree, parametric = parametric, : There are other near singularities as well. 1.01
```

```
## Warning in simpleLoess(y, x, w, span, degree = degree, parametric = parametric, : zero-width neighborhood. make span bigger
```

```
## Warning: Computation failed in `stat_smooth()`:
## NA/NaN/Inf in foreign function call (arg 5)
```

```
## Warning in simpleLoess(y, x, w, span, degree = degree, parametric = parametric, : at -0.005
```

```
## Warning in simpleLoess(y, x, w, span, degree = degree, parametric = parametric, : radius 2.5e-05
```

```
## Warning in simpleLoess(y, x, w, span, degree = degree, parametric = parametric, : all data on boundary of neighborhood. make span bigger
```

```
## Warning in simpleLoess(y, x, w, span, degree = degree, parametric = parametric, : pseudoinverse used at -0.005
```

```
## Warning in simpleLoess(y, x, w, span, degree = degree, parametric = parametric, : neighborhood radius 0.005
```

```
## Warning in simpleLoess(y, x, w, span, degree = degree, parametric = parametric, : reciprocal condition number 1
```

```
## Warning in simpleLoess(y, x, w, span, degree = degree, parametric = parametric, : There are other near singularities as well. 1.01
```

```
## Warning in simpleLoess(y, x, w, span, degree = degree, parametric = parametric, : zero-width neighborhood. make span bigger
```

```
## Warning: Computation failed in `stat_smooth()`:
## NA/NaN/Inf in foreign function call (arg 5)
```

```
## Warning in simpleLoess(y, x, w, span, degree = degree, parametric = parametric, : at -0.005
```

```
## Warning in simpleLoess(y, x, w, span, degree = degree, parametric = parametric, : radius 2.5e-05
```

```
## Warning in simpleLoess(y, x, w, span, degree = degree, parametric = parametric, : all data on boundary of neighborhood. make span bigger
```

```
## Warning in simpleLoess(y, x, w, span, degree = degree, parametric = parametric, : pseudoinverse used at -0.005
```

```
## Warning in simpleLoess(y, x, w, span, degree = degree, parametric = parametric, : neighborhood radius 0.005
```

```
## Warning in simpleLoess(y, x, w, span, degree = degree, parametric = parametric, : reciprocal condition number 1
```

```
## Warning in simpleLoess(y, x, w, span, degree = degree, parametric = parametric, : There are other near singularities as well. 1.01
```

```
## Warning in simpleLoess(y, x, w, span, degree = degree, parametric = parametric, : zero-width neighborhood. make span bigger
```

```
## Warning: Computation failed in `stat_smooth()`:
## NA/NaN/Inf in foreign function call (arg 5)
```

```
## Warning in simpleLoess(y, x, w, span, degree = degree, parametric = parametric, : at -0.005
```

```
## Warning in simpleLoess(y, x, w, span, degree = degree, parametric = parametric, : radius 2.5e-05
```

```
## Warning in simpleLoess(y, x, w, span, degree = degree, parametric = parametric, : all data on boundary of neighborhood. make span bigger
```

```
## Warning in simpleLoess(y, x, w, span, degree = degree, parametric = parametric, : pseudoinverse used at -0.005
```

```
## Warning in simpleLoess(y, x, w, span, degree = degree, parametric = parametric, : neighborhood radius 0.005
```

```
## Warning in simpleLoess(y, x, w, span, degree = degree, parametric = parametric, : reciprocal condition number 1
```

```
## Warning in simpleLoess(y, x, w, span, degree = degree, parametric = parametric, : There are other near singularities as well. 1.01
```

```
## Warning in simpleLoess(y, x, w, span, degree = degree, parametric = parametric, : zero-width neighborhood. make span bigger
```

```
## Warning: Computation failed in `stat_smooth()`:
## NA/NaN/Inf in foreign function call (arg 5)
```

```
## Warning in simpleLoess(y, x, w, span, degree = degree, parametric = parametric, : at -0.005
```

```
## Warning in simpleLoess(y, x, w, span, degree = degree, parametric = parametric, : radius 2.5e-05
```

```
## Warning in simpleLoess(y, x, w, span, degree = degree, parametric = parametric, : all data on boundary of neighborhood. make span bigger
```

```
## Warning in simpleLoess(y, x, w, span, degree = degree, parametric = parametric, : pseudoinverse used at -0.005
```

```
## Warning in simpleLoess(y, x, w, span, degree = degree, parametric = parametric, : neighborhood radius 0.005
```

```
## Warning in simpleLoess(y, x, w, span, degree = degree, parametric = parametric, : reciprocal condition number 1
```

```
## Warning in simpleLoess(y, x, w, span, degree = degree, parametric = parametric, : There are other near singularities as well. 1.01
```

```
## Warning in simpleLoess(y, x, w, span, degree = degree, parametric = parametric, : zero-width neighborhood. make span bigger
```

```
## Warning: Computation failed in `stat_smooth()`:
## NA/NaN/Inf in foreign function call (arg 5)
```

```
## Warning in simpleLoess(y, x, w, span, degree = degree, parametric = parametric, : at -0.005
```

```
## Warning in simpleLoess(y, x, w, span, degree = degree, parametric = parametric, : radius 2.5e-05
```

```
## Warning in simpleLoess(y, x, w, span, degree = degree, parametric = parametric, : all data on boundary of neighborhood. make span bigger
```

```
## Warning in simpleLoess(y, x, w, span, degree = degree, parametric = parametric, : pseudoinverse used at -0.005
```

```
## Warning in simpleLoess(y, x, w, span, degree = degree, parametric = parametric, : neighborhood radius 0.005
```

```
## Warning in simpleLoess(y, x, w, span, degree = degree, parametric = parametric, : reciprocal condition number 1
```

```
## Warning in simpleLoess(y, x, w, span, degree = degree, parametric = parametric, : There are other near singularities as well. 1.01
```

```
## Warning in simpleLoess(y, x, w, span, degree = degree, parametric = parametric, : zero-width neighborhood. make span bigger
```

```
## Warning: Computation failed in `stat_smooth()`:
## NA/NaN/Inf in foreign function call (arg 5)
```

```
## Warning in simpleLoess(y, x, w, span, degree = degree, parametric = parametric, : at -0.005
```

```
## Warning in simpleLoess(y, x, w, span, degree = degree, parametric = parametric, : radius 2.5e-05
```

```
## Warning in simpleLoess(y, x, w, span, degree = degree, parametric = parametric, : all data on boundary of neighborhood. make span bigger
```

```
## Warning in simpleLoess(y, x, w, span, degree = degree, parametric = parametric, : pseudoinverse used at -0.005
```

```
## Warning in simpleLoess(y, x, w, span, degree = degree, parametric = parametric, : neighborhood radius 0.005
```

```
## Warning in simpleLoess(y, x, w, span, degree = degree, parametric = parametric, : reciprocal condition number 1
```

```
## Warning in simpleLoess(y, x, w, span, degree = degree, parametric = parametric, : There are other near singularities as well. 1.01
```

```
## Warning in simpleLoess(y, x, w, span, degree = degree, parametric = parametric, : zero-width neighborhood. make span bigger
```

```
## Warning: Computation failed in `stat_smooth()`:
## NA/NaN/Inf in foreign function call (arg 5)
```

```
## Warning in simpleLoess(y, x, w, span, degree = degree, parametric = parametric, : at -0.005
```

```
## Warning in simpleLoess(y, x, w, span, degree = degree, parametric = parametric, : radius 2.5e-05
```

```
## Warning in simpleLoess(y, x, w, span, degree = degree, parametric = parametric, : all data on boundary of neighborhood. make span bigger
```

```
## Warning in simpleLoess(y, x, w, span, degree = degree, parametric = parametric, : pseudoinverse used at -0.005
```

```
## Warning in simpleLoess(y, x, w, span, degree = degree, parametric = parametric, : neighborhood radius 0.005
```

```
## Warning in simpleLoess(y, x, w, span, degree = degree, parametric = parametric, : reciprocal condition number 1
```

```
## Warning in simpleLoess(y, x, w, span, degree = degree, parametric = parametric, : There are other near singularities as well. 1.01
```

```
## Warning in simpleLoess(y, x, w, span, degree = degree, parametric = parametric, : zero-width neighborhood. make span bigger
```

```
## Warning: Computation failed in `stat_smooth()`:
## NA/NaN/Inf in foreign function call (arg 5)
```

```
## Warning in simpleLoess(y, x, w, span, degree = degree, parametric = parametric, : at -0.005
```

```
## Warning in simpleLoess(y, x, w, span, degree = degree, parametric = parametric, : radius 2.5e-05
```

```
## Warning in simpleLoess(y, x, w, span, degree = degree, parametric = parametric, : all data on boundary of neighborhood. make span bigger
```

```
## Warning in simpleLoess(y, x, w, span, degree = degree, parametric = parametric, : pseudoinverse used at -0.005
```

```
## Warning in simpleLoess(y, x, w, span, degree = degree, parametric = parametric, : neighborhood radius 0.005
```

```
## Warning in simpleLoess(y, x, w, span, degree = degree, parametric = parametric, : reciprocal condition number 1
```

```
## Warning in simpleLoess(y, x, w, span, degree = degree, parametric = parametric, : There are other near singularities as well. 1.01
```

```
## Warning in simpleLoess(y, x, w, span, degree = degree, parametric = parametric, : zero-width neighborhood. make span bigger
```

```
## Warning: Computation failed in `stat_smooth()`:
## NA/NaN/Inf in foreign function call (arg 5)
```

```
## Warning in simpleLoess(y, x, w, span, degree = degree, parametric = parametric, : at -0.005
```

```
## Warning in simpleLoess(y, x, w, span, degree = degree, parametric = parametric, : radius 2.5e-05
```

```
## Warning in simpleLoess(y, x, w, span, degree = degree, parametric = parametric, : all data on boundary of neighborhood. make span bigger
```

```
## Warning in simpleLoess(y, x, w, span, degree = degree, parametric = parametric, : pseudoinverse used at -0.005
```

```
## Warning in simpleLoess(y, x, w, span, degree = degree, parametric = parametric, : neighborhood radius 0.005
```

```
## Warning in simpleLoess(y, x, w, span, degree = degree, parametric = parametric, : reciprocal condition number 1
```

```
## Warning in simpleLoess(y, x, w, span, degree = degree, parametric = parametric, : There are other near singularities as well. 1.01
```

```
## Warning in simpleLoess(y, x, w, span, degree = degree, parametric = parametric, : zero-width neighborhood. make span bigger
```

```
## Warning: Computation failed in `stat_smooth()`:
## NA/NaN/Inf in foreign function call (arg 5)
```

```
## Warning in simpleLoess(y, x, w, span, degree = degree, parametric = parametric, : at -0.005
```

```
## Warning in simpleLoess(y, x, w, span, degree = degree, parametric = parametric, : radius 2.5e-05
```

```
## Warning in simpleLoess(y, x, w, span, degree = degree, parametric = parametric, : all data on boundary of neighborhood. make span bigger
```

```
## Warning in simpleLoess(y, x, w, span, degree = degree, parametric = parametric, : pseudoinverse used at -0.005
```

```
## Warning in simpleLoess(y, x, w, span, degree = degree, parametric = parametric, : neighborhood radius 0.005
```

```
## Warning in simpleLoess(y, x, w, span, degree = degree, parametric = parametric, : reciprocal condition number 1
```

```
## Warning in simpleLoess(y, x, w, span, degree = degree, parametric = parametric, : There are other near singularities as well. 1.01
```

```
## Warning in simpleLoess(y, x, w, span, degree = degree, parametric = parametric, : zero-width neighborhood. make span bigger
```

```
## Warning: Computation failed in `stat_smooth()`:
## NA/NaN/Inf in foreign function call (arg 5)
```

```
## Warning in simpleLoess(y, x, w, span, degree = degree, parametric = parametric, : at -0.005
```

```
## Warning in simpleLoess(y, x, w, span, degree = degree, parametric = parametric, : radius 2.5e-05
```

```
## Warning in simpleLoess(y, x, w, span, degree = degree, parametric = parametric, : all data on boundary of neighborhood. make span bigger
```

```
## Warning in simpleLoess(y, x, w, span, degree = degree, parametric = parametric, : pseudoinverse used at -0.005
```

```
## Warning in simpleLoess(y, x, w, span, degree = degree, parametric = parametric, : neighborhood radius 0.005
```

```
## Warning in simpleLoess(y, x, w, span, degree = degree, parametric = parametric, : reciprocal condition number 1
```

```
## Warning in simpleLoess(y, x, w, span, degree = degree, parametric = parametric, : There are other near singularities as well. 1.01
```

```
## Warning in simpleLoess(y, x, w, span, degree = degree, parametric = parametric, : zero-width neighborhood. make span bigger
```

```
## Warning: Computation failed in `stat_smooth()`:
## NA/NaN/Inf in foreign function call (arg 5)
```

```
## Warning in simpleLoess(y, x, w, span, degree = degree, parametric = parametric, : pseudoinverse used at -0.005
```

```
## Warning in simpleLoess(y, x, w, span, degree = degree, parametric = parametric, : neighborhood radius 1.005
```

```
## Warning in simpleLoess(y, x, w, span, degree = degree, parametric = parametric, : reciprocal condition number 0
```

```
## Warning in simpleLoess(y, x, w, span, degree = degree, parametric = parametric, : There are other near singularities as well. 1.01
```

```
## Warning in predLoess(object$y, object$x, newx = if (is.null(newdata)) object$x else if (is.data.frame(newdata)) as.matrix(model.frame(delete.response(terms(object)), : pseudoinverse used at -0.005
```

```
## Warning in predLoess(object$y, object$x, newx = if (is.null(newdata)) object$x else if (is.data.frame(newdata)) as.matrix(model.frame(delete.response(terms(object)), : neighborhood radius 1.005
```

```
## Warning in predLoess(object$y, object$x, newx = if (is.null(newdata)) object$x else if (is.data.frame(newdata)) as.matrix(model.frame(delete.response(terms(object)), : reciprocal condition number 0
```

```
## Warning in predLoess(object$y, object$x, newx = if (is.null(newdata)) object$x else if (is.data.frame(newdata)) as.matrix(model.frame(delete.response(terms(object)), : There are other near
## singularities as well. 1.01
```

```
## Warning in simpleLoess(y, x, w, span, degree = degree, parametric = parametric, : at -0.005
```

```
## Warning in simpleLoess(y, x, w, span, degree = degree, parametric = parametric, : radius 2.5e-05
```

```
## Warning in simpleLoess(y, x, w, span, degree = degree, parametric = parametric, : all data on boundary of neighborhood. make span bigger
```

```
## Warning in simpleLoess(y, x, w, span, degree = degree, parametric = parametric, : pseudoinverse used at -0.005
```

```
## Warning in simpleLoess(y, x, w, span, degree = degree, parametric = parametric, : neighborhood radius 0.005
```

```
## Warning in simpleLoess(y, x, w, span, degree = degree, parametric = parametric, : reciprocal condition number 1
```

```
## Warning in simpleLoess(y, x, w, span, degree = degree, parametric = parametric, : There are other near singularities as well. 1.01
```

```
## Warning in simpleLoess(y, x, w, span, degree = degree, parametric = parametric, : zero-width neighborhood. make span bigger
```

```
## Warning: Computation failed in `stat_smooth()`:
## NA/NaN/Inf in foreign function call (arg 5)
```

```
## Warning in simpleLoess(y, x, w, span, degree = degree, parametric = parametric, : at -0.005
```

```
## Warning in simpleLoess(y, x, w, span, degree = degree, parametric = parametric, : radius 2.5e-05
```

```
## Warning in simpleLoess(y, x, w, span, degree = degree, parametric = parametric, : all data on boundary of neighborhood. make span bigger
```

```
## Warning in simpleLoess(y, x, w, span, degree = degree, parametric = parametric, : pseudoinverse used at -0.005
```

```
## Warning in simpleLoess(y, x, w, span, degree = degree, parametric = parametric, : neighborhood radius 0.005
```

```
## Warning in simpleLoess(y, x, w, span, degree = degree, parametric = parametric, : reciprocal condition number 1
```

```
## Warning in simpleLoess(y, x, w, span, degree = degree, parametric = parametric, : There are other near singularities as well. 1.01
```

```
## Warning in simpleLoess(y, x, w, span, degree = degree, parametric = parametric, : zero-width neighborhood. make span bigger
```

```
## Warning: Computation failed in `stat_smooth()`:
## NA/NaN/Inf in foreign function call (arg 5)
```

```
## Warning in simpleLoess(y, x, w, span, degree = degree, parametric = parametric, : pseudoinverse used at -0.005
```

```
## Warning in simpleLoess(y, x, w, span, degree = degree, parametric = parametric, : neighborhood radius 1.005
```

```
## Warning in simpleLoess(y, x, w, span, degree = degree, parametric = parametric, : reciprocal condition number 0
```

```
## Warning in simpleLoess(y, x, w, span, degree = degree, parametric = parametric, : There are other near singularities as well. 1.01
```

```
## Warning in predLoess(object$y, object$x, newx = if (is.null(newdata)) object$x else if (is.data.frame(newdata)) as.matrix(model.frame(delete.response(terms(object)), : pseudoinverse used at -0.005
```

```
## Warning in predLoess(object$y, object$x, newx = if (is.null(newdata)) object$x else if (is.data.frame(newdata)) as.matrix(model.frame(delete.response(terms(object)), : neighborhood radius 1.005
```

```
## Warning in predLoess(object$y, object$x, newx = if (is.null(newdata)) object$x else if (is.data.frame(newdata)) as.matrix(model.frame(delete.response(terms(object)), : reciprocal condition number 0
```

```
## Warning in predLoess(object$y, object$x, newx = if (is.null(newdata)) object$x else if (is.data.frame(newdata)) as.matrix(model.frame(delete.response(terms(object)), : There are other near
## singularities as well. 1.01
```

```
## Warning in simpleLoess(y, x, w, span, degree = degree, parametric = parametric, : at -0.005
```

```
## Warning in simpleLoess(y, x, w, span, degree = degree, parametric = parametric, : radius 2.5e-05
```

```
## Warning in simpleLoess(y, x, w, span, degree = degree, parametric = parametric, : all data on boundary of neighborhood. make span bigger
```

```
## Warning in simpleLoess(y, x, w, span, degree = degree, parametric = parametric, : pseudoinverse used at -0.005
```

```
## Warning in simpleLoess(y, x, w, span, degree = degree, parametric = parametric, : neighborhood radius 0.005
```

```
## Warning in simpleLoess(y, x, w, span, degree = degree, parametric = parametric, : reciprocal condition number 1
```

```
## Warning in simpleLoess(y, x, w, span, degree = degree, parametric = parametric, : There are other near singularities as well. 1.01
```

```
## Warning in simpleLoess(y, x, w, span, degree = degree, parametric = parametric, : zero-width neighborhood. make span bigger
```

```
## Warning: Computation failed in `stat_smooth()`:
## NA/NaN/Inf in foreign function call (arg 5)
```

```
## Warning in simpleLoess(y, x, w, span, degree = degree, parametric = parametric, : at -0.005
```

```
## Warning in simpleLoess(y, x, w, span, degree = degree, parametric = parametric, : radius 2.5e-05
```

```
## Warning in simpleLoess(y, x, w, span, degree = degree, parametric = parametric, : all data on boundary of neighborhood. make span bigger
```

```
## Warning in simpleLoess(y, x, w, span, degree = degree, parametric = parametric, : pseudoinverse used at -0.005
```

```
## Warning in simpleLoess(y, x, w, span, degree = degree, parametric = parametric, : neighborhood radius 0.005
```

```
## Warning in simpleLoess(y, x, w, span, degree = degree, parametric = parametric, : reciprocal condition number 1
```

```
## Warning in simpleLoess(y, x, w, span, degree = degree, parametric = parametric, : There are other near singularities as well. 1.01
```

```
## Warning in simpleLoess(y, x, w, span, degree = degree, parametric = parametric, : zero-width neighborhood. make span bigger
```

```
## Warning: Computation failed in `stat_smooth()`:
## NA/NaN/Inf in foreign function call (arg 5)
```

```
## Warning in simpleLoess(y, x, w, span, degree = degree, parametric = parametric, : at -0.005
```

```
## Warning in simpleLoess(y, x, w, span, degree = degree, parametric = parametric, : radius 2.5e-05
```

```
## Warning in simpleLoess(y, x, w, span, degree = degree, parametric = parametric, : all data on boundary of neighborhood. make span bigger
```

```
## Warning in simpleLoess(y, x, w, span, degree = degree, parametric = parametric, : pseudoinverse used at -0.005
```

```
## Warning in simpleLoess(y, x, w, span, degree = degree, parametric = parametric, : neighborhood radius 0.005
```

```
## Warning in simpleLoess(y, x, w, span, degree = degree, parametric = parametric, : reciprocal condition number 1
```

```
## Warning in simpleLoess(y, x, w, span, degree = degree, parametric = parametric, : There are other near singularities as well. 1.01
```

```
## Warning in simpleLoess(y, x, w, span, degree = degree, parametric = parametric, : zero-width neighborhood. make span bigger
```

```
## Warning: Computation failed in `stat_smooth()`:
## NA/NaN/Inf in foreign function call (arg 5)
```

```
## Warning in simpleLoess(y, x, w, span, degree = degree, parametric = parametric, : at -0.005
```

```
## Warning in simpleLoess(y, x, w, span, degree = degree, parametric = parametric, : radius 2.5e-05
```

```
## Warning in simpleLoess(y, x, w, span, degree = degree, parametric = parametric, : all data on boundary of neighborhood. make span bigger
```

```
## Warning in simpleLoess(y, x, w, span, degree = degree, parametric = parametric, : pseudoinverse used at -0.005
```

```
## Warning in simpleLoess(y, x, w, span, degree = degree, parametric = parametric, : neighborhood radius 0.005
```

```
## Warning in simpleLoess(y, x, w, span, degree = degree, parametric = parametric, : reciprocal condition number 1
```

```
## Warning in simpleLoess(y, x, w, span, degree = degree, parametric = parametric, : There are other near singularities as well. 1.01
```

```
## Warning in simpleLoess(y, x, w, span, degree = degree, parametric = parametric, : zero-width neighborhood. make span bigger
```

```
## Warning: Computation failed in `stat_smooth()`:
## NA/NaN/Inf in foreign function call (arg 5)
```

```
## Warning in simpleLoess(y, x, w, span, degree = degree, parametric = parametric, : at -0.005
```

```
## Warning in simpleLoess(y, x, w, span, degree = degree, parametric = parametric, : radius 2.5e-05
```

```
## Warning in simpleLoess(y, x, w, span, degree = degree, parametric = parametric, : all data on boundary of neighborhood. make span bigger
```

```
## Warning in simpleLoess(y, x, w, span, degree = degree, parametric = parametric, : pseudoinverse used at -0.005
```

```
## Warning in simpleLoess(y, x, w, span, degree = degree, parametric = parametric, : neighborhood radius 0.005
```

```
## Warning in simpleLoess(y, x, w, span, degree = degree, parametric = parametric, : reciprocal condition number 1
```

```
## Warning in simpleLoess(y, x, w, span, degree = degree, parametric = parametric, : There are other near singularities as well. 1.01
```

```
## Warning in simpleLoess(y, x, w, span, degree = degree, parametric = parametric, : zero-width neighborhood. make span bigger
```

```
## Warning: Computation failed in `stat_smooth()`:
## NA/NaN/Inf in foreign function call (arg 5)
```

```
## Warning in simpleLoess(y, x, w, span, degree = degree, parametric = parametric, : at -0.005
```

```
## Warning in simpleLoess(y, x, w, span, degree = degree, parametric = parametric, : radius 2.5e-05
```

```
## Warning in simpleLoess(y, x, w, span, degree = degree, parametric = parametric, : all data on boundary of neighborhood. make span bigger
```

```
## Warning in simpleLoess(y, x, w, span, degree = degree, parametric = parametric, : pseudoinverse used at -0.005
```

```
## Warning in simpleLoess(y, x, w, span, degree = degree, parametric = parametric, : neighborhood radius 0.005
```

```
## Warning in simpleLoess(y, x, w, span, degree = degree, parametric = parametric, : reciprocal condition number 1
```

```
## Warning in simpleLoess(y, x, w, span, degree = degree, parametric = parametric, : There are other near singularities as well. 1.01
```

```
## Warning in simpleLoess(y, x, w, span, degree = degree, parametric = parametric, : zero-width neighborhood. make span bigger
```

```
## Warning: Computation failed in `stat_smooth()`:
## NA/NaN/Inf in foreign function call (arg 5)
```

```
## Warning in simpleLoess(y, x, w, span, degree = degree, parametric = parametric, : at -0.005
```

```
## Warning in simpleLoess(y, x, w, span, degree = degree, parametric = parametric, : radius 2.5e-05
```

```
## Warning in simpleLoess(y, x, w, span, degree = degree, parametric = parametric, : all data on boundary of neighborhood. make span bigger
```

```
## Warning in simpleLoess(y, x, w, span, degree = degree, parametric = parametric, : pseudoinverse used at -0.005
```

```
## Warning in simpleLoess(y, x, w, span, degree = degree, parametric = parametric, : neighborhood radius 0.005
```

```
## Warning in simpleLoess(y, x, w, span, degree = degree, parametric = parametric, : reciprocal condition number 1
```

```
## Warning in simpleLoess(y, x, w, span, degree = degree, parametric = parametric, : There are other near singularities as well. 1.01
```

```
## Warning in simpleLoess(y, x, w, span, degree = degree, parametric = parametric, : zero-width neighborhood. make span bigger
```

```
## Warning: Computation failed in `stat_smooth()`:
## NA/NaN/Inf in foreign function call (arg 5)
```

```
## Warning in simpleLoess(y, x, w, span, degree = degree, parametric = parametric, : at -0.005
```

```
## Warning in simpleLoess(y, x, w, span, degree = degree, parametric = parametric, : radius 2.5e-05
```

```
## Warning in simpleLoess(y, x, w, span, degree = degree, parametric = parametric, : all data on boundary of neighborhood. make span bigger
```

```
## Warning in simpleLoess(y, x, w, span, degree = degree, parametric = parametric, : pseudoinverse used at -0.005
```

```
## Warning in simpleLoess(y, x, w, span, degree = degree, parametric = parametric, : neighborhood radius 0.005
```

```
## Warning in simpleLoess(y, x, w, span, degree = degree, parametric = parametric, : reciprocal condition number 1
```

```
## Warning in simpleLoess(y, x, w, span, degree = degree, parametric = parametric, : There are other near singularities as well. 1.01
```

```
## Warning in simpleLoess(y, x, w, span, degree = degree, parametric = parametric, : zero-width neighborhood. make span bigger
```

```
## Warning: Computation failed in `stat_smooth()`:
## NA/NaN/Inf in foreign function call (arg 5)
```

```
## Warning in simpleLoess(y, x, w, span, degree = degree, parametric = parametric, : at -0.005
```

```
## Warning in simpleLoess(y, x, w, span, degree = degree, parametric = parametric, : radius 2.5e-05
```

```
## Warning in simpleLoess(y, x, w, span, degree = degree, parametric = parametric, : all data on boundary of neighborhood. make span bigger
```

```
## Warning in simpleLoess(y, x, w, span, degree = degree, parametric = parametric, : pseudoinverse used at -0.005
```

```
## Warning in simpleLoess(y, x, w, span, degree = degree, parametric = parametric, : neighborhood radius 0.005
```

```
## Warning in simpleLoess(y, x, w, span, degree = degree, parametric = parametric, : reciprocal condition number 1
```

```
## Warning in simpleLoess(y, x, w, span, degree = degree, parametric = parametric, : There are other near singularities as well. 1.01
```

```
## Warning in simpleLoess(y, x, w, span, degree = degree, parametric = parametric, : zero-width neighborhood. make span bigger
```

```
## Warning: Computation failed in `stat_smooth()`:
## NA/NaN/Inf in foreign function call (arg 5)
```

```
## Warning in simpleLoess(y, x, w, span, degree = degree, parametric = parametric, : at -0.005
```

```
## Warning in simpleLoess(y, x, w, span, degree = degree, parametric = parametric, : radius 2.5e-05
```

```
## Warning in simpleLoess(y, x, w, span, degree = degree, parametric = parametric, : all data on boundary of neighborhood. make span bigger
```

```
## Warning in simpleLoess(y, x, w, span, degree = degree, parametric = parametric, : pseudoinverse used at -0.005
```

```
## Warning in simpleLoess(y, x, w, span, degree = degree, parametric = parametric, : neighborhood radius 0.005
```

```
## Warning in simpleLoess(y, x, w, span, degree = degree, parametric = parametric, : reciprocal condition number 1
```

```
## Warning in simpleLoess(y, x, w, span, degree = degree, parametric = parametric, : There are other near singularities as well. 1.01
```

```
## Warning in simpleLoess(y, x, w, span, degree = degree, parametric = parametric, : zero-width neighborhood. make span bigger
```

```
## Warning: Computation failed in `stat_smooth()`:
## NA/NaN/Inf in foreign function call (arg 5)
```

```
## Warning in simpleLoess(y, x, w, span, degree = degree, parametric = parametric, : at -0.005
```

```
## Warning in simpleLoess(y, x, w, span, degree = degree, parametric = parametric, : radius 2.5e-05
```

```
## Warning in simpleLoess(y, x, w, span, degree = degree, parametric = parametric, : all data on boundary of neighborhood. make span bigger
```

```
## Warning in simpleLoess(y, x, w, span, degree = degree, parametric = parametric, : pseudoinverse used at -0.005
```

```
## Warning in simpleLoess(y, x, w, span, degree = degree, parametric = parametric, : neighborhood radius 0.005
```

```
## Warning in simpleLoess(y, x, w, span, degree = degree, parametric = parametric, : reciprocal condition number 1
```

```
## Warning in simpleLoess(y, x, w, span, degree = degree, parametric = parametric, : There are other near singularities as well. 1.01
```

```
## Warning in simpleLoess(y, x, w, span, degree = degree, parametric = parametric, : zero-width neighborhood. make span bigger
```

```
## Warning: Computation failed in `stat_smooth()`:
## NA/NaN/Inf in foreign function call (arg 5)
```

```
## Warning in simpleLoess(y, x, w, span, degree = degree, parametric = parametric, : pseudoinverse used at -0.005
```

```
## Warning in simpleLoess(y, x, w, span, degree = degree, parametric = parametric, : neighborhood radius 1.005
```

```
## Warning in simpleLoess(y, x, w, span, degree = degree, parametric = parametric, : reciprocal condition number 0
```

```
## Warning in simpleLoess(y, x, w, span, degree = degree, parametric = parametric, : There are other near singularities as well. 1.01
```

```
## Warning in predLoess(object$y, object$x, newx = if (is.null(newdata)) object$x else if (is.data.frame(newdata)) as.matrix(model.frame(delete.response(terms(object)), : pseudoinverse used at -0.005
```

```
## Warning in predLoess(object$y, object$x, newx = if (is.null(newdata)) object$x else if (is.data.frame(newdata)) as.matrix(model.frame(delete.response(terms(object)), : neighborhood radius 1.005
```

```
## Warning in predLoess(object$y, object$x, newx = if (is.null(newdata)) object$x else if (is.data.frame(newdata)) as.matrix(model.frame(delete.response(terms(object)), : reciprocal condition number 0
```

```
## Warning in predLoess(object$y, object$x, newx = if (is.null(newdata)) object$x else if (is.data.frame(newdata)) as.matrix(model.frame(delete.response(terms(object)), : There are other near
## singularities as well. 1.01
```

```
## Warning in simpleLoess(y, x, w, span, degree = degree, parametric = parametric, : at -0.005
```

```
## Warning in simpleLoess(y, x, w, span, degree = degree, parametric = parametric, : radius 2.5e-05
```

```
## Warning in simpleLoess(y, x, w, span, degree = degree, parametric = parametric, : all data on boundary of neighborhood. make span bigger
```

```
## Warning in simpleLoess(y, x, w, span, degree = degree, parametric = parametric, : pseudoinverse used at -0.005
```

```
## Warning in simpleLoess(y, x, w, span, degree = degree, parametric = parametric, : neighborhood radius 0.005
```

```
## Warning in simpleLoess(y, x, w, span, degree = degree, parametric = parametric, : reciprocal condition number 1
```

```
## Warning in simpleLoess(y, x, w, span, degree = degree, parametric = parametric, : There are other near singularities as well. 1.01
```

```
## Warning in simpleLoess(y, x, w, span, degree = degree, parametric = parametric, : zero-width neighborhood. make span bigger
```

```
## Warning: Computation failed in `stat_smooth()`:
## NA/NaN/Inf in foreign function call (arg 5)
```

```
## Warning in simpleLoess(y, x, w, span, degree = degree, parametric = parametric, : at -0.005
```

```
## Warning in simpleLoess(y, x, w, span, degree = degree, parametric = parametric, : radius 2.5e-05
```

```
## Warning in simpleLoess(y, x, w, span, degree = degree, parametric = parametric, : all data on boundary of neighborhood. make span bigger
```

```
## Warning in simpleLoess(y, x, w, span, degree = degree, parametric = parametric, : pseudoinverse used at -0.005
```

```
## Warning in simpleLoess(y, x, w, span, degree = degree, parametric = parametric, : neighborhood radius 0.005
```

```
## Warning in simpleLoess(y, x, w, span, degree = degree, parametric = parametric, : reciprocal condition number 1
```

```
## Warning in simpleLoess(y, x, w, span, degree = degree, parametric = parametric, : There are other near singularities as well. 1.01
```

```
## Warning in simpleLoess(y, x, w, span, degree = degree, parametric = parametric, : zero-width neighborhood. make span bigger
```

```
## Warning: Computation failed in `stat_smooth()`:
## NA/NaN/Inf in foreign function call (arg 5)
```

```
## Warning in simpleLoess(y, x, w, span, degree = degree, parametric = parametric, : at -0.005
```

```
## Warning in simpleLoess(y, x, w, span, degree = degree, parametric = parametric, : radius 2.5e-05
```

```
## Warning in simpleLoess(y, x, w, span, degree = degree, parametric = parametric, : all data on boundary of neighborhood. make span bigger
```

```
## Warning in simpleLoess(y, x, w, span, degree = degree, parametric = parametric, : pseudoinverse used at -0.005
```

```
## Warning in simpleLoess(y, x, w, span, degree = degree, parametric = parametric, : neighborhood radius 0.005
```

```
## Warning in simpleLoess(y, x, w, span, degree = degree, parametric = parametric, : reciprocal condition number 1
```

```
## Warning in simpleLoess(y, x, w, span, degree = degree, parametric = parametric, : There are other near singularities as well. 1.01
```

```
## Warning in simpleLoess(y, x, w, span, degree = degree, parametric = parametric, : zero-width neighborhood. make span bigger
```

```
## Warning: Computation failed in `stat_smooth()`:
## NA/NaN/Inf in foreign function call (arg 5)
```

```
## Warning in simpleLoess(y, x, w, span, degree = degree, parametric = parametric, : at -0.005
```

```
## Warning in simpleLoess(y, x, w, span, degree = degree, parametric = parametric, : radius 2.5e-05
```

```
## Warning in simpleLoess(y, x, w, span, degree = degree, parametric = parametric, : all data on boundary of neighborhood. make span bigger
```

```
## Warning in simpleLoess(y, x, w, span, degree = degree, parametric = parametric, : pseudoinverse used at -0.005
```

```
## Warning in simpleLoess(y, x, w, span, degree = degree, parametric = parametric, : neighborhood radius 0.005
```

```
## Warning in simpleLoess(y, x, w, span, degree = degree, parametric = parametric, : reciprocal condition number 1
```

```
## Warning in simpleLoess(y, x, w, span, degree = degree, parametric = parametric, : There are other near singularities as well. 1.01
```

```
## Warning in simpleLoess(y, x, w, span, degree = degree, parametric = parametric, : zero-width neighborhood. make span bigger
```

```
## Warning: Computation failed in `stat_smooth()`:
## NA/NaN/Inf in foreign function call (arg 5)
```

```
## Warning in simpleLoess(y, x, w, span, degree = degree, parametric = parametric, : at -0.005
```

```
## Warning in simpleLoess(y, x, w, span, degree = degree, parametric = parametric, : radius 2.5e-05
```

```
## Warning in simpleLoess(y, x, w, span, degree = degree, parametric = parametric, : all data on boundary of neighborhood. make span bigger
```

```
## Warning in simpleLoess(y, x, w, span, degree = degree, parametric = parametric, : pseudoinverse used at -0.005
```

```
## Warning in simpleLoess(y, x, w, span, degree = degree, parametric = parametric, : neighborhood radius 0.005
```

```
## Warning in simpleLoess(y, x, w, span, degree = degree, parametric = parametric, : reciprocal condition number 1
```

```
## Warning in simpleLoess(y, x, w, span, degree = degree, parametric = parametric, : There are other near singularities as well. 1.01
```

```
## Warning in simpleLoess(y, x, w, span, degree = degree, parametric = parametric, : zero-width neighborhood. make span bigger
```

```
## Warning: Computation failed in `stat_smooth()`:
## NA/NaN/Inf in foreign function call (arg 5)
```

```
## Warning in simpleLoess(y, x, w, span, degree = degree, parametric = parametric, : pseudoinverse used at -0.005
```

```
## Warning in simpleLoess(y, x, w, span, degree = degree, parametric = parametric, : neighborhood radius 1.005
```

```
## Warning in simpleLoess(y, x, w, span, degree = degree, parametric = parametric, : reciprocal condition number 0
```

```
## Warning in simpleLoess(y, x, w, span, degree = degree, parametric = parametric, : There are other near singularities as well. 1.01
```

```
## Warning in predLoess(object$y, object$x, newx = if (is.null(newdata)) object$x else if (is.data.frame(newdata)) as.matrix(model.frame(delete.response(terms(object)), : pseudoinverse used at -0.005
```

```
## Warning in predLoess(object$y, object$x, newx = if (is.null(newdata)) object$x else if (is.data.frame(newdata)) as.matrix(model.frame(delete.response(terms(object)), : neighborhood radius 1.005
```

```
## Warning in predLoess(object$y, object$x, newx = if (is.null(newdata)) object$x else if (is.data.frame(newdata)) as.matrix(model.frame(delete.response(terms(object)), : reciprocal condition number 0
```

```
## Warning in predLoess(object$y, object$x, newx = if (is.null(newdata)) object$x else if (is.data.frame(newdata)) as.matrix(model.frame(delete.response(terms(object)), : There are other near
## singularities as well. 1.01
```

```
## Warning in simpleLoess(y, x, w, span, degree = degree, parametric = parametric, : at -0.005
```

```
## Warning in simpleLoess(y, x, w, span, degree = degree, parametric = parametric, : radius 2.5e-05
```

```
## Warning in simpleLoess(y, x, w, span, degree = degree, parametric = parametric, : all data on boundary of neighborhood. make span bigger
```

```
## Warning in simpleLoess(y, x, w, span, degree = degree, parametric = parametric, : pseudoinverse used at -0.005
```

```
## Warning in simpleLoess(y, x, w, span, degree = degree, parametric = parametric, : neighborhood radius 0.005
```

```
## Warning in simpleLoess(y, x, w, span, degree = degree, parametric = parametric, : reciprocal condition number 1
```

```
## Warning in simpleLoess(y, x, w, span, degree = degree, parametric = parametric, : There are other near singularities as well. 1.01
```

```
## Warning in simpleLoess(y, x, w, span, degree = degree, parametric = parametric, : zero-width neighborhood. make span bigger
```

```
## Warning: Computation failed in `stat_smooth()`:
## NA/NaN/Inf in foreign function call (arg 5)
```

```
## Warning in simpleLoess(y, x, w, span, degree = degree, parametric = parametric, : at -0.005
```

```
## Warning in simpleLoess(y, x, w, span, degree = degree, parametric = parametric, : radius 2.5e-05
```

```
## Warning in simpleLoess(y, x, w, span, degree = degree, parametric = parametric, : all data on boundary of neighborhood. make span bigger
```

```
## Warning in simpleLoess(y, x, w, span, degree = degree, parametric = parametric, : pseudoinverse used at -0.005
```

```
## Warning in simpleLoess(y, x, w, span, degree = degree, parametric = parametric, : neighborhood radius 0.005
```

```
## Warning in simpleLoess(y, x, w, span, degree = degree, parametric = parametric, : reciprocal condition number 1
```

```
## Warning in simpleLoess(y, x, w, span, degree = degree, parametric = parametric, : There are other near singularities as well. 1.01
```

```
## Warning in simpleLoess(y, x, w, span, degree = degree, parametric = parametric, : zero-width neighborhood. make span bigger
```

```
## Warning: Computation failed in `stat_smooth()`:
## NA/NaN/Inf in foreign function call (arg 5)
```

```
## Warning in simpleLoess(y, x, w, span, degree = degree, parametric = parametric, : pseudoinverse used at -0.005
```

```
## Warning in simpleLoess(y, x, w, span, degree = degree, parametric = parametric, : neighborhood radius 1.005
```

```
## Warning in simpleLoess(y, x, w, span, degree = degree, parametric = parametric, : reciprocal condition number 0
```

```
## Warning in simpleLoess(y, x, w, span, degree = degree, parametric = parametric, : There are other near singularities as well. 1.01
```

```
## Warning in predLoess(object$y, object$x, newx = if (is.null(newdata)) object$x else if (is.data.frame(newdata)) as.matrix(model.frame(delete.response(terms(object)), : pseudoinverse used at -0.005
```

```
## Warning in predLoess(object$y, object$x, newx = if (is.null(newdata)) object$x else if (is.data.frame(newdata)) as.matrix(model.frame(delete.response(terms(object)), : neighborhood radius 1.005
```

```
## Warning in predLoess(object$y, object$x, newx = if (is.null(newdata)) object$x else if (is.data.frame(newdata)) as.matrix(model.frame(delete.response(terms(object)), : reciprocal condition number 0
```

```
## Warning in predLoess(object$y, object$x, newx = if (is.null(newdata)) object$x else if (is.data.frame(newdata)) as.matrix(model.frame(delete.response(terms(object)), : There are other near
## singularities as well. 1.01
```

```
## Warning in simpleLoess(y, x, w, span, degree = degree, parametric = parametric, : pseudoinverse used at -0.005
```

```
## Warning in simpleLoess(y, x, w, span, degree = degree, parametric = parametric, : neighborhood radius 1.005
```

```
## Warning in simpleLoess(y, x, w, span, degree = degree, parametric = parametric, : reciprocal condition number 0
```

```
## Warning in simpleLoess(y, x, w, span, degree = degree, parametric = parametric, : There are other near singularities as well. 1.01
```

```
## Warning in predLoess(object$y, object$x, newx = if (is.null(newdata)) object$x else if (is.data.frame(newdata)) as.matrix(model.frame(delete.response(terms(object)), : pseudoinverse used at -0.005
```

```
## Warning in predLoess(object$y, object$x, newx = if (is.null(newdata)) object$x else if (is.data.frame(newdata)) as.matrix(model.frame(delete.response(terms(object)), : neighborhood radius 1.005
```

```
## Warning in predLoess(object$y, object$x, newx = if (is.null(newdata)) object$x else if (is.data.frame(newdata)) as.matrix(model.frame(delete.response(terms(object)), : reciprocal condition number 0
```

```
## Warning in predLoess(object$y, object$x, newx = if (is.null(newdata)) object$x else if (is.data.frame(newdata)) as.matrix(model.frame(delete.response(terms(object)), : There are other near
## singularities as well. 1.01
```

```
## Warning in simpleLoess(y, x, w, span, degree = degree, parametric = parametric, : at -0.005
```

```
## Warning in simpleLoess(y, x, w, span, degree = degree, parametric = parametric, : radius 2.5e-05
```

```
## Warning in simpleLoess(y, x, w, span, degree = degree, parametric = parametric, : all data on boundary of neighborhood. make span bigger
```

```
## Warning in simpleLoess(y, x, w, span, degree = degree, parametric = parametric, : pseudoinverse used at -0.005
```

```
## Warning in simpleLoess(y, x, w, span, degree = degree, parametric = parametric, : neighborhood radius 0.005
```

```
## Warning in simpleLoess(y, x, w, span, degree = degree, parametric = parametric, : reciprocal condition number 1
```

```
## Warning in simpleLoess(y, x, w, span, degree = degree, parametric = parametric, : There are other near singularities as well. 1.01
```

```
## Warning in simpleLoess(y, x, w, span, degree = degree, parametric = parametric, : zero-width neighborhood. make span bigger
```

```
## Warning: Computation failed in `stat_smooth()`:
## NA/NaN/Inf in foreign function call (arg 5)
```

```
## Warning in simpleLoess(y, x, w, span, degree = degree, parametric = parametric, : at -0.005
```

```
## Warning in simpleLoess(y, x, w, span, degree = degree, parametric = parametric, : radius 2.5e-05
```

```
## Warning in simpleLoess(y, x, w, span, degree = degree, parametric = parametric, : all data on boundary of neighborhood. make span bigger
```

```
## Warning in simpleLoess(y, x, w, span, degree = degree, parametric = parametric, : pseudoinverse used at -0.005
```

```
## Warning in simpleLoess(y, x, w, span, degree = degree, parametric = parametric, : neighborhood radius 0.005
```

```
## Warning in simpleLoess(y, x, w, span, degree = degree, parametric = parametric, : reciprocal condition number 1
```

```
## Warning in simpleLoess(y, x, w, span, degree = degree, parametric = parametric, : There are other near singularities as well. 1.01
```

```
## Warning in simpleLoess(y, x, w, span, degree = degree, parametric = parametric, : zero-width neighborhood. make span bigger
```

```
## Warning: Computation failed in `stat_smooth()`:
## NA/NaN/Inf in foreign function call (arg 5)
```

```
## Warning in simpleLoess(y, x, w, span, degree = degree, parametric = parametric, : at -0.005
```

```
## Warning in simpleLoess(y, x, w, span, degree = degree, parametric = parametric, : radius 2.5e-05
```

```
## Warning in simpleLoess(y, x, w, span, degree = degree, parametric = parametric, : all data on boundary of neighborhood. make span bigger
```

```
## Warning in simpleLoess(y, x, w, span, degree = degree, parametric = parametric, : pseudoinverse used at -0.005
```

```
## Warning in simpleLoess(y, x, w, span, degree = degree, parametric = parametric, : neighborhood radius 0.005
```

```
## Warning in simpleLoess(y, x, w, span, degree = degree, parametric = parametric, : reciprocal condition number 1
```

```
## Warning in simpleLoess(y, x, w, span, degree = degree, parametric = parametric, : There are other near singularities as well. 1.01
```

```
## Warning in simpleLoess(y, x, w, span, degree = degree, parametric = parametric, : zero-width neighborhood. make span bigger
```

```
## Warning: Computation failed in `stat_smooth()`:
## NA/NaN/Inf in foreign function call (arg 5)
```

```
## Warning in simpleLoess(y, x, w, span, degree = degree, parametric = parametric, : at -0.005
```

```
## Warning in simpleLoess(y, x, w, span, degree = degree, parametric = parametric, : radius 2.5e-05
```

```
## Warning in simpleLoess(y, x, w, span, degree = degree, parametric = parametric, : all data on boundary of neighborhood. make span bigger
```

```
## Warning in simpleLoess(y, x, w, span, degree = degree, parametric = parametric, : pseudoinverse used at -0.005
```

```
## Warning in simpleLoess(y, x, w, span, degree = degree, parametric = parametric, : neighborhood radius 0.005
```

```
## Warning in simpleLoess(y, x, w, span, degree = degree, parametric = parametric, : reciprocal condition number 1
```

```
## Warning in simpleLoess(y, x, w, span, degree = degree, parametric = parametric, : There are other near singularities as well. 1.01
```

```
## Warning in simpleLoess(y, x, w, span, degree = degree, parametric = parametric, : zero-width neighborhood. make span bigger
```

```
## Warning: Computation failed in `stat_smooth()`:
## NA/NaN/Inf in foreign function call (arg 5)
```

```
## Warning in simpleLoess(y, x, w, span, degree = degree, parametric = parametric, : at -0.005
```

```
## Warning in simpleLoess(y, x, w, span, degree = degree, parametric = parametric, : radius 2.5e-05
```

```
## Warning in simpleLoess(y, x, w, span, degree = degree, parametric = parametric, : all data on boundary of neighborhood. make span bigger
```

```
## Warning in simpleLoess(y, x, w, span, degree = degree, parametric = parametric, : pseudoinverse used at -0.005
```

```
## Warning in simpleLoess(y, x, w, span, degree = degree, parametric = parametric, : neighborhood radius 0.005
```

```
## Warning in simpleLoess(y, x, w, span, degree = degree, parametric = parametric, : reciprocal condition number 1
```

```
## Warning in simpleLoess(y, x, w, span, degree = degree, parametric = parametric, : There are other near singularities as well. 1.01
```

```
## Warning in simpleLoess(y, x, w, span, degree = degree, parametric = parametric, : zero-width neighborhood. make span bigger
```

```
## Warning: Computation failed in `stat_smooth()`:
## NA/NaN/Inf in foreign function call (arg 5)
```

```
## Warning in simpleLoess(y, x, w, span, degree = degree, parametric = parametric, : at -0.005
```

```
## Warning in simpleLoess(y, x, w, span, degree = degree, parametric = parametric, : radius 2.5e-05
```

```
## Warning in simpleLoess(y, x, w, span, degree = degree, parametric = parametric, : all data on boundary of neighborhood. make span bigger
```

```
## Warning in simpleLoess(y, x, w, span, degree = degree, parametric = parametric, : pseudoinverse used at -0.005
```

```
## Warning in simpleLoess(y, x, w, span, degree = degree, parametric = parametric, : neighborhood radius 0.005
```

```
## Warning in simpleLoess(y, x, w, span, degree = degree, parametric = parametric, : reciprocal condition number 1
```

```
## Warning in simpleLoess(y, x, w, span, degree = degree, parametric = parametric, : There are other near singularities as well. 1.01
```

```
## Warning in simpleLoess(y, x, w, span, degree = degree, parametric = parametric, : zero-width neighborhood. make span bigger
```

```
## Warning: Computation failed in `stat_smooth()`:
## NA/NaN/Inf in foreign function call (arg 5)
```

```
## Warning in simpleLoess(y, x, w, span, degree = degree, parametric = parametric, : at -0.005
```

```
## Warning in simpleLoess(y, x, w, span, degree = degree, parametric = parametric, : radius 2.5e-05
```

```
## Warning in simpleLoess(y, x, w, span, degree = degree, parametric = parametric, : all data on boundary of neighborhood. make span bigger
```

```
## Warning in simpleLoess(y, x, w, span, degree = degree, parametric = parametric, : pseudoinverse used at -0.005
```

```
## Warning in simpleLoess(y, x, w, span, degree = degree, parametric = parametric, : neighborhood radius 0.005
```

```
## Warning in simpleLoess(y, x, w, span, degree = degree, parametric = parametric, : reciprocal condition number 1
```

```
## Warning in simpleLoess(y, x, w, span, degree = degree, parametric = parametric, : There are other near singularities as well. 1.01
```

```
## Warning in simpleLoess(y, x, w, span, degree = degree, parametric = parametric, : zero-width neighborhood. make span bigger
```

```
## Warning: Computation failed in `stat_smooth()`:
## NA/NaN/Inf in foreign function call (arg 5)
```

```
## Warning in simpleLoess(y, x, w, span, degree = degree, parametric = parametric, : at -0.005
```

```
## Warning in simpleLoess(y, x, w, span, degree = degree, parametric = parametric, : radius 2.5e-05
```

```
## Warning in simpleLoess(y, x, w, span, degree = degree, parametric = parametric, : all data on boundary of neighborhood. make span bigger
```

```
## Warning in simpleLoess(y, x, w, span, degree = degree, parametric = parametric, : pseudoinverse used at -0.005
```

```
## Warning in simpleLoess(y, x, w, span, degree = degree, parametric = parametric, : neighborhood radius 0.005
```

```
## Warning in simpleLoess(y, x, w, span, degree = degree, parametric = parametric, : reciprocal condition number 1
```

```
## Warning in simpleLoess(y, x, w, span, degree = degree, parametric = parametric, : There are other near singularities as well. 1.01
```

```
## Warning in simpleLoess(y, x, w, span, degree = degree, parametric = parametric, : zero-width neighborhood. make span bigger
```

```
## Warning: Computation failed in `stat_smooth()`:
## NA/NaN/Inf in foreign function call (arg 5)
```

```
## Warning in simpleLoess(y, x, w, span, degree = degree, parametric = parametric, : at -0.005
```

```
## Warning in simpleLoess(y, x, w, span, degree = degree, parametric = parametric, : radius 2.5e-05
```

```
## Warning in simpleLoess(y, x, w, span, degree = degree, parametric = parametric, : all data on boundary of neighborhood. make span bigger
```

```
## Warning in simpleLoess(y, x, w, span, degree = degree, parametric = parametric, : pseudoinverse used at -0.005
```

```
## Warning in simpleLoess(y, x, w, span, degree = degree, parametric = parametric, : neighborhood radius 0.005
```

```
## Warning in simpleLoess(y, x, w, span, degree = degree, parametric = parametric, : reciprocal condition number 1
```

```
## Warning in simpleLoess(y, x, w, span, degree = degree, parametric = parametric, : There are other near singularities as well. 1.01
```

```
## Warning in simpleLoess(y, x, w, span, degree = degree, parametric = parametric, : zero-width neighborhood. make span bigger
```

```
## Warning: Computation failed in `stat_smooth()`:
## NA/NaN/Inf in foreign function call (arg 5)
```

```
## Warning in simpleLoess(y, x, w, span, degree = degree, parametric = parametric, : at -0.005
```

```
## Warning in simpleLoess(y, x, w, span, degree = degree, parametric = parametric, : radius 2.5e-05
```

```
## Warning in simpleLoess(y, x, w, span, degree = degree, parametric = parametric, : all data on boundary of neighborhood. make span bigger
```

```
## Warning in simpleLoess(y, x, w, span, degree = degree, parametric = parametric, : pseudoinverse used at -0.005
```

```
## Warning in simpleLoess(y, x, w, span, degree = degree, parametric = parametric, : neighborhood radius 0.005
```

```
## Warning in simpleLoess(y, x, w, span, degree = degree, parametric = parametric, : reciprocal condition number 1
```

```
## Warning in simpleLoess(y, x, w, span, degree = degree, parametric = parametric, : There are other near singularities as well. 1.01
```

```
## Warning in simpleLoess(y, x, w, span, degree = degree, parametric = parametric, : zero-width neighborhood. make span bigger
```

```
## Warning: Computation failed in `stat_smooth()`:
## NA/NaN/Inf in foreign function call (arg 5)
```

```
## Warning in simpleLoess(y, x, w, span, degree = degree, parametric = parametric, : at -0.005
```

```
## Warning in simpleLoess(y, x, w, span, degree = degree, parametric = parametric, : radius 2.5e-05
```

```
## Warning in simpleLoess(y, x, w, span, degree = degree, parametric = parametric, : all data on boundary of neighborhood. make span bigger
```

```
## Warning in simpleLoess(y, x, w, span, degree = degree, parametric = parametric, : pseudoinverse used at -0.005
```

```
## Warning in simpleLoess(y, x, w, span, degree = degree, parametric = parametric, : neighborhood radius 0.005
```

```
## Warning in simpleLoess(y, x, w, span, degree = degree, parametric = parametric, : reciprocal condition number 1
```

```
## Warning in simpleLoess(y, x, w, span, degree = degree, parametric = parametric, : There are other near singularities as well. 1.01
```

```
## Warning in simpleLoess(y, x, w, span, degree = degree, parametric = parametric, : zero-width neighborhood. make span bigger
```

```
## Warning: Computation failed in `stat_smooth()`:
## NA/NaN/Inf in foreign function call (arg 5)
```

```
## Warning in simpleLoess(y, x, w, span, degree = degree, parametric = parametric, : at -0.005
```

```
## Warning in simpleLoess(y, x, w, span, degree = degree, parametric = parametric, : radius 2.5e-05
```

```
## Warning in simpleLoess(y, x, w, span, degree = degree, parametric = parametric, : all data on boundary of neighborhood. make span bigger
```

```
## Warning in simpleLoess(y, x, w, span, degree = degree, parametric = parametric, : pseudoinverse used at -0.005
```

```
## Warning in simpleLoess(y, x, w, span, degree = degree, parametric = parametric, : neighborhood radius 0.005
```

```
## Warning in simpleLoess(y, x, w, span, degree = degree, parametric = parametric, : reciprocal condition number 1
```

```
## Warning in simpleLoess(y, x, w, span, degree = degree, parametric = parametric, : There are other near singularities as well. 1.01
```

```
## Warning in simpleLoess(y, x, w, span, degree = degree, parametric = parametric, : zero-width neighborhood. make span bigger
```

```
## Warning: Computation failed in `stat_smooth()`:
## NA/NaN/Inf in foreign function call (arg 5)
```

```
## Warning in simpleLoess(y, x, w, span, degree = degree, parametric = parametric, : at -0.005
```

```
## Warning in simpleLoess(y, x, w, span, degree = degree, parametric = parametric, : radius 2.5e-05
```

```
## Warning in simpleLoess(y, x, w, span, degree = degree, parametric = parametric, : all data on boundary of neighborhood. make span bigger
```

```
## Warning in simpleLoess(y, x, w, span, degree = degree, parametric = parametric, : pseudoinverse used at -0.005
```

```
## Warning in simpleLoess(y, x, w, span, degree = degree, parametric = parametric, : neighborhood radius 0.005
```

```
## Warning in simpleLoess(y, x, w, span, degree = degree, parametric = parametric, : reciprocal condition number 1
```

```
## Warning in simpleLoess(y, x, w, span, degree = degree, parametric = parametric, : There are other near singularities as well. 1.01
```

```
## Warning in simpleLoess(y, x, w, span, degree = degree, parametric = parametric, : zero-width neighborhood. make span bigger
```

```
## Warning: Computation failed in `stat_smooth()`:
## NA/NaN/Inf in foreign function call (arg 5)
```

```
## Warning in simpleLoess(y, x, w, span, degree = degree, parametric = parametric, : pseudoinverse used at -0.005
```

```
## Warning in simpleLoess(y, x, w, span, degree = degree, parametric = parametric, : neighborhood radius 1.005
```

```
## Warning in simpleLoess(y, x, w, span, degree = degree, parametric = parametric, : reciprocal condition number 0
```

```
## Warning in simpleLoess(y, x, w, span, degree = degree, parametric = parametric, : There are other near singularities as well. 1.01
```

```
## Warning in predLoess(object$y, object$x, newx = if (is.null(newdata)) object$x else if (is.data.frame(newdata)) as.matrix(model.frame(delete.response(terms(object)), : pseudoinverse used at -0.005
```

```
## Warning in predLoess(object$y, object$x, newx = if (is.null(newdata)) object$x else if (is.data.frame(newdata)) as.matrix(model.frame(delete.response(terms(object)), : neighborhood radius 1.005
```

```
## Warning in predLoess(object$y, object$x, newx = if (is.null(newdata)) object$x else if (is.data.frame(newdata)) as.matrix(model.frame(delete.response(terms(object)), : reciprocal condition number 0
```

```
## Warning in predLoess(object$y, object$x, newx = if (is.null(newdata)) object$x else if (is.data.frame(newdata)) as.matrix(model.frame(delete.response(terms(object)), : There are other near
## singularities as well. 1.01
```

```
## Warning in simpleLoess(y, x, w, span, degree = degree, parametric = parametric, : at -0.005
```

```
## Warning in simpleLoess(y, x, w, span, degree = degree, parametric = parametric, : radius 2.5e-05
```

```
## Warning in simpleLoess(y, x, w, span, degree = degree, parametric = parametric, : all data on boundary of neighborhood. make span bigger
```

```
## Warning in simpleLoess(y, x, w, span, degree = degree, parametric = parametric, : pseudoinverse used at -0.005
```

```
## Warning in simpleLoess(y, x, w, span, degree = degree, parametric = parametric, : neighborhood radius 0.005
```

```
## Warning in simpleLoess(y, x, w, span, degree = degree, parametric = parametric, : reciprocal condition number 1
```

```
## Warning in simpleLoess(y, x, w, span, degree = degree, parametric = parametric, : There are other near singularities as well. 1.01
```

```
## Warning in simpleLoess(y, x, w, span, degree = degree, parametric = parametric, : zero-width neighborhood. make span bigger
```

```
## Warning: Computation failed in `stat_smooth()`:
## NA/NaN/Inf in foreign function call (arg 5)
```

```
## Warning in simpleLoess(y, x, w, span, degree = degree, parametric = parametric, : at -0.005
```

```
## Warning in simpleLoess(y, x, w, span, degree = degree, parametric = parametric, : radius 2.5e-05
```

```
## Warning in simpleLoess(y, x, w, span, degree = degree, parametric = parametric, : all data on boundary of neighborhood. make span bigger
```

```
## Warning in simpleLoess(y, x, w, span, degree = degree, parametric = parametric, : pseudoinverse used at -0.005
```

```
## Warning in simpleLoess(y, x, w, span, degree = degree, parametric = parametric, : neighborhood radius 0.005
```

```
## Warning in simpleLoess(y, x, w, span, degree = degree, parametric = parametric, : reciprocal condition number 1
```

```
## Warning in simpleLoess(y, x, w, span, degree = degree, parametric = parametric, : There are other near singularities as well. 1.01
```

```
## Warning in simpleLoess(y, x, w, span, degree = degree, parametric = parametric, : zero-width neighborhood. make span bigger
```

```
## Warning: Computation failed in `stat_smooth()`:
## NA/NaN/Inf in foreign function call (arg 5)
```

```
## Warning in simpleLoess(y, x, w, span, degree = degree, parametric = parametric, : at -0.005
```

```
## Warning in simpleLoess(y, x, w, span, degree = degree, parametric = parametric, : radius 2.5e-05
```

```
## Warning in simpleLoess(y, x, w, span, degree = degree, parametric = parametric, : all data on boundary of neighborhood. make span bigger
```

```
## Warning in simpleLoess(y, x, w, span, degree = degree, parametric = parametric, : pseudoinverse used at -0.005
```

```
## Warning in simpleLoess(y, x, w, span, degree = degree, parametric = parametric, : neighborhood radius 0.005
```

```
## Warning in simpleLoess(y, x, w, span, degree = degree, parametric = parametric, : reciprocal condition number 1
```

```
## Warning in simpleLoess(y, x, w, span, degree = degree, parametric = parametric, : There are other near singularities as well. 1.01
```

```
## Warning in simpleLoess(y, x, w, span, degree = degree, parametric = parametric, : zero-width neighborhood. make span bigger
```

```
## Warning: Computation failed in `stat_smooth()`:
## NA/NaN/Inf in foreign function call (arg 5)
```

```
## Warning in simpleLoess(y, x, w, span, degree = degree, parametric = parametric, : at -0.005
```

```
## Warning in simpleLoess(y, x, w, span, degree = degree, parametric = parametric, : radius 2.5e-05
```

```
## Warning in simpleLoess(y, x, w, span, degree = degree, parametric = parametric, : all data on boundary of neighborhood. make span bigger
```

```
## Warning in simpleLoess(y, x, w, span, degree = degree, parametric = parametric, : pseudoinverse used at -0.005
```

```
## Warning in simpleLoess(y, x, w, span, degree = degree, parametric = parametric, : neighborhood radius 0.005
```

```
## Warning in simpleLoess(y, x, w, span, degree = degree, parametric = parametric, : reciprocal condition number 1
```

```
## Warning in simpleLoess(y, x, w, span, degree = degree, parametric = parametric, : There are other near singularities as well. 1.01
```

```
## Warning in simpleLoess(y, x, w, span, degree = degree, parametric = parametric, : zero-width neighborhood. make span bigger
```

```
## Warning: Computation failed in `stat_smooth()`:
## NA/NaN/Inf in foreign function call (arg 5)
```

```
## Warning in simpleLoess(y, x, w, span, degree = degree, parametric = parametric, : at -0.005
```

```
## Warning in simpleLoess(y, x, w, span, degree = degree, parametric = parametric, : radius 2.5e-05
```

```
## Warning in simpleLoess(y, x, w, span, degree = degree, parametric = parametric, : all data on boundary of neighborhood. make span bigger
```

```
## Warning in simpleLoess(y, x, w, span, degree = degree, parametric = parametric, : pseudoinverse used at -0.005
```

```
## Warning in simpleLoess(y, x, w, span, degree = degree, parametric = parametric, : neighborhood radius 0.005
```

```
## Warning in simpleLoess(y, x, w, span, degree = degree, parametric = parametric, : reciprocal condition number 1
```

```
## Warning in simpleLoess(y, x, w, span, degree = degree, parametric = parametric, : There are other near singularities as well. 1.01
```

```
## Warning in simpleLoess(y, x, w, span, degree = degree, parametric = parametric, : zero-width neighborhood. make span bigger
```

```
## Warning: Computation failed in `stat_smooth()`:
## NA/NaN/Inf in foreign function call (arg 5)
```

```
## Warning in simpleLoess(y, x, w, span, degree = degree, parametric = parametric, : at -0.005
```

```
## Warning in simpleLoess(y, x, w, span, degree = degree, parametric = parametric, : radius 2.5e-05
```

```
## Warning in simpleLoess(y, x, w, span, degree = degree, parametric = parametric, : all data on boundary of neighborhood. make span bigger
```

```
## Warning in simpleLoess(y, x, w, span, degree = degree, parametric = parametric, : pseudoinverse used at -0.005
```

```
## Warning in simpleLoess(y, x, w, span, degree = degree, parametric = parametric, : neighborhood radius 0.005
```

```
## Warning in simpleLoess(y, x, w, span, degree = degree, parametric = parametric, : reciprocal condition number 1
```

```
## Warning in simpleLoess(y, x, w, span, degree = degree, parametric = parametric, : There are other near singularities as well. 1.01
```

```
## Warning in simpleLoess(y, x, w, span, degree = degree, parametric = parametric, : zero-width neighborhood. make span bigger
```

```
## Warning: Computation failed in `stat_smooth()`:
## NA/NaN/Inf in foreign function call (arg 5)
```

```
## Warning in simpleLoess(y, x, w, span, degree = degree, parametric = parametric, : at -0.005
```

```
## Warning in simpleLoess(y, x, w, span, degree = degree, parametric = parametric, : radius 2.5e-05
```

```
## Warning in simpleLoess(y, x, w, span, degree = degree, parametric = parametric, : all data on boundary of neighborhood. make span bigger
```

```
## Warning in simpleLoess(y, x, w, span, degree = degree, parametric = parametric, : pseudoinverse used at -0.005
```

```
## Warning in simpleLoess(y, x, w, span, degree = degree, parametric = parametric, : neighborhood radius 0.005
```

```
## Warning in simpleLoess(y, x, w, span, degree = degree, parametric = parametric, : reciprocal condition number 1
```

```
## Warning in simpleLoess(y, x, w, span, degree = degree, parametric = parametric, : There are other near singularities as well. 1.01
```

```
## Warning in simpleLoess(y, x, w, span, degree = degree, parametric = parametric, : zero-width neighborhood. make span bigger
```

```
## Warning: Computation failed in `stat_smooth()`:
## NA/NaN/Inf in foreign function call (arg 5)
```

```
## Warning in simpleLoess(y, x, w, span, degree = degree, parametric = parametric, : at -0.005
```

```
## Warning in simpleLoess(y, x, w, span, degree = degree, parametric = parametric, : radius 2.5e-05
```

```
## Warning in simpleLoess(y, x, w, span, degree = degree, parametric = parametric, : all data on boundary of neighborhood. make span bigger
```

```
## Warning in simpleLoess(y, x, w, span, degree = degree, parametric = parametric, : pseudoinverse used at -0.005
```

```
## Warning in simpleLoess(y, x, w, span, degree = degree, parametric = parametric, : neighborhood radius 0.005
```

```
## Warning in simpleLoess(y, x, w, span, degree = degree, parametric = parametric, : reciprocal condition number 1
```

```
## Warning in simpleLoess(y, x, w, span, degree = degree, parametric = parametric, : There are other near singularities as well. 1.01
```

```
## Warning in simpleLoess(y, x, w, span, degree = degree, parametric = parametric, : zero-width neighborhood. make span bigger
```

```
## Warning: Computation failed in `stat_smooth()`:
## NA/NaN/Inf in foreign function call (arg 5)
```

```
## Warning in simpleLoess(y, x, w, span, degree = degree, parametric = parametric, : at -0.005
```

```
## Warning in simpleLoess(y, x, w, span, degree = degree, parametric = parametric, : radius 2.5e-05
```

```
## Warning in simpleLoess(y, x, w, span, degree = degree, parametric = parametric, : all data on boundary of neighborhood. make span bigger
```

```
## Warning in simpleLoess(y, x, w, span, degree = degree, parametric = parametric, : pseudoinverse used at -0.005
```

```
## Warning in simpleLoess(y, x, w, span, degree = degree, parametric = parametric, : neighborhood radius 0.005
```

```
## Warning in simpleLoess(y, x, w, span, degree = degree, parametric = parametric, : reciprocal condition number 1
```

```
## Warning in simpleLoess(y, x, w, span, degree = degree, parametric = parametric, : There are other near singularities as well. 1.01
```

```
## Warning in simpleLoess(y, x, w, span, degree = degree, parametric = parametric, : zero-width neighborhood. make span bigger
```

```
## Warning: Computation failed in `stat_smooth()`:
## NA/NaN/Inf in foreign function call (arg 5)
```

```
## Warning in simpleLoess(y, x, w, span, degree = degree, parametric = parametric, : at -0.005
```

```
## Warning in simpleLoess(y, x, w, span, degree = degree, parametric = parametric, : radius 2.5e-05
```

```
## Warning in simpleLoess(y, x, w, span, degree = degree, parametric = parametric, : all data on boundary of neighborhood. make span bigger
```

```
## Warning in simpleLoess(y, x, w, span, degree = degree, parametric = parametric, : pseudoinverse used at -0.005
```

```
## Warning in simpleLoess(y, x, w, span, degree = degree, parametric = parametric, : neighborhood radius 0.005
```

```
## Warning in simpleLoess(y, x, w, span, degree = degree, parametric = parametric, : reciprocal condition number 1
```

```
## Warning in simpleLoess(y, x, w, span, degree = degree, parametric = parametric, : There are other near singularities as well. 1.01
```

```
## Warning in simpleLoess(y, x, w, span, degree = degree, parametric = parametric, : zero-width neighborhood. make span bigger
```

```
## Warning: Computation failed in `stat_smooth()`:
## NA/NaN/Inf in foreign function call (arg 5)
```

```
## Warning in simpleLoess(y, x, w, span, degree = degree, parametric = parametric, : at -0.005
```

```
## Warning in simpleLoess(y, x, w, span, degree = degree, parametric = parametric, : radius 2.5e-05
```

```
## Warning in simpleLoess(y, x, w, span, degree = degree, parametric = parametric, : all data on boundary of neighborhood. make span bigger
```

```
## Warning in simpleLoess(y, x, w, span, degree = degree, parametric = parametric, : pseudoinverse used at -0.005
```

```
## Warning in simpleLoess(y, x, w, span, degree = degree, parametric = parametric, : neighborhood radius 0.005
```

```
## Warning in simpleLoess(y, x, w, span, degree = degree, parametric = parametric, : reciprocal condition number 1
```

```
## Warning in simpleLoess(y, x, w, span, degree = degree, parametric = parametric, : There are other near singularities as well. 1.01
```

```
## Warning in simpleLoess(y, x, w, span, degree = degree, parametric = parametric, : zero-width neighborhood. make span bigger
```

```
## Warning: Computation failed in `stat_smooth()`:
## NA/NaN/Inf in foreign function call (arg 5)
```

```
## Warning in simpleLoess(y, x, w, span, degree = degree, parametric = parametric, : at -0.005
```

```
## Warning in simpleLoess(y, x, w, span, degree = degree, parametric = parametric, : radius 2.5e-05
```

```
## Warning in simpleLoess(y, x, w, span, degree = degree, parametric = parametric, : all data on boundary of neighborhood. make span bigger
```

```
## Warning in simpleLoess(y, x, w, span, degree = degree, parametric = parametric, : pseudoinverse used at -0.005
```

```
## Warning in simpleLoess(y, x, w, span, degree = degree, parametric = parametric, : neighborhood radius 0.005
```

```
## Warning in simpleLoess(y, x, w, span, degree = degree, parametric = parametric, : reciprocal condition number 1
```

```
## Warning in simpleLoess(y, x, w, span, degree = degree, parametric = parametric, : There are other near singularities as well. 1.01
```

```
## Warning in simpleLoess(y, x, w, span, degree = degree, parametric = parametric, : zero-width neighborhood. make span bigger
```

```
## Warning: Computation failed in `stat_smooth()`:
## NA/NaN/Inf in foreign function call (arg 5)
```

```
## Warning in simpleLoess(y, x, w, span, degree = degree, parametric = parametric, : at -0.005
```

```
## Warning in simpleLoess(y, x, w, span, degree = degree, parametric = parametric, : radius 2.5e-05
```

```
## Warning in simpleLoess(y, x, w, span, degree = degree, parametric = parametric, : all data on boundary of neighborhood. make span bigger
```

```
## Warning in simpleLoess(y, x, w, span, degree = degree, parametric = parametric, : pseudoinverse used at -0.005
```

```
## Warning in simpleLoess(y, x, w, span, degree = degree, parametric = parametric, : neighborhood radius 0.005
```

```
## Warning in simpleLoess(y, x, w, span, degree = degree, parametric = parametric, : reciprocal condition number 1
```

```
## Warning in simpleLoess(y, x, w, span, degree = degree, parametric = parametric, : There are other near singularities as well. 1.01
```

```
## Warning in simpleLoess(y, x, w, span, degree = degree, parametric = parametric, : zero-width neighborhood. make span bigger
```

```
## Warning: Computation failed in `stat_smooth()`:
## NA/NaN/Inf in foreign function call (arg 5)
```

```
## Warning in simpleLoess(y, x, w, span, degree = degree, parametric = parametric, : at -0.005
```

```
## Warning in simpleLoess(y, x, w, span, degree = degree, parametric = parametric, : radius 2.5e-05
```

```
## Warning in simpleLoess(y, x, w, span, degree = degree, parametric = parametric, : all data on boundary of neighborhood. make span bigger
```

```
## Warning in simpleLoess(y, x, w, span, degree = degree, parametric = parametric, : pseudoinverse used at -0.005
```

```
## Warning in simpleLoess(y, x, w, span, degree = degree, parametric = parametric, : neighborhood radius 0.005
```

```
## Warning in simpleLoess(y, x, w, span, degree = degree, parametric = parametric, : reciprocal condition number 1
```

```
## Warning in simpleLoess(y, x, w, span, degree = degree, parametric = parametric, : There are other near singularities as well. 1.01
```

```
## Warning in simpleLoess(y, x, w, span, degree = degree, parametric = parametric, : zero-width neighborhood. make span bigger
```

```
## Warning: Computation failed in `stat_smooth()`:
## NA/NaN/Inf in foreign function call (arg 5)
```

```
## Warning in simpleLoess(y, x, w, span, degree = degree, parametric = parametric, : at -0.005
```

```
## Warning in simpleLoess(y, x, w, span, degree = degree, parametric = parametric, : radius 2.5e-05
```

```
## Warning in simpleLoess(y, x, w, span, degree = degree, parametric = parametric, : all data on boundary of neighborhood. make span bigger
```

```
## Warning in simpleLoess(y, x, w, span, degree = degree, parametric = parametric, : pseudoinverse used at -0.005
```

```
## Warning in simpleLoess(y, x, w, span, degree = degree, parametric = parametric, : neighborhood radius 0.005
```

```
## Warning in simpleLoess(y, x, w, span, degree = degree, parametric = parametric, : reciprocal condition number 1
```

```
## Warning in simpleLoess(y, x, w, span, degree = degree, parametric = parametric, : There are other near singularities as well. 1.01
```

```
## Warning in simpleLoess(y, x, w, span, degree = degree, parametric = parametric, : zero-width neighborhood. make span bigger
```

```
## Warning: Computation failed in `stat_smooth()`:
## NA/NaN/Inf in foreign function call (arg 5)
```

```
## Warning in simpleLoess(y, x, w, span, degree = degree, parametric = parametric, : at -0.005
```

```
## Warning in simpleLoess(y, x, w, span, degree = degree, parametric = parametric, : radius 2.5e-05
```

```
## Warning in simpleLoess(y, x, w, span, degree = degree, parametric = parametric, : all data on boundary of neighborhood. make span bigger
```

```
## Warning in simpleLoess(y, x, w, span, degree = degree, parametric = parametric, : pseudoinverse used at -0.005
```

```
## Warning in simpleLoess(y, x, w, span, degree = degree, parametric = parametric, : neighborhood radius 0.005
```

```
## Warning in simpleLoess(y, x, w, span, degree = degree, parametric = parametric, : reciprocal condition number 1
```

```
## Warning in simpleLoess(y, x, w, span, degree = degree, parametric = parametric, : There are other near singularities as well. 1.01
```

```
## Warning in simpleLoess(y, x, w, span, degree = degree, parametric = parametric, : zero-width neighborhood. make span bigger
```

```
## Warning: Computation failed in `stat_smooth()`:
## NA/NaN/Inf in foreign function call (arg 5)
```

```
## Warning in simpleLoess(y, x, w, span, degree = degree, parametric = parametric, : at -0.005
```

```
## Warning in simpleLoess(y, x, w, span, degree = degree, parametric = parametric, : radius 2.5e-05
```

```
## Warning in simpleLoess(y, x, w, span, degree = degree, parametric = parametric, : all data on boundary of neighborhood. make span bigger
```

```
## Warning in simpleLoess(y, x, w, span, degree = degree, parametric = parametric, : pseudoinverse used at -0.005
```

```
## Warning in simpleLoess(y, x, w, span, degree = degree, parametric = parametric, : neighborhood radius 0.005
```

```
## Warning in simpleLoess(y, x, w, span, degree = degree, parametric = parametric, : reciprocal condition number 1
```

```
## Warning in simpleLoess(y, x, w, span, degree = degree, parametric = parametric, : There are other near singularities as well. 1.01
```

```
## Warning in simpleLoess(y, x, w, span, degree = degree, parametric = parametric, : zero-width neighborhood. make span bigger
```

```
## Warning: Computation failed in `stat_smooth()`:
## NA/NaN/Inf in foreign function call (arg 5)
```

```
## Warning in simpleLoess(y, x, w, span, degree = degree, parametric = parametric, : at -0.005
```

```
## Warning in simpleLoess(y, x, w, span, degree = degree, parametric = parametric, : radius 2.5e-05
```

```
## Warning in simpleLoess(y, x, w, span, degree = degree, parametric = parametric, : all data on boundary of neighborhood. make span bigger
```

```
## Warning in simpleLoess(y, x, w, span, degree = degree, parametric = parametric, : pseudoinverse used at -0.005
```

```
## Warning in simpleLoess(y, x, w, span, degree = degree, parametric = parametric, : neighborhood radius 0.005
```

```
## Warning in simpleLoess(y, x, w, span, degree = degree, parametric = parametric, : reciprocal condition number 1
```

```
## Warning in simpleLoess(y, x, w, span, degree = degree, parametric = parametric, : There are other near singularities as well. 1.01
```

```
## Warning in simpleLoess(y, x, w, span, degree = degree, parametric = parametric, : zero-width neighborhood. make span bigger
```

```
## Warning: Computation failed in `stat_smooth()`:
## NA/NaN/Inf in foreign function call (arg 5)
```

```
## Warning in simpleLoess(y, x, w, span, degree = degree, parametric = parametric, : at -0.005
```

```
## Warning in simpleLoess(y, x, w, span, degree = degree, parametric = parametric, : radius 2.5e-05
```

```
## Warning in simpleLoess(y, x, w, span, degree = degree, parametric = parametric, : all data on boundary of neighborhood. make span bigger
```

```
## Warning in simpleLoess(y, x, w, span, degree = degree, parametric = parametric, : pseudoinverse used at -0.005
```

```
## Warning in simpleLoess(y, x, w, span, degree = degree, parametric = parametric, : neighborhood radius 0.005
```

```
## Warning in simpleLoess(y, x, w, span, degree = degree, parametric = parametric, : reciprocal condition number 1
```

```
## Warning in simpleLoess(y, x, w, span, degree = degree, parametric = parametric, : There are other near singularities as well. 1.01
```

```
## Warning in simpleLoess(y, x, w, span, degree = degree, parametric = parametric, : zero-width neighborhood. make span bigger
```

```
## Warning: Computation failed in `stat_smooth()`:
## NA/NaN/Inf in foreign function call (arg 5)
```

```
## Warning in simpleLoess(y, x, w, span, degree = degree, parametric = parametric, : at -0.005
```

```
## Warning in simpleLoess(y, x, w, span, degree = degree, parametric = parametric, : radius 2.5e-05
```

```
## Warning in simpleLoess(y, x, w, span, degree = degree, parametric = parametric, : all data on boundary of neighborhood. make span bigger
```

```
## Warning in simpleLoess(y, x, w, span, degree = degree, parametric = parametric, : pseudoinverse used at -0.005
```

```
## Warning in simpleLoess(y, x, w, span, degree = degree, parametric = parametric, : neighborhood radius 0.005
```

```
## Warning in simpleLoess(y, x, w, span, degree = degree, parametric = parametric, : reciprocal condition number 1
```

```
## Warning in simpleLoess(y, x, w, span, degree = degree, parametric = parametric, : There are other near singularities as well. 1.01
```

```
## Warning in simpleLoess(y, x, w, span, degree = degree, parametric = parametric, : zero-width neighborhood. make span bigger
```

```
## Warning: Computation failed in `stat_smooth()`:
## NA/NaN/Inf in foreign function call (arg 5)
```

```
## Warning in simpleLoess(y, x, w, span, degree = degree, parametric = parametric, : at -0.005
```

```
## Warning in simpleLoess(y, x, w, span, degree = degree, parametric = parametric, : radius 2.5e-05
```

```
## Warning in simpleLoess(y, x, w, span, degree = degree, parametric = parametric, : all data on boundary of neighborhood. make span bigger
```

```
## Warning in simpleLoess(y, x, w, span, degree = degree, parametric = parametric, : pseudoinverse used at -0.005
```

```
## Warning in simpleLoess(y, x, w, span, degree = degree, parametric = parametric, : neighborhood radius 0.005
```

```
## Warning in simpleLoess(y, x, w, span, degree = degree, parametric = parametric, : reciprocal condition number 1
```

```
## Warning in simpleLoess(y, x, w, span, degree = degree, parametric = parametric, : There are other near singularities as well. 1.01
```

```
## Warning in simpleLoess(y, x, w, span, degree = degree, parametric = parametric, : zero-width neighborhood. make span bigger
```

```
## Warning: Computation failed in `stat_smooth()`:
## NA/NaN/Inf in foreign function call (arg 5)
```

```
## Warning in simpleLoess(y, x, w, span, degree = degree, parametric = parametric, : at -0.005
```

```
## Warning in simpleLoess(y, x, w, span, degree = degree, parametric = parametric, : radius 2.5e-05
```

```
## Warning in simpleLoess(y, x, w, span, degree = degree, parametric = parametric, : all data on boundary of neighborhood. make span bigger
```

```
## Warning in simpleLoess(y, x, w, span, degree = degree, parametric = parametric, : pseudoinverse used at -0.005
```

```
## Warning in simpleLoess(y, x, w, span, degree = degree, parametric = parametric, : neighborhood radius 0.005
```

```
## Warning in simpleLoess(y, x, w, span, degree = degree, parametric = parametric, : reciprocal condition number 1
```

```
## Warning in simpleLoess(y, x, w, span, degree = degree, parametric = parametric, : There are other near singularities as well. 1.01
```

```
## Warning in simpleLoess(y, x, w, span, degree = degree, parametric = parametric, : zero-width neighborhood. make span bigger
```

```
## Warning: Computation failed in `stat_smooth()`:
## NA/NaN/Inf in foreign function call (arg 5)
```

```
## Warning in simpleLoess(y, x, w, span, degree = degree, parametric = parametric, : pseudoinverse used at -0.005
```

```
## Warning in simpleLoess(y, x, w, span, degree = degree, parametric = parametric, : neighborhood radius 1.005
```

```
## Warning in simpleLoess(y, x, w, span, degree = degree, parametric = parametric, : reciprocal condition number 0
```

```
## Warning in simpleLoess(y, x, w, span, degree = degree, parametric = parametric, : There are other near singularities as well. 1.01
```

```
## Warning in predLoess(object$y, object$x, newx = if (is.null(newdata)) object$x else if (is.data.frame(newdata)) as.matrix(model.frame(delete.response(terms(object)), : pseudoinverse used at -0.005
```

```
## Warning in predLoess(object$y, object$x, newx = if (is.null(newdata)) object$x else if (is.data.frame(newdata)) as.matrix(model.frame(delete.response(terms(object)), : neighborhood radius 1.005
```

```
## Warning in predLoess(object$y, object$x, newx = if (is.null(newdata)) object$x else if (is.data.frame(newdata)) as.matrix(model.frame(delete.response(terms(object)), : reciprocal condition number 0
```

```
## Warning in predLoess(object$y, object$x, newx = if (is.null(newdata)) object$x else if (is.data.frame(newdata)) as.matrix(model.frame(delete.response(terms(object)), : There are other near
## singularities as well. 1.01
```

```
## Warning in simpleLoess(y, x, w, span, degree = degree, parametric = parametric, : pseudoinverse used at -0.005
```

```
## Warning in simpleLoess(y, x, w, span, degree = degree, parametric = parametric, : neighborhood radius 1.005
```

```
## Warning in simpleLoess(y, x, w, span, degree = degree, parametric = parametric, : reciprocal condition number 0
```

```
## Warning in simpleLoess(y, x, w, span, degree = degree, parametric = parametric, : There are other near singularities as well. 1.01
```

```
## Warning in predLoess(object$y, object$x, newx = if (is.null(newdata)) object$x else if (is.data.frame(newdata)) as.matrix(model.frame(delete.response(terms(object)), : pseudoinverse used at -0.005
```

```
## Warning in predLoess(object$y, object$x, newx = if (is.null(newdata)) object$x else if (is.data.frame(newdata)) as.matrix(model.frame(delete.response(terms(object)), : neighborhood radius 1.005
```

```
## Warning in predLoess(object$y, object$x, newx = if (is.null(newdata)) object$x else if (is.data.frame(newdata)) as.matrix(model.frame(delete.response(terms(object)), : reciprocal condition number 0
```

```
## Warning in predLoess(object$y, object$x, newx = if (is.null(newdata)) object$x else if (is.data.frame(newdata)) as.matrix(model.frame(delete.response(terms(object)), : There are other near
## singularities as well. 1.01
```

```
## Warning in simpleLoess(y, x, w, span, degree = degree, parametric = parametric, : pseudoinverse used at -0.005
```

```
## Warning in simpleLoess(y, x, w, span, degree = degree, parametric = parametric, : neighborhood radius 1.005
```

```
## Warning in simpleLoess(y, x, w, span, degree = degree, parametric = parametric, : reciprocal condition number 0
```

```
## Warning in simpleLoess(y, x, w, span, degree = degree, parametric = parametric, : There are other near singularities as well. 1.01
```

```
## Warning in predLoess(object$y, object$x, newx = if (is.null(newdata)) object$x else if (is.data.frame(newdata)) as.matrix(model.frame(delete.response(terms(object)), : pseudoinverse used at -0.005
```

```
## Warning in predLoess(object$y, object$x, newx = if (is.null(newdata)) object$x else if (is.data.frame(newdata)) as.matrix(model.frame(delete.response(terms(object)), : neighborhood radius 1.005
```

```
## Warning in predLoess(object$y, object$x, newx = if (is.null(newdata)) object$x else if (is.data.frame(newdata)) as.matrix(model.frame(delete.response(terms(object)), : reciprocal condition number 0
```

```
## Warning in predLoess(object$y, object$x, newx = if (is.null(newdata)) object$x else if (is.data.frame(newdata)) as.matrix(model.frame(delete.response(terms(object)), : There are other near
## singularities as well. 1.01
```

```
## Warning in simpleLoess(y, x, w, span, degree = degree, parametric = parametric, : pseudoinverse used at -0.005
```

```
## Warning in simpleLoess(y, x, w, span, degree = degree, parametric = parametric, : neighborhood radius 1.005
```

```
## Warning in simpleLoess(y, x, w, span, degree = degree, parametric = parametric, : reciprocal condition number 0
```

```
## Warning in simpleLoess(y, x, w, span, degree = degree, parametric = parametric, : There are other near singularities as well. 1.01
```

```
## Warning in predLoess(object$y, object$x, newx = if (is.null(newdata)) object$x else if (is.data.frame(newdata)) as.matrix(model.frame(delete.response(terms(object)), : pseudoinverse used at -0.005
```

```
## Warning in predLoess(object$y, object$x, newx = if (is.null(newdata)) object$x else if (is.data.frame(newdata)) as.matrix(model.frame(delete.response(terms(object)), : neighborhood radius 1.005
```

```
## Warning in predLoess(object$y, object$x, newx = if (is.null(newdata)) object$x else if (is.data.frame(newdata)) as.matrix(model.frame(delete.response(terms(object)), : reciprocal condition number 0
```

```
## Warning in predLoess(object$y, object$x, newx = if (is.null(newdata)) object$x else if (is.data.frame(newdata)) as.matrix(model.frame(delete.response(terms(object)), : There are other near
## singularities as well. 1.01
```

```
## Warning in simpleLoess(y, x, w, span, degree = degree, parametric = parametric, : at -0.005
```

```
## Warning in simpleLoess(y, x, w, span, degree = degree, parametric = parametric, : radius 2.5e-05
```

```
## Warning in simpleLoess(y, x, w, span, degree = degree, parametric = parametric, : all data on boundary of neighborhood. make span bigger
```

```
## Warning in simpleLoess(y, x, w, span, degree = degree, parametric = parametric, : pseudoinverse used at -0.005
```

```
## Warning in simpleLoess(y, x, w, span, degree = degree, parametric = parametric, : neighborhood radius 0.005
```

```
## Warning in simpleLoess(y, x, w, span, degree = degree, parametric = parametric, : reciprocal condition number 1
```

```
## Warning in simpleLoess(y, x, w, span, degree = degree, parametric = parametric, : There are other near singularities as well. 1.01
```

```
## Warning in simpleLoess(y, x, w, span, degree = degree, parametric = parametric, : zero-width neighborhood. make span bigger
```

```
## Warning: Computation failed in `stat_smooth()`:
## NA/NaN/Inf in foreign function call (arg 5)
```

```
## Warning in simpleLoess(y, x, w, span, degree = degree, parametric = parametric, : pseudoinverse used at -0.005
```

```
## Warning in simpleLoess(y, x, w, span, degree = degree, parametric = parametric, : neighborhood radius 1.005
```

```
## Warning in simpleLoess(y, x, w, span, degree = degree, parametric = parametric, : reciprocal condition number 0
```

```
## Warning in simpleLoess(y, x, w, span, degree = degree, parametric = parametric, : There are other near singularities as well. 1.01
```

```
## Warning in predLoess(object$y, object$x, newx = if (is.null(newdata)) object$x else if (is.data.frame(newdata)) as.matrix(model.frame(delete.response(terms(object)), : pseudoinverse used at -0.005
```

```
## Warning in predLoess(object$y, object$x, newx = if (is.null(newdata)) object$x else if (is.data.frame(newdata)) as.matrix(model.frame(delete.response(terms(object)), : neighborhood radius 1.005
```

```
## Warning in predLoess(object$y, object$x, newx = if (is.null(newdata)) object$x else if (is.data.frame(newdata)) as.matrix(model.frame(delete.response(terms(object)), : reciprocal condition number 0
```

```
## Warning in predLoess(object$y, object$x, newx = if (is.null(newdata)) object$x else if (is.data.frame(newdata)) as.matrix(model.frame(delete.response(terms(object)), : There are other near
## singularities as well. 1.01
```

```
## Warning in simpleLoess(y, x, w, span, degree = degree, parametric = parametric, : at -0.005
```

```
## Warning in simpleLoess(y, x, w, span, degree = degree, parametric = parametric, : radius 2.5e-05
```

```
## Warning in simpleLoess(y, x, w, span, degree = degree, parametric = parametric, : all data on boundary of neighborhood. make span bigger
```

```
## Warning in simpleLoess(y, x, w, span, degree = degree, parametric = parametric, : pseudoinverse used at -0.005
```

```
## Warning in simpleLoess(y, x, w, span, degree = degree, parametric = parametric, : neighborhood radius 0.005
```

```
## Warning in simpleLoess(y, x, w, span, degree = degree, parametric = parametric, : reciprocal condition number 1
```

```
## Warning in simpleLoess(y, x, w, span, degree = degree, parametric = parametric, : There are other near singularities as well. 1.01
```

```
## Warning in simpleLoess(y, x, w, span, degree = degree, parametric = parametric, : zero-width neighborhood. make span bigger
```

```
## Warning: Computation failed in `stat_smooth()`:
## NA/NaN/Inf in foreign function call (arg 5)
```

```
## Warning in simpleLoess(y, x, w, span, degree = degree, parametric = parametric, : at -0.005
```

```
## Warning in simpleLoess(y, x, w, span, degree = degree, parametric = parametric, : radius 2.5e-05
```

```
## Warning in simpleLoess(y, x, w, span, degree = degree, parametric = parametric, : all data on boundary of neighborhood. make span bigger
```

```
## Warning in simpleLoess(y, x, w, span, degree = degree, parametric = parametric, : pseudoinverse used at -0.005
```

```
## Warning in simpleLoess(y, x, w, span, degree = degree, parametric = parametric, : neighborhood radius 0.005
```

```
## Warning in simpleLoess(y, x, w, span, degree = degree, parametric = parametric, : reciprocal condition number 1
```

```
## Warning in simpleLoess(y, x, w, span, degree = degree, parametric = parametric, : There are other near singularities as well. 1.01
```

```
## Warning in simpleLoess(y, x, w, span, degree = degree, parametric = parametric, : zero-width neighborhood. make span bigger
```

```
## Warning: Computation failed in `stat_smooth()`:
## NA/NaN/Inf in foreign function call (arg 5)
```

```
## Warning in simpleLoess(y, x, w, span, degree = degree, parametric = parametric, : at -0.005
```

```
## Warning in simpleLoess(y, x, w, span, degree = degree, parametric = parametric, : radius 2.5e-05
```

```
## Warning in simpleLoess(y, x, w, span, degree = degree, parametric = parametric, : all data on boundary of neighborhood. make span bigger
```

```
## Warning in simpleLoess(y, x, w, span, degree = degree, parametric = parametric, : pseudoinverse used at -0.005
```

```
## Warning in simpleLoess(y, x, w, span, degree = degree, parametric = parametric, : neighborhood radius 0.005
```

```
## Warning in simpleLoess(y, x, w, span, degree = degree, parametric = parametric, : reciprocal condition number 1
```

```
## Warning in simpleLoess(y, x, w, span, degree = degree, parametric = parametric, : There are other near singularities as well. 1.01
```

```
## Warning in simpleLoess(y, x, w, span, degree = degree, parametric = parametric, : zero-width neighborhood. make span bigger
```

```
## Warning: Computation failed in `stat_smooth()`:
## NA/NaN/Inf in foreign function call (arg 5)
```

```
## Warning in simpleLoess(y, x, w, span, degree = degree, parametric = parametric, : at -0.005
```

```
## Warning in simpleLoess(y, x, w, span, degree = degree, parametric = parametric, : radius 2.5e-05
```

```
## Warning in simpleLoess(y, x, w, span, degree = degree, parametric = parametric, : all data on boundary of neighborhood. make span bigger
```

```
## Warning in simpleLoess(y, x, w, span, degree = degree, parametric = parametric, : pseudoinverse used at -0.005
```

```
## Warning in simpleLoess(y, x, w, span, degree = degree, parametric = parametric, : neighborhood radius 0.005
```

```
## Warning in simpleLoess(y, x, w, span, degree = degree, parametric = parametric, : reciprocal condition number 1
```

```
## Warning in simpleLoess(y, x, w, span, degree = degree, parametric = parametric, : There are other near singularities as well. 1.01
```

```
## Warning in simpleLoess(y, x, w, span, degree = degree, parametric = parametric, : zero-width neighborhood. make span bigger
```

```
## Warning: Computation failed in `stat_smooth()`:
## NA/NaN/Inf in foreign function call (arg 5)
```

```
## Warning in simpleLoess(y, x, w, span, degree = degree, parametric = parametric, : at -0.005
```

```
## Warning in simpleLoess(y, x, w, span, degree = degree, parametric = parametric, : radius 2.5e-05
```

```
## Warning in simpleLoess(y, x, w, span, degree = degree, parametric = parametric, : all data on boundary of neighborhood. make span bigger
```

```
## Warning in simpleLoess(y, x, w, span, degree = degree, parametric = parametric, : pseudoinverse used at -0.005
```

```
## Warning in simpleLoess(y, x, w, span, degree = degree, parametric = parametric, : neighborhood radius 0.005
```

```
## Warning in simpleLoess(y, x, w, span, degree = degree, parametric = parametric, : reciprocal condition number 1
```

```
## Warning in simpleLoess(y, x, w, span, degree = degree, parametric = parametric, : There are other near singularities as well. 1.01
```

```
## Warning in simpleLoess(y, x, w, span, degree = degree, parametric = parametric, : zero-width neighborhood. make span bigger
```

```
## Warning: Computation failed in `stat_smooth()`:
## NA/NaN/Inf in foreign function call (arg 5)
```

```
## Warning in simpleLoess(y, x, w, span, degree = degree, parametric = parametric, : at -0.005
```

```
## Warning in simpleLoess(y, x, w, span, degree = degree, parametric = parametric, : radius 2.5e-05
```

```
## Warning in simpleLoess(y, x, w, span, degree = degree, parametric = parametric, : all data on boundary of neighborhood. make span bigger
```

```
## Warning in simpleLoess(y, x, w, span, degree = degree, parametric = parametric, : pseudoinverse used at -0.005
```

```
## Warning in simpleLoess(y, x, w, span, degree = degree, parametric = parametric, : neighborhood radius 0.005
```

```
## Warning in simpleLoess(y, x, w, span, degree = degree, parametric = parametric, : reciprocal condition number 1
```

```
## Warning in simpleLoess(y, x, w, span, degree = degree, parametric = parametric, : There are other near singularities as well. 1.01
```

```
## Warning in simpleLoess(y, x, w, span, degree = degree, parametric = parametric, : zero-width neighborhood. make span bigger
```

```
## Warning: Computation failed in `stat_smooth()`:
## NA/NaN/Inf in foreign function call (arg 5)
```

```
## Warning in simpleLoess(y, x, w, span, degree = degree, parametric = parametric, : at -0.005
```

```
## Warning in simpleLoess(y, x, w, span, degree = degree, parametric = parametric, : radius 2.5e-05
```

```
## Warning in simpleLoess(y, x, w, span, degree = degree, parametric = parametric, : all data on boundary of neighborhood. make span bigger
```

```
## Warning in simpleLoess(y, x, w, span, degree = degree, parametric = parametric, : pseudoinverse used at -0.005
```

```
## Warning in simpleLoess(y, x, w, span, degree = degree, parametric = parametric, : neighborhood radius 0.005
```

```
## Warning in simpleLoess(y, x, w, span, degree = degree, parametric = parametric, : reciprocal condition number 1
```

```
## Warning in simpleLoess(y, x, w, span, degree = degree, parametric = parametric, : There are other near singularities as well. 1.01
```

```
## Warning in simpleLoess(y, x, w, span, degree = degree, parametric = parametric, : zero-width neighborhood. make span bigger
```

```
## Warning: Computation failed in `stat_smooth()`:
## NA/NaN/Inf in foreign function call (arg 5)
```

```
## Warning in simpleLoess(y, x, w, span, degree = degree, parametric = parametric, : at -0.005
```

```
## Warning in simpleLoess(y, x, w, span, degree = degree, parametric = parametric, : radius 2.5e-05
```

```
## Warning in simpleLoess(y, x, w, span, degree = degree, parametric = parametric, : all data on boundary of neighborhood. make span bigger
```

```
## Warning in simpleLoess(y, x, w, span, degree = degree, parametric = parametric, : pseudoinverse used at -0.005
```

```
## Warning in simpleLoess(y, x, w, span, degree = degree, parametric = parametric, : neighborhood radius 0.005
```

```
## Warning in simpleLoess(y, x, w, span, degree = degree, parametric = parametric, : reciprocal condition number 1
```

```
## Warning in simpleLoess(y, x, w, span, degree = degree, parametric = parametric, : There are other near singularities as well. 1.01
```

```
## Warning in simpleLoess(y, x, w, span, degree = degree, parametric = parametric, : zero-width neighborhood. make span bigger
```

```
## Warning: Computation failed in `stat_smooth()`:
## NA/NaN/Inf in foreign function call (arg 5)
```

```
## Warning in simpleLoess(y, x, w, span, degree = degree, parametric = parametric, : at -0.005
```

```
## Warning in simpleLoess(y, x, w, span, degree = degree, parametric = parametric, : radius 2.5e-05
```

```
## Warning in simpleLoess(y, x, w, span, degree = degree, parametric = parametric, : all data on boundary of neighborhood. make span bigger
```

```
## Warning in simpleLoess(y, x, w, span, degree = degree, parametric = parametric, : pseudoinverse used at -0.005
```

```
## Warning in simpleLoess(y, x, w, span, degree = degree, parametric = parametric, : neighborhood radius 0.005
```

```
## Warning in simpleLoess(y, x, w, span, degree = degree, parametric = parametric, : reciprocal condition number 1
```

```
## Warning in simpleLoess(y, x, w, span, degree = degree, parametric = parametric, : There are other near singularities as well. 1.01
```

```
## Warning in simpleLoess(y, x, w, span, degree = degree, parametric = parametric, : zero-width neighborhood. make span bigger
```

```
## Warning: Computation failed in `stat_smooth()`:
## NA/NaN/Inf in foreign function call (arg 5)
```

```
## Warning in simpleLoess(y, x, w, span, degree = degree, parametric = parametric, : at -0.005
```

```
## Warning in simpleLoess(y, x, w, span, degree = degree, parametric = parametric, : radius 2.5e-05
```

```
## Warning in simpleLoess(y, x, w, span, degree = degree, parametric = parametric, : all data on boundary of neighborhood. make span bigger
```

```
## Warning in simpleLoess(y, x, w, span, degree = degree, parametric = parametric, : pseudoinverse used at -0.005
```

```
## Warning in simpleLoess(y, x, w, span, degree = degree, parametric = parametric, : neighborhood radius 0.005
```

```
## Warning in simpleLoess(y, x, w, span, degree = degree, parametric = parametric, : reciprocal condition number 1
```

```
## Warning in simpleLoess(y, x, w, span, degree = degree, parametric = parametric, : There are other near singularities as well. 1.01
```

```
## Warning in simpleLoess(y, x, w, span, degree = degree, parametric = parametric, : zero-width neighborhood. make span bigger
```

```
## Warning: Computation failed in `stat_smooth()`:
## NA/NaN/Inf in foreign function call (arg 5)
```

```
## Warning in simpleLoess(y, x, w, span, degree = degree, parametric = parametric, : at -0.005
```

```
## Warning in simpleLoess(y, x, w, span, degree = degree, parametric = parametric, : radius 2.5e-05
```

```
## Warning in simpleLoess(y, x, w, span, degree = degree, parametric = parametric, : all data on boundary of neighborhood. make span bigger
```

```
## Warning in simpleLoess(y, x, w, span, degree = degree, parametric = parametric, : pseudoinverse used at -0.005
```

```
## Warning in simpleLoess(y, x, w, span, degree = degree, parametric = parametric, : neighborhood radius 0.005
```

```
## Warning in simpleLoess(y, x, w, span, degree = degree, parametric = parametric, : reciprocal condition number 1
```

```
## Warning in simpleLoess(y, x, w, span, degree = degree, parametric = parametric, : There are other near singularities as well. 1.01
```

```
## Warning in simpleLoess(y, x, w, span, degree = degree, parametric = parametric, : zero-width neighborhood. make span bigger
```

```
## Warning: Computation failed in `stat_smooth()`:
## NA/NaN/Inf in foreign function call (arg 5)
```

```
## Warning in simpleLoess(y, x, w, span, degree = degree, parametric = parametric, : at -0.005
```

```
## Warning in simpleLoess(y, x, w, span, degree = degree, parametric = parametric, : radius 2.5e-05
```

```
## Warning in simpleLoess(y, x, w, span, degree = degree, parametric = parametric, : all data on boundary of neighborhood. make span bigger
```

```
## Warning in simpleLoess(y, x, w, span, degree = degree, parametric = parametric, : pseudoinverse used at -0.005
```

```
## Warning in simpleLoess(y, x, w, span, degree = degree, parametric = parametric, : neighborhood radius 0.005
```

```
## Warning in simpleLoess(y, x, w, span, degree = degree, parametric = parametric, : reciprocal condition number 1
```

```
## Warning in simpleLoess(y, x, w, span, degree = degree, parametric = parametric, : There are other near singularities as well. 1.01
```

```
## Warning in simpleLoess(y, x, w, span, degree = degree, parametric = parametric, : zero-width neighborhood. make span bigger
```

```
## Warning: Computation failed in `stat_smooth()`:
## NA/NaN/Inf in foreign function call (arg 5)
```

```
## Warning in simpleLoess(y, x, w, span, degree = degree, parametric = parametric, : at -0.005
```

```
## Warning in simpleLoess(y, x, w, span, degree = degree, parametric = parametric, : radius 2.5e-05
```

```
## Warning in simpleLoess(y, x, w, span, degree = degree, parametric = parametric, : all data on boundary of neighborhood. make span bigger
```

```
## Warning in simpleLoess(y, x, w, span, degree = degree, parametric = parametric, : pseudoinverse used at -0.005
```

```
## Warning in simpleLoess(y, x, w, span, degree = degree, parametric = parametric, : neighborhood radius 0.005
```

```
## Warning in simpleLoess(y, x, w, span, degree = degree, parametric = parametric, : reciprocal condition number 1
```

```
## Warning in simpleLoess(y, x, w, span, degree = degree, parametric = parametric, : There are other near singularities as well. 1.01
```

```
## Warning in simpleLoess(y, x, w, span, degree = degree, parametric = parametric, : zero-width neighborhood. make span bigger
```

```
## Warning: Computation failed in `stat_smooth()`:
## NA/NaN/Inf in foreign function call (arg 5)
```

```
## Warning in simpleLoess(y, x, w, span, degree = degree, parametric = parametric, : at -0.005
```

```
## Warning in simpleLoess(y, x, w, span, degree = degree, parametric = parametric, : radius 2.5e-05
```

```
## Warning in simpleLoess(y, x, w, span, degree = degree, parametric = parametric, : all data on boundary of neighborhood. make span bigger
```

```
## Warning in simpleLoess(y, x, w, span, degree = degree, parametric = parametric, : pseudoinverse used at -0.005
```

```
## Warning in simpleLoess(y, x, w, span, degree = degree, parametric = parametric, : neighborhood radius 0.005
```

```
## Warning in simpleLoess(y, x, w, span, degree = degree, parametric = parametric, : reciprocal condition number 1
```

```
## Warning in simpleLoess(y, x, w, span, degree = degree, parametric = parametric, : There are other near singularities as well. 1.01
```

```
## Warning in simpleLoess(y, x, w, span, degree = degree, parametric = parametric, : zero-width neighborhood. make span bigger
```

```
## Warning: Computation failed in `stat_smooth()`:
## NA/NaN/Inf in foreign function call (arg 5)
```

```
## Warning in simpleLoess(y, x, w, span, degree = degree, parametric = parametric, : at -0.005
```

```
## Warning in simpleLoess(y, x, w, span, degree = degree, parametric = parametric, : radius 2.5e-05
```

```
## Warning in simpleLoess(y, x, w, span, degree = degree, parametric = parametric, : all data on boundary of neighborhood. make span bigger
```

```
## Warning in simpleLoess(y, x, w, span, degree = degree, parametric = parametric, : pseudoinverse used at -0.005
```

```
## Warning in simpleLoess(y, x, w, span, degree = degree, parametric = parametric, : neighborhood radius 0.005
```

```
## Warning in simpleLoess(y, x, w, span, degree = degree, parametric = parametric, : reciprocal condition number 1
```

```
## Warning in simpleLoess(y, x, w, span, degree = degree, parametric = parametric, : There are other near singularities as well. 1.01
```

```
## Warning in simpleLoess(y, x, w, span, degree = degree, parametric = parametric, : zero-width neighborhood. make span bigger
```

```
## Warning: Computation failed in `stat_smooth()`:
## NA/NaN/Inf in foreign function call (arg 5)
```

```
## Warning in simpleLoess(y, x, w, span, degree = degree, parametric = parametric, : at -0.005
```

```
## Warning in simpleLoess(y, x, w, span, degree = degree, parametric = parametric, : radius 2.5e-05
```

```
## Warning in simpleLoess(y, x, w, span, degree = degree, parametric = parametric, : all data on boundary of neighborhood. make span bigger
```

```
## Warning in simpleLoess(y, x, w, span, degree = degree, parametric = parametric, : pseudoinverse used at -0.005
```

```
## Warning in simpleLoess(y, x, w, span, degree = degree, parametric = parametric, : neighborhood radius 0.005
```

```
## Warning in simpleLoess(y, x, w, span, degree = degree, parametric = parametric, : reciprocal condition number 1
```

```
## Warning in simpleLoess(y, x, w, span, degree = degree, parametric = parametric, : There are other near singularities as well. 1.01
```

```
## Warning in simpleLoess(y, x, w, span, degree = degree, parametric = parametric, : zero-width neighborhood. make span bigger
```

```
## Warning: Computation failed in `stat_smooth()`:
## NA/NaN/Inf in foreign function call (arg 5)
```

```
## Warning in simpleLoess(y, x, w, span, degree = degree, parametric = parametric, : pseudoinverse used at -0.005
```

```
## Warning in simpleLoess(y, x, w, span, degree = degree, parametric = parametric, : neighborhood radius 1.005
```

```
## Warning in simpleLoess(y, x, w, span, degree = degree, parametric = parametric, : reciprocal condition number 0
```

```
## Warning in simpleLoess(y, x, w, span, degree = degree, parametric = parametric, : There are other near singularities as well. 1.01
```

```
## Warning in predLoess(object$y, object$x, newx = if (is.null(newdata)) object$x else if (is.data.frame(newdata)) as.matrix(model.frame(delete.response(terms(object)), : pseudoinverse used at -0.005
```

```
## Warning in predLoess(object$y, object$x, newx = if (is.null(newdata)) object$x else if (is.data.frame(newdata)) as.matrix(model.frame(delete.response(terms(object)), : neighborhood radius 1.005
```

```
## Warning in predLoess(object$y, object$x, newx = if (is.null(newdata)) object$x else if (is.data.frame(newdata)) as.matrix(model.frame(delete.response(terms(object)), : reciprocal condition number 0
```

```
## Warning in predLoess(object$y, object$x, newx = if (is.null(newdata)) object$x else if (is.data.frame(newdata)) as.matrix(model.frame(delete.response(terms(object)), : There are other near
## singularities as well. 1.01
```

```
## Warning in simpleLoess(y, x, w, span, degree = degree, parametric = parametric, : at -0.005
```

```
## Warning in simpleLoess(y, x, w, span, degree = degree, parametric = parametric, : radius 2.5e-05
```

```
## Warning in simpleLoess(y, x, w, span, degree = degree, parametric = parametric, : all data on boundary of neighborhood. make span bigger
```

```
## Warning in simpleLoess(y, x, w, span, degree = degree, parametric = parametric, : pseudoinverse used at -0.005
```

```
## Warning in simpleLoess(y, x, w, span, degree = degree, parametric = parametric, : neighborhood radius 0.005
```

```
## Warning in simpleLoess(y, x, w, span, degree = degree, parametric = parametric, : reciprocal condition number 1
```

```
## Warning in simpleLoess(y, x, w, span, degree = degree, parametric = parametric, : There are other near singularities as well. 1.01
```

```
## Warning in simpleLoess(y, x, w, span, degree = degree, parametric = parametric, : zero-width neighborhood. make span bigger
```

```
## Warning: Computation failed in `stat_smooth()`:
## NA/NaN/Inf in foreign function call (arg 5)
```

```
## Warning in simpleLoess(y, x, w, span, degree = degree, parametric = parametric, : at -0.005
```

```
## Warning in simpleLoess(y, x, w, span, degree = degree, parametric = parametric, : radius 2.5e-05
```

```
## Warning in simpleLoess(y, x, w, span, degree = degree, parametric = parametric, : all data on boundary of neighborhood. make span bigger
```

```
## Warning in simpleLoess(y, x, w, span, degree = degree, parametric = parametric, : pseudoinverse used at -0.005
```

```
## Warning in simpleLoess(y, x, w, span, degree = degree, parametric = parametric, : neighborhood radius 0.005
```

```
## Warning in simpleLoess(y, x, w, span, degree = degree, parametric = parametric, : reciprocal condition number 1
```

```
## Warning in simpleLoess(y, x, w, span, degree = degree, parametric = parametric, : There are other near singularities as well. 1.01
```

```
## Warning in simpleLoess(y, x, w, span, degree = degree, parametric = parametric, : zero-width neighborhood. make span bigger
```

```
## Warning: Computation failed in `stat_smooth()`:
## NA/NaN/Inf in foreign function call (arg 5)
```

```
## Warning in simpleLoess(y, x, w, span, degree = degree, parametric = parametric, : pseudoinverse used at -0.005
```

```
## Warning in simpleLoess(y, x, w, span, degree = degree, parametric = parametric, : neighborhood radius 1.005
```

```
## Warning in simpleLoess(y, x, w, span, degree = degree, parametric = parametric, : reciprocal condition number 0
```

```
## Warning in simpleLoess(y, x, w, span, degree = degree, parametric = parametric, : There are other near singularities as well. 1.01
```

```
## Warning in predLoess(object$y, object$x, newx = if (is.null(newdata)) object$x else if (is.data.frame(newdata)) as.matrix(model.frame(delete.response(terms(object)), : pseudoinverse used at -0.005
```

```
## Warning in predLoess(object$y, object$x, newx = if (is.null(newdata)) object$x else if (is.data.frame(newdata)) as.matrix(model.frame(delete.response(terms(object)), : neighborhood radius 1.005
```

```
## Warning in predLoess(object$y, object$x, newx = if (is.null(newdata)) object$x else if (is.data.frame(newdata)) as.matrix(model.frame(delete.response(terms(object)), : reciprocal condition number 0
```

```
## Warning in predLoess(object$y, object$x, newx = if (is.null(newdata)) object$x else if (is.data.frame(newdata)) as.matrix(model.frame(delete.response(terms(object)), : There are other near
## singularities as well. 1.01
```

```
## Warning in simpleLoess(y, x, w, span, degree = degree, parametric = parametric, : at -0.005
```

```
## Warning in simpleLoess(y, x, w, span, degree = degree, parametric = parametric, : radius 2.5e-05
```

```
## Warning in simpleLoess(y, x, w, span, degree = degree, parametric = parametric, : all data on boundary of neighborhood. make span bigger
```

```
## Warning in simpleLoess(y, x, w, span, degree = degree, parametric = parametric, : pseudoinverse used at -0.005
```

```
## Warning in simpleLoess(y, x, w, span, degree = degree, parametric = parametric, : neighborhood radius 0.005
```

```
## Warning in simpleLoess(y, x, w, span, degree = degree, parametric = parametric, : reciprocal condition number 1
```

```
## Warning in simpleLoess(y, x, w, span, degree = degree, parametric = parametric, : There are other near singularities as well. 1.01
```

```
## Warning in simpleLoess(y, x, w, span, degree = degree, parametric = parametric, : zero-width neighborhood. make span bigger
```

```
## Warning: Computation failed in `stat_smooth()`:
## NA/NaN/Inf in foreign function call (arg 5)
```

```
## Warning in simpleLoess(y, x, w, span, degree = degree, parametric = parametric, : at -0.005
```

```
## Warning in simpleLoess(y, x, w, span, degree = degree, parametric = parametric, : radius 2.5e-05
```

```
## Warning in simpleLoess(y, x, w, span, degree = degree, parametric = parametric, : all data on boundary of neighborhood. make span bigger
```

```
## Warning in simpleLoess(y, x, w, span, degree = degree, parametric = parametric, : pseudoinverse used at -0.005
```

```
## Warning in simpleLoess(y, x, w, span, degree = degree, parametric = parametric, : neighborhood radius 0.005
```

```
## Warning in simpleLoess(y, x, w, span, degree = degree, parametric = parametric, : reciprocal condition number 1
```

```
## Warning in simpleLoess(y, x, w, span, degree = degree, parametric = parametric, : There are other near singularities as well. 1.01
```

```
## Warning in simpleLoess(y, x, w, span, degree = degree, parametric = parametric, : zero-width neighborhood. make span bigger
```

```
## Warning: Computation failed in `stat_smooth()`:
## NA/NaN/Inf in foreign function call (arg 5)
```

```
## Warning in simpleLoess(y, x, w, span, degree = degree, parametric = parametric, : at -0.005
```

```
## Warning in simpleLoess(y, x, w, span, degree = degree, parametric = parametric, : radius 2.5e-05
```

```
## Warning in simpleLoess(y, x, w, span, degree = degree, parametric = parametric, : all data on boundary of neighborhood. make span bigger
```

```
## Warning in simpleLoess(y, x, w, span, degree = degree, parametric = parametric, : pseudoinverse used at -0.005
```

```
## Warning in simpleLoess(y, x, w, span, degree = degree, parametric = parametric, : neighborhood radius 0.005
```

```
## Warning in simpleLoess(y, x, w, span, degree = degree, parametric = parametric, : reciprocal condition number 1
```

```
## Warning in simpleLoess(y, x, w, span, degree = degree, parametric = parametric, : There are other near singularities as well. 1.01
```

```
## Warning in simpleLoess(y, x, w, span, degree = degree, parametric = parametric, : zero-width neighborhood. make span bigger
```

```
## Warning: Computation failed in `stat_smooth()`:
## NA/NaN/Inf in foreign function call (arg 5)
```

```
## Warning in simpleLoess(y, x, w, span, degree = degree, parametric = parametric, : at -0.005
```

```
## Warning in simpleLoess(y, x, w, span, degree = degree, parametric = parametric, : radius 2.5e-05
```

```
## Warning in simpleLoess(y, x, w, span, degree = degree, parametric = parametric, : all data on boundary of neighborhood. make span bigger
```

```
## Warning in simpleLoess(y, x, w, span, degree = degree, parametric = parametric, : pseudoinverse used at -0.005
```

```
## Warning in simpleLoess(y, x, w, span, degree = degree, parametric = parametric, : neighborhood radius 0.005
```

```
## Warning in simpleLoess(y, x, w, span, degree = degree, parametric = parametric, : reciprocal condition number 1
```

```
## Warning in simpleLoess(y, x, w, span, degree = degree, parametric = parametric, : There are other near singularities as well. 1.01
```

```
## Warning in simpleLoess(y, x, w, span, degree = degree, parametric = parametric, : zero-width neighborhood. make span bigger
```

```
## Warning: Computation failed in `stat_smooth()`:
## NA/NaN/Inf in foreign function call (arg 5)
```

```
## Warning in simpleLoess(y, x, w, span, degree = degree, parametric = parametric, : at -0.005
```

```
## Warning in simpleLoess(y, x, w, span, degree = degree, parametric = parametric, : radius 2.5e-05
```

```
## Warning in simpleLoess(y, x, w, span, degree = degree, parametric = parametric, : all data on boundary of neighborhood. make span bigger
```

```
## Warning in simpleLoess(y, x, w, span, degree = degree, parametric = parametric, : pseudoinverse used at -0.005
```

```
## Warning in simpleLoess(y, x, w, span, degree = degree, parametric = parametric, : neighborhood radius 0.005
```

```
## Warning in simpleLoess(y, x, w, span, degree = degree, parametric = parametric, : reciprocal condition number 1
```

```
## Warning in simpleLoess(y, x, w, span, degree = degree, parametric = parametric, : There are other near singularities as well. 1.01
```

```
## Warning in simpleLoess(y, x, w, span, degree = degree, parametric = parametric, : zero-width neighborhood. make span bigger
```

```
## Warning: Computation failed in `stat_smooth()`:
## NA/NaN/Inf in foreign function call (arg 5)
```

```
## Warning in simpleLoess(y, x, w, span, degree = degree, parametric = parametric, : at -0.005
```

```
## Warning in simpleLoess(y, x, w, span, degree = degree, parametric = parametric, : radius 2.5e-05
```

```
## Warning in simpleLoess(y, x, w, span, degree = degree, parametric = parametric, : all data on boundary of neighborhood. make span bigger
```

```
## Warning in simpleLoess(y, x, w, span, degree = degree, parametric = parametric, : pseudoinverse used at -0.005
```

```
## Warning in simpleLoess(y, x, w, span, degree = degree, parametric = parametric, : neighborhood radius 0.005
```

```
## Warning in simpleLoess(y, x, w, span, degree = degree, parametric = parametric, : reciprocal condition number 1
```

```
## Warning in simpleLoess(y, x, w, span, degree = degree, parametric = parametric, : There are other near singularities as well. 1.01
```

```
## Warning in simpleLoess(y, x, w, span, degree = degree, parametric = parametric, : zero-width neighborhood. make span bigger
```

```
## Warning: Computation failed in `stat_smooth()`:
## NA/NaN/Inf in foreign function call (arg 5)
```

```
## Warning in simpleLoess(y, x, w, span, degree = degree, parametric = parametric, : at -0.005
```

```
## Warning in simpleLoess(y, x, w, span, degree = degree, parametric = parametric, : radius 2.5e-05
```

```
## Warning in simpleLoess(y, x, w, span, degree = degree, parametric = parametric, : all data on boundary of neighborhood. make span bigger
```

```
## Warning in simpleLoess(y, x, w, span, degree = degree, parametric = parametric, : pseudoinverse used at -0.005
```

```
## Warning in simpleLoess(y, x, w, span, degree = degree, parametric = parametric, : neighborhood radius 0.005
```

```
## Warning in simpleLoess(y, x, w, span, degree = degree, parametric = parametric, : reciprocal condition number 1
```

```
## Warning in simpleLoess(y, x, w, span, degree = degree, parametric = parametric, : There are other near singularities as well. 1.01
```

```
## Warning in simpleLoess(y, x, w, span, degree = degree, parametric = parametric, : zero-width neighborhood. make span bigger
```

```
## Warning: Computation failed in `stat_smooth()`:
## NA/NaN/Inf in foreign function call (arg 5)
```

```
## Warning in simpleLoess(y, x, w, span, degree = degree, parametric = parametric, : at -0.005
```

```
## Warning in simpleLoess(y, x, w, span, degree = degree, parametric = parametric, : radius 2.5e-05
```

```
## Warning in simpleLoess(y, x, w, span, degree = degree, parametric = parametric, : all data on boundary of neighborhood. make span bigger
```

```
## Warning in simpleLoess(y, x, w, span, degree = degree, parametric = parametric, : pseudoinverse used at -0.005
```

```
## Warning in simpleLoess(y, x, w, span, degree = degree, parametric = parametric, : neighborhood radius 0.005
```

```
## Warning in simpleLoess(y, x, w, span, degree = degree, parametric = parametric, : reciprocal condition number 1
```

```
## Warning in simpleLoess(y, x, w, span, degree = degree, parametric = parametric, : There are other near singularities as well. 1.01
```

```
## Warning in simpleLoess(y, x, w, span, degree = degree, parametric = parametric, : zero-width neighborhood. make span bigger
```

```
## Warning: Computation failed in `stat_smooth()`:
## NA/NaN/Inf in foreign function call (arg 5)
```

```
## Warning in simpleLoess(y, x, w, span, degree = degree, parametric = parametric, : at -0.005
```

```
## Warning in simpleLoess(y, x, w, span, degree = degree, parametric = parametric, : radius 2.5e-05
```

```
## Warning in simpleLoess(y, x, w, span, degree = degree, parametric = parametric, : all data on boundary of neighborhood. make span bigger
```

```
## Warning in simpleLoess(y, x, w, span, degree = degree, parametric = parametric, : pseudoinverse used at -0.005
```

```
## Warning in simpleLoess(y, x, w, span, degree = degree, parametric = parametric, : neighborhood radius 0.005
```

```
## Warning in simpleLoess(y, x, w, span, degree = degree, parametric = parametric, : reciprocal condition number 1
```

```
## Warning in simpleLoess(y, x, w, span, degree = degree, parametric = parametric, : There are other near singularities as well. 1.01
```

```
## Warning in simpleLoess(y, x, w, span, degree = degree, parametric = parametric, : zero-width neighborhood. make span bigger
```

```
## Warning: Computation failed in `stat_smooth()`:
## NA/NaN/Inf in foreign function call (arg 5)
```

```
## Warning in simpleLoess(y, x, w, span, degree = degree, parametric = parametric, : at -0.005
```

```
## Warning in simpleLoess(y, x, w, span, degree = degree, parametric = parametric, : radius 2.5e-05
```

```
## Warning in simpleLoess(y, x, w, span, degree = degree, parametric = parametric, : all data on boundary of neighborhood. make span bigger
```

```
## Warning in simpleLoess(y, x, w, span, degree = degree, parametric = parametric, : pseudoinverse used at -0.005
```

```
## Warning in simpleLoess(y, x, w, span, degree = degree, parametric = parametric, : neighborhood radius 0.005
```

```
## Warning in simpleLoess(y, x, w, span, degree = degree, parametric = parametric, : reciprocal condition number 1
```

```
## Warning in simpleLoess(y, x, w, span, degree = degree, parametric = parametric, : There are other near singularities as well. 1.01
```

```
## Warning in simpleLoess(y, x, w, span, degree = degree, parametric = parametric, : zero-width neighborhood. make span bigger
```

```
## Warning: Computation failed in `stat_smooth()`:
## NA/NaN/Inf in foreign function call (arg 5)
```

```
## Warning in simpleLoess(y, x, w, span, degree = degree, parametric = parametric, : at -0.005
```

```
## Warning in simpleLoess(y, x, w, span, degree = degree, parametric = parametric, : radius 2.5e-05
```

```
## Warning in simpleLoess(y, x, w, span, degree = degree, parametric = parametric, : all data on boundary of neighborhood. make span bigger
```

```
## Warning in simpleLoess(y, x, w, span, degree = degree, parametric = parametric, : pseudoinverse used at -0.005
```

```
## Warning in simpleLoess(y, x, w, span, degree = degree, parametric = parametric, : neighborhood radius 0.005
```

```
## Warning in simpleLoess(y, x, w, span, degree = degree, parametric = parametric, : reciprocal condition number 1
```

```
## Warning in simpleLoess(y, x, w, span, degree = degree, parametric = parametric, : There are other near singularities as well. 1.01
```

```
## Warning in simpleLoess(y, x, w, span, degree = degree, parametric = parametric, : zero-width neighborhood. make span bigger
```

```
## Warning: Computation failed in `stat_smooth()`:
## NA/NaN/Inf in foreign function call (arg 5)
```

```
## Warning in simpleLoess(y, x, w, span, degree = degree, parametric = parametric, : at -0.005
```

```
## Warning in simpleLoess(y, x, w, span, degree = degree, parametric = parametric, : radius 2.5e-05
```

```
## Warning in simpleLoess(y, x, w, span, degree = degree, parametric = parametric, : all data on boundary of neighborhood. make span bigger
```

```
## Warning in simpleLoess(y, x, w, span, degree = degree, parametric = parametric, : pseudoinverse used at -0.005
```

```
## Warning in simpleLoess(y, x, w, span, degree = degree, parametric = parametric, : neighborhood radius 0.005
```

```
## Warning in simpleLoess(y, x, w, span, degree = degree, parametric = parametric, : reciprocal condition number 1
```

```
## Warning in simpleLoess(y, x, w, span, degree = degree, parametric = parametric, : There are other near singularities as well. 1.01
```

```
## Warning in simpleLoess(y, x, w, span, degree = degree, parametric = parametric, : zero-width neighborhood. make span bigger
```

```
## Warning: Computation failed in `stat_smooth()`:
## NA/NaN/Inf in foreign function call (arg 5)
```

```
## Warning in simpleLoess(y, x, w, span, degree = degree, parametric = parametric, : at -0.005
```

```
## Warning in simpleLoess(y, x, w, span, degree = degree, parametric = parametric, : radius 2.5e-05
```

```
## Warning in simpleLoess(y, x, w, span, degree = degree, parametric = parametric, : all data on boundary of neighborhood. make span bigger
```

```
## Warning in simpleLoess(y, x, w, span, degree = degree, parametric = parametric, : pseudoinverse used at -0.005
```

```
## Warning in simpleLoess(y, x, w, span, degree = degree, parametric = parametric, : neighborhood radius 0.005
```

```
## Warning in simpleLoess(y, x, w, span, degree = degree, parametric = parametric, : reciprocal condition number 1
```

```
## Warning in simpleLoess(y, x, w, span, degree = degree, parametric = parametric, : There are other near singularities as well. 1.01
```

```
## Warning in simpleLoess(y, x, w, span, degree = degree, parametric = parametric, : zero-width neighborhood. make span bigger
```

```
## Warning: Computation failed in `stat_smooth()`:
## NA/NaN/Inf in foreign function call (arg 5)
```

```
## Warning in simpleLoess(y, x, w, span, degree = degree, parametric = parametric, : pseudoinverse used at -0.005
```

```
## Warning in simpleLoess(y, x, w, span, degree = degree, parametric = parametric, : neighborhood radius 1.005
```

```
## Warning in simpleLoess(y, x, w, span, degree = degree, parametric = parametric, : reciprocal condition number 0
```

```
## Warning in simpleLoess(y, x, w, span, degree = degree, parametric = parametric, : There are other near singularities as well. 1.01
```

```
## Warning in predLoess(object$y, object$x, newx = if (is.null(newdata)) object$x else if (is.data.frame(newdata)) as.matrix(model.frame(delete.response(terms(object)), : pseudoinverse used at -0.005
```

```
## Warning in predLoess(object$y, object$x, newx = if (is.null(newdata)) object$x else if (is.data.frame(newdata)) as.matrix(model.frame(delete.response(terms(object)), : neighborhood radius 1.005
```

```
## Warning in predLoess(object$y, object$x, newx = if (is.null(newdata)) object$x else if (is.data.frame(newdata)) as.matrix(model.frame(delete.response(terms(object)), : reciprocal condition number 0
```

```
## Warning in predLoess(object$y, object$x, newx = if (is.null(newdata)) object$x else if (is.data.frame(newdata)) as.matrix(model.frame(delete.response(terms(object)), : There are other near
## singularities as well. 1.01
```

```
## Warning in simpleLoess(y, x, w, span, degree = degree, parametric = parametric, : at -0.005
```

```
## Warning in simpleLoess(y, x, w, span, degree = degree, parametric = parametric, : radius 2.5e-05
```

```
## Warning in simpleLoess(y, x, w, span, degree = degree, parametric = parametric, : all data on boundary of neighborhood. make span bigger
```

```
## Warning in simpleLoess(y, x, w, span, degree = degree, parametric = parametric, : pseudoinverse used at -0.005
```

```
## Warning in simpleLoess(y, x, w, span, degree = degree, parametric = parametric, : neighborhood radius 0.005
```

```
## Warning in simpleLoess(y, x, w, span, degree = degree, parametric = parametric, : reciprocal condition number 1
```

```
## Warning in simpleLoess(y, x, w, span, degree = degree, parametric = parametric, : There are other near singularities as well. 1.01
```

```
## Warning in simpleLoess(y, x, w, span, degree = degree, parametric = parametric, : zero-width neighborhood. make span bigger
```

```
## Warning: Computation failed in `stat_smooth()`:
## NA/NaN/Inf in foreign function call (arg 5)
```

```
## Warning in simpleLoess(y, x, w, span, degree = degree, parametric = parametric, : at -0.005
```

```
## Warning in simpleLoess(y, x, w, span, degree = degree, parametric = parametric, : radius 2.5e-05
```

```
## Warning in simpleLoess(y, x, w, span, degree = degree, parametric = parametric, : all data on boundary of neighborhood. make span bigger
```

```
## Warning in simpleLoess(y, x, w, span, degree = degree, parametric = parametric, : pseudoinverse used at -0.005
```

```
## Warning in simpleLoess(y, x, w, span, degree = degree, parametric = parametric, : neighborhood radius 0.005
```

```
## Warning in simpleLoess(y, x, w, span, degree = degree, parametric = parametric, : reciprocal condition number 1
```

```
## Warning in simpleLoess(y, x, w, span, degree = degree, parametric = parametric, : There are other near singularities as well. 1.01
```

```
## Warning in simpleLoess(y, x, w, span, degree = degree, parametric = parametric, : zero-width neighborhood. make span bigger
```

```
## Warning: Computation failed in `stat_smooth()`:
## NA/NaN/Inf in foreign function call (arg 5)
```

```
## Warning in simpleLoess(y, x, w, span, degree = degree, parametric = parametric, : at -0.005
```

```
## Warning in simpleLoess(y, x, w, span, degree = degree, parametric = parametric, : radius 2.5e-05
```

```
## Warning in simpleLoess(y, x, w, span, degree = degree, parametric = parametric, : all data on boundary of neighborhood. make span bigger
```

```
## Warning in simpleLoess(y, x, w, span, degree = degree, parametric = parametric, : pseudoinverse used at -0.005
```

```
## Warning in simpleLoess(y, x, w, span, degree = degree, parametric = parametric, : neighborhood radius 0.005
```

```
## Warning in simpleLoess(y, x, w, span, degree = degree, parametric = parametric, : reciprocal condition number 1
```

```
## Warning in simpleLoess(y, x, w, span, degree = degree, parametric = parametric, : There are other near singularities as well. 1.01
```

```
## Warning in simpleLoess(y, x, w, span, degree = degree, parametric = parametric, : zero-width neighborhood. make span bigger
```

```
## Warning: Computation failed in `stat_smooth()`:
## NA/NaN/Inf in foreign function call (arg 5)
```

```
## Warning in simpleLoess(y, x, w, span, degree = degree, parametric = parametric, : pseudoinverse used at -0.005
```

```
## Warning in simpleLoess(y, x, w, span, degree = degree, parametric = parametric, : neighborhood radius 1.005
```

```
## Warning in simpleLoess(y, x, w, span, degree = degree, parametric = parametric, : reciprocal condition number 0
```

```
## Warning in simpleLoess(y, x, w, span, degree = degree, parametric = parametric, : There are other near singularities as well. 1.01
```

```
## Warning in predLoess(object$y, object$x, newx = if (is.null(newdata)) object$x else if (is.data.frame(newdata)) as.matrix(model.frame(delete.response(terms(object)), : pseudoinverse used at -0.005
```

```
## Warning in predLoess(object$y, object$x, newx = if (is.null(newdata)) object$x else if (is.data.frame(newdata)) as.matrix(model.frame(delete.response(terms(object)), : neighborhood radius 1.005
```

```
## Warning in predLoess(object$y, object$x, newx = if (is.null(newdata)) object$x else if (is.data.frame(newdata)) as.matrix(model.frame(delete.response(terms(object)), : reciprocal condition number 0
```

```
## Warning in predLoess(object$y, object$x, newx = if (is.null(newdata)) object$x else if (is.data.frame(newdata)) as.matrix(model.frame(delete.response(terms(object)), : There are other near
## singularities as well. 1.01
```

```
## Warning in simpleLoess(y, x, w, span, degree = degree, parametric = parametric, : at -0.005
```

```
## Warning in simpleLoess(y, x, w, span, degree = degree, parametric = parametric, : radius 2.5e-05
```

```
## Warning in simpleLoess(y, x, w, span, degree = degree, parametric = parametric, : all data on boundary of neighborhood. make span bigger
```

```
## Warning in simpleLoess(y, x, w, span, degree = degree, parametric = parametric, : pseudoinverse used at -0.005
```

```
## Warning in simpleLoess(y, x, w, span, degree = degree, parametric = parametric, : neighborhood radius 0.005
```

```
## Warning in simpleLoess(y, x, w, span, degree = degree, parametric = parametric, : reciprocal condition number 1
```

```
## Warning in simpleLoess(y, x, w, span, degree = degree, parametric = parametric, : There are other near singularities as well. 1.01
```

```
## Warning in simpleLoess(y, x, w, span, degree = degree, parametric = parametric, : zero-width neighborhood. make span bigger
```

```
## Warning: Computation failed in `stat_smooth()`:
## NA/NaN/Inf in foreign function call (arg 5)
```

```
## Warning in simpleLoess(y, x, w, span, degree = degree, parametric = parametric, : at -0.005
```

```
## Warning in simpleLoess(y, x, w, span, degree = degree, parametric = parametric, : radius 2.5e-05
```

```
## Warning in simpleLoess(y, x, w, span, degree = degree, parametric = parametric, : all data on boundary of neighborhood. make span bigger
```

```
## Warning in simpleLoess(y, x, w, span, degree = degree, parametric = parametric, : pseudoinverse used at -0.005
```

```
## Warning in simpleLoess(y, x, w, span, degree = degree, parametric = parametric, : neighborhood radius 0.005
```

```
## Warning in simpleLoess(y, x, w, span, degree = degree, parametric = parametric, : reciprocal condition number 1
```

```
## Warning in simpleLoess(y, x, w, span, degree = degree, parametric = parametric, : There are other near singularities as well. 1.01
```

```
## Warning in simpleLoess(y, x, w, span, degree = degree, parametric = parametric, : zero-width neighborhood. make span bigger
```

```
## Warning: Computation failed in `stat_smooth()`:
## NA/NaN/Inf in foreign function call (arg 5)
```

```
## Warning in simpleLoess(y, x, w, span, degree = degree, parametric = parametric, : at -0.005
```

```
## Warning in simpleLoess(y, x, w, span, degree = degree, parametric = parametric, : radius 2.5e-05
```

```
## Warning in simpleLoess(y, x, w, span, degree = degree, parametric = parametric, : all data on boundary of neighborhood. make span bigger
```

```
## Warning in simpleLoess(y, x, w, span, degree = degree, parametric = parametric, : pseudoinverse used at -0.005
```

```
## Warning in simpleLoess(y, x, w, span, degree = degree, parametric = parametric, : neighborhood radius 0.005
```

```
## Warning in simpleLoess(y, x, w, span, degree = degree, parametric = parametric, : reciprocal condition number 1
```

```
## Warning in simpleLoess(y, x, w, span, degree = degree, parametric = parametric, : There are other near singularities as well. 1.01
```

```
## Warning in simpleLoess(y, x, w, span, degree = degree, parametric = parametric, : zero-width neighborhood. make span bigger
```

```
## Warning: Computation failed in `stat_smooth()`:
## NA/NaN/Inf in foreign function call (arg 5)
```

```
## Warning in simpleLoess(y, x, w, span, degree = degree, parametric = parametric, : at -0.005
```

```
## Warning in simpleLoess(y, x, w, span, degree = degree, parametric = parametric, : radius 2.5e-05
```

```
## Warning in simpleLoess(y, x, w, span, degree = degree, parametric = parametric, : all data on boundary of neighborhood. make span bigger
```

```
## Warning in simpleLoess(y, x, w, span, degree = degree, parametric = parametric, : pseudoinverse used at -0.005
```

```
## Warning in simpleLoess(y, x, w, span, degree = degree, parametric = parametric, : neighborhood radius 0.005
```

```
## Warning in simpleLoess(y, x, w, span, degree = degree, parametric = parametric, : reciprocal condition number 1
```

```
## Warning in simpleLoess(y, x, w, span, degree = degree, parametric = parametric, : There are other near singularities as well. 1.01
```

```
## Warning in simpleLoess(y, x, w, span, degree = degree, parametric = parametric, : zero-width neighborhood. make span bigger
```

```
## Warning: Computation failed in `stat_smooth()`:
## NA/NaN/Inf in foreign function call (arg 5)
```

```
## Warning in simpleLoess(y, x, w, span, degree = degree, parametric = parametric, : at -0.005
```

```
## Warning in simpleLoess(y, x, w, span, degree = degree, parametric = parametric, : radius 2.5e-05
```

```
## Warning in simpleLoess(y, x, w, span, degree = degree, parametric = parametric, : all data on boundary of neighborhood. make span bigger
```

```
## Warning in simpleLoess(y, x, w, span, degree = degree, parametric = parametric, : pseudoinverse used at -0.005
```

```
## Warning in simpleLoess(y, x, w, span, degree = degree, parametric = parametric, : neighborhood radius 0.005
```

```
## Warning in simpleLoess(y, x, w, span, degree = degree, parametric = parametric, : reciprocal condition number 1
```

```
## Warning in simpleLoess(y, x, w, span, degree = degree, parametric = parametric, : There are other near singularities as well. 1.01
```

```
## Warning in simpleLoess(y, x, w, span, degree = degree, parametric = parametric, : zero-width neighborhood. make span bigger
```

```
## Warning: Computation failed in `stat_smooth()`:
## NA/NaN/Inf in foreign function call (arg 5)
```

```
## Warning in simpleLoess(y, x, w, span, degree = degree, parametric = parametric, : at -0.005
```

```
## Warning in simpleLoess(y, x, w, span, degree = degree, parametric = parametric, : radius 2.5e-05
```

```
## Warning in simpleLoess(y, x, w, span, degree = degree, parametric = parametric, : all data on boundary of neighborhood. make span bigger
```

```
## Warning in simpleLoess(y, x, w, span, degree = degree, parametric = parametric, : pseudoinverse used at -0.005
```

```
## Warning in simpleLoess(y, x, w, span, degree = degree, parametric = parametric, : neighborhood radius 0.005
```

```
## Warning in simpleLoess(y, x, w, span, degree = degree, parametric = parametric, : reciprocal condition number 1
```

```
## Warning in simpleLoess(y, x, w, span, degree = degree, parametric = parametric, : There are other near singularities as well. 1.01
```

```
## Warning in simpleLoess(y, x, w, span, degree = degree, parametric = parametric, : zero-width neighborhood. make span bigger
```

```
## Warning: Computation failed in `stat_smooth()`:
## NA/NaN/Inf in foreign function call (arg 5)
```

```
## Warning in simpleLoess(y, x, w, span, degree = degree, parametric = parametric, : at -0.005
```

```
## Warning in simpleLoess(y, x, w, span, degree = degree, parametric = parametric, : radius 2.5e-05
```

```
## Warning in simpleLoess(y, x, w, span, degree = degree, parametric = parametric, : all data on boundary of neighborhood. make span bigger
```

```
## Warning in simpleLoess(y, x, w, span, degree = degree, parametric = parametric, : pseudoinverse used at -0.005
```

```
## Warning in simpleLoess(y, x, w, span, degree = degree, parametric = parametric, : neighborhood radius 0.005
```

```
## Warning in simpleLoess(y, x, w, span, degree = degree, parametric = parametric, : reciprocal condition number 1
```

```
## Warning in simpleLoess(y, x, w, span, degree = degree, parametric = parametric, : There are other near singularities as well. 1.01
```

```
## Warning in simpleLoess(y, x, w, span, degree = degree, parametric = parametric, : zero-width neighborhood. make span bigger
```

```
## Warning: Computation failed in `stat_smooth()`:
## NA/NaN/Inf in foreign function call (arg 5)
```

```
## Warning in simpleLoess(y, x, w, span, degree = degree, parametric = parametric, : at -0.005
```

```
## Warning in simpleLoess(y, x, w, span, degree = degree, parametric = parametric, : radius 2.5e-05
```

```
## Warning in simpleLoess(y, x, w, span, degree = degree, parametric = parametric, : all data on boundary of neighborhood. make span bigger
```

```
## Warning in simpleLoess(y, x, w, span, degree = degree, parametric = parametric, : pseudoinverse used at -0.005
```

```
## Warning in simpleLoess(y, x, w, span, degree = degree, parametric = parametric, : neighborhood radius 0.005
```

```
## Warning in simpleLoess(y, x, w, span, degree = degree, parametric = parametric, : reciprocal condition number 1
```

```
## Warning in simpleLoess(y, x, w, span, degree = degree, parametric = parametric, : There are other near singularities as well. 1.01
```

```
## Warning in simpleLoess(y, x, w, span, degree = degree, parametric = parametric, : zero-width neighborhood. make span bigger
```

```
## Warning: Computation failed in `stat_smooth()`:
## NA/NaN/Inf in foreign function call (arg 5)
```

```
## Warning in simpleLoess(y, x, w, span, degree = degree, parametric = parametric, : at -0.005
```

```
## Warning in simpleLoess(y, x, w, span, degree = degree, parametric = parametric, : radius 2.5e-05
```

```
## Warning in simpleLoess(y, x, w, span, degree = degree, parametric = parametric, : all data on boundary of neighborhood. make span bigger
```

```
## Warning in simpleLoess(y, x, w, span, degree = degree, parametric = parametric, : pseudoinverse used at -0.005
```

```
## Warning in simpleLoess(y, x, w, span, degree = degree, parametric = parametric, : neighborhood radius 0.005
```

```
## Warning in simpleLoess(y, x, w, span, degree = degree, parametric = parametric, : reciprocal condition number 1
```

```
## Warning in simpleLoess(y, x, w, span, degree = degree, parametric = parametric, : There are other near singularities as well. 1.01
```

```
## Warning in simpleLoess(y, x, w, span, degree = degree, parametric = parametric, : zero-width neighborhood. make span bigger
```

```
## Warning: Computation failed in `stat_smooth()`:
## NA/NaN/Inf in foreign function call (arg 5)
```

```
## Warning in simpleLoess(y, x, w, span, degree = degree, parametric = parametric, : at -0.005
```

```
## Warning in simpleLoess(y, x, w, span, degree = degree, parametric = parametric, : radius 2.5e-05
```

```
## Warning in simpleLoess(y, x, w, span, degree = degree, parametric = parametric, : all data on boundary of neighborhood. make span bigger
```

```
## Warning in simpleLoess(y, x, w, span, degree = degree, parametric = parametric, : pseudoinverse used at -0.005
```

```
## Warning in simpleLoess(y, x, w, span, degree = degree, parametric = parametric, : neighborhood radius 0.005
```

```
## Warning in simpleLoess(y, x, w, span, degree = degree, parametric = parametric, : reciprocal condition number 1
```

```
## Warning in simpleLoess(y, x, w, span, degree = degree, parametric = parametric, : There are other near singularities as well. 1.01
```

```
## Warning in simpleLoess(y, x, w, span, degree = degree, parametric = parametric, : zero-width neighborhood. make span bigger
```

```
## Warning: Computation failed in `stat_smooth()`:
## NA/NaN/Inf in foreign function call (arg 5)
```

```
## Warning in simpleLoess(y, x, w, span, degree = degree, parametric = parametric, : pseudoinverse used at -0.005
```

```
## Warning in simpleLoess(y, x, w, span, degree = degree, parametric = parametric, : neighborhood radius 1.005
```

```
## Warning in simpleLoess(y, x, w, span, degree = degree, parametric = parametric, : reciprocal condition number 0
```

```
## Warning in simpleLoess(y, x, w, span, degree = degree, parametric = parametric, : There are other near singularities as well. 1.01
```

```
## Warning in predLoess(object$y, object$x, newx = if (is.null(newdata)) object$x else if (is.data.frame(newdata)) as.matrix(model.frame(delete.response(terms(object)), : pseudoinverse used at -0.005
```

```
## Warning in predLoess(object$y, object$x, newx = if (is.null(newdata)) object$x else if (is.data.frame(newdata)) as.matrix(model.frame(delete.response(terms(object)), : neighborhood radius 1.005
```

```
## Warning in predLoess(object$y, object$x, newx = if (is.null(newdata)) object$x else if (is.data.frame(newdata)) as.matrix(model.frame(delete.response(terms(object)), : reciprocal condition number 0
```

```
## Warning in predLoess(object$y, object$x, newx = if (is.null(newdata)) object$x else if (is.data.frame(newdata)) as.matrix(model.frame(delete.response(terms(object)), : There are other near
## singularities as well. 1.01
```

```
## Warning in simpleLoess(y, x, w, span, degree = degree, parametric = parametric, : at -0.005
```

```
## Warning in simpleLoess(y, x, w, span, degree = degree, parametric = parametric, : radius 2.5e-05
```

```
## Warning in simpleLoess(y, x, w, span, degree = degree, parametric = parametric, : all data on boundary of neighborhood. make span bigger
```

```
## Warning in simpleLoess(y, x, w, span, degree = degree, parametric = parametric, : pseudoinverse used at -0.005
```

```
## Warning in simpleLoess(y, x, w, span, degree = degree, parametric = parametric, : neighborhood radius 0.005
```

```
## Warning in simpleLoess(y, x, w, span, degree = degree, parametric = parametric, : reciprocal condition number 1
```

```
## Warning in simpleLoess(y, x, w, span, degree = degree, parametric = parametric, : There are other near singularities as well. 1.01
```

```
## Warning in simpleLoess(y, x, w, span, degree = degree, parametric = parametric, : zero-width neighborhood. make span bigger
```

```
## Warning: Computation failed in `stat_smooth()`:
## NA/NaN/Inf in foreign function call (arg 5)
```

```
## Warning in simpleLoess(y, x, w, span, degree = degree, parametric = parametric, : at -0.005
```

```
## Warning in simpleLoess(y, x, w, span, degree = degree, parametric = parametric, : radius 2.5e-05
```

```
## Warning in simpleLoess(y, x, w, span, degree = degree, parametric = parametric, : all data on boundary of neighborhood. make span bigger
```

```
## Warning in simpleLoess(y, x, w, span, degree = degree, parametric = parametric, : pseudoinverse used at -0.005
```

```
## Warning in simpleLoess(y, x, w, span, degree = degree, parametric = parametric, : neighborhood radius 0.005
```

```
## Warning in simpleLoess(y, x, w, span, degree = degree, parametric = parametric, : reciprocal condition number 1
```

```
## Warning in simpleLoess(y, x, w, span, degree = degree, parametric = parametric, : There are other near singularities as well. 1.01
```

```
## Warning in simpleLoess(y, x, w, span, degree = degree, parametric = parametric, : zero-width neighborhood. make span bigger
```

```
## Warning: Computation failed in `stat_smooth()`:
## NA/NaN/Inf in foreign function call (arg 5)
```

```
## Warning in simpleLoess(y, x, w, span, degree = degree, parametric = parametric, : at -0.005
```

```
## Warning in simpleLoess(y, x, w, span, degree = degree, parametric = parametric, : radius 2.5e-05
```

```
## Warning in simpleLoess(y, x, w, span, degree = degree, parametric = parametric, : all data on boundary of neighborhood. make span bigger
```

```
## Warning in simpleLoess(y, x, w, span, degree = degree, parametric = parametric, : pseudoinverse used at -0.005
```

```
## Warning in simpleLoess(y, x, w, span, degree = degree, parametric = parametric, : neighborhood radius 0.005
```

```
## Warning in simpleLoess(y, x, w, span, degree = degree, parametric = parametric, : reciprocal condition number 1
```

```
## Warning in simpleLoess(y, x, w, span, degree = degree, parametric = parametric, : There are other near singularities as well. 1.01
```

```
## Warning in simpleLoess(y, x, w, span, degree = degree, parametric = parametric, : zero-width neighborhood. make span bigger
```

```
## Warning: Computation failed in `stat_smooth()`:
## NA/NaN/Inf in foreign function call (arg 5)
```

```
## Warning in simpleLoess(y, x, w, span, degree = degree, parametric = parametric, : at -0.005
```

```
## Warning in simpleLoess(y, x, w, span, degree = degree, parametric = parametric, : radius 2.5e-05
```

```
## Warning in simpleLoess(y, x, w, span, degree = degree, parametric = parametric, : all data on boundary of neighborhood. make span bigger
```

```
## Warning in simpleLoess(y, x, w, span, degree = degree, parametric = parametric, : pseudoinverse used at -0.005
```

```
## Warning in simpleLoess(y, x, w, span, degree = degree, parametric = parametric, : neighborhood radius 0.005
```

```
## Warning in simpleLoess(y, x, w, span, degree = degree, parametric = parametric, : reciprocal condition number 1
```

```
## Warning in simpleLoess(y, x, w, span, degree = degree, parametric = parametric, : There are other near singularities as well. 1.01
```

```
## Warning in simpleLoess(y, x, w, span, degree = degree, parametric = parametric, : zero-width neighborhood. make span bigger
```

```
## Warning: Computation failed in `stat_smooth()`:
## NA/NaN/Inf in foreign function call (arg 5)
```

```
## Warning in simpleLoess(y, x, w, span, degree = degree, parametric = parametric, : at -0.005
```

```
## Warning in simpleLoess(y, x, w, span, degree = degree, parametric = parametric, : radius 2.5e-05
```

```
## Warning in simpleLoess(y, x, w, span, degree = degree, parametric = parametric, : all data on boundary of neighborhood. make span bigger
```

```
## Warning in simpleLoess(y, x, w, span, degree = degree, parametric = parametric, : pseudoinverse used at -0.005
```

```
## Warning in simpleLoess(y, x, w, span, degree = degree, parametric = parametric, : neighborhood radius 0.005
```

```
## Warning in simpleLoess(y, x, w, span, degree = degree, parametric = parametric, : reciprocal condition number 1
```

```
## Warning in simpleLoess(y, x, w, span, degree = degree, parametric = parametric, : There are other near singularities as well. 1.01
```

```
## Warning in simpleLoess(y, x, w, span, degree = degree, parametric = parametric, : zero-width neighborhood. make span bigger
```

```
## Warning: Computation failed in `stat_smooth()`:
## NA/NaN/Inf in foreign function call (arg 5)
```

```
## Warning in simpleLoess(y, x, w, span, degree = degree, parametric = parametric, : at -0.005
```

```
## Warning in simpleLoess(y, x, w, span, degree = degree, parametric = parametric, : radius 2.5e-05
```

```
## Warning in simpleLoess(y, x, w, span, degree = degree, parametric = parametric, : all data on boundary of neighborhood. make span bigger
```

```
## Warning in simpleLoess(y, x, w, span, degree = degree, parametric = parametric, : pseudoinverse used at -0.005
```

```
## Warning in simpleLoess(y, x, w, span, degree = degree, parametric = parametric, : neighborhood radius 0.005
```

```
## Warning in simpleLoess(y, x, w, span, degree = degree, parametric = parametric, : reciprocal condition number 1
```

```
## Warning in simpleLoess(y, x, w, span, degree = degree, parametric = parametric, : There are other near singularities as well. 1.01
```

```
## Warning in simpleLoess(y, x, w, span, degree = degree, parametric = parametric, : zero-width neighborhood. make span bigger
```

```
## Warning: Computation failed in `stat_smooth()`:
## NA/NaN/Inf in foreign function call (arg 5)
```

```
## Warning in simpleLoess(y, x, w, span, degree = degree, parametric = parametric, : at -0.005
```

```
## Warning in simpleLoess(y, x, w, span, degree = degree, parametric = parametric, : radius 2.5e-05
```

```
## Warning in simpleLoess(y, x, w, span, degree = degree, parametric = parametric, : all data on boundary of neighborhood. make span bigger
```

```
## Warning in simpleLoess(y, x, w, span, degree = degree, parametric = parametric, : pseudoinverse used at -0.005
```

```
## Warning in simpleLoess(y, x, w, span, degree = degree, parametric = parametric, : neighborhood radius 0.005
```

```
## Warning in simpleLoess(y, x, w, span, degree = degree, parametric = parametric, : reciprocal condition number 1
```

```
## Warning in simpleLoess(y, x, w, span, degree = degree, parametric = parametric, : There are other near singularities as well. 1.01
```

```
## Warning in simpleLoess(y, x, w, span, degree = degree, parametric = parametric, : zero-width neighborhood. make span bigger
```

```
## Warning: Computation failed in `stat_smooth()`:
## NA/NaN/Inf in foreign function call (arg 5)
```

```
## Warning in simpleLoess(y, x, w, span, degree = degree, parametric = parametric, : at -0.005
```

```
## Warning in simpleLoess(y, x, w, span, degree = degree, parametric = parametric, : radius 2.5e-05
```

```
## Warning in simpleLoess(y, x, w, span, degree = degree, parametric = parametric, : all data on boundary of neighborhood. make span bigger
```

```
## Warning in simpleLoess(y, x, w, span, degree = degree, parametric = parametric, : pseudoinverse used at -0.005
```

```
## Warning in simpleLoess(y, x, w, span, degree = degree, parametric = parametric, : neighborhood radius 0.005
```

```
## Warning in simpleLoess(y, x, w, span, degree = degree, parametric = parametric, : reciprocal condition number 1
```

```
## Warning in simpleLoess(y, x, w, span, degree = degree, parametric = parametric, : There are other near singularities as well. 1.01
```

```
## Warning in simpleLoess(y, x, w, span, degree = degree, parametric = parametric, : zero-width neighborhood. make span bigger
```

```
## Warning: Computation failed in `stat_smooth()`:
## NA/NaN/Inf in foreign function call (arg 5)
```

```
## Warning in simpleLoess(y, x, w, span, degree = degree, parametric = parametric, : at -0.005
```

```
## Warning in simpleLoess(y, x, w, span, degree = degree, parametric = parametric, : radius 2.5e-05
```

```
## Warning in simpleLoess(y, x, w, span, degree = degree, parametric = parametric, : all data on boundary of neighborhood. make span bigger
```

```
## Warning in simpleLoess(y, x, w, span, degree = degree, parametric = parametric, : pseudoinverse used at -0.005
```

```
## Warning in simpleLoess(y, x, w, span, degree = degree, parametric = parametric, : neighborhood radius 0.005
```

```
## Warning in simpleLoess(y, x, w, span, degree = degree, parametric = parametric, : reciprocal condition number 1
```

```
## Warning in simpleLoess(y, x, w, span, degree = degree, parametric = parametric, : There are other near singularities as well. 1.01
```

```
## Warning in simpleLoess(y, x, w, span, degree = degree, parametric = parametric, : zero-width neighborhood. make span bigger
```

```
## Warning: Computation failed in `stat_smooth()`:
## NA/NaN/Inf in foreign function call (arg 5)
```

```
## Warning in simpleLoess(y, x, w, span, degree = degree, parametric = parametric, : at -0.005
```

```
## Warning in simpleLoess(y, x, w, span, degree = degree, parametric = parametric, : radius 2.5e-05
```

```
## Warning in simpleLoess(y, x, w, span, degree = degree, parametric = parametric, : all data on boundary of neighborhood. make span bigger
```

```
## Warning in simpleLoess(y, x, w, span, degree = degree, parametric = parametric, : pseudoinverse used at -0.005
```

```
## Warning in simpleLoess(y, x, w, span, degree = degree, parametric = parametric, : neighborhood radius 0.005
```

```
## Warning in simpleLoess(y, x, w, span, degree = degree, parametric = parametric, : reciprocal condition number 1
```

```
## Warning in simpleLoess(y, x, w, span, degree = degree, parametric = parametric, : There are other near singularities as well. 1.01
```

```
## Warning in simpleLoess(y, x, w, span, degree = degree, parametric = parametric, : zero-width neighborhood. make span bigger
```

```
## Warning: Computation failed in `stat_smooth()`:
## NA/NaN/Inf in foreign function call (arg 5)
```

```
## Warning in simpleLoess(y, x, w, span, degree = degree, parametric = parametric, : pseudoinverse used at -0.005
```

```
## Warning in simpleLoess(y, x, w, span, degree = degree, parametric = parametric, : neighborhood radius 1.005
```

```
## Warning in simpleLoess(y, x, w, span, degree = degree, parametric = parametric, : reciprocal condition number 0
```

```
## Warning in simpleLoess(y, x, w, span, degree = degree, parametric = parametric, : There are other near singularities as well. 1.01
```

```
## Warning in predLoess(object$y, object$x, newx = if (is.null(newdata)) object$x else if (is.data.frame(newdata)) as.matrix(model.frame(delete.response(terms(object)), : pseudoinverse used at -0.005
```

```
## Warning in predLoess(object$y, object$x, newx = if (is.null(newdata)) object$x else if (is.data.frame(newdata)) as.matrix(model.frame(delete.response(terms(object)), : neighborhood radius 1.005
```

```
## Warning in predLoess(object$y, object$x, newx = if (is.null(newdata)) object$x else if (is.data.frame(newdata)) as.matrix(model.frame(delete.response(terms(object)), : reciprocal condition number 0
```

```
## Warning in predLoess(object$y, object$x, newx = if (is.null(newdata)) object$x else if (is.data.frame(newdata)) as.matrix(model.frame(delete.response(terms(object)), : There are other near
## singularities as well. 1.01
```

```
## Warning in simpleLoess(y, x, w, span, degree = degree, parametric = parametric, : at -0.005
```

```
## Warning in simpleLoess(y, x, w, span, degree = degree, parametric = parametric, : radius 2.5e-05
```

```
## Warning in simpleLoess(y, x, w, span, degree = degree, parametric = parametric, : all data on boundary of neighborhood. make span bigger
```

```
## Warning in simpleLoess(y, x, w, span, degree = degree, parametric = parametric, : pseudoinverse used at -0.005
```

```
## Warning in simpleLoess(y, x, w, span, degree = degree, parametric = parametric, : neighborhood radius 0.005
```

```
## Warning in simpleLoess(y, x, w, span, degree = degree, parametric = parametric, : reciprocal condition number 1
```

```
## Warning in simpleLoess(y, x, w, span, degree = degree, parametric = parametric, : There are other near singularities as well. 1.01
```

```
## Warning in simpleLoess(y, x, w, span, degree = degree, parametric = parametric, : zero-width neighborhood. make span bigger
```

```
## Warning: Computation failed in `stat_smooth()`:
## NA/NaN/Inf in foreign function call (arg 5)
```

```
## Warning in simpleLoess(y, x, w, span, degree = degree, parametric = parametric, : at -0.005
```

```
## Warning in simpleLoess(y, x, w, span, degree = degree, parametric = parametric, : radius 2.5e-05
```

```
## Warning in simpleLoess(y, x, w, span, degree = degree, parametric = parametric, : all data on boundary of neighborhood. make span bigger
```

```
## Warning in simpleLoess(y, x, w, span, degree = degree, parametric = parametric, : pseudoinverse used at -0.005
```

```
## Warning in simpleLoess(y, x, w, span, degree = degree, parametric = parametric, : neighborhood radius 0.005
```

```
## Warning in simpleLoess(y, x, w, span, degree = degree, parametric = parametric, : reciprocal condition number 1
```

```
## Warning in simpleLoess(y, x, w, span, degree = degree, parametric = parametric, : There are other near singularities as well. 1.01
```

```
## Warning in simpleLoess(y, x, w, span, degree = degree, parametric = parametric, : zero-width neighborhood. make span bigger
```

```
## Warning: Computation failed in `stat_smooth()`:
## NA/NaN/Inf in foreign function call (arg 5)
```

```
## Warning in simpleLoess(y, x, w, span, degree = degree, parametric = parametric, : at -0.005
```

```
## Warning in simpleLoess(y, x, w, span, degree = degree, parametric = parametric, : radius 2.5e-05
```

```
## Warning in simpleLoess(y, x, w, span, degree = degree, parametric = parametric, : all data on boundary of neighborhood. make span bigger
```

```
## Warning in simpleLoess(y, x, w, span, degree = degree, parametric = parametric, : pseudoinverse used at -0.005
```

```
## Warning in simpleLoess(y, x, w, span, degree = degree, parametric = parametric, : neighborhood radius 0.005
```

```
## Warning in simpleLoess(y, x, w, span, degree = degree, parametric = parametric, : reciprocal condition number 1
```

```
## Warning in simpleLoess(y, x, w, span, degree = degree, parametric = parametric, : There are other near singularities as well. 1.01
```

```
## Warning in simpleLoess(y, x, w, span, degree = degree, parametric = parametric, : zero-width neighborhood. make span bigger
```

```
## Warning: Computation failed in `stat_smooth()`:
## NA/NaN/Inf in foreign function call (arg 5)
```

```
## Warning in simpleLoess(y, x, w, span, degree = degree, parametric = parametric, : at -0.005
```

```
## Warning in simpleLoess(y, x, w, span, degree = degree, parametric = parametric, : radius 2.5e-05
```

```
## Warning in simpleLoess(y, x, w, span, degree = degree, parametric = parametric, : all data on boundary of neighborhood. make span bigger
```

```
## Warning in simpleLoess(y, x, w, span, degree = degree, parametric = parametric, : pseudoinverse used at -0.005
```

```
## Warning in simpleLoess(y, x, w, span, degree = degree, parametric = parametric, : neighborhood radius 0.005
```

```
## Warning in simpleLoess(y, x, w, span, degree = degree, parametric = parametric, : reciprocal condition number 1
```

```
## Warning in simpleLoess(y, x, w, span, degree = degree, parametric = parametric, : There are other near singularities as well. 1.01
```

```
## Warning in simpleLoess(y, x, w, span, degree = degree, parametric = parametric, : zero-width neighborhood. make span bigger
```

```
## Warning: Computation failed in `stat_smooth()`:
## NA/NaN/Inf in foreign function call (arg 5)
```

```
## Warning in simpleLoess(y, x, w, span, degree = degree, parametric = parametric, : at -0.005
```

```
## Warning in simpleLoess(y, x, w, span, degree = degree, parametric = parametric, : radius 2.5e-05
```

```
## Warning in simpleLoess(y, x, w, span, degree = degree, parametric = parametric, : all data on boundary of neighborhood. make span bigger
```

```
## Warning in simpleLoess(y, x, w, span, degree = degree, parametric = parametric, : pseudoinverse used at -0.005
```

```
## Warning in simpleLoess(y, x, w, span, degree = degree, parametric = parametric, : neighborhood radius 0.005
```

```
## Warning in simpleLoess(y, x, w, span, degree = degree, parametric = parametric, : reciprocal condition number 1
```

```
## Warning in simpleLoess(y, x, w, span, degree = degree, parametric = parametric, : There are other near singularities as well. 1.01
```

```
## Warning in simpleLoess(y, x, w, span, degree = degree, parametric = parametric, : zero-width neighborhood. make span bigger
```

```
## Warning: Computation failed in `stat_smooth()`:
## NA/NaN/Inf in foreign function call (arg 5)
```

```
## Warning in simpleLoess(y, x, w, span, degree = degree, parametric = parametric, : at -0.005
```

```
## Warning in simpleLoess(y, x, w, span, degree = degree, parametric = parametric, : radius 2.5e-05
```

```
## Warning in simpleLoess(y, x, w, span, degree = degree, parametric = parametric, : all data on boundary of neighborhood. make span bigger
```

```
## Warning in simpleLoess(y, x, w, span, degree = degree, parametric = parametric, : pseudoinverse used at -0.005
```

```
## Warning in simpleLoess(y, x, w, span, degree = degree, parametric = parametric, : neighborhood radius 0.005
```

```
## Warning in simpleLoess(y, x, w, span, degree = degree, parametric = parametric, : reciprocal condition number 1
```

```
## Warning in simpleLoess(y, x, w, span, degree = degree, parametric = parametric, : There are other near singularities as well. 1.01
```

```
## Warning in simpleLoess(y, x, w, span, degree = degree, parametric = parametric, : zero-width neighborhood. make span bigger
```

```
## Warning: Computation failed in `stat_smooth()`:
## NA/NaN/Inf in foreign function call (arg 5)
```

```
## Warning in simpleLoess(y, x, w, span, degree = degree, parametric = parametric, : pseudoinverse used at -0.005
```

```
## Warning in simpleLoess(y, x, w, span, degree = degree, parametric = parametric, : neighborhood radius 1.005
```

```
## Warning in simpleLoess(y, x, w, span, degree = degree, parametric = parametric, : reciprocal condition number 0
```

```
## Warning in simpleLoess(y, x, w, span, degree = degree, parametric = parametric, : There are other near singularities as well. 1.01
```

```
## Warning in predLoess(object$y, object$x, newx = if (is.null(newdata)) object$x else if (is.data.frame(newdata)) as.matrix(model.frame(delete.response(terms(object)), : pseudoinverse used at -0.005
```

```
## Warning in predLoess(object$y, object$x, newx = if (is.null(newdata)) object$x else if (is.data.frame(newdata)) as.matrix(model.frame(delete.response(terms(object)), : neighborhood radius 1.005
```

```
## Warning in predLoess(object$y, object$x, newx = if (is.null(newdata)) object$x else if (is.data.frame(newdata)) as.matrix(model.frame(delete.response(terms(object)), : reciprocal condition number 0
```

```
## Warning in predLoess(object$y, object$x, newx = if (is.null(newdata)) object$x else if (is.data.frame(newdata)) as.matrix(model.frame(delete.response(terms(object)), : There are other near
## singularities as well. 1.01
```

```
## Warning in simpleLoess(y, x, w, span, degree = degree, parametric = parametric, : pseudoinverse used at -0.005
```

```
## Warning in simpleLoess(y, x, w, span, degree = degree, parametric = parametric, : neighborhood radius 1.005
```

```
## Warning in simpleLoess(y, x, w, span, degree = degree, parametric = parametric, : reciprocal condition number 0
```

```
## Warning in simpleLoess(y, x, w, span, degree = degree, parametric = parametric, : There are other near singularities as well. 1.01
```

```
## Warning in predLoess(object$y, object$x, newx = if (is.null(newdata)) object$x else if (is.data.frame(newdata)) as.matrix(model.frame(delete.response(terms(object)), : pseudoinverse used at -0.005
```

```
## Warning in predLoess(object$y, object$x, newx = if (is.null(newdata)) object$x else if (is.data.frame(newdata)) as.matrix(model.frame(delete.response(terms(object)), : neighborhood radius 1.005
```

```
## Warning in predLoess(object$y, object$x, newx = if (is.null(newdata)) object$x else if (is.data.frame(newdata)) as.matrix(model.frame(delete.response(terms(object)), : reciprocal condition number 0
```

```
## Warning in predLoess(object$y, object$x, newx = if (is.null(newdata)) object$x else if (is.data.frame(newdata)) as.matrix(model.frame(delete.response(terms(object)), : There are other near
## singularities as well. 1.01
```

```
## Warning in simpleLoess(y, x, w, span, degree = degree, parametric = parametric, : at -0.005
```

```
## Warning in simpleLoess(y, x, w, span, degree = degree, parametric = parametric, : radius 2.5e-05
```

```
## Warning in simpleLoess(y, x, w, span, degree = degree, parametric = parametric, : all data on boundary of neighborhood. make span bigger
```

```
## Warning in simpleLoess(y, x, w, span, degree = degree, parametric = parametric, : pseudoinverse used at -0.005
```

```
## Warning in simpleLoess(y, x, w, span, degree = degree, parametric = parametric, : neighborhood radius 0.005
```

```
## Warning in simpleLoess(y, x, w, span, degree = degree, parametric = parametric, : reciprocal condition number 1
```

```
## Warning in simpleLoess(y, x, w, span, degree = degree, parametric = parametric, : There are other near singularities as well. 1.01
```

```
## Warning in simpleLoess(y, x, w, span, degree = degree, parametric = parametric, : zero-width neighborhood. make span bigger
```

```
## Warning: Computation failed in `stat_smooth()`:
## NA/NaN/Inf in foreign function call (arg 5)
```

```
## Warning in simpleLoess(y, x, w, span, degree = degree, parametric = parametric, : at -0.005
```

```
## Warning in simpleLoess(y, x, w, span, degree = degree, parametric = parametric, : radius 2.5e-05
```

```
## Warning in simpleLoess(y, x, w, span, degree = degree, parametric = parametric, : all data on boundary of neighborhood. make span bigger
```

```
## Warning in simpleLoess(y, x, w, span, degree = degree, parametric = parametric, : pseudoinverse used at -0.005
```

```
## Warning in simpleLoess(y, x, w, span, degree = degree, parametric = parametric, : neighborhood radius 0.005
```

```
## Warning in simpleLoess(y, x, w, span, degree = degree, parametric = parametric, : reciprocal condition number 1
```

```
## Warning in simpleLoess(y, x, w, span, degree = degree, parametric = parametric, : There are other near singularities as well. 1.01
```

```
## Warning in simpleLoess(y, x, w, span, degree = degree, parametric = parametric, : zero-width neighborhood. make span bigger
```

```
## Warning: Computation failed in `stat_smooth()`:
## NA/NaN/Inf in foreign function call (arg 5)
```

```
## Warning in simpleLoess(y, x, w, span, degree = degree, parametric = parametric, : at -0.005
```

```
## Warning in simpleLoess(y, x, w, span, degree = degree, parametric = parametric, : radius 2.5e-05
```

```
## Warning in simpleLoess(y, x, w, span, degree = degree, parametric = parametric, : all data on boundary of neighborhood. make span bigger
```

```
## Warning in simpleLoess(y, x, w, span, degree = degree, parametric = parametric, : pseudoinverse used at -0.005
```

```
## Warning in simpleLoess(y, x, w, span, degree = degree, parametric = parametric, : neighborhood radius 0.005
```

```
## Warning in simpleLoess(y, x, w, span, degree = degree, parametric = parametric, : reciprocal condition number 1
```

```
## Warning in simpleLoess(y, x, w, span, degree = degree, parametric = parametric, : There are other near singularities as well. 1.01
```

```
## Warning in simpleLoess(y, x, w, span, degree = degree, parametric = parametric, : zero-width neighborhood. make span bigger
```

```
## Warning: Computation failed in `stat_smooth()`:
## NA/NaN/Inf in foreign function call (arg 5)
```

```
## Warning in simpleLoess(y, x, w, span, degree = degree, parametric = parametric, : at -0.005
```

```
## Warning in simpleLoess(y, x, w, span, degree = degree, parametric = parametric, : radius 2.5e-05
```

```
## Warning in simpleLoess(y, x, w, span, degree = degree, parametric = parametric, : all data on boundary of neighborhood. make span bigger
```

```
## Warning in simpleLoess(y, x, w, span, degree = degree, parametric = parametric, : pseudoinverse used at -0.005
```

```
## Warning in simpleLoess(y, x, w, span, degree = degree, parametric = parametric, : neighborhood radius 0.005
```

```
## Warning in simpleLoess(y, x, w, span, degree = degree, parametric = parametric, : reciprocal condition number 1
```

```
## Warning in simpleLoess(y, x, w, span, degree = degree, parametric = parametric, : There are other near singularities as well. 1.01
```

```
## Warning in simpleLoess(y, x, w, span, degree = degree, parametric = parametric, : zero-width neighborhood. make span bigger
```

```
## Warning: Computation failed in `stat_smooth()`:
## NA/NaN/Inf in foreign function call (arg 5)
```

```
## Warning in simpleLoess(y, x, w, span, degree = degree, parametric = parametric, : at -0.005
```

```
## Warning in simpleLoess(y, x, w, span, degree = degree, parametric = parametric, : radius 2.5e-05
```

```
## Warning in simpleLoess(y, x, w, span, degree = degree, parametric = parametric, : all data on boundary of neighborhood. make span bigger
```

```
## Warning in simpleLoess(y, x, w, span, degree = degree, parametric = parametric, : pseudoinverse used at -0.005
```

```
## Warning in simpleLoess(y, x, w, span, degree = degree, parametric = parametric, : neighborhood radius 0.005
```

```
## Warning in simpleLoess(y, x, w, span, degree = degree, parametric = parametric, : reciprocal condition number 1
```

```
## Warning in simpleLoess(y, x, w, span, degree = degree, parametric = parametric, : There are other near singularities as well. 1.01
```

```
## Warning in simpleLoess(y, x, w, span, degree = degree, parametric = parametric, : zero-width neighborhood. make span bigger
```

```
## Warning: Computation failed in `stat_smooth()`:
## NA/NaN/Inf in foreign function call (arg 5)
```

```
## Warning in simpleLoess(y, x, w, span, degree = degree, parametric = parametric, : at -0.005
```

```
## Warning in simpleLoess(y, x, w, span, degree = degree, parametric = parametric, : radius 2.5e-05
```

```
## Warning in simpleLoess(y, x, w, span, degree = degree, parametric = parametric, : all data on boundary of neighborhood. make span bigger
```

```
## Warning in simpleLoess(y, x, w, span, degree = degree, parametric = parametric, : pseudoinverse used at -0.005
```

```
## Warning in simpleLoess(y, x, w, span, degree = degree, parametric = parametric, : neighborhood radius 0.005
```

```
## Warning in simpleLoess(y, x, w, span, degree = degree, parametric = parametric, : reciprocal condition number 1
```

```
## Warning in simpleLoess(y, x, w, span, degree = degree, parametric = parametric, : There are other near singularities as well. 1.01
```

```
## Warning in simpleLoess(y, x, w, span, degree = degree, parametric = parametric, : zero-width neighborhood. make span bigger
```

```
## Warning: Computation failed in `stat_smooth()`:
## NA/NaN/Inf in foreign function call (arg 5)
```

```
## Warning in simpleLoess(y, x, w, span, degree = degree, parametric = parametric, : at -0.005
```

```
## Warning in simpleLoess(y, x, w, span, degree = degree, parametric = parametric, : radius 2.5e-05
```

```
## Warning in simpleLoess(y, x, w, span, degree = degree, parametric = parametric, : all data on boundary of neighborhood. make span bigger
```

```
## Warning in simpleLoess(y, x, w, span, degree = degree, parametric = parametric, : pseudoinverse used at -0.005
```

```
## Warning in simpleLoess(y, x, w, span, degree = degree, parametric = parametric, : neighborhood radius 0.005
```

```
## Warning in simpleLoess(y, x, w, span, degree = degree, parametric = parametric, : reciprocal condition number 1
```

```
## Warning in simpleLoess(y, x, w, span, degree = degree, parametric = parametric, : There are other near singularities as well. 1.01
```

```
## Warning in simpleLoess(y, x, w, span, degree = degree, parametric = parametric, : zero-width neighborhood. make span bigger
```

```
## Warning: Computation failed in `stat_smooth()`:
## NA/NaN/Inf in foreign function call (arg 5)
```

```
## Warning in simpleLoess(y, x, w, span, degree = degree, parametric = parametric, : pseudoinverse used at -0.005
```

```
## Warning in simpleLoess(y, x, w, span, degree = degree, parametric = parametric, : neighborhood radius 1.005
```

```
## Warning in simpleLoess(y, x, w, span, degree = degree, parametric = parametric, : reciprocal condition number 0
```

```
## Warning in simpleLoess(y, x, w, span, degree = degree, parametric = parametric, : There are other near singularities as well. 1.01
```

```
## Warning in predLoess(object$y, object$x, newx = if (is.null(newdata)) object$x else if (is.data.frame(newdata)) as.matrix(model.frame(delete.response(terms(object)), : pseudoinverse used at -0.005
```

```
## Warning in predLoess(object$y, object$x, newx = if (is.null(newdata)) object$x else if (is.data.frame(newdata)) as.matrix(model.frame(delete.response(terms(object)), : neighborhood radius 1.005
```

```
## Warning in predLoess(object$y, object$x, newx = if (is.null(newdata)) object$x else if (is.data.frame(newdata)) as.matrix(model.frame(delete.response(terms(object)), : reciprocal condition number 0
```

```
## Warning in predLoess(object$y, object$x, newx = if (is.null(newdata)) object$x else if (is.data.frame(newdata)) as.matrix(model.frame(delete.response(terms(object)), : There are other near
## singularities as well. 1.01
```

```
## Warning in simpleLoess(y, x, w, span, degree = degree, parametric = parametric, : at -0.005
```

```
## Warning in simpleLoess(y, x, w, span, degree = degree, parametric = parametric, : radius 2.5e-05
```

```
## Warning in simpleLoess(y, x, w, span, degree = degree, parametric = parametric, : all data on boundary of neighborhood. make span bigger
```

```
## Warning in simpleLoess(y, x, w, span, degree = degree, parametric = parametric, : pseudoinverse used at -0.005
```

```
## Warning in simpleLoess(y, x, w, span, degree = degree, parametric = parametric, : neighborhood radius 0.005
```

```
## Warning in simpleLoess(y, x, w, span, degree = degree, parametric = parametric, : reciprocal condition number 1
```

```
## Warning in simpleLoess(y, x, w, span, degree = degree, parametric = parametric, : There are other near singularities as well. 1.01
```

```
## Warning in simpleLoess(y, x, w, span, degree = degree, parametric = parametric, : zero-width neighborhood. make span bigger
```

```
## Warning: Computation failed in `stat_smooth()`:
## NA/NaN/Inf in foreign function call (arg 5)
```

```
## Warning in simpleLoess(y, x, w, span, degree = degree, parametric = parametric, : at -0.005
```

```
## Warning in simpleLoess(y, x, w, span, degree = degree, parametric = parametric, : radius 2.5e-05
```

```
## Warning in simpleLoess(y, x, w, span, degree = degree, parametric = parametric, : all data on boundary of neighborhood. make span bigger
```

```
## Warning in simpleLoess(y, x, w, span, degree = degree, parametric = parametric, : pseudoinverse used at -0.005
```

```
## Warning in simpleLoess(y, x, w, span, degree = degree, parametric = parametric, : neighborhood radius 0.005
```

```
## Warning in simpleLoess(y, x, w, span, degree = degree, parametric = parametric, : reciprocal condition number 1
```

```
## Warning in simpleLoess(y, x, w, span, degree = degree, parametric = parametric, : There are other near singularities as well. 1.01
```

```
## Warning in simpleLoess(y, x, w, span, degree = degree, parametric = parametric, : zero-width neighborhood. make span bigger
```

```
## Warning: Computation failed in `stat_smooth()`:
## NA/NaN/Inf in foreign function call (arg 5)
```

```
## Warning in simpleLoess(y, x, w, span, degree = degree, parametric = parametric, : at -0.005
```

```
## Warning in simpleLoess(y, x, w, span, degree = degree, parametric = parametric, : radius 2.5e-05
```

```
## Warning in simpleLoess(y, x, w, span, degree = degree, parametric = parametric, : all data on boundary of neighborhood. make span bigger
```

```
## Warning in simpleLoess(y, x, w, span, degree = degree, parametric = parametric, : pseudoinverse used at -0.005
```

```
## Warning in simpleLoess(y, x, w, span, degree = degree, parametric = parametric, : neighborhood radius 0.005
```

```
## Warning in simpleLoess(y, x, w, span, degree = degree, parametric = parametric, : reciprocal condition number 1
```

```
## Warning in simpleLoess(y, x, w, span, degree = degree, parametric = parametric, : There are other near singularities as well. 1.01
```

```
## Warning in simpleLoess(y, x, w, span, degree = degree, parametric = parametric, : zero-width neighborhood. make span bigger
```

```
## Warning: Computation failed in `stat_smooth()`:
## NA/NaN/Inf in foreign function call (arg 5)
```

```
## Warning in simpleLoess(y, x, w, span, degree = degree, parametric = parametric, : at -0.005
```

```
## Warning in simpleLoess(y, x, w, span, degree = degree, parametric = parametric, : radius 2.5e-05
```

```
## Warning in simpleLoess(y, x, w, span, degree = degree, parametric = parametric, : all data on boundary of neighborhood. make span bigger
```

```
## Warning in simpleLoess(y, x, w, span, degree = degree, parametric = parametric, : pseudoinverse used at -0.005
```

```
## Warning in simpleLoess(y, x, w, span, degree = degree, parametric = parametric, : neighborhood radius 0.005
```

```
## Warning in simpleLoess(y, x, w, span, degree = degree, parametric = parametric, : reciprocal condition number 1
```

```
## Warning in simpleLoess(y, x, w, span, degree = degree, parametric = parametric, : There are other near singularities as well. 1.01
```

```
## Warning in simpleLoess(y, x, w, span, degree = degree, parametric = parametric, : zero-width neighborhood. make span bigger
```

```
## Warning: Computation failed in `stat_smooth()`:
## NA/NaN/Inf in foreign function call (arg 5)
```

```
## Warning in simpleLoess(y, x, w, span, degree = degree, parametric = parametric, : at -0.005
```

```
## Warning in simpleLoess(y, x, w, span, degree = degree, parametric = parametric, : radius 2.5e-05
```

```
## Warning in simpleLoess(y, x, w, span, degree = degree, parametric = parametric, : all data on boundary of neighborhood. make span bigger
```

```
## Warning in simpleLoess(y, x, w, span, degree = degree, parametric = parametric, : pseudoinverse used at -0.005
```

```
## Warning in simpleLoess(y, x, w, span, degree = degree, parametric = parametric, : neighborhood radius 0.005
```

```
## Warning in simpleLoess(y, x, w, span, degree = degree, parametric = parametric, : reciprocal condition number 1
```

```
## Warning in simpleLoess(y, x, w, span, degree = degree, parametric = parametric, : There are other near singularities as well. 1.01
```

```
## Warning in simpleLoess(y, x, w, span, degree = degree, parametric = parametric, : zero-width neighborhood. make span bigger
```

```
## Warning: Computation failed in `stat_smooth()`:
## NA/NaN/Inf in foreign function call (arg 5)
```

```
## Warning in simpleLoess(y, x, w, span, degree = degree, parametric = parametric, : at -0.005
```

```
## Warning in simpleLoess(y, x, w, span, degree = degree, parametric = parametric, : radius 2.5e-05
```

```
## Warning in simpleLoess(y, x, w, span, degree = degree, parametric = parametric, : all data on boundary of neighborhood. make span bigger
```

```
## Warning in simpleLoess(y, x, w, span, degree = degree, parametric = parametric, : pseudoinverse used at -0.005
```

```
## Warning in simpleLoess(y, x, w, span, degree = degree, parametric = parametric, : neighborhood radius 0.005
```

```
## Warning in simpleLoess(y, x, w, span, degree = degree, parametric = parametric, : reciprocal condition number 1
```

```
## Warning in simpleLoess(y, x, w, span, degree = degree, parametric = parametric, : There are other near singularities as well. 1.01
```

```
## Warning in simpleLoess(y, x, w, span, degree = degree, parametric = parametric, : zero-width neighborhood. make span bigger
```

```
## Warning: Computation failed in `stat_smooth()`:
## NA/NaN/Inf in foreign function call (arg 5)
```

```
## Warning in simpleLoess(y, x, w, span, degree = degree, parametric = parametric, : at -0.005
```

```
## Warning in simpleLoess(y, x, w, span, degree = degree, parametric = parametric, : radius 2.5e-05
```

```
## Warning in simpleLoess(y, x, w, span, degree = degree, parametric = parametric, : all data on boundary of neighborhood. make span bigger
```

```
## Warning in simpleLoess(y, x, w, span, degree = degree, parametric = parametric, : pseudoinverse used at -0.005
```

```
## Warning in simpleLoess(y, x, w, span, degree = degree, parametric = parametric, : neighborhood radius 0.005
```

```
## Warning in simpleLoess(y, x, w, span, degree = degree, parametric = parametric, : reciprocal condition number 1
```

```
## Warning in simpleLoess(y, x, w, span, degree = degree, parametric = parametric, : There are other near singularities as well. 1.01
```

```
## Warning in simpleLoess(y, x, w, span, degree = degree, parametric = parametric, : zero-width neighborhood. make span bigger
```

```
## Warning: Computation failed in `stat_smooth()`:
## NA/NaN/Inf in foreign function call (arg 5)
```

```
## Warning in simpleLoess(y, x, w, span, degree = degree, parametric = parametric, : at -0.005
```

```
## Warning in simpleLoess(y, x, w, span, degree = degree, parametric = parametric, : radius 2.5e-05
```

```
## Warning in simpleLoess(y, x, w, span, degree = degree, parametric = parametric, : all data on boundary of neighborhood. make span bigger
```

```
## Warning in simpleLoess(y, x, w, span, degree = degree, parametric = parametric, : pseudoinverse used at -0.005
```

```
## Warning in simpleLoess(y, x, w, span, degree = degree, parametric = parametric, : neighborhood radius 0.005
```

```
## Warning in simpleLoess(y, x, w, span, degree = degree, parametric = parametric, : reciprocal condition number 1
```

```
## Warning in simpleLoess(y, x, w, span, degree = degree, parametric = parametric, : There are other near singularities as well. 1.01
```

```
## Warning in simpleLoess(y, x, w, span, degree = degree, parametric = parametric, : zero-width neighborhood. make span bigger
```

```
## Warning: Computation failed in `stat_smooth()`:
## NA/NaN/Inf in foreign function call (arg 5)
```

```
## Warning in simpleLoess(y, x, w, span, degree = degree, parametric = parametric, : at -0.005
```

```
## Warning in simpleLoess(y, x, w, span, degree = degree, parametric = parametric, : radius 2.5e-05
```

```
## Warning in simpleLoess(y, x, w, span, degree = degree, parametric = parametric, : all data on boundary of neighborhood. make span bigger
```

```
## Warning in simpleLoess(y, x, w, span, degree = degree, parametric = parametric, : pseudoinverse used at -0.005
```

```
## Warning in simpleLoess(y, x, w, span, degree = degree, parametric = parametric, : neighborhood radius 0.005
```

```
## Warning in simpleLoess(y, x, w, span, degree = degree, parametric = parametric, : reciprocal condition number 1
```

```
## Warning in simpleLoess(y, x, w, span, degree = degree, parametric = parametric, : There are other near singularities as well. 1.01
```

```
## Warning in simpleLoess(y, x, w, span, degree = degree, parametric = parametric, : zero-width neighborhood. make span bigger
```

```
## Warning: Computation failed in `stat_smooth()`:
## NA/NaN/Inf in foreign function call (arg 5)
```

```
## Warning in simpleLoess(y, x, w, span, degree = degree, parametric = parametric, : at -0.005
```

```
## Warning in simpleLoess(y, x, w, span, degree = degree, parametric = parametric, : radius 2.5e-05
```

```
## Warning in simpleLoess(y, x, w, span, degree = degree, parametric = parametric, : all data on boundary of neighborhood. make span bigger
```

```
## Warning in simpleLoess(y, x, w, span, degree = degree, parametric = parametric, : pseudoinverse used at -0.005
```

```
## Warning in simpleLoess(y, x, w, span, degree = degree, parametric = parametric, : neighborhood radius 0.005
```

```
## Warning in simpleLoess(y, x, w, span, degree = degree, parametric = parametric, : reciprocal condition number 1
```

```
## Warning in simpleLoess(y, x, w, span, degree = degree, parametric = parametric, : There are other near singularities as well. 1.01
```

```
## Warning in simpleLoess(y, x, w, span, degree = degree, parametric = parametric, : zero-width neighborhood. make span bigger
```

```
## Warning: Computation failed in `stat_smooth()`:
## NA/NaN/Inf in foreign function call (arg 5)
```

```
## Warning in simpleLoess(y, x, w, span, degree = degree, parametric = parametric, : pseudoinverse used at -0.005
```

```
## Warning in simpleLoess(y, x, w, span, degree = degree, parametric = parametric, : neighborhood radius 1.005
```

```
## Warning in simpleLoess(y, x, w, span, degree = degree, parametric = parametric, : reciprocal condition number 0
```

```
## Warning in simpleLoess(y, x, w, span, degree = degree, parametric = parametric, : There are other near singularities as well. 1.01
```

```
## Warning in predLoess(object$y, object$x, newx = if (is.null(newdata)) object$x else if (is.data.frame(newdata)) as.matrix(model.frame(delete.response(terms(object)), : pseudoinverse used at -0.005
```

```
## Warning in predLoess(object$y, object$x, newx = if (is.null(newdata)) object$x else if (is.data.frame(newdata)) as.matrix(model.frame(delete.response(terms(object)), : neighborhood radius 1.005
```

```
## Warning in predLoess(object$y, object$x, newx = if (is.null(newdata)) object$x else if (is.data.frame(newdata)) as.matrix(model.frame(delete.response(terms(object)), : reciprocal condition number 0
```

```
## Warning in predLoess(object$y, object$x, newx = if (is.null(newdata)) object$x else if (is.data.frame(newdata)) as.matrix(model.frame(delete.response(terms(object)), : There are other near
## singularities as well. 1.01
```

```
## Warning in simpleLoess(y, x, w, span, degree = degree, parametric = parametric, : at -0.005
```

```
## Warning in simpleLoess(y, x, w, span, degree = degree, parametric = parametric, : radius 2.5e-05
```

```
## Warning in simpleLoess(y, x, w, span, degree = degree, parametric = parametric, : all data on boundary of neighborhood. make span bigger
```

```
## Warning in simpleLoess(y, x, w, span, degree = degree, parametric = parametric, : pseudoinverse used at -0.005
```

```
## Warning in simpleLoess(y, x, w, span, degree = degree, parametric = parametric, : neighborhood radius 0.005
```

```
## Warning in simpleLoess(y, x, w, span, degree = degree, parametric = parametric, : reciprocal condition number 1
```

```
## Warning in simpleLoess(y, x, w, span, degree = degree, parametric = parametric, : There are other near singularities as well. 1.01
```

```
## Warning in simpleLoess(y, x, w, span, degree = degree, parametric = parametric, : zero-width neighborhood. make span bigger
```

```
## Warning: Computation failed in `stat_smooth()`:
## NA/NaN/Inf in foreign function call (arg 5)
```

```
## Warning in simpleLoess(y, x, w, span, degree = degree, parametric = parametric, : at -0.005
```

```
## Warning in simpleLoess(y, x, w, span, degree = degree, parametric = parametric, : radius 2.5e-05
```

```
## Warning in simpleLoess(y, x, w, span, degree = degree, parametric = parametric, : all data on boundary of neighborhood. make span bigger
```

```
## Warning in simpleLoess(y, x, w, span, degree = degree, parametric = parametric, : pseudoinverse used at -0.005
```

```
## Warning in simpleLoess(y, x, w, span, degree = degree, parametric = parametric, : neighborhood radius 0.005
```

```
## Warning in simpleLoess(y, x, w, span, degree = degree, parametric = parametric, : reciprocal condition number 1
```

```
## Warning in simpleLoess(y, x, w, span, degree = degree, parametric = parametric, : There are other near singularities as well. 1.01
```

```
## Warning in simpleLoess(y, x, w, span, degree = degree, parametric = parametric, : zero-width neighborhood. make span bigger
```

```
## Warning: Computation failed in `stat_smooth()`:
## NA/NaN/Inf in foreign function call (arg 5)
```

```
## Warning in simpleLoess(y, x, w, span, degree = degree, parametric = parametric, : at -0.005
```

```
## Warning in simpleLoess(y, x, w, span, degree = degree, parametric = parametric, : radius 2.5e-05
```

```
## Warning in simpleLoess(y, x, w, span, degree = degree, parametric = parametric, : all data on boundary of neighborhood. make span bigger
```

```
## Warning in simpleLoess(y, x, w, span, degree = degree, parametric = parametric, : pseudoinverse used at -0.005
```

```
## Warning in simpleLoess(y, x, w, span, degree = degree, parametric = parametric, : neighborhood radius 0.005
```

```
## Warning in simpleLoess(y, x, w, span, degree = degree, parametric = parametric, : reciprocal condition number 1
```

```
## Warning in simpleLoess(y, x, w, span, degree = degree, parametric = parametric, : There are other near singularities as well. 1.01
```

```
## Warning in simpleLoess(y, x, w, span, degree = degree, parametric = parametric, : zero-width neighborhood. make span bigger
```

```
## Warning: Computation failed in `stat_smooth()`:
## NA/NaN/Inf in foreign function call (arg 5)
```

```
## Warning in simpleLoess(y, x, w, span, degree = degree, parametric = parametric, : at -0.005
```

```
## Warning in simpleLoess(y, x, w, span, degree = degree, parametric = parametric, : radius 2.5e-05
```

```
## Warning in simpleLoess(y, x, w, span, degree = degree, parametric = parametric, : all data on boundary of neighborhood. make span bigger
```

```
## Warning in simpleLoess(y, x, w, span, degree = degree, parametric = parametric, : pseudoinverse used at -0.005
```

```
## Warning in simpleLoess(y, x, w, span, degree = degree, parametric = parametric, : neighborhood radius 0.005
```

```
## Warning in simpleLoess(y, x, w, span, degree = degree, parametric = parametric, : reciprocal condition number 1
```

```
## Warning in simpleLoess(y, x, w, span, degree = degree, parametric = parametric, : There are other near singularities as well. 1.01
```

```
## Warning in simpleLoess(y, x, w, span, degree = degree, parametric = parametric, : zero-width neighborhood. make span bigger
```

```
## Warning: Computation failed in `stat_smooth()`:
## NA/NaN/Inf in foreign function call (arg 5)
```

```
## Warning in simpleLoess(y, x, w, span, degree = degree, parametric = parametric, : at -0.005
```

```
## Warning in simpleLoess(y, x, w, span, degree = degree, parametric = parametric, : radius 2.5e-05
```

```
## Warning in simpleLoess(y, x, w, span, degree = degree, parametric = parametric, : all data on boundary of neighborhood. make span bigger
```

```
## Warning in simpleLoess(y, x, w, span, degree = degree, parametric = parametric, : pseudoinverse used at -0.005
```

```
## Warning in simpleLoess(y, x, w, span, degree = degree, parametric = parametric, : neighborhood radius 0.005
```

```
## Warning in simpleLoess(y, x, w, span, degree = degree, parametric = parametric, : reciprocal condition number 1
```

```
## Warning in simpleLoess(y, x, w, span, degree = degree, parametric = parametric, : There are other near singularities as well. 1.01
```

```
## Warning in simpleLoess(y, x, w, span, degree = degree, parametric = parametric, : zero-width neighborhood. make span bigger
```

```
## Warning: Computation failed in `stat_smooth()`:
## NA/NaN/Inf in foreign function call (arg 5)
```

```
## Warning in simpleLoess(y, x, w, span, degree = degree, parametric = parametric, : pseudoinverse used at -0.005
```

```
## Warning in simpleLoess(y, x, w, span, degree = degree, parametric = parametric, : neighborhood radius 1.005
```

```
## Warning in simpleLoess(y, x, w, span, degree = degree, parametric = parametric, : reciprocal condition number 0
```

```
## Warning in simpleLoess(y, x, w, span, degree = degree, parametric = parametric, : There are other near singularities as well. 1.01
```

```
## Warning in predLoess(object$y, object$x, newx = if (is.null(newdata)) object$x else if (is.data.frame(newdata)) as.matrix(model.frame(delete.response(terms(object)), : pseudoinverse used at -0.005
```

```
## Warning in predLoess(object$y, object$x, newx = if (is.null(newdata)) object$x else if (is.data.frame(newdata)) as.matrix(model.frame(delete.response(terms(object)), : neighborhood radius 1.005
```

```
## Warning in predLoess(object$y, object$x, newx = if (is.null(newdata)) object$x else if (is.data.frame(newdata)) as.matrix(model.frame(delete.response(terms(object)), : reciprocal condition number 0
```

```
## Warning in predLoess(object$y, object$x, newx = if (is.null(newdata)) object$x else if (is.data.frame(newdata)) as.matrix(model.frame(delete.response(terms(object)), : There are other near
## singularities as well. 1.01
```

```
## Warning in simpleLoess(y, x, w, span, degree = degree, parametric = parametric, : at -0.005
```

```
## Warning in simpleLoess(y, x, w, span, degree = degree, parametric = parametric, : radius 2.5e-05
```

```
## Warning in simpleLoess(y, x, w, span, degree = degree, parametric = parametric, : all data on boundary of neighborhood. make span bigger
```

```
## Warning in simpleLoess(y, x, w, span, degree = degree, parametric = parametric, : pseudoinverse used at -0.005
```

```
## Warning in simpleLoess(y, x, w, span, degree = degree, parametric = parametric, : neighborhood radius 0.005
```

```
## Warning in simpleLoess(y, x, w, span, degree = degree, parametric = parametric, : reciprocal condition number 1
```

```
## Warning in simpleLoess(y, x, w, span, degree = degree, parametric = parametric, : There are other near singularities as well. 1.01
```

```
## Warning in simpleLoess(y, x, w, span, degree = degree, parametric = parametric, : zero-width neighborhood. make span bigger
```

```
## Warning: Computation failed in `stat_smooth()`:
## NA/NaN/Inf in foreign function call (arg 5)
```

```
## Warning in simpleLoess(y, x, w, span, degree = degree, parametric = parametric, : at -0.005
```

```
## Warning in simpleLoess(y, x, w, span, degree = degree, parametric = parametric, : radius 2.5e-05
```

```
## Warning in simpleLoess(y, x, w, span, degree = degree, parametric = parametric, : all data on boundary of neighborhood. make span bigger
```

```
## Warning in simpleLoess(y, x, w, span, degree = degree, parametric = parametric, : pseudoinverse used at -0.005
```

```
## Warning in simpleLoess(y, x, w, span, degree = degree, parametric = parametric, : neighborhood radius 0.005
```

```
## Warning in simpleLoess(y, x, w, span, degree = degree, parametric = parametric, : reciprocal condition number 1
```

```
## Warning in simpleLoess(y, x, w, span, degree = degree, parametric = parametric, : There are other near singularities as well. 1.01
```

```
## Warning in simpleLoess(y, x, w, span, degree = degree, parametric = parametric, : zero-width neighborhood. make span bigger
```

```
## Warning: Computation failed in `stat_smooth()`:
## NA/NaN/Inf in foreign function call (arg 5)
```

```
## Warning in simpleLoess(y, x, w, span, degree = degree, parametric = parametric, : at -0.005
```

```
## Warning in simpleLoess(y, x, w, span, degree = degree, parametric = parametric, : radius 2.5e-05
```

```
## Warning in simpleLoess(y, x, w, span, degree = degree, parametric = parametric, : all data on boundary of neighborhood. make span bigger
```

```
## Warning in simpleLoess(y, x, w, span, degree = degree, parametric = parametric, : pseudoinverse used at -0.005
```

```
## Warning in simpleLoess(y, x, w, span, degree = degree, parametric = parametric, : neighborhood radius 0.005
```

```
## Warning in simpleLoess(y, x, w, span, degree = degree, parametric = parametric, : reciprocal condition number 1
```

```
## Warning in simpleLoess(y, x, w, span, degree = degree, parametric = parametric, : There are other near singularities as well. 1.01
```

```
## Warning in simpleLoess(y, x, w, span, degree = degree, parametric = parametric, : zero-width neighborhood. make span bigger
```

```
## Warning: Computation failed in `stat_smooth()`:
## NA/NaN/Inf in foreign function call (arg 5)
```

```
## Warning in simpleLoess(y, x, w, span, degree = degree, parametric = parametric, : at -0.005
```

```
## Warning in simpleLoess(y, x, w, span, degree = degree, parametric = parametric, : radius 2.5e-05
```

```
## Warning in simpleLoess(y, x, w, span, degree = degree, parametric = parametric, : all data on boundary of neighborhood. make span bigger
```

```
## Warning in simpleLoess(y, x, w, span, degree = degree, parametric = parametric, : pseudoinverse used at -0.005
```

```
## Warning in simpleLoess(y, x, w, span, degree = degree, parametric = parametric, : neighborhood radius 0.005
```

```
## Warning in simpleLoess(y, x, w, span, degree = degree, parametric = parametric, : reciprocal condition number 1
```

```
## Warning in simpleLoess(y, x, w, span, degree = degree, parametric = parametric, : There are other near singularities as well. 1.01
```

```
## Warning in simpleLoess(y, x, w, span, degree = degree, parametric = parametric, : zero-width neighborhood. make span bigger
```

```
## Warning: Computation failed in `stat_smooth()`:
## NA/NaN/Inf in foreign function call (arg 5)
```

```
## Warning in simpleLoess(y, x, w, span, degree = degree, parametric = parametric, : pseudoinverse used at -0.005
```

```
## Warning in simpleLoess(y, x, w, span, degree = degree, parametric = parametric, : neighborhood radius 1.005
```

```
## Warning in simpleLoess(y, x, w, span, degree = degree, parametric = parametric, : reciprocal condition number 0
```

```
## Warning in simpleLoess(y, x, w, span, degree = degree, parametric = parametric, : There are other near singularities as well. 1.01
```

```
## Warning in predLoess(object$y, object$x, newx = if (is.null(newdata)) object$x else if (is.data.frame(newdata)) as.matrix(model.frame(delete.response(terms(object)), : pseudoinverse used at -0.005
```

```
## Warning in predLoess(object$y, object$x, newx = if (is.null(newdata)) object$x else if (is.data.frame(newdata)) as.matrix(model.frame(delete.response(terms(object)), : neighborhood radius 1.005
```

```
## Warning in predLoess(object$y, object$x, newx = if (is.null(newdata)) object$x else if (is.data.frame(newdata)) as.matrix(model.frame(delete.response(terms(object)), : reciprocal condition number 0
```

```
## Warning in predLoess(object$y, object$x, newx = if (is.null(newdata)) object$x else if (is.data.frame(newdata)) as.matrix(model.frame(delete.response(terms(object)), : There are other near
## singularities as well. 1.01
```

```
## Warning in simpleLoess(y, x, w, span, degree = degree, parametric = parametric, : at -0.005
```

```
## Warning in simpleLoess(y, x, w, span, degree = degree, parametric = parametric, : radius 2.5e-05
```

```
## Warning in simpleLoess(y, x, w, span, degree = degree, parametric = parametric, : all data on boundary of neighborhood. make span bigger
```

```
## Warning in simpleLoess(y, x, w, span, degree = degree, parametric = parametric, : pseudoinverse used at -0.005
```

```
## Warning in simpleLoess(y, x, w, span, degree = degree, parametric = parametric, : neighborhood radius 0.005
```

```
## Warning in simpleLoess(y, x, w, span, degree = degree, parametric = parametric, : reciprocal condition number 1
```

```
## Warning in simpleLoess(y, x, w, span, degree = degree, parametric = parametric, : There are other near singularities as well. 1.01
```

```
## Warning in simpleLoess(y, x, w, span, degree = degree, parametric = parametric, : zero-width neighborhood. make span bigger
```

```
## Warning: Computation failed in `stat_smooth()`:
## NA/NaN/Inf in foreign function call (arg 5)
```

```
## Warning in simpleLoess(y, x, w, span, degree = degree, parametric = parametric, : at -0.005
```

```
## Warning in simpleLoess(y, x, w, span, degree = degree, parametric = parametric, : radius 2.5e-05
```

```
## Warning in simpleLoess(y, x, w, span, degree = degree, parametric = parametric, : all data on boundary of neighborhood. make span bigger
```

```
## Warning in simpleLoess(y, x, w, span, degree = degree, parametric = parametric, : pseudoinverse used at -0.005
```

```
## Warning in simpleLoess(y, x, w, span, degree = degree, parametric = parametric, : neighborhood radius 0.005
```

```
## Warning in simpleLoess(y, x, w, span, degree = degree, parametric = parametric, : reciprocal condition number 1
```

```
## Warning in simpleLoess(y, x, w, span, degree = degree, parametric = parametric, : There are other near singularities as well. 1.01
```

```
## Warning in simpleLoess(y, x, w, span, degree = degree, parametric = parametric, : zero-width neighborhood. make span bigger
```

```
## Warning: Computation failed in `stat_smooth()`:
## NA/NaN/Inf in foreign function call (arg 5)
```

```
## Warning in simpleLoess(y, x, w, span, degree = degree, parametric = parametric, : at -0.005
```

```
## Warning in simpleLoess(y, x, w, span, degree = degree, parametric = parametric, : radius 2.5e-05
```

```
## Warning in simpleLoess(y, x, w, span, degree = degree, parametric = parametric, : all data on boundary of neighborhood. make span bigger
```

```
## Warning in simpleLoess(y, x, w, span, degree = degree, parametric = parametric, : pseudoinverse used at -0.005
```

```
## Warning in simpleLoess(y, x, w, span, degree = degree, parametric = parametric, : neighborhood radius 0.005
```

```
## Warning in simpleLoess(y, x, w, span, degree = degree, parametric = parametric, : reciprocal condition number 1
```

```
## Warning in simpleLoess(y, x, w, span, degree = degree, parametric = parametric, : There are other near singularities as well. 1.01
```

```
## Warning in simpleLoess(y, x, w, span, degree = degree, parametric = parametric, : zero-width neighborhood. make span bigger
```

```
## Warning: Computation failed in `stat_smooth()`:
## NA/NaN/Inf in foreign function call (arg 5)
```

```
## Warning in simpleLoess(y, x, w, span, degree = degree, parametric = parametric, : at -0.005
```

```
## Warning in simpleLoess(y, x, w, span, degree = degree, parametric = parametric, : radius 2.5e-05
```

```
## Warning in simpleLoess(y, x, w, span, degree = degree, parametric = parametric, : all data on boundary of neighborhood. make span bigger
```

```
## Warning in simpleLoess(y, x, w, span, degree = degree, parametric = parametric, : pseudoinverse used at -0.005
```

```
## Warning in simpleLoess(y, x, w, span, degree = degree, parametric = parametric, : neighborhood radius 0.005
```

```
## Warning in simpleLoess(y, x, w, span, degree = degree, parametric = parametric, : reciprocal condition number 1
```

```
## Warning in simpleLoess(y, x, w, span, degree = degree, parametric = parametric, : There are other near singularities as well. 1.01
```

```
## Warning in simpleLoess(y, x, w, span, degree = degree, parametric = parametric, : zero-width neighborhood. make span bigger
```

```
## Warning: Computation failed in `stat_smooth()`:
## NA/NaN/Inf in foreign function call (arg 5)
```

```
## Warning in simpleLoess(y, x, w, span, degree = degree, parametric = parametric, : at -0.005
```

```
## Warning in simpleLoess(y, x, w, span, degree = degree, parametric = parametric, : radius 2.5e-05
```

```
## Warning in simpleLoess(y, x, w, span, degree = degree, parametric = parametric, : all data on boundary of neighborhood. make span bigger
```

```
## Warning in simpleLoess(y, x, w, span, degree = degree, parametric = parametric, : pseudoinverse used at -0.005
```

```
## Warning in simpleLoess(y, x, w, span, degree = degree, parametric = parametric, : neighborhood radius 0.005
```

```
## Warning in simpleLoess(y, x, w, span, degree = degree, parametric = parametric, : reciprocal condition number 1
```

```
## Warning in simpleLoess(y, x, w, span, degree = degree, parametric = parametric, : There are other near singularities as well. 1.01
```

```
## Warning in simpleLoess(y, x, w, span, degree = degree, parametric = parametric, : zero-width neighborhood. make span bigger
```

```
## Warning: Computation failed in `stat_smooth()`:
## NA/NaN/Inf in foreign function call (arg 5)
```

```
## Warning in simpleLoess(y, x, w, span, degree = degree, parametric = parametric, : at -0.005
```

```
## Warning in simpleLoess(y, x, w, span, degree = degree, parametric = parametric, : radius 2.5e-05
```

```
## Warning in simpleLoess(y, x, w, span, degree = degree, parametric = parametric, : all data on boundary of neighborhood. make span bigger
```

```
## Warning in simpleLoess(y, x, w, span, degree = degree, parametric = parametric, : pseudoinverse used at -0.005
```

```
## Warning in simpleLoess(y, x, w, span, degree = degree, parametric = parametric, : neighborhood radius 0.005
```

```
## Warning in simpleLoess(y, x, w, span, degree = degree, parametric = parametric, : reciprocal condition number 1
```

```
## Warning in simpleLoess(y, x, w, span, degree = degree, parametric = parametric, : There are other near singularities as well. 1.01
```

```
## Warning in simpleLoess(y, x, w, span, degree = degree, parametric = parametric, : zero-width neighborhood. make span bigger
```

```
## Warning: Computation failed in `stat_smooth()`:
## NA/NaN/Inf in foreign function call (arg 5)
```

```
## Warning in simpleLoess(y, x, w, span, degree = degree, parametric = parametric, : at -0.005
```

```
## Warning in simpleLoess(y, x, w, span, degree = degree, parametric = parametric, : radius 2.5e-05
```

```
## Warning in simpleLoess(y, x, w, span, degree = degree, parametric = parametric, : all data on boundary of neighborhood. make span bigger
```

```
## Warning in simpleLoess(y, x, w, span, degree = degree, parametric = parametric, : pseudoinverse used at -0.005
```

```
## Warning in simpleLoess(y, x, w, span, degree = degree, parametric = parametric, : neighborhood radius 0.005
```

```
## Warning in simpleLoess(y, x, w, span, degree = degree, parametric = parametric, : reciprocal condition number 1
```

```
## Warning in simpleLoess(y, x, w, span, degree = degree, parametric = parametric, : There are other near singularities as well. 1.01
```

```
## Warning in simpleLoess(y, x, w, span, degree = degree, parametric = parametric, : zero-width neighborhood. make span bigger
```

```
## Warning: Computation failed in `stat_smooth()`:
## NA/NaN/Inf in foreign function call (arg 5)
```

```
## Warning in simpleLoess(y, x, w, span, degree = degree, parametric = parametric, : at -0.005
```

```
## Warning in simpleLoess(y, x, w, span, degree = degree, parametric = parametric, : radius 2.5e-05
```

```
## Warning in simpleLoess(y, x, w, span, degree = degree, parametric = parametric, : all data on boundary of neighborhood. make span bigger
```

```
## Warning in simpleLoess(y, x, w, span, degree = degree, parametric = parametric, : pseudoinverse used at -0.005
```

```
## Warning in simpleLoess(y, x, w, span, degree = degree, parametric = parametric, : neighborhood radius 0.005
```

```
## Warning in simpleLoess(y, x, w, span, degree = degree, parametric = parametric, : reciprocal condition number 1
```

```
## Warning in simpleLoess(y, x, w, span, degree = degree, parametric = parametric, : There are other near singularities as well. 1.01
```

```
## Warning in simpleLoess(y, x, w, span, degree = degree, parametric = parametric, : zero-width neighborhood. make span bigger
```

```
## Warning: Computation failed in `stat_smooth()`:
## NA/NaN/Inf in foreign function call (arg 5)
```

```
## Warning in simpleLoess(y, x, w, span, degree = degree, parametric = parametric, : at -0.005
```

```
## Warning in simpleLoess(y, x, w, span, degree = degree, parametric = parametric, : radius 2.5e-05
```

```
## Warning in simpleLoess(y, x, w, span, degree = degree, parametric = parametric, : all data on boundary of neighborhood. make span bigger
```

```
## Warning in simpleLoess(y, x, w, span, degree = degree, parametric = parametric, : pseudoinverse used at -0.005
```

```
## Warning in simpleLoess(y, x, w, span, degree = degree, parametric = parametric, : neighborhood radius 0.005
```

```
## Warning in simpleLoess(y, x, w, span, degree = degree, parametric = parametric, : reciprocal condition number 1
```

```
## Warning in simpleLoess(y, x, w, span, degree = degree, parametric = parametric, : There are other near singularities as well. 1.01
```

```
## Warning in simpleLoess(y, x, w, span, degree = degree, parametric = parametric, : zero-width neighborhood. make span bigger
```

```
## Warning: Computation failed in `stat_smooth()`:
## NA/NaN/Inf in foreign function call (arg 5)
```

```
## Warning in simpleLoess(y, x, w, span, degree = degree, parametric = parametric, : at -0.005
```

```
## Warning in simpleLoess(y, x, w, span, degree = degree, parametric = parametric, : radius 2.5e-05
```

```
## Warning in simpleLoess(y, x, w, span, degree = degree, parametric = parametric, : all data on boundary of neighborhood. make span bigger
```

```
## Warning in simpleLoess(y, x, w, span, degree = degree, parametric = parametric, : pseudoinverse used at -0.005
```

```
## Warning in simpleLoess(y, x, w, span, degree = degree, parametric = parametric, : neighborhood radius 0.005
```

```
## Warning in simpleLoess(y, x, w, span, degree = degree, parametric = parametric, : reciprocal condition number 1
```

```
## Warning in simpleLoess(y, x, w, span, degree = degree, parametric = parametric, : There are other near singularities as well. 1.01
```

```
## Warning in simpleLoess(y, x, w, span, degree = degree, parametric = parametric, : zero-width neighborhood. make span bigger
```

```
## Warning: Computation failed in `stat_smooth()`:
## NA/NaN/Inf in foreign function call (arg 5)
```

```
## Warning in simpleLoess(y, x, w, span, degree = degree, parametric = parametric, : at -0.005
```

```
## Warning in simpleLoess(y, x, w, span, degree = degree, parametric = parametric, : radius 2.5e-05
```

```
## Warning in simpleLoess(y, x, w, span, degree = degree, parametric = parametric, : all data on boundary of neighborhood. make span bigger
```

```
## Warning in simpleLoess(y, x, w, span, degree = degree, parametric = parametric, : pseudoinverse used at -0.005
```

```
## Warning in simpleLoess(y, x, w, span, degree = degree, parametric = parametric, : neighborhood radius 0.005
```

```
## Warning in simpleLoess(y, x, w, span, degree = degree, parametric = parametric, : reciprocal condition number 1
```

```
## Warning in simpleLoess(y, x, w, span, degree = degree, parametric = parametric, : There are other near singularities as well. 1.01
```

```
## Warning in simpleLoess(y, x, w, span, degree = degree, parametric = parametric, : zero-width neighborhood. make span bigger
```

```
## Warning: Computation failed in `stat_smooth()`:
## NA/NaN/Inf in foreign function call (arg 5)
```

```
## Warning in simpleLoess(y, x, w, span, degree = degree, parametric = parametric, : at -0.005
```

```
## Warning in simpleLoess(y, x, w, span, degree = degree, parametric = parametric, : radius 2.5e-05
```

```
## Warning in simpleLoess(y, x, w, span, degree = degree, parametric = parametric, : all data on boundary of neighborhood. make span bigger
```

```
## Warning in simpleLoess(y, x, w, span, degree = degree, parametric = parametric, : pseudoinverse used at -0.005
```

```
## Warning in simpleLoess(y, x, w, span, degree = degree, parametric = parametric, : neighborhood radius 0.005
```

```
## Warning in simpleLoess(y, x, w, span, degree = degree, parametric = parametric, : reciprocal condition number 1
```

```
## Warning in simpleLoess(y, x, w, span, degree = degree, parametric = parametric, : There are other near singularities as well. 1.01
```

```
## Warning in simpleLoess(y, x, w, span, degree = degree, parametric = parametric, : zero-width neighborhood. make span bigger
```

```
## Warning: Computation failed in `stat_smooth()`:
## NA/NaN/Inf in foreign function call (arg 5)
```

```
## Warning in simpleLoess(y, x, w, span, degree = degree, parametric = parametric, : at -0.005
```

```
## Warning in simpleLoess(y, x, w, span, degree = degree, parametric = parametric, : radius 2.5e-05
```

```
## Warning in simpleLoess(y, x, w, span, degree = degree, parametric = parametric, : all data on boundary of neighborhood. make span bigger
```

```
## Warning in simpleLoess(y, x, w, span, degree = degree, parametric = parametric, : pseudoinverse used at -0.005
```

```
## Warning in simpleLoess(y, x, w, span, degree = degree, parametric = parametric, : neighborhood radius 0.005
```

```
## Warning in simpleLoess(y, x, w, span, degree = degree, parametric = parametric, : reciprocal condition number 1
```

```
## Warning in simpleLoess(y, x, w, span, degree = degree, parametric = parametric, : There are other near singularities as well. 1.01
```

```
## Warning in simpleLoess(y, x, w, span, degree = degree, parametric = parametric, : zero-width neighborhood. make span bigger
```

```
## Warning: Computation failed in `stat_smooth()`:
## NA/NaN/Inf in foreign function call (arg 5)
```

```
## Warning in simpleLoess(y, x, w, span, degree = degree, parametric = parametric, : at -0.005
```

```
## Warning in simpleLoess(y, x, w, span, degree = degree, parametric = parametric, : radius 2.5e-05
```

```
## Warning in simpleLoess(y, x, w, span, degree = degree, parametric = parametric, : all data on boundary of neighborhood. make span bigger
```

```
## Warning in simpleLoess(y, x, w, span, degree = degree, parametric = parametric, : pseudoinverse used at -0.005
```

```
## Warning in simpleLoess(y, x, w, span, degree = degree, parametric = parametric, : neighborhood radius 0.005
```

```
## Warning in simpleLoess(y, x, w, span, degree = degree, parametric = parametric, : reciprocal condition number 1
```

```
## Warning in simpleLoess(y, x, w, span, degree = degree, parametric = parametric, : There are other near singularities as well. 1.01
```

```
## Warning in simpleLoess(y, x, w, span, degree = degree, parametric = parametric, : zero-width neighborhood. make span bigger
```

```
## Warning: Computation failed in `stat_smooth()`:
## NA/NaN/Inf in foreign function call (arg 5)
```

```
## Warning in simpleLoess(y, x, w, span, degree = degree, parametric = parametric, : at -0.005
```

```
## Warning in simpleLoess(y, x, w, span, degree = degree, parametric = parametric, : radius 2.5e-05
```

```
## Warning in simpleLoess(y, x, w, span, degree = degree, parametric = parametric, : all data on boundary of neighborhood. make span bigger
```

```
## Warning in simpleLoess(y, x, w, span, degree = degree, parametric = parametric, : pseudoinverse used at -0.005
```

```
## Warning in simpleLoess(y, x, w, span, degree = degree, parametric = parametric, : neighborhood radius 0.005
```

```
## Warning in simpleLoess(y, x, w, span, degree = degree, parametric = parametric, : reciprocal condition number 1
```

```
## Warning in simpleLoess(y, x, w, span, degree = degree, parametric = parametric, : There are other near singularities as well. 1.01
```

```
## Warning in simpleLoess(y, x, w, span, degree = degree, parametric = parametric, : zero-width neighborhood. make span bigger
```

```
## Warning: Computation failed in `stat_smooth()`:
## NA/NaN/Inf in foreign function call (arg 5)
```

```
## Warning in simpleLoess(y, x, w, span, degree = degree, parametric = parametric, : pseudoinverse used at -0.005
```

```
## Warning in simpleLoess(y, x, w, span, degree = degree, parametric = parametric, : neighborhood radius 1.005
```

```
## Warning in simpleLoess(y, x, w, span, degree = degree, parametric = parametric, : reciprocal condition number 0
```

```
## Warning in simpleLoess(y, x, w, span, degree = degree, parametric = parametric, : There are other near singularities as well. 1.01
```

```
## Warning in predLoess(object$y, object$x, newx = if (is.null(newdata)) object$x else if (is.data.frame(newdata)) as.matrix(model.frame(delete.response(terms(object)), : pseudoinverse used at -0.005
```

```
## Warning in predLoess(object$y, object$x, newx = if (is.null(newdata)) object$x else if (is.data.frame(newdata)) as.matrix(model.frame(delete.response(terms(object)), : neighborhood radius 1.005
```

```
## Warning in predLoess(object$y, object$x, newx = if (is.null(newdata)) object$x else if (is.data.frame(newdata)) as.matrix(model.frame(delete.response(terms(object)), : reciprocal condition number 0
```

```
## Warning in predLoess(object$y, object$x, newx = if (is.null(newdata)) object$x else if (is.data.frame(newdata)) as.matrix(model.frame(delete.response(terms(object)), : There are other near
## singularities as well. 1.01
```

```
## Warning in simpleLoess(y, x, w, span, degree = degree, parametric = parametric, : at -0.005
```

```
## Warning in simpleLoess(y, x, w, span, degree = degree, parametric = parametric, : radius 2.5e-05
```

```
## Warning in simpleLoess(y, x, w, span, degree = degree, parametric = parametric, : all data on boundary of neighborhood. make span bigger
```

```
## Warning in simpleLoess(y, x, w, span, degree = degree, parametric = parametric, : pseudoinverse used at -0.005
```

```
## Warning in simpleLoess(y, x, w, span, degree = degree, parametric = parametric, : neighborhood radius 0.005
```

```
## Warning in simpleLoess(y, x, w, span, degree = degree, parametric = parametric, : reciprocal condition number 1
```

```
## Warning in simpleLoess(y, x, w, span, degree = degree, parametric = parametric, : There are other near singularities as well. 1.01
```

```
## Warning in simpleLoess(y, x, w, span, degree = degree, parametric = parametric, : zero-width neighborhood. make span bigger
```

```
## Warning: Computation failed in `stat_smooth()`:
## NA/NaN/Inf in foreign function call (arg 5)
```

```
## Warning in simpleLoess(y, x, w, span, degree = degree, parametric = parametric, : at -0.005
```

```
## Warning in simpleLoess(y, x, w, span, degree = degree, parametric = parametric, : radius 2.5e-05
```

```
## Warning in simpleLoess(y, x, w, span, degree = degree, parametric = parametric, : all data on boundary of neighborhood. make span bigger
```

```
## Warning in simpleLoess(y, x, w, span, degree = degree, parametric = parametric, : pseudoinverse used at -0.005
```

```
## Warning in simpleLoess(y, x, w, span, degree = degree, parametric = parametric, : neighborhood radius 0.005
```

```
## Warning in simpleLoess(y, x, w, span, degree = degree, parametric = parametric, : reciprocal condition number 1
```

```
## Warning in simpleLoess(y, x, w, span, degree = degree, parametric = parametric, : There are other near singularities as well. 1.01
```

```
## Warning in simpleLoess(y, x, w, span, degree = degree, parametric = parametric, : zero-width neighborhood. make span bigger
```

```
## Warning: Computation failed in `stat_smooth()`:
## NA/NaN/Inf in foreign function call (arg 5)
```

```
## Warning in simpleLoess(y, x, w, span, degree = degree, parametric = parametric, : at -0.005
```

```
## Warning in simpleLoess(y, x, w, span, degree = degree, parametric = parametric, : radius 2.5e-05
```

```
## Warning in simpleLoess(y, x, w, span, degree = degree, parametric = parametric, : all data on boundary of neighborhood. make span bigger
```

```
## Warning in simpleLoess(y, x, w, span, degree = degree, parametric = parametric, : pseudoinverse used at -0.005
```

```
## Warning in simpleLoess(y, x, w, span, degree = degree, parametric = parametric, : neighborhood radius 0.005
```

```
## Warning in simpleLoess(y, x, w, span, degree = degree, parametric = parametric, : reciprocal condition number 1
```

```
## Warning in simpleLoess(y, x, w, span, degree = degree, parametric = parametric, : There are other near singularities as well. 1.01
```

```
## Warning in simpleLoess(y, x, w, span, degree = degree, parametric = parametric, : zero-width neighborhood. make span bigger
```

```
## Warning: Computation failed in `stat_smooth()`:
## NA/NaN/Inf in foreign function call (arg 5)
```

```
## Warning in simpleLoess(y, x, w, span, degree = degree, parametric = parametric, : at -0.005
```

```
## Warning in simpleLoess(y, x, w, span, degree = degree, parametric = parametric, : radius 2.5e-05
```

```
## Warning in simpleLoess(y, x, w, span, degree = degree, parametric = parametric, : all data on boundary of neighborhood. make span bigger
```

```
## Warning in simpleLoess(y, x, w, span, degree = degree, parametric = parametric, : pseudoinverse used at -0.005
```

```
## Warning in simpleLoess(y, x, w, span, degree = degree, parametric = parametric, : neighborhood radius 0.005
```

```
## Warning in simpleLoess(y, x, w, span, degree = degree, parametric = parametric, : reciprocal condition number 1
```

```
## Warning in simpleLoess(y, x, w, span, degree = degree, parametric = parametric, : There are other near singularities as well. 1.01
```

```
## Warning in simpleLoess(y, x, w, span, degree = degree, parametric = parametric, : zero-width neighborhood. make span bigger
```

```
## Warning: Computation failed in `stat_smooth()`:
## NA/NaN/Inf in foreign function call (arg 5)
```

```
## Warning in simpleLoess(y, x, w, span, degree = degree, parametric = parametric, : at -0.005
```

```
## Warning in simpleLoess(y, x, w, span, degree = degree, parametric = parametric, : radius 2.5e-05
```

```
## Warning in simpleLoess(y, x, w, span, degree = degree, parametric = parametric, : all data on boundary of neighborhood. make span bigger
```

```
## Warning in simpleLoess(y, x, w, span, degree = degree, parametric = parametric, : pseudoinverse used at -0.005
```

```
## Warning in simpleLoess(y, x, w, span, degree = degree, parametric = parametric, : neighborhood radius 0.005
```

```
## Warning in simpleLoess(y, x, w, span, degree = degree, parametric = parametric, : reciprocal condition number 1
```

```
## Warning in simpleLoess(y, x, w, span, degree = degree, parametric = parametric, : There are other near singularities as well. 1.01
```

```
## Warning in simpleLoess(y, x, w, span, degree = degree, parametric = parametric, : zero-width neighborhood. make span bigger
```

```
## Warning: Computation failed in `stat_smooth()`:
## NA/NaN/Inf in foreign function call (arg 5)
```

```
## Warning in simpleLoess(y, x, w, span, degree = degree, parametric = parametric, : at -0.005
```

```
## Warning in simpleLoess(y, x, w, span, degree = degree, parametric = parametric, : radius 2.5e-05
```

```
## Warning in simpleLoess(y, x, w, span, degree = degree, parametric = parametric, : all data on boundary of neighborhood. make span bigger
```

```
## Warning in simpleLoess(y, x, w, span, degree = degree, parametric = parametric, : pseudoinverse used at -0.005
```

```
## Warning in simpleLoess(y, x, w, span, degree = degree, parametric = parametric, : neighborhood radius 0.005
```

```
## Warning in simpleLoess(y, x, w, span, degree = degree, parametric = parametric, : reciprocal condition number 1
```

```
## Warning in simpleLoess(y, x, w, span, degree = degree, parametric = parametric, : There are other near singularities as well. 1.01
```

```
## Warning in simpleLoess(y, x, w, span, degree = degree, parametric = parametric, : zero-width neighborhood. make span bigger
```

```
## Warning: Computation failed in `stat_smooth()`:
## NA/NaN/Inf in foreign function call (arg 5)
```

```
## Warning in simpleLoess(y, x, w, span, degree = degree, parametric = parametric, : at -0.005
```

```
## Warning in simpleLoess(y, x, w, span, degree = degree, parametric = parametric, : radius 2.5e-05
```

```
## Warning in simpleLoess(y, x, w, span, degree = degree, parametric = parametric, : all data on boundary of neighborhood. make span bigger
```

```
## Warning in simpleLoess(y, x, w, span, degree = degree, parametric = parametric, : pseudoinverse used at -0.005
```

```
## Warning in simpleLoess(y, x, w, span, degree = degree, parametric = parametric, : neighborhood radius 0.005
```

```
## Warning in simpleLoess(y, x, w, span, degree = degree, parametric = parametric, : reciprocal condition number 1
```

```
## Warning in simpleLoess(y, x, w, span, degree = degree, parametric = parametric, : There are other near singularities as well. 1.01
```

```
## Warning in simpleLoess(y, x, w, span, degree = degree, parametric = parametric, : zero-width neighborhood. make span bigger
```

```
## Warning: Computation failed in `stat_smooth()`:
## NA/NaN/Inf in foreign function call (arg 5)
```

```
## Warning in simpleLoess(y, x, w, span, degree = degree, parametric = parametric, : at -0.005
```

```
## Warning in simpleLoess(y, x, w, span, degree = degree, parametric = parametric, : radius 2.5e-05
```

```
## Warning in simpleLoess(y, x, w, span, degree = degree, parametric = parametric, : all data on boundary of neighborhood. make span bigger
```

```
## Warning in simpleLoess(y, x, w, span, degree = degree, parametric = parametric, : pseudoinverse used at -0.005
```

```
## Warning in simpleLoess(y, x, w, span, degree = degree, parametric = parametric, : neighborhood radius 0.005
```

```
## Warning in simpleLoess(y, x, w, span, degree = degree, parametric = parametric, : reciprocal condition number 1
```

```
## Warning in simpleLoess(y, x, w, span, degree = degree, parametric = parametric, : There are other near singularities as well. 1.01
```

```
## Warning in simpleLoess(y, x, w, span, degree = degree, parametric = parametric, : zero-width neighborhood. make span bigger
```

```
## Warning: Computation failed in `stat_smooth()`:
## NA/NaN/Inf in foreign function call (arg 5)
```

```
## Warning in simpleLoess(y, x, w, span, degree = degree, parametric = parametric, : at -0.005
```

```
## Warning in simpleLoess(y, x, w, span, degree = degree, parametric = parametric, : radius 2.5e-05
```

```
## Warning in simpleLoess(y, x, w, span, degree = degree, parametric = parametric, : all data on boundary of neighborhood. make span bigger
```

```
## Warning in simpleLoess(y, x, w, span, degree = degree, parametric = parametric, : pseudoinverse used at -0.005
```

```
## Warning in simpleLoess(y, x, w, span, degree = degree, parametric = parametric, : neighborhood radius 0.005
```

```
## Warning in simpleLoess(y, x, w, span, degree = degree, parametric = parametric, : reciprocal condition number 1
```

```
## Warning in simpleLoess(y, x, w, span, degree = degree, parametric = parametric, : There are other near singularities as well. 1.01
```

```
## Warning in simpleLoess(y, x, w, span, degree = degree, parametric = parametric, : zero-width neighborhood. make span bigger
```

```
## Warning: Computation failed in `stat_smooth()`:
## NA/NaN/Inf in foreign function call (arg 5)
```

```
## Warning in simpleLoess(y, x, w, span, degree = degree, parametric = parametric, : at -0.005
```

```
## Warning in simpleLoess(y, x, w, span, degree = degree, parametric = parametric, : radius 2.5e-05
```

```
## Warning in simpleLoess(y, x, w, span, degree = degree, parametric = parametric, : all data on boundary of neighborhood. make span bigger
```

```
## Warning in simpleLoess(y, x, w, span, degree = degree, parametric = parametric, : pseudoinverse used at -0.005
```

```
## Warning in simpleLoess(y, x, w, span, degree = degree, parametric = parametric, : neighborhood radius 0.005
```

```
## Warning in simpleLoess(y, x, w, span, degree = degree, parametric = parametric, : reciprocal condition number 1
```

```
## Warning in simpleLoess(y, x, w, span, degree = degree, parametric = parametric, : There are other near singularities as well. 1.01
```

```
## Warning in simpleLoess(y, x, w, span, degree = degree, parametric = parametric, : zero-width neighborhood. make span bigger
```

```
## Warning: Computation failed in `stat_smooth()`:
## NA/NaN/Inf in foreign function call (arg 5)
```

```
## Warning in simpleLoess(y, x, w, span, degree = degree, parametric = parametric, : at -0.005
```

```
## Warning in simpleLoess(y, x, w, span, degree = degree, parametric = parametric, : radius 2.5e-05
```

```
## Warning in simpleLoess(y, x, w, span, degree = degree, parametric = parametric, : all data on boundary of neighborhood. make span bigger
```

```
## Warning in simpleLoess(y, x, w, span, degree = degree, parametric = parametric, : pseudoinverse used at -0.005
```

```
## Warning in simpleLoess(y, x, w, span, degree = degree, parametric = parametric, : neighborhood radius 0.005
```

```
## Warning in simpleLoess(y, x, w, span, degree = degree, parametric = parametric, : reciprocal condition number 1
```

```
## Warning in simpleLoess(y, x, w, span, degree = degree, parametric = parametric, : There are other near singularities as well. 1.01
```

```
## Warning in simpleLoess(y, x, w, span, degree = degree, parametric = parametric, : zero-width neighborhood. make span bigger
```

```
## Warning: Computation failed in `stat_smooth()`:
## NA/NaN/Inf in foreign function call (arg 5)
```

```
## Warning in simpleLoess(y, x, w, span, degree = degree, parametric = parametric, : at -0.005
```

```
## Warning in simpleLoess(y, x, w, span, degree = degree, parametric = parametric, : radius 2.5e-05
```

```
## Warning in simpleLoess(y, x, w, span, degree = degree, parametric = parametric, : all data on boundary of neighborhood. make span bigger
```

```
## Warning in simpleLoess(y, x, w, span, degree = degree, parametric = parametric, : pseudoinverse used at -0.005
```

```
## Warning in simpleLoess(y, x, w, span, degree = degree, parametric = parametric, : neighborhood radius 0.005
```

```
## Warning in simpleLoess(y, x, w, span, degree = degree, parametric = parametric, : reciprocal condition number 1
```

```
## Warning in simpleLoess(y, x, w, span, degree = degree, parametric = parametric, : There are other near singularities as well. 1.01
```

```
## Warning in simpleLoess(y, x, w, span, degree = degree, parametric = parametric, : zero-width neighborhood. make span bigger
```

```
## Warning: Computation failed in `stat_smooth()`:
## NA/NaN/Inf in foreign function call (arg 5)
```

```
## Warning in simpleLoess(y, x, w, span, degree = degree, parametric = parametric, : at -0.005
```

```
## Warning in simpleLoess(y, x, w, span, degree = degree, parametric = parametric, : radius 2.5e-05
```

```
## Warning in simpleLoess(y, x, w, span, degree = degree, parametric = parametric, : all data on boundary of neighborhood. make span bigger
```

```
## Warning in simpleLoess(y, x, w, span, degree = degree, parametric = parametric, : pseudoinverse used at -0.005
```

```
## Warning in simpleLoess(y, x, w, span, degree = degree, parametric = parametric, : neighborhood radius 0.005
```

```
## Warning in simpleLoess(y, x, w, span, degree = degree, parametric = parametric, : reciprocal condition number 1
```

```
## Warning in simpleLoess(y, x, w, span, degree = degree, parametric = parametric, : There are other near singularities as well. 1.01
```

```
## Warning in simpleLoess(y, x, w, span, degree = degree, parametric = parametric, : zero-width neighborhood. make span bigger
```

```
## Warning: Computation failed in `stat_smooth()`:
## NA/NaN/Inf in foreign function call (arg 5)
```

```
## Warning in simpleLoess(y, x, w, span, degree = degree, parametric = parametric, : at -0.005
```

```
## Warning in simpleLoess(y, x, w, span, degree = degree, parametric = parametric, : radius 2.5e-05
```

```
## Warning in simpleLoess(y, x, w, span, degree = degree, parametric = parametric, : all data on boundary of neighborhood. make span bigger
```

```
## Warning in simpleLoess(y, x, w, span, degree = degree, parametric = parametric, : pseudoinverse used at -0.005
```

```
## Warning in simpleLoess(y, x, w, span, degree = degree, parametric = parametric, : neighborhood radius 0.005
```

```
## Warning in simpleLoess(y, x, w, span, degree = degree, parametric = parametric, : reciprocal condition number 1
```

```
## Warning in simpleLoess(y, x, w, span, degree = degree, parametric = parametric, : There are other near singularities as well. 1.01
```

```
## Warning in simpleLoess(y, x, w, span, degree = degree, parametric = parametric, : zero-width neighborhood. make span bigger
```

```
## Warning: Computation failed in `stat_smooth()`:
## NA/NaN/Inf in foreign function call (arg 5)
```

```
## Warning in simpleLoess(y, x, w, span, degree = degree, parametric = parametric, : at -0.005
```

```
## Warning in simpleLoess(y, x, w, span, degree = degree, parametric = parametric, : radius 2.5e-05
```

```
## Warning in simpleLoess(y, x, w, span, degree = degree, parametric = parametric, : all data on boundary of neighborhood. make span bigger
```

```
## Warning in simpleLoess(y, x, w, span, degree = degree, parametric = parametric, : pseudoinverse used at -0.005
```

```
## Warning in simpleLoess(y, x, w, span, degree = degree, parametric = parametric, : neighborhood radius 0.005
```

```
## Warning in simpleLoess(y, x, w, span, degree = degree, parametric = parametric, : reciprocal condition number 1
```

```
## Warning in simpleLoess(y, x, w, span, degree = degree, parametric = parametric, : There are other near singularities as well. 1.01
```

```
## Warning in simpleLoess(y, x, w, span, degree = degree, parametric = parametric, : zero-width neighborhood. make span bigger
```

```
## Warning: Computation failed in `stat_smooth()`:
## NA/NaN/Inf in foreign function call (arg 5)
```

```
## Warning in simpleLoess(y, x, w, span, degree = degree, parametric = parametric, : at -0.005
```

```
## Warning in simpleLoess(y, x, w, span, degree = degree, parametric = parametric, : radius 2.5e-05
```

```
## Warning in simpleLoess(y, x, w, span, degree = degree, parametric = parametric, : all data on boundary of neighborhood. make span bigger
```

```
## Warning in simpleLoess(y, x, w, span, degree = degree, parametric = parametric, : pseudoinverse used at -0.005
```

```
## Warning in simpleLoess(y, x, w, span, degree = degree, parametric = parametric, : neighborhood radius 0.005
```

```
## Warning in simpleLoess(y, x, w, span, degree = degree, parametric = parametric, : reciprocal condition number 1
```

```
## Warning in simpleLoess(y, x, w, span, degree = degree, parametric = parametric, : There are other near singularities as well. 1.01
```

```
## Warning in simpleLoess(y, x, w, span, degree = degree, parametric = parametric, : zero-width neighborhood. make span bigger
```

```
## Warning: Computation failed in `stat_smooth()`:
## NA/NaN/Inf in foreign function call (arg 5)
```

```
## Warning in simpleLoess(y, x, w, span, degree = degree, parametric = parametric, : at -0.005
```

```
## Warning in simpleLoess(y, x, w, span, degree = degree, parametric = parametric, : radius 2.5e-05
```

```
## Warning in simpleLoess(y, x, w, span, degree = degree, parametric = parametric, : all data on boundary of neighborhood. make span bigger
```

```
## Warning in simpleLoess(y, x, w, span, degree = degree, parametric = parametric, : pseudoinverse used at -0.005
```

```
## Warning in simpleLoess(y, x, w, span, degree = degree, parametric = parametric, : neighborhood radius 0.005
```

```
## Warning in simpleLoess(y, x, w, span, degree = degree, parametric = parametric, : reciprocal condition number 1
```

```
## Warning in simpleLoess(y, x, w, span, degree = degree, parametric = parametric, : There are other near singularities as well. 1.01
```

```
## Warning in simpleLoess(y, x, w, span, degree = degree, parametric = parametric, : zero-width neighborhood. make span bigger
```

```
## Warning: Computation failed in `stat_smooth()`:
## NA/NaN/Inf in foreign function call (arg 5)
```

```
## Warning in simpleLoess(y, x, w, span, degree = degree, parametric = parametric, : at -0.005
```

```
## Warning in simpleLoess(y, x, w, span, degree = degree, parametric = parametric, : radius 2.5e-05
```

```
## Warning in simpleLoess(y, x, w, span, degree = degree, parametric = parametric, : all data on boundary of neighborhood. make span bigger
```

```
## Warning in simpleLoess(y, x, w, span, degree = degree, parametric = parametric, : pseudoinverse used at -0.005
```

```
## Warning in simpleLoess(y, x, w, span, degree = degree, parametric = parametric, : neighborhood radius 0.005
```

```
## Warning in simpleLoess(y, x, w, span, degree = degree, parametric = parametric, : reciprocal condition number 1
```

```
## Warning in simpleLoess(y, x, w, span, degree = degree, parametric = parametric, : There are other near singularities as well. 1.01
```

```
## Warning in simpleLoess(y, x, w, span, degree = degree, parametric = parametric, : zero-width neighborhood. make span bigger
```

```
## Warning: Computation failed in `stat_smooth()`:
## NA/NaN/Inf in foreign function call (arg 5)
```

```
## Warning in simpleLoess(y, x, w, span, degree = degree, parametric = parametric, : at -0.005
```

```
## Warning in simpleLoess(y, x, w, span, degree = degree, parametric = parametric, : radius 2.5e-05
```

```
## Warning in simpleLoess(y, x, w, span, degree = degree, parametric = parametric, : all data on boundary of neighborhood. make span bigger
```

```
## Warning in simpleLoess(y, x, w, span, degree = degree, parametric = parametric, : pseudoinverse used at -0.005
```

```
## Warning in simpleLoess(y, x, w, span, degree = degree, parametric = parametric, : neighborhood radius 0.005
```

```
## Warning in simpleLoess(y, x, w, span, degree = degree, parametric = parametric, : reciprocal condition number 1
```

```
## Warning in simpleLoess(y, x, w, span, degree = degree, parametric = parametric, : There are other near singularities as well. 1.01
```

```
## Warning in simpleLoess(y, x, w, span, degree = degree, parametric = parametric, : zero-width neighborhood. make span bigger
```

```
## Warning: Computation failed in `stat_smooth()`:
## NA/NaN/Inf in foreign function call (arg 5)
```

```
## Warning in simpleLoess(y, x, w, span, degree = degree, parametric = parametric, : at -0.005
```

```
## Warning in simpleLoess(y, x, w, span, degree = degree, parametric = parametric, : radius 2.5e-05
```

```
## Warning in simpleLoess(y, x, w, span, degree = degree, parametric = parametric, : all data on boundary of neighborhood. make span bigger
```

```
## Warning in simpleLoess(y, x, w, span, degree = degree, parametric = parametric, : pseudoinverse used at -0.005
```

```
## Warning in simpleLoess(y, x, w, span, degree = degree, parametric = parametric, : neighborhood radius 0.005
```

```
## Warning in simpleLoess(y, x, w, span, degree = degree, parametric = parametric, : reciprocal condition number 1
```

```
## Warning in simpleLoess(y, x, w, span, degree = degree, parametric = parametric, : There are other near singularities as well. 1.01
```

```
## Warning in simpleLoess(y, x, w, span, degree = degree, parametric = parametric, : zero-width neighborhood. make span bigger
```

```
## Warning: Computation failed in `stat_smooth()`:
## NA/NaN/Inf in foreign function call (arg 5)
```

```
## Warning in simpleLoess(y, x, w, span, degree = degree, parametric = parametric, : at -0.005
```

```
## Warning in simpleLoess(y, x, w, span, degree = degree, parametric = parametric, : radius 2.5e-05
```

```
## Warning in simpleLoess(y, x, w, span, degree = degree, parametric = parametric, : all data on boundary of neighborhood. make span bigger
```

```
## Warning in simpleLoess(y, x, w, span, degree = degree, parametric = parametric, : pseudoinverse used at -0.005
```

```
## Warning in simpleLoess(y, x, w, span, degree = degree, parametric = parametric, : neighborhood radius 0.005
```

```
## Warning in simpleLoess(y, x, w, span, degree = degree, parametric = parametric, : reciprocal condition number 1
```

```
## Warning in simpleLoess(y, x, w, span, degree = degree, parametric = parametric, : There are other near singularities as well. 1.01
```

```
## Warning in simpleLoess(y, x, w, span, degree = degree, parametric = parametric, : zero-width neighborhood. make span bigger
```

```
## Warning: Computation failed in `stat_smooth()`:
## NA/NaN/Inf in foreign function call (arg 5)
```

```
## Warning in simpleLoess(y, x, w, span, degree = degree, parametric = parametric, : at -0.005
```

```
## Warning in simpleLoess(y, x, w, span, degree = degree, parametric = parametric, : radius 2.5e-05
```

```
## Warning in simpleLoess(y, x, w, span, degree = degree, parametric = parametric, : all data on boundary of neighborhood. make span bigger
```

```
## Warning in simpleLoess(y, x, w, span, degree = degree, parametric = parametric, : pseudoinverse used at -0.005
```

```
## Warning in simpleLoess(y, x, w, span, degree = degree, parametric = parametric, : neighborhood radius 0.005
```

```
## Warning in simpleLoess(y, x, w, span, degree = degree, parametric = parametric, : reciprocal condition number 1
```

```
## Warning in simpleLoess(y, x, w, span, degree = degree, parametric = parametric, : There are other near singularities as well. 1.01
```

```
## Warning in simpleLoess(y, x, w, span, degree = degree, parametric = parametric, : zero-width neighborhood. make span bigger
```

```
## Warning: Computation failed in `stat_smooth()`:
## NA/NaN/Inf in foreign function call (arg 5)
```

```
## Warning in simpleLoess(y, x, w, span, degree = degree, parametric = parametric, : at -0.005
```

```
## Warning in simpleLoess(y, x, w, span, degree = degree, parametric = parametric, : radius 2.5e-05
```

```
## Warning in simpleLoess(y, x, w, span, degree = degree, parametric = parametric, : all data on boundary of neighborhood. make span bigger
```

```
## Warning in simpleLoess(y, x, w, span, degree = degree, parametric = parametric, : pseudoinverse used at -0.005
```

```
## Warning in simpleLoess(y, x, w, span, degree = degree, parametric = parametric, : neighborhood radius 0.005
```

```
## Warning in simpleLoess(y, x, w, span, degree = degree, parametric = parametric, : reciprocal condition number 1
```

```
## Warning in simpleLoess(y, x, w, span, degree = degree, parametric = parametric, : There are other near singularities as well. 1.01
```

```
## Warning in simpleLoess(y, x, w, span, degree = degree, parametric = parametric, : zero-width neighborhood. make span bigger
```

```
## Warning: Computation failed in `stat_smooth()`:
## NA/NaN/Inf in foreign function call (arg 5)
```

```
## Warning in simpleLoess(y, x, w, span, degree = degree, parametric = parametric, : at -0.005
```

```
## Warning in simpleLoess(y, x, w, span, degree = degree, parametric = parametric, : radius 2.5e-05
```

```
## Warning in simpleLoess(y, x, w, span, degree = degree, parametric = parametric, : all data on boundary of neighborhood. make span bigger
```

```
## Warning in simpleLoess(y, x, w, span, degree = degree, parametric = parametric, : pseudoinverse used at -0.005
```

```
## Warning in simpleLoess(y, x, w, span, degree = degree, parametric = parametric, : neighborhood radius 0.005
```

```
## Warning in simpleLoess(y, x, w, span, degree = degree, parametric = parametric, : reciprocal condition number 1
```

```
## Warning in simpleLoess(y, x, w, span, degree = degree, parametric = parametric, : There are other near singularities as well. 1.01
```

```
## Warning in simpleLoess(y, x, w, span, degree = degree, parametric = parametric, : zero-width neighborhood. make span bigger
```

```
## Warning: Computation failed in `stat_smooth()`:
## NA/NaN/Inf in foreign function call (arg 5)
```

```
## Warning in simpleLoess(y, x, w, span, degree = degree, parametric = parametric, : at -0.005
```

```
## Warning in simpleLoess(y, x, w, span, degree = degree, parametric = parametric, : radius 2.5e-05
```

```
## Warning in simpleLoess(y, x, w, span, degree = degree, parametric = parametric, : all data on boundary of neighborhood. make span bigger
```

```
## Warning in simpleLoess(y, x, w, span, degree = degree, parametric = parametric, : pseudoinverse used at -0.005
```

```
## Warning in simpleLoess(y, x, w, span, degree = degree, parametric = parametric, : neighborhood radius 0.005
```

```
## Warning in simpleLoess(y, x, w, span, degree = degree, parametric = parametric, : reciprocal condition number 1
```

```
## Warning in simpleLoess(y, x, w, span, degree = degree, parametric = parametric, : There are other near singularities as well. 1.01
```

```
## Warning in simpleLoess(y, x, w, span, degree = degree, parametric = parametric, : zero-width neighborhood. make span bigger
```

```
## Warning: Computation failed in `stat_smooth()`:
## NA/NaN/Inf in foreign function call (arg 5)
```

```
## Warning in simpleLoess(y, x, w, span, degree = degree, parametric = parametric, : at -0.005
```

```
## Warning in simpleLoess(y, x, w, span, degree = degree, parametric = parametric, : radius 2.5e-05
```

```
## Warning in simpleLoess(y, x, w, span, degree = degree, parametric = parametric, : all data on boundary of neighborhood. make span bigger
```

```
## Warning in simpleLoess(y, x, w, span, degree = degree, parametric = parametric, : pseudoinverse used at -0.005
```

```
## Warning in simpleLoess(y, x, w, span, degree = degree, parametric = parametric, : neighborhood radius 0.005
```

```
## Warning in simpleLoess(y, x, w, span, degree = degree, parametric = parametric, : reciprocal condition number 1
```

```
## Warning in simpleLoess(y, x, w, span, degree = degree, parametric = parametric, : There are other near singularities as well. 1.01
```

```
## Warning in simpleLoess(y, x, w, span, degree = degree, parametric = parametric, : zero-width neighborhood. make span bigger
```

```
## Warning: Computation failed in `stat_smooth()`:
## NA/NaN/Inf in foreign function call (arg 5)
```

```
## Warning in simpleLoess(y, x, w, span, degree = degree, parametric = parametric, : pseudoinverse used at -0.005
```

```
## Warning in simpleLoess(y, x, w, span, degree = degree, parametric = parametric, : neighborhood radius 1.005
```

```
## Warning in simpleLoess(y, x, w, span, degree = degree, parametric = parametric, : reciprocal condition number 0
```

```
## Warning in simpleLoess(y, x, w, span, degree = degree, parametric = parametric, : There are other near singularities as well. 1.01
```

```
## Warning in predLoess(object$y, object$x, newx = if (is.null(newdata)) object$x else if (is.data.frame(newdata)) as.matrix(model.frame(delete.response(terms(object)), : pseudoinverse used at -0.005
```

```
## Warning in predLoess(object$y, object$x, newx = if (is.null(newdata)) object$x else if (is.data.frame(newdata)) as.matrix(model.frame(delete.response(terms(object)), : neighborhood radius 1.005
```

```
## Warning in predLoess(object$y, object$x, newx = if (is.null(newdata)) object$x else if (is.data.frame(newdata)) as.matrix(model.frame(delete.response(terms(object)), : reciprocal condition number 0
```

```
## Warning in predLoess(object$y, object$x, newx = if (is.null(newdata)) object$x else if (is.data.frame(newdata)) as.matrix(model.frame(delete.response(terms(object)), : There are other near
## singularities as well. 1.01
```

```
## Warning in simpleLoess(y, x, w, span, degree = degree, parametric = parametric, : at -0.005
```

```
## Warning in simpleLoess(y, x, w, span, degree = degree, parametric = parametric, : radius 2.5e-05
```

```
## Warning in simpleLoess(y, x, w, span, degree = degree, parametric = parametric, : all data on boundary of neighborhood. make span bigger
```

```
## Warning in simpleLoess(y, x, w, span, degree = degree, parametric = parametric, : pseudoinverse used at -0.005
```

```
## Warning in simpleLoess(y, x, w, span, degree = degree, parametric = parametric, : neighborhood radius 0.005
```

```
## Warning in simpleLoess(y, x, w, span, degree = degree, parametric = parametric, : reciprocal condition number 1
```

```
## Warning in simpleLoess(y, x, w, span, degree = degree, parametric = parametric, : There are other near singularities as well. 1.01
```

```
## Warning in simpleLoess(y, x, w, span, degree = degree, parametric = parametric, : zero-width neighborhood. make span bigger
```

```
## Warning: Computation failed in `stat_smooth()`:
## NA/NaN/Inf in foreign function call (arg 5)
```

```
## Warning in simpleLoess(y, x, w, span, degree = degree, parametric = parametric, : pseudoinverse used at -0.005
```

```
## Warning in simpleLoess(y, x, w, span, degree = degree, parametric = parametric, : neighborhood radius 1.005
```

```
## Warning in simpleLoess(y, x, w, span, degree = degree, parametric = parametric, : reciprocal condition number 0
```

```
## Warning in simpleLoess(y, x, w, span, degree = degree, parametric = parametric, : There are other near singularities as well. 1.01
```

```
## Warning in predLoess(object$y, object$x, newx = if (is.null(newdata)) object$x else if (is.data.frame(newdata)) as.matrix(model.frame(delete.response(terms(object)), : pseudoinverse used at -0.005
```

```
## Warning in predLoess(object$y, object$x, newx = if (is.null(newdata)) object$x else if (is.data.frame(newdata)) as.matrix(model.frame(delete.response(terms(object)), : neighborhood radius 1.005
```

```
## Warning in predLoess(object$y, object$x, newx = if (is.null(newdata)) object$x else if (is.data.frame(newdata)) as.matrix(model.frame(delete.response(terms(object)), : reciprocal condition number 0
```

```
## Warning in predLoess(object$y, object$x, newx = if (is.null(newdata)) object$x else if (is.data.frame(newdata)) as.matrix(model.frame(delete.response(terms(object)), : There are other near
## singularities as well. 1.01
```

```
## Warning in simpleLoess(y, x, w, span, degree = degree, parametric = parametric, : at -0.005
```

```
## Warning in simpleLoess(y, x, w, span, degree = degree, parametric = parametric, : radius 2.5e-05
```

```
## Warning in simpleLoess(y, x, w, span, degree = degree, parametric = parametric, : all data on boundary of neighborhood. make span bigger
```

```
## Warning in simpleLoess(y, x, w, span, degree = degree, parametric = parametric, : pseudoinverse used at -0.005
```

```
## Warning in simpleLoess(y, x, w, span, degree = degree, parametric = parametric, : neighborhood radius 0.005
```

```
## Warning in simpleLoess(y, x, w, span, degree = degree, parametric = parametric, : reciprocal condition number 1
```

```
## Warning in simpleLoess(y, x, w, span, degree = degree, parametric = parametric, : There are other near singularities as well. 1.01
```

```
## Warning in simpleLoess(y, x, w, span, degree = degree, parametric = parametric, : zero-width neighborhood. make span bigger
```

```
## Warning: Computation failed in `stat_smooth()`:
## NA/NaN/Inf in foreign function call (arg 5)
```

```
## Warning in simpleLoess(y, x, w, span, degree = degree, parametric = parametric, : at -0.005
```

```
## Warning in simpleLoess(y, x, w, span, degree = degree, parametric = parametric, : radius 2.5e-05
```

```
## Warning in simpleLoess(y, x, w, span, degree = degree, parametric = parametric, : all data on boundary of neighborhood. make span bigger
```

```
## Warning in simpleLoess(y, x, w, span, degree = degree, parametric = parametric, : pseudoinverse used at -0.005
```

```
## Warning in simpleLoess(y, x, w, span, degree = degree, parametric = parametric, : neighborhood radius 0.005
```

```
## Warning in simpleLoess(y, x, w, span, degree = degree, parametric = parametric, : reciprocal condition number 1
```

```
## Warning in simpleLoess(y, x, w, span, degree = degree, parametric = parametric, : There are other near singularities as well. 1.01
```

```
## Warning in simpleLoess(y, x, w, span, degree = degree, parametric = parametric, : zero-width neighborhood. make span bigger
```

```
## Warning: Computation failed in `stat_smooth()`:
## NA/NaN/Inf in foreign function call (arg 5)
```

```
## Warning in simpleLoess(y, x, w, span, degree = degree, parametric = parametric, : at -0.005
```

```
## Warning in simpleLoess(y, x, w, span, degree = degree, parametric = parametric, : radius 2.5e-05
```

```
## Warning in simpleLoess(y, x, w, span, degree = degree, parametric = parametric, : all data on boundary of neighborhood. make span bigger
```

```
## Warning in simpleLoess(y, x, w, span, degree = degree, parametric = parametric, : pseudoinverse used at -0.005
```

```
## Warning in simpleLoess(y, x, w, span, degree = degree, parametric = parametric, : neighborhood radius 0.005
```

```
## Warning in simpleLoess(y, x, w, span, degree = degree, parametric = parametric, : reciprocal condition number 1
```

```
## Warning in simpleLoess(y, x, w, span, degree = degree, parametric = parametric, : There are other near singularities as well. 1.01
```

```
## Warning in simpleLoess(y, x, w, span, degree = degree, parametric = parametric, : zero-width neighborhood. make span bigger
```

```
## Warning: Computation failed in `stat_smooth()`:
## NA/NaN/Inf in foreign function call (arg 5)
```

```
## Warning in simpleLoess(y, x, w, span, degree = degree, parametric = parametric, : at -0.005
```

```
## Warning in simpleLoess(y, x, w, span, degree = degree, parametric = parametric, : radius 2.5e-05
```

```
## Warning in simpleLoess(y, x, w, span, degree = degree, parametric = parametric, : all data on boundary of neighborhood. make span bigger
```

```
## Warning in simpleLoess(y, x, w, span, degree = degree, parametric = parametric, : pseudoinverse used at -0.005
```

```
## Warning in simpleLoess(y, x, w, span, degree = degree, parametric = parametric, : neighborhood radius 0.005
```

```
## Warning in simpleLoess(y, x, w, span, degree = degree, parametric = parametric, : reciprocal condition number 1
```

```
## Warning in simpleLoess(y, x, w, span, degree = degree, parametric = parametric, : There are other near singularities as well. 1.01
```

```
## Warning in simpleLoess(y, x, w, span, degree = degree, parametric = parametric, : zero-width neighborhood. make span bigger
```

```
## Warning: Computation failed in `stat_smooth()`:
## NA/NaN/Inf in foreign function call (arg 5)
```

```
## Warning in simpleLoess(y, x, w, span, degree = degree, parametric = parametric, : at -0.005
```

```
## Warning in simpleLoess(y, x, w, span, degree = degree, parametric = parametric, : radius 2.5e-05
```

```
## Warning in simpleLoess(y, x, w, span, degree = degree, parametric = parametric, : all data on boundary of neighborhood. make span bigger
```

```
## Warning in simpleLoess(y, x, w, span, degree = degree, parametric = parametric, : pseudoinverse used at -0.005
```

```
## Warning in simpleLoess(y, x, w, span, degree = degree, parametric = parametric, : neighborhood radius 0.005
```

```
## Warning in simpleLoess(y, x, w, span, degree = degree, parametric = parametric, : reciprocal condition number 1
```

```
## Warning in simpleLoess(y, x, w, span, degree = degree, parametric = parametric, : There are other near singularities as well. 1.01
```

```
## Warning in simpleLoess(y, x, w, span, degree = degree, parametric = parametric, : zero-width neighborhood. make span bigger
```

```
## Warning: Computation failed in `stat_smooth()`:
## NA/NaN/Inf in foreign function call (arg 5)
```

```
## Warning in simpleLoess(y, x, w, span, degree = degree, parametric = parametric, : at -0.005
```

```
## Warning in simpleLoess(y, x, w, span, degree = degree, parametric = parametric, : radius 2.5e-05
```

```
## Warning in simpleLoess(y, x, w, span, degree = degree, parametric = parametric, : all data on boundary of neighborhood. make span bigger
```

```
## Warning in simpleLoess(y, x, w, span, degree = degree, parametric = parametric, : pseudoinverse used at -0.005
```

```
## Warning in simpleLoess(y, x, w, span, degree = degree, parametric = parametric, : neighborhood radius 0.005
```

```
## Warning in simpleLoess(y, x, w, span, degree = degree, parametric = parametric, : reciprocal condition number 1
```

```
## Warning in simpleLoess(y, x, w, span, degree = degree, parametric = parametric, : There are other near singularities as well. 1.01
```

```
## Warning in simpleLoess(y, x, w, span, degree = degree, parametric = parametric, : zero-width neighborhood. make span bigger
```

```
## Warning: Computation failed in `stat_smooth()`:
## NA/NaN/Inf in foreign function call (arg 5)
```

```
## Warning in simpleLoess(y, x, w, span, degree = degree, parametric = parametric, : pseudoinverse used at -0.005
```

```
## Warning in simpleLoess(y, x, w, span, degree = degree, parametric = parametric, : neighborhood radius 1.005
```

```
## Warning in simpleLoess(y, x, w, span, degree = degree, parametric = parametric, : reciprocal condition number 0
```

```
## Warning in simpleLoess(y, x, w, span, degree = degree, parametric = parametric, : There are other near singularities as well. 1.01
```

```
## Warning in predLoess(object$y, object$x, newx = if (is.null(newdata)) object$x else if (is.data.frame(newdata)) as.matrix(model.frame(delete.response(terms(object)), : pseudoinverse used at -0.005
```

```
## Warning in predLoess(object$y, object$x, newx = if (is.null(newdata)) object$x else if (is.data.frame(newdata)) as.matrix(model.frame(delete.response(terms(object)), : neighborhood radius 1.005
```

```
## Warning in predLoess(object$y, object$x, newx = if (is.null(newdata)) object$x else if (is.data.frame(newdata)) as.matrix(model.frame(delete.response(terms(object)), : reciprocal condition number 0
```

```
## Warning in predLoess(object$y, object$x, newx = if (is.null(newdata)) object$x else if (is.data.frame(newdata)) as.matrix(model.frame(delete.response(terms(object)), : There are other near
## singularities as well. 1.01
```

```
## Warning in simpleLoess(y, x, w, span, degree = degree, parametric = parametric, : pseudoinverse used at -0.005
```

```
## Warning in simpleLoess(y, x, w, span, degree = degree, parametric = parametric, : neighborhood radius 1.005
```

```
## Warning in simpleLoess(y, x, w, span, degree = degree, parametric = parametric, : reciprocal condition number 0
```

```
## Warning in simpleLoess(y, x, w, span, degree = degree, parametric = parametric, : There are other near singularities as well. 1.01
```

```
## Warning in predLoess(object$y, object$x, newx = if (is.null(newdata)) object$x else if (is.data.frame(newdata)) as.matrix(model.frame(delete.response(terms(object)), : pseudoinverse used at -0.005
```

```
## Warning in predLoess(object$y, object$x, newx = if (is.null(newdata)) object$x else if (is.data.frame(newdata)) as.matrix(model.frame(delete.response(terms(object)), : neighborhood radius 1.005
```

```
## Warning in predLoess(object$y, object$x, newx = if (is.null(newdata)) object$x else if (is.data.frame(newdata)) as.matrix(model.frame(delete.response(terms(object)), : reciprocal condition number 0
```

```
## Warning in predLoess(object$y, object$x, newx = if (is.null(newdata)) object$x else if (is.data.frame(newdata)) as.matrix(model.frame(delete.response(terms(object)), : There are other near
## singularities as well. 1.01
```

```
## Warning in simpleLoess(y, x, w, span, degree = degree, parametric = parametric, : pseudoinverse used at -0.005
```

```
## Warning in simpleLoess(y, x, w, span, degree = degree, parametric = parametric, : neighborhood radius 1.005
```

```
## Warning in simpleLoess(y, x, w, span, degree = degree, parametric = parametric, : reciprocal condition number 0
```

```
## Warning in simpleLoess(y, x, w, span, degree = degree, parametric = parametric, : There are other near singularities as well. 1.01
```

```
## Warning in predLoess(object$y, object$x, newx = if (is.null(newdata)) object$x else if (is.data.frame(newdata)) as.matrix(model.frame(delete.response(terms(object)), : pseudoinverse used at -0.005
```

```
## Warning in predLoess(object$y, object$x, newx = if (is.null(newdata)) object$x else if (is.data.frame(newdata)) as.matrix(model.frame(delete.response(terms(object)), : neighborhood radius 1.005
```

```
## Warning in predLoess(object$y, object$x, newx = if (is.null(newdata)) object$x else if (is.data.frame(newdata)) as.matrix(model.frame(delete.response(terms(object)), : reciprocal condition number 0
```

```
## Warning in predLoess(object$y, object$x, newx = if (is.null(newdata)) object$x else if (is.data.frame(newdata)) as.matrix(model.frame(delete.response(terms(object)), : There are other near
## singularities as well. 1.01
```

```
## Warning in simpleLoess(y, x, w, span, degree = degree, parametric = parametric, : at -0.005
```

```
## Warning in simpleLoess(y, x, w, span, degree = degree, parametric = parametric, : radius 2.5e-05
```

```
## Warning in simpleLoess(y, x, w, span, degree = degree, parametric = parametric, : all data on boundary of neighborhood. make span bigger
```

```
## Warning in simpleLoess(y, x, w, span, degree = degree, parametric = parametric, : pseudoinverse used at -0.005
```

```
## Warning in simpleLoess(y, x, w, span, degree = degree, parametric = parametric, : neighborhood radius 0.005
```

```
## Warning in simpleLoess(y, x, w, span, degree = degree, parametric = parametric, : reciprocal condition number 1
```

```
## Warning in simpleLoess(y, x, w, span, degree = degree, parametric = parametric, : There are other near singularities as well. 1.01
```

```
## Warning in simpleLoess(y, x, w, span, degree = degree, parametric = parametric, : zero-width neighborhood. make span bigger
```

```
## Warning: Computation failed in `stat_smooth()`:
## NA/NaN/Inf in foreign function call (arg 5)
```

```
## Warning in simpleLoess(y, x, w, span, degree = degree, parametric = parametric, : at -0.005
```

```
## Warning in simpleLoess(y, x, w, span, degree = degree, parametric = parametric, : radius 2.5e-05
```

```
## Warning in simpleLoess(y, x, w, span, degree = degree, parametric = parametric, : all data on boundary of neighborhood. make span bigger
```

```
## Warning in simpleLoess(y, x, w, span, degree = degree, parametric = parametric, : pseudoinverse used at -0.005
```

```
## Warning in simpleLoess(y, x, w, span, degree = degree, parametric = parametric, : neighborhood radius 0.005
```

```
## Warning in simpleLoess(y, x, w, span, degree = degree, parametric = parametric, : reciprocal condition number 1
```

```
## Warning in simpleLoess(y, x, w, span, degree = degree, parametric = parametric, : There are other near singularities as well. 1.01
```

```
## Warning in simpleLoess(y, x, w, span, degree = degree, parametric = parametric, : zero-width neighborhood. make span bigger
```

```
## Warning: Computation failed in `stat_smooth()`:
## NA/NaN/Inf in foreign function call (arg 5)
```

```
## Warning in simpleLoess(y, x, w, span, degree = degree, parametric = parametric, : at -0.005
```

```
## Warning in simpleLoess(y, x, w, span, degree = degree, parametric = parametric, : radius 2.5e-05
```

```
## Warning in simpleLoess(y, x, w, span, degree = degree, parametric = parametric, : all data on boundary of neighborhood. make span bigger
```

```
## Warning in simpleLoess(y, x, w, span, degree = degree, parametric = parametric, : pseudoinverse used at -0.005
```

```
## Warning in simpleLoess(y, x, w, span, degree = degree, parametric = parametric, : neighborhood radius 0.005
```

```
## Warning in simpleLoess(y, x, w, span, degree = degree, parametric = parametric, : reciprocal condition number 1
```

```
## Warning in simpleLoess(y, x, w, span, degree = degree, parametric = parametric, : There are other near singularities as well. 1.01
```

```
## Warning in simpleLoess(y, x, w, span, degree = degree, parametric = parametric, : zero-width neighborhood. make span bigger
```

```
## Warning: Computation failed in `stat_smooth()`:
## NA/NaN/Inf in foreign function call (arg 5)
```

```
## Warning in simpleLoess(y, x, w, span, degree = degree, parametric = parametric, : at -0.005
```

```
## Warning in simpleLoess(y, x, w, span, degree = degree, parametric = parametric, : radius 2.5e-05
```

```
## Warning in simpleLoess(y, x, w, span, degree = degree, parametric = parametric, : all data on boundary of neighborhood. make span bigger
```

```
## Warning in simpleLoess(y, x, w, span, degree = degree, parametric = parametric, : pseudoinverse used at -0.005
```

```
## Warning in simpleLoess(y, x, w, span, degree = degree, parametric = parametric, : neighborhood radius 0.005
```

```
## Warning in simpleLoess(y, x, w, span, degree = degree, parametric = parametric, : reciprocal condition number 1
```

```
## Warning in simpleLoess(y, x, w, span, degree = degree, parametric = parametric, : There are other near singularities as well. 1.01
```

```
## Warning in simpleLoess(y, x, w, span, degree = degree, parametric = parametric, : zero-width neighborhood. make span bigger
```

```
## Warning: Computation failed in `stat_smooth()`:
## NA/NaN/Inf in foreign function call (arg 5)
```

```
## Warning in simpleLoess(y, x, w, span, degree = degree, parametric = parametric, : at -0.005
```

```
## Warning in simpleLoess(y, x, w, span, degree = degree, parametric = parametric, : radius 2.5e-05
```

```
## Warning in simpleLoess(y, x, w, span, degree = degree, parametric = parametric, : all data on boundary of neighborhood. make span bigger
```

```
## Warning in simpleLoess(y, x, w, span, degree = degree, parametric = parametric, : pseudoinverse used at -0.005
```

```
## Warning in simpleLoess(y, x, w, span, degree = degree, parametric = parametric, : neighborhood radius 0.005
```

```
## Warning in simpleLoess(y, x, w, span, degree = degree, parametric = parametric, : reciprocal condition number 1
```

```
## Warning in simpleLoess(y, x, w, span, degree = degree, parametric = parametric, : There are other near singularities as well. 1.01
```

```
## Warning in simpleLoess(y, x, w, span, degree = degree, parametric = parametric, : zero-width neighborhood. make span bigger
```

```
## Warning: Computation failed in `stat_smooth()`:
## NA/NaN/Inf in foreign function call (arg 5)
```

```
## Warning in simpleLoess(y, x, w, span, degree = degree, parametric = parametric, : pseudoinverse used at -0.005
```

```
## Warning in simpleLoess(y, x, w, span, degree = degree, parametric = parametric, : neighborhood radius 1.005
```

```
## Warning in simpleLoess(y, x, w, span, degree = degree, parametric = parametric, : reciprocal condition number 0
```

```
## Warning in simpleLoess(y, x, w, span, degree = degree, parametric = parametric, : There are other near singularities as well. 1.01
```

```
## Warning in predLoess(object$y, object$x, newx = if (is.null(newdata)) object$x else if (is.data.frame(newdata)) as.matrix(model.frame(delete.response(terms(object)), : pseudoinverse used at -0.005
```

```
## Warning in predLoess(object$y, object$x, newx = if (is.null(newdata)) object$x else if (is.data.frame(newdata)) as.matrix(model.frame(delete.response(terms(object)), : neighborhood radius 1.005
```

```
## Warning in predLoess(object$y, object$x, newx = if (is.null(newdata)) object$x else if (is.data.frame(newdata)) as.matrix(model.frame(delete.response(terms(object)), : reciprocal condition number 0
```

```
## Warning in predLoess(object$y, object$x, newx = if (is.null(newdata)) object$x else if (is.data.frame(newdata)) as.matrix(model.frame(delete.response(terms(object)), : There are other near
## singularities as well. 1.01
```

```
## Warning in simpleLoess(y, x, w, span, degree = degree, parametric = parametric, : pseudoinverse used at -0.005
```

```
## Warning in simpleLoess(y, x, w, span, degree = degree, parametric = parametric, : neighborhood radius 1.005
```

```
## Warning in simpleLoess(y, x, w, span, degree = degree, parametric = parametric, : reciprocal condition number 0
```

```
## Warning in simpleLoess(y, x, w, span, degree = degree, parametric = parametric, : There are other near singularities as well. 1.01
```

```
## Warning in predLoess(object$y, object$x, newx = if (is.null(newdata)) object$x else if (is.data.frame(newdata)) as.matrix(model.frame(delete.response(terms(object)), : pseudoinverse used at -0.005
```

```
## Warning in predLoess(object$y, object$x, newx = if (is.null(newdata)) object$x else if (is.data.frame(newdata)) as.matrix(model.frame(delete.response(terms(object)), : neighborhood radius 1.005
```

```
## Warning in predLoess(object$y, object$x, newx = if (is.null(newdata)) object$x else if (is.data.frame(newdata)) as.matrix(model.frame(delete.response(terms(object)), : reciprocal condition number 0
```

```
## Warning in predLoess(object$y, object$x, newx = if (is.null(newdata)) object$x else if (is.data.frame(newdata)) as.matrix(model.frame(delete.response(terms(object)), : There are other near
## singularities as well. 1.01
```

```
## Warning in simpleLoess(y, x, w, span, degree = degree, parametric = parametric, : at -0.005
```

```
## Warning in simpleLoess(y, x, w, span, degree = degree, parametric = parametric, : radius 2.5e-05
```

```
## Warning in simpleLoess(y, x, w, span, degree = degree, parametric = parametric, : all data on boundary of neighborhood. make span bigger
```

```
## Warning in simpleLoess(y, x, w, span, degree = degree, parametric = parametric, : pseudoinverse used at -0.005
```

```
## Warning in simpleLoess(y, x, w, span, degree = degree, parametric = parametric, : neighborhood radius 0.005
```

```
## Warning in simpleLoess(y, x, w, span, degree = degree, parametric = parametric, : reciprocal condition number 1
```

```
## Warning in simpleLoess(y, x, w, span, degree = degree, parametric = parametric, : There are other near singularities as well. 1.01
```

```
## Warning in simpleLoess(y, x, w, span, degree = degree, parametric = parametric, : zero-width neighborhood. make span bigger
```

```
## Warning: Computation failed in `stat_smooth()`:
## NA/NaN/Inf in foreign function call (arg 5)
```

```
## Warning in simpleLoess(y, x, w, span, degree = degree, parametric = parametric, : at -0.005
```

```
## Warning in simpleLoess(y, x, w, span, degree = degree, parametric = parametric, : radius 2.5e-05
```

```
## Warning in simpleLoess(y, x, w, span, degree = degree, parametric = parametric, : all data on boundary of neighborhood. make span bigger
```

```
## Warning in simpleLoess(y, x, w, span, degree = degree, parametric = parametric, : pseudoinverse used at -0.005
```

```
## Warning in simpleLoess(y, x, w, span, degree = degree, parametric = parametric, : neighborhood radius 0.005
```

```
## Warning in simpleLoess(y, x, w, span, degree = degree, parametric = parametric, : reciprocal condition number 1
```

```
## Warning in simpleLoess(y, x, w, span, degree = degree, parametric = parametric, : There are other near singularities as well. 1.01
```

```
## Warning in simpleLoess(y, x, w, span, degree = degree, parametric = parametric, : zero-width neighborhood. make span bigger
```

```
## Warning: Computation failed in `stat_smooth()`:
## NA/NaN/Inf in foreign function call (arg 5)
```

```
## Warning in simpleLoess(y, x, w, span, degree = degree, parametric = parametric, : at -0.005
```

```
## Warning in simpleLoess(y, x, w, span, degree = degree, parametric = parametric, : radius 2.5e-05
```

```
## Warning in simpleLoess(y, x, w, span, degree = degree, parametric = parametric, : all data on boundary of neighborhood. make span bigger
```

```
## Warning in simpleLoess(y, x, w, span, degree = degree, parametric = parametric, : pseudoinverse used at -0.005
```

```
## Warning in simpleLoess(y, x, w, span, degree = degree, parametric = parametric, : neighborhood radius 0.005
```

```
## Warning in simpleLoess(y, x, w, span, degree = degree, parametric = parametric, : reciprocal condition number 1
```

```
## Warning in simpleLoess(y, x, w, span, degree = degree, parametric = parametric, : There are other near singularities as well. 1.01
```

```
## Warning in simpleLoess(y, x, w, span, degree = degree, parametric = parametric, : zero-width neighborhood. make span bigger
```

```
## Warning: Computation failed in `stat_smooth()`:
## NA/NaN/Inf in foreign function call (arg 5)
```

```
## Warning in simpleLoess(y, x, w, span, degree = degree, parametric = parametric, : at -0.005
```

```
## Warning in simpleLoess(y, x, w, span, degree = degree, parametric = parametric, : radius 2.5e-05
```

```
## Warning in simpleLoess(y, x, w, span, degree = degree, parametric = parametric, : all data on boundary of neighborhood. make span bigger
```

```
## Warning in simpleLoess(y, x, w, span, degree = degree, parametric = parametric, : pseudoinverse used at -0.005
```

```
## Warning in simpleLoess(y, x, w, span, degree = degree, parametric = parametric, : neighborhood radius 0.005
```

```
## Warning in simpleLoess(y, x, w, span, degree = degree, parametric = parametric, : reciprocal condition number 1
```

```
## Warning in simpleLoess(y, x, w, span, degree = degree, parametric = parametric, : There are other near singularities as well. 1.01
```

```
## Warning in simpleLoess(y, x, w, span, degree = degree, parametric = parametric, : zero-width neighborhood. make span bigger
```

```
## Warning: Computation failed in `stat_smooth()`:
## NA/NaN/Inf in foreign function call (arg 5)
```

```
## Warning in simpleLoess(y, x, w, span, degree = degree, parametric = parametric, : at -0.005
```

```
## Warning in simpleLoess(y, x, w, span, degree = degree, parametric = parametric, : radius 2.5e-05
```

```
## Warning in simpleLoess(y, x, w, span, degree = degree, parametric = parametric, : all data on boundary of neighborhood. make span bigger
```

```
## Warning in simpleLoess(y, x, w, span, degree = degree, parametric = parametric, : pseudoinverse used at -0.005
```

```
## Warning in simpleLoess(y, x, w, span, degree = degree, parametric = parametric, : neighborhood radius 0.005
```

```
## Warning in simpleLoess(y, x, w, span, degree = degree, parametric = parametric, : reciprocal condition number 1
```

```
## Warning in simpleLoess(y, x, w, span, degree = degree, parametric = parametric, : There are other near singularities as well. 1.01
```

```
## Warning in simpleLoess(y, x, w, span, degree = degree, parametric = parametric, : zero-width neighborhood. make span bigger
```

```
## Warning: Computation failed in `stat_smooth()`:
## NA/NaN/Inf in foreign function call (arg 5)
```

```
## Warning in simpleLoess(y, x, w, span, degree = degree, parametric = parametric, : at -0.005
```

```
## Warning in simpleLoess(y, x, w, span, degree = degree, parametric = parametric, : radius 2.5e-05
```

```
## Warning in simpleLoess(y, x, w, span, degree = degree, parametric = parametric, : all data on boundary of neighborhood. make span bigger
```

```
## Warning in simpleLoess(y, x, w, span, degree = degree, parametric = parametric, : pseudoinverse used at -0.005
```

```
## Warning in simpleLoess(y, x, w, span, degree = degree, parametric = parametric, : neighborhood radius 0.005
```

```
## Warning in simpleLoess(y, x, w, span, degree = degree, parametric = parametric, : reciprocal condition number 1
```

```
## Warning in simpleLoess(y, x, w, span, degree = degree, parametric = parametric, : There are other near singularities as well. 1.01
```

```
## Warning in simpleLoess(y, x, w, span, degree = degree, parametric = parametric, : zero-width neighborhood. make span bigger
```

```
## Warning: Computation failed in `stat_smooth()`:
## NA/NaN/Inf in foreign function call (arg 5)
```

```
## Warning in simpleLoess(y, x, w, span, degree = degree, parametric = parametric, : at -0.005
```

```
## Warning in simpleLoess(y, x, w, span, degree = degree, parametric = parametric, : radius 2.5e-05
```

```
## Warning in simpleLoess(y, x, w, span, degree = degree, parametric = parametric, : all data on boundary of neighborhood. make span bigger
```

```
## Warning in simpleLoess(y, x, w, span, degree = degree, parametric = parametric, : pseudoinverse used at -0.005
```

```
## Warning in simpleLoess(y, x, w, span, degree = degree, parametric = parametric, : neighborhood radius 0.005
```

```
## Warning in simpleLoess(y, x, w, span, degree = degree, parametric = parametric, : reciprocal condition number 1
```

```
## Warning in simpleLoess(y, x, w, span, degree = degree, parametric = parametric, : There are other near singularities as well. 1.01
```

```
## Warning in simpleLoess(y, x, w, span, degree = degree, parametric = parametric, : zero-width neighborhood. make span bigger
```

```
## Warning: Computation failed in `stat_smooth()`:
## NA/NaN/Inf in foreign function call (arg 5)
```

```
## Warning in simpleLoess(y, x, w, span, degree = degree, parametric = parametric, : at -0.005
```

```
## Warning in simpleLoess(y, x, w, span, degree = degree, parametric = parametric, : radius 2.5e-05
```

```
## Warning in simpleLoess(y, x, w, span, degree = degree, parametric = parametric, : all data on boundary of neighborhood. make span bigger
```

```
## Warning in simpleLoess(y, x, w, span, degree = degree, parametric = parametric, : pseudoinverse used at -0.005
```

```
## Warning in simpleLoess(y, x, w, span, degree = degree, parametric = parametric, : neighborhood radius 0.005
```

```
## Warning in simpleLoess(y, x, w, span, degree = degree, parametric = parametric, : reciprocal condition number 1
```

```
## Warning in simpleLoess(y, x, w, span, degree = degree, parametric = parametric, : There are other near singularities as well. 1.01
```

```
## Warning in simpleLoess(y, x, w, span, degree = degree, parametric = parametric, : zero-width neighborhood. make span bigger
```

```
## Warning: Computation failed in `stat_smooth()`:
## NA/NaN/Inf in foreign function call (arg 5)
```

```
## Warning in simpleLoess(y, x, w, span, degree = degree, parametric = parametric, : at -0.005
```

```
## Warning in simpleLoess(y, x, w, span, degree = degree, parametric = parametric, : radius 2.5e-05
```

```
## Warning in simpleLoess(y, x, w, span, degree = degree, parametric = parametric, : all data on boundary of neighborhood. make span bigger
```

```
## Warning in simpleLoess(y, x, w, span, degree = degree, parametric = parametric, : pseudoinverse used at -0.005
```

```
## Warning in simpleLoess(y, x, w, span, degree = degree, parametric = parametric, : neighborhood radius 0.005
```

```
## Warning in simpleLoess(y, x, w, span, degree = degree, parametric = parametric, : reciprocal condition number 1
```

```
## Warning in simpleLoess(y, x, w, span, degree = degree, parametric = parametric, : There are other near singularities as well. 1.01
```

```
## Warning in simpleLoess(y, x, w, span, degree = degree, parametric = parametric, : zero-width neighborhood. make span bigger
```

```
## Warning: Computation failed in `stat_smooth()`:
## NA/NaN/Inf in foreign function call (arg 5)
```

```
## Warning in simpleLoess(y, x, w, span, degree = degree, parametric = parametric, : at -0.005
```

```
## Warning in simpleLoess(y, x, w, span, degree = degree, parametric = parametric, : radius 2.5e-05
```

```
## Warning in simpleLoess(y, x, w, span, degree = degree, parametric = parametric, : all data on boundary of neighborhood. make span bigger
```

```
## Warning in simpleLoess(y, x, w, span, degree = degree, parametric = parametric, : pseudoinverse used at -0.005
```

```
## Warning in simpleLoess(y, x, w, span, degree = degree, parametric = parametric, : neighborhood radius 0.005
```

```
## Warning in simpleLoess(y, x, w, span, degree = degree, parametric = parametric, : reciprocal condition number 1
```

```
## Warning in simpleLoess(y, x, w, span, degree = degree, parametric = parametric, : There are other near singularities as well. 1.01
```

```
## Warning in simpleLoess(y, x, w, span, degree = degree, parametric = parametric, : zero-width neighborhood. make span bigger
```

```
## Warning: Computation failed in `stat_smooth()`:
## NA/NaN/Inf in foreign function call (arg 5)
```

```
## Warning in simpleLoess(y, x, w, span, degree = degree, parametric = parametric, : at -0.005
```

```
## Warning in simpleLoess(y, x, w, span, degree = degree, parametric = parametric, : radius 2.5e-05
```

```
## Warning in simpleLoess(y, x, w, span, degree = degree, parametric = parametric, : all data on boundary of neighborhood. make span bigger
```

```
## Warning in simpleLoess(y, x, w, span, degree = degree, parametric = parametric, : pseudoinverse used at -0.005
```

```
## Warning in simpleLoess(y, x, w, span, degree = degree, parametric = parametric, : neighborhood radius 0.005
```

```
## Warning in simpleLoess(y, x, w, span, degree = degree, parametric = parametric, : reciprocal condition number 1
```

```
## Warning in simpleLoess(y, x, w, span, degree = degree, parametric = parametric, : There are other near singularities as well. 1.01
```

```
## Warning in simpleLoess(y, x, w, span, degree = degree, parametric = parametric, : zero-width neighborhood. make span bigger
```

```
## Warning: Computation failed in `stat_smooth()`:
## NA/NaN/Inf in foreign function call (arg 5)
```

```
## Warning in simpleLoess(y, x, w, span, degree = degree, parametric = parametric, : at -0.005
```

```
## Warning in simpleLoess(y, x, w, span, degree = degree, parametric = parametric, : radius 2.5e-05
```

```
## Warning in simpleLoess(y, x, w, span, degree = degree, parametric = parametric, : all data on boundary of neighborhood. make span bigger
```

```
## Warning in simpleLoess(y, x, w, span, degree = degree, parametric = parametric, : pseudoinverse used at -0.005
```

```
## Warning in simpleLoess(y, x, w, span, degree = degree, parametric = parametric, : neighborhood radius 0.005
```

```
## Warning in simpleLoess(y, x, w, span, degree = degree, parametric = parametric, : reciprocal condition number 1
```

```
## Warning in simpleLoess(y, x, w, span, degree = degree, parametric = parametric, : There are other near singularities as well. 1.01
```

```
## Warning in simpleLoess(y, x, w, span, degree = degree, parametric = parametric, : zero-width neighborhood. make span bigger
```

```
## Warning: Computation failed in `stat_smooth()`:
## NA/NaN/Inf in foreign function call (arg 5)
```

```
## Warning in simpleLoess(y, x, w, span, degree = degree, parametric = parametric, : at -0.005
```

```
## Warning in simpleLoess(y, x, w, span, degree = degree, parametric = parametric, : radius 2.5e-05
```

```
## Warning in simpleLoess(y, x, w, span, degree = degree, parametric = parametric, : all data on boundary of neighborhood. make span bigger
```

```
## Warning in simpleLoess(y, x, w, span, degree = degree, parametric = parametric, : pseudoinverse used at -0.005
```

```
## Warning in simpleLoess(y, x, w, span, degree = degree, parametric = parametric, : neighborhood radius 0.005
```

```
## Warning in simpleLoess(y, x, w, span, degree = degree, parametric = parametric, : reciprocal condition number 1
```

```
## Warning in simpleLoess(y, x, w, span, degree = degree, parametric = parametric, : There are other near singularities as well. 1.01
```

```
## Warning in simpleLoess(y, x, w, span, degree = degree, parametric = parametric, : zero-width neighborhood. make span bigger
```

```
## Warning: Computation failed in `stat_smooth()`:
## NA/NaN/Inf in foreign function call (arg 5)
```

```
## Warning in simpleLoess(y, x, w, span, degree = degree, parametric = parametric, : at -0.005
```

```
## Warning in simpleLoess(y, x, w, span, degree = degree, parametric = parametric, : radius 2.5e-05
```

```
## Warning in simpleLoess(y, x, w, span, degree = degree, parametric = parametric, : all data on boundary of neighborhood. make span bigger
```

```
## Warning in simpleLoess(y, x, w, span, degree = degree, parametric = parametric, : pseudoinverse used at -0.005
```

```
## Warning in simpleLoess(y, x, w, span, degree = degree, parametric = parametric, : neighborhood radius 0.005
```

```
## Warning in simpleLoess(y, x, w, span, degree = degree, parametric = parametric, : reciprocal condition number 1
```

```
## Warning in simpleLoess(y, x, w, span, degree = degree, parametric = parametric, : There are other near singularities as well. 1.01
```

```
## Warning in simpleLoess(y, x, w, span, degree = degree, parametric = parametric, : zero-width neighborhood. make span bigger
```

```
## Warning: Computation failed in `stat_smooth()`:
## NA/NaN/Inf in foreign function call (arg 5)
```

```
## Warning in simpleLoess(y, x, w, span, degree = degree, parametric = parametric, : pseudoinverse used at -0.005
```

```
## Warning in simpleLoess(y, x, w, span, degree = degree, parametric = parametric, : neighborhood radius 1.005
```

```
## Warning in simpleLoess(y, x, w, span, degree = degree, parametric = parametric, : reciprocal condition number 0
```

```
## Warning in simpleLoess(y, x, w, span, degree = degree, parametric = parametric, : There are other near singularities as well. 1.01
```

```
## Warning in predLoess(object$y, object$x, newx = if (is.null(newdata)) object$x else if (is.data.frame(newdata)) as.matrix(model.frame(delete.response(terms(object)), : pseudoinverse used at -0.005
```

```
## Warning in predLoess(object$y, object$x, newx = if (is.null(newdata)) object$x else if (is.data.frame(newdata)) as.matrix(model.frame(delete.response(terms(object)), : neighborhood radius 1.005
```

```
## Warning in predLoess(object$y, object$x, newx = if (is.null(newdata)) object$x else if (is.data.frame(newdata)) as.matrix(model.frame(delete.response(terms(object)), : reciprocal condition number 0
```

```
## Warning in predLoess(object$y, object$x, newx = if (is.null(newdata)) object$x else if (is.data.frame(newdata)) as.matrix(model.frame(delete.response(terms(object)), : There are other near
## singularities as well. 1.01
```

```
## Warning in simpleLoess(y, x, w, span, degree = degree, parametric = parametric, : at -0.005
```

```
## Warning in simpleLoess(y, x, w, span, degree = degree, parametric = parametric, : radius 2.5e-05
```

```
## Warning in simpleLoess(y, x, w, span, degree = degree, parametric = parametric, : all data on boundary of neighborhood. make span bigger
```

```
## Warning in simpleLoess(y, x, w, span, degree = degree, parametric = parametric, : pseudoinverse used at -0.005
```

```
## Warning in simpleLoess(y, x, w, span, degree = degree, parametric = parametric, : neighborhood radius 0.005
```

```
## Warning in simpleLoess(y, x, w, span, degree = degree, parametric = parametric, : reciprocal condition number 1
```

```
## Warning in simpleLoess(y, x, w, span, degree = degree, parametric = parametric, : There are other near singularities as well. 1.01
```

```
## Warning in simpleLoess(y, x, w, span, degree = degree, parametric = parametric, : zero-width neighborhood. make span bigger
```

```
## Warning: Computation failed in `stat_smooth()`:
## NA/NaN/Inf in foreign function call (arg 5)
```

```
## Warning in simpleLoess(y, x, w, span, degree = degree, parametric = parametric, : at -0.005
```

```
## Warning in simpleLoess(y, x, w, span, degree = degree, parametric = parametric, : radius 2.5e-05
```

```
## Warning in simpleLoess(y, x, w, span, degree = degree, parametric = parametric, : all data on boundary of neighborhood. make span bigger
```

```
## Warning in simpleLoess(y, x, w, span, degree = degree, parametric = parametric, : pseudoinverse used at -0.005
```

```
## Warning in simpleLoess(y, x, w, span, degree = degree, parametric = parametric, : neighborhood radius 0.005
```

```
## Warning in simpleLoess(y, x, w, span, degree = degree, parametric = parametric, : reciprocal condition number 1
```

```
## Warning in simpleLoess(y, x, w, span, degree = degree, parametric = parametric, : There are other near singularities as well. 1.01
```

```
## Warning in simpleLoess(y, x, w, span, degree = degree, parametric = parametric, : zero-width neighborhood. make span bigger
```

```
## Warning: Computation failed in `stat_smooth()`:
## NA/NaN/Inf in foreign function call (arg 5)
```

```
## Warning in simpleLoess(y, x, w, span, degree = degree, parametric = parametric, : at -0.005
```

```
## Warning in simpleLoess(y, x, w, span, degree = degree, parametric = parametric, : radius 2.5e-05
```

```
## Warning in simpleLoess(y, x, w, span, degree = degree, parametric = parametric, : all data on boundary of neighborhood. make span bigger
```

```
## Warning in simpleLoess(y, x, w, span, degree = degree, parametric = parametric, : pseudoinverse used at -0.005
```

```
## Warning in simpleLoess(y, x, w, span, degree = degree, parametric = parametric, : neighborhood radius 0.005
```

```
## Warning in simpleLoess(y, x, w, span, degree = degree, parametric = parametric, : reciprocal condition number 1
```

```
## Warning in simpleLoess(y, x, w, span, degree = degree, parametric = parametric, : There are other near singularities as well. 1.01
```

```
## Warning in simpleLoess(y, x, w, span, degree = degree, parametric = parametric, : zero-width neighborhood. make span bigger
```

```
## Warning: Computation failed in `stat_smooth()`:
## NA/NaN/Inf in foreign function call (arg 5)
```

```
## Warning in simpleLoess(y, x, w, span, degree = degree, parametric = parametric, : at -0.005
```

```
## Warning in simpleLoess(y, x, w, span, degree = degree, parametric = parametric, : radius 2.5e-05
```

```
## Warning in simpleLoess(y, x, w, span, degree = degree, parametric = parametric, : all data on boundary of neighborhood. make span bigger
```

```
## Warning in simpleLoess(y, x, w, span, degree = degree, parametric = parametric, : pseudoinverse used at -0.005
```

```
## Warning in simpleLoess(y, x, w, span, degree = degree, parametric = parametric, : neighborhood radius 0.005
```

```
## Warning in simpleLoess(y, x, w, span, degree = degree, parametric = parametric, : reciprocal condition number 1
```

```
## Warning in simpleLoess(y, x, w, span, degree = degree, parametric = parametric, : There are other near singularities as well. 1.01
```

```
## Warning in simpleLoess(y, x, w, span, degree = degree, parametric = parametric, : zero-width neighborhood. make span bigger
```

```
## Warning: Computation failed in `stat_smooth()`:
## NA/NaN/Inf in foreign function call (arg 5)
```

```
## Warning in simpleLoess(y, x, w, span, degree = degree, parametric = parametric, : at -0.005
```

```
## Warning in simpleLoess(y, x, w, span, degree = degree, parametric = parametric, : radius 2.5e-05
```

```
## Warning in simpleLoess(y, x, w, span, degree = degree, parametric = parametric, : all data on boundary of neighborhood. make span bigger
```

```
## Warning in simpleLoess(y, x, w, span, degree = degree, parametric = parametric, : pseudoinverse used at -0.005
```

```
## Warning in simpleLoess(y, x, w, span, degree = degree, parametric = parametric, : neighborhood radius 0.005
```

```
## Warning in simpleLoess(y, x, w, span, degree = degree, parametric = parametric, : reciprocal condition number 1
```

```
## Warning in simpleLoess(y, x, w, span, degree = degree, parametric = parametric, : There are other near singularities as well. 1.01
```

```
## Warning in simpleLoess(y, x, w, span, degree = degree, parametric = parametric, : zero-width neighborhood. make span bigger
```

```
## Warning: Computation failed in `stat_smooth()`:
## NA/NaN/Inf in foreign function call (arg 5)
```

```
## Warning in simpleLoess(y, x, w, span, degree = degree, parametric = parametric, : at -0.005
```

```
## Warning in simpleLoess(y, x, w, span, degree = degree, parametric = parametric, : radius 2.5e-05
```

```
## Warning in simpleLoess(y, x, w, span, degree = degree, parametric = parametric, : all data on boundary of neighborhood. make span bigger
```

```
## Warning in simpleLoess(y, x, w, span, degree = degree, parametric = parametric, : pseudoinverse used at -0.005
```

```
## Warning in simpleLoess(y, x, w, span, degree = degree, parametric = parametric, : neighborhood radius 0.005
```

```
## Warning in simpleLoess(y, x, w, span, degree = degree, parametric = parametric, : reciprocal condition number 1
```

```
## Warning in simpleLoess(y, x, w, span, degree = degree, parametric = parametric, : There are other near singularities as well. 1.01
```

```
## Warning in simpleLoess(y, x, w, span, degree = degree, parametric = parametric, : zero-width neighborhood. make span bigger
```

```
## Warning: Computation failed in `stat_smooth()`:
## NA/NaN/Inf in foreign function call (arg 5)
```

```
## Warning in simpleLoess(y, x, w, span, degree = degree, parametric = parametric, : at -0.005
```

```
## Warning in simpleLoess(y, x, w, span, degree = degree, parametric = parametric, : radius 2.5e-05
```

```
## Warning in simpleLoess(y, x, w, span, degree = degree, parametric = parametric, : all data on boundary of neighborhood. make span bigger
```

```
## Warning in simpleLoess(y, x, w, span, degree = degree, parametric = parametric, : pseudoinverse used at -0.005
```

```
## Warning in simpleLoess(y, x, w, span, degree = degree, parametric = parametric, : neighborhood radius 0.005
```

```
## Warning in simpleLoess(y, x, w, span, degree = degree, parametric = parametric, : reciprocal condition number 1
```

```
## Warning in simpleLoess(y, x, w, span, degree = degree, parametric = parametric, : There are other near singularities as well. 1.01
```

```
## Warning in simpleLoess(y, x, w, span, degree = degree, parametric = parametric, : zero-width neighborhood. make span bigger
```

```
## Warning: Computation failed in `stat_smooth()`:
## NA/NaN/Inf in foreign function call (arg 5)
```

```
## Warning in simpleLoess(y, x, w, span, degree = degree, parametric = parametric, : at -0.005
```

```
## Warning in simpleLoess(y, x, w, span, degree = degree, parametric = parametric, : radius 2.5e-05
```

```
## Warning in simpleLoess(y, x, w, span, degree = degree, parametric = parametric, : all data on boundary of neighborhood. make span bigger
```

```
## Warning in simpleLoess(y, x, w, span, degree = degree, parametric = parametric, : pseudoinverse used at -0.005
```

```
## Warning in simpleLoess(y, x, w, span, degree = degree, parametric = parametric, : neighborhood radius 0.005
```

```
## Warning in simpleLoess(y, x, w, span, degree = degree, parametric = parametric, : reciprocal condition number 1
```

```
## Warning in simpleLoess(y, x, w, span, degree = degree, parametric = parametric, : There are other near singularities as well. 1.01
```

```
## Warning in simpleLoess(y, x, w, span, degree = degree, parametric = parametric, : zero-width neighborhood. make span bigger
```

```
## Warning: Computation failed in `stat_smooth()`:
## NA/NaN/Inf in foreign function call (arg 5)
```

```
## Warning in simpleLoess(y, x, w, span, degree = degree, parametric = parametric, : at -0.005
```

```
## Warning in simpleLoess(y, x, w, span, degree = degree, parametric = parametric, : radius 2.5e-05
```

```
## Warning in simpleLoess(y, x, w, span, degree = degree, parametric = parametric, : all data on boundary of neighborhood. make span bigger
```

```
## Warning in simpleLoess(y, x, w, span, degree = degree, parametric = parametric, : pseudoinverse used at -0.005
```

```
## Warning in simpleLoess(y, x, w, span, degree = degree, parametric = parametric, : neighborhood radius 0.005
```

```
## Warning in simpleLoess(y, x, w, span, degree = degree, parametric = parametric, : reciprocal condition number 1
```

```
## Warning in simpleLoess(y, x, w, span, degree = degree, parametric = parametric, : There are other near singularities as well. 1.01
```

```
## Warning in simpleLoess(y, x, w, span, degree = degree, parametric = parametric, : zero-width neighborhood. make span bigger
```

```
## Warning: Computation failed in `stat_smooth()`:
## NA/NaN/Inf in foreign function call (arg 5)
```

```
## Warning in simpleLoess(y, x, w, span, degree = degree, parametric = parametric, : at -0.005
```

```
## Warning in simpleLoess(y, x, w, span, degree = degree, parametric = parametric, : radius 2.5e-05
```

```
## Warning in simpleLoess(y, x, w, span, degree = degree, parametric = parametric, : all data on boundary of neighborhood. make span bigger
```

```
## Warning in simpleLoess(y, x, w, span, degree = degree, parametric = parametric, : pseudoinverse used at -0.005
```

```
## Warning in simpleLoess(y, x, w, span, degree = degree, parametric = parametric, : neighborhood radius 0.005
```

```
## Warning in simpleLoess(y, x, w, span, degree = degree, parametric = parametric, : reciprocal condition number 1
```

```
## Warning in simpleLoess(y, x, w, span, degree = degree, parametric = parametric, : There are other near singularities as well. 1.01
```

```
## Warning in simpleLoess(y, x, w, span, degree = degree, parametric = parametric, : zero-width neighborhood. make span bigger
```

```
## Warning: Computation failed in `stat_smooth()`:
## NA/NaN/Inf in foreign function call (arg 5)
```

```
## Warning in simpleLoess(y, x, w, span, degree = degree, parametric = parametric, : at -0.005
```

```
## Warning in simpleLoess(y, x, w, span, degree = degree, parametric = parametric, : radius 2.5e-05
```

```
## Warning in simpleLoess(y, x, w, span, degree = degree, parametric = parametric, : all data on boundary of neighborhood. make span bigger
```

```
## Warning in simpleLoess(y, x, w, span, degree = degree, parametric = parametric, : pseudoinverse used at -0.005
```

```
## Warning in simpleLoess(y, x, w, span, degree = degree, parametric = parametric, : neighborhood radius 0.005
```

```
## Warning in simpleLoess(y, x, w, span, degree = degree, parametric = parametric, : reciprocal condition number 1
```

```
## Warning in simpleLoess(y, x, w, span, degree = degree, parametric = parametric, : There are other near singularities as well. 1.01
```

```
## Warning in simpleLoess(y, x, w, span, degree = degree, parametric = parametric, : zero-width neighborhood. make span bigger
```

```
## Warning: Computation failed in `stat_smooth()`:
## NA/NaN/Inf in foreign function call (arg 5)
```

```
## Warning in simpleLoess(y, x, w, span, degree = degree, parametric = parametric, : at -0.005
```

```
## Warning in simpleLoess(y, x, w, span, degree = degree, parametric = parametric, : radius 2.5e-05
```

```
## Warning in simpleLoess(y, x, w, span, degree = degree, parametric = parametric, : all data on boundary of neighborhood. make span bigger
```

```
## Warning in simpleLoess(y, x, w, span, degree = degree, parametric = parametric, : pseudoinverse used at -0.005
```

```
## Warning in simpleLoess(y, x, w, span, degree = degree, parametric = parametric, : neighborhood radius 0.005
```

```
## Warning in simpleLoess(y, x, w, span, degree = degree, parametric = parametric, : reciprocal condition number 1
```

```
## Warning in simpleLoess(y, x, w, span, degree = degree, parametric = parametric, : There are other near singularities as well. 1.01
```

```
## Warning in simpleLoess(y, x, w, span, degree = degree, parametric = parametric, : zero-width neighborhood. make span bigger
```

```
## Warning: Computation failed in `stat_smooth()`:
## NA/NaN/Inf in foreign function call (arg 5)
```

```
## Warning in simpleLoess(y, x, w, span, degree = degree, parametric = parametric, : pseudoinverse used at -0.005
```

```
## Warning in simpleLoess(y, x, w, span, degree = degree, parametric = parametric, : neighborhood radius 1.005
```

```
## Warning in simpleLoess(y, x, w, span, degree = degree, parametric = parametric, : reciprocal condition number 0
```

```
## Warning in simpleLoess(y, x, w, span, degree = degree, parametric = parametric, : There are other near singularities as well. 1.01
```

```
## Warning in predLoess(object$y, object$x, newx = if (is.null(newdata)) object$x else if (is.data.frame(newdata)) as.matrix(model.frame(delete.response(terms(object)), : pseudoinverse used at -0.005
```

```
## Warning in predLoess(object$y, object$x, newx = if (is.null(newdata)) object$x else if (is.data.frame(newdata)) as.matrix(model.frame(delete.response(terms(object)), : neighborhood radius 1.005
```

```
## Warning in predLoess(object$y, object$x, newx = if (is.null(newdata)) object$x else if (is.data.frame(newdata)) as.matrix(model.frame(delete.response(terms(object)), : reciprocal condition number 0
```

```
## Warning in predLoess(object$y, object$x, newx = if (is.null(newdata)) object$x else if (is.data.frame(newdata)) as.matrix(model.frame(delete.response(terms(object)), : There are other near
## singularities as well. 1.01
```

```
## Warning in simpleLoess(y, x, w, span, degree = degree, parametric = parametric, : pseudoinverse used at -0.005
```

```
## Warning in simpleLoess(y, x, w, span, degree = degree, parametric = parametric, : neighborhood radius 1.005
```

```
## Warning in simpleLoess(y, x, w, span, degree = degree, parametric = parametric, : reciprocal condition number 0
```

```
## Warning in simpleLoess(y, x, w, span, degree = degree, parametric = parametric, : There are other near singularities as well. 1.01
```

```
## Warning in predLoess(object$y, object$x, newx = if (is.null(newdata)) object$x else if (is.data.frame(newdata)) as.matrix(model.frame(delete.response(terms(object)), : pseudoinverse used at -0.005
```

```
## Warning in predLoess(object$y, object$x, newx = if (is.null(newdata)) object$x else if (is.data.frame(newdata)) as.matrix(model.frame(delete.response(terms(object)), : neighborhood radius 1.005
```

```
## Warning in predLoess(object$y, object$x, newx = if (is.null(newdata)) object$x else if (is.data.frame(newdata)) as.matrix(model.frame(delete.response(terms(object)), : reciprocal condition number 0
```

```
## Warning in predLoess(object$y, object$x, newx = if (is.null(newdata)) object$x else if (is.data.frame(newdata)) as.matrix(model.frame(delete.response(terms(object)), : There are other near
## singularities as well. 1.01
```

```
## Warning in simpleLoess(y, x, w, span, degree = degree, parametric = parametric, : at -0.005
```

```
## Warning in simpleLoess(y, x, w, span, degree = degree, parametric = parametric, : radius 2.5e-05
```

```
## Warning in simpleLoess(y, x, w, span, degree = degree, parametric = parametric, : all data on boundary of neighborhood. make span bigger
```

```
## Warning in simpleLoess(y, x, w, span, degree = degree, parametric = parametric, : pseudoinverse used at -0.005
```

```
## Warning in simpleLoess(y, x, w, span, degree = degree, parametric = parametric, : neighborhood radius 0.005
```

```
## Warning in simpleLoess(y, x, w, span, degree = degree, parametric = parametric, : reciprocal condition number 1
```

```
## Warning in simpleLoess(y, x, w, span, degree = degree, parametric = parametric, : There are other near singularities as well. 1.01
```

```
## Warning in simpleLoess(y, x, w, span, degree = degree, parametric = parametric, : zero-width neighborhood. make span bigger
```

```
## Warning: Computation failed in `stat_smooth()`:
## NA/NaN/Inf in foreign function call (arg 5)
```

```
## Warning in simpleLoess(y, x, w, span, degree = degree, parametric = parametric, : pseudoinverse used at -0.005
```

```
## Warning in simpleLoess(y, x, w, span, degree = degree, parametric = parametric, : neighborhood radius 1.005
```

```
## Warning in simpleLoess(y, x, w, span, degree = degree, parametric = parametric, : reciprocal condition number 0
```

```
## Warning in simpleLoess(y, x, w, span, degree = degree, parametric = parametric, : There are other near singularities as well. 1.01
```

```
## Warning in predLoess(object$y, object$x, newx = if (is.null(newdata)) object$x else if (is.data.frame(newdata)) as.matrix(model.frame(delete.response(terms(object)), : pseudoinverse used at -0.005
```

```
## Warning in predLoess(object$y, object$x, newx = if (is.null(newdata)) object$x else if (is.data.frame(newdata)) as.matrix(model.frame(delete.response(terms(object)), : neighborhood radius 1.005
```

```
## Warning in predLoess(object$y, object$x, newx = if (is.null(newdata)) object$x else if (is.data.frame(newdata)) as.matrix(model.frame(delete.response(terms(object)), : reciprocal condition number 0
```

```
## Warning in predLoess(object$y, object$x, newx = if (is.null(newdata)) object$x else if (is.data.frame(newdata)) as.matrix(model.frame(delete.response(terms(object)), : There are other near
## singularities as well. 1.01
```

```
## Warning in simpleLoess(y, x, w, span, degree = degree, parametric = parametric, : pseudoinverse used at -0.005
```

```
## Warning in simpleLoess(y, x, w, span, degree = degree, parametric = parametric, : neighborhood radius 1.005
```

```
## Warning in simpleLoess(y, x, w, span, degree = degree, parametric = parametric, : reciprocal condition number 0
```

```
## Warning in simpleLoess(y, x, w, span, degree = degree, parametric = parametric, : There are other near singularities as well. 1.01
```

```
## Warning in predLoess(object$y, object$x, newx = if (is.null(newdata)) object$x else if (is.data.frame(newdata)) as.matrix(model.frame(delete.response(terms(object)), : pseudoinverse used at -0.005
```

```
## Warning in predLoess(object$y, object$x, newx = if (is.null(newdata)) object$x else if (is.data.frame(newdata)) as.matrix(model.frame(delete.response(terms(object)), : neighborhood radius 1.005
```

```
## Warning in predLoess(object$y, object$x, newx = if (is.null(newdata)) object$x else if (is.data.frame(newdata)) as.matrix(model.frame(delete.response(terms(object)), : reciprocal condition number 0
```

```
## Warning in predLoess(object$y, object$x, newx = if (is.null(newdata)) object$x else if (is.data.frame(newdata)) as.matrix(model.frame(delete.response(terms(object)), : There are other near
## singularities as well. 1.01
```

```
## Warning in simpleLoess(y, x, w, span, degree = degree, parametric = parametric, : pseudoinverse used at -0.005
```

```
## Warning in simpleLoess(y, x, w, span, degree = degree, parametric = parametric, : neighborhood radius 1.005
```

```
## Warning in simpleLoess(y, x, w, span, degree = degree, parametric = parametric, : reciprocal condition number 0
```

```
## Warning in simpleLoess(y, x, w, span, degree = degree, parametric = parametric, : There are other near singularities as well. 1.01
```

```
## Warning in predLoess(object$y, object$x, newx = if (is.null(newdata)) object$x else if (is.data.frame(newdata)) as.matrix(model.frame(delete.response(terms(object)), : pseudoinverse used at -0.005
```

```
## Warning in predLoess(object$y, object$x, newx = if (is.null(newdata)) object$x else if (is.data.frame(newdata)) as.matrix(model.frame(delete.response(terms(object)), : neighborhood radius 1.005
```

```
## Warning in predLoess(object$y, object$x, newx = if (is.null(newdata)) object$x else if (is.data.frame(newdata)) as.matrix(model.frame(delete.response(terms(object)), : reciprocal condition number 0
```

```
## Warning in predLoess(object$y, object$x, newx = if (is.null(newdata)) object$x else if (is.data.frame(newdata)) as.matrix(model.frame(delete.response(terms(object)), : There are other near
## singularities as well. 1.01
```

```
## Warning in simpleLoess(y, x, w, span, degree = degree, parametric = parametric, : at -0.005
```

```
## Warning in simpleLoess(y, x, w, span, degree = degree, parametric = parametric, : radius 2.5e-05
```

```
## Warning in simpleLoess(y, x, w, span, degree = degree, parametric = parametric, : all data on boundary of neighborhood. make span bigger
```

```
## Warning in simpleLoess(y, x, w, span, degree = degree, parametric = parametric, : pseudoinverse used at -0.005
```

```
## Warning in simpleLoess(y, x, w, span, degree = degree, parametric = parametric, : neighborhood radius 0.005
```

```
## Warning in simpleLoess(y, x, w, span, degree = degree, parametric = parametric, : reciprocal condition number 1
```

```
## Warning in simpleLoess(y, x, w, span, degree = degree, parametric = parametric, : There are other near singularities as well. 1.01
```

```
## Warning in simpleLoess(y, x, w, span, degree = degree, parametric = parametric, : zero-width neighborhood. make span bigger
```

```
## Warning: Computation failed in `stat_smooth()`:
## NA/NaN/Inf in foreign function call (arg 5)
```

```
## Warning in simpleLoess(y, x, w, span, degree = degree, parametric = parametric, : at -0.005
```

```
## Warning in simpleLoess(y, x, w, span, degree = degree, parametric = parametric, : radius 2.5e-05
```

```
## Warning in simpleLoess(y, x, w, span, degree = degree, parametric = parametric, : all data on boundary of neighborhood. make span bigger
```

```
## Warning in simpleLoess(y, x, w, span, degree = degree, parametric = parametric, : pseudoinverse used at -0.005
```

```
## Warning in simpleLoess(y, x, w, span, degree = degree, parametric = parametric, : neighborhood radius 0.005
```

```
## Warning in simpleLoess(y, x, w, span, degree = degree, parametric = parametric, : reciprocal condition number 1
```

```
## Warning in simpleLoess(y, x, w, span, degree = degree, parametric = parametric, : There are other near singularities as well. 1.01
```

```
## Warning in simpleLoess(y, x, w, span, degree = degree, parametric = parametric, : zero-width neighborhood. make span bigger
```

```
## Warning: Computation failed in `stat_smooth()`:
## NA/NaN/Inf in foreign function call (arg 5)
```

```
## Warning in simpleLoess(y, x, w, span, degree = degree, parametric = parametric, : at -0.005
```

```
## Warning in simpleLoess(y, x, w, span, degree = degree, parametric = parametric, : radius 2.5e-05
```

```
## Warning in simpleLoess(y, x, w, span, degree = degree, parametric = parametric, : all data on boundary of neighborhood. make span bigger
```

```
## Warning in simpleLoess(y, x, w, span, degree = degree, parametric = parametric, : pseudoinverse used at -0.005
```

```
## Warning in simpleLoess(y, x, w, span, degree = degree, parametric = parametric, : neighborhood radius 0.005
```

```
## Warning in simpleLoess(y, x, w, span, degree = degree, parametric = parametric, : reciprocal condition number 1
```

```
## Warning in simpleLoess(y, x, w, span, degree = degree, parametric = parametric, : There are other near singularities as well. 1.01
```

```
## Warning in simpleLoess(y, x, w, span, degree = degree, parametric = parametric, : zero-width neighborhood. make span bigger
```

```
## Warning: Computation failed in `stat_smooth()`:
## NA/NaN/Inf in foreign function call (arg 5)
```

```
## Warning in simpleLoess(y, x, w, span, degree = degree, parametric = parametric, : at -0.005
```

```
## Warning in simpleLoess(y, x, w, span, degree = degree, parametric = parametric, : radius 2.5e-05
```

```
## Warning in simpleLoess(y, x, w, span, degree = degree, parametric = parametric, : all data on boundary of neighborhood. make span bigger
```

```
## Warning in simpleLoess(y, x, w, span, degree = degree, parametric = parametric, : pseudoinverse used at -0.005
```

```
## Warning in simpleLoess(y, x, w, span, degree = degree, parametric = parametric, : neighborhood radius 0.005
```

```
## Warning in simpleLoess(y, x, w, span, degree = degree, parametric = parametric, : reciprocal condition number 1
```

```
## Warning in simpleLoess(y, x, w, span, degree = degree, parametric = parametric, : There are other near singularities as well. 1.01
```

```
## Warning in simpleLoess(y, x, w, span, degree = degree, parametric = parametric, : zero-width neighborhood. make span bigger
```

```
## Warning: Computation failed in `stat_smooth()`:
## NA/NaN/Inf in foreign function call (arg 5)
```

```
## Warning in simpleLoess(y, x, w, span, degree = degree, parametric = parametric, : at -0.005
```

```
## Warning in simpleLoess(y, x, w, span, degree = degree, parametric = parametric, : radius 2.5e-05
```

```
## Warning in simpleLoess(y, x, w, span, degree = degree, parametric = parametric, : all data on boundary of neighborhood. make span bigger
```

```
## Warning in simpleLoess(y, x, w, span, degree = degree, parametric = parametric, : pseudoinverse used at -0.005
```

```
## Warning in simpleLoess(y, x, w, span, degree = degree, parametric = parametric, : neighborhood radius 0.005
```

```
## Warning in simpleLoess(y, x, w, span, degree = degree, parametric = parametric, : reciprocal condition number 1
```

```
## Warning in simpleLoess(y, x, w, span, degree = degree, parametric = parametric, : There are other near singularities as well. 1.01
```

```
## Warning in simpleLoess(y, x, w, span, degree = degree, parametric = parametric, : zero-width neighborhood. make span bigger
```

```
## Warning: Computation failed in `stat_smooth()`:
## NA/NaN/Inf in foreign function call (arg 5)
```

```
## Warning in simpleLoess(y, x, w, span, degree = degree, parametric = parametric, : at -0.005
```

```
## Warning in simpleLoess(y, x, w, span, degree = degree, parametric = parametric, : radius 2.5e-05
```

```
## Warning in simpleLoess(y, x, w, span, degree = degree, parametric = parametric, : all data on boundary of neighborhood. make span bigger
```

```
## Warning in simpleLoess(y, x, w, span, degree = degree, parametric = parametric, : pseudoinverse used at -0.005
```

```
## Warning in simpleLoess(y, x, w, span, degree = degree, parametric = parametric, : neighborhood radius 0.005
```

```
## Warning in simpleLoess(y, x, w, span, degree = degree, parametric = parametric, : reciprocal condition number 1
```

```
## Warning in simpleLoess(y, x, w, span, degree = degree, parametric = parametric, : There are other near singularities as well. 1.01
```

```
## Warning in simpleLoess(y, x, w, span, degree = degree, parametric = parametric, : zero-width neighborhood. make span bigger
```

```
## Warning: Computation failed in `stat_smooth()`:
## NA/NaN/Inf in foreign function call (arg 5)
```

```
## Warning in simpleLoess(y, x, w, span, degree = degree, parametric = parametric, : at -0.005
```

```
## Warning in simpleLoess(y, x, w, span, degree = degree, parametric = parametric, : radius 2.5e-05
```

```
## Warning in simpleLoess(y, x, w, span, degree = degree, parametric = parametric, : all data on boundary of neighborhood. make span bigger
```

```
## Warning in simpleLoess(y, x, w, span, degree = degree, parametric = parametric, : pseudoinverse used at -0.005
```

```
## Warning in simpleLoess(y, x, w, span, degree = degree, parametric = parametric, : neighborhood radius 0.005
```

```
## Warning in simpleLoess(y, x, w, span, degree = degree, parametric = parametric, : reciprocal condition number 1
```

```
## Warning in simpleLoess(y, x, w, span, degree = degree, parametric = parametric, : There are other near singularities as well. 1.01
```

```
## Warning in simpleLoess(y, x, w, span, degree = degree, parametric = parametric, : zero-width neighborhood. make span bigger
```

```
## Warning: Computation failed in `stat_smooth()`:
## NA/NaN/Inf in foreign function call (arg 5)
```

```
## Warning in simpleLoess(y, x, w, span, degree = degree, parametric = parametric, : at -0.005
```

```
## Warning in simpleLoess(y, x, w, span, degree = degree, parametric = parametric, : radius 2.5e-05
```

```
## Warning in simpleLoess(y, x, w, span, degree = degree, parametric = parametric, : all data on boundary of neighborhood. make span bigger
```

```
## Warning in simpleLoess(y, x, w, span, degree = degree, parametric = parametric, : pseudoinverse used at -0.005
```

```
## Warning in simpleLoess(y, x, w, span, degree = degree, parametric = parametric, : neighborhood radius 0.005
```

```
## Warning in simpleLoess(y, x, w, span, degree = degree, parametric = parametric, : reciprocal condition number 1
```

```
## Warning in simpleLoess(y, x, w, span, degree = degree, parametric = parametric, : There are other near singularities as well. 1.01
```

```
## Warning in simpleLoess(y, x, w, span, degree = degree, parametric = parametric, : zero-width neighborhood. make span bigger
```

```
## Warning: Computation failed in `stat_smooth()`:
## NA/NaN/Inf in foreign function call (arg 5)
```

```
## Warning in simpleLoess(y, x, w, span, degree = degree, parametric = parametric, : at -0.005
```

```
## Warning in simpleLoess(y, x, w, span, degree = degree, parametric = parametric, : radius 2.5e-05
```

```
## Warning in simpleLoess(y, x, w, span, degree = degree, parametric = parametric, : all data on boundary of neighborhood. make span bigger
```

```
## Warning in simpleLoess(y, x, w, span, degree = degree, parametric = parametric, : pseudoinverse used at -0.005
```

```
## Warning in simpleLoess(y, x, w, span, degree = degree, parametric = parametric, : neighborhood radius 0.005
```

```
## Warning in simpleLoess(y, x, w, span, degree = degree, parametric = parametric, : reciprocal condition number 1
```

```
## Warning in simpleLoess(y, x, w, span, degree = degree, parametric = parametric, : There are other near singularities as well. 1.01
```

```
## Warning in simpleLoess(y, x, w, span, degree = degree, parametric = parametric, : zero-width neighborhood. make span bigger
```

```
## Warning: Computation failed in `stat_smooth()`:
## NA/NaN/Inf in foreign function call (arg 5)
```

```
## Warning in simpleLoess(y, x, w, span, degree = degree, parametric = parametric, : at -0.005
```

```
## Warning in simpleLoess(y, x, w, span, degree = degree, parametric = parametric, : radius 2.5e-05
```

```
## Warning in simpleLoess(y, x, w, span, degree = degree, parametric = parametric, : all data on boundary of neighborhood. make span bigger
```

```
## Warning in simpleLoess(y, x, w, span, degree = degree, parametric = parametric, : pseudoinverse used at -0.005
```

```
## Warning in simpleLoess(y, x, w, span, degree = degree, parametric = parametric, : neighborhood radius 0.005
```

```
## Warning in simpleLoess(y, x, w, span, degree = degree, parametric = parametric, : reciprocal condition number 1
```

```
## Warning in simpleLoess(y, x, w, span, degree = degree, parametric = parametric, : There are other near singularities as well. 1.01
```

```
## Warning in simpleLoess(y, x, w, span, degree = degree, parametric = parametric, : zero-width neighborhood. make span bigger
```

```
## Warning: Computation failed in `stat_smooth()`:
## NA/NaN/Inf in foreign function call (arg 5)
```

```
## Warning in simpleLoess(y, x, w, span, degree = degree, parametric = parametric, : at -0.005
```

```
## Warning in simpleLoess(y, x, w, span, degree = degree, parametric = parametric, : radius 2.5e-05
```

```
## Warning in simpleLoess(y, x, w, span, degree = degree, parametric = parametric, : all data on boundary of neighborhood. make span bigger
```

```
## Warning in simpleLoess(y, x, w, span, degree = degree, parametric = parametric, : pseudoinverse used at -0.005
```

```
## Warning in simpleLoess(y, x, w, span, degree = degree, parametric = parametric, : neighborhood radius 0.005
```

```
## Warning in simpleLoess(y, x, w, span, degree = degree, parametric = parametric, : reciprocal condition number 1
```

```
## Warning in simpleLoess(y, x, w, span, degree = degree, parametric = parametric, : There are other near singularities as well. 1.01
```

```
## Warning in simpleLoess(y, x, w, span, degree = degree, parametric = parametric, : zero-width neighborhood. make span bigger
```

```
## Warning: Computation failed in `stat_smooth()`:
## NA/NaN/Inf in foreign function call (arg 5)
```

```
## Warning in simpleLoess(y, x, w, span, degree = degree, parametric = parametric, : at -0.005
```

```
## Warning in simpleLoess(y, x, w, span, degree = degree, parametric = parametric, : radius 2.5e-05
```

```
## Warning in simpleLoess(y, x, w, span, degree = degree, parametric = parametric, : all data on boundary of neighborhood. make span bigger
```

```
## Warning in simpleLoess(y, x, w, span, degree = degree, parametric = parametric, : pseudoinverse used at -0.005
```

```
## Warning in simpleLoess(y, x, w, span, degree = degree, parametric = parametric, : neighborhood radius 0.005
```

```
## Warning in simpleLoess(y, x, w, span, degree = degree, parametric = parametric, : reciprocal condition number 1
```

```
## Warning in simpleLoess(y, x, w, span, degree = degree, parametric = parametric, : There are other near singularities as well. 1.01
```

```
## Warning in simpleLoess(y, x, w, span, degree = degree, parametric = parametric, : zero-width neighborhood. make span bigger
```

```
## Warning: Computation failed in `stat_smooth()`:
## NA/NaN/Inf in foreign function call (arg 5)
```

```
## Warning in simpleLoess(y, x, w, span, degree = degree, parametric = parametric, : at -0.005
```

```
## Warning in simpleLoess(y, x, w, span, degree = degree, parametric = parametric, : radius 2.5e-05
```

```
## Warning in simpleLoess(y, x, w, span, degree = degree, parametric = parametric, : all data on boundary of neighborhood. make span bigger
```

```
## Warning in simpleLoess(y, x, w, span, degree = degree, parametric = parametric, : pseudoinverse used at -0.005
```

```
## Warning in simpleLoess(y, x, w, span, degree = degree, parametric = parametric, : neighborhood radius 0.005
```

```
## Warning in simpleLoess(y, x, w, span, degree = degree, parametric = parametric, : reciprocal condition number 1
```

```
## Warning in simpleLoess(y, x, w, span, degree = degree, parametric = parametric, : There are other near singularities as well. 1.01
```

```
## Warning in simpleLoess(y, x, w, span, degree = degree, parametric = parametric, : zero-width neighborhood. make span bigger
```

```
## Warning: Computation failed in `stat_smooth()`:
## NA/NaN/Inf in foreign function call (arg 5)
```

```
## Warning in simpleLoess(y, x, w, span, degree = degree, parametric = parametric, : at -0.005
```

```
## Warning in simpleLoess(y, x, w, span, degree = degree, parametric = parametric, : radius 2.5e-05
```

```
## Warning in simpleLoess(y, x, w, span, degree = degree, parametric = parametric, : all data on boundary of neighborhood. make span bigger
```

```
## Warning in simpleLoess(y, x, w, span, degree = degree, parametric = parametric, : pseudoinverse used at -0.005
```

```
## Warning in simpleLoess(y, x, w, span, degree = degree, parametric = parametric, : neighborhood radius 0.005
```

```
## Warning in simpleLoess(y, x, w, span, degree = degree, parametric = parametric, : reciprocal condition number 1
```

```
## Warning in simpleLoess(y, x, w, span, degree = degree, parametric = parametric, : There are other near singularities as well. 1.01
```

```
## Warning in simpleLoess(y, x, w, span, degree = degree, parametric = parametric, : zero-width neighborhood. make span bigger
```

```
## Warning: Computation failed in `stat_smooth()`:
## NA/NaN/Inf in foreign function call (arg 5)
```

```
## Warning in simpleLoess(y, x, w, span, degree = degree, parametric = parametric, : at -0.005
```

```
## Warning in simpleLoess(y, x, w, span, degree = degree, parametric = parametric, : radius 2.5e-05
```

```
## Warning in simpleLoess(y, x, w, span, degree = degree, parametric = parametric, : all data on boundary of neighborhood. make span bigger
```

```
## Warning in simpleLoess(y, x, w, span, degree = degree, parametric = parametric, : pseudoinverse used at -0.005
```

```
## Warning in simpleLoess(y, x, w, span, degree = degree, parametric = parametric, : neighborhood radius 0.005
```

```
## Warning in simpleLoess(y, x, w, span, degree = degree, parametric = parametric, : reciprocal condition number 1
```

```
## Warning in simpleLoess(y, x, w, span, degree = degree, parametric = parametric, : There are other near singularities as well. 1.01
```

```
## Warning in simpleLoess(y, x, w, span, degree = degree, parametric = parametric, : zero-width neighborhood. make span bigger
```

```
## Warning: Computation failed in `stat_smooth()`:
## NA/NaN/Inf in foreign function call (arg 5)
```

```
## Warning in simpleLoess(y, x, w, span, degree = degree, parametric = parametric, : at -0.005
```

```
## Warning in simpleLoess(y, x, w, span, degree = degree, parametric = parametric, : radius 2.5e-05
```

```
## Warning in simpleLoess(y, x, w, span, degree = degree, parametric = parametric, : all data on boundary of neighborhood. make span bigger
```

```
## Warning in simpleLoess(y, x, w, span, degree = degree, parametric = parametric, : pseudoinverse used at -0.005
```

```
## Warning in simpleLoess(y, x, w, span, degree = degree, parametric = parametric, : neighborhood radius 0.005
```

```
## Warning in simpleLoess(y, x, w, span, degree = degree, parametric = parametric, : reciprocal condition number 1
```

```
## Warning in simpleLoess(y, x, w, span, degree = degree, parametric = parametric, : There are other near singularities as well. 1.01
```

```
## Warning in simpleLoess(y, x, w, span, degree = degree, parametric = parametric, : zero-width neighborhood. make span bigger
```

```
## Warning: Computation failed in `stat_smooth()`:
## NA/NaN/Inf in foreign function call (arg 5)
```

```
## Warning in simpleLoess(y, x, w, span, degree = degree, parametric = parametric, : at -0.005
```

```
## Warning in simpleLoess(y, x, w, span, degree = degree, parametric = parametric, : radius 2.5e-05
```

```
## Warning in simpleLoess(y, x, w, span, degree = degree, parametric = parametric, : all data on boundary of neighborhood. make span bigger
```

```
## Warning in simpleLoess(y, x, w, span, degree = degree, parametric = parametric, : pseudoinverse used at -0.005
```

```
## Warning in simpleLoess(y, x, w, span, degree = degree, parametric = parametric, : neighborhood radius 0.005
```

```
## Warning in simpleLoess(y, x, w, span, degree = degree, parametric = parametric, : reciprocal condition number 1
```

```
## Warning in simpleLoess(y, x, w, span, degree = degree, parametric = parametric, : There are other near singularities as well. 1.01
```

```
## Warning in simpleLoess(y, x, w, span, degree = degree, parametric = parametric, : zero-width neighborhood. make span bigger
```

```
## Warning: Computation failed in `stat_smooth()`:
## NA/NaN/Inf in foreign function call (arg 5)
```

```
## Warning in simpleLoess(y, x, w, span, degree = degree, parametric = parametric, : at -0.005
```

```
## Warning in simpleLoess(y, x, w, span, degree = degree, parametric = parametric, : radius 2.5e-05
```

```
## Warning in simpleLoess(y, x, w, span, degree = degree, parametric = parametric, : all data on boundary of neighborhood. make span bigger
```

```
## Warning in simpleLoess(y, x, w, span, degree = degree, parametric = parametric, : pseudoinverse used at -0.005
```

```
## Warning in simpleLoess(y, x, w, span, degree = degree, parametric = parametric, : neighborhood radius 0.005
```

```
## Warning in simpleLoess(y, x, w, span, degree = degree, parametric = parametric, : reciprocal condition number 1
```

```
## Warning in simpleLoess(y, x, w, span, degree = degree, parametric = parametric, : There are other near singularities as well. 1.01
```

```
## Warning in simpleLoess(y, x, w, span, degree = degree, parametric = parametric, : zero-width neighborhood. make span bigger
```

```
## Warning: Computation failed in `stat_smooth()`:
## NA/NaN/Inf in foreign function call (arg 5)
```

```
## Warning in simpleLoess(y, x, w, span, degree = degree, parametric = parametric, : at -0.005
```

```
## Warning in simpleLoess(y, x, w, span, degree = degree, parametric = parametric, : radius 2.5e-05
```

```
## Warning in simpleLoess(y, x, w, span, degree = degree, parametric = parametric, : all data on boundary of neighborhood. make span bigger
```

```
## Warning in simpleLoess(y, x, w, span, degree = degree, parametric = parametric, : pseudoinverse used at -0.005
```

```
## Warning in simpleLoess(y, x, w, span, degree = degree, parametric = parametric, : neighborhood radius 0.005
```

```
## Warning in simpleLoess(y, x, w, span, degree = degree, parametric = parametric, : reciprocal condition number 1
```

```
## Warning in simpleLoess(y, x, w, span, degree = degree, parametric = parametric, : There are other near singularities as well. 1.01
```

```
## Warning in simpleLoess(y, x, w, span, degree = degree, parametric = parametric, : zero-width neighborhood. make span bigger
```

```
## Warning: Computation failed in `stat_smooth()`:
## NA/NaN/Inf in foreign function call (arg 5)
```

```
## Warning in simpleLoess(y, x, w, span, degree = degree, parametric = parametric, : at -0.005
```

```
## Warning in simpleLoess(y, x, w, span, degree = degree, parametric = parametric, : radius 2.5e-05
```

```
## Warning in simpleLoess(y, x, w, span, degree = degree, parametric = parametric, : all data on boundary of neighborhood. make span bigger
```

```
## Warning in simpleLoess(y, x, w, span, degree = degree, parametric = parametric, : pseudoinverse used at -0.005
```

```
## Warning in simpleLoess(y, x, w, span, degree = degree, parametric = parametric, : neighborhood radius 0.005
```

```
## Warning in simpleLoess(y, x, w, span, degree = degree, parametric = parametric, : reciprocal condition number 1
```

```
## Warning in simpleLoess(y, x, w, span, degree = degree, parametric = parametric, : There are other near singularities as well. 1.01
```

```
## Warning in simpleLoess(y, x, w, span, degree = degree, parametric = parametric, : zero-width neighborhood. make span bigger
```

```
## Warning: Computation failed in `stat_smooth()`:
## NA/NaN/Inf in foreign function call (arg 5)
```

```
## Warning in simpleLoess(y, x, w, span, degree = degree, parametric = parametric, : at -0.005
```

```
## Warning in simpleLoess(y, x, w, span, degree = degree, parametric = parametric, : radius 2.5e-05
```

```
## Warning in simpleLoess(y, x, w, span, degree = degree, parametric = parametric, : all data on boundary of neighborhood. make span bigger
```

```
## Warning in simpleLoess(y, x, w, span, degree = degree, parametric = parametric, : pseudoinverse used at -0.005
```

```
## Warning in simpleLoess(y, x, w, span, degree = degree, parametric = parametric, : neighborhood radius 0.005
```

```
## Warning in simpleLoess(y, x, w, span, degree = degree, parametric = parametric, : reciprocal condition number 1
```

```
## Warning in simpleLoess(y, x, w, span, degree = degree, parametric = parametric, : There are other near singularities as well. 1.01
```

```
## Warning in simpleLoess(y, x, w, span, degree = degree, parametric = parametric, : zero-width neighborhood. make span bigger
```

```
## Warning: Computation failed in `stat_smooth()`:
## NA/NaN/Inf in foreign function call (arg 5)
```

```
## Warning in simpleLoess(y, x, w, span, degree = degree, parametric = parametric, : at -0.005
```

```
## Warning in simpleLoess(y, x, w, span, degree = degree, parametric = parametric, : radius 2.5e-05
```

```
## Warning in simpleLoess(y, x, w, span, degree = degree, parametric = parametric, : all data on boundary of neighborhood. make span bigger
```

```
## Warning in simpleLoess(y, x, w, span, degree = degree, parametric = parametric, : pseudoinverse used at -0.005
```

```
## Warning in simpleLoess(y, x, w, span, degree = degree, parametric = parametric, : neighborhood radius 0.005
```

```
## Warning in simpleLoess(y, x, w, span, degree = degree, parametric = parametric, : reciprocal condition number 1
```

```
## Warning in simpleLoess(y, x, w, span, degree = degree, parametric = parametric, : There are other near singularities as well. 1.01
```

```
## Warning in simpleLoess(y, x, w, span, degree = degree, parametric = parametric, : zero-width neighborhood. make span bigger
```

```
## Warning: Computation failed in `stat_smooth()`:
## NA/NaN/Inf in foreign function call (arg 5)
```

```
## Warning in simpleLoess(y, x, w, span, degree = degree, parametric = parametric, : at -0.005
```

```
## Warning in simpleLoess(y, x, w, span, degree = degree, parametric = parametric, : radius 2.5e-05
```

```
## Warning in simpleLoess(y, x, w, span, degree = degree, parametric = parametric, : all data on boundary of neighborhood. make span bigger
```

```
## Warning in simpleLoess(y, x, w, span, degree = degree, parametric = parametric, : pseudoinverse used at -0.005
```

```
## Warning in simpleLoess(y, x, w, span, degree = degree, parametric = parametric, : neighborhood radius 0.005
```

```
## Warning in simpleLoess(y, x, w, span, degree = degree, parametric = parametric, : reciprocal condition number 1
```

```
## Warning in simpleLoess(y, x, w, span, degree = degree, parametric = parametric, : There are other near singularities as well. 1.01
```

```
## Warning in simpleLoess(y, x, w, span, degree = degree, parametric = parametric, : zero-width neighborhood. make span bigger
```

```
## Warning: Computation failed in `stat_smooth()`:
## NA/NaN/Inf in foreign function call (arg 5)
```

```
## Warning in simpleLoess(y, x, w, span, degree = degree, parametric = parametric, : pseudoinverse used at -0.005
```

```
## Warning in simpleLoess(y, x, w, span, degree = degree, parametric = parametric, : neighborhood radius 1.005
```

```
## Warning in simpleLoess(y, x, w, span, degree = degree, parametric = parametric, : reciprocal condition number 0
```

```
## Warning in simpleLoess(y, x, w, span, degree = degree, parametric = parametric, : There are other near singularities as well. 1.01
```

```
## Warning in predLoess(object$y, object$x, newx = if (is.null(newdata)) object$x else if (is.data.frame(newdata)) as.matrix(model.frame(delete.response(terms(object)), : pseudoinverse used at -0.005
```

```
## Warning in predLoess(object$y, object$x, newx = if (is.null(newdata)) object$x else if (is.data.frame(newdata)) as.matrix(model.frame(delete.response(terms(object)), : neighborhood radius 1.005
```

```
## Warning in predLoess(object$y, object$x, newx = if (is.null(newdata)) object$x else if (is.data.frame(newdata)) as.matrix(model.frame(delete.response(terms(object)), : reciprocal condition number 0
```

```
## Warning in predLoess(object$y, object$x, newx = if (is.null(newdata)) object$x else if (is.data.frame(newdata)) as.matrix(model.frame(delete.response(terms(object)), : There are other near
## singularities as well. 1.01
```

```
## Warning in simpleLoess(y, x, w, span, degree = degree, parametric = parametric, : at -0.005
```

```
## Warning in simpleLoess(y, x, w, span, degree = degree, parametric = parametric, : radius 2.5e-05
```

```
## Warning in simpleLoess(y, x, w, span, degree = degree, parametric = parametric, : all data on boundary of neighborhood. make span bigger
```

```
## Warning in simpleLoess(y, x, w, span, degree = degree, parametric = parametric, : pseudoinverse used at -0.005
```

```
## Warning in simpleLoess(y, x, w, span, degree = degree, parametric = parametric, : neighborhood radius 0.005
```

```
## Warning in simpleLoess(y, x, w, span, degree = degree, parametric = parametric, : reciprocal condition number 1
```

```
## Warning in simpleLoess(y, x, w, span, degree = degree, parametric = parametric, : There are other near singularities as well. 1.01
```

```
## Warning in simpleLoess(y, x, w, span, degree = degree, parametric = parametric, : zero-width neighborhood. make span bigger
```

```
## Warning: Computation failed in `stat_smooth()`:
## NA/NaN/Inf in foreign function call (arg 5)
```

```
## Warning in simpleLoess(y, x, w, span, degree = degree, parametric = parametric, : at -0.005
```

```
## Warning in simpleLoess(y, x, w, span, degree = degree, parametric = parametric, : radius 2.5e-05
```

```
## Warning in simpleLoess(y, x, w, span, degree = degree, parametric = parametric, : all data on boundary of neighborhood. make span bigger
```

```
## Warning in simpleLoess(y, x, w, span, degree = degree, parametric = parametric, : pseudoinverse used at -0.005
```

```
## Warning in simpleLoess(y, x, w, span, degree = degree, parametric = parametric, : neighborhood radius 0.005
```

```
## Warning in simpleLoess(y, x, w, span, degree = degree, parametric = parametric, : reciprocal condition number 1
```

```
## Warning in simpleLoess(y, x, w, span, degree = degree, parametric = parametric, : There are other near singularities as well. 1.01
```

```
## Warning in simpleLoess(y, x, w, span, degree = degree, parametric = parametric, : zero-width neighborhood. make span bigger
```

```
## Warning: Computation failed in `stat_smooth()`:
## NA/NaN/Inf in foreign function call (arg 5)
```

```
## Warning in simpleLoess(y, x, w, span, degree = degree, parametric = parametric, : at -0.005
```

```
## Warning in simpleLoess(y, x, w, span, degree = degree, parametric = parametric, : radius 2.5e-05
```

```
## Warning in simpleLoess(y, x, w, span, degree = degree, parametric = parametric, : all data on boundary of neighborhood. make span bigger
```

```
## Warning in simpleLoess(y, x, w, span, degree = degree, parametric = parametric, : pseudoinverse used at -0.005
```

```
## Warning in simpleLoess(y, x, w, span, degree = degree, parametric = parametric, : neighborhood radius 0.005
```

```
## Warning in simpleLoess(y, x, w, span, degree = degree, parametric = parametric, : reciprocal condition number 1
```

```
## Warning in simpleLoess(y, x, w, span, degree = degree, parametric = parametric, : There are other near singularities as well. 1.01
```

```
## Warning in simpleLoess(y, x, w, span, degree = degree, parametric = parametric, : zero-width neighborhood. make span bigger
```

```
## Warning: Computation failed in `stat_smooth()`:
## NA/NaN/Inf in foreign function call (arg 5)
```

```
## Warning in simpleLoess(y, x, w, span, degree = degree, parametric = parametric, : at -0.005
```

```
## Warning in simpleLoess(y, x, w, span, degree = degree, parametric = parametric, : radius 2.5e-05
```

```
## Warning in simpleLoess(y, x, w, span, degree = degree, parametric = parametric, : all data on boundary of neighborhood. make span bigger
```

```
## Warning in simpleLoess(y, x, w, span, degree = degree, parametric = parametric, : pseudoinverse used at -0.005
```

```
## Warning in simpleLoess(y, x, w, span, degree = degree, parametric = parametric, : neighborhood radius 0.005
```

```
## Warning in simpleLoess(y, x, w, span, degree = degree, parametric = parametric, : reciprocal condition number 1
```

```
## Warning in simpleLoess(y, x, w, span, degree = degree, parametric = parametric, : There are other near singularities as well. 1.01
```

```
## Warning in simpleLoess(y, x, w, span, degree = degree, parametric = parametric, : zero-width neighborhood. make span bigger
```

```
## Warning: Computation failed in `stat_smooth()`:
## NA/NaN/Inf in foreign function call (arg 5)
```

```
## Warning in simpleLoess(y, x, w, span, degree = degree, parametric = parametric, : at -0.005
```

```
## Warning in simpleLoess(y, x, w, span, degree = degree, parametric = parametric, : radius 2.5e-05
```

```
## Warning in simpleLoess(y, x, w, span, degree = degree, parametric = parametric, : all data on boundary of neighborhood. make span bigger
```

```
## Warning in simpleLoess(y, x, w, span, degree = degree, parametric = parametric, : pseudoinverse used at -0.005
```

```
## Warning in simpleLoess(y, x, w, span, degree = degree, parametric = parametric, : neighborhood radius 0.005
```

```
## Warning in simpleLoess(y, x, w, span, degree = degree, parametric = parametric, : reciprocal condition number 1
```

```
## Warning in simpleLoess(y, x, w, span, degree = degree, parametric = parametric, : There are other near singularities as well. 1.01
```

```
## Warning in simpleLoess(y, x, w, span, degree = degree, parametric = parametric, : zero-width neighborhood. make span bigger
```

```
## Warning: Computation failed in `stat_smooth()`:
## NA/NaN/Inf in foreign function call (arg 5)
```

```
## Warning in simpleLoess(y, x, w, span, degree = degree, parametric = parametric, : at -0.005
```

```
## Warning in simpleLoess(y, x, w, span, degree = degree, parametric = parametric, : radius 2.5e-05
```

```
## Warning in simpleLoess(y, x, w, span, degree = degree, parametric = parametric, : all data on boundary of neighborhood. make span bigger
```

```
## Warning in simpleLoess(y, x, w, span, degree = degree, parametric = parametric, : pseudoinverse used at -0.005
```

```
## Warning in simpleLoess(y, x, w, span, degree = degree, parametric = parametric, : neighborhood radius 0.005
```

```
## Warning in simpleLoess(y, x, w, span, degree = degree, parametric = parametric, : reciprocal condition number 1
```

```
## Warning in simpleLoess(y, x, w, span, degree = degree, parametric = parametric, : There are other near singularities as well. 1.01
```

```
## Warning in simpleLoess(y, x, w, span, degree = degree, parametric = parametric, : zero-width neighborhood. make span bigger
```

```
## Warning: Computation failed in `stat_smooth()`:
## NA/NaN/Inf in foreign function call (arg 5)
```

```
## Warning in simpleLoess(y, x, w, span, degree = degree, parametric = parametric, : at -0.005
```

```
## Warning in simpleLoess(y, x, w, span, degree = degree, parametric = parametric, : radius 2.5e-05
```

```
## Warning in simpleLoess(y, x, w, span, degree = degree, parametric = parametric, : all data on boundary of neighborhood. make span bigger
```

```
## Warning in simpleLoess(y, x, w, span, degree = degree, parametric = parametric, : pseudoinverse used at -0.005
```

```
## Warning in simpleLoess(y, x, w, span, degree = degree, parametric = parametric, : neighborhood radius 0.005
```

```
## Warning in simpleLoess(y, x, w, span, degree = degree, parametric = parametric, : reciprocal condition number 1
```

```
## Warning in simpleLoess(y, x, w, span, degree = degree, parametric = parametric, : There are other near singularities as well. 1.01
```

```
## Warning in simpleLoess(y, x, w, span, degree = degree, parametric = parametric, : zero-width neighborhood. make span bigger
```

```
## Warning: Computation failed in `stat_smooth()`:
## NA/NaN/Inf in foreign function call (arg 5)
```

```
## Warning in simpleLoess(y, x, w, span, degree = degree, parametric = parametric, : pseudoinverse used at -0.005
```

```
## Warning in simpleLoess(y, x, w, span, degree = degree, parametric = parametric, : neighborhood radius 1.005
```

```
## Warning in simpleLoess(y, x, w, span, degree = degree, parametric = parametric, : reciprocal condition number 0
```

```
## Warning in simpleLoess(y, x, w, span, degree = degree, parametric = parametric, : There are other near singularities as well. 1.01
```

```
## Warning in predLoess(object$y, object$x, newx = if (is.null(newdata)) object$x else if (is.data.frame(newdata)) as.matrix(model.frame(delete.response(terms(object)), : pseudoinverse used at -0.005
```

```
## Warning in predLoess(object$y, object$x, newx = if (is.null(newdata)) object$x else if (is.data.frame(newdata)) as.matrix(model.frame(delete.response(terms(object)), : neighborhood radius 1.005
```

```
## Warning in predLoess(object$y, object$x, newx = if (is.null(newdata)) object$x else if (is.data.frame(newdata)) as.matrix(model.frame(delete.response(terms(object)), : reciprocal condition number 0
```

```
## Warning in predLoess(object$y, object$x, newx = if (is.null(newdata)) object$x else if (is.data.frame(newdata)) as.matrix(model.frame(delete.response(terms(object)), : There are other near
## singularities as well. 1.01
```

```
## Warning in simpleLoess(y, x, w, span, degree = degree, parametric = parametric, : at -0.005
```

```
## Warning in simpleLoess(y, x, w, span, degree = degree, parametric = parametric, : radius 2.5e-05
```

```
## Warning in simpleLoess(y, x, w, span, degree = degree, parametric = parametric, : all data on boundary of neighborhood. make span bigger
```

```
## Warning in simpleLoess(y, x, w, span, degree = degree, parametric = parametric, : pseudoinverse used at -0.005
```

```
## Warning in simpleLoess(y, x, w, span, degree = degree, parametric = parametric, : neighborhood radius 0.005
```

```
## Warning in simpleLoess(y, x, w, span, degree = degree, parametric = parametric, : reciprocal condition number 1
```

```
## Warning in simpleLoess(y, x, w, span, degree = degree, parametric = parametric, : There are other near singularities as well. 1.01
```

```
## Warning in simpleLoess(y, x, w, span, degree = degree, parametric = parametric, : zero-width neighborhood. make span bigger
```

```
## Warning: Computation failed in `stat_smooth()`:
## NA/NaN/Inf in foreign function call (arg 5)
```

```
## Warning in simpleLoess(y, x, w, span, degree = degree, parametric = parametric, : at -0.005
```

```
## Warning in simpleLoess(y, x, w, span, degree = degree, parametric = parametric, : radius 2.5e-05
```

```
## Warning in simpleLoess(y, x, w, span, degree = degree, parametric = parametric, : all data on boundary of neighborhood. make span bigger
```

```
## Warning in simpleLoess(y, x, w, span, degree = degree, parametric = parametric, : pseudoinverse used at -0.005
```

```
## Warning in simpleLoess(y, x, w, span, degree = degree, parametric = parametric, : neighborhood radius 0.005
```

```
## Warning in simpleLoess(y, x, w, span, degree = degree, parametric = parametric, : reciprocal condition number 1
```

```
## Warning in simpleLoess(y, x, w, span, degree = degree, parametric = parametric, : There are other near singularities as well. 1.01
```

```
## Warning in simpleLoess(y, x, w, span, degree = degree, parametric = parametric, : zero-width neighborhood. make span bigger
```

```
## Warning: Computation failed in `stat_smooth()`:
## NA/NaN/Inf in foreign function call (arg 5)
```

```
## Warning in simpleLoess(y, x, w, span, degree = degree, parametric = parametric, : at -0.005
```

```
## Warning in simpleLoess(y, x, w, span, degree = degree, parametric = parametric, : radius 2.5e-05
```

```
## Warning in simpleLoess(y, x, w, span, degree = degree, parametric = parametric, : all data on boundary of neighborhood. make span bigger
```

```
## Warning in simpleLoess(y, x, w, span, degree = degree, parametric = parametric, : pseudoinverse used at -0.005
```

```
## Warning in simpleLoess(y, x, w, span, degree = degree, parametric = parametric, : neighborhood radius 0.005
```

```
## Warning in simpleLoess(y, x, w, span, degree = degree, parametric = parametric, : reciprocal condition number 1
```

```
## Warning in simpleLoess(y, x, w, span, degree = degree, parametric = parametric, : There are other near singularities as well. 1.01
```

```
## Warning in simpleLoess(y, x, w, span, degree = degree, parametric = parametric, : zero-width neighborhood. make span bigger
```

```
## Warning: Computation failed in `stat_smooth()`:
## NA/NaN/Inf in foreign function call (arg 5)
```

```
## Warning in simpleLoess(y, x, w, span, degree = degree, parametric = parametric, : at -0.005
```

```
## Warning in simpleLoess(y, x, w, span, degree = degree, parametric = parametric, : radius 2.5e-05
```

```
## Warning in simpleLoess(y, x, w, span, degree = degree, parametric = parametric, : all data on boundary of neighborhood. make span bigger
```

```
## Warning in simpleLoess(y, x, w, span, degree = degree, parametric = parametric, : pseudoinverse used at -0.005
```

```
## Warning in simpleLoess(y, x, w, span, degree = degree, parametric = parametric, : neighborhood radius 0.005
```

```
## Warning in simpleLoess(y, x, w, span, degree = degree, parametric = parametric, : reciprocal condition number 1
```

```
## Warning in simpleLoess(y, x, w, span, degree = degree, parametric = parametric, : There are other near singularities as well. 1.01
```

```
## Warning in simpleLoess(y, x, w, span, degree = degree, parametric = parametric, : zero-width neighborhood. make span bigger
```

```
## Warning: Computation failed in `stat_smooth()`:
## NA/NaN/Inf in foreign function call (arg 5)
```

```
## Warning in simpleLoess(y, x, w, span, degree = degree, parametric = parametric, : at -0.005
```

```
## Warning in simpleLoess(y, x, w, span, degree = degree, parametric = parametric, : radius 2.5e-05
```

```
## Warning in simpleLoess(y, x, w, span, degree = degree, parametric = parametric, : all data on boundary of neighborhood. make span bigger
```

```
## Warning in simpleLoess(y, x, w, span, degree = degree, parametric = parametric, : pseudoinverse used at -0.005
```

```
## Warning in simpleLoess(y, x, w, span, degree = degree, parametric = parametric, : neighborhood radius 0.005
```

```
## Warning in simpleLoess(y, x, w, span, degree = degree, parametric = parametric, : reciprocal condition number 1
```

```
## Warning in simpleLoess(y, x, w, span, degree = degree, parametric = parametric, : There are other near singularities as well. 1.01
```

```
## Warning in simpleLoess(y, x, w, span, degree = degree, parametric = parametric, : zero-width neighborhood. make span bigger
```

```
## Warning: Computation failed in `stat_smooth()`:
## NA/NaN/Inf in foreign function call (arg 5)
```

```
## Warning in simpleLoess(y, x, w, span, degree = degree, parametric = parametric, : at -0.005
```

```
## Warning in simpleLoess(y, x, w, span, degree = degree, parametric = parametric, : radius 2.5e-05
```

```
## Warning in simpleLoess(y, x, w, span, degree = degree, parametric = parametric, : all data on boundary of neighborhood. make span bigger
```

```
## Warning in simpleLoess(y, x, w, span, degree = degree, parametric = parametric, : pseudoinverse used at -0.005
```

```
## Warning in simpleLoess(y, x, w, span, degree = degree, parametric = parametric, : neighborhood radius 0.005
```

```
## Warning in simpleLoess(y, x, w, span, degree = degree, parametric = parametric, : reciprocal condition number 1
```

```
## Warning in simpleLoess(y, x, w, span, degree = degree, parametric = parametric, : There are other near singularities as well. 1.01
```

```
## Warning in simpleLoess(y, x, w, span, degree = degree, parametric = parametric, : zero-width neighborhood. make span bigger
```

```
## Warning: Computation failed in `stat_smooth()`:
## NA/NaN/Inf in foreign function call (arg 5)
```

```
## Warning in simpleLoess(y, x, w, span, degree = degree, parametric = parametric, : pseudoinverse used at -0.005
```

```
## Warning in simpleLoess(y, x, w, span, degree = degree, parametric = parametric, : neighborhood radius 1.005
```

```
## Warning in simpleLoess(y, x, w, span, degree = degree, parametric = parametric, : reciprocal condition number 0
```

```
## Warning in simpleLoess(y, x, w, span, degree = degree, parametric = parametric, : There are other near singularities as well. 1.01
```

```
## Warning in predLoess(object$y, object$x, newx = if (is.null(newdata)) object$x else if (is.data.frame(newdata)) as.matrix(model.frame(delete.response(terms(object)), : pseudoinverse used at -0.005
```

```
## Warning in predLoess(object$y, object$x, newx = if (is.null(newdata)) object$x else if (is.data.frame(newdata)) as.matrix(model.frame(delete.response(terms(object)), : neighborhood radius 1.005
```

```
## Warning in predLoess(object$y, object$x, newx = if (is.null(newdata)) object$x else if (is.data.frame(newdata)) as.matrix(model.frame(delete.response(terms(object)), : reciprocal condition number 0
```

```
## Warning in predLoess(object$y, object$x, newx = if (is.null(newdata)) object$x else if (is.data.frame(newdata)) as.matrix(model.frame(delete.response(terms(object)), : There are other near
## singularities as well. 1.01
```

```
## Warning in simpleLoess(y, x, w, span, degree = degree, parametric = parametric, : at -0.005
```

```
## Warning in simpleLoess(y, x, w, span, degree = degree, parametric = parametric, : radius 2.5e-05
```

```
## Warning in simpleLoess(y, x, w, span, degree = degree, parametric = parametric, : all data on boundary of neighborhood. make span bigger
```

```
## Warning in simpleLoess(y, x, w, span, degree = degree, parametric = parametric, : pseudoinverse used at -0.005
```

```
## Warning in simpleLoess(y, x, w, span, degree = degree, parametric = parametric, : neighborhood radius 0.005
```

```
## Warning in simpleLoess(y, x, w, span, degree = degree, parametric = parametric, : reciprocal condition number 1
```

```
## Warning in simpleLoess(y, x, w, span, degree = degree, parametric = parametric, : There are other near singularities as well. 1.01
```

```
## Warning in simpleLoess(y, x, w, span, degree = degree, parametric = parametric, : zero-width neighborhood. make span bigger
```

```
## Warning: Computation failed in `stat_smooth()`:
## NA/NaN/Inf in foreign function call (arg 5)
```

```
## Warning in simpleLoess(y, x, w, span, degree = degree, parametric = parametric, : at -0.005
```

```
## Warning in simpleLoess(y, x, w, span, degree = degree, parametric = parametric, : radius 2.5e-05
```

```
## Warning in simpleLoess(y, x, w, span, degree = degree, parametric = parametric, : all data on boundary of neighborhood. make span bigger
```

```
## Warning in simpleLoess(y, x, w, span, degree = degree, parametric = parametric, : pseudoinverse used at -0.005
```

```
## Warning in simpleLoess(y, x, w, span, degree = degree, parametric = parametric, : neighborhood radius 0.005
```

```
## Warning in simpleLoess(y, x, w, span, degree = degree, parametric = parametric, : reciprocal condition number 1
```

```
## Warning in simpleLoess(y, x, w, span, degree = degree, parametric = parametric, : There are other near singularities as well. 1.01
```

```
## Warning in simpleLoess(y, x, w, span, degree = degree, parametric = parametric, : zero-width neighborhood. make span bigger
```

```
## Warning: Computation failed in `stat_smooth()`:
## NA/NaN/Inf in foreign function call (arg 5)
```

```
## Warning in simpleLoess(y, x, w, span, degree = degree, parametric = parametric, : at -0.005
```

```
## Warning in simpleLoess(y, x, w, span, degree = degree, parametric = parametric, : radius 2.5e-05
```

```
## Warning in simpleLoess(y, x, w, span, degree = degree, parametric = parametric, : all data on boundary of neighborhood. make span bigger
```

```
## Warning in simpleLoess(y, x, w, span, degree = degree, parametric = parametric, : pseudoinverse used at -0.005
```

```
## Warning in simpleLoess(y, x, w, span, degree = degree, parametric = parametric, : neighborhood radius 0.005
```

```
## Warning in simpleLoess(y, x, w, span, degree = degree, parametric = parametric, : reciprocal condition number 1
```

```
## Warning in simpleLoess(y, x, w, span, degree = degree, parametric = parametric, : There are other near singularities as well. 1.01
```

```
## Warning in simpleLoess(y, x, w, span, degree = degree, parametric = parametric, : zero-width neighborhood. make span bigger
```

```
## Warning: Computation failed in `stat_smooth()`:
## NA/NaN/Inf in foreign function call (arg 5)
```

```
## Warning in simpleLoess(y, x, w, span, degree = degree, parametric = parametric, : at -0.005
```

```
## Warning in simpleLoess(y, x, w, span, degree = degree, parametric = parametric, : radius 2.5e-05
```

```
## Warning in simpleLoess(y, x, w, span, degree = degree, parametric = parametric, : all data on boundary of neighborhood. make span bigger
```

```
## Warning in simpleLoess(y, x, w, span, degree = degree, parametric = parametric, : pseudoinverse used at -0.005
```

```
## Warning in simpleLoess(y, x, w, span, degree = degree, parametric = parametric, : neighborhood radius 0.005
```

```
## Warning in simpleLoess(y, x, w, span, degree = degree, parametric = parametric, : reciprocal condition number 1
```

```
## Warning in simpleLoess(y, x, w, span, degree = degree, parametric = parametric, : There are other near singularities as well. 1.01
```

```
## Warning in simpleLoess(y, x, w, span, degree = degree, parametric = parametric, : zero-width neighborhood. make span bigger
```

```
## Warning: Computation failed in `stat_smooth()`:
## NA/NaN/Inf in foreign function call (arg 5)
```

```
## Warning in simpleLoess(y, x, w, span, degree = degree, parametric = parametric, : at -0.005
```

```
## Warning in simpleLoess(y, x, w, span, degree = degree, parametric = parametric, : radius 2.5e-05
```

```
## Warning in simpleLoess(y, x, w, span, degree = degree, parametric = parametric, : all data on boundary of neighborhood. make span bigger
```

```
## Warning in simpleLoess(y, x, w, span, degree = degree, parametric = parametric, : pseudoinverse used at -0.005
```

```
## Warning in simpleLoess(y, x, w, span, degree = degree, parametric = parametric, : neighborhood radius 0.005
```

```
## Warning in simpleLoess(y, x, w, span, degree = degree, parametric = parametric, : reciprocal condition number 1
```

```
## Warning in simpleLoess(y, x, w, span, degree = degree, parametric = parametric, : There are other near singularities as well. 1.01
```

```
## Warning in simpleLoess(y, x, w, span, degree = degree, parametric = parametric, : zero-width neighborhood. make span bigger
```

```
## Warning: Computation failed in `stat_smooth()`:
## NA/NaN/Inf in foreign function call (arg 5)
```

```
## Warning in simpleLoess(y, x, w, span, degree = degree, parametric = parametric, : at -0.005
```

```
## Warning in simpleLoess(y, x, w, span, degree = degree, parametric = parametric, : radius 2.5e-05
```

```
## Warning in simpleLoess(y, x, w, span, degree = degree, parametric = parametric, : all data on boundary of neighborhood. make span bigger
```

```
## Warning in simpleLoess(y, x, w, span, degree = degree, parametric = parametric, : pseudoinverse used at -0.005
```

```
## Warning in simpleLoess(y, x, w, span, degree = degree, parametric = parametric, : neighborhood radius 0.005
```

```
## Warning in simpleLoess(y, x, w, span, degree = degree, parametric = parametric, : reciprocal condition number 1
```

```
## Warning in simpleLoess(y, x, w, span, degree = degree, parametric = parametric, : There are other near singularities as well. 1.01
```

```
## Warning in simpleLoess(y, x, w, span, degree = degree, parametric = parametric, : zero-width neighborhood. make span bigger
```

```
## Warning: Computation failed in `stat_smooth()`:
## NA/NaN/Inf in foreign function call (arg 5)
```

```
## Warning in simpleLoess(y, x, w, span, degree = degree, parametric = parametric, : at -0.005
```

```
## Warning in simpleLoess(y, x, w, span, degree = degree, parametric = parametric, : radius 2.5e-05
```

```
## Warning in simpleLoess(y, x, w, span, degree = degree, parametric = parametric, : all data on boundary of neighborhood. make span bigger
```

```
## Warning in simpleLoess(y, x, w, span, degree = degree, parametric = parametric, : pseudoinverse used at -0.005
```

```
## Warning in simpleLoess(y, x, w, span, degree = degree, parametric = parametric, : neighborhood radius 0.005
```

```
## Warning in simpleLoess(y, x, w, span, degree = degree, parametric = parametric, : reciprocal condition number 1
```

```
## Warning in simpleLoess(y, x, w, span, degree = degree, parametric = parametric, : There are other near singularities as well. 1.01
```

```
## Warning in simpleLoess(y, x, w, span, degree = degree, parametric = parametric, : zero-width neighborhood. make span bigger
```

```
## Warning: Computation failed in `stat_smooth()`:
## NA/NaN/Inf in foreign function call (arg 5)
```

```
## Warning in simpleLoess(y, x, w, span, degree = degree, parametric = parametric, : at -0.005
```

```
## Warning in simpleLoess(y, x, w, span, degree = degree, parametric = parametric, : radius 2.5e-05
```

```
## Warning in simpleLoess(y, x, w, span, degree = degree, parametric = parametric, : all data on boundary of neighborhood. make span bigger
```

```
## Warning in simpleLoess(y, x, w, span, degree = degree, parametric = parametric, : pseudoinverse used at -0.005
```

```
## Warning in simpleLoess(y, x, w, span, degree = degree, parametric = parametric, : neighborhood radius 0.005
```

```
## Warning in simpleLoess(y, x, w, span, degree = degree, parametric = parametric, : reciprocal condition number 1
```

```
## Warning in simpleLoess(y, x, w, span, degree = degree, parametric = parametric, : There are other near singularities as well. 1.01
```

```
## Warning in simpleLoess(y, x, w, span, degree = degree, parametric = parametric, : zero-width neighborhood. make span bigger
```

```
## Warning: Computation failed in `stat_smooth()`:
## NA/NaN/Inf in foreign function call (arg 5)
```

```
## Warning in simpleLoess(y, x, w, span, degree = degree, parametric = parametric, : at -0.005
```

```
## Warning in simpleLoess(y, x, w, span, degree = degree, parametric = parametric, : radius 2.5e-05
```

```
## Warning in simpleLoess(y, x, w, span, degree = degree, parametric = parametric, : all data on boundary of neighborhood. make span bigger
```

```
## Warning in simpleLoess(y, x, w, span, degree = degree, parametric = parametric, : pseudoinverse used at -0.005
```

```
## Warning in simpleLoess(y, x, w, span, degree = degree, parametric = parametric, : neighborhood radius 0.005
```

```
## Warning in simpleLoess(y, x, w, span, degree = degree, parametric = parametric, : reciprocal condition number 1
```

```
## Warning in simpleLoess(y, x, w, span, degree = degree, parametric = parametric, : There are other near singularities as well. 1.01
```

```
## Warning in simpleLoess(y, x, w, span, degree = degree, parametric = parametric, : zero-width neighborhood. make span bigger
```

```
## Warning: Computation failed in `stat_smooth()`:
## NA/NaN/Inf in foreign function call (arg 5)
```

```
## Warning in simpleLoess(y, x, w, span, degree = degree, parametric = parametric, : at -0.005
```

```
## Warning in simpleLoess(y, x, w, span, degree = degree, parametric = parametric, : radius 2.5e-05
```

```
## Warning in simpleLoess(y, x, w, span, degree = degree, parametric = parametric, : all data on boundary of neighborhood. make span bigger
```

```
## Warning in simpleLoess(y, x, w, span, degree = degree, parametric = parametric, : pseudoinverse used at -0.005
```

```
## Warning in simpleLoess(y, x, w, span, degree = degree, parametric = parametric, : neighborhood radius 0.005
```

```
## Warning in simpleLoess(y, x, w, span, degree = degree, parametric = parametric, : reciprocal condition number 1
```

```
## Warning in simpleLoess(y, x, w, span, degree = degree, parametric = parametric, : There are other near singularities as well. 1.01
```

```
## Warning in simpleLoess(y, x, w, span, degree = degree, parametric = parametric, : zero-width neighborhood. make span bigger
```

```
## Warning: Computation failed in `stat_smooth()`:
## NA/NaN/Inf in foreign function call (arg 5)
```

```
## Warning in simpleLoess(y, x, w, span, degree = degree, parametric = parametric, : at -0.005
```

```
## Warning in simpleLoess(y, x, w, span, degree = degree, parametric = parametric, : radius 2.5e-05
```

```
## Warning in simpleLoess(y, x, w, span, degree = degree, parametric = parametric, : all data on boundary of neighborhood. make span bigger
```

```
## Warning in simpleLoess(y, x, w, span, degree = degree, parametric = parametric, : pseudoinverse used at -0.005
```

```
## Warning in simpleLoess(y, x, w, span, degree = degree, parametric = parametric, : neighborhood radius 0.005
```

```
## Warning in simpleLoess(y, x, w, span, degree = degree, parametric = parametric, : reciprocal condition number 1
```

```
## Warning in simpleLoess(y, x, w, span, degree = degree, parametric = parametric, : There are other near singularities as well. 1.01
```

```
## Warning in simpleLoess(y, x, w, span, degree = degree, parametric = parametric, : zero-width neighborhood. make span bigger
```

```
## Warning: Computation failed in `stat_smooth()`:
## NA/NaN/Inf in foreign function call (arg 5)
```

```
## Warning in simpleLoess(y, x, w, span, degree = degree, parametric = parametric, : at -0.005
```

```
## Warning in simpleLoess(y, x, w, span, degree = degree, parametric = parametric, : radius 2.5e-05
```

```
## Warning in simpleLoess(y, x, w, span, degree = degree, parametric = parametric, : all data on boundary of neighborhood. make span bigger
```

```
## Warning in simpleLoess(y, x, w, span, degree = degree, parametric = parametric, : pseudoinverse used at -0.005
```

```
## Warning in simpleLoess(y, x, w, span, degree = degree, parametric = parametric, : neighborhood radius 0.005
```

```
## Warning in simpleLoess(y, x, w, span, degree = degree, parametric = parametric, : reciprocal condition number 1
```

```
## Warning in simpleLoess(y, x, w, span, degree = degree, parametric = parametric, : There are other near singularities as well. 1.01
```

```
## Warning in simpleLoess(y, x, w, span, degree = degree, parametric = parametric, : zero-width neighborhood. make span bigger
```

```
## Warning: Computation failed in `stat_smooth()`:
## NA/NaN/Inf in foreign function call (arg 5)
```

```
## Warning in simpleLoess(y, x, w, span, degree = degree, parametric = parametric, : pseudoinverse used at -0.005
```

```
## Warning in simpleLoess(y, x, w, span, degree = degree, parametric = parametric, : neighborhood radius 1.005
```

```
## Warning in simpleLoess(y, x, w, span, degree = degree, parametric = parametric, : reciprocal condition number 0
```

```
## Warning in simpleLoess(y, x, w, span, degree = degree, parametric = parametric, : There are other near singularities as well. 1.01
```

```
## Warning in predLoess(object$y, object$x, newx = if (is.null(newdata)) object$x else if (is.data.frame(newdata)) as.matrix(model.frame(delete.response(terms(object)), : pseudoinverse used at -0.005
```

```
## Warning in predLoess(object$y, object$x, newx = if (is.null(newdata)) object$x else if (is.data.frame(newdata)) as.matrix(model.frame(delete.response(terms(object)), : neighborhood radius 1.005
```

```
## Warning in predLoess(object$y, object$x, newx = if (is.null(newdata)) object$x else if (is.data.frame(newdata)) as.matrix(model.frame(delete.response(terms(object)), : reciprocal condition number 0
```

```
## Warning in predLoess(object$y, object$x, newx = if (is.null(newdata)) object$x else if (is.data.frame(newdata)) as.matrix(model.frame(delete.response(terms(object)), : There are other near
## singularities as well. 1.01
```

```
## Warning in simpleLoess(y, x, w, span, degree = degree, parametric = parametric, : at -0.005
```

```
## Warning in simpleLoess(y, x, w, span, degree = degree, parametric = parametric, : radius 2.5e-05
```

```
## Warning in simpleLoess(y, x, w, span, degree = degree, parametric = parametric, : all data on boundary of neighborhood. make span bigger
```

```
## Warning in simpleLoess(y, x, w, span, degree = degree, parametric = parametric, : pseudoinverse used at -0.005
```

```
## Warning in simpleLoess(y, x, w, span, degree = degree, parametric = parametric, : neighborhood radius 0.005
```

```
## Warning in simpleLoess(y, x, w, span, degree = degree, parametric = parametric, : reciprocal condition number 1
```

```
## Warning in simpleLoess(y, x, w, span, degree = degree, parametric = parametric, : There are other near singularities as well. 1.01
```

```
## Warning in simpleLoess(y, x, w, span, degree = degree, parametric = parametric, : zero-width neighborhood. make span bigger
```

```
## Warning: Computation failed in `stat_smooth()`:
## NA/NaN/Inf in foreign function call (arg 5)
```

```
## Warning in simpleLoess(y, x, w, span, degree = degree, parametric = parametric, : pseudoinverse used at -0.005
```

```
## Warning in simpleLoess(y, x, w, span, degree = degree, parametric = parametric, : neighborhood radius 1.005
```

```
## Warning in simpleLoess(y, x, w, span, degree = degree, parametric = parametric, : reciprocal condition number 0
```

```
## Warning in simpleLoess(y, x, w, span, degree = degree, parametric = parametric, : There are other near singularities as well. 1.01
```

```
## Warning in predLoess(object$y, object$x, newx = if (is.null(newdata)) object$x else if (is.data.frame(newdata)) as.matrix(model.frame(delete.response(terms(object)), : pseudoinverse used at -0.005
```

```
## Warning in predLoess(object$y, object$x, newx = if (is.null(newdata)) object$x else if (is.data.frame(newdata)) as.matrix(model.frame(delete.response(terms(object)), : neighborhood radius 1.005
```

```
## Warning in predLoess(object$y, object$x, newx = if (is.null(newdata)) object$x else if (is.data.frame(newdata)) as.matrix(model.frame(delete.response(terms(object)), : reciprocal condition number 0
```

```
## Warning in predLoess(object$y, object$x, newx = if (is.null(newdata)) object$x else if (is.data.frame(newdata)) as.matrix(model.frame(delete.response(terms(object)), : There are other near
## singularities as well. 1.01
```

```
## Warning in simpleLoess(y, x, w, span, degree = degree, parametric = parametric, : at -0.005
```

```
## Warning in simpleLoess(y, x, w, span, degree = degree, parametric = parametric, : radius 2.5e-05
```

```
## Warning in simpleLoess(y, x, w, span, degree = degree, parametric = parametric, : all data on boundary of neighborhood. make span bigger
```

```
## Warning in simpleLoess(y, x, w, span, degree = degree, parametric = parametric, : pseudoinverse used at -0.005
```

```
## Warning in simpleLoess(y, x, w, span, degree = degree, parametric = parametric, : neighborhood radius 0.005
```

```
## Warning in simpleLoess(y, x, w, span, degree = degree, parametric = parametric, : reciprocal condition number 1
```

```
## Warning in simpleLoess(y, x, w, span, degree = degree, parametric = parametric, : There are other near singularities as well. 1.01
```

```
## Warning in simpleLoess(y, x, w, span, degree = degree, parametric = parametric, : zero-width neighborhood. make span bigger
```

```
## Warning: Computation failed in `stat_smooth()`:
## NA/NaN/Inf in foreign function call (arg 5)
```

```
## Warning in simpleLoess(y, x, w, span, degree = degree, parametric = parametric, : pseudoinverse used at -0.005
```

```
## Warning in simpleLoess(y, x, w, span, degree = degree, parametric = parametric, : neighborhood radius 1.005
```

```
## Warning in simpleLoess(y, x, w, span, degree = degree, parametric = parametric, : reciprocal condition number 0
```

```
## Warning in simpleLoess(y, x, w, span, degree = degree, parametric = parametric, : There are other near singularities as well. 1.01
```

```
## Warning in predLoess(object$y, object$x, newx = if (is.null(newdata)) object$x else if (is.data.frame(newdata)) as.matrix(model.frame(delete.response(terms(object)), : pseudoinverse used at -0.005
```

```
## Warning in predLoess(object$y, object$x, newx = if (is.null(newdata)) object$x else if (is.data.frame(newdata)) as.matrix(model.frame(delete.response(terms(object)), : neighborhood radius 1.005
```

```
## Warning in predLoess(object$y, object$x, newx = if (is.null(newdata)) object$x else if (is.data.frame(newdata)) as.matrix(model.frame(delete.response(terms(object)), : reciprocal condition number 0
```

```
## Warning in predLoess(object$y, object$x, newx = if (is.null(newdata)) object$x else if (is.data.frame(newdata)) as.matrix(model.frame(delete.response(terms(object)), : There are other near
## singularities as well. 1.01
```

```
## Warning in simpleLoess(y, x, w, span, degree = degree, parametric = parametric, : at -0.005
```

```
## Warning in simpleLoess(y, x, w, span, degree = degree, parametric = parametric, : radius 2.5e-05
```

```
## Warning in simpleLoess(y, x, w, span, degree = degree, parametric = parametric, : all data on boundary of neighborhood. make span bigger
```

```
## Warning in simpleLoess(y, x, w, span, degree = degree, parametric = parametric, : pseudoinverse used at -0.005
```

```
## Warning in simpleLoess(y, x, w, span, degree = degree, parametric = parametric, : neighborhood radius 0.005
```

```
## Warning in simpleLoess(y, x, w, span, degree = degree, parametric = parametric, : reciprocal condition number 1
```

```
## Warning in simpleLoess(y, x, w, span, degree = degree, parametric = parametric, : There are other near singularities as well. 1.01
```

```
## Warning in simpleLoess(y, x, w, span, degree = degree, parametric = parametric, : zero-width neighborhood. make span bigger
```

```
## Warning: Computation failed in `stat_smooth()`:
## NA/NaN/Inf in foreign function call (arg 5)
```

```
## Warning in simpleLoess(y, x, w, span, degree = degree, parametric = parametric, : at -0.005
```

```
## Warning in simpleLoess(y, x, w, span, degree = degree, parametric = parametric, : radius 2.5e-05
```

```
## Warning in simpleLoess(y, x, w, span, degree = degree, parametric = parametric, : all data on boundary of neighborhood. make span bigger
```

```
## Warning in simpleLoess(y, x, w, span, degree = degree, parametric = parametric, : pseudoinverse used at -0.005
```

```
## Warning in simpleLoess(y, x, w, span, degree = degree, parametric = parametric, : neighborhood radius 0.005
```

```
## Warning in simpleLoess(y, x, w, span, degree = degree, parametric = parametric, : reciprocal condition number 1
```

```
## Warning in simpleLoess(y, x, w, span, degree = degree, parametric = parametric, : There are other near singularities as well. 1.01
```

```
## Warning in simpleLoess(y, x, w, span, degree = degree, parametric = parametric, : zero-width neighborhood. make span bigger
```

```
## Warning: Computation failed in `stat_smooth()`:
## NA/NaN/Inf in foreign function call (arg 5)
```

```
## Warning in simpleLoess(y, x, w, span, degree = degree, parametric = parametric, : at -0.005
```

```
## Warning in simpleLoess(y, x, w, span, degree = degree, parametric = parametric, : radius 2.5e-05
```

```
## Warning in simpleLoess(y, x, w, span, degree = degree, parametric = parametric, : all data on boundary of neighborhood. make span bigger
```

```
## Warning in simpleLoess(y, x, w, span, degree = degree, parametric = parametric, : pseudoinverse used at -0.005
```

```
## Warning in simpleLoess(y, x, w, span, degree = degree, parametric = parametric, : neighborhood radius 0.005
```

```
## Warning in simpleLoess(y, x, w, span, degree = degree, parametric = parametric, : reciprocal condition number 1
```

```
## Warning in simpleLoess(y, x, w, span, degree = degree, parametric = parametric, : There are other near singularities as well. 1.01
```

```
## Warning in simpleLoess(y, x, w, span, degree = degree, parametric = parametric, : zero-width neighborhood. make span bigger
```

```
## Warning: Computation failed in `stat_smooth()`:
## NA/NaN/Inf in foreign function call (arg 5)
```

```
## Warning in simpleLoess(y, x, w, span, degree = degree, parametric = parametric, : at -0.005
```

```
## Warning in simpleLoess(y, x, w, span, degree = degree, parametric = parametric, : radius 2.5e-05
```

```
## Warning in simpleLoess(y, x, w, span, degree = degree, parametric = parametric, : all data on boundary of neighborhood. make span bigger
```

```
## Warning in simpleLoess(y, x, w, span, degree = degree, parametric = parametric, : pseudoinverse used at -0.005
```

```
## Warning in simpleLoess(y, x, w, span, degree = degree, parametric = parametric, : neighborhood radius 0.005
```

```
## Warning in simpleLoess(y, x, w, span, degree = degree, parametric = parametric, : reciprocal condition number 1
```

```
## Warning in simpleLoess(y, x, w, span, degree = degree, parametric = parametric, : There are other near singularities as well. 1.01
```

```
## Warning in simpleLoess(y, x, w, span, degree = degree, parametric = parametric, : zero-width neighborhood. make span bigger
```

```
## Warning: Computation failed in `stat_smooth()`:
## NA/NaN/Inf in foreign function call (arg 5)
```

```
## Warning in simpleLoess(y, x, w, span, degree = degree, parametric = parametric, : at -0.005
```

```
## Warning in simpleLoess(y, x, w, span, degree = degree, parametric = parametric, : radius 2.5e-05
```

```
## Warning in simpleLoess(y, x, w, span, degree = degree, parametric = parametric, : all data on boundary of neighborhood. make span bigger
```

```
## Warning in simpleLoess(y, x, w, span, degree = degree, parametric = parametric, : pseudoinverse used at -0.005
```

```
## Warning in simpleLoess(y, x, w, span, degree = degree, parametric = parametric, : neighborhood radius 0.005
```

```
## Warning in simpleLoess(y, x, w, span, degree = degree, parametric = parametric, : reciprocal condition number 1
```

```
## Warning in simpleLoess(y, x, w, span, degree = degree, parametric = parametric, : There are other near singularities as well. 1.01
```

```
## Warning in simpleLoess(y, x, w, span, degree = degree, parametric = parametric, : zero-width neighborhood. make span bigger
```

```
## Warning: Computation failed in `stat_smooth()`:
## NA/NaN/Inf in foreign function call (arg 5)
```

```
## Warning in simpleLoess(y, x, w, span, degree = degree, parametric = parametric, : at -0.005
```

```
## Warning in simpleLoess(y, x, w, span, degree = degree, parametric = parametric, : radius 2.5e-05
```

```
## Warning in simpleLoess(y, x, w, span, degree = degree, parametric = parametric, : all data on boundary of neighborhood. make span bigger
```

```
## Warning in simpleLoess(y, x, w, span, degree = degree, parametric = parametric, : pseudoinverse used at -0.005
```

```
## Warning in simpleLoess(y, x, w, span, degree = degree, parametric = parametric, : neighborhood radius 0.005
```

```
## Warning in simpleLoess(y, x, w, span, degree = degree, parametric = parametric, : reciprocal condition number 1
```

```
## Warning in simpleLoess(y, x, w, span, degree = degree, parametric = parametric, : There are other near singularities as well. 1.01
```

```
## Warning in simpleLoess(y, x, w, span, degree = degree, parametric = parametric, : zero-width neighborhood. make span bigger
```

```
## Warning: Computation failed in `stat_smooth()`:
## NA/NaN/Inf in foreign function call (arg 5)
```

```
## Warning in simpleLoess(y, x, w, span, degree = degree, parametric = parametric, : at -0.005
```

```
## Warning in simpleLoess(y, x, w, span, degree = degree, parametric = parametric, : radius 2.5e-05
```

```
## Warning in simpleLoess(y, x, w, span, degree = degree, parametric = parametric, : all data on boundary of neighborhood. make span bigger
```

```
## Warning in simpleLoess(y, x, w, span, degree = degree, parametric = parametric, : pseudoinverse used at -0.005
```

```
## Warning in simpleLoess(y, x, w, span, degree = degree, parametric = parametric, : neighborhood radius 0.005
```

```
## Warning in simpleLoess(y, x, w, span, degree = degree, parametric = parametric, : reciprocal condition number 1
```

```
## Warning in simpleLoess(y, x, w, span, degree = degree, parametric = parametric, : There are other near singularities as well. 1.01
```

```
## Warning in simpleLoess(y, x, w, span, degree = degree, parametric = parametric, : zero-width neighborhood. make span bigger
```

```
## Warning: Computation failed in `stat_smooth()`:
## NA/NaN/Inf in foreign function call (arg 5)
```

```
## Warning in simpleLoess(y, x, w, span, degree = degree, parametric = parametric, : at -0.005
```

```
## Warning in simpleLoess(y, x, w, span, degree = degree, parametric = parametric, : radius 2.5e-05
```

```
## Warning in simpleLoess(y, x, w, span, degree = degree, parametric = parametric, : all data on boundary of neighborhood. make span bigger
```

```
## Warning in simpleLoess(y, x, w, span, degree = degree, parametric = parametric, : pseudoinverse used at -0.005
```

```
## Warning in simpleLoess(y, x, w, span, degree = degree, parametric = parametric, : neighborhood radius 0.005
```

```
## Warning in simpleLoess(y, x, w, span, degree = degree, parametric = parametric, : reciprocal condition number 1
```

```
## Warning in simpleLoess(y, x, w, span, degree = degree, parametric = parametric, : There are other near singularities as well. 1.01
```

```
## Warning in simpleLoess(y, x, w, span, degree = degree, parametric = parametric, : zero-width neighborhood. make span bigger
```

```
## Warning: Computation failed in `stat_smooth()`:
## NA/NaN/Inf in foreign function call (arg 5)
```

```
## Warning in simpleLoess(y, x, w, span, degree = degree, parametric = parametric, : at -0.005
```

```
## Warning in simpleLoess(y, x, w, span, degree = degree, parametric = parametric, : radius 2.5e-05
```

```
## Warning in simpleLoess(y, x, w, span, degree = degree, parametric = parametric, : all data on boundary of neighborhood. make span bigger
```

```
## Warning in simpleLoess(y, x, w, span, degree = degree, parametric = parametric, : pseudoinverse used at -0.005
```

```
## Warning in simpleLoess(y, x, w, span, degree = degree, parametric = parametric, : neighborhood radius 0.005
```

```
## Warning in simpleLoess(y, x, w, span, degree = degree, parametric = parametric, : reciprocal condition number 1
```

```
## Warning in simpleLoess(y, x, w, span, degree = degree, parametric = parametric, : There are other near singularities as well. 1.01
```

```
## Warning in simpleLoess(y, x, w, span, degree = degree, parametric = parametric, : zero-width neighborhood. make span bigger
```

```
## Warning: Computation failed in `stat_smooth()`:
## NA/NaN/Inf in foreign function call (arg 5)
```

```
## Warning in simpleLoess(y, x, w, span, degree = degree, parametric = parametric, : at -0.005
```

```
## Warning in simpleLoess(y, x, w, span, degree = degree, parametric = parametric, : radius 2.5e-05
```

```
## Warning in simpleLoess(y, x, w, span, degree = degree, parametric = parametric, : all data on boundary of neighborhood. make span bigger
```

```
## Warning in simpleLoess(y, x, w, span, degree = degree, parametric = parametric, : pseudoinverse used at -0.005
```

```
## Warning in simpleLoess(y, x, w, span, degree = degree, parametric = parametric, : neighborhood radius 0.005
```

```
## Warning in simpleLoess(y, x, w, span, degree = degree, parametric = parametric, : reciprocal condition number 1
```

```
## Warning in simpleLoess(y, x, w, span, degree = degree, parametric = parametric, : There are other near singularities as well. 1.01
```

```
## Warning in simpleLoess(y, x, w, span, degree = degree, parametric = parametric, : zero-width neighborhood. make span bigger
```

```
## Warning: Computation failed in `stat_smooth()`:
## NA/NaN/Inf in foreign function call (arg 5)
```

```
## Warning in simpleLoess(y, x, w, span, degree = degree, parametric = parametric, : at -0.005
```

```
## Warning in simpleLoess(y, x, w, span, degree = degree, parametric = parametric, : radius 2.5e-05
```

```
## Warning in simpleLoess(y, x, w, span, degree = degree, parametric = parametric, : all data on boundary of neighborhood. make span bigger
```

```
## Warning in simpleLoess(y, x, w, span, degree = degree, parametric = parametric, : pseudoinverse used at -0.005
```

```
## Warning in simpleLoess(y, x, w, span, degree = degree, parametric = parametric, : neighborhood radius 0.005
```

```
## Warning in simpleLoess(y, x, w, span, degree = degree, parametric = parametric, : reciprocal condition number 1
```

```
## Warning in simpleLoess(y, x, w, span, degree = degree, parametric = parametric, : There are other near singularities as well. 1.01
```

```
## Warning in simpleLoess(y, x, w, span, degree = degree, parametric = parametric, : zero-width neighborhood. make span bigger
```

```
## Warning: Computation failed in `stat_smooth()`:
## NA/NaN/Inf in foreign function call (arg 5)
```

```
## Warning in simpleLoess(y, x, w, span, degree = degree, parametric = parametric, : at -0.005
```

```
## Warning in simpleLoess(y, x, w, span, degree = degree, parametric = parametric, : radius 2.5e-05
```

```
## Warning in simpleLoess(y, x, w, span, degree = degree, parametric = parametric, : all data on boundary of neighborhood. make span bigger
```

```
## Warning in simpleLoess(y, x, w, span, degree = degree, parametric = parametric, : pseudoinverse used at -0.005
```

```
## Warning in simpleLoess(y, x, w, span, degree = degree, parametric = parametric, : neighborhood radius 0.005
```

```
## Warning in simpleLoess(y, x, w, span, degree = degree, parametric = parametric, : reciprocal condition number 1
```

```
## Warning in simpleLoess(y, x, w, span, degree = degree, parametric = parametric, : There are other near singularities as well. 1.01
```

```
## Warning in simpleLoess(y, x, w, span, degree = degree, parametric = parametric, : zero-width neighborhood. make span bigger
```

```
## Warning: Computation failed in `stat_smooth()`:
## NA/NaN/Inf in foreign function call (arg 5)
```

```
## Warning in simpleLoess(y, x, w, span, degree = degree, parametric = parametric, : at -0.005
```

```
## Warning in simpleLoess(y, x, w, span, degree = degree, parametric = parametric, : radius 2.5e-05
```

```
## Warning in simpleLoess(y, x, w, span, degree = degree, parametric = parametric, : all data on boundary of neighborhood. make span bigger
```

```
## Warning in simpleLoess(y, x, w, span, degree = degree, parametric = parametric, : pseudoinverse used at -0.005
```

```
## Warning in simpleLoess(y, x, w, span, degree = degree, parametric = parametric, : neighborhood radius 0.005
```

```
## Warning in simpleLoess(y, x, w, span, degree = degree, parametric = parametric, : reciprocal condition number 1
```

```
## Warning in simpleLoess(y, x, w, span, degree = degree, parametric = parametric, : There are other near singularities as well. 1.01
```

```
## Warning in simpleLoess(y, x, w, span, degree = degree, parametric = parametric, : zero-width neighborhood. make span bigger
```

```
## Warning: Computation failed in `stat_smooth()`:
## NA/NaN/Inf in foreign function call (arg 5)
```

```
## Warning in simpleLoess(y, x, w, span, degree = degree, parametric = parametric, : at -0.005
```

```
## Warning in simpleLoess(y, x, w, span, degree = degree, parametric = parametric, : radius 2.5e-05
```

```
## Warning in simpleLoess(y, x, w, span, degree = degree, parametric = parametric, : all data on boundary of neighborhood. make span bigger
```

```
## Warning in simpleLoess(y, x, w, span, degree = degree, parametric = parametric, : pseudoinverse used at -0.005
```

```
## Warning in simpleLoess(y, x, w, span, degree = degree, parametric = parametric, : neighborhood radius 0.005
```

```
## Warning in simpleLoess(y, x, w, span, degree = degree, parametric = parametric, : reciprocal condition number 1
```

```
## Warning in simpleLoess(y, x, w, span, degree = degree, parametric = parametric, : There are other near singularities as well. 1.01
```

```
## Warning in simpleLoess(y, x, w, span, degree = degree, parametric = parametric, : zero-width neighborhood. make span bigger
```

```
## Warning: Computation failed in `stat_smooth()`:
## NA/NaN/Inf in foreign function call (arg 5)
```

```
## Warning in simpleLoess(y, x, w, span, degree = degree, parametric = parametric, : pseudoinverse used at -0.005
```

```
## Warning in simpleLoess(y, x, w, span, degree = degree, parametric = parametric, : neighborhood radius 1.005
```

```
## Warning in simpleLoess(y, x, w, span, degree = degree, parametric = parametric, : reciprocal condition number 0
```

```
## Warning in simpleLoess(y, x, w, span, degree = degree, parametric = parametric, : There are other near singularities as well. 1.01
```

```
## Warning in predLoess(object$y, object$x, newx = if (is.null(newdata)) object$x else if (is.data.frame(newdata)) as.matrix(model.frame(delete.response(terms(object)), : pseudoinverse used at -0.005
```

```
## Warning in predLoess(object$y, object$x, newx = if (is.null(newdata)) object$x else if (is.data.frame(newdata)) as.matrix(model.frame(delete.response(terms(object)), : neighborhood radius 1.005
```

```
## Warning in predLoess(object$y, object$x, newx = if (is.null(newdata)) object$x else if (is.data.frame(newdata)) as.matrix(model.frame(delete.response(terms(object)), : reciprocal condition number 0
```

```
## Warning in predLoess(object$y, object$x, newx = if (is.null(newdata)) object$x else if (is.data.frame(newdata)) as.matrix(model.frame(delete.response(terms(object)), : There are other near
## singularities as well. 1.01
```

```
## Warning in simpleLoess(y, x, w, span, degree = degree, parametric = parametric, : at -0.005
```

```
## Warning in simpleLoess(y, x, w, span, degree = degree, parametric = parametric, : radius 2.5e-05
```

```
## Warning in simpleLoess(y, x, w, span, degree = degree, parametric = parametric, : all data on boundary of neighborhood. make span bigger
```

```
## Warning in simpleLoess(y, x, w, span, degree = degree, parametric = parametric, : pseudoinverse used at -0.005
```

```
## Warning in simpleLoess(y, x, w, span, degree = degree, parametric = parametric, : neighborhood radius 0.005
```

```
## Warning in simpleLoess(y, x, w, span, degree = degree, parametric = parametric, : reciprocal condition number 1
```

```
## Warning in simpleLoess(y, x, w, span, degree = degree, parametric = parametric, : There are other near singularities as well. 1.01
```

```
## Warning in simpleLoess(y, x, w, span, degree = degree, parametric = parametric, : zero-width neighborhood. make span bigger
```

```
## Warning: Computation failed in `stat_smooth()`:
## NA/NaN/Inf in foreign function call (arg 5)
```

```
## Warning in simpleLoess(y, x, w, span, degree = degree, parametric = parametric, : at -0.005
```

```
## Warning in simpleLoess(y, x, w, span, degree = degree, parametric = parametric, : radius 2.5e-05
```

```
## Warning in simpleLoess(y, x, w, span, degree = degree, parametric = parametric, : all data on boundary of neighborhood. make span bigger
```

```
## Warning in simpleLoess(y, x, w, span, degree = degree, parametric = parametric, : pseudoinverse used at -0.005
```

```
## Warning in simpleLoess(y, x, w, span, degree = degree, parametric = parametric, : neighborhood radius 0.005
```

```
## Warning in simpleLoess(y, x, w, span, degree = degree, parametric = parametric, : reciprocal condition number 1
```

```
## Warning in simpleLoess(y, x, w, span, degree = degree, parametric = parametric, : There are other near singularities as well. 1.01
```

```
## Warning in simpleLoess(y, x, w, span, degree = degree, parametric = parametric, : zero-width neighborhood. make span bigger
```

```
## Warning: Computation failed in `stat_smooth()`:
## NA/NaN/Inf in foreign function call (arg 5)
```

```
## Warning in simpleLoess(y, x, w, span, degree = degree, parametric = parametric, : at -0.005
```

```
## Warning in simpleLoess(y, x, w, span, degree = degree, parametric = parametric, : radius 2.5e-05
```

```
## Warning in simpleLoess(y, x, w, span, degree = degree, parametric = parametric, : all data on boundary of neighborhood. make span bigger
```

```
## Warning in simpleLoess(y, x, w, span, degree = degree, parametric = parametric, : pseudoinverse used at -0.005
```

```
## Warning in simpleLoess(y, x, w, span, degree = degree, parametric = parametric, : neighborhood radius 0.005
```

```
## Warning in simpleLoess(y, x, w, span, degree = degree, parametric = parametric, : reciprocal condition number 1
```

```
## Warning in simpleLoess(y, x, w, span, degree = degree, parametric = parametric, : There are other near singularities as well. 1.01
```

```
## Warning in simpleLoess(y, x, w, span, degree = degree, parametric = parametric, : zero-width neighborhood. make span bigger
```

```
## Warning: Computation failed in `stat_smooth()`:
## NA/NaN/Inf in foreign function call (arg 5)
```

```
## Warning in simpleLoess(y, x, w, span, degree = degree, parametric = parametric, : at -0.005
```

```
## Warning in simpleLoess(y, x, w, span, degree = degree, parametric = parametric, : radius 2.5e-05
```

```
## Warning in simpleLoess(y, x, w, span, degree = degree, parametric = parametric, : all data on boundary of neighborhood. make span bigger
```

```
## Warning in simpleLoess(y, x, w, span, degree = degree, parametric = parametric, : pseudoinverse used at -0.005
```

```
## Warning in simpleLoess(y, x, w, span, degree = degree, parametric = parametric, : neighborhood radius 0.005
```

```
## Warning in simpleLoess(y, x, w, span, degree = degree, parametric = parametric, : reciprocal condition number 1
```

```
## Warning in simpleLoess(y, x, w, span, degree = degree, parametric = parametric, : There are other near singularities as well. 1.01
```

```
## Warning in simpleLoess(y, x, w, span, degree = degree, parametric = parametric, : zero-width neighborhood. make span bigger
```

```
## Warning: Computation failed in `stat_smooth()`:
## NA/NaN/Inf in foreign function call (arg 5)
```

```
## Warning in simpleLoess(y, x, w, span, degree = degree, parametric = parametric, : at -0.005
```

```
## Warning in simpleLoess(y, x, w, span, degree = degree, parametric = parametric, : radius 2.5e-05
```

```
## Warning in simpleLoess(y, x, w, span, degree = degree, parametric = parametric, : all data on boundary of neighborhood. make span bigger
```

```
## Warning in simpleLoess(y, x, w, span, degree = degree, parametric = parametric, : pseudoinverse used at -0.005
```

```
## Warning in simpleLoess(y, x, w, span, degree = degree, parametric = parametric, : neighborhood radius 0.005
```

```
## Warning in simpleLoess(y, x, w, span, degree = degree, parametric = parametric, : reciprocal condition number 1
```

```
## Warning in simpleLoess(y, x, w, span, degree = degree, parametric = parametric, : There are other near singularities as well. 1.01
```

```
## Warning in simpleLoess(y, x, w, span, degree = degree, parametric = parametric, : zero-width neighborhood. make span bigger
```

```
## Warning: Computation failed in `stat_smooth()`:
## NA/NaN/Inf in foreign function call (arg 5)
```

```
## Warning in simpleLoess(y, x, w, span, degree = degree, parametric = parametric, : at -0.005
```

```
## Warning in simpleLoess(y, x, w, span, degree = degree, parametric = parametric, : radius 2.5e-05
```

```
## Warning in simpleLoess(y, x, w, span, degree = degree, parametric = parametric, : all data on boundary of neighborhood. make span bigger
```

```
## Warning in simpleLoess(y, x, w, span, degree = degree, parametric = parametric, : pseudoinverse used at -0.005
```

```
## Warning in simpleLoess(y, x, w, span, degree = degree, parametric = parametric, : neighborhood radius 0.005
```

```
## Warning in simpleLoess(y, x, w, span, degree = degree, parametric = parametric, : reciprocal condition number 1
```

```
## Warning in simpleLoess(y, x, w, span, degree = degree, parametric = parametric, : There are other near singularities as well. 1.01
```

```
## Warning in simpleLoess(y, x, w, span, degree = degree, parametric = parametric, : zero-width neighborhood. make span bigger
```

```
## Warning: Computation failed in `stat_smooth()`:
## NA/NaN/Inf in foreign function call (arg 5)
```

```
## Warning in simpleLoess(y, x, w, span, degree = degree, parametric = parametric, : at -0.005
```

```
## Warning in simpleLoess(y, x, w, span, degree = degree, parametric = parametric, : radius 2.5e-05
```

```
## Warning in simpleLoess(y, x, w, span, degree = degree, parametric = parametric, : all data on boundary of neighborhood. make span bigger
```

```
## Warning in simpleLoess(y, x, w, span, degree = degree, parametric = parametric, : pseudoinverse used at -0.005
```

```
## Warning in simpleLoess(y, x, w, span, degree = degree, parametric = parametric, : neighborhood radius 0.005
```

```
## Warning in simpleLoess(y, x, w, span, degree = degree, parametric = parametric, : reciprocal condition number 1
```

```
## Warning in simpleLoess(y, x, w, span, degree = degree, parametric = parametric, : There are other near singularities as well. 1.01
```

```
## Warning in simpleLoess(y, x, w, span, degree = degree, parametric = parametric, : zero-width neighborhood. make span bigger
```

```
## Warning: Computation failed in `stat_smooth()`:
## NA/NaN/Inf in foreign function call (arg 5)
```

```
## Warning in simpleLoess(y, x, w, span, degree = degree, parametric = parametric, : at -0.005
```

```
## Warning in simpleLoess(y, x, w, span, degree = degree, parametric = parametric, : radius 2.5e-05
```

```
## Warning in simpleLoess(y, x, w, span, degree = degree, parametric = parametric, : all data on boundary of neighborhood. make span bigger
```

```
## Warning in simpleLoess(y, x, w, span, degree = degree, parametric = parametric, : pseudoinverse used at -0.005
```

```
## Warning in simpleLoess(y, x, w, span, degree = degree, parametric = parametric, : neighborhood radius 0.005
```

```
## Warning in simpleLoess(y, x, w, span, degree = degree, parametric = parametric, : reciprocal condition number 1
```

```
## Warning in simpleLoess(y, x, w, span, degree = degree, parametric = parametric, : There are other near singularities as well. 1.01
```

```
## Warning in simpleLoess(y, x, w, span, degree = degree, parametric = parametric, : zero-width neighborhood. make span bigger
```

```
## Warning: Computation failed in `stat_smooth()`:
## NA/NaN/Inf in foreign function call (arg 5)
```

```
## Warning in simpleLoess(y, x, w, span, degree = degree, parametric = parametric, : at -0.005
```

```
## Warning in simpleLoess(y, x, w, span, degree = degree, parametric = parametric, : radius 2.5e-05
```

```
## Warning in simpleLoess(y, x, w, span, degree = degree, parametric = parametric, : all data on boundary of neighborhood. make span bigger
```

```
## Warning in simpleLoess(y, x, w, span, degree = degree, parametric = parametric, : pseudoinverse used at -0.005
```

```
## Warning in simpleLoess(y, x, w, span, degree = degree, parametric = parametric, : neighborhood radius 0.005
```

```
## Warning in simpleLoess(y, x, w, span, degree = degree, parametric = parametric, : reciprocal condition number 1
```

```
## Warning in simpleLoess(y, x, w, span, degree = degree, parametric = parametric, : There are other near singularities as well. 1.01
```

```
## Warning in simpleLoess(y, x, w, span, degree = degree, parametric = parametric, : zero-width neighborhood. make span bigger
```

```
## Warning: Computation failed in `stat_smooth()`:
## NA/NaN/Inf in foreign function call (arg 5)
```

```
## Warning in simpleLoess(y, x, w, span, degree = degree, parametric = parametric, : at -0.005
```

```
## Warning in simpleLoess(y, x, w, span, degree = degree, parametric = parametric, : radius 2.5e-05
```

```
## Warning in simpleLoess(y, x, w, span, degree = degree, parametric = parametric, : all data on boundary of neighborhood. make span bigger
```

```
## Warning in simpleLoess(y, x, w, span, degree = degree, parametric = parametric, : pseudoinverse used at -0.005
```

```
## Warning in simpleLoess(y, x, w, span, degree = degree, parametric = parametric, : neighborhood radius 0.005
```

```
## Warning in simpleLoess(y, x, w, span, degree = degree, parametric = parametric, : reciprocal condition number 1
```

```
## Warning in simpleLoess(y, x, w, span, degree = degree, parametric = parametric, : There are other near singularities as well. 1.01
```

```
## Warning in simpleLoess(y, x, w, span, degree = degree, parametric = parametric, : zero-width neighborhood. make span bigger
```

```
## Warning: Computation failed in `stat_smooth()`:
## NA/NaN/Inf in foreign function call (arg 5)
```

```
## Warning in simpleLoess(y, x, w, span, degree = degree, parametric = parametric, : pseudoinverse used at -0.005
```

```
## Warning in simpleLoess(y, x, w, span, degree = degree, parametric = parametric, : neighborhood radius 1.005
```

```
## Warning in simpleLoess(y, x, w, span, degree = degree, parametric = parametric, : reciprocal condition number 0
```

```
## Warning in simpleLoess(y, x, w, span, degree = degree, parametric = parametric, : There are other near singularities as well. 1.01
```

```
## Warning in predLoess(object$y, object$x, newx = if (is.null(newdata)) object$x else if (is.data.frame(newdata)) as.matrix(model.frame(delete.response(terms(object)), : pseudoinverse used at -0.005
```

```
## Warning in predLoess(object$y, object$x, newx = if (is.null(newdata)) object$x else if (is.data.frame(newdata)) as.matrix(model.frame(delete.response(terms(object)), : neighborhood radius 1.005
```

```
## Warning in predLoess(object$y, object$x, newx = if (is.null(newdata)) object$x else if (is.data.frame(newdata)) as.matrix(model.frame(delete.response(terms(object)), : reciprocal condition number 0
```

```
## Warning in predLoess(object$y, object$x, newx = if (is.null(newdata)) object$x else if (is.data.frame(newdata)) as.matrix(model.frame(delete.response(terms(object)), : There are other near
## singularities as well. 1.01
```

```
## Warning in simpleLoess(y, x, w, span, degree = degree, parametric = parametric, : at -0.005
```

```
## Warning in simpleLoess(y, x, w, span, degree = degree, parametric = parametric, : radius 2.5e-05
```

```
## Warning in simpleLoess(y, x, w, span, degree = degree, parametric = parametric, : all data on boundary of neighborhood. make span bigger
```

```
## Warning in simpleLoess(y, x, w, span, degree = degree, parametric = parametric, : pseudoinverse used at -0.005
```

```
## Warning in simpleLoess(y, x, w, span, degree = degree, parametric = parametric, : neighborhood radius 0.005
```

```
## Warning in simpleLoess(y, x, w, span, degree = degree, parametric = parametric, : reciprocal condition number 1
```

```
## Warning in simpleLoess(y, x, w, span, degree = degree, parametric = parametric, : There are other near singularities as well. 1.01
```

```
## Warning in simpleLoess(y, x, w, span, degree = degree, parametric = parametric, : zero-width neighborhood. make span bigger
```

```
## Warning: Computation failed in `stat_smooth()`:
## NA/NaN/Inf in foreign function call (arg 5)
```

```
## Warning in simpleLoess(y, x, w, span, degree = degree, parametric = parametric, : at -0.005
```

```
## Warning in simpleLoess(y, x, w, span, degree = degree, parametric = parametric, : radius 2.5e-05
```

```
## Warning in simpleLoess(y, x, w, span, degree = degree, parametric = parametric, : all data on boundary of neighborhood. make span bigger
```

```
## Warning in simpleLoess(y, x, w, span, degree = degree, parametric = parametric, : pseudoinverse used at -0.005
```

```
## Warning in simpleLoess(y, x, w, span, degree = degree, parametric = parametric, : neighborhood radius 0.005
```

```
## Warning in simpleLoess(y, x, w, span, degree = degree, parametric = parametric, : reciprocal condition number 1
```

```
## Warning in simpleLoess(y, x, w, span, degree = degree, parametric = parametric, : There are other near singularities as well. 1.01
```

```
## Warning in simpleLoess(y, x, w, span, degree = degree, parametric = parametric, : zero-width neighborhood. make span bigger
```

```
## Warning: Computation failed in `stat_smooth()`:
## NA/NaN/Inf in foreign function call (arg 5)
```

```
## Warning in simpleLoess(y, x, w, span, degree = degree, parametric = parametric, : at -0.005
```

```
## Warning in simpleLoess(y, x, w, span, degree = degree, parametric = parametric, : radius 2.5e-05
```

```
## Warning in simpleLoess(y, x, w, span, degree = degree, parametric = parametric, : all data on boundary of neighborhood. make span bigger
```

```
## Warning in simpleLoess(y, x, w, span, degree = degree, parametric = parametric, : pseudoinverse used at -0.005
```

```
## Warning in simpleLoess(y, x, w, span, degree = degree, parametric = parametric, : neighborhood radius 0.005
```

```
## Warning in simpleLoess(y, x, w, span, degree = degree, parametric = parametric, : reciprocal condition number 1
```

```
## Warning in simpleLoess(y, x, w, span, degree = degree, parametric = parametric, : There are other near singularities as well. 1.01
```

```
## Warning in simpleLoess(y, x, w, span, degree = degree, parametric = parametric, : zero-width neighborhood. make span bigger
```

```
## Warning: Computation failed in `stat_smooth()`:
## NA/NaN/Inf in foreign function call (arg 5)
```

```
## Warning in simpleLoess(y, x, w, span, degree = degree, parametric = parametric, : at -0.005
```

```
## Warning in simpleLoess(y, x, w, span, degree = degree, parametric = parametric, : radius 2.5e-05
```

```
## Warning in simpleLoess(y, x, w, span, degree = degree, parametric = parametric, : all data on boundary of neighborhood. make span bigger
```

```
## Warning in simpleLoess(y, x, w, span, degree = degree, parametric = parametric, : pseudoinverse used at -0.005
```

```
## Warning in simpleLoess(y, x, w, span, degree = degree, parametric = parametric, : neighborhood radius 0.005
```

```
## Warning in simpleLoess(y, x, w, span, degree = degree, parametric = parametric, : reciprocal condition number 1
```

```
## Warning in simpleLoess(y, x, w, span, degree = degree, parametric = parametric, : There are other near singularities as well. 1.01
```

```
## Warning in simpleLoess(y, x, w, span, degree = degree, parametric = parametric, : zero-width neighborhood. make span bigger
```

```
## Warning: Computation failed in `stat_smooth()`:
## NA/NaN/Inf in foreign function call (arg 5)
```

```
## Warning in simpleLoess(y, x, w, span, degree = degree, parametric = parametric, : at -0.005
```

```
## Warning in simpleLoess(y, x, w, span, degree = degree, parametric = parametric, : radius 2.5e-05
```

```
## Warning in simpleLoess(y, x, w, span, degree = degree, parametric = parametric, : all data on boundary of neighborhood. make span bigger
```

```
## Warning in simpleLoess(y, x, w, span, degree = degree, parametric = parametric, : pseudoinverse used at -0.005
```

```
## Warning in simpleLoess(y, x, w, span, degree = degree, parametric = parametric, : neighborhood radius 0.005
```

```
## Warning in simpleLoess(y, x, w, span, degree = degree, parametric = parametric, : reciprocal condition number 1
```

```
## Warning in simpleLoess(y, x, w, span, degree = degree, parametric = parametric, : There are other near singularities as well. 1.01
```

```
## Warning in simpleLoess(y, x, w, span, degree = degree, parametric = parametric, : zero-width neighborhood. make span bigger
```

```
## Warning: Computation failed in `stat_smooth()`:
## NA/NaN/Inf in foreign function call (arg 5)
```

```
## Warning in simpleLoess(y, x, w, span, degree = degree, parametric = parametric, : at -0.005
```

```
## Warning in simpleLoess(y, x, w, span, degree = degree, parametric = parametric, : radius 2.5e-05
```

```
## Warning in simpleLoess(y, x, w, span, degree = degree, parametric = parametric, : all data on boundary of neighborhood. make span bigger
```

```
## Warning in simpleLoess(y, x, w, span, degree = degree, parametric = parametric, : pseudoinverse used at -0.005
```

```
## Warning in simpleLoess(y, x, w, span, degree = degree, parametric = parametric, : neighborhood radius 0.005
```

```
## Warning in simpleLoess(y, x, w, span, degree = degree, parametric = parametric, : reciprocal condition number 1
```

```
## Warning in simpleLoess(y, x, w, span, degree = degree, parametric = parametric, : There are other near singularities as well. 1.01
```

```
## Warning in simpleLoess(y, x, w, span, degree = degree, parametric = parametric, : zero-width neighborhood. make span bigger
```

```
## Warning: Computation failed in `stat_smooth()`:
## NA/NaN/Inf in foreign function call (arg 5)
```

```
## Warning in simpleLoess(y, x, w, span, degree = degree, parametric = parametric, : at -0.005
```

```
## Warning in simpleLoess(y, x, w, span, degree = degree, parametric = parametric, : radius 2.5e-05
```

```
## Warning in simpleLoess(y, x, w, span, degree = degree, parametric = parametric, : all data on boundary of neighborhood. make span bigger
```

```
## Warning in simpleLoess(y, x, w, span, degree = degree, parametric = parametric, : pseudoinverse used at -0.005
```

```
## Warning in simpleLoess(y, x, w, span, degree = degree, parametric = parametric, : neighborhood radius 0.005
```

```
## Warning in simpleLoess(y, x, w, span, degree = degree, parametric = parametric, : reciprocal condition number 1
```

```
## Warning in simpleLoess(y, x, w, span, degree = degree, parametric = parametric, : There are other near singularities as well. 1.01
```

```
## Warning in simpleLoess(y, x, w, span, degree = degree, parametric = parametric, : zero-width neighborhood. make span bigger
```

```
## Warning: Computation failed in `stat_smooth()`:
## NA/NaN/Inf in foreign function call (arg 5)
```

```
## Warning in simpleLoess(y, x, w, span, degree = degree, parametric = parametric, : at -0.005
```

```
## Warning in simpleLoess(y, x, w, span, degree = degree, parametric = parametric, : radius 2.5e-05
```

```
## Warning in simpleLoess(y, x, w, span, degree = degree, parametric = parametric, : all data on boundary of neighborhood. make span bigger
```

```
## Warning in simpleLoess(y, x, w, span, degree = degree, parametric = parametric, : pseudoinverse used at -0.005
```

```
## Warning in simpleLoess(y, x, w, span, degree = degree, parametric = parametric, : neighborhood radius 0.005
```

```
## Warning in simpleLoess(y, x, w, span, degree = degree, parametric = parametric, : reciprocal condition number 1
```

```
## Warning in simpleLoess(y, x, w, span, degree = degree, parametric = parametric, : There are other near singularities as well. 1.01
```

```
## Warning in simpleLoess(y, x, w, span, degree = degree, parametric = parametric, : zero-width neighborhood. make span bigger
```

```
## Warning: Computation failed in `stat_smooth()`:
## NA/NaN/Inf in foreign function call (arg 5)
```

```
## Warning in simpleLoess(y, x, w, span, degree = degree, parametric = parametric, : at -0.005
```

```
## Warning in simpleLoess(y, x, w, span, degree = degree, parametric = parametric, : radius 2.5e-05
```

```
## Warning in simpleLoess(y, x, w, span, degree = degree, parametric = parametric, : all data on boundary of neighborhood. make span bigger
```

```
## Warning in simpleLoess(y, x, w, span, degree = degree, parametric = parametric, : pseudoinverse used at -0.005
```

```
## Warning in simpleLoess(y, x, w, span, degree = degree, parametric = parametric, : neighborhood radius 0.005
```

```
## Warning in simpleLoess(y, x, w, span, degree = degree, parametric = parametric, : reciprocal condition number 1
```

```
## Warning in simpleLoess(y, x, w, span, degree = degree, parametric = parametric, : There are other near singularities as well. 1.01
```

```
## Warning in simpleLoess(y, x, w, span, degree = degree, parametric = parametric, : zero-width neighborhood. make span bigger
```

```
## Warning: Computation failed in `stat_smooth()`:
## NA/NaN/Inf in foreign function call (arg 5)
```

```
## Warning in simpleLoess(y, x, w, span, degree = degree, parametric = parametric, : at -0.005
```

```
## Warning in simpleLoess(y, x, w, span, degree = degree, parametric = parametric, : radius 2.5e-05
```

```
## Warning in simpleLoess(y, x, w, span, degree = degree, parametric = parametric, : all data on boundary of neighborhood. make span bigger
```

```
## Warning in simpleLoess(y, x, w, span, degree = degree, parametric = parametric, : pseudoinverse used at -0.005
```

```
## Warning in simpleLoess(y, x, w, span, degree = degree, parametric = parametric, : neighborhood radius 0.005
```

```
## Warning in simpleLoess(y, x, w, span, degree = degree, parametric = parametric, : reciprocal condition number 1
```

```
## Warning in simpleLoess(y, x, w, span, degree = degree, parametric = parametric, : There are other near singularities as well. 1.01
```

```
## Warning in simpleLoess(y, x, w, span, degree = degree, parametric = parametric, : zero-width neighborhood. make span bigger
```

```
## Warning: Computation failed in `stat_smooth()`:
## NA/NaN/Inf in foreign function call (arg 5)
```

```
## Warning in simpleLoess(y, x, w, span, degree = degree, parametric = parametric, : at -0.005
```

```
## Warning in simpleLoess(y, x, w, span, degree = degree, parametric = parametric, : radius 2.5e-05
```

```
## Warning in simpleLoess(y, x, w, span, degree = degree, parametric = parametric, : all data on boundary of neighborhood. make span bigger
```

```
## Warning in simpleLoess(y, x, w, span, degree = degree, parametric = parametric, : pseudoinverse used at -0.005
```

```
## Warning in simpleLoess(y, x, w, span, degree = degree, parametric = parametric, : neighborhood radius 0.005
```

```
## Warning in simpleLoess(y, x, w, span, degree = degree, parametric = parametric, : reciprocal condition number 1
```

```
## Warning in simpleLoess(y, x, w, span, degree = degree, parametric = parametric, : There are other near singularities as well. 1.01
```

```
## Warning in simpleLoess(y, x, w, span, degree = degree, parametric = parametric, : zero-width neighborhood. make span bigger
```

```
## Warning: Computation failed in `stat_smooth()`:
## NA/NaN/Inf in foreign function call (arg 5)
```

```
## Warning in simpleLoess(y, x, w, span, degree = degree, parametric = parametric, : at -0.005
```

```
## Warning in simpleLoess(y, x, w, span, degree = degree, parametric = parametric, : radius 2.5e-05
```

```
## Warning in simpleLoess(y, x, w, span, degree = degree, parametric = parametric, : all data on boundary of neighborhood. make span bigger
```

```
## Warning in simpleLoess(y, x, w, span, degree = degree, parametric = parametric, : pseudoinverse used at -0.005
```

```
## Warning in simpleLoess(y, x, w, span, degree = degree, parametric = parametric, : neighborhood radius 0.005
```

```
## Warning in simpleLoess(y, x, w, span, degree = degree, parametric = parametric, : reciprocal condition number 1
```

```
## Warning in simpleLoess(y, x, w, span, degree = degree, parametric = parametric, : There are other near singularities as well. 1.01
```

```
## Warning in simpleLoess(y, x, w, span, degree = degree, parametric = parametric, : zero-width neighborhood. make span bigger
```

```
## Warning: Computation failed in `stat_smooth()`:
## NA/NaN/Inf in foreign function call (arg 5)
```

```
## Warning in simpleLoess(y, x, w, span, degree = degree, parametric = parametric, : at -0.005
```

```
## Warning in simpleLoess(y, x, w, span, degree = degree, parametric = parametric, : radius 2.5e-05
```

```
## Warning in simpleLoess(y, x, w, span, degree = degree, parametric = parametric, : all data on boundary of neighborhood. make span bigger
```

```
## Warning in simpleLoess(y, x, w, span, degree = degree, parametric = parametric, : pseudoinverse used at -0.005
```

```
## Warning in simpleLoess(y, x, w, span, degree = degree, parametric = parametric, : neighborhood radius 0.005
```

```
## Warning in simpleLoess(y, x, w, span, degree = degree, parametric = parametric, : reciprocal condition number 1
```

```
## Warning in simpleLoess(y, x, w, span, degree = degree, parametric = parametric, : There are other near singularities as well. 1.01
```

```
## Warning in simpleLoess(y, x, w, span, degree = degree, parametric = parametric, : zero-width neighborhood. make span bigger
```

```
## Warning: Computation failed in `stat_smooth()`:
## NA/NaN/Inf in foreign function call (arg 5)
```

```
## Warning in simpleLoess(y, x, w, span, degree = degree, parametric = parametric, : at -0.005
```

```
## Warning in simpleLoess(y, x, w, span, degree = degree, parametric = parametric, : radius 2.5e-05
```

```
## Warning in simpleLoess(y, x, w, span, degree = degree, parametric = parametric, : all data on boundary of neighborhood. make span bigger
```

```
## Warning in simpleLoess(y, x, w, span, degree = degree, parametric = parametric, : pseudoinverse used at -0.005
```

```
## Warning in simpleLoess(y, x, w, span, degree = degree, parametric = parametric, : neighborhood radius 0.005
```

```
## Warning in simpleLoess(y, x, w, span, degree = degree, parametric = parametric, : reciprocal condition number 1
```

```
## Warning in simpleLoess(y, x, w, span, degree = degree, parametric = parametric, : There are other near singularities as well. 1.01
```

```
## Warning in simpleLoess(y, x, w, span, degree = degree, parametric = parametric, : zero-width neighborhood. make span bigger
```

```
## Warning: Computation failed in `stat_smooth()`:
## NA/NaN/Inf in foreign function call (arg 5)
```

```
## Warning in simpleLoess(y, x, w, span, degree = degree, parametric = parametric, : at -0.005
```

```
## Warning in simpleLoess(y, x, w, span, degree = degree, parametric = parametric, : radius 2.5e-05
```

```
## Warning in simpleLoess(y, x, w, span, degree = degree, parametric = parametric, : all data on boundary of neighborhood. make span bigger
```

```
## Warning in simpleLoess(y, x, w, span, degree = degree, parametric = parametric, : pseudoinverse used at -0.005
```

```
## Warning in simpleLoess(y, x, w, span, degree = degree, parametric = parametric, : neighborhood radius 0.005
```

```
## Warning in simpleLoess(y, x, w, span, degree = degree, parametric = parametric, : reciprocal condition number 1
```

```
## Warning in simpleLoess(y, x, w, span, degree = degree, parametric = parametric, : There are other near singularities as well. 1.01
```

```
## Warning in simpleLoess(y, x, w, span, degree = degree, parametric = parametric, : zero-width neighborhood. make span bigger
```

```
## Warning: Computation failed in `stat_smooth()`:
## NA/NaN/Inf in foreign function call (arg 5)
```

```
## Warning in simpleLoess(y, x, w, span, degree = degree, parametric = parametric, : at -0.005
```

```
## Warning in simpleLoess(y, x, w, span, degree = degree, parametric = parametric, : radius 2.5e-05
```

```
## Warning in simpleLoess(y, x, w, span, degree = degree, parametric = parametric, : all data on boundary of neighborhood. make span bigger
```

```
## Warning in simpleLoess(y, x, w, span, degree = degree, parametric = parametric, : pseudoinverse used at -0.005
```

```
## Warning in simpleLoess(y, x, w, span, degree = degree, parametric = parametric, : neighborhood radius 0.005
```

```
## Warning in simpleLoess(y, x, w, span, degree = degree, parametric = parametric, : reciprocal condition number 1
```

```
## Warning in simpleLoess(y, x, w, span, degree = degree, parametric = parametric, : There are other near singularities as well. 1.01
```

```
## Warning in simpleLoess(y, x, w, span, degree = degree, parametric = parametric, : zero-width neighborhood. make span bigger
```

```
## Warning: Computation failed in `stat_smooth()`:
## NA/NaN/Inf in foreign function call (arg 5)
```

```
## Warning in simpleLoess(y, x, w, span, degree = degree, parametric = parametric, : at -0.005
```

```
## Warning in simpleLoess(y, x, w, span, degree = degree, parametric = parametric, : radius 2.5e-05
```

```
## Warning in simpleLoess(y, x, w, span, degree = degree, parametric = parametric, : all data on boundary of neighborhood. make span bigger
```

```
## Warning in simpleLoess(y, x, w, span, degree = degree, parametric = parametric, : pseudoinverse used at -0.005
```

```
## Warning in simpleLoess(y, x, w, span, degree = degree, parametric = parametric, : neighborhood radius 0.005
```

```
## Warning in simpleLoess(y, x, w, span, degree = degree, parametric = parametric, : reciprocal condition number 1
```

```
## Warning in simpleLoess(y, x, w, span, degree = degree, parametric = parametric, : There are other near singularities as well. 1.01
```

```
## Warning in simpleLoess(y, x, w, span, degree = degree, parametric = parametric, : zero-width neighborhood. make span bigger
```

```
## Warning: Computation failed in `stat_smooth()`:
## NA/NaN/Inf in foreign function call (arg 5)
```

```
## Warning in simpleLoess(y, x, w, span, degree = degree, parametric = parametric, : at -0.005
```

```
## Warning in simpleLoess(y, x, w, span, degree = degree, parametric = parametric, : radius 2.5e-05
```

```
## Warning in simpleLoess(y, x, w, span, degree = degree, parametric = parametric, : all data on boundary of neighborhood. make span bigger
```

```
## Warning in simpleLoess(y, x, w, span, degree = degree, parametric = parametric, : pseudoinverse used at -0.005
```

```
## Warning in simpleLoess(y, x, w, span, degree = degree, parametric = parametric, : neighborhood radius 0.005
```

```
## Warning in simpleLoess(y, x, w, span, degree = degree, parametric = parametric, : reciprocal condition number 1
```

```
## Warning in simpleLoess(y, x, w, span, degree = degree, parametric = parametric, : There are other near singularities as well. 1.01
```

```
## Warning in simpleLoess(y, x, w, span, degree = degree, parametric = parametric, : zero-width neighborhood. make span bigger
```

```
## Warning: Computation failed in `stat_smooth()`:
## NA/NaN/Inf in foreign function call (arg 5)
```

```
## Warning in simpleLoess(y, x, w, span, degree = degree, parametric = parametric, : at -0.005
```

```
## Warning in simpleLoess(y, x, w, span, degree = degree, parametric = parametric, : radius 2.5e-05
```

```
## Warning in simpleLoess(y, x, w, span, degree = degree, parametric = parametric, : all data on boundary of neighborhood. make span bigger
```

```
## Warning in simpleLoess(y, x, w, span, degree = degree, parametric = parametric, : pseudoinverse used at -0.005
```

```
## Warning in simpleLoess(y, x, w, span, degree = degree, parametric = parametric, : neighborhood radius 0.005
```

```
## Warning in simpleLoess(y, x, w, span, degree = degree, parametric = parametric, : reciprocal condition number 1
```

```
## Warning in simpleLoess(y, x, w, span, degree = degree, parametric = parametric, : There are other near singularities as well. 1.01
```

```
## Warning in simpleLoess(y, x, w, span, degree = degree, parametric = parametric, : zero-width neighborhood. make span bigger
```

```
## Warning: Computation failed in `stat_smooth()`:
## NA/NaN/Inf in foreign function call (arg 5)
```

```
## Warning in simpleLoess(y, x, w, span, degree = degree, parametric = parametric, : at -0.005
```

```
## Warning in simpleLoess(y, x, w, span, degree = degree, parametric = parametric, : radius 2.5e-05
```

```
## Warning in simpleLoess(y, x, w, span, degree = degree, parametric = parametric, : all data on boundary of neighborhood. make span bigger
```

```
## Warning in simpleLoess(y, x, w, span, degree = degree, parametric = parametric, : pseudoinverse used at -0.005
```

```
## Warning in simpleLoess(y, x, w, span, degree = degree, parametric = parametric, : neighborhood radius 0.005
```

```
## Warning in simpleLoess(y, x, w, span, degree = degree, parametric = parametric, : reciprocal condition number 1
```

```
## Warning in simpleLoess(y, x, w, span, degree = degree, parametric = parametric, : There are other near singularities as well. 1.01
```

```
## Warning in simpleLoess(y, x, w, span, degree = degree, parametric = parametric, : zero-width neighborhood. make span bigger
```

```
## Warning: Computation failed in `stat_smooth()`:
## NA/NaN/Inf in foreign function call (arg 5)
```

```
## Warning in simpleLoess(y, x, w, span, degree = degree, parametric = parametric, : at -0.005
```

```
## Warning in simpleLoess(y, x, w, span, degree = degree, parametric = parametric, : radius 2.5e-05
```

```
## Warning in simpleLoess(y, x, w, span, degree = degree, parametric = parametric, : all data on boundary of neighborhood. make span bigger
```

```
## Warning in simpleLoess(y, x, w, span, degree = degree, parametric = parametric, : pseudoinverse used at -0.005
```

```
## Warning in simpleLoess(y, x, w, span, degree = degree, parametric = parametric, : neighborhood radius 0.005
```

```
## Warning in simpleLoess(y, x, w, span, degree = degree, parametric = parametric, : reciprocal condition number 1
```

```
## Warning in simpleLoess(y, x, w, span, degree = degree, parametric = parametric, : There are other near singularities as well. 1.01
```

```
## Warning in simpleLoess(y, x, w, span, degree = degree, parametric = parametric, : zero-width neighborhood. make span bigger
```

```
## Warning: Computation failed in `stat_smooth()`:
## NA/NaN/Inf in foreign function call (arg 5)
```

```
## Warning in simpleLoess(y, x, w, span, degree = degree, parametric = parametric, : at -0.005
```

```
## Warning in simpleLoess(y, x, w, span, degree = degree, parametric = parametric, : radius 2.5e-05
```

```
## Warning in simpleLoess(y, x, w, span, degree = degree, parametric = parametric, : all data on boundary of neighborhood. make span bigger
```

```
## Warning in simpleLoess(y, x, w, span, degree = degree, parametric = parametric, : pseudoinverse used at -0.005
```

```
## Warning in simpleLoess(y, x, w, span, degree = degree, parametric = parametric, : neighborhood radius 0.005
```

```
## Warning in simpleLoess(y, x, w, span, degree = degree, parametric = parametric, : reciprocal condition number 1
```

```
## Warning in simpleLoess(y, x, w, span, degree = degree, parametric = parametric, : There are other near singularities as well. 1.01
```

```
## Warning in simpleLoess(y, x, w, span, degree = degree, parametric = parametric, : zero-width neighborhood. make span bigger
```

```
## Warning: Computation failed in `stat_smooth()`:
## NA/NaN/Inf in foreign function call (arg 5)
```

```
## Warning in simpleLoess(y, x, w, span, degree = degree, parametric = parametric, : at -0.005
```

```
## Warning in simpleLoess(y, x, w, span, degree = degree, parametric = parametric, : radius 2.5e-05
```

```
## Warning in simpleLoess(y, x, w, span, degree = degree, parametric = parametric, : all data on boundary of neighborhood. make span bigger
```

```
## Warning in simpleLoess(y, x, w, span, degree = degree, parametric = parametric, : pseudoinverse used at -0.005
```

```
## Warning in simpleLoess(y, x, w, span, degree = degree, parametric = parametric, : neighborhood radius 0.005
```

```
## Warning in simpleLoess(y, x, w, span, degree = degree, parametric = parametric, : reciprocal condition number 1
```

```
## Warning in simpleLoess(y, x, w, span, degree = degree, parametric = parametric, : There are other near singularities as well. 1.01
```

```
## Warning in simpleLoess(y, x, w, span, degree = degree, parametric = parametric, : zero-width neighborhood. make span bigger
```

```
## Warning: Computation failed in `stat_smooth()`:
## NA/NaN/Inf in foreign function call (arg 5)
```

```
## Warning in simpleLoess(y, x, w, span, degree = degree, parametric = parametric, : at -0.005
```

```
## Warning in simpleLoess(y, x, w, span, degree = degree, parametric = parametric, : radius 2.5e-05
```

```
## Warning in simpleLoess(y, x, w, span, degree = degree, parametric = parametric, : all data on boundary of neighborhood. make span bigger
```

```
## Warning in simpleLoess(y, x, w, span, degree = degree, parametric = parametric, : pseudoinverse used at -0.005
```

```
## Warning in simpleLoess(y, x, w, span, degree = degree, parametric = parametric, : neighborhood radius 0.005
```

```
## Warning in simpleLoess(y, x, w, span, degree = degree, parametric = parametric, : reciprocal condition number 1
```

```
## Warning in simpleLoess(y, x, w, span, degree = degree, parametric = parametric, : There are other near singularities as well. 1.01
```

```
## Warning in simpleLoess(y, x, w, span, degree = degree, parametric = parametric, : zero-width neighborhood. make span bigger
```

```
## Warning: Computation failed in `stat_smooth()`:
## NA/NaN/Inf in foreign function call (arg 5)
```

```
## Warning in simpleLoess(y, x, w, span, degree = degree, parametric = parametric, : at -0.005
```

```
## Warning in simpleLoess(y, x, w, span, degree = degree, parametric = parametric, : radius 2.5e-05
```

```
## Warning in simpleLoess(y, x, w, span, degree = degree, parametric = parametric, : all data on boundary of neighborhood. make span bigger
```

```
## Warning in simpleLoess(y, x, w, span, degree = degree, parametric = parametric, : pseudoinverse used at -0.005
```

```
## Warning in simpleLoess(y, x, w, span, degree = degree, parametric = parametric, : neighborhood radius 0.005
```

```
## Warning in simpleLoess(y, x, w, span, degree = degree, parametric = parametric, : reciprocal condition number 1
```

```
## Warning in simpleLoess(y, x, w, span, degree = degree, parametric = parametric, : There are other near singularities as well. 1.01
```

```
## Warning in simpleLoess(y, x, w, span, degree = degree, parametric = parametric, : zero-width neighborhood. make span bigger
```

```
## Warning: Computation failed in `stat_smooth()`:
## NA/NaN/Inf in foreign function call (arg 5)
```

```
## Warning in simpleLoess(y, x, w, span, degree = degree, parametric = parametric, : at -0.005
```

```
## Warning in simpleLoess(y, x, w, span, degree = degree, parametric = parametric, : radius 2.5e-05
```

```
## Warning in simpleLoess(y, x, w, span, degree = degree, parametric = parametric, : all data on boundary of neighborhood. make span bigger
```

```
## Warning in simpleLoess(y, x, w, span, degree = degree, parametric = parametric, : pseudoinverse used at -0.005
```

```
## Warning in simpleLoess(y, x, w, span, degree = degree, parametric = parametric, : neighborhood radius 0.005
```

```
## Warning in simpleLoess(y, x, w, span, degree = degree, parametric = parametric, : reciprocal condition number 1
```

```
## Warning in simpleLoess(y, x, w, span, degree = degree, parametric = parametric, : There are other near singularities as well. 1.01
```

```
## Warning in simpleLoess(y, x, w, span, degree = degree, parametric = parametric, : zero-width neighborhood. make span bigger
```

```
## Warning: Computation failed in `stat_smooth()`:
## NA/NaN/Inf in foreign function call (arg 5)
```

```
## Warning in simpleLoess(y, x, w, span, degree = degree, parametric = parametric, : at -0.005
```

```
## Warning in simpleLoess(y, x, w, span, degree = degree, parametric = parametric, : radius 2.5e-05
```

```
## Warning in simpleLoess(y, x, w, span, degree = degree, parametric = parametric, : all data on boundary of neighborhood. make span bigger
```

```
## Warning in simpleLoess(y, x, w, span, degree = degree, parametric = parametric, : pseudoinverse used at -0.005
```

```
## Warning in simpleLoess(y, x, w, span, degree = degree, parametric = parametric, : neighborhood radius 0.005
```

```
## Warning in simpleLoess(y, x, w, span, degree = degree, parametric = parametric, : reciprocal condition number 1
```

```
## Warning in simpleLoess(y, x, w, span, degree = degree, parametric = parametric, : There are other near singularities as well. 1.01
```

```
## Warning in simpleLoess(y, x, w, span, degree = degree, parametric = parametric, : zero-width neighborhood. make span bigger
```

```
## Warning: Computation failed in `stat_smooth()`:
## NA/NaN/Inf in foreign function call (arg 5)
```

```
## Warning in simpleLoess(y, x, w, span, degree = degree, parametric = parametric, : at -0.005
```

```
## Warning in simpleLoess(y, x, w, span, degree = degree, parametric = parametric, : radius 2.5e-05
```

```
## Warning in simpleLoess(y, x, w, span, degree = degree, parametric = parametric, : all data on boundary of neighborhood. make span bigger
```

```
## Warning in simpleLoess(y, x, w, span, degree = degree, parametric = parametric, : pseudoinverse used at -0.005
```

```
## Warning in simpleLoess(y, x, w, span, degree = degree, parametric = parametric, : neighborhood radius 0.005
```

```
## Warning in simpleLoess(y, x, w, span, degree = degree, parametric = parametric, : reciprocal condition number 1
```

```
## Warning in simpleLoess(y, x, w, span, degree = degree, parametric = parametric, : There are other near singularities as well. 1.01
```

```
## Warning in simpleLoess(y, x, w, span, degree = degree, parametric = parametric, : zero-width neighborhood. make span bigger
```

```
## Warning: Computation failed in `stat_smooth()`:
## NA/NaN/Inf in foreign function call (arg 5)
```

```
## Warning in simpleLoess(y, x, w, span, degree = degree, parametric = parametric, : at -0.005
```

```
## Warning in simpleLoess(y, x, w, span, degree = degree, parametric = parametric, : radius 2.5e-05
```

```
## Warning in simpleLoess(y, x, w, span, degree = degree, parametric = parametric, : all data on boundary of neighborhood. make span bigger
```

```
## Warning in simpleLoess(y, x, w, span, degree = degree, parametric = parametric, : pseudoinverse used at -0.005
```

```
## Warning in simpleLoess(y, x, w, span, degree = degree, parametric = parametric, : neighborhood radius 0.005
```

```
## Warning in simpleLoess(y, x, w, span, degree = degree, parametric = parametric, : reciprocal condition number 1
```

```
## Warning in simpleLoess(y, x, w, span, degree = degree, parametric = parametric, : There are other near singularities as well. 1.01
```

```
## Warning in simpleLoess(y, x, w, span, degree = degree, parametric = parametric, : zero-width neighborhood. make span bigger
```

```
## Warning: Computation failed in `stat_smooth()`:
## NA/NaN/Inf in foreign function call (arg 5)
```

```
## Warning in simpleLoess(y, x, w, span, degree = degree, parametric = parametric, : at -0.005
```

```
## Warning in simpleLoess(y, x, w, span, degree = degree, parametric = parametric, : radius 2.5e-05
```

```
## Warning in simpleLoess(y, x, w, span, degree = degree, parametric = parametric, : all data on boundary of neighborhood. make span bigger
```

```
## Warning in simpleLoess(y, x, w, span, degree = degree, parametric = parametric, : pseudoinverse used at -0.005
```

```
## Warning in simpleLoess(y, x, w, span, degree = degree, parametric = parametric, : neighborhood radius 0.005
```

```
## Warning in simpleLoess(y, x, w, span, degree = degree, parametric = parametric, : reciprocal condition number 1
```

```
## Warning in simpleLoess(y, x, w, span, degree = degree, parametric = parametric, : There are other near singularities as well. 1.01
```

```
## Warning in simpleLoess(y, x, w, span, degree = degree, parametric = parametric, : zero-width neighborhood. make span bigger
```

```
## Warning: Computation failed in `stat_smooth()`:
## NA/NaN/Inf in foreign function call (arg 5)
```

```
## Warning in simpleLoess(y, x, w, span, degree = degree, parametric = parametric, : at -0.005
```

```
## Warning in simpleLoess(y, x, w, span, degree = degree, parametric = parametric, : radius 2.5e-05
```

```
## Warning in simpleLoess(y, x, w, span, degree = degree, parametric = parametric, : all data on boundary of neighborhood. make span bigger
```

```
## Warning in simpleLoess(y, x, w, span, degree = degree, parametric = parametric, : pseudoinverse used at -0.005
```

```
## Warning in simpleLoess(y, x, w, span, degree = degree, parametric = parametric, : neighborhood radius 0.005
```

```
## Warning in simpleLoess(y, x, w, span, degree = degree, parametric = parametric, : reciprocal condition number 1
```

```
## Warning in simpleLoess(y, x, w, span, degree = degree, parametric = parametric, : There are other near singularities as well. 1.01
```

```
## Warning in simpleLoess(y, x, w, span, degree = degree, parametric = parametric, : zero-width neighborhood. make span bigger
```

```
## Warning: Computation failed in `stat_smooth()`:
## NA/NaN/Inf in foreign function call (arg 5)
```

```
## Warning in simpleLoess(y, x, w, span, degree = degree, parametric = parametric, : at -0.005
```

```
## Warning in simpleLoess(y, x, w, span, degree = degree, parametric = parametric, : radius 2.5e-05
```

```
## Warning in simpleLoess(y, x, w, span, degree = degree, parametric = parametric, : all data on boundary of neighborhood. make span bigger
```

```
## Warning in simpleLoess(y, x, w, span, degree = degree, parametric = parametric, : pseudoinverse used at -0.005
```

```
## Warning in simpleLoess(y, x, w, span, degree = degree, parametric = parametric, : neighborhood radius 0.005
```

```
## Warning in simpleLoess(y, x, w, span, degree = degree, parametric = parametric, : reciprocal condition number 1
```

```
## Warning in simpleLoess(y, x, w, span, degree = degree, parametric = parametric, : There are other near singularities as well. 1.01
```

```
## Warning in simpleLoess(y, x, w, span, degree = degree, parametric = parametric, : zero-width neighborhood. make span bigger
```

```
## Warning: Computation failed in `stat_smooth()`:
## NA/NaN/Inf in foreign function call (arg 5)
```

```
## Warning in simpleLoess(y, x, w, span, degree = degree, parametric = parametric, : at -0.005
```

```
## Warning in simpleLoess(y, x, w, span, degree = degree, parametric = parametric, : radius 2.5e-05
```

```
## Warning in simpleLoess(y, x, w, span, degree = degree, parametric = parametric, : all data on boundary of neighborhood. make span bigger
```

```
## Warning in simpleLoess(y, x, w, span, degree = degree, parametric = parametric, : pseudoinverse used at -0.005
```

```
## Warning in simpleLoess(y, x, w, span, degree = degree, parametric = parametric, : neighborhood radius 0.005
```

```
## Warning in simpleLoess(y, x, w, span, degree = degree, parametric = parametric, : reciprocal condition number 1
```

```
## Warning in simpleLoess(y, x, w, span, degree = degree, parametric = parametric, : There are other near singularities as well. 1.01
```

```
## Warning in simpleLoess(y, x, w, span, degree = degree, parametric = parametric, : zero-width neighborhood. make span bigger
```

```
## Warning: Computation failed in `stat_smooth()`:
## NA/NaN/Inf in foreign function call (arg 5)
```

```
## Warning in simpleLoess(y, x, w, span, degree = degree, parametric = parametric, : at -0.005
```

```
## Warning in simpleLoess(y, x, w, span, degree = degree, parametric = parametric, : radius 2.5e-05
```

```
## Warning in simpleLoess(y, x, w, span, degree = degree, parametric = parametric, : all data on boundary of neighborhood. make span bigger
```

```
## Warning in simpleLoess(y, x, w, span, degree = degree, parametric = parametric, : pseudoinverse used at -0.005
```

```
## Warning in simpleLoess(y, x, w, span, degree = degree, parametric = parametric, : neighborhood radius 0.005
```

```
## Warning in simpleLoess(y, x, w, span, degree = degree, parametric = parametric, : reciprocal condition number 1
```

```
## Warning in simpleLoess(y, x, w, span, degree = degree, parametric = parametric, : There are other near singularities as well. 1.01
```

```
## Warning in simpleLoess(y, x, w, span, degree = degree, parametric = parametric, : zero-width neighborhood. make span bigger
```

```
## Warning: Computation failed in `stat_smooth()`:
## NA/NaN/Inf in foreign function call (arg 5)
```

```
## Warning in simpleLoess(y, x, w, span, degree = degree, parametric = parametric, : at -0.005
```

```
## Warning in simpleLoess(y, x, w, span, degree = degree, parametric = parametric, : radius 2.5e-05
```

```
## Warning in simpleLoess(y, x, w, span, degree = degree, parametric = parametric, : all data on boundary of neighborhood. make span bigger
```

```
## Warning in simpleLoess(y, x, w, span, degree = degree, parametric = parametric, : pseudoinverse used at -0.005
```

```
## Warning in simpleLoess(y, x, w, span, degree = degree, parametric = parametric, : neighborhood radius 0.005
```

```
## Warning in simpleLoess(y, x, w, span, degree = degree, parametric = parametric, : reciprocal condition number 1
```

```
## Warning in simpleLoess(y, x, w, span, degree = degree, parametric = parametric, : There are other near singularities as well. 1.01
```

```
## Warning in simpleLoess(y, x, w, span, degree = degree, parametric = parametric, : zero-width neighborhood. make span bigger
```

```
## Warning: Computation failed in `stat_smooth()`:
## NA/NaN/Inf in foreign function call (arg 5)
```

```
## Warning in simpleLoess(y, x, w, span, degree = degree, parametric = parametric, : at -0.005
```

```
## Warning in simpleLoess(y, x, w, span, degree = degree, parametric = parametric, : radius 2.5e-05
```

```
## Warning in simpleLoess(y, x, w, span, degree = degree, parametric = parametric, : all data on boundary of neighborhood. make span bigger
```

```
## Warning in simpleLoess(y, x, w, span, degree = degree, parametric = parametric, : pseudoinverse used at -0.005
```

```
## Warning in simpleLoess(y, x, w, span, degree = degree, parametric = parametric, : neighborhood radius 0.005
```

```
## Warning in simpleLoess(y, x, w, span, degree = degree, parametric = parametric, : reciprocal condition number 1
```

```
## Warning in simpleLoess(y, x, w, span, degree = degree, parametric = parametric, : There are other near singularities as well. 1.01
```

```
## Warning in simpleLoess(y, x, w, span, degree = degree, parametric = parametric, : zero-width neighborhood. make span bigger
```

```
## Warning: Computation failed in `stat_smooth()`:
## NA/NaN/Inf in foreign function call (arg 5)
```

```
## Warning in simpleLoess(y, x, w, span, degree = degree, parametric = parametric, : at -0.005
```

```
## Warning in simpleLoess(y, x, w, span, degree = degree, parametric = parametric, : radius 2.5e-05
```

```
## Warning in simpleLoess(y, x, w, span, degree = degree, parametric = parametric, : all data on boundary of neighborhood. make span bigger
```

```
## Warning in simpleLoess(y, x, w, span, degree = degree, parametric = parametric, : pseudoinverse used at -0.005
```

```
## Warning in simpleLoess(y, x, w, span, degree = degree, parametric = parametric, : neighborhood radius 0.005
```

```
## Warning in simpleLoess(y, x, w, span, degree = degree, parametric = parametric, : reciprocal condition number 1
```

```
## Warning in simpleLoess(y, x, w, span, degree = degree, parametric = parametric, : There are other near singularities as well. 1.01
```

```
## Warning in simpleLoess(y, x, w, span, degree = degree, parametric = parametric, : zero-width neighborhood. make span bigger
```

```
## Warning: Computation failed in `stat_smooth()`:
## NA/NaN/Inf in foreign function call (arg 5)
```

```
## Warning in simpleLoess(y, x, w, span, degree = degree, parametric = parametric, : at -0.005
```

```
## Warning in simpleLoess(y, x, w, span, degree = degree, parametric = parametric, : radius 2.5e-05
```

```
## Warning in simpleLoess(y, x, w, span, degree = degree, parametric = parametric, : all data on boundary of neighborhood. make span bigger
```

```
## Warning in simpleLoess(y, x, w, span, degree = degree, parametric = parametric, : pseudoinverse used at -0.005
```

```
## Warning in simpleLoess(y, x, w, span, degree = degree, parametric = parametric, : neighborhood radius 0.005
```

```
## Warning in simpleLoess(y, x, w, span, degree = degree, parametric = parametric, : reciprocal condition number 1
```

```
## Warning in simpleLoess(y, x, w, span, degree = degree, parametric = parametric, : There are other near singularities as well. 1.01
```

```
## Warning in simpleLoess(y, x, w, span, degree = degree, parametric = parametric, : zero-width neighborhood. make span bigger
```

```
## Warning: Computation failed in `stat_smooth()`:
## NA/NaN/Inf in foreign function call (arg 5)
```

```
## Warning in simpleLoess(y, x, w, span, degree = degree, parametric = parametric, : at -0.005
```

```
## Warning in simpleLoess(y, x, w, span, degree = degree, parametric = parametric, : radius 2.5e-05
```

```
## Warning in simpleLoess(y, x, w, span, degree = degree, parametric = parametric, : all data on boundary of neighborhood. make span bigger
```

```
## Warning in simpleLoess(y, x, w, span, degree = degree, parametric = parametric, : pseudoinverse used at -0.005
```

```
## Warning in simpleLoess(y, x, w, span, degree = degree, parametric = parametric, : neighborhood radius 0.005
```

```
## Warning in simpleLoess(y, x, w, span, degree = degree, parametric = parametric, : reciprocal condition number 1
```

```
## Warning in simpleLoess(y, x, w, span, degree = degree, parametric = parametric, : There are other near singularities as well. 1.01
```

```
## Warning in simpleLoess(y, x, w, span, degree = degree, parametric = parametric, : zero-width neighborhood. make span bigger
```

```
## Warning: Computation failed in `stat_smooth()`:
## NA/NaN/Inf in foreign function call (arg 5)
```

```
## Warning in simpleLoess(y, x, w, span, degree = degree, parametric = parametric, : at -0.005
```

```
## Warning in simpleLoess(y, x, w, span, degree = degree, parametric = parametric, : radius 2.5e-05
```

```
## Warning in simpleLoess(y, x, w, span, degree = degree, parametric = parametric, : all data on boundary of neighborhood. make span bigger
```

```
## Warning in simpleLoess(y, x, w, span, degree = degree, parametric = parametric, : pseudoinverse used at -0.005
```

```
## Warning in simpleLoess(y, x, w, span, degree = degree, parametric = parametric, : neighborhood radius 0.005
```

```
## Warning in simpleLoess(y, x, w, span, degree = degree, parametric = parametric, : reciprocal condition number 1
```

```
## Warning in simpleLoess(y, x, w, span, degree = degree, parametric = parametric, : There are other near singularities as well. 1.01
```

```
## Warning in simpleLoess(y, x, w, span, degree = degree, parametric = parametric, : zero-width neighborhood. make span bigger
```

```
## Warning: Computation failed in `stat_smooth()`:
## NA/NaN/Inf in foreign function call (arg 5)
```

```
## Warning in simpleLoess(y, x, w, span, degree = degree, parametric = parametric, : at -0.005
```

```
## Warning in simpleLoess(y, x, w, span, degree = degree, parametric = parametric, : radius 2.5e-05
```

```
## Warning in simpleLoess(y, x, w, span, degree = degree, parametric = parametric, : all data on boundary of neighborhood. make span bigger
```

```
## Warning in simpleLoess(y, x, w, span, degree = degree, parametric = parametric, : pseudoinverse used at -0.005
```

```
## Warning in simpleLoess(y, x, w, span, degree = degree, parametric = parametric, : neighborhood radius 0.005
```

```
## Warning in simpleLoess(y, x, w, span, degree = degree, parametric = parametric, : reciprocal condition number 1
```

```
## Warning in simpleLoess(y, x, w, span, degree = degree, parametric = parametric, : There are other near singularities as well. 1.01
```

```
## Warning in simpleLoess(y, x, w, span, degree = degree, parametric = parametric, : zero-width neighborhood. make span bigger
```

```
## Warning: Computation failed in `stat_smooth()`:
## NA/NaN/Inf in foreign function call (arg 5)
```

```
## Warning in simpleLoess(y, x, w, span, degree = degree, parametric = parametric, : at -0.005
```

```
## Warning in simpleLoess(y, x, w, span, degree = degree, parametric = parametric, : radius 2.5e-05
```

```
## Warning in simpleLoess(y, x, w, span, degree = degree, parametric = parametric, : all data on boundary of neighborhood. make span bigger
```

```
## Warning in simpleLoess(y, x, w, span, degree = degree, parametric = parametric, : pseudoinverse used at -0.005
```

```
## Warning in simpleLoess(y, x, w, span, degree = degree, parametric = parametric, : neighborhood radius 0.005
```

```
## Warning in simpleLoess(y, x, w, span, degree = degree, parametric = parametric, : reciprocal condition number 1
```

```
## Warning in simpleLoess(y, x, w, span, degree = degree, parametric = parametric, : There are other near singularities as well. 1.01
```

```
## Warning in simpleLoess(y, x, w, span, degree = degree, parametric = parametric, : zero-width neighborhood. make span bigger
```

```
## Warning: Computation failed in `stat_smooth()`:
## NA/NaN/Inf in foreign function call (arg 5)
```

```
## Warning in simpleLoess(y, x, w, span, degree = degree, parametric = parametric, : pseudoinverse used at -0.005
```

```
## Warning in simpleLoess(y, x, w, span, degree = degree, parametric = parametric, : neighborhood radius 1.005
```

```
## Warning in simpleLoess(y, x, w, span, degree = degree, parametric = parametric, : reciprocal condition number 0
```

```
## Warning in simpleLoess(y, x, w, span, degree = degree, parametric = parametric, : There are other near singularities as well. 1.01
```

```
## Warning in predLoess(object$y, object$x, newx = if (is.null(newdata)) object$x else if (is.data.frame(newdata)) as.matrix(model.frame(delete.response(terms(object)), : pseudoinverse used at -0.005
```

```
## Warning in predLoess(object$y, object$x, newx = if (is.null(newdata)) object$x else if (is.data.frame(newdata)) as.matrix(model.frame(delete.response(terms(object)), : neighborhood radius 1.005
```

```
## Warning in predLoess(object$y, object$x, newx = if (is.null(newdata)) object$x else if (is.data.frame(newdata)) as.matrix(model.frame(delete.response(terms(object)), : reciprocal condition number 0
```

```
## Warning in predLoess(object$y, object$x, newx = if (is.null(newdata)) object$x else if (is.data.frame(newdata)) as.matrix(model.frame(delete.response(terms(object)), : There are other near
## singularities as well. 1.01
```

```
## Warning in simpleLoess(y, x, w, span, degree = degree, parametric = parametric, : at -0.005
```

```
## Warning in simpleLoess(y, x, w, span, degree = degree, parametric = parametric, : radius 2.5e-05
```

```
## Warning in simpleLoess(y, x, w, span, degree = degree, parametric = parametric, : all data on boundary of neighborhood. make span bigger
```

```
## Warning in simpleLoess(y, x, w, span, degree = degree, parametric = parametric, : pseudoinverse used at -0.005
```

```
## Warning in simpleLoess(y, x, w, span, degree = degree, parametric = parametric, : neighborhood radius 0.005
```

```
## Warning in simpleLoess(y, x, w, span, degree = degree, parametric = parametric, : reciprocal condition number 1
```

```
## Warning in simpleLoess(y, x, w, span, degree = degree, parametric = parametric, : There are other near singularities as well. 1.01
```

```
## Warning in simpleLoess(y, x, w, span, degree = degree, parametric = parametric, : zero-width neighborhood. make span bigger
```

```
## Warning: Computation failed in `stat_smooth()`:
## NA/NaN/Inf in foreign function call (arg 5)
```

```
## Warning in simpleLoess(y, x, w, span, degree = degree, parametric = parametric, : at -0.005
```

```
## Warning in simpleLoess(y, x, w, span, degree = degree, parametric = parametric, : radius 2.5e-05
```

```
## Warning in simpleLoess(y, x, w, span, degree = degree, parametric = parametric, : all data on boundary of neighborhood. make span bigger
```

```
## Warning in simpleLoess(y, x, w, span, degree = degree, parametric = parametric, : pseudoinverse used at -0.005
```

```
## Warning in simpleLoess(y, x, w, span, degree = degree, parametric = parametric, : neighborhood radius 0.005
```

```
## Warning in simpleLoess(y, x, w, span, degree = degree, parametric = parametric, : reciprocal condition number 1
```

```
## Warning in simpleLoess(y, x, w, span, degree = degree, parametric = parametric, : There are other near singularities as well. 1.01
```

```
## Warning in simpleLoess(y, x, w, span, degree = degree, parametric = parametric, : zero-width neighborhood. make span bigger
```

```
## Warning: Computation failed in `stat_smooth()`:
## NA/NaN/Inf in foreign function call (arg 5)
```

```
## Warning in simpleLoess(y, x, w, span, degree = degree, parametric = parametric, : at -0.005
```

```
## Warning in simpleLoess(y, x, w, span, degree = degree, parametric = parametric, : radius 2.5e-05
```

```
## Warning in simpleLoess(y, x, w, span, degree = degree, parametric = parametric, : all data on boundary of neighborhood. make span bigger
```

```
## Warning in simpleLoess(y, x, w, span, degree = degree, parametric = parametric, : pseudoinverse used at -0.005
```

```
## Warning in simpleLoess(y, x, w, span, degree = degree, parametric = parametric, : neighborhood radius 0.005
```

```
## Warning in simpleLoess(y, x, w, span, degree = degree, parametric = parametric, : reciprocal condition number 1
```

```
## Warning in simpleLoess(y, x, w, span, degree = degree, parametric = parametric, : There are other near singularities as well. 1.01
```

```
## Warning in simpleLoess(y, x, w, span, degree = degree, parametric = parametric, : zero-width neighborhood. make span bigger
```

```
## Warning: Computation failed in `stat_smooth()`:
## NA/NaN/Inf in foreign function call (arg 5)
```

```
## Warning in simpleLoess(y, x, w, span, degree = degree, parametric = parametric, : at -0.005
```

```
## Warning in simpleLoess(y, x, w, span, degree = degree, parametric = parametric, : radius 2.5e-05
```

```
## Warning in simpleLoess(y, x, w, span, degree = degree, parametric = parametric, : all data on boundary of neighborhood. make span bigger
```

```
## Warning in simpleLoess(y, x, w, span, degree = degree, parametric = parametric, : pseudoinverse used at -0.005
```

```
## Warning in simpleLoess(y, x, w, span, degree = degree, parametric = parametric, : neighborhood radius 0.005
```

```
## Warning in simpleLoess(y, x, w, span, degree = degree, parametric = parametric, : reciprocal condition number 1
```

```
## Warning in simpleLoess(y, x, w, span, degree = degree, parametric = parametric, : There are other near singularities as well. 1.01
```

```
## Warning in simpleLoess(y, x, w, span, degree = degree, parametric = parametric, : zero-width neighborhood. make span bigger
```

```
## Warning: Computation failed in `stat_smooth()`:
## NA/NaN/Inf in foreign function call (arg 5)
```

```
## Warning in simpleLoess(y, x, w, span, degree = degree, parametric = parametric, : at -0.005
```

```
## Warning in simpleLoess(y, x, w, span, degree = degree, parametric = parametric, : radius 2.5e-05
```

```
## Warning in simpleLoess(y, x, w, span, degree = degree, parametric = parametric, : all data on boundary of neighborhood. make span bigger
```

```
## Warning in simpleLoess(y, x, w, span, degree = degree, parametric = parametric, : pseudoinverse used at -0.005
```

```
## Warning in simpleLoess(y, x, w, span, degree = degree, parametric = parametric, : neighborhood radius 0.005
```

```
## Warning in simpleLoess(y, x, w, span, degree = degree, parametric = parametric, : reciprocal condition number 1
```

```
## Warning in simpleLoess(y, x, w, span, degree = degree, parametric = parametric, : There are other near singularities as well. 1.01
```

```
## Warning in simpleLoess(y, x, w, span, degree = degree, parametric = parametric, : zero-width neighborhood. make span bigger
```

```
## Warning: Computation failed in `stat_smooth()`:
## NA/NaN/Inf in foreign function call (arg 5)
```

```
## Warning in simpleLoess(y, x, w, span, degree = degree, parametric = parametric, : at -0.005
```

```
## Warning in simpleLoess(y, x, w, span, degree = degree, parametric = parametric, : radius 2.5e-05
```

```
## Warning in simpleLoess(y, x, w, span, degree = degree, parametric = parametric, : all data on boundary of neighborhood. make span bigger
```

```
## Warning in simpleLoess(y, x, w, span, degree = degree, parametric = parametric, : pseudoinverse used at -0.005
```

```
## Warning in simpleLoess(y, x, w, span, degree = degree, parametric = parametric, : neighborhood radius 0.005
```

```
## Warning in simpleLoess(y, x, w, span, degree = degree, parametric = parametric, : reciprocal condition number 1
```

```
## Warning in simpleLoess(y, x, w, span, degree = degree, parametric = parametric, : There are other near singularities as well. 1.01
```

```
## Warning in simpleLoess(y, x, w, span, degree = degree, parametric = parametric, : zero-width neighborhood. make span bigger
```

```
## Warning: Computation failed in `stat_smooth()`:
## NA/NaN/Inf in foreign function call (arg 5)
```

```
## Warning in simpleLoess(y, x, w, span, degree = degree, parametric = parametric, : at -0.005
```

```
## Warning in simpleLoess(y, x, w, span, degree = degree, parametric = parametric, : radius 2.5e-05
```

```
## Warning in simpleLoess(y, x, w, span, degree = degree, parametric = parametric, : all data on boundary of neighborhood. make span bigger
```

```
## Warning in simpleLoess(y, x, w, span, degree = degree, parametric = parametric, : pseudoinverse used at -0.005
```

```
## Warning in simpleLoess(y, x, w, span, degree = degree, parametric = parametric, : neighborhood radius 0.005
```

```
## Warning in simpleLoess(y, x, w, span, degree = degree, parametric = parametric, : reciprocal condition number 1
```

```
## Warning in simpleLoess(y, x, w, span, degree = degree, parametric = parametric, : There are other near singularities as well. 1.01
```

```
## Warning in simpleLoess(y, x, w, span, degree = degree, parametric = parametric, : zero-width neighborhood. make span bigger
```

```
## Warning: Computation failed in `stat_smooth()`:
## NA/NaN/Inf in foreign function call (arg 5)
```

```
## Warning in simpleLoess(y, x, w, span, degree = degree, parametric = parametric, : at -0.005
```

```
## Warning in simpleLoess(y, x, w, span, degree = degree, parametric = parametric, : radius 2.5e-05
```

```
## Warning in simpleLoess(y, x, w, span, degree = degree, parametric = parametric, : all data on boundary of neighborhood. make span bigger
```

```
## Warning in simpleLoess(y, x, w, span, degree = degree, parametric = parametric, : pseudoinverse used at -0.005
```

```
## Warning in simpleLoess(y, x, w, span, degree = degree, parametric = parametric, : neighborhood radius 0.005
```

```
## Warning in simpleLoess(y, x, w, span, degree = degree, parametric = parametric, : reciprocal condition number 1
```

```
## Warning in simpleLoess(y, x, w, span, degree = degree, parametric = parametric, : There are other near singularities as well. 1.01
```

```
## Warning in simpleLoess(y, x, w, span, degree = degree, parametric = parametric, : zero-width neighborhood. make span bigger
```

```
## Warning: Computation failed in `stat_smooth()`:
## NA/NaN/Inf in foreign function call (arg 5)
```

```
## Warning in simpleLoess(y, x, w, span, degree = degree, parametric = parametric, : at -0.005
```

```
## Warning in simpleLoess(y, x, w, span, degree = degree, parametric = parametric, : radius 2.5e-05
```

```
## Warning in simpleLoess(y, x, w, span, degree = degree, parametric = parametric, : all data on boundary of neighborhood. make span bigger
```

```
## Warning in simpleLoess(y, x, w, span, degree = degree, parametric = parametric, : pseudoinverse used at -0.005
```

```
## Warning in simpleLoess(y, x, w, span, degree = degree, parametric = parametric, : neighborhood radius 0.005
```

```
## Warning in simpleLoess(y, x, w, span, degree = degree, parametric = parametric, : reciprocal condition number 1
```

```
## Warning in simpleLoess(y, x, w, span, degree = degree, parametric = parametric, : There are other near singularities as well. 1.01
```

```
## Warning in simpleLoess(y, x, w, span, degree = degree, parametric = parametric, : zero-width neighborhood. make span bigger
```

```
## Warning: Computation failed in `stat_smooth()`:
## NA/NaN/Inf in foreign function call (arg 5)
```

```
## Warning in simpleLoess(y, x, w, span, degree = degree, parametric = parametric, : at -0.005
```

```
## Warning in simpleLoess(y, x, w, span, degree = degree, parametric = parametric, : radius 2.5e-05
```

```
## Warning in simpleLoess(y, x, w, span, degree = degree, parametric = parametric, : all data on boundary of neighborhood. make span bigger
```

```
## Warning in simpleLoess(y, x, w, span, degree = degree, parametric = parametric, : pseudoinverse used at -0.005
```

```
## Warning in simpleLoess(y, x, w, span, degree = degree, parametric = parametric, : neighborhood radius 0.005
```

```
## Warning in simpleLoess(y, x, w, span, degree = degree, parametric = parametric, : reciprocal condition number 1
```

```
## Warning in simpleLoess(y, x, w, span, degree = degree, parametric = parametric, : There are other near singularities as well. 1.01
```

```
## Warning in simpleLoess(y, x, w, span, degree = degree, parametric = parametric, : zero-width neighborhood. make span bigger
```

```
## Warning: Computation failed in `stat_smooth()`:
## NA/NaN/Inf in foreign function call (arg 5)
```

```
## Warning in simpleLoess(y, x, w, span, degree = degree, parametric = parametric, : at -0.005
```

```
## Warning in simpleLoess(y, x, w, span, degree = degree, parametric = parametric, : radius 2.5e-05
```

```
## Warning in simpleLoess(y, x, w, span, degree = degree, parametric = parametric, : all data on boundary of neighborhood. make span bigger
```

```
## Warning in simpleLoess(y, x, w, span, degree = degree, parametric = parametric, : pseudoinverse used at -0.005
```

```
## Warning in simpleLoess(y, x, w, span, degree = degree, parametric = parametric, : neighborhood radius 0.005
```

```
## Warning in simpleLoess(y, x, w, span, degree = degree, parametric = parametric, : reciprocal condition number 1
```

```
## Warning in simpleLoess(y, x, w, span, degree = degree, parametric = parametric, : There are other near singularities as well. 1.01
```

```
## Warning in simpleLoess(y, x, w, span, degree = degree, parametric = parametric, : zero-width neighborhood. make span bigger
```

```
## Warning: Computation failed in `stat_smooth()`:
## NA/NaN/Inf in foreign function call (arg 5)
```

```
## Warning in simpleLoess(y, x, w, span, degree = degree, parametric = parametric, : pseudoinverse used at -0.005
```

```
## Warning in simpleLoess(y, x, w, span, degree = degree, parametric = parametric, : neighborhood radius 1.005
```

```
## Warning in simpleLoess(y, x, w, span, degree = degree, parametric = parametric, : reciprocal condition number 0
```

```
## Warning in simpleLoess(y, x, w, span, degree = degree, parametric = parametric, : There are other near singularities as well. 1.01
```

```
## Warning in predLoess(object$y, object$x, newx = if (is.null(newdata)) object$x else if (is.data.frame(newdata)) as.matrix(model.frame(delete.response(terms(object)), : pseudoinverse used at -0.005
```

```
## Warning in predLoess(object$y, object$x, newx = if (is.null(newdata)) object$x else if (is.data.frame(newdata)) as.matrix(model.frame(delete.response(terms(object)), : neighborhood radius 1.005
```

```
## Warning in predLoess(object$y, object$x, newx = if (is.null(newdata)) object$x else if (is.data.frame(newdata)) as.matrix(model.frame(delete.response(terms(object)), : reciprocal condition number 0
```

```
## Warning in predLoess(object$y, object$x, newx = if (is.null(newdata)) object$x else if (is.data.frame(newdata)) as.matrix(model.frame(delete.response(terms(object)), : There are other near
## singularities as well. 1.01
```

```
## Warning in simpleLoess(y, x, w, span, degree = degree, parametric = parametric, : at -0.005
```

```
## Warning in simpleLoess(y, x, w, span, degree = degree, parametric = parametric, : radius 2.5e-05
```

```
## Warning in simpleLoess(y, x, w, span, degree = degree, parametric = parametric, : all data on boundary of neighborhood. make span bigger
```

```
## Warning in simpleLoess(y, x, w, span, degree = degree, parametric = parametric, : pseudoinverse used at -0.005
```

```
## Warning in simpleLoess(y, x, w, span, degree = degree, parametric = parametric, : neighborhood radius 0.005
```

```
## Warning in simpleLoess(y, x, w, span, degree = degree, parametric = parametric, : reciprocal condition number 1
```

```
## Warning in simpleLoess(y, x, w, span, degree = degree, parametric = parametric, : There are other near singularities as well. 1.01
```

```
## Warning in simpleLoess(y, x, w, span, degree = degree, parametric = parametric, : zero-width neighborhood. make span bigger
```

```
## Warning: Computation failed in `stat_smooth()`:
## NA/NaN/Inf in foreign function call (arg 5)
```

```
## Warning in simpleLoess(y, x, w, span, degree = degree, parametric = parametric, : at -0.005
```

```
## Warning in simpleLoess(y, x, w, span, degree = degree, parametric = parametric, : radius 2.5e-05
```

```
## Warning in simpleLoess(y, x, w, span, degree = degree, parametric = parametric, : all data on boundary of neighborhood. make span bigger
```

```
## Warning in simpleLoess(y, x, w, span, degree = degree, parametric = parametric, : pseudoinverse used at -0.005
```

```
## Warning in simpleLoess(y, x, w, span, degree = degree, parametric = parametric, : neighborhood radius 0.005
```

```
## Warning in simpleLoess(y, x, w, span, degree = degree, parametric = parametric, : reciprocal condition number 1
```

```
## Warning in simpleLoess(y, x, w, span, degree = degree, parametric = parametric, : There are other near singularities as well. 1.01
```

```
## Warning in simpleLoess(y, x, w, span, degree = degree, parametric = parametric, : zero-width neighborhood. make span bigger
```

```
## Warning: Computation failed in `stat_smooth()`:
## NA/NaN/Inf in foreign function call (arg 5)
```

```
## Warning in simpleLoess(y, x, w, span, degree = degree, parametric = parametric, : at -0.005
```

```
## Warning in simpleLoess(y, x, w, span, degree = degree, parametric = parametric, : radius 2.5e-05
```

```
## Warning in simpleLoess(y, x, w, span, degree = degree, parametric = parametric, : all data on boundary of neighborhood. make span bigger
```

```
## Warning in simpleLoess(y, x, w, span, degree = degree, parametric = parametric, : pseudoinverse used at -0.005
```

```
## Warning in simpleLoess(y, x, w, span, degree = degree, parametric = parametric, : neighborhood radius 0.005
```

```
## Warning in simpleLoess(y, x, w, span, degree = degree, parametric = parametric, : reciprocal condition number 1
```

```
## Warning in simpleLoess(y, x, w, span, degree = degree, parametric = parametric, : There are other near singularities as well. 1.01
```

```
## Warning in simpleLoess(y, x, w, span, degree = degree, parametric = parametric, : zero-width neighborhood. make span bigger
```

```
## Warning: Computation failed in `stat_smooth()`:
## NA/NaN/Inf in foreign function call (arg 5)
```

```
## Warning in simpleLoess(y, x, w, span, degree = degree, parametric = parametric, : at -0.005
```

```
## Warning in simpleLoess(y, x, w, span, degree = degree, parametric = parametric, : radius 2.5e-05
```

```
## Warning in simpleLoess(y, x, w, span, degree = degree, parametric = parametric, : all data on boundary of neighborhood. make span bigger
```

```
## Warning in simpleLoess(y, x, w, span, degree = degree, parametric = parametric, : pseudoinverse used at -0.005
```

```
## Warning in simpleLoess(y, x, w, span, degree = degree, parametric = parametric, : neighborhood radius 0.005
```

```
## Warning in simpleLoess(y, x, w, span, degree = degree, parametric = parametric, : reciprocal condition number 1
```

```
## Warning in simpleLoess(y, x, w, span, degree = degree, parametric = parametric, : There are other near singularities as well. 1.01
```

```
## Warning in simpleLoess(y, x, w, span, degree = degree, parametric = parametric, : zero-width neighborhood. make span bigger
```

```
## Warning: Computation failed in `stat_smooth()`:
## NA/NaN/Inf in foreign function call (arg 5)
```

```
## Warning in simpleLoess(y, x, w, span, degree = degree, parametric = parametric, : at -0.005
```

```
## Warning in simpleLoess(y, x, w, span, degree = degree, parametric = parametric, : radius 2.5e-05
```

```
## Warning in simpleLoess(y, x, w, span, degree = degree, parametric = parametric, : all data on boundary of neighborhood. make span bigger
```

```
## Warning in simpleLoess(y, x, w, span, degree = degree, parametric = parametric, : pseudoinverse used at -0.005
```

```
## Warning in simpleLoess(y, x, w, span, degree = degree, parametric = parametric, : neighborhood radius 0.005
```

```
## Warning in simpleLoess(y, x, w, span, degree = degree, parametric = parametric, : reciprocal condition number 1
```

```
## Warning in simpleLoess(y, x, w, span, degree = degree, parametric = parametric, : There are other near singularities as well. 1.01
```

```
## Warning in simpleLoess(y, x, w, span, degree = degree, parametric = parametric, : zero-width neighborhood. make span bigger
```

```
## Warning: Computation failed in `stat_smooth()`:
## NA/NaN/Inf in foreign function call (arg 5)
```

```
## Warning in simpleLoess(y, x, w, span, degree = degree, parametric = parametric, : pseudoinverse used at -0.005
```

```
## Warning in simpleLoess(y, x, w, span, degree = degree, parametric = parametric, : neighborhood radius 1.005
```

```
## Warning in simpleLoess(y, x, w, span, degree = degree, parametric = parametric, : reciprocal condition number 0
```

```
## Warning in simpleLoess(y, x, w, span, degree = degree, parametric = parametric, : There are other near singularities as well. 1.01
```

```
## Warning in predLoess(object$y, object$x, newx = if (is.null(newdata)) object$x else if (is.data.frame(newdata)) as.matrix(model.frame(delete.response(terms(object)), : pseudoinverse used at -0.005
```

```
## Warning in predLoess(object$y, object$x, newx = if (is.null(newdata)) object$x else if (is.data.frame(newdata)) as.matrix(model.frame(delete.response(terms(object)), : neighborhood radius 1.005
```

```
## Warning in predLoess(object$y, object$x, newx = if (is.null(newdata)) object$x else if (is.data.frame(newdata)) as.matrix(model.frame(delete.response(terms(object)), : reciprocal condition number 0
```

```
## Warning in predLoess(object$y, object$x, newx = if (is.null(newdata)) object$x else if (is.data.frame(newdata)) as.matrix(model.frame(delete.response(terms(object)), : There are other near
## singularities as well. 1.01
```

```
## Warning in simpleLoess(y, x, w, span, degree = degree, parametric = parametric, : at -0.005
```

```
## Warning in simpleLoess(y, x, w, span, degree = degree, parametric = parametric, : radius 2.5e-05
```

```
## Warning in simpleLoess(y, x, w, span, degree = degree, parametric = parametric, : all data on boundary of neighborhood. make span bigger
```

```
## Warning in simpleLoess(y, x, w, span, degree = degree, parametric = parametric, : pseudoinverse used at -0.005
```

```
## Warning in simpleLoess(y, x, w, span, degree = degree, parametric = parametric, : neighborhood radius 0.005
```

```
## Warning in simpleLoess(y, x, w, span, degree = degree, parametric = parametric, : reciprocal condition number 1
```

```
## Warning in simpleLoess(y, x, w, span, degree = degree, parametric = parametric, : There are other near singularities as well. 1.01
```

```
## Warning in simpleLoess(y, x, w, span, degree = degree, parametric = parametric, : zero-width neighborhood. make span bigger
```

```
## Warning: Computation failed in `stat_smooth()`:
## NA/NaN/Inf in foreign function call (arg 5)
```

```
## Warning in simpleLoess(y, x, w, span, degree = degree, parametric = parametric, : at -0.005
```

```
## Warning in simpleLoess(y, x, w, span, degree = degree, parametric = parametric, : radius 2.5e-05
```

```
## Warning in simpleLoess(y, x, w, span, degree = degree, parametric = parametric, : all data on boundary of neighborhood. make span bigger
```

```
## Warning in simpleLoess(y, x, w, span, degree = degree, parametric = parametric, : pseudoinverse used at -0.005
```

```
## Warning in simpleLoess(y, x, w, span, degree = degree, parametric = parametric, : neighborhood radius 0.005
```

```
## Warning in simpleLoess(y, x, w, span, degree = degree, parametric = parametric, : reciprocal condition number 1
```

```
## Warning in simpleLoess(y, x, w, span, degree = degree, parametric = parametric, : There are other near singularities as well. 1.01
```

```
## Warning in simpleLoess(y, x, w, span, degree = degree, parametric = parametric, : zero-width neighborhood. make span bigger
```

```
## Warning: Computation failed in `stat_smooth()`:
## NA/NaN/Inf in foreign function call (arg 5)
```

```
## Warning in simpleLoess(y, x, w, span, degree = degree, parametric = parametric, : at -0.005
```

```
## Warning in simpleLoess(y, x, w, span, degree = degree, parametric = parametric, : radius 2.5e-05
```

```
## Warning in simpleLoess(y, x, w, span, degree = degree, parametric = parametric, : all data on boundary of neighborhood. make span bigger
```

```
## Warning in simpleLoess(y, x, w, span, degree = degree, parametric = parametric, : pseudoinverse used at -0.005
```

```
## Warning in simpleLoess(y, x, w, span, degree = degree, parametric = parametric, : neighborhood radius 0.005
```

```
## Warning in simpleLoess(y, x, w, span, degree = degree, parametric = parametric, : reciprocal condition number 1
```

```
## Warning in simpleLoess(y, x, w, span, degree = degree, parametric = parametric, : There are other near singularities as well. 1.01
```

```
## Warning in simpleLoess(y, x, w, span, degree = degree, parametric = parametric, : zero-width neighborhood. make span bigger
```

```
## Warning: Computation failed in `stat_smooth()`:
## NA/NaN/Inf in foreign function call (arg 5)
```

```
## Warning in simpleLoess(y, x, w, span, degree = degree, parametric = parametric, : at -0.005
```

```
## Warning in simpleLoess(y, x, w, span, degree = degree, parametric = parametric, : radius 2.5e-05
```

```
## Warning in simpleLoess(y, x, w, span, degree = degree, parametric = parametric, : all data on boundary of neighborhood. make span bigger
```

```
## Warning in simpleLoess(y, x, w, span, degree = degree, parametric = parametric, : pseudoinverse used at -0.005
```

```
## Warning in simpleLoess(y, x, w, span, degree = degree, parametric = parametric, : neighborhood radius 0.005
```

```
## Warning in simpleLoess(y, x, w, span, degree = degree, parametric = parametric, : reciprocal condition number 1
```

```
## Warning in simpleLoess(y, x, w, span, degree = degree, parametric = parametric, : There are other near singularities as well. 1.01
```

```
## Warning in simpleLoess(y, x, w, span, degree = degree, parametric = parametric, : zero-width neighborhood. make span bigger
```

```
## Warning: Computation failed in `stat_smooth()`:
## NA/NaN/Inf in foreign function call (arg 5)
```

```
## Warning in simpleLoess(y, x, w, span, degree = degree, parametric = parametric, : at -0.005
```

```
## Warning in simpleLoess(y, x, w, span, degree = degree, parametric = parametric, : radius 2.5e-05
```

```
## Warning in simpleLoess(y, x, w, span, degree = degree, parametric = parametric, : all data on boundary of neighborhood. make span bigger
```

```
## Warning in simpleLoess(y, x, w, span, degree = degree, parametric = parametric, : pseudoinverse used at -0.005
```

```
## Warning in simpleLoess(y, x, w, span, degree = degree, parametric = parametric, : neighborhood radius 0.005
```

```
## Warning in simpleLoess(y, x, w, span, degree = degree, parametric = parametric, : reciprocal condition number 1
```

```
## Warning in simpleLoess(y, x, w, span, degree = degree, parametric = parametric, : There are other near singularities as well. 1.01
```

```
## Warning in simpleLoess(y, x, w, span, degree = degree, parametric = parametric, : zero-width neighborhood. make span bigger
```

```
## Warning: Computation failed in `stat_smooth()`:
## NA/NaN/Inf in foreign function call (arg 5)
```

```
## Warning in simpleLoess(y, x, w, span, degree = degree, parametric = parametric, : at -0.005
```

```
## Warning in simpleLoess(y, x, w, span, degree = degree, parametric = parametric, : radius 2.5e-05
```

```
## Warning in simpleLoess(y, x, w, span, degree = degree, parametric = parametric, : all data on boundary of neighborhood. make span bigger
```

```
## Warning in simpleLoess(y, x, w, span, degree = degree, parametric = parametric, : pseudoinverse used at -0.005
```

```
## Warning in simpleLoess(y, x, w, span, degree = degree, parametric = parametric, : neighborhood radius 0.005
```

```
## Warning in simpleLoess(y, x, w, span, degree = degree, parametric = parametric, : reciprocal condition number 1
```

```
## Warning in simpleLoess(y, x, w, span, degree = degree, parametric = parametric, : There are other near singularities as well. 1.01
```

```
## Warning in simpleLoess(y, x, w, span, degree = degree, parametric = parametric, : zero-width neighborhood. make span bigger
```

```
## Warning: Computation failed in `stat_smooth()`:
## NA/NaN/Inf in foreign function call (arg 5)
```

```
## Warning in simpleLoess(y, x, w, span, degree = degree, parametric = parametric, : at -0.005
```

```
## Warning in simpleLoess(y, x, w, span, degree = degree, parametric = parametric, : radius 2.5e-05
```

```
## Warning in simpleLoess(y, x, w, span, degree = degree, parametric = parametric, : all data on boundary of neighborhood. make span bigger
```

```
## Warning in simpleLoess(y, x, w, span, degree = degree, parametric = parametric, : pseudoinverse used at -0.005
```

```
## Warning in simpleLoess(y, x, w, span, degree = degree, parametric = parametric, : neighborhood radius 0.005
```

```
## Warning in simpleLoess(y, x, w, span, degree = degree, parametric = parametric, : reciprocal condition number 1
```

```
## Warning in simpleLoess(y, x, w, span, degree = degree, parametric = parametric, : There are other near singularities as well. 1.01
```

```
## Warning in simpleLoess(y, x, w, span, degree = degree, parametric = parametric, : zero-width neighborhood. make span bigger
```

```
## Warning: Computation failed in `stat_smooth()`:
## NA/NaN/Inf in foreign function call (arg 5)
```

```
## Warning in simpleLoess(y, x, w, span, degree = degree, parametric = parametric, : at -0.005
```

```
## Warning in simpleLoess(y, x, w, span, degree = degree, parametric = parametric, : radius 2.5e-05
```

```
## Warning in simpleLoess(y, x, w, span, degree = degree, parametric = parametric, : all data on boundary of neighborhood. make span bigger
```

```
## Warning in simpleLoess(y, x, w, span, degree = degree, parametric = parametric, : pseudoinverse used at -0.005
```

```
## Warning in simpleLoess(y, x, w, span, degree = degree, parametric = parametric, : neighborhood radius 0.005
```

```
## Warning in simpleLoess(y, x, w, span, degree = degree, parametric = parametric, : reciprocal condition number 1
```

```
## Warning in simpleLoess(y, x, w, span, degree = degree, parametric = parametric, : There are other near singularities as well. 1.01
```

```
## Warning in simpleLoess(y, x, w, span, degree = degree, parametric = parametric, : zero-width neighborhood. make span bigger
```

```
## Warning: Computation failed in `stat_smooth()`:
## NA/NaN/Inf in foreign function call (arg 5)
```

```
## Warning in simpleLoess(y, x, w, span, degree = degree, parametric = parametric, : pseudoinverse used at -0.005
```

```
## Warning in simpleLoess(y, x, w, span, degree = degree, parametric = parametric, : neighborhood radius 1.005
```

```
## Warning in simpleLoess(y, x, w, span, degree = degree, parametric = parametric, : reciprocal condition number 0
```

```
## Warning in simpleLoess(y, x, w, span, degree = degree, parametric = parametric, : There are other near singularities as well. 1.01
```

```
## Warning in predLoess(object$y, object$x, newx = if (is.null(newdata)) object$x else if (is.data.frame(newdata)) as.matrix(model.frame(delete.response(terms(object)), : pseudoinverse used at -0.005
```

```
## Warning in predLoess(object$y, object$x, newx = if (is.null(newdata)) object$x else if (is.data.frame(newdata)) as.matrix(model.frame(delete.response(terms(object)), : neighborhood radius 1.005
```

```
## Warning in predLoess(object$y, object$x, newx = if (is.null(newdata)) object$x else if (is.data.frame(newdata)) as.matrix(model.frame(delete.response(terms(object)), : reciprocal condition number 0
```

```
## Warning in predLoess(object$y, object$x, newx = if (is.null(newdata)) object$x else if (is.data.frame(newdata)) as.matrix(model.frame(delete.response(terms(object)), : There are other near
## singularities as well. 1.01
```

```
## Warning in simpleLoess(y, x, w, span, degree = degree, parametric = parametric, : at -0.005
```

```
## Warning in simpleLoess(y, x, w, span, degree = degree, parametric = parametric, : radius 2.5e-05
```

```
## Warning in simpleLoess(y, x, w, span, degree = degree, parametric = parametric, : all data on boundary of neighborhood. make span bigger
```

```
## Warning in simpleLoess(y, x, w, span, degree = degree, parametric = parametric, : pseudoinverse used at -0.005
```

```
## Warning in simpleLoess(y, x, w, span, degree = degree, parametric = parametric, : neighborhood radius 0.005
```

```
## Warning in simpleLoess(y, x, w, span, degree = degree, parametric = parametric, : reciprocal condition number 1
```

```
## Warning in simpleLoess(y, x, w, span, degree = degree, parametric = parametric, : There are other near singularities as well. 1.01
```

```
## Warning in simpleLoess(y, x, w, span, degree = degree, parametric = parametric, : zero-width neighborhood. make span bigger
```

```
## Warning: Computation failed in `stat_smooth()`:
## NA/NaN/Inf in foreign function call (arg 5)
```

```
## Warning in simpleLoess(y, x, w, span, degree = degree, parametric = parametric, : pseudoinverse used at -0.005
```

```
## Warning in simpleLoess(y, x, w, span, degree = degree, parametric = parametric, : neighborhood radius 1.005
```

```
## Warning in simpleLoess(y, x, w, span, degree = degree, parametric = parametric, : reciprocal condition number 0
```

```
## Warning in simpleLoess(y, x, w, span, degree = degree, parametric = parametric, : There are other near singularities as well. 1.01
```

```
## Warning in predLoess(object$y, object$x, newx = if (is.null(newdata)) object$x else if (is.data.frame(newdata)) as.matrix(model.frame(delete.response(terms(object)), : pseudoinverse used at -0.005
```

```
## Warning in predLoess(object$y, object$x, newx = if (is.null(newdata)) object$x else if (is.data.frame(newdata)) as.matrix(model.frame(delete.response(terms(object)), : neighborhood radius 1.005
```

```
## Warning in predLoess(object$y, object$x, newx = if (is.null(newdata)) object$x else if (is.data.frame(newdata)) as.matrix(model.frame(delete.response(terms(object)), : reciprocal condition number 0
```

```
## Warning in predLoess(object$y, object$x, newx = if (is.null(newdata)) object$x else if (is.data.frame(newdata)) as.matrix(model.frame(delete.response(terms(object)), : There are other near
## singularities as well. 1.01
```

```
## Warning in simpleLoess(y, x, w, span, degree = degree, parametric = parametric, : at -0.005
```

```
## Warning in simpleLoess(y, x, w, span, degree = degree, parametric = parametric, : radius 2.5e-05
```

```
## Warning in simpleLoess(y, x, w, span, degree = degree, parametric = parametric, : all data on boundary of neighborhood. make span bigger
```

```
## Warning in simpleLoess(y, x, w, span, degree = degree, parametric = parametric, : pseudoinverse used at -0.005
```

```
## Warning in simpleLoess(y, x, w, span, degree = degree, parametric = parametric, : neighborhood radius 0.005
```

```
## Warning in simpleLoess(y, x, w, span, degree = degree, parametric = parametric, : reciprocal condition number 1
```

```
## Warning in simpleLoess(y, x, w, span, degree = degree, parametric = parametric, : There are other near singularities as well. 1.01
```

```
## Warning in simpleLoess(y, x, w, span, degree = degree, parametric = parametric, : zero-width neighborhood. make span bigger
```

```
## Warning: Computation failed in `stat_smooth()`:
## NA/NaN/Inf in foreign function call (arg 5)
```

```
## Warning in simpleLoess(y, x, w, span, degree = degree, parametric = parametric, : at -0.005
```

```
## Warning in simpleLoess(y, x, w, span, degree = degree, parametric = parametric, : radius 2.5e-05
```

```
## Warning in simpleLoess(y, x, w, span, degree = degree, parametric = parametric, : all data on boundary of neighborhood. make span bigger
```

```
## Warning in simpleLoess(y, x, w, span, degree = degree, parametric = parametric, : pseudoinverse used at -0.005
```

```
## Warning in simpleLoess(y, x, w, span, degree = degree, parametric = parametric, : neighborhood radius 0.005
```

```
## Warning in simpleLoess(y, x, w, span, degree = degree, parametric = parametric, : reciprocal condition number 1
```

```
## Warning in simpleLoess(y, x, w, span, degree = degree, parametric = parametric, : There are other near singularities as well. 1.01
```

```
## Warning in simpleLoess(y, x, w, span, degree = degree, parametric = parametric, : zero-width neighborhood. make span bigger
```

```
## Warning: Computation failed in `stat_smooth()`:
## NA/NaN/Inf in foreign function call (arg 5)
```

```
## Warning in simpleLoess(y, x, w, span, degree = degree, parametric = parametric, : at -0.005
```

```
## Warning in simpleLoess(y, x, w, span, degree = degree, parametric = parametric, : radius 2.5e-05
```

```
## Warning in simpleLoess(y, x, w, span, degree = degree, parametric = parametric, : all data on boundary of neighborhood. make span bigger
```

```
## Warning in simpleLoess(y, x, w, span, degree = degree, parametric = parametric, : pseudoinverse used at -0.005
```

```
## Warning in simpleLoess(y, x, w, span, degree = degree, parametric = parametric, : neighborhood radius 0.005
```

```
## Warning in simpleLoess(y, x, w, span, degree = degree, parametric = parametric, : reciprocal condition number 1
```

```
## Warning in simpleLoess(y, x, w, span, degree = degree, parametric = parametric, : There are other near singularities as well. 1.01
```

```
## Warning in simpleLoess(y, x, w, span, degree = degree, parametric = parametric, : zero-width neighborhood. make span bigger
```

```
## Warning: Computation failed in `stat_smooth()`:
## NA/NaN/Inf in foreign function call (arg 5)
```

```
## Warning in simpleLoess(y, x, w, span, degree = degree, parametric = parametric, : at -0.005
```

```
## Warning in simpleLoess(y, x, w, span, degree = degree, parametric = parametric, : radius 2.5e-05
```

```
## Warning in simpleLoess(y, x, w, span, degree = degree, parametric = parametric, : all data on boundary of neighborhood. make span bigger
```

```
## Warning in simpleLoess(y, x, w, span, degree = degree, parametric = parametric, : pseudoinverse used at -0.005
```

```
## Warning in simpleLoess(y, x, w, span, degree = degree, parametric = parametric, : neighborhood radius 0.005
```

```
## Warning in simpleLoess(y, x, w, span, degree = degree, parametric = parametric, : reciprocal condition number 1
```

```
## Warning in simpleLoess(y, x, w, span, degree = degree, parametric = parametric, : There are other near singularities as well. 1.01
```

```
## Warning in simpleLoess(y, x, w, span, degree = degree, parametric = parametric, : zero-width neighborhood. make span bigger
```

```
## Warning: Computation failed in `stat_smooth()`:
## NA/NaN/Inf in foreign function call (arg 5)
```

```
## Warning in simpleLoess(y, x, w, span, degree = degree, parametric = parametric, : at -0.005
```

```
## Warning in simpleLoess(y, x, w, span, degree = degree, parametric = parametric, : radius 2.5e-05
```

```
## Warning in simpleLoess(y, x, w, span, degree = degree, parametric = parametric, : all data on boundary of neighborhood. make span bigger
```

```
## Warning in simpleLoess(y, x, w, span, degree = degree, parametric = parametric, : pseudoinverse used at -0.005
```

```
## Warning in simpleLoess(y, x, w, span, degree = degree, parametric = parametric, : neighborhood radius 0.005
```

```
## Warning in simpleLoess(y, x, w, span, degree = degree, parametric = parametric, : reciprocal condition number 1
```

```
## Warning in simpleLoess(y, x, w, span, degree = degree, parametric = parametric, : There are other near singularities as well. 1.01
```

```
## Warning in simpleLoess(y, x, w, span, degree = degree, parametric = parametric, : zero-width neighborhood. make span bigger
```

```
## Warning: Computation failed in `stat_smooth()`:
## NA/NaN/Inf in foreign function call (arg 5)
```

```
## Warning in simpleLoess(y, x, w, span, degree = degree, parametric = parametric, : at -0.005
```

```
## Warning in simpleLoess(y, x, w, span, degree = degree, parametric = parametric, : radius 2.5e-05
```

```
## Warning in simpleLoess(y, x, w, span, degree = degree, parametric = parametric, : all data on boundary of neighborhood. make span bigger
```

```
## Warning in simpleLoess(y, x, w, span, degree = degree, parametric = parametric, : pseudoinverse used at -0.005
```

```
## Warning in simpleLoess(y, x, w, span, degree = degree, parametric = parametric, : neighborhood radius 0.005
```

```
## Warning in simpleLoess(y, x, w, span, degree = degree, parametric = parametric, : reciprocal condition number 1
```

```
## Warning in simpleLoess(y, x, w, span, degree = degree, parametric = parametric, : There are other near singularities as well. 1.01
```

```
## Warning in simpleLoess(y, x, w, span, degree = degree, parametric = parametric, : zero-width neighborhood. make span bigger
```

```
## Warning: Computation failed in `stat_smooth()`:
## NA/NaN/Inf in foreign function call (arg 5)
```

```
## Warning in simpleLoess(y, x, w, span, degree = degree, parametric = parametric, : at -0.005
```

```
## Warning in simpleLoess(y, x, w, span, degree = degree, parametric = parametric, : radius 2.5e-05
```

```
## Warning in simpleLoess(y, x, w, span, degree = degree, parametric = parametric, : all data on boundary of neighborhood. make span bigger
```

```
## Warning in simpleLoess(y, x, w, span, degree = degree, parametric = parametric, : pseudoinverse used at -0.005
```

```
## Warning in simpleLoess(y, x, w, span, degree = degree, parametric = parametric, : neighborhood radius 0.005
```

```
## Warning in simpleLoess(y, x, w, span, degree = degree, parametric = parametric, : reciprocal condition number 1
```

```
## Warning in simpleLoess(y, x, w, span, degree = degree, parametric = parametric, : There are other near singularities as well. 1.01
```

```
## Warning in simpleLoess(y, x, w, span, degree = degree, parametric = parametric, : zero-width neighborhood. make span bigger
```

```
## Warning: Computation failed in `stat_smooth()`:
## NA/NaN/Inf in foreign function call (arg 5)
```

```
## Warning in simpleLoess(y, x, w, span, degree = degree, parametric = parametric, : pseudoinverse used at -0.005
```

```
## Warning in simpleLoess(y, x, w, span, degree = degree, parametric = parametric, : neighborhood radius 1.005
```

```
## Warning in simpleLoess(y, x, w, span, degree = degree, parametric = parametric, : reciprocal condition number 0
```

```
## Warning in simpleLoess(y, x, w, span, degree = degree, parametric = parametric, : There are other near singularities as well. 1.01
```

```
## Warning in predLoess(object$y, object$x, newx = if (is.null(newdata)) object$x else if (is.data.frame(newdata)) as.matrix(model.frame(delete.response(terms(object)), : pseudoinverse used at -0.005
```

```
## Warning in predLoess(object$y, object$x, newx = if (is.null(newdata)) object$x else if (is.data.frame(newdata)) as.matrix(model.frame(delete.response(terms(object)), : neighborhood radius 1.005
```

```
## Warning in predLoess(object$y, object$x, newx = if (is.null(newdata)) object$x else if (is.data.frame(newdata)) as.matrix(model.frame(delete.response(terms(object)), : reciprocal condition number 0
```

```
## Warning in predLoess(object$y, object$x, newx = if (is.null(newdata)) object$x else if (is.data.frame(newdata)) as.matrix(model.frame(delete.response(terms(object)), : There are other near
## singularities as well. 1.01
```

```
## Warning in simpleLoess(y, x, w, span, degree = degree, parametric = parametric, : at -0.005
```

```
## Warning in simpleLoess(y, x, w, span, degree = degree, parametric = parametric, : radius 2.5e-05
```

```
## Warning in simpleLoess(y, x, w, span, degree = degree, parametric = parametric, : all data on boundary of neighborhood. make span bigger
```

```
## Warning in simpleLoess(y, x, w, span, degree = degree, parametric = parametric, : pseudoinverse used at -0.005
```

```
## Warning in simpleLoess(y, x, w, span, degree = degree, parametric = parametric, : neighborhood radius 0.005
```

```
## Warning in simpleLoess(y, x, w, span, degree = degree, parametric = parametric, : reciprocal condition number 1
```

```
## Warning in simpleLoess(y, x, w, span, degree = degree, parametric = parametric, : There are other near singularities as well. 1.01
```

```
## Warning in simpleLoess(y, x, w, span, degree = degree, parametric = parametric, : zero-width neighborhood. make span bigger
```

```
## Warning: Computation failed in `stat_smooth()`:
## NA/NaN/Inf in foreign function call (arg 5)
```

```
## Warning in simpleLoess(y, x, w, span, degree = degree, parametric = parametric, : at -0.005
```

```
## Warning in simpleLoess(y, x, w, span, degree = degree, parametric = parametric, : radius 2.5e-05
```

```
## Warning in simpleLoess(y, x, w, span, degree = degree, parametric = parametric, : all data on boundary of neighborhood. make span bigger
```

```
## Warning in simpleLoess(y, x, w, span, degree = degree, parametric = parametric, : pseudoinverse used at -0.005
```

```
## Warning in simpleLoess(y, x, w, span, degree = degree, parametric = parametric, : neighborhood radius 0.005
```

```
## Warning in simpleLoess(y, x, w, span, degree = degree, parametric = parametric, : reciprocal condition number 1
```

```
## Warning in simpleLoess(y, x, w, span, degree = degree, parametric = parametric, : There are other near singularities as well. 1.01
```

```
## Warning in simpleLoess(y, x, w, span, degree = degree, parametric = parametric, : zero-width neighborhood. make span bigger
```

```
## Warning: Computation failed in `stat_smooth()`:
## NA/NaN/Inf in foreign function call (arg 5)
```

```
## Warning in simpleLoess(y, x, w, span, degree = degree, parametric = parametric, : at -0.005
```

```
## Warning in simpleLoess(y, x, w, span, degree = degree, parametric = parametric, : radius 2.5e-05
```

```
## Warning in simpleLoess(y, x, w, span, degree = degree, parametric = parametric, : all data on boundary of neighborhood. make span bigger
```

```
## Warning in simpleLoess(y, x, w, span, degree = degree, parametric = parametric, : pseudoinverse used at -0.005
```

```
## Warning in simpleLoess(y, x, w, span, degree = degree, parametric = parametric, : neighborhood radius 0.005
```

```
## Warning in simpleLoess(y, x, w, span, degree = degree, parametric = parametric, : reciprocal condition number 1
```

```
## Warning in simpleLoess(y, x, w, span, degree = degree, parametric = parametric, : There are other near singularities as well. 1.01
```

```
## Warning in simpleLoess(y, x, w, span, degree = degree, parametric = parametric, : zero-width neighborhood. make span bigger
```

```
## Warning: Computation failed in `stat_smooth()`:
## NA/NaN/Inf in foreign function call (arg 5)
```

```
## Warning in simpleLoess(y, x, w, span, degree = degree, parametric = parametric, : at -0.005
```

```
## Warning in simpleLoess(y, x, w, span, degree = degree, parametric = parametric, : radius 2.5e-05
```

```
## Warning in simpleLoess(y, x, w, span, degree = degree, parametric = parametric, : all data on boundary of neighborhood. make span bigger
```

```
## Warning in simpleLoess(y, x, w, span, degree = degree, parametric = parametric, : pseudoinverse used at -0.005
```

```
## Warning in simpleLoess(y, x, w, span, degree = degree, parametric = parametric, : neighborhood radius 0.005
```

```
## Warning in simpleLoess(y, x, w, span, degree = degree, parametric = parametric, : reciprocal condition number 1
```

```
## Warning in simpleLoess(y, x, w, span, degree = degree, parametric = parametric, : There are other near singularities as well. 1.01
```

```
## Warning in simpleLoess(y, x, w, span, degree = degree, parametric = parametric, : zero-width neighborhood. make span bigger
```

```
## Warning: Computation failed in `stat_smooth()`:
## NA/NaN/Inf in foreign function call (arg 5)
```

```
## Warning in simpleLoess(y, x, w, span, degree = degree, parametric = parametric, : at -0.005
```

```
## Warning in simpleLoess(y, x, w, span, degree = degree, parametric = parametric, : radius 2.5e-05
```

```
## Warning in simpleLoess(y, x, w, span, degree = degree, parametric = parametric, : all data on boundary of neighborhood. make span bigger
```

```
## Warning in simpleLoess(y, x, w, span, degree = degree, parametric = parametric, : pseudoinverse used at -0.005
```

```
## Warning in simpleLoess(y, x, w, span, degree = degree, parametric = parametric, : neighborhood radius 0.005
```

```
## Warning in simpleLoess(y, x, w, span, degree = degree, parametric = parametric, : reciprocal condition number 1
```

```
## Warning in simpleLoess(y, x, w, span, degree = degree, parametric = parametric, : There are other near singularities as well. 1.01
```

```
## Warning in simpleLoess(y, x, w, span, degree = degree, parametric = parametric, : zero-width neighborhood. make span bigger
```

```
## Warning: Computation failed in `stat_smooth()`:
## NA/NaN/Inf in foreign function call (arg 5)
```

```
## Warning in simpleLoess(y, x, w, span, degree = degree, parametric = parametric, : at -0.005
```

```
## Warning in simpleLoess(y, x, w, span, degree = degree, parametric = parametric, : radius 2.5e-05
```

```
## Warning in simpleLoess(y, x, w, span, degree = degree, parametric = parametric, : all data on boundary of neighborhood. make span bigger
```

```
## Warning in simpleLoess(y, x, w, span, degree = degree, parametric = parametric, : pseudoinverse used at -0.005
```

```
## Warning in simpleLoess(y, x, w, span, degree = degree, parametric = parametric, : neighborhood radius 0.005
```

```
## Warning in simpleLoess(y, x, w, span, degree = degree, parametric = parametric, : reciprocal condition number 1
```

```
## Warning in simpleLoess(y, x, w, span, degree = degree, parametric = parametric, : There are other near singularities as well. 1.01
```

```
## Warning in simpleLoess(y, x, w, span, degree = degree, parametric = parametric, : zero-width neighborhood. make span bigger
```

```
## Warning: Computation failed in `stat_smooth()`:
## NA/NaN/Inf in foreign function call (arg 5)
```

```
## Warning in simpleLoess(y, x, w, span, degree = degree, parametric = parametric, : at -0.005
```

```
## Warning in simpleLoess(y, x, w, span, degree = degree, parametric = parametric, : radius 2.5e-05
```

```
## Warning in simpleLoess(y, x, w, span, degree = degree, parametric = parametric, : all data on boundary of neighborhood. make span bigger
```

```
## Warning in simpleLoess(y, x, w, span, degree = degree, parametric = parametric, : pseudoinverse used at -0.005
```

```
## Warning in simpleLoess(y, x, w, span, degree = degree, parametric = parametric, : neighborhood radius 0.005
```

```
## Warning in simpleLoess(y, x, w, span, degree = degree, parametric = parametric, : reciprocal condition number 1
```

```
## Warning in simpleLoess(y, x, w, span, degree = degree, parametric = parametric, : There are other near singularities as well. 1.01
```

```
## Warning in simpleLoess(y, x, w, span, degree = degree, parametric = parametric, : zero-width neighborhood. make span bigger
```

```
## Warning: Computation failed in `stat_smooth()`:
## NA/NaN/Inf in foreign function call (arg 5)
```

```
## Warning in simpleLoess(y, x, w, span, degree = degree, parametric = parametric, : at -0.005
```

```
## Warning in simpleLoess(y, x, w, span, degree = degree, parametric = parametric, : radius 2.5e-05
```

```
## Warning in simpleLoess(y, x, w, span, degree = degree, parametric = parametric, : all data on boundary of neighborhood. make span bigger
```

```
## Warning in simpleLoess(y, x, w, span, degree = degree, parametric = parametric, : pseudoinverse used at -0.005
```

```
## Warning in simpleLoess(y, x, w, span, degree = degree, parametric = parametric, : neighborhood radius 0.005
```

```
## Warning in simpleLoess(y, x, w, span, degree = degree, parametric = parametric, : reciprocal condition number 1
```

```
## Warning in simpleLoess(y, x, w, span, degree = degree, parametric = parametric, : There are other near singularities as well. 1.01
```

```
## Warning in simpleLoess(y, x, w, span, degree = degree, parametric = parametric, : zero-width neighborhood. make span bigger
```

```
## Warning: Computation failed in `stat_smooth()`:
## NA/NaN/Inf in foreign function call (arg 5)
```

```
## Warning in simpleLoess(y, x, w, span, degree = degree, parametric = parametric, : at -0.005
```

```
## Warning in simpleLoess(y, x, w, span, degree = degree, parametric = parametric, : radius 2.5e-05
```

```
## Warning in simpleLoess(y, x, w, span, degree = degree, parametric = parametric, : all data on boundary of neighborhood. make span bigger
```

```
## Warning in simpleLoess(y, x, w, span, degree = degree, parametric = parametric, : pseudoinverse used at -0.005
```

```
## Warning in simpleLoess(y, x, w, span, degree = degree, parametric = parametric, : neighborhood radius 0.005
```

```
## Warning in simpleLoess(y, x, w, span, degree = degree, parametric = parametric, : reciprocal condition number 1
```

```
## Warning in simpleLoess(y, x, w, span, degree = degree, parametric = parametric, : There are other near singularities as well. 1.01
```

```
## Warning in simpleLoess(y, x, w, span, degree = degree, parametric = parametric, : zero-width neighborhood. make span bigger
```

```
## Warning: Computation failed in `stat_smooth()`:
## NA/NaN/Inf in foreign function call (arg 5)
```

```
## Warning in simpleLoess(y, x, w, span, degree = degree, parametric = parametric, : at -0.005
```

```
## Warning in simpleLoess(y, x, w, span, degree = degree, parametric = parametric, : radius 2.5e-05
```

```
## Warning in simpleLoess(y, x, w, span, degree = degree, parametric = parametric, : all data on boundary of neighborhood. make span bigger
```

```
## Warning in simpleLoess(y, x, w, span, degree = degree, parametric = parametric, : pseudoinverse used at -0.005
```

```
## Warning in simpleLoess(y, x, w, span, degree = degree, parametric = parametric, : neighborhood radius 0.005
```

```
## Warning in simpleLoess(y, x, w, span, degree = degree, parametric = parametric, : reciprocal condition number 1
```

```
## Warning in simpleLoess(y, x, w, span, degree = degree, parametric = parametric, : There are other near singularities as well. 1.01
```

```
## Warning in simpleLoess(y, x, w, span, degree = degree, parametric = parametric, : zero-width neighborhood. make span bigger
```

```
## Warning: Computation failed in `stat_smooth()`:
## NA/NaN/Inf in foreign function call (arg 5)
```

```
## Warning in simpleLoess(y, x, w, span, degree = degree, parametric = parametric, : at -0.005
```

```
## Warning in simpleLoess(y, x, w, span, degree = degree, parametric = parametric, : radius 2.5e-05
```

```
## Warning in simpleLoess(y, x, w, span, degree = degree, parametric = parametric, : all data on boundary of neighborhood. make span bigger
```

```
## Warning in simpleLoess(y, x, w, span, degree = degree, parametric = parametric, : pseudoinverse used at -0.005
```

```
## Warning in simpleLoess(y, x, w, span, degree = degree, parametric = parametric, : neighborhood radius 0.005
```

```
## Warning in simpleLoess(y, x, w, span, degree = degree, parametric = parametric, : reciprocal condition number 1
```

```
## Warning in simpleLoess(y, x, w, span, degree = degree, parametric = parametric, : There are other near singularities as well. 1.01
```

```
## Warning in simpleLoess(y, x, w, span, degree = degree, parametric = parametric, : zero-width neighborhood. make span bigger
```

```
## Warning: Computation failed in `stat_smooth()`:
## NA/NaN/Inf in foreign function call (arg 5)
```

```
## Warning in simpleLoess(y, x, w, span, degree = degree, parametric = parametric, : at -0.005
```

```
## Warning in simpleLoess(y, x, w, span, degree = degree, parametric = parametric, : radius 2.5e-05
```

```
## Warning in simpleLoess(y, x, w, span, degree = degree, parametric = parametric, : all data on boundary of neighborhood. make span bigger
```

```
## Warning in simpleLoess(y, x, w, span, degree = degree, parametric = parametric, : pseudoinverse used at -0.005
```

```
## Warning in simpleLoess(y, x, w, span, degree = degree, parametric = parametric, : neighborhood radius 0.005
```

```
## Warning in simpleLoess(y, x, w, span, degree = degree, parametric = parametric, : reciprocal condition number 1
```

```
## Warning in simpleLoess(y, x, w, span, degree = degree, parametric = parametric, : There are other near singularities as well. 1.01
```

```
## Warning in simpleLoess(y, x, w, span, degree = degree, parametric = parametric, : zero-width neighborhood. make span bigger
```

```
## Warning: Computation failed in `stat_smooth()`:
## NA/NaN/Inf in foreign function call (arg 5)
```

```
## Warning in simpleLoess(y, x, w, span, degree = degree, parametric = parametric, : at -0.005
```

```
## Warning in simpleLoess(y, x, w, span, degree = degree, parametric = parametric, : radius 2.5e-05
```

```
## Warning in simpleLoess(y, x, w, span, degree = degree, parametric = parametric, : all data on boundary of neighborhood. make span bigger
```

```
## Warning in simpleLoess(y, x, w, span, degree = degree, parametric = parametric, : pseudoinverse used at -0.005
```

```
## Warning in simpleLoess(y, x, w, span, degree = degree, parametric = parametric, : neighborhood radius 0.005
```

```
## Warning in simpleLoess(y, x, w, span, degree = degree, parametric = parametric, : reciprocal condition number 1
```

```
## Warning in simpleLoess(y, x, w, span, degree = degree, parametric = parametric, : There are other near singularities as well. 1.01
```

```
## Warning in simpleLoess(y, x, w, span, degree = degree, parametric = parametric, : zero-width neighborhood. make span bigger
```

```
## Warning: Computation failed in `stat_smooth()`:
## NA/NaN/Inf in foreign function call (arg 5)
```

```
## Warning in simpleLoess(y, x, w, span, degree = degree, parametric = parametric, : at -0.005
```

```
## Warning in simpleLoess(y, x, w, span, degree = degree, parametric = parametric, : radius 2.5e-05
```

```
## Warning in simpleLoess(y, x, w, span, degree = degree, parametric = parametric, : all data on boundary of neighborhood. make span bigger
```

```
## Warning in simpleLoess(y, x, w, span, degree = degree, parametric = parametric, : pseudoinverse used at -0.005
```

```
## Warning in simpleLoess(y, x, w, span, degree = degree, parametric = parametric, : neighborhood radius 0.005
```

```
## Warning in simpleLoess(y, x, w, span, degree = degree, parametric = parametric, : reciprocal condition number 1
```

```
## Warning in simpleLoess(y, x, w, span, degree = degree, parametric = parametric, : There are other near singularities as well. 1.01
```

```
## Warning in simpleLoess(y, x, w, span, degree = degree, parametric = parametric, : zero-width neighborhood. make span bigger
```

```
## Warning: Computation failed in `stat_smooth()`:
## NA/NaN/Inf in foreign function call (arg 5)
```

```
## Warning in simpleLoess(y, x, w, span, degree = degree, parametric = parametric, : at -0.005
```

```
## Warning in simpleLoess(y, x, w, span, degree = degree, parametric = parametric, : radius 2.5e-05
```

```
## Warning in simpleLoess(y, x, w, span, degree = degree, parametric = parametric, : all data on boundary of neighborhood. make span bigger
```

```
## Warning in simpleLoess(y, x, w, span, degree = degree, parametric = parametric, : pseudoinverse used at -0.005
```

```
## Warning in simpleLoess(y, x, w, span, degree = degree, parametric = parametric, : neighborhood radius 0.005
```

```
## Warning in simpleLoess(y, x, w, span, degree = degree, parametric = parametric, : reciprocal condition number 1
```

```
## Warning in simpleLoess(y, x, w, span, degree = degree, parametric = parametric, : There are other near singularities as well. 1.01
```

```
## Warning in simpleLoess(y, x, w, span, degree = degree, parametric = parametric, : zero-width neighborhood. make span bigger
```

```
## Warning: Computation failed in `stat_smooth()`:
## NA/NaN/Inf in foreign function call (arg 5)
```

```
## Warning in simpleLoess(y, x, w, span, degree = degree, parametric = parametric, : at -0.005
```

```
## Warning in simpleLoess(y, x, w, span, degree = degree, parametric = parametric, : radius 2.5e-05
```

```
## Warning in simpleLoess(y, x, w, span, degree = degree, parametric = parametric, : all data on boundary of neighborhood. make span bigger
```

```
## Warning in simpleLoess(y, x, w, span, degree = degree, parametric = parametric, : pseudoinverse used at -0.005
```

```
## Warning in simpleLoess(y, x, w, span, degree = degree, parametric = parametric, : neighborhood radius 0.005
```

```
## Warning in simpleLoess(y, x, w, span, degree = degree, parametric = parametric, : reciprocal condition number 1
```

```
## Warning in simpleLoess(y, x, w, span, degree = degree, parametric = parametric, : There are other near singularities as well. 1.01
```

```
## Warning in simpleLoess(y, x, w, span, degree = degree, parametric = parametric, : zero-width neighborhood. make span bigger
```

```
## Warning: Computation failed in `stat_smooth()`:
## NA/NaN/Inf in foreign function call (arg 5)
```

```
## Warning in simpleLoess(y, x, w, span, degree = degree, parametric = parametric, : at -0.005
```

```
## Warning in simpleLoess(y, x, w, span, degree = degree, parametric = parametric, : radius 2.5e-05
```

```
## Warning in simpleLoess(y, x, w, span, degree = degree, parametric = parametric, : all data on boundary of neighborhood. make span bigger
```

```
## Warning in simpleLoess(y, x, w, span, degree = degree, parametric = parametric, : pseudoinverse used at -0.005
```

```
## Warning in simpleLoess(y, x, w, span, degree = degree, parametric = parametric, : neighborhood radius 0.005
```

```
## Warning in simpleLoess(y, x, w, span, degree = degree, parametric = parametric, : reciprocal condition number 1
```

```
## Warning in simpleLoess(y, x, w, span, degree = degree, parametric = parametric, : There are other near singularities as well. 1.01
```

```
## Warning in simpleLoess(y, x, w, span, degree = degree, parametric = parametric, : zero-width neighborhood. make span bigger
```

```
## Warning: Computation failed in `stat_smooth()`:
## NA/NaN/Inf in foreign function call (arg 5)
```

```
## Warning in simpleLoess(y, x, w, span, degree = degree, parametric = parametric, : at -0.005
```

```
## Warning in simpleLoess(y, x, w, span, degree = degree, parametric = parametric, : radius 2.5e-05
```

```
## Warning in simpleLoess(y, x, w, span, degree = degree, parametric = parametric, : all data on boundary of neighborhood. make span bigger
```

```
## Warning in simpleLoess(y, x, w, span, degree = degree, parametric = parametric, : pseudoinverse used at -0.005
```

```
## Warning in simpleLoess(y, x, w, span, degree = degree, parametric = parametric, : neighborhood radius 0.005
```

```
## Warning in simpleLoess(y, x, w, span, degree = degree, parametric = parametric, : reciprocal condition number 1
```

```
## Warning in simpleLoess(y, x, w, span, degree = degree, parametric = parametric, : There are other near singularities as well. 1.01
```

```
## Warning in simpleLoess(y, x, w, span, degree = degree, parametric = parametric, : zero-width neighborhood. make span bigger
```

```
## Warning: Computation failed in `stat_smooth()`:
## NA/NaN/Inf in foreign function call (arg 5)
```

```
## Warning in simpleLoess(y, x, w, span, degree = degree, parametric = parametric, : pseudoinverse used at -0.005
```

```
## Warning in simpleLoess(y, x, w, span, degree = degree, parametric = parametric, : neighborhood radius 1.005
```

```
## Warning in simpleLoess(y, x, w, span, degree = degree, parametric = parametric, : reciprocal condition number 0
```

```
## Warning in simpleLoess(y, x, w, span, degree = degree, parametric = parametric, : There are other near singularities as well. 1.01
```

```
## Warning in predLoess(object$y, object$x, newx = if (is.null(newdata)) object$x else if (is.data.frame(newdata)) as.matrix(model.frame(delete.response(terms(object)), : pseudoinverse used at -0.005
```

```
## Warning in predLoess(object$y, object$x, newx = if (is.null(newdata)) object$x else if (is.data.frame(newdata)) as.matrix(model.frame(delete.response(terms(object)), : neighborhood radius 1.005
```

```
## Warning in predLoess(object$y, object$x, newx = if (is.null(newdata)) object$x else if (is.data.frame(newdata)) as.matrix(model.frame(delete.response(terms(object)), : reciprocal condition number 0
```

```
## Warning in predLoess(object$y, object$x, newx = if (is.null(newdata)) object$x else if (is.data.frame(newdata)) as.matrix(model.frame(delete.response(terms(object)), : There are other near
## singularities as well. 1.01
```

```
## Warning in simpleLoess(y, x, w, span, degree = degree, parametric = parametric, : at -0.005
```

```
## Warning in simpleLoess(y, x, w, span, degree = degree, parametric = parametric, : radius 2.5e-05
```

```
## Warning in simpleLoess(y, x, w, span, degree = degree, parametric = parametric, : all data on boundary of neighborhood. make span bigger
```

```
## Warning in simpleLoess(y, x, w, span, degree = degree, parametric = parametric, : pseudoinverse used at -0.005
```

```
## Warning in simpleLoess(y, x, w, span, degree = degree, parametric = parametric, : neighborhood radius 0.005
```

```
## Warning in simpleLoess(y, x, w, span, degree = degree, parametric = parametric, : reciprocal condition number 1
```

```
## Warning in simpleLoess(y, x, w, span, degree = degree, parametric = parametric, : There are other near singularities as well. 1.01
```

```
## Warning in simpleLoess(y, x, w, span, degree = degree, parametric = parametric, : zero-width neighborhood. make span bigger
```

```
## Warning: Computation failed in `stat_smooth()`:
## NA/NaN/Inf in foreign function call (arg 5)
```

```
## Warning in simpleLoess(y, x, w, span, degree = degree, parametric = parametric, : at -0.005
```

```
## Warning in simpleLoess(y, x, w, span, degree = degree, parametric = parametric, : radius 2.5e-05
```

```
## Warning in simpleLoess(y, x, w, span, degree = degree, parametric = parametric, : all data on boundary of neighborhood. make span bigger
```

```
## Warning in simpleLoess(y, x, w, span, degree = degree, parametric = parametric, : pseudoinverse used at -0.005
```

```
## Warning in simpleLoess(y, x, w, span, degree = degree, parametric = parametric, : neighborhood radius 0.005
```

```
## Warning in simpleLoess(y, x, w, span, degree = degree, parametric = parametric, : reciprocal condition number 1
```

```
## Warning in simpleLoess(y, x, w, span, degree = degree, parametric = parametric, : There are other near singularities as well. 1.01
```

```
## Warning in simpleLoess(y, x, w, span, degree = degree, parametric = parametric, : zero-width neighborhood. make span bigger
```

```
## Warning: Computation failed in `stat_smooth()`:
## NA/NaN/Inf in foreign function call (arg 5)
```

```
## Warning in simpleLoess(y, x, w, span, degree = degree, parametric = parametric, : at -0.005
```

```
## Warning in simpleLoess(y, x, w, span, degree = degree, parametric = parametric, : radius 2.5e-05
```

```
## Warning in simpleLoess(y, x, w, span, degree = degree, parametric = parametric, : all data on boundary of neighborhood. make span bigger
```

```
## Warning in simpleLoess(y, x, w, span, degree = degree, parametric = parametric, : pseudoinverse used at -0.005
```

```
## Warning in simpleLoess(y, x, w, span, degree = degree, parametric = parametric, : neighborhood radius 0.005
```

```
## Warning in simpleLoess(y, x, w, span, degree = degree, parametric = parametric, : reciprocal condition number 1
```

```
## Warning in simpleLoess(y, x, w, span, degree = degree, parametric = parametric, : There are other near singularities as well. 1.01
```

```
## Warning in simpleLoess(y, x, w, span, degree = degree, parametric = parametric, : zero-width neighborhood. make span bigger
```

```
## Warning: Computation failed in `stat_smooth()`:
## NA/NaN/Inf in foreign function call (arg 5)
```

```
## Warning in simpleLoess(y, x, w, span, degree = degree, parametric = parametric, : at -0.005
```

```
## Warning in simpleLoess(y, x, w, span, degree = degree, parametric = parametric, : radius 2.5e-05
```

```
## Warning in simpleLoess(y, x, w, span, degree = degree, parametric = parametric, : all data on boundary of neighborhood. make span bigger
```

```
## Warning in simpleLoess(y, x, w, span, degree = degree, parametric = parametric, : pseudoinverse used at -0.005
```

```
## Warning in simpleLoess(y, x, w, span, degree = degree, parametric = parametric, : neighborhood radius 0.005
```

```
## Warning in simpleLoess(y, x, w, span, degree = degree, parametric = parametric, : reciprocal condition number 1
```

```
## Warning in simpleLoess(y, x, w, span, degree = degree, parametric = parametric, : There are other near singularities as well. 1.01
```

```
## Warning in simpleLoess(y, x, w, span, degree = degree, parametric = parametric, : zero-width neighborhood. make span bigger
```

```
## Warning: Computation failed in `stat_smooth()`:
## NA/NaN/Inf in foreign function call (arg 5)
```

```
## Warning in simpleLoess(y, x, w, span, degree = degree, parametric = parametric, : at -0.005
```

```
## Warning in simpleLoess(y, x, w, span, degree = degree, parametric = parametric, : radius 2.5e-05
```

```
## Warning in simpleLoess(y, x, w, span, degree = degree, parametric = parametric, : all data on boundary of neighborhood. make span bigger
```

```
## Warning in simpleLoess(y, x, w, span, degree = degree, parametric = parametric, : pseudoinverse used at -0.005
```

```
## Warning in simpleLoess(y, x, w, span, degree = degree, parametric = parametric, : neighborhood radius 0.005
```

```
## Warning in simpleLoess(y, x, w, span, degree = degree, parametric = parametric, : reciprocal condition number 1
```

```
## Warning in simpleLoess(y, x, w, span, degree = degree, parametric = parametric, : There are other near singularities as well. 1.01
```

```
## Warning in simpleLoess(y, x, w, span, degree = degree, parametric = parametric, : zero-width neighborhood. make span bigger
```

```
## Warning: Computation failed in `stat_smooth()`:
## NA/NaN/Inf in foreign function call (arg 5)
```

```
## Warning in simpleLoess(y, x, w, span, degree = degree, parametric = parametric, : at -0.005
```

```
## Warning in simpleLoess(y, x, w, span, degree = degree, parametric = parametric, : radius 2.5e-05
```

```
## Warning in simpleLoess(y, x, w, span, degree = degree, parametric = parametric, : all data on boundary of neighborhood. make span bigger
```

```
## Warning in simpleLoess(y, x, w, span, degree = degree, parametric = parametric, : pseudoinverse used at -0.005
```

```
## Warning in simpleLoess(y, x, w, span, degree = degree, parametric = parametric, : neighborhood radius 0.005
```

```
## Warning in simpleLoess(y, x, w, span, degree = degree, parametric = parametric, : reciprocal condition number 1
```

```
## Warning in simpleLoess(y, x, w, span, degree = degree, parametric = parametric, : There are other near singularities as well. 1.01
```

```
## Warning in simpleLoess(y, x, w, span, degree = degree, parametric = parametric, : zero-width neighborhood. make span bigger
```

```
## Warning: Computation failed in `stat_smooth()`:
## NA/NaN/Inf in foreign function call (arg 5)
```

```
## Warning in simpleLoess(y, x, w, span, degree = degree, parametric = parametric, : at -0.005
```

```
## Warning in simpleLoess(y, x, w, span, degree = degree, parametric = parametric, : radius 2.5e-05
```

```
## Warning in simpleLoess(y, x, w, span, degree = degree, parametric = parametric, : all data on boundary of neighborhood. make span bigger
```

```
## Warning in simpleLoess(y, x, w, span, degree = degree, parametric = parametric, : pseudoinverse used at -0.005
```

```
## Warning in simpleLoess(y, x, w, span, degree = degree, parametric = parametric, : neighborhood radius 0.005
```

```
## Warning in simpleLoess(y, x, w, span, degree = degree, parametric = parametric, : reciprocal condition number 1
```

```
## Warning in simpleLoess(y, x, w, span, degree = degree, parametric = parametric, : There are other near singularities as well. 1.01
```

```
## Warning in simpleLoess(y, x, w, span, degree = degree, parametric = parametric, : zero-width neighborhood. make span bigger
```

```
## Warning: Computation failed in `stat_smooth()`:
## NA/NaN/Inf in foreign function call (arg 5)
```

```
## Warning in simpleLoess(y, x, w, span, degree = degree, parametric = parametric, : at -0.005
```

```
## Warning in simpleLoess(y, x, w, span, degree = degree, parametric = parametric, : radius 2.5e-05
```

```
## Warning in simpleLoess(y, x, w, span, degree = degree, parametric = parametric, : all data on boundary of neighborhood. make span bigger
```

```
## Warning in simpleLoess(y, x, w, span, degree = degree, parametric = parametric, : pseudoinverse used at -0.005
```

```
## Warning in simpleLoess(y, x, w, span, degree = degree, parametric = parametric, : neighborhood radius 0.005
```

```
## Warning in simpleLoess(y, x, w, span, degree = degree, parametric = parametric, : reciprocal condition number 1
```

```
## Warning in simpleLoess(y, x, w, span, degree = degree, parametric = parametric, : There are other near singularities as well. 1.01
```

```
## Warning in simpleLoess(y, x, w, span, degree = degree, parametric = parametric, : zero-width neighborhood. make span bigger
```

```
## Warning: Computation failed in `stat_smooth()`:
## NA/NaN/Inf in foreign function call (arg 5)
```

```
## Warning in simpleLoess(y, x, w, span, degree = degree, parametric = parametric, : at -0.005
```

```
## Warning in simpleLoess(y, x, w, span, degree = degree, parametric = parametric, : radius 2.5e-05
```

```
## Warning in simpleLoess(y, x, w, span, degree = degree, parametric = parametric, : all data on boundary of neighborhood. make span bigger
```

```
## Warning in simpleLoess(y, x, w, span, degree = degree, parametric = parametric, : pseudoinverse used at -0.005
```

```
## Warning in simpleLoess(y, x, w, span, degree = degree, parametric = parametric, : neighborhood radius 0.005
```

```
## Warning in simpleLoess(y, x, w, span, degree = degree, parametric = parametric, : reciprocal condition number 1
```

```
## Warning in simpleLoess(y, x, w, span, degree = degree, parametric = parametric, : There are other near singularities as well. 1.01
```

```
## Warning in simpleLoess(y, x, w, span, degree = degree, parametric = parametric, : zero-width neighborhood. make span bigger
```

```
## Warning: Computation failed in `stat_smooth()`:
## NA/NaN/Inf in foreign function call (arg 5)
```

```
## Warning in simpleLoess(y, x, w, span, degree = degree, parametric = parametric, : at -0.005
```

```
## Warning in simpleLoess(y, x, w, span, degree = degree, parametric = parametric, : radius 2.5e-05
```

```
## Warning in simpleLoess(y, x, w, span, degree = degree, parametric = parametric, : all data on boundary of neighborhood. make span bigger
```

```
## Warning in simpleLoess(y, x, w, span, degree = degree, parametric = parametric, : pseudoinverse used at -0.005
```

```
## Warning in simpleLoess(y, x, w, span, degree = degree, parametric = parametric, : neighborhood radius 0.005
```

```
## Warning in simpleLoess(y, x, w, span, degree = degree, parametric = parametric, : reciprocal condition number 1
```

```
## Warning in simpleLoess(y, x, w, span, degree = degree, parametric = parametric, : There are other near singularities as well. 1.01
```

```
## Warning in simpleLoess(y, x, w, span, degree = degree, parametric = parametric, : zero-width neighborhood. make span bigger
```

```
## Warning: Computation failed in `stat_smooth()`:
## NA/NaN/Inf in foreign function call (arg 5)
```

```
## Warning in simpleLoess(y, x, w, span, degree = degree, parametric = parametric, : at -0.005
```

```
## Warning in simpleLoess(y, x, w, span, degree = degree, parametric = parametric, : radius 2.5e-05
```

```
## Warning in simpleLoess(y, x, w, span, degree = degree, parametric = parametric, : all data on boundary of neighborhood. make span bigger
```

```
## Warning in simpleLoess(y, x, w, span, degree = degree, parametric = parametric, : pseudoinverse used at -0.005
```

```
## Warning in simpleLoess(y, x, w, span, degree = degree, parametric = parametric, : neighborhood radius 0.005
```

```
## Warning in simpleLoess(y, x, w, span, degree = degree, parametric = parametric, : reciprocal condition number 1
```

```
## Warning in simpleLoess(y, x, w, span, degree = degree, parametric = parametric, : There are other near singularities as well. 1.01
```

```
## Warning in simpleLoess(y, x, w, span, degree = degree, parametric = parametric, : zero-width neighborhood. make span bigger
```

```
## Warning: Computation failed in `stat_smooth()`:
## NA/NaN/Inf in foreign function call (arg 5)
```

```
## Warning in simpleLoess(y, x, w, span, degree = degree, parametric = parametric, : at -0.005
```

```
## Warning in simpleLoess(y, x, w, span, degree = degree, parametric = parametric, : radius 2.5e-05
```

```
## Warning in simpleLoess(y, x, w, span, degree = degree, parametric = parametric, : all data on boundary of neighborhood. make span bigger
```

```
## Warning in simpleLoess(y, x, w, span, degree = degree, parametric = parametric, : pseudoinverse used at -0.005
```

```
## Warning in simpleLoess(y, x, w, span, degree = degree, parametric = parametric, : neighborhood radius 0.005
```

```
## Warning in simpleLoess(y, x, w, span, degree = degree, parametric = parametric, : reciprocal condition number 1
```

```
## Warning in simpleLoess(y, x, w, span, degree = degree, parametric = parametric, : There are other near singularities as well. 1.01
```

```
## Warning in simpleLoess(y, x, w, span, degree = degree, parametric = parametric, : zero-width neighborhood. make span bigger
```

```
## Warning: Computation failed in `stat_smooth()`:
## NA/NaN/Inf in foreign function call (arg 5)
```

```
## Warning in simpleLoess(y, x, w, span, degree = degree, parametric = parametric, : at -0.005
```

```
## Warning in simpleLoess(y, x, w, span, degree = degree, parametric = parametric, : radius 2.5e-05
```

```
## Warning in simpleLoess(y, x, w, span, degree = degree, parametric = parametric, : all data on boundary of neighborhood. make span bigger
```

```
## Warning in simpleLoess(y, x, w, span, degree = degree, parametric = parametric, : pseudoinverse used at -0.005
```

```
## Warning in simpleLoess(y, x, w, span, degree = degree, parametric = parametric, : neighborhood radius 0.005
```

```
## Warning in simpleLoess(y, x, w, span, degree = degree, parametric = parametric, : reciprocal condition number 1
```

```
## Warning in simpleLoess(y, x, w, span, degree = degree, parametric = parametric, : There are other near singularities as well. 1.01
```

```
## Warning in simpleLoess(y, x, w, span, degree = degree, parametric = parametric, : zero-width neighborhood. make span bigger
```

```
## Warning: Computation failed in `stat_smooth()`:
## NA/NaN/Inf in foreign function call (arg 5)
```

```
## Warning in simpleLoess(y, x, w, span, degree = degree, parametric = parametric, : at -0.005
```

```
## Warning in simpleLoess(y, x, w, span, degree = degree, parametric = parametric, : radius 2.5e-05
```

```
## Warning in simpleLoess(y, x, w, span, degree = degree, parametric = parametric, : all data on boundary of neighborhood. make span bigger
```

```
## Warning in simpleLoess(y, x, w, span, degree = degree, parametric = parametric, : pseudoinverse used at -0.005
```

```
## Warning in simpleLoess(y, x, w, span, degree = degree, parametric = parametric, : neighborhood radius 0.005
```

```
## Warning in simpleLoess(y, x, w, span, degree = degree, parametric = parametric, : reciprocal condition number 1
```

```
## Warning in simpleLoess(y, x, w, span, degree = degree, parametric = parametric, : There are other near singularities as well. 1.01
```

```
## Warning in simpleLoess(y, x, w, span, degree = degree, parametric = parametric, : zero-width neighborhood. make span bigger
```

```
## Warning: Computation failed in `stat_smooth()`:
## NA/NaN/Inf in foreign function call (arg 5)
```

```
## Warning in simpleLoess(y, x, w, span, degree = degree, parametric = parametric, : at -0.005
```

```
## Warning in simpleLoess(y, x, w, span, degree = degree, parametric = parametric, : radius 2.5e-05
```

```
## Warning in simpleLoess(y, x, w, span, degree = degree, parametric = parametric, : all data on boundary of neighborhood. make span bigger
```

```
## Warning in simpleLoess(y, x, w, span, degree = degree, parametric = parametric, : pseudoinverse used at -0.005
```

```
## Warning in simpleLoess(y, x, w, span, degree = degree, parametric = parametric, : neighborhood radius 0.005
```

```
## Warning in simpleLoess(y, x, w, span, degree = degree, parametric = parametric, : reciprocal condition number 1
```

```
## Warning in simpleLoess(y, x, w, span, degree = degree, parametric = parametric, : There are other near singularities as well. 1.01
```

```
## Warning in simpleLoess(y, x, w, span, degree = degree, parametric = parametric, : zero-width neighborhood. make span bigger
```

```
## Warning: Computation failed in `stat_smooth()`:
## NA/NaN/Inf in foreign function call (arg 5)
```

```
## Warning in simpleLoess(y, x, w, span, degree = degree, parametric = parametric, : at -0.005
```

```
## Warning in simpleLoess(y, x, w, span, degree = degree, parametric = parametric, : radius 2.5e-05
```

```
## Warning in simpleLoess(y, x, w, span, degree = degree, parametric = parametric, : all data on boundary of neighborhood. make span bigger
```

```
## Warning in simpleLoess(y, x, w, span, degree = degree, parametric = parametric, : pseudoinverse used at -0.005
```

```
## Warning in simpleLoess(y, x, w, span, degree = degree, parametric = parametric, : neighborhood radius 0.005
```

```
## Warning in simpleLoess(y, x, w, span, degree = degree, parametric = parametric, : reciprocal condition number 1
```

```
## Warning in simpleLoess(y, x, w, span, degree = degree, parametric = parametric, : There are other near singularities as well. 1.01
```

```
## Warning in simpleLoess(y, x, w, span, degree = degree, parametric = parametric, : zero-width neighborhood. make span bigger
```

```
## Warning: Computation failed in `stat_smooth()`:
## NA/NaN/Inf in foreign function call (arg 5)
```

```
## Warning in simpleLoess(y, x, w, span, degree = degree, parametric = parametric, : at -0.005
```

```
## Warning in simpleLoess(y, x, w, span, degree = degree, parametric = parametric, : radius 2.5e-05
```

```
## Warning in simpleLoess(y, x, w, span, degree = degree, parametric = parametric, : all data on boundary of neighborhood. make span bigger
```

```
## Warning in simpleLoess(y, x, w, span, degree = degree, parametric = parametric, : pseudoinverse used at -0.005
```

```
## Warning in simpleLoess(y, x, w, span, degree = degree, parametric = parametric, : neighborhood radius 0.005
```

```
## Warning in simpleLoess(y, x, w, span, degree = degree, parametric = parametric, : reciprocal condition number 1
```

```
## Warning in simpleLoess(y, x, w, span, degree = degree, parametric = parametric, : There are other near singularities as well. 1.01
```

```
## Warning in simpleLoess(y, x, w, span, degree = degree, parametric = parametric, : zero-width neighborhood. make span bigger
```

```
## Warning: Computation failed in `stat_smooth()`:
## NA/NaN/Inf in foreign function call (arg 5)
```

```
## Warning in simpleLoess(y, x, w, span, degree = degree, parametric = parametric, : at -0.005
```

```
## Warning in simpleLoess(y, x, w, span, degree = degree, parametric = parametric, : radius 2.5e-05
```

```
## Warning in simpleLoess(y, x, w, span, degree = degree, parametric = parametric, : all data on boundary of neighborhood. make span bigger
```

```
## Warning in simpleLoess(y, x, w, span, degree = degree, parametric = parametric, : pseudoinverse used at -0.005
```

```
## Warning in simpleLoess(y, x, w, span, degree = degree, parametric = parametric, : neighborhood radius 0.005
```

```
## Warning in simpleLoess(y, x, w, span, degree = degree, parametric = parametric, : reciprocal condition number 1
```

```
## Warning in simpleLoess(y, x, w, span, degree = degree, parametric = parametric, : There are other near singularities as well. 1.01
```

```
## Warning in simpleLoess(y, x, w, span, degree = degree, parametric = parametric, : zero-width neighborhood. make span bigger
```

```
## Warning: Computation failed in `stat_smooth()`:
## NA/NaN/Inf in foreign function call (arg 5)
```

```
## Warning in simpleLoess(y, x, w, span, degree = degree, parametric = parametric, : at -0.005
```

```
## Warning in simpleLoess(y, x, w, span, degree = degree, parametric = parametric, : radius 2.5e-05
```

```
## Warning in simpleLoess(y, x, w, span, degree = degree, parametric = parametric, : all data on boundary of neighborhood. make span bigger
```

```
## Warning in simpleLoess(y, x, w, span, degree = degree, parametric = parametric, : pseudoinverse used at -0.005
```

```
## Warning in simpleLoess(y, x, w, span, degree = degree, parametric = parametric, : neighborhood radius 0.005
```

```
## Warning in simpleLoess(y, x, w, span, degree = degree, parametric = parametric, : reciprocal condition number 1
```

```
## Warning in simpleLoess(y, x, w, span, degree = degree, parametric = parametric, : There are other near singularities as well. 1.01
```

```
## Warning in simpleLoess(y, x, w, span, degree = degree, parametric = parametric, : zero-width neighborhood. make span bigger
```

```
## Warning: Computation failed in `stat_smooth()`:
## NA/NaN/Inf in foreign function call (arg 5)
```

```
## Warning in simpleLoess(y, x, w, span, degree = degree, parametric = parametric, : at -0.005
```

```
## Warning in simpleLoess(y, x, w, span, degree = degree, parametric = parametric, : radius 2.5e-05
```

```
## Warning in simpleLoess(y, x, w, span, degree = degree, parametric = parametric, : all data on boundary of neighborhood. make span bigger
```

```
## Warning in simpleLoess(y, x, w, span, degree = degree, parametric = parametric, : pseudoinverse used at -0.005
```

```
## Warning in simpleLoess(y, x, w, span, degree = degree, parametric = parametric, : neighborhood radius 0.005
```

```
## Warning in simpleLoess(y, x, w, span, degree = degree, parametric = parametric, : reciprocal condition number 1
```

```
## Warning in simpleLoess(y, x, w, span, degree = degree, parametric = parametric, : There are other near singularities as well. 1.01
```

```
## Warning in simpleLoess(y, x, w, span, degree = degree, parametric = parametric, : zero-width neighborhood. make span bigger
```

```
## Warning: Computation failed in `stat_smooth()`:
## NA/NaN/Inf in foreign function call (arg 5)
```

```
## Warning in simpleLoess(y, x, w, span, degree = degree, parametric = parametric, : at -0.005
```

```
## Warning in simpleLoess(y, x, w, span, degree = degree, parametric = parametric, : radius 2.5e-05
```

```
## Warning in simpleLoess(y, x, w, span, degree = degree, parametric = parametric, : all data on boundary of neighborhood. make span bigger
```

```
## Warning in simpleLoess(y, x, w, span, degree = degree, parametric = parametric, : pseudoinverse used at -0.005
```

```
## Warning in simpleLoess(y, x, w, span, degree = degree, parametric = parametric, : neighborhood radius 0.005
```

```
## Warning in simpleLoess(y, x, w, span, degree = degree, parametric = parametric, : reciprocal condition number 1
```

```
## Warning in simpleLoess(y, x, w, span, degree = degree, parametric = parametric, : There are other near singularities as well. 1.01
```

```
## Warning in simpleLoess(y, x, w, span, degree = degree, parametric = parametric, : zero-width neighborhood. make span bigger
```

```
## Warning: Computation failed in `stat_smooth()`:
## NA/NaN/Inf in foreign function call (arg 5)
```

```
## Warning in simpleLoess(y, x, w, span, degree = degree, parametric = parametric, : pseudoinverse used at -0.005
```

```
## Warning in simpleLoess(y, x, w, span, degree = degree, parametric = parametric, : neighborhood radius 1.005
```

```
## Warning in simpleLoess(y, x, w, span, degree = degree, parametric = parametric, : reciprocal condition number 0
```

```
## Warning in simpleLoess(y, x, w, span, degree = degree, parametric = parametric, : There are other near singularities as well. 1.01
```

```
## Warning in predLoess(object$y, object$x, newx = if (is.null(newdata)) object$x else if (is.data.frame(newdata)) as.matrix(model.frame(delete.response(terms(object)), : pseudoinverse used at -0.005
```

```
## Warning in predLoess(object$y, object$x, newx = if (is.null(newdata)) object$x else if (is.data.frame(newdata)) as.matrix(model.frame(delete.response(terms(object)), : neighborhood radius 1.005
```

```
## Warning in predLoess(object$y, object$x, newx = if (is.null(newdata)) object$x else if (is.data.frame(newdata)) as.matrix(model.frame(delete.response(terms(object)), : reciprocal condition number 0
```

```
## Warning in predLoess(object$y, object$x, newx = if (is.null(newdata)) object$x else if (is.data.frame(newdata)) as.matrix(model.frame(delete.response(terms(object)), : There are other near
## singularities as well. 1.01
```

```
## Warning in simpleLoess(y, x, w, span, degree = degree, parametric = parametric, : pseudoinverse used at -0.005
```

```
## Warning in simpleLoess(y, x, w, span, degree = degree, parametric = parametric, : neighborhood radius 1.005
```

```
## Warning in simpleLoess(y, x, w, span, degree = degree, parametric = parametric, : reciprocal condition number 0
```

```
## Warning in simpleLoess(y, x, w, span, degree = degree, parametric = parametric, : There are other near singularities as well. 1.01
```

```
## Warning in predLoess(object$y, object$x, newx = if (is.null(newdata)) object$x else if (is.data.frame(newdata)) as.matrix(model.frame(delete.response(terms(object)), : pseudoinverse used at -0.005
```

```
## Warning in predLoess(object$y, object$x, newx = if (is.null(newdata)) object$x else if (is.data.frame(newdata)) as.matrix(model.frame(delete.response(terms(object)), : neighborhood radius 1.005
```

```
## Warning in predLoess(object$y, object$x, newx = if (is.null(newdata)) object$x else if (is.data.frame(newdata)) as.matrix(model.frame(delete.response(terms(object)), : reciprocal condition number 0
```

```
## Warning in predLoess(object$y, object$x, newx = if (is.null(newdata)) object$x else if (is.data.frame(newdata)) as.matrix(model.frame(delete.response(terms(object)), : There are other near
## singularities as well. 1.01
```

```
## Warning in simpleLoess(y, x, w, span, degree = degree, parametric = parametric, : at -0.005
```

```
## Warning in simpleLoess(y, x, w, span, degree = degree, parametric = parametric, : radius 2.5e-05
```

```
## Warning in simpleLoess(y, x, w, span, degree = degree, parametric = parametric, : all data on boundary of neighborhood. make span bigger
```

```
## Warning in simpleLoess(y, x, w, span, degree = degree, parametric = parametric, : pseudoinverse used at -0.005
```

```
## Warning in simpleLoess(y, x, w, span, degree = degree, parametric = parametric, : neighborhood radius 0.005
```

```
## Warning in simpleLoess(y, x, w, span, degree = degree, parametric = parametric, : reciprocal condition number 1
```

```
## Warning in simpleLoess(y, x, w, span, degree = degree, parametric = parametric, : There are other near singularities as well. 1.01
```

```
## Warning in simpleLoess(y, x, w, span, degree = degree, parametric = parametric, : zero-width neighborhood. make span bigger
```

```
## Warning: Computation failed in `stat_smooth()`:
## NA/NaN/Inf in foreign function call (arg 5)
```

```
## Warning in simpleLoess(y, x, w, span, degree = degree, parametric = parametric, : at -0.005
```

```
## Warning in simpleLoess(y, x, w, span, degree = degree, parametric = parametric, : radius 2.5e-05
```

```
## Warning in simpleLoess(y, x, w, span, degree = degree, parametric = parametric, : all data on boundary of neighborhood. make span bigger
```

```
## Warning in simpleLoess(y, x, w, span, degree = degree, parametric = parametric, : pseudoinverse used at -0.005
```

```
## Warning in simpleLoess(y, x, w, span, degree = degree, parametric = parametric, : neighborhood radius 0.005
```

```
## Warning in simpleLoess(y, x, w, span, degree = degree, parametric = parametric, : reciprocal condition number 1
```

```
## Warning in simpleLoess(y, x, w, span, degree = degree, parametric = parametric, : There are other near singularities as well. 1.01
```

```
## Warning in simpleLoess(y, x, w, span, degree = degree, parametric = parametric, : zero-width neighborhood. make span bigger
```

```
## Warning: Computation failed in `stat_smooth()`:
## NA/NaN/Inf in foreign function call (arg 5)
```

```
## Warning in simpleLoess(y, x, w, span, degree = degree, parametric = parametric, : pseudoinverse used at -0.005
```

```
## Warning in simpleLoess(y, x, w, span, degree = degree, parametric = parametric, : neighborhood radius 1.005
```

```
## Warning in simpleLoess(y, x, w, span, degree = degree, parametric = parametric, : reciprocal condition number 0
```

```
## Warning in simpleLoess(y, x, w, span, degree = degree, parametric = parametric, : There are other near singularities as well. 1.01
```

```
## Warning in predLoess(object$y, object$x, newx = if (is.null(newdata)) object$x else if (is.data.frame(newdata)) as.matrix(model.frame(delete.response(terms(object)), : pseudoinverse used at -0.005
```

```
## Warning in predLoess(object$y, object$x, newx = if (is.null(newdata)) object$x else if (is.data.frame(newdata)) as.matrix(model.frame(delete.response(terms(object)), : neighborhood radius 1.005
```

```
## Warning in predLoess(object$y, object$x, newx = if (is.null(newdata)) object$x else if (is.data.frame(newdata)) as.matrix(model.frame(delete.response(terms(object)), : reciprocal condition number 0
```

```
## Warning in predLoess(object$y, object$x, newx = if (is.null(newdata)) object$x else if (is.data.frame(newdata)) as.matrix(model.frame(delete.response(terms(object)), : There are other near
## singularities as well. 1.01
```

```
## Warning in simpleLoess(y, x, w, span, degree = degree, parametric = parametric, : at -0.005
```

```
## Warning in simpleLoess(y, x, w, span, degree = degree, parametric = parametric, : radius 2.5e-05
```

```
## Warning in simpleLoess(y, x, w, span, degree = degree, parametric = parametric, : all data on boundary of neighborhood. make span bigger
```

```
## Warning in simpleLoess(y, x, w, span, degree = degree, parametric = parametric, : pseudoinverse used at -0.005
```

```
## Warning in simpleLoess(y, x, w, span, degree = degree, parametric = parametric, : neighborhood radius 0.005
```

```
## Warning in simpleLoess(y, x, w, span, degree = degree, parametric = parametric, : reciprocal condition number 1
```

```
## Warning in simpleLoess(y, x, w, span, degree = degree, parametric = parametric, : There are other near singularities as well. 1.01
```

```
## Warning in simpleLoess(y, x, w, span, degree = degree, parametric = parametric, : zero-width neighborhood. make span bigger
```

```
## Warning: Computation failed in `stat_smooth()`:
## NA/NaN/Inf in foreign function call (arg 5)
```

```
## Warning in simpleLoess(y, x, w, span, degree = degree, parametric = parametric, : at -0.005
```

```
## Warning in simpleLoess(y, x, w, span, degree = degree, parametric = parametric, : radius 2.5e-05
```

```
## Warning in simpleLoess(y, x, w, span, degree = degree, parametric = parametric, : all data on boundary of neighborhood. make span bigger
```

```
## Warning in simpleLoess(y, x, w, span, degree = degree, parametric = parametric, : pseudoinverse used at -0.005
```

```
## Warning in simpleLoess(y, x, w, span, degree = degree, parametric = parametric, : neighborhood radius 0.005
```

```
## Warning in simpleLoess(y, x, w, span, degree = degree, parametric = parametric, : reciprocal condition number 1
```

```
## Warning in simpleLoess(y, x, w, span, degree = degree, parametric = parametric, : There are other near singularities as well. 1.01
```

```
## Warning in simpleLoess(y, x, w, span, degree = degree, parametric = parametric, : zero-width neighborhood. make span bigger
```

```
## Warning: Computation failed in `stat_smooth()`:
## NA/NaN/Inf in foreign function call (arg 5)
```

```
## Warning in simpleLoess(y, x, w, span, degree = degree, parametric = parametric, : at -0.005
```

```
## Warning in simpleLoess(y, x, w, span, degree = degree, parametric = parametric, : radius 2.5e-05
```

```
## Warning in simpleLoess(y, x, w, span, degree = degree, parametric = parametric, : all data on boundary of neighborhood. make span bigger
```

```
## Warning in simpleLoess(y, x, w, span, degree = degree, parametric = parametric, : pseudoinverse used at -0.005
```

```
## Warning in simpleLoess(y, x, w, span, degree = degree, parametric = parametric, : neighborhood radius 0.005
```

```
## Warning in simpleLoess(y, x, w, span, degree = degree, parametric = parametric, : reciprocal condition number 1
```

```
## Warning in simpleLoess(y, x, w, span, degree = degree, parametric = parametric, : There are other near singularities as well. 1.01
```

```
## Warning in simpleLoess(y, x, w, span, degree = degree, parametric = parametric, : zero-width neighborhood. make span bigger
```

```
## Warning: Computation failed in `stat_smooth()`:
## NA/NaN/Inf in foreign function call (arg 5)
```

```
## Warning in simpleLoess(y, x, w, span, degree = degree, parametric = parametric, : at -0.005
```

```
## Warning in simpleLoess(y, x, w, span, degree = degree, parametric = parametric, : radius 2.5e-05
```

```
## Warning in simpleLoess(y, x, w, span, degree = degree, parametric = parametric, : all data on boundary of neighborhood. make span bigger
```

```
## Warning in simpleLoess(y, x, w, span, degree = degree, parametric = parametric, : pseudoinverse used at -0.005
```

```
## Warning in simpleLoess(y, x, w, span, degree = degree, parametric = parametric, : neighborhood radius 0.005
```

```
## Warning in simpleLoess(y, x, w, span, degree = degree, parametric = parametric, : reciprocal condition number 1
```

```
## Warning in simpleLoess(y, x, w, span, degree = degree, parametric = parametric, : There are other near singularities as well. 1.01
```

```
## Warning in simpleLoess(y, x, w, span, degree = degree, parametric = parametric, : zero-width neighborhood. make span bigger
```

```
## Warning: Computation failed in `stat_smooth()`:
## NA/NaN/Inf in foreign function call (arg 5)
```

```
## Warning in simpleLoess(y, x, w, span, degree = degree, parametric = parametric, : at -0.005
```

```
## Warning in simpleLoess(y, x, w, span, degree = degree, parametric = parametric, : radius 2.5e-05
```

```
## Warning in simpleLoess(y, x, w, span, degree = degree, parametric = parametric, : all data on boundary of neighborhood. make span bigger
```

```
## Warning in simpleLoess(y, x, w, span, degree = degree, parametric = parametric, : pseudoinverse used at -0.005
```

```
## Warning in simpleLoess(y, x, w, span, degree = degree, parametric = parametric, : neighborhood radius 0.005
```

```
## Warning in simpleLoess(y, x, w, span, degree = degree, parametric = parametric, : reciprocal condition number 1
```

```
## Warning in simpleLoess(y, x, w, span, degree = degree, parametric = parametric, : There are other near singularities as well. 1.01
```

```
## Warning in simpleLoess(y, x, w, span, degree = degree, parametric = parametric, : zero-width neighborhood. make span bigger
```

```
## Warning: Computation failed in `stat_smooth()`:
## NA/NaN/Inf in foreign function call (arg 5)
```

```
## Warning in simpleLoess(y, x, w, span, degree = degree, parametric = parametric, : at -0.005
```

```
## Warning in simpleLoess(y, x, w, span, degree = degree, parametric = parametric, : radius 2.5e-05
```

```
## Warning in simpleLoess(y, x, w, span, degree = degree, parametric = parametric, : all data on boundary of neighborhood. make span bigger
```

```
## Warning in simpleLoess(y, x, w, span, degree = degree, parametric = parametric, : pseudoinverse used at -0.005
```

```
## Warning in simpleLoess(y, x, w, span, degree = degree, parametric = parametric, : neighborhood radius 0.005
```

```
## Warning in simpleLoess(y, x, w, span, degree = degree, parametric = parametric, : reciprocal condition number 1
```

```
## Warning in simpleLoess(y, x, w, span, degree = degree, parametric = parametric, : There are other near singularities as well. 1.01
```

```
## Warning in simpleLoess(y, x, w, span, degree = degree, parametric = parametric, : zero-width neighborhood. make span bigger
```

```
## Warning: Computation failed in `stat_smooth()`:
## NA/NaN/Inf in foreign function call (arg 5)
```

```
## Warning in simpleLoess(y, x, w, span, degree = degree, parametric = parametric, : at -0.005
```

```
## Warning in simpleLoess(y, x, w, span, degree = degree, parametric = parametric, : radius 2.5e-05
```

```
## Warning in simpleLoess(y, x, w, span, degree = degree, parametric = parametric, : all data on boundary of neighborhood. make span bigger
```

```
## Warning in simpleLoess(y, x, w, span, degree = degree, parametric = parametric, : pseudoinverse used at -0.005
```

```
## Warning in simpleLoess(y, x, w, span, degree = degree, parametric = parametric, : neighborhood radius 0.005
```

```
## Warning in simpleLoess(y, x, w, span, degree = degree, parametric = parametric, : reciprocal condition number 1
```

```
## Warning in simpleLoess(y, x, w, span, degree = degree, parametric = parametric, : There are other near singularities as well. 1.01
```

```
## Warning in simpleLoess(y, x, w, span, degree = degree, parametric = parametric, : zero-width neighborhood. make span bigger
```

```
## Warning: Computation failed in `stat_smooth()`:
## NA/NaN/Inf in foreign function call (arg 5)
```

```
## Warning in simpleLoess(y, x, w, span, degree = degree, parametric = parametric, : at -0.005
```

```
## Warning in simpleLoess(y, x, w, span, degree = degree, parametric = parametric, : radius 2.5e-05
```

```
## Warning in simpleLoess(y, x, w, span, degree = degree, parametric = parametric, : all data on boundary of neighborhood. make span bigger
```

```
## Warning in simpleLoess(y, x, w, span, degree = degree, parametric = parametric, : pseudoinverse used at -0.005
```

```
## Warning in simpleLoess(y, x, w, span, degree = degree, parametric = parametric, : neighborhood radius 0.005
```

```
## Warning in simpleLoess(y, x, w, span, degree = degree, parametric = parametric, : reciprocal condition number 1
```

```
## Warning in simpleLoess(y, x, w, span, degree = degree, parametric = parametric, : There are other near singularities as well. 1.01
```

```
## Warning in simpleLoess(y, x, w, span, degree = degree, parametric = parametric, : zero-width neighborhood. make span bigger
```

```
## Warning: Computation failed in `stat_smooth()`:
## NA/NaN/Inf in foreign function call (arg 5)
```

```
## Warning in simpleLoess(y, x, w, span, degree = degree, parametric = parametric, : at -0.005
```

```
## Warning in simpleLoess(y, x, w, span, degree = degree, parametric = parametric, : radius 2.5e-05
```

```
## Warning in simpleLoess(y, x, w, span, degree = degree, parametric = parametric, : all data on boundary of neighborhood. make span bigger
```

```
## Warning in simpleLoess(y, x, w, span, degree = degree, parametric = parametric, : pseudoinverse used at -0.005
```

```
## Warning in simpleLoess(y, x, w, span, degree = degree, parametric = parametric, : neighborhood radius 0.005
```

```
## Warning in simpleLoess(y, x, w, span, degree = degree, parametric = parametric, : reciprocal condition number 1
```

```
## Warning in simpleLoess(y, x, w, span, degree = degree, parametric = parametric, : There are other near singularities as well. 1.01
```

```
## Warning in simpleLoess(y, x, w, span, degree = degree, parametric = parametric, : zero-width neighborhood. make span bigger
```

```
## Warning: Computation failed in `stat_smooth()`:
## NA/NaN/Inf in foreign function call (arg 5)
```

```
## Warning in simpleLoess(y, x, w, span, degree = degree, parametric = parametric, : pseudoinverse used at -0.005
```

```
## Warning in simpleLoess(y, x, w, span, degree = degree, parametric = parametric, : neighborhood radius 1.005
```

```
## Warning in simpleLoess(y, x, w, span, degree = degree, parametric = parametric, : reciprocal condition number 0
```

```
## Warning in simpleLoess(y, x, w, span, degree = degree, parametric = parametric, : There are other near singularities as well. 1.01
```

```
## Warning in predLoess(object$y, object$x, newx = if (is.null(newdata)) object$x else if (is.data.frame(newdata)) as.matrix(model.frame(delete.response(terms(object)), : pseudoinverse used at -0.005
```

```
## Warning in predLoess(object$y, object$x, newx = if (is.null(newdata)) object$x else if (is.data.frame(newdata)) as.matrix(model.frame(delete.response(terms(object)), : neighborhood radius 1.005
```

```
## Warning in predLoess(object$y, object$x, newx = if (is.null(newdata)) object$x else if (is.data.frame(newdata)) as.matrix(model.frame(delete.response(terms(object)), : reciprocal condition number 0
```

```
## Warning in predLoess(object$y, object$x, newx = if (is.null(newdata)) object$x else if (is.data.frame(newdata)) as.matrix(model.frame(delete.response(terms(object)), : There are other near
## singularities as well. 1.01
```

```
## Warning in simpleLoess(y, x, w, span, degree = degree, parametric = parametric, : at -0.005
```

```
## Warning in simpleLoess(y, x, w, span, degree = degree, parametric = parametric, : radius 2.5e-05
```

```
## Warning in simpleLoess(y, x, w, span, degree = degree, parametric = parametric, : all data on boundary of neighborhood. make span bigger
```

```
## Warning in simpleLoess(y, x, w, span, degree = degree, parametric = parametric, : pseudoinverse used at -0.005
```

```
## Warning in simpleLoess(y, x, w, span, degree = degree, parametric = parametric, : neighborhood radius 0.005
```

```
## Warning in simpleLoess(y, x, w, span, degree = degree, parametric = parametric, : reciprocal condition number 1
```

```
## Warning in simpleLoess(y, x, w, span, degree = degree, parametric = parametric, : There are other near singularities as well. 1.01
```

```
## Warning in simpleLoess(y, x, w, span, degree = degree, parametric = parametric, : zero-width neighborhood. make span bigger
```

```
## Warning: Computation failed in `stat_smooth()`:
## NA/NaN/Inf in foreign function call (arg 5)
```

```
## Warning in simpleLoess(y, x, w, span, degree = degree, parametric = parametric, : at -0.005
```

```
## Warning in simpleLoess(y, x, w, span, degree = degree, parametric = parametric, : radius 2.5e-05
```

```
## Warning in simpleLoess(y, x, w, span, degree = degree, parametric = parametric, : all data on boundary of neighborhood. make span bigger
```

```
## Warning in simpleLoess(y, x, w, span, degree = degree, parametric = parametric, : pseudoinverse used at -0.005
```

```
## Warning in simpleLoess(y, x, w, span, degree = degree, parametric = parametric, : neighborhood radius 0.005
```

```
## Warning in simpleLoess(y, x, w, span, degree = degree, parametric = parametric, : reciprocal condition number 1
```

```
## Warning in simpleLoess(y, x, w, span, degree = degree, parametric = parametric, : There are other near singularities as well. 1.01
```

```
## Warning in simpleLoess(y, x, w, span, degree = degree, parametric = parametric, : zero-width neighborhood. make span bigger
```

```
## Warning: Computation failed in `stat_smooth()`:
## NA/NaN/Inf in foreign function call (arg 5)
```

```
## Warning in simpleLoess(y, x, w, span, degree = degree, parametric = parametric, : at -0.005
```

```
## Warning in simpleLoess(y, x, w, span, degree = degree, parametric = parametric, : radius 2.5e-05
```

```
## Warning in simpleLoess(y, x, w, span, degree = degree, parametric = parametric, : all data on boundary of neighborhood. make span bigger
```

```
## Warning in simpleLoess(y, x, w, span, degree = degree, parametric = parametric, : pseudoinverse used at -0.005
```

```
## Warning in simpleLoess(y, x, w, span, degree = degree, parametric = parametric, : neighborhood radius 0.005
```

```
## Warning in simpleLoess(y, x, w, span, degree = degree, parametric = parametric, : reciprocal condition number 1
```

```
## Warning in simpleLoess(y, x, w, span, degree = degree, parametric = parametric, : There are other near singularities as well. 1.01
```

```
## Warning in simpleLoess(y, x, w, span, degree = degree, parametric = parametric, : zero-width neighborhood. make span bigger
```

```
## Warning: Computation failed in `stat_smooth()`:
## NA/NaN/Inf in foreign function call (arg 5)
```

```
## Warning in simpleLoess(y, x, w, span, degree = degree, parametric = parametric, : at -0.005
```

```
## Warning in simpleLoess(y, x, w, span, degree = degree, parametric = parametric, : radius 2.5e-05
```

```
## Warning in simpleLoess(y, x, w, span, degree = degree, parametric = parametric, : all data on boundary of neighborhood. make span bigger
```

```
## Warning in simpleLoess(y, x, w, span, degree = degree, parametric = parametric, : pseudoinverse used at -0.005
```

```
## Warning in simpleLoess(y, x, w, span, degree = degree, parametric = parametric, : neighborhood radius 0.005
```

```
## Warning in simpleLoess(y, x, w, span, degree = degree, parametric = parametric, : reciprocal condition number 1
```

```
## Warning in simpleLoess(y, x, w, span, degree = degree, parametric = parametric, : There are other near singularities as well. 1.01
```

```
## Warning in simpleLoess(y, x, w, span, degree = degree, parametric = parametric, : zero-width neighborhood. make span bigger
```

```
## Warning: Computation failed in `stat_smooth()`:
## NA/NaN/Inf in foreign function call (arg 5)
```

```
## Warning in simpleLoess(y, x, w, span, degree = degree, parametric = parametric, : at -0.005
```

```
## Warning in simpleLoess(y, x, w, span, degree = degree, parametric = parametric, : radius 2.5e-05
```

```
## Warning in simpleLoess(y, x, w, span, degree = degree, parametric = parametric, : all data on boundary of neighborhood. make span bigger
```

```
## Warning in simpleLoess(y, x, w, span, degree = degree, parametric = parametric, : pseudoinverse used at -0.005
```

```
## Warning in simpleLoess(y, x, w, span, degree = degree, parametric = parametric, : neighborhood radius 0.005
```

```
## Warning in simpleLoess(y, x, w, span, degree = degree, parametric = parametric, : reciprocal condition number 1
```

```
## Warning in simpleLoess(y, x, w, span, degree = degree, parametric = parametric, : There are other near singularities as well. 1.01
```

```
## Warning in simpleLoess(y, x, w, span, degree = degree, parametric = parametric, : zero-width neighborhood. make span bigger
```

```
## Warning: Computation failed in `stat_smooth()`:
## NA/NaN/Inf in foreign function call (arg 5)
```

```
## Warning in simpleLoess(y, x, w, span, degree = degree, parametric = parametric, : pseudoinverse used at -0.005
```

```
## Warning in simpleLoess(y, x, w, span, degree = degree, parametric = parametric, : neighborhood radius 1.005
```

```
## Warning in simpleLoess(y, x, w, span, degree = degree, parametric = parametric, : reciprocal condition number 0
```

```
## Warning in simpleLoess(y, x, w, span, degree = degree, parametric = parametric, : There are other near singularities as well. 1.01
```

```
## Warning in predLoess(object$y, object$x, newx = if (is.null(newdata)) object$x else if (is.data.frame(newdata)) as.matrix(model.frame(delete.response(terms(object)), : pseudoinverse used at -0.005
```

```
## Warning in predLoess(object$y, object$x, newx = if (is.null(newdata)) object$x else if (is.data.frame(newdata)) as.matrix(model.frame(delete.response(terms(object)), : neighborhood radius 1.005
```

```
## Warning in predLoess(object$y, object$x, newx = if (is.null(newdata)) object$x else if (is.data.frame(newdata)) as.matrix(model.frame(delete.response(terms(object)), : reciprocal condition number 0
```

```
## Warning in predLoess(object$y, object$x, newx = if (is.null(newdata)) object$x else if (is.data.frame(newdata)) as.matrix(model.frame(delete.response(terms(object)), : There are other near
## singularities as well. 1.01
```

```
## Warning in simpleLoess(y, x, w, span, degree = degree, parametric = parametric, : at -0.005
```

```
## Warning in simpleLoess(y, x, w, span, degree = degree, parametric = parametric, : radius 2.5e-05
```

```
## Warning in simpleLoess(y, x, w, span, degree = degree, parametric = parametric, : all data on boundary of neighborhood. make span bigger
```

```
## Warning in simpleLoess(y, x, w, span, degree = degree, parametric = parametric, : pseudoinverse used at -0.005
```

```
## Warning in simpleLoess(y, x, w, span, degree = degree, parametric = parametric, : neighborhood radius 0.005
```

```
## Warning in simpleLoess(y, x, w, span, degree = degree, parametric = parametric, : reciprocal condition number 1
```

```
## Warning in simpleLoess(y, x, w, span, degree = degree, parametric = parametric, : There are other near singularities as well. 1.01
```

```
## Warning in simpleLoess(y, x, w, span, degree = degree, parametric = parametric, : zero-width neighborhood. make span bigger
```

```
## Warning: Computation failed in `stat_smooth()`:
## NA/NaN/Inf in foreign function call (arg 5)
```

```
## Warning in simpleLoess(y, x, w, span, degree = degree, parametric = parametric, : at -0.005
```

```
## Warning in simpleLoess(y, x, w, span, degree = degree, parametric = parametric, : radius 2.5e-05
```

```
## Warning in simpleLoess(y, x, w, span, degree = degree, parametric = parametric, : all data on boundary of neighborhood. make span bigger
```

```
## Warning in simpleLoess(y, x, w, span, degree = degree, parametric = parametric, : pseudoinverse used at -0.005
```

```
## Warning in simpleLoess(y, x, w, span, degree = degree, parametric = parametric, : neighborhood radius 0.005
```

```
## Warning in simpleLoess(y, x, w, span, degree = degree, parametric = parametric, : reciprocal condition number 1
```

```
## Warning in simpleLoess(y, x, w, span, degree = degree, parametric = parametric, : There are other near singularities as well. 1.01
```

```
## Warning in simpleLoess(y, x, w, span, degree = degree, parametric = parametric, : zero-width neighborhood. make span bigger
```

```
## Warning: Computation failed in `stat_smooth()`:
## NA/NaN/Inf in foreign function call (arg 5)
```

```
## Warning in simpleLoess(y, x, w, span, degree = degree, parametric = parametric, : at -0.005
```

```
## Warning in simpleLoess(y, x, w, span, degree = degree, parametric = parametric, : radius 2.5e-05
```

```
## Warning in simpleLoess(y, x, w, span, degree = degree, parametric = parametric, : all data on boundary of neighborhood. make span bigger
```

```
## Warning in simpleLoess(y, x, w, span, degree = degree, parametric = parametric, : pseudoinverse used at -0.005
```

```
## Warning in simpleLoess(y, x, w, span, degree = degree, parametric = parametric, : neighborhood radius 0.005
```

```
## Warning in simpleLoess(y, x, w, span, degree = degree, parametric = parametric, : reciprocal condition number 1
```

```
## Warning in simpleLoess(y, x, w, span, degree = degree, parametric = parametric, : There are other near singularities as well. 1.01
```

```
## Warning in simpleLoess(y, x, w, span, degree = degree, parametric = parametric, : zero-width neighborhood. make span bigger
```

```
## Warning: Computation failed in `stat_smooth()`:
## NA/NaN/Inf in foreign function call (arg 5)
```

```
## Warning in simpleLoess(y, x, w, span, degree = degree, parametric = parametric, : at -0.005
```

```
## Warning in simpleLoess(y, x, w, span, degree = degree, parametric = parametric, : radius 2.5e-05
```

```
## Warning in simpleLoess(y, x, w, span, degree = degree, parametric = parametric, : all data on boundary of neighborhood. make span bigger
```

```
## Warning in simpleLoess(y, x, w, span, degree = degree, parametric = parametric, : pseudoinverse used at -0.005
```

```
## Warning in simpleLoess(y, x, w, span, degree = degree, parametric = parametric, : neighborhood radius 0.005
```

```
## Warning in simpleLoess(y, x, w, span, degree = degree, parametric = parametric, : reciprocal condition number 1
```

```
## Warning in simpleLoess(y, x, w, span, degree = degree, parametric = parametric, : There are other near singularities as well. 1.01
```

```
## Warning in simpleLoess(y, x, w, span, degree = degree, parametric = parametric, : zero-width neighborhood. make span bigger
```

```
## Warning: Computation failed in `stat_smooth()`:
## NA/NaN/Inf in foreign function call (arg 5)
```

```
## Warning in simpleLoess(y, x, w, span, degree = degree, parametric = parametric, : pseudoinverse used at -0.005
```

```
## Warning in simpleLoess(y, x, w, span, degree = degree, parametric = parametric, : neighborhood radius 1.005
```

```
## Warning in simpleLoess(y, x, w, span, degree = degree, parametric = parametric, : reciprocal condition number 0
```

```
## Warning in simpleLoess(y, x, w, span, degree = degree, parametric = parametric, : There are other near singularities as well. 1.01
```

```
## Warning in predLoess(object$y, object$x, newx = if (is.null(newdata)) object$x else if (is.data.frame(newdata)) as.matrix(model.frame(delete.response(terms(object)), : pseudoinverse used at -0.005
```

```
## Warning in predLoess(object$y, object$x, newx = if (is.null(newdata)) object$x else if (is.data.frame(newdata)) as.matrix(model.frame(delete.response(terms(object)), : neighborhood radius 1.005
```

```
## Warning in predLoess(object$y, object$x, newx = if (is.null(newdata)) object$x else if (is.data.frame(newdata)) as.matrix(model.frame(delete.response(terms(object)), : reciprocal condition number 0
```

```
## Warning in predLoess(object$y, object$x, newx = if (is.null(newdata)) object$x else if (is.data.frame(newdata)) as.matrix(model.frame(delete.response(terms(object)), : There are other near
## singularities as well. 1.01
```

```
## Warning in simpleLoess(y, x, w, span, degree = degree, parametric = parametric, : pseudoinverse used at -0.005
```

```
## Warning in simpleLoess(y, x, w, span, degree = degree, parametric = parametric, : neighborhood radius 1.005
```

```
## Warning in simpleLoess(y, x, w, span, degree = degree, parametric = parametric, : reciprocal condition number 0
```

```
## Warning in simpleLoess(y, x, w, span, degree = degree, parametric = parametric, : There are other near singularities as well. 1.01
```

```
## Warning in predLoess(object$y, object$x, newx = if (is.null(newdata)) object$x else if (is.data.frame(newdata)) as.matrix(model.frame(delete.response(terms(object)), : pseudoinverse used at -0.005
```

```
## Warning in predLoess(object$y, object$x, newx = if (is.null(newdata)) object$x else if (is.data.frame(newdata)) as.matrix(model.frame(delete.response(terms(object)), : neighborhood radius 1.005
```

```
## Warning in predLoess(object$y, object$x, newx = if (is.null(newdata)) object$x else if (is.data.frame(newdata)) as.matrix(model.frame(delete.response(terms(object)), : reciprocal condition number 0
```

```
## Warning in predLoess(object$y, object$x, newx = if (is.null(newdata)) object$x else if (is.data.frame(newdata)) as.matrix(model.frame(delete.response(terms(object)), : There are other near
## singularities as well. 1.01
```

```
## Warning in simpleLoess(y, x, w, span, degree = degree, parametric = parametric, : at -0.005
```

```
## Warning in simpleLoess(y, x, w, span, degree = degree, parametric = parametric, : radius 2.5e-05
```

```
## Warning in simpleLoess(y, x, w, span, degree = degree, parametric = parametric, : all data on boundary of neighborhood. make span bigger
```

```
## Warning in simpleLoess(y, x, w, span, degree = degree, parametric = parametric, : pseudoinverse used at -0.005
```

```
## Warning in simpleLoess(y, x, w, span, degree = degree, parametric = parametric, : neighborhood radius 0.005
```

```
## Warning in simpleLoess(y, x, w, span, degree = degree, parametric = parametric, : reciprocal condition number 1
```

```
## Warning in simpleLoess(y, x, w, span, degree = degree, parametric = parametric, : There are other near singularities as well. 1.01
```

```
## Warning in simpleLoess(y, x, w, span, degree = degree, parametric = parametric, : zero-width neighborhood. make span bigger
```

```
## Warning: Computation failed in `stat_smooth()`:
## NA/NaN/Inf in foreign function call (arg 5)
```

```
## Warning in simpleLoess(y, x, w, span, degree = degree, parametric = parametric, : at -0.005
```

```
## Warning in simpleLoess(y, x, w, span, degree = degree, parametric = parametric, : radius 2.5e-05
```

```
## Warning in simpleLoess(y, x, w, span, degree = degree, parametric = parametric, : all data on boundary of neighborhood. make span bigger
```

```
## Warning in simpleLoess(y, x, w, span, degree = degree, parametric = parametric, : pseudoinverse used at -0.005
```

```
## Warning in simpleLoess(y, x, w, span, degree = degree, parametric = parametric, : neighborhood radius 0.005
```

```
## Warning in simpleLoess(y, x, w, span, degree = degree, parametric = parametric, : reciprocal condition number 1
```

```
## Warning in simpleLoess(y, x, w, span, degree = degree, parametric = parametric, : There are other near singularities as well. 1.01
```

```
## Warning in simpleLoess(y, x, w, span, degree = degree, parametric = parametric, : zero-width neighborhood. make span bigger
```

```
## Warning: Computation failed in `stat_smooth()`:
## NA/NaN/Inf in foreign function call (arg 5)
```

```
## Warning in simpleLoess(y, x, w, span, degree = degree, parametric = parametric, : at -0.005
```

```
## Warning in simpleLoess(y, x, w, span, degree = degree, parametric = parametric, : radius 2.5e-05
```

```
## Warning in simpleLoess(y, x, w, span, degree = degree, parametric = parametric, : all data on boundary of neighborhood. make span bigger
```

```
## Warning in simpleLoess(y, x, w, span, degree = degree, parametric = parametric, : pseudoinverse used at -0.005
```

```
## Warning in simpleLoess(y, x, w, span, degree = degree, parametric = parametric, : neighborhood radius 0.005
```

```
## Warning in simpleLoess(y, x, w, span, degree = degree, parametric = parametric, : reciprocal condition number 1
```

```
## Warning in simpleLoess(y, x, w, span, degree = degree, parametric = parametric, : There are other near singularities as well. 1.01
```

```
## Warning in simpleLoess(y, x, w, span, degree = degree, parametric = parametric, : zero-width neighborhood. make span bigger
```

```
## Warning: Computation failed in `stat_smooth()`:
## NA/NaN/Inf in foreign function call (arg 5)
```

```
## Warning in simpleLoess(y, x, w, span, degree = degree, parametric = parametric, : at -0.005
```

```
## Warning in simpleLoess(y, x, w, span, degree = degree, parametric = parametric, : radius 2.5e-05
```

```
## Warning in simpleLoess(y, x, w, span, degree = degree, parametric = parametric, : all data on boundary of neighborhood. make span bigger
```

```
## Warning in simpleLoess(y, x, w, span, degree = degree, parametric = parametric, : pseudoinverse used at -0.005
```

```
## Warning in simpleLoess(y, x, w, span, degree = degree, parametric = parametric, : neighborhood radius 0.005
```

```
## Warning in simpleLoess(y, x, w, span, degree = degree, parametric = parametric, : reciprocal condition number 1
```

```
## Warning in simpleLoess(y, x, w, span, degree = degree, parametric = parametric, : There are other near singularities as well. 1.01
```

```
## Warning in simpleLoess(y, x, w, span, degree = degree, parametric = parametric, : zero-width neighborhood. make span bigger
```

```
## Warning: Computation failed in `stat_smooth()`:
## NA/NaN/Inf in foreign function call (arg 5)
```

```
## Warning in simpleLoess(y, x, w, span, degree = degree, parametric = parametric, : at -0.005
```

```
## Warning in simpleLoess(y, x, w, span, degree = degree, parametric = parametric, : radius 2.5e-05
```

```
## Warning in simpleLoess(y, x, w, span, degree = degree, parametric = parametric, : all data on boundary of neighborhood. make span bigger
```

```
## Warning in simpleLoess(y, x, w, span, degree = degree, parametric = parametric, : pseudoinverse used at -0.005
```

```
## Warning in simpleLoess(y, x, w, span, degree = degree, parametric = parametric, : neighborhood radius 0.005
```

```
## Warning in simpleLoess(y, x, w, span, degree = degree, parametric = parametric, : reciprocal condition number 1
```

```
## Warning in simpleLoess(y, x, w, span, degree = degree, parametric = parametric, : There are other near singularities as well. 1.01
```

```
## Warning in simpleLoess(y, x, w, span, degree = degree, parametric = parametric, : zero-width neighborhood. make span bigger
```

```
## Warning: Computation failed in `stat_smooth()`:
## NA/NaN/Inf in foreign function call (arg 5)
```

```
## Warning in simpleLoess(y, x, w, span, degree = degree, parametric = parametric, : at -0.005
```

```
## Warning in simpleLoess(y, x, w, span, degree = degree, parametric = parametric, : radius 2.5e-05
```

```
## Warning in simpleLoess(y, x, w, span, degree = degree, parametric = parametric, : all data on boundary of neighborhood. make span bigger
```

```
## Warning in simpleLoess(y, x, w, span, degree = degree, parametric = parametric, : pseudoinverse used at -0.005
```

```
## Warning in simpleLoess(y, x, w, span, degree = degree, parametric = parametric, : neighborhood radius 0.005
```

```
## Warning in simpleLoess(y, x, w, span, degree = degree, parametric = parametric, : reciprocal condition number 1
```

```
## Warning in simpleLoess(y, x, w, span, degree = degree, parametric = parametric, : There are other near singularities as well. 1.01
```

```
## Warning in simpleLoess(y, x, w, span, degree = degree, parametric = parametric, : zero-width neighborhood. make span bigger
```

```
## Warning: Computation failed in `stat_smooth()`:
## NA/NaN/Inf in foreign function call (arg 5)
```

```
## Warning in simpleLoess(y, x, w, span, degree = degree, parametric = parametric, : at -0.005
```

```
## Warning in simpleLoess(y, x, w, span, degree = degree, parametric = parametric, : radius 2.5e-05
```

```
## Warning in simpleLoess(y, x, w, span, degree = degree, parametric = parametric, : all data on boundary of neighborhood. make span bigger
```

```
## Warning in simpleLoess(y, x, w, span, degree = degree, parametric = parametric, : pseudoinverse used at -0.005
```

```
## Warning in simpleLoess(y, x, w, span, degree = degree, parametric = parametric, : neighborhood radius 0.005
```

```
## Warning in simpleLoess(y, x, w, span, degree = degree, parametric = parametric, : reciprocal condition number 1
```

```
## Warning in simpleLoess(y, x, w, span, degree = degree, parametric = parametric, : There are other near singularities as well. 1.01
```

```
## Warning in simpleLoess(y, x, w, span, degree = degree, parametric = parametric, : zero-width neighborhood. make span bigger
```

```
## Warning: Computation failed in `stat_smooth()`:
## NA/NaN/Inf in foreign function call (arg 5)
```

```
## Warning in simpleLoess(y, x, w, span, degree = degree, parametric = parametric, : at -0.005
```

```
## Warning in simpleLoess(y, x, w, span, degree = degree, parametric = parametric, : radius 2.5e-05
```

```
## Warning in simpleLoess(y, x, w, span, degree = degree, parametric = parametric, : all data on boundary of neighborhood. make span bigger
```

```
## Warning in simpleLoess(y, x, w, span, degree = degree, parametric = parametric, : pseudoinverse used at -0.005
```

```
## Warning in simpleLoess(y, x, w, span, degree = degree, parametric = parametric, : neighborhood radius 0.005
```

```
## Warning in simpleLoess(y, x, w, span, degree = degree, parametric = parametric, : reciprocal condition number 1
```

```
## Warning in simpleLoess(y, x, w, span, degree = degree, parametric = parametric, : There are other near singularities as well. 1.01
```

```
## Warning in simpleLoess(y, x, w, span, degree = degree, parametric = parametric, : zero-width neighborhood. make span bigger
```

```
## Warning: Computation failed in `stat_smooth()`:
## NA/NaN/Inf in foreign function call (arg 5)
```

```
## Warning in simpleLoess(y, x, w, span, degree = degree, parametric = parametric, : at -0.005
```

```
## Warning in simpleLoess(y, x, w, span, degree = degree, parametric = parametric, : radius 2.5e-05
```

```
## Warning in simpleLoess(y, x, w, span, degree = degree, parametric = parametric, : all data on boundary of neighborhood. make span bigger
```

```
## Warning in simpleLoess(y, x, w, span, degree = degree, parametric = parametric, : pseudoinverse used at -0.005
```

```
## Warning in simpleLoess(y, x, w, span, degree = degree, parametric = parametric, : neighborhood radius 0.005
```

```
## Warning in simpleLoess(y, x, w, span, degree = degree, parametric = parametric, : reciprocal condition number 1
```

```
## Warning in simpleLoess(y, x, w, span, degree = degree, parametric = parametric, : There are other near singularities as well. 1.01
```

```
## Warning in simpleLoess(y, x, w, span, degree = degree, parametric = parametric, : zero-width neighborhood. make span bigger
```

```
## Warning: Computation failed in `stat_smooth()`:
## NA/NaN/Inf in foreign function call (arg 5)
```

```
## Warning in simpleLoess(y, x, w, span, degree = degree, parametric = parametric, : at -0.005
```

```
## Warning in simpleLoess(y, x, w, span, degree = degree, parametric = parametric, : radius 2.5e-05
```

```
## Warning in simpleLoess(y, x, w, span, degree = degree, parametric = parametric, : all data on boundary of neighborhood. make span bigger
```

```
## Warning in simpleLoess(y, x, w, span, degree = degree, parametric = parametric, : pseudoinverse used at -0.005
```

```
## Warning in simpleLoess(y, x, w, span, degree = degree, parametric = parametric, : neighborhood radius 0.005
```

```
## Warning in simpleLoess(y, x, w, span, degree = degree, parametric = parametric, : reciprocal condition number 1
```

```
## Warning in simpleLoess(y, x, w, span, degree = degree, parametric = parametric, : There are other near singularities as well. 1.01
```

```
## Warning in simpleLoess(y, x, w, span, degree = degree, parametric = parametric, : zero-width neighborhood. make span bigger
```

```
## Warning: Computation failed in `stat_smooth()`:
## NA/NaN/Inf in foreign function call (arg 5)
```

```
## Warning in simpleLoess(y, x, w, span, degree = degree, parametric = parametric, : at -0.005
```

```
## Warning in simpleLoess(y, x, w, span, degree = degree, parametric = parametric, : radius 2.5e-05
```

```
## Warning in simpleLoess(y, x, w, span, degree = degree, parametric = parametric, : all data on boundary of neighborhood. make span bigger
```

```
## Warning in simpleLoess(y, x, w, span, degree = degree, parametric = parametric, : pseudoinverse used at -0.005
```

```
## Warning in simpleLoess(y, x, w, span, degree = degree, parametric = parametric, : neighborhood radius 0.005
```

```
## Warning in simpleLoess(y, x, w, span, degree = degree, parametric = parametric, : reciprocal condition number 1
```

```
## Warning in simpleLoess(y, x, w, span, degree = degree, parametric = parametric, : There are other near singularities as well. 1.01
```

```
## Warning in simpleLoess(y, x, w, span, degree = degree, parametric = parametric, : zero-width neighborhood. make span bigger
```

```
## Warning: Computation failed in `stat_smooth()`:
## NA/NaN/Inf in foreign function call (arg 5)
```

```
## Warning in simpleLoess(y, x, w, span, degree = degree, parametric = parametric, : at -0.005
```

```
## Warning in simpleLoess(y, x, w, span, degree = degree, parametric = parametric, : radius 2.5e-05
```

```
## Warning in simpleLoess(y, x, w, span, degree = degree, parametric = parametric, : all data on boundary of neighborhood. make span bigger
```

```
## Warning in simpleLoess(y, x, w, span, degree = degree, parametric = parametric, : pseudoinverse used at -0.005
```

```
## Warning in simpleLoess(y, x, w, span, degree = degree, parametric = parametric, : neighborhood radius 0.005
```

```
## Warning in simpleLoess(y, x, w, span, degree = degree, parametric = parametric, : reciprocal condition number 1
```

```
## Warning in simpleLoess(y, x, w, span, degree = degree, parametric = parametric, : There are other near singularities as well. 1.01
```

```
## Warning in simpleLoess(y, x, w, span, degree = degree, parametric = parametric, : zero-width neighborhood. make span bigger
```

```
## Warning: Computation failed in `stat_smooth()`:
## NA/NaN/Inf in foreign function call (arg 5)
```

```
## Warning in simpleLoess(y, x, w, span, degree = degree, parametric = parametric, : at -0.005
```

```
## Warning in simpleLoess(y, x, w, span, degree = degree, parametric = parametric, : radius 2.5e-05
```

```
## Warning in simpleLoess(y, x, w, span, degree = degree, parametric = parametric, : all data on boundary of neighborhood. make span bigger
```

```
## Warning in simpleLoess(y, x, w, span, degree = degree, parametric = parametric, : pseudoinverse used at -0.005
```

```
## Warning in simpleLoess(y, x, w, span, degree = degree, parametric = parametric, : neighborhood radius 0.005
```

```
## Warning in simpleLoess(y, x, w, span, degree = degree, parametric = parametric, : reciprocal condition number 1
```

```
## Warning in simpleLoess(y, x, w, span, degree = degree, parametric = parametric, : There are other near singularities as well. 1.01
```

```
## Warning in simpleLoess(y, x, w, span, degree = degree, parametric = parametric, : zero-width neighborhood. make span bigger
```

```
## Warning: Computation failed in `stat_smooth()`:
## NA/NaN/Inf in foreign function call (arg 5)
```

```
## Warning in simpleLoess(y, x, w, span, degree = degree, parametric = parametric, : at -0.005
```

```
## Warning in simpleLoess(y, x, w, span, degree = degree, parametric = parametric, : radius 2.5e-05
```

```
## Warning in simpleLoess(y, x, w, span, degree = degree, parametric = parametric, : all data on boundary of neighborhood. make span bigger
```

```
## Warning in simpleLoess(y, x, w, span, degree = degree, parametric = parametric, : pseudoinverse used at -0.005
```

```
## Warning in simpleLoess(y, x, w, span, degree = degree, parametric = parametric, : neighborhood radius 0.005
```

```
## Warning in simpleLoess(y, x, w, span, degree = degree, parametric = parametric, : reciprocal condition number 1
```

```
## Warning in simpleLoess(y, x, w, span, degree = degree, parametric = parametric, : There are other near singularities as well. 1.01
```

```
## Warning in simpleLoess(y, x, w, span, degree = degree, parametric = parametric, : zero-width neighborhood. make span bigger
```

```
## Warning: Computation failed in `stat_smooth()`:
## NA/NaN/Inf in foreign function call (arg 5)
```

```
## Warning in simpleLoess(y, x, w, span, degree = degree, parametric = parametric, : at -0.005
```

```
## Warning in simpleLoess(y, x, w, span, degree = degree, parametric = parametric, : radius 2.5e-05
```

```
## Warning in simpleLoess(y, x, w, span, degree = degree, parametric = parametric, : all data on boundary of neighborhood. make span bigger
```

```
## Warning in simpleLoess(y, x, w, span, degree = degree, parametric = parametric, : pseudoinverse used at -0.005
```

```
## Warning in simpleLoess(y, x, w, span, degree = degree, parametric = parametric, : neighborhood radius 0.005
```

```
## Warning in simpleLoess(y, x, w, span, degree = degree, parametric = parametric, : reciprocal condition number 1
```

```
## Warning in simpleLoess(y, x, w, span, degree = degree, parametric = parametric, : There are other near singularities as well. 1.01
```

```
## Warning in simpleLoess(y, x, w, span, degree = degree, parametric = parametric, : zero-width neighborhood. make span bigger
```

```
## Warning: Computation failed in `stat_smooth()`:
## NA/NaN/Inf in foreign function call (arg 5)
```

```
## Warning in simpleLoess(y, x, w, span, degree = degree, parametric = parametric, : at -0.005
```

```
## Warning in simpleLoess(y, x, w, span, degree = degree, parametric = parametric, : radius 2.5e-05
```

```
## Warning in simpleLoess(y, x, w, span, degree = degree, parametric = parametric, : all data on boundary of neighborhood. make span bigger
```

```
## Warning in simpleLoess(y, x, w, span, degree = degree, parametric = parametric, : pseudoinverse used at -0.005
```

```
## Warning in simpleLoess(y, x, w, span, degree = degree, parametric = parametric, : neighborhood radius 0.005
```

```
## Warning in simpleLoess(y, x, w, span, degree = degree, parametric = parametric, : reciprocal condition number 1
```

```
## Warning in simpleLoess(y, x, w, span, degree = degree, parametric = parametric, : There are other near singularities as well. 1.01
```

```
## Warning in simpleLoess(y, x, w, span, degree = degree, parametric = parametric, : zero-width neighborhood. make span bigger
```

```
## Warning: Computation failed in `stat_smooth()`:
## NA/NaN/Inf in foreign function call (arg 5)
```

```
## Warning in simpleLoess(y, x, w, span, degree = degree, parametric = parametric, : at -0.005
```

```
## Warning in simpleLoess(y, x, w, span, degree = degree, parametric = parametric, : radius 2.5e-05
```

```
## Warning in simpleLoess(y, x, w, span, degree = degree, parametric = parametric, : all data on boundary of neighborhood. make span bigger
```

```
## Warning in simpleLoess(y, x, w, span, degree = degree, parametric = parametric, : pseudoinverse used at -0.005
```

```
## Warning in simpleLoess(y, x, w, span, degree = degree, parametric = parametric, : neighborhood radius 0.005
```

```
## Warning in simpleLoess(y, x, w, span, degree = degree, parametric = parametric, : reciprocal condition number 1
```

```
## Warning in simpleLoess(y, x, w, span, degree = degree, parametric = parametric, : There are other near singularities as well. 1.01
```

```
## Warning in simpleLoess(y, x, w, span, degree = degree, parametric = parametric, : zero-width neighborhood. make span bigger
```

```
## Warning: Computation failed in `stat_smooth()`:
## NA/NaN/Inf in foreign function call (arg 5)
```

```
## Warning in simpleLoess(y, x, w, span, degree = degree, parametric = parametric, : at -0.005
```

```
## Warning in simpleLoess(y, x, w, span, degree = degree, parametric = parametric, : radius 2.5e-05
```

```
## Warning in simpleLoess(y, x, w, span, degree = degree, parametric = parametric, : all data on boundary of neighborhood. make span bigger
```

```
## Warning in simpleLoess(y, x, w, span, degree = degree, parametric = parametric, : pseudoinverse used at -0.005
```

```
## Warning in simpleLoess(y, x, w, span, degree = degree, parametric = parametric, : neighborhood radius 0.005
```

```
## Warning in simpleLoess(y, x, w, span, degree = degree, parametric = parametric, : reciprocal condition number 1
```

```
## Warning in simpleLoess(y, x, w, span, degree = degree, parametric = parametric, : There are other near singularities as well. 1.01
```

```
## Warning in simpleLoess(y, x, w, span, degree = degree, parametric = parametric, : zero-width neighborhood. make span bigger
```

```
## Warning: Computation failed in `stat_smooth()`:
## NA/NaN/Inf in foreign function call (arg 5)
```

```
## Warning in simpleLoess(y, x, w, span, degree = degree, parametric = parametric, : at -0.005
```

```
## Warning in simpleLoess(y, x, w, span, degree = degree, parametric = parametric, : radius 2.5e-05
```

```
## Warning in simpleLoess(y, x, w, span, degree = degree, parametric = parametric, : all data on boundary of neighborhood. make span bigger
```

```
## Warning in simpleLoess(y, x, w, span, degree = degree, parametric = parametric, : pseudoinverse used at -0.005
```

```
## Warning in simpleLoess(y, x, w, span, degree = degree, parametric = parametric, : neighborhood radius 0.005
```

```
## Warning in simpleLoess(y, x, w, span, degree = degree, parametric = parametric, : reciprocal condition number 1
```

```
## Warning in simpleLoess(y, x, w, span, degree = degree, parametric = parametric, : There are other near singularities as well. 1.01
```

```
## Warning in simpleLoess(y, x, w, span, degree = degree, parametric = parametric, : zero-width neighborhood. make span bigger
```

```
## Warning: Computation failed in `stat_smooth()`:
## NA/NaN/Inf in foreign function call (arg 5)
```

```
## Warning in simpleLoess(y, x, w, span, degree = degree, parametric = parametric, : pseudoinverse used at -0.005
```

```
## Warning in simpleLoess(y, x, w, span, degree = degree, parametric = parametric, : neighborhood radius 1.005
```

```
## Warning in simpleLoess(y, x, w, span, degree = degree, parametric = parametric, : reciprocal condition number 0
```

```
## Warning in simpleLoess(y, x, w, span, degree = degree, parametric = parametric, : There are other near singularities as well. 1.01
```

```
## Warning in predLoess(object$y, object$x, newx = if (is.null(newdata)) object$x else if (is.data.frame(newdata)) as.matrix(model.frame(delete.response(terms(object)), : pseudoinverse used at -0.005
```

```
## Warning in predLoess(object$y, object$x, newx = if (is.null(newdata)) object$x else if (is.data.frame(newdata)) as.matrix(model.frame(delete.response(terms(object)), : neighborhood radius 1.005
```

```
## Warning in predLoess(object$y, object$x, newx = if (is.null(newdata)) object$x else if (is.data.frame(newdata)) as.matrix(model.frame(delete.response(terms(object)), : reciprocal condition number 0
```

```
## Warning in predLoess(object$y, object$x, newx = if (is.null(newdata)) object$x else if (is.data.frame(newdata)) as.matrix(model.frame(delete.response(terms(object)), : There are other near
## singularities as well. 1.01
```

```
## Warning in simpleLoess(y, x, w, span, degree = degree, parametric = parametric, : at -0.005
```

```
## Warning in simpleLoess(y, x, w, span, degree = degree, parametric = parametric, : radius 2.5e-05
```

```
## Warning in simpleLoess(y, x, w, span, degree = degree, parametric = parametric, : all data on boundary of neighborhood. make span bigger
```

```
## Warning in simpleLoess(y, x, w, span, degree = degree, parametric = parametric, : pseudoinverse used at -0.005
```

```
## Warning in simpleLoess(y, x, w, span, degree = degree, parametric = parametric, : neighborhood radius 0.005
```

```
## Warning in simpleLoess(y, x, w, span, degree = degree, parametric = parametric, : reciprocal condition number 1
```

```
## Warning in simpleLoess(y, x, w, span, degree = degree, parametric = parametric, : There are other near singularities as well. 1.01
```

```
## Warning in simpleLoess(y, x, w, span, degree = degree, parametric = parametric, : zero-width neighborhood. make span bigger
```

```
## Warning: Computation failed in `stat_smooth()`:
## NA/NaN/Inf in foreign function call (arg 5)
```

```
## Warning in simpleLoess(y, x, w, span, degree = degree, parametric = parametric, : at -0.005
```

```
## Warning in simpleLoess(y, x, w, span, degree = degree, parametric = parametric, : radius 2.5e-05
```

```
## Warning in simpleLoess(y, x, w, span, degree = degree, parametric = parametric, : all data on boundary of neighborhood. make span bigger
```

```
## Warning in simpleLoess(y, x, w, span, degree = degree, parametric = parametric, : pseudoinverse used at -0.005
```

```
## Warning in simpleLoess(y, x, w, span, degree = degree, parametric = parametric, : neighborhood radius 0.005
```

```
## Warning in simpleLoess(y, x, w, span, degree = degree, parametric = parametric, : reciprocal condition number 1
```

```
## Warning in simpleLoess(y, x, w, span, degree = degree, parametric = parametric, : There are other near singularities as well. 1.01
```

```
## Warning in simpleLoess(y, x, w, span, degree = degree, parametric = parametric, : zero-width neighborhood. make span bigger
```

```
## Warning: Computation failed in `stat_smooth()`:
## NA/NaN/Inf in foreign function call (arg 5)
```

```
## Warning in simpleLoess(y, x, w, span, degree = degree, parametric = parametric, : at -0.005
```

```
## Warning in simpleLoess(y, x, w, span, degree = degree, parametric = parametric, : radius 2.5e-05
```

```
## Warning in simpleLoess(y, x, w, span, degree = degree, parametric = parametric, : all data on boundary of neighborhood. make span bigger
```

```
## Warning in simpleLoess(y, x, w, span, degree = degree, parametric = parametric, : pseudoinverse used at -0.005
```

```
## Warning in simpleLoess(y, x, w, span, degree = degree, parametric = parametric, : neighborhood radius 0.005
```

```
## Warning in simpleLoess(y, x, w, span, degree = degree, parametric = parametric, : reciprocal condition number 1
```

```
## Warning in simpleLoess(y, x, w, span, degree = degree, parametric = parametric, : There are other near singularities as well. 1.01
```

```
## Warning in simpleLoess(y, x, w, span, degree = degree, parametric = parametric, : zero-width neighborhood. make span bigger
```

```
## Warning: Computation failed in `stat_smooth()`:
## NA/NaN/Inf in foreign function call (arg 5)
```

```
## Warning in simpleLoess(y, x, w, span, degree = degree, parametric = parametric, : at -0.005
```

```
## Warning in simpleLoess(y, x, w, span, degree = degree, parametric = parametric, : radius 2.5e-05
```

```
## Warning in simpleLoess(y, x, w, span, degree = degree, parametric = parametric, : all data on boundary of neighborhood. make span bigger
```

```
## Warning in simpleLoess(y, x, w, span, degree = degree, parametric = parametric, : pseudoinverse used at -0.005
```

```
## Warning in simpleLoess(y, x, w, span, degree = degree, parametric = parametric, : neighborhood radius 0.005
```

```
## Warning in simpleLoess(y, x, w, span, degree = degree, parametric = parametric, : reciprocal condition number 1
```

```
## Warning in simpleLoess(y, x, w, span, degree = degree, parametric = parametric, : There are other near singularities as well. 1.01
```

```
## Warning in simpleLoess(y, x, w, span, degree = degree, parametric = parametric, : zero-width neighborhood. make span bigger
```

```
## Warning: Computation failed in `stat_smooth()`:
## NA/NaN/Inf in foreign function call (arg 5)
```

```
## Warning in simpleLoess(y, x, w, span, degree = degree, parametric = parametric, : at -0.005
```

```
## Warning in simpleLoess(y, x, w, span, degree = degree, parametric = parametric, : radius 2.5e-05
```

```
## Warning in simpleLoess(y, x, w, span, degree = degree, parametric = parametric, : all data on boundary of neighborhood. make span bigger
```

```
## Warning in simpleLoess(y, x, w, span, degree = degree, parametric = parametric, : pseudoinverse used at -0.005
```

```
## Warning in simpleLoess(y, x, w, span, degree = degree, parametric = parametric, : neighborhood radius 0.005
```

```
## Warning in simpleLoess(y, x, w, span, degree = degree, parametric = parametric, : reciprocal condition number 1
```

```
## Warning in simpleLoess(y, x, w, span, degree = degree, parametric = parametric, : There are other near singularities as well. 1.01
```

```
## Warning in simpleLoess(y, x, w, span, degree = degree, parametric = parametric, : zero-width neighborhood. make span bigger
```

```
## Warning: Computation failed in `stat_smooth()`:
## NA/NaN/Inf in foreign function call (arg 5)
```

```
## Warning in simpleLoess(y, x, w, span, degree = degree, parametric = parametric, : at -0.005
```

```
## Warning in simpleLoess(y, x, w, span, degree = degree, parametric = parametric, : radius 2.5e-05
```

```
## Warning in simpleLoess(y, x, w, span, degree = degree, parametric = parametric, : all data on boundary of neighborhood. make span bigger
```

```
## Warning in simpleLoess(y, x, w, span, degree = degree, parametric = parametric, : pseudoinverse used at -0.005
```

```
## Warning in simpleLoess(y, x, w, span, degree = degree, parametric = parametric, : neighborhood radius 0.005
```

```
## Warning in simpleLoess(y, x, w, span, degree = degree, parametric = parametric, : reciprocal condition number 1
```

```
## Warning in simpleLoess(y, x, w, span, degree = degree, parametric = parametric, : There are other near singularities as well. 1.01
```

```
## Warning in simpleLoess(y, x, w, span, degree = degree, parametric = parametric, : zero-width neighborhood. make span bigger
```

```
## Warning: Computation failed in `stat_smooth()`:
## NA/NaN/Inf in foreign function call (arg 5)
```

```
## Warning in simpleLoess(y, x, w, span, degree = degree, parametric = parametric, : at -0.005
```

```
## Warning in simpleLoess(y, x, w, span, degree = degree, parametric = parametric, : radius 2.5e-05
```

```
## Warning in simpleLoess(y, x, w, span, degree = degree, parametric = parametric, : all data on boundary of neighborhood. make span bigger
```

```
## Warning in simpleLoess(y, x, w, span, degree = degree, parametric = parametric, : pseudoinverse used at -0.005
```

```
## Warning in simpleLoess(y, x, w, span, degree = degree, parametric = parametric, : neighborhood radius 0.005
```

```
## Warning in simpleLoess(y, x, w, span, degree = degree, parametric = parametric, : reciprocal condition number 1
```

```
## Warning in simpleLoess(y, x, w, span, degree = degree, parametric = parametric, : There are other near singularities as well. 1.01
```

```
## Warning in simpleLoess(y, x, w, span, degree = degree, parametric = parametric, : zero-width neighborhood. make span bigger
```

```
## Warning: Computation failed in `stat_smooth()`:
## NA/NaN/Inf in foreign function call (arg 5)
```

```
## Warning in simpleLoess(y, x, w, span, degree = degree, parametric = parametric, : at -0.005
```

```
## Warning in simpleLoess(y, x, w, span, degree = degree, parametric = parametric, : radius 2.5e-05
```

```
## Warning in simpleLoess(y, x, w, span, degree = degree, parametric = parametric, : all data on boundary of neighborhood. make span bigger
```

```
## Warning in simpleLoess(y, x, w, span, degree = degree, parametric = parametric, : pseudoinverse used at -0.005
```

```
## Warning in simpleLoess(y, x, w, span, degree = degree, parametric = parametric, : neighborhood radius 0.005
```

```
## Warning in simpleLoess(y, x, w, span, degree = degree, parametric = parametric, : reciprocal condition number 1
```

```
## Warning in simpleLoess(y, x, w, span, degree = degree, parametric = parametric, : There are other near singularities as well. 1.01
```

```
## Warning in simpleLoess(y, x, w, span, degree = degree, parametric = parametric, : zero-width neighborhood. make span bigger
```

```
## Warning: Computation failed in `stat_smooth()`:
## NA/NaN/Inf in foreign function call (arg 5)
```

```
## Warning in simpleLoess(y, x, w, span, degree = degree, parametric = parametric, : at -0.005
```

```
## Warning in simpleLoess(y, x, w, span, degree = degree, parametric = parametric, : radius 2.5e-05
```

```
## Warning in simpleLoess(y, x, w, span, degree = degree, parametric = parametric, : all data on boundary of neighborhood. make span bigger
```

```
## Warning in simpleLoess(y, x, w, span, degree = degree, parametric = parametric, : pseudoinverse used at -0.005
```

```
## Warning in simpleLoess(y, x, w, span, degree = degree, parametric = parametric, : neighborhood radius 0.005
```

```
## Warning in simpleLoess(y, x, w, span, degree = degree, parametric = parametric, : reciprocal condition number 1
```

```
## Warning in simpleLoess(y, x, w, span, degree = degree, parametric = parametric, : There are other near singularities as well. 1.01
```

```
## Warning in simpleLoess(y, x, w, span, degree = degree, parametric = parametric, : zero-width neighborhood. make span bigger
```

```
## Warning: Computation failed in `stat_smooth()`:
## NA/NaN/Inf in foreign function call (arg 5)
```

```
## Warning in simpleLoess(y, x, w, span, degree = degree, parametric = parametric, : at -0.005
```

```
## Warning in simpleLoess(y, x, w, span, degree = degree, parametric = parametric, : radius 2.5e-05
```

```
## Warning in simpleLoess(y, x, w, span, degree = degree, parametric = parametric, : all data on boundary of neighborhood. make span bigger
```

```
## Warning in simpleLoess(y, x, w, span, degree = degree, parametric = parametric, : pseudoinverse used at -0.005
```

```
## Warning in simpleLoess(y, x, w, span, degree = degree, parametric = parametric, : neighborhood radius 0.005
```

```
## Warning in simpleLoess(y, x, w, span, degree = degree, parametric = parametric, : reciprocal condition number 1
```

```
## Warning in simpleLoess(y, x, w, span, degree = degree, parametric = parametric, : There are other near singularities as well. 1.01
```

```
## Warning in simpleLoess(y, x, w, span, degree = degree, parametric = parametric, : zero-width neighborhood. make span bigger
```

```
## Warning: Computation failed in `stat_smooth()`:
## NA/NaN/Inf in foreign function call (arg 5)
```

```
## Warning in simpleLoess(y, x, w, span, degree = degree, parametric = parametric, : at -0.005
```

```
## Warning in simpleLoess(y, x, w, span, degree = degree, parametric = parametric, : radius 2.5e-05
```

```
## Warning in simpleLoess(y, x, w, span, degree = degree, parametric = parametric, : all data on boundary of neighborhood. make span bigger
```

```
## Warning in simpleLoess(y, x, w, span, degree = degree, parametric = parametric, : pseudoinverse used at -0.005
```

```
## Warning in simpleLoess(y, x, w, span, degree = degree, parametric = parametric, : neighborhood radius 0.005
```

```
## Warning in simpleLoess(y, x, w, span, degree = degree, parametric = parametric, : reciprocal condition number 1
```

```
## Warning in simpleLoess(y, x, w, span, degree = degree, parametric = parametric, : There are other near singularities as well. 1.01
```

```
## Warning in simpleLoess(y, x, w, span, degree = degree, parametric = parametric, : zero-width neighborhood. make span bigger
```

```
## Warning: Computation failed in `stat_smooth()`:
## NA/NaN/Inf in foreign function call (arg 5)
```

```
## Warning in simpleLoess(y, x, w, span, degree = degree, parametric = parametric, : at -0.005
```

```
## Warning in simpleLoess(y, x, w, span, degree = degree, parametric = parametric, : radius 2.5e-05
```

```
## Warning in simpleLoess(y, x, w, span, degree = degree, parametric = parametric, : all data on boundary of neighborhood. make span bigger
```

```
## Warning in simpleLoess(y, x, w, span, degree = degree, parametric = parametric, : pseudoinverse used at -0.005
```

```
## Warning in simpleLoess(y, x, w, span, degree = degree, parametric = parametric, : neighborhood radius 0.005
```

```
## Warning in simpleLoess(y, x, w, span, degree = degree, parametric = parametric, : reciprocal condition number 1
```

```
## Warning in simpleLoess(y, x, w, span, degree = degree, parametric = parametric, : There are other near singularities as well. 1.01
```

```
## Warning in simpleLoess(y, x, w, span, degree = degree, parametric = parametric, : zero-width neighborhood. make span bigger
```

```
## Warning: Computation failed in `stat_smooth()`:
## NA/NaN/Inf in foreign function call (arg 5)
```

```
## Warning in simpleLoess(y, x, w, span, degree = degree, parametric = parametric, : at -0.005
```

```
## Warning in simpleLoess(y, x, w, span, degree = degree, parametric = parametric, : radius 2.5e-05
```

```
## Warning in simpleLoess(y, x, w, span, degree = degree, parametric = parametric, : all data on boundary of neighborhood. make span bigger
```

```
## Warning in simpleLoess(y, x, w, span, degree = degree, parametric = parametric, : pseudoinverse used at -0.005
```

```
## Warning in simpleLoess(y, x, w, span, degree = degree, parametric = parametric, : neighborhood radius 0.005
```

```
## Warning in simpleLoess(y, x, w, span, degree = degree, parametric = parametric, : reciprocal condition number 1
```

```
## Warning in simpleLoess(y, x, w, span, degree = degree, parametric = parametric, : There are other near singularities as well. 1.01
```

```
## Warning in simpleLoess(y, x, w, span, degree = degree, parametric = parametric, : zero-width neighborhood. make span bigger
```

```
## Warning: Computation failed in `stat_smooth()`:
## NA/NaN/Inf in foreign function call (arg 5)
```

```
## Warning in simpleLoess(y, x, w, span, degree = degree, parametric = parametric, : at -0.005
```

```
## Warning in simpleLoess(y, x, w, span, degree = degree, parametric = parametric, : radius 2.5e-05
```

```
## Warning in simpleLoess(y, x, w, span, degree = degree, parametric = parametric, : all data on boundary of neighborhood. make span bigger
```

```
## Warning in simpleLoess(y, x, w, span, degree = degree, parametric = parametric, : pseudoinverse used at -0.005
```

```
## Warning in simpleLoess(y, x, w, span, degree = degree, parametric = parametric, : neighborhood radius 0.005
```

```
## Warning in simpleLoess(y, x, w, span, degree = degree, parametric = parametric, : reciprocal condition number 1
```

```
## Warning in simpleLoess(y, x, w, span, degree = degree, parametric = parametric, : There are other near singularities as well. 1.01
```

```
## Warning in simpleLoess(y, x, w, span, degree = degree, parametric = parametric, : zero-width neighborhood. make span bigger
```

```
## Warning: Computation failed in `stat_smooth()`:
## NA/NaN/Inf in foreign function call (arg 5)
```

```
## Warning in simpleLoess(y, x, w, span, degree = degree, parametric = parametric, : at -0.005
```

```
## Warning in simpleLoess(y, x, w, span, degree = degree, parametric = parametric, : radius 2.5e-05
```

```
## Warning in simpleLoess(y, x, w, span, degree = degree, parametric = parametric, : all data on boundary of neighborhood. make span bigger
```

```
## Warning in simpleLoess(y, x, w, span, degree = degree, parametric = parametric, : pseudoinverse used at -0.005
```

```
## Warning in simpleLoess(y, x, w, span, degree = degree, parametric = parametric, : neighborhood radius 0.005
```

```
## Warning in simpleLoess(y, x, w, span, degree = degree, parametric = parametric, : reciprocal condition number 1
```

```
## Warning in simpleLoess(y, x, w, span, degree = degree, parametric = parametric, : There are other near singularities as well. 1.01
```

```
## Warning in simpleLoess(y, x, w, span, degree = degree, parametric = parametric, : zero-width neighborhood. make span bigger
```

```
## Warning: Computation failed in `stat_smooth()`:
## NA/NaN/Inf in foreign function call (arg 5)
```

```
## Warning in simpleLoess(y, x, w, span, degree = degree, parametric = parametric, : at -0.005
```

```
## Warning in simpleLoess(y, x, w, span, degree = degree, parametric = parametric, : radius 2.5e-05
```

```
## Warning in simpleLoess(y, x, w, span, degree = degree, parametric = parametric, : all data on boundary of neighborhood. make span bigger
```

```
## Warning in simpleLoess(y, x, w, span, degree = degree, parametric = parametric, : pseudoinverse used at -0.005
```

```
## Warning in simpleLoess(y, x, w, span, degree = degree, parametric = parametric, : neighborhood radius 0.005
```

```
## Warning in simpleLoess(y, x, w, span, degree = degree, parametric = parametric, : reciprocal condition number 1
```

```
## Warning in simpleLoess(y, x, w, span, degree = degree, parametric = parametric, : There are other near singularities as well. 1.01
```

```
## Warning in simpleLoess(y, x, w, span, degree = degree, parametric = parametric, : zero-width neighborhood. make span bigger
```

```
## Warning: Computation failed in `stat_smooth()`:
## NA/NaN/Inf in foreign function call (arg 5)
```

```
## Warning in simpleLoess(y, x, w, span, degree = degree, parametric = parametric, : at -0.005
```

```
## Warning in simpleLoess(y, x, w, span, degree = degree, parametric = parametric, : radius 2.5e-05
```

```
## Warning in simpleLoess(y, x, w, span, degree = degree, parametric = parametric, : all data on boundary of neighborhood. make span bigger
```

```
## Warning in simpleLoess(y, x, w, span, degree = degree, parametric = parametric, : pseudoinverse used at -0.005
```

```
## Warning in simpleLoess(y, x, w, span, degree = degree, parametric = parametric, : neighborhood radius 0.005
```

```
## Warning in simpleLoess(y, x, w, span, degree = degree, parametric = parametric, : reciprocal condition number 1
```

```
## Warning in simpleLoess(y, x, w, span, degree = degree, parametric = parametric, : There are other near singularities as well. 1.01
```

```
## Warning in simpleLoess(y, x, w, span, degree = degree, parametric = parametric, : zero-width neighborhood. make span bigger
```

```
## Warning: Computation failed in `stat_smooth()`:
## NA/NaN/Inf in foreign function call (arg 5)
```

```
## Warning in simpleLoess(y, x, w, span, degree = degree, parametric = parametric, : at -0.005
```

```
## Warning in simpleLoess(y, x, w, span, degree = degree, parametric = parametric, : radius 2.5e-05
```

```
## Warning in simpleLoess(y, x, w, span, degree = degree, parametric = parametric, : all data on boundary of neighborhood. make span bigger
```

```
## Warning in simpleLoess(y, x, w, span, degree = degree, parametric = parametric, : pseudoinverse used at -0.005
```

```
## Warning in simpleLoess(y, x, w, span, degree = degree, parametric = parametric, : neighborhood radius 0.005
```

```
## Warning in simpleLoess(y, x, w, span, degree = degree, parametric = parametric, : reciprocal condition number 1
```

```
## Warning in simpleLoess(y, x, w, span, degree = degree, parametric = parametric, : There are other near singularities as well. 1.01
```

```
## Warning in simpleLoess(y, x, w, span, degree = degree, parametric = parametric, : zero-width neighborhood. make span bigger
```

```
## Warning: Computation failed in `stat_smooth()`:
## NA/NaN/Inf in foreign function call (arg 5)
```

```
## Warning in simpleLoess(y, x, w, span, degree = degree, parametric = parametric, : at -0.005
```

```
## Warning in simpleLoess(y, x, w, span, degree = degree, parametric = parametric, : radius 2.5e-05
```

```
## Warning in simpleLoess(y, x, w, span, degree = degree, parametric = parametric, : all data on boundary of neighborhood. make span bigger
```

```
## Warning in simpleLoess(y, x, w, span, degree = degree, parametric = parametric, : pseudoinverse used at -0.005
```

```
## Warning in simpleLoess(y, x, w, span, degree = degree, parametric = parametric, : neighborhood radius 0.005
```

```
## Warning in simpleLoess(y, x, w, span, degree = degree, parametric = parametric, : reciprocal condition number 1
```

```
## Warning in simpleLoess(y, x, w, span, degree = degree, parametric = parametric, : There are other near singularities as well. 1.01
```

```
## Warning in simpleLoess(y, x, w, span, degree = degree, parametric = parametric, : zero-width neighborhood. make span bigger
```

```
## Warning: Computation failed in `stat_smooth()`:
## NA/NaN/Inf in foreign function call (arg 5)
```

```
## Warning in simpleLoess(y, x, w, span, degree = degree, parametric = parametric, : pseudoinverse used at -0.005
```

```
## Warning in simpleLoess(y, x, w, span, degree = degree, parametric = parametric, : neighborhood radius 1.005
```

```
## Warning in simpleLoess(y, x, w, span, degree = degree, parametric = parametric, : reciprocal condition number 0
```

```
## Warning in simpleLoess(y, x, w, span, degree = degree, parametric = parametric, : There are other near singularities as well. 1.01
```

```
## Warning in predLoess(object$y, object$x, newx = if (is.null(newdata)) object$x else if (is.data.frame(newdata)) as.matrix(model.frame(delete.response(terms(object)), : pseudoinverse used at -0.005
```

```
## Warning in predLoess(object$y, object$x, newx = if (is.null(newdata)) object$x else if (is.data.frame(newdata)) as.matrix(model.frame(delete.response(terms(object)), : neighborhood radius 1.005
```

```
## Warning in predLoess(object$y, object$x, newx = if (is.null(newdata)) object$x else if (is.data.frame(newdata)) as.matrix(model.frame(delete.response(terms(object)), : reciprocal condition number 0
```

```
## Warning in predLoess(object$y, object$x, newx = if (is.null(newdata)) object$x else if (is.data.frame(newdata)) as.matrix(model.frame(delete.response(terms(object)), : There are other near
## singularities as well. 1.01
```

```
## Warning in simpleLoess(y, x, w, span, degree = degree, parametric = parametric, : at -0.005
```

```
## Warning in simpleLoess(y, x, w, span, degree = degree, parametric = parametric, : radius 2.5e-05
```

```
## Warning in simpleLoess(y, x, w, span, degree = degree, parametric = parametric, : all data on boundary of neighborhood. make span bigger
```

```
## Warning in simpleLoess(y, x, w, span, degree = degree, parametric = parametric, : pseudoinverse used at -0.005
```

```
## Warning in simpleLoess(y, x, w, span, degree = degree, parametric = parametric, : neighborhood radius 0.005
```

```
## Warning in simpleLoess(y, x, w, span, degree = degree, parametric = parametric, : reciprocal condition number 1
```

```
## Warning in simpleLoess(y, x, w, span, degree = degree, parametric = parametric, : There are other near singularities as well. 1.01
```

```
## Warning in simpleLoess(y, x, w, span, degree = degree, parametric = parametric, : zero-width neighborhood. make span bigger
```

```
## Warning: Computation failed in `stat_smooth()`:
## NA/NaN/Inf in foreign function call (arg 5)
```

```
## Warning in simpleLoess(y, x, w, span, degree = degree, parametric = parametric, : at -0.005
```

```
## Warning in simpleLoess(y, x, w, span, degree = degree, parametric = parametric, : radius 2.5e-05
```

```
## Warning in simpleLoess(y, x, w, span, degree = degree, parametric = parametric, : all data on boundary of neighborhood. make span bigger
```

```
## Warning in simpleLoess(y, x, w, span, degree = degree, parametric = parametric, : pseudoinverse used at -0.005
```

```
## Warning in simpleLoess(y, x, w, span, degree = degree, parametric = parametric, : neighborhood radius 0.005
```

```
## Warning in simpleLoess(y, x, w, span, degree = degree, parametric = parametric, : reciprocal condition number 1
```

```
## Warning in simpleLoess(y, x, w, span, degree = degree, parametric = parametric, : There are other near singularities as well. 1.01
```

```
## Warning in simpleLoess(y, x, w, span, degree = degree, parametric = parametric, : zero-width neighborhood. make span bigger
```

```
## Warning: Computation failed in `stat_smooth()`:
## NA/NaN/Inf in foreign function call (arg 5)
```

```
## Warning in simpleLoess(y, x, w, span, degree = degree, parametric = parametric, : at -0.005
```

```
## Warning in simpleLoess(y, x, w, span, degree = degree, parametric = parametric, : radius 2.5e-05
```

```
## Warning in simpleLoess(y, x, w, span, degree = degree, parametric = parametric, : all data on boundary of neighborhood. make span bigger
```

```
## Warning in simpleLoess(y, x, w, span, degree = degree, parametric = parametric, : pseudoinverse used at -0.005
```

```
## Warning in simpleLoess(y, x, w, span, degree = degree, parametric = parametric, : neighborhood radius 0.005
```

```
## Warning in simpleLoess(y, x, w, span, degree = degree, parametric = parametric, : reciprocal condition number 1
```

```
## Warning in simpleLoess(y, x, w, span, degree = degree, parametric = parametric, : There are other near singularities as well. 1.01
```

```
## Warning in simpleLoess(y, x, w, span, degree = degree, parametric = parametric, : zero-width neighborhood. make span bigger
```

```
## Warning: Computation failed in `stat_smooth()`:
## NA/NaN/Inf in foreign function call (arg 5)
```

```
## Warning in simpleLoess(y, x, w, span, degree = degree, parametric = parametric, : at -0.005
```

```
## Warning in simpleLoess(y, x, w, span, degree = degree, parametric = parametric, : radius 2.5e-05
```

```
## Warning in simpleLoess(y, x, w, span, degree = degree, parametric = parametric, : all data on boundary of neighborhood. make span bigger
```

```
## Warning in simpleLoess(y, x, w, span, degree = degree, parametric = parametric, : pseudoinverse used at -0.005
```

```
## Warning in simpleLoess(y, x, w, span, degree = degree, parametric = parametric, : neighborhood radius 0.005
```

```
## Warning in simpleLoess(y, x, w, span, degree = degree, parametric = parametric, : reciprocal condition number 1
```

```
## Warning in simpleLoess(y, x, w, span, degree = degree, parametric = parametric, : There are other near singularities as well. 1.01
```

```
## Warning in simpleLoess(y, x, w, span, degree = degree, parametric = parametric, : zero-width neighborhood. make span bigger
```

```
## Warning: Computation failed in `stat_smooth()`:
## NA/NaN/Inf in foreign function call (arg 5)
```

```
## Warning in simpleLoess(y, x, w, span, degree = degree, parametric = parametric, : at -0.005
```

```
## Warning in simpleLoess(y, x, w, span, degree = degree, parametric = parametric, : radius 2.5e-05
```

```
## Warning in simpleLoess(y, x, w, span, degree = degree, parametric = parametric, : all data on boundary of neighborhood. make span bigger
```

```
## Warning in simpleLoess(y, x, w, span, degree = degree, parametric = parametric, : pseudoinverse used at -0.005
```

```
## Warning in simpleLoess(y, x, w, span, degree = degree, parametric = parametric, : neighborhood radius 0.005
```

```
## Warning in simpleLoess(y, x, w, span, degree = degree, parametric = parametric, : reciprocal condition number 1
```

```
## Warning in simpleLoess(y, x, w, span, degree = degree, parametric = parametric, : There are other near singularities as well. 1.01
```

```
## Warning in simpleLoess(y, x, w, span, degree = degree, parametric = parametric, : zero-width neighborhood. make span bigger
```

```
## Warning: Computation failed in `stat_smooth()`:
## NA/NaN/Inf in foreign function call (arg 5)
```

```
## Warning in simpleLoess(y, x, w, span, degree = degree, parametric = parametric, : at -0.005
```

```
## Warning in simpleLoess(y, x, w, span, degree = degree, parametric = parametric, : radius 2.5e-05
```

```
## Warning in simpleLoess(y, x, w, span, degree = degree, parametric = parametric, : all data on boundary of neighborhood. make span bigger
```

```
## Warning in simpleLoess(y, x, w, span, degree = degree, parametric = parametric, : pseudoinverse used at -0.005
```

```
## Warning in simpleLoess(y, x, w, span, degree = degree, parametric = parametric, : neighborhood radius 0.005
```

```
## Warning in simpleLoess(y, x, w, span, degree = degree, parametric = parametric, : reciprocal condition number 1
```

```
## Warning in simpleLoess(y, x, w, span, degree = degree, parametric = parametric, : There are other near singularities as well. 1.01
```

```
## Warning in simpleLoess(y, x, w, span, degree = degree, parametric = parametric, : zero-width neighborhood. make span bigger
```

```
## Warning: Computation failed in `stat_smooth()`:
## NA/NaN/Inf in foreign function call (arg 5)
```

```
## Warning in simpleLoess(y, x, w, span, degree = degree, parametric = parametric, : at -0.005
```

```
## Warning in simpleLoess(y, x, w, span, degree = degree, parametric = parametric, : radius 2.5e-05
```

```
## Warning in simpleLoess(y, x, w, span, degree = degree, parametric = parametric, : all data on boundary of neighborhood. make span bigger
```

```
## Warning in simpleLoess(y, x, w, span, degree = degree, parametric = parametric, : pseudoinverse used at -0.005
```

```
## Warning in simpleLoess(y, x, w, span, degree = degree, parametric = parametric, : neighborhood radius 0.005
```

```
## Warning in simpleLoess(y, x, w, span, degree = degree, parametric = parametric, : reciprocal condition number 1
```

```
## Warning in simpleLoess(y, x, w, span, degree = degree, parametric = parametric, : There are other near singularities as well. 1.01
```

```
## Warning in simpleLoess(y, x, w, span, degree = degree, parametric = parametric, : zero-width neighborhood. make span bigger
```

```
## Warning: Computation failed in `stat_smooth()`:
## NA/NaN/Inf in foreign function call (arg 5)
```

```
## Warning in simpleLoess(y, x, w, span, degree = degree, parametric = parametric, : pseudoinverse used at -0.005
```

```
## Warning in simpleLoess(y, x, w, span, degree = degree, parametric = parametric, : neighborhood radius 1.005
```

```
## Warning in simpleLoess(y, x, w, span, degree = degree, parametric = parametric, : reciprocal condition number 0
```

```
## Warning in simpleLoess(y, x, w, span, degree = degree, parametric = parametric, : There are other near singularities as well. 1.01
```

```
## Warning in predLoess(object$y, object$x, newx = if (is.null(newdata)) object$x else if (is.data.frame(newdata)) as.matrix(model.frame(delete.response(terms(object)), : pseudoinverse used at -0.005
```

```
## Warning in predLoess(object$y, object$x, newx = if (is.null(newdata)) object$x else if (is.data.frame(newdata)) as.matrix(model.frame(delete.response(terms(object)), : neighborhood radius 1.005
```

```
## Warning in predLoess(object$y, object$x, newx = if (is.null(newdata)) object$x else if (is.data.frame(newdata)) as.matrix(model.frame(delete.response(terms(object)), : reciprocal condition number 0
```

```
## Warning in predLoess(object$y, object$x, newx = if (is.null(newdata)) object$x else if (is.data.frame(newdata)) as.matrix(model.frame(delete.response(terms(object)), : There are other near
## singularities as well. 1.01
```

```
## Warning in simpleLoess(y, x, w, span, degree = degree, parametric = parametric, : at -0.005
```

```
## Warning in simpleLoess(y, x, w, span, degree = degree, parametric = parametric, : radius 2.5e-05
```

```
## Warning in simpleLoess(y, x, w, span, degree = degree, parametric = parametric, : all data on boundary of neighborhood. make span bigger
```

```
## Warning in simpleLoess(y, x, w, span, degree = degree, parametric = parametric, : pseudoinverse used at -0.005
```

```
## Warning in simpleLoess(y, x, w, span, degree = degree, parametric = parametric, : neighborhood radius 0.005
```

```
## Warning in simpleLoess(y, x, w, span, degree = degree, parametric = parametric, : reciprocal condition number 1
```

```
## Warning in simpleLoess(y, x, w, span, degree = degree, parametric = parametric, : There are other near singularities as well. 1.01
```

```
## Warning in simpleLoess(y, x, w, span, degree = degree, parametric = parametric, : zero-width neighborhood. make span bigger
```

```
## Warning: Computation failed in `stat_smooth()`:
## NA/NaN/Inf in foreign function call (arg 5)
```

```
## Warning in simpleLoess(y, x, w, span, degree = degree, parametric = parametric, : at -0.005
```

```
## Warning in simpleLoess(y, x, w, span, degree = degree, parametric = parametric, : radius 2.5e-05
```

```
## Warning in simpleLoess(y, x, w, span, degree = degree, parametric = parametric, : all data on boundary of neighborhood. make span bigger
```

```
## Warning in simpleLoess(y, x, w, span, degree = degree, parametric = parametric, : pseudoinverse used at -0.005
```

```
## Warning in simpleLoess(y, x, w, span, degree = degree, parametric = parametric, : neighborhood radius 0.005
```

```
## Warning in simpleLoess(y, x, w, span, degree = degree, parametric = parametric, : reciprocal condition number 1
```

```
## Warning in simpleLoess(y, x, w, span, degree = degree, parametric = parametric, : There are other near singularities as well. 1.01
```

```
## Warning in simpleLoess(y, x, w, span, degree = degree, parametric = parametric, : zero-width neighborhood. make span bigger
```

```
## Warning: Computation failed in `stat_smooth()`:
## NA/NaN/Inf in foreign function call (arg 5)
```

```
## Warning in simpleLoess(y, x, w, span, degree = degree, parametric = parametric, : at -0.005
```

```
## Warning in simpleLoess(y, x, w, span, degree = degree, parametric = parametric, : radius 2.5e-05
```

```
## Warning in simpleLoess(y, x, w, span, degree = degree, parametric = parametric, : all data on boundary of neighborhood. make span bigger
```

```
## Warning in simpleLoess(y, x, w, span, degree = degree, parametric = parametric, : pseudoinverse used at -0.005
```

```
## Warning in simpleLoess(y, x, w, span, degree = degree, parametric = parametric, : neighborhood radius 0.005
```

```
## Warning in simpleLoess(y, x, w, span, degree = degree, parametric = parametric, : reciprocal condition number 1
```

```
## Warning in simpleLoess(y, x, w, span, degree = degree, parametric = parametric, : There are other near singularities as well. 1.01
```

```
## Warning in simpleLoess(y, x, w, span, degree = degree, parametric = parametric, : zero-width neighborhood. make span bigger
```

```
## Warning: Computation failed in `stat_smooth()`:
## NA/NaN/Inf in foreign function call (arg 5)
```

```
## Warning in simpleLoess(y, x, w, span, degree = degree, parametric = parametric, : pseudoinverse used at -0.005
```

```
## Warning in simpleLoess(y, x, w, span, degree = degree, parametric = parametric, : neighborhood radius 1.005
```

```
## Warning in simpleLoess(y, x, w, span, degree = degree, parametric = parametric, : reciprocal condition number 0
```

```
## Warning in simpleLoess(y, x, w, span, degree = degree, parametric = parametric, : There are other near singularities as well. 1.01
```

```
## Warning in predLoess(object$y, object$x, newx = if (is.null(newdata)) object$x else if (is.data.frame(newdata)) as.matrix(model.frame(delete.response(terms(object)), : pseudoinverse used at -0.005
```

```
## Warning in predLoess(object$y, object$x, newx = if (is.null(newdata)) object$x else if (is.data.frame(newdata)) as.matrix(model.frame(delete.response(terms(object)), : neighborhood radius 1.005
```

```
## Warning in predLoess(object$y, object$x, newx = if (is.null(newdata)) object$x else if (is.data.frame(newdata)) as.matrix(model.frame(delete.response(terms(object)), : reciprocal condition number 0
```

```
## Warning in predLoess(object$y, object$x, newx = if (is.null(newdata)) object$x else if (is.data.frame(newdata)) as.matrix(model.frame(delete.response(terms(object)), : There are other near
## singularities as well. 1.01
```

```
## Warning in simpleLoess(y, x, w, span, degree = degree, parametric = parametric, : at -0.005
```

```
## Warning in simpleLoess(y, x, w, span, degree = degree, parametric = parametric, : radius 2.5e-05
```

```
## Warning in simpleLoess(y, x, w, span, degree = degree, parametric = parametric, : all data on boundary of neighborhood. make span bigger
```

```
## Warning in simpleLoess(y, x, w, span, degree = degree, parametric = parametric, : pseudoinverse used at -0.005
```

```
## Warning in simpleLoess(y, x, w, span, degree = degree, parametric = parametric, : neighborhood radius 0.005
```

```
## Warning in simpleLoess(y, x, w, span, degree = degree, parametric = parametric, : reciprocal condition number 1
```

```
## Warning in simpleLoess(y, x, w, span, degree = degree, parametric = parametric, : There are other near singularities as well. 1.01
```

```
## Warning in simpleLoess(y, x, w, span, degree = degree, parametric = parametric, : zero-width neighborhood. make span bigger
```

```
## Warning: Computation failed in `stat_smooth()`:
## NA/NaN/Inf in foreign function call (arg 5)
```

```
## Warning in simpleLoess(y, x, w, span, degree = degree, parametric = parametric, : at -0.005
```

```
## Warning in simpleLoess(y, x, w, span, degree = degree, parametric = parametric, : radius 2.5e-05
```

```
## Warning in simpleLoess(y, x, w, span, degree = degree, parametric = parametric, : all data on boundary of neighborhood. make span bigger
```

```
## Warning in simpleLoess(y, x, w, span, degree = degree, parametric = parametric, : pseudoinverse used at -0.005
```

```
## Warning in simpleLoess(y, x, w, span, degree = degree, parametric = parametric, : neighborhood radius 0.005
```

```
## Warning in simpleLoess(y, x, w, span, degree = degree, parametric = parametric, : reciprocal condition number 1
```

```
## Warning in simpleLoess(y, x, w, span, degree = degree, parametric = parametric, : There are other near singularities as well. 1.01
```

```
## Warning in simpleLoess(y, x, w, span, degree = degree, parametric = parametric, : zero-width neighborhood. make span bigger
```

```
## Warning: Computation failed in `stat_smooth()`:
## NA/NaN/Inf in foreign function call (arg 5)
```

```
## Warning in simpleLoess(y, x, w, span, degree = degree, parametric = parametric, : at -0.005
```

```
## Warning in simpleLoess(y, x, w, span, degree = degree, parametric = parametric, : radius 2.5e-05
```

```
## Warning in simpleLoess(y, x, w, span, degree = degree, parametric = parametric, : all data on boundary of neighborhood. make span bigger
```

```
## Warning in simpleLoess(y, x, w, span, degree = degree, parametric = parametric, : pseudoinverse used at -0.005
```

```
## Warning in simpleLoess(y, x, w, span, degree = degree, parametric = parametric, : neighborhood radius 0.005
```

```
## Warning in simpleLoess(y, x, w, span, degree = degree, parametric = parametric, : reciprocal condition number 1
```

```
## Warning in simpleLoess(y, x, w, span, degree = degree, parametric = parametric, : There are other near singularities as well. 1.01
```

```
## Warning in simpleLoess(y, x, w, span, degree = degree, parametric = parametric, : zero-width neighborhood. make span bigger
```

```
## Warning: Computation failed in `stat_smooth()`:
## NA/NaN/Inf in foreign function call (arg 5)
```

```
## Warning in simpleLoess(y, x, w, span, degree = degree, parametric = parametric, : at -0.005
```

```
## Warning in simpleLoess(y, x, w, span, degree = degree, parametric = parametric, : radius 2.5e-05
```

```
## Warning in simpleLoess(y, x, w, span, degree = degree, parametric = parametric, : all data on boundary of neighborhood. make span bigger
```

```
## Warning in simpleLoess(y, x, w, span, degree = degree, parametric = parametric, : pseudoinverse used at -0.005
```

```
## Warning in simpleLoess(y, x, w, span, degree = degree, parametric = parametric, : neighborhood radius 0.005
```

```
## Warning in simpleLoess(y, x, w, span, degree = degree, parametric = parametric, : reciprocal condition number 1
```

```
## Warning in simpleLoess(y, x, w, span, degree = degree, parametric = parametric, : There are other near singularities as well. 1.01
```

```
## Warning in simpleLoess(y, x, w, span, degree = degree, parametric = parametric, : zero-width neighborhood. make span bigger
```

```
## Warning: Computation failed in `stat_smooth()`:
## NA/NaN/Inf in foreign function call (arg 5)
```

```
## Warning in simpleLoess(y, x, w, span, degree = degree, parametric = parametric, : at -0.005
```

```
## Warning in simpleLoess(y, x, w, span, degree = degree, parametric = parametric, : radius 2.5e-05
```

```
## Warning in simpleLoess(y, x, w, span, degree = degree, parametric = parametric, : all data on boundary of neighborhood. make span bigger
```

```
## Warning in simpleLoess(y, x, w, span, degree = degree, parametric = parametric, : pseudoinverse used at -0.005
```

```
## Warning in simpleLoess(y, x, w, span, degree = degree, parametric = parametric, : neighborhood radius 0.005
```

```
## Warning in simpleLoess(y, x, w, span, degree = degree, parametric = parametric, : reciprocal condition number 1
```

```
## Warning in simpleLoess(y, x, w, span, degree = degree, parametric = parametric, : There are other near singularities as well. 1.01
```

```
## Warning in simpleLoess(y, x, w, span, degree = degree, parametric = parametric, : zero-width neighborhood. make span bigger
```

```
## Warning: Computation failed in `stat_smooth()`:
## NA/NaN/Inf in foreign function call (arg 5)
```

```
## Warning in simpleLoess(y, x, w, span, degree = degree, parametric = parametric, : pseudoinverse used at -0.005
```

```
## Warning in simpleLoess(y, x, w, span, degree = degree, parametric = parametric, : neighborhood radius 1.005
```

```
## Warning in simpleLoess(y, x, w, span, degree = degree, parametric = parametric, : reciprocal condition number 0
```

```
## Warning in simpleLoess(y, x, w, span, degree = degree, parametric = parametric, : There are other near singularities as well. 1.01
```

```
## Warning in predLoess(object$y, object$x, newx = if (is.null(newdata)) object$x else if (is.data.frame(newdata)) as.matrix(model.frame(delete.response(terms(object)), : pseudoinverse used at -0.005
```

```
## Warning in predLoess(object$y, object$x, newx = if (is.null(newdata)) object$x else if (is.data.frame(newdata)) as.matrix(model.frame(delete.response(terms(object)), : neighborhood radius 1.005
```

```
## Warning in predLoess(object$y, object$x, newx = if (is.null(newdata)) object$x else if (is.data.frame(newdata)) as.matrix(model.frame(delete.response(terms(object)), : reciprocal condition number 0
```

```
## Warning in predLoess(object$y, object$x, newx = if (is.null(newdata)) object$x else if (is.data.frame(newdata)) as.matrix(model.frame(delete.response(terms(object)), : There are other near
## singularities as well. 1.01
```

```
## Warning in simpleLoess(y, x, w, span, degree = degree, parametric = parametric, : at -0.005
```

```
## Warning in simpleLoess(y, x, w, span, degree = degree, parametric = parametric, : radius 2.5e-05
```

```
## Warning in simpleLoess(y, x, w, span, degree = degree, parametric = parametric, : all data on boundary of neighborhood. make span bigger
```

```
## Warning in simpleLoess(y, x, w, span, degree = degree, parametric = parametric, : pseudoinverse used at -0.005
```

```
## Warning in simpleLoess(y, x, w, span, degree = degree, parametric = parametric, : neighborhood radius 0.005
```

```
## Warning in simpleLoess(y, x, w, span, degree = degree, parametric = parametric, : reciprocal condition number 1
```

```
## Warning in simpleLoess(y, x, w, span, degree = degree, parametric = parametric, : There are other near singularities as well. 1.01
```

```
## Warning in simpleLoess(y, x, w, span, degree = degree, parametric = parametric, : zero-width neighborhood. make span bigger
```

```
## Warning: Computation failed in `stat_smooth()`:
## NA/NaN/Inf in foreign function call (arg 5)
```

```
## Warning in simpleLoess(y, x, w, span, degree = degree, parametric = parametric, : at -0.005
```

```
## Warning in simpleLoess(y, x, w, span, degree = degree, parametric = parametric, : radius 2.5e-05
```

```
## Warning in simpleLoess(y, x, w, span, degree = degree, parametric = parametric, : all data on boundary of neighborhood. make span bigger
```

```
## Warning in simpleLoess(y, x, w, span, degree = degree, parametric = parametric, : pseudoinverse used at -0.005
```

```
## Warning in simpleLoess(y, x, w, span, degree = degree, parametric = parametric, : neighborhood radius 0.005
```

```
## Warning in simpleLoess(y, x, w, span, degree = degree, parametric = parametric, : reciprocal condition number 1
```

```
## Warning in simpleLoess(y, x, w, span, degree = degree, parametric = parametric, : There are other near singularities as well. 1.01
```

```
## Warning in simpleLoess(y, x, w, span, degree = degree, parametric = parametric, : zero-width neighborhood. make span bigger
```

```
## Warning: Computation failed in `stat_smooth()`:
## NA/NaN/Inf in foreign function call (arg 5)
```

```
## Warning in simpleLoess(y, x, w, span, degree = degree, parametric = parametric, : at -0.005
```

```
## Warning in simpleLoess(y, x, w, span, degree = degree, parametric = parametric, : radius 2.5e-05
```

```
## Warning in simpleLoess(y, x, w, span, degree = degree, parametric = parametric, : all data on boundary of neighborhood. make span bigger
```

```
## Warning in simpleLoess(y, x, w, span, degree = degree, parametric = parametric, : pseudoinverse used at -0.005
```

```
## Warning in simpleLoess(y, x, w, span, degree = degree, parametric = parametric, : neighborhood radius 0.005
```

```
## Warning in simpleLoess(y, x, w, span, degree = degree, parametric = parametric, : reciprocal condition number 1
```

```
## Warning in simpleLoess(y, x, w, span, degree = degree, parametric = parametric, : There are other near singularities as well. 1.01
```

```
## Warning in simpleLoess(y, x, w, span, degree = degree, parametric = parametric, : zero-width neighborhood. make span bigger
```

```
## Warning: Computation failed in `stat_smooth()`:
## NA/NaN/Inf in foreign function call (arg 5)
```

```
## Warning in simpleLoess(y, x, w, span, degree = degree, parametric = parametric, : at -0.005
```

```
## Warning in simpleLoess(y, x, w, span, degree = degree, parametric = parametric, : radius 2.5e-05
```

```
## Warning in simpleLoess(y, x, w, span, degree = degree, parametric = parametric, : all data on boundary of neighborhood. make span bigger
```

```
## Warning in simpleLoess(y, x, w, span, degree = degree, parametric = parametric, : pseudoinverse used at -0.005
```

```
## Warning in simpleLoess(y, x, w, span, degree = degree, parametric = parametric, : neighborhood radius 0.005
```

```
## Warning in simpleLoess(y, x, w, span, degree = degree, parametric = parametric, : reciprocal condition number 1
```

```
## Warning in simpleLoess(y, x, w, span, degree = degree, parametric = parametric, : There are other near singularities as well. 1.01
```

```
## Warning in simpleLoess(y, x, w, span, degree = degree, parametric = parametric, : zero-width neighborhood. make span bigger
```

```
## Warning: Computation failed in `stat_smooth()`:
## NA/NaN/Inf in foreign function call (arg 5)
```

```
## Warning in simpleLoess(y, x, w, span, degree = degree, parametric = parametric, : at -0.005
```

```
## Warning in simpleLoess(y, x, w, span, degree = degree, parametric = parametric, : radius 2.5e-05
```

```
## Warning in simpleLoess(y, x, w, span, degree = degree, parametric = parametric, : all data on boundary of neighborhood. make span bigger
```

```
## Warning in simpleLoess(y, x, w, span, degree = degree, parametric = parametric, : pseudoinverse used at -0.005
```

```
## Warning in simpleLoess(y, x, w, span, degree = degree, parametric = parametric, : neighborhood radius 0.005
```

```
## Warning in simpleLoess(y, x, w, span, degree = degree, parametric = parametric, : reciprocal condition number 1
```

```
## Warning in simpleLoess(y, x, w, span, degree = degree, parametric = parametric, : There are other near singularities as well. 1.01
```

```
## Warning in simpleLoess(y, x, w, span, degree = degree, parametric = parametric, : zero-width neighborhood. make span bigger
```

```
## Warning: Computation failed in `stat_smooth()`:
## NA/NaN/Inf in foreign function call (arg 5)
```

```
## Warning in simpleLoess(y, x, w, span, degree = degree, parametric = parametric, : at -0.005
```

```
## Warning in simpleLoess(y, x, w, span, degree = degree, parametric = parametric, : radius 2.5e-05
```

```
## Warning in simpleLoess(y, x, w, span, degree = degree, parametric = parametric, : all data on boundary of neighborhood. make span bigger
```

```
## Warning in simpleLoess(y, x, w, span, degree = degree, parametric = parametric, : pseudoinverse used at -0.005
```

```
## Warning in simpleLoess(y, x, w, span, degree = degree, parametric = parametric, : neighborhood radius 0.005
```

```
## Warning in simpleLoess(y, x, w, span, degree = degree, parametric = parametric, : reciprocal condition number 1
```

```
## Warning in simpleLoess(y, x, w, span, degree = degree, parametric = parametric, : There are other near singularities as well. 1.01
```

```
## Warning in simpleLoess(y, x, w, span, degree = degree, parametric = parametric, : zero-width neighborhood. make span bigger
```

```
## Warning: Computation failed in `stat_smooth()`:
## NA/NaN/Inf in foreign function call (arg 5)
```

```
## Warning in simpleLoess(y, x, w, span, degree = degree, parametric = parametric, : at -0.005
```

```
## Warning in simpleLoess(y, x, w, span, degree = degree, parametric = parametric, : radius 2.5e-05
```

```
## Warning in simpleLoess(y, x, w, span, degree = degree, parametric = parametric, : all data on boundary of neighborhood. make span bigger
```

```
## Warning in simpleLoess(y, x, w, span, degree = degree, parametric = parametric, : pseudoinverse used at -0.005
```

```
## Warning in simpleLoess(y, x, w, span, degree = degree, parametric = parametric, : neighborhood radius 0.005
```

```
## Warning in simpleLoess(y, x, w, span, degree = degree, parametric = parametric, : reciprocal condition number 1
```

```
## Warning in simpleLoess(y, x, w, span, degree = degree, parametric = parametric, : There are other near singularities as well. 1.01
```

```
## Warning in simpleLoess(y, x, w, span, degree = degree, parametric = parametric, : zero-width neighborhood. make span bigger
```

```
## Warning: Computation failed in `stat_smooth()`:
## NA/NaN/Inf in foreign function call (arg 5)
```

```
## Warning in simpleLoess(y, x, w, span, degree = degree, parametric = parametric, : at -0.005
```

```
## Warning in simpleLoess(y, x, w, span, degree = degree, parametric = parametric, : radius 2.5e-05
```

```
## Warning in simpleLoess(y, x, w, span, degree = degree, parametric = parametric, : all data on boundary of neighborhood. make span bigger
```

```
## Warning in simpleLoess(y, x, w, span, degree = degree, parametric = parametric, : pseudoinverse used at -0.005
```

```
## Warning in simpleLoess(y, x, w, span, degree = degree, parametric = parametric, : neighborhood radius 0.005
```

```
## Warning in simpleLoess(y, x, w, span, degree = degree, parametric = parametric, : reciprocal condition number 1
```

```
## Warning in simpleLoess(y, x, w, span, degree = degree, parametric = parametric, : There are other near singularities as well. 1.01
```

```
## Warning in simpleLoess(y, x, w, span, degree = degree, parametric = parametric, : zero-width neighborhood. make span bigger
```

```
## Warning: Computation failed in `stat_smooth()`:
## NA/NaN/Inf in foreign function call (arg 5)
```

```
## Warning in simpleLoess(y, x, w, span, degree = degree, parametric = parametric, : at -0.005
```

```
## Warning in simpleLoess(y, x, w, span, degree = degree, parametric = parametric, : radius 2.5e-05
```

```
## Warning in simpleLoess(y, x, w, span, degree = degree, parametric = parametric, : all data on boundary of neighborhood. make span bigger
```

```
## Warning in simpleLoess(y, x, w, span, degree = degree, parametric = parametric, : pseudoinverse used at -0.005
```

```
## Warning in simpleLoess(y, x, w, span, degree = degree, parametric = parametric, : neighborhood radius 0.005
```

```
## Warning in simpleLoess(y, x, w, span, degree = degree, parametric = parametric, : reciprocal condition number 1
```

```
## Warning in simpleLoess(y, x, w, span, degree = degree, parametric = parametric, : There are other near singularities as well. 1.01
```

```
## Warning in simpleLoess(y, x, w, span, degree = degree, parametric = parametric, : zero-width neighborhood. make span bigger
```

```
## Warning: Computation failed in `stat_smooth()`:
## NA/NaN/Inf in foreign function call (arg 5)
```

```
## Warning in simpleLoess(y, x, w, span, degree = degree, parametric = parametric, : at -0.005
```

```
## Warning in simpleLoess(y, x, w, span, degree = degree, parametric = parametric, : radius 2.5e-05
```

```
## Warning in simpleLoess(y, x, w, span, degree = degree, parametric = parametric, : all data on boundary of neighborhood. make span bigger
```

```
## Warning in simpleLoess(y, x, w, span, degree = degree, parametric = parametric, : pseudoinverse used at -0.005
```

```
## Warning in simpleLoess(y, x, w, span, degree = degree, parametric = parametric, : neighborhood radius 0.005
```

```
## Warning in simpleLoess(y, x, w, span, degree = degree, parametric = parametric, : reciprocal condition number 1
```

```
## Warning in simpleLoess(y, x, w, span, degree = degree, parametric = parametric, : There are other near singularities as well. 1.01
```

```
## Warning in simpleLoess(y, x, w, span, degree = degree, parametric = parametric, : zero-width neighborhood. make span bigger
```

```
## Warning: Computation failed in `stat_smooth()`:
## NA/NaN/Inf in foreign function call (arg 5)
```

```
## Warning in simpleLoess(y, x, w, span, degree = degree, parametric = parametric, : at -0.005
```

```
## Warning in simpleLoess(y, x, w, span, degree = degree, parametric = parametric, : radius 2.5e-05
```

```
## Warning in simpleLoess(y, x, w, span, degree = degree, parametric = parametric, : all data on boundary of neighborhood. make span bigger
```

```
## Warning in simpleLoess(y, x, w, span, degree = degree, parametric = parametric, : pseudoinverse used at -0.005
```

```
## Warning in simpleLoess(y, x, w, span, degree = degree, parametric = parametric, : neighborhood radius 0.005
```

```
## Warning in simpleLoess(y, x, w, span, degree = degree, parametric = parametric, : reciprocal condition number 1
```

```
## Warning in simpleLoess(y, x, w, span, degree = degree, parametric = parametric, : There are other near singularities as well. 1.01
```

```
## Warning in simpleLoess(y, x, w, span, degree = degree, parametric = parametric, : zero-width neighborhood. make span bigger
```

```
## Warning: Computation failed in `stat_smooth()`:
## NA/NaN/Inf in foreign function call (arg 5)
```

```
## Warning in simpleLoess(y, x, w, span, degree = degree, parametric = parametric, : at -0.005
```

```
## Warning in simpleLoess(y, x, w, span, degree = degree, parametric = parametric, : radius 2.5e-05
```

```
## Warning in simpleLoess(y, x, w, span, degree = degree, parametric = parametric, : all data on boundary of neighborhood. make span bigger
```

```
## Warning in simpleLoess(y, x, w, span, degree = degree, parametric = parametric, : pseudoinverse used at -0.005
```

```
## Warning in simpleLoess(y, x, w, span, degree = degree, parametric = parametric, : neighborhood radius 0.005
```

```
## Warning in simpleLoess(y, x, w, span, degree = degree, parametric = parametric, : reciprocal condition number 1
```

```
## Warning in simpleLoess(y, x, w, span, degree = degree, parametric = parametric, : There are other near singularities as well. 1.01
```

```
## Warning in simpleLoess(y, x, w, span, degree = degree, parametric = parametric, : zero-width neighborhood. make span bigger
```

```
## Warning: Computation failed in `stat_smooth()`:
## NA/NaN/Inf in foreign function call (arg 5)
```

```
## Warning in simpleLoess(y, x, w, span, degree = degree, parametric = parametric, : at -0.005
```

```
## Warning in simpleLoess(y, x, w, span, degree = degree, parametric = parametric, : radius 2.5e-05
```

```
## Warning in simpleLoess(y, x, w, span, degree = degree, parametric = parametric, : all data on boundary of neighborhood. make span bigger
```

```
## Warning in simpleLoess(y, x, w, span, degree = degree, parametric = parametric, : pseudoinverse used at -0.005
```

```
## Warning in simpleLoess(y, x, w, span, degree = degree, parametric = parametric, : neighborhood radius 0.005
```

```
## Warning in simpleLoess(y, x, w, span, degree = degree, parametric = parametric, : reciprocal condition number 1
```

```
## Warning in simpleLoess(y, x, w, span, degree = degree, parametric = parametric, : There are other near singularities as well. 1.01
```

```
## Warning in simpleLoess(y, x, w, span, degree = degree, parametric = parametric, : zero-width neighborhood. make span bigger
```

```
## Warning: Computation failed in `stat_smooth()`:
## NA/NaN/Inf in foreign function call (arg 5)
```

```
## Warning in simpleLoess(y, x, w, span, degree = degree, parametric = parametric, : at -0.005
```

```
## Warning in simpleLoess(y, x, w, span, degree = degree, parametric = parametric, : radius 2.5e-05
```

```
## Warning in simpleLoess(y, x, w, span, degree = degree, parametric = parametric, : all data on boundary of neighborhood. make span bigger
```

```
## Warning in simpleLoess(y, x, w, span, degree = degree, parametric = parametric, : pseudoinverse used at -0.005
```

```
## Warning in simpleLoess(y, x, w, span, degree = degree, parametric = parametric, : neighborhood radius 0.005
```

```
## Warning in simpleLoess(y, x, w, span, degree = degree, parametric = parametric, : reciprocal condition number 1
```

```
## Warning in simpleLoess(y, x, w, span, degree = degree, parametric = parametric, : There are other near singularities as well. 1.01
```

```
## Warning in simpleLoess(y, x, w, span, degree = degree, parametric = parametric, : zero-width neighborhood. make span bigger
```

```
## Warning: Computation failed in `stat_smooth()`:
## NA/NaN/Inf in foreign function call (arg 5)
```

```
## Warning in simpleLoess(y, x, w, span, degree = degree, parametric = parametric, : at -0.005
```

```
## Warning in simpleLoess(y, x, w, span, degree = degree, parametric = parametric, : radius 2.5e-05
```

```
## Warning in simpleLoess(y, x, w, span, degree = degree, parametric = parametric, : all data on boundary of neighborhood. make span bigger
```

```
## Warning in simpleLoess(y, x, w, span, degree = degree, parametric = parametric, : pseudoinverse used at -0.005
```

```
## Warning in simpleLoess(y, x, w, span, degree = degree, parametric = parametric, : neighborhood radius 0.005
```

```
## Warning in simpleLoess(y, x, w, span, degree = degree, parametric = parametric, : reciprocal condition number 1
```

```
## Warning in simpleLoess(y, x, w, span, degree = degree, parametric = parametric, : There are other near singularities as well. 1.01
```

```
## Warning in simpleLoess(y, x, w, span, degree = degree, parametric = parametric, : zero-width neighborhood. make span bigger
```

```
## Warning: Computation failed in `stat_smooth()`:
## NA/NaN/Inf in foreign function call (arg 5)
```

```
## Warning in simpleLoess(y, x, w, span, degree = degree, parametric = parametric, : pseudoinverse used at -0.005
```

```
## Warning in simpleLoess(y, x, w, span, degree = degree, parametric = parametric, : neighborhood radius 1.005
```

```
## Warning in simpleLoess(y, x, w, span, degree = degree, parametric = parametric, : reciprocal condition number 0
```

```
## Warning in simpleLoess(y, x, w, span, degree = degree, parametric = parametric, : There are other near singularities as well. 1.01
```

```
## Warning in predLoess(object$y, object$x, newx = if (is.null(newdata)) object$x else if (is.data.frame(newdata)) as.matrix(model.frame(delete.response(terms(object)), : pseudoinverse used at -0.005
```

```
## Warning in predLoess(object$y, object$x, newx = if (is.null(newdata)) object$x else if (is.data.frame(newdata)) as.matrix(model.frame(delete.response(terms(object)), : neighborhood radius 1.005
```

```
## Warning in predLoess(object$y, object$x, newx = if (is.null(newdata)) object$x else if (is.data.frame(newdata)) as.matrix(model.frame(delete.response(terms(object)), : reciprocal condition number 0
```

```
## Warning in predLoess(object$y, object$x, newx = if (is.null(newdata)) object$x else if (is.data.frame(newdata)) as.matrix(model.frame(delete.response(terms(object)), : There are other near
## singularities as well. 1.01
```

```
## Warning in simpleLoess(y, x, w, span, degree = degree, parametric = parametric, : at -0.005
```

```
## Warning in simpleLoess(y, x, w, span, degree = degree, parametric = parametric, : radius 2.5e-05
```

```
## Warning in simpleLoess(y, x, w, span, degree = degree, parametric = parametric, : all data on boundary of neighborhood. make span bigger
```

```
## Warning in simpleLoess(y, x, w, span, degree = degree, parametric = parametric, : pseudoinverse used at -0.005
```

```
## Warning in simpleLoess(y, x, w, span, degree = degree, parametric = parametric, : neighborhood radius 0.005
```

```
## Warning in simpleLoess(y, x, w, span, degree = degree, parametric = parametric, : reciprocal condition number 1
```

```
## Warning in simpleLoess(y, x, w, span, degree = degree, parametric = parametric, : There are other near singularities as well. 1.01
```

```
## Warning in simpleLoess(y, x, w, span, degree = degree, parametric = parametric, : zero-width neighborhood. make span bigger
```

```
## Warning: Computation failed in `stat_smooth()`:
## NA/NaN/Inf in foreign function call (arg 5)
```

```
## Warning in simpleLoess(y, x, w, span, degree = degree, parametric = parametric, : at -0.005
```

```
## Warning in simpleLoess(y, x, w, span, degree = degree, parametric = parametric, : radius 2.5e-05
```

```
## Warning in simpleLoess(y, x, w, span, degree = degree, parametric = parametric, : all data on boundary of neighborhood. make span bigger
```

```
## Warning in simpleLoess(y, x, w, span, degree = degree, parametric = parametric, : pseudoinverse used at -0.005
```

```
## Warning in simpleLoess(y, x, w, span, degree = degree, parametric = parametric, : neighborhood radius 0.005
```

```
## Warning in simpleLoess(y, x, w, span, degree = degree, parametric = parametric, : reciprocal condition number 1
```

```
## Warning in simpleLoess(y, x, w, span, degree = degree, parametric = parametric, : There are other near singularities as well. 1.01
```

```
## Warning in simpleLoess(y, x, w, span, degree = degree, parametric = parametric, : zero-width neighborhood. make span bigger
```

```
## Warning: Computation failed in `stat_smooth()`:
## NA/NaN/Inf in foreign function call (arg 5)
```

```
## Warning in simpleLoess(y, x, w, span, degree = degree, parametric = parametric, : at -0.005
```

```
## Warning in simpleLoess(y, x, w, span, degree = degree, parametric = parametric, : radius 2.5e-05
```

```
## Warning in simpleLoess(y, x, w, span, degree = degree, parametric = parametric, : all data on boundary of neighborhood. make span bigger
```

```
## Warning in simpleLoess(y, x, w, span, degree = degree, parametric = parametric, : pseudoinverse used at -0.005
```

```
## Warning in simpleLoess(y, x, w, span, degree = degree, parametric = parametric, : neighborhood radius 0.005
```

```
## Warning in simpleLoess(y, x, w, span, degree = degree, parametric = parametric, : reciprocal condition number 1
```

```
## Warning in simpleLoess(y, x, w, span, degree = degree, parametric = parametric, : There are other near singularities as well. 1.01
```

```
## Warning in simpleLoess(y, x, w, span, degree = degree, parametric = parametric, : zero-width neighborhood. make span bigger
```

```
## Warning: Computation failed in `stat_smooth()`:
## NA/NaN/Inf in foreign function call (arg 5)
```

```
## Warning in simpleLoess(y, x, w, span, degree = degree, parametric = parametric, : at -0.005
```

```
## Warning in simpleLoess(y, x, w, span, degree = degree, parametric = parametric, : radius 2.5e-05
```

```
## Warning in simpleLoess(y, x, w, span, degree = degree, parametric = parametric, : all data on boundary of neighborhood. make span bigger
```

```
## Warning in simpleLoess(y, x, w, span, degree = degree, parametric = parametric, : pseudoinverse used at -0.005
```

```
## Warning in simpleLoess(y, x, w, span, degree = degree, parametric = parametric, : neighborhood radius 0.005
```

```
## Warning in simpleLoess(y, x, w, span, degree = degree, parametric = parametric, : reciprocal condition number 1
```

```
## Warning in simpleLoess(y, x, w, span, degree = degree, parametric = parametric, : There are other near singularities as well. 1.01
```

```
## Warning in simpleLoess(y, x, w, span, degree = degree, parametric = parametric, : zero-width neighborhood. make span bigger
```

```
## Warning: Computation failed in `stat_smooth()`:
## NA/NaN/Inf in foreign function call (arg 5)
```

```
## Warning in simpleLoess(y, x, w, span, degree = degree, parametric = parametric, : at -0.005
```

```
## Warning in simpleLoess(y, x, w, span, degree = degree, parametric = parametric, : radius 2.5e-05
```

```
## Warning in simpleLoess(y, x, w, span, degree = degree, parametric = parametric, : all data on boundary of neighborhood. make span bigger
```

```
## Warning in simpleLoess(y, x, w, span, degree = degree, parametric = parametric, : pseudoinverse used at -0.005
```

```
## Warning in simpleLoess(y, x, w, span, degree = degree, parametric = parametric, : neighborhood radius 0.005
```

```
## Warning in simpleLoess(y, x, w, span, degree = degree, parametric = parametric, : reciprocal condition number 1
```

```
## Warning in simpleLoess(y, x, w, span, degree = degree, parametric = parametric, : There are other near singularities as well. 1.01
```

```
## Warning in simpleLoess(y, x, w, span, degree = degree, parametric = parametric, : zero-width neighborhood. make span bigger
```

```
## Warning: Computation failed in `stat_smooth()`:
## NA/NaN/Inf in foreign function call (arg 5)
```

```
## Warning in simpleLoess(y, x, w, span, degree = degree, parametric = parametric, : pseudoinverse used at -0.005
```

```
## Warning in simpleLoess(y, x, w, span, degree = degree, parametric = parametric, : neighborhood radius 1.005
```

```
## Warning in simpleLoess(y, x, w, span, degree = degree, parametric = parametric, : reciprocal condition number 0
```

```
## Warning in simpleLoess(y, x, w, span, degree = degree, parametric = parametric, : There are other near singularities as well. 1.01
```

```
## Warning in predLoess(object$y, object$x, newx = if (is.null(newdata)) object$x else if (is.data.frame(newdata)) as.matrix(model.frame(delete.response(terms(object)), : pseudoinverse used at -0.005
```

```
## Warning in predLoess(object$y, object$x, newx = if (is.null(newdata)) object$x else if (is.data.frame(newdata)) as.matrix(model.frame(delete.response(terms(object)), : neighborhood radius 1.005
```

```
## Warning in predLoess(object$y, object$x, newx = if (is.null(newdata)) object$x else if (is.data.frame(newdata)) as.matrix(model.frame(delete.response(terms(object)), : reciprocal condition number 0
```

```
## Warning in predLoess(object$y, object$x, newx = if (is.null(newdata)) object$x else if (is.data.frame(newdata)) as.matrix(model.frame(delete.response(terms(object)), : There are other near
## singularities as well. 1.01
```

```
## Warning in simpleLoess(y, x, w, span, degree = degree, parametric = parametric, : at -0.005
```

```
## Warning in simpleLoess(y, x, w, span, degree = degree, parametric = parametric, : radius 2.5e-05
```

```
## Warning in simpleLoess(y, x, w, span, degree = degree, parametric = parametric, : all data on boundary of neighborhood. make span bigger
```

```
## Warning in simpleLoess(y, x, w, span, degree = degree, parametric = parametric, : pseudoinverse used at -0.005
```

```
## Warning in simpleLoess(y, x, w, span, degree = degree, parametric = parametric, : neighborhood radius 0.005
```

```
## Warning in simpleLoess(y, x, w, span, degree = degree, parametric = parametric, : reciprocal condition number 1
```

```
## Warning in simpleLoess(y, x, w, span, degree = degree, parametric = parametric, : There are other near singularities as well. 1.01
```

```
## Warning in simpleLoess(y, x, w, span, degree = degree, parametric = parametric, : zero-width neighborhood. make span bigger
```

```
## Warning: Computation failed in `stat_smooth()`:
## NA/NaN/Inf in foreign function call (arg 5)
```

```
## Warning in simpleLoess(y, x, w, span, degree = degree, parametric = parametric, : at -0.005
```

```
## Warning in simpleLoess(y, x, w, span, degree = degree, parametric = parametric, : radius 2.5e-05
```

```
## Warning in simpleLoess(y, x, w, span, degree = degree, parametric = parametric, : all data on boundary of neighborhood. make span bigger
```

```
## Warning in simpleLoess(y, x, w, span, degree = degree, parametric = parametric, : pseudoinverse used at -0.005
```

```
## Warning in simpleLoess(y, x, w, span, degree = degree, parametric = parametric, : neighborhood radius 0.005
```

```
## Warning in simpleLoess(y, x, w, span, degree = degree, parametric = parametric, : reciprocal condition number 1
```

```
## Warning in simpleLoess(y, x, w, span, degree = degree, parametric = parametric, : There are other near singularities as well. 1.01
```

```
## Warning in simpleLoess(y, x, w, span, degree = degree, parametric = parametric, : zero-width neighborhood. make span bigger
```

```
## Warning: Computation failed in `stat_smooth()`:
## NA/NaN/Inf in foreign function call (arg 5)
```

```
## Warning in simpleLoess(y, x, w, span, degree = degree, parametric = parametric, : at -0.005
```

```
## Warning in simpleLoess(y, x, w, span, degree = degree, parametric = parametric, : radius 2.5e-05
```

```
## Warning in simpleLoess(y, x, w, span, degree = degree, parametric = parametric, : all data on boundary of neighborhood. make span bigger
```

```
## Warning in simpleLoess(y, x, w, span, degree = degree, parametric = parametric, : pseudoinverse used at -0.005
```

```
## Warning in simpleLoess(y, x, w, span, degree = degree, parametric = parametric, : neighborhood radius 0.005
```

```
## Warning in simpleLoess(y, x, w, span, degree = degree, parametric = parametric, : reciprocal condition number 1
```

```
## Warning in simpleLoess(y, x, w, span, degree = degree, parametric = parametric, : There are other near singularities as well. 1.01
```

```
## Warning in simpleLoess(y, x, w, span, degree = degree, parametric = parametric, : zero-width neighborhood. make span bigger
```

```
## Warning: Computation failed in `stat_smooth()`:
## NA/NaN/Inf in foreign function call (arg 5)
```

```
## Warning in simpleLoess(y, x, w, span, degree = degree, parametric = parametric, : at -0.005
```

```
## Warning in simpleLoess(y, x, w, span, degree = degree, parametric = parametric, : radius 2.5e-05
```

```
## Warning in simpleLoess(y, x, w, span, degree = degree, parametric = parametric, : all data on boundary of neighborhood. make span bigger
```

```
## Warning in simpleLoess(y, x, w, span, degree = degree, parametric = parametric, : pseudoinverse used at -0.005
```

```
## Warning in simpleLoess(y, x, w, span, degree = degree, parametric = parametric, : neighborhood radius 0.005
```

```
## Warning in simpleLoess(y, x, w, span, degree = degree, parametric = parametric, : reciprocal condition number 1
```

```
## Warning in simpleLoess(y, x, w, span, degree = degree, parametric = parametric, : There are other near singularities as well. 1.01
```

```
## Warning in simpleLoess(y, x, w, span, degree = degree, parametric = parametric, : zero-width neighborhood. make span bigger
```

```
## Warning: Computation failed in `stat_smooth()`:
## NA/NaN/Inf in foreign function call (arg 5)
```

```
## Warning in simpleLoess(y, x, w, span, degree = degree, parametric = parametric, : at -0.005
```

```
## Warning in simpleLoess(y, x, w, span, degree = degree, parametric = parametric, : radius 2.5e-05
```

```
## Warning in simpleLoess(y, x, w, span, degree = degree, parametric = parametric, : all data on boundary of neighborhood. make span bigger
```

```
## Warning in simpleLoess(y, x, w, span, degree = degree, parametric = parametric, : pseudoinverse used at -0.005
```

```
## Warning in simpleLoess(y, x, w, span, degree = degree, parametric = parametric, : neighborhood radius 0.005
```

```
## Warning in simpleLoess(y, x, w, span, degree = degree, parametric = parametric, : reciprocal condition number 1
```

```
## Warning in simpleLoess(y, x, w, span, degree = degree, parametric = parametric, : There are other near singularities as well. 1.01
```

```
## Warning in simpleLoess(y, x, w, span, degree = degree, parametric = parametric, : zero-width neighborhood. make span bigger
```

```
## Warning: Computation failed in `stat_smooth()`:
## NA/NaN/Inf in foreign function call (arg 5)
```

```
## Warning in simpleLoess(y, x, w, span, degree = degree, parametric = parametric, : at -0.005
```

```
## Warning in simpleLoess(y, x, w, span, degree = degree, parametric = parametric, : radius 2.5e-05
```

```
## Warning in simpleLoess(y, x, w, span, degree = degree, parametric = parametric, : all data on boundary of neighborhood. make span bigger
```

```
## Warning in simpleLoess(y, x, w, span, degree = degree, parametric = parametric, : pseudoinverse used at -0.005
```

```
## Warning in simpleLoess(y, x, w, span, degree = degree, parametric = parametric, : neighborhood radius 0.005
```

```
## Warning in simpleLoess(y, x, w, span, degree = degree, parametric = parametric, : reciprocal condition number 1
```

```
## Warning in simpleLoess(y, x, w, span, degree = degree, parametric = parametric, : There are other near singularities as well. 1.01
```

```
## Warning in simpleLoess(y, x, w, span, degree = degree, parametric = parametric, : zero-width neighborhood. make span bigger
```

```
## Warning: Computation failed in `stat_smooth()`:
## NA/NaN/Inf in foreign function call (arg 5)
```

```
## Warning in simpleLoess(y, x, w, span, degree = degree, parametric = parametric, : at -0.005
```

```
## Warning in simpleLoess(y, x, w, span, degree = degree, parametric = parametric, : radius 2.5e-05
```

```
## Warning in simpleLoess(y, x, w, span, degree = degree, parametric = parametric, : all data on boundary of neighborhood. make span bigger
```

```
## Warning in simpleLoess(y, x, w, span, degree = degree, parametric = parametric, : pseudoinverse used at -0.005
```

```
## Warning in simpleLoess(y, x, w, span, degree = degree, parametric = parametric, : neighborhood radius 0.005
```

```
## Warning in simpleLoess(y, x, w, span, degree = degree, parametric = parametric, : reciprocal condition number 1
```

```
## Warning in simpleLoess(y, x, w, span, degree = degree, parametric = parametric, : There are other near singularities as well. 1.01
```

```
## Warning in simpleLoess(y, x, w, span, degree = degree, parametric = parametric, : zero-width neighborhood. make span bigger
```

```
## Warning: Computation failed in `stat_smooth()`:
## NA/NaN/Inf in foreign function call (arg 5)
```

```
## Warning in simpleLoess(y, x, w, span, degree = degree, parametric = parametric, : at -0.005
```

```
## Warning in simpleLoess(y, x, w, span, degree = degree, parametric = parametric, : radius 2.5e-05
```

```
## Warning in simpleLoess(y, x, w, span, degree = degree, parametric = parametric, : all data on boundary of neighborhood. make span bigger
```

```
## Warning in simpleLoess(y, x, w, span, degree = degree, parametric = parametric, : pseudoinverse used at -0.005
```

```
## Warning in simpleLoess(y, x, w, span, degree = degree, parametric = parametric, : neighborhood radius 0.005
```

```
## Warning in simpleLoess(y, x, w, span, degree = degree, parametric = parametric, : reciprocal condition number 1
```

```
## Warning in simpleLoess(y, x, w, span, degree = degree, parametric = parametric, : There are other near singularities as well. 1.01
```

```
## Warning in simpleLoess(y, x, w, span, degree = degree, parametric = parametric, : zero-width neighborhood. make span bigger
```

```
## Warning: Computation failed in `stat_smooth()`:
## NA/NaN/Inf in foreign function call (arg 5)
```

```
## Warning in simpleLoess(y, x, w, span, degree = degree, parametric = parametric, : pseudoinverse used at -0.005
```

```
## Warning in simpleLoess(y, x, w, span, degree = degree, parametric = parametric, : neighborhood radius 1.005
```

```
## Warning in simpleLoess(y, x, w, span, degree = degree, parametric = parametric, : reciprocal condition number 0
```

```
## Warning in simpleLoess(y, x, w, span, degree = degree, parametric = parametric, : There are other near singularities as well. 1.01
```

```
## Warning in predLoess(object$y, object$x, newx = if (is.null(newdata)) object$x else if (is.data.frame(newdata)) as.matrix(model.frame(delete.response(terms(object)), : pseudoinverse used at -0.005
```

```
## Warning in predLoess(object$y, object$x, newx = if (is.null(newdata)) object$x else if (is.data.frame(newdata)) as.matrix(model.frame(delete.response(terms(object)), : neighborhood radius 1.005
```

```
## Warning in predLoess(object$y, object$x, newx = if (is.null(newdata)) object$x else if (is.data.frame(newdata)) as.matrix(model.frame(delete.response(terms(object)), : reciprocal condition number 0
```

```
## Warning in predLoess(object$y, object$x, newx = if (is.null(newdata)) object$x else if (is.data.frame(newdata)) as.matrix(model.frame(delete.response(terms(object)), : There are other near
## singularities as well. 1.01
```

```
## Warning in simpleLoess(y, x, w, span, degree = degree, parametric = parametric, : at -0.005
```

```
## Warning in simpleLoess(y, x, w, span, degree = degree, parametric = parametric, : radius 2.5e-05
```

```
## Warning in simpleLoess(y, x, w, span, degree = degree, parametric = parametric, : all data on boundary of neighborhood. make span bigger
```

```
## Warning in simpleLoess(y, x, w, span, degree = degree, parametric = parametric, : pseudoinverse used at -0.005
```

```
## Warning in simpleLoess(y, x, w, span, degree = degree, parametric = parametric, : neighborhood radius 0.005
```

```
## Warning in simpleLoess(y, x, w, span, degree = degree, parametric = parametric, : reciprocal condition number 1
```

```
## Warning in simpleLoess(y, x, w, span, degree = degree, parametric = parametric, : There are other near singularities as well. 1.01
```

```
## Warning in simpleLoess(y, x, w, span, degree = degree, parametric = parametric, : zero-width neighborhood. make span bigger
```

```
## Warning: Computation failed in `stat_smooth()`:
## NA/NaN/Inf in foreign function call (arg 5)
```

```
## Warning in simpleLoess(y, x, w, span, degree = degree, parametric = parametric, : at -0.005
```

```
## Warning in simpleLoess(y, x, w, span, degree = degree, parametric = parametric, : radius 2.5e-05
```

```
## Warning in simpleLoess(y, x, w, span, degree = degree, parametric = parametric, : all data on boundary of neighborhood. make span bigger
```

```
## Warning in simpleLoess(y, x, w, span, degree = degree, parametric = parametric, : pseudoinverse used at -0.005
```

```
## Warning in simpleLoess(y, x, w, span, degree = degree, parametric = parametric, : neighborhood radius 0.005
```

```
## Warning in simpleLoess(y, x, w, span, degree = degree, parametric = parametric, : reciprocal condition number 1
```

```
## Warning in simpleLoess(y, x, w, span, degree = degree, parametric = parametric, : There are other near singularities as well. 1.01
```

```
## Warning in simpleLoess(y, x, w, span, degree = degree, parametric = parametric, : zero-width neighborhood. make span bigger
```

```
## Warning: Computation failed in `stat_smooth()`:
## NA/NaN/Inf in foreign function call (arg 5)
```

```
## Warning in simpleLoess(y, x, w, span, degree = degree, parametric = parametric, : at -0.005
```

```
## Warning in simpleLoess(y, x, w, span, degree = degree, parametric = parametric, : radius 2.5e-05
```

```
## Warning in simpleLoess(y, x, w, span, degree = degree, parametric = parametric, : all data on boundary of neighborhood. make span bigger
```

```
## Warning in simpleLoess(y, x, w, span, degree = degree, parametric = parametric, : pseudoinverse used at -0.005
```

```
## Warning in simpleLoess(y, x, w, span, degree = degree, parametric = parametric, : neighborhood radius 0.005
```

```
## Warning in simpleLoess(y, x, w, span, degree = degree, parametric = parametric, : reciprocal condition number 1
```

```
## Warning in simpleLoess(y, x, w, span, degree = degree, parametric = parametric, : There are other near singularities as well. 1.01
```

```
## Warning in simpleLoess(y, x, w, span, degree = degree, parametric = parametric, : zero-width neighborhood. make span bigger
```

```
## Warning: Computation failed in `stat_smooth()`:
## NA/NaN/Inf in foreign function call (arg 5)
```

```
## Warning in simpleLoess(y, x, w, span, degree = degree, parametric = parametric, : at -0.005
```

```
## Warning in simpleLoess(y, x, w, span, degree = degree, parametric = parametric, : radius 2.5e-05
```

```
## Warning in simpleLoess(y, x, w, span, degree = degree, parametric = parametric, : all data on boundary of neighborhood. make span bigger
```

```
## Warning in simpleLoess(y, x, w, span, degree = degree, parametric = parametric, : pseudoinverse used at -0.005
```

```
## Warning in simpleLoess(y, x, w, span, degree = degree, parametric = parametric, : neighborhood radius 0.005
```

```
## Warning in simpleLoess(y, x, w, span, degree = degree, parametric = parametric, : reciprocal condition number 1
```

```
## Warning in simpleLoess(y, x, w, span, degree = degree, parametric = parametric, : There are other near singularities as well. 1.01
```

```
## Warning in simpleLoess(y, x, w, span, degree = degree, parametric = parametric, : zero-width neighborhood. make span bigger
```

```
## Warning: Computation failed in `stat_smooth()`:
## NA/NaN/Inf in foreign function call (arg 5)
```

```
## Warning in simpleLoess(y, x, w, span, degree = degree, parametric = parametric, : at -0.005
```

```
## Warning in simpleLoess(y, x, w, span, degree = degree, parametric = parametric, : radius 2.5e-05
```

```
## Warning in simpleLoess(y, x, w, span, degree = degree, parametric = parametric, : all data on boundary of neighborhood. make span bigger
```

```
## Warning in simpleLoess(y, x, w, span, degree = degree, parametric = parametric, : pseudoinverse used at -0.005
```

```
## Warning in simpleLoess(y, x, w, span, degree = degree, parametric = parametric, : neighborhood radius 0.005
```

```
## Warning in simpleLoess(y, x, w, span, degree = degree, parametric = parametric, : reciprocal condition number 1
```

```
## Warning in simpleLoess(y, x, w, span, degree = degree, parametric = parametric, : There are other near singularities as well. 1.01
```

```
## Warning in simpleLoess(y, x, w, span, degree = degree, parametric = parametric, : zero-width neighborhood. make span bigger
```

```
## Warning: Computation failed in `stat_smooth()`:
## NA/NaN/Inf in foreign function call (arg 5)
```

```
## Warning in simpleLoess(y, x, w, span, degree = degree, parametric = parametric, : at -0.005
```

```
## Warning in simpleLoess(y, x, w, span, degree = degree, parametric = parametric, : radius 2.5e-05
```

```
## Warning in simpleLoess(y, x, w, span, degree = degree, parametric = parametric, : all data on boundary of neighborhood. make span bigger
```

```
## Warning in simpleLoess(y, x, w, span, degree = degree, parametric = parametric, : pseudoinverse used at -0.005
```

```
## Warning in simpleLoess(y, x, w, span, degree = degree, parametric = parametric, : neighborhood radius 0.005
```

```
## Warning in simpleLoess(y, x, w, span, degree = degree, parametric = parametric, : reciprocal condition number 1
```

```
## Warning in simpleLoess(y, x, w, span, degree = degree, parametric = parametric, : There are other near singularities as well. 1.01
```

```
## Warning in simpleLoess(y, x, w, span, degree = degree, parametric = parametric, : zero-width neighborhood. make span bigger
```

```
## Warning: Computation failed in `stat_smooth()`:
## NA/NaN/Inf in foreign function call (arg 5)
```

```
## Warning in simpleLoess(y, x, w, span, degree = degree, parametric = parametric, : at -0.005
```

```
## Warning in simpleLoess(y, x, w, span, degree = degree, parametric = parametric, : radius 2.5e-05
```

```
## Warning in simpleLoess(y, x, w, span, degree = degree, parametric = parametric, : all data on boundary of neighborhood. make span bigger
```

```
## Warning in simpleLoess(y, x, w, span, degree = degree, parametric = parametric, : pseudoinverse used at -0.005
```

```
## Warning in simpleLoess(y, x, w, span, degree = degree, parametric = parametric, : neighborhood radius 0.005
```

```
## Warning in simpleLoess(y, x, w, span, degree = degree, parametric = parametric, : reciprocal condition number 1
```

```
## Warning in simpleLoess(y, x, w, span, degree = degree, parametric = parametric, : There are other near singularities as well. 1.01
```

```
## Warning in simpleLoess(y, x, w, span, degree = degree, parametric = parametric, : zero-width neighborhood. make span bigger
```

```
## Warning: Computation failed in `stat_smooth()`:
## NA/NaN/Inf in foreign function call (arg 5)
```

```
## Warning in simpleLoess(y, x, w, span, degree = degree, parametric = parametric, : at -0.005
```

```
## Warning in simpleLoess(y, x, w, span, degree = degree, parametric = parametric, : radius 2.5e-05
```

```
## Warning in simpleLoess(y, x, w, span, degree = degree, parametric = parametric, : all data on boundary of neighborhood. make span bigger
```

```
## Warning in simpleLoess(y, x, w, span, degree = degree, parametric = parametric, : pseudoinverse used at -0.005
```

```
## Warning in simpleLoess(y, x, w, span, degree = degree, parametric = parametric, : neighborhood radius 0.005
```

```
## Warning in simpleLoess(y, x, w, span, degree = degree, parametric = parametric, : reciprocal condition number 1
```

```
## Warning in simpleLoess(y, x, w, span, degree = degree, parametric = parametric, : There are other near singularities as well. 1.01
```

```
## Warning in simpleLoess(y, x, w, span, degree = degree, parametric = parametric, : zero-width neighborhood. make span bigger
```

```
## Warning: Computation failed in `stat_smooth()`:
## NA/NaN/Inf in foreign function call (arg 5)
```

```
## Warning in simpleLoess(y, x, w, span, degree = degree, parametric = parametric, : at -0.005
```

```
## Warning in simpleLoess(y, x, w, span, degree = degree, parametric = parametric, : radius 2.5e-05
```

```
## Warning in simpleLoess(y, x, w, span, degree = degree, parametric = parametric, : all data on boundary of neighborhood. make span bigger
```

```
## Warning in simpleLoess(y, x, w, span, degree = degree, parametric = parametric, : pseudoinverse used at -0.005
```

```
## Warning in simpleLoess(y, x, w, span, degree = degree, parametric = parametric, : neighborhood radius 0.005
```

```
## Warning in simpleLoess(y, x, w, span, degree = degree, parametric = parametric, : reciprocal condition number 1
```

```
## Warning in simpleLoess(y, x, w, span, degree = degree, parametric = parametric, : There are other near singularities as well. 1.01
```

```
## Warning in simpleLoess(y, x, w, span, degree = degree, parametric = parametric, : zero-width neighborhood. make span bigger
```

```
## Warning: Computation failed in `stat_smooth()`:
## NA/NaN/Inf in foreign function call (arg 5)
```

```
## Warning in simpleLoess(y, x, w, span, degree = degree, parametric = parametric, : pseudoinverse used at -0.005
```

```
## Warning in simpleLoess(y, x, w, span, degree = degree, parametric = parametric, : neighborhood radius 1.005
```

```
## Warning in simpleLoess(y, x, w, span, degree = degree, parametric = parametric, : reciprocal condition number 0
```

```
## Warning in simpleLoess(y, x, w, span, degree = degree, parametric = parametric, : There are other near singularities as well. 1.01
```

```
## Warning in predLoess(object$y, object$x, newx = if (is.null(newdata)) object$x else if (is.data.frame(newdata)) as.matrix(model.frame(delete.response(terms(object)), : pseudoinverse used at -0.005
```

```
## Warning in predLoess(object$y, object$x, newx = if (is.null(newdata)) object$x else if (is.data.frame(newdata)) as.matrix(model.frame(delete.response(terms(object)), : neighborhood radius 1.005
```

```
## Warning in predLoess(object$y, object$x, newx = if (is.null(newdata)) object$x else if (is.data.frame(newdata)) as.matrix(model.frame(delete.response(terms(object)), : reciprocal condition number 0
```

```
## Warning in predLoess(object$y, object$x, newx = if (is.null(newdata)) object$x else if (is.data.frame(newdata)) as.matrix(model.frame(delete.response(terms(object)), : There are other near
## singularities as well. 1.01
```

```
## Warning in simpleLoess(y, x, w, span, degree = degree, parametric = parametric, : at -0.005
```

```
## Warning in simpleLoess(y, x, w, span, degree = degree, parametric = parametric, : radius 2.5e-05
```

```
## Warning in simpleLoess(y, x, w, span, degree = degree, parametric = parametric, : all data on boundary of neighborhood. make span bigger
```

```
## Warning in simpleLoess(y, x, w, span, degree = degree, parametric = parametric, : pseudoinverse used at -0.005
```

```
## Warning in simpleLoess(y, x, w, span, degree = degree, parametric = parametric, : neighborhood radius 0.005
```

```
## Warning in simpleLoess(y, x, w, span, degree = degree, parametric = parametric, : reciprocal condition number 1
```

```
## Warning in simpleLoess(y, x, w, span, degree = degree, parametric = parametric, : There are other near singularities as well. 1.01
```

```
## Warning in simpleLoess(y, x, w, span, degree = degree, parametric = parametric, : zero-width neighborhood. make span bigger
```

```
## Warning: Computation failed in `stat_smooth()`:
## NA/NaN/Inf in foreign function call (arg 5)
```

```
## Warning in simpleLoess(y, x, w, span, degree = degree, parametric = parametric, : at -0.005
```

```
## Warning in simpleLoess(y, x, w, span, degree = degree, parametric = parametric, : radius 2.5e-05
```

```
## Warning in simpleLoess(y, x, w, span, degree = degree, parametric = parametric, : all data on boundary of neighborhood. make span bigger
```

```
## Warning in simpleLoess(y, x, w, span, degree = degree, parametric = parametric, : pseudoinverse used at -0.005
```

```
## Warning in simpleLoess(y, x, w, span, degree = degree, parametric = parametric, : neighborhood radius 0.005
```

```
## Warning in simpleLoess(y, x, w, span, degree = degree, parametric = parametric, : reciprocal condition number 1
```

```
## Warning in simpleLoess(y, x, w, span, degree = degree, parametric = parametric, : There are other near singularities as well. 1.01
```

```
## Warning in simpleLoess(y, x, w, span, degree = degree, parametric = parametric, : zero-width neighborhood. make span bigger
```

```
## Warning: Computation failed in `stat_smooth()`:
## NA/NaN/Inf in foreign function call (arg 5)
```

```
## Warning in simpleLoess(y, x, w, span, degree = degree, parametric = parametric, : at -0.005
```

```
## Warning in simpleLoess(y, x, w, span, degree = degree, parametric = parametric, : radius 2.5e-05
```

```
## Warning in simpleLoess(y, x, w, span, degree = degree, parametric = parametric, : all data on boundary of neighborhood. make span bigger
```

```
## Warning in simpleLoess(y, x, w, span, degree = degree, parametric = parametric, : pseudoinverse used at -0.005
```

```
## Warning in simpleLoess(y, x, w, span, degree = degree, parametric = parametric, : neighborhood radius 0.005
```

```
## Warning in simpleLoess(y, x, w, span, degree = degree, parametric = parametric, : reciprocal condition number 1
```

```
## Warning in simpleLoess(y, x, w, span, degree = degree, parametric = parametric, : There are other near singularities as well. 1.01
```

```
## Warning in simpleLoess(y, x, w, span, degree = degree, parametric = parametric, : zero-width neighborhood. make span bigger
```

```
## Warning: Computation failed in `stat_smooth()`:
## NA/NaN/Inf in foreign function call (arg 5)
```

```
## Warning in simpleLoess(y, x, w, span, degree = degree, parametric = parametric, : at -0.005
```

```
## Warning in simpleLoess(y, x, w, span, degree = degree, parametric = parametric, : radius 2.5e-05
```

```
## Warning in simpleLoess(y, x, w, span, degree = degree, parametric = parametric, : all data on boundary of neighborhood. make span bigger
```

```
## Warning in simpleLoess(y, x, w, span, degree = degree, parametric = parametric, : pseudoinverse used at -0.005
```

```
## Warning in simpleLoess(y, x, w, span, degree = degree, parametric = parametric, : neighborhood radius 0.005
```

```
## Warning in simpleLoess(y, x, w, span, degree = degree, parametric = parametric, : reciprocal condition number 1
```

```
## Warning in simpleLoess(y, x, w, span, degree = degree, parametric = parametric, : There are other near singularities as well. 1.01
```

```
## Warning in simpleLoess(y, x, w, span, degree = degree, parametric = parametric, : zero-width neighborhood. make span bigger
```

```
## Warning: Computation failed in `stat_smooth()`:
## NA/NaN/Inf in foreign function call (arg 5)
```

```
## Warning in simpleLoess(y, x, w, span, degree = degree, parametric = parametric, : at -0.005
```

```
## Warning in simpleLoess(y, x, w, span, degree = degree, parametric = parametric, : radius 2.5e-05
```

```
## Warning in simpleLoess(y, x, w, span, degree = degree, parametric = parametric, : all data on boundary of neighborhood. make span bigger
```

```
## Warning in simpleLoess(y, x, w, span, degree = degree, parametric = parametric, : pseudoinverse used at -0.005
```

```
## Warning in simpleLoess(y, x, w, span, degree = degree, parametric = parametric, : neighborhood radius 0.005
```

```
## Warning in simpleLoess(y, x, w, span, degree = degree, parametric = parametric, : reciprocal condition number 1
```

```
## Warning in simpleLoess(y, x, w, span, degree = degree, parametric = parametric, : There are other near singularities as well. 1.01
```

```
## Warning in simpleLoess(y, x, w, span, degree = degree, parametric = parametric, : zero-width neighborhood. make span bigger
```

```
## Warning: Computation failed in `stat_smooth()`:
## NA/NaN/Inf in foreign function call (arg 5)
```

```
## Warning in simpleLoess(y, x, w, span, degree = degree, parametric = parametric, : at -0.005
```

```
## Warning in simpleLoess(y, x, w, span, degree = degree, parametric = parametric, : radius 2.5e-05
```

```
## Warning in simpleLoess(y, x, w, span, degree = degree, parametric = parametric, : all data on boundary of neighborhood. make span bigger
```

```
## Warning in simpleLoess(y, x, w, span, degree = degree, parametric = parametric, : pseudoinverse used at -0.005
```

```
## Warning in simpleLoess(y, x, w, span, degree = degree, parametric = parametric, : neighborhood radius 0.005
```

```
## Warning in simpleLoess(y, x, w, span, degree = degree, parametric = parametric, : reciprocal condition number 1
```

```
## Warning in simpleLoess(y, x, w, span, degree = degree, parametric = parametric, : There are other near singularities as well. 1.01
```

```
## Warning in simpleLoess(y, x, w, span, degree = degree, parametric = parametric, : zero-width neighborhood. make span bigger
```

```
## Warning: Computation failed in `stat_smooth()`:
## NA/NaN/Inf in foreign function call (arg 5)
```

```
## Warning in simpleLoess(y, x, w, span, degree = degree, parametric = parametric, : at -0.005
```

```
## Warning in simpleLoess(y, x, w, span, degree = degree, parametric = parametric, : radius 2.5e-05
```

```
## Warning in simpleLoess(y, x, w, span, degree = degree, parametric = parametric, : all data on boundary of neighborhood. make span bigger
```

```
## Warning in simpleLoess(y, x, w, span, degree = degree, parametric = parametric, : pseudoinverse used at -0.005
```

```
## Warning in simpleLoess(y, x, w, span, degree = degree, parametric = parametric, : neighborhood radius 0.005
```

```
## Warning in simpleLoess(y, x, w, span, degree = degree, parametric = parametric, : reciprocal condition number 1
```

```
## Warning in simpleLoess(y, x, w, span, degree = degree, parametric = parametric, : There are other near singularities as well. 1.01
```

```
## Warning in simpleLoess(y, x, w, span, degree = degree, parametric = parametric, : zero-width neighborhood. make span bigger
```

```
## Warning: Computation failed in `stat_smooth()`:
## NA/NaN/Inf in foreign function call (arg 5)
```

```
## Warning in simpleLoess(y, x, w, span, degree = degree, parametric = parametric, : pseudoinverse used at -0.005
```

```
## Warning in simpleLoess(y, x, w, span, degree = degree, parametric = parametric, : neighborhood radius 1.005
```

```
## Warning in simpleLoess(y, x, w, span, degree = degree, parametric = parametric, : reciprocal condition number 0
```

```
## Warning in simpleLoess(y, x, w, span, degree = degree, parametric = parametric, : There are other near singularities as well. 1.01
```

```
## Warning in predLoess(object$y, object$x, newx = if (is.null(newdata)) object$x else if (is.data.frame(newdata)) as.matrix(model.frame(delete.response(terms(object)), : pseudoinverse used at -0.005
```

```
## Warning in predLoess(object$y, object$x, newx = if (is.null(newdata)) object$x else if (is.data.frame(newdata)) as.matrix(model.frame(delete.response(terms(object)), : neighborhood radius 1.005
```

```
## Warning in predLoess(object$y, object$x, newx = if (is.null(newdata)) object$x else if (is.data.frame(newdata)) as.matrix(model.frame(delete.response(terms(object)), : reciprocal condition number 0
```

```
## Warning in predLoess(object$y, object$x, newx = if (is.null(newdata)) object$x else if (is.data.frame(newdata)) as.matrix(model.frame(delete.response(terms(object)), : There are other near
## singularities as well. 1.01
```

```
## Warning in simpleLoess(y, x, w, span, degree = degree, parametric = parametric, : pseudoinverse used at -0.005
```

```
## Warning in simpleLoess(y, x, w, span, degree = degree, parametric = parametric, : neighborhood radius 1.005
```

```
## Warning in simpleLoess(y, x, w, span, degree = degree, parametric = parametric, : reciprocal condition number 0
```

```
## Warning in simpleLoess(y, x, w, span, degree = degree, parametric = parametric, : There are other near singularities as well. 1.01
```

```
## Warning in predLoess(object$y, object$x, newx = if (is.null(newdata)) object$x else if (is.data.frame(newdata)) as.matrix(model.frame(delete.response(terms(object)), : pseudoinverse used at -0.005
```

```
## Warning in predLoess(object$y, object$x, newx = if (is.null(newdata)) object$x else if (is.data.frame(newdata)) as.matrix(model.frame(delete.response(terms(object)), : neighborhood radius 1.005
```

```
## Warning in predLoess(object$y, object$x, newx = if (is.null(newdata)) object$x else if (is.data.frame(newdata)) as.matrix(model.frame(delete.response(terms(object)), : reciprocal condition number 0
```

```
## Warning in predLoess(object$y, object$x, newx = if (is.null(newdata)) object$x else if (is.data.frame(newdata)) as.matrix(model.frame(delete.response(terms(object)), : There are other near
## singularities as well. 1.01
```

```
## Warning in simpleLoess(y, x, w, span, degree = degree, parametric = parametric, : at -0.005
```

```
## Warning in simpleLoess(y, x, w, span, degree = degree, parametric = parametric, : radius 2.5e-05
```

```
## Warning in simpleLoess(y, x, w, span, degree = degree, parametric = parametric, : all data on boundary of neighborhood. make span bigger
```

```
## Warning in simpleLoess(y, x, w, span, degree = degree, parametric = parametric, : pseudoinverse used at -0.005
```

```
## Warning in simpleLoess(y, x, w, span, degree = degree, parametric = parametric, : neighborhood radius 0.005
```

```
## Warning in simpleLoess(y, x, w, span, degree = degree, parametric = parametric, : reciprocal condition number 1
```

```
## Warning in simpleLoess(y, x, w, span, degree = degree, parametric = parametric, : There are other near singularities as well. 1.01
```

```
## Warning in simpleLoess(y, x, w, span, degree = degree, parametric = parametric, : zero-width neighborhood. make span bigger
```

```
## Warning: Computation failed in `stat_smooth()`:
## NA/NaN/Inf in foreign function call (arg 5)
```

```
## Warning in simpleLoess(y, x, w, span, degree = degree, parametric = parametric, : at -0.005
```

```
## Warning in simpleLoess(y, x, w, span, degree = degree, parametric = parametric, : radius 2.5e-05
```

```
## Warning in simpleLoess(y, x, w, span, degree = degree, parametric = parametric, : all data on boundary of neighborhood. make span bigger
```

```
## Warning in simpleLoess(y, x, w, span, degree = degree, parametric = parametric, : pseudoinverse used at -0.005
```

```
## Warning in simpleLoess(y, x, w, span, degree = degree, parametric = parametric, : neighborhood radius 0.005
```

```
## Warning in simpleLoess(y, x, w, span, degree = degree, parametric = parametric, : reciprocal condition number 1
```

```
## Warning in simpleLoess(y, x, w, span, degree = degree, parametric = parametric, : There are other near singularities as well. 1.01
```

```
## Warning in simpleLoess(y, x, w, span, degree = degree, parametric = parametric, : zero-width neighborhood. make span bigger
```

```
## Warning: Computation failed in `stat_smooth()`:
## NA/NaN/Inf in foreign function call (arg 5)
```

```
## Warning in simpleLoess(y, x, w, span, degree = degree, parametric = parametric, : at -0.005
```

```
## Warning in simpleLoess(y, x, w, span, degree = degree, parametric = parametric, : radius 2.5e-05
```

```
## Warning in simpleLoess(y, x, w, span, degree = degree, parametric = parametric, : all data on boundary of neighborhood. make span bigger
```

```
## Warning in simpleLoess(y, x, w, span, degree = degree, parametric = parametric, : pseudoinverse used at -0.005
```

```
## Warning in simpleLoess(y, x, w, span, degree = degree, parametric = parametric, : neighborhood radius 0.005
```

```
## Warning in simpleLoess(y, x, w, span, degree = degree, parametric = parametric, : reciprocal condition number 1
```

```
## Warning in simpleLoess(y, x, w, span, degree = degree, parametric = parametric, : There are other near singularities as well. 1.01
```

```
## Warning in simpleLoess(y, x, w, span, degree = degree, parametric = parametric, : zero-width neighborhood. make span bigger
```

```
## Warning: Computation failed in `stat_smooth()`:
## NA/NaN/Inf in foreign function call (arg 5)
```

```
## Warning in simpleLoess(y, x, w, span, degree = degree, parametric = parametric, : at -0.005
```

```
## Warning in simpleLoess(y, x, w, span, degree = degree, parametric = parametric, : radius 2.5e-05
```

```
## Warning in simpleLoess(y, x, w, span, degree = degree, parametric = parametric, : all data on boundary of neighborhood. make span bigger
```

```
## Warning in simpleLoess(y, x, w, span, degree = degree, parametric = parametric, : pseudoinverse used at -0.005
```

```
## Warning in simpleLoess(y, x, w, span, degree = degree, parametric = parametric, : neighborhood radius 0.005
```

```
## Warning in simpleLoess(y, x, w, span, degree = degree, parametric = parametric, : reciprocal condition number 1
```

```
## Warning in simpleLoess(y, x, w, span, degree = degree, parametric = parametric, : There are other near singularities as well. 1.01
```

```
## Warning in simpleLoess(y, x, w, span, degree = degree, parametric = parametric, : zero-width neighborhood. make span bigger
```

```
## Warning: Computation failed in `stat_smooth()`:
## NA/NaN/Inf in foreign function call (arg 5)
```

```
## Warning in simpleLoess(y, x, w, span, degree = degree, parametric = parametric, : at -0.005
```

```
## Warning in simpleLoess(y, x, w, span, degree = degree, parametric = parametric, : radius 2.5e-05
```

```
## Warning in simpleLoess(y, x, w, span, degree = degree, parametric = parametric, : all data on boundary of neighborhood. make span bigger
```

```
## Warning in simpleLoess(y, x, w, span, degree = degree, parametric = parametric, : pseudoinverse used at -0.005
```

```
## Warning in simpleLoess(y, x, w, span, degree = degree, parametric = parametric, : neighborhood radius 0.005
```

```
## Warning in simpleLoess(y, x, w, span, degree = degree, parametric = parametric, : reciprocal condition number 1
```

```
## Warning in simpleLoess(y, x, w, span, degree = degree, parametric = parametric, : There are other near singularities as well. 1.01
```

```
## Warning in simpleLoess(y, x, w, span, degree = degree, parametric = parametric, : zero-width neighborhood. make span bigger
```

```
## Warning: Computation failed in `stat_smooth()`:
## NA/NaN/Inf in foreign function call (arg 5)
```

```
## Warning in simpleLoess(y, x, w, span, degree = degree, parametric = parametric, : pseudoinverse used at -0.005
```

```
## Warning in simpleLoess(y, x, w, span, degree = degree, parametric = parametric, : neighborhood radius 1.005
```

```
## Warning in simpleLoess(y, x, w, span, degree = degree, parametric = parametric, : reciprocal condition number 0
```

```
## Warning in simpleLoess(y, x, w, span, degree = degree, parametric = parametric, : There are other near singularities as well. 1.01
```

```
## Warning in predLoess(object$y, object$x, newx = if (is.null(newdata)) object$x else if (is.data.frame(newdata)) as.matrix(model.frame(delete.response(terms(object)), : pseudoinverse used at -0.005
```

```
## Warning in predLoess(object$y, object$x, newx = if (is.null(newdata)) object$x else if (is.data.frame(newdata)) as.matrix(model.frame(delete.response(terms(object)), : neighborhood radius 1.005
```

```
## Warning in predLoess(object$y, object$x, newx = if (is.null(newdata)) object$x else if (is.data.frame(newdata)) as.matrix(model.frame(delete.response(terms(object)), : reciprocal condition number 0
```

```
## Warning in predLoess(object$y, object$x, newx = if (is.null(newdata)) object$x else if (is.data.frame(newdata)) as.matrix(model.frame(delete.response(terms(object)), : There are other near
## singularities as well. 1.01
```

```
## Warning in simpleLoess(y, x, w, span, degree = degree, parametric = parametric, : at -0.005
```

```
## Warning in simpleLoess(y, x, w, span, degree = degree, parametric = parametric, : radius 2.5e-05
```

```
## Warning in simpleLoess(y, x, w, span, degree = degree, parametric = parametric, : all data on boundary of neighborhood. make span bigger
```

```
## Warning in simpleLoess(y, x, w, span, degree = degree, parametric = parametric, : pseudoinverse used at -0.005
```

```
## Warning in simpleLoess(y, x, w, span, degree = degree, parametric = parametric, : neighborhood radius 0.005
```

```
## Warning in simpleLoess(y, x, w, span, degree = degree, parametric = parametric, : reciprocal condition number 1
```

```
## Warning in simpleLoess(y, x, w, span, degree = degree, parametric = parametric, : There are other near singularities as well. 1.01
```

```
## Warning in simpleLoess(y, x, w, span, degree = degree, parametric = parametric, : zero-width neighborhood. make span bigger
```

```
## Warning: Computation failed in `stat_smooth()`:
## NA/NaN/Inf in foreign function call (arg 5)
```

```
## Warning in simpleLoess(y, x, w, span, degree = degree, parametric = parametric, : at -0.005
```

```
## Warning in simpleLoess(y, x, w, span, degree = degree, parametric = parametric, : radius 2.5e-05
```

```
## Warning in simpleLoess(y, x, w, span, degree = degree, parametric = parametric, : all data on boundary of neighborhood. make span bigger
```

```
## Warning in simpleLoess(y, x, w, span, degree = degree, parametric = parametric, : pseudoinverse used at -0.005
```

```
## Warning in simpleLoess(y, x, w, span, degree = degree, parametric = parametric, : neighborhood radius 0.005
```

```
## Warning in simpleLoess(y, x, w, span, degree = degree, parametric = parametric, : reciprocal condition number 1
```

```
## Warning in simpleLoess(y, x, w, span, degree = degree, parametric = parametric, : There are other near singularities as well. 1.01
```

```
## Warning in simpleLoess(y, x, w, span, degree = degree, parametric = parametric, : zero-width neighborhood. make span bigger
```

```
## Warning: Computation failed in `stat_smooth()`:
## NA/NaN/Inf in foreign function call (arg 5)
```

```
## Warning in simpleLoess(y, x, w, span, degree = degree, parametric = parametric, : pseudoinverse used at -0.005
```

```
## Warning in simpleLoess(y, x, w, span, degree = degree, parametric = parametric, : neighborhood radius 1.005
```

```
## Warning in simpleLoess(y, x, w, span, degree = degree, parametric = parametric, : reciprocal condition number 0
```

```
## Warning in simpleLoess(y, x, w, span, degree = degree, parametric = parametric, : There are other near singularities as well. 1.01
```

```
## Warning in predLoess(object$y, object$x, newx = if (is.null(newdata)) object$x else if (is.data.frame(newdata)) as.matrix(model.frame(delete.response(terms(object)), : pseudoinverse used at -0.005
```

```
## Warning in predLoess(object$y, object$x, newx = if (is.null(newdata)) object$x else if (is.data.frame(newdata)) as.matrix(model.frame(delete.response(terms(object)), : neighborhood radius 1.005
```

```
## Warning in predLoess(object$y, object$x, newx = if (is.null(newdata)) object$x else if (is.data.frame(newdata)) as.matrix(model.frame(delete.response(terms(object)), : reciprocal condition number 0
```

```
## Warning in predLoess(object$y, object$x, newx = if (is.null(newdata)) object$x else if (is.data.frame(newdata)) as.matrix(model.frame(delete.response(terms(object)), : There are other near
## singularities as well. 1.01
```

```
## Warning in simpleLoess(y, x, w, span, degree = degree, parametric = parametric, : at -0.005
```

```
## Warning in simpleLoess(y, x, w, span, degree = degree, parametric = parametric, : radius 2.5e-05
```

```
## Warning in simpleLoess(y, x, w, span, degree = degree, parametric = parametric, : all data on boundary of neighborhood. make span bigger
```

```
## Warning in simpleLoess(y, x, w, span, degree = degree, parametric = parametric, : pseudoinverse used at -0.005
```

```
## Warning in simpleLoess(y, x, w, span, degree = degree, parametric = parametric, : neighborhood radius 0.005
```

```
## Warning in simpleLoess(y, x, w, span, degree = degree, parametric = parametric, : reciprocal condition number 1
```

```
## Warning in simpleLoess(y, x, w, span, degree = degree, parametric = parametric, : There are other near singularities as well. 1.01
```

```
## Warning in simpleLoess(y, x, w, span, degree = degree, parametric = parametric, : zero-width neighborhood. make span bigger
```

```
## Warning: Computation failed in `stat_smooth()`:
## NA/NaN/Inf in foreign function call (arg 5)
```

```
## Warning in simpleLoess(y, x, w, span, degree = degree, parametric = parametric, : at -0.005
```

```
## Warning in simpleLoess(y, x, w, span, degree = degree, parametric = parametric, : radius 2.5e-05
```

```
## Warning in simpleLoess(y, x, w, span, degree = degree, parametric = parametric, : all data on boundary of neighborhood. make span bigger
```

```
## Warning in simpleLoess(y, x, w, span, degree = degree, parametric = parametric, : pseudoinverse used at -0.005
```

```
## Warning in simpleLoess(y, x, w, span, degree = degree, parametric = parametric, : neighborhood radius 0.005
```

```
## Warning in simpleLoess(y, x, w, span, degree = degree, parametric = parametric, : reciprocal condition number 1
```

```
## Warning in simpleLoess(y, x, w, span, degree = degree, parametric = parametric, : There are other near singularities as well. 1.01
```

```
## Warning in simpleLoess(y, x, w, span, degree = degree, parametric = parametric, : zero-width neighborhood. make span bigger
```

```
## Warning: Computation failed in `stat_smooth()`:
## NA/NaN/Inf in foreign function call (arg 5)
```

```
## Warning in simpleLoess(y, x, w, span, degree = degree, parametric = parametric, : at -0.005
```

```
## Warning in simpleLoess(y, x, w, span, degree = degree, parametric = parametric, : radius 2.5e-05
```

```
## Warning in simpleLoess(y, x, w, span, degree = degree, parametric = parametric, : all data on boundary of neighborhood. make span bigger
```

```
## Warning in simpleLoess(y, x, w, span, degree = degree, parametric = parametric, : pseudoinverse used at -0.005
```

```
## Warning in simpleLoess(y, x, w, span, degree = degree, parametric = parametric, : neighborhood radius 0.005
```

```
## Warning in simpleLoess(y, x, w, span, degree = degree, parametric = parametric, : reciprocal condition number 1
```

```
## Warning in simpleLoess(y, x, w, span, degree = degree, parametric = parametric, : There are other near singularities as well. 1.01
```

```
## Warning in simpleLoess(y, x, w, span, degree = degree, parametric = parametric, : zero-width neighborhood. make span bigger
```

```
## Warning: Computation failed in `stat_smooth()`:
## NA/NaN/Inf in foreign function call (arg 5)
```

```
## Warning in simpleLoess(y, x, w, span, degree = degree, parametric = parametric, : at -0.005
```

```
## Warning in simpleLoess(y, x, w, span, degree = degree, parametric = parametric, : radius 2.5e-05
```

```
## Warning in simpleLoess(y, x, w, span, degree = degree, parametric = parametric, : all data on boundary of neighborhood. make span bigger
```

```
## Warning in simpleLoess(y, x, w, span, degree = degree, parametric = parametric, : pseudoinverse used at -0.005
```

```
## Warning in simpleLoess(y, x, w, span, degree = degree, parametric = parametric, : neighborhood radius 0.005
```

```
## Warning in simpleLoess(y, x, w, span, degree = degree, parametric = parametric, : reciprocal condition number 1
```

```
## Warning in simpleLoess(y, x, w, span, degree = degree, parametric = parametric, : There are other near singularities as well. 1.01
```

```
## Warning in simpleLoess(y, x, w, span, degree = degree, parametric = parametric, : zero-width neighborhood. make span bigger
```

```
## Warning: Computation failed in `stat_smooth()`:
## NA/NaN/Inf in foreign function call (arg 5)
```

```
## Warning in simpleLoess(y, x, w, span, degree = degree, parametric = parametric, : at -0.005
```

```
## Warning in simpleLoess(y, x, w, span, degree = degree, parametric = parametric, : radius 2.5e-05
```

```
## Warning in simpleLoess(y, x, w, span, degree = degree, parametric = parametric, : all data on boundary of neighborhood. make span bigger
```

```
## Warning in simpleLoess(y, x, w, span, degree = degree, parametric = parametric, : pseudoinverse used at -0.005
```

```
## Warning in simpleLoess(y, x, w, span, degree = degree, parametric = parametric, : neighborhood radius 0.005
```

```
## Warning in simpleLoess(y, x, w, span, degree = degree, parametric = parametric, : reciprocal condition number 1
```

```
## Warning in simpleLoess(y, x, w, span, degree = degree, parametric = parametric, : There are other near singularities as well. 1.01
```

```
## Warning in simpleLoess(y, x, w, span, degree = degree, parametric = parametric, : zero-width neighborhood. make span bigger
```

```
## Warning: Computation failed in `stat_smooth()`:
## NA/NaN/Inf in foreign function call (arg 5)
```

```
## Warning in simpleLoess(y, x, w, span, degree = degree, parametric = parametric, : at -0.005
```

```
## Warning in simpleLoess(y, x, w, span, degree = degree, parametric = parametric, : radius 2.5e-05
```

```
## Warning in simpleLoess(y, x, w, span, degree = degree, parametric = parametric, : all data on boundary of neighborhood. make span bigger
```

```
## Warning in simpleLoess(y, x, w, span, degree = degree, parametric = parametric, : pseudoinverse used at -0.005
```

```
## Warning in simpleLoess(y, x, w, span, degree = degree, parametric = parametric, : neighborhood radius 0.005
```

```
## Warning in simpleLoess(y, x, w, span, degree = degree, parametric = parametric, : reciprocal condition number 1
```

```
## Warning in simpleLoess(y, x, w, span, degree = degree, parametric = parametric, : There are other near singularities as well. 1.01
```

```
## Warning in simpleLoess(y, x, w, span, degree = degree, parametric = parametric, : zero-width neighborhood. make span bigger
```

```
## Warning: Computation failed in `stat_smooth()`:
## NA/NaN/Inf in foreign function call (arg 5)
```

```
## Warning in simpleLoess(y, x, w, span, degree = degree, parametric = parametric, : at -0.005
```

```
## Warning in simpleLoess(y, x, w, span, degree = degree, parametric = parametric, : radius 2.5e-05
```

```
## Warning in simpleLoess(y, x, w, span, degree = degree, parametric = parametric, : all data on boundary of neighborhood. make span bigger
```

```
## Warning in simpleLoess(y, x, w, span, degree = degree, parametric = parametric, : pseudoinverse used at -0.005
```

```
## Warning in simpleLoess(y, x, w, span, degree = degree, parametric = parametric, : neighborhood radius 0.005
```

```
## Warning in simpleLoess(y, x, w, span, degree = degree, parametric = parametric, : reciprocal condition number 1
```

```
## Warning in simpleLoess(y, x, w, span, degree = degree, parametric = parametric, : There are other near singularities as well. 1.01
```

```
## Warning in simpleLoess(y, x, w, span, degree = degree, parametric = parametric, : zero-width neighborhood. make span bigger
```

```
## Warning: Computation failed in `stat_smooth()`:
## NA/NaN/Inf in foreign function call (arg 5)
```

```
## Warning in simpleLoess(y, x, w, span, degree = degree, parametric = parametric, : at -0.005
```

```
## Warning in simpleLoess(y, x, w, span, degree = degree, parametric = parametric, : radius 2.5e-05
```

```
## Warning in simpleLoess(y, x, w, span, degree = degree, parametric = parametric, : all data on boundary of neighborhood. make span bigger
```

```
## Warning in simpleLoess(y, x, w, span, degree = degree, parametric = parametric, : pseudoinverse used at -0.005
```

```
## Warning in simpleLoess(y, x, w, span, degree = degree, parametric = parametric, : neighborhood radius 0.005
```

```
## Warning in simpleLoess(y, x, w, span, degree = degree, parametric = parametric, : reciprocal condition number 1
```

```
## Warning in simpleLoess(y, x, w, span, degree = degree, parametric = parametric, : There are other near singularities as well. 1.01
```

```
## Warning in simpleLoess(y, x, w, span, degree = degree, parametric = parametric, : zero-width neighborhood. make span bigger
```

```
## Warning: Computation failed in `stat_smooth()`:
## NA/NaN/Inf in foreign function call (arg 5)
```

```
## Warning in simpleLoess(y, x, w, span, degree = degree, parametric = parametric, : at -0.005
```

```
## Warning in simpleLoess(y, x, w, span, degree = degree, parametric = parametric, : radius 2.5e-05
```

```
## Warning in simpleLoess(y, x, w, span, degree = degree, parametric = parametric, : all data on boundary of neighborhood. make span bigger
```

```
## Warning in simpleLoess(y, x, w, span, degree = degree, parametric = parametric, : pseudoinverse used at -0.005
```

```
## Warning in simpleLoess(y, x, w, span, degree = degree, parametric = parametric, : neighborhood radius 0.005
```

```
## Warning in simpleLoess(y, x, w, span, degree = degree, parametric = parametric, : reciprocal condition number 1
```

```
## Warning in simpleLoess(y, x, w, span, degree = degree, parametric = parametric, : There are other near singularities as well. 1.01
```

```
## Warning in simpleLoess(y, x, w, span, degree = degree, parametric = parametric, : zero-width neighborhood. make span bigger
```

```
## Warning: Computation failed in `stat_smooth()`:
## NA/NaN/Inf in foreign function call (arg 5)
```

```
## Warning in simpleLoess(y, x, w, span, degree = degree, parametric = parametric, : at -0.005
```

```
## Warning in simpleLoess(y, x, w, span, degree = degree, parametric = parametric, : radius 2.5e-05
```

```
## Warning in simpleLoess(y, x, w, span, degree = degree, parametric = parametric, : all data on boundary of neighborhood. make span bigger
```

```
## Warning in simpleLoess(y, x, w, span, degree = degree, parametric = parametric, : pseudoinverse used at -0.005
```

```
## Warning in simpleLoess(y, x, w, span, degree = degree, parametric = parametric, : neighborhood radius 0.005
```

```
## Warning in simpleLoess(y, x, w, span, degree = degree, parametric = parametric, : reciprocal condition number 1
```

```
## Warning in simpleLoess(y, x, w, span, degree = degree, parametric = parametric, : There are other near singularities as well. 1.01
```

```
## Warning in simpleLoess(y, x, w, span, degree = degree, parametric = parametric, : zero-width neighborhood. make span bigger
```

```
## Warning: Computation failed in `stat_smooth()`:
## NA/NaN/Inf in foreign function call (arg 5)
```

```
## Warning in simpleLoess(y, x, w, span, degree = degree, parametric = parametric, : at -0.005
```

```
## Warning in simpleLoess(y, x, w, span, degree = degree, parametric = parametric, : radius 2.5e-05
```

```
## Warning in simpleLoess(y, x, w, span, degree = degree, parametric = parametric, : all data on boundary of neighborhood. make span bigger
```

```
## Warning in simpleLoess(y, x, w, span, degree = degree, parametric = parametric, : pseudoinverse used at -0.005
```

```
## Warning in simpleLoess(y, x, w, span, degree = degree, parametric = parametric, : neighborhood radius 0.005
```

```
## Warning in simpleLoess(y, x, w, span, degree = degree, parametric = parametric, : reciprocal condition number 1
```

```
## Warning in simpleLoess(y, x, w, span, degree = degree, parametric = parametric, : There are other near singularities as well. 1.01
```

```
## Warning in simpleLoess(y, x, w, span, degree = degree, parametric = parametric, : zero-width neighborhood. make span bigger
```

```
## Warning: Computation failed in `stat_smooth()`:
## NA/NaN/Inf in foreign function call (arg 5)
```

```
## Warning in simpleLoess(y, x, w, span, degree = degree, parametric = parametric, : at -0.005
```

```
## Warning in simpleLoess(y, x, w, span, degree = degree, parametric = parametric, : radius 2.5e-05
```

```
## Warning in simpleLoess(y, x, w, span, degree = degree, parametric = parametric, : all data on boundary of neighborhood. make span bigger
```

```
## Warning in simpleLoess(y, x, w, span, degree = degree, parametric = parametric, : pseudoinverse used at -0.005
```

```
## Warning in simpleLoess(y, x, w, span, degree = degree, parametric = parametric, : neighborhood radius 0.005
```

```
## Warning in simpleLoess(y, x, w, span, degree = degree, parametric = parametric, : reciprocal condition number 1
```

```
## Warning in simpleLoess(y, x, w, span, degree = degree, parametric = parametric, : There are other near singularities as well. 1.01
```

```
## Warning in simpleLoess(y, x, w, span, degree = degree, parametric = parametric, : zero-width neighborhood. make span bigger
```

```
## Warning: Computation failed in `stat_smooth()`:
## NA/NaN/Inf in foreign function call (arg 5)
```

```
## Warning in simpleLoess(y, x, w, span, degree = degree, parametric = parametric, : at -0.005
```

```
## Warning in simpleLoess(y, x, w, span, degree = degree, parametric = parametric, : radius 2.5e-05
```

```
## Warning in simpleLoess(y, x, w, span, degree = degree, parametric = parametric, : all data on boundary of neighborhood. make span bigger
```

```
## Warning in simpleLoess(y, x, w, span, degree = degree, parametric = parametric, : pseudoinverse used at -0.005
```

```
## Warning in simpleLoess(y, x, w, span, degree = degree, parametric = parametric, : neighborhood radius 0.005
```

```
## Warning in simpleLoess(y, x, w, span, degree = degree, parametric = parametric, : reciprocal condition number 1
```

```
## Warning in simpleLoess(y, x, w, span, degree = degree, parametric = parametric, : There are other near singularities as well. 1.01
```

```
## Warning in simpleLoess(y, x, w, span, degree = degree, parametric = parametric, : zero-width neighborhood. make span bigger
```

```
## Warning: Computation failed in `stat_smooth()`:
## NA/NaN/Inf in foreign function call (arg 5)
```

```
## Warning in simpleLoess(y, x, w, span, degree = degree, parametric = parametric, : at -0.005
```

```
## Warning in simpleLoess(y, x, w, span, degree = degree, parametric = parametric, : radius 2.5e-05
```

```
## Warning in simpleLoess(y, x, w, span, degree = degree, parametric = parametric, : all data on boundary of neighborhood. make span bigger
```

```
## Warning in simpleLoess(y, x, w, span, degree = degree, parametric = parametric, : pseudoinverse used at -0.005
```

```
## Warning in simpleLoess(y, x, w, span, degree = degree, parametric = parametric, : neighborhood radius 0.005
```

```
## Warning in simpleLoess(y, x, w, span, degree = degree, parametric = parametric, : reciprocal condition number 1
```

```
## Warning in simpleLoess(y, x, w, span, degree = degree, parametric = parametric, : There are other near singularities as well. 1.01
```

```
## Warning in simpleLoess(y, x, w, span, degree = degree, parametric = parametric, : zero-width neighborhood. make span bigger
```

```
## Warning: Computation failed in `stat_smooth()`:
## NA/NaN/Inf in foreign function call (arg 5)
```

```
## Warning in simpleLoess(y, x, w, span, degree = degree, parametric = parametric, : at -0.005
```

```
## Warning in simpleLoess(y, x, w, span, degree = degree, parametric = parametric, : radius 2.5e-05
```

```
## Warning in simpleLoess(y, x, w, span, degree = degree, parametric = parametric, : all data on boundary of neighborhood. make span bigger
```

```
## Warning in simpleLoess(y, x, w, span, degree = degree, parametric = parametric, : pseudoinverse used at -0.005
```

```
## Warning in simpleLoess(y, x, w, span, degree = degree, parametric = parametric, : neighborhood radius 0.005
```

```
## Warning in simpleLoess(y, x, w, span, degree = degree, parametric = parametric, : reciprocal condition number 1
```

```
## Warning in simpleLoess(y, x, w, span, degree = degree, parametric = parametric, : There are other near singularities as well. 1.01
```

```
## Warning in simpleLoess(y, x, w, span, degree = degree, parametric = parametric, : zero-width neighborhood. make span bigger
```

```
## Warning: Computation failed in `stat_smooth()`:
## NA/NaN/Inf in foreign function call (arg 5)
```

```
## Warning in simpleLoess(y, x, w, span, degree = degree, parametric = parametric, : at -0.005
```

```
## Warning in simpleLoess(y, x, w, span, degree = degree, parametric = parametric, : radius 2.5e-05
```

```
## Warning in simpleLoess(y, x, w, span, degree = degree, parametric = parametric, : all data on boundary of neighborhood. make span bigger
```

```
## Warning in simpleLoess(y, x, w, span, degree = degree, parametric = parametric, : pseudoinverse used at -0.005
```

```
## Warning in simpleLoess(y, x, w, span, degree = degree, parametric = parametric, : neighborhood radius 0.005
```

```
## Warning in simpleLoess(y, x, w, span, degree = degree, parametric = parametric, : reciprocal condition number 1
```

```
## Warning in simpleLoess(y, x, w, span, degree = degree, parametric = parametric, : There are other near singularities as well. 1.01
```

```
## Warning in simpleLoess(y, x, w, span, degree = degree, parametric = parametric, : zero-width neighborhood. make span bigger
```

```
## Warning: Computation failed in `stat_smooth()`:
## NA/NaN/Inf in foreign function call (arg 5)
```

```
## Warning in simpleLoess(y, x, w, span, degree = degree, parametric = parametric, : at -0.005
```

```
## Warning in simpleLoess(y, x, w, span, degree = degree, parametric = parametric, : radius 2.5e-05
```

```
## Warning in simpleLoess(y, x, w, span, degree = degree, parametric = parametric, : all data on boundary of neighborhood. make span bigger
```

```
## Warning in simpleLoess(y, x, w, span, degree = degree, parametric = parametric, : pseudoinverse used at -0.005
```

```
## Warning in simpleLoess(y, x, w, span, degree = degree, parametric = parametric, : neighborhood radius 0.005
```

```
## Warning in simpleLoess(y, x, w, span, degree = degree, parametric = parametric, : reciprocal condition number 1
```

```
## Warning in simpleLoess(y, x, w, span, degree = degree, parametric = parametric, : There are other near singularities as well. 1.01
```

```
## Warning in simpleLoess(y, x, w, span, degree = degree, parametric = parametric, : zero-width neighborhood. make span bigger
```

```
## Warning: Computation failed in `stat_smooth()`:
## NA/NaN/Inf in foreign function call (arg 5)
```

```
## Warning in simpleLoess(y, x, w, span, degree = degree, parametric = parametric, : at -0.005
```

```
## Warning in simpleLoess(y, x, w, span, degree = degree, parametric = parametric, : radius 2.5e-05
```

```
## Warning in simpleLoess(y, x, w, span, degree = degree, parametric = parametric, : all data on boundary of neighborhood. make span bigger
```

```
## Warning in simpleLoess(y, x, w, span, degree = degree, parametric = parametric, : pseudoinverse used at -0.005
```

```
## Warning in simpleLoess(y, x, w, span, degree = degree, parametric = parametric, : neighborhood radius 0.005
```

```
## Warning in simpleLoess(y, x, w, span, degree = degree, parametric = parametric, : reciprocal condition number 1
```

```
## Warning in simpleLoess(y, x, w, span, degree = degree, parametric = parametric, : There are other near singularities as well. 1.01
```

```
## Warning in simpleLoess(y, x, w, span, degree = degree, parametric = parametric, : zero-width neighborhood. make span bigger
```

```
## Warning: Computation failed in `stat_smooth()`:
## NA/NaN/Inf in foreign function call (arg 5)
```

```
## Warning in simpleLoess(y, x, w, span, degree = degree, parametric = parametric, : at -0.005
```

```
## Warning in simpleLoess(y, x, w, span, degree = degree, parametric = parametric, : radius 2.5e-05
```

```
## Warning in simpleLoess(y, x, w, span, degree = degree, parametric = parametric, : all data on boundary of neighborhood. make span bigger
```

```
## Warning in simpleLoess(y, x, w, span, degree = degree, parametric = parametric, : pseudoinverse used at -0.005
```

```
## Warning in simpleLoess(y, x, w, span, degree = degree, parametric = parametric, : neighborhood radius 0.005
```

```
## Warning in simpleLoess(y, x, w, span, degree = degree, parametric = parametric, : reciprocal condition number 1
```

```
## Warning in simpleLoess(y, x, w, span, degree = degree, parametric = parametric, : There are other near singularities as well. 1.01
```

```
## Warning in simpleLoess(y, x, w, span, degree = degree, parametric = parametric, : zero-width neighborhood. make span bigger
```

```
## Warning: Computation failed in `stat_smooth()`:
## NA/NaN/Inf in foreign function call (arg 5)
```

```
## Warning in simpleLoess(y, x, w, span, degree = degree, parametric = parametric, : pseudoinverse used at -0.005
```

```
## Warning in simpleLoess(y, x, w, span, degree = degree, parametric = parametric, : neighborhood radius 1.005
```

```
## Warning in simpleLoess(y, x, w, span, degree = degree, parametric = parametric, : reciprocal condition number 0
```

```
## Warning in simpleLoess(y, x, w, span, degree = degree, parametric = parametric, : There are other near singularities as well. 1.01
```

```
## Warning in predLoess(object$y, object$x, newx = if (is.null(newdata)) object$x else if (is.data.frame(newdata)) as.matrix(model.frame(delete.response(terms(object)), : pseudoinverse used at -0.005
```

```
## Warning in predLoess(object$y, object$x, newx = if (is.null(newdata)) object$x else if (is.data.frame(newdata)) as.matrix(model.frame(delete.response(terms(object)), : neighborhood radius 1.005
```

```
## Warning in predLoess(object$y, object$x, newx = if (is.null(newdata)) object$x else if (is.data.frame(newdata)) as.matrix(model.frame(delete.response(terms(object)), : reciprocal condition number 0
```

```
## Warning in predLoess(object$y, object$x, newx = if (is.null(newdata)) object$x else if (is.data.frame(newdata)) as.matrix(model.frame(delete.response(terms(object)), : There are other near
## singularities as well. 1.01
```

```
## Warning in simpleLoess(y, x, w, span, degree = degree, parametric = parametric, : at -0.005
```

```
## Warning in simpleLoess(y, x, w, span, degree = degree, parametric = parametric, : radius 2.5e-05
```

```
## Warning in simpleLoess(y, x, w, span, degree = degree, parametric = parametric, : all data on boundary of neighborhood. make span bigger
```

```
## Warning in simpleLoess(y, x, w, span, degree = degree, parametric = parametric, : pseudoinverse used at -0.005
```

```
## Warning in simpleLoess(y, x, w, span, degree = degree, parametric = parametric, : neighborhood radius 0.005
```

```
## Warning in simpleLoess(y, x, w, span, degree = degree, parametric = parametric, : reciprocal condition number 1
```

```
## Warning in simpleLoess(y, x, w, span, degree = degree, parametric = parametric, : There are other near singularities as well. 1.01
```

```
## Warning in simpleLoess(y, x, w, span, degree = degree, parametric = parametric, : zero-width neighborhood. make span bigger
```

```
## Warning: Computation failed in `stat_smooth()`:
## NA/NaN/Inf in foreign function call (arg 5)
```

```
## Warning in simpleLoess(y, x, w, span, degree = degree, parametric = parametric, : at -0.005
```

```
## Warning in simpleLoess(y, x, w, span, degree = degree, parametric = parametric, : radius 2.5e-05
```

```
## Warning in simpleLoess(y, x, w, span, degree = degree, parametric = parametric, : all data on boundary of neighborhood. make span bigger
```

```
## Warning in simpleLoess(y, x, w, span, degree = degree, parametric = parametric, : pseudoinverse used at -0.005
```

```
## Warning in simpleLoess(y, x, w, span, degree = degree, parametric = parametric, : neighborhood radius 0.005
```

```
## Warning in simpleLoess(y, x, w, span, degree = degree, parametric = parametric, : reciprocal condition number 1
```

```
## Warning in simpleLoess(y, x, w, span, degree = degree, parametric = parametric, : There are other near singularities as well. 1.01
```

```
## Warning in simpleLoess(y, x, w, span, degree = degree, parametric = parametric, : zero-width neighborhood. make span bigger
```

```
## Warning: Computation failed in `stat_smooth()`:
## NA/NaN/Inf in foreign function call (arg 5)
```

```
## Warning in simpleLoess(y, x, w, span, degree = degree, parametric = parametric, : at -0.005
```

```
## Warning in simpleLoess(y, x, w, span, degree = degree, parametric = parametric, : radius 2.5e-05
```

```
## Warning in simpleLoess(y, x, w, span, degree = degree, parametric = parametric, : all data on boundary of neighborhood. make span bigger
```

```
## Warning in simpleLoess(y, x, w, span, degree = degree, parametric = parametric, : pseudoinverse used at -0.005
```

```
## Warning in simpleLoess(y, x, w, span, degree = degree, parametric = parametric, : neighborhood radius 0.005
```

```
## Warning in simpleLoess(y, x, w, span, degree = degree, parametric = parametric, : reciprocal condition number 1
```

```
## Warning in simpleLoess(y, x, w, span, degree = degree, parametric = parametric, : There are other near singularities as well. 1.01
```

```
## Warning in simpleLoess(y, x, w, span, degree = degree, parametric = parametric, : zero-width neighborhood. make span bigger
```

```
## Warning: Computation failed in `stat_smooth()`:
## NA/NaN/Inf in foreign function call (arg 5)
```

```
## Warning in simpleLoess(y, x, w, span, degree = degree, parametric = parametric, : at -0.005
```

```
## Warning in simpleLoess(y, x, w, span, degree = degree, parametric = parametric, : radius 2.5e-05
```

```
## Warning in simpleLoess(y, x, w, span, degree = degree, parametric = parametric, : all data on boundary of neighborhood. make span bigger
```

```
## Warning in simpleLoess(y, x, w, span, degree = degree, parametric = parametric, : pseudoinverse used at -0.005
```

```
## Warning in simpleLoess(y, x, w, span, degree = degree, parametric = parametric, : neighborhood radius 0.005
```

```
## Warning in simpleLoess(y, x, w, span, degree = degree, parametric = parametric, : reciprocal condition number 1
```

```
## Warning in simpleLoess(y, x, w, span, degree = degree, parametric = parametric, : There are other near singularities as well. 1.01
```

```
## Warning in simpleLoess(y, x, w, span, degree = degree, parametric = parametric, : zero-width neighborhood. make span bigger
```

```
## Warning: Computation failed in `stat_smooth()`:
## NA/NaN/Inf in foreign function call (arg 5)
```

```
## Warning in simpleLoess(y, x, w, span, degree = degree, parametric = parametric, : at -0.005
```

```
## Warning in simpleLoess(y, x, w, span, degree = degree, parametric = parametric, : radius 2.5e-05
```

```
## Warning in simpleLoess(y, x, w, span, degree = degree, parametric = parametric, : all data on boundary of neighborhood. make span bigger
```

```
## Warning in simpleLoess(y, x, w, span, degree = degree, parametric = parametric, : pseudoinverse used at -0.005
```

```
## Warning in simpleLoess(y, x, w, span, degree = degree, parametric = parametric, : neighborhood radius 0.005
```

```
## Warning in simpleLoess(y, x, w, span, degree = degree, parametric = parametric, : reciprocal condition number 1
```

```
## Warning in simpleLoess(y, x, w, span, degree = degree, parametric = parametric, : There are other near singularities as well. 1.01
```

```
## Warning in simpleLoess(y, x, w, span, degree = degree, parametric = parametric, : zero-width neighborhood. make span bigger
```

```
## Warning: Computation failed in `stat_smooth()`:
## NA/NaN/Inf in foreign function call (arg 5)
```

```
## Warning in simpleLoess(y, x, w, span, degree = degree, parametric = parametric, : at -0.005
```

```
## Warning in simpleLoess(y, x, w, span, degree = degree, parametric = parametric, : radius 2.5e-05
```

```
## Warning in simpleLoess(y, x, w, span, degree = degree, parametric = parametric, : all data on boundary of neighborhood. make span bigger
```

```
## Warning in simpleLoess(y, x, w, span, degree = degree, parametric = parametric, : pseudoinverse used at -0.005
```

```
## Warning in simpleLoess(y, x, w, span, degree = degree, parametric = parametric, : neighborhood radius 0.005
```

```
## Warning in simpleLoess(y, x, w, span, degree = degree, parametric = parametric, : reciprocal condition number 1
```

```
## Warning in simpleLoess(y, x, w, span, degree = degree, parametric = parametric, : There are other near singularities as well. 1.01
```

```
## Warning in simpleLoess(y, x, w, span, degree = degree, parametric = parametric, : zero-width neighborhood. make span bigger
```

```
## Warning: Computation failed in `stat_smooth()`:
## NA/NaN/Inf in foreign function call (arg 5)
```

```
## Warning in simpleLoess(y, x, w, span, degree = degree, parametric = parametric, : pseudoinverse used at -0.005
```

```
## Warning in simpleLoess(y, x, w, span, degree = degree, parametric = parametric, : neighborhood radius 1.005
```

```
## Warning in simpleLoess(y, x, w, span, degree = degree, parametric = parametric, : reciprocal condition number 0
```

```
## Warning in simpleLoess(y, x, w, span, degree = degree, parametric = parametric, : There are other near singularities as well. 1.01
```

```
## Warning in predLoess(object$y, object$x, newx = if (is.null(newdata)) object$x else if (is.data.frame(newdata)) as.matrix(model.frame(delete.response(terms(object)), : pseudoinverse used at -0.005
```

```
## Warning in predLoess(object$y, object$x, newx = if (is.null(newdata)) object$x else if (is.data.frame(newdata)) as.matrix(model.frame(delete.response(terms(object)), : neighborhood radius 1.005
```

```
## Warning in predLoess(object$y, object$x, newx = if (is.null(newdata)) object$x else if (is.data.frame(newdata)) as.matrix(model.frame(delete.response(terms(object)), : reciprocal condition number 0
```

```
## Warning in predLoess(object$y, object$x, newx = if (is.null(newdata)) object$x else if (is.data.frame(newdata)) as.matrix(model.frame(delete.response(terms(object)), : There are other near
## singularities as well. 1.01
```

```
## Warning in simpleLoess(y, x, w, span, degree = degree, parametric = parametric, : at -0.005
```

```
## Warning in simpleLoess(y, x, w, span, degree = degree, parametric = parametric, : radius 2.5e-05
```

```
## Warning in simpleLoess(y, x, w, span, degree = degree, parametric = parametric, : all data on boundary of neighborhood. make span bigger
```

```
## Warning in simpleLoess(y, x, w, span, degree = degree, parametric = parametric, : pseudoinverse used at -0.005
```

```
## Warning in simpleLoess(y, x, w, span, degree = degree, parametric = parametric, : neighborhood radius 0.005
```

```
## Warning in simpleLoess(y, x, w, span, degree = degree, parametric = parametric, : reciprocal condition number 1
```

```
## Warning in simpleLoess(y, x, w, span, degree = degree, parametric = parametric, : There are other near singularities as well. 1.01
```

```
## Warning in simpleLoess(y, x, w, span, degree = degree, parametric = parametric, : zero-width neighborhood. make span bigger
```

```
## Warning: Computation failed in `stat_smooth()`:
## NA/NaN/Inf in foreign function call (arg 5)
```

```
## Warning in simpleLoess(y, x, w, span, degree = degree, parametric = parametric, : at -0.005
```

```
## Warning in simpleLoess(y, x, w, span, degree = degree, parametric = parametric, : radius 2.5e-05
```

```
## Warning in simpleLoess(y, x, w, span, degree = degree, parametric = parametric, : all data on boundary of neighborhood. make span bigger
```

```
## Warning in simpleLoess(y, x, w, span, degree = degree, parametric = parametric, : pseudoinverse used at -0.005
```

```
## Warning in simpleLoess(y, x, w, span, degree = degree, parametric = parametric, : neighborhood radius 0.005
```

```
## Warning in simpleLoess(y, x, w, span, degree = degree, parametric = parametric, : reciprocal condition number 1
```

```
## Warning in simpleLoess(y, x, w, span, degree = degree, parametric = parametric, : There are other near singularities as well. 1.01
```

```
## Warning in simpleLoess(y, x, w, span, degree = degree, parametric = parametric, : zero-width neighborhood. make span bigger
```

```
## Warning: Computation failed in `stat_smooth()`:
## NA/NaN/Inf in foreign function call (arg 5)
```

```
## Warning in simpleLoess(y, x, w, span, degree = degree, parametric = parametric, : at -0.005
```

```
## Warning in simpleLoess(y, x, w, span, degree = degree, parametric = parametric, : radius 2.5e-05
```

```
## Warning in simpleLoess(y, x, w, span, degree = degree, parametric = parametric, : all data on boundary of neighborhood. make span bigger
```

```
## Warning in simpleLoess(y, x, w, span, degree = degree, parametric = parametric, : pseudoinverse used at -0.005
```

```
## Warning in simpleLoess(y, x, w, span, degree = degree, parametric = parametric, : neighborhood radius 0.005
```

```
## Warning in simpleLoess(y, x, w, span, degree = degree, parametric = parametric, : reciprocal condition number 1
```

```
## Warning in simpleLoess(y, x, w, span, degree = degree, parametric = parametric, : There are other near singularities as well. 1.01
```

```
## Warning in simpleLoess(y, x, w, span, degree = degree, parametric = parametric, : zero-width neighborhood. make span bigger
```

```
## Warning: Computation failed in `stat_smooth()`:
## NA/NaN/Inf in foreign function call (arg 5)
```

```
## Warning in simpleLoess(y, x, w, span, degree = degree, parametric = parametric, : at -0.005
```

```
## Warning in simpleLoess(y, x, w, span, degree = degree, parametric = parametric, : radius 2.5e-05
```

```
## Warning in simpleLoess(y, x, w, span, degree = degree, parametric = parametric, : all data on boundary of neighborhood. make span bigger
```

```
## Warning in simpleLoess(y, x, w, span, degree = degree, parametric = parametric, : pseudoinverse used at -0.005
```

```
## Warning in simpleLoess(y, x, w, span, degree = degree, parametric = parametric, : neighborhood radius 0.005
```

```
## Warning in simpleLoess(y, x, w, span, degree = degree, parametric = parametric, : reciprocal condition number 1
```

```
## Warning in simpleLoess(y, x, w, span, degree = degree, parametric = parametric, : There are other near singularities as well. 1.01
```

```
## Warning in simpleLoess(y, x, w, span, degree = degree, parametric = parametric, : zero-width neighborhood. make span bigger
```

```
## Warning: Computation failed in `stat_smooth()`:
## NA/NaN/Inf in foreign function call (arg 5)
```

```
## Warning in simpleLoess(y, x, w, span, degree = degree, parametric = parametric, : pseudoinverse used at -0.005
```

```
## Warning in simpleLoess(y, x, w, span, degree = degree, parametric = parametric, : neighborhood radius 1.005
```

```
## Warning in simpleLoess(y, x, w, span, degree = degree, parametric = parametric, : reciprocal condition number 0
```

```
## Warning in simpleLoess(y, x, w, span, degree = degree, parametric = parametric, : There are other near singularities as well. 1.01
```

```
## Warning in predLoess(object$y, object$x, newx = if (is.null(newdata)) object$x else if (is.data.frame(newdata)) as.matrix(model.frame(delete.response(terms(object)), : pseudoinverse used at -0.005
```

```
## Warning in predLoess(object$y, object$x, newx = if (is.null(newdata)) object$x else if (is.data.frame(newdata)) as.matrix(model.frame(delete.response(terms(object)), : neighborhood radius 1.005
```

```
## Warning in predLoess(object$y, object$x, newx = if (is.null(newdata)) object$x else if (is.data.frame(newdata)) as.matrix(model.frame(delete.response(terms(object)), : reciprocal condition number 0
```

```
## Warning in predLoess(object$y, object$x, newx = if (is.null(newdata)) object$x else if (is.data.frame(newdata)) as.matrix(model.frame(delete.response(terms(object)), : There are other near
## singularities as well. 1.01
```

```
## Warning in simpleLoess(y, x, w, span, degree = degree, parametric = parametric, : at -0.005
```

```
## Warning in simpleLoess(y, x, w, span, degree = degree, parametric = parametric, : radius 2.5e-05
```

```
## Warning in simpleLoess(y, x, w, span, degree = degree, parametric = parametric, : all data on boundary of neighborhood. make span bigger
```

```
## Warning in simpleLoess(y, x, w, span, degree = degree, parametric = parametric, : pseudoinverse used at -0.005
```

```
## Warning in simpleLoess(y, x, w, span, degree = degree, parametric = parametric, : neighborhood radius 0.005
```

```
## Warning in simpleLoess(y, x, w, span, degree = degree, parametric = parametric, : reciprocal condition number 1
```

```
## Warning in simpleLoess(y, x, w, span, degree = degree, parametric = parametric, : There are other near singularities as well. 1.01
```

```
## Warning in simpleLoess(y, x, w, span, degree = degree, parametric = parametric, : zero-width neighborhood. make span bigger
```

```
## Warning: Computation failed in `stat_smooth()`:
## NA/NaN/Inf in foreign function call (arg 5)
```

```
## Warning in simpleLoess(y, x, w, span, degree = degree, parametric = parametric, : at -0.005
```

```
## Warning in simpleLoess(y, x, w, span, degree = degree, parametric = parametric, : radius 2.5e-05
```

```
## Warning in simpleLoess(y, x, w, span, degree = degree, parametric = parametric, : all data on boundary of neighborhood. make span bigger
```

```
## Warning in simpleLoess(y, x, w, span, degree = degree, parametric = parametric, : pseudoinverse used at -0.005
```

```
## Warning in simpleLoess(y, x, w, span, degree = degree, parametric = parametric, : neighborhood radius 0.005
```

```
## Warning in simpleLoess(y, x, w, span, degree = degree, parametric = parametric, : reciprocal condition number 1
```

```
## Warning in simpleLoess(y, x, w, span, degree = degree, parametric = parametric, : There are other near singularities as well. 1.01
```

```
## Warning in simpleLoess(y, x, w, span, degree = degree, parametric = parametric, : zero-width neighborhood. make span bigger
```

```
## Warning: Computation failed in `stat_smooth()`:
## NA/NaN/Inf in foreign function call (arg 5)
```

```
## Warning in simpleLoess(y, x, w, span, degree = degree, parametric = parametric, : pseudoinverse used at -0.005
```

```
## Warning in simpleLoess(y, x, w, span, degree = degree, parametric = parametric, : neighborhood radius 1.005
```

```
## Warning in simpleLoess(y, x, w, span, degree = degree, parametric = parametric, : reciprocal condition number 0
```

```
## Warning in simpleLoess(y, x, w, span, degree = degree, parametric = parametric, : There are other near singularities as well. 1.01
```

```
## Warning in predLoess(object$y, object$x, newx = if (is.null(newdata)) object$x else if (is.data.frame(newdata)) as.matrix(model.frame(delete.response(terms(object)), : pseudoinverse used at -0.005
```

```
## Warning in predLoess(object$y, object$x, newx = if (is.null(newdata)) object$x else if (is.data.frame(newdata)) as.matrix(model.frame(delete.response(terms(object)), : neighborhood radius 1.005
```

```
## Warning in predLoess(object$y, object$x, newx = if (is.null(newdata)) object$x else if (is.data.frame(newdata)) as.matrix(model.frame(delete.response(terms(object)), : reciprocal condition number 0
```

```
## Warning in predLoess(object$y, object$x, newx = if (is.null(newdata)) object$x else if (is.data.frame(newdata)) as.matrix(model.frame(delete.response(terms(object)), : There are other near
## singularities as well. 1.01
```

```
## Warning in simpleLoess(y, x, w, span, degree = degree, parametric = parametric, : pseudoinverse used at -0.005
```

```
## Warning in simpleLoess(y, x, w, span, degree = degree, parametric = parametric, : neighborhood radius 1.005
```

```
## Warning in simpleLoess(y, x, w, span, degree = degree, parametric = parametric, : reciprocal condition number 0
```

```
## Warning in simpleLoess(y, x, w, span, degree = degree, parametric = parametric, : There are other near singularities as well. 1.01
```

```
## Warning in predLoess(object$y, object$x, newx = if (is.null(newdata)) object$x else if (is.data.frame(newdata)) as.matrix(model.frame(delete.response(terms(object)), : pseudoinverse used at -0.005
```

```
## Warning in predLoess(object$y, object$x, newx = if (is.null(newdata)) object$x else if (is.data.frame(newdata)) as.matrix(model.frame(delete.response(terms(object)), : neighborhood radius 1.005
```

```
## Warning in predLoess(object$y, object$x, newx = if (is.null(newdata)) object$x else if (is.data.frame(newdata)) as.matrix(model.frame(delete.response(terms(object)), : reciprocal condition number 0
```

```
## Warning in predLoess(object$y, object$x, newx = if (is.null(newdata)) object$x else if (is.data.frame(newdata)) as.matrix(model.frame(delete.response(terms(object)), : There are other near
## singularities as well. 1.01
```

```
## Warning in simpleLoess(y, x, w, span, degree = degree, parametric = parametric, : at -0.005
```

```
## Warning in simpleLoess(y, x, w, span, degree = degree, parametric = parametric, : radius 2.5e-05
```

```
## Warning in simpleLoess(y, x, w, span, degree = degree, parametric = parametric, : all data on boundary of neighborhood. make span bigger
```

```
## Warning in simpleLoess(y, x, w, span, degree = degree, parametric = parametric, : pseudoinverse used at -0.005
```

```
## Warning in simpleLoess(y, x, w, span, degree = degree, parametric = parametric, : neighborhood radius 0.005
```

```
## Warning in simpleLoess(y, x, w, span, degree = degree, parametric = parametric, : reciprocal condition number 1
```

```
## Warning in simpleLoess(y, x, w, span, degree = degree, parametric = parametric, : There are other near singularities as well. 1.01
```

```
## Warning in simpleLoess(y, x, w, span, degree = degree, parametric = parametric, : zero-width neighborhood. make span bigger
```

```
## Warning: Computation failed in `stat_smooth()`:
## NA/NaN/Inf in foreign function call (arg 5)
```

```
## Warning in simpleLoess(y, x, w, span, degree = degree, parametric = parametric, : at -0.005
```

```
## Warning in simpleLoess(y, x, w, span, degree = degree, parametric = parametric, : radius 2.5e-05
```

```
## Warning in simpleLoess(y, x, w, span, degree = degree, parametric = parametric, : all data on boundary of neighborhood. make span bigger
```

```
## Warning in simpleLoess(y, x, w, span, degree = degree, parametric = parametric, : pseudoinverse used at -0.005
```

```
## Warning in simpleLoess(y, x, w, span, degree = degree, parametric = parametric, : neighborhood radius 0.005
```

```
## Warning in simpleLoess(y, x, w, span, degree = degree, parametric = parametric, : reciprocal condition number 1
```

```
## Warning in simpleLoess(y, x, w, span, degree = degree, parametric = parametric, : There are other near singularities as well. 1.01
```

```
## Warning in simpleLoess(y, x, w, span, degree = degree, parametric = parametric, : zero-width neighborhood. make span bigger
```

```
## Warning: Computation failed in `stat_smooth()`:
## NA/NaN/Inf in foreign function call (arg 5)
```

```
## Warning in simpleLoess(y, x, w, span, degree = degree, parametric = parametric, : at -0.005
```

```
## Warning in simpleLoess(y, x, w, span, degree = degree, parametric = parametric, : radius 2.5e-05
```

```
## Warning in simpleLoess(y, x, w, span, degree = degree, parametric = parametric, : all data on boundary of neighborhood. make span bigger
```

```
## Warning in simpleLoess(y, x, w, span, degree = degree, parametric = parametric, : pseudoinverse used at -0.005
```

```
## Warning in simpleLoess(y, x, w, span, degree = degree, parametric = parametric, : neighborhood radius 0.005
```

```
## Warning in simpleLoess(y, x, w, span, degree = degree, parametric = parametric, : reciprocal condition number 1
```

```
## Warning in simpleLoess(y, x, w, span, degree = degree, parametric = parametric, : There are other near singularities as well. 1.01
```

```
## Warning in simpleLoess(y, x, w, span, degree = degree, parametric = parametric, : zero-width neighborhood. make span bigger
```

```
## Warning: Computation failed in `stat_smooth()`:
## NA/NaN/Inf in foreign function call (arg 5)
```

```
## Warning in simpleLoess(y, x, w, span, degree = degree, parametric = parametric, : at -0.005
```

```
## Warning in simpleLoess(y, x, w, span, degree = degree, parametric = parametric, : radius 2.5e-05
```

```
## Warning in simpleLoess(y, x, w, span, degree = degree, parametric = parametric, : all data on boundary of neighborhood. make span bigger
```

```
## Warning in simpleLoess(y, x, w, span, degree = degree, parametric = parametric, : pseudoinverse used at -0.005
```

```
## Warning in simpleLoess(y, x, w, span, degree = degree, parametric = parametric, : neighborhood radius 0.005
```

```
## Warning in simpleLoess(y, x, w, span, degree = degree, parametric = parametric, : reciprocal condition number 1
```

```
## Warning in simpleLoess(y, x, w, span, degree = degree, parametric = parametric, : There are other near singularities as well. 1.01
```

```
## Warning in simpleLoess(y, x, w, span, degree = degree, parametric = parametric, : zero-width neighborhood. make span bigger
```

```
## Warning: Computation failed in `stat_smooth()`:
## NA/NaN/Inf in foreign function call (arg 5)
```

```
## Warning in simpleLoess(y, x, w, span, degree = degree, parametric = parametric, : at -0.005
```

```
## Warning in simpleLoess(y, x, w, span, degree = degree, parametric = parametric, : radius 2.5e-05
```

```
## Warning in simpleLoess(y, x, w, span, degree = degree, parametric = parametric, : all data on boundary of neighborhood. make span bigger
```

```
## Warning in simpleLoess(y, x, w, span, degree = degree, parametric = parametric, : pseudoinverse used at -0.005
```

```
## Warning in simpleLoess(y, x, w, span, degree = degree, parametric = parametric, : neighborhood radius 0.005
```

```
## Warning in simpleLoess(y, x, w, span, degree = degree, parametric = parametric, : reciprocal condition number 1
```

```
## Warning in simpleLoess(y, x, w, span, degree = degree, parametric = parametric, : There are other near singularities as well. 1.01
```

```
## Warning in simpleLoess(y, x, w, span, degree = degree, parametric = parametric, : zero-width neighborhood. make span bigger
```

```
## Warning: Computation failed in `stat_smooth()`:
## NA/NaN/Inf in foreign function call (arg 5)
```

```
## Warning in simpleLoess(y, x, w, span, degree = degree, parametric = parametric, : at -0.005
```

```
## Warning in simpleLoess(y, x, w, span, degree = degree, parametric = parametric, : radius 2.5e-05
```

```
## Warning in simpleLoess(y, x, w, span, degree = degree, parametric = parametric, : all data on boundary of neighborhood. make span bigger
```

```
## Warning in simpleLoess(y, x, w, span, degree = degree, parametric = parametric, : pseudoinverse used at -0.005
```

```
## Warning in simpleLoess(y, x, w, span, degree = degree, parametric = parametric, : neighborhood radius 0.005
```

```
## Warning in simpleLoess(y, x, w, span, degree = degree, parametric = parametric, : reciprocal condition number 1
```

```
## Warning in simpleLoess(y, x, w, span, degree = degree, parametric = parametric, : There are other near singularities as well. 1.01
```

```
## Warning in simpleLoess(y, x, w, span, degree = degree, parametric = parametric, : zero-width neighborhood. make span bigger
```

```
## Warning: Computation failed in `stat_smooth()`:
## NA/NaN/Inf in foreign function call (arg 5)
```

```
## Warning in simpleLoess(y, x, w, span, degree = degree, parametric = parametric, : at -0.005
```

```
## Warning in simpleLoess(y, x, w, span, degree = degree, parametric = parametric, : radius 2.5e-05
```

```
## Warning in simpleLoess(y, x, w, span, degree = degree, parametric = parametric, : all data on boundary of neighborhood. make span bigger
```

```
## Warning in simpleLoess(y, x, w, span, degree = degree, parametric = parametric, : pseudoinverse used at -0.005
```

```
## Warning in simpleLoess(y, x, w, span, degree = degree, parametric = parametric, : neighborhood radius 0.005
```

```
## Warning in simpleLoess(y, x, w, span, degree = degree, parametric = parametric, : reciprocal condition number 1
```

```
## Warning in simpleLoess(y, x, w, span, degree = degree, parametric = parametric, : There are other near singularities as well. 1.01
```

```
## Warning in simpleLoess(y, x, w, span, degree = degree, parametric = parametric, : zero-width neighborhood. make span bigger
```

```
## Warning: Computation failed in `stat_smooth()`:
## NA/NaN/Inf in foreign function call (arg 5)
```

```
## Warning in simpleLoess(y, x, w, span, degree = degree, parametric = parametric, : at -0.005
```

```
## Warning in simpleLoess(y, x, w, span, degree = degree, parametric = parametric, : radius 2.5e-05
```

```
## Warning in simpleLoess(y, x, w, span, degree = degree, parametric = parametric, : all data on boundary of neighborhood. make span bigger
```

```
## Warning in simpleLoess(y, x, w, span, degree = degree, parametric = parametric, : pseudoinverse used at -0.005
```

```
## Warning in simpleLoess(y, x, w, span, degree = degree, parametric = parametric, : neighborhood radius 0.005
```

```
## Warning in simpleLoess(y, x, w, span, degree = degree, parametric = parametric, : reciprocal condition number 1
```

```
## Warning in simpleLoess(y, x, w, span, degree = degree, parametric = parametric, : There are other near singularities as well. 1.01
```

```
## Warning in simpleLoess(y, x, w, span, degree = degree, parametric = parametric, : zero-width neighborhood. make span bigger
```

```
## Warning: Computation failed in `stat_smooth()`:
## NA/NaN/Inf in foreign function call (arg 5)
```

```
## Warning in simpleLoess(y, x, w, span, degree = degree, parametric = parametric, : at -0.005
```

```
## Warning in simpleLoess(y, x, w, span, degree = degree, parametric = parametric, : radius 2.5e-05
```

```
## Warning in simpleLoess(y, x, w, span, degree = degree, parametric = parametric, : all data on boundary of neighborhood. make span bigger
```

```
## Warning in simpleLoess(y, x, w, span, degree = degree, parametric = parametric, : pseudoinverse used at -0.005
```

```
## Warning in simpleLoess(y, x, w, span, degree = degree, parametric = parametric, : neighborhood radius 0.005
```

```
## Warning in simpleLoess(y, x, w, span, degree = degree, parametric = parametric, : reciprocal condition number 1
```

```
## Warning in simpleLoess(y, x, w, span, degree = degree, parametric = parametric, : There are other near singularities as well. 1.01
```

```
## Warning in simpleLoess(y, x, w, span, degree = degree, parametric = parametric, : zero-width neighborhood. make span bigger
```

```
## Warning: Computation failed in `stat_smooth()`:
## NA/NaN/Inf in foreign function call (arg 5)
```

```
## Warning in simpleLoess(y, x, w, span, degree = degree, parametric = parametric, : at -0.005
```

```
## Warning in simpleLoess(y, x, w, span, degree = degree, parametric = parametric, : radius 2.5e-05
```

```
## Warning in simpleLoess(y, x, w, span, degree = degree, parametric = parametric, : all data on boundary of neighborhood. make span bigger
```

```
## Warning in simpleLoess(y, x, w, span, degree = degree, parametric = parametric, : pseudoinverse used at -0.005
```

```
## Warning in simpleLoess(y, x, w, span, degree = degree, parametric = parametric, : neighborhood radius 0.005
```

```
## Warning in simpleLoess(y, x, w, span, degree = degree, parametric = parametric, : reciprocal condition number 1
```

```
## Warning in simpleLoess(y, x, w, span, degree = degree, parametric = parametric, : There are other near singularities as well. 1.01
```

```
## Warning in simpleLoess(y, x, w, span, degree = degree, parametric = parametric, : zero-width neighborhood. make span bigger
```

```
## Warning: Computation failed in `stat_smooth()`:
## NA/NaN/Inf in foreign function call (arg 5)
```

```
## Warning in simpleLoess(y, x, w, span, degree = degree, parametric = parametric, : pseudoinverse used at -0.005
```

```
## Warning in simpleLoess(y, x, w, span, degree = degree, parametric = parametric, : neighborhood radius 1.005
```

```
## Warning in simpleLoess(y, x, w, span, degree = degree, parametric = parametric, : reciprocal condition number 0
```

```
## Warning in simpleLoess(y, x, w, span, degree = degree, parametric = parametric, : There are other near singularities as well. 1.01
```

```
## Warning in predLoess(object$y, object$x, newx = if (is.null(newdata)) object$x else if (is.data.frame(newdata)) as.matrix(model.frame(delete.response(terms(object)), : pseudoinverse used at -0.005
```

```
## Warning in predLoess(object$y, object$x, newx = if (is.null(newdata)) object$x else if (is.data.frame(newdata)) as.matrix(model.frame(delete.response(terms(object)), : neighborhood radius 1.005
```

```
## Warning in predLoess(object$y, object$x, newx = if (is.null(newdata)) object$x else if (is.data.frame(newdata)) as.matrix(model.frame(delete.response(terms(object)), : reciprocal condition number 0
```

```
## Warning in predLoess(object$y, object$x, newx = if (is.null(newdata)) object$x else if (is.data.frame(newdata)) as.matrix(model.frame(delete.response(terms(object)), : There are other near
## singularities as well. 1.01
```

```
## Warning in simpleLoess(y, x, w, span, degree = degree, parametric = parametric, : at -0.005
```

```
## Warning in simpleLoess(y, x, w, span, degree = degree, parametric = parametric, : radius 2.5e-05
```

```
## Warning in simpleLoess(y, x, w, span, degree = degree, parametric = parametric, : all data on boundary of neighborhood. make span bigger
```

```
## Warning in simpleLoess(y, x, w, span, degree = degree, parametric = parametric, : pseudoinverse used at -0.005
```

```
## Warning in simpleLoess(y, x, w, span, degree = degree, parametric = parametric, : neighborhood radius 0.005
```

```
## Warning in simpleLoess(y, x, w, span, degree = degree, parametric = parametric, : reciprocal condition number 1
```

```
## Warning in simpleLoess(y, x, w, span, degree = degree, parametric = parametric, : There are other near singularities as well. 1.01
```

```
## Warning in simpleLoess(y, x, w, span, degree = degree, parametric = parametric, : zero-width neighborhood. make span bigger
```

```
## Warning: Computation failed in `stat_smooth()`:
## NA/NaN/Inf in foreign function call (arg 5)
```

```
## Warning in simpleLoess(y, x, w, span, degree = degree, parametric = parametric, : at -0.005
```

```
## Warning in simpleLoess(y, x, w, span, degree = degree, parametric = parametric, : radius 2.5e-05
```

```
## Warning in simpleLoess(y, x, w, span, degree = degree, parametric = parametric, : all data on boundary of neighborhood. make span bigger
```

```
## Warning in simpleLoess(y, x, w, span, degree = degree, parametric = parametric, : pseudoinverse used at -0.005
```

```
## Warning in simpleLoess(y, x, w, span, degree = degree, parametric = parametric, : neighborhood radius 0.005
```

```
## Warning in simpleLoess(y, x, w, span, degree = degree, parametric = parametric, : reciprocal condition number 1
```

```
## Warning in simpleLoess(y, x, w, span, degree = degree, parametric = parametric, : There are other near singularities as well. 1.01
```

```
## Warning in simpleLoess(y, x, w, span, degree = degree, parametric = parametric, : zero-width neighborhood. make span bigger
```

```
## Warning: Computation failed in `stat_smooth()`:
## NA/NaN/Inf in foreign function call (arg 5)
```

```
## Warning in simpleLoess(y, x, w, span, degree = degree, parametric = parametric, : at -0.005
```

```
## Warning in simpleLoess(y, x, w, span, degree = degree, parametric = parametric, : radius 2.5e-05
```

```
## Warning in simpleLoess(y, x, w, span, degree = degree, parametric = parametric, : all data on boundary of neighborhood. make span bigger
```

```
## Warning in simpleLoess(y, x, w, span, degree = degree, parametric = parametric, : pseudoinverse used at -0.005
```

```
## Warning in simpleLoess(y, x, w, span, degree = degree, parametric = parametric, : neighborhood radius 0.005
```

```
## Warning in simpleLoess(y, x, w, span, degree = degree, parametric = parametric, : reciprocal condition number 1
```

```
## Warning in simpleLoess(y, x, w, span, degree = degree, parametric = parametric, : There are other near singularities as well. 1.01
```

```
## Warning in simpleLoess(y, x, w, span, degree = degree, parametric = parametric, : zero-width neighborhood. make span bigger
```

```
## Warning: Computation failed in `stat_smooth()`:
## NA/NaN/Inf in foreign function call (arg 5)
```

```
## Warning in simpleLoess(y, x, w, span, degree = degree, parametric = parametric, : at -0.005
```

```
## Warning in simpleLoess(y, x, w, span, degree = degree, parametric = parametric, : radius 2.5e-05
```

```
## Warning in simpleLoess(y, x, w, span, degree = degree, parametric = parametric, : all data on boundary of neighborhood. make span bigger
```

```
## Warning in simpleLoess(y, x, w, span, degree = degree, parametric = parametric, : pseudoinverse used at -0.005
```

```
## Warning in simpleLoess(y, x, w, span, degree = degree, parametric = parametric, : neighborhood radius 0.005
```

```
## Warning in simpleLoess(y, x, w, span, degree = degree, parametric = parametric, : reciprocal condition number 1
```

```
## Warning in simpleLoess(y, x, w, span, degree = degree, parametric = parametric, : There are other near singularities as well. 1.01
```

```
## Warning in simpleLoess(y, x, w, span, degree = degree, parametric = parametric, : zero-width neighborhood. make span bigger
```

```
## Warning: Computation failed in `stat_smooth()`:
## NA/NaN/Inf in foreign function call (arg 5)
```

```
## Warning in simpleLoess(y, x, w, span, degree = degree, parametric = parametric, : at -0.005
```

```
## Warning in simpleLoess(y, x, w, span, degree = degree, parametric = parametric, : radius 2.5e-05
```

```
## Warning in simpleLoess(y, x, w, span, degree = degree, parametric = parametric, : all data on boundary of neighborhood. make span bigger
```

```
## Warning in simpleLoess(y, x, w, span, degree = degree, parametric = parametric, : pseudoinverse used at -0.005
```

```
## Warning in simpleLoess(y, x, w, span, degree = degree, parametric = parametric, : neighborhood radius 0.005
```

```
## Warning in simpleLoess(y, x, w, span, degree = degree, parametric = parametric, : reciprocal condition number 1
```

```
## Warning in simpleLoess(y, x, w, span, degree = degree, parametric = parametric, : There are other near singularities as well. 1.01
```

```
## Warning in simpleLoess(y, x, w, span, degree = degree, parametric = parametric, : zero-width neighborhood. make span bigger
```

```
## Warning: Computation failed in `stat_smooth()`:
## NA/NaN/Inf in foreign function call (arg 5)
```

```
## Warning in simpleLoess(y, x, w, span, degree = degree, parametric = parametric, : at -0.005
```

```
## Warning in simpleLoess(y, x, w, span, degree = degree, parametric = parametric, : radius 2.5e-05
```

```
## Warning in simpleLoess(y, x, w, span, degree = degree, parametric = parametric, : all data on boundary of neighborhood. make span bigger
```

```
## Warning in simpleLoess(y, x, w, span, degree = degree, parametric = parametric, : pseudoinverse used at -0.005
```

```
## Warning in simpleLoess(y, x, w, span, degree = degree, parametric = parametric, : neighborhood radius 0.005
```

```
## Warning in simpleLoess(y, x, w, span, degree = degree, parametric = parametric, : reciprocal condition number 1
```

```
## Warning in simpleLoess(y, x, w, span, degree = degree, parametric = parametric, : There are other near singularities as well. 1.01
```

```
## Warning in simpleLoess(y, x, w, span, degree = degree, parametric = parametric, : zero-width neighborhood. make span bigger
```

```
## Warning: Computation failed in `stat_smooth()`:
## NA/NaN/Inf in foreign function call (arg 5)
```

```
## Warning in simpleLoess(y, x, w, span, degree = degree, parametric = parametric, : at -0.005
```

```
## Warning in simpleLoess(y, x, w, span, degree = degree, parametric = parametric, : radius 2.5e-05
```

```
## Warning in simpleLoess(y, x, w, span, degree = degree, parametric = parametric, : all data on boundary of neighborhood. make span bigger
```

```
## Warning in simpleLoess(y, x, w, span, degree = degree, parametric = parametric, : pseudoinverse used at -0.005
```

```
## Warning in simpleLoess(y, x, w, span, degree = degree, parametric = parametric, : neighborhood radius 0.005
```

```
## Warning in simpleLoess(y, x, w, span, degree = degree, parametric = parametric, : reciprocal condition number 1
```

```
## Warning in simpleLoess(y, x, w, span, degree = degree, parametric = parametric, : There are other near singularities as well. 1.01
```

```
## Warning in simpleLoess(y, x, w, span, degree = degree, parametric = parametric, : zero-width neighborhood. make span bigger
```

```
## Warning: Computation failed in `stat_smooth()`:
## NA/NaN/Inf in foreign function call (arg 5)
```

```
## Warning in simpleLoess(y, x, w, span, degree = degree, parametric = parametric, : pseudoinverse used at -0.005
```

```
## Warning in simpleLoess(y, x, w, span, degree = degree, parametric = parametric, : neighborhood radius 1.005
```

```
## Warning in simpleLoess(y, x, w, span, degree = degree, parametric = parametric, : reciprocal condition number 0
```

```
## Warning in simpleLoess(y, x, w, span, degree = degree, parametric = parametric, : There are other near singularities as well. 1.01
```

```
## Warning in predLoess(object$y, object$x, newx = if (is.null(newdata)) object$x else if (is.data.frame(newdata)) as.matrix(model.frame(delete.response(terms(object)), : pseudoinverse used at -0.005
```

```
## Warning in predLoess(object$y, object$x, newx = if (is.null(newdata)) object$x else if (is.data.frame(newdata)) as.matrix(model.frame(delete.response(terms(object)), : neighborhood radius 1.005
```

```
## Warning in predLoess(object$y, object$x, newx = if (is.null(newdata)) object$x else if (is.data.frame(newdata)) as.matrix(model.frame(delete.response(terms(object)), : reciprocal condition number 0
```

```
## Warning in predLoess(object$y, object$x, newx = if (is.null(newdata)) object$x else if (is.data.frame(newdata)) as.matrix(model.frame(delete.response(terms(object)), : There are other near
## singularities as well. 1.01
```

```
## Warning in simpleLoess(y, x, w, span, degree = degree, parametric = parametric, : pseudoinverse used at -0.005
```

```
## Warning in simpleLoess(y, x, w, span, degree = degree, parametric = parametric, : neighborhood radius 1.005
```

```
## Warning in simpleLoess(y, x, w, span, degree = degree, parametric = parametric, : reciprocal condition number 0
```

```
## Warning in simpleLoess(y, x, w, span, degree = degree, parametric = parametric, : There are other near singularities as well. 1.01
```

```
## Warning in predLoess(object$y, object$x, newx = if (is.null(newdata)) object$x else if (is.data.frame(newdata)) as.matrix(model.frame(delete.response(terms(object)), : pseudoinverse used at -0.005
```

```
## Warning in predLoess(object$y, object$x, newx = if (is.null(newdata)) object$x else if (is.data.frame(newdata)) as.matrix(model.frame(delete.response(terms(object)), : neighborhood radius 1.005
```

```
## Warning in predLoess(object$y, object$x, newx = if (is.null(newdata)) object$x else if (is.data.frame(newdata)) as.matrix(model.frame(delete.response(terms(object)), : reciprocal condition number 0
```

```
## Warning in predLoess(object$y, object$x, newx = if (is.null(newdata)) object$x else if (is.data.frame(newdata)) as.matrix(model.frame(delete.response(terms(object)), : There are other near
## singularities as well. 1.01
```

```
## Warning in simpleLoess(y, x, w, span, degree = degree, parametric = parametric, : at -0.005
```

```
## Warning in simpleLoess(y, x, w, span, degree = degree, parametric = parametric, : radius 2.5e-05
```

```
## Warning in simpleLoess(y, x, w, span, degree = degree, parametric = parametric, : all data on boundary of neighborhood. make span bigger
```

```
## Warning in simpleLoess(y, x, w, span, degree = degree, parametric = parametric, : pseudoinverse used at -0.005
```

```
## Warning in simpleLoess(y, x, w, span, degree = degree, parametric = parametric, : neighborhood radius 0.005
```

```
## Warning in simpleLoess(y, x, w, span, degree = degree, parametric = parametric, : reciprocal condition number 1
```

```
## Warning in simpleLoess(y, x, w, span, degree = degree, parametric = parametric, : There are other near singularities as well. 1.01
```

```
## Warning in simpleLoess(y, x, w, span, degree = degree, parametric = parametric, : zero-width neighborhood. make span bigger
```

```
## Warning: Computation failed in `stat_smooth()`:
## NA/NaN/Inf in foreign function call (arg 5)
```

```
## Warning in simpleLoess(y, x, w, span, degree = degree, parametric = parametric, : at -0.005
```

```
## Warning in simpleLoess(y, x, w, span, degree = degree, parametric = parametric, : radius 2.5e-05
```

```
## Warning in simpleLoess(y, x, w, span, degree = degree, parametric = parametric, : all data on boundary of neighborhood. make span bigger
```

```
## Warning in simpleLoess(y, x, w, span, degree = degree, parametric = parametric, : pseudoinverse used at -0.005
```

```
## Warning in simpleLoess(y, x, w, span, degree = degree, parametric = parametric, : neighborhood radius 0.005
```

```
## Warning in simpleLoess(y, x, w, span, degree = degree, parametric = parametric, : reciprocal condition number 1
```

```
## Warning in simpleLoess(y, x, w, span, degree = degree, parametric = parametric, : There are other near singularities as well. 1.01
```

```
## Warning in simpleLoess(y, x, w, span, degree = degree, parametric = parametric, : zero-width neighborhood. make span bigger
```

```
## Warning: Computation failed in `stat_smooth()`:
## NA/NaN/Inf in foreign function call (arg 5)
```

```
## Warning in simpleLoess(y, x, w, span, degree = degree, parametric = parametric, : pseudoinverse used at -0.005
```

```
## Warning in simpleLoess(y, x, w, span, degree = degree, parametric = parametric, : neighborhood radius 1.005
```

```
## Warning in simpleLoess(y, x, w, span, degree = degree, parametric = parametric, : reciprocal condition number 0
```

```
## Warning in simpleLoess(y, x, w, span, degree = degree, parametric = parametric, : There are other near singularities as well. 1.01
```

```
## Warning in predLoess(object$y, object$x, newx = if (is.null(newdata)) object$x else if (is.data.frame(newdata)) as.matrix(model.frame(delete.response(terms(object)), : pseudoinverse used at -0.005
```

```
## Warning in predLoess(object$y, object$x, newx = if (is.null(newdata)) object$x else if (is.data.frame(newdata)) as.matrix(model.frame(delete.response(terms(object)), : neighborhood radius 1.005
```

```
## Warning in predLoess(object$y, object$x, newx = if (is.null(newdata)) object$x else if (is.data.frame(newdata)) as.matrix(model.frame(delete.response(terms(object)), : reciprocal condition number 0
```

```
## Warning in predLoess(object$y, object$x, newx = if (is.null(newdata)) object$x else if (is.data.frame(newdata)) as.matrix(model.frame(delete.response(terms(object)), : There are other near
## singularities as well. 1.01
```

```
## Warning in simpleLoess(y, x, w, span, degree = degree, parametric = parametric, : at -0.005
```

```
## Warning in simpleLoess(y, x, w, span, degree = degree, parametric = parametric, : radius 2.5e-05
```

```
## Warning in simpleLoess(y, x, w, span, degree = degree, parametric = parametric, : all data on boundary of neighborhood. make span bigger
```

```
## Warning in simpleLoess(y, x, w, span, degree = degree, parametric = parametric, : pseudoinverse used at -0.005
```

```
## Warning in simpleLoess(y, x, w, span, degree = degree, parametric = parametric, : neighborhood radius 0.005
```

```
## Warning in simpleLoess(y, x, w, span, degree = degree, parametric = parametric, : reciprocal condition number 1
```

```
## Warning in simpleLoess(y, x, w, span, degree = degree, parametric = parametric, : There are other near singularities as well. 1.01
```

```
## Warning in simpleLoess(y, x, w, span, degree = degree, parametric = parametric, : zero-width neighborhood. make span bigger
```

```
## Warning: Computation failed in `stat_smooth()`:
## NA/NaN/Inf in foreign function call (arg 5)
```

```
## Warning in simpleLoess(y, x, w, span, degree = degree, parametric = parametric, : at -0.005
```

```
## Warning in simpleLoess(y, x, w, span, degree = degree, parametric = parametric, : radius 2.5e-05
```

```
## Warning in simpleLoess(y, x, w, span, degree = degree, parametric = parametric, : all data on boundary of neighborhood. make span bigger
```

```
## Warning in simpleLoess(y, x, w, span, degree = degree, parametric = parametric, : pseudoinverse used at -0.005
```

```
## Warning in simpleLoess(y, x, w, span, degree = degree, parametric = parametric, : neighborhood radius 0.005
```

```
## Warning in simpleLoess(y, x, w, span, degree = degree, parametric = parametric, : reciprocal condition number 1
```

```
## Warning in simpleLoess(y, x, w, span, degree = degree, parametric = parametric, : There are other near singularities as well. 1.01
```

```
## Warning in simpleLoess(y, x, w, span, degree = degree, parametric = parametric, : zero-width neighborhood. make span bigger
```

```
## Warning: Computation failed in `stat_smooth()`:
## NA/NaN/Inf in foreign function call (arg 5)
```

```
## Warning in simpleLoess(y, x, w, span, degree = degree, parametric = parametric, : at -0.005
```

```
## Warning in simpleLoess(y, x, w, span, degree = degree, parametric = parametric, : radius 2.5e-05
```

```
## Warning in simpleLoess(y, x, w, span, degree = degree, parametric = parametric, : all data on boundary of neighborhood. make span bigger
```

```
## Warning in simpleLoess(y, x, w, span, degree = degree, parametric = parametric, : pseudoinverse used at -0.005
```

```
## Warning in simpleLoess(y, x, w, span, degree = degree, parametric = parametric, : neighborhood radius 0.005
```

```
## Warning in simpleLoess(y, x, w, span, degree = degree, parametric = parametric, : reciprocal condition number 1
```

```
## Warning in simpleLoess(y, x, w, span, degree = degree, parametric = parametric, : There are other near singularities as well. 1.01
```

```
## Warning in simpleLoess(y, x, w, span, degree = degree, parametric = parametric, : zero-width neighborhood. make span bigger
```

```
## Warning: Computation failed in `stat_smooth()`:
## NA/NaN/Inf in foreign function call (arg 5)
```

```
## Warning in simpleLoess(y, x, w, span, degree = degree, parametric = parametric, : at -0.005
```

```
## Warning in simpleLoess(y, x, w, span, degree = degree, parametric = parametric, : radius 2.5e-05
```

```
## Warning in simpleLoess(y, x, w, span, degree = degree, parametric = parametric, : all data on boundary of neighborhood. make span bigger
```

```
## Warning in simpleLoess(y, x, w, span, degree = degree, parametric = parametric, : pseudoinverse used at -0.005
```

```
## Warning in simpleLoess(y, x, w, span, degree = degree, parametric = parametric, : neighborhood radius 0.005
```

```
## Warning in simpleLoess(y, x, w, span, degree = degree, parametric = parametric, : reciprocal condition number 1
```

```
## Warning in simpleLoess(y, x, w, span, degree = degree, parametric = parametric, : There are other near singularities as well. 1.01
```

```
## Warning in simpleLoess(y, x, w, span, degree = degree, parametric = parametric, : zero-width neighborhood. make span bigger
```

```
## Warning: Computation failed in `stat_smooth()`:
## NA/NaN/Inf in foreign function call (arg 5)
```

```
## Warning in simpleLoess(y, x, w, span, degree = degree, parametric = parametric, : at -0.005
```

```
## Warning in simpleLoess(y, x, w, span, degree = degree, parametric = parametric, : radius 2.5e-05
```

```
## Warning in simpleLoess(y, x, w, span, degree = degree, parametric = parametric, : all data on boundary of neighborhood. make span bigger
```

```
## Warning in simpleLoess(y, x, w, span, degree = degree, parametric = parametric, : pseudoinverse used at -0.005
```

```
## Warning in simpleLoess(y, x, w, span, degree = degree, parametric = parametric, : neighborhood radius 0.005
```

```
## Warning in simpleLoess(y, x, w, span, degree = degree, parametric = parametric, : reciprocal condition number 1
```

```
## Warning in simpleLoess(y, x, w, span, degree = degree, parametric = parametric, : There are other near singularities as well. 1.01
```

```
## Warning in simpleLoess(y, x, w, span, degree = degree, parametric = parametric, : zero-width neighborhood. make span bigger
```

```
## Warning: Computation failed in `stat_smooth()`:
## NA/NaN/Inf in foreign function call (arg 5)
```

```
## Warning in simpleLoess(y, x, w, span, degree = degree, parametric = parametric, : at -0.005
```

```
## Warning in simpleLoess(y, x, w, span, degree = degree, parametric = parametric, : radius 2.5e-05
```

```
## Warning in simpleLoess(y, x, w, span, degree = degree, parametric = parametric, : all data on boundary of neighborhood. make span bigger
```

```
## Warning in simpleLoess(y, x, w, span, degree = degree, parametric = parametric, : pseudoinverse used at -0.005
```

```
## Warning in simpleLoess(y, x, w, span, degree = degree, parametric = parametric, : neighborhood radius 0.005
```

```
## Warning in simpleLoess(y, x, w, span, degree = degree, parametric = parametric, : reciprocal condition number 1
```

```
## Warning in simpleLoess(y, x, w, span, degree = degree, parametric = parametric, : There are other near singularities as well. 1.01
```

```
## Warning in simpleLoess(y, x, w, span, degree = degree, parametric = parametric, : zero-width neighborhood. make span bigger
```

```
## Warning: Computation failed in `stat_smooth()`:
## NA/NaN/Inf in foreign function call (arg 5)
```

```
## Warning in simpleLoess(y, x, w, span, degree = degree, parametric = parametric, : at -0.005
```

```
## Warning in simpleLoess(y, x, w, span, degree = degree, parametric = parametric, : radius 2.5e-05
```

```
## Warning in simpleLoess(y, x, w, span, degree = degree, parametric = parametric, : all data on boundary of neighborhood. make span bigger
```

```
## Warning in simpleLoess(y, x, w, span, degree = degree, parametric = parametric, : pseudoinverse used at -0.005
```

```
## Warning in simpleLoess(y, x, w, span, degree = degree, parametric = parametric, : neighborhood radius 0.005
```

```
## Warning in simpleLoess(y, x, w, span, degree = degree, parametric = parametric, : reciprocal condition number 1
```

```
## Warning in simpleLoess(y, x, w, span, degree = degree, parametric = parametric, : There are other near singularities as well. 1.01
```

```
## Warning in simpleLoess(y, x, w, span, degree = degree, parametric = parametric, : zero-width neighborhood. make span bigger
```

```
## Warning: Computation failed in `stat_smooth()`:
## NA/NaN/Inf in foreign function call (arg 5)
```

```
## Warning in simpleLoess(y, x, w, span, degree = degree, parametric = parametric, : pseudoinverse used at -0.005
```

```
## Warning in simpleLoess(y, x, w, span, degree = degree, parametric = parametric, : neighborhood radius 1.005
```

```
## Warning in simpleLoess(y, x, w, span, degree = degree, parametric = parametric, : reciprocal condition number 0
```

```
## Warning in simpleLoess(y, x, w, span, degree = degree, parametric = parametric, : There are other near singularities as well. 1.01
```

```
## Warning in predLoess(object$y, object$x, newx = if (is.null(newdata)) object$x else if (is.data.frame(newdata)) as.matrix(model.frame(delete.response(terms(object)), : pseudoinverse used at -0.005
```

```
## Warning in predLoess(object$y, object$x, newx = if (is.null(newdata)) object$x else if (is.data.frame(newdata)) as.matrix(model.frame(delete.response(terms(object)), : neighborhood radius 1.005
```

```
## Warning in predLoess(object$y, object$x, newx = if (is.null(newdata)) object$x else if (is.data.frame(newdata)) as.matrix(model.frame(delete.response(terms(object)), : reciprocal condition number 0
```

```
## Warning in predLoess(object$y, object$x, newx = if (is.null(newdata)) object$x else if (is.data.frame(newdata)) as.matrix(model.frame(delete.response(terms(object)), : There are other near
## singularities as well. 1.01
```

```
## Warning in simpleLoess(y, x, w, span, degree = degree, parametric = parametric, : at -0.005
```

```
## Warning in simpleLoess(y, x, w, span, degree = degree, parametric = parametric, : radius 2.5e-05
```

```
## Warning in simpleLoess(y, x, w, span, degree = degree, parametric = parametric, : all data on boundary of neighborhood. make span bigger
```

```
## Warning in simpleLoess(y, x, w, span, degree = degree, parametric = parametric, : pseudoinverse used at -0.005
```

```
## Warning in simpleLoess(y, x, w, span, degree = degree, parametric = parametric, : neighborhood radius 0.005
```

```
## Warning in simpleLoess(y, x, w, span, degree = degree, parametric = parametric, : reciprocal condition number 1
```

```
## Warning in simpleLoess(y, x, w, span, degree = degree, parametric = parametric, : There are other near singularities as well. 1.01
```

```
## Warning in simpleLoess(y, x, w, span, degree = degree, parametric = parametric, : zero-width neighborhood. make span bigger
```

```
## Warning: Computation failed in `stat_smooth()`:
## NA/NaN/Inf in foreign function call (arg 5)
```

```
## Warning in simpleLoess(y, x, w, span, degree = degree, parametric = parametric, : at -0.005
```

```
## Warning in simpleLoess(y, x, w, span, degree = degree, parametric = parametric, : radius 2.5e-05
```

```
## Warning in simpleLoess(y, x, w, span, degree = degree, parametric = parametric, : all data on boundary of neighborhood. make span bigger
```

```
## Warning in simpleLoess(y, x, w, span, degree = degree, parametric = parametric, : pseudoinverse used at -0.005
```

```
## Warning in simpleLoess(y, x, w, span, degree = degree, parametric = parametric, : neighborhood radius 0.005
```

```
## Warning in simpleLoess(y, x, w, span, degree = degree, parametric = parametric, : reciprocal condition number 1
```

```
## Warning in simpleLoess(y, x, w, span, degree = degree, parametric = parametric, : There are other near singularities as well. 1.01
```

```
## Warning in simpleLoess(y, x, w, span, degree = degree, parametric = parametric, : zero-width neighborhood. make span bigger
```

```
## Warning: Computation failed in `stat_smooth()`:
## NA/NaN/Inf in foreign function call (arg 5)
```

```
## Warning in simpleLoess(y, x, w, span, degree = degree, parametric = parametric, : pseudoinverse used at -0.005
```

```
## Warning in simpleLoess(y, x, w, span, degree = degree, parametric = parametric, : neighborhood radius 1.005
```

```
## Warning in simpleLoess(y, x, w, span, degree = degree, parametric = parametric, : reciprocal condition number 0
```

```
## Warning in simpleLoess(y, x, w, span, degree = degree, parametric = parametric, : There are other near singularities as well. 1.01
```

```
## Warning in predLoess(object$y, object$x, newx = if (is.null(newdata)) object$x else if (is.data.frame(newdata)) as.matrix(model.frame(delete.response(terms(object)), : pseudoinverse used at -0.005
```

```
## Warning in predLoess(object$y, object$x, newx = if (is.null(newdata)) object$x else if (is.data.frame(newdata)) as.matrix(model.frame(delete.response(terms(object)), : neighborhood radius 1.005
```

```
## Warning in predLoess(object$y, object$x, newx = if (is.null(newdata)) object$x else if (is.data.frame(newdata)) as.matrix(model.frame(delete.response(terms(object)), : reciprocal condition number 0
```

```
## Warning in predLoess(object$y, object$x, newx = if (is.null(newdata)) object$x else if (is.data.frame(newdata)) as.matrix(model.frame(delete.response(terms(object)), : There are other near
## singularities as well. 1.01
```

```
## Warning in simpleLoess(y, x, w, span, degree = degree, parametric = parametric, : at -0.005
```

```
## Warning in simpleLoess(y, x, w, span, degree = degree, parametric = parametric, : radius 2.5e-05
```

```
## Warning in simpleLoess(y, x, w, span, degree = degree, parametric = parametric, : all data on boundary of neighborhood. make span bigger
```

```
## Warning in simpleLoess(y, x, w, span, degree = degree, parametric = parametric, : pseudoinverse used at -0.005
```

```
## Warning in simpleLoess(y, x, w, span, degree = degree, parametric = parametric, : neighborhood radius 0.005
```

```
## Warning in simpleLoess(y, x, w, span, degree = degree, parametric = parametric, : reciprocal condition number 1
```

```
## Warning in simpleLoess(y, x, w, span, degree = degree, parametric = parametric, : There are other near singularities as well. 1.01
```

```
## Warning in simpleLoess(y, x, w, span, degree = degree, parametric = parametric, : zero-width neighborhood. make span bigger
```

```
## Warning: Computation failed in `stat_smooth()`:
## NA/NaN/Inf in foreign function call (arg 5)
```

```
## Warning in simpleLoess(y, x, w, span, degree = degree, parametric = parametric, : at -0.005
```

```
## Warning in simpleLoess(y, x, w, span, degree = degree, parametric = parametric, : radius 2.5e-05
```

```
## Warning in simpleLoess(y, x, w, span, degree = degree, parametric = parametric, : all data on boundary of neighborhood. make span bigger
```

```
## Warning in simpleLoess(y, x, w, span, degree = degree, parametric = parametric, : pseudoinverse used at -0.005
```

```
## Warning in simpleLoess(y, x, w, span, degree = degree, parametric = parametric, : neighborhood radius 0.005
```

```
## Warning in simpleLoess(y, x, w, span, degree = degree, parametric = parametric, : reciprocal condition number 1
```

```
## Warning in simpleLoess(y, x, w, span, degree = degree, parametric = parametric, : There are other near singularities as well. 1.01
```

```
## Warning in simpleLoess(y, x, w, span, degree = degree, parametric = parametric, : zero-width neighborhood. make span bigger
```

```
## Warning: Computation failed in `stat_smooth()`:
## NA/NaN/Inf in foreign function call (arg 5)
```

```
## Warning in simpleLoess(y, x, w, span, degree = degree, parametric = parametric, : at -0.005
```

```
## Warning in simpleLoess(y, x, w, span, degree = degree, parametric = parametric, : radius 2.5e-05
```

```
## Warning in simpleLoess(y, x, w, span, degree = degree, parametric = parametric, : all data on boundary of neighborhood. make span bigger
```

```
## Warning in simpleLoess(y, x, w, span, degree = degree, parametric = parametric, : pseudoinverse used at -0.005
```

```
## Warning in simpleLoess(y, x, w, span, degree = degree, parametric = parametric, : neighborhood radius 0.005
```

```
## Warning in simpleLoess(y, x, w, span, degree = degree, parametric = parametric, : reciprocal condition number 1
```

```
## Warning in simpleLoess(y, x, w, span, degree = degree, parametric = parametric, : There are other near singularities as well. 1.01
```

```
## Warning in simpleLoess(y, x, w, span, degree = degree, parametric = parametric, : zero-width neighborhood. make span bigger
```

```
## Warning: Computation failed in `stat_smooth()`:
## NA/NaN/Inf in foreign function call (arg 5)
```

```
## Warning in simpleLoess(y, x, w, span, degree = degree, parametric = parametric, : at -0.005
```

```
## Warning in simpleLoess(y, x, w, span, degree = degree, parametric = parametric, : radius 2.5e-05
```

```
## Warning in simpleLoess(y, x, w, span, degree = degree, parametric = parametric, : all data on boundary of neighborhood. make span bigger
```

```
## Warning in simpleLoess(y, x, w, span, degree = degree, parametric = parametric, : pseudoinverse used at -0.005
```

```
## Warning in simpleLoess(y, x, w, span, degree = degree, parametric = parametric, : neighborhood radius 0.005
```

```
## Warning in simpleLoess(y, x, w, span, degree = degree, parametric = parametric, : reciprocal condition number 1
```

```
## Warning in simpleLoess(y, x, w, span, degree = degree, parametric = parametric, : There are other near singularities as well. 1.01
```

```
## Warning in simpleLoess(y, x, w, span, degree = degree, parametric = parametric, : zero-width neighborhood. make span bigger
```

```
## Warning: Computation failed in `stat_smooth()`:
## NA/NaN/Inf in foreign function call (arg 5)
```

```
## Warning in simpleLoess(y, x, w, span, degree = degree, parametric = parametric, : pseudoinverse used at -0.005
```

```
## Warning in simpleLoess(y, x, w, span, degree = degree, parametric = parametric, : neighborhood radius 1.005
```

```
## Warning in simpleLoess(y, x, w, span, degree = degree, parametric = parametric, : reciprocal condition number 0
```

```
## Warning in simpleLoess(y, x, w, span, degree = degree, parametric = parametric, : There are other near singularities as well. 1.01
```

```
## Warning in predLoess(object$y, object$x, newx = if (is.null(newdata)) object$x else if (is.data.frame(newdata)) as.matrix(model.frame(delete.response(terms(object)), : pseudoinverse used at -0.005
```

```
## Warning in predLoess(object$y, object$x, newx = if (is.null(newdata)) object$x else if (is.data.frame(newdata)) as.matrix(model.frame(delete.response(terms(object)), : neighborhood radius 1.005
```

```
## Warning in predLoess(object$y, object$x, newx = if (is.null(newdata)) object$x else if (is.data.frame(newdata)) as.matrix(model.frame(delete.response(terms(object)), : reciprocal condition number 0
```

```
## Warning in predLoess(object$y, object$x, newx = if (is.null(newdata)) object$x else if (is.data.frame(newdata)) as.matrix(model.frame(delete.response(terms(object)), : There are other near
## singularities as well. 1.01
```

```
## Warning in simpleLoess(y, x, w, span, degree = degree, parametric = parametric, : at -0.005
```

```
## Warning in simpleLoess(y, x, w, span, degree = degree, parametric = parametric, : radius 2.5e-05
```

```
## Warning in simpleLoess(y, x, w, span, degree = degree, parametric = parametric, : all data on boundary of neighborhood. make span bigger
```

```
## Warning in simpleLoess(y, x, w, span, degree = degree, parametric = parametric, : pseudoinverse used at -0.005
```

```
## Warning in simpleLoess(y, x, w, span, degree = degree, parametric = parametric, : neighborhood radius 0.005
```

```
## Warning in simpleLoess(y, x, w, span, degree = degree, parametric = parametric, : reciprocal condition number 1
```

```
## Warning in simpleLoess(y, x, w, span, degree = degree, parametric = parametric, : There are other near singularities as well. 1.01
```

```
## Warning in simpleLoess(y, x, w, span, degree = degree, parametric = parametric, : zero-width neighborhood. make span bigger
```

```
## Warning: Computation failed in `stat_smooth()`:
## NA/NaN/Inf in foreign function call (arg 5)
```

```
## Warning in simpleLoess(y, x, w, span, degree = degree, parametric = parametric, : at -0.005
```

```
## Warning in simpleLoess(y, x, w, span, degree = degree, parametric = parametric, : radius 2.5e-05
```

```
## Warning in simpleLoess(y, x, w, span, degree = degree, parametric = parametric, : all data on boundary of neighborhood. make span bigger
```

```
## Warning in simpleLoess(y, x, w, span, degree = degree, parametric = parametric, : pseudoinverse used at -0.005
```

```
## Warning in simpleLoess(y, x, w, span, degree = degree, parametric = parametric, : neighborhood radius 0.005
```

```
## Warning in simpleLoess(y, x, w, span, degree = degree, parametric = parametric, : reciprocal condition number 1
```

```
## Warning in simpleLoess(y, x, w, span, degree = degree, parametric = parametric, : There are other near singularities as well. 1.01
```

```
## Warning in simpleLoess(y, x, w, span, degree = degree, parametric = parametric, : zero-width neighborhood. make span bigger
```

```
## Warning: Computation failed in `stat_smooth()`:
## NA/NaN/Inf in foreign function call (arg 5)
```

```
## Warning in simpleLoess(y, x, w, span, degree = degree, parametric = parametric, : at -0.005
```

```
## Warning in simpleLoess(y, x, w, span, degree = degree, parametric = parametric, : radius 2.5e-05
```

```
## Warning in simpleLoess(y, x, w, span, degree = degree, parametric = parametric, : all data on boundary of neighborhood. make span bigger
```

```
## Warning in simpleLoess(y, x, w, span, degree = degree, parametric = parametric, : pseudoinverse used at -0.005
```

```
## Warning in simpleLoess(y, x, w, span, degree = degree, parametric = parametric, : neighborhood radius 0.005
```

```
## Warning in simpleLoess(y, x, w, span, degree = degree, parametric = parametric, : reciprocal condition number 1
```

```
## Warning in simpleLoess(y, x, w, span, degree = degree, parametric = parametric, : There are other near singularities as well. 1.01
```

```
## Warning in simpleLoess(y, x, w, span, degree = degree, parametric = parametric, : zero-width neighborhood. make span bigger
```

```
## Warning: Computation failed in `stat_smooth()`:
## NA/NaN/Inf in foreign function call (arg 5)
```

```
## Warning in simpleLoess(y, x, w, span, degree = degree, parametric = parametric, : at -0.005
```

```
## Warning in simpleLoess(y, x, w, span, degree = degree, parametric = parametric, : radius 2.5e-05
```

```
## Warning in simpleLoess(y, x, w, span, degree = degree, parametric = parametric, : all data on boundary of neighborhood. make span bigger
```

```
## Warning in simpleLoess(y, x, w, span, degree = degree, parametric = parametric, : pseudoinverse used at -0.005
```

```
## Warning in simpleLoess(y, x, w, span, degree = degree, parametric = parametric, : neighborhood radius 0.005
```

```
## Warning in simpleLoess(y, x, w, span, degree = degree, parametric = parametric, : reciprocal condition number 1
```

```
## Warning in simpleLoess(y, x, w, span, degree = degree, parametric = parametric, : There are other near singularities as well. 1.01
```

```
## Warning in simpleLoess(y, x, w, span, degree = degree, parametric = parametric, : zero-width neighborhood. make span bigger
```

```
## Warning: Computation failed in `stat_smooth()`:
## NA/NaN/Inf in foreign function call (arg 5)
```

```
## Warning in simpleLoess(y, x, w, span, degree = degree, parametric = parametric, : at -0.005
```

```
## Warning in simpleLoess(y, x, w, span, degree = degree, parametric = parametric, : radius 2.5e-05
```

```
## Warning in simpleLoess(y, x, w, span, degree = degree, parametric = parametric, : all data on boundary of neighborhood. make span bigger
```

```
## Warning in simpleLoess(y, x, w, span, degree = degree, parametric = parametric, : pseudoinverse used at -0.005
```

```
## Warning in simpleLoess(y, x, w, span, degree = degree, parametric = parametric, : neighborhood radius 0.005
```

```
## Warning in simpleLoess(y, x, w, span, degree = degree, parametric = parametric, : reciprocal condition number 1
```

```
## Warning in simpleLoess(y, x, w, span, degree = degree, parametric = parametric, : There are other near singularities as well. 1.01
```

```
## Warning in simpleLoess(y, x, w, span, degree = degree, parametric = parametric, : zero-width neighborhood. make span bigger
```

```
## Warning: Computation failed in `stat_smooth()`:
## NA/NaN/Inf in foreign function call (arg 5)
```

```
## Warning in simpleLoess(y, x, w, span, degree = degree, parametric = parametric, : at -0.005
```

```
## Warning in simpleLoess(y, x, w, span, degree = degree, parametric = parametric, : radius 2.5e-05
```

```
## Warning in simpleLoess(y, x, w, span, degree = degree, parametric = parametric, : all data on boundary of neighborhood. make span bigger
```

```
## Warning in simpleLoess(y, x, w, span, degree = degree, parametric = parametric, : pseudoinverse used at -0.005
```

```
## Warning in simpleLoess(y, x, w, span, degree = degree, parametric = parametric, : neighborhood radius 0.005
```

```
## Warning in simpleLoess(y, x, w, span, degree = degree, parametric = parametric, : reciprocal condition number 1
```

```
## Warning in simpleLoess(y, x, w, span, degree = degree, parametric = parametric, : There are other near singularities as well. 1.01
```

```
## Warning in simpleLoess(y, x, w, span, degree = degree, parametric = parametric, : zero-width neighborhood. make span bigger
```

```
## Warning: Computation failed in `stat_smooth()`:
## NA/NaN/Inf in foreign function call (arg 5)
```

```
## Warning in simpleLoess(y, x, w, span, degree = degree, parametric = parametric, : at -0.005
```

```
## Warning in simpleLoess(y, x, w, span, degree = degree, parametric = parametric, : radius 2.5e-05
```

```
## Warning in simpleLoess(y, x, w, span, degree = degree, parametric = parametric, : all data on boundary of neighborhood. make span bigger
```

```
## Warning in simpleLoess(y, x, w, span, degree = degree, parametric = parametric, : pseudoinverse used at -0.005
```

```
## Warning in simpleLoess(y, x, w, span, degree = degree, parametric = parametric, : neighborhood radius 0.005
```

```
## Warning in simpleLoess(y, x, w, span, degree = degree, parametric = parametric, : reciprocal condition number 1
```

```
## Warning in simpleLoess(y, x, w, span, degree = degree, parametric = parametric, : There are other near singularities as well. 1.01
```

```
## Warning in simpleLoess(y, x, w, span, degree = degree, parametric = parametric, : zero-width neighborhood. make span bigger
```

```
## Warning: Computation failed in `stat_smooth()`:
## NA/NaN/Inf in foreign function call (arg 5)
```

```
## Warning in simpleLoess(y, x, w, span, degree = degree, parametric = parametric, : at -0.005
```

```
## Warning in simpleLoess(y, x, w, span, degree = degree, parametric = parametric, : radius 2.5e-05
```

```
## Warning in simpleLoess(y, x, w, span, degree = degree, parametric = parametric, : all data on boundary of neighborhood. make span bigger
```

```
## Warning in simpleLoess(y, x, w, span, degree = degree, parametric = parametric, : pseudoinverse used at -0.005
```

```
## Warning in simpleLoess(y, x, w, span, degree = degree, parametric = parametric, : neighborhood radius 0.005
```

```
## Warning in simpleLoess(y, x, w, span, degree = degree, parametric = parametric, : reciprocal condition number 1
```

```
## Warning in simpleLoess(y, x, w, span, degree = degree, parametric = parametric, : There are other near singularities as well. 1.01
```

```
## Warning in simpleLoess(y, x, w, span, degree = degree, parametric = parametric, : zero-width neighborhood. make span bigger
```

```
## Warning: Computation failed in `stat_smooth()`:
## NA/NaN/Inf in foreign function call (arg 5)
```

```
## Warning in simpleLoess(y, x, w, span, degree = degree, parametric = parametric, : pseudoinverse used at -0.005
```

```
## Warning in simpleLoess(y, x, w, span, degree = degree, parametric = parametric, : neighborhood radius 1.005
```

```
## Warning in simpleLoess(y, x, w, span, degree = degree, parametric = parametric, : reciprocal condition number 0
```

```
## Warning in simpleLoess(y, x, w, span, degree = degree, parametric = parametric, : There are other near singularities as well. 1.01
```

```
## Warning in predLoess(object$y, object$x, newx = if (is.null(newdata)) object$x else if (is.data.frame(newdata)) as.matrix(model.frame(delete.response(terms(object)), : pseudoinverse used at -0.005
```

```
## Warning in predLoess(object$y, object$x, newx = if (is.null(newdata)) object$x else if (is.data.frame(newdata)) as.matrix(model.frame(delete.response(terms(object)), : neighborhood radius 1.005
```

```
## Warning in predLoess(object$y, object$x, newx = if (is.null(newdata)) object$x else if (is.data.frame(newdata)) as.matrix(model.frame(delete.response(terms(object)), : reciprocal condition number 0
```

```
## Warning in predLoess(object$y, object$x, newx = if (is.null(newdata)) object$x else if (is.data.frame(newdata)) as.matrix(model.frame(delete.response(terms(object)), : There are other near
## singularities as well. 1.01
```

```
## Warning in simpleLoess(y, x, w, span, degree = degree, parametric = parametric, : at -0.005
```

```
## Warning in simpleLoess(y, x, w, span, degree = degree, parametric = parametric, : radius 2.5e-05
```

```
## Warning in simpleLoess(y, x, w, span, degree = degree, parametric = parametric, : all data on boundary of neighborhood. make span bigger
```

```
## Warning in simpleLoess(y, x, w, span, degree = degree, parametric = parametric, : pseudoinverse used at -0.005
```

```
## Warning in simpleLoess(y, x, w, span, degree = degree, parametric = parametric, : neighborhood radius 0.005
```

```
## Warning in simpleLoess(y, x, w, span, degree = degree, parametric = parametric, : reciprocal condition number 1
```

```
## Warning in simpleLoess(y, x, w, span, degree = degree, parametric = parametric, : There are other near singularities as well. 1.01
```

```
## Warning in simpleLoess(y, x, w, span, degree = degree, parametric = parametric, : zero-width neighborhood. make span bigger
```

```
## Warning: Computation failed in `stat_smooth()`:
## NA/NaN/Inf in foreign function call (arg 5)
```

```
## Warning in simpleLoess(y, x, w, span, degree = degree, parametric = parametric, : pseudoinverse used at -0.005
```

```
## Warning in simpleLoess(y, x, w, span, degree = degree, parametric = parametric, : neighborhood radius 1.005
```

```
## Warning in simpleLoess(y, x, w, span, degree = degree, parametric = parametric, : reciprocal condition number 0
```

```
## Warning in simpleLoess(y, x, w, span, degree = degree, parametric = parametric, : There are other near singularities as well. 1.01
```

```
## Warning in predLoess(object$y, object$x, newx = if (is.null(newdata)) object$x else if (is.data.frame(newdata)) as.matrix(model.frame(delete.response(terms(object)), : pseudoinverse used at -0.005
```

```
## Warning in predLoess(object$y, object$x, newx = if (is.null(newdata)) object$x else if (is.data.frame(newdata)) as.matrix(model.frame(delete.response(terms(object)), : neighborhood radius 1.005
```

```
## Warning in predLoess(object$y, object$x, newx = if (is.null(newdata)) object$x else if (is.data.frame(newdata)) as.matrix(model.frame(delete.response(terms(object)), : reciprocal condition number 0
```

```
## Warning in predLoess(object$y, object$x, newx = if (is.null(newdata)) object$x else if (is.data.frame(newdata)) as.matrix(model.frame(delete.response(terms(object)), : There are other near
## singularities as well. 1.01
```

```
## Warning in simpleLoess(y, x, w, span, degree = degree, parametric = parametric, : at -0.005
```

```
## Warning in simpleLoess(y, x, w, span, degree = degree, parametric = parametric, : radius 2.5e-05
```

```
## Warning in simpleLoess(y, x, w, span, degree = degree, parametric = parametric, : all data on boundary of neighborhood. make span bigger
```

```
## Warning in simpleLoess(y, x, w, span, degree = degree, parametric = parametric, : pseudoinverse used at -0.005
```

```
## Warning in simpleLoess(y, x, w, span, degree = degree, parametric = parametric, : neighborhood radius 0.005
```

```
## Warning in simpleLoess(y, x, w, span, degree = degree, parametric = parametric, : reciprocal condition number 1
```

```
## Warning in simpleLoess(y, x, w, span, degree = degree, parametric = parametric, : There are other near singularities as well. 1.01
```

```
## Warning in simpleLoess(y, x, w, span, degree = degree, parametric = parametric, : zero-width neighborhood. make span bigger
```

```
## Warning: Computation failed in `stat_smooth()`:
## NA/NaN/Inf in foreign function call (arg 5)
```

```
## Warning in simpleLoess(y, x, w, span, degree = degree, parametric = parametric, : at -0.005
```

```
## Warning in simpleLoess(y, x, w, span, degree = degree, parametric = parametric, : radius 2.5e-05
```

```
## Warning in simpleLoess(y, x, w, span, degree = degree, parametric = parametric, : all data on boundary of neighborhood. make span bigger
```

```
## Warning in simpleLoess(y, x, w, span, degree = degree, parametric = parametric, : pseudoinverse used at -0.005
```

```
## Warning in simpleLoess(y, x, w, span, degree = degree, parametric = parametric, : neighborhood radius 0.005
```

```
## Warning in simpleLoess(y, x, w, span, degree = degree, parametric = parametric, : reciprocal condition number 1
```

```
## Warning in simpleLoess(y, x, w, span, degree = degree, parametric = parametric, : There are other near singularities as well. 1.01
```

```
## Warning in simpleLoess(y, x, w, span, degree = degree, parametric = parametric, : zero-width neighborhood. make span bigger
```

```
## Warning: Computation failed in `stat_smooth()`:
## NA/NaN/Inf in foreign function call (arg 5)
```

```
## Warning in simpleLoess(y, x, w, span, degree = degree, parametric = parametric, : at -0.005
```

```
## Warning in simpleLoess(y, x, w, span, degree = degree, parametric = parametric, : radius 2.5e-05
```

```
## Warning in simpleLoess(y, x, w, span, degree = degree, parametric = parametric, : all data on boundary of neighborhood. make span bigger
```

```
## Warning in simpleLoess(y, x, w, span, degree = degree, parametric = parametric, : pseudoinverse used at -0.005
```

```
## Warning in simpleLoess(y, x, w, span, degree = degree, parametric = parametric, : neighborhood radius 0.005
```

```
## Warning in simpleLoess(y, x, w, span, degree = degree, parametric = parametric, : reciprocal condition number 1
```

```
## Warning in simpleLoess(y, x, w, span, degree = degree, parametric = parametric, : There are other near singularities as well. 1.01
```

```
## Warning in simpleLoess(y, x, w, span, degree = degree, parametric = parametric, : zero-width neighborhood. make span bigger
```

```
## Warning: Computation failed in `stat_smooth()`:
## NA/NaN/Inf in foreign function call (arg 5)
```

```
## Warning in simpleLoess(y, x, w, span, degree = degree, parametric = parametric, : at -0.005
```

```
## Warning in simpleLoess(y, x, w, span, degree = degree, parametric = parametric, : radius 2.5e-05
```

```
## Warning in simpleLoess(y, x, w, span, degree = degree, parametric = parametric, : all data on boundary of neighborhood. make span bigger
```

```
## Warning in simpleLoess(y, x, w, span, degree = degree, parametric = parametric, : pseudoinverse used at -0.005
```

```
## Warning in simpleLoess(y, x, w, span, degree = degree, parametric = parametric, : neighborhood radius 0.005
```

```
## Warning in simpleLoess(y, x, w, span, degree = degree, parametric = parametric, : reciprocal condition number 1
```

```
## Warning in simpleLoess(y, x, w, span, degree = degree, parametric = parametric, : There are other near singularities as well. 1.01
```

```
## Warning in simpleLoess(y, x, w, span, degree = degree, parametric = parametric, : zero-width neighborhood. make span bigger
```

```
## Warning: Computation failed in `stat_smooth()`:
## NA/NaN/Inf in foreign function call (arg 5)
```

```
## Warning in simpleLoess(y, x, w, span, degree = degree, parametric = parametric, : at -0.005
```

```
## Warning in simpleLoess(y, x, w, span, degree = degree, parametric = parametric, : radius 2.5e-05
```

```
## Warning in simpleLoess(y, x, w, span, degree = degree, parametric = parametric, : all data on boundary of neighborhood. make span bigger
```

```
## Warning in simpleLoess(y, x, w, span, degree = degree, parametric = parametric, : pseudoinverse used at -0.005
```

```
## Warning in simpleLoess(y, x, w, span, degree = degree, parametric = parametric, : neighborhood radius 0.005
```

```
## Warning in simpleLoess(y, x, w, span, degree = degree, parametric = parametric, : reciprocal condition number 1
```

```
## Warning in simpleLoess(y, x, w, span, degree = degree, parametric = parametric, : There are other near singularities as well. 1.01
```

```
## Warning in simpleLoess(y, x, w, span, degree = degree, parametric = parametric, : zero-width neighborhood. make span bigger
```

```
## Warning: Computation failed in `stat_smooth()`:
## NA/NaN/Inf in foreign function call (arg 5)
```

```
## Warning in simpleLoess(y, x, w, span, degree = degree, parametric = parametric, : at -0.005
```

```
## Warning in simpleLoess(y, x, w, span, degree = degree, parametric = parametric, : radius 2.5e-05
```

```
## Warning in simpleLoess(y, x, w, span, degree = degree, parametric = parametric, : all data on boundary of neighborhood. make span bigger
```

```
## Warning in simpleLoess(y, x, w, span, degree = degree, parametric = parametric, : pseudoinverse used at -0.005
```

```
## Warning in simpleLoess(y, x, w, span, degree = degree, parametric = parametric, : neighborhood radius 0.005
```

```
## Warning in simpleLoess(y, x, w, span, degree = degree, parametric = parametric, : reciprocal condition number 1
```

```
## Warning in simpleLoess(y, x, w, span, degree = degree, parametric = parametric, : There are other near singularities as well. 1.01
```

```
## Warning in simpleLoess(y, x, w, span, degree = degree, parametric = parametric, : zero-width neighborhood. make span bigger
```

```
## Warning: Computation failed in `stat_smooth()`:
## NA/NaN/Inf in foreign function call (arg 5)
```

```
## Warning in simpleLoess(y, x, w, span, degree = degree, parametric = parametric, : at -0.005
```

```
## Warning in simpleLoess(y, x, w, span, degree = degree, parametric = parametric, : radius 2.5e-05
```

```
## Warning in simpleLoess(y, x, w, span, degree = degree, parametric = parametric, : all data on boundary of neighborhood. make span bigger
```

```
## Warning in simpleLoess(y, x, w, span, degree = degree, parametric = parametric, : pseudoinverse used at -0.005
```

```
## Warning in simpleLoess(y, x, w, span, degree = degree, parametric = parametric, : neighborhood radius 0.005
```

```
## Warning in simpleLoess(y, x, w, span, degree = degree, parametric = parametric, : reciprocal condition number 1
```

```
## Warning in simpleLoess(y, x, w, span, degree = degree, parametric = parametric, : There are other near singularities as well. 1.01
```

```
## Warning in simpleLoess(y, x, w, span, degree = degree, parametric = parametric, : zero-width neighborhood. make span bigger
```

```
## Warning: Computation failed in `stat_smooth()`:
## NA/NaN/Inf in foreign function call (arg 5)
```

```
## Warning in simpleLoess(y, x, w, span, degree = degree, parametric = parametric, : at -0.005
```

```
## Warning in simpleLoess(y, x, w, span, degree = degree, parametric = parametric, : radius 2.5e-05
```

```
## Warning in simpleLoess(y, x, w, span, degree = degree, parametric = parametric, : all data on boundary of neighborhood. make span bigger
```

```
## Warning in simpleLoess(y, x, w, span, degree = degree, parametric = parametric, : pseudoinverse used at -0.005
```

```
## Warning in simpleLoess(y, x, w, span, degree = degree, parametric = parametric, : neighborhood radius 0.005
```

```
## Warning in simpleLoess(y, x, w, span, degree = degree, parametric = parametric, : reciprocal condition number 1
```

```
## Warning in simpleLoess(y, x, w, span, degree = degree, parametric = parametric, : There are other near singularities as well. 1.01
```

```
## Warning in simpleLoess(y, x, w, span, degree = degree, parametric = parametric, : zero-width neighborhood. make span bigger
```

```
## Warning: Computation failed in `stat_smooth()`:
## NA/NaN/Inf in foreign function call (arg 5)
```

```
## Warning in simpleLoess(y, x, w, span, degree = degree, parametric = parametric, : pseudoinverse used at -0.005
```

```
## Warning in simpleLoess(y, x, w, span, degree = degree, parametric = parametric, : neighborhood radius 1.005
```

```
## Warning in simpleLoess(y, x, w, span, degree = degree, parametric = parametric, : reciprocal condition number 0
```

```
## Warning in simpleLoess(y, x, w, span, degree = degree, parametric = parametric, : There are other near singularities as well. 1.01
```

```
## Warning in predLoess(object$y, object$x, newx = if (is.null(newdata)) object$x else if (is.data.frame(newdata)) as.matrix(model.frame(delete.response(terms(object)), : pseudoinverse used at -0.005
```

```
## Warning in predLoess(object$y, object$x, newx = if (is.null(newdata)) object$x else if (is.data.frame(newdata)) as.matrix(model.frame(delete.response(terms(object)), : neighborhood radius 1.005
```

```
## Warning in predLoess(object$y, object$x, newx = if (is.null(newdata)) object$x else if (is.data.frame(newdata)) as.matrix(model.frame(delete.response(terms(object)), : reciprocal condition number 0
```

```
## Warning in predLoess(object$y, object$x, newx = if (is.null(newdata)) object$x else if (is.data.frame(newdata)) as.matrix(model.frame(delete.response(terms(object)), : There are other near
## singularities as well. 1.01
```

```
## Warning in simpleLoess(y, x, w, span, degree = degree, parametric = parametric, : at -0.005
```

```
## Warning in simpleLoess(y, x, w, span, degree = degree, parametric = parametric, : radius 2.5e-05
```

```
## Warning in simpleLoess(y, x, w, span, degree = degree, parametric = parametric, : all data on boundary of neighborhood. make span bigger
```

```
## Warning in simpleLoess(y, x, w, span, degree = degree, parametric = parametric, : pseudoinverse used at -0.005
```

```
## Warning in simpleLoess(y, x, w, span, degree = degree, parametric = parametric, : neighborhood radius 0.005
```

```
## Warning in simpleLoess(y, x, w, span, degree = degree, parametric = parametric, : reciprocal condition number 1
```

```
## Warning in simpleLoess(y, x, w, span, degree = degree, parametric = parametric, : There are other near singularities as well. 1.01
```

```
## Warning in simpleLoess(y, x, w, span, degree = degree, parametric = parametric, : zero-width neighborhood. make span bigger
```

```
## Warning: Computation failed in `stat_smooth()`:
## NA/NaN/Inf in foreign function call (arg 5)
```

```
## Warning in simpleLoess(y, x, w, span, degree = degree, parametric = parametric, : at -0.005
```

```
## Warning in simpleLoess(y, x, w, span, degree = degree, parametric = parametric, : radius 2.5e-05
```

```
## Warning in simpleLoess(y, x, w, span, degree = degree, parametric = parametric, : all data on boundary of neighborhood. make span bigger
```

```
## Warning in simpleLoess(y, x, w, span, degree = degree, parametric = parametric, : pseudoinverse used at -0.005
```

```
## Warning in simpleLoess(y, x, w, span, degree = degree, parametric = parametric, : neighborhood radius 0.005
```

```
## Warning in simpleLoess(y, x, w, span, degree = degree, parametric = parametric, : reciprocal condition number 1
```

```
## Warning in simpleLoess(y, x, w, span, degree = degree, parametric = parametric, : There are other near singularities as well. 1.01
```

```
## Warning in simpleLoess(y, x, w, span, degree = degree, parametric = parametric, : zero-width neighborhood. make span bigger
```

```
## Warning: Computation failed in `stat_smooth()`:
## NA/NaN/Inf in foreign function call (arg 5)
```

```
## Warning in simpleLoess(y, x, w, span, degree = degree, parametric = parametric, : at -0.005
```

```
## Warning in simpleLoess(y, x, w, span, degree = degree, parametric = parametric, : radius 2.5e-05
```

```
## Warning in simpleLoess(y, x, w, span, degree = degree, parametric = parametric, : all data on boundary of neighborhood. make span bigger
```

```
## Warning in simpleLoess(y, x, w, span, degree = degree, parametric = parametric, : pseudoinverse used at -0.005
```

```
## Warning in simpleLoess(y, x, w, span, degree = degree, parametric = parametric, : neighborhood radius 0.005
```

```
## Warning in simpleLoess(y, x, w, span, degree = degree, parametric = parametric, : reciprocal condition number 1
```

```
## Warning in simpleLoess(y, x, w, span, degree = degree, parametric = parametric, : There are other near singularities as well. 1.01
```

```
## Warning in simpleLoess(y, x, w, span, degree = degree, parametric = parametric, : zero-width neighborhood. make span bigger
```

```
## Warning: Computation failed in `stat_smooth()`:
## NA/NaN/Inf in foreign function call (arg 5)
```

```
## Warning in simpleLoess(y, x, w, span, degree = degree, parametric = parametric, : pseudoinverse used at -0.005
```

```
## Warning in simpleLoess(y, x, w, span, degree = degree, parametric = parametric, : neighborhood radius 1.005
```

```
## Warning in simpleLoess(y, x, w, span, degree = degree, parametric = parametric, : reciprocal condition number 0
```

```
## Warning in simpleLoess(y, x, w, span, degree = degree, parametric = parametric, : There are other near singularities as well. 1.01
```

```
## Warning in predLoess(object$y, object$x, newx = if (is.null(newdata)) object$x else if (is.data.frame(newdata)) as.matrix(model.frame(delete.response(terms(object)), : pseudoinverse used at -0.005
```

```
## Warning in predLoess(object$y, object$x, newx = if (is.null(newdata)) object$x else if (is.data.frame(newdata)) as.matrix(model.frame(delete.response(terms(object)), : neighborhood radius 1.005
```

```
## Warning in predLoess(object$y, object$x, newx = if (is.null(newdata)) object$x else if (is.data.frame(newdata)) as.matrix(model.frame(delete.response(terms(object)), : reciprocal condition number 0
```

```
## Warning in predLoess(object$y, object$x, newx = if (is.null(newdata)) object$x else if (is.data.frame(newdata)) as.matrix(model.frame(delete.response(terms(object)), : There are other near
## singularities as well. 1.01
```

```
## Warning in simpleLoess(y, x, w, span, degree = degree, parametric = parametric, : at -0.005
```

```
## Warning in simpleLoess(y, x, w, span, degree = degree, parametric = parametric, : radius 2.5e-05
```

```
## Warning in simpleLoess(y, x, w, span, degree = degree, parametric = parametric, : all data on boundary of neighborhood. make span bigger
```

```
## Warning in simpleLoess(y, x, w, span, degree = degree, parametric = parametric, : pseudoinverse used at -0.005
```

```
## Warning in simpleLoess(y, x, w, span, degree = degree, parametric = parametric, : neighborhood radius 0.005
```

```
## Warning in simpleLoess(y, x, w, span, degree = degree, parametric = parametric, : reciprocal condition number 1
```

```
## Warning in simpleLoess(y, x, w, span, degree = degree, parametric = parametric, : There are other near singularities as well. 1.01
```

```
## Warning in simpleLoess(y, x, w, span, degree = degree, parametric = parametric, : zero-width neighborhood. make span bigger
```

```
## Warning: Computation failed in `stat_smooth()`:
## NA/NaN/Inf in foreign function call (arg 5)
```

```
## Warning in simpleLoess(y, x, w, span, degree = degree, parametric = parametric, : at -0.005
```

```
## Warning in simpleLoess(y, x, w, span, degree = degree, parametric = parametric, : radius 2.5e-05
```

```
## Warning in simpleLoess(y, x, w, span, degree = degree, parametric = parametric, : all data on boundary of neighborhood. make span bigger
```

```
## Warning in simpleLoess(y, x, w, span, degree = degree, parametric = parametric, : pseudoinverse used at -0.005
```

```
## Warning in simpleLoess(y, x, w, span, degree = degree, parametric = parametric, : neighborhood radius 0.005
```

```
## Warning in simpleLoess(y, x, w, span, degree = degree, parametric = parametric, : reciprocal condition number 1
```

```
## Warning in simpleLoess(y, x, w, span, degree = degree, parametric = parametric, : There are other near singularities as well. 1.01
```

```
## Warning in simpleLoess(y, x, w, span, degree = degree, parametric = parametric, : zero-width neighborhood. make span bigger
```

```
## Warning: Computation failed in `stat_smooth()`:
## NA/NaN/Inf in foreign function call (arg 5)
```

```
## Warning in simpleLoess(y, x, w, span, degree = degree, parametric = parametric, : at -0.005
```

```
## Warning in simpleLoess(y, x, w, span, degree = degree, parametric = parametric, : radius 2.5e-05
```

```
## Warning in simpleLoess(y, x, w, span, degree = degree, parametric = parametric, : all data on boundary of neighborhood. make span bigger
```

```
## Warning in simpleLoess(y, x, w, span, degree = degree, parametric = parametric, : pseudoinverse used at -0.005
```

```
## Warning in simpleLoess(y, x, w, span, degree = degree, parametric = parametric, : neighborhood radius 0.005
```

```
## Warning in simpleLoess(y, x, w, span, degree = degree, parametric = parametric, : reciprocal condition number 1
```

```
## Warning in simpleLoess(y, x, w, span, degree = degree, parametric = parametric, : There are other near singularities as well. 1.01
```

```
## Warning in simpleLoess(y, x, w, span, degree = degree, parametric = parametric, : zero-width neighborhood. make span bigger
```

```
## Warning: Computation failed in `stat_smooth()`:
## NA/NaN/Inf in foreign function call (arg 5)
```

```
## Warning in simpleLoess(y, x, w, span, degree = degree, parametric = parametric, : at -0.005
```

```
## Warning in simpleLoess(y, x, w, span, degree = degree, parametric = parametric, : radius 2.5e-05
```

```
## Warning in simpleLoess(y, x, w, span, degree = degree, parametric = parametric, : all data on boundary of neighborhood. make span bigger
```

```
## Warning in simpleLoess(y, x, w, span, degree = degree, parametric = parametric, : pseudoinverse used at -0.005
```

```
## Warning in simpleLoess(y, x, w, span, degree = degree, parametric = parametric, : neighborhood radius 0.005
```

```
## Warning in simpleLoess(y, x, w, span, degree = degree, parametric = parametric, : reciprocal condition number 1
```

```
## Warning in simpleLoess(y, x, w, span, degree = degree, parametric = parametric, : There are other near singularities as well. 1.01
```

```
## Warning in simpleLoess(y, x, w, span, degree = degree, parametric = parametric, : zero-width neighborhood. make span bigger
```

```
## Warning: Computation failed in `stat_smooth()`:
## NA/NaN/Inf in foreign function call (arg 5)
```

```
## Warning in simpleLoess(y, x, w, span, degree = degree, parametric = parametric, : at -0.005
```

```
## Warning in simpleLoess(y, x, w, span, degree = degree, parametric = parametric, : radius 2.5e-05
```

```
## Warning in simpleLoess(y, x, w, span, degree = degree, parametric = parametric, : all data on boundary of neighborhood. make span bigger
```

```
## Warning in simpleLoess(y, x, w, span, degree = degree, parametric = parametric, : pseudoinverse used at -0.005
```

```
## Warning in simpleLoess(y, x, w, span, degree = degree, parametric = parametric, : neighborhood radius 0.005
```

```
## Warning in simpleLoess(y, x, w, span, degree = degree, parametric = parametric, : reciprocal condition number 1
```

```
## Warning in simpleLoess(y, x, w, span, degree = degree, parametric = parametric, : There are other near singularities as well. 1.01
```

```
## Warning in simpleLoess(y, x, w, span, degree = degree, parametric = parametric, : zero-width neighborhood. make span bigger
```

```
## Warning: Computation failed in `stat_smooth()`:
## NA/NaN/Inf in foreign function call (arg 5)
```

```
## Warning in simpleLoess(y, x, w, span, degree = degree, parametric = parametric, : at -0.005
```

```
## Warning in simpleLoess(y, x, w, span, degree = degree, parametric = parametric, : radius 2.5e-05
```

```
## Warning in simpleLoess(y, x, w, span, degree = degree, parametric = parametric, : all data on boundary of neighborhood. make span bigger
```

```
## Warning in simpleLoess(y, x, w, span, degree = degree, parametric = parametric, : pseudoinverse used at -0.005
```

```
## Warning in simpleLoess(y, x, w, span, degree = degree, parametric = parametric, : neighborhood radius 0.005
```

```
## Warning in simpleLoess(y, x, w, span, degree = degree, parametric = parametric, : reciprocal condition number 1
```

```
## Warning in simpleLoess(y, x, w, span, degree = degree, parametric = parametric, : There are other near singularities as well. 1.01
```

```
## Warning in simpleLoess(y, x, w, span, degree = degree, parametric = parametric, : zero-width neighborhood. make span bigger
```

```
## Warning: Computation failed in `stat_smooth()`:
## NA/NaN/Inf in foreign function call (arg 5)
```

```
## Warning in simpleLoess(y, x, w, span, degree = degree, parametric = parametric, : pseudoinverse used at -0.005
```

```
## Warning in simpleLoess(y, x, w, span, degree = degree, parametric = parametric, : neighborhood radius 1.005
```

```
## Warning in simpleLoess(y, x, w, span, degree = degree, parametric = parametric, : reciprocal condition number 0
```

```
## Warning in simpleLoess(y, x, w, span, degree = degree, parametric = parametric, : There are other near singularities as well. 1.01
```

```
## Warning in predLoess(object$y, object$x, newx = if (is.null(newdata)) object$x else if (is.data.frame(newdata)) as.matrix(model.frame(delete.response(terms(object)), : pseudoinverse used at -0.005
```

```
## Warning in predLoess(object$y, object$x, newx = if (is.null(newdata)) object$x else if (is.data.frame(newdata)) as.matrix(model.frame(delete.response(terms(object)), : neighborhood radius 1.005
```

```
## Warning in predLoess(object$y, object$x, newx = if (is.null(newdata)) object$x else if (is.data.frame(newdata)) as.matrix(model.frame(delete.response(terms(object)), : reciprocal condition number 0
```

```
## Warning in predLoess(object$y, object$x, newx = if (is.null(newdata)) object$x else if (is.data.frame(newdata)) as.matrix(model.frame(delete.response(terms(object)), : There are other near
## singularities as well. 1.01
```

```
## Warning in simpleLoess(y, x, w, span, degree = degree, parametric = parametric, : at -0.005
```

```
## Warning in simpleLoess(y, x, w, span, degree = degree, parametric = parametric, : radius 2.5e-05
```

```
## Warning in simpleLoess(y, x, w, span, degree = degree, parametric = parametric, : all data on boundary of neighborhood. make span bigger
```

```
## Warning in simpleLoess(y, x, w, span, degree = degree, parametric = parametric, : pseudoinverse used at -0.005
```

```
## Warning in simpleLoess(y, x, w, span, degree = degree, parametric = parametric, : neighborhood radius 0.005
```

```
## Warning in simpleLoess(y, x, w, span, degree = degree, parametric = parametric, : reciprocal condition number 1
```

```
## Warning in simpleLoess(y, x, w, span, degree = degree, parametric = parametric, : There are other near singularities as well. 1.01
```

```
## Warning in simpleLoess(y, x, w, span, degree = degree, parametric = parametric, : zero-width neighborhood. make span bigger
```

```
## Warning: Computation failed in `stat_smooth()`:
## NA/NaN/Inf in foreign function call (arg 5)
```

```
## Warning in simpleLoess(y, x, w, span, degree = degree, parametric = parametric, : at -0.005
```

```
## Warning in simpleLoess(y, x, w, span, degree = degree, parametric = parametric, : radius 2.5e-05
```

```
## Warning in simpleLoess(y, x, w, span, degree = degree, parametric = parametric, : all data on boundary of neighborhood. make span bigger
```

```
## Warning in simpleLoess(y, x, w, span, degree = degree, parametric = parametric, : pseudoinverse used at -0.005
```

```
## Warning in simpleLoess(y, x, w, span, degree = degree, parametric = parametric, : neighborhood radius 0.005
```

```
## Warning in simpleLoess(y, x, w, span, degree = degree, parametric = parametric, : reciprocal condition number 1
```

```
## Warning in simpleLoess(y, x, w, span, degree = degree, parametric = parametric, : There are other near singularities as well. 1.01
```

```
## Warning in simpleLoess(y, x, w, span, degree = degree, parametric = parametric, : zero-width neighborhood. make span bigger
```

```
## Warning: Computation failed in `stat_smooth()`:
## NA/NaN/Inf in foreign function call (arg 5)
```

```
## Warning in simpleLoess(y, x, w, span, degree = degree, parametric = parametric, : at -0.005
```

```
## Warning in simpleLoess(y, x, w, span, degree = degree, parametric = parametric, : radius 2.5e-05
```

```
## Warning in simpleLoess(y, x, w, span, degree = degree, parametric = parametric, : all data on boundary of neighborhood. make span bigger
```

```
## Warning in simpleLoess(y, x, w, span, degree = degree, parametric = parametric, : pseudoinverse used at -0.005
```

```
## Warning in simpleLoess(y, x, w, span, degree = degree, parametric = parametric, : neighborhood radius 0.005
```

```
## Warning in simpleLoess(y, x, w, span, degree = degree, parametric = parametric, : reciprocal condition number 1
```

```
## Warning in simpleLoess(y, x, w, span, degree = degree, parametric = parametric, : There are other near singularities as well. 1.01
```

```
## Warning in simpleLoess(y, x, w, span, degree = degree, parametric = parametric, : zero-width neighborhood. make span bigger
```

```
## Warning: Computation failed in `stat_smooth()`:
## NA/NaN/Inf in foreign function call (arg 5)
```

```
## Warning in simpleLoess(y, x, w, span, degree = degree, parametric = parametric, : at -0.005
```

```
## Warning in simpleLoess(y, x, w, span, degree = degree, parametric = parametric, : radius 2.5e-05
```

```
## Warning in simpleLoess(y, x, w, span, degree = degree, parametric = parametric, : all data on boundary of neighborhood. make span bigger
```

```
## Warning in simpleLoess(y, x, w, span, degree = degree, parametric = parametric, : pseudoinverse used at -0.005
```

```
## Warning in simpleLoess(y, x, w, span, degree = degree, parametric = parametric, : neighborhood radius 0.005
```

```
## Warning in simpleLoess(y, x, w, span, degree = degree, parametric = parametric, : reciprocal condition number 1
```

```
## Warning in simpleLoess(y, x, w, span, degree = degree, parametric = parametric, : There are other near singularities as well. 1.01
```

```
## Warning in simpleLoess(y, x, w, span, degree = degree, parametric = parametric, : zero-width neighborhood. make span bigger
```

```
## Warning: Computation failed in `stat_smooth()`:
## NA/NaN/Inf in foreign function call (arg 5)
```

```
## Warning in simpleLoess(y, x, w, span, degree = degree, parametric = parametric, : at -0.005
```

```
## Warning in simpleLoess(y, x, w, span, degree = degree, parametric = parametric, : radius 2.5e-05
```

```
## Warning in simpleLoess(y, x, w, span, degree = degree, parametric = parametric, : all data on boundary of neighborhood. make span bigger
```

```
## Warning in simpleLoess(y, x, w, span, degree = degree, parametric = parametric, : pseudoinverse used at -0.005
```

```
## Warning in simpleLoess(y, x, w, span, degree = degree, parametric = parametric, : neighborhood radius 0.005
```

```
## Warning in simpleLoess(y, x, w, span, degree = degree, parametric = parametric, : reciprocal condition number 1
```

```
## Warning in simpleLoess(y, x, w, span, degree = degree, parametric = parametric, : There are other near singularities as well. 1.01
```

```
## Warning in simpleLoess(y, x, w, span, degree = degree, parametric = parametric, : zero-width neighborhood. make span bigger
```

```
## Warning: Computation failed in `stat_smooth()`:
## NA/NaN/Inf in foreign function call (arg 5)
```

```
## Warning in simpleLoess(y, x, w, span, degree = degree, parametric = parametric, : at -0.005
```

```
## Warning in simpleLoess(y, x, w, span, degree = degree, parametric = parametric, : radius 2.5e-05
```

```
## Warning in simpleLoess(y, x, w, span, degree = degree, parametric = parametric, : all data on boundary of neighborhood. make span bigger
```

```
## Warning in simpleLoess(y, x, w, span, degree = degree, parametric = parametric, : pseudoinverse used at -0.005
```

```
## Warning in simpleLoess(y, x, w, span, degree = degree, parametric = parametric, : neighborhood radius 0.005
```

```
## Warning in simpleLoess(y, x, w, span, degree = degree, parametric = parametric, : reciprocal condition number 1
```

```
## Warning in simpleLoess(y, x, w, span, degree = degree, parametric = parametric, : There are other near singularities as well. 1.01
```

```
## Warning in simpleLoess(y, x, w, span, degree = degree, parametric = parametric, : zero-width neighborhood. make span bigger
```

```
## Warning: Computation failed in `stat_smooth()`:
## NA/NaN/Inf in foreign function call (arg 5)
```

```
## Warning in simpleLoess(y, x, w, span, degree = degree, parametric = parametric, : at -0.005
```

```
## Warning in simpleLoess(y, x, w, span, degree = degree, parametric = parametric, : radius 2.5e-05
```

```
## Warning in simpleLoess(y, x, w, span, degree = degree, parametric = parametric, : all data on boundary of neighborhood. make span bigger
```

```
## Warning in simpleLoess(y, x, w, span, degree = degree, parametric = parametric, : pseudoinverse used at -0.005
```

```
## Warning in simpleLoess(y, x, w, span, degree = degree, parametric = parametric, : neighborhood radius 0.005
```

```
## Warning in simpleLoess(y, x, w, span, degree = degree, parametric = parametric, : reciprocal condition number 1
```

```
## Warning in simpleLoess(y, x, w, span, degree = degree, parametric = parametric, : There are other near singularities as well. 1.01
```

```
## Warning in simpleLoess(y, x, w, span, degree = degree, parametric = parametric, : zero-width neighborhood. make span bigger
```

```
## Warning: Computation failed in `stat_smooth()`:
## NA/NaN/Inf in foreign function call (arg 5)
```

```
## Warning in simpleLoess(y, x, w, span, degree = degree, parametric = parametric, : at -0.005
```

```
## Warning in simpleLoess(y, x, w, span, degree = degree, parametric = parametric, : radius 2.5e-05
```

```
## Warning in simpleLoess(y, x, w, span, degree = degree, parametric = parametric, : all data on boundary of neighborhood. make span bigger
```

```
## Warning in simpleLoess(y, x, w, span, degree = degree, parametric = parametric, : pseudoinverse used at -0.005
```

```
## Warning in simpleLoess(y, x, w, span, degree = degree, parametric = parametric, : neighborhood radius 0.005
```

```
## Warning in simpleLoess(y, x, w, span, degree = degree, parametric = parametric, : reciprocal condition number 1
```

```
## Warning in simpleLoess(y, x, w, span, degree = degree, parametric = parametric, : There are other near singularities as well. 1.01
```

```
## Warning in simpleLoess(y, x, w, span, degree = degree, parametric = parametric, : zero-width neighborhood. make span bigger
```

```
## Warning: Computation failed in `stat_smooth()`:
## NA/NaN/Inf in foreign function call (arg 5)
```

```
## Warning in simpleLoess(y, x, w, span, degree = degree, parametric = parametric, : at -0.005
```

```
## Warning in simpleLoess(y, x, w, span, degree = degree, parametric = parametric, : radius 2.5e-05
```

```
## Warning in simpleLoess(y, x, w, span, degree = degree, parametric = parametric, : all data on boundary of neighborhood. make span bigger
```

```
## Warning in simpleLoess(y, x, w, span, degree = degree, parametric = parametric, : pseudoinverse used at -0.005
```

```
## Warning in simpleLoess(y, x, w, span, degree = degree, parametric = parametric, : neighborhood radius 0.005
```

```
## Warning in simpleLoess(y, x, w, span, degree = degree, parametric = parametric, : reciprocal condition number 1
```

```
## Warning in simpleLoess(y, x, w, span, degree = degree, parametric = parametric, : There are other near singularities as well. 1.01
```

```
## Warning in simpleLoess(y, x, w, span, degree = degree, parametric = parametric, : zero-width neighborhood. make span bigger
```

```
## Warning: Computation failed in `stat_smooth()`:
## NA/NaN/Inf in foreign function call (arg 5)
```

```
## Warning in simpleLoess(y, x, w, span, degree = degree, parametric = parametric, : at -0.005
```

```
## Warning in simpleLoess(y, x, w, span, degree = degree, parametric = parametric, : radius 2.5e-05
```

```
## Warning in simpleLoess(y, x, w, span, degree = degree, parametric = parametric, : all data on boundary of neighborhood. make span bigger
```

```
## Warning in simpleLoess(y, x, w, span, degree = degree, parametric = parametric, : pseudoinverse used at -0.005
```

```
## Warning in simpleLoess(y, x, w, span, degree = degree, parametric = parametric, : neighborhood radius 0.005
```

```
## Warning in simpleLoess(y, x, w, span, degree = degree, parametric = parametric, : reciprocal condition number 1
```

```
## Warning in simpleLoess(y, x, w, span, degree = degree, parametric = parametric, : There are other near singularities as well. 1.01
```

```
## Warning in simpleLoess(y, x, w, span, degree = degree, parametric = parametric, : zero-width neighborhood. make span bigger
```

```
## Warning: Computation failed in `stat_smooth()`:
## NA/NaN/Inf in foreign function call (arg 5)
```

```
## Warning in simpleLoess(y, x, w, span, degree = degree, parametric = parametric, : at -0.005
```

```
## Warning in simpleLoess(y, x, w, span, degree = degree, parametric = parametric, : radius 2.5e-05
```

```
## Warning in simpleLoess(y, x, w, span, degree = degree, parametric = parametric, : all data on boundary of neighborhood. make span bigger
```

```
## Warning in simpleLoess(y, x, w, span, degree = degree, parametric = parametric, : pseudoinverse used at -0.005
```

```
## Warning in simpleLoess(y, x, w, span, degree = degree, parametric = parametric, : neighborhood radius 0.005
```

```
## Warning in simpleLoess(y, x, w, span, degree = degree, parametric = parametric, : reciprocal condition number 1
```

```
## Warning in simpleLoess(y, x, w, span, degree = degree, parametric = parametric, : There are other near singularities as well. 1.01
```

```
## Warning in simpleLoess(y, x, w, span, degree = degree, parametric = parametric, : zero-width neighborhood. make span bigger
```

```
## Warning: Computation failed in `stat_smooth()`:
## NA/NaN/Inf in foreign function call (arg 5)
```

```
## Warning in simpleLoess(y, x, w, span, degree = degree, parametric = parametric, : at -0.005
```

```
## Warning in simpleLoess(y, x, w, span, degree = degree, parametric = parametric, : radius 2.5e-05
```

```
## Warning in simpleLoess(y, x, w, span, degree = degree, parametric = parametric, : all data on boundary of neighborhood. make span bigger
```

```
## Warning in simpleLoess(y, x, w, span, degree = degree, parametric = parametric, : pseudoinverse used at -0.005
```

```
## Warning in simpleLoess(y, x, w, span, degree = degree, parametric = parametric, : neighborhood radius 0.005
```

```
## Warning in simpleLoess(y, x, w, span, degree = degree, parametric = parametric, : reciprocal condition number 1
```

```
## Warning in simpleLoess(y, x, w, span, degree = degree, parametric = parametric, : There are other near singularities as well. 1.01
```

```
## Warning in simpleLoess(y, x, w, span, degree = degree, parametric = parametric, : zero-width neighborhood. make span bigger
```

```
## Warning: Computation failed in `stat_smooth()`:
## NA/NaN/Inf in foreign function call (arg 5)
```

```
## Warning in simpleLoess(y, x, w, span, degree = degree, parametric = parametric, : at -0.005
```

```
## Warning in simpleLoess(y, x, w, span, degree = degree, parametric = parametric, : radius 2.5e-05
```

```
## Warning in simpleLoess(y, x, w, span, degree = degree, parametric = parametric, : all data on boundary of neighborhood. make span bigger
```

```
## Warning in simpleLoess(y, x, w, span, degree = degree, parametric = parametric, : pseudoinverse used at -0.005
```

```
## Warning in simpleLoess(y, x, w, span, degree = degree, parametric = parametric, : neighborhood radius 0.005
```

```
## Warning in simpleLoess(y, x, w, span, degree = degree, parametric = parametric, : reciprocal condition number 1
```

```
## Warning in simpleLoess(y, x, w, span, degree = degree, parametric = parametric, : There are other near singularities as well. 1.01
```

```
## Warning in simpleLoess(y, x, w, span, degree = degree, parametric = parametric, : zero-width neighborhood. make span bigger
```

```
## Warning: Computation failed in `stat_smooth()`:
## NA/NaN/Inf in foreign function call (arg 5)
```

```
## Warning in simpleLoess(y, x, w, span, degree = degree, parametric = parametric, : at -0.005
```

```
## Warning in simpleLoess(y, x, w, span, degree = degree, parametric = parametric, : radius 2.5e-05
```

```
## Warning in simpleLoess(y, x, w, span, degree = degree, parametric = parametric, : all data on boundary of neighborhood. make span bigger
```

```
## Warning in simpleLoess(y, x, w, span, degree = degree, parametric = parametric, : pseudoinverse used at -0.005
```

```
## Warning in simpleLoess(y, x, w, span, degree = degree, parametric = parametric, : neighborhood radius 0.005
```

```
## Warning in simpleLoess(y, x, w, span, degree = degree, parametric = parametric, : reciprocal condition number 1
```

```
## Warning in simpleLoess(y, x, w, span, degree = degree, parametric = parametric, : There are other near singularities as well. 1.01
```

```
## Warning in simpleLoess(y, x, w, span, degree = degree, parametric = parametric, : zero-width neighborhood. make span bigger
```

```
## Warning: Computation failed in `stat_smooth()`:
## NA/NaN/Inf in foreign function call (arg 5)
```

```
## Warning in simpleLoess(y, x, w, span, degree = degree, parametric = parametric, : pseudoinverse used at -0.005
```

```
## Warning in simpleLoess(y, x, w, span, degree = degree, parametric = parametric, : neighborhood radius 1.005
```

```
## Warning in simpleLoess(y, x, w, span, degree = degree, parametric = parametric, : reciprocal condition number 0
```

```
## Warning in simpleLoess(y, x, w, span, degree = degree, parametric = parametric, : There are other near singularities as well. 1.01
```

```
## Warning in predLoess(object$y, object$x, newx = if (is.null(newdata)) object$x else if (is.data.frame(newdata)) as.matrix(model.frame(delete.response(terms(object)), : pseudoinverse used at -0.005
```

```
## Warning in predLoess(object$y, object$x, newx = if (is.null(newdata)) object$x else if (is.data.frame(newdata)) as.matrix(model.frame(delete.response(terms(object)), : neighborhood radius 1.005
```

```
## Warning in predLoess(object$y, object$x, newx = if (is.null(newdata)) object$x else if (is.data.frame(newdata)) as.matrix(model.frame(delete.response(terms(object)), : reciprocal condition number 0
```

```
## Warning in predLoess(object$y, object$x, newx = if (is.null(newdata)) object$x else if (is.data.frame(newdata)) as.matrix(model.frame(delete.response(terms(object)), : There are other near
## singularities as well. 1.01
```

```
## Warning in simpleLoess(y, x, w, span, degree = degree, parametric = parametric, : at -0.005
```

```
## Warning in simpleLoess(y, x, w, span, degree = degree, parametric = parametric, : radius 2.5e-05
```

```
## Warning in simpleLoess(y, x, w, span, degree = degree, parametric = parametric, : all data on boundary of neighborhood. make span bigger
```

```
## Warning in simpleLoess(y, x, w, span, degree = degree, parametric = parametric, : pseudoinverse used at -0.005
```

```
## Warning in simpleLoess(y, x, w, span, degree = degree, parametric = parametric, : neighborhood radius 0.005
```

```
## Warning in simpleLoess(y, x, w, span, degree = degree, parametric = parametric, : reciprocal condition number 1
```

```
## Warning in simpleLoess(y, x, w, span, degree = degree, parametric = parametric, : There are other near singularities as well. 1.01
```

```
## Warning in simpleLoess(y, x, w, span, degree = degree, parametric = parametric, : zero-width neighborhood. make span bigger
```

```
## Warning: Computation failed in `stat_smooth()`:
## NA/NaN/Inf in foreign function call (arg 5)
```

```
## Warning in simpleLoess(y, x, w, span, degree = degree, parametric = parametric, : at -0.005
```

```
## Warning in simpleLoess(y, x, w, span, degree = degree, parametric = parametric, : radius 2.5e-05
```

```
## Warning in simpleLoess(y, x, w, span, degree = degree, parametric = parametric, : all data on boundary of neighborhood. make span bigger
```

```
## Warning in simpleLoess(y, x, w, span, degree = degree, parametric = parametric, : pseudoinverse used at -0.005
```

```
## Warning in simpleLoess(y, x, w, span, degree = degree, parametric = parametric, : neighborhood radius 0.005
```

```
## Warning in simpleLoess(y, x, w, span, degree = degree, parametric = parametric, : reciprocal condition number 1
```

```
## Warning in simpleLoess(y, x, w, span, degree = degree, parametric = parametric, : There are other near singularities as well. 1.01
```

```
## Warning in simpleLoess(y, x, w, span, degree = degree, parametric = parametric, : zero-width neighborhood. make span bigger
```

```
## Warning: Computation failed in `stat_smooth()`:
## NA/NaN/Inf in foreign function call (arg 5)
```

```
## Warning in simpleLoess(y, x, w, span, degree = degree, parametric = parametric, : pseudoinverse used at -0.005
```

```
## Warning in simpleLoess(y, x, w, span, degree = degree, parametric = parametric, : neighborhood radius 1.005
```

```
## Warning in simpleLoess(y, x, w, span, degree = degree, parametric = parametric, : reciprocal condition number 0
```

```
## Warning in simpleLoess(y, x, w, span, degree = degree, parametric = parametric, : There are other near singularities as well. 1.01
```

```
## Warning in predLoess(object$y, object$x, newx = if (is.null(newdata)) object$x else if (is.data.frame(newdata)) as.matrix(model.frame(delete.response(terms(object)), : pseudoinverse used at -0.005
```

```
## Warning in predLoess(object$y, object$x, newx = if (is.null(newdata)) object$x else if (is.data.frame(newdata)) as.matrix(model.frame(delete.response(terms(object)), : neighborhood radius 1.005
```

```
## Warning in predLoess(object$y, object$x, newx = if (is.null(newdata)) object$x else if (is.data.frame(newdata)) as.matrix(model.frame(delete.response(terms(object)), : reciprocal condition number 0
```

```
## Warning in predLoess(object$y, object$x, newx = if (is.null(newdata)) object$x else if (is.data.frame(newdata)) as.matrix(model.frame(delete.response(terms(object)), : There are other near
## singularities as well. 1.01
```

```
## Warning in simpleLoess(y, x, w, span, degree = degree, parametric = parametric, : pseudoinverse used at -0.005
```

```
## Warning in simpleLoess(y, x, w, span, degree = degree, parametric = parametric, : neighborhood radius 1.005
```

```
## Warning in simpleLoess(y, x, w, span, degree = degree, parametric = parametric, : reciprocal condition number 0
```

```
## Warning in simpleLoess(y, x, w, span, degree = degree, parametric = parametric, : There are other near singularities as well. 1.01
```

```
## Warning in predLoess(object$y, object$x, newx = if (is.null(newdata)) object$x else if (is.data.frame(newdata)) as.matrix(model.frame(delete.response(terms(object)), : pseudoinverse used at -0.005
```

```
## Warning in predLoess(object$y, object$x, newx = if (is.null(newdata)) object$x else if (is.data.frame(newdata)) as.matrix(model.frame(delete.response(terms(object)), : neighborhood radius 1.005
```

```
## Warning in predLoess(object$y, object$x, newx = if (is.null(newdata)) object$x else if (is.data.frame(newdata)) as.matrix(model.frame(delete.response(terms(object)), : reciprocal condition number 0
```

```
## Warning in predLoess(object$y, object$x, newx = if (is.null(newdata)) object$x else if (is.data.frame(newdata)) as.matrix(model.frame(delete.response(terms(object)), : There are other near
## singularities as well. 1.01
```

```
## Warning in simpleLoess(y, x, w, span, degree = degree, parametric = parametric, : at -0.005
```

```
## Warning in simpleLoess(y, x, w, span, degree = degree, parametric = parametric, : radius 2.5e-05
```

```
## Warning in simpleLoess(y, x, w, span, degree = degree, parametric = parametric, : all data on boundary of neighborhood. make span bigger
```

```
## Warning in simpleLoess(y, x, w, span, degree = degree, parametric = parametric, : pseudoinverse used at -0.005
```

```
## Warning in simpleLoess(y, x, w, span, degree = degree, parametric = parametric, : neighborhood radius 0.005
```

```
## Warning in simpleLoess(y, x, w, span, degree = degree, parametric = parametric, : reciprocal condition number 1
```

```
## Warning in simpleLoess(y, x, w, span, degree = degree, parametric = parametric, : There are other near singularities as well. 1.01
```

```
## Warning in simpleLoess(y, x, w, span, degree = degree, parametric = parametric, : zero-width neighborhood. make span bigger
```

```
## Warning: Computation failed in `stat_smooth()`:
## NA/NaN/Inf in foreign function call (arg 5)
```

```
## Warning in simpleLoess(y, x, w, span, degree = degree, parametric = parametric, : at -0.005
```

```
## Warning in simpleLoess(y, x, w, span, degree = degree, parametric = parametric, : radius 2.5e-05
```

```
## Warning in simpleLoess(y, x, w, span, degree = degree, parametric = parametric, : all data on boundary of neighborhood. make span bigger
```

```
## Warning in simpleLoess(y, x, w, span, degree = degree, parametric = parametric, : pseudoinverse used at -0.005
```

```
## Warning in simpleLoess(y, x, w, span, degree = degree, parametric = parametric, : neighborhood radius 0.005
```

```
## Warning in simpleLoess(y, x, w, span, degree = degree, parametric = parametric, : reciprocal condition number 1
```

```
## Warning in simpleLoess(y, x, w, span, degree = degree, parametric = parametric, : There are other near singularities as well. 1.01
```

```
## Warning in simpleLoess(y, x, w, span, degree = degree, parametric = parametric, : zero-width neighborhood. make span bigger
```

```
## Warning: Computation failed in `stat_smooth()`:
## NA/NaN/Inf in foreign function call (arg 5)
```

```
## Warning in simpleLoess(y, x, w, span, degree = degree, parametric = parametric, : at -0.005
```

```
## Warning in simpleLoess(y, x, w, span, degree = degree, parametric = parametric, : radius 2.5e-05
```

```
## Warning in simpleLoess(y, x, w, span, degree = degree, parametric = parametric, : all data on boundary of neighborhood. make span bigger
```

```
## Warning in simpleLoess(y, x, w, span, degree = degree, parametric = parametric, : pseudoinverse used at -0.005
```

```
## Warning in simpleLoess(y, x, w, span, degree = degree, parametric = parametric, : neighborhood radius 0.005
```

```
## Warning in simpleLoess(y, x, w, span, degree = degree, parametric = parametric, : reciprocal condition number 1
```

```
## Warning in simpleLoess(y, x, w, span, degree = degree, parametric = parametric, : There are other near singularities as well. 1.01
```

```
## Warning in simpleLoess(y, x, w, span, degree = degree, parametric = parametric, : zero-width neighborhood. make span bigger
```

```
## Warning: Computation failed in `stat_smooth()`:
## NA/NaN/Inf in foreign function call (arg 5)
```

```
## Warning in simpleLoess(y, x, w, span, degree = degree, parametric = parametric, : at -0.005
```

```
## Warning in simpleLoess(y, x, w, span, degree = degree, parametric = parametric, : radius 2.5e-05
```

```
## Warning in simpleLoess(y, x, w, span, degree = degree, parametric = parametric, : all data on boundary of neighborhood. make span bigger
```

```
## Warning in simpleLoess(y, x, w, span, degree = degree, parametric = parametric, : pseudoinverse used at -0.005
```

```
## Warning in simpleLoess(y, x, w, span, degree = degree, parametric = parametric, : neighborhood radius 0.005
```

```
## Warning in simpleLoess(y, x, w, span, degree = degree, parametric = parametric, : reciprocal condition number 1
```

```
## Warning in simpleLoess(y, x, w, span, degree = degree, parametric = parametric, : There are other near singularities as well. 1.01
```

```
## Warning in simpleLoess(y, x, w, span, degree = degree, parametric = parametric, : zero-width neighborhood. make span bigger
```

```
## Warning: Computation failed in `stat_smooth()`:
## NA/NaN/Inf in foreign function call (arg 5)
```

```
## Warning in simpleLoess(y, x, w, span, degree = degree, parametric = parametric, : pseudoinverse used at -0.005
```

```
## Warning in simpleLoess(y, x, w, span, degree = degree, parametric = parametric, : neighborhood radius 1.005
```

```
## Warning in simpleLoess(y, x, w, span, degree = degree, parametric = parametric, : reciprocal condition number 0
```

```
## Warning in simpleLoess(y, x, w, span, degree = degree, parametric = parametric, : There are other near singularities as well. 1.01
```

```
## Warning in predLoess(object$y, object$x, newx = if (is.null(newdata)) object$x else if (is.data.frame(newdata)) as.matrix(model.frame(delete.response(terms(object)), : pseudoinverse used at -0.005
```

```
## Warning in predLoess(object$y, object$x, newx = if (is.null(newdata)) object$x else if (is.data.frame(newdata)) as.matrix(model.frame(delete.response(terms(object)), : neighborhood radius 1.005
```

```
## Warning in predLoess(object$y, object$x, newx = if (is.null(newdata)) object$x else if (is.data.frame(newdata)) as.matrix(model.frame(delete.response(terms(object)), : reciprocal condition number 0
```

```
## Warning in predLoess(object$y, object$x, newx = if (is.null(newdata)) object$x else if (is.data.frame(newdata)) as.matrix(model.frame(delete.response(terms(object)), : There are other near
## singularities as well. 1.01
```

```
## Warning in simpleLoess(y, x, w, span, degree = degree, parametric = parametric, : at -0.005
```

```
## Warning in simpleLoess(y, x, w, span, degree = degree, parametric = parametric, : radius 2.5e-05
```

```
## Warning in simpleLoess(y, x, w, span, degree = degree, parametric = parametric, : all data on boundary of neighborhood. make span bigger
```

```
## Warning in simpleLoess(y, x, w, span, degree = degree, parametric = parametric, : pseudoinverse used at -0.005
```

```
## Warning in simpleLoess(y, x, w, span, degree = degree, parametric = parametric, : neighborhood radius 0.005
```

```
## Warning in simpleLoess(y, x, w, span, degree = degree, parametric = parametric, : reciprocal condition number 1
```

```
## Warning in simpleLoess(y, x, w, span, degree = degree, parametric = parametric, : There are other near singularities as well. 1.01
```

```
## Warning in simpleLoess(y, x, w, span, degree = degree, parametric = parametric, : zero-width neighborhood. make span bigger
```

```
## Warning: Computation failed in `stat_smooth()`:
## NA/NaN/Inf in foreign function call (arg 5)
```

```
## Warning in simpleLoess(y, x, w, span, degree = degree, parametric = parametric, : at -0.005
```

```
## Warning in simpleLoess(y, x, w, span, degree = degree, parametric = parametric, : radius 2.5e-05
```

```
## Warning in simpleLoess(y, x, w, span, degree = degree, parametric = parametric, : all data on boundary of neighborhood. make span bigger
```

```
## Warning in simpleLoess(y, x, w, span, degree = degree, parametric = parametric, : pseudoinverse used at -0.005
```

```
## Warning in simpleLoess(y, x, w, span, degree = degree, parametric = parametric, : neighborhood radius 0.005
```

```
## Warning in simpleLoess(y, x, w, span, degree = degree, parametric = parametric, : reciprocal condition number 1
```

```
## Warning in simpleLoess(y, x, w, span, degree = degree, parametric = parametric, : There are other near singularities as well. 1.01
```

```
## Warning in simpleLoess(y, x, w, span, degree = degree, parametric = parametric, : zero-width neighborhood. make span bigger
```

```
## Warning: Computation failed in `stat_smooth()`:
## NA/NaN/Inf in foreign function call (arg 5)
```

```
## Warning in simpleLoess(y, x, w, span, degree = degree, parametric = parametric, : at -0.005
```

```
## Warning in simpleLoess(y, x, w, span, degree = degree, parametric = parametric, : radius 2.5e-05
```

```
## Warning in simpleLoess(y, x, w, span, degree = degree, parametric = parametric, : all data on boundary of neighborhood. make span bigger
```

```
## Warning in simpleLoess(y, x, w, span, degree = degree, parametric = parametric, : pseudoinverse used at -0.005
```

```
## Warning in simpleLoess(y, x, w, span, degree = degree, parametric = parametric, : neighborhood radius 0.005
```

```
## Warning in simpleLoess(y, x, w, span, degree = degree, parametric = parametric, : reciprocal condition number 1
```

```
## Warning in simpleLoess(y, x, w, span, degree = degree, parametric = parametric, : There are other near singularities as well. 1.01
```

```
## Warning in simpleLoess(y, x, w, span, degree = degree, parametric = parametric, : zero-width neighborhood. make span bigger
```

```
## Warning: Computation failed in `stat_smooth()`:
## NA/NaN/Inf in foreign function call (arg 5)
```

```
## Warning in simpleLoess(y, x, w, span, degree = degree, parametric = parametric, : at -0.005
```

```
## Warning in simpleLoess(y, x, w, span, degree = degree, parametric = parametric, : radius 2.5e-05
```

```
## Warning in simpleLoess(y, x, w, span, degree = degree, parametric = parametric, : all data on boundary of neighborhood. make span bigger
```

```
## Warning in simpleLoess(y, x, w, span, degree = degree, parametric = parametric, : pseudoinverse used at -0.005
```

```
## Warning in simpleLoess(y, x, w, span, degree = degree, parametric = parametric, : neighborhood radius 0.005
```

```
## Warning in simpleLoess(y, x, w, span, degree = degree, parametric = parametric, : reciprocal condition number 1
```

```
## Warning in simpleLoess(y, x, w, span, degree = degree, parametric = parametric, : There are other near singularities as well. 1.01
```

```
## Warning in simpleLoess(y, x, w, span, degree = degree, parametric = parametric, : zero-width neighborhood. make span bigger
```

```
## Warning: Computation failed in `stat_smooth()`:
## NA/NaN/Inf in foreign function call (arg 5)
```

```
## Warning in simpleLoess(y, x, w, span, degree = degree, parametric = parametric, : at -0.005
```

```
## Warning in simpleLoess(y, x, w, span, degree = degree, parametric = parametric, : radius 2.5e-05
```

```
## Warning in simpleLoess(y, x, w, span, degree = degree, parametric = parametric, : all data on boundary of neighborhood. make span bigger
```

```
## Warning in simpleLoess(y, x, w, span, degree = degree, parametric = parametric, : pseudoinverse used at -0.005
```

```
## Warning in simpleLoess(y, x, w, span, degree = degree, parametric = parametric, : neighborhood radius 0.005
```

```
## Warning in simpleLoess(y, x, w, span, degree = degree, parametric = parametric, : reciprocal condition number 1
```

```
## Warning in simpleLoess(y, x, w, span, degree = degree, parametric = parametric, : There are other near singularities as well. 1.01
```

```
## Warning in simpleLoess(y, x, w, span, degree = degree, parametric = parametric, : zero-width neighborhood. make span bigger
```

```
## Warning: Computation failed in `stat_smooth()`:
## NA/NaN/Inf in foreign function call (arg 5)
```

```
## Warning in simpleLoess(y, x, w, span, degree = degree, parametric = parametric, : at -0.005
```

```
## Warning in simpleLoess(y, x, w, span, degree = degree, parametric = parametric, : radius 2.5e-05
```

```
## Warning in simpleLoess(y, x, w, span, degree = degree, parametric = parametric, : all data on boundary of neighborhood. make span bigger
```

```
## Warning in simpleLoess(y, x, w, span, degree = degree, parametric = parametric, : pseudoinverse used at -0.005
```

```
## Warning in simpleLoess(y, x, w, span, degree = degree, parametric = parametric, : neighborhood radius 0.005
```

```
## Warning in simpleLoess(y, x, w, span, degree = degree, parametric = parametric, : reciprocal condition number 1
```

```
## Warning in simpleLoess(y, x, w, span, degree = degree, parametric = parametric, : There are other near singularities as well. 1.01
```

```
## Warning in simpleLoess(y, x, w, span, degree = degree, parametric = parametric, : zero-width neighborhood. make span bigger
```

```
## Warning: Computation failed in `stat_smooth()`:
## NA/NaN/Inf in foreign function call (arg 5)
```

```
## Warning in simpleLoess(y, x, w, span, degree = degree, parametric = parametric, : at -0.005
```

```
## Warning in simpleLoess(y, x, w, span, degree = degree, parametric = parametric, : radius 2.5e-05
```

```
## Warning in simpleLoess(y, x, w, span, degree = degree, parametric = parametric, : all data on boundary of neighborhood. make span bigger
```

```
## Warning in simpleLoess(y, x, w, span, degree = degree, parametric = parametric, : pseudoinverse used at -0.005
```

```
## Warning in simpleLoess(y, x, w, span, degree = degree, parametric = parametric, : neighborhood radius 0.005
```

```
## Warning in simpleLoess(y, x, w, span, degree = degree, parametric = parametric, : reciprocal condition number 1
```

```
## Warning in simpleLoess(y, x, w, span, degree = degree, parametric = parametric, : There are other near singularities as well. 1.01
```

```
## Warning in simpleLoess(y, x, w, span, degree = degree, parametric = parametric, : zero-width neighborhood. make span bigger
```

```
## Warning: Computation failed in `stat_smooth()`:
## NA/NaN/Inf in foreign function call (arg 5)
```

```
## Warning in simpleLoess(y, x, w, span, degree = degree, parametric = parametric, : at -0.005
```

```
## Warning in simpleLoess(y, x, w, span, degree = degree, parametric = parametric, : radius 2.5e-05
```

```
## Warning in simpleLoess(y, x, w, span, degree = degree, parametric = parametric, : all data on boundary of neighborhood. make span bigger
```

```
## Warning in simpleLoess(y, x, w, span, degree = degree, parametric = parametric, : pseudoinverse used at -0.005
```

```
## Warning in simpleLoess(y, x, w, span, degree = degree, parametric = parametric, : neighborhood radius 0.005
```

```
## Warning in simpleLoess(y, x, w, span, degree = degree, parametric = parametric, : reciprocal condition number 1
```

```
## Warning in simpleLoess(y, x, w, span, degree = degree, parametric = parametric, : There are other near singularities as well. 1.01
```

```
## Warning in simpleLoess(y, x, w, span, degree = degree, parametric = parametric, : zero-width neighborhood. make span bigger
```

```
## Warning: Computation failed in `stat_smooth()`:
## NA/NaN/Inf in foreign function call (arg 5)
```

```
## Warning in simpleLoess(y, x, w, span, degree = degree, parametric = parametric, : at -0.005
```

```
## Warning in simpleLoess(y, x, w, span, degree = degree, parametric = parametric, : radius 2.5e-05
```

```
## Warning in simpleLoess(y, x, w, span, degree = degree, parametric = parametric, : all data on boundary of neighborhood. make span bigger
```

```
## Warning in simpleLoess(y, x, w, span, degree = degree, parametric = parametric, : pseudoinverse used at -0.005
```

```
## Warning in simpleLoess(y, x, w, span, degree = degree, parametric = parametric, : neighborhood radius 0.005
```

```
## Warning in simpleLoess(y, x, w, span, degree = degree, parametric = parametric, : reciprocal condition number 1
```

```
## Warning in simpleLoess(y, x, w, span, degree = degree, parametric = parametric, : There are other near singularities as well. 1.01
```

```
## Warning in simpleLoess(y, x, w, span, degree = degree, parametric = parametric, : zero-width neighborhood. make span bigger
```

```
## Warning: Computation failed in `stat_smooth()`:
## NA/NaN/Inf in foreign function call (arg 5)
```

```
## Warning in simpleLoess(y, x, w, span, degree = degree, parametric = parametric, : at -0.005
```

```
## Warning in simpleLoess(y, x, w, span, degree = degree, parametric = parametric, : radius 2.5e-05
```

```
## Warning in simpleLoess(y, x, w, span, degree = degree, parametric = parametric, : all data on boundary of neighborhood. make span bigger
```

```
## Warning in simpleLoess(y, x, w, span, degree = degree, parametric = parametric, : pseudoinverse used at -0.005
```

```
## Warning in simpleLoess(y, x, w, span, degree = degree, parametric = parametric, : neighborhood radius 0.005
```

```
## Warning in simpleLoess(y, x, w, span, degree = degree, parametric = parametric, : reciprocal condition number 1
```

```
## Warning in simpleLoess(y, x, w, span, degree = degree, parametric = parametric, : There are other near singularities as well. 1.01
```

```
## Warning in simpleLoess(y, x, w, span, degree = degree, parametric = parametric, : zero-width neighborhood. make span bigger
```

```
## Warning: Computation failed in `stat_smooth()`:
## NA/NaN/Inf in foreign function call (arg 5)
```

```
## Warning in simpleLoess(y, x, w, span, degree = degree, parametric = parametric, : at -0.005
```

```
## Warning in simpleLoess(y, x, w, span, degree = degree, parametric = parametric, : radius 2.5e-05
```

```
## Warning in simpleLoess(y, x, w, span, degree = degree, parametric = parametric, : all data on boundary of neighborhood. make span bigger
```

```
## Warning in simpleLoess(y, x, w, span, degree = degree, parametric = parametric, : pseudoinverse used at -0.005
```

```
## Warning in simpleLoess(y, x, w, span, degree = degree, parametric = parametric, : neighborhood radius 0.005
```

```
## Warning in simpleLoess(y, x, w, span, degree = degree, parametric = parametric, : reciprocal condition number 1
```

```
## Warning in simpleLoess(y, x, w, span, degree = degree, parametric = parametric, : There are other near singularities as well. 1.01
```

```
## Warning in simpleLoess(y, x, w, span, degree = degree, parametric = parametric, : zero-width neighborhood. make span bigger
```

```
## Warning: Computation failed in `stat_smooth()`:
## NA/NaN/Inf in foreign function call (arg 5)
```

```
## Warning in simpleLoess(y, x, w, span, degree = degree, parametric = parametric, : at -0.005
```

```
## Warning in simpleLoess(y, x, w, span, degree = degree, parametric = parametric, : radius 2.5e-05
```

```
## Warning in simpleLoess(y, x, w, span, degree = degree, parametric = parametric, : all data on boundary of neighborhood. make span bigger
```

```
## Warning in simpleLoess(y, x, w, span, degree = degree, parametric = parametric, : pseudoinverse used at -0.005
```

```
## Warning in simpleLoess(y, x, w, span, degree = degree, parametric = parametric, : neighborhood radius 0.005
```

```
## Warning in simpleLoess(y, x, w, span, degree = degree, parametric = parametric, : reciprocal condition number 1
```

```
## Warning in simpleLoess(y, x, w, span, degree = degree, parametric = parametric, : There are other near singularities as well. 1.01
```

```
## Warning in simpleLoess(y, x, w, span, degree = degree, parametric = parametric, : zero-width neighborhood. make span bigger
```

```
## Warning: Computation failed in `stat_smooth()`:
## NA/NaN/Inf in foreign function call (arg 5)
```

```
## Warning in simpleLoess(y, x, w, span, degree = degree, parametric = parametric, : at -0.005
```

```
## Warning in simpleLoess(y, x, w, span, degree = degree, parametric = parametric, : radius 2.5e-05
```

```
## Warning in simpleLoess(y, x, w, span, degree = degree, parametric = parametric, : all data on boundary of neighborhood. make span bigger
```

```
## Warning in simpleLoess(y, x, w, span, degree = degree, parametric = parametric, : pseudoinverse used at -0.005
```

```
## Warning in simpleLoess(y, x, w, span, degree = degree, parametric = parametric, : neighborhood radius 0.005
```

```
## Warning in simpleLoess(y, x, w, span, degree = degree, parametric = parametric, : reciprocal condition number 1
```

```
## Warning in simpleLoess(y, x, w, span, degree = degree, parametric = parametric, : There are other near singularities as well. 1.01
```

```
## Warning in simpleLoess(y, x, w, span, degree = degree, parametric = parametric, : zero-width neighborhood. make span bigger
```

```
## Warning: Computation failed in `stat_smooth()`:
## NA/NaN/Inf in foreign function call (arg 5)
```

```
## Warning in simpleLoess(y, x, w, span, degree = degree, parametric = parametric, : at -0.005
```

```
## Warning in simpleLoess(y, x, w, span, degree = degree, parametric = parametric, : radius 2.5e-05
```

```
## Warning in simpleLoess(y, x, w, span, degree = degree, parametric = parametric, : all data on boundary of neighborhood. make span bigger
```

```
## Warning in simpleLoess(y, x, w, span, degree = degree, parametric = parametric, : pseudoinverse used at -0.005
```

```
## Warning in simpleLoess(y, x, w, span, degree = degree, parametric = parametric, : neighborhood radius 0.005
```

```
## Warning in simpleLoess(y, x, w, span, degree = degree, parametric = parametric, : reciprocal condition number 1
```

```
## Warning in simpleLoess(y, x, w, span, degree = degree, parametric = parametric, : There are other near singularities as well. 1.01
```

```
## Warning in simpleLoess(y, x, w, span, degree = degree, parametric = parametric, : zero-width neighborhood. make span bigger
```

```
## Warning: Computation failed in `stat_smooth()`:
## NA/NaN/Inf in foreign function call (arg 5)
```

```
## Warning in simpleLoess(y, x, w, span, degree = degree, parametric = parametric, : at -0.005
```

```
## Warning in simpleLoess(y, x, w, span, degree = degree, parametric = parametric, : radius 2.5e-05
```

```
## Warning in simpleLoess(y, x, w, span, degree = degree, parametric = parametric, : all data on boundary of neighborhood. make span bigger
```

```
## Warning in simpleLoess(y, x, w, span, degree = degree, parametric = parametric, : pseudoinverse used at -0.005
```

```
## Warning in simpleLoess(y, x, w, span, degree = degree, parametric = parametric, : neighborhood radius 0.005
```

```
## Warning in simpleLoess(y, x, w, span, degree = degree, parametric = parametric, : reciprocal condition number 1
```

```
## Warning in simpleLoess(y, x, w, span, degree = degree, parametric = parametric, : There are other near singularities as well. 1.01
```

```
## Warning in simpleLoess(y, x, w, span, degree = degree, parametric = parametric, : zero-width neighborhood. make span bigger
```

```
## Warning: Computation failed in `stat_smooth()`:
## NA/NaN/Inf in foreign function call (arg 5)
```

```
## Warning in simpleLoess(y, x, w, span, degree = degree, parametric = parametric, : at -0.005
```

```
## Warning in simpleLoess(y, x, w, span, degree = degree, parametric = parametric, : radius 2.5e-05
```

```
## Warning in simpleLoess(y, x, w, span, degree = degree, parametric = parametric, : all data on boundary of neighborhood. make span bigger
```

```
## Warning in simpleLoess(y, x, w, span, degree = degree, parametric = parametric, : pseudoinverse used at -0.005
```

```
## Warning in simpleLoess(y, x, w, span, degree = degree, parametric = parametric, : neighborhood radius 0.005
```

```
## Warning in simpleLoess(y, x, w, span, degree = degree, parametric = parametric, : reciprocal condition number 1
```

```
## Warning in simpleLoess(y, x, w, span, degree = degree, parametric = parametric, : There are other near singularities as well. 1.01
```

```
## Warning in simpleLoess(y, x, w, span, degree = degree, parametric = parametric, : zero-width neighborhood. make span bigger
```

```
## Warning: Computation failed in `stat_smooth()`:
## NA/NaN/Inf in foreign function call (arg 5)
```

```
## Warning in simpleLoess(y, x, w, span, degree = degree, parametric = parametric, : at -0.005
```

```
## Warning in simpleLoess(y, x, w, span, degree = degree, parametric = parametric, : radius 2.5e-05
```

```
## Warning in simpleLoess(y, x, w, span, degree = degree, parametric = parametric, : all data on boundary of neighborhood. make span bigger
```

```
## Warning in simpleLoess(y, x, w, span, degree = degree, parametric = parametric, : pseudoinverse used at -0.005
```

```
## Warning in simpleLoess(y, x, w, span, degree = degree, parametric = parametric, : neighborhood radius 0.005
```

```
## Warning in simpleLoess(y, x, w, span, degree = degree, parametric = parametric, : reciprocal condition number 1
```

```
## Warning in simpleLoess(y, x, w, span, degree = degree, parametric = parametric, : There are other near singularities as well. 1.01
```

```
## Warning in simpleLoess(y, x, w, span, degree = degree, parametric = parametric, : zero-width neighborhood. make span bigger
```

```
## Warning: Computation failed in `stat_smooth()`:
## NA/NaN/Inf in foreign function call (arg 5)
```

```
## Warning in simpleLoess(y, x, w, span, degree = degree, parametric = parametric, : at -0.005
```

```
## Warning in simpleLoess(y, x, w, span, degree = degree, parametric = parametric, : radius 2.5e-05
```

```
## Warning in simpleLoess(y, x, w, span, degree = degree, parametric = parametric, : all data on boundary of neighborhood. make span bigger
```

```
## Warning in simpleLoess(y, x, w, span, degree = degree, parametric = parametric, : pseudoinverse used at -0.005
```

```
## Warning in simpleLoess(y, x, w, span, degree = degree, parametric = parametric, : neighborhood radius 0.005
```

```
## Warning in simpleLoess(y, x, w, span, degree = degree, parametric = parametric, : reciprocal condition number 1
```

```
## Warning in simpleLoess(y, x, w, span, degree = degree, parametric = parametric, : There are other near singularities as well. 1.01
```

```
## Warning in simpleLoess(y, x, w, span, degree = degree, parametric = parametric, : zero-width neighborhood. make span bigger
```

```
## Warning: Computation failed in `stat_smooth()`:
## NA/NaN/Inf in foreign function call (arg 5)
```

```
## Warning in simpleLoess(y, x, w, span, degree = degree, parametric = parametric, : at -0.005
```

```
## Warning in simpleLoess(y, x, w, span, degree = degree, parametric = parametric, : radius 2.5e-05
```

```
## Warning in simpleLoess(y, x, w, span, degree = degree, parametric = parametric, : all data on boundary of neighborhood. make span bigger
```

```
## Warning in simpleLoess(y, x, w, span, degree = degree, parametric = parametric, : pseudoinverse used at -0.005
```

```
## Warning in simpleLoess(y, x, w, span, degree = degree, parametric = parametric, : neighborhood radius 0.005
```

```
## Warning in simpleLoess(y, x, w, span, degree = degree, parametric = parametric, : reciprocal condition number 1
```

```
## Warning in simpleLoess(y, x, w, span, degree = degree, parametric = parametric, : There are other near singularities as well. 1.01
```

```
## Warning in simpleLoess(y, x, w, span, degree = degree, parametric = parametric, : zero-width neighborhood. make span bigger
```

```
## Warning: Computation failed in `stat_smooth()`:
## NA/NaN/Inf in foreign function call (arg 5)
```

```
## Warning in simpleLoess(y, x, w, span, degree = degree, parametric = parametric, : at -0.005
```

```
## Warning in simpleLoess(y, x, w, span, degree = degree, parametric = parametric, : radius 2.5e-05
```

```
## Warning in simpleLoess(y, x, w, span, degree = degree, parametric = parametric, : all data on boundary of neighborhood. make span bigger
```

```
## Warning in simpleLoess(y, x, w, span, degree = degree, parametric = parametric, : pseudoinverse used at -0.005
```

```
## Warning in simpleLoess(y, x, w, span, degree = degree, parametric = parametric, : neighborhood radius 0.005
```

```
## Warning in simpleLoess(y, x, w, span, degree = degree, parametric = parametric, : reciprocal condition number 1
```

```
## Warning in simpleLoess(y, x, w, span, degree = degree, parametric = parametric, : There are other near singularities as well. 1.01
```

```
## Warning in simpleLoess(y, x, w, span, degree = degree, parametric = parametric, : zero-width neighborhood. make span bigger
```

```
## Warning: Computation failed in `stat_smooth()`:
## NA/NaN/Inf in foreign function call (arg 5)
```

```
## Warning in simpleLoess(y, x, w, span, degree = degree, parametric = parametric, : at -0.005
```

```
## Warning in simpleLoess(y, x, w, span, degree = degree, parametric = parametric, : radius 2.5e-05
```

```
## Warning in simpleLoess(y, x, w, span, degree = degree, parametric = parametric, : all data on boundary of neighborhood. make span bigger
```

```
## Warning in simpleLoess(y, x, w, span, degree = degree, parametric = parametric, : pseudoinverse used at -0.005
```

```
## Warning in simpleLoess(y, x, w, span, degree = degree, parametric = parametric, : neighborhood radius 0.005
```

```
## Warning in simpleLoess(y, x, w, span, degree = degree, parametric = parametric, : reciprocal condition number 1
```

```
## Warning in simpleLoess(y, x, w, span, degree = degree, parametric = parametric, : There are other near singularities as well. 1.01
```

```
## Warning in simpleLoess(y, x, w, span, degree = degree, parametric = parametric, : zero-width neighborhood. make span bigger
```

```
## Warning: Computation failed in `stat_smooth()`:
## NA/NaN/Inf in foreign function call (arg 5)
```

```
## Warning in simpleLoess(y, x, w, span, degree = degree, parametric = parametric, : at -0.005
```

```
## Warning in simpleLoess(y, x, w, span, degree = degree, parametric = parametric, : radius 2.5e-05
```

```
## Warning in simpleLoess(y, x, w, span, degree = degree, parametric = parametric, : all data on boundary of neighborhood. make span bigger
```

```
## Warning in simpleLoess(y, x, w, span, degree = degree, parametric = parametric, : pseudoinverse used at -0.005
```

```
## Warning in simpleLoess(y, x, w, span, degree = degree, parametric = parametric, : neighborhood radius 0.005
```

```
## Warning in simpleLoess(y, x, w, span, degree = degree, parametric = parametric, : reciprocal condition number 1
```

```
## Warning in simpleLoess(y, x, w, span, degree = degree, parametric = parametric, : There are other near singularities as well. 1.01
```

```
## Warning in simpleLoess(y, x, w, span, degree = degree, parametric = parametric, : zero-width neighborhood. make span bigger
```

```
## Warning: Computation failed in `stat_smooth()`:
## NA/NaN/Inf in foreign function call (arg 5)
```

```
## Warning in simpleLoess(y, x, w, span, degree = degree, parametric = parametric, : at -0.005
```

```
## Warning in simpleLoess(y, x, w, span, degree = degree, parametric = parametric, : radius 2.5e-05
```

```
## Warning in simpleLoess(y, x, w, span, degree = degree, parametric = parametric, : all data on boundary of neighborhood. make span bigger
```

```
## Warning in simpleLoess(y, x, w, span, degree = degree, parametric = parametric, : pseudoinverse used at -0.005
```

```
## Warning in simpleLoess(y, x, w, span, degree = degree, parametric = parametric, : neighborhood radius 0.005
```

```
## Warning in simpleLoess(y, x, w, span, degree = degree, parametric = parametric, : reciprocal condition number 1
```

```
## Warning in simpleLoess(y, x, w, span, degree = degree, parametric = parametric, : There are other near singularities as well. 1.01
```

```
## Warning in simpleLoess(y, x, w, span, degree = degree, parametric = parametric, : zero-width neighborhood. make span bigger
```

```
## Warning: Computation failed in `stat_smooth()`:
## NA/NaN/Inf in foreign function call (arg 5)
```

```
## Warning in simpleLoess(y, x, w, span, degree = degree, parametric = parametric, : at -0.005
```

```
## Warning in simpleLoess(y, x, w, span, degree = degree, parametric = parametric, : radius 2.5e-05
```

```
## Warning in simpleLoess(y, x, w, span, degree = degree, parametric = parametric, : all data on boundary of neighborhood. make span bigger
```

```
## Warning in simpleLoess(y, x, w, span, degree = degree, parametric = parametric, : pseudoinverse used at -0.005
```

```
## Warning in simpleLoess(y, x, w, span, degree = degree, parametric = parametric, : neighborhood radius 0.005
```

```
## Warning in simpleLoess(y, x, w, span, degree = degree, parametric = parametric, : reciprocal condition number 1
```

```
## Warning in simpleLoess(y, x, w, span, degree = degree, parametric = parametric, : There are other near singularities as well. 1.01
```

```
## Warning in simpleLoess(y, x, w, span, degree = degree, parametric = parametric, : zero-width neighborhood. make span bigger
```

```
## Warning: Computation failed in `stat_smooth()`:
## NA/NaN/Inf in foreign function call (arg 5)
```

```
## Warning in simpleLoess(y, x, w, span, degree = degree, parametric = parametric, : at -0.005
```

```
## Warning in simpleLoess(y, x, w, span, degree = degree, parametric = parametric, : radius 2.5e-05
```

```
## Warning in simpleLoess(y, x, w, span, degree = degree, parametric = parametric, : all data on boundary of neighborhood. make span bigger
```

```
## Warning in simpleLoess(y, x, w, span, degree = degree, parametric = parametric, : pseudoinverse used at -0.005
```

```
## Warning in simpleLoess(y, x, w, span, degree = degree, parametric = parametric, : neighborhood radius 0.005
```

```
## Warning in simpleLoess(y, x, w, span, degree = degree, parametric = parametric, : reciprocal condition number 1
```

```
## Warning in simpleLoess(y, x, w, span, degree = degree, parametric = parametric, : There are other near singularities as well. 1.01
```

```
## Warning in simpleLoess(y, x, w, span, degree = degree, parametric = parametric, : zero-width neighborhood. make span bigger
```

```
## Warning: Computation failed in `stat_smooth()`:
## NA/NaN/Inf in foreign function call (arg 5)
```

```
## Warning in simpleLoess(y, x, w, span, degree = degree, parametric = parametric, : at -0.005
```

```
## Warning in simpleLoess(y, x, w, span, degree = degree, parametric = parametric, : radius 2.5e-05
```

```
## Warning in simpleLoess(y, x, w, span, degree = degree, parametric = parametric, : all data on boundary of neighborhood. make span bigger
```

```
## Warning in simpleLoess(y, x, w, span, degree = degree, parametric = parametric, : pseudoinverse used at -0.005
```

```
## Warning in simpleLoess(y, x, w, span, degree = degree, parametric = parametric, : neighborhood radius 0.005
```

```
## Warning in simpleLoess(y, x, w, span, degree = degree, parametric = parametric, : reciprocal condition number 1
```

```
## Warning in simpleLoess(y, x, w, span, degree = degree, parametric = parametric, : There are other near singularities as well. 1.01
```

```
## Warning in simpleLoess(y, x, w, span, degree = degree, parametric = parametric, : zero-width neighborhood. make span bigger
```

```
## Warning: Computation failed in `stat_smooth()`:
## NA/NaN/Inf in foreign function call (arg 5)
```

```
## Warning in simpleLoess(y, x, w, span, degree = degree, parametric = parametric, : pseudoinverse used at -0.005
```

```
## Warning in simpleLoess(y, x, w, span, degree = degree, parametric = parametric, : neighborhood radius 1.005
```

```
## Warning in simpleLoess(y, x, w, span, degree = degree, parametric = parametric, : reciprocal condition number 0
```

```
## Warning in simpleLoess(y, x, w, span, degree = degree, parametric = parametric, : There are other near singularities as well. 1.01
```

```
## Warning in predLoess(object$y, object$x, newx = if (is.null(newdata)) object$x else if (is.data.frame(newdata)) as.matrix(model.frame(delete.response(terms(object)), : pseudoinverse used at -0.005
```

```
## Warning in predLoess(object$y, object$x, newx = if (is.null(newdata)) object$x else if (is.data.frame(newdata)) as.matrix(model.frame(delete.response(terms(object)), : neighborhood radius 1.005
```

```
## Warning in predLoess(object$y, object$x, newx = if (is.null(newdata)) object$x else if (is.data.frame(newdata)) as.matrix(model.frame(delete.response(terms(object)), : reciprocal condition number 0
```

```
## Warning in predLoess(object$y, object$x, newx = if (is.null(newdata)) object$x else if (is.data.frame(newdata)) as.matrix(model.frame(delete.response(terms(object)), : There are other near
## singularities as well. 1.01
```

```
## Warning in simpleLoess(y, x, w, span, degree = degree, parametric = parametric, : pseudoinverse used at -0.005
```

```
## Warning in simpleLoess(y, x, w, span, degree = degree, parametric = parametric, : neighborhood radius 1.005
```

```
## Warning in simpleLoess(y, x, w, span, degree = degree, parametric = parametric, : reciprocal condition number 0
```

```
## Warning in simpleLoess(y, x, w, span, degree = degree, parametric = parametric, : There are other near singularities as well. 1.01
```

```
## Warning in predLoess(object$y, object$x, newx = if (is.null(newdata)) object$x else if (is.data.frame(newdata)) as.matrix(model.frame(delete.response(terms(object)), : pseudoinverse used at -0.005
```

```
## Warning in predLoess(object$y, object$x, newx = if (is.null(newdata)) object$x else if (is.data.frame(newdata)) as.matrix(model.frame(delete.response(terms(object)), : neighborhood radius 1.005
```

```
## Warning in predLoess(object$y, object$x, newx = if (is.null(newdata)) object$x else if (is.data.frame(newdata)) as.matrix(model.frame(delete.response(terms(object)), : reciprocal condition number 0
```

```
## Warning in predLoess(object$y, object$x, newx = if (is.null(newdata)) object$x else if (is.data.frame(newdata)) as.matrix(model.frame(delete.response(terms(object)), : There are other near
## singularities as well. 1.01
```

```
## Warning in simpleLoess(y, x, w, span, degree = degree, parametric = parametric, : at -0.005
```

```
## Warning in simpleLoess(y, x, w, span, degree = degree, parametric = parametric, : radius 2.5e-05
```

```
## Warning in simpleLoess(y, x, w, span, degree = degree, parametric = parametric, : all data on boundary of neighborhood. make span bigger
```

```
## Warning in simpleLoess(y, x, w, span, degree = degree, parametric = parametric, : pseudoinverse used at -0.005
```

```
## Warning in simpleLoess(y, x, w, span, degree = degree, parametric = parametric, : neighborhood radius 0.005
```

```
## Warning in simpleLoess(y, x, w, span, degree = degree, parametric = parametric, : reciprocal condition number 1
```

```
## Warning in simpleLoess(y, x, w, span, degree = degree, parametric = parametric, : There are other near singularities as well. 1.01
```

```
## Warning in simpleLoess(y, x, w, span, degree = degree, parametric = parametric, : zero-width neighborhood. make span bigger
```

```
## Warning: Computation failed in `stat_smooth()`:
## NA/NaN/Inf in foreign function call (arg 5)
```

```
## Warning in simpleLoess(y, x, w, span, degree = degree, parametric = parametric, : pseudoinverse used at -0.005
```

```
## Warning in simpleLoess(y, x, w, span, degree = degree, parametric = parametric, : neighborhood radius 1.005
```

```
## Warning in simpleLoess(y, x, w, span, degree = degree, parametric = parametric, : reciprocal condition number 0
```

```
## Warning in simpleLoess(y, x, w, span, degree = degree, parametric = parametric, : There are other near singularities as well. 1.01
```

```
## Warning in predLoess(object$y, object$x, newx = if (is.null(newdata)) object$x else if (is.data.frame(newdata)) as.matrix(model.frame(delete.response(terms(object)), : pseudoinverse used at -0.005
```

```
## Warning in predLoess(object$y, object$x, newx = if (is.null(newdata)) object$x else if (is.data.frame(newdata)) as.matrix(model.frame(delete.response(terms(object)), : neighborhood radius 1.005
```

```
## Warning in predLoess(object$y, object$x, newx = if (is.null(newdata)) object$x else if (is.data.frame(newdata)) as.matrix(model.frame(delete.response(terms(object)), : reciprocal condition number 0
```

```
## Warning in predLoess(object$y, object$x, newx = if (is.null(newdata)) object$x else if (is.data.frame(newdata)) as.matrix(model.frame(delete.response(terms(object)), : There are other near
## singularities as well. 1.01
```

```
## Warning in simpleLoess(y, x, w, span, degree = degree, parametric = parametric, : at -0.005
```

```
## Warning in simpleLoess(y, x, w, span, degree = degree, parametric = parametric, : radius 2.5e-05
```

```
## Warning in simpleLoess(y, x, w, span, degree = degree, parametric = parametric, : all data on boundary of neighborhood. make span bigger
```

```
## Warning in simpleLoess(y, x, w, span, degree = degree, parametric = parametric, : pseudoinverse used at -0.005
```

```
## Warning in simpleLoess(y, x, w, span, degree = degree, parametric = parametric, : neighborhood radius 0.005
```

```
## Warning in simpleLoess(y, x, w, span, degree = degree, parametric = parametric, : reciprocal condition number 1
```

```
## Warning in simpleLoess(y, x, w, span, degree = degree, parametric = parametric, : There are other near singularities as well. 1.01
```

```
## Warning in simpleLoess(y, x, w, span, degree = degree, parametric = parametric, : zero-width neighborhood. make span bigger
```

```
## Warning: Computation failed in `stat_smooth()`:
## NA/NaN/Inf in foreign function call (arg 5)
```

```
## Warning in simpleLoess(y, x, w, span, degree = degree, parametric = parametric, : at -0.005
```

```
## Warning in simpleLoess(y, x, w, span, degree = degree, parametric = parametric, : radius 2.5e-05
```

```
## Warning in simpleLoess(y, x, w, span, degree = degree, parametric = parametric, : all data on boundary of neighborhood. make span bigger
```

```
## Warning in simpleLoess(y, x, w, span, degree = degree, parametric = parametric, : pseudoinverse used at -0.005
```

```
## Warning in simpleLoess(y, x, w, span, degree = degree, parametric = parametric, : neighborhood radius 0.005
```

```
## Warning in simpleLoess(y, x, w, span, degree = degree, parametric = parametric, : reciprocal condition number 1
```

```
## Warning in simpleLoess(y, x, w, span, degree = degree, parametric = parametric, : There are other near singularities as well. 1.01
```

```
## Warning in simpleLoess(y, x, w, span, degree = degree, parametric = parametric, : zero-width neighborhood. make span bigger
```

```
## Warning: Computation failed in `stat_smooth()`:
## NA/NaN/Inf in foreign function call (arg 5)
```

```
## Warning in simpleLoess(y, x, w, span, degree = degree, parametric = parametric, : at -0.005
```

```
## Warning in simpleLoess(y, x, w, span, degree = degree, parametric = parametric, : radius 2.5e-05
```

```
## Warning in simpleLoess(y, x, w, span, degree = degree, parametric = parametric, : all data on boundary of neighborhood. make span bigger
```

```
## Warning in simpleLoess(y, x, w, span, degree = degree, parametric = parametric, : pseudoinverse used at -0.005
```

```
## Warning in simpleLoess(y, x, w, span, degree = degree, parametric = parametric, : neighborhood radius 0.005
```

```
## Warning in simpleLoess(y, x, w, span, degree = degree, parametric = parametric, : reciprocal condition number 1
```

```
## Warning in simpleLoess(y, x, w, span, degree = degree, parametric = parametric, : There are other near singularities as well. 1.01
```

```
## Warning in simpleLoess(y, x, w, span, degree = degree, parametric = parametric, : zero-width neighborhood. make span bigger
```

```
## Warning: Computation failed in `stat_smooth()`:
## NA/NaN/Inf in foreign function call (arg 5)
```

```
## Warning in simpleLoess(y, x, w, span, degree = degree, parametric = parametric, : at -0.005
```

```
## Warning in simpleLoess(y, x, w, span, degree = degree, parametric = parametric, : radius 2.5e-05
```

```
## Warning in simpleLoess(y, x, w, span, degree = degree, parametric = parametric, : all data on boundary of neighborhood. make span bigger
```

```
## Warning in simpleLoess(y, x, w, span, degree = degree, parametric = parametric, : pseudoinverse used at -0.005
```

```
## Warning in simpleLoess(y, x, w, span, degree = degree, parametric = parametric, : neighborhood radius 0.005
```

```
## Warning in simpleLoess(y, x, w, span, degree = degree, parametric = parametric, : reciprocal condition number 1
```

```
## Warning in simpleLoess(y, x, w, span, degree = degree, parametric = parametric, : There are other near singularities as well. 1.01
```

```
## Warning in simpleLoess(y, x, w, span, degree = degree, parametric = parametric, : zero-width neighborhood. make span bigger
```

```
## Warning: Computation failed in `stat_smooth()`:
## NA/NaN/Inf in foreign function call (arg 5)
```

```
## Warning in simpleLoess(y, x, w, span, degree = degree, parametric = parametric, : at -0.005
```

```
## Warning in simpleLoess(y, x, w, span, degree = degree, parametric = parametric, : radius 2.5e-05
```

```
## Warning in simpleLoess(y, x, w, span, degree = degree, parametric = parametric, : all data on boundary of neighborhood. make span bigger
```

```
## Warning in simpleLoess(y, x, w, span, degree = degree, parametric = parametric, : pseudoinverse used at -0.005
```

```
## Warning in simpleLoess(y, x, w, span, degree = degree, parametric = parametric, : neighborhood radius 0.005
```

```
## Warning in simpleLoess(y, x, w, span, degree = degree, parametric = parametric, : reciprocal condition number 1
```

```
## Warning in simpleLoess(y, x, w, span, degree = degree, parametric = parametric, : There are other near singularities as well. 1.01
```

```
## Warning in simpleLoess(y, x, w, span, degree = degree, parametric = parametric, : zero-width neighborhood. make span bigger
```

```
## Warning: Computation failed in `stat_smooth()`:
## NA/NaN/Inf in foreign function call (arg 5)
```

```
## Warning in simpleLoess(y, x, w, span, degree = degree, parametric = parametric, : at -0.005
```

```
## Warning in simpleLoess(y, x, w, span, degree = degree, parametric = parametric, : radius 2.5e-05
```

```
## Warning in simpleLoess(y, x, w, span, degree = degree, parametric = parametric, : all data on boundary of neighborhood. make span bigger
```

```
## Warning in simpleLoess(y, x, w, span, degree = degree, parametric = parametric, : pseudoinverse used at -0.005
```

```
## Warning in simpleLoess(y, x, w, span, degree = degree, parametric = parametric, : neighborhood radius 0.005
```

```
## Warning in simpleLoess(y, x, w, span, degree = degree, parametric = parametric, : reciprocal condition number 1
```

```
## Warning in simpleLoess(y, x, w, span, degree = degree, parametric = parametric, : There are other near singularities as well. 1.01
```

```
## Warning in simpleLoess(y, x, w, span, degree = degree, parametric = parametric, : zero-width neighborhood. make span bigger
```

```
## Warning: Computation failed in `stat_smooth()`:
## NA/NaN/Inf in foreign function call (arg 5)
```

```
## Warning in simpleLoess(y, x, w, span, degree = degree, parametric = parametric, : at -0.005
```

```
## Warning in simpleLoess(y, x, w, span, degree = degree, parametric = parametric, : radius 2.5e-05
```

```
## Warning in simpleLoess(y, x, w, span, degree = degree, parametric = parametric, : all data on boundary of neighborhood. make span bigger
```

```
## Warning in simpleLoess(y, x, w, span, degree = degree, parametric = parametric, : pseudoinverse used at -0.005
```

```
## Warning in simpleLoess(y, x, w, span, degree = degree, parametric = parametric, : neighborhood radius 0.005
```

```
## Warning in simpleLoess(y, x, w, span, degree = degree, parametric = parametric, : reciprocal condition number 1
```

```
## Warning in simpleLoess(y, x, w, span, degree = degree, parametric = parametric, : There are other near singularities as well. 1.01
```

```
## Warning in simpleLoess(y, x, w, span, degree = degree, parametric = parametric, : zero-width neighborhood. make span bigger
```

```
## Warning: Computation failed in `stat_smooth()`:
## NA/NaN/Inf in foreign function call (arg 5)
```

```
## Warning in simpleLoess(y, x, w, span, degree = degree, parametric = parametric, : at -0.005
```

```
## Warning in simpleLoess(y, x, w, span, degree = degree, parametric = parametric, : radius 2.5e-05
```

```
## Warning in simpleLoess(y, x, w, span, degree = degree, parametric = parametric, : all data on boundary of neighborhood. make span bigger
```

```
## Warning in simpleLoess(y, x, w, span, degree = degree, parametric = parametric, : pseudoinverse used at -0.005
```

```
## Warning in simpleLoess(y, x, w, span, degree = degree, parametric = parametric, : neighborhood radius 0.005
```

```
## Warning in simpleLoess(y, x, w, span, degree = degree, parametric = parametric, : reciprocal condition number 1
```

```
## Warning in simpleLoess(y, x, w, span, degree = degree, parametric = parametric, : There are other near singularities as well. 1.01
```

```
## Warning in simpleLoess(y, x, w, span, degree = degree, parametric = parametric, : zero-width neighborhood. make span bigger
```

```
## Warning: Computation failed in `stat_smooth()`:
## NA/NaN/Inf in foreign function call (arg 5)
```

```
## Warning in simpleLoess(y, x, w, span, degree = degree, parametric = parametric, : at -0.005
```

```
## Warning in simpleLoess(y, x, w, span, degree = degree, parametric = parametric, : radius 2.5e-05
```

```
## Warning in simpleLoess(y, x, w, span, degree = degree, parametric = parametric, : all data on boundary of neighborhood. make span bigger
```

```
## Warning in simpleLoess(y, x, w, span, degree = degree, parametric = parametric, : pseudoinverse used at -0.005
```

```
## Warning in simpleLoess(y, x, w, span, degree = degree, parametric = parametric, : neighborhood radius 0.005
```

```
## Warning in simpleLoess(y, x, w, span, degree = degree, parametric = parametric, : reciprocal condition number 1
```

```
## Warning in simpleLoess(y, x, w, span, degree = degree, parametric = parametric, : There are other near singularities as well. 1.01
```

```
## Warning in simpleLoess(y, x, w, span, degree = degree, parametric = parametric, : zero-width neighborhood. make span bigger
```

```
## Warning: Computation failed in `stat_smooth()`:
## NA/NaN/Inf in foreign function call (arg 5)
```

```
## Warning in simpleLoess(y, x, w, span, degree = degree, parametric = parametric, : at -0.005
```

```
## Warning in simpleLoess(y, x, w, span, degree = degree, parametric = parametric, : radius 2.5e-05
```

```
## Warning in simpleLoess(y, x, w, span, degree = degree, parametric = parametric, : all data on boundary of neighborhood. make span bigger
```

```
## Warning in simpleLoess(y, x, w, span, degree = degree, parametric = parametric, : pseudoinverse used at -0.005
```

```
## Warning in simpleLoess(y, x, w, span, degree = degree, parametric = parametric, : neighborhood radius 0.005
```

```
## Warning in simpleLoess(y, x, w, span, degree = degree, parametric = parametric, : reciprocal condition number 1
```

```
## Warning in simpleLoess(y, x, w, span, degree = degree, parametric = parametric, : There are other near singularities as well. 1.01
```

```
## Warning in simpleLoess(y, x, w, span, degree = degree, parametric = parametric, : zero-width neighborhood. make span bigger
```

```
## Warning: Computation failed in `stat_smooth()`:
## NA/NaN/Inf in foreign function call (arg 5)
```

```
## Warning in simpleLoess(y, x, w, span, degree = degree, parametric = parametric, : at -0.005
```

```
## Warning in simpleLoess(y, x, w, span, degree = degree, parametric = parametric, : radius 2.5e-05
```

```
## Warning in simpleLoess(y, x, w, span, degree = degree, parametric = parametric, : all data on boundary of neighborhood. make span bigger
```

```
## Warning in simpleLoess(y, x, w, span, degree = degree, parametric = parametric, : pseudoinverse used at -0.005
```

```
## Warning in simpleLoess(y, x, w, span, degree = degree, parametric = parametric, : neighborhood radius 0.005
```

```
## Warning in simpleLoess(y, x, w, span, degree = degree, parametric = parametric, : reciprocal condition number 1
```

```
## Warning in simpleLoess(y, x, w, span, degree = degree, parametric = parametric, : There are other near singularities as well. 1.01
```

```
## Warning in simpleLoess(y, x, w, span, degree = degree, parametric = parametric, : zero-width neighborhood. make span bigger
```

```
## Warning: Computation failed in `stat_smooth()`:
## NA/NaN/Inf in foreign function call (arg 5)
```

```
## Warning in simpleLoess(y, x, w, span, degree = degree, parametric = parametric, : at -0.005
```

```
## Warning in simpleLoess(y, x, w, span, degree = degree, parametric = parametric, : radius 2.5e-05
```

```
## Warning in simpleLoess(y, x, w, span, degree = degree, parametric = parametric, : all data on boundary of neighborhood. make span bigger
```

```
## Warning in simpleLoess(y, x, w, span, degree = degree, parametric = parametric, : pseudoinverse used at -0.005
```

```
## Warning in simpleLoess(y, x, w, span, degree = degree, parametric = parametric, : neighborhood radius 0.005
```

```
## Warning in simpleLoess(y, x, w, span, degree = degree, parametric = parametric, : reciprocal condition number 1
```

```
## Warning in simpleLoess(y, x, w, span, degree = degree, parametric = parametric, : There are other near singularities as well. 1.01
```

```
## Warning in simpleLoess(y, x, w, span, degree = degree, parametric = parametric, : zero-width neighborhood. make span bigger
```

```
## Warning: Computation failed in `stat_smooth()`:
## NA/NaN/Inf in foreign function call (arg 5)
```

```
## Warning in simpleLoess(y, x, w, span, degree = degree, parametric = parametric, : at -0.005
```

```
## Warning in simpleLoess(y, x, w, span, degree = degree, parametric = parametric, : radius 2.5e-05
```

```
## Warning in simpleLoess(y, x, w, span, degree = degree, parametric = parametric, : all data on boundary of neighborhood. make span bigger
```

```
## Warning in simpleLoess(y, x, w, span, degree = degree, parametric = parametric, : pseudoinverse used at -0.005
```

```
## Warning in simpleLoess(y, x, w, span, degree = degree, parametric = parametric, : neighborhood radius 0.005
```

```
## Warning in simpleLoess(y, x, w, span, degree = degree, parametric = parametric, : reciprocal condition number 1
```

```
## Warning in simpleLoess(y, x, w, span, degree = degree, parametric = parametric, : There are other near singularities as well. 1.01
```

```
## Warning in simpleLoess(y, x, w, span, degree = degree, parametric = parametric, : zero-width neighborhood. make span bigger
```

```
## Warning: Computation failed in `stat_smooth()`:
## NA/NaN/Inf in foreign function call (arg 5)
```

```
## Warning in simpleLoess(y, x, w, span, degree = degree, parametric = parametric, : at -0.005
```

```
## Warning in simpleLoess(y, x, w, span, degree = degree, parametric = parametric, : radius 2.5e-05
```

```
## Warning in simpleLoess(y, x, w, span, degree = degree, parametric = parametric, : all data on boundary of neighborhood. make span bigger
```

```
## Warning in simpleLoess(y, x, w, span, degree = degree, parametric = parametric, : pseudoinverse used at -0.005
```

```
## Warning in simpleLoess(y, x, w, span, degree = degree, parametric = parametric, : neighborhood radius 0.005
```

```
## Warning in simpleLoess(y, x, w, span, degree = degree, parametric = parametric, : reciprocal condition number 1
```

```
## Warning in simpleLoess(y, x, w, span, degree = degree, parametric = parametric, : There are other near singularities as well. 1.01
```

```
## Warning in simpleLoess(y, x, w, span, degree = degree, parametric = parametric, : zero-width neighborhood. make span bigger
```

```
## Warning: Computation failed in `stat_smooth()`:
## NA/NaN/Inf in foreign function call (arg 5)
```

```
## Warning in simpleLoess(y, x, w, span, degree = degree, parametric = parametric, : pseudoinverse used at -0.005
```

```
## Warning in simpleLoess(y, x, w, span, degree = degree, parametric = parametric, : neighborhood radius 1.005
```

```
## Warning in simpleLoess(y, x, w, span, degree = degree, parametric = parametric, : reciprocal condition number 0
```

```
## Warning in simpleLoess(y, x, w, span, degree = degree, parametric = parametric, : There are other near singularities as well. 1.01
```

```
## Warning in predLoess(object$y, object$x, newx = if (is.null(newdata)) object$x else if (is.data.frame(newdata)) as.matrix(model.frame(delete.response(terms(object)), : pseudoinverse used at -0.005
```

```
## Warning in predLoess(object$y, object$x, newx = if (is.null(newdata)) object$x else if (is.data.frame(newdata)) as.matrix(model.frame(delete.response(terms(object)), : neighborhood radius 1.005
```

```
## Warning in predLoess(object$y, object$x, newx = if (is.null(newdata)) object$x else if (is.data.frame(newdata)) as.matrix(model.frame(delete.response(terms(object)), : reciprocal condition number 0
```

```
## Warning in predLoess(object$y, object$x, newx = if (is.null(newdata)) object$x else if (is.data.frame(newdata)) as.matrix(model.frame(delete.response(terms(object)), : There are other near
## singularities as well. 1.01
```

```
## Warning in simpleLoess(y, x, w, span, degree = degree, parametric = parametric, : at -0.005
```

```
## Warning in simpleLoess(y, x, w, span, degree = degree, parametric = parametric, : radius 2.5e-05
```

```
## Warning in simpleLoess(y, x, w, span, degree = degree, parametric = parametric, : all data on boundary of neighborhood. make span bigger
```

```
## Warning in simpleLoess(y, x, w, span, degree = degree, parametric = parametric, : pseudoinverse used at -0.005
```

```
## Warning in simpleLoess(y, x, w, span, degree = degree, parametric = parametric, : neighborhood radius 0.005
```

```
## Warning in simpleLoess(y, x, w, span, degree = degree, parametric = parametric, : reciprocal condition number 1
```

```
## Warning in simpleLoess(y, x, w, span, degree = degree, parametric = parametric, : There are other near singularities as well. 1.01
```

```
## Warning in simpleLoess(y, x, w, span, degree = degree, parametric = parametric, : zero-width neighborhood. make span bigger
```

```
## Warning: Computation failed in `stat_smooth()`:
## NA/NaN/Inf in foreign function call (arg 5)
```

```
## Warning in simpleLoess(y, x, w, span, degree = degree, parametric = parametric, : at -0.005
```

```
## Warning in simpleLoess(y, x, w, span, degree = degree, parametric = parametric, : radius 2.5e-05
```

```
## Warning in simpleLoess(y, x, w, span, degree = degree, parametric = parametric, : all data on boundary of neighborhood. make span bigger
```

```
## Warning in simpleLoess(y, x, w, span, degree = degree, parametric = parametric, : pseudoinverse used at -0.005
```

```
## Warning in simpleLoess(y, x, w, span, degree = degree, parametric = parametric, : neighborhood radius 0.005
```

```
## Warning in simpleLoess(y, x, w, span, degree = degree, parametric = parametric, : reciprocal condition number 1
```

```
## Warning in simpleLoess(y, x, w, span, degree = degree, parametric = parametric, : There are other near singularities as well. 1.01
```

```
## Warning in simpleLoess(y, x, w, span, degree = degree, parametric = parametric, : zero-width neighborhood. make span bigger
```

```
## Warning: Computation failed in `stat_smooth()`:
## NA/NaN/Inf in foreign function call (arg 5)
```

```
## Warning in simpleLoess(y, x, w, span, degree = degree, parametric = parametric, : at -0.005
```

```
## Warning in simpleLoess(y, x, w, span, degree = degree, parametric = parametric, : radius 2.5e-05
```

```
## Warning in simpleLoess(y, x, w, span, degree = degree, parametric = parametric, : all data on boundary of neighborhood. make span bigger
```

```
## Warning in simpleLoess(y, x, w, span, degree = degree, parametric = parametric, : pseudoinverse used at -0.005
```

```
## Warning in simpleLoess(y, x, w, span, degree = degree, parametric = parametric, : neighborhood radius 0.005
```

```
## Warning in simpleLoess(y, x, w, span, degree = degree, parametric = parametric, : reciprocal condition number 1
```

```
## Warning in simpleLoess(y, x, w, span, degree = degree, parametric = parametric, : There are other near singularities as well. 1.01
```

```
## Warning in simpleLoess(y, x, w, span, degree = degree, parametric = parametric, : zero-width neighborhood. make span bigger
```

```
## Warning: Computation failed in `stat_smooth()`:
## NA/NaN/Inf in foreign function call (arg 5)
```

```
## Warning in simpleLoess(y, x, w, span, degree = degree, parametric = parametric, : at -0.005
```

```
## Warning in simpleLoess(y, x, w, span, degree = degree, parametric = parametric, : radius 2.5e-05
```

```
## Warning in simpleLoess(y, x, w, span, degree = degree, parametric = parametric, : all data on boundary of neighborhood. make span bigger
```

```
## Warning in simpleLoess(y, x, w, span, degree = degree, parametric = parametric, : pseudoinverse used at -0.005
```

```
## Warning in simpleLoess(y, x, w, span, degree = degree, parametric = parametric, : neighborhood radius 0.005
```

```
## Warning in simpleLoess(y, x, w, span, degree = degree, parametric = parametric, : reciprocal condition number 1
```

```
## Warning in simpleLoess(y, x, w, span, degree = degree, parametric = parametric, : There are other near singularities as well. 1.01
```

```
## Warning in simpleLoess(y, x, w, span, degree = degree, parametric = parametric, : zero-width neighborhood. make span bigger
```

```
## Warning: Computation failed in `stat_smooth()`:
## NA/NaN/Inf in foreign function call (arg 5)
```

```
## Warning in simpleLoess(y, x, w, span, degree = degree, parametric = parametric, : at -0.005
```

```
## Warning in simpleLoess(y, x, w, span, degree = degree, parametric = parametric, : radius 2.5e-05
```

```
## Warning in simpleLoess(y, x, w, span, degree = degree, parametric = parametric, : all data on boundary of neighborhood. make span bigger
```

```
## Warning in simpleLoess(y, x, w, span, degree = degree, parametric = parametric, : pseudoinverse used at -0.005
```

```
## Warning in simpleLoess(y, x, w, span, degree = degree, parametric = parametric, : neighborhood radius 0.005
```

```
## Warning in simpleLoess(y, x, w, span, degree = degree, parametric = parametric, : reciprocal condition number 1
```

```
## Warning in simpleLoess(y, x, w, span, degree = degree, parametric = parametric, : There are other near singularities as well. 1.01
```

```
## Warning in simpleLoess(y, x, w, span, degree = degree, parametric = parametric, : zero-width neighborhood. make span bigger
```

```
## Warning: Computation failed in `stat_smooth()`:
## NA/NaN/Inf in foreign function call (arg 5)
```

```
## Warning in simpleLoess(y, x, w, span, degree = degree, parametric = parametric, : at -0.005
```

```
## Warning in simpleLoess(y, x, w, span, degree = degree, parametric = parametric, : radius 2.5e-05
```

```
## Warning in simpleLoess(y, x, w, span, degree = degree, parametric = parametric, : all data on boundary of neighborhood. make span bigger
```

```
## Warning in simpleLoess(y, x, w, span, degree = degree, parametric = parametric, : pseudoinverse used at -0.005
```

```
## Warning in simpleLoess(y, x, w, span, degree = degree, parametric = parametric, : neighborhood radius 0.005
```

```
## Warning in simpleLoess(y, x, w, span, degree = degree, parametric = parametric, : reciprocal condition number 1
```

```
## Warning in simpleLoess(y, x, w, span, degree = degree, parametric = parametric, : There are other near singularities as well. 1.01
```

```
## Warning in simpleLoess(y, x, w, span, degree = degree, parametric = parametric, : zero-width neighborhood. make span bigger
```

```
## Warning: Computation failed in `stat_smooth()`:
## NA/NaN/Inf in foreign function call (arg 5)
```

```
## Warning in simpleLoess(y, x, w, span, degree = degree, parametric = parametric, : at -0.005
```

```
## Warning in simpleLoess(y, x, w, span, degree = degree, parametric = parametric, : radius 2.5e-05
```

```
## Warning in simpleLoess(y, x, w, span, degree = degree, parametric = parametric, : all data on boundary of neighborhood. make span bigger
```

```
## Warning in simpleLoess(y, x, w, span, degree = degree, parametric = parametric, : pseudoinverse used at -0.005
```

```
## Warning in simpleLoess(y, x, w, span, degree = degree, parametric = parametric, : neighborhood radius 0.005
```

```
## Warning in simpleLoess(y, x, w, span, degree = degree, parametric = parametric, : reciprocal condition number 1
```

```
## Warning in simpleLoess(y, x, w, span, degree = degree, parametric = parametric, : There are other near singularities as well. 1.01
```

```
## Warning in simpleLoess(y, x, w, span, degree = degree, parametric = parametric, : zero-width neighborhood. make span bigger
```

```
## Warning: Computation failed in `stat_smooth()`:
## NA/NaN/Inf in foreign function call (arg 5)
```

```
## Warning in simpleLoess(y, x, w, span, degree = degree, parametric = parametric, : at -0.005
```

```
## Warning in simpleLoess(y, x, w, span, degree = degree, parametric = parametric, : radius 2.5e-05
```

```
## Warning in simpleLoess(y, x, w, span, degree = degree, parametric = parametric, : all data on boundary of neighborhood. make span bigger
```

```
## Warning in simpleLoess(y, x, w, span, degree = degree, parametric = parametric, : pseudoinverse used at -0.005
```

```
## Warning in simpleLoess(y, x, w, span, degree = degree, parametric = parametric, : neighborhood radius 0.005
```

```
## Warning in simpleLoess(y, x, w, span, degree = degree, parametric = parametric, : reciprocal condition number 1
```

```
## Warning in simpleLoess(y, x, w, span, degree = degree, parametric = parametric, : There are other near singularities as well. 1.01
```

```
## Warning in simpleLoess(y, x, w, span, degree = degree, parametric = parametric, : zero-width neighborhood. make span bigger
```

```
## Warning: Computation failed in `stat_smooth()`:
## NA/NaN/Inf in foreign function call (arg 5)
```

```
## Warning in simpleLoess(y, x, w, span, degree = degree, parametric = parametric, : at -0.005
```

```
## Warning in simpleLoess(y, x, w, span, degree = degree, parametric = parametric, : radius 2.5e-05
```

```
## Warning in simpleLoess(y, x, w, span, degree = degree, parametric = parametric, : all data on boundary of neighborhood. make span bigger
```

```
## Warning in simpleLoess(y, x, w, span, degree = degree, parametric = parametric, : pseudoinverse used at -0.005
```

```
## Warning in simpleLoess(y, x, w, span, degree = degree, parametric = parametric, : neighborhood radius 0.005
```

```
## Warning in simpleLoess(y, x, w, span, degree = degree, parametric = parametric, : reciprocal condition number 1
```

```
## Warning in simpleLoess(y, x, w, span, degree = degree, parametric = parametric, : There are other near singularities as well. 1.01
```

```
## Warning in simpleLoess(y, x, w, span, degree = degree, parametric = parametric, : zero-width neighborhood. make span bigger
```

```
## Warning: Computation failed in `stat_smooth()`:
## NA/NaN/Inf in foreign function call (arg 5)
```

```
## Warning in simpleLoess(y, x, w, span, degree = degree, parametric = parametric, : at -0.005
```

```
## Warning in simpleLoess(y, x, w, span, degree = degree, parametric = parametric, : radius 2.5e-05
```

```
## Warning in simpleLoess(y, x, w, span, degree = degree, parametric = parametric, : all data on boundary of neighborhood. make span bigger
```

```
## Warning in simpleLoess(y, x, w, span, degree = degree, parametric = parametric, : pseudoinverse used at -0.005
```

```
## Warning in simpleLoess(y, x, w, span, degree = degree, parametric = parametric, : neighborhood radius 0.005
```

```
## Warning in simpleLoess(y, x, w, span, degree = degree, parametric = parametric, : reciprocal condition number 1
```

```
## Warning in simpleLoess(y, x, w, span, degree = degree, parametric = parametric, : There are other near singularities as well. 1.01
```

```
## Warning in simpleLoess(y, x, w, span, degree = degree, parametric = parametric, : zero-width neighborhood. make span bigger
```

```
## Warning: Computation failed in `stat_smooth()`:
## NA/NaN/Inf in foreign function call (arg 5)
```

```
## Warning in simpleLoess(y, x, w, span, degree = degree, parametric = parametric, : at -0.005
```

```
## Warning in simpleLoess(y, x, w, span, degree = degree, parametric = parametric, : radius 2.5e-05
```

```
## Warning in simpleLoess(y, x, w, span, degree = degree, parametric = parametric, : all data on boundary of neighborhood. make span bigger
```

```
## Warning in simpleLoess(y, x, w, span, degree = degree, parametric = parametric, : pseudoinverse used at -0.005
```

```
## Warning in simpleLoess(y, x, w, span, degree = degree, parametric = parametric, : neighborhood radius 0.005
```

```
## Warning in simpleLoess(y, x, w, span, degree = degree, parametric = parametric, : reciprocal condition number 1
```

```
## Warning in simpleLoess(y, x, w, span, degree = degree, parametric = parametric, : There are other near singularities as well. 1.01
```

```
## Warning in simpleLoess(y, x, w, span, degree = degree, parametric = parametric, : zero-width neighborhood. make span bigger
```

```
## Warning: Computation failed in `stat_smooth()`:
## NA/NaN/Inf in foreign function call (arg 5)
```

```
## Warning in simpleLoess(y, x, w, span, degree = degree, parametric = parametric, : pseudoinverse used at -0.005
```

```
## Warning in simpleLoess(y, x, w, span, degree = degree, parametric = parametric, : neighborhood radius 1.005
```

```
## Warning in simpleLoess(y, x, w, span, degree = degree, parametric = parametric, : reciprocal condition number 0
```

```
## Warning in simpleLoess(y, x, w, span, degree = degree, parametric = parametric, : There are other near singularities as well. 1.01
```

```
## Warning in predLoess(object$y, object$x, newx = if (is.null(newdata)) object$x else if (is.data.frame(newdata)) as.matrix(model.frame(delete.response(terms(object)), : pseudoinverse used at -0.005
```

```
## Warning in predLoess(object$y, object$x, newx = if (is.null(newdata)) object$x else if (is.data.frame(newdata)) as.matrix(model.frame(delete.response(terms(object)), : neighborhood radius 1.005
```

```
## Warning in predLoess(object$y, object$x, newx = if (is.null(newdata)) object$x else if (is.data.frame(newdata)) as.matrix(model.frame(delete.response(terms(object)), : reciprocal condition number 0
```

```
## Warning in predLoess(object$y, object$x, newx = if (is.null(newdata)) object$x else if (is.data.frame(newdata)) as.matrix(model.frame(delete.response(terms(object)), : There are other near
## singularities as well. 1.01
```

```
## Warning in simpleLoess(y, x, w, span, degree = degree, parametric = parametric, : at -0.005
```

```
## Warning in simpleLoess(y, x, w, span, degree = degree, parametric = parametric, : radius 2.5e-05
```

```
## Warning in simpleLoess(y, x, w, span, degree = degree, parametric = parametric, : all data on boundary of neighborhood. make span bigger
```

```
## Warning in simpleLoess(y, x, w, span, degree = degree, parametric = parametric, : pseudoinverse used at -0.005
```

```
## Warning in simpleLoess(y, x, w, span, degree = degree, parametric = parametric, : neighborhood radius 0.005
```

```
## Warning in simpleLoess(y, x, w, span, degree = degree, parametric = parametric, : reciprocal condition number 1
```

```
## Warning in simpleLoess(y, x, w, span, degree = degree, parametric = parametric, : There are other near singularities as well. 1.01
```

```
## Warning in simpleLoess(y, x, w, span, degree = degree, parametric = parametric, : zero-width neighborhood. make span bigger
```

```
## Warning: Computation failed in `stat_smooth()`:
## NA/NaN/Inf in foreign function call (arg 5)
```

```
## Warning in simpleLoess(y, x, w, span, degree = degree, parametric = parametric, : pseudoinverse used at -0.005
```

```
## Warning in simpleLoess(y, x, w, span, degree = degree, parametric = parametric, : neighborhood radius 1.005
```

```
## Warning in simpleLoess(y, x, w, span, degree = degree, parametric = parametric, : reciprocal condition number 0
```

```
## Warning in simpleLoess(y, x, w, span, degree = degree, parametric = parametric, : There are other near singularities as well. 1.01
```

```
## Warning in predLoess(object$y, object$x, newx = if (is.null(newdata)) object$x else if (is.data.frame(newdata)) as.matrix(model.frame(delete.response(terms(object)), : pseudoinverse used at -0.005
```

```
## Warning in predLoess(object$y, object$x, newx = if (is.null(newdata)) object$x else if (is.data.frame(newdata)) as.matrix(model.frame(delete.response(terms(object)), : neighborhood radius 1.005
```

```
## Warning in predLoess(object$y, object$x, newx = if (is.null(newdata)) object$x else if (is.data.frame(newdata)) as.matrix(model.frame(delete.response(terms(object)), : reciprocal condition number 0
```

```
## Warning in predLoess(object$y, object$x, newx = if (is.null(newdata)) object$x else if (is.data.frame(newdata)) as.matrix(model.frame(delete.response(terms(object)), : There are other near
## singularities as well. 1.01
```

```
## Warning in simpleLoess(y, x, w, span, degree = degree, parametric = parametric, : pseudoinverse used at -0.005
```

```
## Warning in simpleLoess(y, x, w, span, degree = degree, parametric = parametric, : neighborhood radius 1.005
```

```
## Warning in simpleLoess(y, x, w, span, degree = degree, parametric = parametric, : reciprocal condition number 0
```

```
## Warning in simpleLoess(y, x, w, span, degree = degree, parametric = parametric, : There are other near singularities as well. 1.01
```

```
## Warning in predLoess(object$y, object$x, newx = if (is.null(newdata)) object$x else if (is.data.frame(newdata)) as.matrix(model.frame(delete.response(terms(object)), : pseudoinverse used at -0.005
```

```
## Warning in predLoess(object$y, object$x, newx = if (is.null(newdata)) object$x else if (is.data.frame(newdata)) as.matrix(model.frame(delete.response(terms(object)), : neighborhood radius 1.005
```

```
## Warning in predLoess(object$y, object$x, newx = if (is.null(newdata)) object$x else if (is.data.frame(newdata)) as.matrix(model.frame(delete.response(terms(object)), : reciprocal condition number 0
```

```
## Warning in predLoess(object$y, object$x, newx = if (is.null(newdata)) object$x else if (is.data.frame(newdata)) as.matrix(model.frame(delete.response(terms(object)), : There are other near
## singularities as well. 1.01
```

```
## Warning in simpleLoess(y, x, w, span, degree = degree, parametric = parametric, : pseudoinverse used at -0.005
```

```
## Warning in simpleLoess(y, x, w, span, degree = degree, parametric = parametric, : neighborhood radius 1.005
```

```
## Warning in simpleLoess(y, x, w, span, degree = degree, parametric = parametric, : reciprocal condition number 0
```

```
## Warning in simpleLoess(y, x, w, span, degree = degree, parametric = parametric, : There are other near singularities as well. 1.01
```

```
## Warning in predLoess(object$y, object$x, newx = if (is.null(newdata)) object$x else if (is.data.frame(newdata)) as.matrix(model.frame(delete.response(terms(object)), : pseudoinverse used at -0.005
```

```
## Warning in predLoess(object$y, object$x, newx = if (is.null(newdata)) object$x else if (is.data.frame(newdata)) as.matrix(model.frame(delete.response(terms(object)), : neighborhood radius 1.005
```

```
## Warning in predLoess(object$y, object$x, newx = if (is.null(newdata)) object$x else if (is.data.frame(newdata)) as.matrix(model.frame(delete.response(terms(object)), : reciprocal condition number 0
```

```
## Warning in predLoess(object$y, object$x, newx = if (is.null(newdata)) object$x else if (is.data.frame(newdata)) as.matrix(model.frame(delete.response(terms(object)), : There are other near
## singularities as well. 1.01
```

```
## Warning in simpleLoess(y, x, w, span, degree = degree, parametric = parametric, : at -0.005
```

```
## Warning in simpleLoess(y, x, w, span, degree = degree, parametric = parametric, : radius 2.5e-05
```

```
## Warning in simpleLoess(y, x, w, span, degree = degree, parametric = parametric, : all data on boundary of neighborhood. make span bigger
```

```
## Warning in simpleLoess(y, x, w, span, degree = degree, parametric = parametric, : pseudoinverse used at -0.005
```

```
## Warning in simpleLoess(y, x, w, span, degree = degree, parametric = parametric, : neighborhood radius 0.005
```

```
## Warning in simpleLoess(y, x, w, span, degree = degree, parametric = parametric, : reciprocal condition number 1
```

```
## Warning in simpleLoess(y, x, w, span, degree = degree, parametric = parametric, : There are other near singularities as well. 1.01
```

```
## Warning in simpleLoess(y, x, w, span, degree = degree, parametric = parametric, : zero-width neighborhood. make span bigger
```

```
## Warning: Computation failed in `stat_smooth()`:
## NA/NaN/Inf in foreign function call (arg 5)
```

```
## Warning in simpleLoess(y, x, w, span, degree = degree, parametric = parametric, : pseudoinverse used at -0.005
```

```
## Warning in simpleLoess(y, x, w, span, degree = degree, parametric = parametric, : neighborhood radius 1.005
```

```
## Warning in simpleLoess(y, x, w, span, degree = degree, parametric = parametric, : reciprocal condition number 0
```

```
## Warning in simpleLoess(y, x, w, span, degree = degree, parametric = parametric, : There are other near singularities as well. 1.01
```

```
## Warning in predLoess(object$y, object$x, newx = if (is.null(newdata)) object$x else if (is.data.frame(newdata)) as.matrix(model.frame(delete.response(terms(object)), : pseudoinverse used at -0.005
```

```
## Warning in predLoess(object$y, object$x, newx = if (is.null(newdata)) object$x else if (is.data.frame(newdata)) as.matrix(model.frame(delete.response(terms(object)), : neighborhood radius 1.005
```

```
## Warning in predLoess(object$y, object$x, newx = if (is.null(newdata)) object$x else if (is.data.frame(newdata)) as.matrix(model.frame(delete.response(terms(object)), : reciprocal condition number 0
```

```
## Warning in predLoess(object$y, object$x, newx = if (is.null(newdata)) object$x else if (is.data.frame(newdata)) as.matrix(model.frame(delete.response(terms(object)), : There are other near
## singularities as well. 1.01
```

```
## Warning in simpleLoess(y, x, w, span, degree = degree, parametric = parametric, : at -0.005
```

```
## Warning in simpleLoess(y, x, w, span, degree = degree, parametric = parametric, : radius 2.5e-05
```

```
## Warning in simpleLoess(y, x, w, span, degree = degree, parametric = parametric, : all data on boundary of neighborhood. make span bigger
```

```
## Warning in simpleLoess(y, x, w, span, degree = degree, parametric = parametric, : pseudoinverse used at -0.005
```

```
## Warning in simpleLoess(y, x, w, span, degree = degree, parametric = parametric, : neighborhood radius 0.005
```

```
## Warning in simpleLoess(y, x, w, span, degree = degree, parametric = parametric, : reciprocal condition number 1
```

```
## Warning in simpleLoess(y, x, w, span, degree = degree, parametric = parametric, : There are other near singularities as well. 1.01
```

```
## Warning in simpleLoess(y, x, w, span, degree = degree, parametric = parametric, : zero-width neighborhood. make span bigger
```

```
## Warning: Computation failed in `stat_smooth()`:
## NA/NaN/Inf in foreign function call (arg 5)
```

```
## Warning in simpleLoess(y, x, w, span, degree = degree, parametric = parametric, : at -0.005
```

```
## Warning in simpleLoess(y, x, w, span, degree = degree, parametric = parametric, : radius 2.5e-05
```

```
## Warning in simpleLoess(y, x, w, span, degree = degree, parametric = parametric, : all data on boundary of neighborhood. make span bigger
```

```
## Warning in simpleLoess(y, x, w, span, degree = degree, parametric = parametric, : pseudoinverse used at -0.005
```

```
## Warning in simpleLoess(y, x, w, span, degree = degree, parametric = parametric, : neighborhood radius 0.005
```

```
## Warning in simpleLoess(y, x, w, span, degree = degree, parametric = parametric, : reciprocal condition number 1
```

```
## Warning in simpleLoess(y, x, w, span, degree = degree, parametric = parametric, : There are other near singularities as well. 1.01
```

```
## Warning in simpleLoess(y, x, w, span, degree = degree, parametric = parametric, : zero-width neighborhood. make span bigger
```

```
## Warning: Computation failed in `stat_smooth()`:
## NA/NaN/Inf in foreign function call (arg 5)
```

```
## Warning in simpleLoess(y, x, w, span, degree = degree, parametric = parametric, : at -0.005
```

```
## Warning in simpleLoess(y, x, w, span, degree = degree, parametric = parametric, : radius 2.5e-05
```

```
## Warning in simpleLoess(y, x, w, span, degree = degree, parametric = parametric, : all data on boundary of neighborhood. make span bigger
```

```
## Warning in simpleLoess(y, x, w, span, degree = degree, parametric = parametric, : pseudoinverse used at -0.005
```

```
## Warning in simpleLoess(y, x, w, span, degree = degree, parametric = parametric, : neighborhood radius 0.005
```

```
## Warning in simpleLoess(y, x, w, span, degree = degree, parametric = parametric, : reciprocal condition number 1
```

```
## Warning in simpleLoess(y, x, w, span, degree = degree, parametric = parametric, : There are other near singularities as well. 1.01
```

```
## Warning in simpleLoess(y, x, w, span, degree = degree, parametric = parametric, : zero-width neighborhood. make span bigger
```

```
## Warning: Computation failed in `stat_smooth()`:
## NA/NaN/Inf in foreign function call (arg 5)
```

```
## Warning in simpleLoess(y, x, w, span, degree = degree, parametric = parametric, : pseudoinverse used at -0.005
```

```
## Warning in simpleLoess(y, x, w, span, degree = degree, parametric = parametric, : neighborhood radius 1.005
```

```
## Warning in simpleLoess(y, x, w, span, degree = degree, parametric = parametric, : reciprocal condition number 0
```

```
## Warning in simpleLoess(y, x, w, span, degree = degree, parametric = parametric, : There are other near singularities as well. 1.01
```

```
## Warning in predLoess(object$y, object$x, newx = if (is.null(newdata)) object$x else if (is.data.frame(newdata)) as.matrix(model.frame(delete.response(terms(object)), : pseudoinverse used at -0.005
```

```
## Warning in predLoess(object$y, object$x, newx = if (is.null(newdata)) object$x else if (is.data.frame(newdata)) as.matrix(model.frame(delete.response(terms(object)), : neighborhood radius 1.005
```

```
## Warning in predLoess(object$y, object$x, newx = if (is.null(newdata)) object$x else if (is.data.frame(newdata)) as.matrix(model.frame(delete.response(terms(object)), : reciprocal condition number 0
```

```
## Warning in predLoess(object$y, object$x, newx = if (is.null(newdata)) object$x else if (is.data.frame(newdata)) as.matrix(model.frame(delete.response(terms(object)), : There are other near
## singularities as well. 1.01
```

```
## Warning in simpleLoess(y, x, w, span, degree = degree, parametric = parametric, : pseudoinverse used at -0.005
```

```
## Warning in simpleLoess(y, x, w, span, degree = degree, parametric = parametric, : neighborhood radius 1.005
```

```
## Warning in simpleLoess(y, x, w, span, degree = degree, parametric = parametric, : reciprocal condition number 0
```

```
## Warning in simpleLoess(y, x, w, span, degree = degree, parametric = parametric, : There are other near singularities as well. 1.01
```

```
## Warning in predLoess(object$y, object$x, newx = if (is.null(newdata)) object$x else if (is.data.frame(newdata)) as.matrix(model.frame(delete.response(terms(object)), : pseudoinverse used at -0.005
```

```
## Warning in predLoess(object$y, object$x, newx = if (is.null(newdata)) object$x else if (is.data.frame(newdata)) as.matrix(model.frame(delete.response(terms(object)), : neighborhood radius 1.005
```

```
## Warning in predLoess(object$y, object$x, newx = if (is.null(newdata)) object$x else if (is.data.frame(newdata)) as.matrix(model.frame(delete.response(terms(object)), : reciprocal condition number 0
```

```
## Warning in predLoess(object$y, object$x, newx = if (is.null(newdata)) object$x else if (is.data.frame(newdata)) as.matrix(model.frame(delete.response(terms(object)), : There are other near
## singularities as well. 1.01
```

```
## Warning in simpleLoess(y, x, w, span, degree = degree, parametric = parametric, : at -0.005
```

```
## Warning in simpleLoess(y, x, w, span, degree = degree, parametric = parametric, : radius 2.5e-05
```

```
## Warning in simpleLoess(y, x, w, span, degree = degree, parametric = parametric, : all data on boundary of neighborhood. make span bigger
```

```
## Warning in simpleLoess(y, x, w, span, degree = degree, parametric = parametric, : pseudoinverse used at -0.005
```

```
## Warning in simpleLoess(y, x, w, span, degree = degree, parametric = parametric, : neighborhood radius 0.005
```

```
## Warning in simpleLoess(y, x, w, span, degree = degree, parametric = parametric, : reciprocal condition number 1
```

```
## Warning in simpleLoess(y, x, w, span, degree = degree, parametric = parametric, : There are other near singularities as well. 1.01
```

```
## Warning in simpleLoess(y, x, w, span, degree = degree, parametric = parametric, : zero-width neighborhood. make span bigger
```

```
## Warning: Computation failed in `stat_smooth()`:
## NA/NaN/Inf in foreign function call (arg 5)
```

```
## Warning in simpleLoess(y, x, w, span, degree = degree, parametric = parametric, : at -0.005
```

```
## Warning in simpleLoess(y, x, w, span, degree = degree, parametric = parametric, : radius 2.5e-05
```

```
## Warning in simpleLoess(y, x, w, span, degree = degree, parametric = parametric, : all data on boundary of neighborhood. make span bigger
```

```
## Warning in simpleLoess(y, x, w, span, degree = degree, parametric = parametric, : pseudoinverse used at -0.005
```

```
## Warning in simpleLoess(y, x, w, span, degree = degree, parametric = parametric, : neighborhood radius 0.005
```

```
## Warning in simpleLoess(y, x, w, span, degree = degree, parametric = parametric, : reciprocal condition number 1
```

```
## Warning in simpleLoess(y, x, w, span, degree = degree, parametric = parametric, : There are other near singularities as well. 1.01
```

```
## Warning in simpleLoess(y, x, w, span, degree = degree, parametric = parametric, : zero-width neighborhood. make span bigger
```

```
## Warning: Computation failed in `stat_smooth()`:
## NA/NaN/Inf in foreign function call (arg 5)
```

```
## Warning in simpleLoess(y, x, w, span, degree = degree, parametric = parametric, : at -0.005
```

```
## Warning in simpleLoess(y, x, w, span, degree = degree, parametric = parametric, : radius 2.5e-05
```

```
## Warning in simpleLoess(y, x, w, span, degree = degree, parametric = parametric, : all data on boundary of neighborhood. make span bigger
```

```
## Warning in simpleLoess(y, x, w, span, degree = degree, parametric = parametric, : pseudoinverse used at -0.005
```

```
## Warning in simpleLoess(y, x, w, span, degree = degree, parametric = parametric, : neighborhood radius 0.005
```

```
## Warning in simpleLoess(y, x, w, span, degree = degree, parametric = parametric, : reciprocal condition number 1
```

```
## Warning in simpleLoess(y, x, w, span, degree = degree, parametric = parametric, : There are other near singularities as well. 1.01
```

```
## Warning in simpleLoess(y, x, w, span, degree = degree, parametric = parametric, : zero-width neighborhood. make span bigger
```

```
## Warning: Computation failed in `stat_smooth()`:
## NA/NaN/Inf in foreign function call (arg 5)
```

```
## Warning in simpleLoess(y, x, w, span, degree = degree, parametric = parametric, : at -0.005
```

```
## Warning in simpleLoess(y, x, w, span, degree = degree, parametric = parametric, : radius 2.5e-05
```

```
## Warning in simpleLoess(y, x, w, span, degree = degree, parametric = parametric, : all data on boundary of neighborhood. make span bigger
```

```
## Warning in simpleLoess(y, x, w, span, degree = degree, parametric = parametric, : pseudoinverse used at -0.005
```

```
## Warning in simpleLoess(y, x, w, span, degree = degree, parametric = parametric, : neighborhood radius 0.005
```

```
## Warning in simpleLoess(y, x, w, span, degree = degree, parametric = parametric, : reciprocal condition number 1
```

```
## Warning in simpleLoess(y, x, w, span, degree = degree, parametric = parametric, : There are other near singularities as well. 1.01
```

```
## Warning in simpleLoess(y, x, w, span, degree = degree, parametric = parametric, : zero-width neighborhood. make span bigger
```

```
## Warning: Computation failed in `stat_smooth()`:
## NA/NaN/Inf in foreign function call (arg 5)
```

```
## Warning in simpleLoess(y, x, w, span, degree = degree, parametric = parametric, : pseudoinverse used at -0.005
```

```
## Warning in simpleLoess(y, x, w, span, degree = degree, parametric = parametric, : neighborhood radius 1.005
```

```
## Warning in simpleLoess(y, x, w, span, degree = degree, parametric = parametric, : reciprocal condition number 0
```

```
## Warning in simpleLoess(y, x, w, span, degree = degree, parametric = parametric, : There are other near singularities as well. 1.01
```

```
## Warning in predLoess(object$y, object$x, newx = if (is.null(newdata)) object$x else if (is.data.frame(newdata)) as.matrix(model.frame(delete.response(terms(object)), : pseudoinverse used at -0.005
```

```
## Warning in predLoess(object$y, object$x, newx = if (is.null(newdata)) object$x else if (is.data.frame(newdata)) as.matrix(model.frame(delete.response(terms(object)), : neighborhood radius 1.005
```

```
## Warning in predLoess(object$y, object$x, newx = if (is.null(newdata)) object$x else if (is.data.frame(newdata)) as.matrix(model.frame(delete.response(terms(object)), : reciprocal condition number 0
```

```
## Warning in predLoess(object$y, object$x, newx = if (is.null(newdata)) object$x else if (is.data.frame(newdata)) as.matrix(model.frame(delete.response(terms(object)), : There are other near
## singularities as well. 1.01
```

```
## Warning in simpleLoess(y, x, w, span, degree = degree, parametric = parametric, : at -0.005
```

```
## Warning in simpleLoess(y, x, w, span, degree = degree, parametric = parametric, : radius 2.5e-05
```

```
## Warning in simpleLoess(y, x, w, span, degree = degree, parametric = parametric, : all data on boundary of neighborhood. make span bigger
```

```
## Warning in simpleLoess(y, x, w, span, degree = degree, parametric = parametric, : pseudoinverse used at -0.005
```

```
## Warning in simpleLoess(y, x, w, span, degree = degree, parametric = parametric, : neighborhood radius 0.005
```

```
## Warning in simpleLoess(y, x, w, span, degree = degree, parametric = parametric, : reciprocal condition number 1
```

```
## Warning in simpleLoess(y, x, w, span, degree = degree, parametric = parametric, : There are other near singularities as well. 1.01
```

```
## Warning in simpleLoess(y, x, w, span, degree = degree, parametric = parametric, : zero-width neighborhood. make span bigger
```

```
## Warning: Computation failed in `stat_smooth()`:
## NA/NaN/Inf in foreign function call (arg 5)
```

```
## Warning in simpleLoess(y, x, w, span, degree = degree, parametric = parametric, : pseudoinverse used at -0.005
```

```
## Warning in simpleLoess(y, x, w, span, degree = degree, parametric = parametric, : neighborhood radius 1.005
```

```
## Warning in simpleLoess(y, x, w, span, degree = degree, parametric = parametric, : reciprocal condition number 0
```

```
## Warning in simpleLoess(y, x, w, span, degree = degree, parametric = parametric, : There are other near singularities as well. 1.01
```

```
## Warning in predLoess(object$y, object$x, newx = if (is.null(newdata)) object$x else if (is.data.frame(newdata)) as.matrix(model.frame(delete.response(terms(object)), : pseudoinverse used at -0.005
```

```
## Warning in predLoess(object$y, object$x, newx = if (is.null(newdata)) object$x else if (is.data.frame(newdata)) as.matrix(model.frame(delete.response(terms(object)), : neighborhood radius 1.005
```

```
## Warning in predLoess(object$y, object$x, newx = if (is.null(newdata)) object$x else if (is.data.frame(newdata)) as.matrix(model.frame(delete.response(terms(object)), : reciprocal condition number 0
```

```
## Warning in predLoess(object$y, object$x, newx = if (is.null(newdata)) object$x else if (is.data.frame(newdata)) as.matrix(model.frame(delete.response(terms(object)), : There are other near
## singularities as well. 1.01
```

```
## Warning in simpleLoess(y, x, w, span, degree = degree, parametric = parametric, : pseudoinverse used at -0.005
```

```
## Warning in simpleLoess(y, x, w, span, degree = degree, parametric = parametric, : neighborhood radius 1.005
```

```
## Warning in simpleLoess(y, x, w, span, degree = degree, parametric = parametric, : reciprocal condition number 0
```

```
## Warning in simpleLoess(y, x, w, span, degree = degree, parametric = parametric, : There are other near singularities as well. 1.01
```

```
## Warning in predLoess(object$y, object$x, newx = if (is.null(newdata)) object$x else if (is.data.frame(newdata)) as.matrix(model.frame(delete.response(terms(object)), : pseudoinverse used at -0.005
```

```
## Warning in predLoess(object$y, object$x, newx = if (is.null(newdata)) object$x else if (is.data.frame(newdata)) as.matrix(model.frame(delete.response(terms(object)), : neighborhood radius 1.005
```

```
## Warning in predLoess(object$y, object$x, newx = if (is.null(newdata)) object$x else if (is.data.frame(newdata)) as.matrix(model.frame(delete.response(terms(object)), : reciprocal condition number 0
```

```
## Warning in predLoess(object$y, object$x, newx = if (is.null(newdata)) object$x else if (is.data.frame(newdata)) as.matrix(model.frame(delete.response(terms(object)), : There are other near
## singularities as well. 1.01
```

```
## Warning in simpleLoess(y, x, w, span, degree = degree, parametric = parametric, : at -0.005
```

```
## Warning in simpleLoess(y, x, w, span, degree = degree, parametric = parametric, : radius 2.5e-05
```

```
## Warning in simpleLoess(y, x, w, span, degree = degree, parametric = parametric, : all data on boundary of neighborhood. make span bigger
```

```
## Warning in simpleLoess(y, x, w, span, degree = degree, parametric = parametric, : pseudoinverse used at -0.005
```

```
## Warning in simpleLoess(y, x, w, span, degree = degree, parametric = parametric, : neighborhood radius 0.005
```

```
## Warning in simpleLoess(y, x, w, span, degree = degree, parametric = parametric, : reciprocal condition number 1
```

```
## Warning in simpleLoess(y, x, w, span, degree = degree, parametric = parametric, : There are other near singularities as well. 1.01
```

```
## Warning in simpleLoess(y, x, w, span, degree = degree, parametric = parametric, : zero-width neighborhood. make span bigger
```

```
## Warning: Computation failed in `stat_smooth()`:
## NA/NaN/Inf in foreign function call (arg 5)
```

```
## Warning in simpleLoess(y, x, w, span, degree = degree, parametric = parametric, : at -0.005
```

```
## Warning in simpleLoess(y, x, w, span, degree = degree, parametric = parametric, : radius 2.5e-05
```

```
## Warning in simpleLoess(y, x, w, span, degree = degree, parametric = parametric, : all data on boundary of neighborhood. make span bigger
```

```
## Warning in simpleLoess(y, x, w, span, degree = degree, parametric = parametric, : pseudoinverse used at -0.005
```

```
## Warning in simpleLoess(y, x, w, span, degree = degree, parametric = parametric, : neighborhood radius 0.005
```

```
## Warning in simpleLoess(y, x, w, span, degree = degree, parametric = parametric, : reciprocal condition number 1
```

```
## Warning in simpleLoess(y, x, w, span, degree = degree, parametric = parametric, : There are other near singularities as well. 1.01
```

```
## Warning in simpleLoess(y, x, w, span, degree = degree, parametric = parametric, : zero-width neighborhood. make span bigger
```

```
## Warning: Computation failed in `stat_smooth()`:
## NA/NaN/Inf in foreign function call (arg 5)
```

```
## Warning in simpleLoess(y, x, w, span, degree = degree, parametric = parametric, : pseudoinverse used at -0.005
```

```
## Warning in simpleLoess(y, x, w, span, degree = degree, parametric = parametric, : neighborhood radius 1.005
```

```
## Warning in simpleLoess(y, x, w, span, degree = degree, parametric = parametric, : reciprocal condition number 0
```

```
## Warning in simpleLoess(y, x, w, span, degree = degree, parametric = parametric, : There are other near singularities as well. 1.01
```

```
## Warning in predLoess(object$y, object$x, newx = if (is.null(newdata)) object$x else if (is.data.frame(newdata)) as.matrix(model.frame(delete.response(terms(object)), : pseudoinverse used at -0.005
```

```
## Warning in predLoess(object$y, object$x, newx = if (is.null(newdata)) object$x else if (is.data.frame(newdata)) as.matrix(model.frame(delete.response(terms(object)), : neighborhood radius 1.005
```

```
## Warning in predLoess(object$y, object$x, newx = if (is.null(newdata)) object$x else if (is.data.frame(newdata)) as.matrix(model.frame(delete.response(terms(object)), : reciprocal condition number 0
```

```
## Warning in predLoess(object$y, object$x, newx = if (is.null(newdata)) object$x else if (is.data.frame(newdata)) as.matrix(model.frame(delete.response(terms(object)), : There are other near
## singularities as well. 1.01
```

```
## Warning in simpleLoess(y, x, w, span, degree = degree, parametric = parametric, : at -0.005
```

```
## Warning in simpleLoess(y, x, w, span, degree = degree, parametric = parametric, : radius 2.5e-05
```

```
## Warning in simpleLoess(y, x, w, span, degree = degree, parametric = parametric, : all data on boundary of neighborhood. make span bigger
```

```
## Warning in simpleLoess(y, x, w, span, degree = degree, parametric = parametric, : pseudoinverse used at -0.005
```

```
## Warning in simpleLoess(y, x, w, span, degree = degree, parametric = parametric, : neighborhood radius 0.005
```

```
## Warning in simpleLoess(y, x, w, span, degree = degree, parametric = parametric, : reciprocal condition number 1
```

```
## Warning in simpleLoess(y, x, w, span, degree = degree, parametric = parametric, : There are other near singularities as well. 1.01
```

```
## Warning in simpleLoess(y, x, w, span, degree = degree, parametric = parametric, : zero-width neighborhood. make span bigger
```

```
## Warning: Computation failed in `stat_smooth()`:
## NA/NaN/Inf in foreign function call (arg 5)
```

```
## Warning in simpleLoess(y, x, w, span, degree = degree, parametric = parametric, : at -0.005
```

```
## Warning in simpleLoess(y, x, w, span, degree = degree, parametric = parametric, : radius 2.5e-05
```

```
## Warning in simpleLoess(y, x, w, span, degree = degree, parametric = parametric, : all data on boundary of neighborhood. make span bigger
```

```
## Warning in simpleLoess(y, x, w, span, degree = degree, parametric = parametric, : pseudoinverse used at -0.005
```

```
## Warning in simpleLoess(y, x, w, span, degree = degree, parametric = parametric, : neighborhood radius 0.005
```

```
## Warning in simpleLoess(y, x, w, span, degree = degree, parametric = parametric, : reciprocal condition number 1
```

```
## Warning in simpleLoess(y, x, w, span, degree = degree, parametric = parametric, : There are other near singularities as well. 1.01
```

```
## Warning in simpleLoess(y, x, w, span, degree = degree, parametric = parametric, : zero-width neighborhood. make span bigger
```

```
## Warning: Computation failed in `stat_smooth()`:
## NA/NaN/Inf in foreign function call (arg 5)
```

```
## Warning in simpleLoess(y, x, w, span, degree = degree, parametric = parametric, : at -0.005
```

```
## Warning in simpleLoess(y, x, w, span, degree = degree, parametric = parametric, : radius 2.5e-05
```

```
## Warning in simpleLoess(y, x, w, span, degree = degree, parametric = parametric, : all data on boundary of neighborhood. make span bigger
```

```
## Warning in simpleLoess(y, x, w, span, degree = degree, parametric = parametric, : pseudoinverse used at -0.005
```

```
## Warning in simpleLoess(y, x, w, span, degree = degree, parametric = parametric, : neighborhood radius 0.005
```

```
## Warning in simpleLoess(y, x, w, span, degree = degree, parametric = parametric, : reciprocal condition number 1
```

```
## Warning in simpleLoess(y, x, w, span, degree = degree, parametric = parametric, : There are other near singularities as well. 1.01
```

```
## Warning in simpleLoess(y, x, w, span, degree = degree, parametric = parametric, : zero-width neighborhood. make span bigger
```

```
## Warning: Computation failed in `stat_smooth()`:
## NA/NaN/Inf in foreign function call (arg 5)
```

```
## Warning in simpleLoess(y, x, w, span, degree = degree, parametric = parametric, : at -0.005
```

```
## Warning in simpleLoess(y, x, w, span, degree = degree, parametric = parametric, : radius 2.5e-05
```

```
## Warning in simpleLoess(y, x, w, span, degree = degree, parametric = parametric, : all data on boundary of neighborhood. make span bigger
```

```
## Warning in simpleLoess(y, x, w, span, degree = degree, parametric = parametric, : pseudoinverse used at -0.005
```

```
## Warning in simpleLoess(y, x, w, span, degree = degree, parametric = parametric, : neighborhood radius 0.005
```

```
## Warning in simpleLoess(y, x, w, span, degree = degree, parametric = parametric, : reciprocal condition number 1
```

```
## Warning in simpleLoess(y, x, w, span, degree = degree, parametric = parametric, : There are other near singularities as well. 1.01
```

```
## Warning in simpleLoess(y, x, w, span, degree = degree, parametric = parametric, : zero-width neighborhood. make span bigger
```

```
## Warning: Computation failed in `stat_smooth()`:
## NA/NaN/Inf in foreign function call (arg 5)
```

```
## Warning in simpleLoess(y, x, w, span, degree = degree, parametric = parametric, : at -0.005
```

```
## Warning in simpleLoess(y, x, w, span, degree = degree, parametric = parametric, : radius 2.5e-05
```

```
## Warning in simpleLoess(y, x, w, span, degree = degree, parametric = parametric, : all data on boundary of neighborhood. make span bigger
```

```
## Warning in simpleLoess(y, x, w, span, degree = degree, parametric = parametric, : pseudoinverse used at -0.005
```

```
## Warning in simpleLoess(y, x, w, span, degree = degree, parametric = parametric, : neighborhood radius 0.005
```

```
## Warning in simpleLoess(y, x, w, span, degree = degree, parametric = parametric, : reciprocal condition number 1
```

```
## Warning in simpleLoess(y, x, w, span, degree = degree, parametric = parametric, : There are other near singularities as well. 1.01
```

```
## Warning in simpleLoess(y, x, w, span, degree = degree, parametric = parametric, : zero-width neighborhood. make span bigger
```

```
## Warning: Computation failed in `stat_smooth()`:
## NA/NaN/Inf in foreign function call (arg 5)
```

```
## Warning in simpleLoess(y, x, w, span, degree = degree, parametric = parametric, : at -0.005
```

```
## Warning in simpleLoess(y, x, w, span, degree = degree, parametric = parametric, : radius 2.5e-05
```

```
## Warning in simpleLoess(y, x, w, span, degree = degree, parametric = parametric, : all data on boundary of neighborhood. make span bigger
```

```
## Warning in simpleLoess(y, x, w, span, degree = degree, parametric = parametric, : pseudoinverse used at -0.005
```

```
## Warning in simpleLoess(y, x, w, span, degree = degree, parametric = parametric, : neighborhood radius 0.005
```

```
## Warning in simpleLoess(y, x, w, span, degree = degree, parametric = parametric, : reciprocal condition number 1
```

```
## Warning in simpleLoess(y, x, w, span, degree = degree, parametric = parametric, : There are other near singularities as well. 1.01
```

```
## Warning in simpleLoess(y, x, w, span, degree = degree, parametric = parametric, : zero-width neighborhood. make span bigger
```

```
## Warning: Computation failed in `stat_smooth()`:
## NA/NaN/Inf in foreign function call (arg 5)
```

```
## Warning in simpleLoess(y, x, w, span, degree = degree, parametric = parametric, : at -0.005
```

```
## Warning in simpleLoess(y, x, w, span, degree = degree, parametric = parametric, : radius 2.5e-05
```

```
## Warning in simpleLoess(y, x, w, span, degree = degree, parametric = parametric, : all data on boundary of neighborhood. make span bigger
```

```
## Warning in simpleLoess(y, x, w, span, degree = degree, parametric = parametric, : pseudoinverse used at -0.005
```

```
## Warning in simpleLoess(y, x, w, span, degree = degree, parametric = parametric, : neighborhood radius 0.005
```

```
## Warning in simpleLoess(y, x, w, span, degree = degree, parametric = parametric, : reciprocal condition number 1
```

```
## Warning in simpleLoess(y, x, w, span, degree = degree, parametric = parametric, : There are other near singularities as well. 1.01
```

```
## Warning in simpleLoess(y, x, w, span, degree = degree, parametric = parametric, : zero-width neighborhood. make span bigger
```

```
## Warning: Computation failed in `stat_smooth()`:
## NA/NaN/Inf in foreign function call (arg 5)
```

```
## Warning in simpleLoess(y, x, w, span, degree = degree, parametric = parametric, : at -0.005
```

```
## Warning in simpleLoess(y, x, w, span, degree = degree, parametric = parametric, : radius 2.5e-05
```

```
## Warning in simpleLoess(y, x, w, span, degree = degree, parametric = parametric, : all data on boundary of neighborhood. make span bigger
```

```
## Warning in simpleLoess(y, x, w, span, degree = degree, parametric = parametric, : pseudoinverse used at -0.005
```

```
## Warning in simpleLoess(y, x, w, span, degree = degree, parametric = parametric, : neighborhood radius 0.005
```

```
## Warning in simpleLoess(y, x, w, span, degree = degree, parametric = parametric, : reciprocal condition number 1
```

```
## Warning in simpleLoess(y, x, w, span, degree = degree, parametric = parametric, : There are other near singularities as well. 1.01
```

```
## Warning in simpleLoess(y, x, w, span, degree = degree, parametric = parametric, : zero-width neighborhood. make span bigger
```

```
## Warning: Computation failed in `stat_smooth()`:
## NA/NaN/Inf in foreign function call (arg 5)
```

```
## Warning in simpleLoess(y, x, w, span, degree = degree, parametric = parametric, : at -0.005
```

```
## Warning in simpleLoess(y, x, w, span, degree = degree, parametric = parametric, : radius 2.5e-05
```

```
## Warning in simpleLoess(y, x, w, span, degree = degree, parametric = parametric, : all data on boundary of neighborhood. make span bigger
```

```
## Warning in simpleLoess(y, x, w, span, degree = degree, parametric = parametric, : pseudoinverse used at -0.005
```

```
## Warning in simpleLoess(y, x, w, span, degree = degree, parametric = parametric, : neighborhood radius 0.005
```

```
## Warning in simpleLoess(y, x, w, span, degree = degree, parametric = parametric, : reciprocal condition number 1
```

```
## Warning in simpleLoess(y, x, w, span, degree = degree, parametric = parametric, : There are other near singularities as well. 1.01
```

```
## Warning in simpleLoess(y, x, w, span, degree = degree, parametric = parametric, : zero-width neighborhood. make span bigger
```

```
## Warning: Computation failed in `stat_smooth()`:
## NA/NaN/Inf in foreign function call (arg 5)
```

```
## Warning in simpleLoess(y, x, w, span, degree = degree, parametric = parametric, : at -0.005
```

```
## Warning in simpleLoess(y, x, w, span, degree = degree, parametric = parametric, : radius 2.5e-05
```

```
## Warning in simpleLoess(y, x, w, span, degree = degree, parametric = parametric, : all data on boundary of neighborhood. make span bigger
```

```
## Warning in simpleLoess(y, x, w, span, degree = degree, parametric = parametric, : pseudoinverse used at -0.005
```

```
## Warning in simpleLoess(y, x, w, span, degree = degree, parametric = parametric, : neighborhood radius 0.005
```

```
## Warning in simpleLoess(y, x, w, span, degree = degree, parametric = parametric, : reciprocal condition number 1
```

```
## Warning in simpleLoess(y, x, w, span, degree = degree, parametric = parametric, : There are other near singularities as well. 1.01
```

```
## Warning in simpleLoess(y, x, w, span, degree = degree, parametric = parametric, : zero-width neighborhood. make span bigger
```

```
## Warning: Computation failed in `stat_smooth()`:
## NA/NaN/Inf in foreign function call (arg 5)
```

```
## Warning in simpleLoess(y, x, w, span, degree = degree, parametric = parametric, : at -0.005
```

```
## Warning in simpleLoess(y, x, w, span, degree = degree, parametric = parametric, : radius 2.5e-05
```

```
## Warning in simpleLoess(y, x, w, span, degree = degree, parametric = parametric, : all data on boundary of neighborhood. make span bigger
```

```
## Warning in simpleLoess(y, x, w, span, degree = degree, parametric = parametric, : pseudoinverse used at -0.005
```

```
## Warning in simpleLoess(y, x, w, span, degree = degree, parametric = parametric, : neighborhood radius 0.005
```

```
## Warning in simpleLoess(y, x, w, span, degree = degree, parametric = parametric, : reciprocal condition number 1
```

```
## Warning in simpleLoess(y, x, w, span, degree = degree, parametric = parametric, : There are other near singularities as well. 1.01
```

```
## Warning in simpleLoess(y, x, w, span, degree = degree, parametric = parametric, : zero-width neighborhood. make span bigger
```

```
## Warning: Computation failed in `stat_smooth()`:
## NA/NaN/Inf in foreign function call (arg 5)
```

```
## Warning in simpleLoess(y, x, w, span, degree = degree, parametric = parametric, : pseudoinverse used at -0.005
```

```
## Warning in simpleLoess(y, x, w, span, degree = degree, parametric = parametric, : neighborhood radius 1.005
```

```
## Warning in simpleLoess(y, x, w, span, degree = degree, parametric = parametric, : reciprocal condition number 0
```

```
## Warning in simpleLoess(y, x, w, span, degree = degree, parametric = parametric, : There are other near singularities as well. 1.01
```

```
## Warning in predLoess(object$y, object$x, newx = if (is.null(newdata)) object$x else if (is.data.frame(newdata)) as.matrix(model.frame(delete.response(terms(object)), : pseudoinverse used at -0.005
```

```
## Warning in predLoess(object$y, object$x, newx = if (is.null(newdata)) object$x else if (is.data.frame(newdata)) as.matrix(model.frame(delete.response(terms(object)), : neighborhood radius 1.005
```

```
## Warning in predLoess(object$y, object$x, newx = if (is.null(newdata)) object$x else if (is.data.frame(newdata)) as.matrix(model.frame(delete.response(terms(object)), : reciprocal condition number 0
```

```
## Warning in predLoess(object$y, object$x, newx = if (is.null(newdata)) object$x else if (is.data.frame(newdata)) as.matrix(model.frame(delete.response(terms(object)), : There are other near
## singularities as well. 1.01
```

```
## Warning in simpleLoess(y, x, w, span, degree = degree, parametric = parametric, : at -0.005
```

```
## Warning in simpleLoess(y, x, w, span, degree = degree, parametric = parametric, : radius 2.5e-05
```

```
## Warning in simpleLoess(y, x, w, span, degree = degree, parametric = parametric, : all data on boundary of neighborhood. make span bigger
```

```
## Warning in simpleLoess(y, x, w, span, degree = degree, parametric = parametric, : pseudoinverse used at -0.005
```

```
## Warning in simpleLoess(y, x, w, span, degree = degree, parametric = parametric, : neighborhood radius 0.005
```

```
## Warning in simpleLoess(y, x, w, span, degree = degree, parametric = parametric, : reciprocal condition number 1
```

```
## Warning in simpleLoess(y, x, w, span, degree = degree, parametric = parametric, : There are other near singularities as well. 1.01
```

```
## Warning in simpleLoess(y, x, w, span, degree = degree, parametric = parametric, : zero-width neighborhood. make span bigger
```

```
## Warning: Computation failed in `stat_smooth()`:
## NA/NaN/Inf in foreign function call (arg 5)
```

```
## Warning in simpleLoess(y, x, w, span, degree = degree, parametric = parametric, : at -0.005
```

```
## Warning in simpleLoess(y, x, w, span, degree = degree, parametric = parametric, : radius 2.5e-05
```

```
## Warning in simpleLoess(y, x, w, span, degree = degree, parametric = parametric, : all data on boundary of neighborhood. make span bigger
```

```
## Warning in simpleLoess(y, x, w, span, degree = degree, parametric = parametric, : pseudoinverse used at -0.005
```

```
## Warning in simpleLoess(y, x, w, span, degree = degree, parametric = parametric, : neighborhood radius 0.005
```

```
## Warning in simpleLoess(y, x, w, span, degree = degree, parametric = parametric, : reciprocal condition number 1
```

```
## Warning in simpleLoess(y, x, w, span, degree = degree, parametric = parametric, : There are other near singularities as well. 1.01
```

```
## Warning in simpleLoess(y, x, w, span, degree = degree, parametric = parametric, : zero-width neighborhood. make span bigger
```

```
## Warning: Computation failed in `stat_smooth()`:
## NA/NaN/Inf in foreign function call (arg 5)
```

```
## Warning in simpleLoess(y, x, w, span, degree = degree, parametric = parametric, : pseudoinverse used at -0.005
```

```
## Warning in simpleLoess(y, x, w, span, degree = degree, parametric = parametric, : neighborhood radius 1.005
```

```
## Warning in simpleLoess(y, x, w, span, degree = degree, parametric = parametric, : reciprocal condition number 0
```

```
## Warning in simpleLoess(y, x, w, span, degree = degree, parametric = parametric, : There are other near singularities as well. 1.01
```

```
## Warning in predLoess(object$y, object$x, newx = if (is.null(newdata)) object$x else if (is.data.frame(newdata)) as.matrix(model.frame(delete.response(terms(object)), : pseudoinverse used at -0.005
```

```
## Warning in predLoess(object$y, object$x, newx = if (is.null(newdata)) object$x else if (is.data.frame(newdata)) as.matrix(model.frame(delete.response(terms(object)), : neighborhood radius 1.005
```

```
## Warning in predLoess(object$y, object$x, newx = if (is.null(newdata)) object$x else if (is.data.frame(newdata)) as.matrix(model.frame(delete.response(terms(object)), : reciprocal condition number 0
```

```
## Warning in predLoess(object$y, object$x, newx = if (is.null(newdata)) object$x else if (is.data.frame(newdata)) as.matrix(model.frame(delete.response(terms(object)), : There are other near
## singularities as well. 1.01
```

```
## Warning in simpleLoess(y, x, w, span, degree = degree, parametric = parametric, : at -0.005
```

```
## Warning in simpleLoess(y, x, w, span, degree = degree, parametric = parametric, : radius 2.5e-05
```

```
## Warning in simpleLoess(y, x, w, span, degree = degree, parametric = parametric, : all data on boundary of neighborhood. make span bigger
```

```
## Warning in simpleLoess(y, x, w, span, degree = degree, parametric = parametric, : pseudoinverse used at -0.005
```

```
## Warning in simpleLoess(y, x, w, span, degree = degree, parametric = parametric, : neighborhood radius 0.005
```

```
## Warning in simpleLoess(y, x, w, span, degree = degree, parametric = parametric, : reciprocal condition number 1
```

```
## Warning in simpleLoess(y, x, w, span, degree = degree, parametric = parametric, : There are other near singularities as well. 1.01
```

```
## Warning in simpleLoess(y, x, w, span, degree = degree, parametric = parametric, : zero-width neighborhood. make span bigger
```

```
## Warning: Computation failed in `stat_smooth()`:
## NA/NaN/Inf in foreign function call (arg 5)
```

```
## Warning in simpleLoess(y, x, w, span, degree = degree, parametric = parametric, : at -0.005
```

```
## Warning in simpleLoess(y, x, w, span, degree = degree, parametric = parametric, : radius 2.5e-05
```

```
## Warning in simpleLoess(y, x, w, span, degree = degree, parametric = parametric, : all data on boundary of neighborhood. make span bigger
```

```
## Warning in simpleLoess(y, x, w, span, degree = degree, parametric = parametric, : pseudoinverse used at -0.005
```

```
## Warning in simpleLoess(y, x, w, span, degree = degree, parametric = parametric, : neighborhood radius 0.005
```

```
## Warning in simpleLoess(y, x, w, span, degree = degree, parametric = parametric, : reciprocal condition number 1
```

```
## Warning in simpleLoess(y, x, w, span, degree = degree, parametric = parametric, : There are other near singularities as well. 1.01
```

```
## Warning in simpleLoess(y, x, w, span, degree = degree, parametric = parametric, : zero-width neighborhood. make span bigger
```

```
## Warning: Computation failed in `stat_smooth()`:
## NA/NaN/Inf in foreign function call (arg 5)
```

```
## Warning in simpleLoess(y, x, w, span, degree = degree, parametric = parametric, : at -0.005
```

```
## Warning in simpleLoess(y, x, w, span, degree = degree, parametric = parametric, : radius 2.5e-05
```

```
## Warning in simpleLoess(y, x, w, span, degree = degree, parametric = parametric, : all data on boundary of neighborhood. make span bigger
```

```
## Warning in simpleLoess(y, x, w, span, degree = degree, parametric = parametric, : pseudoinverse used at -0.005
```

```
## Warning in simpleLoess(y, x, w, span, degree = degree, parametric = parametric, : neighborhood radius 0.005
```

```
## Warning in simpleLoess(y, x, w, span, degree = degree, parametric = parametric, : reciprocal condition number 1
```

```
## Warning in simpleLoess(y, x, w, span, degree = degree, parametric = parametric, : There are other near singularities as well. 1.01
```

```
## Warning in simpleLoess(y, x, w, span, degree = degree, parametric = parametric, : zero-width neighborhood. make span bigger
```

```
## Warning: Computation failed in `stat_smooth()`:
## NA/NaN/Inf in foreign function call (arg 5)
```

```
## Warning in simpleLoess(y, x, w, span, degree = degree, parametric = parametric, : pseudoinverse used at -0.005
```

```
## Warning in simpleLoess(y, x, w, span, degree = degree, parametric = parametric, : neighborhood radius 1.005
```

```
## Warning in simpleLoess(y, x, w, span, degree = degree, parametric = parametric, : reciprocal condition number 0
```

```
## Warning in simpleLoess(y, x, w, span, degree = degree, parametric = parametric, : There are other near singularities as well. 1.01
```

```
## Warning in predLoess(object$y, object$x, newx = if (is.null(newdata)) object$x else if (is.data.frame(newdata)) as.matrix(model.frame(delete.response(terms(object)), : pseudoinverse used at -0.005
```

```
## Warning in predLoess(object$y, object$x, newx = if (is.null(newdata)) object$x else if (is.data.frame(newdata)) as.matrix(model.frame(delete.response(terms(object)), : neighborhood radius 1.005
```

```
## Warning in predLoess(object$y, object$x, newx = if (is.null(newdata)) object$x else if (is.data.frame(newdata)) as.matrix(model.frame(delete.response(terms(object)), : reciprocal condition number 0
```

```
## Warning in predLoess(object$y, object$x, newx = if (is.null(newdata)) object$x else if (is.data.frame(newdata)) as.matrix(model.frame(delete.response(terms(object)), : There are other near
## singularities as well. 1.01
```

```
## Warning in simpleLoess(y, x, w, span, degree = degree, parametric = parametric, : at -0.005
```

```
## Warning in simpleLoess(y, x, w, span, degree = degree, parametric = parametric, : radius 2.5e-05
```

```
## Warning in simpleLoess(y, x, w, span, degree = degree, parametric = parametric, : all data on boundary of neighborhood. make span bigger
```

```
## Warning in simpleLoess(y, x, w, span, degree = degree, parametric = parametric, : pseudoinverse used at -0.005
```

```
## Warning in simpleLoess(y, x, w, span, degree = degree, parametric = parametric, : neighborhood radius 0.005
```

```
## Warning in simpleLoess(y, x, w, span, degree = degree, parametric = parametric, : reciprocal condition number 1
```

```
## Warning in simpleLoess(y, x, w, span, degree = degree, parametric = parametric, : There are other near singularities as well. 1.01
```

```
## Warning in simpleLoess(y, x, w, span, degree = degree, parametric = parametric, : zero-width neighborhood. make span bigger
```

```
## Warning: Computation failed in `stat_smooth()`:
## NA/NaN/Inf in foreign function call (arg 5)
```

```
## Warning in simpleLoess(y, x, w, span, degree = degree, parametric = parametric, : at -0.005
```

```
## Warning in simpleLoess(y, x, w, span, degree = degree, parametric = parametric, : radius 2.5e-05
```

```
## Warning in simpleLoess(y, x, w, span, degree = degree, parametric = parametric, : all data on boundary of neighborhood. make span bigger
```

```
## Warning in simpleLoess(y, x, w, span, degree = degree, parametric = parametric, : pseudoinverse used at -0.005
```

```
## Warning in simpleLoess(y, x, w, span, degree = degree, parametric = parametric, : neighborhood radius 0.005
```

```
## Warning in simpleLoess(y, x, w, span, degree = degree, parametric = parametric, : reciprocal condition number 1
```

```
## Warning in simpleLoess(y, x, w, span, degree = degree, parametric = parametric, : There are other near singularities as well. 1.01
```

```
## Warning in simpleLoess(y, x, w, span, degree = degree, parametric = parametric, : zero-width neighborhood. make span bigger
```

```
## Warning: Computation failed in `stat_smooth()`:
## NA/NaN/Inf in foreign function call (arg 5)
```

```
## Warning in simpleLoess(y, x, w, span, degree = degree, parametric = parametric, : at -0.005
```

```
## Warning in simpleLoess(y, x, w, span, degree = degree, parametric = parametric, : radius 2.5e-05
```

```
## Warning in simpleLoess(y, x, w, span, degree = degree, parametric = parametric, : all data on boundary of neighborhood. make span bigger
```

```
## Warning in simpleLoess(y, x, w, span, degree = degree, parametric = parametric, : pseudoinverse used at -0.005
```

```
## Warning in simpleLoess(y, x, w, span, degree = degree, parametric = parametric, : neighborhood radius 0.005
```

```
## Warning in simpleLoess(y, x, w, span, degree = degree, parametric = parametric, : reciprocal condition number 1
```

```
## Warning in simpleLoess(y, x, w, span, degree = degree, parametric = parametric, : There are other near singularities as well. 1.01
```

```
## Warning in simpleLoess(y, x, w, span, degree = degree, parametric = parametric, : zero-width neighborhood. make span bigger
```

```
## Warning: Computation failed in `stat_smooth()`:
## NA/NaN/Inf in foreign function call (arg 5)
```

```
## Warning in simpleLoess(y, x, w, span, degree = degree, parametric = parametric, : at -0.005
```

```
## Warning in simpleLoess(y, x, w, span, degree = degree, parametric = parametric, : radius 2.5e-05
```

```
## Warning in simpleLoess(y, x, w, span, degree = degree, parametric = parametric, : all data on boundary of neighborhood. make span bigger
```

```
## Warning in simpleLoess(y, x, w, span, degree = degree, parametric = parametric, : pseudoinverse used at -0.005
```

```
## Warning in simpleLoess(y, x, w, span, degree = degree, parametric = parametric, : neighborhood radius 0.005
```

```
## Warning in simpleLoess(y, x, w, span, degree = degree, parametric = parametric, : reciprocal condition number 1
```

```
## Warning in simpleLoess(y, x, w, span, degree = degree, parametric = parametric, : There are other near singularities as well. 1.01
```

```
## Warning in simpleLoess(y, x, w, span, degree = degree, parametric = parametric, : zero-width neighborhood. make span bigger
```

```
## Warning: Computation failed in `stat_smooth()`:
## NA/NaN/Inf in foreign function call (arg 5)
```

```
## Warning in simpleLoess(y, x, w, span, degree = degree, parametric = parametric, : at -0.005
```

```
## Warning in simpleLoess(y, x, w, span, degree = degree, parametric = parametric, : radius 2.5e-05
```

```
## Warning in simpleLoess(y, x, w, span, degree = degree, parametric = parametric, : all data on boundary of neighborhood. make span bigger
```

```
## Warning in simpleLoess(y, x, w, span, degree = degree, parametric = parametric, : pseudoinverse used at -0.005
```

```
## Warning in simpleLoess(y, x, w, span, degree = degree, parametric = parametric, : neighborhood radius 0.005
```

```
## Warning in simpleLoess(y, x, w, span, degree = degree, parametric = parametric, : reciprocal condition number 1
```

```
## Warning in simpleLoess(y, x, w, span, degree = degree, parametric = parametric, : There are other near singularities as well. 1.01
```

```
## Warning in simpleLoess(y, x, w, span, degree = degree, parametric = parametric, : zero-width neighborhood. make span bigger
```

```
## Warning: Computation failed in `stat_smooth()`:
## NA/NaN/Inf in foreign function call (arg 5)
```

```
## Warning in simpleLoess(y, x, w, span, degree = degree, parametric = parametric, : at -0.005
```

```
## Warning in simpleLoess(y, x, w, span, degree = degree, parametric = parametric, : radius 2.5e-05
```

```
## Warning in simpleLoess(y, x, w, span, degree = degree, parametric = parametric, : all data on boundary of neighborhood. make span bigger
```

```
## Warning in simpleLoess(y, x, w, span, degree = degree, parametric = parametric, : pseudoinverse used at -0.005
```

```
## Warning in simpleLoess(y, x, w, span, degree = degree, parametric = parametric, : neighborhood radius 0.005
```

```
## Warning in simpleLoess(y, x, w, span, degree = degree, parametric = parametric, : reciprocal condition number 1
```

```
## Warning in simpleLoess(y, x, w, span, degree = degree, parametric = parametric, : There are other near singularities as well. 1.01
```

```
## Warning in simpleLoess(y, x, w, span, degree = degree, parametric = parametric, : zero-width neighborhood. make span bigger
```

```
## Warning: Computation failed in `stat_smooth()`:
## NA/NaN/Inf in foreign function call (arg 5)
```

```
## Warning in simpleLoess(y, x, w, span, degree = degree, parametric = parametric, : pseudoinverse used at -0.005
```

```
## Warning in simpleLoess(y, x, w, span, degree = degree, parametric = parametric, : neighborhood radius 1.005
```

```
## Warning in simpleLoess(y, x, w, span, degree = degree, parametric = parametric, : reciprocal condition number 0
```

```
## Warning in simpleLoess(y, x, w, span, degree = degree, parametric = parametric, : There are other near singularities as well. 1.01
```

```
## Warning in predLoess(object$y, object$x, newx = if (is.null(newdata)) object$x else if (is.data.frame(newdata)) as.matrix(model.frame(delete.response(terms(object)), : pseudoinverse used at -0.005
```

```
## Warning in predLoess(object$y, object$x, newx = if (is.null(newdata)) object$x else if (is.data.frame(newdata)) as.matrix(model.frame(delete.response(terms(object)), : neighborhood radius 1.005
```

```
## Warning in predLoess(object$y, object$x, newx = if (is.null(newdata)) object$x else if (is.data.frame(newdata)) as.matrix(model.frame(delete.response(terms(object)), : reciprocal condition number 0
```

```
## Warning in predLoess(object$y, object$x, newx = if (is.null(newdata)) object$x else if (is.data.frame(newdata)) as.matrix(model.frame(delete.response(terms(object)), : There are other near
## singularities as well. 1.01
```

```
## Warning in simpleLoess(y, x, w, span, degree = degree, parametric = parametric, : at -0.005
```

```
## Warning in simpleLoess(y, x, w, span, degree = degree, parametric = parametric, : radius 2.5e-05
```

```
## Warning in simpleLoess(y, x, w, span, degree = degree, parametric = parametric, : all data on boundary of neighborhood. make span bigger
```

```
## Warning in simpleLoess(y, x, w, span, degree = degree, parametric = parametric, : pseudoinverse used at -0.005
```

```
## Warning in simpleLoess(y, x, w, span, degree = degree, parametric = parametric, : neighborhood radius 0.005
```

```
## Warning in simpleLoess(y, x, w, span, degree = degree, parametric = parametric, : reciprocal condition number 1
```

```
## Warning in simpleLoess(y, x, w, span, degree = degree, parametric = parametric, : There are other near singularities as well. 1.01
```

```
## Warning in simpleLoess(y, x, w, span, degree = degree, parametric = parametric, : zero-width neighborhood. make span bigger
```

```
## Warning: Computation failed in `stat_smooth()`:
## NA/NaN/Inf in foreign function call (arg 5)
```

```
## Warning in simpleLoess(y, x, w, span, degree = degree, parametric = parametric, : at -0.005
```

```
## Warning in simpleLoess(y, x, w, span, degree = degree, parametric = parametric, : radius 2.5e-05
```

```
## Warning in simpleLoess(y, x, w, span, degree = degree, parametric = parametric, : all data on boundary of neighborhood. make span bigger
```

```
## Warning in simpleLoess(y, x, w, span, degree = degree, parametric = parametric, : pseudoinverse used at -0.005
```

```
## Warning in simpleLoess(y, x, w, span, degree = degree, parametric = parametric, : neighborhood radius 0.005
```

```
## Warning in simpleLoess(y, x, w, span, degree = degree, parametric = parametric, : reciprocal condition number 1
```

```
## Warning in simpleLoess(y, x, w, span, degree = degree, parametric = parametric, : There are other near singularities as well. 1.01
```

```
## Warning in simpleLoess(y, x, w, span, degree = degree, parametric = parametric, : zero-width neighborhood. make span bigger
```

```
## Warning: Computation failed in `stat_smooth()`:
## NA/NaN/Inf in foreign function call (arg 5)
```

```
## Warning in simpleLoess(y, x, w, span, degree = degree, parametric = parametric, : at -0.005
```

```
## Warning in simpleLoess(y, x, w, span, degree = degree, parametric = parametric, : radius 2.5e-05
```

```
## Warning in simpleLoess(y, x, w, span, degree = degree, parametric = parametric, : all data on boundary of neighborhood. make span bigger
```

```
## Warning in simpleLoess(y, x, w, span, degree = degree, parametric = parametric, : pseudoinverse used at -0.005
```

```
## Warning in simpleLoess(y, x, w, span, degree = degree, parametric = parametric, : neighborhood radius 0.005
```

```
## Warning in simpleLoess(y, x, w, span, degree = degree, parametric = parametric, : reciprocal condition number 1
```

```
## Warning in simpleLoess(y, x, w, span, degree = degree, parametric = parametric, : There are other near singularities as well. 1.01
```

```
## Warning in simpleLoess(y, x, w, span, degree = degree, parametric = parametric, : zero-width neighborhood. make span bigger
```

```
## Warning: Computation failed in `stat_smooth()`:
## NA/NaN/Inf in foreign function call (arg 5)
```

```
## Warning in simpleLoess(y, x, w, span, degree = degree, parametric = parametric, : at -0.005
```

```
## Warning in simpleLoess(y, x, w, span, degree = degree, parametric = parametric, : radius 2.5e-05
```

```
## Warning in simpleLoess(y, x, w, span, degree = degree, parametric = parametric, : all data on boundary of neighborhood. make span bigger
```

```
## Warning in simpleLoess(y, x, w, span, degree = degree, parametric = parametric, : pseudoinverse used at -0.005
```

```
## Warning in simpleLoess(y, x, w, span, degree = degree, parametric = parametric, : neighborhood radius 0.005
```

```
## Warning in simpleLoess(y, x, w, span, degree = degree, parametric = parametric, : reciprocal condition number 1
```

```
## Warning in simpleLoess(y, x, w, span, degree = degree, parametric = parametric, : There are other near singularities as well. 1.01
```

```
## Warning in simpleLoess(y, x, w, span, degree = degree, parametric = parametric, : zero-width neighborhood. make span bigger
```

```
## Warning: Computation failed in `stat_smooth()`:
## NA/NaN/Inf in foreign function call (arg 5)
```

```
## Warning in simpleLoess(y, x, w, span, degree = degree, parametric = parametric, : at -0.005
```

```
## Warning in simpleLoess(y, x, w, span, degree = degree, parametric = parametric, : radius 2.5e-05
```

```
## Warning in simpleLoess(y, x, w, span, degree = degree, parametric = parametric, : all data on boundary of neighborhood. make span bigger
```

```
## Warning in simpleLoess(y, x, w, span, degree = degree, parametric = parametric, : pseudoinverse used at -0.005
```

```
## Warning in simpleLoess(y, x, w, span, degree = degree, parametric = parametric, : neighborhood radius 0.005
```

```
## Warning in simpleLoess(y, x, w, span, degree = degree, parametric = parametric, : reciprocal condition number 1
```

```
## Warning in simpleLoess(y, x, w, span, degree = degree, parametric = parametric, : There are other near singularities as well. 1.01
```

```
## Warning in simpleLoess(y, x, w, span, degree = degree, parametric = parametric, : zero-width neighborhood. make span bigger
```

```
## Warning: Computation failed in `stat_smooth()`:
## NA/NaN/Inf in foreign function call (arg 5)
```

```
## Warning in simpleLoess(y, x, w, span, degree = degree, parametric = parametric, : at -0.005
```

```
## Warning in simpleLoess(y, x, w, span, degree = degree, parametric = parametric, : radius 2.5e-05
```

```
## Warning in simpleLoess(y, x, w, span, degree = degree, parametric = parametric, : all data on boundary of neighborhood. make span bigger
```

```
## Warning in simpleLoess(y, x, w, span, degree = degree, parametric = parametric, : pseudoinverse used at -0.005
```

```
## Warning in simpleLoess(y, x, w, span, degree = degree, parametric = parametric, : neighborhood radius 0.005
```

```
## Warning in simpleLoess(y, x, w, span, degree = degree, parametric = parametric, : reciprocal condition number 1
```

```
## Warning in simpleLoess(y, x, w, span, degree = degree, parametric = parametric, : There are other near singularities as well. 1.01
```

```
## Warning in simpleLoess(y, x, w, span, degree = degree, parametric = parametric, : zero-width neighborhood. make span bigger
```

```
## Warning: Computation failed in `stat_smooth()`:
## NA/NaN/Inf in foreign function call (arg 5)
```

```
## Warning in simpleLoess(y, x, w, span, degree = degree, parametric = parametric, : at -0.005
```

```
## Warning in simpleLoess(y, x, w, span, degree = degree, parametric = parametric, : radius 2.5e-05
```

```
## Warning in simpleLoess(y, x, w, span, degree = degree, parametric = parametric, : all data on boundary of neighborhood. make span bigger
```

```
## Warning in simpleLoess(y, x, w, span, degree = degree, parametric = parametric, : pseudoinverse used at -0.005
```

```
## Warning in simpleLoess(y, x, w, span, degree = degree, parametric = parametric, : neighborhood radius 0.005
```

```
## Warning in simpleLoess(y, x, w, span, degree = degree, parametric = parametric, : reciprocal condition number 1
```

```
## Warning in simpleLoess(y, x, w, span, degree = degree, parametric = parametric, : There are other near singularities as well. 1.01
```

```
## Warning in simpleLoess(y, x, w, span, degree = degree, parametric = parametric, : zero-width neighborhood. make span bigger
```

```
## Warning: Computation failed in `stat_smooth()`:
## NA/NaN/Inf in foreign function call (arg 5)
```

```
## Warning in simpleLoess(y, x, w, span, degree = degree, parametric = parametric, : pseudoinverse used at -0.005
```

```
## Warning in simpleLoess(y, x, w, span, degree = degree, parametric = parametric, : neighborhood radius 1.005
```

```
## Warning in simpleLoess(y, x, w, span, degree = degree, parametric = parametric, : reciprocal condition number 0
```

```
## Warning in simpleLoess(y, x, w, span, degree = degree, parametric = parametric, : There are other near singularities as well. 1.01
```

```
## Warning in predLoess(object$y, object$x, newx = if (is.null(newdata)) object$x else if (is.data.frame(newdata)) as.matrix(model.frame(delete.response(terms(object)), : pseudoinverse used at -0.005
```

```
## Warning in predLoess(object$y, object$x, newx = if (is.null(newdata)) object$x else if (is.data.frame(newdata)) as.matrix(model.frame(delete.response(terms(object)), : neighborhood radius 1.005
```

```
## Warning in predLoess(object$y, object$x, newx = if (is.null(newdata)) object$x else if (is.data.frame(newdata)) as.matrix(model.frame(delete.response(terms(object)), : reciprocal condition number 0
```

```
## Warning in predLoess(object$y, object$x, newx = if (is.null(newdata)) object$x else if (is.data.frame(newdata)) as.matrix(model.frame(delete.response(terms(object)), : There are other near
## singularities as well. 1.01
```

```
## Warning in simpleLoess(y, x, w, span, degree = degree, parametric = parametric, : at -0.005
```

```
## Warning in simpleLoess(y, x, w, span, degree = degree, parametric = parametric, : radius 2.5e-05
```

```
## Warning in simpleLoess(y, x, w, span, degree = degree, parametric = parametric, : all data on boundary of neighborhood. make span bigger
```

```
## Warning in simpleLoess(y, x, w, span, degree = degree, parametric = parametric, : pseudoinverse used at -0.005
```

```
## Warning in simpleLoess(y, x, w, span, degree = degree, parametric = parametric, : neighborhood radius 0.005
```

```
## Warning in simpleLoess(y, x, w, span, degree = degree, parametric = parametric, : reciprocal condition number 1
```

```
## Warning in simpleLoess(y, x, w, span, degree = degree, parametric = parametric, : There are other near singularities as well. 1.01
```

```
## Warning in simpleLoess(y, x, w, span, degree = degree, parametric = parametric, : zero-width neighborhood. make span bigger
```

```
## Warning: Computation failed in `stat_smooth()`:
## NA/NaN/Inf in foreign function call (arg 5)
```

```
## Warning in simpleLoess(y, x, w, span, degree = degree, parametric = parametric, : at -0.005
```

```
## Warning in simpleLoess(y, x, w, span, degree = degree, parametric = parametric, : radius 2.5e-05
```

```
## Warning in simpleLoess(y, x, w, span, degree = degree, parametric = parametric, : all data on boundary of neighborhood. make span bigger
```

```
## Warning in simpleLoess(y, x, w, span, degree = degree, parametric = parametric, : pseudoinverse used at -0.005
```

```
## Warning in simpleLoess(y, x, w, span, degree = degree, parametric = parametric, : neighborhood radius 0.005
```

```
## Warning in simpleLoess(y, x, w, span, degree = degree, parametric = parametric, : reciprocal condition number 1
```

```
## Warning in simpleLoess(y, x, w, span, degree = degree, parametric = parametric, : There are other near singularities as well. 1.01
```

```
## Warning in simpleLoess(y, x, w, span, degree = degree, parametric = parametric, : zero-width neighborhood. make span bigger
```

```
## Warning: Computation failed in `stat_smooth()`:
## NA/NaN/Inf in foreign function call (arg 5)
```

```
## Warning in simpleLoess(y, x, w, span, degree = degree, parametric = parametric, : at -0.005
```

```
## Warning in simpleLoess(y, x, w, span, degree = degree, parametric = parametric, : radius 2.5e-05
```

```
## Warning in simpleLoess(y, x, w, span, degree = degree, parametric = parametric, : all data on boundary of neighborhood. make span bigger
```

```
## Warning in simpleLoess(y, x, w, span, degree = degree, parametric = parametric, : pseudoinverse used at -0.005
```

```
## Warning in simpleLoess(y, x, w, span, degree = degree, parametric = parametric, : neighborhood radius 0.005
```

```
## Warning in simpleLoess(y, x, w, span, degree = degree, parametric = parametric, : reciprocal condition number 1
```

```
## Warning in simpleLoess(y, x, w, span, degree = degree, parametric = parametric, : There are other near singularities as well. 1.01
```

```
## Warning in simpleLoess(y, x, w, span, degree = degree, parametric = parametric, : zero-width neighborhood. make span bigger
```

```
## Warning: Computation failed in `stat_smooth()`:
## NA/NaN/Inf in foreign function call (arg 5)
```

```
## Warning in simpleLoess(y, x, w, span, degree = degree, parametric = parametric, : at -0.005
```

```
## Warning in simpleLoess(y, x, w, span, degree = degree, parametric = parametric, : radius 2.5e-05
```

```
## Warning in simpleLoess(y, x, w, span, degree = degree, parametric = parametric, : all data on boundary of neighborhood. make span bigger
```

```
## Warning in simpleLoess(y, x, w, span, degree = degree, parametric = parametric, : pseudoinverse used at -0.005
```

```
## Warning in simpleLoess(y, x, w, span, degree = degree, parametric = parametric, : neighborhood radius 0.005
```

```
## Warning in simpleLoess(y, x, w, span, degree = degree, parametric = parametric, : reciprocal condition number 1
```

```
## Warning in simpleLoess(y, x, w, span, degree = degree, parametric = parametric, : There are other near singularities as well. 1.01
```

```
## Warning in simpleLoess(y, x, w, span, degree = degree, parametric = parametric, : zero-width neighborhood. make span bigger
```

```
## Warning: Computation failed in `stat_smooth()`:
## NA/NaN/Inf in foreign function call (arg 5)
```

```
## Warning in simpleLoess(y, x, w, span, degree = degree, parametric = parametric, : at -0.005
```

```
## Warning in simpleLoess(y, x, w, span, degree = degree, parametric = parametric, : radius 2.5e-05
```

```
## Warning in simpleLoess(y, x, w, span, degree = degree, parametric = parametric, : all data on boundary of neighborhood. make span bigger
```

```
## Warning in simpleLoess(y, x, w, span, degree = degree, parametric = parametric, : pseudoinverse used at -0.005
```

```
## Warning in simpleLoess(y, x, w, span, degree = degree, parametric = parametric, : neighborhood radius 0.005
```

```
## Warning in simpleLoess(y, x, w, span, degree = degree, parametric = parametric, : reciprocal condition number 1
```

```
## Warning in simpleLoess(y, x, w, span, degree = degree, parametric = parametric, : There are other near singularities as well. 1.01
```

```
## Warning in simpleLoess(y, x, w, span, degree = degree, parametric = parametric, : zero-width neighborhood. make span bigger
```

```
## Warning: Computation failed in `stat_smooth()`:
## NA/NaN/Inf in foreign function call (arg 5)
```

```
## Warning in simpleLoess(y, x, w, span, degree = degree, parametric = parametric, : at -0.005
```

```
## Warning in simpleLoess(y, x, w, span, degree = degree, parametric = parametric, : radius 2.5e-05
```

```
## Warning in simpleLoess(y, x, w, span, degree = degree, parametric = parametric, : all data on boundary of neighborhood. make span bigger
```

```
## Warning in simpleLoess(y, x, w, span, degree = degree, parametric = parametric, : pseudoinverse used at -0.005
```

```
## Warning in simpleLoess(y, x, w, span, degree = degree, parametric = parametric, : neighborhood radius 0.005
```

```
## Warning in simpleLoess(y, x, w, span, degree = degree, parametric = parametric, : reciprocal condition number 1
```

```
## Warning in simpleLoess(y, x, w, span, degree = degree, parametric = parametric, : There are other near singularities as well. 1.01
```

```
## Warning in simpleLoess(y, x, w, span, degree = degree, parametric = parametric, : zero-width neighborhood. make span bigger
```

```
## Warning: Computation failed in `stat_smooth()`:
## NA/NaN/Inf in foreign function call (arg 5)
```

```
## Warning in simpleLoess(y, x, w, span, degree = degree, parametric = parametric, : at -0.005
```

```
## Warning in simpleLoess(y, x, w, span, degree = degree, parametric = parametric, : radius 2.5e-05
```

```
## Warning in simpleLoess(y, x, w, span, degree = degree, parametric = parametric, : all data on boundary of neighborhood. make span bigger
```

```
## Warning in simpleLoess(y, x, w, span, degree = degree, parametric = parametric, : pseudoinverse used at -0.005
```

```
## Warning in simpleLoess(y, x, w, span, degree = degree, parametric = parametric, : neighborhood radius 0.005
```

```
## Warning in simpleLoess(y, x, w, span, degree = degree, parametric = parametric, : reciprocal condition number 1
```

```
## Warning in simpleLoess(y, x, w, span, degree = degree, parametric = parametric, : There are other near singularities as well. 1.01
```

```
## Warning in simpleLoess(y, x, w, span, degree = degree, parametric = parametric, : zero-width neighborhood. make span bigger
```

```
## Warning: Computation failed in `stat_smooth()`:
## NA/NaN/Inf in foreign function call (arg 5)
```

```
## Warning in simpleLoess(y, x, w, span, degree = degree, parametric = parametric, : at -0.005
```

```
## Warning in simpleLoess(y, x, w, span, degree = degree, parametric = parametric, : radius 2.5e-05
```

```
## Warning in simpleLoess(y, x, w, span, degree = degree, parametric = parametric, : all data on boundary of neighborhood. make span bigger
```

```
## Warning in simpleLoess(y, x, w, span, degree = degree, parametric = parametric, : pseudoinverse used at -0.005
```

```
## Warning in simpleLoess(y, x, w, span, degree = degree, parametric = parametric, : neighborhood radius 0.005
```

```
## Warning in simpleLoess(y, x, w, span, degree = degree, parametric = parametric, : reciprocal condition number 1
```

```
## Warning in simpleLoess(y, x, w, span, degree = degree, parametric = parametric, : There are other near singularities as well. 1.01
```

```
## Warning in simpleLoess(y, x, w, span, degree = degree, parametric = parametric, : zero-width neighborhood. make span bigger
```

```
## Warning: Computation failed in `stat_smooth()`:
## NA/NaN/Inf in foreign function call (arg 5)
```

```
## Warning in simpleLoess(y, x, w, span, degree = degree, parametric = parametric, : at -0.005
```

```
## Warning in simpleLoess(y, x, w, span, degree = degree, parametric = parametric, : radius 2.5e-05
```

```
## Warning in simpleLoess(y, x, w, span, degree = degree, parametric = parametric, : all data on boundary of neighborhood. make span bigger
```

```
## Warning in simpleLoess(y, x, w, span, degree = degree, parametric = parametric, : pseudoinverse used at -0.005
```

```
## Warning in simpleLoess(y, x, w, span, degree = degree, parametric = parametric, : neighborhood radius 0.005
```

```
## Warning in simpleLoess(y, x, w, span, degree = degree, parametric = parametric, : reciprocal condition number 1
```

```
## Warning in simpleLoess(y, x, w, span, degree = degree, parametric = parametric, : There are other near singularities as well. 1.01
```

```
## Warning in simpleLoess(y, x, w, span, degree = degree, parametric = parametric, : zero-width neighborhood. make span bigger
```

```
## Warning: Computation failed in `stat_smooth()`:
## NA/NaN/Inf in foreign function call (arg 5)
```

```
## Warning in simpleLoess(y, x, w, span, degree = degree, parametric = parametric, : pseudoinverse used at -0.005
```

```
## Warning in simpleLoess(y, x, w, span, degree = degree, parametric = parametric, : neighborhood radius 1.005
```

```
## Warning in simpleLoess(y, x, w, span, degree = degree, parametric = parametric, : reciprocal condition number 0
```

```
## Warning in simpleLoess(y, x, w, span, degree = degree, parametric = parametric, : There are other near singularities as well. 1.01
```

```
## Warning in predLoess(object$y, object$x, newx = if (is.null(newdata)) object$x else if (is.data.frame(newdata)) as.matrix(model.frame(delete.response(terms(object)), : pseudoinverse used at -0.005
```

```
## Warning in predLoess(object$y, object$x, newx = if (is.null(newdata)) object$x else if (is.data.frame(newdata)) as.matrix(model.frame(delete.response(terms(object)), : neighborhood radius 1.005
```

```
## Warning in predLoess(object$y, object$x, newx = if (is.null(newdata)) object$x else if (is.data.frame(newdata)) as.matrix(model.frame(delete.response(terms(object)), : reciprocal condition number 0
```

```
## Warning in predLoess(object$y, object$x, newx = if (is.null(newdata)) object$x else if (is.data.frame(newdata)) as.matrix(model.frame(delete.response(terms(object)), : There are other near
## singularities as well. 1.01
```

```
## Warning in simpleLoess(y, x, w, span, degree = degree, parametric = parametric, : at -0.005
```

```
## Warning in simpleLoess(y, x, w, span, degree = degree, parametric = parametric, : radius 2.5e-05
```

```
## Warning in simpleLoess(y, x, w, span, degree = degree, parametric = parametric, : all data on boundary of neighborhood. make span bigger
```

```
## Warning in simpleLoess(y, x, w, span, degree = degree, parametric = parametric, : pseudoinverse used at -0.005
```

```
## Warning in simpleLoess(y, x, w, span, degree = degree, parametric = parametric, : neighborhood radius 0.005
```

```
## Warning in simpleLoess(y, x, w, span, degree = degree, parametric = parametric, : reciprocal condition number 1
```

```
## Warning in simpleLoess(y, x, w, span, degree = degree, parametric = parametric, : There are other near singularities as well. 1.01
```

```
## Warning in simpleLoess(y, x, w, span, degree = degree, parametric = parametric, : zero-width neighborhood. make span bigger
```

```
## Warning: Computation failed in `stat_smooth()`:
## NA/NaN/Inf in foreign function call (arg 5)
```

```
## Warning in simpleLoess(y, x, w, span, degree = degree, parametric = parametric, : at -0.005
```

```
## Warning in simpleLoess(y, x, w, span, degree = degree, parametric = parametric, : radius 2.5e-05
```

```
## Warning in simpleLoess(y, x, w, span, degree = degree, parametric = parametric, : all data on boundary of neighborhood. make span bigger
```

```
## Warning in simpleLoess(y, x, w, span, degree = degree, parametric = parametric, : pseudoinverse used at -0.005
```

```
## Warning in simpleLoess(y, x, w, span, degree = degree, parametric = parametric, : neighborhood radius 0.005
```

```
## Warning in simpleLoess(y, x, w, span, degree = degree, parametric = parametric, : reciprocal condition number 1
```

```
## Warning in simpleLoess(y, x, w, span, degree = degree, parametric = parametric, : There are other near singularities as well. 1.01
```

```
## Warning in simpleLoess(y, x, w, span, degree = degree, parametric = parametric, : zero-width neighborhood. make span bigger
```

```
## Warning: Computation failed in `stat_smooth()`:
## NA/NaN/Inf in foreign function call (arg 5)
```

```
## Warning in simpleLoess(y, x, w, span, degree = degree, parametric = parametric, : at -0.005
```

```
## Warning in simpleLoess(y, x, w, span, degree = degree, parametric = parametric, : radius 2.5e-05
```

```
## Warning in simpleLoess(y, x, w, span, degree = degree, parametric = parametric, : all data on boundary of neighborhood. make span bigger
```

```
## Warning in simpleLoess(y, x, w, span, degree = degree, parametric = parametric, : pseudoinverse used at -0.005
```

```
## Warning in simpleLoess(y, x, w, span, degree = degree, parametric = parametric, : neighborhood radius 0.005
```

```
## Warning in simpleLoess(y, x, w, span, degree = degree, parametric = parametric, : reciprocal condition number 1
```

```
## Warning in simpleLoess(y, x, w, span, degree = degree, parametric = parametric, : There are other near singularities as well. 1.01
```

```
## Warning in simpleLoess(y, x, w, span, degree = degree, parametric = parametric, : zero-width neighborhood. make span bigger
```

```
## Warning: Computation failed in `stat_smooth()`:
## NA/NaN/Inf in foreign function call (arg 5)
```

```
## Warning in simpleLoess(y, x, w, span, degree = degree, parametric = parametric, : pseudoinverse used at -0.005
```

```
## Warning in simpleLoess(y, x, w, span, degree = degree, parametric = parametric, : neighborhood radius 1.005
```

```
## Warning in simpleLoess(y, x, w, span, degree = degree, parametric = parametric, : reciprocal condition number 0
```

```
## Warning in simpleLoess(y, x, w, span, degree = degree, parametric = parametric, : There are other near singularities as well. 1.01
```

```
## Warning in predLoess(object$y, object$x, newx = if (is.null(newdata)) object$x else if (is.data.frame(newdata)) as.matrix(model.frame(delete.response(terms(object)), : pseudoinverse used at -0.005
```

```
## Warning in predLoess(object$y, object$x, newx = if (is.null(newdata)) object$x else if (is.data.frame(newdata)) as.matrix(model.frame(delete.response(terms(object)), : neighborhood radius 1.005
```

```
## Warning in predLoess(object$y, object$x, newx = if (is.null(newdata)) object$x else if (is.data.frame(newdata)) as.matrix(model.frame(delete.response(terms(object)), : reciprocal condition number 0
```

```
## Warning in predLoess(object$y, object$x, newx = if (is.null(newdata)) object$x else if (is.data.frame(newdata)) as.matrix(model.frame(delete.response(terms(object)), : There are other near
## singularities as well. 1.01
```

```
## Warning in simpleLoess(y, x, w, span, degree = degree, parametric = parametric, : at -0.005
```

```
## Warning in simpleLoess(y, x, w, span, degree = degree, parametric = parametric, : radius 2.5e-05
```

```
## Warning in simpleLoess(y, x, w, span, degree = degree, parametric = parametric, : all data on boundary of neighborhood. make span bigger
```

```
## Warning in simpleLoess(y, x, w, span, degree = degree, parametric = parametric, : pseudoinverse used at -0.005
```

```
## Warning in simpleLoess(y, x, w, span, degree = degree, parametric = parametric, : neighborhood radius 0.005
```

```
## Warning in simpleLoess(y, x, w, span, degree = degree, parametric = parametric, : reciprocal condition number 1
```

```
## Warning in simpleLoess(y, x, w, span, degree = degree, parametric = parametric, : There are other near singularities as well. 1.01
```

```
## Warning in simpleLoess(y, x, w, span, degree = degree, parametric = parametric, : zero-width neighborhood. make span bigger
```

```
## Warning: Computation failed in `stat_smooth()`:
## NA/NaN/Inf in foreign function call (arg 5)
```

```
## Warning in simpleLoess(y, x, w, span, degree = degree, parametric = parametric, : at -0.005
```

```
## Warning in simpleLoess(y, x, w, span, degree = degree, parametric = parametric, : radius 2.5e-05
```

```
## Warning in simpleLoess(y, x, w, span, degree = degree, parametric = parametric, : all data on boundary of neighborhood. make span bigger
```

```
## Warning in simpleLoess(y, x, w, span, degree = degree, parametric = parametric, : pseudoinverse used at -0.005
```

```
## Warning in simpleLoess(y, x, w, span, degree = degree, parametric = parametric, : neighborhood radius 0.005
```

```
## Warning in simpleLoess(y, x, w, span, degree = degree, parametric = parametric, : reciprocal condition number 1
```

```
## Warning in simpleLoess(y, x, w, span, degree = degree, parametric = parametric, : There are other near singularities as well. 1.01
```

```
## Warning in simpleLoess(y, x, w, span, degree = degree, parametric = parametric, : zero-width neighborhood. make span bigger
```

```
## Warning: Computation failed in `stat_smooth()`:
## NA/NaN/Inf in foreign function call (arg 5)
```

```
## Warning in simpleLoess(y, x, w, span, degree = degree, parametric = parametric, : at -0.005
```

```
## Warning in simpleLoess(y, x, w, span, degree = degree, parametric = parametric, : radius 2.5e-05
```

```
## Warning in simpleLoess(y, x, w, span, degree = degree, parametric = parametric, : all data on boundary of neighborhood. make span bigger
```

```
## Warning in simpleLoess(y, x, w, span, degree = degree, parametric = parametric, : pseudoinverse used at -0.005
```

```
## Warning in simpleLoess(y, x, w, span, degree = degree, parametric = parametric, : neighborhood radius 0.005
```

```
## Warning in simpleLoess(y, x, w, span, degree = degree, parametric = parametric, : reciprocal condition number 1
```

```
## Warning in simpleLoess(y, x, w, span, degree = degree, parametric = parametric, : There are other near singularities as well. 1.01
```

```
## Warning in simpleLoess(y, x, w, span, degree = degree, parametric = parametric, : zero-width neighborhood. make span bigger
```

```
## Warning: Computation failed in `stat_smooth()`:
## NA/NaN/Inf in foreign function call (arg 5)
```

```
## Warning in simpleLoess(y, x, w, span, degree = degree, parametric = parametric, : at -0.005
```

```
## Warning in simpleLoess(y, x, w, span, degree = degree, parametric = parametric, : radius 2.5e-05
```

```
## Warning in simpleLoess(y, x, w, span, degree = degree, parametric = parametric, : all data on boundary of neighborhood. make span bigger
```

```
## Warning in simpleLoess(y, x, w, span, degree = degree, parametric = parametric, : pseudoinverse used at -0.005
```

```
## Warning in simpleLoess(y, x, w, span, degree = degree, parametric = parametric, : neighborhood radius 0.005
```

```
## Warning in simpleLoess(y, x, w, span, degree = degree, parametric = parametric, : reciprocal condition number 1
```

```
## Warning in simpleLoess(y, x, w, span, degree = degree, parametric = parametric, : There are other near singularities as well. 1.01
```

```
## Warning in simpleLoess(y, x, w, span, degree = degree, parametric = parametric, : zero-width neighborhood. make span bigger
```

```
## Warning: Computation failed in `stat_smooth()`:
## NA/NaN/Inf in foreign function call (arg 5)
```

```
## Warning in simpleLoess(y, x, w, span, degree = degree, parametric = parametric, : at -0.005
```

```
## Warning in simpleLoess(y, x, w, span, degree = degree, parametric = parametric, : radius 2.5e-05
```

```
## Warning in simpleLoess(y, x, w, span, degree = degree, parametric = parametric, : all data on boundary of neighborhood. make span bigger
```

```
## Warning in simpleLoess(y, x, w, span, degree = degree, parametric = parametric, : pseudoinverse used at -0.005
```

```
## Warning in simpleLoess(y, x, w, span, degree = degree, parametric = parametric, : neighborhood radius 0.005
```

```
## Warning in simpleLoess(y, x, w, span, degree = degree, parametric = parametric, : reciprocal condition number 1
```

```
## Warning in simpleLoess(y, x, w, span, degree = degree, parametric = parametric, : There are other near singularities as well. 1.01
```

```
## Warning in simpleLoess(y, x, w, span, degree = degree, parametric = parametric, : zero-width neighborhood. make span bigger
```

```
## Warning: Computation failed in `stat_smooth()`:
## NA/NaN/Inf in foreign function call (arg 5)
```

```
## Warning in simpleLoess(y, x, w, span, degree = degree, parametric = parametric, : at -0.005
```

```
## Warning in simpleLoess(y, x, w, span, degree = degree, parametric = parametric, : radius 2.5e-05
```

```
## Warning in simpleLoess(y, x, w, span, degree = degree, parametric = parametric, : all data on boundary of neighborhood. make span bigger
```

```
## Warning in simpleLoess(y, x, w, span, degree = degree, parametric = parametric, : pseudoinverse used at -0.005
```

```
## Warning in simpleLoess(y, x, w, span, degree = degree, parametric = parametric, : neighborhood radius 0.005
```

```
## Warning in simpleLoess(y, x, w, span, degree = degree, parametric = parametric, : reciprocal condition number 1
```

```
## Warning in simpleLoess(y, x, w, span, degree = degree, parametric = parametric, : There are other near singularities as well. 1.01
```

```
## Warning in simpleLoess(y, x, w, span, degree = degree, parametric = parametric, : zero-width neighborhood. make span bigger
```

```
## Warning: Computation failed in `stat_smooth()`:
## NA/NaN/Inf in foreign function call (arg 5)
```

```
## Warning in simpleLoess(y, x, w, span, degree = degree, parametric = parametric, : at -0.005
```

```
## Warning in simpleLoess(y, x, w, span, degree = degree, parametric = parametric, : radius 2.5e-05
```

```
## Warning in simpleLoess(y, x, w, span, degree = degree, parametric = parametric, : all data on boundary of neighborhood. make span bigger
```

```
## Warning in simpleLoess(y, x, w, span, degree = degree, parametric = parametric, : pseudoinverse used at -0.005
```

```
## Warning in simpleLoess(y, x, w, span, degree = degree, parametric = parametric, : neighborhood radius 0.005
```

```
## Warning in simpleLoess(y, x, w, span, degree = degree, parametric = parametric, : reciprocal condition number 1
```

```
## Warning in simpleLoess(y, x, w, span, degree = degree, parametric = parametric, : There are other near singularities as well. 1.01
```

```
## Warning in simpleLoess(y, x, w, span, degree = degree, parametric = parametric, : zero-width neighborhood. make span bigger
```

```
## Warning: Computation failed in `stat_smooth()`:
## NA/NaN/Inf in foreign function call (arg 5)
```

```
## Warning in simpleLoess(y, x, w, span, degree = degree, parametric = parametric, : at -0.005
```

```
## Warning in simpleLoess(y, x, w, span, degree = degree, parametric = parametric, : radius 2.5e-05
```

```
## Warning in simpleLoess(y, x, w, span, degree = degree, parametric = parametric, : all data on boundary of neighborhood. make span bigger
```

```
## Warning in simpleLoess(y, x, w, span, degree = degree, parametric = parametric, : pseudoinverse used at -0.005
```

```
## Warning in simpleLoess(y, x, w, span, degree = degree, parametric = parametric, : neighborhood radius 0.005
```

```
## Warning in simpleLoess(y, x, w, span, degree = degree, parametric = parametric, : reciprocal condition number 1
```

```
## Warning in simpleLoess(y, x, w, span, degree = degree, parametric = parametric, : There are other near singularities as well. 1.01
```

```
## Warning in simpleLoess(y, x, w, span, degree = degree, parametric = parametric, : zero-width neighborhood. make span bigger
```

```
## Warning: Computation failed in `stat_smooth()`:
## NA/NaN/Inf in foreign function call (arg 5)
```

```
## Warning in simpleLoess(y, x, w, span, degree = degree, parametric = parametric, : at -0.005
```

```
## Warning in simpleLoess(y, x, w, span, degree = degree, parametric = parametric, : radius 2.5e-05
```

```
## Warning in simpleLoess(y, x, w, span, degree = degree, parametric = parametric, : all data on boundary of neighborhood. make span bigger
```

```
## Warning in simpleLoess(y, x, w, span, degree = degree, parametric = parametric, : pseudoinverse used at -0.005
```

```
## Warning in simpleLoess(y, x, w, span, degree = degree, parametric = parametric, : neighborhood radius 0.005
```

```
## Warning in simpleLoess(y, x, w, span, degree = degree, parametric = parametric, : reciprocal condition number 1
```

```
## Warning in simpleLoess(y, x, w, span, degree = degree, parametric = parametric, : There are other near singularities as well. 1.01
```

```
## Warning in simpleLoess(y, x, w, span, degree = degree, parametric = parametric, : zero-width neighborhood. make span bigger
```

```
## Warning: Computation failed in `stat_smooth()`:
## NA/NaN/Inf in foreign function call (arg 5)
```

```
## Warning in simpleLoess(y, x, w, span, degree = degree, parametric = parametric, : at -0.005
```

```
## Warning in simpleLoess(y, x, w, span, degree = degree, parametric = parametric, : radius 2.5e-05
```

```
## Warning in simpleLoess(y, x, w, span, degree = degree, parametric = parametric, : all data on boundary of neighborhood. make span bigger
```

```
## Warning in simpleLoess(y, x, w, span, degree = degree, parametric = parametric, : pseudoinverse used at -0.005
```

```
## Warning in simpleLoess(y, x, w, span, degree = degree, parametric = parametric, : neighborhood radius 0.005
```

```
## Warning in simpleLoess(y, x, w, span, degree = degree, parametric = parametric, : reciprocal condition number 1
```

```
## Warning in simpleLoess(y, x, w, span, degree = degree, parametric = parametric, : There are other near singularities as well. 1.01
```

```
## Warning in simpleLoess(y, x, w, span, degree = degree, parametric = parametric, : zero-width neighborhood. make span bigger
```

```
## Warning: Computation failed in `stat_smooth()`:
## NA/NaN/Inf in foreign function call (arg 5)
```

```
## Warning in simpleLoess(y, x, w, span, degree = degree, parametric = parametric, : at -0.005
```

```
## Warning in simpleLoess(y, x, w, span, degree = degree, parametric = parametric, : radius 2.5e-05
```

```
## Warning in simpleLoess(y, x, w, span, degree = degree, parametric = parametric, : all data on boundary of neighborhood. make span bigger
```

```
## Warning in simpleLoess(y, x, w, span, degree = degree, parametric = parametric, : pseudoinverse used at -0.005
```

```
## Warning in simpleLoess(y, x, w, span, degree = degree, parametric = parametric, : neighborhood radius 0.005
```

```
## Warning in simpleLoess(y, x, w, span, degree = degree, parametric = parametric, : reciprocal condition number 1
```

```
## Warning in simpleLoess(y, x, w, span, degree = degree, parametric = parametric, : There are other near singularities as well. 1.01
```

```
## Warning in simpleLoess(y, x, w, span, degree = degree, parametric = parametric, : zero-width neighborhood. make span bigger
```

```
## Warning: Computation failed in `stat_smooth()`:
## NA/NaN/Inf in foreign function call (arg 5)
```

```
## Warning in simpleLoess(y, x, w, span, degree = degree, parametric = parametric, : pseudoinverse used at -0.005
```

```
## Warning in simpleLoess(y, x, w, span, degree = degree, parametric = parametric, : neighborhood radius 1.005
```

```
## Warning in simpleLoess(y, x, w, span, degree = degree, parametric = parametric, : reciprocal condition number 0
```

```
## Warning in simpleLoess(y, x, w, span, degree = degree, parametric = parametric, : There are other near singularities as well. 1.01
```

```
## Warning in predLoess(object$y, object$x, newx = if (is.null(newdata)) object$x else if (is.data.frame(newdata)) as.matrix(model.frame(delete.response(terms(object)), : pseudoinverse used at -0.005
```

```
## Warning in predLoess(object$y, object$x, newx = if (is.null(newdata)) object$x else if (is.data.frame(newdata)) as.matrix(model.frame(delete.response(terms(object)), : neighborhood radius 1.005
```

```
## Warning in predLoess(object$y, object$x, newx = if (is.null(newdata)) object$x else if (is.data.frame(newdata)) as.matrix(model.frame(delete.response(terms(object)), : reciprocal condition number 0
```

```
## Warning in predLoess(object$y, object$x, newx = if (is.null(newdata)) object$x else if (is.data.frame(newdata)) as.matrix(model.frame(delete.response(terms(object)), : There are other near
## singularities as well. 1.01
```

```
## Warning in simpleLoess(y, x, w, span, degree = degree, parametric = parametric, : at -0.005
```

```
## Warning in simpleLoess(y, x, w, span, degree = degree, parametric = parametric, : radius 2.5e-05
```

```
## Warning in simpleLoess(y, x, w, span, degree = degree, parametric = parametric, : all data on boundary of neighborhood. make span bigger
```

```
## Warning in simpleLoess(y, x, w, span, degree = degree, parametric = parametric, : pseudoinverse used at -0.005
```

```
## Warning in simpleLoess(y, x, w, span, degree = degree, parametric = parametric, : neighborhood radius 0.005
```

```
## Warning in simpleLoess(y, x, w, span, degree = degree, parametric = parametric, : reciprocal condition number 1
```

```
## Warning in simpleLoess(y, x, w, span, degree = degree, parametric = parametric, : There are other near singularities as well. 1.01
```

```
## Warning in simpleLoess(y, x, w, span, degree = degree, parametric = parametric, : zero-width neighborhood. make span bigger
```

```
## Warning: Computation failed in `stat_smooth()`:
## NA/NaN/Inf in foreign function call (arg 5)
```

```
## Warning in simpleLoess(y, x, w, span, degree = degree, parametric = parametric, : at -0.005
```

```
## Warning in simpleLoess(y, x, w, span, degree = degree, parametric = parametric, : radius 2.5e-05
```

```
## Warning in simpleLoess(y, x, w, span, degree = degree, parametric = parametric, : all data on boundary of neighborhood. make span bigger
```

```
## Warning in simpleLoess(y, x, w, span, degree = degree, parametric = parametric, : pseudoinverse used at -0.005
```

```
## Warning in simpleLoess(y, x, w, span, degree = degree, parametric = parametric, : neighborhood radius 0.005
```

```
## Warning in simpleLoess(y, x, w, span, degree = degree, parametric = parametric, : reciprocal condition number 1
```

```
## Warning in simpleLoess(y, x, w, span, degree = degree, parametric = parametric, : There are other near singularities as well. 1.01
```

```
## Warning in simpleLoess(y, x, w, span, degree = degree, parametric = parametric, : zero-width neighborhood. make span bigger
```

```
## Warning: Computation failed in `stat_smooth()`:
## NA/NaN/Inf in foreign function call (arg 5)
```

```
## Warning in simpleLoess(y, x, w, span, degree = degree, parametric = parametric, : at -0.005
```

```
## Warning in simpleLoess(y, x, w, span, degree = degree, parametric = parametric, : radius 2.5e-05
```

```
## Warning in simpleLoess(y, x, w, span, degree = degree, parametric = parametric, : all data on boundary of neighborhood. make span bigger
```

```
## Warning in simpleLoess(y, x, w, span, degree = degree, parametric = parametric, : pseudoinverse used at -0.005
```

```
## Warning in simpleLoess(y, x, w, span, degree = degree, parametric = parametric, : neighborhood radius 0.005
```

```
## Warning in simpleLoess(y, x, w, span, degree = degree, parametric = parametric, : reciprocal condition number 1
```

```
## Warning in simpleLoess(y, x, w, span, degree = degree, parametric = parametric, : There are other near singularities as well. 1.01
```

```
## Warning in simpleLoess(y, x, w, span, degree = degree, parametric = parametric, : zero-width neighborhood. make span bigger
```

```
## Warning: Computation failed in `stat_smooth()`:
## NA/NaN/Inf in foreign function call (arg 5)
```

```
## Warning in simpleLoess(y, x, w, span, degree = degree, parametric = parametric, : at -0.005
```

```
## Warning in simpleLoess(y, x, w, span, degree = degree, parametric = parametric, : radius 2.5e-05
```

```
## Warning in simpleLoess(y, x, w, span, degree = degree, parametric = parametric, : all data on boundary of neighborhood. make span bigger
```

```
## Warning in simpleLoess(y, x, w, span, degree = degree, parametric = parametric, : pseudoinverse used at -0.005
```

```
## Warning in simpleLoess(y, x, w, span, degree = degree, parametric = parametric, : neighborhood radius 0.005
```

```
## Warning in simpleLoess(y, x, w, span, degree = degree, parametric = parametric, : reciprocal condition number 1
```

```
## Warning in simpleLoess(y, x, w, span, degree = degree, parametric = parametric, : There are other near singularities as well. 1.01
```

```
## Warning in simpleLoess(y, x, w, span, degree = degree, parametric = parametric, : zero-width neighborhood. make span bigger
```

```
## Warning: Computation failed in `stat_smooth()`:
## NA/NaN/Inf in foreign function call (arg 5)
```

```
## Warning in simpleLoess(y, x, w, span, degree = degree, parametric = parametric, : at -0.005
```

```
## Warning in simpleLoess(y, x, w, span, degree = degree, parametric = parametric, : radius 2.5e-05
```

```
## Warning in simpleLoess(y, x, w, span, degree = degree, parametric = parametric, : all data on boundary of neighborhood. make span bigger
```

```
## Warning in simpleLoess(y, x, w, span, degree = degree, parametric = parametric, : pseudoinverse used at -0.005
```

```
## Warning in simpleLoess(y, x, w, span, degree = degree, parametric = parametric, : neighborhood radius 0.005
```

```
## Warning in simpleLoess(y, x, w, span, degree = degree, parametric = parametric, : reciprocal condition number 1
```

```
## Warning in simpleLoess(y, x, w, span, degree = degree, parametric = parametric, : There are other near singularities as well. 1.01
```

```
## Warning in simpleLoess(y, x, w, span, degree = degree, parametric = parametric, : zero-width neighborhood. make span bigger
```

```
## Warning: Computation failed in `stat_smooth()`:
## NA/NaN/Inf in foreign function call (arg 5)
```

```
## Warning in simpleLoess(y, x, w, span, degree = degree, parametric = parametric, : at -0.005
```

```
## Warning in simpleLoess(y, x, w, span, degree = degree, parametric = parametric, : radius 2.5e-05
```

```
## Warning in simpleLoess(y, x, w, span, degree = degree, parametric = parametric, : all data on boundary of neighborhood. make span bigger
```

```
## Warning in simpleLoess(y, x, w, span, degree = degree, parametric = parametric, : pseudoinverse used at -0.005
```

```
## Warning in simpleLoess(y, x, w, span, degree = degree, parametric = parametric, : neighborhood radius 0.005
```

```
## Warning in simpleLoess(y, x, w, span, degree = degree, parametric = parametric, : reciprocal condition number 1
```

```
## Warning in simpleLoess(y, x, w, span, degree = degree, parametric = parametric, : There are other near singularities as well. 1.01
```

```
## Warning in simpleLoess(y, x, w, span, degree = degree, parametric = parametric, : zero-width neighborhood. make span bigger
```

```
## Warning: Computation failed in `stat_smooth()`:
## NA/NaN/Inf in foreign function call (arg 5)
```

```
## Warning in simpleLoess(y, x, w, span, degree = degree, parametric = parametric, : at -0.005
```

```
## Warning in simpleLoess(y, x, w, span, degree = degree, parametric = parametric, : radius 2.5e-05
```

```
## Warning in simpleLoess(y, x, w, span, degree = degree, parametric = parametric, : all data on boundary of neighborhood. make span bigger
```

```
## Warning in simpleLoess(y, x, w, span, degree = degree, parametric = parametric, : pseudoinverse used at -0.005
```

```
## Warning in simpleLoess(y, x, w, span, degree = degree, parametric = parametric, : neighborhood radius 0.005
```

```
## Warning in simpleLoess(y, x, w, span, degree = degree, parametric = parametric, : reciprocal condition number 1
```

```
## Warning in simpleLoess(y, x, w, span, degree = degree, parametric = parametric, : There are other near singularities as well. 1.01
```

```
## Warning in simpleLoess(y, x, w, span, degree = degree, parametric = parametric, : zero-width neighborhood. make span bigger
```

```
## Warning: Computation failed in `stat_smooth()`:
## NA/NaN/Inf in foreign function call (arg 5)
```

```
## Warning in simpleLoess(y, x, w, span, degree = degree, parametric = parametric, : pseudoinverse used at -0.005
```

```
## Warning in simpleLoess(y, x, w, span, degree = degree, parametric = parametric, : neighborhood radius 1.005
```

```
## Warning in simpleLoess(y, x, w, span, degree = degree, parametric = parametric, : reciprocal condition number 0
```

```
## Warning in simpleLoess(y, x, w, span, degree = degree, parametric = parametric, : There are other near singularities as well. 1.01
```

```
## Warning in predLoess(object$y, object$x, newx = if (is.null(newdata)) object$x else if (is.data.frame(newdata)) as.matrix(model.frame(delete.response(terms(object)), : pseudoinverse used at -0.005
```

```
## Warning in predLoess(object$y, object$x, newx = if (is.null(newdata)) object$x else if (is.data.frame(newdata)) as.matrix(model.frame(delete.response(terms(object)), : neighborhood radius 1.005
```

```
## Warning in predLoess(object$y, object$x, newx = if (is.null(newdata)) object$x else if (is.data.frame(newdata)) as.matrix(model.frame(delete.response(terms(object)), : reciprocal condition number 0
```

```
## Warning in predLoess(object$y, object$x, newx = if (is.null(newdata)) object$x else if (is.data.frame(newdata)) as.matrix(model.frame(delete.response(terms(object)), : There are other near
## singularities as well. 1.01
```

```
## Warning in simpleLoess(y, x, w, span, degree = degree, parametric = parametric, : pseudoinverse used at -0.005
```

```
## Warning in simpleLoess(y, x, w, span, degree = degree, parametric = parametric, : neighborhood radius 1.005
```

```
## Warning in simpleLoess(y, x, w, span, degree = degree, parametric = parametric, : reciprocal condition number 0
```

```
## Warning in simpleLoess(y, x, w, span, degree = degree, parametric = parametric, : There are other near singularities as well. 1.01
```

```
## Warning in predLoess(object$y, object$x, newx = if (is.null(newdata)) object$x else if (is.data.frame(newdata)) as.matrix(model.frame(delete.response(terms(object)), : pseudoinverse used at -0.005
```

```
## Warning in predLoess(object$y, object$x, newx = if (is.null(newdata)) object$x else if (is.data.frame(newdata)) as.matrix(model.frame(delete.response(terms(object)), : neighborhood radius 1.005
```

```
## Warning in predLoess(object$y, object$x, newx = if (is.null(newdata)) object$x else if (is.data.frame(newdata)) as.matrix(model.frame(delete.response(terms(object)), : reciprocal condition number 0
```

```
## Warning in predLoess(object$y, object$x, newx = if (is.null(newdata)) object$x else if (is.data.frame(newdata)) as.matrix(model.frame(delete.response(terms(object)), : There are other near
## singularities as well. 1.01
```

```
## Warning in simpleLoess(y, x, w, span, degree = degree, parametric = parametric, : at -0.005
```

```
## Warning in simpleLoess(y, x, w, span, degree = degree, parametric = parametric, : radius 2.5e-05
```

```
## Warning in simpleLoess(y, x, w, span, degree = degree, parametric = parametric, : all data on boundary of neighborhood. make span bigger
```

```
## Warning in simpleLoess(y, x, w, span, degree = degree, parametric = parametric, : pseudoinverse used at -0.005
```

```
## Warning in simpleLoess(y, x, w, span, degree = degree, parametric = parametric, : neighborhood radius 0.005
```

```
## Warning in simpleLoess(y, x, w, span, degree = degree, parametric = parametric, : reciprocal condition number 1
```

```
## Warning in simpleLoess(y, x, w, span, degree = degree, parametric = parametric, : There are other near singularities as well. 1.01
```

```
## Warning in simpleLoess(y, x, w, span, degree = degree, parametric = parametric, : zero-width neighborhood. make span bigger
```

```
## Warning: Computation failed in `stat_smooth()`:
## NA/NaN/Inf in foreign function call (arg 5)
```

```
## Warning in simpleLoess(y, x, w, span, degree = degree, parametric = parametric, : at -0.005
```

```
## Warning in simpleLoess(y, x, w, span, degree = degree, parametric = parametric, : radius 2.5e-05
```

```
## Warning in simpleLoess(y, x, w, span, degree = degree, parametric = parametric, : all data on boundary of neighborhood. make span bigger
```

```
## Warning in simpleLoess(y, x, w, span, degree = degree, parametric = parametric, : pseudoinverse used at -0.005
```

```
## Warning in simpleLoess(y, x, w, span, degree = degree, parametric = parametric, : neighborhood radius 0.005
```

```
## Warning in simpleLoess(y, x, w, span, degree = degree, parametric = parametric, : reciprocal condition number 1
```

```
## Warning in simpleLoess(y, x, w, span, degree = degree, parametric = parametric, : There are other near singularities as well. 1.01
```

```
## Warning in simpleLoess(y, x, w, span, degree = degree, parametric = parametric, : zero-width neighborhood. make span bigger
```

```
## Warning: Computation failed in `stat_smooth()`:
## NA/NaN/Inf in foreign function call (arg 5)
```

```
## Warning in simpleLoess(y, x, w, span, degree = degree, parametric = parametric, : at -0.005
```

```
## Warning in simpleLoess(y, x, w, span, degree = degree, parametric = parametric, : radius 2.5e-05
```

```
## Warning in simpleLoess(y, x, w, span, degree = degree, parametric = parametric, : all data on boundary of neighborhood. make span bigger
```

```
## Warning in simpleLoess(y, x, w, span, degree = degree, parametric = parametric, : pseudoinverse used at -0.005
```

```
## Warning in simpleLoess(y, x, w, span, degree = degree, parametric = parametric, : neighborhood radius 0.005
```

```
## Warning in simpleLoess(y, x, w, span, degree = degree, parametric = parametric, : reciprocal condition number 1
```

```
## Warning in simpleLoess(y, x, w, span, degree = degree, parametric = parametric, : There are other near singularities as well. 1.01
```

```
## Warning in simpleLoess(y, x, w, span, degree = degree, parametric = parametric, : zero-width neighborhood. make span bigger
```

```
## Warning: Computation failed in `stat_smooth()`:
## NA/NaN/Inf in foreign function call (arg 5)
```

```
## Warning in simpleLoess(y, x, w, span, degree = degree, parametric = parametric, : at -0.005
```

```
## Warning in simpleLoess(y, x, w, span, degree = degree, parametric = parametric, : radius 2.5e-05
```

```
## Warning in simpleLoess(y, x, w, span, degree = degree, parametric = parametric, : all data on boundary of neighborhood. make span bigger
```

```
## Warning in simpleLoess(y, x, w, span, degree = degree, parametric = parametric, : pseudoinverse used at -0.005
```

```
## Warning in simpleLoess(y, x, w, span, degree = degree, parametric = parametric, : neighborhood radius 0.005
```

```
## Warning in simpleLoess(y, x, w, span, degree = degree, parametric = parametric, : reciprocal condition number 1
```

```
## Warning in simpleLoess(y, x, w, span, degree = degree, parametric = parametric, : There are other near singularities as well. 1.01
```

```
## Warning in simpleLoess(y, x, w, span, degree = degree, parametric = parametric, : zero-width neighborhood. make span bigger
```

```
## Warning: Computation failed in `stat_smooth()`:
## NA/NaN/Inf in foreign function call (arg 5)
```

```
## Warning in simpleLoess(y, x, w, span, degree = degree, parametric = parametric, : at -0.005
```

```
## Warning in simpleLoess(y, x, w, span, degree = degree, parametric = parametric, : radius 2.5e-05
```

```
## Warning in simpleLoess(y, x, w, span, degree = degree, parametric = parametric, : all data on boundary of neighborhood. make span bigger
```

```
## Warning in simpleLoess(y, x, w, span, degree = degree, parametric = parametric, : pseudoinverse used at -0.005
```

```
## Warning in simpleLoess(y, x, w, span, degree = degree, parametric = parametric, : neighborhood radius 0.005
```

```
## Warning in simpleLoess(y, x, w, span, degree = degree, parametric = parametric, : reciprocal condition number 1
```

```
## Warning in simpleLoess(y, x, w, span, degree = degree, parametric = parametric, : There are other near singularities as well. 1.01
```

```
## Warning in simpleLoess(y, x, w, span, degree = degree, parametric = parametric, : zero-width neighborhood. make span bigger
```

```
## Warning: Computation failed in `stat_smooth()`:
## NA/NaN/Inf in foreign function call (arg 5)
```

```
## Warning in simpleLoess(y, x, w, span, degree = degree, parametric = parametric, : at -0.005
```

```
## Warning in simpleLoess(y, x, w, span, degree = degree, parametric = parametric, : radius 2.5e-05
```

```
## Warning in simpleLoess(y, x, w, span, degree = degree, parametric = parametric, : all data on boundary of neighborhood. make span bigger
```

```
## Warning in simpleLoess(y, x, w, span, degree = degree, parametric = parametric, : pseudoinverse used at -0.005
```

```
## Warning in simpleLoess(y, x, w, span, degree = degree, parametric = parametric, : neighborhood radius 0.005
```

```
## Warning in simpleLoess(y, x, w, span, degree = degree, parametric = parametric, : reciprocal condition number 1
```

```
## Warning in simpleLoess(y, x, w, span, degree = degree, parametric = parametric, : There are other near singularities as well. 1.01
```

```
## Warning in simpleLoess(y, x, w, span, degree = degree, parametric = parametric, : zero-width neighborhood. make span bigger
```

```
## Warning: Computation failed in `stat_smooth()`:
## NA/NaN/Inf in foreign function call (arg 5)
```

```
## Warning in simpleLoess(y, x, w, span, degree = degree, parametric = parametric, : at -0.005
```

```
## Warning in simpleLoess(y, x, w, span, degree = degree, parametric = parametric, : radius 2.5e-05
```

```
## Warning in simpleLoess(y, x, w, span, degree = degree, parametric = parametric, : all data on boundary of neighborhood. make span bigger
```

```
## Warning in simpleLoess(y, x, w, span, degree = degree, parametric = parametric, : pseudoinverse used at -0.005
```

```
## Warning in simpleLoess(y, x, w, span, degree = degree, parametric = parametric, : neighborhood radius 0.005
```

```
## Warning in simpleLoess(y, x, w, span, degree = degree, parametric = parametric, : reciprocal condition number 1
```

```
## Warning in simpleLoess(y, x, w, span, degree = degree, parametric = parametric, : There are other near singularities as well. 1.01
```

```
## Warning in simpleLoess(y, x, w, span, degree = degree, parametric = parametric, : zero-width neighborhood. make span bigger
```

```
## Warning: Computation failed in `stat_smooth()`:
## NA/NaN/Inf in foreign function call (arg 5)
```

```
## Warning in simpleLoess(y, x, w, span, degree = degree, parametric = parametric, : pseudoinverse used at -0.005
```

```
## Warning in simpleLoess(y, x, w, span, degree = degree, parametric = parametric, : neighborhood radius 1.005
```

```
## Warning in simpleLoess(y, x, w, span, degree = degree, parametric = parametric, : reciprocal condition number 0
```

```
## Warning in simpleLoess(y, x, w, span, degree = degree, parametric = parametric, : There are other near singularities as well. 1.01
```

```
## Warning in predLoess(object$y, object$x, newx = if (is.null(newdata)) object$x else if (is.data.frame(newdata)) as.matrix(model.frame(delete.response(terms(object)), : pseudoinverse used at -0.005
```

```
## Warning in predLoess(object$y, object$x, newx = if (is.null(newdata)) object$x else if (is.data.frame(newdata)) as.matrix(model.frame(delete.response(terms(object)), : neighborhood radius 1.005
```

```
## Warning in predLoess(object$y, object$x, newx = if (is.null(newdata)) object$x else if (is.data.frame(newdata)) as.matrix(model.frame(delete.response(terms(object)), : reciprocal condition number 0
```

```
## Warning in predLoess(object$y, object$x, newx = if (is.null(newdata)) object$x else if (is.data.frame(newdata)) as.matrix(model.frame(delete.response(terms(object)), : There are other near
## singularities as well. 1.01
```

```
## Warning in simpleLoess(y, x, w, span, degree = degree, parametric = parametric, : at -0.005
```

```
## Warning in simpleLoess(y, x, w, span, degree = degree, parametric = parametric, : radius 2.5e-05
```

```
## Warning in simpleLoess(y, x, w, span, degree = degree, parametric = parametric, : all data on boundary of neighborhood. make span bigger
```

```
## Warning in simpleLoess(y, x, w, span, degree = degree, parametric = parametric, : pseudoinverse used at -0.005
```

```
## Warning in simpleLoess(y, x, w, span, degree = degree, parametric = parametric, : neighborhood radius 0.005
```

```
## Warning in simpleLoess(y, x, w, span, degree = degree, parametric = parametric, : reciprocal condition number 1
```

```
## Warning in simpleLoess(y, x, w, span, degree = degree, parametric = parametric, : There are other near singularities as well. 1.01
```

```
## Warning in simpleLoess(y, x, w, span, degree = degree, parametric = parametric, : zero-width neighborhood. make span bigger
```

```
## Warning: Computation failed in `stat_smooth()`:
## NA/NaN/Inf in foreign function call (arg 5)
```

```
## Warning in simpleLoess(y, x, w, span, degree = degree, parametric = parametric, : at -0.005
```

```
## Warning in simpleLoess(y, x, w, span, degree = degree, parametric = parametric, : radius 2.5e-05
```

```
## Warning in simpleLoess(y, x, w, span, degree = degree, parametric = parametric, : all data on boundary of neighborhood. make span bigger
```

```
## Warning in simpleLoess(y, x, w, span, degree = degree, parametric = parametric, : pseudoinverse used at -0.005
```

```
## Warning in simpleLoess(y, x, w, span, degree = degree, parametric = parametric, : neighborhood radius 0.005
```

```
## Warning in simpleLoess(y, x, w, span, degree = degree, parametric = parametric, : reciprocal condition number 1
```

```
## Warning in simpleLoess(y, x, w, span, degree = degree, parametric = parametric, : There are other near singularities as well. 1.01
```

```
## Warning in simpleLoess(y, x, w, span, degree = degree, parametric = parametric, : zero-width neighborhood. make span bigger
```

```
## Warning: Computation failed in `stat_smooth()`:
## NA/NaN/Inf in foreign function call (arg 5)
```

```
## Warning in simpleLoess(y, x, w, span, degree = degree, parametric = parametric, : at -0.005
```

```
## Warning in simpleLoess(y, x, w, span, degree = degree, parametric = parametric, : radius 2.5e-05
```

```
## Warning in simpleLoess(y, x, w, span, degree = degree, parametric = parametric, : all data on boundary of neighborhood. make span bigger
```

```
## Warning in simpleLoess(y, x, w, span, degree = degree, parametric = parametric, : pseudoinverse used at -0.005
```

```
## Warning in simpleLoess(y, x, w, span, degree = degree, parametric = parametric, : neighborhood radius 0.005
```

```
## Warning in simpleLoess(y, x, w, span, degree = degree, parametric = parametric, : reciprocal condition number 1
```

```
## Warning in simpleLoess(y, x, w, span, degree = degree, parametric = parametric, : There are other near singularities as well. 1.01
```

```
## Warning in simpleLoess(y, x, w, span, degree = degree, parametric = parametric, : zero-width neighborhood. make span bigger
```

```
## Warning: Computation failed in `stat_smooth()`:
## NA/NaN/Inf in foreign function call (arg 5)
```

```
## Warning in simpleLoess(y, x, w, span, degree = degree, parametric = parametric, : at -0.005
```

```
## Warning in simpleLoess(y, x, w, span, degree = degree, parametric = parametric, : radius 2.5e-05
```

```
## Warning in simpleLoess(y, x, w, span, degree = degree, parametric = parametric, : all data on boundary of neighborhood. make span bigger
```

```
## Warning in simpleLoess(y, x, w, span, degree = degree, parametric = parametric, : pseudoinverse used at -0.005
```

```
## Warning in simpleLoess(y, x, w, span, degree = degree, parametric = parametric, : neighborhood radius 0.005
```

```
## Warning in simpleLoess(y, x, w, span, degree = degree, parametric = parametric, : reciprocal condition number 1
```

```
## Warning in simpleLoess(y, x, w, span, degree = degree, parametric = parametric, : There are other near singularities as well. 1.01
```

```
## Warning in simpleLoess(y, x, w, span, degree = degree, parametric = parametric, : zero-width neighborhood. make span bigger
```

```
## Warning: Computation failed in `stat_smooth()`:
## NA/NaN/Inf in foreign function call (arg 5)
```

```
## Warning in simpleLoess(y, x, w, span, degree = degree, parametric = parametric, : at -0.005
```

```
## Warning in simpleLoess(y, x, w, span, degree = degree, parametric = parametric, : radius 2.5e-05
```

```
## Warning in simpleLoess(y, x, w, span, degree = degree, parametric = parametric, : all data on boundary of neighborhood. make span bigger
```

```
## Warning in simpleLoess(y, x, w, span, degree = degree, parametric = parametric, : pseudoinverse used at -0.005
```

```
## Warning in simpleLoess(y, x, w, span, degree = degree, parametric = parametric, : neighborhood radius 0.005
```

```
## Warning in simpleLoess(y, x, w, span, degree = degree, parametric = parametric, : reciprocal condition number 1
```

```
## Warning in simpleLoess(y, x, w, span, degree = degree, parametric = parametric, : There are other near singularities as well. 1.01
```

```
## Warning in simpleLoess(y, x, w, span, degree = degree, parametric = parametric, : zero-width neighborhood. make span bigger
```

```
## Warning: Computation failed in `stat_smooth()`:
## NA/NaN/Inf in foreign function call (arg 5)
```

```
## Warning in simpleLoess(y, x, w, span, degree = degree, parametric = parametric, : at -0.005
```

```
## Warning in simpleLoess(y, x, w, span, degree = degree, parametric = parametric, : radius 2.5e-05
```

```
## Warning in simpleLoess(y, x, w, span, degree = degree, parametric = parametric, : all data on boundary of neighborhood. make span bigger
```

```
## Warning in simpleLoess(y, x, w, span, degree = degree, parametric = parametric, : pseudoinverse used at -0.005
```

```
## Warning in simpleLoess(y, x, w, span, degree = degree, parametric = parametric, : neighborhood radius 0.005
```

```
## Warning in simpleLoess(y, x, w, span, degree = degree, parametric = parametric, : reciprocal condition number 1
```

```
## Warning in simpleLoess(y, x, w, span, degree = degree, parametric = parametric, : There are other near singularities as well. 1.01
```

```
## Warning in simpleLoess(y, x, w, span, degree = degree, parametric = parametric, : zero-width neighborhood. make span bigger
```

```
## Warning: Computation failed in `stat_smooth()`:
## NA/NaN/Inf in foreign function call (arg 5)
```

```
## Warning in simpleLoess(y, x, w, span, degree = degree, parametric = parametric, : pseudoinverse used at -0.005
```

```
## Warning in simpleLoess(y, x, w, span, degree = degree, parametric = parametric, : neighborhood radius 1.005
```

```
## Warning in simpleLoess(y, x, w, span, degree = degree, parametric = parametric, : reciprocal condition number 0
```

```
## Warning in simpleLoess(y, x, w, span, degree = degree, parametric = parametric, : There are other near singularities as well. 1.01
```

```
## Warning in predLoess(object$y, object$x, newx = if (is.null(newdata)) object$x else if (is.data.frame(newdata)) as.matrix(model.frame(delete.response(terms(object)), : pseudoinverse used at -0.005
```

```
## Warning in predLoess(object$y, object$x, newx = if (is.null(newdata)) object$x else if (is.data.frame(newdata)) as.matrix(model.frame(delete.response(terms(object)), : neighborhood radius 1.005
```

```
## Warning in predLoess(object$y, object$x, newx = if (is.null(newdata)) object$x else if (is.data.frame(newdata)) as.matrix(model.frame(delete.response(terms(object)), : reciprocal condition number 0
```

```
## Warning in predLoess(object$y, object$x, newx = if (is.null(newdata)) object$x else if (is.data.frame(newdata)) as.matrix(model.frame(delete.response(terms(object)), : There are other near
## singularities as well. 1.01
```

```
## Warning in simpleLoess(y, x, w, span, degree = degree, parametric = parametric, : pseudoinverse used at -0.005
```

```
## Warning in simpleLoess(y, x, w, span, degree = degree, parametric = parametric, : neighborhood radius 1.005
```

```
## Warning in simpleLoess(y, x, w, span, degree = degree, parametric = parametric, : reciprocal condition number 0
```

```
## Warning in simpleLoess(y, x, w, span, degree = degree, parametric = parametric, : There are other near singularities as well. 1.01
```

```
## Warning in predLoess(object$y, object$x, newx = if (is.null(newdata)) object$x else if (is.data.frame(newdata)) as.matrix(model.frame(delete.response(terms(object)), : pseudoinverse used at -0.005
```

```
## Warning in predLoess(object$y, object$x, newx = if (is.null(newdata)) object$x else if (is.data.frame(newdata)) as.matrix(model.frame(delete.response(terms(object)), : neighborhood radius 1.005
```

```
## Warning in predLoess(object$y, object$x, newx = if (is.null(newdata)) object$x else if (is.data.frame(newdata)) as.matrix(model.frame(delete.response(terms(object)), : reciprocal condition number 0
```

```
## Warning in predLoess(object$y, object$x, newx = if (is.null(newdata)) object$x else if (is.data.frame(newdata)) as.matrix(model.frame(delete.response(terms(object)), : There are other near
## singularities as well. 1.01
```

```
## Warning in simpleLoess(y, x, w, span, degree = degree, parametric = parametric, : at -0.005
```

```
## Warning in simpleLoess(y, x, w, span, degree = degree, parametric = parametric, : radius 2.5e-05
```

```
## Warning in simpleLoess(y, x, w, span, degree = degree, parametric = parametric, : all data on boundary of neighborhood. make span bigger
```

```
## Warning in simpleLoess(y, x, w, span, degree = degree, parametric = parametric, : pseudoinverse used at -0.005
```

```
## Warning in simpleLoess(y, x, w, span, degree = degree, parametric = parametric, : neighborhood radius 0.005
```

```
## Warning in simpleLoess(y, x, w, span, degree = degree, parametric = parametric, : reciprocal condition number 1
```

```
## Warning in simpleLoess(y, x, w, span, degree = degree, parametric = parametric, : There are other near singularities as well. 1.01
```

```
## Warning in simpleLoess(y, x, w, span, degree = degree, parametric = parametric, : zero-width neighborhood. make span bigger
```

```
## Warning: Computation failed in `stat_smooth()`:
## NA/NaN/Inf in foreign function call (arg 5)
```

```
## Warning in simpleLoess(y, x, w, span, degree = degree, parametric = parametric, : at -0.005
```

```
## Warning in simpleLoess(y, x, w, span, degree = degree, parametric = parametric, : radius 2.5e-05
```

```
## Warning in simpleLoess(y, x, w, span, degree = degree, parametric = parametric, : all data on boundary of neighborhood. make span bigger
```

```
## Warning in simpleLoess(y, x, w, span, degree = degree, parametric = parametric, : pseudoinverse used at -0.005
```

```
## Warning in simpleLoess(y, x, w, span, degree = degree, parametric = parametric, : neighborhood radius 0.005
```

```
## Warning in simpleLoess(y, x, w, span, degree = degree, parametric = parametric, : reciprocal condition number 1
```

```
## Warning in simpleLoess(y, x, w, span, degree = degree, parametric = parametric, : There are other near singularities as well. 1.01
```

```
## Warning in simpleLoess(y, x, w, span, degree = degree, parametric = parametric, : zero-width neighborhood. make span bigger
```

```
## Warning: Computation failed in `stat_smooth()`:
## NA/NaN/Inf in foreign function call (arg 5)
```

```
## Warning in simpleLoess(y, x, w, span, degree = degree, parametric = parametric, : at -0.005
```

```
## Warning in simpleLoess(y, x, w, span, degree = degree, parametric = parametric, : radius 2.5e-05
```

```
## Warning in simpleLoess(y, x, w, span, degree = degree, parametric = parametric, : all data on boundary of neighborhood. make span bigger
```

```
## Warning in simpleLoess(y, x, w, span, degree = degree, parametric = parametric, : pseudoinverse used at -0.005
```

```
## Warning in simpleLoess(y, x, w, span, degree = degree, parametric = parametric, : neighborhood radius 0.005
```

```
## Warning in simpleLoess(y, x, w, span, degree = degree, parametric = parametric, : reciprocal condition number 1
```

```
## Warning in simpleLoess(y, x, w, span, degree = degree, parametric = parametric, : There are other near singularities as well. 1.01
```

```
## Warning in simpleLoess(y, x, w, span, degree = degree, parametric = parametric, : zero-width neighborhood. make span bigger
```

```
## Warning: Computation failed in `stat_smooth()`:
## NA/NaN/Inf in foreign function call (arg 5)
```

```
## Warning in simpleLoess(y, x, w, span, degree = degree, parametric = parametric, : at -0.005
```

```
## Warning in simpleLoess(y, x, w, span, degree = degree, parametric = parametric, : radius 2.5e-05
```

```
## Warning in simpleLoess(y, x, w, span, degree = degree, parametric = parametric, : all data on boundary of neighborhood. make span bigger
```

```
## Warning in simpleLoess(y, x, w, span, degree = degree, parametric = parametric, : pseudoinverse used at -0.005
```

```
## Warning in simpleLoess(y, x, w, span, degree = degree, parametric = parametric, : neighborhood radius 0.005
```

```
## Warning in simpleLoess(y, x, w, span, degree = degree, parametric = parametric, : reciprocal condition number 1
```

```
## Warning in simpleLoess(y, x, w, span, degree = degree, parametric = parametric, : There are other near singularities as well. 1.01
```

```
## Warning in simpleLoess(y, x, w, span, degree = degree, parametric = parametric, : zero-width neighborhood. make span bigger
```

```
## Warning: Computation failed in `stat_smooth()`:
## NA/NaN/Inf in foreign function call (arg 5)
```

```
## Warning in simpleLoess(y, x, w, span, degree = degree, parametric = parametric, : at -0.005
```

```
## Warning in simpleLoess(y, x, w, span, degree = degree, parametric = parametric, : radius 2.5e-05
```

```
## Warning in simpleLoess(y, x, w, span, degree = degree, parametric = parametric, : all data on boundary of neighborhood. make span bigger
```

```
## Warning in simpleLoess(y, x, w, span, degree = degree, parametric = parametric, : pseudoinverse used at -0.005
```

```
## Warning in simpleLoess(y, x, w, span, degree = degree, parametric = parametric, : neighborhood radius 0.005
```

```
## Warning in simpleLoess(y, x, w, span, degree = degree, parametric = parametric, : reciprocal condition number 1
```

```
## Warning in simpleLoess(y, x, w, span, degree = degree, parametric = parametric, : There are other near singularities as well. 1.01
```

```
## Warning in simpleLoess(y, x, w, span, degree = degree, parametric = parametric, : zero-width neighborhood. make span bigger
```

```
## Warning: Computation failed in `stat_smooth()`:
## NA/NaN/Inf in foreign function call (arg 5)
```

```
## Warning in simpleLoess(y, x, w, span, degree = degree, parametric = parametric, : at -0.005
```

```
## Warning in simpleLoess(y, x, w, span, degree = degree, parametric = parametric, : radius 2.5e-05
```

```
## Warning in simpleLoess(y, x, w, span, degree = degree, parametric = parametric, : all data on boundary of neighborhood. make span bigger
```

```
## Warning in simpleLoess(y, x, w, span, degree = degree, parametric = parametric, : pseudoinverse used at -0.005
```

```
## Warning in simpleLoess(y, x, w, span, degree = degree, parametric = parametric, : neighborhood radius 0.005
```

```
## Warning in simpleLoess(y, x, w, span, degree = degree, parametric = parametric, : reciprocal condition number 1
```

```
## Warning in simpleLoess(y, x, w, span, degree = degree, parametric = parametric, : There are other near singularities as well. 1.01
```

```
## Warning in simpleLoess(y, x, w, span, degree = degree, parametric = parametric, : zero-width neighborhood. make span bigger
```

```
## Warning: Computation failed in `stat_smooth()`:
## NA/NaN/Inf in foreign function call (arg 5)
```

```
## Warning in simpleLoess(y, x, w, span, degree = degree, parametric = parametric, : at -0.005
```

```
## Warning in simpleLoess(y, x, w, span, degree = degree, parametric = parametric, : radius 2.5e-05
```

```
## Warning in simpleLoess(y, x, w, span, degree = degree, parametric = parametric, : all data on boundary of neighborhood. make span bigger
```

```
## Warning in simpleLoess(y, x, w, span, degree = degree, parametric = parametric, : pseudoinverse used at -0.005
```

```
## Warning in simpleLoess(y, x, w, span, degree = degree, parametric = parametric, : neighborhood radius 0.005
```

```
## Warning in simpleLoess(y, x, w, span, degree = degree, parametric = parametric, : reciprocal condition number 1
```

```
## Warning in simpleLoess(y, x, w, span, degree = degree, parametric = parametric, : There are other near singularities as well. 1.01
```

```
## Warning in simpleLoess(y, x, w, span, degree = degree, parametric = parametric, : zero-width neighborhood. make span bigger
```

```
## Warning: Computation failed in `stat_smooth()`:
## NA/NaN/Inf in foreign function call (arg 5)
```

```
## Warning in simpleLoess(y, x, w, span, degree = degree, parametric = parametric, : at -0.005
```

```
## Warning in simpleLoess(y, x, w, span, degree = degree, parametric = parametric, : radius 2.5e-05
```

```
## Warning in simpleLoess(y, x, w, span, degree = degree, parametric = parametric, : all data on boundary of neighborhood. make span bigger
```

```
## Warning in simpleLoess(y, x, w, span, degree = degree, parametric = parametric, : pseudoinverse used at -0.005
```

```
## Warning in simpleLoess(y, x, w, span, degree = degree, parametric = parametric, : neighborhood radius 0.005
```

```
## Warning in simpleLoess(y, x, w, span, degree = degree, parametric = parametric, : reciprocal condition number 1
```

```
## Warning in simpleLoess(y, x, w, span, degree = degree, parametric = parametric, : There are other near singularities as well. 1.01
```

```
## Warning in simpleLoess(y, x, w, span, degree = degree, parametric = parametric, : zero-width neighborhood. make span bigger
```

```
## Warning: Computation failed in `stat_smooth()`:
## NA/NaN/Inf in foreign function call (arg 5)
```

```
## Warning in simpleLoess(y, x, w, span, degree = degree, parametric = parametric, : at -0.005
```

```
## Warning in simpleLoess(y, x, w, span, degree = degree, parametric = parametric, : radius 2.5e-05
```

```
## Warning in simpleLoess(y, x, w, span, degree = degree, parametric = parametric, : all data on boundary of neighborhood. make span bigger
```

```
## Warning in simpleLoess(y, x, w, span, degree = degree, parametric = parametric, : pseudoinverse used at -0.005
```

```
## Warning in simpleLoess(y, x, w, span, degree = degree, parametric = parametric, : neighborhood radius 0.005
```

```
## Warning in simpleLoess(y, x, w, span, degree = degree, parametric = parametric, : reciprocal condition number 1
```

```
## Warning in simpleLoess(y, x, w, span, degree = degree, parametric = parametric, : There are other near singularities as well. 1.01
```

```
## Warning in simpleLoess(y, x, w, span, degree = degree, parametric = parametric, : zero-width neighborhood. make span bigger
```

```
## Warning: Computation failed in `stat_smooth()`:
## NA/NaN/Inf in foreign function call (arg 5)
```

```
## Warning in simpleLoess(y, x, w, span, degree = degree, parametric = parametric, : at -0.005
```

```
## Warning in simpleLoess(y, x, w, span, degree = degree, parametric = parametric, : radius 2.5e-05
```

```
## Warning in simpleLoess(y, x, w, span, degree = degree, parametric = parametric, : all data on boundary of neighborhood. make span bigger
```

```
## Warning in simpleLoess(y, x, w, span, degree = degree, parametric = parametric, : pseudoinverse used at -0.005
```

```
## Warning in simpleLoess(y, x, w, span, degree = degree, parametric = parametric, : neighborhood radius 0.005
```

```
## Warning in simpleLoess(y, x, w, span, degree = degree, parametric = parametric, : reciprocal condition number 1
```

```
## Warning in simpleLoess(y, x, w, span, degree = degree, parametric = parametric, : There are other near singularities as well. 1.01
```

```
## Warning in simpleLoess(y, x, w, span, degree = degree, parametric = parametric, : zero-width neighborhood. make span bigger
```

```
## Warning: Computation failed in `stat_smooth()`:
## NA/NaN/Inf in foreign function call (arg 5)
```

```
## Warning in simpleLoess(y, x, w, span, degree = degree, parametric = parametric, : at -0.005
```

```
## Warning in simpleLoess(y, x, w, span, degree = degree, parametric = parametric, : radius 2.5e-05
```

```
## Warning in simpleLoess(y, x, w, span, degree = degree, parametric = parametric, : all data on boundary of neighborhood. make span bigger
```

```
## Warning in simpleLoess(y, x, w, span, degree = degree, parametric = parametric, : pseudoinverse used at -0.005
```

```
## Warning in simpleLoess(y, x, w, span, degree = degree, parametric = parametric, : neighborhood radius 0.005
```

```
## Warning in simpleLoess(y, x, w, span, degree = degree, parametric = parametric, : reciprocal condition number 1
```

```
## Warning in simpleLoess(y, x, w, span, degree = degree, parametric = parametric, : There are other near singularities as well. 1.01
```

```
## Warning in simpleLoess(y, x, w, span, degree = degree, parametric = parametric, : zero-width neighborhood. make span bigger
```

```
## Warning: Computation failed in `stat_smooth()`:
## NA/NaN/Inf in foreign function call (arg 5)
```

```
## Warning in simpleLoess(y, x, w, span, degree = degree, parametric = parametric, : pseudoinverse used at -0.005
```

```
## Warning in simpleLoess(y, x, w, span, degree = degree, parametric = parametric, : neighborhood radius 1.005
```

```
## Warning in simpleLoess(y, x, w, span, degree = degree, parametric = parametric, : reciprocal condition number 0
```

```
## Warning in simpleLoess(y, x, w, span, degree = degree, parametric = parametric, : There are other near singularities as well. 1.01
```

```
## Warning in predLoess(object$y, object$x, newx = if (is.null(newdata)) object$x else if (is.data.frame(newdata)) as.matrix(model.frame(delete.response(terms(object)), : pseudoinverse used at -0.005
```

```
## Warning in predLoess(object$y, object$x, newx = if (is.null(newdata)) object$x else if (is.data.frame(newdata)) as.matrix(model.frame(delete.response(terms(object)), : neighborhood radius 1.005
```

```
## Warning in predLoess(object$y, object$x, newx = if (is.null(newdata)) object$x else if (is.data.frame(newdata)) as.matrix(model.frame(delete.response(terms(object)), : reciprocal condition number 0
```

```
## Warning in predLoess(object$y, object$x, newx = if (is.null(newdata)) object$x else if (is.data.frame(newdata)) as.matrix(model.frame(delete.response(terms(object)), : There are other near
## singularities as well. 1.01
```

```
## Warning in simpleLoess(y, x, w, span, degree = degree, parametric = parametric, : at -0.005
```

```
## Warning in simpleLoess(y, x, w, span, degree = degree, parametric = parametric, : radius 2.5e-05
```

```
## Warning in simpleLoess(y, x, w, span, degree = degree, parametric = parametric, : all data on boundary of neighborhood. make span bigger
```

```
## Warning in simpleLoess(y, x, w, span, degree = degree, parametric = parametric, : pseudoinverse used at -0.005
```

```
## Warning in simpleLoess(y, x, w, span, degree = degree, parametric = parametric, : neighborhood radius 0.005
```

```
## Warning in simpleLoess(y, x, w, span, degree = degree, parametric = parametric, : reciprocal condition number 1
```

```
## Warning in simpleLoess(y, x, w, span, degree = degree, parametric = parametric, : There are other near singularities as well. 1.01
```

```
## Warning in simpleLoess(y, x, w, span, degree = degree, parametric = parametric, : zero-width neighborhood. make span bigger
```

```
## Warning: Computation failed in `stat_smooth()`:
## NA/NaN/Inf in foreign function call (arg 5)
```

```
## Warning in simpleLoess(y, x, w, span, degree = degree, parametric = parametric, : at -0.005
```

```
## Warning in simpleLoess(y, x, w, span, degree = degree, parametric = parametric, : radius 2.5e-05
```

```
## Warning in simpleLoess(y, x, w, span, degree = degree, parametric = parametric, : all data on boundary of neighborhood. make span bigger
```

```
## Warning in simpleLoess(y, x, w, span, degree = degree, parametric = parametric, : pseudoinverse used at -0.005
```

```
## Warning in simpleLoess(y, x, w, span, degree = degree, parametric = parametric, : neighborhood radius 0.005
```

```
## Warning in simpleLoess(y, x, w, span, degree = degree, parametric = parametric, : reciprocal condition number 1
```

```
## Warning in simpleLoess(y, x, w, span, degree = degree, parametric = parametric, : There are other near singularities as well. 1.01
```

```
## Warning in simpleLoess(y, x, w, span, degree = degree, parametric = parametric, : zero-width neighborhood. make span bigger
```

```
## Warning: Computation failed in `stat_smooth()`:
## NA/NaN/Inf in foreign function call (arg 5)
```

```
## Warning in simpleLoess(y, x, w, span, degree = degree, parametric = parametric, : at -0.005
```

```
## Warning in simpleLoess(y, x, w, span, degree = degree, parametric = parametric, : radius 2.5e-05
```

```
## Warning in simpleLoess(y, x, w, span, degree = degree, parametric = parametric, : all data on boundary of neighborhood. make span bigger
```

```
## Warning in simpleLoess(y, x, w, span, degree = degree, parametric = parametric, : pseudoinverse used at -0.005
```

```
## Warning in simpleLoess(y, x, w, span, degree = degree, parametric = parametric, : neighborhood radius 0.005
```

```
## Warning in simpleLoess(y, x, w, span, degree = degree, parametric = parametric, : reciprocal condition number 1
```

```
## Warning in simpleLoess(y, x, w, span, degree = degree, parametric = parametric, : There are other near singularities as well. 1.01
```

```
## Warning in simpleLoess(y, x, w, span, degree = degree, parametric = parametric, : zero-width neighborhood. make span bigger
```

```
## Warning: Computation failed in `stat_smooth()`:
## NA/NaN/Inf in foreign function call (arg 5)
```

```
## Warning in simpleLoess(y, x, w, span, degree = degree, parametric = parametric, : at -0.005
```

```
## Warning in simpleLoess(y, x, w, span, degree = degree, parametric = parametric, : radius 2.5e-05
```

```
## Warning in simpleLoess(y, x, w, span, degree = degree, parametric = parametric, : all data on boundary of neighborhood. make span bigger
```

```
## Warning in simpleLoess(y, x, w, span, degree = degree, parametric = parametric, : pseudoinverse used at -0.005
```

```
## Warning in simpleLoess(y, x, w, span, degree = degree, parametric = parametric, : neighborhood radius 0.005
```

```
## Warning in simpleLoess(y, x, w, span, degree = degree, parametric = parametric, : reciprocal condition number 1
```

```
## Warning in simpleLoess(y, x, w, span, degree = degree, parametric = parametric, : There are other near singularities as well. 1.01
```

```
## Warning in simpleLoess(y, x, w, span, degree = degree, parametric = parametric, : zero-width neighborhood. make span bigger
```

```
## Warning: Computation failed in `stat_smooth()`:
## NA/NaN/Inf in foreign function call (arg 5)
```

```
## Warning in simpleLoess(y, x, w, span, degree = degree, parametric = parametric, : at -0.005
```

```
## Warning in simpleLoess(y, x, w, span, degree = degree, parametric = parametric, : radius 2.5e-05
```

```
## Warning in simpleLoess(y, x, w, span, degree = degree, parametric = parametric, : all data on boundary of neighborhood. make span bigger
```

```
## Warning in simpleLoess(y, x, w, span, degree = degree, parametric = parametric, : pseudoinverse used at -0.005
```

```
## Warning in simpleLoess(y, x, w, span, degree = degree, parametric = parametric, : neighborhood radius 0.005
```

```
## Warning in simpleLoess(y, x, w, span, degree = degree, parametric = parametric, : reciprocal condition number 1
```

```
## Warning in simpleLoess(y, x, w, span, degree = degree, parametric = parametric, : There are other near singularities as well. 1.01
```

```
## Warning in simpleLoess(y, x, w, span, degree = degree, parametric = parametric, : zero-width neighborhood. make span bigger
```

```
## Warning: Computation failed in `stat_smooth()`:
## NA/NaN/Inf in foreign function call (arg 5)
```

```
## Warning in simpleLoess(y, x, w, span, degree = degree, parametric = parametric, : at -0.005
```

```
## Warning in simpleLoess(y, x, w, span, degree = degree, parametric = parametric, : radius 2.5e-05
```

```
## Warning in simpleLoess(y, x, w, span, degree = degree, parametric = parametric, : all data on boundary of neighborhood. make span bigger
```

```
## Warning in simpleLoess(y, x, w, span, degree = degree, parametric = parametric, : pseudoinverse used at -0.005
```

```
## Warning in simpleLoess(y, x, w, span, degree = degree, parametric = parametric, : neighborhood radius 0.005
```

```
## Warning in simpleLoess(y, x, w, span, degree = degree, parametric = parametric, : reciprocal condition number 1
```

```
## Warning in simpleLoess(y, x, w, span, degree = degree, parametric = parametric, : There are other near singularities as well. 1.01
```

```
## Warning in simpleLoess(y, x, w, span, degree = degree, parametric = parametric, : zero-width neighborhood. make span bigger
```

```
## Warning: Computation failed in `stat_smooth()`:
## NA/NaN/Inf in foreign function call (arg 5)
```

```
## Warning in simpleLoess(y, x, w, span, degree = degree, parametric = parametric, : at -0.005
```

```
## Warning in simpleLoess(y, x, w, span, degree = degree, parametric = parametric, : radius 2.5e-05
```

```
## Warning in simpleLoess(y, x, w, span, degree = degree, parametric = parametric, : all data on boundary of neighborhood. make span bigger
```

```
## Warning in simpleLoess(y, x, w, span, degree = degree, parametric = parametric, : pseudoinverse used at -0.005
```

```
## Warning in simpleLoess(y, x, w, span, degree = degree, parametric = parametric, : neighborhood radius 0.005
```

```
## Warning in simpleLoess(y, x, w, span, degree = degree, parametric = parametric, : reciprocal condition number 1
```

```
## Warning in simpleLoess(y, x, w, span, degree = degree, parametric = parametric, : There are other near singularities as well. 1.01
```

```
## Warning in simpleLoess(y, x, w, span, degree = degree, parametric = parametric, : zero-width neighborhood. make span bigger
```

```
## Warning: Computation failed in `stat_smooth()`:
## NA/NaN/Inf in foreign function call (arg 5)
```

```
## Warning in simpleLoess(y, x, w, span, degree = degree, parametric = parametric, : at -0.005
```

```
## Warning in simpleLoess(y, x, w, span, degree = degree, parametric = parametric, : radius 2.5e-05
```

```
## Warning in simpleLoess(y, x, w, span, degree = degree, parametric = parametric, : all data on boundary of neighborhood. make span bigger
```

```
## Warning in simpleLoess(y, x, w, span, degree = degree, parametric = parametric, : pseudoinverse used at -0.005
```

```
## Warning in simpleLoess(y, x, w, span, degree = degree, parametric = parametric, : neighborhood radius 0.005
```

```
## Warning in simpleLoess(y, x, w, span, degree = degree, parametric = parametric, : reciprocal condition number 1
```

```
## Warning in simpleLoess(y, x, w, span, degree = degree, parametric = parametric, : There are other near singularities as well. 1.01
```

```
## Warning in simpleLoess(y, x, w, span, degree = degree, parametric = parametric, : zero-width neighborhood. make span bigger
```

```
## Warning: Computation failed in `stat_smooth()`:
## NA/NaN/Inf in foreign function call (arg 5)
```

```
## Warning in simpleLoess(y, x, w, span, degree = degree, parametric = parametric, : at -0.005
```

```
## Warning in simpleLoess(y, x, w, span, degree = degree, parametric = parametric, : radius 2.5e-05
```

```
## Warning in simpleLoess(y, x, w, span, degree = degree, parametric = parametric, : all data on boundary of neighborhood. make span bigger
```

```
## Warning in simpleLoess(y, x, w, span, degree = degree, parametric = parametric, : pseudoinverse used at -0.005
```

```
## Warning in simpleLoess(y, x, w, span, degree = degree, parametric = parametric, : neighborhood radius 0.005
```

```
## Warning in simpleLoess(y, x, w, span, degree = degree, parametric = parametric, : reciprocal condition number 1
```

```
## Warning in simpleLoess(y, x, w, span, degree = degree, parametric = parametric, : There are other near singularities as well. 1.01
```

```
## Warning in simpleLoess(y, x, w, span, degree = degree, parametric = parametric, : zero-width neighborhood. make span bigger
```

```
## Warning: Computation failed in `stat_smooth()`:
## NA/NaN/Inf in foreign function call (arg 5)
```

```
## Warning in simpleLoess(y, x, w, span, degree = degree, parametric = parametric, : at -0.005
```

```
## Warning in simpleLoess(y, x, w, span, degree = degree, parametric = parametric, : radius 2.5e-05
```

```
## Warning in simpleLoess(y, x, w, span, degree = degree, parametric = parametric, : all data on boundary of neighborhood. make span bigger
```

```
## Warning in simpleLoess(y, x, w, span, degree = degree, parametric = parametric, : pseudoinverse used at -0.005
```

```
## Warning in simpleLoess(y, x, w, span, degree = degree, parametric = parametric, : neighborhood radius 0.005
```

```
## Warning in simpleLoess(y, x, w, span, degree = degree, parametric = parametric, : reciprocal condition number 1
```

```
## Warning in simpleLoess(y, x, w, span, degree = degree, parametric = parametric, : There are other near singularities as well. 1.01
```

```
## Warning in simpleLoess(y, x, w, span, degree = degree, parametric = parametric, : zero-width neighborhood. make span bigger
```

```
## Warning: Computation failed in `stat_smooth()`:
## NA/NaN/Inf in foreign function call (arg 5)
```

```
## Warning in simpleLoess(y, x, w, span, degree = degree, parametric = parametric, : at -0.005
```

```
## Warning in simpleLoess(y, x, w, span, degree = degree, parametric = parametric, : radius 2.5e-05
```

```
## Warning in simpleLoess(y, x, w, span, degree = degree, parametric = parametric, : all data on boundary of neighborhood. make span bigger
```

```
## Warning in simpleLoess(y, x, w, span, degree = degree, parametric = parametric, : pseudoinverse used at -0.005
```

```
## Warning in simpleLoess(y, x, w, span, degree = degree, parametric = parametric, : neighborhood radius 0.005
```

```
## Warning in simpleLoess(y, x, w, span, degree = degree, parametric = parametric, : reciprocal condition number 1
```

```
## Warning in simpleLoess(y, x, w, span, degree = degree, parametric = parametric, : There are other near singularities as well. 1.01
```

```
## Warning in simpleLoess(y, x, w, span, degree = degree, parametric = parametric, : zero-width neighborhood. make span bigger
```

```
## Warning: Computation failed in `stat_smooth()`:
## NA/NaN/Inf in foreign function call (arg 5)
```

```
## Warning in simpleLoess(y, x, w, span, degree = degree, parametric = parametric, : at -0.005
```

```
## Warning in simpleLoess(y, x, w, span, degree = degree, parametric = parametric, : radius 2.5e-05
```

```
## Warning in simpleLoess(y, x, w, span, degree = degree, parametric = parametric, : all data on boundary of neighborhood. make span bigger
```

```
## Warning in simpleLoess(y, x, w, span, degree = degree, parametric = parametric, : pseudoinverse used at -0.005
```

```
## Warning in simpleLoess(y, x, w, span, degree = degree, parametric = parametric, : neighborhood radius 0.005
```

```
## Warning in simpleLoess(y, x, w, span, degree = degree, parametric = parametric, : reciprocal condition number 1
```

```
## Warning in simpleLoess(y, x, w, span, degree = degree, parametric = parametric, : There are other near singularities as well. 1.01
```

```
## Warning in simpleLoess(y, x, w, span, degree = degree, parametric = parametric, : zero-width neighborhood. make span bigger
```

```
## Warning: Computation failed in `stat_smooth()`:
## NA/NaN/Inf in foreign function call (arg 5)
```

```
## Warning in simpleLoess(y, x, w, span, degree = degree, parametric = parametric, : at -0.005
```

```
## Warning in simpleLoess(y, x, w, span, degree = degree, parametric = parametric, : radius 2.5e-05
```

```
## Warning in simpleLoess(y, x, w, span, degree = degree, parametric = parametric, : all data on boundary of neighborhood. make span bigger
```

```
## Warning in simpleLoess(y, x, w, span, degree = degree, parametric = parametric, : pseudoinverse used at -0.005
```

```
## Warning in simpleLoess(y, x, w, span, degree = degree, parametric = parametric, : neighborhood radius 0.005
```

```
## Warning in simpleLoess(y, x, w, span, degree = degree, parametric = parametric, : reciprocal condition number 1
```

```
## Warning in simpleLoess(y, x, w, span, degree = degree, parametric = parametric, : There are other near singularities as well. 1.01
```

```
## Warning in simpleLoess(y, x, w, span, degree = degree, parametric = parametric, : zero-width neighborhood. make span bigger
```

```
## Warning: Computation failed in `stat_smooth()`:
## NA/NaN/Inf in foreign function call (arg 5)
```

```
## Warning in simpleLoess(y, x, w, span, degree = degree, parametric = parametric, : at -0.005
```

```
## Warning in simpleLoess(y, x, w, span, degree = degree, parametric = parametric, : radius 2.5e-05
```

```
## Warning in simpleLoess(y, x, w, span, degree = degree, parametric = parametric, : all data on boundary of neighborhood. make span bigger
```

```
## Warning in simpleLoess(y, x, w, span, degree = degree, parametric = parametric, : pseudoinverse used at -0.005
```

```
## Warning in simpleLoess(y, x, w, span, degree = degree, parametric = parametric, : neighborhood radius 0.005
```

```
## Warning in simpleLoess(y, x, w, span, degree = degree, parametric = parametric, : reciprocal condition number 1
```

```
## Warning in simpleLoess(y, x, w, span, degree = degree, parametric = parametric, : There are other near singularities as well. 1.01
```

```
## Warning in simpleLoess(y, x, w, span, degree = degree, parametric = parametric, : zero-width neighborhood. make span bigger
```

```
## Warning: Computation failed in `stat_smooth()`:
## NA/NaN/Inf in foreign function call (arg 5)
```

```
## Warning in simpleLoess(y, x, w, span, degree = degree, parametric = parametric, : at -0.005
```

```
## Warning in simpleLoess(y, x, w, span, degree = degree, parametric = parametric, : radius 2.5e-05
```

```
## Warning in simpleLoess(y, x, w, span, degree = degree, parametric = parametric, : all data on boundary of neighborhood. make span bigger
```

```
## Warning in simpleLoess(y, x, w, span, degree = degree, parametric = parametric, : pseudoinverse used at -0.005
```

```
## Warning in simpleLoess(y, x, w, span, degree = degree, parametric = parametric, : neighborhood radius 0.005
```

```
## Warning in simpleLoess(y, x, w, span, degree = degree, parametric = parametric, : reciprocal condition number 1
```

```
## Warning in simpleLoess(y, x, w, span, degree = degree, parametric = parametric, : There are other near singularities as well. 1.01
```

```
## Warning in simpleLoess(y, x, w, span, degree = degree, parametric = parametric, : zero-width neighborhood. make span bigger
```

```
## Warning: Computation failed in `stat_smooth()`:
## NA/NaN/Inf in foreign function call (arg 5)
```

```
## Warning in simpleLoess(y, x, w, span, degree = degree, parametric = parametric, : at -0.005
```

```
## Warning in simpleLoess(y, x, w, span, degree = degree, parametric = parametric, : radius 2.5e-05
```

```
## Warning in simpleLoess(y, x, w, span, degree = degree, parametric = parametric, : all data on boundary of neighborhood. make span bigger
```

```
## Warning in simpleLoess(y, x, w, span, degree = degree, parametric = parametric, : pseudoinverse used at -0.005
```

```
## Warning in simpleLoess(y, x, w, span, degree = degree, parametric = parametric, : neighborhood radius 0.005
```

```
## Warning in simpleLoess(y, x, w, span, degree = degree, parametric = parametric, : reciprocal condition number 1
```

```
## Warning in simpleLoess(y, x, w, span, degree = degree, parametric = parametric, : There are other near singularities as well. 1.01
```

```
## Warning in simpleLoess(y, x, w, span, degree = degree, parametric = parametric, : zero-width neighborhood. make span bigger
```

```
## Warning: Computation failed in `stat_smooth()`:
## NA/NaN/Inf in foreign function call (arg 5)
```

```
## Warning in simpleLoess(y, x, w, span, degree = degree, parametric = parametric, : at -0.005
```

```
## Warning in simpleLoess(y, x, w, span, degree = degree, parametric = parametric, : radius 2.5e-05
```

```
## Warning in simpleLoess(y, x, w, span, degree = degree, parametric = parametric, : all data on boundary of neighborhood. make span bigger
```

```
## Warning in simpleLoess(y, x, w, span, degree = degree, parametric = parametric, : pseudoinverse used at -0.005
```

```
## Warning in simpleLoess(y, x, w, span, degree = degree, parametric = parametric, : neighborhood radius 0.005
```

```
## Warning in simpleLoess(y, x, w, span, degree = degree, parametric = parametric, : reciprocal condition number 1
```

```
## Warning in simpleLoess(y, x, w, span, degree = degree, parametric = parametric, : There are other near singularities as well. 1.01
```

```
## Warning in simpleLoess(y, x, w, span, degree = degree, parametric = parametric, : zero-width neighborhood. make span bigger
```

```
## Warning: Computation failed in `stat_smooth()`:
## NA/NaN/Inf in foreign function call (arg 5)
```

```
## Warning in simpleLoess(y, x, w, span, degree = degree, parametric = parametric, : at -0.005
```

```
## Warning in simpleLoess(y, x, w, span, degree = degree, parametric = parametric, : radius 2.5e-05
```

```
## Warning in simpleLoess(y, x, w, span, degree = degree, parametric = parametric, : all data on boundary of neighborhood. make span bigger
```

```
## Warning in simpleLoess(y, x, w, span, degree = degree, parametric = parametric, : pseudoinverse used at -0.005
```

```
## Warning in simpleLoess(y, x, w, span, degree = degree, parametric = parametric, : neighborhood radius 0.005
```

```
## Warning in simpleLoess(y, x, w, span, degree = degree, parametric = parametric, : reciprocal condition number 1
```

```
## Warning in simpleLoess(y, x, w, span, degree = degree, parametric = parametric, : There are other near singularities as well. 1.01
```

```
## Warning in simpleLoess(y, x, w, span, degree = degree, parametric = parametric, : zero-width neighborhood. make span bigger
```

```
## Warning: Computation failed in `stat_smooth()`:
## NA/NaN/Inf in foreign function call (arg 5)
```

```
## Warning in simpleLoess(y, x, w, span, degree = degree, parametric = parametric, : pseudoinverse used at -0.005
```

```
## Warning in simpleLoess(y, x, w, span, degree = degree, parametric = parametric, : neighborhood radius 1.005
```

```
## Warning in simpleLoess(y, x, w, span, degree = degree, parametric = parametric, : reciprocal condition number 0
```

```
## Warning in simpleLoess(y, x, w, span, degree = degree, parametric = parametric, : There are other near singularities as well. 1.01
```

```
## Warning in predLoess(object$y, object$x, newx = if (is.null(newdata)) object$x else if (is.data.frame(newdata)) as.matrix(model.frame(delete.response(terms(object)), : pseudoinverse used at -0.005
```

```
## Warning in predLoess(object$y, object$x, newx = if (is.null(newdata)) object$x else if (is.data.frame(newdata)) as.matrix(model.frame(delete.response(terms(object)), : neighborhood radius 1.005
```

```
## Warning in predLoess(object$y, object$x, newx = if (is.null(newdata)) object$x else if (is.data.frame(newdata)) as.matrix(model.frame(delete.response(terms(object)), : reciprocal condition number 0
```

```
## Warning in predLoess(object$y, object$x, newx = if (is.null(newdata)) object$x else if (is.data.frame(newdata)) as.matrix(model.frame(delete.response(terms(object)), : There are other near
## singularities as well. 1.01
```

```
## Warning in simpleLoess(y, x, w, span, degree = degree, parametric = parametric, : at -0.005
```

```
## Warning in simpleLoess(y, x, w, span, degree = degree, parametric = parametric, : radius 2.5e-05
```

```
## Warning in simpleLoess(y, x, w, span, degree = degree, parametric = parametric, : all data on boundary of neighborhood. make span bigger
```

```
## Warning in simpleLoess(y, x, w, span, degree = degree, parametric = parametric, : pseudoinverse used at -0.005
```

```
## Warning in simpleLoess(y, x, w, span, degree = degree, parametric = parametric, : neighborhood radius 0.005
```

```
## Warning in simpleLoess(y, x, w, span, degree = degree, parametric = parametric, : reciprocal condition number 1
```

```
## Warning in simpleLoess(y, x, w, span, degree = degree, parametric = parametric, : There are other near singularities as well. 1.01
```

```
## Warning in simpleLoess(y, x, w, span, degree = degree, parametric = parametric, : zero-width neighborhood. make span bigger
```

```
## Warning: Computation failed in `stat_smooth()`:
## NA/NaN/Inf in foreign function call (arg 5)
```

```
## Warning in simpleLoess(y, x, w, span, degree = degree, parametric = parametric, : at -0.005
```

```
## Warning in simpleLoess(y, x, w, span, degree = degree, parametric = parametric, : radius 2.5e-05
```

```
## Warning in simpleLoess(y, x, w, span, degree = degree, parametric = parametric, : all data on boundary of neighborhood. make span bigger
```

```
## Warning in simpleLoess(y, x, w, span, degree = degree, parametric = parametric, : pseudoinverse used at -0.005
```

```
## Warning in simpleLoess(y, x, w, span, degree = degree, parametric = parametric, : neighborhood radius 0.005
```

```
## Warning in simpleLoess(y, x, w, span, degree = degree, parametric = parametric, : reciprocal condition number 1
```

```
## Warning in simpleLoess(y, x, w, span, degree = degree, parametric = parametric, : There are other near singularities as well. 1.01
```

```
## Warning in simpleLoess(y, x, w, span, degree = degree, parametric = parametric, : zero-width neighborhood. make span bigger
```

```
## Warning: Computation failed in `stat_smooth()`:
## NA/NaN/Inf in foreign function call (arg 5)
```

```
## Warning in simpleLoess(y, x, w, span, degree = degree, parametric = parametric, : at -0.005
```

```
## Warning in simpleLoess(y, x, w, span, degree = degree, parametric = parametric, : radius 2.5e-05
```

```
## Warning in simpleLoess(y, x, w, span, degree = degree, parametric = parametric, : all data on boundary of neighborhood. make span bigger
```

```
## Warning in simpleLoess(y, x, w, span, degree = degree, parametric = parametric, : pseudoinverse used at -0.005
```

```
## Warning in simpleLoess(y, x, w, span, degree = degree, parametric = parametric, : neighborhood radius 0.005
```

```
## Warning in simpleLoess(y, x, w, span, degree = degree, parametric = parametric, : reciprocal condition number 1
```

```
## Warning in simpleLoess(y, x, w, span, degree = degree, parametric = parametric, : There are other near singularities as well. 1.01
```

```
## Warning in simpleLoess(y, x, w, span, degree = degree, parametric = parametric, : zero-width neighborhood. make span bigger
```

```
## Warning: Computation failed in `stat_smooth()`:
## NA/NaN/Inf in foreign function call (arg 5)
```

```
## Warning in simpleLoess(y, x, w, span, degree = degree, parametric = parametric, : at -0.005
```

```
## Warning in simpleLoess(y, x, w, span, degree = degree, parametric = parametric, : radius 2.5e-05
```

```
## Warning in simpleLoess(y, x, w, span, degree = degree, parametric = parametric, : all data on boundary of neighborhood. make span bigger
```

```
## Warning in simpleLoess(y, x, w, span, degree = degree, parametric = parametric, : pseudoinverse used at -0.005
```

```
## Warning in simpleLoess(y, x, w, span, degree = degree, parametric = parametric, : neighborhood radius 0.005
```

```
## Warning in simpleLoess(y, x, w, span, degree = degree, parametric = parametric, : reciprocal condition number 1
```

```
## Warning in simpleLoess(y, x, w, span, degree = degree, parametric = parametric, : There are other near singularities as well. 1.01
```

```
## Warning in simpleLoess(y, x, w, span, degree = degree, parametric = parametric, : zero-width neighborhood. make span bigger
```

```
## Warning: Computation failed in `stat_smooth()`:
## NA/NaN/Inf in foreign function call (arg 5)
```

```
## Warning in simpleLoess(y, x, w, span, degree = degree, parametric = parametric, : at -0.005
```

```
## Warning in simpleLoess(y, x, w, span, degree = degree, parametric = parametric, : radius 2.5e-05
```

```
## Warning in simpleLoess(y, x, w, span, degree = degree, parametric = parametric, : all data on boundary of neighborhood. make span bigger
```

```
## Warning in simpleLoess(y, x, w, span, degree = degree, parametric = parametric, : pseudoinverse used at -0.005
```

```
## Warning in simpleLoess(y, x, w, span, degree = degree, parametric = parametric, : neighborhood radius 0.005
```

```
## Warning in simpleLoess(y, x, w, span, degree = degree, parametric = parametric, : reciprocal condition number 1
```

```
## Warning in simpleLoess(y, x, w, span, degree = degree, parametric = parametric, : There are other near singularities as well. 1.01
```

```
## Warning in simpleLoess(y, x, w, span, degree = degree, parametric = parametric, : zero-width neighborhood. make span bigger
```

```
## Warning: Computation failed in `stat_smooth()`:
## NA/NaN/Inf in foreign function call (arg 5)
```

```
## Warning in simpleLoess(y, x, w, span, degree = degree, parametric = parametric, : at -0.005
```

```
## Warning in simpleLoess(y, x, w, span, degree = degree, parametric = parametric, : radius 2.5e-05
```

```
## Warning in simpleLoess(y, x, w, span, degree = degree, parametric = parametric, : all data on boundary of neighborhood. make span bigger
```

```
## Warning in simpleLoess(y, x, w, span, degree = degree, parametric = parametric, : pseudoinverse used at -0.005
```

```
## Warning in simpleLoess(y, x, w, span, degree = degree, parametric = parametric, : neighborhood radius 0.005
```

```
## Warning in simpleLoess(y, x, w, span, degree = degree, parametric = parametric, : reciprocal condition number 1
```

```
## Warning in simpleLoess(y, x, w, span, degree = degree, parametric = parametric, : There are other near singularities as well. 1.01
```

```
## Warning in simpleLoess(y, x, w, span, degree = degree, parametric = parametric, : zero-width neighborhood. make span bigger
```

```
## Warning: Computation failed in `stat_smooth()`:
## NA/NaN/Inf in foreign function call (arg 5)
```

```
## Warning in simpleLoess(y, x, w, span, degree = degree, parametric = parametric, : at -0.005
```

```
## Warning in simpleLoess(y, x, w, span, degree = degree, parametric = parametric, : radius 2.5e-05
```

```
## Warning in simpleLoess(y, x, w, span, degree = degree, parametric = parametric, : all data on boundary of neighborhood. make span bigger
```

```
## Warning in simpleLoess(y, x, w, span, degree = degree, parametric = parametric, : pseudoinverse used at -0.005
```

```
## Warning in simpleLoess(y, x, w, span, degree = degree, parametric = parametric, : neighborhood radius 0.005
```

```
## Warning in simpleLoess(y, x, w, span, degree = degree, parametric = parametric, : reciprocal condition number 1
```

```
## Warning in simpleLoess(y, x, w, span, degree = degree, parametric = parametric, : There are other near singularities as well. 1.01
```

```
## Warning in simpleLoess(y, x, w, span, degree = degree, parametric = parametric, : zero-width neighborhood. make span bigger
```

```
## Warning: Computation failed in `stat_smooth()`:
## NA/NaN/Inf in foreign function call (arg 5)
```

```
## Warning in simpleLoess(y, x, w, span, degree = degree, parametric = parametric, : at -0.005
```

```
## Warning in simpleLoess(y, x, w, span, degree = degree, parametric = parametric, : radius 2.5e-05
```

```
## Warning in simpleLoess(y, x, w, span, degree = degree, parametric = parametric, : all data on boundary of neighborhood. make span bigger
```

```
## Warning in simpleLoess(y, x, w, span, degree = degree, parametric = parametric, : pseudoinverse used at -0.005
```

```
## Warning in simpleLoess(y, x, w, span, degree = degree, parametric = parametric, : neighborhood radius 0.005
```

```
## Warning in simpleLoess(y, x, w, span, degree = degree, parametric = parametric, : reciprocal condition number 1
```

```
## Warning in simpleLoess(y, x, w, span, degree = degree, parametric = parametric, : There are other near singularities as well. 1.01
```

```
## Warning in simpleLoess(y, x, w, span, degree = degree, parametric = parametric, : zero-width neighborhood. make span bigger
```

```
## Warning: Computation failed in `stat_smooth()`:
## NA/NaN/Inf in foreign function call (arg 5)
```

```
## Warning in simpleLoess(y, x, w, span, degree = degree, parametric = parametric, : at -0.005
```

```
## Warning in simpleLoess(y, x, w, span, degree = degree, parametric = parametric, : radius 2.5e-05
```

```
## Warning in simpleLoess(y, x, w, span, degree = degree, parametric = parametric, : all data on boundary of neighborhood. make span bigger
```

```
## Warning in simpleLoess(y, x, w, span, degree = degree, parametric = parametric, : pseudoinverse used at -0.005
```

```
## Warning in simpleLoess(y, x, w, span, degree = degree, parametric = parametric, : neighborhood radius 0.005
```

```
## Warning in simpleLoess(y, x, w, span, degree = degree, parametric = parametric, : reciprocal condition number 1
```

```
## Warning in simpleLoess(y, x, w, span, degree = degree, parametric = parametric, : There are other near singularities as well. 1.01
```

```
## Warning in simpleLoess(y, x, w, span, degree = degree, parametric = parametric, : zero-width neighborhood. make span bigger
```

```
## Warning: Computation failed in `stat_smooth()`:
## NA/NaN/Inf in foreign function call (arg 5)
```

```
## Warning in simpleLoess(y, x, w, span, degree = degree, parametric = parametric, : pseudoinverse used at -0.005
```

```
## Warning in simpleLoess(y, x, w, span, degree = degree, parametric = parametric, : neighborhood radius 1.005
```

```
## Warning in simpleLoess(y, x, w, span, degree = degree, parametric = parametric, : reciprocal condition number 0
```

```
## Warning in simpleLoess(y, x, w, span, degree = degree, parametric = parametric, : There are other near singularities as well. 1.01
```

```
## Warning in predLoess(object$y, object$x, newx = if (is.null(newdata)) object$x else if (is.data.frame(newdata)) as.matrix(model.frame(delete.response(terms(object)), : pseudoinverse used at -0.005
```

```
## Warning in predLoess(object$y, object$x, newx = if (is.null(newdata)) object$x else if (is.data.frame(newdata)) as.matrix(model.frame(delete.response(terms(object)), : neighborhood radius 1.005
```

```
## Warning in predLoess(object$y, object$x, newx = if (is.null(newdata)) object$x else if (is.data.frame(newdata)) as.matrix(model.frame(delete.response(terms(object)), : reciprocal condition number 0
```

```
## Warning in predLoess(object$y, object$x, newx = if (is.null(newdata)) object$x else if (is.data.frame(newdata)) as.matrix(model.frame(delete.response(terms(object)), : There are other near
## singularities as well. 1.01
```

```
## Warning in simpleLoess(y, x, w, span, degree = degree, parametric = parametric, : at -0.005
```

```
## Warning in simpleLoess(y, x, w, span, degree = degree, parametric = parametric, : radius 2.5e-05
```

```
## Warning in simpleLoess(y, x, w, span, degree = degree, parametric = parametric, : all data on boundary of neighborhood. make span bigger
```

```
## Warning in simpleLoess(y, x, w, span, degree = degree, parametric = parametric, : pseudoinverse used at -0.005
```

```
## Warning in simpleLoess(y, x, w, span, degree = degree, parametric = parametric, : neighborhood radius 0.005
```

```
## Warning in simpleLoess(y, x, w, span, degree = degree, parametric = parametric, : reciprocal condition number 1
```

```
## Warning in simpleLoess(y, x, w, span, degree = degree, parametric = parametric, : There are other near singularities as well. 1.01
```

```
## Warning in simpleLoess(y, x, w, span, degree = degree, parametric = parametric, : zero-width neighborhood. make span bigger
```

```
## Warning: Computation failed in `stat_smooth()`:
## NA/NaN/Inf in foreign function call (arg 5)
```

```
## Warning in simpleLoess(y, x, w, span, degree = degree, parametric = parametric, : at -0.005
```

```
## Warning in simpleLoess(y, x, w, span, degree = degree, parametric = parametric, : radius 2.5e-05
```

```
## Warning in simpleLoess(y, x, w, span, degree = degree, parametric = parametric, : all data on boundary of neighborhood. make span bigger
```

```
## Warning in simpleLoess(y, x, w, span, degree = degree, parametric = parametric, : pseudoinverse used at -0.005
```

```
## Warning in simpleLoess(y, x, w, span, degree = degree, parametric = parametric, : neighborhood radius 0.005
```

```
## Warning in simpleLoess(y, x, w, span, degree = degree, parametric = parametric, : reciprocal condition number 1
```

```
## Warning in simpleLoess(y, x, w, span, degree = degree, parametric = parametric, : There are other near singularities as well. 1.01
```

```
## Warning in simpleLoess(y, x, w, span, degree = degree, parametric = parametric, : zero-width neighborhood. make span bigger
```

```
## Warning: Computation failed in `stat_smooth()`:
## NA/NaN/Inf in foreign function call (arg 5)
```

```
## Warning in simpleLoess(y, x, w, span, degree = degree, parametric = parametric, : at -0.005
```

```
## Warning in simpleLoess(y, x, w, span, degree = degree, parametric = parametric, : radius 2.5e-05
```

```
## Warning in simpleLoess(y, x, w, span, degree = degree, parametric = parametric, : all data on boundary of neighborhood. make span bigger
```

```
## Warning in simpleLoess(y, x, w, span, degree = degree, parametric = parametric, : pseudoinverse used at -0.005
```

```
## Warning in simpleLoess(y, x, w, span, degree = degree, parametric = parametric, : neighborhood radius 0.005
```

```
## Warning in simpleLoess(y, x, w, span, degree = degree, parametric = parametric, : reciprocal condition number 1
```

```
## Warning in simpleLoess(y, x, w, span, degree = degree, parametric = parametric, : There are other near singularities as well. 1.01
```

```
## Warning in simpleLoess(y, x, w, span, degree = degree, parametric = parametric, : zero-width neighborhood. make span bigger
```

```
## Warning: Computation failed in `stat_smooth()`:
## NA/NaN/Inf in foreign function call (arg 5)
```

```
## Warning in simpleLoess(y, x, w, span, degree = degree, parametric = parametric, : at -0.005
```

```
## Warning in simpleLoess(y, x, w, span, degree = degree, parametric = parametric, : radius 2.5e-05
```

```
## Warning in simpleLoess(y, x, w, span, degree = degree, parametric = parametric, : all data on boundary of neighborhood. make span bigger
```

```
## Warning in simpleLoess(y, x, w, span, degree = degree, parametric = parametric, : pseudoinverse used at -0.005
```

```
## Warning in simpleLoess(y, x, w, span, degree = degree, parametric = parametric, : neighborhood radius 0.005
```

```
## Warning in simpleLoess(y, x, w, span, degree = degree, parametric = parametric, : reciprocal condition number 1
```

```
## Warning in simpleLoess(y, x, w, span, degree = degree, parametric = parametric, : There are other near singularities as well. 1.01
```

```
## Warning in simpleLoess(y, x, w, span, degree = degree, parametric = parametric, : zero-width neighborhood. make span bigger
```

```
## Warning: Computation failed in `stat_smooth()`:
## NA/NaN/Inf in foreign function call (arg 5)
```

```
## Warning in simpleLoess(y, x, w, span, degree = degree, parametric = parametric, : at -0.005
```

```
## Warning in simpleLoess(y, x, w, span, degree = degree, parametric = parametric, : radius 2.5e-05
```

```
## Warning in simpleLoess(y, x, w, span, degree = degree, parametric = parametric, : all data on boundary of neighborhood. make span bigger
```

```
## Warning in simpleLoess(y, x, w, span, degree = degree, parametric = parametric, : pseudoinverse used at -0.005
```

```
## Warning in simpleLoess(y, x, w, span, degree = degree, parametric = parametric, : neighborhood radius 0.005
```

```
## Warning in simpleLoess(y, x, w, span, degree = degree, parametric = parametric, : reciprocal condition number 1
```

```
## Warning in simpleLoess(y, x, w, span, degree = degree, parametric = parametric, : There are other near singularities as well. 1.01
```

```
## Warning in simpleLoess(y, x, w, span, degree = degree, parametric = parametric, : zero-width neighborhood. make span bigger
```

```
## Warning: Computation failed in `stat_smooth()`:
## NA/NaN/Inf in foreign function call (arg 5)
```

```
## Warning in simpleLoess(y, x, w, span, degree = degree, parametric = parametric, : at -0.005
```

```
## Warning in simpleLoess(y, x, w, span, degree = degree, parametric = parametric, : radius 2.5e-05
```

```
## Warning in simpleLoess(y, x, w, span, degree = degree, parametric = parametric, : all data on boundary of neighborhood. make span bigger
```

```
## Warning in simpleLoess(y, x, w, span, degree = degree, parametric = parametric, : pseudoinverse used at -0.005
```

```
## Warning in simpleLoess(y, x, w, span, degree = degree, parametric = parametric, : neighborhood radius 0.005
```

```
## Warning in simpleLoess(y, x, w, span, degree = degree, parametric = parametric, : reciprocal condition number 1
```

```
## Warning in simpleLoess(y, x, w, span, degree = degree, parametric = parametric, : There are other near singularities as well. 1.01
```

```
## Warning in simpleLoess(y, x, w, span, degree = degree, parametric = parametric, : zero-width neighborhood. make span bigger
```

```
## Warning: Computation failed in `stat_smooth()`:
## NA/NaN/Inf in foreign function call (arg 5)
```

```
## Warning in simpleLoess(y, x, w, span, degree = degree, parametric = parametric, : at -0.005
```

```
## Warning in simpleLoess(y, x, w, span, degree = degree, parametric = parametric, : radius 2.5e-05
```

```
## Warning in simpleLoess(y, x, w, span, degree = degree, parametric = parametric, : all data on boundary of neighborhood. make span bigger
```

```
## Warning in simpleLoess(y, x, w, span, degree = degree, parametric = parametric, : pseudoinverse used at -0.005
```

```
## Warning in simpleLoess(y, x, w, span, degree = degree, parametric = parametric, : neighborhood radius 0.005
```

```
## Warning in simpleLoess(y, x, w, span, degree = degree, parametric = parametric, : reciprocal condition number 1
```

```
## Warning in simpleLoess(y, x, w, span, degree = degree, parametric = parametric, : There are other near singularities as well. 1.01
```

```
## Warning in simpleLoess(y, x, w, span, degree = degree, parametric = parametric, : zero-width neighborhood. make span bigger
```

```
## Warning: Computation failed in `stat_smooth()`:
## NA/NaN/Inf in foreign function call (arg 5)
```

```
## Warning in simpleLoess(y, x, w, span, degree = degree, parametric = parametric, : at -0.005
```

```
## Warning in simpleLoess(y, x, w, span, degree = degree, parametric = parametric, : radius 2.5e-05
```

```
## Warning in simpleLoess(y, x, w, span, degree = degree, parametric = parametric, : all data on boundary of neighborhood. make span bigger
```

```
## Warning in simpleLoess(y, x, w, span, degree = degree, parametric = parametric, : pseudoinverse used at -0.005
```

```
## Warning in simpleLoess(y, x, w, span, degree = degree, parametric = parametric, : neighborhood radius 0.005
```

```
## Warning in simpleLoess(y, x, w, span, degree = degree, parametric = parametric, : reciprocal condition number 1
```

```
## Warning in simpleLoess(y, x, w, span, degree = degree, parametric = parametric, : There are other near singularities as well. 1.01
```

```
## Warning in simpleLoess(y, x, w, span, degree = degree, parametric = parametric, : zero-width neighborhood. make span bigger
```

```
## Warning: Computation failed in `stat_smooth()`:
## NA/NaN/Inf in foreign function call (arg 5)
```

```
## Warning in simpleLoess(y, x, w, span, degree = degree, parametric = parametric, : at -0.005
```

```
## Warning in simpleLoess(y, x, w, span, degree = degree, parametric = parametric, : radius 2.5e-05
```

```
## Warning in simpleLoess(y, x, w, span, degree = degree, parametric = parametric, : all data on boundary of neighborhood. make span bigger
```

```
## Warning in simpleLoess(y, x, w, span, degree = degree, parametric = parametric, : pseudoinverse used at -0.005
```

```
## Warning in simpleLoess(y, x, w, span, degree = degree, parametric = parametric, : neighborhood radius 0.005
```

```
## Warning in simpleLoess(y, x, w, span, degree = degree, parametric = parametric, : reciprocal condition number 1
```

```
## Warning in simpleLoess(y, x, w, span, degree = degree, parametric = parametric, : There are other near singularities as well. 1.01
```

```
## Warning in simpleLoess(y, x, w, span, degree = degree, parametric = parametric, : zero-width neighborhood. make span bigger
```

```
## Warning: Computation failed in `stat_smooth()`:
## NA/NaN/Inf in foreign function call (arg 5)
```

```
## Warning in simpleLoess(y, x, w, span, degree = degree, parametric = parametric, : at -0.005
```

```
## Warning in simpleLoess(y, x, w, span, degree = degree, parametric = parametric, : radius 2.5e-05
```

```
## Warning in simpleLoess(y, x, w, span, degree = degree, parametric = parametric, : all data on boundary of neighborhood. make span bigger
```

```
## Warning in simpleLoess(y, x, w, span, degree = degree, parametric = parametric, : pseudoinverse used at -0.005
```

```
## Warning in simpleLoess(y, x, w, span, degree = degree, parametric = parametric, : neighborhood radius 0.005
```

```
## Warning in simpleLoess(y, x, w, span, degree = degree, parametric = parametric, : reciprocal condition number 1
```

```
## Warning in simpleLoess(y, x, w, span, degree = degree, parametric = parametric, : There are other near singularities as well. 1.01
```

```
## Warning in simpleLoess(y, x, w, span, degree = degree, parametric = parametric, : zero-width neighborhood. make span bigger
```

```
## Warning: Computation failed in `stat_smooth()`:
## NA/NaN/Inf in foreign function call (arg 5)
```

```
## Warning in simpleLoess(y, x, w, span, degree = degree, parametric = parametric, : at -0.005
```

```
## Warning in simpleLoess(y, x, w, span, degree = degree, parametric = parametric, : radius 2.5e-05
```

```
## Warning in simpleLoess(y, x, w, span, degree = degree, parametric = parametric, : all data on boundary of neighborhood. make span bigger
```

```
## Warning in simpleLoess(y, x, w, span, degree = degree, parametric = parametric, : pseudoinverse used at -0.005
```

```
## Warning in simpleLoess(y, x, w, span, degree = degree, parametric = parametric, : neighborhood radius 0.005
```

```
## Warning in simpleLoess(y, x, w, span, degree = degree, parametric = parametric, : reciprocal condition number 1
```

```
## Warning in simpleLoess(y, x, w, span, degree = degree, parametric = parametric, : There are other near singularities as well. 1.01
```

```
## Warning in simpleLoess(y, x, w, span, degree = degree, parametric = parametric, : zero-width neighborhood. make span bigger
```

```
## Warning: Computation failed in `stat_smooth()`:
## NA/NaN/Inf in foreign function call (arg 5)
```

```
## Warning in simpleLoess(y, x, w, span, degree = degree, parametric = parametric, : at -0.005
```

```
## Warning in simpleLoess(y, x, w, span, degree = degree, parametric = parametric, : radius 2.5e-05
```

```
## Warning in simpleLoess(y, x, w, span, degree = degree, parametric = parametric, : all data on boundary of neighborhood. make span bigger
```

```
## Warning in simpleLoess(y, x, w, span, degree = degree, parametric = parametric, : pseudoinverse used at -0.005
```

```
## Warning in simpleLoess(y, x, w, span, degree = degree, parametric = parametric, : neighborhood radius 0.005
```

```
## Warning in simpleLoess(y, x, w, span, degree = degree, parametric = parametric, : reciprocal condition number 1
```

```
## Warning in simpleLoess(y, x, w, span, degree = degree, parametric = parametric, : There are other near singularities as well. 1.01
```

```
## Warning in simpleLoess(y, x, w, span, degree = degree, parametric = parametric, : zero-width neighborhood. make span bigger
```

```
## Warning: Computation failed in `stat_smooth()`:
## NA/NaN/Inf in foreign function call (arg 5)
```

```
## Warning in simpleLoess(y, x, w, span, degree = degree, parametric = parametric, : at -0.005
```

```
## Warning in simpleLoess(y, x, w, span, degree = degree, parametric = parametric, : radius 2.5e-05
```

```
## Warning in simpleLoess(y, x, w, span, degree = degree, parametric = parametric, : all data on boundary of neighborhood. make span bigger
```

```
## Warning in simpleLoess(y, x, w, span, degree = degree, parametric = parametric, : pseudoinverse used at -0.005
```

```
## Warning in simpleLoess(y, x, w, span, degree = degree, parametric = parametric, : neighborhood radius 0.005
```

```
## Warning in simpleLoess(y, x, w, span, degree = degree, parametric = parametric, : reciprocal condition number 1
```

```
## Warning in simpleLoess(y, x, w, span, degree = degree, parametric = parametric, : There are other near singularities as well. 1.01
```

```
## Warning in simpleLoess(y, x, w, span, degree = degree, parametric = parametric, : zero-width neighborhood. make span bigger
```

```
## Warning: Computation failed in `stat_smooth()`:
## NA/NaN/Inf in foreign function call (arg 5)
```

```
## Warning in simpleLoess(y, x, w, span, degree = degree, parametric = parametric, : pseudoinverse used at -0.005
```

```
## Warning in simpleLoess(y, x, w, span, degree = degree, parametric = parametric, : neighborhood radius 1.005
```

```
## Warning in simpleLoess(y, x, w, span, degree = degree, parametric = parametric, : reciprocal condition number 0
```

```
## Warning in simpleLoess(y, x, w, span, degree = degree, parametric = parametric, : There are other near singularities as well. 1.01
```

```
## Warning in predLoess(object$y, object$x, newx = if (is.null(newdata)) object$x else if (is.data.frame(newdata)) as.matrix(model.frame(delete.response(terms(object)), : pseudoinverse used at -0.005
```

```
## Warning in predLoess(object$y, object$x, newx = if (is.null(newdata)) object$x else if (is.data.frame(newdata)) as.matrix(model.frame(delete.response(terms(object)), : neighborhood radius 1.005
```

```
## Warning in predLoess(object$y, object$x, newx = if (is.null(newdata)) object$x else if (is.data.frame(newdata)) as.matrix(model.frame(delete.response(terms(object)), : reciprocal condition number 0
```

```
## Warning in predLoess(object$y, object$x, newx = if (is.null(newdata)) object$x else if (is.data.frame(newdata)) as.matrix(model.frame(delete.response(terms(object)), : There are other near
## singularities as well. 1.01
```

```
## Warning in simpleLoess(y, x, w, span, degree = degree, parametric = parametric, : at -0.005
```

```
## Warning in simpleLoess(y, x, w, span, degree = degree, parametric = parametric, : radius 2.5e-05
```

```
## Warning in simpleLoess(y, x, w, span, degree = degree, parametric = parametric, : all data on boundary of neighborhood. make span bigger
```

```
## Warning in simpleLoess(y, x, w, span, degree = degree, parametric = parametric, : pseudoinverse used at -0.005
```

```
## Warning in simpleLoess(y, x, w, span, degree = degree, parametric = parametric, : neighborhood radius 0.005
```

```
## Warning in simpleLoess(y, x, w, span, degree = degree, parametric = parametric, : reciprocal condition number 1
```

```
## Warning in simpleLoess(y, x, w, span, degree = degree, parametric = parametric, : There are other near singularities as well. 1.01
```

```
## Warning in simpleLoess(y, x, w, span, degree = degree, parametric = parametric, : zero-width neighborhood. make span bigger
```

```
## Warning: Computation failed in `stat_smooth()`:
## NA/NaN/Inf in foreign function call (arg 5)
```

```
## Warning in simpleLoess(y, x, w, span, degree = degree, parametric = parametric, : at -0.005
```

```
## Warning in simpleLoess(y, x, w, span, degree = degree, parametric = parametric, : radius 2.5e-05
```

```
## Warning in simpleLoess(y, x, w, span, degree = degree, parametric = parametric, : all data on boundary of neighborhood. make span bigger
```

```
## Warning in simpleLoess(y, x, w, span, degree = degree, parametric = parametric, : pseudoinverse used at -0.005
```

```
## Warning in simpleLoess(y, x, w, span, degree = degree, parametric = parametric, : neighborhood radius 0.005
```

```
## Warning in simpleLoess(y, x, w, span, degree = degree, parametric = parametric, : reciprocal condition number 1
```

```
## Warning in simpleLoess(y, x, w, span, degree = degree, parametric = parametric, : There are other near singularities as well. 1.01
```

```
## Warning in simpleLoess(y, x, w, span, degree = degree, parametric = parametric, : zero-width neighborhood. make span bigger
```

```
## Warning: Computation failed in `stat_smooth()`:
## NA/NaN/Inf in foreign function call (arg 5)
```

```
## Warning in simpleLoess(y, x, w, span, degree = degree, parametric = parametric, : at -0.005
```

```
## Warning in simpleLoess(y, x, w, span, degree = degree, parametric = parametric, : radius 2.5e-05
```

```
## Warning in simpleLoess(y, x, w, span, degree = degree, parametric = parametric, : all data on boundary of neighborhood. make span bigger
```

```
## Warning in simpleLoess(y, x, w, span, degree = degree, parametric = parametric, : pseudoinverse used at -0.005
```

```
## Warning in simpleLoess(y, x, w, span, degree = degree, parametric = parametric, : neighborhood radius 0.005
```

```
## Warning in simpleLoess(y, x, w, span, degree = degree, parametric = parametric, : reciprocal condition number 1
```

```
## Warning in simpleLoess(y, x, w, span, degree = degree, parametric = parametric, : There are other near singularities as well. 1.01
```

```
## Warning in simpleLoess(y, x, w, span, degree = degree, parametric = parametric, : zero-width neighborhood. make span bigger
```

```
## Warning: Computation failed in `stat_smooth()`:
## NA/NaN/Inf in foreign function call (arg 5)
```

```
## Warning in simpleLoess(y, x, w, span, degree = degree, parametric = parametric, : at -0.005
```

```
## Warning in simpleLoess(y, x, w, span, degree = degree, parametric = parametric, : radius 2.5e-05
```

```
## Warning in simpleLoess(y, x, w, span, degree = degree, parametric = parametric, : all data on boundary of neighborhood. make span bigger
```

```
## Warning in simpleLoess(y, x, w, span, degree = degree, parametric = parametric, : pseudoinverse used at -0.005
```

```
## Warning in simpleLoess(y, x, w, span, degree = degree, parametric = parametric, : neighborhood radius 0.005
```

```
## Warning in simpleLoess(y, x, w, span, degree = degree, parametric = parametric, : reciprocal condition number 1
```

```
## Warning in simpleLoess(y, x, w, span, degree = degree, parametric = parametric, : There are other near singularities as well. 1.01
```

```
## Warning in simpleLoess(y, x, w, span, degree = degree, parametric = parametric, : zero-width neighborhood. make span bigger
```

```
## Warning: Computation failed in `stat_smooth()`:
## NA/NaN/Inf in foreign function call (arg 5)
```

```
## Warning in simpleLoess(y, x, w, span, degree = degree, parametric = parametric, : at -0.005
```

```
## Warning in simpleLoess(y, x, w, span, degree = degree, parametric = parametric, : radius 2.5e-05
```

```
## Warning in simpleLoess(y, x, w, span, degree = degree, parametric = parametric, : all data on boundary of neighborhood. make span bigger
```

```
## Warning in simpleLoess(y, x, w, span, degree = degree, parametric = parametric, : pseudoinverse used at -0.005
```

```
## Warning in simpleLoess(y, x, w, span, degree = degree, parametric = parametric, : neighborhood radius 0.005
```

```
## Warning in simpleLoess(y, x, w, span, degree = degree, parametric = parametric, : reciprocal condition number 1
```

```
## Warning in simpleLoess(y, x, w, span, degree = degree, parametric = parametric, : There are other near singularities as well. 1.01
```

```
## Warning in simpleLoess(y, x, w, span, degree = degree, parametric = parametric, : zero-width neighborhood. make span bigger
```

```
## Warning: Computation failed in `stat_smooth()`:
## NA/NaN/Inf in foreign function call (arg 5)
```

```
## Warning in simpleLoess(y, x, w, span, degree = degree, parametric = parametric, : at -0.005
```

```
## Warning in simpleLoess(y, x, w, span, degree = degree, parametric = parametric, : radius 2.5e-05
```

```
## Warning in simpleLoess(y, x, w, span, degree = degree, parametric = parametric, : all data on boundary of neighborhood. make span bigger
```

```
## Warning in simpleLoess(y, x, w, span, degree = degree, parametric = parametric, : pseudoinverse used at -0.005
```

```
## Warning in simpleLoess(y, x, w, span, degree = degree, parametric = parametric, : neighborhood radius 0.005
```

```
## Warning in simpleLoess(y, x, w, span, degree = degree, parametric = parametric, : reciprocal condition number 1
```

```
## Warning in simpleLoess(y, x, w, span, degree = degree, parametric = parametric, : There are other near singularities as well. 1.01
```

```
## Warning in simpleLoess(y, x, w, span, degree = degree, parametric = parametric, : zero-width neighborhood. make span bigger
```

```
## Warning: Computation failed in `stat_smooth()`:
## NA/NaN/Inf in foreign function call (arg 5)
```

```
## Warning in simpleLoess(y, x, w, span, degree = degree, parametric = parametric, : at -0.005
```

```
## Warning in simpleLoess(y, x, w, span, degree = degree, parametric = parametric, : radius 2.5e-05
```

```
## Warning in simpleLoess(y, x, w, span, degree = degree, parametric = parametric, : all data on boundary of neighborhood. make span bigger
```

```
## Warning in simpleLoess(y, x, w, span, degree = degree, parametric = parametric, : pseudoinverse used at -0.005
```

```
## Warning in simpleLoess(y, x, w, span, degree = degree, parametric = parametric, : neighborhood radius 0.005
```

```
## Warning in simpleLoess(y, x, w, span, degree = degree, parametric = parametric, : reciprocal condition number 1
```

```
## Warning in simpleLoess(y, x, w, span, degree = degree, parametric = parametric, : There are other near singularities as well. 1.01
```

```
## Warning in simpleLoess(y, x, w, span, degree = degree, parametric = parametric, : zero-width neighborhood. make span bigger
```

```
## Warning: Computation failed in `stat_smooth()`:
## NA/NaN/Inf in foreign function call (arg 5)
```

```
## Warning in simpleLoess(y, x, w, span, degree = degree, parametric = parametric, : at -0.005
```

```
## Warning in simpleLoess(y, x, w, span, degree = degree, parametric = parametric, : radius 2.5e-05
```

```
## Warning in simpleLoess(y, x, w, span, degree = degree, parametric = parametric, : all data on boundary of neighborhood. make span bigger
```

```
## Warning in simpleLoess(y, x, w, span, degree = degree, parametric = parametric, : pseudoinverse used at -0.005
```

```
## Warning in simpleLoess(y, x, w, span, degree = degree, parametric = parametric, : neighborhood radius 0.005
```

```
## Warning in simpleLoess(y, x, w, span, degree = degree, parametric = parametric, : reciprocal condition number 1
```

```
## Warning in simpleLoess(y, x, w, span, degree = degree, parametric = parametric, : There are other near singularities as well. 1.01
```

```
## Warning in simpleLoess(y, x, w, span, degree = degree, parametric = parametric, : zero-width neighborhood. make span bigger
```

```
## Warning: Computation failed in `stat_smooth()`:
## NA/NaN/Inf in foreign function call (arg 5)
```

```
## Warning in simpleLoess(y, x, w, span, degree = degree, parametric = parametric, : at -0.005
```

```
## Warning in simpleLoess(y, x, w, span, degree = degree, parametric = parametric, : radius 2.5e-05
```

```
## Warning in simpleLoess(y, x, w, span, degree = degree, parametric = parametric, : all data on boundary of neighborhood. make span bigger
```

```
## Warning in simpleLoess(y, x, w, span, degree = degree, parametric = parametric, : pseudoinverse used at -0.005
```

```
## Warning in simpleLoess(y, x, w, span, degree = degree, parametric = parametric, : neighborhood radius 0.005
```

```
## Warning in simpleLoess(y, x, w, span, degree = degree, parametric = parametric, : reciprocal condition number 1
```

```
## Warning in simpleLoess(y, x, w, span, degree = degree, parametric = parametric, : There are other near singularities as well. 1.01
```

```
## Warning in simpleLoess(y, x, w, span, degree = degree, parametric = parametric, : zero-width neighborhood. make span bigger
```

```
## Warning: Computation failed in `stat_smooth()`:
## NA/NaN/Inf in foreign function call (arg 5)
```

```
## Warning in simpleLoess(y, x, w, span, degree = degree, parametric = parametric, : at -0.005
```

```
## Warning in simpleLoess(y, x, w, span, degree = degree, parametric = parametric, : radius 2.5e-05
```

```
## Warning in simpleLoess(y, x, w, span, degree = degree, parametric = parametric, : all data on boundary of neighborhood. make span bigger
```

```
## Warning in simpleLoess(y, x, w, span, degree = degree, parametric = parametric, : pseudoinverse used at -0.005
```

```
## Warning in simpleLoess(y, x, w, span, degree = degree, parametric = parametric, : neighborhood radius 0.005
```

```
## Warning in simpleLoess(y, x, w, span, degree = degree, parametric = parametric, : reciprocal condition number 1
```

```
## Warning in simpleLoess(y, x, w, span, degree = degree, parametric = parametric, : There are other near singularities as well. 1.01
```

```
## Warning in simpleLoess(y, x, w, span, degree = degree, parametric = parametric, : zero-width neighborhood. make span bigger
```

```
## Warning: Computation failed in `stat_smooth()`:
## NA/NaN/Inf in foreign function call (arg 5)
```

```
## Warning in simpleLoess(y, x, w, span, degree = degree, parametric = parametric, : at -0.005
```

```
## Warning in simpleLoess(y, x, w, span, degree = degree, parametric = parametric, : radius 2.5e-05
```

```
## Warning in simpleLoess(y, x, w, span, degree = degree, parametric = parametric, : all data on boundary of neighborhood. make span bigger
```

```
## Warning in simpleLoess(y, x, w, span, degree = degree, parametric = parametric, : pseudoinverse used at -0.005
```

```
## Warning in simpleLoess(y, x, w, span, degree = degree, parametric = parametric, : neighborhood radius 0.005
```

```
## Warning in simpleLoess(y, x, w, span, degree = degree, parametric = parametric, : reciprocal condition number 1
```

```
## Warning in simpleLoess(y, x, w, span, degree = degree, parametric = parametric, : There are other near singularities as well. 1.01
```

```
## Warning in simpleLoess(y, x, w, span, degree = degree, parametric = parametric, : zero-width neighborhood. make span bigger
```

```
## Warning: Computation failed in `stat_smooth()`:
## NA/NaN/Inf in foreign function call (arg 5)
```

```
## Warning in simpleLoess(y, x, w, span, degree = degree, parametric = parametric, : pseudoinverse used at -0.005
```

```
## Warning in simpleLoess(y, x, w, span, degree = degree, parametric = parametric, : neighborhood radius 1.005
```

```
## Warning in simpleLoess(y, x, w, span, degree = degree, parametric = parametric, : reciprocal condition number 0
```

```
## Warning in simpleLoess(y, x, w, span, degree = degree, parametric = parametric, : There are other near singularities as well. 1.01
```

```
## Warning in predLoess(object$y, object$x, newx = if (is.null(newdata)) object$x else if (is.data.frame(newdata)) as.matrix(model.frame(delete.response(terms(object)), : pseudoinverse used at -0.005
```

```
## Warning in predLoess(object$y, object$x, newx = if (is.null(newdata)) object$x else if (is.data.frame(newdata)) as.matrix(model.frame(delete.response(terms(object)), : neighborhood radius 1.005
```

```
## Warning in predLoess(object$y, object$x, newx = if (is.null(newdata)) object$x else if (is.data.frame(newdata)) as.matrix(model.frame(delete.response(terms(object)), : reciprocal condition number 0
```

```
## Warning in predLoess(object$y, object$x, newx = if (is.null(newdata)) object$x else if (is.data.frame(newdata)) as.matrix(model.frame(delete.response(terms(object)), : There are other near
## singularities as well. 1.01
```

```
## Warning in simpleLoess(y, x, w, span, degree = degree, parametric = parametric, : at -0.005
```

```
## Warning in simpleLoess(y, x, w, span, degree = degree, parametric = parametric, : radius 2.5e-05
```

```
## Warning in simpleLoess(y, x, w, span, degree = degree, parametric = parametric, : all data on boundary of neighborhood. make span bigger
```

```
## Warning in simpleLoess(y, x, w, span, degree = degree, parametric = parametric, : pseudoinverse used at -0.005
```

```
## Warning in simpleLoess(y, x, w, span, degree = degree, parametric = parametric, : neighborhood radius 0.005
```

```
## Warning in simpleLoess(y, x, w, span, degree = degree, parametric = parametric, : reciprocal condition number 1
```

```
## Warning in simpleLoess(y, x, w, span, degree = degree, parametric = parametric, : There are other near singularities as well. 1.01
```

```
## Warning in simpleLoess(y, x, w, span, degree = degree, parametric = parametric, : zero-width neighborhood. make span bigger
```

```
## Warning: Computation failed in `stat_smooth()`:
## NA/NaN/Inf in foreign function call (arg 5)
```

```
## Warning in simpleLoess(y, x, w, span, degree = degree, parametric = parametric, : at -0.005
```

```
## Warning in simpleLoess(y, x, w, span, degree = degree, parametric = parametric, : radius 2.5e-05
```

```
## Warning in simpleLoess(y, x, w, span, degree = degree, parametric = parametric, : all data on boundary of neighborhood. make span bigger
```

```
## Warning in simpleLoess(y, x, w, span, degree = degree, parametric = parametric, : pseudoinverse used at -0.005
```

```
## Warning in simpleLoess(y, x, w, span, degree = degree, parametric = parametric, : neighborhood radius 0.005
```

```
## Warning in simpleLoess(y, x, w, span, degree = degree, parametric = parametric, : reciprocal condition number 1
```

```
## Warning in simpleLoess(y, x, w, span, degree = degree, parametric = parametric, : There are other near singularities as well. 1.01
```

```
## Warning in simpleLoess(y, x, w, span, degree = degree, parametric = parametric, : zero-width neighborhood. make span bigger
```

```
## Warning: Computation failed in `stat_smooth()`:
## NA/NaN/Inf in foreign function call (arg 5)
```

```
## Warning in simpleLoess(y, x, w, span, degree = degree, parametric = parametric, : at -0.005
```

```
## Warning in simpleLoess(y, x, w, span, degree = degree, parametric = parametric, : radius 2.5e-05
```

```
## Warning in simpleLoess(y, x, w, span, degree = degree, parametric = parametric, : all data on boundary of neighborhood. make span bigger
```

```
## Warning in simpleLoess(y, x, w, span, degree = degree, parametric = parametric, : pseudoinverse used at -0.005
```

```
## Warning in simpleLoess(y, x, w, span, degree = degree, parametric = parametric, : neighborhood radius 0.005
```

```
## Warning in simpleLoess(y, x, w, span, degree = degree, parametric = parametric, : reciprocal condition number 1
```

```
## Warning in simpleLoess(y, x, w, span, degree = degree, parametric = parametric, : There are other near singularities as well. 1.01
```

```
## Warning in simpleLoess(y, x, w, span, degree = degree, parametric = parametric, : zero-width neighborhood. make span bigger
```

```
## Warning: Computation failed in `stat_smooth()`:
## NA/NaN/Inf in foreign function call (arg 5)
```

```
## Warning in simpleLoess(y, x, w, span, degree = degree, parametric = parametric, : at -0.005
```

```
## Warning in simpleLoess(y, x, w, span, degree = degree, parametric = parametric, : radius 2.5e-05
```

```
## Warning in simpleLoess(y, x, w, span, degree = degree, parametric = parametric, : all data on boundary of neighborhood. make span bigger
```

```
## Warning in simpleLoess(y, x, w, span, degree = degree, parametric = parametric, : pseudoinverse used at -0.005
```

```
## Warning in simpleLoess(y, x, w, span, degree = degree, parametric = parametric, : neighborhood radius 0.005
```

```
## Warning in simpleLoess(y, x, w, span, degree = degree, parametric = parametric, : reciprocal condition number 1
```

```
## Warning in simpleLoess(y, x, w, span, degree = degree, parametric = parametric, : There are other near singularities as well. 1.01
```

```
## Warning in simpleLoess(y, x, w, span, degree = degree, parametric = parametric, : zero-width neighborhood. make span bigger
```

```
## Warning: Computation failed in `stat_smooth()`:
## NA/NaN/Inf in foreign function call (arg 5)
```

```
## Warning in simpleLoess(y, x, w, span, degree = degree, parametric = parametric, : at -0.005
```

```
## Warning in simpleLoess(y, x, w, span, degree = degree, parametric = parametric, : radius 2.5e-05
```

```
## Warning in simpleLoess(y, x, w, span, degree = degree, parametric = parametric, : all data on boundary of neighborhood. make span bigger
```

```
## Warning in simpleLoess(y, x, w, span, degree = degree, parametric = parametric, : pseudoinverse used at -0.005
```

```
## Warning in simpleLoess(y, x, w, span, degree = degree, parametric = parametric, : neighborhood radius 0.005
```

```
## Warning in simpleLoess(y, x, w, span, degree = degree, parametric = parametric, : reciprocal condition number 1
```

```
## Warning in simpleLoess(y, x, w, span, degree = degree, parametric = parametric, : There are other near singularities as well. 1.01
```

```
## Warning in simpleLoess(y, x, w, span, degree = degree, parametric = parametric, : zero-width neighborhood. make span bigger
```

```
## Warning: Computation failed in `stat_smooth()`:
## NA/NaN/Inf in foreign function call (arg 5)
```

```
## Warning in simpleLoess(y, x, w, span, degree = degree, parametric = parametric, : at -0.005
```

```
## Warning in simpleLoess(y, x, w, span, degree = degree, parametric = parametric, : radius 2.5e-05
```

```
## Warning in simpleLoess(y, x, w, span, degree = degree, parametric = parametric, : all data on boundary of neighborhood. make span bigger
```

```
## Warning in simpleLoess(y, x, w, span, degree = degree, parametric = parametric, : pseudoinverse used at -0.005
```

```
## Warning in simpleLoess(y, x, w, span, degree = degree, parametric = parametric, : neighborhood radius 0.005
```

```
## Warning in simpleLoess(y, x, w, span, degree = degree, parametric = parametric, : reciprocal condition number 1
```

```
## Warning in simpleLoess(y, x, w, span, degree = degree, parametric = parametric, : There are other near singularities as well. 1.01
```

```
## Warning in simpleLoess(y, x, w, span, degree = degree, parametric = parametric, : zero-width neighborhood. make span bigger
```

```
## Warning: Computation failed in `stat_smooth()`:
## NA/NaN/Inf in foreign function call (arg 5)
```

```
## Warning in simpleLoess(y, x, w, span, degree = degree, parametric = parametric, : at -0.005
```

```
## Warning in simpleLoess(y, x, w, span, degree = degree, parametric = parametric, : radius 2.5e-05
```

```
## Warning in simpleLoess(y, x, w, span, degree = degree, parametric = parametric, : all data on boundary of neighborhood. make span bigger
```

```
## Warning in simpleLoess(y, x, w, span, degree = degree, parametric = parametric, : pseudoinverse used at -0.005
```

```
## Warning in simpleLoess(y, x, w, span, degree = degree, parametric = parametric, : neighborhood radius 0.005
```

```
## Warning in simpleLoess(y, x, w, span, degree = degree, parametric = parametric, : reciprocal condition number 1
```

```
## Warning in simpleLoess(y, x, w, span, degree = degree, parametric = parametric, : There are other near singularities as well. 1.01
```

```
## Warning in simpleLoess(y, x, w, span, degree = degree, parametric = parametric, : zero-width neighborhood. make span bigger
```

```
## Warning: Computation failed in `stat_smooth()`:
## NA/NaN/Inf in foreign function call (arg 5)
```

```
## Warning in simpleLoess(y, x, w, span, degree = degree, parametric = parametric, : at -0.005
```

```
## Warning in simpleLoess(y, x, w, span, degree = degree, parametric = parametric, : radius 2.5e-05
```

```
## Warning in simpleLoess(y, x, w, span, degree = degree, parametric = parametric, : all data on boundary of neighborhood. make span bigger
```

```
## Warning in simpleLoess(y, x, w, span, degree = degree, parametric = parametric, : pseudoinverse used at -0.005
```

```
## Warning in simpleLoess(y, x, w, span, degree = degree, parametric = parametric, : neighborhood radius 0.005
```

```
## Warning in simpleLoess(y, x, w, span, degree = degree, parametric = parametric, : reciprocal condition number 1
```

```
## Warning in simpleLoess(y, x, w, span, degree = degree, parametric = parametric, : There are other near singularities as well. 1.01
```

```
## Warning in simpleLoess(y, x, w, span, degree = degree, parametric = parametric, : zero-width neighborhood. make span bigger
```

```
## Warning: Computation failed in `stat_smooth()`:
## NA/NaN/Inf in foreign function call (arg 5)
```

```
## Warning in simpleLoess(y, x, w, span, degree = degree, parametric = parametric, : at -0.005
```

```
## Warning in simpleLoess(y, x, w, span, degree = degree, parametric = parametric, : radius 2.5e-05
```

```
## Warning in simpleLoess(y, x, w, span, degree = degree, parametric = parametric, : all data on boundary of neighborhood. make span bigger
```

```
## Warning in simpleLoess(y, x, w, span, degree = degree, parametric = parametric, : pseudoinverse used at -0.005
```

```
## Warning in simpleLoess(y, x, w, span, degree = degree, parametric = parametric, : neighborhood radius 0.005
```

```
## Warning in simpleLoess(y, x, w, span, degree = degree, parametric = parametric, : reciprocal condition number 1
```

```
## Warning in simpleLoess(y, x, w, span, degree = degree, parametric = parametric, : There are other near singularities as well. 1.01
```

```
## Warning in simpleLoess(y, x, w, span, degree = degree, parametric = parametric, : zero-width neighborhood. make span bigger
```

```
## Warning: Computation failed in `stat_smooth()`:
## NA/NaN/Inf in foreign function call (arg 5)
```

```
## Warning in simpleLoess(y, x, w, span, degree = degree, parametric = parametric, : at -0.005
```

```
## Warning in simpleLoess(y, x, w, span, degree = degree, parametric = parametric, : radius 2.5e-05
```

```
## Warning in simpleLoess(y, x, w, span, degree = degree, parametric = parametric, : all data on boundary of neighborhood. make span bigger
```

```
## Warning in simpleLoess(y, x, w, span, degree = degree, parametric = parametric, : pseudoinverse used at -0.005
```

```
## Warning in simpleLoess(y, x, w, span, degree = degree, parametric = parametric, : neighborhood radius 0.005
```

```
## Warning in simpleLoess(y, x, w, span, degree = degree, parametric = parametric, : reciprocal condition number 1
```

```
## Warning in simpleLoess(y, x, w, span, degree = degree, parametric = parametric, : There are other near singularities as well. 1.01
```

```
## Warning in simpleLoess(y, x, w, span, degree = degree, parametric = parametric, : zero-width neighborhood. make span bigger
```

```
## Warning: Computation failed in `stat_smooth()`:
## NA/NaN/Inf in foreign function call (arg 5)
```

```
## Warning in simpleLoess(y, x, w, span, degree = degree, parametric = parametric, : pseudoinverse used at -0.005
```

```
## Warning in simpleLoess(y, x, w, span, degree = degree, parametric = parametric, : neighborhood radius 1.005
```

```
## Warning in simpleLoess(y, x, w, span, degree = degree, parametric = parametric, : reciprocal condition number 0
```

```
## Warning in simpleLoess(y, x, w, span, degree = degree, parametric = parametric, : There are other near singularities as well. 1.01
```

```
## Warning in predLoess(object$y, object$x, newx = if (is.null(newdata)) object$x else if (is.data.frame(newdata)) as.matrix(model.frame(delete.response(terms(object)), : pseudoinverse used at -0.005
```

```
## Warning in predLoess(object$y, object$x, newx = if (is.null(newdata)) object$x else if (is.data.frame(newdata)) as.matrix(model.frame(delete.response(terms(object)), : neighborhood radius 1.005
```

```
## Warning in predLoess(object$y, object$x, newx = if (is.null(newdata)) object$x else if (is.data.frame(newdata)) as.matrix(model.frame(delete.response(terms(object)), : reciprocal condition number 0
```

```
## Warning in predLoess(object$y, object$x, newx = if (is.null(newdata)) object$x else if (is.data.frame(newdata)) as.matrix(model.frame(delete.response(terms(object)), : There are other near
## singularities as well. 1.01
```

```
## Warning in simpleLoess(y, x, w, span, degree = degree, parametric = parametric, : at -0.005
```

```
## Warning in simpleLoess(y, x, w, span, degree = degree, parametric = parametric, : radius 2.5e-05
```

```
## Warning in simpleLoess(y, x, w, span, degree = degree, parametric = parametric, : all data on boundary of neighborhood. make span bigger
```

```
## Warning in simpleLoess(y, x, w, span, degree = degree, parametric = parametric, : pseudoinverse used at -0.005
```

```
## Warning in simpleLoess(y, x, w, span, degree = degree, parametric = parametric, : neighborhood radius 0.005
```

```
## Warning in simpleLoess(y, x, w, span, degree = degree, parametric = parametric, : reciprocal condition number 1
```

```
## Warning in simpleLoess(y, x, w, span, degree = degree, parametric = parametric, : There are other near singularities as well. 1.01
```

```
## Warning in simpleLoess(y, x, w, span, degree = degree, parametric = parametric, : zero-width neighborhood. make span bigger
```

```
## Warning: Computation failed in `stat_smooth()`:
## NA/NaN/Inf in foreign function call (arg 5)
```

```
## Warning in simpleLoess(y, x, w, span, degree = degree, parametric = parametric, : at -0.005
```

```
## Warning in simpleLoess(y, x, w, span, degree = degree, parametric = parametric, : radius 2.5e-05
```

```
## Warning in simpleLoess(y, x, w, span, degree = degree, parametric = parametric, : all data on boundary of neighborhood. make span bigger
```

```
## Warning in simpleLoess(y, x, w, span, degree = degree, parametric = parametric, : pseudoinverse used at -0.005
```

```
## Warning in simpleLoess(y, x, w, span, degree = degree, parametric = parametric, : neighborhood radius 0.005
```

```
## Warning in simpleLoess(y, x, w, span, degree = degree, parametric = parametric, : reciprocal condition number 1
```

```
## Warning in simpleLoess(y, x, w, span, degree = degree, parametric = parametric, : There are other near singularities as well. 1.01
```

```
## Warning in simpleLoess(y, x, w, span, degree = degree, parametric = parametric, : zero-width neighborhood. make span bigger
```

```
## Warning: Computation failed in `stat_smooth()`:
## NA/NaN/Inf in foreign function call (arg 5)
```

```
## Warning in simpleLoess(y, x, w, span, degree = degree, parametric = parametric, : at -0.005
```

```
## Warning in simpleLoess(y, x, w, span, degree = degree, parametric = parametric, : radius 2.5e-05
```

```
## Warning in simpleLoess(y, x, w, span, degree = degree, parametric = parametric, : all data on boundary of neighborhood. make span bigger
```

```
## Warning in simpleLoess(y, x, w, span, degree = degree, parametric = parametric, : pseudoinverse used at -0.005
```

```
## Warning in simpleLoess(y, x, w, span, degree = degree, parametric = parametric, : neighborhood radius 0.005
```

```
## Warning in simpleLoess(y, x, w, span, degree = degree, parametric = parametric, : reciprocal condition number 1
```

```
## Warning in simpleLoess(y, x, w, span, degree = degree, parametric = parametric, : There are other near singularities as well. 1.01
```

```
## Warning in simpleLoess(y, x, w, span, degree = degree, parametric = parametric, : zero-width neighborhood. make span bigger
```

```
## Warning: Computation failed in `stat_smooth()`:
## NA/NaN/Inf in foreign function call (arg 5)
```

```
## Warning in simpleLoess(y, x, w, span, degree = degree, parametric = parametric, : at -0.005
```

```
## Warning in simpleLoess(y, x, w, span, degree = degree, parametric = parametric, : radius 2.5e-05
```

```
## Warning in simpleLoess(y, x, w, span, degree = degree, parametric = parametric, : all data on boundary of neighborhood. make span bigger
```

```
## Warning in simpleLoess(y, x, w, span, degree = degree, parametric = parametric, : pseudoinverse used at -0.005
```

```
## Warning in simpleLoess(y, x, w, span, degree = degree, parametric = parametric, : neighborhood radius 0.005
```

```
## Warning in simpleLoess(y, x, w, span, degree = degree, parametric = parametric, : reciprocal condition number 1
```

```
## Warning in simpleLoess(y, x, w, span, degree = degree, parametric = parametric, : There are other near singularities as well. 1.01
```

```
## Warning in simpleLoess(y, x, w, span, degree = degree, parametric = parametric, : zero-width neighborhood. make span bigger
```

```
## Warning: Computation failed in `stat_smooth()`:
## NA/NaN/Inf in foreign function call (arg 5)
```

```
## Warning in simpleLoess(y, x, w, span, degree = degree, parametric = parametric, : at -0.005
```

```
## Warning in simpleLoess(y, x, w, span, degree = degree, parametric = parametric, : radius 2.5e-05
```

```
## Warning in simpleLoess(y, x, w, span, degree = degree, parametric = parametric, : all data on boundary of neighborhood. make span bigger
```

```
## Warning in simpleLoess(y, x, w, span, degree = degree, parametric = parametric, : pseudoinverse used at -0.005
```

```
## Warning in simpleLoess(y, x, w, span, degree = degree, parametric = parametric, : neighborhood radius 0.005
```

```
## Warning in simpleLoess(y, x, w, span, degree = degree, parametric = parametric, : reciprocal condition number 1
```

```
## Warning in simpleLoess(y, x, w, span, degree = degree, parametric = parametric, : There are other near singularities as well. 1.01
```

```
## Warning in simpleLoess(y, x, w, span, degree = degree, parametric = parametric, : zero-width neighborhood. make span bigger
```

```
## Warning: Computation failed in `stat_smooth()`:
## NA/NaN/Inf in foreign function call (arg 5)
```

```
## Warning in simpleLoess(y, x, w, span, degree = degree, parametric = parametric, : at -0.005
```

```
## Warning in simpleLoess(y, x, w, span, degree = degree, parametric = parametric, : radius 2.5e-05
```

```
## Warning in simpleLoess(y, x, w, span, degree = degree, parametric = parametric, : all data on boundary of neighborhood. make span bigger
```

```
## Warning in simpleLoess(y, x, w, span, degree = degree, parametric = parametric, : pseudoinverse used at -0.005
```

```
## Warning in simpleLoess(y, x, w, span, degree = degree, parametric = parametric, : neighborhood radius 0.005
```

```
## Warning in simpleLoess(y, x, w, span, degree = degree, parametric = parametric, : reciprocal condition number 1
```

```
## Warning in simpleLoess(y, x, w, span, degree = degree, parametric = parametric, : There are other near singularities as well. 1.01
```

```
## Warning in simpleLoess(y, x, w, span, degree = degree, parametric = parametric, : zero-width neighborhood. make span bigger
```

```
## Warning: Computation failed in `stat_smooth()`:
## NA/NaN/Inf in foreign function call (arg 5)
```

```
## Warning in simpleLoess(y, x, w, span, degree = degree, parametric = parametric, : at -0.005
```

```
## Warning in simpleLoess(y, x, w, span, degree = degree, parametric = parametric, : radius 2.5e-05
```

```
## Warning in simpleLoess(y, x, w, span, degree = degree, parametric = parametric, : all data on boundary of neighborhood. make span bigger
```

```
## Warning in simpleLoess(y, x, w, span, degree = degree, parametric = parametric, : pseudoinverse used at -0.005
```

```
## Warning in simpleLoess(y, x, w, span, degree = degree, parametric = parametric, : neighborhood radius 0.005
```

```
## Warning in simpleLoess(y, x, w, span, degree = degree, parametric = parametric, : reciprocal condition number 1
```

```
## Warning in simpleLoess(y, x, w, span, degree = degree, parametric = parametric, : There are other near singularities as well. 1.01
```

```
## Warning in simpleLoess(y, x, w, span, degree = degree, parametric = parametric, : zero-width neighborhood. make span bigger
```

```
## Warning: Computation failed in `stat_smooth()`:
## NA/NaN/Inf in foreign function call (arg 5)
```

```
## Warning in simpleLoess(y, x, w, span, degree = degree, parametric = parametric, : at -0.005
```

```
## Warning in simpleLoess(y, x, w, span, degree = degree, parametric = parametric, : radius 2.5e-05
```

```
## Warning in simpleLoess(y, x, w, span, degree = degree, parametric = parametric, : all data on boundary of neighborhood. make span bigger
```

```
## Warning in simpleLoess(y, x, w, span, degree = degree, parametric = parametric, : pseudoinverse used at -0.005
```

```
## Warning in simpleLoess(y, x, w, span, degree = degree, parametric = parametric, : neighborhood radius 0.005
```

```
## Warning in simpleLoess(y, x, w, span, degree = degree, parametric = parametric, : reciprocal condition number 1
```

```
## Warning in simpleLoess(y, x, w, span, degree = degree, parametric = parametric, : There are other near singularities as well. 1.01
```

```
## Warning in simpleLoess(y, x, w, span, degree = degree, parametric = parametric, : zero-width neighborhood. make span bigger
```

```
## Warning: Computation failed in `stat_smooth()`:
## NA/NaN/Inf in foreign function call (arg 5)
```

```
## Warning in simpleLoess(y, x, w, span, degree = degree, parametric = parametric, : at -0.005
```

```
## Warning in simpleLoess(y, x, w, span, degree = degree, parametric = parametric, : radius 2.5e-05
```

```
## Warning in simpleLoess(y, x, w, span, degree = degree, parametric = parametric, : all data on boundary of neighborhood. make span bigger
```

```
## Warning in simpleLoess(y, x, w, span, degree = degree, parametric = parametric, : pseudoinverse used at -0.005
```

```
## Warning in simpleLoess(y, x, w, span, degree = degree, parametric = parametric, : neighborhood radius 0.005
```

```
## Warning in simpleLoess(y, x, w, span, degree = degree, parametric = parametric, : reciprocal condition number 1
```

```
## Warning in simpleLoess(y, x, w, span, degree = degree, parametric = parametric, : There are other near singularities as well. 1.01
```

```
## Warning in simpleLoess(y, x, w, span, degree = degree, parametric = parametric, : zero-width neighborhood. make span bigger
```

```
## Warning: Computation failed in `stat_smooth()`:
## NA/NaN/Inf in foreign function call (arg 5)
```

```
## Warning in simpleLoess(y, x, w, span, degree = degree, parametric = parametric, : at -0.005
```

```
## Warning in simpleLoess(y, x, w, span, degree = degree, parametric = parametric, : radius 2.5e-05
```

```
## Warning in simpleLoess(y, x, w, span, degree = degree, parametric = parametric, : all data on boundary of neighborhood. make span bigger
```

```
## Warning in simpleLoess(y, x, w, span, degree = degree, parametric = parametric, : pseudoinverse used at -0.005
```

```
## Warning in simpleLoess(y, x, w, span, degree = degree, parametric = parametric, : neighborhood radius 0.005
```

```
## Warning in simpleLoess(y, x, w, span, degree = degree, parametric = parametric, : reciprocal condition number 1
```

```
## Warning in simpleLoess(y, x, w, span, degree = degree, parametric = parametric, : There are other near singularities as well. 1.01
```

```
## Warning in simpleLoess(y, x, w, span, degree = degree, parametric = parametric, : zero-width neighborhood. make span bigger
```

```
## Warning: Computation failed in `stat_smooth()`:
## NA/NaN/Inf in foreign function call (arg 5)
```

```
## Warning in simpleLoess(y, x, w, span, degree = degree, parametric = parametric, : at -0.005
```

```
## Warning in simpleLoess(y, x, w, span, degree = degree, parametric = parametric, : radius 2.5e-05
```

```
## Warning in simpleLoess(y, x, w, span, degree = degree, parametric = parametric, : all data on boundary of neighborhood. make span bigger
```

```
## Warning in simpleLoess(y, x, w, span, degree = degree, parametric = parametric, : pseudoinverse used at -0.005
```

```
## Warning in simpleLoess(y, x, w, span, degree = degree, parametric = parametric, : neighborhood radius 0.005
```

```
## Warning in simpleLoess(y, x, w, span, degree = degree, parametric = parametric, : reciprocal condition number 1
```

```
## Warning in simpleLoess(y, x, w, span, degree = degree, parametric = parametric, : There are other near singularities as well. 1.01
```

```
## Warning in simpleLoess(y, x, w, span, degree = degree, parametric = parametric, : zero-width neighborhood. make span bigger
```

```
## Warning: Computation failed in `stat_smooth()`:
## NA/NaN/Inf in foreign function call (arg 5)
```

```
## Warning in simpleLoess(y, x, w, span, degree = degree, parametric = parametric, : at -0.005
```

```
## Warning in simpleLoess(y, x, w, span, degree = degree, parametric = parametric, : radius 2.5e-05
```

```
## Warning in simpleLoess(y, x, w, span, degree = degree, parametric = parametric, : all data on boundary of neighborhood. make span bigger
```

```
## Warning in simpleLoess(y, x, w, span, degree = degree, parametric = parametric, : pseudoinverse used at -0.005
```

```
## Warning in simpleLoess(y, x, w, span, degree = degree, parametric = parametric, : neighborhood radius 0.005
```

```
## Warning in simpleLoess(y, x, w, span, degree = degree, parametric = parametric, : reciprocal condition number 1
```

```
## Warning in simpleLoess(y, x, w, span, degree = degree, parametric = parametric, : There are other near singularities as well. 1.01
```

```
## Warning in simpleLoess(y, x, w, span, degree = degree, parametric = parametric, : zero-width neighborhood. make span bigger
```

```
## Warning: Computation failed in `stat_smooth()`:
## NA/NaN/Inf in foreign function call (arg 5)
```

```
## Warning in simpleLoess(y, x, w, span, degree = degree, parametric = parametric, : at -0.005
```

```
## Warning in simpleLoess(y, x, w, span, degree = degree, parametric = parametric, : radius 2.5e-05
```

```
## Warning in simpleLoess(y, x, w, span, degree = degree, parametric = parametric, : all data on boundary of neighborhood. make span bigger
```

```
## Warning in simpleLoess(y, x, w, span, degree = degree, parametric = parametric, : pseudoinverse used at -0.005
```

```
## Warning in simpleLoess(y, x, w, span, degree = degree, parametric = parametric, : neighborhood radius 0.005
```

```
## Warning in simpleLoess(y, x, w, span, degree = degree, parametric = parametric, : reciprocal condition number 1
```

```
## Warning in simpleLoess(y, x, w, span, degree = degree, parametric = parametric, : There are other near singularities as well. 1.01
```

```
## Warning in simpleLoess(y, x, w, span, degree = degree, parametric = parametric, : zero-width neighborhood. make span bigger
```

```
## Warning: Computation failed in `stat_smooth()`:
## NA/NaN/Inf in foreign function call (arg 5)
```

```
## Warning in simpleLoess(y, x, w, span, degree = degree, parametric = parametric, : at -0.005
```

```
## Warning in simpleLoess(y, x, w, span, degree = degree, parametric = parametric, : radius 2.5e-05
```

```
## Warning in simpleLoess(y, x, w, span, degree = degree, parametric = parametric, : all data on boundary of neighborhood. make span bigger
```

```
## Warning in simpleLoess(y, x, w, span, degree = degree, parametric = parametric, : pseudoinverse used at -0.005
```

```
## Warning in simpleLoess(y, x, w, span, degree = degree, parametric = parametric, : neighborhood radius 0.005
```

```
## Warning in simpleLoess(y, x, w, span, degree = degree, parametric = parametric, : reciprocal condition number 1
```

```
## Warning in simpleLoess(y, x, w, span, degree = degree, parametric = parametric, : There are other near singularities as well. 1.01
```

```
## Warning in simpleLoess(y, x, w, span, degree = degree, parametric = parametric, : zero-width neighborhood. make span bigger
```

```
## Warning: Computation failed in `stat_smooth()`:
## NA/NaN/Inf in foreign function call (arg 5)
```

```
## Warning in simpleLoess(y, x, w, span, degree = degree, parametric = parametric, : pseudoinverse used at -0.005
```

```
## Warning in simpleLoess(y, x, w, span, degree = degree, parametric = parametric, : neighborhood radius 1.005
```

```
## Warning in simpleLoess(y, x, w, span, degree = degree, parametric = parametric, : reciprocal condition number 0
```

```
## Warning in simpleLoess(y, x, w, span, degree = degree, parametric = parametric, : There are other near singularities as well. 1.01
```

```
## Warning in predLoess(object$y, object$x, newx = if (is.null(newdata)) object$x else if (is.data.frame(newdata)) as.matrix(model.frame(delete.response(terms(object)), : pseudoinverse used at -0.005
```

```
## Warning in predLoess(object$y, object$x, newx = if (is.null(newdata)) object$x else if (is.data.frame(newdata)) as.matrix(model.frame(delete.response(terms(object)), : neighborhood radius 1.005
```

```
## Warning in predLoess(object$y, object$x, newx = if (is.null(newdata)) object$x else if (is.data.frame(newdata)) as.matrix(model.frame(delete.response(terms(object)), : reciprocal condition number 0
```

```
## Warning in predLoess(object$y, object$x, newx = if (is.null(newdata)) object$x else if (is.data.frame(newdata)) as.matrix(model.frame(delete.response(terms(object)), : There are other near
## singularities as well. 1.01
```

```
## Warning in simpleLoess(y, x, w, span, degree = degree, parametric = parametric, : at -0.005
```

```
## Warning in simpleLoess(y, x, w, span, degree = degree, parametric = parametric, : radius 2.5e-05
```

```
## Warning in simpleLoess(y, x, w, span, degree = degree, parametric = parametric, : all data on boundary of neighborhood. make span bigger
```

```
## Warning in simpleLoess(y, x, w, span, degree = degree, parametric = parametric, : pseudoinverse used at -0.005
```

```
## Warning in simpleLoess(y, x, w, span, degree = degree, parametric = parametric, : neighborhood radius 0.005
```

```
## Warning in simpleLoess(y, x, w, span, degree = degree, parametric = parametric, : reciprocal condition number 1
```

```
## Warning in simpleLoess(y, x, w, span, degree = degree, parametric = parametric, : There are other near singularities as well. 1.01
```

```
## Warning in simpleLoess(y, x, w, span, degree = degree, parametric = parametric, : zero-width neighborhood. make span bigger
```

```
## Warning: Computation failed in `stat_smooth()`:
## NA/NaN/Inf in foreign function call (arg 5)
```

```
## Warning in simpleLoess(y, x, w, span, degree = degree, parametric = parametric, : pseudoinverse used at -0.005
```

```
## Warning in simpleLoess(y, x, w, span, degree = degree, parametric = parametric, : neighborhood radius 1.005
```

```
## Warning in simpleLoess(y, x, w, span, degree = degree, parametric = parametric, : reciprocal condition number 0
```

```
## Warning in simpleLoess(y, x, w, span, degree = degree, parametric = parametric, : There are other near singularities as well. 1.01
```

```
## Warning in predLoess(object$y, object$x, newx = if (is.null(newdata)) object$x else if (is.data.frame(newdata)) as.matrix(model.frame(delete.response(terms(object)), : pseudoinverse used at -0.005
```

```
## Warning in predLoess(object$y, object$x, newx = if (is.null(newdata)) object$x else if (is.data.frame(newdata)) as.matrix(model.frame(delete.response(terms(object)), : neighborhood radius 1.005
```

```
## Warning in predLoess(object$y, object$x, newx = if (is.null(newdata)) object$x else if (is.data.frame(newdata)) as.matrix(model.frame(delete.response(terms(object)), : reciprocal condition number 0
```

```
## Warning in predLoess(object$y, object$x, newx = if (is.null(newdata)) object$x else if (is.data.frame(newdata)) as.matrix(model.frame(delete.response(terms(object)), : There are other near
## singularities as well. 1.01
```

```
## Warning in simpleLoess(y, x, w, span, degree = degree, parametric = parametric, : at -0.005
```

```
## Warning in simpleLoess(y, x, w, span, degree = degree, parametric = parametric, : radius 2.5e-05
```

```
## Warning in simpleLoess(y, x, w, span, degree = degree, parametric = parametric, : all data on boundary of neighborhood. make span bigger
```

```
## Warning in simpleLoess(y, x, w, span, degree = degree, parametric = parametric, : pseudoinverse used at -0.005
```

```
## Warning in simpleLoess(y, x, w, span, degree = degree, parametric = parametric, : neighborhood radius 0.005
```

```
## Warning in simpleLoess(y, x, w, span, degree = degree, parametric = parametric, : reciprocal condition number 1
```

```
## Warning in simpleLoess(y, x, w, span, degree = degree, parametric = parametric, : There are other near singularities as well. 1.01
```

```
## Warning in simpleLoess(y, x, w, span, degree = degree, parametric = parametric, : zero-width neighborhood. make span bigger
```

```
## Warning: Computation failed in `stat_smooth()`:
## NA/NaN/Inf in foreign function call (arg 5)
```

```
## Warning in simpleLoess(y, x, w, span, degree = degree, parametric = parametric, : at -0.005
```

```
## Warning in simpleLoess(y, x, w, span, degree = degree, parametric = parametric, : radius 2.5e-05
```

```
## Warning in simpleLoess(y, x, w, span, degree = degree, parametric = parametric, : all data on boundary of neighborhood. make span bigger
```

```
## Warning in simpleLoess(y, x, w, span, degree = degree, parametric = parametric, : pseudoinverse used at -0.005
```

```
## Warning in simpleLoess(y, x, w, span, degree = degree, parametric = parametric, : neighborhood radius 0.005
```

```
## Warning in simpleLoess(y, x, w, span, degree = degree, parametric = parametric, : reciprocal condition number 1
```

```
## Warning in simpleLoess(y, x, w, span, degree = degree, parametric = parametric, : There are other near singularities as well. 1.01
```

```
## Warning in simpleLoess(y, x, w, span, degree = degree, parametric = parametric, : zero-width neighborhood. make span bigger
```

```
## Warning: Computation failed in `stat_smooth()`:
## NA/NaN/Inf in foreign function call (arg 5)
```

```
## Warning in simpleLoess(y, x, w, span, degree = degree, parametric = parametric, : at -0.005
```

```
## Warning in simpleLoess(y, x, w, span, degree = degree, parametric = parametric, : radius 2.5e-05
```

```
## Warning in simpleLoess(y, x, w, span, degree = degree, parametric = parametric, : all data on boundary of neighborhood. make span bigger
```

```
## Warning in simpleLoess(y, x, w, span, degree = degree, parametric = parametric, : pseudoinverse used at -0.005
```

```
## Warning in simpleLoess(y, x, w, span, degree = degree, parametric = parametric, : neighborhood radius 0.005
```

```
## Warning in simpleLoess(y, x, w, span, degree = degree, parametric = parametric, : reciprocal condition number 1
```

```
## Warning in simpleLoess(y, x, w, span, degree = degree, parametric = parametric, : There are other near singularities as well. 1.01
```

```
## Warning in simpleLoess(y, x, w, span, degree = degree, parametric = parametric, : zero-width neighborhood. make span bigger
```

```
## Warning: Computation failed in `stat_smooth()`:
## NA/NaN/Inf in foreign function call (arg 5)
```

```
## Warning in simpleLoess(y, x, w, span, degree = degree, parametric = parametric, : at -0.005
```

```
## Warning in simpleLoess(y, x, w, span, degree = degree, parametric = parametric, : radius 2.5e-05
```

```
## Warning in simpleLoess(y, x, w, span, degree = degree, parametric = parametric, : all data on boundary of neighborhood. make span bigger
```

```
## Warning in simpleLoess(y, x, w, span, degree = degree, parametric = parametric, : pseudoinverse used at -0.005
```

```
## Warning in simpleLoess(y, x, w, span, degree = degree, parametric = parametric, : neighborhood radius 0.005
```

```
## Warning in simpleLoess(y, x, w, span, degree = degree, parametric = parametric, : reciprocal condition number 1
```

```
## Warning in simpleLoess(y, x, w, span, degree = degree, parametric = parametric, : There are other near singularities as well. 1.01
```

```
## Warning in simpleLoess(y, x, w, span, degree = degree, parametric = parametric, : zero-width neighborhood. make span bigger
```

```
## Warning: Computation failed in `stat_smooth()`:
## NA/NaN/Inf in foreign function call (arg 5)
```

```
## Warning in simpleLoess(y, x, w, span, degree = degree, parametric = parametric, : at -0.005
```

```
## Warning in simpleLoess(y, x, w, span, degree = degree, parametric = parametric, : radius 2.5e-05
```

```
## Warning in simpleLoess(y, x, w, span, degree = degree, parametric = parametric, : all data on boundary of neighborhood. make span bigger
```

```
## Warning in simpleLoess(y, x, w, span, degree = degree, parametric = parametric, : pseudoinverse used at -0.005
```

```
## Warning in simpleLoess(y, x, w, span, degree = degree, parametric = parametric, : neighborhood radius 0.005
```

```
## Warning in simpleLoess(y, x, w, span, degree = degree, parametric = parametric, : reciprocal condition number 1
```

```
## Warning in simpleLoess(y, x, w, span, degree = degree, parametric = parametric, : There are other near singularities as well. 1.01
```

```
## Warning in simpleLoess(y, x, w, span, degree = degree, parametric = parametric, : zero-width neighborhood. make span bigger
```

```
## Warning: Computation failed in `stat_smooth()`:
## NA/NaN/Inf in foreign function call (arg 5)
```

```
## Warning in simpleLoess(y, x, w, span, degree = degree, parametric = parametric, : at -0.005
```

```
## Warning in simpleLoess(y, x, w, span, degree = degree, parametric = parametric, : radius 2.5e-05
```

```
## Warning in simpleLoess(y, x, w, span, degree = degree, parametric = parametric, : all data on boundary of neighborhood. make span bigger
```

```
## Warning in simpleLoess(y, x, w, span, degree = degree, parametric = parametric, : pseudoinverse used at -0.005
```

```
## Warning in simpleLoess(y, x, w, span, degree = degree, parametric = parametric, : neighborhood radius 0.005
```

```
## Warning in simpleLoess(y, x, w, span, degree = degree, parametric = parametric, : reciprocal condition number 1
```

```
## Warning in simpleLoess(y, x, w, span, degree = degree, parametric = parametric, : There are other near singularities as well. 1.01
```

```
## Warning in simpleLoess(y, x, w, span, degree = degree, parametric = parametric, : zero-width neighborhood. make span bigger
```

```
## Warning: Computation failed in `stat_smooth()`:
## NA/NaN/Inf in foreign function call (arg 5)
```

```
## Warning in simpleLoess(y, x, w, span, degree = degree, parametric = parametric, : at -0.005
```

```
## Warning in simpleLoess(y, x, w, span, degree = degree, parametric = parametric, : radius 2.5e-05
```

```
## Warning in simpleLoess(y, x, w, span, degree = degree, parametric = parametric, : all data on boundary of neighborhood. make span bigger
```

```
## Warning in simpleLoess(y, x, w, span, degree = degree, parametric = parametric, : pseudoinverse used at -0.005
```

```
## Warning in simpleLoess(y, x, w, span, degree = degree, parametric = parametric, : neighborhood radius 0.005
```

```
## Warning in simpleLoess(y, x, w, span, degree = degree, parametric = parametric, : reciprocal condition number 1
```

```
## Warning in simpleLoess(y, x, w, span, degree = degree, parametric = parametric, : There are other near singularities as well. 1.01
```

```
## Warning in simpleLoess(y, x, w, span, degree = degree, parametric = parametric, : zero-width neighborhood. make span bigger
```

```
## Warning: Computation failed in `stat_smooth()`:
## NA/NaN/Inf in foreign function call (arg 5)
```

```
## Warning in simpleLoess(y, x, w, span, degree = degree, parametric = parametric, : at -0.005
```

```
## Warning in simpleLoess(y, x, w, span, degree = degree, parametric = parametric, : radius 2.5e-05
```

```
## Warning in simpleLoess(y, x, w, span, degree = degree, parametric = parametric, : all data on boundary of neighborhood. make span bigger
```

```
## Warning in simpleLoess(y, x, w, span, degree = degree, parametric = parametric, : pseudoinverse used at -0.005
```

```
## Warning in simpleLoess(y, x, w, span, degree = degree, parametric = parametric, : neighborhood radius 0.005
```

```
## Warning in simpleLoess(y, x, w, span, degree = degree, parametric = parametric, : reciprocal condition number 1
```

```
## Warning in simpleLoess(y, x, w, span, degree = degree, parametric = parametric, : There are other near singularities as well. 1.01
```

```
## Warning in simpleLoess(y, x, w, span, degree = degree, parametric = parametric, : zero-width neighborhood. make span bigger
```

```
## Warning: Computation failed in `stat_smooth()`:
## NA/NaN/Inf in foreign function call (arg 5)
```

```
## Warning in simpleLoess(y, x, w, span, degree = degree, parametric = parametric, : at -0.005
```

```
## Warning in simpleLoess(y, x, w, span, degree = degree, parametric = parametric, : radius 2.5e-05
```

```
## Warning in simpleLoess(y, x, w, span, degree = degree, parametric = parametric, : all data on boundary of neighborhood. make span bigger
```

```
## Warning in simpleLoess(y, x, w, span, degree = degree, parametric = parametric, : pseudoinverse used at -0.005
```

```
## Warning in simpleLoess(y, x, w, span, degree = degree, parametric = parametric, : neighborhood radius 0.005
```

```
## Warning in simpleLoess(y, x, w, span, degree = degree, parametric = parametric, : reciprocal condition number 1
```

```
## Warning in simpleLoess(y, x, w, span, degree = degree, parametric = parametric, : There are other near singularities as well. 1.01
```

```
## Warning in simpleLoess(y, x, w, span, degree = degree, parametric = parametric, : zero-width neighborhood. make span bigger
```

```
## Warning: Computation failed in `stat_smooth()`:
## NA/NaN/Inf in foreign function call (arg 5)
```

```
## Warning in simpleLoess(y, x, w, span, degree = degree, parametric = parametric, : at -0.005
```

```
## Warning in simpleLoess(y, x, w, span, degree = degree, parametric = parametric, : radius 2.5e-05
```

```
## Warning in simpleLoess(y, x, w, span, degree = degree, parametric = parametric, : all data on boundary of neighborhood. make span bigger
```

```
## Warning in simpleLoess(y, x, w, span, degree = degree, parametric = parametric, : pseudoinverse used at -0.005
```

```
## Warning in simpleLoess(y, x, w, span, degree = degree, parametric = parametric, : neighborhood radius 0.005
```

```
## Warning in simpleLoess(y, x, w, span, degree = degree, parametric = parametric, : reciprocal condition number 1
```

```
## Warning in simpleLoess(y, x, w, span, degree = degree, parametric = parametric, : There are other near singularities as well. 1.01
```

```
## Warning in simpleLoess(y, x, w, span, degree = degree, parametric = parametric, : zero-width neighborhood. make span bigger
```

```
## Warning: Computation failed in `stat_smooth()`:
## NA/NaN/Inf in foreign function call (arg 5)
```

```
## Warning in simpleLoess(y, x, w, span, degree = degree, parametric = parametric, : at -0.005
```

```
## Warning in simpleLoess(y, x, w, span, degree = degree, parametric = parametric, : radius 2.5e-05
```

```
## Warning in simpleLoess(y, x, w, span, degree = degree, parametric = parametric, : all data on boundary of neighborhood. make span bigger
```

```
## Warning in simpleLoess(y, x, w, span, degree = degree, parametric = parametric, : pseudoinverse used at -0.005
```

```
## Warning in simpleLoess(y, x, w, span, degree = degree, parametric = parametric, : neighborhood radius 0.005
```

```
## Warning in simpleLoess(y, x, w, span, degree = degree, parametric = parametric, : reciprocal condition number 1
```

```
## Warning in simpleLoess(y, x, w, span, degree = degree, parametric = parametric, : There are other near singularities as well. 1.01
```

```
## Warning in simpleLoess(y, x, w, span, degree = degree, parametric = parametric, : zero-width neighborhood. make span bigger
```

```
## Warning: Computation failed in `stat_smooth()`:
## NA/NaN/Inf in foreign function call (arg 5)
```

```
## Warning in simpleLoess(y, x, w, span, degree = degree, parametric = parametric, : at -0.005
```

```
## Warning in simpleLoess(y, x, w, span, degree = degree, parametric = parametric, : radius 2.5e-05
```

```
## Warning in simpleLoess(y, x, w, span, degree = degree, parametric = parametric, : all data on boundary of neighborhood. make span bigger
```

```
## Warning in simpleLoess(y, x, w, span, degree = degree, parametric = parametric, : pseudoinverse used at -0.005
```

```
## Warning in simpleLoess(y, x, w, span, degree = degree, parametric = parametric, : neighborhood radius 0.005
```

```
## Warning in simpleLoess(y, x, w, span, degree = degree, parametric = parametric, : reciprocal condition number 1
```

```
## Warning in simpleLoess(y, x, w, span, degree = degree, parametric = parametric, : There are other near singularities as well. 1.01
```

```
## Warning in simpleLoess(y, x, w, span, degree = degree, parametric = parametric, : zero-width neighborhood. make span bigger
```

```
## Warning: Computation failed in `stat_smooth()`:
## NA/NaN/Inf in foreign function call (arg 5)
```

```
## Warning in simpleLoess(y, x, w, span, degree = degree, parametric = parametric, : at -0.005
```

```
## Warning in simpleLoess(y, x, w, span, degree = degree, parametric = parametric, : radius 2.5e-05
```

```
## Warning in simpleLoess(y, x, w, span, degree = degree, parametric = parametric, : all data on boundary of neighborhood. make span bigger
```

```
## Warning in simpleLoess(y, x, w, span, degree = degree, parametric = parametric, : pseudoinverse used at -0.005
```

```
## Warning in simpleLoess(y, x, w, span, degree = degree, parametric = parametric, : neighborhood radius 0.005
```

```
## Warning in simpleLoess(y, x, w, span, degree = degree, parametric = parametric, : reciprocal condition number 1
```

```
## Warning in simpleLoess(y, x, w, span, degree = degree, parametric = parametric, : There are other near singularities as well. 1.01
```

```
## Warning in simpleLoess(y, x, w, span, degree = degree, parametric = parametric, : zero-width neighborhood. make span bigger
```

```
## Warning: Computation failed in `stat_smooth()`:
## NA/NaN/Inf in foreign function call (arg 5)
```

```
## Warning in simpleLoess(y, x, w, span, degree = degree, parametric = parametric, : at -0.005
```

```
## Warning in simpleLoess(y, x, w, span, degree = degree, parametric = parametric, : radius 2.5e-05
```

```
## Warning in simpleLoess(y, x, w, span, degree = degree, parametric = parametric, : all data on boundary of neighborhood. make span bigger
```

```
## Warning in simpleLoess(y, x, w, span, degree = degree, parametric = parametric, : pseudoinverse used at -0.005
```

```
## Warning in simpleLoess(y, x, w, span, degree = degree, parametric = parametric, : neighborhood radius 0.005
```

```
## Warning in simpleLoess(y, x, w, span, degree = degree, parametric = parametric, : reciprocal condition number 1
```

```
## Warning in simpleLoess(y, x, w, span, degree = degree, parametric = parametric, : There are other near singularities as well. 1.01
```

```
## Warning in simpleLoess(y, x, w, span, degree = degree, parametric = parametric, : zero-width neighborhood. make span bigger
```

```
## Warning: Computation failed in `stat_smooth()`:
## NA/NaN/Inf in foreign function call (arg 5)
```

```
## Warning in simpleLoess(y, x, w, span, degree = degree, parametric = parametric, : at -0.005
```

```
## Warning in simpleLoess(y, x, w, span, degree = degree, parametric = parametric, : radius 2.5e-05
```

```
## Warning in simpleLoess(y, x, w, span, degree = degree, parametric = parametric, : all data on boundary of neighborhood. make span bigger
```

```
## Warning in simpleLoess(y, x, w, span, degree = degree, parametric = parametric, : pseudoinverse used at -0.005
```

```
## Warning in simpleLoess(y, x, w, span, degree = degree, parametric = parametric, : neighborhood radius 0.005
```

```
## Warning in simpleLoess(y, x, w, span, degree = degree, parametric = parametric, : reciprocal condition number 1
```

```
## Warning in simpleLoess(y, x, w, span, degree = degree, parametric = parametric, : There are other near singularities as well. 1.01
```

```
## Warning in simpleLoess(y, x, w, span, degree = degree, parametric = parametric, : zero-width neighborhood. make span bigger
```

```
## Warning: Computation failed in `stat_smooth()`:
## NA/NaN/Inf in foreign function call (arg 5)
```

```
## Warning in simpleLoess(y, x, w, span, degree = degree, parametric = parametric, : at -0.005
```

```
## Warning in simpleLoess(y, x, w, span, degree = degree, parametric = parametric, : radius 2.5e-05
```

```
## Warning in simpleLoess(y, x, w, span, degree = degree, parametric = parametric, : all data on boundary of neighborhood. make span bigger
```

```
## Warning in simpleLoess(y, x, w, span, degree = degree, parametric = parametric, : pseudoinverse used at -0.005
```

```
## Warning in simpleLoess(y, x, w, span, degree = degree, parametric = parametric, : neighborhood radius 0.005
```

```
## Warning in simpleLoess(y, x, w, span, degree = degree, parametric = parametric, : reciprocal condition number 1
```

```
## Warning in simpleLoess(y, x, w, span, degree = degree, parametric = parametric, : There are other near singularities as well. 1.01
```

```
## Warning in simpleLoess(y, x, w, span, degree = degree, parametric = parametric, : zero-width neighborhood. make span bigger
```

```
## Warning: Computation failed in `stat_smooth()`:
## NA/NaN/Inf in foreign function call (arg 5)
```

```
## Warning in simpleLoess(y, x, w, span, degree = degree, parametric = parametric, : at -0.005
```

```
## Warning in simpleLoess(y, x, w, span, degree = degree, parametric = parametric, : radius 2.5e-05
```

```
## Warning in simpleLoess(y, x, w, span, degree = degree, parametric = parametric, : all data on boundary of neighborhood. make span bigger
```

```
## Warning in simpleLoess(y, x, w, span, degree = degree, parametric = parametric, : pseudoinverse used at -0.005
```

```
## Warning in simpleLoess(y, x, w, span, degree = degree, parametric = parametric, : neighborhood radius 0.005
```

```
## Warning in simpleLoess(y, x, w, span, degree = degree, parametric = parametric, : reciprocal condition number 1
```

```
## Warning in simpleLoess(y, x, w, span, degree = degree, parametric = parametric, : There are other near singularities as well. 1.01
```

```
## Warning in simpleLoess(y, x, w, span, degree = degree, parametric = parametric, : zero-width neighborhood. make span bigger
```

```
## Warning: Computation failed in `stat_smooth()`:
## NA/NaN/Inf in foreign function call (arg 5)
```

```
## Warning in simpleLoess(y, x, w, span, degree = degree, parametric = parametric, : at -0.005
```

```
## Warning in simpleLoess(y, x, w, span, degree = degree, parametric = parametric, : radius 2.5e-05
```

```
## Warning in simpleLoess(y, x, w, span, degree = degree, parametric = parametric, : all data on boundary of neighborhood. make span bigger
```

```
## Warning in simpleLoess(y, x, w, span, degree = degree, parametric = parametric, : pseudoinverse used at -0.005
```

```
## Warning in simpleLoess(y, x, w, span, degree = degree, parametric = parametric, : neighborhood radius 0.005
```

```
## Warning in simpleLoess(y, x, w, span, degree = degree, parametric = parametric, : reciprocal condition number 1
```

```
## Warning in simpleLoess(y, x, w, span, degree = degree, parametric = parametric, : There are other near singularities as well. 1.01
```

```
## Warning in simpleLoess(y, x, w, span, degree = degree, parametric = parametric, : zero-width neighborhood. make span bigger
```

```
## Warning: Computation failed in `stat_smooth()`:
## NA/NaN/Inf in foreign function call (arg 5)
```

```
## Warning in simpleLoess(y, x, w, span, degree = degree, parametric = parametric, : at -0.005
```

```
## Warning in simpleLoess(y, x, w, span, degree = degree, parametric = parametric, : radius 2.5e-05
```

```
## Warning in simpleLoess(y, x, w, span, degree = degree, parametric = parametric, : all data on boundary of neighborhood. make span bigger
```

```
## Warning in simpleLoess(y, x, w, span, degree = degree, parametric = parametric, : pseudoinverse used at -0.005
```

```
## Warning in simpleLoess(y, x, w, span, degree = degree, parametric = parametric, : neighborhood radius 0.005
```

```
## Warning in simpleLoess(y, x, w, span, degree = degree, parametric = parametric, : reciprocal condition number 1
```

```
## Warning in simpleLoess(y, x, w, span, degree = degree, parametric = parametric, : There are other near singularities as well. 1.01
```

```
## Warning in simpleLoess(y, x, w, span, degree = degree, parametric = parametric, : zero-width neighborhood. make span bigger
```

```
## Warning: Computation failed in `stat_smooth()`:
## NA/NaN/Inf in foreign function call (arg 5)
```

```
## Warning in simpleLoess(y, x, w, span, degree = degree, parametric = parametric, : at -0.005
```

```
## Warning in simpleLoess(y, x, w, span, degree = degree, parametric = parametric, : radius 2.5e-05
```

```
## Warning in simpleLoess(y, x, w, span, degree = degree, parametric = parametric, : all data on boundary of neighborhood. make span bigger
```

```
## Warning in simpleLoess(y, x, w, span, degree = degree, parametric = parametric, : pseudoinverse used at -0.005
```

```
## Warning in simpleLoess(y, x, w, span, degree = degree, parametric = parametric, : neighborhood radius 0.005
```

```
## Warning in simpleLoess(y, x, w, span, degree = degree, parametric = parametric, : reciprocal condition number 1
```

```
## Warning in simpleLoess(y, x, w, span, degree = degree, parametric = parametric, : There are other near singularities as well. 1.01
```

```
## Warning in simpleLoess(y, x, w, span, degree = degree, parametric = parametric, : zero-width neighborhood. make span bigger
```

```
## Warning: Computation failed in `stat_smooth()`:
## NA/NaN/Inf in foreign function call (arg 5)
```

```
## Warning in simpleLoess(y, x, w, span, degree = degree, parametric = parametric, : at -0.005
```

```
## Warning in simpleLoess(y, x, w, span, degree = degree, parametric = parametric, : radius 2.5e-05
```

```
## Warning in simpleLoess(y, x, w, span, degree = degree, parametric = parametric, : all data on boundary of neighborhood. make span bigger
```

```
## Warning in simpleLoess(y, x, w, span, degree = degree, parametric = parametric, : pseudoinverse used at -0.005
```

```
## Warning in simpleLoess(y, x, w, span, degree = degree, parametric = parametric, : neighborhood radius 0.005
```

```
## Warning in simpleLoess(y, x, w, span, degree = degree, parametric = parametric, : reciprocal condition number 1
```

```
## Warning in simpleLoess(y, x, w, span, degree = degree, parametric = parametric, : There are other near singularities as well. 1.01
```

```
## Warning in simpleLoess(y, x, w, span, degree = degree, parametric = parametric, : zero-width neighborhood. make span bigger
```

```
## Warning: Computation failed in `stat_smooth()`:
## NA/NaN/Inf in foreign function call (arg 5)
```

```
## Warning in simpleLoess(y, x, w, span, degree = degree, parametric = parametric, : at -0.005
```

```
## Warning in simpleLoess(y, x, w, span, degree = degree, parametric = parametric, : radius 2.5e-05
```

```
## Warning in simpleLoess(y, x, w, span, degree = degree, parametric = parametric, : all data on boundary of neighborhood. make span bigger
```

```
## Warning in simpleLoess(y, x, w, span, degree = degree, parametric = parametric, : pseudoinverse used at -0.005
```

```
## Warning in simpleLoess(y, x, w, span, degree = degree, parametric = parametric, : neighborhood radius 0.005
```

```
## Warning in simpleLoess(y, x, w, span, degree = degree, parametric = parametric, : reciprocal condition number 1
```

```
## Warning in simpleLoess(y, x, w, span, degree = degree, parametric = parametric, : There are other near singularities as well. 1.01
```

```
## Warning in simpleLoess(y, x, w, span, degree = degree, parametric = parametric, : zero-width neighborhood. make span bigger
```

```
## Warning: Computation failed in `stat_smooth()`:
## NA/NaN/Inf in foreign function call (arg 5)
```

```
## Warning in simpleLoess(y, x, w, span, degree = degree, parametric = parametric, : at -0.005
```

```
## Warning in simpleLoess(y, x, w, span, degree = degree, parametric = parametric, : radius 2.5e-05
```

```
## Warning in simpleLoess(y, x, w, span, degree = degree, parametric = parametric, : all data on boundary of neighborhood. make span bigger
```

```
## Warning in simpleLoess(y, x, w, span, degree = degree, parametric = parametric, : pseudoinverse used at -0.005
```

```
## Warning in simpleLoess(y, x, w, span, degree = degree, parametric = parametric, : neighborhood radius 0.005
```

```
## Warning in simpleLoess(y, x, w, span, degree = degree, parametric = parametric, : reciprocal condition number 1
```

```
## Warning in simpleLoess(y, x, w, span, degree = degree, parametric = parametric, : There are other near singularities as well. 1.01
```

```
## Warning in simpleLoess(y, x, w, span, degree = degree, parametric = parametric, : zero-width neighborhood. make span bigger
```

```
## Warning: Computation failed in `stat_smooth()`:
## NA/NaN/Inf in foreign function call (arg 5)
```

```
## Warning in simpleLoess(y, x, w, span, degree = degree, parametric = parametric, : pseudoinverse used at -0.005
```

```
## Warning in simpleLoess(y, x, w, span, degree = degree, parametric = parametric, : neighborhood radius 1.005
```

```
## Warning in simpleLoess(y, x, w, span, degree = degree, parametric = parametric, : reciprocal condition number 0
```

```
## Warning in simpleLoess(y, x, w, span, degree = degree, parametric = parametric, : There are other near singularities as well. 1.01
```

```
## Warning in predLoess(object$y, object$x, newx = if (is.null(newdata)) object$x else if (is.data.frame(newdata)) as.matrix(model.frame(delete.response(terms(object)), : pseudoinverse used at -0.005
```

```
## Warning in predLoess(object$y, object$x, newx = if (is.null(newdata)) object$x else if (is.data.frame(newdata)) as.matrix(model.frame(delete.response(terms(object)), : neighborhood radius 1.005
```

```
## Warning in predLoess(object$y, object$x, newx = if (is.null(newdata)) object$x else if (is.data.frame(newdata)) as.matrix(model.frame(delete.response(terms(object)), : reciprocal condition number 0
```

```
## Warning in predLoess(object$y, object$x, newx = if (is.null(newdata)) object$x else if (is.data.frame(newdata)) as.matrix(model.frame(delete.response(terms(object)), : There are other near
## singularities as well. 1.01
```

```
## Warning in simpleLoess(y, x, w, span, degree = degree, parametric = parametric, : at -0.005
```

```
## Warning in simpleLoess(y, x, w, span, degree = degree, parametric = parametric, : radius 2.5e-05
```

```
## Warning in simpleLoess(y, x, w, span, degree = degree, parametric = parametric, : all data on boundary of neighborhood. make span bigger
```

```
## Warning in simpleLoess(y, x, w, span, degree = degree, parametric = parametric, : pseudoinverse used at -0.005
```

```
## Warning in simpleLoess(y, x, w, span, degree = degree, parametric = parametric, : neighborhood radius 0.005
```

```
## Warning in simpleLoess(y, x, w, span, degree = degree, parametric = parametric, : reciprocal condition number 1
```

```
## Warning in simpleLoess(y, x, w, span, degree = degree, parametric = parametric, : There are other near singularities as well. 1.01
```

```
## Warning in simpleLoess(y, x, w, span, degree = degree, parametric = parametric, : zero-width neighborhood. make span bigger
```

```
## Warning: Computation failed in `stat_smooth()`:
## NA/NaN/Inf in foreign function call (arg 5)
```

```
## Warning in simpleLoess(y, x, w, span, degree = degree, parametric = parametric, : at -0.005
```

```
## Warning in simpleLoess(y, x, w, span, degree = degree, parametric = parametric, : radius 2.5e-05
```

```
## Warning in simpleLoess(y, x, w, span, degree = degree, parametric = parametric, : all data on boundary of neighborhood. make span bigger
```

```
## Warning in simpleLoess(y, x, w, span, degree = degree, parametric = parametric, : pseudoinverse used at -0.005
```

```
## Warning in simpleLoess(y, x, w, span, degree = degree, parametric = parametric, : neighborhood radius 0.005
```

```
## Warning in simpleLoess(y, x, w, span, degree = degree, parametric = parametric, : reciprocal condition number 1
```

```
## Warning in simpleLoess(y, x, w, span, degree = degree, parametric = parametric, : There are other near singularities as well. 1.01
```

```
## Warning in simpleLoess(y, x, w, span, degree = degree, parametric = parametric, : zero-width neighborhood. make span bigger
```

```
## Warning: Computation failed in `stat_smooth()`:
## NA/NaN/Inf in foreign function call (arg 5)
```

```
## Warning in simpleLoess(y, x, w, span, degree = degree, parametric = parametric, : at -0.005
```

```
## Warning in simpleLoess(y, x, w, span, degree = degree, parametric = parametric, : radius 2.5e-05
```

```
## Warning in simpleLoess(y, x, w, span, degree = degree, parametric = parametric, : all data on boundary of neighborhood. make span bigger
```

```
## Warning in simpleLoess(y, x, w, span, degree = degree, parametric = parametric, : pseudoinverse used at -0.005
```

```
## Warning in simpleLoess(y, x, w, span, degree = degree, parametric = parametric, : neighborhood radius 0.005
```

```
## Warning in simpleLoess(y, x, w, span, degree = degree, parametric = parametric, : reciprocal condition number 1
```

```
## Warning in simpleLoess(y, x, w, span, degree = degree, parametric = parametric, : There are other near singularities as well. 1.01
```

```
## Warning in simpleLoess(y, x, w, span, degree = degree, parametric = parametric, : zero-width neighborhood. make span bigger
```

```
## Warning: Computation failed in `stat_smooth()`:
## NA/NaN/Inf in foreign function call (arg 5)
```

```
## Warning in simpleLoess(y, x, w, span, degree = degree, parametric = parametric, : at -0.005
```

```
## Warning in simpleLoess(y, x, w, span, degree = degree, parametric = parametric, : radius 2.5e-05
```

```
## Warning in simpleLoess(y, x, w, span, degree = degree, parametric = parametric, : all data on boundary of neighborhood. make span bigger
```

```
## Warning in simpleLoess(y, x, w, span, degree = degree, parametric = parametric, : pseudoinverse used at -0.005
```

```
## Warning in simpleLoess(y, x, w, span, degree = degree, parametric = parametric, : neighborhood radius 0.005
```

```
## Warning in simpleLoess(y, x, w, span, degree = degree, parametric = parametric, : reciprocal condition number 1
```

```
## Warning in simpleLoess(y, x, w, span, degree = degree, parametric = parametric, : There are other near singularities as well. 1.01
```

```
## Warning in simpleLoess(y, x, w, span, degree = degree, parametric = parametric, : zero-width neighborhood. make span bigger
```

```
## Warning: Computation failed in `stat_smooth()`:
## NA/NaN/Inf in foreign function call (arg 5)
```

```
## Warning in simpleLoess(y, x, w, span, degree = degree, parametric = parametric, : at -0.005
```

```
## Warning in simpleLoess(y, x, w, span, degree = degree, parametric = parametric, : radius 2.5e-05
```

```
## Warning in simpleLoess(y, x, w, span, degree = degree, parametric = parametric, : all data on boundary of neighborhood. make span bigger
```

```
## Warning in simpleLoess(y, x, w, span, degree = degree, parametric = parametric, : pseudoinverse used at -0.005
```

```
## Warning in simpleLoess(y, x, w, span, degree = degree, parametric = parametric, : neighborhood radius 0.005
```

```
## Warning in simpleLoess(y, x, w, span, degree = degree, parametric = parametric, : reciprocal condition number 1
```

```
## Warning in simpleLoess(y, x, w, span, degree = degree, parametric = parametric, : There are other near singularities as well. 1.01
```

```
## Warning in simpleLoess(y, x, w, span, degree = degree, parametric = parametric, : zero-width neighborhood. make span bigger
```

```
## Warning: Computation failed in `stat_smooth()`:
## NA/NaN/Inf in foreign function call (arg 5)
```

```
## Warning in simpleLoess(y, x, w, span, degree = degree, parametric = parametric, : at -0.005
```

```
## Warning in simpleLoess(y, x, w, span, degree = degree, parametric = parametric, : radius 2.5e-05
```

```
## Warning in simpleLoess(y, x, w, span, degree = degree, parametric = parametric, : all data on boundary of neighborhood. make span bigger
```

```
## Warning in simpleLoess(y, x, w, span, degree = degree, parametric = parametric, : pseudoinverse used at -0.005
```

```
## Warning in simpleLoess(y, x, w, span, degree = degree, parametric = parametric, : neighborhood radius 0.005
```

```
## Warning in simpleLoess(y, x, w, span, degree = degree, parametric = parametric, : reciprocal condition number 1
```

```
## Warning in simpleLoess(y, x, w, span, degree = degree, parametric = parametric, : There are other near singularities as well. 1.01
```

```
## Warning in simpleLoess(y, x, w, span, degree = degree, parametric = parametric, : zero-width neighborhood. make span bigger
```

```
## Warning: Computation failed in `stat_smooth()`:
## NA/NaN/Inf in foreign function call (arg 5)
```

```
## Warning in simpleLoess(y, x, w, span, degree = degree, parametric = parametric, : at -0.005
```

```
## Warning in simpleLoess(y, x, w, span, degree = degree, parametric = parametric, : radius 2.5e-05
```

```
## Warning in simpleLoess(y, x, w, span, degree = degree, parametric = parametric, : all data on boundary of neighborhood. make span bigger
```

```
## Warning in simpleLoess(y, x, w, span, degree = degree, parametric = parametric, : pseudoinverse used at -0.005
```

```
## Warning in simpleLoess(y, x, w, span, degree = degree, parametric = parametric, : neighborhood radius 0.005
```

```
## Warning in simpleLoess(y, x, w, span, degree = degree, parametric = parametric, : reciprocal condition number 1
```

```
## Warning in simpleLoess(y, x, w, span, degree = degree, parametric = parametric, : There are other near singularities as well. 1.01
```

```
## Warning in simpleLoess(y, x, w, span, degree = degree, parametric = parametric, : zero-width neighborhood. make span bigger
```

```
## Warning: Computation failed in `stat_smooth()`:
## NA/NaN/Inf in foreign function call (arg 5)
```

```
## Warning in simpleLoess(y, x, w, span, degree = degree, parametric = parametric, : at -0.005
```

```
## Warning in simpleLoess(y, x, w, span, degree = degree, parametric = parametric, : radius 2.5e-05
```

```
## Warning in simpleLoess(y, x, w, span, degree = degree, parametric = parametric, : all data on boundary of neighborhood. make span bigger
```

```
## Warning in simpleLoess(y, x, w, span, degree = degree, parametric = parametric, : pseudoinverse used at -0.005
```

```
## Warning in simpleLoess(y, x, w, span, degree = degree, parametric = parametric, : neighborhood radius 0.005
```

```
## Warning in simpleLoess(y, x, w, span, degree = degree, parametric = parametric, : reciprocal condition number 1
```

```
## Warning in simpleLoess(y, x, w, span, degree = degree, parametric = parametric, : There are other near singularities as well. 1.01
```

```
## Warning in simpleLoess(y, x, w, span, degree = degree, parametric = parametric, : zero-width neighborhood. make span bigger
```

```
## Warning: Computation failed in `stat_smooth()`:
## NA/NaN/Inf in foreign function call (arg 5)
```

```
## Warning in simpleLoess(y, x, w, span, degree = degree, parametric = parametric, : at -0.005
```

```
## Warning in simpleLoess(y, x, w, span, degree = degree, parametric = parametric, : radius 2.5e-05
```

```
## Warning in simpleLoess(y, x, w, span, degree = degree, parametric = parametric, : all data on boundary of neighborhood. make span bigger
```

```
## Warning in simpleLoess(y, x, w, span, degree = degree, parametric = parametric, : pseudoinverse used at -0.005
```

```
## Warning in simpleLoess(y, x, w, span, degree = degree, parametric = parametric, : neighborhood radius 0.005
```

```
## Warning in simpleLoess(y, x, w, span, degree = degree, parametric = parametric, : reciprocal condition number 1
```

```
## Warning in simpleLoess(y, x, w, span, degree = degree, parametric = parametric, : There are other near singularities as well. 1.01
```

```
## Warning in simpleLoess(y, x, w, span, degree = degree, parametric = parametric, : zero-width neighborhood. make span bigger
```

```
## Warning: Computation failed in `stat_smooth()`:
## NA/NaN/Inf in foreign function call (arg 5)
```

```
## Warning in simpleLoess(y, x, w, span, degree = degree, parametric = parametric, : pseudoinverse used at -0.005
```

```
## Warning in simpleLoess(y, x, w, span, degree = degree, parametric = parametric, : neighborhood radius 1.005
```

```
## Warning in simpleLoess(y, x, w, span, degree = degree, parametric = parametric, : reciprocal condition number 0
```

```
## Warning in simpleLoess(y, x, w, span, degree = degree, parametric = parametric, : There are other near singularities as well. 1.01
```

```
## Warning in predLoess(object$y, object$x, newx = if (is.null(newdata)) object$x else if (is.data.frame(newdata)) as.matrix(model.frame(delete.response(terms(object)), : pseudoinverse used at -0.005
```

```
## Warning in predLoess(object$y, object$x, newx = if (is.null(newdata)) object$x else if (is.data.frame(newdata)) as.matrix(model.frame(delete.response(terms(object)), : neighborhood radius 1.005
```

```
## Warning in predLoess(object$y, object$x, newx = if (is.null(newdata)) object$x else if (is.data.frame(newdata)) as.matrix(model.frame(delete.response(terms(object)), : reciprocal condition number 0
```

```
## Warning in predLoess(object$y, object$x, newx = if (is.null(newdata)) object$x else if (is.data.frame(newdata)) as.matrix(model.frame(delete.response(terms(object)), : There are other near
## singularities as well. 1.01
```

```
## Warning in simpleLoess(y, x, w, span, degree = degree, parametric = parametric, : at -0.005
```

```
## Warning in simpleLoess(y, x, w, span, degree = degree, parametric = parametric, : radius 2.5e-05
```

```
## Warning in simpleLoess(y, x, w, span, degree = degree, parametric = parametric, : all data on boundary of neighborhood. make span bigger
```

```
## Warning in simpleLoess(y, x, w, span, degree = degree, parametric = parametric, : pseudoinverse used at -0.005
```

```
## Warning in simpleLoess(y, x, w, span, degree = degree, parametric = parametric, : neighborhood radius 0.005
```

```
## Warning in simpleLoess(y, x, w, span, degree = degree, parametric = parametric, : reciprocal condition number 1
```

```
## Warning in simpleLoess(y, x, w, span, degree = degree, parametric = parametric, : There are other near singularities as well. 1.01
```

```
## Warning in simpleLoess(y, x, w, span, degree = degree, parametric = parametric, : zero-width neighborhood. make span bigger
```

```
## Warning: Computation failed in `stat_smooth()`:
## NA/NaN/Inf in foreign function call (arg 5)
```

```
## Warning in simpleLoess(y, x, w, span, degree = degree, parametric = parametric, : at -0.005
```

```
## Warning in simpleLoess(y, x, w, span, degree = degree, parametric = parametric, : radius 2.5e-05
```

```
## Warning in simpleLoess(y, x, w, span, degree = degree, parametric = parametric, : all data on boundary of neighborhood. make span bigger
```

```
## Warning in simpleLoess(y, x, w, span, degree = degree, parametric = parametric, : pseudoinverse used at -0.005
```

```
## Warning in simpleLoess(y, x, w, span, degree = degree, parametric = parametric, : neighborhood radius 0.005
```

```
## Warning in simpleLoess(y, x, w, span, degree = degree, parametric = parametric, : reciprocal condition number 1
```

```
## Warning in simpleLoess(y, x, w, span, degree = degree, parametric = parametric, : There are other near singularities as well. 1.01
```

```
## Warning in simpleLoess(y, x, w, span, degree = degree, parametric = parametric, : zero-width neighborhood. make span bigger
```

```
## Warning: Computation failed in `stat_smooth()`:
## NA/NaN/Inf in foreign function call (arg 5)
```

```
## Warning in simpleLoess(y, x, w, span, degree = degree, parametric = parametric, : pseudoinverse used at -0.005
```

```
## Warning in simpleLoess(y, x, w, span, degree = degree, parametric = parametric, : neighborhood radius 1.005
```

```
## Warning in simpleLoess(y, x, w, span, degree = degree, parametric = parametric, : reciprocal condition number 0
```

```
## Warning in simpleLoess(y, x, w, span, degree = degree, parametric = parametric, : There are other near singularities as well. 1.01
```

```
## Warning in predLoess(object$y, object$x, newx = if (is.null(newdata)) object$x else if (is.data.frame(newdata)) as.matrix(model.frame(delete.response(terms(object)), : pseudoinverse used at -0.005
```

```
## Warning in predLoess(object$y, object$x, newx = if (is.null(newdata)) object$x else if (is.data.frame(newdata)) as.matrix(model.frame(delete.response(terms(object)), : neighborhood radius 1.005
```

```
## Warning in predLoess(object$y, object$x, newx = if (is.null(newdata)) object$x else if (is.data.frame(newdata)) as.matrix(model.frame(delete.response(terms(object)), : reciprocal condition number 0
```

```
## Warning in predLoess(object$y, object$x, newx = if (is.null(newdata)) object$x else if (is.data.frame(newdata)) as.matrix(model.frame(delete.response(terms(object)), : There are other near
## singularities as well. 1.01
```

```
## Warning in simpleLoess(y, x, w, span, degree = degree, parametric = parametric, : at -0.005
```

```
## Warning in simpleLoess(y, x, w, span, degree = degree, parametric = parametric, : radius 2.5e-05
```

```
## Warning in simpleLoess(y, x, w, span, degree = degree, parametric = parametric, : all data on boundary of neighborhood. make span bigger
```

```
## Warning in simpleLoess(y, x, w, span, degree = degree, parametric = parametric, : pseudoinverse used at -0.005
```

```
## Warning in simpleLoess(y, x, w, span, degree = degree, parametric = parametric, : neighborhood radius 0.005
```

```
## Warning in simpleLoess(y, x, w, span, degree = degree, parametric = parametric, : reciprocal condition number 1
```

```
## Warning in simpleLoess(y, x, w, span, degree = degree, parametric = parametric, : There are other near singularities as well. 1.01
```

```
## Warning in simpleLoess(y, x, w, span, degree = degree, parametric = parametric, : zero-width neighborhood. make span bigger
```

```
## Warning: Computation failed in `stat_smooth()`:
## NA/NaN/Inf in foreign function call (arg 5)
```

```
## Warning in simpleLoess(y, x, w, span, degree = degree, parametric = parametric, : at -0.005
```

```
## Warning in simpleLoess(y, x, w, span, degree = degree, parametric = parametric, : radius 2.5e-05
```

```
## Warning in simpleLoess(y, x, w, span, degree = degree, parametric = parametric, : all data on boundary of neighborhood. make span bigger
```

```
## Warning in simpleLoess(y, x, w, span, degree = degree, parametric = parametric, : pseudoinverse used at -0.005
```

```
## Warning in simpleLoess(y, x, w, span, degree = degree, parametric = parametric, : neighborhood radius 0.005
```

```
## Warning in simpleLoess(y, x, w, span, degree = degree, parametric = parametric, : reciprocal condition number 1
```

```
## Warning in simpleLoess(y, x, w, span, degree = degree, parametric = parametric, : There are other near singularities as well. 1.01
```

```
## Warning in simpleLoess(y, x, w, span, degree = degree, parametric = parametric, : zero-width neighborhood. make span bigger
```

```
## Warning: Computation failed in `stat_smooth()`:
## NA/NaN/Inf in foreign function call (arg 5)
```

```
## Warning in simpleLoess(y, x, w, span, degree = degree, parametric = parametric, : at -0.005
```

```
## Warning in simpleLoess(y, x, w, span, degree = degree, parametric = parametric, : radius 2.5e-05
```

```
## Warning in simpleLoess(y, x, w, span, degree = degree, parametric = parametric, : all data on boundary of neighborhood. make span bigger
```

```
## Warning in simpleLoess(y, x, w, span, degree = degree, parametric = parametric, : pseudoinverse used at -0.005
```

```
## Warning in simpleLoess(y, x, w, span, degree = degree, parametric = parametric, : neighborhood radius 0.005
```

```
## Warning in simpleLoess(y, x, w, span, degree = degree, parametric = parametric, : reciprocal condition number 1
```

```
## Warning in simpleLoess(y, x, w, span, degree = degree, parametric = parametric, : There are other near singularities as well. 1.01
```

```
## Warning in simpleLoess(y, x, w, span, degree = degree, parametric = parametric, : zero-width neighborhood. make span bigger
```

```
## Warning: Computation failed in `stat_smooth()`:
## NA/NaN/Inf in foreign function call (arg 5)
```

```
## Warning in simpleLoess(y, x, w, span, degree = degree, parametric = parametric, : at -0.005
```

```
## Warning in simpleLoess(y, x, w, span, degree = degree, parametric = parametric, : radius 2.5e-05
```

```
## Warning in simpleLoess(y, x, w, span, degree = degree, parametric = parametric, : all data on boundary of neighborhood. make span bigger
```

```
## Warning in simpleLoess(y, x, w, span, degree = degree, parametric = parametric, : pseudoinverse used at -0.005
```

```
## Warning in simpleLoess(y, x, w, span, degree = degree, parametric = parametric, : neighborhood radius 0.005
```

```
## Warning in simpleLoess(y, x, w, span, degree = degree, parametric = parametric, : reciprocal condition number 1
```

```
## Warning in simpleLoess(y, x, w, span, degree = degree, parametric = parametric, : There are other near singularities as well. 1.01
```

```
## Warning in simpleLoess(y, x, w, span, degree = degree, parametric = parametric, : zero-width neighborhood. make span bigger
```

```
## Warning: Computation failed in `stat_smooth()`:
## NA/NaN/Inf in foreign function call (arg 5)
```

```
## Warning in simpleLoess(y, x, w, span, degree = degree, parametric = parametric, : at -0.005
```

```
## Warning in simpleLoess(y, x, w, span, degree = degree, parametric = parametric, : radius 2.5e-05
```

```
## Warning in simpleLoess(y, x, w, span, degree = degree, parametric = parametric, : all data on boundary of neighborhood. make span bigger
```

```
## Warning in simpleLoess(y, x, w, span, degree = degree, parametric = parametric, : pseudoinverse used at -0.005
```

```
## Warning in simpleLoess(y, x, w, span, degree = degree, parametric = parametric, : neighborhood radius 0.005
```

```
## Warning in simpleLoess(y, x, w, span, degree = degree, parametric = parametric, : reciprocal condition number 1
```

```
## Warning in simpleLoess(y, x, w, span, degree = degree, parametric = parametric, : There are other near singularities as well. 1.01
```

```
## Warning in simpleLoess(y, x, w, span, degree = degree, parametric = parametric, : zero-width neighborhood. make span bigger
```

```
## Warning: Computation failed in `stat_smooth()`:
## NA/NaN/Inf in foreign function call (arg 5)
```

```
## Warning in simpleLoess(y, x, w, span, degree = degree, parametric = parametric, : pseudoinverse used at -0.005
```

```
## Warning in simpleLoess(y, x, w, span, degree = degree, parametric = parametric, : neighborhood radius 1.005
```

```
## Warning in simpleLoess(y, x, w, span, degree = degree, parametric = parametric, : reciprocal condition number 0
```

```
## Warning in simpleLoess(y, x, w, span, degree = degree, parametric = parametric, : There are other near singularities as well. 1.01
```

```
## Warning in predLoess(object$y, object$x, newx = if (is.null(newdata)) object$x else if (is.data.frame(newdata)) as.matrix(model.frame(delete.response(terms(object)), : pseudoinverse used at -0.005
```

```
## Warning in predLoess(object$y, object$x, newx = if (is.null(newdata)) object$x else if (is.data.frame(newdata)) as.matrix(model.frame(delete.response(terms(object)), : neighborhood radius 1.005
```

```
## Warning in predLoess(object$y, object$x, newx = if (is.null(newdata)) object$x else if (is.data.frame(newdata)) as.matrix(model.frame(delete.response(terms(object)), : reciprocal condition number 0
```

```
## Warning in predLoess(object$y, object$x, newx = if (is.null(newdata)) object$x else if (is.data.frame(newdata)) as.matrix(model.frame(delete.response(terms(object)), : There are other near
## singularities as well. 1.01
```

```
## Warning in simpleLoess(y, x, w, span, degree = degree, parametric = parametric, : at -0.005
```

```
## Warning in simpleLoess(y, x, w, span, degree = degree, parametric = parametric, : radius 2.5e-05
```

```
## Warning in simpleLoess(y, x, w, span, degree = degree, parametric = parametric, : all data on boundary of neighborhood. make span bigger
```

```
## Warning in simpleLoess(y, x, w, span, degree = degree, parametric = parametric, : pseudoinverse used at -0.005
```

```
## Warning in simpleLoess(y, x, w, span, degree = degree, parametric = parametric, : neighborhood radius 0.005
```

```
## Warning in simpleLoess(y, x, w, span, degree = degree, parametric = parametric, : reciprocal condition number 1
```

```
## Warning in simpleLoess(y, x, w, span, degree = degree, parametric = parametric, : There are other near singularities as well. 1.01
```

```
## Warning in simpleLoess(y, x, w, span, degree = degree, parametric = parametric, : zero-width neighborhood. make span bigger
```

```
## Warning: Computation failed in `stat_smooth()`:
## NA/NaN/Inf in foreign function call (arg 5)
```

```
## Warning in simpleLoess(y, x, w, span, degree = degree, parametric = parametric, : at -0.005
```

```
## Warning in simpleLoess(y, x, w, span, degree = degree, parametric = parametric, : radius 2.5e-05
```

```
## Warning in simpleLoess(y, x, w, span, degree = degree, parametric = parametric, : all data on boundary of neighborhood. make span bigger
```

```
## Warning in simpleLoess(y, x, w, span, degree = degree, parametric = parametric, : pseudoinverse used at -0.005
```

```
## Warning in simpleLoess(y, x, w, span, degree = degree, parametric = parametric, : neighborhood radius 0.005
```

```
## Warning in simpleLoess(y, x, w, span, degree = degree, parametric = parametric, : reciprocal condition number 1
```

```
## Warning in simpleLoess(y, x, w, span, degree = degree, parametric = parametric, : There are other near singularities as well. 1.01
```

```
## Warning in simpleLoess(y, x, w, span, degree = degree, parametric = parametric, : zero-width neighborhood. make span bigger
```

```
## Warning: Computation failed in `stat_smooth()`:
## NA/NaN/Inf in foreign function call (arg 5)
```

```
## Warning in simpleLoess(y, x, w, span, degree = degree, parametric = parametric, : at -0.005
```

```
## Warning in simpleLoess(y, x, w, span, degree = degree, parametric = parametric, : radius 2.5e-05
```

```
## Warning in simpleLoess(y, x, w, span, degree = degree, parametric = parametric, : all data on boundary of neighborhood. make span bigger
```

```
## Warning in simpleLoess(y, x, w, span, degree = degree, parametric = parametric, : pseudoinverse used at -0.005
```

```
## Warning in simpleLoess(y, x, w, span, degree = degree, parametric = parametric, : neighborhood radius 0.005
```

```
## Warning in simpleLoess(y, x, w, span, degree = degree, parametric = parametric, : reciprocal condition number 1
```

```
## Warning in simpleLoess(y, x, w, span, degree = degree, parametric = parametric, : There are other near singularities as well. 1.01
```

```
## Warning in simpleLoess(y, x, w, span, degree = degree, parametric = parametric, : zero-width neighborhood. make span bigger
```

```
## Warning: Computation failed in `stat_smooth()`:
## NA/NaN/Inf in foreign function call (arg 5)
```

```
## Warning in simpleLoess(y, x, w, span, degree = degree, parametric = parametric, : at -0.005
```

```
## Warning in simpleLoess(y, x, w, span, degree = degree, parametric = parametric, : radius 2.5e-05
```

```
## Warning in simpleLoess(y, x, w, span, degree = degree, parametric = parametric, : all data on boundary of neighborhood. make span bigger
```

```
## Warning in simpleLoess(y, x, w, span, degree = degree, parametric = parametric, : pseudoinverse used at -0.005
```

```
## Warning in simpleLoess(y, x, w, span, degree = degree, parametric = parametric, : neighborhood radius 0.005
```

```
## Warning in simpleLoess(y, x, w, span, degree = degree, parametric = parametric, : reciprocal condition number 1
```

```
## Warning in simpleLoess(y, x, w, span, degree = degree, parametric = parametric, : There are other near singularities as well. 1.01
```

```
## Warning in simpleLoess(y, x, w, span, degree = degree, parametric = parametric, : zero-width neighborhood. make span bigger
```

```
## Warning: Computation failed in `stat_smooth()`:
## NA/NaN/Inf in foreign function call (arg 5)
```

```
## Warning in simpleLoess(y, x, w, span, degree = degree, parametric = parametric, : at -0.005
```

```
## Warning in simpleLoess(y, x, w, span, degree = degree, parametric = parametric, : radius 2.5e-05
```

```
## Warning in simpleLoess(y, x, w, span, degree = degree, parametric = parametric, : all data on boundary of neighborhood. make span bigger
```

```
## Warning in simpleLoess(y, x, w, span, degree = degree, parametric = parametric, : pseudoinverse used at -0.005
```

```
## Warning in simpleLoess(y, x, w, span, degree = degree, parametric = parametric, : neighborhood radius 0.005
```

```
## Warning in simpleLoess(y, x, w, span, degree = degree, parametric = parametric, : reciprocal condition number 1
```

```
## Warning in simpleLoess(y, x, w, span, degree = degree, parametric = parametric, : There are other near singularities as well. 1.01
```

```
## Warning in simpleLoess(y, x, w, span, degree = degree, parametric = parametric, : zero-width neighborhood. make span bigger
```

```
## Warning: Computation failed in `stat_smooth()`:
## NA/NaN/Inf in foreign function call (arg 5)
```

```
## Warning in simpleLoess(y, x, w, span, degree = degree, parametric = parametric, : at -0.005
```

```
## Warning in simpleLoess(y, x, w, span, degree = degree, parametric = parametric, : radius 2.5e-05
```

```
## Warning in simpleLoess(y, x, w, span, degree = degree, parametric = parametric, : all data on boundary of neighborhood. make span bigger
```

```
## Warning in simpleLoess(y, x, w, span, degree = degree, parametric = parametric, : pseudoinverse used at -0.005
```

```
## Warning in simpleLoess(y, x, w, span, degree = degree, parametric = parametric, : neighborhood radius 0.005
```

```
## Warning in simpleLoess(y, x, w, span, degree = degree, parametric = parametric, : reciprocal condition number 1
```

```
## Warning in simpleLoess(y, x, w, span, degree = degree, parametric = parametric, : There are other near singularities as well. 1.01
```

```
## Warning in simpleLoess(y, x, w, span, degree = degree, parametric = parametric, : zero-width neighborhood. make span bigger
```

```
## Warning: Computation failed in `stat_smooth()`:
## NA/NaN/Inf in foreign function call (arg 5)
```

```
## Warning in simpleLoess(y, x, w, span, degree = degree, parametric = parametric, : at -0.005
```

```
## Warning in simpleLoess(y, x, w, span, degree = degree, parametric = parametric, : radius 2.5e-05
```

```
## Warning in simpleLoess(y, x, w, span, degree = degree, parametric = parametric, : all data on boundary of neighborhood. make span bigger
```

```
## Warning in simpleLoess(y, x, w, span, degree = degree, parametric = parametric, : pseudoinverse used at -0.005
```

```
## Warning in simpleLoess(y, x, w, span, degree = degree, parametric = parametric, : neighborhood radius 0.005
```

```
## Warning in simpleLoess(y, x, w, span, degree = degree, parametric = parametric, : reciprocal condition number 1
```

```
## Warning in simpleLoess(y, x, w, span, degree = degree, parametric = parametric, : There are other near singularities as well. 1.01
```

```
## Warning in simpleLoess(y, x, w, span, degree = degree, parametric = parametric, : zero-width neighborhood. make span bigger
```

```
## Warning: Computation failed in `stat_smooth()`:
## NA/NaN/Inf in foreign function call (arg 5)
```

```
## Warning in simpleLoess(y, x, w, span, degree = degree, parametric = parametric, : at -0.005
```

```
## Warning in simpleLoess(y, x, w, span, degree = degree, parametric = parametric, : radius 2.5e-05
```

```
## Warning in simpleLoess(y, x, w, span, degree = degree, parametric = parametric, : all data on boundary of neighborhood. make span bigger
```

```
## Warning in simpleLoess(y, x, w, span, degree = degree, parametric = parametric, : pseudoinverse used at -0.005
```

```
## Warning in simpleLoess(y, x, w, span, degree = degree, parametric = parametric, : neighborhood radius 0.005
```

```
## Warning in simpleLoess(y, x, w, span, degree = degree, parametric = parametric, : reciprocal condition number 1
```

```
## Warning in simpleLoess(y, x, w, span, degree = degree, parametric = parametric, : There are other near singularities as well. 1.01
```

```
## Warning in simpleLoess(y, x, w, span, degree = degree, parametric = parametric, : zero-width neighborhood. make span bigger
```

```
## Warning: Computation failed in `stat_smooth()`:
## NA/NaN/Inf in foreign function call (arg 5)
```

```
## Warning in simpleLoess(y, x, w, span, degree = degree, parametric = parametric, : at -0.005
```

```
## Warning in simpleLoess(y, x, w, span, degree = degree, parametric = parametric, : radius 2.5e-05
```

```
## Warning in simpleLoess(y, x, w, span, degree = degree, parametric = parametric, : all data on boundary of neighborhood. make span bigger
```

```
## Warning in simpleLoess(y, x, w, span, degree = degree, parametric = parametric, : pseudoinverse used at -0.005
```

```
## Warning in simpleLoess(y, x, w, span, degree = degree, parametric = parametric, : neighborhood radius 0.005
```

```
## Warning in simpleLoess(y, x, w, span, degree = degree, parametric = parametric, : reciprocal condition number 1
```

```
## Warning in simpleLoess(y, x, w, span, degree = degree, parametric = parametric, : There are other near singularities as well. 1.01
```

```
## Warning in simpleLoess(y, x, w, span, degree = degree, parametric = parametric, : zero-width neighborhood. make span bigger
```

```
## Warning: Computation failed in `stat_smooth()`:
## NA/NaN/Inf in foreign function call (arg 5)
```

```
## Warning in simpleLoess(y, x, w, span, degree = degree, parametric = parametric, : at -0.005
```

```
## Warning in simpleLoess(y, x, w, span, degree = degree, parametric = parametric, : radius 2.5e-05
```

```
## Warning in simpleLoess(y, x, w, span, degree = degree, parametric = parametric, : all data on boundary of neighborhood. make span bigger
```

```
## Warning in simpleLoess(y, x, w, span, degree = degree, parametric = parametric, : pseudoinverse used at -0.005
```

```
## Warning in simpleLoess(y, x, w, span, degree = degree, parametric = parametric, : neighborhood radius 0.005
```

```
## Warning in simpleLoess(y, x, w, span, degree = degree, parametric = parametric, : reciprocal condition number 1
```

```
## Warning in simpleLoess(y, x, w, span, degree = degree, parametric = parametric, : There are other near singularities as well. 1.01
```

```
## Warning in simpleLoess(y, x, w, span, degree = degree, parametric = parametric, : zero-width neighborhood. make span bigger
```

```
## Warning: Computation failed in `stat_smooth()`:
## NA/NaN/Inf in foreign function call (arg 5)
```

```
## Warning in simpleLoess(y, x, w, span, degree = degree, parametric = parametric, : at -0.005
```

```
## Warning in simpleLoess(y, x, w, span, degree = degree, parametric = parametric, : radius 2.5e-05
```

```
## Warning in simpleLoess(y, x, w, span, degree = degree, parametric = parametric, : all data on boundary of neighborhood. make span bigger
```

```
## Warning in simpleLoess(y, x, w, span, degree = degree, parametric = parametric, : pseudoinverse used at -0.005
```

```
## Warning in simpleLoess(y, x, w, span, degree = degree, parametric = parametric, : neighborhood radius 0.005
```

```
## Warning in simpleLoess(y, x, w, span, degree = degree, parametric = parametric, : reciprocal condition number 1
```

```
## Warning in simpleLoess(y, x, w, span, degree = degree, parametric = parametric, : There are other near singularities as well. 1.01
```

```
## Warning in simpleLoess(y, x, w, span, degree = degree, parametric = parametric, : zero-width neighborhood. make span bigger
```

```
## Warning: Computation failed in `stat_smooth()`:
## NA/NaN/Inf in foreign function call (arg 5)
```

```
## Warning in simpleLoess(y, x, w, span, degree = degree, parametric = parametric, : at -0.005
```

```
## Warning in simpleLoess(y, x, w, span, degree = degree, parametric = parametric, : radius 2.5e-05
```

```
## Warning in simpleLoess(y, x, w, span, degree = degree, parametric = parametric, : all data on boundary of neighborhood. make span bigger
```

```
## Warning in simpleLoess(y, x, w, span, degree = degree, parametric = parametric, : pseudoinverse used at -0.005
```

```
## Warning in simpleLoess(y, x, w, span, degree = degree, parametric = parametric, : neighborhood radius 0.005
```

```
## Warning in simpleLoess(y, x, w, span, degree = degree, parametric = parametric, : reciprocal condition number 1
```

```
## Warning in simpleLoess(y, x, w, span, degree = degree, parametric = parametric, : There are other near singularities as well. 1.01
```

```
## Warning in simpleLoess(y, x, w, span, degree = degree, parametric = parametric, : zero-width neighborhood. make span bigger
```

```
## Warning: Computation failed in `stat_smooth()`:
## NA/NaN/Inf in foreign function call (arg 5)
```

```
## Warning in simpleLoess(y, x, w, span, degree = degree, parametric = parametric, : pseudoinverse used at -0.005
```

```
## Warning in simpleLoess(y, x, w, span, degree = degree, parametric = parametric, : neighborhood radius 1.005
```

```
## Warning in simpleLoess(y, x, w, span, degree = degree, parametric = parametric, : reciprocal condition number 0
```

```
## Warning in simpleLoess(y, x, w, span, degree = degree, parametric = parametric, : There are other near singularities as well. 1.01
```

```
## Warning in predLoess(object$y, object$x, newx = if (is.null(newdata)) object$x else if (is.data.frame(newdata)) as.matrix(model.frame(delete.response(terms(object)), : pseudoinverse used at -0.005
```

```
## Warning in predLoess(object$y, object$x, newx = if (is.null(newdata)) object$x else if (is.data.frame(newdata)) as.matrix(model.frame(delete.response(terms(object)), : neighborhood radius 1.005
```

```
## Warning in predLoess(object$y, object$x, newx = if (is.null(newdata)) object$x else if (is.data.frame(newdata)) as.matrix(model.frame(delete.response(terms(object)), : reciprocal condition number 0
```

```
## Warning in predLoess(object$y, object$x, newx = if (is.null(newdata)) object$x else if (is.data.frame(newdata)) as.matrix(model.frame(delete.response(terms(object)), : There are other near
## singularities as well. 1.01
```

```
## Warning in simpleLoess(y, x, w, span, degree = degree, parametric = parametric, : at -0.005
```

```
## Warning in simpleLoess(y, x, w, span, degree = degree, parametric = parametric, : radius 2.5e-05
```

```
## Warning in simpleLoess(y, x, w, span, degree = degree, parametric = parametric, : all data on boundary of neighborhood. make span bigger
```

```
## Warning in simpleLoess(y, x, w, span, degree = degree, parametric = parametric, : pseudoinverse used at -0.005
```

```
## Warning in simpleLoess(y, x, w, span, degree = degree, parametric = parametric, : neighborhood radius 0.005
```

```
## Warning in simpleLoess(y, x, w, span, degree = degree, parametric = parametric, : reciprocal condition number 1
```

```
## Warning in simpleLoess(y, x, w, span, degree = degree, parametric = parametric, : There are other near singularities as well. 1.01
```

```
## Warning in simpleLoess(y, x, w, span, degree = degree, parametric = parametric, : zero-width neighborhood. make span bigger
```

```
## Warning: Computation failed in `stat_smooth()`:
## NA/NaN/Inf in foreign function call (arg 5)
```

```
## Warning in simpleLoess(y, x, w, span, degree = degree, parametric = parametric, : at -0.005
```

```
## Warning in simpleLoess(y, x, w, span, degree = degree, parametric = parametric, : radius 2.5e-05
```

```
## Warning in simpleLoess(y, x, w, span, degree = degree, parametric = parametric, : all data on boundary of neighborhood. make span bigger
```

```
## Warning in simpleLoess(y, x, w, span, degree = degree, parametric = parametric, : pseudoinverse used at -0.005
```

```
## Warning in simpleLoess(y, x, w, span, degree = degree, parametric = parametric, : neighborhood radius 0.005
```

```
## Warning in simpleLoess(y, x, w, span, degree = degree, parametric = parametric, : reciprocal condition number 1
```

```
## Warning in simpleLoess(y, x, w, span, degree = degree, parametric = parametric, : There are other near singularities as well. 1.01
```

```
## Warning in simpleLoess(y, x, w, span, degree = degree, parametric = parametric, : zero-width neighborhood. make span bigger
```

```
## Warning: Computation failed in `stat_smooth()`:
## NA/NaN/Inf in foreign function call (arg 5)
```

```
## Warning in simpleLoess(y, x, w, span, degree = degree, parametric = parametric, : at -0.005
```

```
## Warning in simpleLoess(y, x, w, span, degree = degree, parametric = parametric, : radius 2.5e-05
```

```
## Warning in simpleLoess(y, x, w, span, degree = degree, parametric = parametric, : all data on boundary of neighborhood. make span bigger
```

```
## Warning in simpleLoess(y, x, w, span, degree = degree, parametric = parametric, : pseudoinverse used at -0.005
```

```
## Warning in simpleLoess(y, x, w, span, degree = degree, parametric = parametric, : neighborhood radius 0.005
```

```
## Warning in simpleLoess(y, x, w, span, degree = degree, parametric = parametric, : reciprocal condition number 1
```

```
## Warning in simpleLoess(y, x, w, span, degree = degree, parametric = parametric, : There are other near singularities as well. 1.01
```

```
## Warning in simpleLoess(y, x, w, span, degree = degree, parametric = parametric, : zero-width neighborhood. make span bigger
```

```
## Warning: Computation failed in `stat_smooth()`:
## NA/NaN/Inf in foreign function call (arg 5)
```

```
## Warning in simpleLoess(y, x, w, span, degree = degree, parametric = parametric, : at -0.005
```

```
## Warning in simpleLoess(y, x, w, span, degree = degree, parametric = parametric, : radius 2.5e-05
```

```
## Warning in simpleLoess(y, x, w, span, degree = degree, parametric = parametric, : all data on boundary of neighborhood. make span bigger
```

```
## Warning in simpleLoess(y, x, w, span, degree = degree, parametric = parametric, : pseudoinverse used at -0.005
```

```
## Warning in simpleLoess(y, x, w, span, degree = degree, parametric = parametric, : neighborhood radius 0.005
```

```
## Warning in simpleLoess(y, x, w, span, degree = degree, parametric = parametric, : reciprocal condition number 1
```

```
## Warning in simpleLoess(y, x, w, span, degree = degree, parametric = parametric, : There are other near singularities as well. 1.01
```

```
## Warning in simpleLoess(y, x, w, span, degree = degree, parametric = parametric, : zero-width neighborhood. make span bigger
```

```
## Warning: Computation failed in `stat_smooth()`:
## NA/NaN/Inf in foreign function call (arg 5)
```

```
## Warning in simpleLoess(y, x, w, span, degree = degree, parametric = parametric, : at -0.005
```

```
## Warning in simpleLoess(y, x, w, span, degree = degree, parametric = parametric, : radius 2.5e-05
```

```
## Warning in simpleLoess(y, x, w, span, degree = degree, parametric = parametric, : all data on boundary of neighborhood. make span bigger
```

```
## Warning in simpleLoess(y, x, w, span, degree = degree, parametric = parametric, : pseudoinverse used at -0.005
```

```
## Warning in simpleLoess(y, x, w, span, degree = degree, parametric = parametric, : neighborhood radius 0.005
```

```
## Warning in simpleLoess(y, x, w, span, degree = degree, parametric = parametric, : reciprocal condition number 1
```

```
## Warning in simpleLoess(y, x, w, span, degree = degree, parametric = parametric, : There are other near singularities as well. 1.01
```

```
## Warning in simpleLoess(y, x, w, span, degree = degree, parametric = parametric, : zero-width neighborhood. make span bigger
```

```
## Warning: Computation failed in `stat_smooth()`:
## NA/NaN/Inf in foreign function call (arg 5)
```

```
## Warning in simpleLoess(y, x, w, span, degree = degree, parametric = parametric, : at -0.005
```

```
## Warning in simpleLoess(y, x, w, span, degree = degree, parametric = parametric, : radius 2.5e-05
```

```
## Warning in simpleLoess(y, x, w, span, degree = degree, parametric = parametric, : all data on boundary of neighborhood. make span bigger
```

```
## Warning in simpleLoess(y, x, w, span, degree = degree, parametric = parametric, : pseudoinverse used at -0.005
```

```
## Warning in simpleLoess(y, x, w, span, degree = degree, parametric = parametric, : neighborhood radius 0.005
```

```
## Warning in simpleLoess(y, x, w, span, degree = degree, parametric = parametric, : reciprocal condition number 1
```

```
## Warning in simpleLoess(y, x, w, span, degree = degree, parametric = parametric, : There are other near singularities as well. 1.01
```

```
## Warning in simpleLoess(y, x, w, span, degree = degree, parametric = parametric, : zero-width neighborhood. make span bigger
```

```
## Warning: Computation failed in `stat_smooth()`:
## NA/NaN/Inf in foreign function call (arg 5)
```

```
## Warning in simpleLoess(y, x, w, span, degree = degree, parametric = parametric, : at -0.005
```

```
## Warning in simpleLoess(y, x, w, span, degree = degree, parametric = parametric, : radius 2.5e-05
```

```
## Warning in simpleLoess(y, x, w, span, degree = degree, parametric = parametric, : all data on boundary of neighborhood. make span bigger
```

```
## Warning in simpleLoess(y, x, w, span, degree = degree, parametric = parametric, : pseudoinverse used at -0.005
```

```
## Warning in simpleLoess(y, x, w, span, degree = degree, parametric = parametric, : neighborhood radius 0.005
```

```
## Warning in simpleLoess(y, x, w, span, degree = degree, parametric = parametric, : reciprocal condition number 1
```

```
## Warning in simpleLoess(y, x, w, span, degree = degree, parametric = parametric, : There are other near singularities as well. 1.01
```

```
## Warning in simpleLoess(y, x, w, span, degree = degree, parametric = parametric, : zero-width neighborhood. make span bigger
```

```
## Warning: Computation failed in `stat_smooth()`:
## NA/NaN/Inf in foreign function call (arg 5)
```

```
## Warning in simpleLoess(y, x, w, span, degree = degree, parametric = parametric, : pseudoinverse used at -0.005
```

```
## Warning in simpleLoess(y, x, w, span, degree = degree, parametric = parametric, : neighborhood radius 1.005
```

```
## Warning in simpleLoess(y, x, w, span, degree = degree, parametric = parametric, : reciprocal condition number 0
```

```
## Warning in simpleLoess(y, x, w, span, degree = degree, parametric = parametric, : There are other near singularities as well. 1.01
```

```
## Warning in predLoess(object$y, object$x, newx = if (is.null(newdata)) object$x else if (is.data.frame(newdata)) as.matrix(model.frame(delete.response(terms(object)), : pseudoinverse used at -0.005
```

```
## Warning in predLoess(object$y, object$x, newx = if (is.null(newdata)) object$x else if (is.data.frame(newdata)) as.matrix(model.frame(delete.response(terms(object)), : neighborhood radius 1.005
```

```
## Warning in predLoess(object$y, object$x, newx = if (is.null(newdata)) object$x else if (is.data.frame(newdata)) as.matrix(model.frame(delete.response(terms(object)), : reciprocal condition number 0
```

```
## Warning in predLoess(object$y, object$x, newx = if (is.null(newdata)) object$x else if (is.data.frame(newdata)) as.matrix(model.frame(delete.response(terms(object)), : There are other near
## singularities as well. 1.01
```

```
## Warning in simpleLoess(y, x, w, span, degree = degree, parametric = parametric, : at -0.005
```

```
## Warning in simpleLoess(y, x, w, span, degree = degree, parametric = parametric, : radius 2.5e-05
```

```
## Warning in simpleLoess(y, x, w, span, degree = degree, parametric = parametric, : all data on boundary of neighborhood. make span bigger
```

```
## Warning in simpleLoess(y, x, w, span, degree = degree, parametric = parametric, : pseudoinverse used at -0.005
```

```
## Warning in simpleLoess(y, x, w, span, degree = degree, parametric = parametric, : neighborhood radius 0.005
```

```
## Warning in simpleLoess(y, x, w, span, degree = degree, parametric = parametric, : reciprocal condition number 1
```

```
## Warning in simpleLoess(y, x, w, span, degree = degree, parametric = parametric, : There are other near singularities as well. 1.01
```

```
## Warning in simpleLoess(y, x, w, span, degree = degree, parametric = parametric, : zero-width neighborhood. make span bigger
```

```
## Warning: Computation failed in `stat_smooth()`:
## NA/NaN/Inf in foreign function call (arg 5)
```

```
## Warning in simpleLoess(y, x, w, span, degree = degree, parametric = parametric, : at -0.005
```

```
## Warning in simpleLoess(y, x, w, span, degree = degree, parametric = parametric, : radius 2.5e-05
```

```
## Warning in simpleLoess(y, x, w, span, degree = degree, parametric = parametric, : all data on boundary of neighborhood. make span bigger
```

```
## Warning in simpleLoess(y, x, w, span, degree = degree, parametric = parametric, : pseudoinverse used at -0.005
```

```
## Warning in simpleLoess(y, x, w, span, degree = degree, parametric = parametric, : neighborhood radius 0.005
```

```
## Warning in simpleLoess(y, x, w, span, degree = degree, parametric = parametric, : reciprocal condition number 1
```

```
## Warning in simpleLoess(y, x, w, span, degree = degree, parametric = parametric, : There are other near singularities as well. 1.01
```

```
## Warning in simpleLoess(y, x, w, span, degree = degree, parametric = parametric, : zero-width neighborhood. make span bigger
```

```
## Warning: Computation failed in `stat_smooth()`:
## NA/NaN/Inf in foreign function call (arg 5)
```

```
## Warning in simpleLoess(y, x, w, span, degree = degree, parametric = parametric, : at -0.005
```

```
## Warning in simpleLoess(y, x, w, span, degree = degree, parametric = parametric, : radius 2.5e-05
```

```
## Warning in simpleLoess(y, x, w, span, degree = degree, parametric = parametric, : all data on boundary of neighborhood. make span bigger
```

```
## Warning in simpleLoess(y, x, w, span, degree = degree, parametric = parametric, : pseudoinverse used at -0.005
```

```
## Warning in simpleLoess(y, x, w, span, degree = degree, parametric = parametric, : neighborhood radius 0.005
```

```
## Warning in simpleLoess(y, x, w, span, degree = degree, parametric = parametric, : reciprocal condition number 1
```

```
## Warning in simpleLoess(y, x, w, span, degree = degree, parametric = parametric, : There are other near singularities as well. 1.01
```

```
## Warning in simpleLoess(y, x, w, span, degree = degree, parametric = parametric, : zero-width neighborhood. make span bigger
```

```
## Warning: Computation failed in `stat_smooth()`:
## NA/NaN/Inf in foreign function call (arg 5)
```

```
## Warning in simpleLoess(y, x, w, span, degree = degree, parametric = parametric, : at -0.005
```

```
## Warning in simpleLoess(y, x, w, span, degree = degree, parametric = parametric, : radius 2.5e-05
```

```
## Warning in simpleLoess(y, x, w, span, degree = degree, parametric = parametric, : all data on boundary of neighborhood. make span bigger
```

```
## Warning in simpleLoess(y, x, w, span, degree = degree, parametric = parametric, : pseudoinverse used at -0.005
```

```
## Warning in simpleLoess(y, x, w, span, degree = degree, parametric = parametric, : neighborhood radius 0.005
```

```
## Warning in simpleLoess(y, x, w, span, degree = degree, parametric = parametric, : reciprocal condition number 1
```

```
## Warning in simpleLoess(y, x, w, span, degree = degree, parametric = parametric, : There are other near singularities as well. 1.01
```

```
## Warning in simpleLoess(y, x, w, span, degree = degree, parametric = parametric, : zero-width neighborhood. make span bigger
```

```
## Warning: Computation failed in `stat_smooth()`:
## NA/NaN/Inf in foreign function call (arg 5)
```

```
## Warning in simpleLoess(y, x, w, span, degree = degree, parametric = parametric, : at -0.005
```

```
## Warning in simpleLoess(y, x, w, span, degree = degree, parametric = parametric, : radius 2.5e-05
```

```
## Warning in simpleLoess(y, x, w, span, degree = degree, parametric = parametric, : all data on boundary of neighborhood. make span bigger
```

```
## Warning in simpleLoess(y, x, w, span, degree = degree, parametric = parametric, : pseudoinverse used at -0.005
```

```
## Warning in simpleLoess(y, x, w, span, degree = degree, parametric = parametric, : neighborhood radius 0.005
```

```
## Warning in simpleLoess(y, x, w, span, degree = degree, parametric = parametric, : reciprocal condition number 1
```

```
## Warning in simpleLoess(y, x, w, span, degree = degree, parametric = parametric, : There are other near singularities as well. 1.01
```

```
## Warning in simpleLoess(y, x, w, span, degree = degree, parametric = parametric, : zero-width neighborhood. make span bigger
```

```
## Warning: Computation failed in `stat_smooth()`:
## NA/NaN/Inf in foreign function call (arg 5)
```

```
## Warning in simpleLoess(y, x, w, span, degree = degree, parametric = parametric, : at -0.005
```

```
## Warning in simpleLoess(y, x, w, span, degree = degree, parametric = parametric, : radius 2.5e-05
```

```
## Warning in simpleLoess(y, x, w, span, degree = degree, parametric = parametric, : all data on boundary of neighborhood. make span bigger
```

```
## Warning in simpleLoess(y, x, w, span, degree = degree, parametric = parametric, : pseudoinverse used at -0.005
```

```
## Warning in simpleLoess(y, x, w, span, degree = degree, parametric = parametric, : neighborhood radius 0.005
```

```
## Warning in simpleLoess(y, x, w, span, degree = degree, parametric = parametric, : reciprocal condition number 1
```

```
## Warning in simpleLoess(y, x, w, span, degree = degree, parametric = parametric, : There are other near singularities as well. 1.01
```

```
## Warning in simpleLoess(y, x, w, span, degree = degree, parametric = parametric, : zero-width neighborhood. make span bigger
```

```
## Warning: Computation failed in `stat_smooth()`:
## NA/NaN/Inf in foreign function call (arg 5)
```

```
## Warning in simpleLoess(y, x, w, span, degree = degree, parametric = parametric, : at -0.005
```

```
## Warning in simpleLoess(y, x, w, span, degree = degree, parametric = parametric, : radius 2.5e-05
```

```
## Warning in simpleLoess(y, x, w, span, degree = degree, parametric = parametric, : all data on boundary of neighborhood. make span bigger
```

```
## Warning in simpleLoess(y, x, w, span, degree = degree, parametric = parametric, : pseudoinverse used at -0.005
```

```
## Warning in simpleLoess(y, x, w, span, degree = degree, parametric = parametric, : neighborhood radius 0.005
```

```
## Warning in simpleLoess(y, x, w, span, degree = degree, parametric = parametric, : reciprocal condition number 1
```

```
## Warning in simpleLoess(y, x, w, span, degree = degree, parametric = parametric, : There are other near singularities as well. 1.01
```

```
## Warning in simpleLoess(y, x, w, span, degree = degree, parametric = parametric, : zero-width neighborhood. make span bigger
```

```
## Warning: Computation failed in `stat_smooth()`:
## NA/NaN/Inf in foreign function call (arg 5)
```

```
## Warning in simpleLoess(y, x, w, span, degree = degree, parametric = parametric, : at -0.005
```

```
## Warning in simpleLoess(y, x, w, span, degree = degree, parametric = parametric, : radius 2.5e-05
```

```
## Warning in simpleLoess(y, x, w, span, degree = degree, parametric = parametric, : all data on boundary of neighborhood. make span bigger
```

```
## Warning in simpleLoess(y, x, w, span, degree = degree, parametric = parametric, : pseudoinverse used at -0.005
```

```
## Warning in simpleLoess(y, x, w, span, degree = degree, parametric = parametric, : neighborhood radius 0.005
```

```
## Warning in simpleLoess(y, x, w, span, degree = degree, parametric = parametric, : reciprocal condition number 1
```

```
## Warning in simpleLoess(y, x, w, span, degree = degree, parametric = parametric, : There are other near singularities as well. 1.01
```

```
## Warning in simpleLoess(y, x, w, span, degree = degree, parametric = parametric, : zero-width neighborhood. make span bigger
```

```
## Warning: Computation failed in `stat_smooth()`:
## NA/NaN/Inf in foreign function call (arg 5)
```

```
## Warning in simpleLoess(y, x, w, span, degree = degree, parametric = parametric, : pseudoinverse used at -0.005
```

```
## Warning in simpleLoess(y, x, w, span, degree = degree, parametric = parametric, : neighborhood radius 1.005
```

```
## Warning in simpleLoess(y, x, w, span, degree = degree, parametric = parametric, : reciprocal condition number 0
```

```
## Warning in simpleLoess(y, x, w, span, degree = degree, parametric = parametric, : There are other near singularities as well. 1.01
```

```
## Warning in predLoess(object$y, object$x, newx = if (is.null(newdata)) object$x else if (is.data.frame(newdata)) as.matrix(model.frame(delete.response(terms(object)), : pseudoinverse used at -0.005
```

```
## Warning in predLoess(object$y, object$x, newx = if (is.null(newdata)) object$x else if (is.data.frame(newdata)) as.matrix(model.frame(delete.response(terms(object)), : neighborhood radius 1.005
```

```
## Warning in predLoess(object$y, object$x, newx = if (is.null(newdata)) object$x else if (is.data.frame(newdata)) as.matrix(model.frame(delete.response(terms(object)), : reciprocal condition number 0
```

```
## Warning in predLoess(object$y, object$x, newx = if (is.null(newdata)) object$x else if (is.data.frame(newdata)) as.matrix(model.frame(delete.response(terms(object)), : There are other near
## singularities as well. 1.01
```

```
## Warning in simpleLoess(y, x, w, span, degree = degree, parametric = parametric, : at -0.005
```

```
## Warning in simpleLoess(y, x, w, span, degree = degree, parametric = parametric, : radius 2.5e-05
```

```
## Warning in simpleLoess(y, x, w, span, degree = degree, parametric = parametric, : all data on boundary of neighborhood. make span bigger
```

```
## Warning in simpleLoess(y, x, w, span, degree = degree, parametric = parametric, : pseudoinverse used at -0.005
```

```
## Warning in simpleLoess(y, x, w, span, degree = degree, parametric = parametric, : neighborhood radius 0.005
```

```
## Warning in simpleLoess(y, x, w, span, degree = degree, parametric = parametric, : reciprocal condition number 1
```

```
## Warning in simpleLoess(y, x, w, span, degree = degree, parametric = parametric, : There are other near singularities as well. 1.01
```

```
## Warning in simpleLoess(y, x, w, span, degree = degree, parametric = parametric, : zero-width neighborhood. make span bigger
```

```
## Warning: Computation failed in `stat_smooth()`:
## NA/NaN/Inf in foreign function call (arg 5)
```

```
## Warning in simpleLoess(y, x, w, span, degree = degree, parametric = parametric, : at -0.005
```

```
## Warning in simpleLoess(y, x, w, span, degree = degree, parametric = parametric, : radius 2.5e-05
```

```
## Warning in simpleLoess(y, x, w, span, degree = degree, parametric = parametric, : all data on boundary of neighborhood. make span bigger
```

```
## Warning in simpleLoess(y, x, w, span, degree = degree, parametric = parametric, : pseudoinverse used at -0.005
```

```
## Warning in simpleLoess(y, x, w, span, degree = degree, parametric = parametric, : neighborhood radius 0.005
```

```
## Warning in simpleLoess(y, x, w, span, degree = degree, parametric = parametric, : reciprocal condition number 1
```

```
## Warning in simpleLoess(y, x, w, span, degree = degree, parametric = parametric, : There are other near singularities as well. 1.01
```

```
## Warning in simpleLoess(y, x, w, span, degree = degree, parametric = parametric, : zero-width neighborhood. make span bigger
```

```
## Warning: Computation failed in `stat_smooth()`:
## NA/NaN/Inf in foreign function call (arg 5)
```

```
## Warning in simpleLoess(y, x, w, span, degree = degree, parametric = parametric, : at -0.005
```

```
## Warning in simpleLoess(y, x, w, span, degree = degree, parametric = parametric, : radius 2.5e-05
```

```
## Warning in simpleLoess(y, x, w, span, degree = degree, parametric = parametric, : all data on boundary of neighborhood. make span bigger
```

```
## Warning in simpleLoess(y, x, w, span, degree = degree, parametric = parametric, : pseudoinverse used at -0.005
```

```
## Warning in simpleLoess(y, x, w, span, degree = degree, parametric = parametric, : neighborhood radius 0.005
```

```
## Warning in simpleLoess(y, x, w, span, degree = degree, parametric = parametric, : reciprocal condition number 1
```

```
## Warning in simpleLoess(y, x, w, span, degree = degree, parametric = parametric, : There are other near singularities as well. 1.01
```

```
## Warning in simpleLoess(y, x, w, span, degree = degree, parametric = parametric, : zero-width neighborhood. make span bigger
```

```
## Warning: Computation failed in `stat_smooth()`:
## NA/NaN/Inf in foreign function call (arg 5)
```

```
## Warning in simpleLoess(y, x, w, span, degree = degree, parametric = parametric, : at -0.005
```

```
## Warning in simpleLoess(y, x, w, span, degree = degree, parametric = parametric, : radius 2.5e-05
```

```
## Warning in simpleLoess(y, x, w, span, degree = degree, parametric = parametric, : all data on boundary of neighborhood. make span bigger
```

```
## Warning in simpleLoess(y, x, w, span, degree = degree, parametric = parametric, : pseudoinverse used at -0.005
```

```
## Warning in simpleLoess(y, x, w, span, degree = degree, parametric = parametric, : neighborhood radius 0.005
```

```
## Warning in simpleLoess(y, x, w, span, degree = degree, parametric = parametric, : reciprocal condition number 1
```

```
## Warning in simpleLoess(y, x, w, span, degree = degree, parametric = parametric, : There are other near singularities as well. 1.01
```

```
## Warning in simpleLoess(y, x, w, span, degree = degree, parametric = parametric, : zero-width neighborhood. make span bigger
```

```
## Warning: Computation failed in `stat_smooth()`:
## NA/NaN/Inf in foreign function call (arg 5)
```

```
## Warning in simpleLoess(y, x, w, span, degree = degree, parametric = parametric, : at -0.005
```

```
## Warning in simpleLoess(y, x, w, span, degree = degree, parametric = parametric, : radius 2.5e-05
```

```
## Warning in simpleLoess(y, x, w, span, degree = degree, parametric = parametric, : all data on boundary of neighborhood. make span bigger
```

```
## Warning in simpleLoess(y, x, w, span, degree = degree, parametric = parametric, : pseudoinverse used at -0.005
```

```
## Warning in simpleLoess(y, x, w, span, degree = degree, parametric = parametric, : neighborhood radius 0.005
```

```
## Warning in simpleLoess(y, x, w, span, degree = degree, parametric = parametric, : reciprocal condition number 1
```

```
## Warning in simpleLoess(y, x, w, span, degree = degree, parametric = parametric, : There are other near singularities as well. 1.01
```

```
## Warning in simpleLoess(y, x, w, span, degree = degree, parametric = parametric, : zero-width neighborhood. make span bigger
```

```
## Warning: Computation failed in `stat_smooth()`:
## NA/NaN/Inf in foreign function call (arg 5)
```

```
## Warning in simpleLoess(y, x, w, span, degree = degree, parametric = parametric, : at -0.005
```

```
## Warning in simpleLoess(y, x, w, span, degree = degree, parametric = parametric, : radius 2.5e-05
```

```
## Warning in simpleLoess(y, x, w, span, degree = degree, parametric = parametric, : all data on boundary of neighborhood. make span bigger
```

```
## Warning in simpleLoess(y, x, w, span, degree = degree, parametric = parametric, : pseudoinverse used at -0.005
```

```
## Warning in simpleLoess(y, x, w, span, degree = degree, parametric = parametric, : neighborhood radius 0.005
```

```
## Warning in simpleLoess(y, x, w, span, degree = degree, parametric = parametric, : reciprocal condition number 1
```

```
## Warning in simpleLoess(y, x, w, span, degree = degree, parametric = parametric, : There are other near singularities as well. 1.01
```

```
## Warning in simpleLoess(y, x, w, span, degree = degree, parametric = parametric, : zero-width neighborhood. make span bigger
```

```
## Warning: Computation failed in `stat_smooth()`:
## NA/NaN/Inf in foreign function call (arg 5)
```

```
## Warning in simpleLoess(y, x, w, span, degree = degree, parametric = parametric, : at -0.005
```

```
## Warning in simpleLoess(y, x, w, span, degree = degree, parametric = parametric, : radius 2.5e-05
```

```
## Warning in simpleLoess(y, x, w, span, degree = degree, parametric = parametric, : all data on boundary of neighborhood. make span bigger
```

```
## Warning in simpleLoess(y, x, w, span, degree = degree, parametric = parametric, : pseudoinverse used at -0.005
```

```
## Warning in simpleLoess(y, x, w, span, degree = degree, parametric = parametric, : neighborhood radius 0.005
```

```
## Warning in simpleLoess(y, x, w, span, degree = degree, parametric = parametric, : reciprocal condition number 1
```

```
## Warning in simpleLoess(y, x, w, span, degree = degree, parametric = parametric, : There are other near singularities as well. 1.01
```

```
## Warning in simpleLoess(y, x, w, span, degree = degree, parametric = parametric, : zero-width neighborhood. make span bigger
```

```
## Warning: Computation failed in `stat_smooth()`:
## NA/NaN/Inf in foreign function call (arg 5)
```

```
## Warning in simpleLoess(y, x, w, span, degree = degree, parametric = parametric, : at -0.005
```

```
## Warning in simpleLoess(y, x, w, span, degree = degree, parametric = parametric, : radius 2.5e-05
```

```
## Warning in simpleLoess(y, x, w, span, degree = degree, parametric = parametric, : all data on boundary of neighborhood. make span bigger
```

```
## Warning in simpleLoess(y, x, w, span, degree = degree, parametric = parametric, : pseudoinverse used at -0.005
```

```
## Warning in simpleLoess(y, x, w, span, degree = degree, parametric = parametric, : neighborhood radius 0.005
```

```
## Warning in simpleLoess(y, x, w, span, degree = degree, parametric = parametric, : reciprocal condition number 1
```

```
## Warning in simpleLoess(y, x, w, span, degree = degree, parametric = parametric, : There are other near singularities as well. 1.01
```

```
## Warning in simpleLoess(y, x, w, span, degree = degree, parametric = parametric, : zero-width neighborhood. make span bigger
```

```
## Warning: Computation failed in `stat_smooth()`:
## NA/NaN/Inf in foreign function call (arg 5)
```

```
## Warning in simpleLoess(y, x, w, span, degree = degree, parametric = parametric, : at -0.005
```

```
## Warning in simpleLoess(y, x, w, span, degree = degree, parametric = parametric, : radius 2.5e-05
```

```
## Warning in simpleLoess(y, x, w, span, degree = degree, parametric = parametric, : all data on boundary of neighborhood. make span bigger
```

```
## Warning in simpleLoess(y, x, w, span, degree = degree, parametric = parametric, : pseudoinverse used at -0.005
```

```
## Warning in simpleLoess(y, x, w, span, degree = degree, parametric = parametric, : neighborhood radius 0.005
```

```
## Warning in simpleLoess(y, x, w, span, degree = degree, parametric = parametric, : reciprocal condition number 1
```

```
## Warning in simpleLoess(y, x, w, span, degree = degree, parametric = parametric, : There are other near singularities as well. 1.01
```

```
## Warning in simpleLoess(y, x, w, span, degree = degree, parametric = parametric, : zero-width neighborhood. make span bigger
```

```
## Warning: Computation failed in `stat_smooth()`:
## NA/NaN/Inf in foreign function call (arg 5)
```

```
## Warning in simpleLoess(y, x, w, span, degree = degree, parametric = parametric, : at -0.005
```

```
## Warning in simpleLoess(y, x, w, span, degree = degree, parametric = parametric, : radius 2.5e-05
```

```
## Warning in simpleLoess(y, x, w, span, degree = degree, parametric = parametric, : all data on boundary of neighborhood. make span bigger
```

```
## Warning in simpleLoess(y, x, w, span, degree = degree, parametric = parametric, : pseudoinverse used at -0.005
```

```
## Warning in simpleLoess(y, x, w, span, degree = degree, parametric = parametric, : neighborhood radius 0.005
```

```
## Warning in simpleLoess(y, x, w, span, degree = degree, parametric = parametric, : reciprocal condition number 1
```

```
## Warning in simpleLoess(y, x, w, span, degree = degree, parametric = parametric, : There are other near singularities as well. 1.01
```

```
## Warning in simpleLoess(y, x, w, span, degree = degree, parametric = parametric, : zero-width neighborhood. make span bigger
```

```
## Warning: Computation failed in `stat_smooth()`:
## NA/NaN/Inf in foreign function call (arg 5)
```

```
## Warning in simpleLoess(y, x, w, span, degree = degree, parametric = parametric, : at -0.005
```

```
## Warning in simpleLoess(y, x, w, span, degree = degree, parametric = parametric, : radius 2.5e-05
```

```
## Warning in simpleLoess(y, x, w, span, degree = degree, parametric = parametric, : all data on boundary of neighborhood. make span bigger
```

```
## Warning in simpleLoess(y, x, w, span, degree = degree, parametric = parametric, : pseudoinverse used at -0.005
```

```
## Warning in simpleLoess(y, x, w, span, degree = degree, parametric = parametric, : neighborhood radius 0.005
```

```
## Warning in simpleLoess(y, x, w, span, degree = degree, parametric = parametric, : reciprocal condition number 1
```

```
## Warning in simpleLoess(y, x, w, span, degree = degree, parametric = parametric, : There are other near singularities as well. 1.01
```

```
## Warning in simpleLoess(y, x, w, span, degree = degree, parametric = parametric, : zero-width neighborhood. make span bigger
```

```
## Warning: Computation failed in `stat_smooth()`:
## NA/NaN/Inf in foreign function call (arg 5)
```

```
## Warning in simpleLoess(y, x, w, span, degree = degree, parametric = parametric, : pseudoinverse used at -0.005
```

```
## Warning in simpleLoess(y, x, w, span, degree = degree, parametric = parametric, : neighborhood radius 1.005
```

```
## Warning in simpleLoess(y, x, w, span, degree = degree, parametric = parametric, : reciprocal condition number 0
```

```
## Warning in simpleLoess(y, x, w, span, degree = degree, parametric = parametric, : There are other near singularities as well. 1.01
```

```
## Warning in predLoess(object$y, object$x, newx = if (is.null(newdata)) object$x else if (is.data.frame(newdata)) as.matrix(model.frame(delete.response(terms(object)), : pseudoinverse used at -0.005
```

```
## Warning in predLoess(object$y, object$x, newx = if (is.null(newdata)) object$x else if (is.data.frame(newdata)) as.matrix(model.frame(delete.response(terms(object)), : neighborhood radius 1.005
```

```
## Warning in predLoess(object$y, object$x, newx = if (is.null(newdata)) object$x else if (is.data.frame(newdata)) as.matrix(model.frame(delete.response(terms(object)), : reciprocal condition number 0
```

```
## Warning in predLoess(object$y, object$x, newx = if (is.null(newdata)) object$x else if (is.data.frame(newdata)) as.matrix(model.frame(delete.response(terms(object)), : There are other near
## singularities as well. 1.01
```

```
## Warning in simpleLoess(y, x, w, span, degree = degree, parametric = parametric, : at -0.005
```

```
## Warning in simpleLoess(y, x, w, span, degree = degree, parametric = parametric, : radius 2.5e-05
```

```
## Warning in simpleLoess(y, x, w, span, degree = degree, parametric = parametric, : all data on boundary of neighborhood. make span bigger
```

```
## Warning in simpleLoess(y, x, w, span, degree = degree, parametric = parametric, : pseudoinverse used at -0.005
```

```
## Warning in simpleLoess(y, x, w, span, degree = degree, parametric = parametric, : neighborhood radius 0.005
```

```
## Warning in simpleLoess(y, x, w, span, degree = degree, parametric = parametric, : reciprocal condition number 1
```

```
## Warning in simpleLoess(y, x, w, span, degree = degree, parametric = parametric, : There are other near singularities as well. 1.01
```

```
## Warning in simpleLoess(y, x, w, span, degree = degree, parametric = parametric, : zero-width neighborhood. make span bigger
```

```
## Warning: Computation failed in `stat_smooth()`:
## NA/NaN/Inf in foreign function call (arg 5)
```

```
## Warning in simpleLoess(y, x, w, span, degree = degree, parametric = parametric, : at -0.005
```

```
## Warning in simpleLoess(y, x, w, span, degree = degree, parametric = parametric, : radius 2.5e-05
```

```
## Warning in simpleLoess(y, x, w, span, degree = degree, parametric = parametric, : all data on boundary of neighborhood. make span bigger
```

```
## Warning in simpleLoess(y, x, w, span, degree = degree, parametric = parametric, : pseudoinverse used at -0.005
```

```
## Warning in simpleLoess(y, x, w, span, degree = degree, parametric = parametric, : neighborhood radius 0.005
```

```
## Warning in simpleLoess(y, x, w, span, degree = degree, parametric = parametric, : reciprocal condition number 1
```

```
## Warning in simpleLoess(y, x, w, span, degree = degree, parametric = parametric, : There are other near singularities as well. 1.01
```

```
## Warning in simpleLoess(y, x, w, span, degree = degree, parametric = parametric, : zero-width neighborhood. make span bigger
```

```
## Warning: Computation failed in `stat_smooth()`:
## NA/NaN/Inf in foreign function call (arg 5)
```

```
## Warning in simpleLoess(y, x, w, span, degree = degree, parametric = parametric, : at -0.005
```

```
## Warning in simpleLoess(y, x, w, span, degree = degree, parametric = parametric, : radius 2.5e-05
```

```
## Warning in simpleLoess(y, x, w, span, degree = degree, parametric = parametric, : all data on boundary of neighborhood. make span bigger
```

```
## Warning in simpleLoess(y, x, w, span, degree = degree, parametric = parametric, : pseudoinverse used at -0.005
```

```
## Warning in simpleLoess(y, x, w, span, degree = degree, parametric = parametric, : neighborhood radius 0.005
```

```
## Warning in simpleLoess(y, x, w, span, degree = degree, parametric = parametric, : reciprocal condition number 1
```

```
## Warning in simpleLoess(y, x, w, span, degree = degree, parametric = parametric, : There are other near singularities as well. 1.01
```

```
## Warning in simpleLoess(y, x, w, span, degree = degree, parametric = parametric, : zero-width neighborhood. make span bigger
```

```
## Warning: Computation failed in `stat_smooth()`:
## NA/NaN/Inf in foreign function call (arg 5)
```

```
## Warning in simpleLoess(y, x, w, span, degree = degree, parametric = parametric, : at -0.005
```

```
## Warning in simpleLoess(y, x, w, span, degree = degree, parametric = parametric, : radius 2.5e-05
```

```
## Warning in simpleLoess(y, x, w, span, degree = degree, parametric = parametric, : all data on boundary of neighborhood. make span bigger
```

```
## Warning in simpleLoess(y, x, w, span, degree = degree, parametric = parametric, : pseudoinverse used at -0.005
```

```
## Warning in simpleLoess(y, x, w, span, degree = degree, parametric = parametric, : neighborhood radius 0.005
```

```
## Warning in simpleLoess(y, x, w, span, degree = degree, parametric = parametric, : reciprocal condition number 1
```

```
## Warning in simpleLoess(y, x, w, span, degree = degree, parametric = parametric, : There are other near singularities as well. 1.01
```

```
## Warning in simpleLoess(y, x, w, span, degree = degree, parametric = parametric, : zero-width neighborhood. make span bigger
```

```
## Warning: Computation failed in `stat_smooth()`:
## NA/NaN/Inf in foreign function call (arg 5)
```

```
## Warning in simpleLoess(y, x, w, span, degree = degree, parametric = parametric, : pseudoinverse used at -0.005
```

```
## Warning in simpleLoess(y, x, w, span, degree = degree, parametric = parametric, : neighborhood radius 1.005
```

```
## Warning in simpleLoess(y, x, w, span, degree = degree, parametric = parametric, : reciprocal condition number 0
```

```
## Warning in simpleLoess(y, x, w, span, degree = degree, parametric = parametric, : There are other near singularities as well. 1.01
```

```
## Warning in predLoess(object$y, object$x, newx = if (is.null(newdata)) object$x else if (is.data.frame(newdata)) as.matrix(model.frame(delete.response(terms(object)), : pseudoinverse used at -0.005
```

```
## Warning in predLoess(object$y, object$x, newx = if (is.null(newdata)) object$x else if (is.data.frame(newdata)) as.matrix(model.frame(delete.response(terms(object)), : neighborhood radius 1.005
```

```
## Warning in predLoess(object$y, object$x, newx = if (is.null(newdata)) object$x else if (is.data.frame(newdata)) as.matrix(model.frame(delete.response(terms(object)), : reciprocal condition number 0
```

```
## Warning in predLoess(object$y, object$x, newx = if (is.null(newdata)) object$x else if (is.data.frame(newdata)) as.matrix(model.frame(delete.response(terms(object)), : There are other near
## singularities as well. 1.01
```

```
## Warning in simpleLoess(y, x, w, span, degree = degree, parametric = parametric, : at -0.005
```

```
## Warning in simpleLoess(y, x, w, span, degree = degree, parametric = parametric, : radius 2.5e-05
```

```
## Warning in simpleLoess(y, x, w, span, degree = degree, parametric = parametric, : all data on boundary of neighborhood. make span bigger
```

```
## Warning in simpleLoess(y, x, w, span, degree = degree, parametric = parametric, : pseudoinverse used at -0.005
```

```
## Warning in simpleLoess(y, x, w, span, degree = degree, parametric = parametric, : neighborhood radius 0.005
```

```
## Warning in simpleLoess(y, x, w, span, degree = degree, parametric = parametric, : reciprocal condition number 1
```

```
## Warning in simpleLoess(y, x, w, span, degree = degree, parametric = parametric, : There are other near singularities as well. 1.01
```

```
## Warning in simpleLoess(y, x, w, span, degree = degree, parametric = parametric, : zero-width neighborhood. make span bigger
```

```
## Warning: Computation failed in `stat_smooth()`:
## NA/NaN/Inf in foreign function call (arg 5)
```

```
## Warning in simpleLoess(y, x, w, span, degree = degree, parametric = parametric, : pseudoinverse used at -0.005
```

```
## Warning in simpleLoess(y, x, w, span, degree = degree, parametric = parametric, : neighborhood radius 1.005
```

```
## Warning in simpleLoess(y, x, w, span, degree = degree, parametric = parametric, : reciprocal condition number 0
```

```
## Warning in simpleLoess(y, x, w, span, degree = degree, parametric = parametric, : There are other near singularities as well. 1.01
```

```
## Warning in predLoess(object$y, object$x, newx = if (is.null(newdata)) object$x else if (is.data.frame(newdata)) as.matrix(model.frame(delete.response(terms(object)), : pseudoinverse used at -0.005
```

```
## Warning in predLoess(object$y, object$x, newx = if (is.null(newdata)) object$x else if (is.data.frame(newdata)) as.matrix(model.frame(delete.response(terms(object)), : neighborhood radius 1.005
```

```
## Warning in predLoess(object$y, object$x, newx = if (is.null(newdata)) object$x else if (is.data.frame(newdata)) as.matrix(model.frame(delete.response(terms(object)), : reciprocal condition number 0
```

```
## Warning in predLoess(object$y, object$x, newx = if (is.null(newdata)) object$x else if (is.data.frame(newdata)) as.matrix(model.frame(delete.response(terms(object)), : There are other near
## singularities as well. 1.01
```

```
## Warning in simpleLoess(y, x, w, span, degree = degree, parametric = parametric, : at -0.005
```

```
## Warning in simpleLoess(y, x, w, span, degree = degree, parametric = parametric, : radius 2.5e-05
```

```
## Warning in simpleLoess(y, x, w, span, degree = degree, parametric = parametric, : all data on boundary of neighborhood. make span bigger
```

```
## Warning in simpleLoess(y, x, w, span, degree = degree, parametric = parametric, : pseudoinverse used at -0.005
```

```
## Warning in simpleLoess(y, x, w, span, degree = degree, parametric = parametric, : neighborhood radius 0.005
```

```
## Warning in simpleLoess(y, x, w, span, degree = degree, parametric = parametric, : reciprocal condition number 1
```

```
## Warning in simpleLoess(y, x, w, span, degree = degree, parametric = parametric, : There are other near singularities as well. 1.01
```

```
## Warning in simpleLoess(y, x, w, span, degree = degree, parametric = parametric, : zero-width neighborhood. make span bigger
```

```
## Warning: Computation failed in `stat_smooth()`:
## NA/NaN/Inf in foreign function call (arg 5)
```

```
## Warning in simpleLoess(y, x, w, span, degree = degree, parametric = parametric, : at -0.005
```

```
## Warning in simpleLoess(y, x, w, span, degree = degree, parametric = parametric, : radius 2.5e-05
```

```
## Warning in simpleLoess(y, x, w, span, degree = degree, parametric = parametric, : all data on boundary of neighborhood. make span bigger
```

```
## Warning in simpleLoess(y, x, w, span, degree = degree, parametric = parametric, : pseudoinverse used at -0.005
```

```
## Warning in simpleLoess(y, x, w, span, degree = degree, parametric = parametric, : neighborhood radius 0.005
```

```
## Warning in simpleLoess(y, x, w, span, degree = degree, parametric = parametric, : reciprocal condition number 1
```

```
## Warning in simpleLoess(y, x, w, span, degree = degree, parametric = parametric, : There are other near singularities as well. 1.01
```

```
## Warning in simpleLoess(y, x, w, span, degree = degree, parametric = parametric, : zero-width neighborhood. make span bigger
```

```
## Warning: Computation failed in `stat_smooth()`:
## NA/NaN/Inf in foreign function call (arg 5)
```

```
## Warning in simpleLoess(y, x, w, span, degree = degree, parametric = parametric, : at -0.005
```

```
## Warning in simpleLoess(y, x, w, span, degree = degree, parametric = parametric, : radius 2.5e-05
```

```
## Warning in simpleLoess(y, x, w, span, degree = degree, parametric = parametric, : all data on boundary of neighborhood. make span bigger
```

```
## Warning in simpleLoess(y, x, w, span, degree = degree, parametric = parametric, : pseudoinverse used at -0.005
```

```
## Warning in simpleLoess(y, x, w, span, degree = degree, parametric = parametric, : neighborhood radius 0.005
```

```
## Warning in simpleLoess(y, x, w, span, degree = degree, parametric = parametric, : reciprocal condition number 1
```

```
## Warning in simpleLoess(y, x, w, span, degree = degree, parametric = parametric, : There are other near singularities as well. 1.01
```

```
## Warning in simpleLoess(y, x, w, span, degree = degree, parametric = parametric, : zero-width neighborhood. make span bigger
```

```
## Warning: Computation failed in `stat_smooth()`:
## NA/NaN/Inf in foreign function call (arg 5)
```

```
## Warning in simpleLoess(y, x, w, span, degree = degree, parametric = parametric, : at -0.005
```

```
## Warning in simpleLoess(y, x, w, span, degree = degree, parametric = parametric, : radius 2.5e-05
```

```
## Warning in simpleLoess(y, x, w, span, degree = degree, parametric = parametric, : all data on boundary of neighborhood. make span bigger
```

```
## Warning in simpleLoess(y, x, w, span, degree = degree, parametric = parametric, : pseudoinverse used at -0.005
```

```
## Warning in simpleLoess(y, x, w, span, degree = degree, parametric = parametric, : neighborhood radius 0.005
```

```
## Warning in simpleLoess(y, x, w, span, degree = degree, parametric = parametric, : reciprocal condition number 1
```

```
## Warning in simpleLoess(y, x, w, span, degree = degree, parametric = parametric, : There are other near singularities as well. 1.01
```

```
## Warning in simpleLoess(y, x, w, span, degree = degree, parametric = parametric, : zero-width neighborhood. make span bigger
```

```
## Warning: Computation failed in `stat_smooth()`:
## NA/NaN/Inf in foreign function call (arg 5)
```

```
## Warning in simpleLoess(y, x, w, span, degree = degree, parametric = parametric, : pseudoinverse used at -0.005
```

```
## Warning in simpleLoess(y, x, w, span, degree = degree, parametric = parametric, : neighborhood radius 1.005
```

```
## Warning in simpleLoess(y, x, w, span, degree = degree, parametric = parametric, : reciprocal condition number 0
```

```
## Warning in simpleLoess(y, x, w, span, degree = degree, parametric = parametric, : There are other near singularities as well. 1.01
```

```
## Warning in predLoess(object$y, object$x, newx = if (is.null(newdata)) object$x else if (is.data.frame(newdata)) as.matrix(model.frame(delete.response(terms(object)), : pseudoinverse used at -0.005
```

```
## Warning in predLoess(object$y, object$x, newx = if (is.null(newdata)) object$x else if (is.data.frame(newdata)) as.matrix(model.frame(delete.response(terms(object)), : neighborhood radius 1.005
```

```
## Warning in predLoess(object$y, object$x, newx = if (is.null(newdata)) object$x else if (is.data.frame(newdata)) as.matrix(model.frame(delete.response(terms(object)), : reciprocal condition number 0
```

```
## Warning in predLoess(object$y, object$x, newx = if (is.null(newdata)) object$x else if (is.data.frame(newdata)) as.matrix(model.frame(delete.response(terms(object)), : There are other near
## singularities as well. 1.01
```

```
## Warning in simpleLoess(y, x, w, span, degree = degree, parametric = parametric, : at -0.005
```

```
## Warning in simpleLoess(y, x, w, span, degree = degree, parametric = parametric, : radius 2.5e-05
```

```
## Warning in simpleLoess(y, x, w, span, degree = degree, parametric = parametric, : all data on boundary of neighborhood. make span bigger
```

```
## Warning in simpleLoess(y, x, w, span, degree = degree, parametric = parametric, : pseudoinverse used at -0.005
```

```
## Warning in simpleLoess(y, x, w, span, degree = degree, parametric = parametric, : neighborhood radius 0.005
```

```
## Warning in simpleLoess(y, x, w, span, degree = degree, parametric = parametric, : reciprocal condition number 1
```

```
## Warning in simpleLoess(y, x, w, span, degree = degree, parametric = parametric, : There are other near singularities as well. 1.01
```

```
## Warning in simpleLoess(y, x, w, span, degree = degree, parametric = parametric, : zero-width neighborhood. make span bigger
```

```
## Warning: Computation failed in `stat_smooth()`:
## NA/NaN/Inf in foreign function call (arg 5)
```

```
## Warning in simpleLoess(y, x, w, span, degree = degree, parametric = parametric, : at -0.005
```

```
## Warning in simpleLoess(y, x, w, span, degree = degree, parametric = parametric, : radius 2.5e-05
```

```
## Warning in simpleLoess(y, x, w, span, degree = degree, parametric = parametric, : all data on boundary of neighborhood. make span bigger
```

```
## Warning in simpleLoess(y, x, w, span, degree = degree, parametric = parametric, : pseudoinverse used at -0.005
```

```
## Warning in simpleLoess(y, x, w, span, degree = degree, parametric = parametric, : neighborhood radius 0.005
```

```
## Warning in simpleLoess(y, x, w, span, degree = degree, parametric = parametric, : reciprocal condition number 1
```

```
## Warning in simpleLoess(y, x, w, span, degree = degree, parametric = parametric, : There are other near singularities as well. 1.01
```

```
## Warning in simpleLoess(y, x, w, span, degree = degree, parametric = parametric, : zero-width neighborhood. make span bigger
```

```
## Warning: Computation failed in `stat_smooth()`:
## NA/NaN/Inf in foreign function call (arg 5)
```

```
## Warning in simpleLoess(y, x, w, span, degree = degree, parametric = parametric, : at -0.005
```

```
## Warning in simpleLoess(y, x, w, span, degree = degree, parametric = parametric, : radius 2.5e-05
```

```
## Warning in simpleLoess(y, x, w, span, degree = degree, parametric = parametric, : all data on boundary of neighborhood. make span bigger
```

```
## Warning in simpleLoess(y, x, w, span, degree = degree, parametric = parametric, : pseudoinverse used at -0.005
```

```
## Warning in simpleLoess(y, x, w, span, degree = degree, parametric = parametric, : neighborhood radius 0.005
```

```
## Warning in simpleLoess(y, x, w, span, degree = degree, parametric = parametric, : reciprocal condition number 1
```

```
## Warning in simpleLoess(y, x, w, span, degree = degree, parametric = parametric, : There are other near singularities as well. 1.01
```

```
## Warning in simpleLoess(y, x, w, span, degree = degree, parametric = parametric, : zero-width neighborhood. make span bigger
```

```
## Warning: Computation failed in `stat_smooth()`:
## NA/NaN/Inf in foreign function call (arg 5)
```

```
## Warning in simpleLoess(y, x, w, span, degree = degree, parametric = parametric, : at -0.005
```

```
## Warning in simpleLoess(y, x, w, span, degree = degree, parametric = parametric, : radius 2.5e-05
```

```
## Warning in simpleLoess(y, x, w, span, degree = degree, parametric = parametric, : all data on boundary of neighborhood. make span bigger
```

```
## Warning in simpleLoess(y, x, w, span, degree = degree, parametric = parametric, : pseudoinverse used at -0.005
```

```
## Warning in simpleLoess(y, x, w, span, degree = degree, parametric = parametric, : neighborhood radius 0.005
```

```
## Warning in simpleLoess(y, x, w, span, degree = degree, parametric = parametric, : reciprocal condition number 1
```

```
## Warning in simpleLoess(y, x, w, span, degree = degree, parametric = parametric, : There are other near singularities as well. 1.01
```

```
## Warning in simpleLoess(y, x, w, span, degree = degree, parametric = parametric, : zero-width neighborhood. make span bigger
```

```
## Warning: Computation failed in `stat_smooth()`:
## NA/NaN/Inf in foreign function call (arg 5)
```

```
## Warning in simpleLoess(y, x, w, span, degree = degree, parametric = parametric, : pseudoinverse used at -0.005
```

```
## Warning in simpleLoess(y, x, w, span, degree = degree, parametric = parametric, : neighborhood radius 1.005
```

```
## Warning in simpleLoess(y, x, w, span, degree = degree, parametric = parametric, : reciprocal condition number 0
```

```
## Warning in simpleLoess(y, x, w, span, degree = degree, parametric = parametric, : There are other near singularities as well. 1.01
```

```
## Warning in predLoess(object$y, object$x, newx = if (is.null(newdata)) object$x else if (is.data.frame(newdata)) as.matrix(model.frame(delete.response(terms(object)), : pseudoinverse used at -0.005
```

```
## Warning in predLoess(object$y, object$x, newx = if (is.null(newdata)) object$x else if (is.data.frame(newdata)) as.matrix(model.frame(delete.response(terms(object)), : neighborhood radius 1.005
```

```
## Warning in predLoess(object$y, object$x, newx = if (is.null(newdata)) object$x else if (is.data.frame(newdata)) as.matrix(model.frame(delete.response(terms(object)), : reciprocal condition number 0
```

```
## Warning in predLoess(object$y, object$x, newx = if (is.null(newdata)) object$x else if (is.data.frame(newdata)) as.matrix(model.frame(delete.response(terms(object)), : There are other near
## singularities as well. 1.01
```

```
## Warning in simpleLoess(y, x, w, span, degree = degree, parametric = parametric, : at -0.005
```

```
## Warning in simpleLoess(y, x, w, span, degree = degree, parametric = parametric, : radius 2.5e-05
```

```
## Warning in simpleLoess(y, x, w, span, degree = degree, parametric = parametric, : all data on boundary of neighborhood. make span bigger
```

```
## Warning in simpleLoess(y, x, w, span, degree = degree, parametric = parametric, : pseudoinverse used at -0.005
```

```
## Warning in simpleLoess(y, x, w, span, degree = degree, parametric = parametric, : neighborhood radius 0.005
```

```
## Warning in simpleLoess(y, x, w, span, degree = degree, parametric = parametric, : reciprocal condition number 1
```

```
## Warning in simpleLoess(y, x, w, span, degree = degree, parametric = parametric, : There are other near singularities as well. 1.01
```

```
## Warning in simpleLoess(y, x, w, span, degree = degree, parametric = parametric, : zero-width neighborhood. make span bigger
```

```
## Warning: Computation failed in `stat_smooth()`:
## NA/NaN/Inf in foreign function call (arg 5)
```

```
## Warning in simpleLoess(y, x, w, span, degree = degree, parametric = parametric, : at -0.005
```

```
## Warning in simpleLoess(y, x, w, span, degree = degree, parametric = parametric, : radius 2.5e-05
```

```
## Warning in simpleLoess(y, x, w, span, degree = degree, parametric = parametric, : all data on boundary of neighborhood. make span bigger
```

```
## Warning in simpleLoess(y, x, w, span, degree = degree, parametric = parametric, : pseudoinverse used at -0.005
```

```
## Warning in simpleLoess(y, x, w, span, degree = degree, parametric = parametric, : neighborhood radius 0.005
```

```
## Warning in simpleLoess(y, x, w, span, degree = degree, parametric = parametric, : reciprocal condition number 1
```

```
## Warning in simpleLoess(y, x, w, span, degree = degree, parametric = parametric, : There are other near singularities as well. 1.01
```

```
## Warning in simpleLoess(y, x, w, span, degree = degree, parametric = parametric, : zero-width neighborhood. make span bigger
```

```
## Warning: Computation failed in `stat_smooth()`:
## NA/NaN/Inf in foreign function call (arg 5)
```

```
## Warning in simpleLoess(y, x, w, span, degree = degree, parametric = parametric, : at -0.005
```

```
## Warning in simpleLoess(y, x, w, span, degree = degree, parametric = parametric, : radius 2.5e-05
```

```
## Warning in simpleLoess(y, x, w, span, degree = degree, parametric = parametric, : all data on boundary of neighborhood. make span bigger
```

```
## Warning in simpleLoess(y, x, w, span, degree = degree, parametric = parametric, : pseudoinverse used at -0.005
```

```
## Warning in simpleLoess(y, x, w, span, degree = degree, parametric = parametric, : neighborhood radius 0.005
```

```
## Warning in simpleLoess(y, x, w, span, degree = degree, parametric = parametric, : reciprocal condition number 1
```

```
## Warning in simpleLoess(y, x, w, span, degree = degree, parametric = parametric, : There are other near singularities as well. 1.01
```

```
## Warning in simpleLoess(y, x, w, span, degree = degree, parametric = parametric, : zero-width neighborhood. make span bigger
```

```
## Warning: Computation failed in `stat_smooth()`:
## NA/NaN/Inf in foreign function call (arg 5)
```

```
## Warning in simpleLoess(y, x, w, span, degree = degree, parametric = parametric, : at -0.005
```

```
## Warning in simpleLoess(y, x, w, span, degree = degree, parametric = parametric, : radius 2.5e-05
```

```
## Warning in simpleLoess(y, x, w, span, degree = degree, parametric = parametric, : all data on boundary of neighborhood. make span bigger
```

```
## Warning in simpleLoess(y, x, w, span, degree = degree, parametric = parametric, : pseudoinverse used at -0.005
```

```
## Warning in simpleLoess(y, x, w, span, degree = degree, parametric = parametric, : neighborhood radius 0.005
```

```
## Warning in simpleLoess(y, x, w, span, degree = degree, parametric = parametric, : reciprocal condition number 1
```

```
## Warning in simpleLoess(y, x, w, span, degree = degree, parametric = parametric, : There are other near singularities as well. 1.01
```

```
## Warning in simpleLoess(y, x, w, span, degree = degree, parametric = parametric, : zero-width neighborhood. make span bigger
```

```
## Warning: Computation failed in `stat_smooth()`:
## NA/NaN/Inf in foreign function call (arg 5)
```

```
## Warning in simpleLoess(y, x, w, span, degree = degree, parametric = parametric, : at -0.005
```

```
## Warning in simpleLoess(y, x, w, span, degree = degree, parametric = parametric, : radius 2.5e-05
```

```
## Warning in simpleLoess(y, x, w, span, degree = degree, parametric = parametric, : all data on boundary of neighborhood. make span bigger
```

```
## Warning in simpleLoess(y, x, w, span, degree = degree, parametric = parametric, : pseudoinverse used at -0.005
```

```
## Warning in simpleLoess(y, x, w, span, degree = degree, parametric = parametric, : neighborhood radius 0.005
```

```
## Warning in simpleLoess(y, x, w, span, degree = degree, parametric = parametric, : reciprocal condition number 1
```

```
## Warning in simpleLoess(y, x, w, span, degree = degree, parametric = parametric, : There are other near singularities as well. 1.01
```

```
## Warning in simpleLoess(y, x, w, span, degree = degree, parametric = parametric, : zero-width neighborhood. make span bigger
```

```
## Warning: Computation failed in `stat_smooth()`:
## NA/NaN/Inf in foreign function call (arg 5)
```

```
## Warning in simpleLoess(y, x, w, span, degree = degree, parametric = parametric, : at -0.005
```

```
## Warning in simpleLoess(y, x, w, span, degree = degree, parametric = parametric, : radius 2.5e-05
```

```
## Warning in simpleLoess(y, x, w, span, degree = degree, parametric = parametric, : all data on boundary of neighborhood. make span bigger
```

```
## Warning in simpleLoess(y, x, w, span, degree = degree, parametric = parametric, : pseudoinverse used at -0.005
```

```
## Warning in simpleLoess(y, x, w, span, degree = degree, parametric = parametric, : neighborhood radius 0.005
```

```
## Warning in simpleLoess(y, x, w, span, degree = degree, parametric = parametric, : reciprocal condition number 1
```

```
## Warning in simpleLoess(y, x, w, span, degree = degree, parametric = parametric, : There are other near singularities as well. 1.01
```

```
## Warning in simpleLoess(y, x, w, span, degree = degree, parametric = parametric, : zero-width neighborhood. make span bigger
```

```
## Warning: Computation failed in `stat_smooth()`:
## NA/NaN/Inf in foreign function call (arg 5)
```

```
## Warning in simpleLoess(y, x, w, span, degree = degree, parametric = parametric, : at -0.005
```

```
## Warning in simpleLoess(y, x, w, span, degree = degree, parametric = parametric, : radius 2.5e-05
```

```
## Warning in simpleLoess(y, x, w, span, degree = degree, parametric = parametric, : all data on boundary of neighborhood. make span bigger
```

```
## Warning in simpleLoess(y, x, w, span, degree = degree, parametric = parametric, : pseudoinverse used at -0.005
```

```
## Warning in simpleLoess(y, x, w, span, degree = degree, parametric = parametric, : neighborhood radius 0.005
```

```
## Warning in simpleLoess(y, x, w, span, degree = degree, parametric = parametric, : reciprocal condition number 1
```

```
## Warning in simpleLoess(y, x, w, span, degree = degree, parametric = parametric, : There are other near singularities as well. 1.01
```

```
## Warning in simpleLoess(y, x, w, span, degree = degree, parametric = parametric, : zero-width neighborhood. make span bigger
```

```
## Warning: Computation failed in `stat_smooth()`:
## NA/NaN/Inf in foreign function call (arg 5)
```

```
## Warning in simpleLoess(y, x, w, span, degree = degree, parametric = parametric, : at -0.005
```

```
## Warning in simpleLoess(y, x, w, span, degree = degree, parametric = parametric, : radius 2.5e-05
```

```
## Warning in simpleLoess(y, x, w, span, degree = degree, parametric = parametric, : all data on boundary of neighborhood. make span bigger
```

```
## Warning in simpleLoess(y, x, w, span, degree = degree, parametric = parametric, : pseudoinverse used at -0.005
```

```
## Warning in simpleLoess(y, x, w, span, degree = degree, parametric = parametric, : neighborhood radius 0.005
```

```
## Warning in simpleLoess(y, x, w, span, degree = degree, parametric = parametric, : reciprocal condition number 1
```

```
## Warning in simpleLoess(y, x, w, span, degree = degree, parametric = parametric, : There are other near singularities as well. 1.01
```

```
## Warning in simpleLoess(y, x, w, span, degree = degree, parametric = parametric, : zero-width neighborhood. make span bigger
```

```
## Warning: Computation failed in `stat_smooth()`:
## NA/NaN/Inf in foreign function call (arg 5)
```

```
## Warning in simpleLoess(y, x, w, span, degree = degree, parametric = parametric, : at -0.005
```

```
## Warning in simpleLoess(y, x, w, span, degree = degree, parametric = parametric, : radius 2.5e-05
```

```
## Warning in simpleLoess(y, x, w, span, degree = degree, parametric = parametric, : all data on boundary of neighborhood. make span bigger
```

```
## Warning in simpleLoess(y, x, w, span, degree = degree, parametric = parametric, : pseudoinverse used at -0.005
```

```
## Warning in simpleLoess(y, x, w, span, degree = degree, parametric = parametric, : neighborhood radius 0.005
```

```
## Warning in simpleLoess(y, x, w, span, degree = degree, parametric = parametric, : reciprocal condition number 1
```

```
## Warning in simpleLoess(y, x, w, span, degree = degree, parametric = parametric, : There are other near singularities as well. 1.01
```

```
## Warning in simpleLoess(y, x, w, span, degree = degree, parametric = parametric, : zero-width neighborhood. make span bigger
```

```
## Warning: Computation failed in `stat_smooth()`:
## NA/NaN/Inf in foreign function call (arg 5)
```

```
## Warning in simpleLoess(y, x, w, span, degree = degree, parametric = parametric, : at -0.005
```

```
## Warning in simpleLoess(y, x, w, span, degree = degree, parametric = parametric, : radius 2.5e-05
```

```
## Warning in simpleLoess(y, x, w, span, degree = degree, parametric = parametric, : all data on boundary of neighborhood. make span bigger
```

```
## Warning in simpleLoess(y, x, w, span, degree = degree, parametric = parametric, : pseudoinverse used at -0.005
```

```
## Warning in simpleLoess(y, x, w, span, degree = degree, parametric = parametric, : neighborhood radius 0.005
```

```
## Warning in simpleLoess(y, x, w, span, degree = degree, parametric = parametric, : reciprocal condition number 1
```

```
## Warning in simpleLoess(y, x, w, span, degree = degree, parametric = parametric, : There are other near singularities as well. 1.01
```

```
## Warning in simpleLoess(y, x, w, span, degree = degree, parametric = parametric, : zero-width neighborhood. make span bigger
```

```
## Warning: Computation failed in `stat_smooth()`:
## NA/NaN/Inf in foreign function call (arg 5)
```

```
## Warning in simpleLoess(y, x, w, span, degree = degree, parametric = parametric, : at -0.005
```

```
## Warning in simpleLoess(y, x, w, span, degree = degree, parametric = parametric, : radius 2.5e-05
```

```
## Warning in simpleLoess(y, x, w, span, degree = degree, parametric = parametric, : all data on boundary of neighborhood. make span bigger
```

```
## Warning in simpleLoess(y, x, w, span, degree = degree, parametric = parametric, : pseudoinverse used at -0.005
```

```
## Warning in simpleLoess(y, x, w, span, degree = degree, parametric = parametric, : neighborhood radius 0.005
```

```
## Warning in simpleLoess(y, x, w, span, degree = degree, parametric = parametric, : reciprocal condition number 1
```

```
## Warning in simpleLoess(y, x, w, span, degree = degree, parametric = parametric, : There are other near singularities as well. 1.01
```

```
## Warning in simpleLoess(y, x, w, span, degree = degree, parametric = parametric, : zero-width neighborhood. make span bigger
```

```
## Warning: Computation failed in `stat_smooth()`:
## NA/NaN/Inf in foreign function call (arg 5)
```

```
## Warning in simpleLoess(y, x, w, span, degree = degree, parametric = parametric, : at -0.005
```

```
## Warning in simpleLoess(y, x, w, span, degree = degree, parametric = parametric, : radius 2.5e-05
```

```
## Warning in simpleLoess(y, x, w, span, degree = degree, parametric = parametric, : all data on boundary of neighborhood. make span bigger
```

```
## Warning in simpleLoess(y, x, w, span, degree = degree, parametric = parametric, : pseudoinverse used at -0.005
```

```
## Warning in simpleLoess(y, x, w, span, degree = degree, parametric = parametric, : neighborhood radius 0.005
```

```
## Warning in simpleLoess(y, x, w, span, degree = degree, parametric = parametric, : reciprocal condition number 1
```

```
## Warning in simpleLoess(y, x, w, span, degree = degree, parametric = parametric, : There are other near singularities as well. 1.01
```

```
## Warning in simpleLoess(y, x, w, span, degree = degree, parametric = parametric, : zero-width neighborhood. make span bigger
```

```
## Warning: Computation failed in `stat_smooth()`:
## NA/NaN/Inf in foreign function call (arg 5)
```

```
## Warning in simpleLoess(y, x, w, span, degree = degree, parametric = parametric, : at -0.005
```

```
## Warning in simpleLoess(y, x, w, span, degree = degree, parametric = parametric, : radius 2.5e-05
```

```
## Warning in simpleLoess(y, x, w, span, degree = degree, parametric = parametric, : all data on boundary of neighborhood. make span bigger
```

```
## Warning in simpleLoess(y, x, w, span, degree = degree, parametric = parametric, : pseudoinverse used at -0.005
```

```
## Warning in simpleLoess(y, x, w, span, degree = degree, parametric = parametric, : neighborhood radius 0.005
```

```
## Warning in simpleLoess(y, x, w, span, degree = degree, parametric = parametric, : reciprocal condition number 1
```

```
## Warning in simpleLoess(y, x, w, span, degree = degree, parametric = parametric, : There are other near singularities as well. 1.01
```

```
## Warning in simpleLoess(y, x, w, span, degree = degree, parametric = parametric, : zero-width neighborhood. make span bigger
```

```
## Warning: Computation failed in `stat_smooth()`:
## NA/NaN/Inf in foreign function call (arg 5)
```

```
## Warning in simpleLoess(y, x, w, span, degree = degree, parametric = parametric, : at -0.005
```

```
## Warning in simpleLoess(y, x, w, span, degree = degree, parametric = parametric, : radius 2.5e-05
```

```
## Warning in simpleLoess(y, x, w, span, degree = degree, parametric = parametric, : all data on boundary of neighborhood. make span bigger
```

```
## Warning in simpleLoess(y, x, w, span, degree = degree, parametric = parametric, : pseudoinverse used at -0.005
```

```
## Warning in simpleLoess(y, x, w, span, degree = degree, parametric = parametric, : neighborhood radius 0.005
```

```
## Warning in simpleLoess(y, x, w, span, degree = degree, parametric = parametric, : reciprocal condition number 1
```

```
## Warning in simpleLoess(y, x, w, span, degree = degree, parametric = parametric, : There are other near singularities as well. 1.01
```

```
## Warning in simpleLoess(y, x, w, span, degree = degree, parametric = parametric, : zero-width neighborhood. make span bigger
```

```
## Warning: Computation failed in `stat_smooth()`:
## NA/NaN/Inf in foreign function call (arg 5)
```

```
## Warning in simpleLoess(y, x, w, span, degree = degree, parametric = parametric, : at -0.005
```

```
## Warning in simpleLoess(y, x, w, span, degree = degree, parametric = parametric, : radius 2.5e-05
```

```
## Warning in simpleLoess(y, x, w, span, degree = degree, parametric = parametric, : all data on boundary of neighborhood. make span bigger
```

```
## Warning in simpleLoess(y, x, w, span, degree = degree, parametric = parametric, : pseudoinverse used at -0.005
```

```
## Warning in simpleLoess(y, x, w, span, degree = degree, parametric = parametric, : neighborhood radius 0.005
```

```
## Warning in simpleLoess(y, x, w, span, degree = degree, parametric = parametric, : reciprocal condition number 1
```

```
## Warning in simpleLoess(y, x, w, span, degree = degree, parametric = parametric, : There are other near singularities as well. 1.01
```

```
## Warning in simpleLoess(y, x, w, span, degree = degree, parametric = parametric, : zero-width neighborhood. make span bigger
```

```
## Warning: Computation failed in `stat_smooth()`:
## NA/NaN/Inf in foreign function call (arg 5)
```

```
## Warning in simpleLoess(y, x, w, span, degree = degree, parametric = parametric, : at -0.005
```

```
## Warning in simpleLoess(y, x, w, span, degree = degree, parametric = parametric, : radius 2.5e-05
```

```
## Warning in simpleLoess(y, x, w, span, degree = degree, parametric = parametric, : all data on boundary of neighborhood. make span bigger
```

```
## Warning in simpleLoess(y, x, w, span, degree = degree, parametric = parametric, : pseudoinverse used at -0.005
```

```
## Warning in simpleLoess(y, x, w, span, degree = degree, parametric = parametric, : neighborhood radius 0.005
```

```
## Warning in simpleLoess(y, x, w, span, degree = degree, parametric = parametric, : reciprocal condition number 1
```

```
## Warning in simpleLoess(y, x, w, span, degree = degree, parametric = parametric, : There are other near singularities as well. 1.01
```

```
## Warning in simpleLoess(y, x, w, span, degree = degree, parametric = parametric, : zero-width neighborhood. make span bigger
```

```
## Warning: Computation failed in `stat_smooth()`:
## NA/NaN/Inf in foreign function call (arg 5)
```

```
## Warning in simpleLoess(y, x, w, span, degree = degree, parametric = parametric, : at -0.005
```

```
## Warning in simpleLoess(y, x, w, span, degree = degree, parametric = parametric, : radius 2.5e-05
```

```
## Warning in simpleLoess(y, x, w, span, degree = degree, parametric = parametric, : all data on boundary of neighborhood. make span bigger
```

```
## Warning in simpleLoess(y, x, w, span, degree = degree, parametric = parametric, : pseudoinverse used at -0.005
```

```
## Warning in simpleLoess(y, x, w, span, degree = degree, parametric = parametric, : neighborhood radius 0.005
```

```
## Warning in simpleLoess(y, x, w, span, degree = degree, parametric = parametric, : reciprocal condition number 1
```

```
## Warning in simpleLoess(y, x, w, span, degree = degree, parametric = parametric, : There are other near singularities as well. 1.01
```

```
## Warning in simpleLoess(y, x, w, span, degree = degree, parametric = parametric, : zero-width neighborhood. make span bigger
```

```
## Warning: Computation failed in `stat_smooth()`:
## NA/NaN/Inf in foreign function call (arg 5)
```

```
## Warning in simpleLoess(y, x, w, span, degree = degree, parametric = parametric, : at -0.005
```

```
## Warning in simpleLoess(y, x, w, span, degree = degree, parametric = parametric, : radius 2.5e-05
```

```
## Warning in simpleLoess(y, x, w, span, degree = degree, parametric = parametric, : all data on boundary of neighborhood. make span bigger
```

```
## Warning in simpleLoess(y, x, w, span, degree = degree, parametric = parametric, : pseudoinverse used at -0.005
```

```
## Warning in simpleLoess(y, x, w, span, degree = degree, parametric = parametric, : neighborhood radius 0.005
```

```
## Warning in simpleLoess(y, x, w, span, degree = degree, parametric = parametric, : reciprocal condition number 1
```

```
## Warning in simpleLoess(y, x, w, span, degree = degree, parametric = parametric, : There are other near singularities as well. 1.01
```

```
## Warning in simpleLoess(y, x, w, span, degree = degree, parametric = parametric, : zero-width neighborhood. make span bigger
```

```
## Warning: Computation failed in `stat_smooth()`:
## NA/NaN/Inf in foreign function call (arg 5)
```

```
## Warning in simpleLoess(y, x, w, span, degree = degree, parametric = parametric, : at -0.005
```

```
## Warning in simpleLoess(y, x, w, span, degree = degree, parametric = parametric, : radius 2.5e-05
```

```
## Warning in simpleLoess(y, x, w, span, degree = degree, parametric = parametric, : all data on boundary of neighborhood. make span bigger
```

```
## Warning in simpleLoess(y, x, w, span, degree = degree, parametric = parametric, : pseudoinverse used at -0.005
```

```
## Warning in simpleLoess(y, x, w, span, degree = degree, parametric = parametric, : neighborhood radius 0.005
```

```
## Warning in simpleLoess(y, x, w, span, degree = degree, parametric = parametric, : reciprocal condition number 1
```

```
## Warning in simpleLoess(y, x, w, span, degree = degree, parametric = parametric, : There are other near singularities as well. 1.01
```

```
## Warning in simpleLoess(y, x, w, span, degree = degree, parametric = parametric, : zero-width neighborhood. make span bigger
```

```
## Warning: Computation failed in `stat_smooth()`:
## NA/NaN/Inf in foreign function call (arg 5)
```

```
## Warning in simpleLoess(y, x, w, span, degree = degree, parametric = parametric, : at -0.005
```

```
## Warning in simpleLoess(y, x, w, span, degree = degree, parametric = parametric, : radius 2.5e-05
```

```
## Warning in simpleLoess(y, x, w, span, degree = degree, parametric = parametric, : all data on boundary of neighborhood. make span bigger
```

```
## Warning in simpleLoess(y, x, w, span, degree = degree, parametric = parametric, : pseudoinverse used at -0.005
```

```
## Warning in simpleLoess(y, x, w, span, degree = degree, parametric = parametric, : neighborhood radius 0.005
```

```
## Warning in simpleLoess(y, x, w, span, degree = degree, parametric = parametric, : reciprocal condition number 1
```

```
## Warning in simpleLoess(y, x, w, span, degree = degree, parametric = parametric, : There are other near singularities as well. 1.01
```

```
## Warning in simpleLoess(y, x, w, span, degree = degree, parametric = parametric, : zero-width neighborhood. make span bigger
```

```
## Warning: Computation failed in `stat_smooth()`:
## NA/NaN/Inf in foreign function call (arg 5)
```

```
## Warning in simpleLoess(y, x, w, span, degree = degree, parametric = parametric, : at -0.005
```

```
## Warning in simpleLoess(y, x, w, span, degree = degree, parametric = parametric, : radius 2.5e-05
```

```
## Warning in simpleLoess(y, x, w, span, degree = degree, parametric = parametric, : all data on boundary of neighborhood. make span bigger
```

```
## Warning in simpleLoess(y, x, w, span, degree = degree, parametric = parametric, : pseudoinverse used at -0.005
```

```
## Warning in simpleLoess(y, x, w, span, degree = degree, parametric = parametric, : neighborhood radius 0.005
```

```
## Warning in simpleLoess(y, x, w, span, degree = degree, parametric = parametric, : reciprocal condition number 1
```

```
## Warning in simpleLoess(y, x, w, span, degree = degree, parametric = parametric, : There are other near singularities as well. 1.01
```

```
## Warning in simpleLoess(y, x, w, span, degree = degree, parametric = parametric, : zero-width neighborhood. make span bigger
```

```
## Warning: Computation failed in `stat_smooth()`:
## NA/NaN/Inf in foreign function call (arg 5)
```

```
## Warning in simpleLoess(y, x, w, span, degree = degree, parametric = parametric, : at -0.005
```

```
## Warning in simpleLoess(y, x, w, span, degree = degree, parametric = parametric, : radius 2.5e-05
```

```
## Warning in simpleLoess(y, x, w, span, degree = degree, parametric = parametric, : all data on boundary of neighborhood. make span bigger
```

```
## Warning in simpleLoess(y, x, w, span, degree = degree, parametric = parametric, : pseudoinverse used at -0.005
```

```
## Warning in simpleLoess(y, x, w, span, degree = degree, parametric = parametric, : neighborhood radius 0.005
```

```
## Warning in simpleLoess(y, x, w, span, degree = degree, parametric = parametric, : reciprocal condition number 1
```

```
## Warning in simpleLoess(y, x, w, span, degree = degree, parametric = parametric, : There are other near singularities as well. 1.01
```

```
## Warning in simpleLoess(y, x, w, span, degree = degree, parametric = parametric, : zero-width neighborhood. make span bigger
```

```
## Warning: Computation failed in `stat_smooth()`:
## NA/NaN/Inf in foreign function call (arg 5)
```

```
## Warning in simpleLoess(y, x, w, span, degree = degree, parametric = parametric, : at -0.005
```

```
## Warning in simpleLoess(y, x, w, span, degree = degree, parametric = parametric, : radius 2.5e-05
```

```
## Warning in simpleLoess(y, x, w, span, degree = degree, parametric = parametric, : all data on boundary of neighborhood. make span bigger
```

```
## Warning in simpleLoess(y, x, w, span, degree = degree, parametric = parametric, : pseudoinverse used at -0.005
```

```
## Warning in simpleLoess(y, x, w, span, degree = degree, parametric = parametric, : neighborhood radius 0.005
```

```
## Warning in simpleLoess(y, x, w, span, degree = degree, parametric = parametric, : reciprocal condition number 1
```

```
## Warning in simpleLoess(y, x, w, span, degree = degree, parametric = parametric, : There are other near singularities as well. 1.01
```

```
## Warning in simpleLoess(y, x, w, span, degree = degree, parametric = parametric, : zero-width neighborhood. make span bigger
```

```
## Warning: Computation failed in `stat_smooth()`:
## NA/NaN/Inf in foreign function call (arg 5)
```

```
## Warning in simpleLoess(y, x, w, span, degree = degree, parametric = parametric, : at -0.005
```

```
## Warning in simpleLoess(y, x, w, span, degree = degree, parametric = parametric, : radius 2.5e-05
```

```
## Warning in simpleLoess(y, x, w, span, degree = degree, parametric = parametric, : all data on boundary of neighborhood. make span bigger
```

```
## Warning in simpleLoess(y, x, w, span, degree = degree, parametric = parametric, : pseudoinverse used at -0.005
```

```
## Warning in simpleLoess(y, x, w, span, degree = degree, parametric = parametric, : neighborhood radius 0.005
```

```
## Warning in simpleLoess(y, x, w, span, degree = degree, parametric = parametric, : reciprocal condition number 1
```

```
## Warning in simpleLoess(y, x, w, span, degree = degree, parametric = parametric, : There are other near singularities as well. 1.01
```

```
## Warning in simpleLoess(y, x, w, span, degree = degree, parametric = parametric, : zero-width neighborhood. make span bigger
```

```
## Warning: Computation failed in `stat_smooth()`:
## NA/NaN/Inf in foreign function call (arg 5)
```

```
## Warning in simpleLoess(y, x, w, span, degree = degree, parametric = parametric, : at -0.005
```

```
## Warning in simpleLoess(y, x, w, span, degree = degree, parametric = parametric, : radius 2.5e-05
```

```
## Warning in simpleLoess(y, x, w, span, degree = degree, parametric = parametric, : all data on boundary of neighborhood. make span bigger
```

```
## Warning in simpleLoess(y, x, w, span, degree = degree, parametric = parametric, : pseudoinverse used at -0.005
```

```
## Warning in simpleLoess(y, x, w, span, degree = degree, parametric = parametric, : neighborhood radius 0.005
```

```
## Warning in simpleLoess(y, x, w, span, degree = degree, parametric = parametric, : reciprocal condition number 1
```

```
## Warning in simpleLoess(y, x, w, span, degree = degree, parametric = parametric, : There are other near singularities as well. 1.01
```

```
## Warning in simpleLoess(y, x, w, span, degree = degree, parametric = parametric, : zero-width neighborhood. make span bigger
```

```
## Warning: Computation failed in `stat_smooth()`:
## NA/NaN/Inf in foreign function call (arg 5)
```

```
## Warning in simpleLoess(y, x, w, span, degree = degree, parametric = parametric, : at -0.005
```

```
## Warning in simpleLoess(y, x, w, span, degree = degree, parametric = parametric, : radius 2.5e-05
```

```
## Warning in simpleLoess(y, x, w, span, degree = degree, parametric = parametric, : all data on boundary of neighborhood. make span bigger
```

```
## Warning in simpleLoess(y, x, w, span, degree = degree, parametric = parametric, : pseudoinverse used at -0.005
```

```
## Warning in simpleLoess(y, x, w, span, degree = degree, parametric = parametric, : neighborhood radius 0.005
```

```
## Warning in simpleLoess(y, x, w, span, degree = degree, parametric = parametric, : reciprocal condition number 1
```

```
## Warning in simpleLoess(y, x, w, span, degree = degree, parametric = parametric, : There are other near singularities as well. 1.01
```

```
## Warning in simpleLoess(y, x, w, span, degree = degree, parametric = parametric, : zero-width neighborhood. make span bigger
```

```
## Warning: Computation failed in `stat_smooth()`:
## NA/NaN/Inf in foreign function call (arg 5)
```

```
## Warning in simpleLoess(y, x, w, span, degree = degree, parametric = parametric, : at -0.005
```

```
## Warning in simpleLoess(y, x, w, span, degree = degree, parametric = parametric, : radius 2.5e-05
```

```
## Warning in simpleLoess(y, x, w, span, degree = degree, parametric = parametric, : all data on boundary of neighborhood. make span bigger
```

```
## Warning in simpleLoess(y, x, w, span, degree = degree, parametric = parametric, : pseudoinverse used at -0.005
```

```
## Warning in simpleLoess(y, x, w, span, degree = degree, parametric = parametric, : neighborhood radius 0.005
```

```
## Warning in simpleLoess(y, x, w, span, degree = degree, parametric = parametric, : reciprocal condition number 1
```

```
## Warning in simpleLoess(y, x, w, span, degree = degree, parametric = parametric, : There are other near singularities as well. 1.01
```

```
## Warning in simpleLoess(y, x, w, span, degree = degree, parametric = parametric, : zero-width neighborhood. make span bigger
```

```
## Warning: Computation failed in `stat_smooth()`:
## NA/NaN/Inf in foreign function call (arg 5)
```

```
## Warning in simpleLoess(y, x, w, span, degree = degree, parametric = parametric, : at -0.005
```

```
## Warning in simpleLoess(y, x, w, span, degree = degree, parametric = parametric, : radius 2.5e-05
```

```
## Warning in simpleLoess(y, x, w, span, degree = degree, parametric = parametric, : all data on boundary of neighborhood. make span bigger
```

```
## Warning in simpleLoess(y, x, w, span, degree = degree, parametric = parametric, : pseudoinverse used at -0.005
```

```
## Warning in simpleLoess(y, x, w, span, degree = degree, parametric = parametric, : neighborhood radius 0.005
```

```
## Warning in simpleLoess(y, x, w, span, degree = degree, parametric = parametric, : reciprocal condition number 1
```

```
## Warning in simpleLoess(y, x, w, span, degree = degree, parametric = parametric, : There are other near singularities as well. 1.01
```

```
## Warning in simpleLoess(y, x, w, span, degree = degree, parametric = parametric, : zero-width neighborhood. make span bigger
```

```
## Warning: Computation failed in `stat_smooth()`:
## NA/NaN/Inf in foreign function call (arg 5)
```

```
## Warning in simpleLoess(y, x, w, span, degree = degree, parametric = parametric, : at -0.005
```

```
## Warning in simpleLoess(y, x, w, span, degree = degree, parametric = parametric, : radius 2.5e-05
```

```
## Warning in simpleLoess(y, x, w, span, degree = degree, parametric = parametric, : all data on boundary of neighborhood. make span bigger
```

```
## Warning in simpleLoess(y, x, w, span, degree = degree, parametric = parametric, : pseudoinverse used at -0.005
```

```
## Warning in simpleLoess(y, x, w, span, degree = degree, parametric = parametric, : neighborhood radius 0.005
```

```
## Warning in simpleLoess(y, x, w, span, degree = degree, parametric = parametric, : reciprocal condition number 1
```

```
## Warning in simpleLoess(y, x, w, span, degree = degree, parametric = parametric, : There are other near singularities as well. 1.01
```

```
## Warning in simpleLoess(y, x, w, span, degree = degree, parametric = parametric, : zero-width neighborhood. make span bigger
```

```
## Warning: Computation failed in `stat_smooth()`:
## NA/NaN/Inf in foreign function call (arg 5)
```

```
## Warning in simpleLoess(y, x, w, span, degree = degree, parametric = parametric, : at -0.005
```

```
## Warning in simpleLoess(y, x, w, span, degree = degree, parametric = parametric, : radius 2.5e-05
```

```
## Warning in simpleLoess(y, x, w, span, degree = degree, parametric = parametric, : all data on boundary of neighborhood. make span bigger
```

```
## Warning in simpleLoess(y, x, w, span, degree = degree, parametric = parametric, : pseudoinverse used at -0.005
```

```
## Warning in simpleLoess(y, x, w, span, degree = degree, parametric = parametric, : neighborhood radius 0.005
```

```
## Warning in simpleLoess(y, x, w, span, degree = degree, parametric = parametric, : reciprocal condition number 1
```

```
## Warning in simpleLoess(y, x, w, span, degree = degree, parametric = parametric, : There are other near singularities as well. 1.01
```

```
## Warning in simpleLoess(y, x, w, span, degree = degree, parametric = parametric, : zero-width neighborhood. make span bigger
```

```
## Warning: Computation failed in `stat_smooth()`:
## NA/NaN/Inf in foreign function call (arg 5)
```

```
## Warning in simpleLoess(y, x, w, span, degree = degree, parametric = parametric, : at -0.005
```

```
## Warning in simpleLoess(y, x, w, span, degree = degree, parametric = parametric, : radius 2.5e-05
```

```
## Warning in simpleLoess(y, x, w, span, degree = degree, parametric = parametric, : all data on boundary of neighborhood. make span bigger
```

```
## Warning in simpleLoess(y, x, w, span, degree = degree, parametric = parametric, : pseudoinverse used at -0.005
```

```
## Warning in simpleLoess(y, x, w, span, degree = degree, parametric = parametric, : neighborhood radius 0.005
```

```
## Warning in simpleLoess(y, x, w, span, degree = degree, parametric = parametric, : reciprocal condition number 1
```

```
## Warning in simpleLoess(y, x, w, span, degree = degree, parametric = parametric, : There are other near singularities as well. 1.01
```

```
## Warning in simpleLoess(y, x, w, span, degree = degree, parametric = parametric, : zero-width neighborhood. make span bigger
```

```
## Warning: Computation failed in `stat_smooth()`:
## NA/NaN/Inf in foreign function call (arg 5)
```

```
## Warning in simpleLoess(y, x, w, span, degree = degree, parametric = parametric, : pseudoinverse used at -0.005
```

```
## Warning in simpleLoess(y, x, w, span, degree = degree, parametric = parametric, : neighborhood radius 1.005
```

```
## Warning in simpleLoess(y, x, w, span, degree = degree, parametric = parametric, : reciprocal condition number 0
```

```
## Warning in simpleLoess(y, x, w, span, degree = degree, parametric = parametric, : There are other near singularities as well. 1.01
```

```
## Warning in predLoess(object$y, object$x, newx = if (is.null(newdata)) object$x else if (is.data.frame(newdata)) as.matrix(model.frame(delete.response(terms(object)), : pseudoinverse used at -0.005
```

```
## Warning in predLoess(object$y, object$x, newx = if (is.null(newdata)) object$x else if (is.data.frame(newdata)) as.matrix(model.frame(delete.response(terms(object)), : neighborhood radius 1.005
```

```
## Warning in predLoess(object$y, object$x, newx = if (is.null(newdata)) object$x else if (is.data.frame(newdata)) as.matrix(model.frame(delete.response(terms(object)), : reciprocal condition number 0
```

```
## Warning in predLoess(object$y, object$x, newx = if (is.null(newdata)) object$x else if (is.data.frame(newdata)) as.matrix(model.frame(delete.response(terms(object)), : There are other near
## singularities as well. 1.01
```

```
## Warning in simpleLoess(y, x, w, span, degree = degree, parametric = parametric, : at -0.005
```

```
## Warning in simpleLoess(y, x, w, span, degree = degree, parametric = parametric, : radius 2.5e-05
```

```
## Warning in simpleLoess(y, x, w, span, degree = degree, parametric = parametric, : all data on boundary of neighborhood. make span bigger
```

```
## Warning in simpleLoess(y, x, w, span, degree = degree, parametric = parametric, : pseudoinverse used at -0.005
```

```
## Warning in simpleLoess(y, x, w, span, degree = degree, parametric = parametric, : neighborhood radius 0.005
```

```
## Warning in simpleLoess(y, x, w, span, degree = degree, parametric = parametric, : reciprocal condition number 1
```

```
## Warning in simpleLoess(y, x, w, span, degree = degree, parametric = parametric, : There are other near singularities as well. 1.01
```

```
## Warning in simpleLoess(y, x, w, span, degree = degree, parametric = parametric, : zero-width neighborhood. make span bigger
```

```
## Warning: Computation failed in `stat_smooth()`:
## NA/NaN/Inf in foreign function call (arg 5)
```

```
## Warning in simpleLoess(y, x, w, span, degree = degree, parametric = parametric, : at -0.005
```

```
## Warning in simpleLoess(y, x, w, span, degree = degree, parametric = parametric, : radius 2.5e-05
```

```
## Warning in simpleLoess(y, x, w, span, degree = degree, parametric = parametric, : all data on boundary of neighborhood. make span bigger
```

```
## Warning in simpleLoess(y, x, w, span, degree = degree, parametric = parametric, : pseudoinverse used at -0.005
```

```
## Warning in simpleLoess(y, x, w, span, degree = degree, parametric = parametric, : neighborhood radius 0.005
```

```
## Warning in simpleLoess(y, x, w, span, degree = degree, parametric = parametric, : reciprocal condition number 1
```

```
## Warning in simpleLoess(y, x, w, span, degree = degree, parametric = parametric, : There are other near singularities as well. 1.01
```

```
## Warning in simpleLoess(y, x, w, span, degree = degree, parametric = parametric, : zero-width neighborhood. make span bigger
```

```
## Warning: Computation failed in `stat_smooth()`:
## NA/NaN/Inf in foreign function call (arg 5)
```

```
## Warning in simpleLoess(y, x, w, span, degree = degree, parametric = parametric, : at -0.005
```

```
## Warning in simpleLoess(y, x, w, span, degree = degree, parametric = parametric, : radius 2.5e-05
```

```
## Warning in simpleLoess(y, x, w, span, degree = degree, parametric = parametric, : all data on boundary of neighborhood. make span bigger
```

```
## Warning in simpleLoess(y, x, w, span, degree = degree, parametric = parametric, : pseudoinverse used at -0.005
```

```
## Warning in simpleLoess(y, x, w, span, degree = degree, parametric = parametric, : neighborhood radius 0.005
```

```
## Warning in simpleLoess(y, x, w, span, degree = degree, parametric = parametric, : reciprocal condition number 1
```

```
## Warning in simpleLoess(y, x, w, span, degree = degree, parametric = parametric, : There are other near singularities as well. 1.01
```

```
## Warning in simpleLoess(y, x, w, span, degree = degree, parametric = parametric, : zero-width neighborhood. make span bigger
```

```
## Warning: Computation failed in `stat_smooth()`:
## NA/NaN/Inf in foreign function call (arg 5)
```

```
## Warning in simpleLoess(y, x, w, span, degree = degree, parametric = parametric, : at -0.005
```

```
## Warning in simpleLoess(y, x, w, span, degree = degree, parametric = parametric, : radius 2.5e-05
```

```
## Warning in simpleLoess(y, x, w, span, degree = degree, parametric = parametric, : all data on boundary of neighborhood. make span bigger
```

```
## Warning in simpleLoess(y, x, w, span, degree = degree, parametric = parametric, : pseudoinverse used at -0.005
```

```
## Warning in simpleLoess(y, x, w, span, degree = degree, parametric = parametric, : neighborhood radius 0.005
```

```
## Warning in simpleLoess(y, x, w, span, degree = degree, parametric = parametric, : reciprocal condition number 1
```

```
## Warning in simpleLoess(y, x, w, span, degree = degree, parametric = parametric, : There are other near singularities as well. 1.01
```

```
## Warning in simpleLoess(y, x, w, span, degree = degree, parametric = parametric, : zero-width neighborhood. make span bigger
```

```
## Warning: Computation failed in `stat_smooth()`:
## NA/NaN/Inf in foreign function call (arg 5)
```

```
## Warning in simpleLoess(y, x, w, span, degree = degree, parametric = parametric, : at -0.005
```

```
## Warning in simpleLoess(y, x, w, span, degree = degree, parametric = parametric, : radius 2.5e-05
```

```
## Warning in simpleLoess(y, x, w, span, degree = degree, parametric = parametric, : all data on boundary of neighborhood. make span bigger
```

```
## Warning in simpleLoess(y, x, w, span, degree = degree, parametric = parametric, : pseudoinverse used at -0.005
```

```
## Warning in simpleLoess(y, x, w, span, degree = degree, parametric = parametric, : neighborhood radius 0.005
```

```
## Warning in simpleLoess(y, x, w, span, degree = degree, parametric = parametric, : reciprocal condition number 1
```

```
## Warning in simpleLoess(y, x, w, span, degree = degree, parametric = parametric, : There are other near singularities as well. 1.01
```

```
## Warning in simpleLoess(y, x, w, span, degree = degree, parametric = parametric, : zero-width neighborhood. make span bigger
```

```
## Warning: Computation failed in `stat_smooth()`:
## NA/NaN/Inf in foreign function call (arg 5)
```

```
## Warning in simpleLoess(y, x, w, span, degree = degree, parametric = parametric, : at -0.005
```

```
## Warning in simpleLoess(y, x, w, span, degree = degree, parametric = parametric, : radius 2.5e-05
```

```
## Warning in simpleLoess(y, x, w, span, degree = degree, parametric = parametric, : all data on boundary of neighborhood. make span bigger
```

```
## Warning in simpleLoess(y, x, w, span, degree = degree, parametric = parametric, : pseudoinverse used at -0.005
```

```
## Warning in simpleLoess(y, x, w, span, degree = degree, parametric = parametric, : neighborhood radius 0.005
```

```
## Warning in simpleLoess(y, x, w, span, degree = degree, parametric = parametric, : reciprocal condition number 1
```

```
## Warning in simpleLoess(y, x, w, span, degree = degree, parametric = parametric, : There are other near singularities as well. 1.01
```

```
## Warning in simpleLoess(y, x, w, span, degree = degree, parametric = parametric, : zero-width neighborhood. make span bigger
```

```
## Warning: Computation failed in `stat_smooth()`:
## NA/NaN/Inf in foreign function call (arg 5)
```

```
## Warning in simpleLoess(y, x, w, span, degree = degree, parametric = parametric, : at -0.005
```

```
## Warning in simpleLoess(y, x, w, span, degree = degree, parametric = parametric, : radius 2.5e-05
```

```
## Warning in simpleLoess(y, x, w, span, degree = degree, parametric = parametric, : all data on boundary of neighborhood. make span bigger
```

```
## Warning in simpleLoess(y, x, w, span, degree = degree, parametric = parametric, : pseudoinverse used at -0.005
```

```
## Warning in simpleLoess(y, x, w, span, degree = degree, parametric = parametric, : neighborhood radius 0.005
```

```
## Warning in simpleLoess(y, x, w, span, degree = degree, parametric = parametric, : reciprocal condition number 1
```

```
## Warning in simpleLoess(y, x, w, span, degree = degree, parametric = parametric, : There are other near singularities as well. 1.01
```

```
## Warning in simpleLoess(y, x, w, span, degree = degree, parametric = parametric, : zero-width neighborhood. make span bigger
```

```
## Warning: Computation failed in `stat_smooth()`:
## NA/NaN/Inf in foreign function call (arg 5)
```

```
## Warning in simpleLoess(y, x, w, span, degree = degree, parametric = parametric, : at -0.005
```

```
## Warning in simpleLoess(y, x, w, span, degree = degree, parametric = parametric, : radius 2.5e-05
```

```
## Warning in simpleLoess(y, x, w, span, degree = degree, parametric = parametric, : all data on boundary of neighborhood. make span bigger
```

```
## Warning in simpleLoess(y, x, w, span, degree = degree, parametric = parametric, : pseudoinverse used at -0.005
```

```
## Warning in simpleLoess(y, x, w, span, degree = degree, parametric = parametric, : neighborhood radius 0.005
```

```
## Warning in simpleLoess(y, x, w, span, degree = degree, parametric = parametric, : reciprocal condition number 1
```

```
## Warning in simpleLoess(y, x, w, span, degree = degree, parametric = parametric, : There are other near singularities as well. 1.01
```

```
## Warning in simpleLoess(y, x, w, span, degree = degree, parametric = parametric, : zero-width neighborhood. make span bigger
```

```
## Warning: Computation failed in `stat_smooth()`:
## NA/NaN/Inf in foreign function call (arg 5)
```

```
## Warning in simpleLoess(y, x, w, span, degree = degree, parametric = parametric, : at -0.005
```

```
## Warning in simpleLoess(y, x, w, span, degree = degree, parametric = parametric, : radius 2.5e-05
```

```
## Warning in simpleLoess(y, x, w, span, degree = degree, parametric = parametric, : all data on boundary of neighborhood. make span bigger
```

```
## Warning in simpleLoess(y, x, w, span, degree = degree, parametric = parametric, : pseudoinverse used at -0.005
```

```
## Warning in simpleLoess(y, x, w, span, degree = degree, parametric = parametric, : neighborhood radius 0.005
```

```
## Warning in simpleLoess(y, x, w, span, degree = degree, parametric = parametric, : reciprocal condition number 1
```

```
## Warning in simpleLoess(y, x, w, span, degree = degree, parametric = parametric, : There are other near singularities as well. 1.01
```

```
## Warning in simpleLoess(y, x, w, span, degree = degree, parametric = parametric, : zero-width neighborhood. make span bigger
```

```
## Warning: Computation failed in `stat_smooth()`:
## NA/NaN/Inf in foreign function call (arg 5)
```

```
## Warning in simpleLoess(y, x, w, span, degree = degree, parametric = parametric, : at -0.005
```

```
## Warning in simpleLoess(y, x, w, span, degree = degree, parametric = parametric, : radius 2.5e-05
```

```
## Warning in simpleLoess(y, x, w, span, degree = degree, parametric = parametric, : all data on boundary of neighborhood. make span bigger
```

```
## Warning in simpleLoess(y, x, w, span, degree = degree, parametric = parametric, : pseudoinverse used at -0.005
```

```
## Warning in simpleLoess(y, x, w, span, degree = degree, parametric = parametric, : neighborhood radius 0.005
```

```
## Warning in simpleLoess(y, x, w, span, degree = degree, parametric = parametric, : reciprocal condition number 1
```

```
## Warning in simpleLoess(y, x, w, span, degree = degree, parametric = parametric, : There are other near singularities as well. 1.01
```

```
## Warning in simpleLoess(y, x, w, span, degree = degree, parametric = parametric, : zero-width neighborhood. make span bigger
```

```
## Warning: Computation failed in `stat_smooth()`:
## NA/NaN/Inf in foreign function call (arg 5)
```

```
## Warning in simpleLoess(y, x, w, span, degree = degree, parametric = parametric, : at -0.005
```

```
## Warning in simpleLoess(y, x, w, span, degree = degree, parametric = parametric, : radius 2.5e-05
```

```
## Warning in simpleLoess(y, x, w, span, degree = degree, parametric = parametric, : all data on boundary of neighborhood. make span bigger
```

```
## Warning in simpleLoess(y, x, w, span, degree = degree, parametric = parametric, : pseudoinverse used at -0.005
```

```
## Warning in simpleLoess(y, x, w, span, degree = degree, parametric = parametric, : neighborhood radius 0.005
```

```
## Warning in simpleLoess(y, x, w, span, degree = degree, parametric = parametric, : reciprocal condition number 1
```

```
## Warning in simpleLoess(y, x, w, span, degree = degree, parametric = parametric, : There are other near singularities as well. 1.01
```

```
## Warning in simpleLoess(y, x, w, span, degree = degree, parametric = parametric, : zero-width neighborhood. make span bigger
```

```
## Warning: Computation failed in `stat_smooth()`:
## NA/NaN/Inf in foreign function call (arg 5)
```

```
## Warning in simpleLoess(y, x, w, span, degree = degree, parametric = parametric, : at -0.005
```

```
## Warning in simpleLoess(y, x, w, span, degree = degree, parametric = parametric, : radius 2.5e-05
```

```
## Warning in simpleLoess(y, x, w, span, degree = degree, parametric = parametric, : all data on boundary of neighborhood. make span bigger
```

```
## Warning in simpleLoess(y, x, w, span, degree = degree, parametric = parametric, : pseudoinverse used at -0.005
```

```
## Warning in simpleLoess(y, x, w, span, degree = degree, parametric = parametric, : neighborhood radius 0.005
```

```
## Warning in simpleLoess(y, x, w, span, degree = degree, parametric = parametric, : reciprocal condition number 1
```

```
## Warning in simpleLoess(y, x, w, span, degree = degree, parametric = parametric, : There are other near singularities as well. 1.01
```

```
## Warning in simpleLoess(y, x, w, span, degree = degree, parametric = parametric, : zero-width neighborhood. make span bigger
```

```
## Warning: Computation failed in `stat_smooth()`:
## NA/NaN/Inf in foreign function call (arg 5)
```

```
## Warning in simpleLoess(y, x, w, span, degree = degree, parametric = parametric, : at -0.005
```

```
## Warning in simpleLoess(y, x, w, span, degree = degree, parametric = parametric, : radius 2.5e-05
```

```
## Warning in simpleLoess(y, x, w, span, degree = degree, parametric = parametric, : all data on boundary of neighborhood. make span bigger
```

```
## Warning in simpleLoess(y, x, w, span, degree = degree, parametric = parametric, : pseudoinverse used at -0.005
```

```
## Warning in simpleLoess(y, x, w, span, degree = degree, parametric = parametric, : neighborhood radius 0.005
```

```
## Warning in simpleLoess(y, x, w, span, degree = degree, parametric = parametric, : reciprocal condition number 1
```

```
## Warning in simpleLoess(y, x, w, span, degree = degree, parametric = parametric, : There are other near singularities as well. 1.01
```

```
## Warning in simpleLoess(y, x, w, span, degree = degree, parametric = parametric, : zero-width neighborhood. make span bigger
```

```
## Warning: Computation failed in `stat_smooth()`:
## NA/NaN/Inf in foreign function call (arg 5)
```

```
## Warning in simpleLoess(y, x, w, span, degree = degree, parametric = parametric, : at -0.005
```

```
## Warning in simpleLoess(y, x, w, span, degree = degree, parametric = parametric, : radius 2.5e-05
```

```
## Warning in simpleLoess(y, x, w, span, degree = degree, parametric = parametric, : all data on boundary of neighborhood. make span bigger
```

```
## Warning in simpleLoess(y, x, w, span, degree = degree, parametric = parametric, : pseudoinverse used at -0.005
```

```
## Warning in simpleLoess(y, x, w, span, degree = degree, parametric = parametric, : neighborhood radius 0.005
```

```
## Warning in simpleLoess(y, x, w, span, degree = degree, parametric = parametric, : reciprocal condition number 1
```

```
## Warning in simpleLoess(y, x, w, span, degree = degree, parametric = parametric, : There are other near singularities as well. 1.01
```

```
## Warning in simpleLoess(y, x, w, span, degree = degree, parametric = parametric, : zero-width neighborhood. make span bigger
```

```
## Warning: Computation failed in `stat_smooth()`:
## NA/NaN/Inf in foreign function call (arg 5)
```

```
## Warning in simpleLoess(y, x, w, span, degree = degree, parametric = parametric, : at -0.005
```

```
## Warning in simpleLoess(y, x, w, span, degree = degree, parametric = parametric, : radius 2.5e-05
```

```
## Warning in simpleLoess(y, x, w, span, degree = degree, parametric = parametric, : all data on boundary of neighborhood. make span bigger
```

```
## Warning in simpleLoess(y, x, w, span, degree = degree, parametric = parametric, : pseudoinverse used at -0.005
```

```
## Warning in simpleLoess(y, x, w, span, degree = degree, parametric = parametric, : neighborhood radius 0.005
```

```
## Warning in simpleLoess(y, x, w, span, degree = degree, parametric = parametric, : reciprocal condition number 1
```

```
## Warning in simpleLoess(y, x, w, span, degree = degree, parametric = parametric, : There are other near singularities as well. 1.01
```

```
## Warning in simpleLoess(y, x, w, span, degree = degree, parametric = parametric, : zero-width neighborhood. make span bigger
```

```
## Warning: Computation failed in `stat_smooth()`:
## NA/NaN/Inf in foreign function call (arg 5)
```

```
## Warning in simpleLoess(y, x, w, span, degree = degree, parametric = parametric, : at -0.005
```

```
## Warning in simpleLoess(y, x, w, span, degree = degree, parametric = parametric, : radius 2.5e-05
```

```
## Warning in simpleLoess(y, x, w, span, degree = degree, parametric = parametric, : all data on boundary of neighborhood. make span bigger
```

```
## Warning in simpleLoess(y, x, w, span, degree = degree, parametric = parametric, : pseudoinverse used at -0.005
```

```
## Warning in simpleLoess(y, x, w, span, degree = degree, parametric = parametric, : neighborhood radius 0.005
```

```
## Warning in simpleLoess(y, x, w, span, degree = degree, parametric = parametric, : reciprocal condition number 1
```

```
## Warning in simpleLoess(y, x, w, span, degree = degree, parametric = parametric, : There are other near singularities as well. 1.01
```

```
## Warning in simpleLoess(y, x, w, span, degree = degree, parametric = parametric, : zero-width neighborhood. make span bigger
```

```
## Warning: Computation failed in `stat_smooth()`:
## NA/NaN/Inf in foreign function call (arg 5)
```

```
## Warning in simpleLoess(y, x, w, span, degree = degree, parametric = parametric, : at -0.005
```

```
## Warning in simpleLoess(y, x, w, span, degree = degree, parametric = parametric, : radius 2.5e-05
```

```
## Warning in simpleLoess(y, x, w, span, degree = degree, parametric = parametric, : all data on boundary of neighborhood. make span bigger
```

```
## Warning in simpleLoess(y, x, w, span, degree = degree, parametric = parametric, : pseudoinverse used at -0.005
```

```
## Warning in simpleLoess(y, x, w, span, degree = degree, parametric = parametric, : neighborhood radius 0.005
```

```
## Warning in simpleLoess(y, x, w, span, degree = degree, parametric = parametric, : reciprocal condition number 1
```

```
## Warning in simpleLoess(y, x, w, span, degree = degree, parametric = parametric, : There are other near singularities as well. 1.01
```

```
## Warning in simpleLoess(y, x, w, span, degree = degree, parametric = parametric, : zero-width neighborhood. make span bigger
```

```
## Warning: Computation failed in `stat_smooth()`:
## NA/NaN/Inf in foreign function call (arg 5)
```

```
## Warning in simpleLoess(y, x, w, span, degree = degree, parametric = parametric, : pseudoinverse used at -0.005
```

```
## Warning in simpleLoess(y, x, w, span, degree = degree, parametric = parametric, : neighborhood radius 1.005
```

```
## Warning in simpleLoess(y, x, w, span, degree = degree, parametric = parametric, : reciprocal condition number 0
```

```
## Warning in simpleLoess(y, x, w, span, degree = degree, parametric = parametric, : There are other near singularities as well. 1.01
```

```
## Warning in predLoess(object$y, object$x, newx = if (is.null(newdata)) object$x else if (is.data.frame(newdata)) as.matrix(model.frame(delete.response(terms(object)), : pseudoinverse used at -0.005
```

```
## Warning in predLoess(object$y, object$x, newx = if (is.null(newdata)) object$x else if (is.data.frame(newdata)) as.matrix(model.frame(delete.response(terms(object)), : neighborhood radius 1.005
```

```
## Warning in predLoess(object$y, object$x, newx = if (is.null(newdata)) object$x else if (is.data.frame(newdata)) as.matrix(model.frame(delete.response(terms(object)), : reciprocal condition number 0
```

```
## Warning in predLoess(object$y, object$x, newx = if (is.null(newdata)) object$x else if (is.data.frame(newdata)) as.matrix(model.frame(delete.response(terms(object)), : There are other near
## singularities as well. 1.01
```

```
## Warning in simpleLoess(y, x, w, span, degree = degree, parametric = parametric, : at -0.005
```

```
## Warning in simpleLoess(y, x, w, span, degree = degree, parametric = parametric, : radius 2.5e-05
```

```
## Warning in simpleLoess(y, x, w, span, degree = degree, parametric = parametric, : all data on boundary of neighborhood. make span bigger
```

```
## Warning in simpleLoess(y, x, w, span, degree = degree, parametric = parametric, : pseudoinverse used at -0.005
```

```
## Warning in simpleLoess(y, x, w, span, degree = degree, parametric = parametric, : neighborhood radius 0.005
```

```
## Warning in simpleLoess(y, x, w, span, degree = degree, parametric = parametric, : reciprocal condition number 1
```

```
## Warning in simpleLoess(y, x, w, span, degree = degree, parametric = parametric, : There are other near singularities as well. 1.01
```

```
## Warning in simpleLoess(y, x, w, span, degree = degree, parametric = parametric, : zero-width neighborhood. make span bigger
```

```
## Warning: Computation failed in `stat_smooth()`:
## NA/NaN/Inf in foreign function call (arg 5)
```

```
## Warning in simpleLoess(y, x, w, span, degree = degree, parametric = parametric, : at -0.005
```

```
## Warning in simpleLoess(y, x, w, span, degree = degree, parametric = parametric, : radius 2.5e-05
```

```
## Warning in simpleLoess(y, x, w, span, degree = degree, parametric = parametric, : all data on boundary of neighborhood. make span bigger
```

```
## Warning in simpleLoess(y, x, w, span, degree = degree, parametric = parametric, : pseudoinverse used at -0.005
```

```
## Warning in simpleLoess(y, x, w, span, degree = degree, parametric = parametric, : neighborhood radius 0.005
```

```
## Warning in simpleLoess(y, x, w, span, degree = degree, parametric = parametric, : reciprocal condition number 1
```

```
## Warning in simpleLoess(y, x, w, span, degree = degree, parametric = parametric, : There are other near singularities as well. 1.01
```

```
## Warning in simpleLoess(y, x, w, span, degree = degree, parametric = parametric, : zero-width neighborhood. make span bigger
```

```
## Warning: Computation failed in `stat_smooth()`:
## NA/NaN/Inf in foreign function call (arg 5)
```

```
## Warning in simpleLoess(y, x, w, span, degree = degree, parametric = parametric, : at -0.005
```

```
## Warning in simpleLoess(y, x, w, span, degree = degree, parametric = parametric, : radius 2.5e-05
```

```
## Warning in simpleLoess(y, x, w, span, degree = degree, parametric = parametric, : all data on boundary of neighborhood. make span bigger
```

```
## Warning in simpleLoess(y, x, w, span, degree = degree, parametric = parametric, : pseudoinverse used at -0.005
```

```
## Warning in simpleLoess(y, x, w, span, degree = degree, parametric = parametric, : neighborhood radius 0.005
```

```
## Warning in simpleLoess(y, x, w, span, degree = degree, parametric = parametric, : reciprocal condition number 1
```

```
## Warning in simpleLoess(y, x, w, span, degree = degree, parametric = parametric, : There are other near singularities as well. 1.01
```

```
## Warning in simpleLoess(y, x, w, span, degree = degree, parametric = parametric, : zero-width neighborhood. make span bigger
```

```
## Warning: Computation failed in `stat_smooth()`:
## NA/NaN/Inf in foreign function call (arg 5)
```

```
## Warning in simpleLoess(y, x, w, span, degree = degree, parametric = parametric, : at -0.005
```

```
## Warning in simpleLoess(y, x, w, span, degree = degree, parametric = parametric, : radius 2.5e-05
```

```
## Warning in simpleLoess(y, x, w, span, degree = degree, parametric = parametric, : all data on boundary of neighborhood. make span bigger
```

```
## Warning in simpleLoess(y, x, w, span, degree = degree, parametric = parametric, : pseudoinverse used at -0.005
```

```
## Warning in simpleLoess(y, x, w, span, degree = degree, parametric = parametric, : neighborhood radius 0.005
```

```
## Warning in simpleLoess(y, x, w, span, degree = degree, parametric = parametric, : reciprocal condition number 1
```

```
## Warning in simpleLoess(y, x, w, span, degree = degree, parametric = parametric, : There are other near singularities as well. 1.01
```

```
## Warning in simpleLoess(y, x, w, span, degree = degree, parametric = parametric, : zero-width neighborhood. make span bigger
```

```
## Warning: Computation failed in `stat_smooth()`:
## NA/NaN/Inf in foreign function call (arg 5)
```

```
## Warning in simpleLoess(y, x, w, span, degree = degree, parametric = parametric, : at -0.005
```

```
## Warning in simpleLoess(y, x, w, span, degree = degree, parametric = parametric, : radius 2.5e-05
```

```
## Warning in simpleLoess(y, x, w, span, degree = degree, parametric = parametric, : all data on boundary of neighborhood. make span bigger
```

```
## Warning in simpleLoess(y, x, w, span, degree = degree, parametric = parametric, : pseudoinverse used at -0.005
```

```
## Warning in simpleLoess(y, x, w, span, degree = degree, parametric = parametric, : neighborhood radius 0.005
```

```
## Warning in simpleLoess(y, x, w, span, degree = degree, parametric = parametric, : reciprocal condition number 1
```

```
## Warning in simpleLoess(y, x, w, span, degree = degree, parametric = parametric, : There are other near singularities as well. 1.01
```

```
## Warning in simpleLoess(y, x, w, span, degree = degree, parametric = parametric, : zero-width neighborhood. make span bigger
```

```
## Warning: Computation failed in `stat_smooth()`:
## NA/NaN/Inf in foreign function call (arg 5)
```

```
## Warning in simpleLoess(y, x, w, span, degree = degree, parametric = parametric, : at -0.005
```

```
## Warning in simpleLoess(y, x, w, span, degree = degree, parametric = parametric, : radius 2.5e-05
```

```
## Warning in simpleLoess(y, x, w, span, degree = degree, parametric = parametric, : all data on boundary of neighborhood. make span bigger
```

```
## Warning in simpleLoess(y, x, w, span, degree = degree, parametric = parametric, : pseudoinverse used at -0.005
```

```
## Warning in simpleLoess(y, x, w, span, degree = degree, parametric = parametric, : neighborhood radius 0.005
```

```
## Warning in simpleLoess(y, x, w, span, degree = degree, parametric = parametric, : reciprocal condition number 1
```

```
## Warning in simpleLoess(y, x, w, span, degree = degree, parametric = parametric, : There are other near singularities as well. 1.01
```

```
## Warning in simpleLoess(y, x, w, span, degree = degree, parametric = parametric, : zero-width neighborhood. make span bigger
```

```
## Warning: Computation failed in `stat_smooth()`:
## NA/NaN/Inf in foreign function call (arg 5)
```

```
## Warning in simpleLoess(y, x, w, span, degree = degree, parametric = parametric, : at -0.005
```

```
## Warning in simpleLoess(y, x, w, span, degree = degree, parametric = parametric, : radius 2.5e-05
```

```
## Warning in simpleLoess(y, x, w, span, degree = degree, parametric = parametric, : all data on boundary of neighborhood. make span bigger
```

```
## Warning in simpleLoess(y, x, w, span, degree = degree, parametric = parametric, : pseudoinverse used at -0.005
```

```
## Warning in simpleLoess(y, x, w, span, degree = degree, parametric = parametric, : neighborhood radius 0.005
```

```
## Warning in simpleLoess(y, x, w, span, degree = degree, parametric = parametric, : reciprocal condition number 1
```

```
## Warning in simpleLoess(y, x, w, span, degree = degree, parametric = parametric, : There are other near singularities as well. 1.01
```

```
## Warning in simpleLoess(y, x, w, span, degree = degree, parametric = parametric, : zero-width neighborhood. make span bigger
```

```
## Warning: Computation failed in `stat_smooth()`:
## NA/NaN/Inf in foreign function call (arg 5)
```

```
## Warning in simpleLoess(y, x, w, span, degree = degree, parametric = parametric, : pseudoinverse used at -0.005
```

```
## Warning in simpleLoess(y, x, w, span, degree = degree, parametric = parametric, : neighborhood radius 1.005
```

```
## Warning in simpleLoess(y, x, w, span, degree = degree, parametric = parametric, : reciprocal condition number 0
```

```
## Warning in simpleLoess(y, x, w, span, degree = degree, parametric = parametric, : There are other near singularities as well. 1.01
```

```
## Warning in predLoess(object$y, object$x, newx = if (is.null(newdata)) object$x else if (is.data.frame(newdata)) as.matrix(model.frame(delete.response(terms(object)), : pseudoinverse used at -0.005
```

```
## Warning in predLoess(object$y, object$x, newx = if (is.null(newdata)) object$x else if (is.data.frame(newdata)) as.matrix(model.frame(delete.response(terms(object)), : neighborhood radius 1.005
```

```
## Warning in predLoess(object$y, object$x, newx = if (is.null(newdata)) object$x else if (is.data.frame(newdata)) as.matrix(model.frame(delete.response(terms(object)), : reciprocal condition number 0
```

```
## Warning in predLoess(object$y, object$x, newx = if (is.null(newdata)) object$x else if (is.data.frame(newdata)) as.matrix(model.frame(delete.response(terms(object)), : There are other near
## singularities as well. 1.01
```

```
## Warning in simpleLoess(y, x, w, span, degree = degree, parametric = parametric, : pseudoinverse used at -0.005
```

```
## Warning in simpleLoess(y, x, w, span, degree = degree, parametric = parametric, : neighborhood radius 1.005
```

```
## Warning in simpleLoess(y, x, w, span, degree = degree, parametric = parametric, : reciprocal condition number 0
```

```
## Warning in simpleLoess(y, x, w, span, degree = degree, parametric = parametric, : There are other near singularities as well. 1.01
```

```
## Warning in predLoess(object$y, object$x, newx = if (is.null(newdata)) object$x else if (is.data.frame(newdata)) as.matrix(model.frame(delete.response(terms(object)), : pseudoinverse used at -0.005
```

```
## Warning in predLoess(object$y, object$x, newx = if (is.null(newdata)) object$x else if (is.data.frame(newdata)) as.matrix(model.frame(delete.response(terms(object)), : neighborhood radius 1.005
```

```
## Warning in predLoess(object$y, object$x, newx = if (is.null(newdata)) object$x else if (is.data.frame(newdata)) as.matrix(model.frame(delete.response(terms(object)), : reciprocal condition number 0
```

```
## Warning in predLoess(object$y, object$x, newx = if (is.null(newdata)) object$x else if (is.data.frame(newdata)) as.matrix(model.frame(delete.response(terms(object)), : There are other near
## singularities as well. 1.01
```

```
## Warning in simpleLoess(y, x, w, span, degree = degree, parametric = parametric, : at -0.005
```

```
## Warning in simpleLoess(y, x, w, span, degree = degree, parametric = parametric, : radius 2.5e-05
```

```
## Warning in simpleLoess(y, x, w, span, degree = degree, parametric = parametric, : all data on boundary of neighborhood. make span bigger
```

```
## Warning in simpleLoess(y, x, w, span, degree = degree, parametric = parametric, : pseudoinverse used at -0.005
```

```
## Warning in simpleLoess(y, x, w, span, degree = degree, parametric = parametric, : neighborhood radius 0.005
```

```
## Warning in simpleLoess(y, x, w, span, degree = degree, parametric = parametric, : reciprocal condition number 1
```

```
## Warning in simpleLoess(y, x, w, span, degree = degree, parametric = parametric, : There are other near singularities as well. 1.01
```

```
## Warning in simpleLoess(y, x, w, span, degree = degree, parametric = parametric, : zero-width neighborhood. make span bigger
```

```
## Warning: Computation failed in `stat_smooth()`:
## NA/NaN/Inf in foreign function call (arg 5)
```

```
## Warning in simpleLoess(y, x, w, span, degree = degree, parametric = parametric, : at -0.005
```

```
## Warning in simpleLoess(y, x, w, span, degree = degree, parametric = parametric, : radius 2.5e-05
```

```
## Warning in simpleLoess(y, x, w, span, degree = degree, parametric = parametric, : all data on boundary of neighborhood. make span bigger
```

```
## Warning in simpleLoess(y, x, w, span, degree = degree, parametric = parametric, : pseudoinverse used at -0.005
```

```
## Warning in simpleLoess(y, x, w, span, degree = degree, parametric = parametric, : neighborhood radius 0.005
```

```
## Warning in simpleLoess(y, x, w, span, degree = degree, parametric = parametric, : reciprocal condition number 1
```

```
## Warning in simpleLoess(y, x, w, span, degree = degree, parametric = parametric, : There are other near singularities as well. 1.01
```

```
## Warning in simpleLoess(y, x, w, span, degree = degree, parametric = parametric, : zero-width neighborhood. make span bigger
```

```
## Warning: Computation failed in `stat_smooth()`:
## NA/NaN/Inf in foreign function call (arg 5)
```

```
## Warning in simpleLoess(y, x, w, span, degree = degree, parametric = parametric, : at -0.005
```

```
## Warning in simpleLoess(y, x, w, span, degree = degree, parametric = parametric, : radius 2.5e-05
```

```
## Warning in simpleLoess(y, x, w, span, degree = degree, parametric = parametric, : all data on boundary of neighborhood. make span bigger
```

```
## Warning in simpleLoess(y, x, w, span, degree = degree, parametric = parametric, : pseudoinverse used at -0.005
```

```
## Warning in simpleLoess(y, x, w, span, degree = degree, parametric = parametric, : neighborhood radius 0.005
```

```
## Warning in simpleLoess(y, x, w, span, degree = degree, parametric = parametric, : reciprocal condition number 1
```

```
## Warning in simpleLoess(y, x, w, span, degree = degree, parametric = parametric, : There are other near singularities as well. 1.01
```

```
## Warning in simpleLoess(y, x, w, span, degree = degree, parametric = parametric, : zero-width neighborhood. make span bigger
```

```
## Warning: Computation failed in `stat_smooth()`:
## NA/NaN/Inf in foreign function call (arg 5)
```

```
## Warning in simpleLoess(y, x, w, span, degree = degree, parametric = parametric, : pseudoinverse used at -0.005
```

```
## Warning in simpleLoess(y, x, w, span, degree = degree, parametric = parametric, : neighborhood radius 1.005
```

```
## Warning in simpleLoess(y, x, w, span, degree = degree, parametric = parametric, : reciprocal condition number 0
```

```
## Warning in simpleLoess(y, x, w, span, degree = degree, parametric = parametric, : There are other near singularities as well. 1.01
```

```
## Warning in predLoess(object$y, object$x, newx = if (is.null(newdata)) object$x else if (is.data.frame(newdata)) as.matrix(model.frame(delete.response(terms(object)), : pseudoinverse used at -0.005
```

```
## Warning in predLoess(object$y, object$x, newx = if (is.null(newdata)) object$x else if (is.data.frame(newdata)) as.matrix(model.frame(delete.response(terms(object)), : neighborhood radius 1.005
```

```
## Warning in predLoess(object$y, object$x, newx = if (is.null(newdata)) object$x else if (is.data.frame(newdata)) as.matrix(model.frame(delete.response(terms(object)), : reciprocal condition number 0
```

```
## Warning in predLoess(object$y, object$x, newx = if (is.null(newdata)) object$x else if (is.data.frame(newdata)) as.matrix(model.frame(delete.response(terms(object)), : There are other near
## singularities as well. 1.01
```

```
## Warning in simpleLoess(y, x, w, span, degree = degree, parametric = parametric, : at -0.005
```

```
## Warning in simpleLoess(y, x, w, span, degree = degree, parametric = parametric, : radius 2.5e-05
```

```
## Warning in simpleLoess(y, x, w, span, degree = degree, parametric = parametric, : all data on boundary of neighborhood. make span bigger
```

```
## Warning in simpleLoess(y, x, w, span, degree = degree, parametric = parametric, : pseudoinverse used at -0.005
```

```
## Warning in simpleLoess(y, x, w, span, degree = degree, parametric = parametric, : neighborhood radius 0.005
```

```
## Warning in simpleLoess(y, x, w, span, degree = degree, parametric = parametric, : reciprocal condition number 1
```

```
## Warning in simpleLoess(y, x, w, span, degree = degree, parametric = parametric, : There are other near singularities as well. 1.01
```

```
## Warning in simpleLoess(y, x, w, span, degree = degree, parametric = parametric, : zero-width neighborhood. make span bigger
```

```
## Warning: Computation failed in `stat_smooth()`:
## NA/NaN/Inf in foreign function call (arg 5)
```

```
## Warning in simpleLoess(y, x, w, span, degree = degree, parametric = parametric, : at -0.005
```

```
## Warning in simpleLoess(y, x, w, span, degree = degree, parametric = parametric, : radius 2.5e-05
```

```
## Warning in simpleLoess(y, x, w, span, degree = degree, parametric = parametric, : all data on boundary of neighborhood. make span bigger
```

```
## Warning in simpleLoess(y, x, w, span, degree = degree, parametric = parametric, : pseudoinverse used at -0.005
```

```
## Warning in simpleLoess(y, x, w, span, degree = degree, parametric = parametric, : neighborhood radius 0.005
```

```
## Warning in simpleLoess(y, x, w, span, degree = degree, parametric = parametric, : reciprocal condition number 1
```

```
## Warning in simpleLoess(y, x, w, span, degree = degree, parametric = parametric, : There are other near singularities as well. 1.01
```

```
## Warning in simpleLoess(y, x, w, span, degree = degree, parametric = parametric, : zero-width neighborhood. make span bigger
```

```
## Warning: Computation failed in `stat_smooth()`:
## NA/NaN/Inf in foreign function call (arg 5)
```

```
## Warning in simpleLoess(y, x, w, span, degree = degree, parametric = parametric, : at -0.005
```

```
## Warning in simpleLoess(y, x, w, span, degree = degree, parametric = parametric, : radius 2.5e-05
```

```
## Warning in simpleLoess(y, x, w, span, degree = degree, parametric = parametric, : all data on boundary of neighborhood. make span bigger
```

```
## Warning in simpleLoess(y, x, w, span, degree = degree, parametric = parametric, : pseudoinverse used at -0.005
```

```
## Warning in simpleLoess(y, x, w, span, degree = degree, parametric = parametric, : neighborhood radius 0.005
```

```
## Warning in simpleLoess(y, x, w, span, degree = degree, parametric = parametric, : reciprocal condition number 1
```

```
## Warning in simpleLoess(y, x, w, span, degree = degree, parametric = parametric, : There are other near singularities as well. 1.01
```

```
## Warning in simpleLoess(y, x, w, span, degree = degree, parametric = parametric, : zero-width neighborhood. make span bigger
```

```
## Warning: Computation failed in `stat_smooth()`:
## NA/NaN/Inf in foreign function call (arg 5)
```

```
## Warning in simpleLoess(y, x, w, span, degree = degree, parametric = parametric, : at -0.005
```

```
## Warning in simpleLoess(y, x, w, span, degree = degree, parametric = parametric, : radius 2.5e-05
```

```
## Warning in simpleLoess(y, x, w, span, degree = degree, parametric = parametric, : all data on boundary of neighborhood. make span bigger
```

```
## Warning in simpleLoess(y, x, w, span, degree = degree, parametric = parametric, : pseudoinverse used at -0.005
```

```
## Warning in simpleLoess(y, x, w, span, degree = degree, parametric = parametric, : neighborhood radius 0.005
```

```
## Warning in simpleLoess(y, x, w, span, degree = degree, parametric = parametric, : reciprocal condition number 1
```

```
## Warning in simpleLoess(y, x, w, span, degree = degree, parametric = parametric, : There are other near singularities as well. 1.01
```

```
## Warning in simpleLoess(y, x, w, span, degree = degree, parametric = parametric, : zero-width neighborhood. make span bigger
```

```
## Warning: Computation failed in `stat_smooth()`:
## NA/NaN/Inf in foreign function call (arg 5)
```

```
## Warning in simpleLoess(y, x, w, span, degree = degree, parametric = parametric, : at -0.005
```

```
## Warning in simpleLoess(y, x, w, span, degree = degree, parametric = parametric, : radius 2.5e-05
```

```
## Warning in simpleLoess(y, x, w, span, degree = degree, parametric = parametric, : all data on boundary of neighborhood. make span bigger
```

```
## Warning in simpleLoess(y, x, w, span, degree = degree, parametric = parametric, : pseudoinverse used at -0.005
```

```
## Warning in simpleLoess(y, x, w, span, degree = degree, parametric = parametric, : neighborhood radius 0.005
```

```
## Warning in simpleLoess(y, x, w, span, degree = degree, parametric = parametric, : reciprocal condition number 1
```

```
## Warning in simpleLoess(y, x, w, span, degree = degree, parametric = parametric, : There are other near singularities as well. 1.01
```

```
## Warning in simpleLoess(y, x, w, span, degree = degree, parametric = parametric, : zero-width neighborhood. make span bigger
```

```
## Warning: Computation failed in `stat_smooth()`:
## NA/NaN/Inf in foreign function call (arg 5)
```

```
## Warning in simpleLoess(y, x, w, span, degree = degree, parametric = parametric, : at -0.005
```

```
## Warning in simpleLoess(y, x, w, span, degree = degree, parametric = parametric, : radius 2.5e-05
```

```
## Warning in simpleLoess(y, x, w, span, degree = degree, parametric = parametric, : all data on boundary of neighborhood. make span bigger
```

```
## Warning in simpleLoess(y, x, w, span, degree = degree, parametric = parametric, : pseudoinverse used at -0.005
```

```
## Warning in simpleLoess(y, x, w, span, degree = degree, parametric = parametric, : neighborhood radius 0.005
```

```
## Warning in simpleLoess(y, x, w, span, degree = degree, parametric = parametric, : reciprocal condition number 1
```

```
## Warning in simpleLoess(y, x, w, span, degree = degree, parametric = parametric, : There are other near singularities as well. 1.01
```

```
## Warning in simpleLoess(y, x, w, span, degree = degree, parametric = parametric, : zero-width neighborhood. make span bigger
```

```
## Warning: Computation failed in `stat_smooth()`:
## NA/NaN/Inf in foreign function call (arg 5)
```

```
## Warning in simpleLoess(y, x, w, span, degree = degree, parametric = parametric, : at -0.005
```

```
## Warning in simpleLoess(y, x, w, span, degree = degree, parametric = parametric, : radius 2.5e-05
```

```
## Warning in simpleLoess(y, x, w, span, degree = degree, parametric = parametric, : all data on boundary of neighborhood. make span bigger
```

```
## Warning in simpleLoess(y, x, w, span, degree = degree, parametric = parametric, : pseudoinverse used at -0.005
```

```
## Warning in simpleLoess(y, x, w, span, degree = degree, parametric = parametric, : neighborhood radius 0.005
```

```
## Warning in simpleLoess(y, x, w, span, degree = degree, parametric = parametric, : reciprocal condition number 1
```

```
## Warning in simpleLoess(y, x, w, span, degree = degree, parametric = parametric, : There are other near singularities as well. 1.01
```

```
## Warning in simpleLoess(y, x, w, span, degree = degree, parametric = parametric, : zero-width neighborhood. make span bigger
```

```
## Warning: Computation failed in `stat_smooth()`:
## NA/NaN/Inf in foreign function call (arg 5)
```

```
## Warning in simpleLoess(y, x, w, span, degree = degree, parametric = parametric, : at -0.005
```

```
## Warning in simpleLoess(y, x, w, span, degree = degree, parametric = parametric, : radius 2.5e-05
```

```
## Warning in simpleLoess(y, x, w, span, degree = degree, parametric = parametric, : all data on boundary of neighborhood. make span bigger
```

```
## Warning in simpleLoess(y, x, w, span, degree = degree, parametric = parametric, : pseudoinverse used at -0.005
```

```
## Warning in simpleLoess(y, x, w, span, degree = degree, parametric = parametric, : neighborhood radius 0.005
```

```
## Warning in simpleLoess(y, x, w, span, degree = degree, parametric = parametric, : reciprocal condition number 1
```

```
## Warning in simpleLoess(y, x, w, span, degree = degree, parametric = parametric, : There are other near singularities as well. 1.01
```

```
## Warning in simpleLoess(y, x, w, span, degree = degree, parametric = parametric, : zero-width neighborhood. make span bigger
```

```
## Warning: Computation failed in `stat_smooth()`:
## NA/NaN/Inf in foreign function call (arg 5)
```

```
## Warning in simpleLoess(y, x, w, span, degree = degree, parametric = parametric, : at -0.005
```

```
## Warning in simpleLoess(y, x, w, span, degree = degree, parametric = parametric, : radius 2.5e-05
```

```
## Warning in simpleLoess(y, x, w, span, degree = degree, parametric = parametric, : all data on boundary of neighborhood. make span bigger
```

```
## Warning in simpleLoess(y, x, w, span, degree = degree, parametric = parametric, : pseudoinverse used at -0.005
```

```
## Warning in simpleLoess(y, x, w, span, degree = degree, parametric = parametric, : neighborhood radius 0.005
```

```
## Warning in simpleLoess(y, x, w, span, degree = degree, parametric = parametric, : reciprocal condition number 1
```

```
## Warning in simpleLoess(y, x, w, span, degree = degree, parametric = parametric, : There are other near singularities as well. 1.01
```

```
## Warning in simpleLoess(y, x, w, span, degree = degree, parametric = parametric, : zero-width neighborhood. make span bigger
```

```
## Warning: Computation failed in `stat_smooth()`:
## NA/NaN/Inf in foreign function call (arg 5)
```

```
## Warning in simpleLoess(y, x, w, span, degree = degree, parametric = parametric, : at -0.005
```

```
## Warning in simpleLoess(y, x, w, span, degree = degree, parametric = parametric, : radius 2.5e-05
```

```
## Warning in simpleLoess(y, x, w, span, degree = degree, parametric = parametric, : all data on boundary of neighborhood. make span bigger
```

```
## Warning in simpleLoess(y, x, w, span, degree = degree, parametric = parametric, : pseudoinverse used at -0.005
```

```
## Warning in simpleLoess(y, x, w, span, degree = degree, parametric = parametric, : neighborhood radius 0.005
```

```
## Warning in simpleLoess(y, x, w, span, degree = degree, parametric = parametric, : reciprocal condition number 1
```

```
## Warning in simpleLoess(y, x, w, span, degree = degree, parametric = parametric, : There are other near singularities as well. 1.01
```

```
## Warning in simpleLoess(y, x, w, span, degree = degree, parametric = parametric, : zero-width neighborhood. make span bigger
```

```
## Warning: Computation failed in `stat_smooth()`:
## NA/NaN/Inf in foreign function call (arg 5)
```

```
## Warning in simpleLoess(y, x, w, span, degree = degree, parametric = parametric, : at -0.005
```

```
## Warning in simpleLoess(y, x, w, span, degree = degree, parametric = parametric, : radius 2.5e-05
```

```
## Warning in simpleLoess(y, x, w, span, degree = degree, parametric = parametric, : all data on boundary of neighborhood. make span bigger
```

```
## Warning in simpleLoess(y, x, w, span, degree = degree, parametric = parametric, : pseudoinverse used at -0.005
```

```
## Warning in simpleLoess(y, x, w, span, degree = degree, parametric = parametric, : neighborhood radius 0.005
```

```
## Warning in simpleLoess(y, x, w, span, degree = degree, parametric = parametric, : reciprocal condition number 1
```

```
## Warning in simpleLoess(y, x, w, span, degree = degree, parametric = parametric, : There are other near singularities as well. 1.01
```

```
## Warning in simpleLoess(y, x, w, span, degree = degree, parametric = parametric, : zero-width neighborhood. make span bigger
```

```
## Warning: Computation failed in `stat_smooth()`:
## NA/NaN/Inf in foreign function call (arg 5)
```

```
## Warning in simpleLoess(y, x, w, span, degree = degree, parametric = parametric, : at -0.005
```

```
## Warning in simpleLoess(y, x, w, span, degree = degree, parametric = parametric, : radius 2.5e-05
```

```
## Warning in simpleLoess(y, x, w, span, degree = degree, parametric = parametric, : all data on boundary of neighborhood. make span bigger
```

```
## Warning in simpleLoess(y, x, w, span, degree = degree, parametric = parametric, : pseudoinverse used at -0.005
```

```
## Warning in simpleLoess(y, x, w, span, degree = degree, parametric = parametric, : neighborhood radius 0.005
```

```
## Warning in simpleLoess(y, x, w, span, degree = degree, parametric = parametric, : reciprocal condition number 1
```

```
## Warning in simpleLoess(y, x, w, span, degree = degree, parametric = parametric, : There are other near singularities as well. 1.01
```

```
## Warning in simpleLoess(y, x, w, span, degree = degree, parametric = parametric, : zero-width neighborhood. make span bigger
```

```
## Warning: Computation failed in `stat_smooth()`:
## NA/NaN/Inf in foreign function call (arg 5)
```

```
## Warning in simpleLoess(y, x, w, span, degree = degree, parametric = parametric, : at -0.005
```

```
## Warning in simpleLoess(y, x, w, span, degree = degree, parametric = parametric, : radius 2.5e-05
```

```
## Warning in simpleLoess(y, x, w, span, degree = degree, parametric = parametric, : all data on boundary of neighborhood. make span bigger
```

```
## Warning in simpleLoess(y, x, w, span, degree = degree, parametric = parametric, : pseudoinverse used at -0.005
```

```
## Warning in simpleLoess(y, x, w, span, degree = degree, parametric = parametric, : neighborhood radius 0.005
```

```
## Warning in simpleLoess(y, x, w, span, degree = degree, parametric = parametric, : reciprocal condition number 1
```

```
## Warning in simpleLoess(y, x, w, span, degree = degree, parametric = parametric, : There are other near singularities as well. 1.01
```

```
## Warning in simpleLoess(y, x, w, span, degree = degree, parametric = parametric, : zero-width neighborhood. make span bigger
```

```
## Warning: Computation failed in `stat_smooth()`:
## NA/NaN/Inf in foreign function call (arg 5)
```

```
## Warning in simpleLoess(y, x, w, span, degree = degree, parametric = parametric, : at -0.005
```

```
## Warning in simpleLoess(y, x, w, span, degree = degree, parametric = parametric, : radius 2.5e-05
```

```
## Warning in simpleLoess(y, x, w, span, degree = degree, parametric = parametric, : all data on boundary of neighborhood. make span bigger
```

```
## Warning in simpleLoess(y, x, w, span, degree = degree, parametric = parametric, : pseudoinverse used at -0.005
```

```
## Warning in simpleLoess(y, x, w, span, degree = degree, parametric = parametric, : neighborhood radius 0.005
```

```
## Warning in simpleLoess(y, x, w, span, degree = degree, parametric = parametric, : reciprocal condition number 1
```

```
## Warning in simpleLoess(y, x, w, span, degree = degree, parametric = parametric, : There are other near singularities as well. 1.01
```

```
## Warning in simpleLoess(y, x, w, span, degree = degree, parametric = parametric, : zero-width neighborhood. make span bigger
```

```
## Warning: Computation failed in `stat_smooth()`:
## NA/NaN/Inf in foreign function call (arg 5)
```

```
## Warning in simpleLoess(y, x, w, span, degree = degree, parametric = parametric, : at -0.005
```

```
## Warning in simpleLoess(y, x, w, span, degree = degree, parametric = parametric, : radius 2.5e-05
```

```
## Warning in simpleLoess(y, x, w, span, degree = degree, parametric = parametric, : all data on boundary of neighborhood. make span bigger
```

```
## Warning in simpleLoess(y, x, w, span, degree = degree, parametric = parametric, : pseudoinverse used at -0.005
```

```
## Warning in simpleLoess(y, x, w, span, degree = degree, parametric = parametric, : neighborhood radius 0.005
```

```
## Warning in simpleLoess(y, x, w, span, degree = degree, parametric = parametric, : reciprocal condition number 1
```

```
## Warning in simpleLoess(y, x, w, span, degree = degree, parametric = parametric, : There are other near singularities as well. 1.01
```

```
## Warning in simpleLoess(y, x, w, span, degree = degree, parametric = parametric, : zero-width neighborhood. make span bigger
```

```
## Warning: Computation failed in `stat_smooth()`:
## NA/NaN/Inf in foreign function call (arg 5)
```

```
## Warning in simpleLoess(y, x, w, span, degree = degree, parametric = parametric, : at -0.005
```

```
## Warning in simpleLoess(y, x, w, span, degree = degree, parametric = parametric, : radius 2.5e-05
```

```
## Warning in simpleLoess(y, x, w, span, degree = degree, parametric = parametric, : all data on boundary of neighborhood. make span bigger
```

```
## Warning in simpleLoess(y, x, w, span, degree = degree, parametric = parametric, : pseudoinverse used at -0.005
```

```
## Warning in simpleLoess(y, x, w, span, degree = degree, parametric = parametric, : neighborhood radius 0.005
```

```
## Warning in simpleLoess(y, x, w, span, degree = degree, parametric = parametric, : reciprocal condition number 1
```

```
## Warning in simpleLoess(y, x, w, span, degree = degree, parametric = parametric, : There are other near singularities as well. 1.01
```

```
## Warning in simpleLoess(y, x, w, span, degree = degree, parametric = parametric, : zero-width neighborhood. make span bigger
```

```
## Warning: Computation failed in `stat_smooth()`:
## NA/NaN/Inf in foreign function call (arg 5)
```

```
## Warning in simpleLoess(y, x, w, span, degree = degree, parametric = parametric, : at -0.005
```

```
## Warning in simpleLoess(y, x, w, span, degree = degree, parametric = parametric, : radius 2.5e-05
```

```
## Warning in simpleLoess(y, x, w, span, degree = degree, parametric = parametric, : all data on boundary of neighborhood. make span bigger
```

```
## Warning in simpleLoess(y, x, w, span, degree = degree, parametric = parametric, : pseudoinverse used at -0.005
```

```
## Warning in simpleLoess(y, x, w, span, degree = degree, parametric = parametric, : neighborhood radius 0.005
```

```
## Warning in simpleLoess(y, x, w, span, degree = degree, parametric = parametric, : reciprocal condition number 1
```

```
## Warning in simpleLoess(y, x, w, span, degree = degree, parametric = parametric, : There are other near singularities as well. 1.01
```

```
## Warning in simpleLoess(y, x, w, span, degree = degree, parametric = parametric, : zero-width neighborhood. make span bigger
```

```
## Warning: Computation failed in `stat_smooth()`:
## NA/NaN/Inf in foreign function call (arg 5)
```

```
## Warning in simpleLoess(y, x, w, span, degree = degree, parametric = parametric, : at -0.005
```

```
## Warning in simpleLoess(y, x, w, span, degree = degree, parametric = parametric, : radius 2.5e-05
```

```
## Warning in simpleLoess(y, x, w, span, degree = degree, parametric = parametric, : all data on boundary of neighborhood. make span bigger
```

```
## Warning in simpleLoess(y, x, w, span, degree = degree, parametric = parametric, : pseudoinverse used at -0.005
```

```
## Warning in simpleLoess(y, x, w, span, degree = degree, parametric = parametric, : neighborhood radius 0.005
```

```
## Warning in simpleLoess(y, x, w, span, degree = degree, parametric = parametric, : reciprocal condition number 1
```

```
## Warning in simpleLoess(y, x, w, span, degree = degree, parametric = parametric, : There are other near singularities as well. 1.01
```

```
## Warning in simpleLoess(y, x, w, span, degree = degree, parametric = parametric, : zero-width neighborhood. make span bigger
```

```
## Warning: Computation failed in `stat_smooth()`:
## NA/NaN/Inf in foreign function call (arg 5)
```

```
## Warning in simpleLoess(y, x, w, span, degree = degree, parametric = parametric, : at -0.005
```

```
## Warning in simpleLoess(y, x, w, span, degree = degree, parametric = parametric, : radius 2.5e-05
```

```
## Warning in simpleLoess(y, x, w, span, degree = degree, parametric = parametric, : all data on boundary of neighborhood. make span bigger
```

```
## Warning in simpleLoess(y, x, w, span, degree = degree, parametric = parametric, : pseudoinverse used at -0.005
```

```
## Warning in simpleLoess(y, x, w, span, degree = degree, parametric = parametric, : neighborhood radius 0.005
```

```
## Warning in simpleLoess(y, x, w, span, degree = degree, parametric = parametric, : reciprocal condition number 1
```

```
## Warning in simpleLoess(y, x, w, span, degree = degree, parametric = parametric, : There are other near singularities as well. 1.01
```

```
## Warning in simpleLoess(y, x, w, span, degree = degree, parametric = parametric, : zero-width neighborhood. make span bigger
```

```
## Warning: Computation failed in `stat_smooth()`:
## NA/NaN/Inf in foreign function call (arg 5)
```

```
## Warning in simpleLoess(y, x, w, span, degree = degree, parametric = parametric, : at -0.005
```

```
## Warning in simpleLoess(y, x, w, span, degree = degree, parametric = parametric, : radius 2.5e-05
```

```
## Warning in simpleLoess(y, x, w, span, degree = degree, parametric = parametric, : all data on boundary of neighborhood. make span bigger
```

```
## Warning in simpleLoess(y, x, w, span, degree = degree, parametric = parametric, : pseudoinverse used at -0.005
```

```
## Warning in simpleLoess(y, x, w, span, degree = degree, parametric = parametric, : neighborhood radius 0.005
```

```
## Warning in simpleLoess(y, x, w, span, degree = degree, parametric = parametric, : reciprocal condition number 1
```

```
## Warning in simpleLoess(y, x, w, span, degree = degree, parametric = parametric, : There are other near singularities as well. 1.01
```

```
## Warning in simpleLoess(y, x, w, span, degree = degree, parametric = parametric, : zero-width neighborhood. make span bigger
```

```
## Warning: Computation failed in `stat_smooth()`:
## NA/NaN/Inf in foreign function call (arg 5)
```

```
## Warning in simpleLoess(y, x, w, span, degree = degree, parametric = parametric, : at -0.005
```

```
## Warning in simpleLoess(y, x, w, span, degree = degree, parametric = parametric, : radius 2.5e-05
```

```
## Warning in simpleLoess(y, x, w, span, degree = degree, parametric = parametric, : all data on boundary of neighborhood. make span bigger
```

```
## Warning in simpleLoess(y, x, w, span, degree = degree, parametric = parametric, : pseudoinverse used at -0.005
```

```
## Warning in simpleLoess(y, x, w, span, degree = degree, parametric = parametric, : neighborhood radius 0.005
```

```
## Warning in simpleLoess(y, x, w, span, degree = degree, parametric = parametric, : reciprocal condition number 1
```

```
## Warning in simpleLoess(y, x, w, span, degree = degree, parametric = parametric, : There are other near singularities as well. 1.01
```

```
## Warning in simpleLoess(y, x, w, span, degree = degree, parametric = parametric, : zero-width neighborhood. make span bigger
```

```
## Warning: Computation failed in `stat_smooth()`:
## NA/NaN/Inf in foreign function call (arg 5)
```

```
## Warning in simpleLoess(y, x, w, span, degree = degree, parametric = parametric, : at -0.005
```

```
## Warning in simpleLoess(y, x, w, span, degree = degree, parametric = parametric, : radius 2.5e-05
```

```
## Warning in simpleLoess(y, x, w, span, degree = degree, parametric = parametric, : all data on boundary of neighborhood. make span bigger
```

```
## Warning in simpleLoess(y, x, w, span, degree = degree, parametric = parametric, : pseudoinverse used at -0.005
```

```
## Warning in simpleLoess(y, x, w, span, degree = degree, parametric = parametric, : neighborhood radius 0.005
```

```
## Warning in simpleLoess(y, x, w, span, degree = degree, parametric = parametric, : reciprocal condition number 1
```

```
## Warning in simpleLoess(y, x, w, span, degree = degree, parametric = parametric, : There are other near singularities as well. 1.01
```

```
## Warning in simpleLoess(y, x, w, span, degree = degree, parametric = parametric, : zero-width neighborhood. make span bigger
```

```
## Warning: Computation failed in `stat_smooth()`:
## NA/NaN/Inf in foreign function call (arg 5)
```

```
## Warning in simpleLoess(y, x, w, span, degree = degree, parametric = parametric, : at -0.005
```

```
## Warning in simpleLoess(y, x, w, span, degree = degree, parametric = parametric, : radius 2.5e-05
```

```
## Warning in simpleLoess(y, x, w, span, degree = degree, parametric = parametric, : all data on boundary of neighborhood. make span bigger
```

```
## Warning in simpleLoess(y, x, w, span, degree = degree, parametric = parametric, : pseudoinverse used at -0.005
```

```
## Warning in simpleLoess(y, x, w, span, degree = degree, parametric = parametric, : neighborhood radius 0.005
```

```
## Warning in simpleLoess(y, x, w, span, degree = degree, parametric = parametric, : reciprocal condition number 1
```

```
## Warning in simpleLoess(y, x, w, span, degree = degree, parametric = parametric, : There are other near singularities as well. 1.01
```

```
## Warning in simpleLoess(y, x, w, span, degree = degree, parametric = parametric, : zero-width neighborhood. make span bigger
```

```
## Warning: Computation failed in `stat_smooth()`:
## NA/NaN/Inf in foreign function call (arg 5)
```

```
## Warning in simpleLoess(y, x, w, span, degree = degree, parametric = parametric, : at -0.005
```

```
## Warning in simpleLoess(y, x, w, span, degree = degree, parametric = parametric, : radius 2.5e-05
```

```
## Warning in simpleLoess(y, x, w, span, degree = degree, parametric = parametric, : all data on boundary of neighborhood. make span bigger
```

```
## Warning in simpleLoess(y, x, w, span, degree = degree, parametric = parametric, : pseudoinverse used at -0.005
```

```
## Warning in simpleLoess(y, x, w, span, degree = degree, parametric = parametric, : neighborhood radius 0.005
```

```
## Warning in simpleLoess(y, x, w, span, degree = degree, parametric = parametric, : reciprocal condition number 1
```

```
## Warning in simpleLoess(y, x, w, span, degree = degree, parametric = parametric, : There are other near singularities as well. 1.01
```

```
## Warning in simpleLoess(y, x, w, span, degree = degree, parametric = parametric, : zero-width neighborhood. make span bigger
```

```
## Warning: Computation failed in `stat_smooth()`:
## NA/NaN/Inf in foreign function call (arg 5)
```

```
## Warning in simpleLoess(y, x, w, span, degree = degree, parametric = parametric, : at -0.005
```

```
## Warning in simpleLoess(y, x, w, span, degree = degree, parametric = parametric, : radius 2.5e-05
```

```
## Warning in simpleLoess(y, x, w, span, degree = degree, parametric = parametric, : all data on boundary of neighborhood. make span bigger
```

```
## Warning in simpleLoess(y, x, w, span, degree = degree, parametric = parametric, : pseudoinverse used at -0.005
```

```
## Warning in simpleLoess(y, x, w, span, degree = degree, parametric = parametric, : neighborhood radius 0.005
```

```
## Warning in simpleLoess(y, x, w, span, degree = degree, parametric = parametric, : reciprocal condition number 1
```

```
## Warning in simpleLoess(y, x, w, span, degree = degree, parametric = parametric, : There are other near singularities as well. 1.01
```

```
## Warning in simpleLoess(y, x, w, span, degree = degree, parametric = parametric, : zero-width neighborhood. make span bigger
```

```
## Warning: Computation failed in `stat_smooth()`:
## NA/NaN/Inf in foreign function call (arg 5)
```

```
## Warning in simpleLoess(y, x, w, span, degree = degree, parametric = parametric, : at -0.005
```

```
## Warning in simpleLoess(y, x, w, span, degree = degree, parametric = parametric, : radius 2.5e-05
```

```
## Warning in simpleLoess(y, x, w, span, degree = degree, parametric = parametric, : all data on boundary of neighborhood. make span bigger
```

```
## Warning in simpleLoess(y, x, w, span, degree = degree, parametric = parametric, : pseudoinverse used at -0.005
```

```
## Warning in simpleLoess(y, x, w, span, degree = degree, parametric = parametric, : neighborhood radius 0.005
```

```
## Warning in simpleLoess(y, x, w, span, degree = degree, parametric = parametric, : reciprocal condition number 1
```

```
## Warning in simpleLoess(y, x, w, span, degree = degree, parametric = parametric, : There are other near singularities as well. 1.01
```

```
## Warning in simpleLoess(y, x, w, span, degree = degree, parametric = parametric, : zero-width neighborhood. make span bigger
```

```
## Warning: Computation failed in `stat_smooth()`:
## NA/NaN/Inf in foreign function call (arg 5)
```

```
## Warning in simpleLoess(y, x, w, span, degree = degree, parametric = parametric, : at -0.005
```

```
## Warning in simpleLoess(y, x, w, span, degree = degree, parametric = parametric, : radius 2.5e-05
```

```
## Warning in simpleLoess(y, x, w, span, degree = degree, parametric = parametric, : all data on boundary of neighborhood. make span bigger
```

```
## Warning in simpleLoess(y, x, w, span, degree = degree, parametric = parametric, : pseudoinverse used at -0.005
```

```
## Warning in simpleLoess(y, x, w, span, degree = degree, parametric = parametric, : neighborhood radius 0.005
```

```
## Warning in simpleLoess(y, x, w, span, degree = degree, parametric = parametric, : reciprocal condition number 1
```

```
## Warning in simpleLoess(y, x, w, span, degree = degree, parametric = parametric, : There are other near singularities as well. 1.01
```

```
## Warning in simpleLoess(y, x, w, span, degree = degree, parametric = parametric, : zero-width neighborhood. make span bigger
```

```
## Warning: Computation failed in `stat_smooth()`:
## NA/NaN/Inf in foreign function call (arg 5)
```

```
## Warning in simpleLoess(y, x, w, span, degree = degree, parametric = parametric, : at -0.005
```

```
## Warning in simpleLoess(y, x, w, span, degree = degree, parametric = parametric, : radius 2.5e-05
```

```
## Warning in simpleLoess(y, x, w, span, degree = degree, parametric = parametric, : all data on boundary of neighborhood. make span bigger
```

```
## Warning in simpleLoess(y, x, w, span, degree = degree, parametric = parametric, : pseudoinverse used at -0.005
```

```
## Warning in simpleLoess(y, x, w, span, degree = degree, parametric = parametric, : neighborhood radius 0.005
```

```
## Warning in simpleLoess(y, x, w, span, degree = degree, parametric = parametric, : reciprocal condition number 1
```

```
## Warning in simpleLoess(y, x, w, span, degree = degree, parametric = parametric, : There are other near singularities as well. 1.01
```

```
## Warning in simpleLoess(y, x, w, span, degree = degree, parametric = parametric, : zero-width neighborhood. make span bigger
```

```
## Warning: Computation failed in `stat_smooth()`:
## NA/NaN/Inf in foreign function call (arg 5)
```

```
## Warning in simpleLoess(y, x, w, span, degree = degree, parametric = parametric, : at -0.005
```

```
## Warning in simpleLoess(y, x, w, span, degree = degree, parametric = parametric, : radius 2.5e-05
```

```
## Warning in simpleLoess(y, x, w, span, degree = degree, parametric = parametric, : all data on boundary of neighborhood. make span bigger
```

```
## Warning in simpleLoess(y, x, w, span, degree = degree, parametric = parametric, : pseudoinverse used at -0.005
```

```
## Warning in simpleLoess(y, x, w, span, degree = degree, parametric = parametric, : neighborhood radius 0.005
```

```
## Warning in simpleLoess(y, x, w, span, degree = degree, parametric = parametric, : reciprocal condition number 1
```

```
## Warning in simpleLoess(y, x, w, span, degree = degree, parametric = parametric, : There are other near singularities as well. 1.01
```

```
## Warning in simpleLoess(y, x, w, span, degree = degree, parametric = parametric, : zero-width neighborhood. make span bigger
```

```
## Warning: Computation failed in `stat_smooth()`:
## NA/NaN/Inf in foreign function call (arg 5)
```

```
## Warning in simpleLoess(y, x, w, span, degree = degree, parametric = parametric, : at -0.005
```

```
## Warning in simpleLoess(y, x, w, span, degree = degree, parametric = parametric, : radius 2.5e-05
```

```
## Warning in simpleLoess(y, x, w, span, degree = degree, parametric = parametric, : all data on boundary of neighborhood. make span bigger
```

```
## Warning in simpleLoess(y, x, w, span, degree = degree, parametric = parametric, : pseudoinverse used at -0.005
```

```
## Warning in simpleLoess(y, x, w, span, degree = degree, parametric = parametric, : neighborhood radius 0.005
```

```
## Warning in simpleLoess(y, x, w, span, degree = degree, parametric = parametric, : reciprocal condition number 1
```

```
## Warning in simpleLoess(y, x, w, span, degree = degree, parametric = parametric, : There are other near singularities as well. 1.01
```

```
## Warning in simpleLoess(y, x, w, span, degree = degree, parametric = parametric, : zero-width neighborhood. make span bigger
```

```
## Warning: Computation failed in `stat_smooth()`:
## NA/NaN/Inf in foreign function call (arg 5)
```

```
## Warning in simpleLoess(y, x, w, span, degree = degree, parametric = parametric, : pseudoinverse used at -0.005
```

```
## Warning in simpleLoess(y, x, w, span, degree = degree, parametric = parametric, : neighborhood radius 1.005
```

```
## Warning in simpleLoess(y, x, w, span, degree = degree, parametric = parametric, : reciprocal condition number 0
```

```
## Warning in simpleLoess(y, x, w, span, degree = degree, parametric = parametric, : There are other near singularities as well. 1.01
```

```
## Warning in predLoess(object$y, object$x, newx = if (is.null(newdata)) object$x else if (is.data.frame(newdata)) as.matrix(model.frame(delete.response(terms(object)), : pseudoinverse used at -0.005
```

```
## Warning in predLoess(object$y, object$x, newx = if (is.null(newdata)) object$x else if (is.data.frame(newdata)) as.matrix(model.frame(delete.response(terms(object)), : neighborhood radius 1.005
```

```
## Warning in predLoess(object$y, object$x, newx = if (is.null(newdata)) object$x else if (is.data.frame(newdata)) as.matrix(model.frame(delete.response(terms(object)), : reciprocal condition number 0
```

```
## Warning in predLoess(object$y, object$x, newx = if (is.null(newdata)) object$x else if (is.data.frame(newdata)) as.matrix(model.frame(delete.response(terms(object)), : There are other near
## singularities as well. 1.01
```

```
## Warning in simpleLoess(y, x, w, span, degree = degree, parametric = parametric, : at -0.005
```

```
## Warning in simpleLoess(y, x, w, span, degree = degree, parametric = parametric, : radius 2.5e-05
```

```
## Warning in simpleLoess(y, x, w, span, degree = degree, parametric = parametric, : all data on boundary of neighborhood. make span bigger
```

```
## Warning in simpleLoess(y, x, w, span, degree = degree, parametric = parametric, : pseudoinverse used at -0.005
```

```
## Warning in simpleLoess(y, x, w, span, degree = degree, parametric = parametric, : neighborhood radius 0.005
```

```
## Warning in simpleLoess(y, x, w, span, degree = degree, parametric = parametric, : reciprocal condition number 1
```

```
## Warning in simpleLoess(y, x, w, span, degree = degree, parametric = parametric, : There are other near singularities as well. 1.01
```

```
## Warning in simpleLoess(y, x, w, span, degree = degree, parametric = parametric, : zero-width neighborhood. make span bigger
```

```
## Warning: Computation failed in `stat_smooth()`:
## NA/NaN/Inf in foreign function call (arg 5)
```

```
## Warning in simpleLoess(y, x, w, span, degree = degree, parametric = parametric, : at -0.005
```

```
## Warning in simpleLoess(y, x, w, span, degree = degree, parametric = parametric, : radius 2.5e-05
```

```
## Warning in simpleLoess(y, x, w, span, degree = degree, parametric = parametric, : all data on boundary of neighborhood. make span bigger
```

```
## Warning in simpleLoess(y, x, w, span, degree = degree, parametric = parametric, : pseudoinverse used at -0.005
```

```
## Warning in simpleLoess(y, x, w, span, degree = degree, parametric = parametric, : neighborhood radius 0.005
```

```
## Warning in simpleLoess(y, x, w, span, degree = degree, parametric = parametric, : reciprocal condition number 1
```

```
## Warning in simpleLoess(y, x, w, span, degree = degree, parametric = parametric, : There are other near singularities as well. 1.01
```

```
## Warning in simpleLoess(y, x, w, span, degree = degree, parametric = parametric, : zero-width neighborhood. make span bigger
```

```
## Warning: Computation failed in `stat_smooth()`:
## NA/NaN/Inf in foreign function call (arg 5)
```

```
## Warning in simpleLoess(y, x, w, span, degree = degree, parametric = parametric, : at -0.005
```

```
## Warning in simpleLoess(y, x, w, span, degree = degree, parametric = parametric, : radius 2.5e-05
```

```
## Warning in simpleLoess(y, x, w, span, degree = degree, parametric = parametric, : all data on boundary of neighborhood. make span bigger
```

```
## Warning in simpleLoess(y, x, w, span, degree = degree, parametric = parametric, : pseudoinverse used at -0.005
```

```
## Warning in simpleLoess(y, x, w, span, degree = degree, parametric = parametric, : neighborhood radius 0.005
```

```
## Warning in simpleLoess(y, x, w, span, degree = degree, parametric = parametric, : reciprocal condition number 1
```

```
## Warning in simpleLoess(y, x, w, span, degree = degree, parametric = parametric, : There are other near singularities as well. 1.01
```

```
## Warning in simpleLoess(y, x, w, span, degree = degree, parametric = parametric, : zero-width neighborhood. make span bigger
```

```
## Warning: Computation failed in `stat_smooth()`:
## NA/NaN/Inf in foreign function call (arg 5)
```

```
## Warning in simpleLoess(y, x, w, span, degree = degree, parametric = parametric, : at -0.005
```

```
## Warning in simpleLoess(y, x, w, span, degree = degree, parametric = parametric, : radius 2.5e-05
```

```
## Warning in simpleLoess(y, x, w, span, degree = degree, parametric = parametric, : all data on boundary of neighborhood. make span bigger
```

```
## Warning in simpleLoess(y, x, w, span, degree = degree, parametric = parametric, : pseudoinverse used at -0.005
```

```
## Warning in simpleLoess(y, x, w, span, degree = degree, parametric = parametric, : neighborhood radius 0.005
```

```
## Warning in simpleLoess(y, x, w, span, degree = degree, parametric = parametric, : reciprocal condition number 1
```

```
## Warning in simpleLoess(y, x, w, span, degree = degree, parametric = parametric, : There are other near singularities as well. 1.01
```

```
## Warning in simpleLoess(y, x, w, span, degree = degree, parametric = parametric, : zero-width neighborhood. make span bigger
```

```
## Warning: Computation failed in `stat_smooth()`:
## NA/NaN/Inf in foreign function call (arg 5)
```

```
## Warning in simpleLoess(y, x, w, span, degree = degree, parametric = parametric, : at -0.005
```

```
## Warning in simpleLoess(y, x, w, span, degree = degree, parametric = parametric, : radius 2.5e-05
```

```
## Warning in simpleLoess(y, x, w, span, degree = degree, parametric = parametric, : all data on boundary of neighborhood. make span bigger
```

```
## Warning in simpleLoess(y, x, w, span, degree = degree, parametric = parametric, : pseudoinverse used at -0.005
```

```
## Warning in simpleLoess(y, x, w, span, degree = degree, parametric = parametric, : neighborhood radius 0.005
```

```
## Warning in simpleLoess(y, x, w, span, degree = degree, parametric = parametric, : reciprocal condition number 1
```

```
## Warning in simpleLoess(y, x, w, span, degree = degree, parametric = parametric, : There are other near singularities as well. 1.01
```

```
## Warning in simpleLoess(y, x, w, span, degree = degree, parametric = parametric, : zero-width neighborhood. make span bigger
```

```
## Warning: Computation failed in `stat_smooth()`:
## NA/NaN/Inf in foreign function call (arg 5)
```

```
## Warning in simpleLoess(y, x, w, span, degree = degree, parametric = parametric, : at -0.005
```

```
## Warning in simpleLoess(y, x, w, span, degree = degree, parametric = parametric, : radius 2.5e-05
```

```
## Warning in simpleLoess(y, x, w, span, degree = degree, parametric = parametric, : all data on boundary of neighborhood. make span bigger
```

```
## Warning in simpleLoess(y, x, w, span, degree = degree, parametric = parametric, : pseudoinverse used at -0.005
```

```
## Warning in simpleLoess(y, x, w, span, degree = degree, parametric = parametric, : neighborhood radius 0.005
```

```
## Warning in simpleLoess(y, x, w, span, degree = degree, parametric = parametric, : reciprocal condition number 1
```

```
## Warning in simpleLoess(y, x, w, span, degree = degree, parametric = parametric, : There are other near singularities as well. 1.01
```

```
## Warning in simpleLoess(y, x, w, span, degree = degree, parametric = parametric, : zero-width neighborhood. make span bigger
```

```
## Warning: Computation failed in `stat_smooth()`:
## NA/NaN/Inf in foreign function call (arg 5)
```

```
## Warning in simpleLoess(y, x, w, span, degree = degree, parametric = parametric, : at -0.005
```

```
## Warning in simpleLoess(y, x, w, span, degree = degree, parametric = parametric, : radius 2.5e-05
```

```
## Warning in simpleLoess(y, x, w, span, degree = degree, parametric = parametric, : all data on boundary of neighborhood. make span bigger
```

```
## Warning in simpleLoess(y, x, w, span, degree = degree, parametric = parametric, : pseudoinverse used at -0.005
```

```
## Warning in simpleLoess(y, x, w, span, degree = degree, parametric = parametric, : neighborhood radius 0.005
```

```
## Warning in simpleLoess(y, x, w, span, degree = degree, parametric = parametric, : reciprocal condition number 1
```

```
## Warning in simpleLoess(y, x, w, span, degree = degree, parametric = parametric, : There are other near singularities as well. 1.01
```

```
## Warning in simpleLoess(y, x, w, span, degree = degree, parametric = parametric, : zero-width neighborhood. make span bigger
```

```
## Warning: Computation failed in `stat_smooth()`:
## NA/NaN/Inf in foreign function call (arg 5)
```

```
## Warning in simpleLoess(y, x, w, span, degree = degree, parametric = parametric, : at -0.005
```

```
## Warning in simpleLoess(y, x, w, span, degree = degree, parametric = parametric, : radius 2.5e-05
```

```
## Warning in simpleLoess(y, x, w, span, degree = degree, parametric = parametric, : all data on boundary of neighborhood. make span bigger
```

```
## Warning in simpleLoess(y, x, w, span, degree = degree, parametric = parametric, : pseudoinverse used at -0.005
```

```
## Warning in simpleLoess(y, x, w, span, degree = degree, parametric = parametric, : neighborhood radius 0.005
```

```
## Warning in simpleLoess(y, x, w, span, degree = degree, parametric = parametric, : reciprocal condition number 1
```

```
## Warning in simpleLoess(y, x, w, span, degree = degree, parametric = parametric, : There are other near singularities as well. 1.01
```

```
## Warning in simpleLoess(y, x, w, span, degree = degree, parametric = parametric, : zero-width neighborhood. make span bigger
```

```
## Warning: Computation failed in `stat_smooth()`:
## NA/NaN/Inf in foreign function call (arg 5)
```

```
## Warning in simpleLoess(y, x, w, span, degree = degree, parametric = parametric, : at -0.005
```

```
## Warning in simpleLoess(y, x, w, span, degree = degree, parametric = parametric, : radius 2.5e-05
```

```
## Warning in simpleLoess(y, x, w, span, degree = degree, parametric = parametric, : all data on boundary of neighborhood. make span bigger
```

```
## Warning in simpleLoess(y, x, w, span, degree = degree, parametric = parametric, : pseudoinverse used at -0.005
```

```
## Warning in simpleLoess(y, x, w, span, degree = degree, parametric = parametric, : neighborhood radius 0.005
```

```
## Warning in simpleLoess(y, x, w, span, degree = degree, parametric = parametric, : reciprocal condition number 1
```

```
## Warning in simpleLoess(y, x, w, span, degree = degree, parametric = parametric, : There are other near singularities as well. 1.01
```

```
## Warning in simpleLoess(y, x, w, span, degree = degree, parametric = parametric, : zero-width neighborhood. make span bigger
```

```
## Warning: Computation failed in `stat_smooth()`:
## NA/NaN/Inf in foreign function call (arg 5)
```

```
## Warning in simpleLoess(y, x, w, span, degree = degree, parametric = parametric, : at -0.005
```

```
## Warning in simpleLoess(y, x, w, span, degree = degree, parametric = parametric, : radius 2.5e-05
```

```
## Warning in simpleLoess(y, x, w, span, degree = degree, parametric = parametric, : all data on boundary of neighborhood. make span bigger
```

```
## Warning in simpleLoess(y, x, w, span, degree = degree, parametric = parametric, : pseudoinverse used at -0.005
```

```
## Warning in simpleLoess(y, x, w, span, degree = degree, parametric = parametric, : neighborhood radius 0.005
```

```
## Warning in simpleLoess(y, x, w, span, degree = degree, parametric = parametric, : reciprocal condition number 1
```

```
## Warning in simpleLoess(y, x, w, span, degree = degree, parametric = parametric, : There are other near singularities as well. 1.01
```

```
## Warning in simpleLoess(y, x, w, span, degree = degree, parametric = parametric, : zero-width neighborhood. make span bigger
```

```
## Warning: Computation failed in `stat_smooth()`:
## NA/NaN/Inf in foreign function call (arg 5)
```

```
## Warning in simpleLoess(y, x, w, span, degree = degree, parametric = parametric, : at -0.005
```

```
## Warning in simpleLoess(y, x, w, span, degree = degree, parametric = parametric, : radius 2.5e-05
```

```
## Warning in simpleLoess(y, x, w, span, degree = degree, parametric = parametric, : all data on boundary of neighborhood. make span bigger
```

```
## Warning in simpleLoess(y, x, w, span, degree = degree, parametric = parametric, : pseudoinverse used at -0.005
```

```
## Warning in simpleLoess(y, x, w, span, degree = degree, parametric = parametric, : neighborhood radius 0.005
```

```
## Warning in simpleLoess(y, x, w, span, degree = degree, parametric = parametric, : reciprocal condition number 1
```

```
## Warning in simpleLoess(y, x, w, span, degree = degree, parametric = parametric, : There are other near singularities as well. 1.01
```

```
## Warning in simpleLoess(y, x, w, span, degree = degree, parametric = parametric, : zero-width neighborhood. make span bigger
```

```
## Warning: Computation failed in `stat_smooth()`:
## NA/NaN/Inf in foreign function call (arg 5)
```

```
## Warning in simpleLoess(y, x, w, span, degree = degree, parametric = parametric, : at -0.005
```

```
## Warning in simpleLoess(y, x, w, span, degree = degree, parametric = parametric, : radius 2.5e-05
```

```
## Warning in simpleLoess(y, x, w, span, degree = degree, parametric = parametric, : all data on boundary of neighborhood. make span bigger
```

```
## Warning in simpleLoess(y, x, w, span, degree = degree, parametric = parametric, : pseudoinverse used at -0.005
```

```
## Warning in simpleLoess(y, x, w, span, degree = degree, parametric = parametric, : neighborhood radius 0.005
```

```
## Warning in simpleLoess(y, x, w, span, degree = degree, parametric = parametric, : reciprocal condition number 1
```

```
## Warning in simpleLoess(y, x, w, span, degree = degree, parametric = parametric, : There are other near singularities as well. 1.01
```

```
## Warning in simpleLoess(y, x, w, span, degree = degree, parametric = parametric, : zero-width neighborhood. make span bigger
```

```
## Warning: Computation failed in `stat_smooth()`:
## NA/NaN/Inf in foreign function call (arg 5)
```

```
## Warning in simpleLoess(y, x, w, span, degree = degree, parametric = parametric, : at -0.005
```

```
## Warning in simpleLoess(y, x, w, span, degree = degree, parametric = parametric, : radius 2.5e-05
```

```
## Warning in simpleLoess(y, x, w, span, degree = degree, parametric = parametric, : all data on boundary of neighborhood. make span bigger
```

```
## Warning in simpleLoess(y, x, w, span, degree = degree, parametric = parametric, : pseudoinverse used at -0.005
```

```
## Warning in simpleLoess(y, x, w, span, degree = degree, parametric = parametric, : neighborhood radius 0.005
```

```
## Warning in simpleLoess(y, x, w, span, degree = degree, parametric = parametric, : reciprocal condition number 1
```

```
## Warning in simpleLoess(y, x, w, span, degree = degree, parametric = parametric, : There are other near singularities as well. 1.01
```

```
## Warning in simpleLoess(y, x, w, span, degree = degree, parametric = parametric, : zero-width neighborhood. make span bigger
```

```
## Warning: Computation failed in `stat_smooth()`:
## NA/NaN/Inf in foreign function call (arg 5)
```

```
## Warning in simpleLoess(y, x, w, span, degree = degree, parametric = parametric, : at -0.005
```

```
## Warning in simpleLoess(y, x, w, span, degree = degree, parametric = parametric, : radius 2.5e-05
```

```
## Warning in simpleLoess(y, x, w, span, degree = degree, parametric = parametric, : all data on boundary of neighborhood. make span bigger
```

```
## Warning in simpleLoess(y, x, w, span, degree = degree, parametric = parametric, : pseudoinverse used at -0.005
```

```
## Warning in simpleLoess(y, x, w, span, degree = degree, parametric = parametric, : neighborhood radius 0.005
```

```
## Warning in simpleLoess(y, x, w, span, degree = degree, parametric = parametric, : reciprocal condition number 1
```

```
## Warning in simpleLoess(y, x, w, span, degree = degree, parametric = parametric, : There are other near singularities as well. 1.01
```

```
## Warning in simpleLoess(y, x, w, span, degree = degree, parametric = parametric, : zero-width neighborhood. make span bigger
```

```
## Warning: Computation failed in `stat_smooth()`:
## NA/NaN/Inf in foreign function call (arg 5)
```

```
## Warning in simpleLoess(y, x, w, span, degree = degree, parametric = parametric, : at -0.005
```

```
## Warning in simpleLoess(y, x, w, span, degree = degree, parametric = parametric, : radius 2.5e-05
```

```
## Warning in simpleLoess(y, x, w, span, degree = degree, parametric = parametric, : all data on boundary of neighborhood. make span bigger
```

```
## Warning in simpleLoess(y, x, w, span, degree = degree, parametric = parametric, : pseudoinverse used at -0.005
```

```
## Warning in simpleLoess(y, x, w, span, degree = degree, parametric = parametric, : neighborhood radius 0.005
```

```
## Warning in simpleLoess(y, x, w, span, degree = degree, parametric = parametric, : reciprocal condition number 1
```

```
## Warning in simpleLoess(y, x, w, span, degree = degree, parametric = parametric, : There are other near singularities as well. 1.01
```

```
## Warning in simpleLoess(y, x, w, span, degree = degree, parametric = parametric, : zero-width neighborhood. make span bigger
```

```
## Warning: Computation failed in `stat_smooth()`:
## NA/NaN/Inf in foreign function call (arg 5)
```

```
## Warning in simpleLoess(y, x, w, span, degree = degree, parametric = parametric, : at -0.005
```

```
## Warning in simpleLoess(y, x, w, span, degree = degree, parametric = parametric, : radius 2.5e-05
```

```
## Warning in simpleLoess(y, x, w, span, degree = degree, parametric = parametric, : all data on boundary of neighborhood. make span bigger
```

```
## Warning in simpleLoess(y, x, w, span, degree = degree, parametric = parametric, : pseudoinverse used at -0.005
```

```
## Warning in simpleLoess(y, x, w, span, degree = degree, parametric = parametric, : neighborhood radius 0.005
```

```
## Warning in simpleLoess(y, x, w, span, degree = degree, parametric = parametric, : reciprocal condition number 1
```

```
## Warning in simpleLoess(y, x, w, span, degree = degree, parametric = parametric, : There are other near singularities as well. 1.01
```

```
## Warning in simpleLoess(y, x, w, span, degree = degree, parametric = parametric, : zero-width neighborhood. make span bigger
```

```
## Warning: Computation failed in `stat_smooth()`:
## NA/NaN/Inf in foreign function call (arg 5)
```

```
## Warning in simpleLoess(y, x, w, span, degree = degree, parametric = parametric, : at -0.005
```

```
## Warning in simpleLoess(y, x, w, span, degree = degree, parametric = parametric, : radius 2.5e-05
```

```
## Warning in simpleLoess(y, x, w, span, degree = degree, parametric = parametric, : all data on boundary of neighborhood. make span bigger
```

```
## Warning in simpleLoess(y, x, w, span, degree = degree, parametric = parametric, : pseudoinverse used at -0.005
```

```
## Warning in simpleLoess(y, x, w, span, degree = degree, parametric = parametric, : neighborhood radius 0.005
```

```
## Warning in simpleLoess(y, x, w, span, degree = degree, parametric = parametric, : reciprocal condition number 1
```

```
## Warning in simpleLoess(y, x, w, span, degree = degree, parametric = parametric, : There are other near singularities as well. 1.01
```

```
## Warning in simpleLoess(y, x, w, span, degree = degree, parametric = parametric, : zero-width neighborhood. make span bigger
```

```
## Warning: Computation failed in `stat_smooth()`:
## NA/NaN/Inf in foreign function call (arg 5)
```

```
## Warning in simpleLoess(y, x, w, span, degree = degree, parametric = parametric, : at -0.005
```

```
## Warning in simpleLoess(y, x, w, span, degree = degree, parametric = parametric, : radius 2.5e-05
```

```
## Warning in simpleLoess(y, x, w, span, degree = degree, parametric = parametric, : all data on boundary of neighborhood. make span bigger
```

```
## Warning in simpleLoess(y, x, w, span, degree = degree, parametric = parametric, : pseudoinverse used at -0.005
```

```
## Warning in simpleLoess(y, x, w, span, degree = degree, parametric = parametric, : neighborhood radius 0.005
```

```
## Warning in simpleLoess(y, x, w, span, degree = degree, parametric = parametric, : reciprocal condition number 1
```

```
## Warning in simpleLoess(y, x, w, span, degree = degree, parametric = parametric, : There are other near singularities as well. 1.01
```

```
## Warning in simpleLoess(y, x, w, span, degree = degree, parametric = parametric, : zero-width neighborhood. make span bigger
```

```
## Warning: Computation failed in `stat_smooth()`:
## NA/NaN/Inf in foreign function call (arg 5)
```

```
## Warning in simpleLoess(y, x, w, span, degree = degree, parametric = parametric, : at -0.005
```

```
## Warning in simpleLoess(y, x, w, span, degree = degree, parametric = parametric, : radius 2.5e-05
```

```
## Warning in simpleLoess(y, x, w, span, degree = degree, parametric = parametric, : all data on boundary of neighborhood. make span bigger
```

```
## Warning in simpleLoess(y, x, w, span, degree = degree, parametric = parametric, : pseudoinverse used at -0.005
```

```
## Warning in simpleLoess(y, x, w, span, degree = degree, parametric = parametric, : neighborhood radius 0.005
```

```
## Warning in simpleLoess(y, x, w, span, degree = degree, parametric = parametric, : reciprocal condition number 1
```

```
## Warning in simpleLoess(y, x, w, span, degree = degree, parametric = parametric, : There are other near singularities as well. 1.01
```

```
## Warning in simpleLoess(y, x, w, span, degree = degree, parametric = parametric, : zero-width neighborhood. make span bigger
```

```
## Warning: Computation failed in `stat_smooth()`:
## NA/NaN/Inf in foreign function call (arg 5)
```

```
## Warning in simpleLoess(y, x, w, span, degree = degree, parametric = parametric, : at -0.005
```

```
## Warning in simpleLoess(y, x, w, span, degree = degree, parametric = parametric, : radius 2.5e-05
```

```
## Warning in simpleLoess(y, x, w, span, degree = degree, parametric = parametric, : all data on boundary of neighborhood. make span bigger
```

```
## Warning in simpleLoess(y, x, w, span, degree = degree, parametric = parametric, : pseudoinverse used at -0.005
```

```
## Warning in simpleLoess(y, x, w, span, degree = degree, parametric = parametric, : neighborhood radius 0.005
```

```
## Warning in simpleLoess(y, x, w, span, degree = degree, parametric = parametric, : reciprocal condition number 1
```

```
## Warning in simpleLoess(y, x, w, span, degree = degree, parametric = parametric, : There are other near singularities as well. 1.01
```

```
## Warning in simpleLoess(y, x, w, span, degree = degree, parametric = parametric, : zero-width neighborhood. make span bigger
```

```
## Warning: Computation failed in `stat_smooth()`:
## NA/NaN/Inf in foreign function call (arg 5)
```

```
## Warning in simpleLoess(y, x, w, span, degree = degree, parametric = parametric, : pseudoinverse used at -0.005
```

```
## Warning in simpleLoess(y, x, w, span, degree = degree, parametric = parametric, : neighborhood radius 1.005
```

```
## Warning in simpleLoess(y, x, w, span, degree = degree, parametric = parametric, : reciprocal condition number 0
```

```
## Warning in simpleLoess(y, x, w, span, degree = degree, parametric = parametric, : There are other near singularities as well. 1.01
```

```
## Warning in predLoess(object$y, object$x, newx = if (is.null(newdata)) object$x else if (is.data.frame(newdata)) as.matrix(model.frame(delete.response(terms(object)), : pseudoinverse used at -0.005
```

```
## Warning in predLoess(object$y, object$x, newx = if (is.null(newdata)) object$x else if (is.data.frame(newdata)) as.matrix(model.frame(delete.response(terms(object)), : neighborhood radius 1.005
```

```
## Warning in predLoess(object$y, object$x, newx = if (is.null(newdata)) object$x else if (is.data.frame(newdata)) as.matrix(model.frame(delete.response(terms(object)), : reciprocal condition number 0
```

```
## Warning in predLoess(object$y, object$x, newx = if (is.null(newdata)) object$x else if (is.data.frame(newdata)) as.matrix(model.frame(delete.response(terms(object)), : There are other near
## singularities as well. 1.01
```

```
## Warning in simpleLoess(y, x, w, span, degree = degree, parametric = parametric, : at -0.005
```

```
## Warning in simpleLoess(y, x, w, span, degree = degree, parametric = parametric, : radius 2.5e-05
```

```
## Warning in simpleLoess(y, x, w, span, degree = degree, parametric = parametric, : all data on boundary of neighborhood. make span bigger
```

```
## Warning in simpleLoess(y, x, w, span, degree = degree, parametric = parametric, : pseudoinverse used at -0.005
```

```
## Warning in simpleLoess(y, x, w, span, degree = degree, parametric = parametric, : neighborhood radius 0.005
```

```
## Warning in simpleLoess(y, x, w, span, degree = degree, parametric = parametric, : reciprocal condition number 1
```

```
## Warning in simpleLoess(y, x, w, span, degree = degree, parametric = parametric, : There are other near singularities as well. 1.01
```

```
## Warning in simpleLoess(y, x, w, span, degree = degree, parametric = parametric, : zero-width neighborhood. make span bigger
```

```
## Warning: Computation failed in `stat_smooth()`:
## NA/NaN/Inf in foreign function call (arg 5)
```

```
## Warning in simpleLoess(y, x, w, span, degree = degree, parametric = parametric, : pseudoinverse used at -0.005
```

```
## Warning in simpleLoess(y, x, w, span, degree = degree, parametric = parametric, : neighborhood radius 1.005
```

```
## Warning in simpleLoess(y, x, w, span, degree = degree, parametric = parametric, : reciprocal condition number 0
```

```
## Warning in simpleLoess(y, x, w, span, degree = degree, parametric = parametric, : There are other near singularities as well. 1.01
```

```
## Warning in predLoess(object$y, object$x, newx = if (is.null(newdata)) object$x else if (is.data.frame(newdata)) as.matrix(model.frame(delete.response(terms(object)), : pseudoinverse used at -0.005
```

```
## Warning in predLoess(object$y, object$x, newx = if (is.null(newdata)) object$x else if (is.data.frame(newdata)) as.matrix(model.frame(delete.response(terms(object)), : neighborhood radius 1.005
```

```
## Warning in predLoess(object$y, object$x, newx = if (is.null(newdata)) object$x else if (is.data.frame(newdata)) as.matrix(model.frame(delete.response(terms(object)), : reciprocal condition number 0
```

```
## Warning in predLoess(object$y, object$x, newx = if (is.null(newdata)) object$x else if (is.data.frame(newdata)) as.matrix(model.frame(delete.response(terms(object)), : There are other near
## singularities as well. 1.01
```

```
## Warning in simpleLoess(y, x, w, span, degree = degree, parametric = parametric, : at -0.005
```

```
## Warning in simpleLoess(y, x, w, span, degree = degree, parametric = parametric, : radius 2.5e-05
```

```
## Warning in simpleLoess(y, x, w, span, degree = degree, parametric = parametric, : all data on boundary of neighborhood. make span bigger
```

```
## Warning in simpleLoess(y, x, w, span, degree = degree, parametric = parametric, : pseudoinverse used at -0.005
```

```
## Warning in simpleLoess(y, x, w, span, degree = degree, parametric = parametric, : neighborhood radius 0.005
```

```
## Warning in simpleLoess(y, x, w, span, degree = degree, parametric = parametric, : reciprocal condition number 1
```

```
## Warning in simpleLoess(y, x, w, span, degree = degree, parametric = parametric, : There are other near singularities as well. 1.01
```

```
## Warning in simpleLoess(y, x, w, span, degree = degree, parametric = parametric, : zero-width neighborhood. make span bigger
```

```
## Warning: Computation failed in `stat_smooth()`:
## NA/NaN/Inf in foreign function call (arg 5)
```

```
## Warning in simpleLoess(y, x, w, span, degree = degree, parametric = parametric, : at -0.005
```

```
## Warning in simpleLoess(y, x, w, span, degree = degree, parametric = parametric, : radius 2.5e-05
```

```
## Warning in simpleLoess(y, x, w, span, degree = degree, parametric = parametric, : all data on boundary of neighborhood. make span bigger
```

```
## Warning in simpleLoess(y, x, w, span, degree = degree, parametric = parametric, : pseudoinverse used at -0.005
```

```
## Warning in simpleLoess(y, x, w, span, degree = degree, parametric = parametric, : neighborhood radius 0.005
```

```
## Warning in simpleLoess(y, x, w, span, degree = degree, parametric = parametric, : reciprocal condition number 1
```

```
## Warning in simpleLoess(y, x, w, span, degree = degree, parametric = parametric, : There are other near singularities as well. 1.01
```

```
## Warning in simpleLoess(y, x, w, span, degree = degree, parametric = parametric, : zero-width neighborhood. make span bigger
```

```
## Warning: Computation failed in `stat_smooth()`:
## NA/NaN/Inf in foreign function call (arg 5)
```

```
## Warning in simpleLoess(y, x, w, span, degree = degree, parametric = parametric, : at -0.005
```

```
## Warning in simpleLoess(y, x, w, span, degree = degree, parametric = parametric, : radius 2.5e-05
```

```
## Warning in simpleLoess(y, x, w, span, degree = degree, parametric = parametric, : all data on boundary of neighborhood. make span bigger
```

```
## Warning in simpleLoess(y, x, w, span, degree = degree, parametric = parametric, : pseudoinverse used at -0.005
```

```
## Warning in simpleLoess(y, x, w, span, degree = degree, parametric = parametric, : neighborhood radius 0.005
```

```
## Warning in simpleLoess(y, x, w, span, degree = degree, parametric = parametric, : reciprocal condition number 1
```

```
## Warning in simpleLoess(y, x, w, span, degree = degree, parametric = parametric, : There are other near singularities as well. 1.01
```

```
## Warning in simpleLoess(y, x, w, span, degree = degree, parametric = parametric, : zero-width neighborhood. make span bigger
```

```
## Warning: Computation failed in `stat_smooth()`:
## NA/NaN/Inf in foreign function call (arg 5)
```

```
## Warning in simpleLoess(y, x, w, span, degree = degree, parametric = parametric, : at -0.005
```

```
## Warning in simpleLoess(y, x, w, span, degree = degree, parametric = parametric, : radius 2.5e-05
```

```
## Warning in simpleLoess(y, x, w, span, degree = degree, parametric = parametric, : all data on boundary of neighborhood. make span bigger
```

```
## Warning in simpleLoess(y, x, w, span, degree = degree, parametric = parametric, : pseudoinverse used at -0.005
```

```
## Warning in simpleLoess(y, x, w, span, degree = degree, parametric = parametric, : neighborhood radius 0.005
```

```
## Warning in simpleLoess(y, x, w, span, degree = degree, parametric = parametric, : reciprocal condition number 1
```

```
## Warning in simpleLoess(y, x, w, span, degree = degree, parametric = parametric, : There are other near singularities as well. 1.01
```

```
## Warning in simpleLoess(y, x, w, span, degree = degree, parametric = parametric, : zero-width neighborhood. make span bigger
```

```
## Warning: Computation failed in `stat_smooth()`:
## NA/NaN/Inf in foreign function call (arg 5)
```

```
## Warning in simpleLoess(y, x, w, span, degree = degree, parametric = parametric, : at -0.005
```

```
## Warning in simpleLoess(y, x, w, span, degree = degree, parametric = parametric, : radius 2.5e-05
```

```
## Warning in simpleLoess(y, x, w, span, degree = degree, parametric = parametric, : all data on boundary of neighborhood. make span bigger
```

```
## Warning in simpleLoess(y, x, w, span, degree = degree, parametric = parametric, : pseudoinverse used at -0.005
```

```
## Warning in simpleLoess(y, x, w, span, degree = degree, parametric = parametric, : neighborhood radius 0.005
```

```
## Warning in simpleLoess(y, x, w, span, degree = degree, parametric = parametric, : reciprocal condition number 1
```

```
## Warning in simpleLoess(y, x, w, span, degree = degree, parametric = parametric, : There are other near singularities as well. 1.01
```

```
## Warning in simpleLoess(y, x, w, span, degree = degree, parametric = parametric, : zero-width neighborhood. make span bigger
```

```
## Warning: Computation failed in `stat_smooth()`:
## NA/NaN/Inf in foreign function call (arg 5)
```

```
## Warning in simpleLoess(y, x, w, span, degree = degree, parametric = parametric, : at -0.005
```

```
## Warning in simpleLoess(y, x, w, span, degree = degree, parametric = parametric, : radius 2.5e-05
```

```
## Warning in simpleLoess(y, x, w, span, degree = degree, parametric = parametric, : all data on boundary of neighborhood. make span bigger
```

```
## Warning in simpleLoess(y, x, w, span, degree = degree, parametric = parametric, : pseudoinverse used at -0.005
```

```
## Warning in simpleLoess(y, x, w, span, degree = degree, parametric = parametric, : neighborhood radius 0.005
```

```
## Warning in simpleLoess(y, x, w, span, degree = degree, parametric = parametric, : reciprocal condition number 1
```

```
## Warning in simpleLoess(y, x, w, span, degree = degree, parametric = parametric, : There are other near singularities as well. 1.01
```

```
## Warning in simpleLoess(y, x, w, span, degree = degree, parametric = parametric, : zero-width neighborhood. make span bigger
```

```
## Warning: Computation failed in `stat_smooth()`:
## NA/NaN/Inf in foreign function call (arg 5)
```

```
## Warning in simpleLoess(y, x, w, span, degree = degree, parametric = parametric, : at -0.005
```

```
## Warning in simpleLoess(y, x, w, span, degree = degree, parametric = parametric, : radius 2.5e-05
```

```
## Warning in simpleLoess(y, x, w, span, degree = degree, parametric = parametric, : all data on boundary of neighborhood. make span bigger
```

```
## Warning in simpleLoess(y, x, w, span, degree = degree, parametric = parametric, : pseudoinverse used at -0.005
```

```
## Warning in simpleLoess(y, x, w, span, degree = degree, parametric = parametric, : neighborhood radius 0.005
```

```
## Warning in simpleLoess(y, x, w, span, degree = degree, parametric = parametric, : reciprocal condition number 1
```

```
## Warning in simpleLoess(y, x, w, span, degree = degree, parametric = parametric, : There are other near singularities as well. 1.01
```

```
## Warning in simpleLoess(y, x, w, span, degree = degree, parametric = parametric, : zero-width neighborhood. make span bigger
```

```
## Warning: Computation failed in `stat_smooth()`:
## NA/NaN/Inf in foreign function call (arg 5)
```

```
## Warning in simpleLoess(y, x, w, span, degree = degree, parametric = parametric, : at -0.005
```

```
## Warning in simpleLoess(y, x, w, span, degree = degree, parametric = parametric, : radius 2.5e-05
```

```
## Warning in simpleLoess(y, x, w, span, degree = degree, parametric = parametric, : all data on boundary of neighborhood. make span bigger
```

```
## Warning in simpleLoess(y, x, w, span, degree = degree, parametric = parametric, : pseudoinverse used at -0.005
```

```
## Warning in simpleLoess(y, x, w, span, degree = degree, parametric = parametric, : neighborhood radius 0.005
```

```
## Warning in simpleLoess(y, x, w, span, degree = degree, parametric = parametric, : reciprocal condition number 1
```

```
## Warning in simpleLoess(y, x, w, span, degree = degree, parametric = parametric, : There are other near singularities as well. 1.01
```

```
## Warning in simpleLoess(y, x, w, span, degree = degree, parametric = parametric, : zero-width neighborhood. make span bigger
```

```
## Warning: Computation failed in `stat_smooth()`:
## NA/NaN/Inf in foreign function call (arg 5)
```

```
## Warning in simpleLoess(y, x, w, span, degree = degree, parametric = parametric, : at -0.005
```

```
## Warning in simpleLoess(y, x, w, span, degree = degree, parametric = parametric, : radius 2.5e-05
```

```
## Warning in simpleLoess(y, x, w, span, degree = degree, parametric = parametric, : all data on boundary of neighborhood. make span bigger
```

```
## Warning in simpleLoess(y, x, w, span, degree = degree, parametric = parametric, : pseudoinverse used at -0.005
```

```
## Warning in simpleLoess(y, x, w, span, degree = degree, parametric = parametric, : neighborhood radius 0.005
```

```
## Warning in simpleLoess(y, x, w, span, degree = degree, parametric = parametric, : reciprocal condition number 1
```

```
## Warning in simpleLoess(y, x, w, span, degree = degree, parametric = parametric, : There are other near singularities as well. 1.01
```

```
## Warning in simpleLoess(y, x, w, span, degree = degree, parametric = parametric, : zero-width neighborhood. make span bigger
```

```
## Warning: Computation failed in `stat_smooth()`:
## NA/NaN/Inf in foreign function call (arg 5)
```

```
## Warning in simpleLoess(y, x, w, span, degree = degree, parametric = parametric, : at -0.005
```

```
## Warning in simpleLoess(y, x, w, span, degree = degree, parametric = parametric, : radius 2.5e-05
```

```
## Warning in simpleLoess(y, x, w, span, degree = degree, parametric = parametric, : all data on boundary of neighborhood. make span bigger
```

```
## Warning in simpleLoess(y, x, w, span, degree = degree, parametric = parametric, : pseudoinverse used at -0.005
```

```
## Warning in simpleLoess(y, x, w, span, degree = degree, parametric = parametric, : neighborhood radius 0.005
```

```
## Warning in simpleLoess(y, x, w, span, degree = degree, parametric = parametric, : reciprocal condition number 1
```

```
## Warning in simpleLoess(y, x, w, span, degree = degree, parametric = parametric, : There are other near singularities as well. 1.01
```

```
## Warning in simpleLoess(y, x, w, span, degree = degree, parametric = parametric, : zero-width neighborhood. make span bigger
```

```
## Warning: Computation failed in `stat_smooth()`:
## NA/NaN/Inf in foreign function call (arg 5)
```

```
## Warning in simpleLoess(y, x, w, span, degree = degree, parametric = parametric, : at -0.005
```

```
## Warning in simpleLoess(y, x, w, span, degree = degree, parametric = parametric, : radius 2.5e-05
```

```
## Warning in simpleLoess(y, x, w, span, degree = degree, parametric = parametric, : all data on boundary of neighborhood. make span bigger
```

```
## Warning in simpleLoess(y, x, w, span, degree = degree, parametric = parametric, : pseudoinverse used at -0.005
```

```
## Warning in simpleLoess(y, x, w, span, degree = degree, parametric = parametric, : neighborhood radius 0.005
```

```
## Warning in simpleLoess(y, x, w, span, degree = degree, parametric = parametric, : reciprocal condition number 1
```

```
## Warning in simpleLoess(y, x, w, span, degree = degree, parametric = parametric, : There are other near singularities as well. 1.01
```

```
## Warning in simpleLoess(y, x, w, span, degree = degree, parametric = parametric, : zero-width neighborhood. make span bigger
```

```
## Warning: Computation failed in `stat_smooth()`:
## NA/NaN/Inf in foreign function call (arg 5)
```

```
## Warning in simpleLoess(y, x, w, span, degree = degree, parametric = parametric, : pseudoinverse used at -0.005
```

```
## Warning in simpleLoess(y, x, w, span, degree = degree, parametric = parametric, : neighborhood radius 1.005
```

```
## Warning in simpleLoess(y, x, w, span, degree = degree, parametric = parametric, : reciprocal condition number 0
```

```
## Warning in simpleLoess(y, x, w, span, degree = degree, parametric = parametric, : There are other near singularities as well. 1.01
```

```
## Warning in predLoess(object$y, object$x, newx = if (is.null(newdata)) object$x else if (is.data.frame(newdata)) as.matrix(model.frame(delete.response(terms(object)), : pseudoinverse used at -0.005
```

```
## Warning in predLoess(object$y, object$x, newx = if (is.null(newdata)) object$x else if (is.data.frame(newdata)) as.matrix(model.frame(delete.response(terms(object)), : neighborhood radius 1.005
```

```
## Warning in predLoess(object$y, object$x, newx = if (is.null(newdata)) object$x else if (is.data.frame(newdata)) as.matrix(model.frame(delete.response(terms(object)), : reciprocal condition number 0
```

```
## Warning in predLoess(object$y, object$x, newx = if (is.null(newdata)) object$x else if (is.data.frame(newdata)) as.matrix(model.frame(delete.response(terms(object)), : There are other near
## singularities as well. 1.01
```

```
## Warning in simpleLoess(y, x, w, span, degree = degree, parametric = parametric, : at -0.005
```

```
## Warning in simpleLoess(y, x, w, span, degree = degree, parametric = parametric, : radius 2.5e-05
```

```
## Warning in simpleLoess(y, x, w, span, degree = degree, parametric = parametric, : all data on boundary of neighborhood. make span bigger
```

```
## Warning in simpleLoess(y, x, w, span, degree = degree, parametric = parametric, : pseudoinverse used at -0.005
```

```
## Warning in simpleLoess(y, x, w, span, degree = degree, parametric = parametric, : neighborhood radius 0.005
```

```
## Warning in simpleLoess(y, x, w, span, degree = degree, parametric = parametric, : reciprocal condition number 1
```

```
## Warning in simpleLoess(y, x, w, span, degree = degree, parametric = parametric, : There are other near singularities as well. 1.01
```

```
## Warning in simpleLoess(y, x, w, span, degree = degree, parametric = parametric, : zero-width neighborhood. make span bigger
```

```
## Warning: Computation failed in `stat_smooth()`:
## NA/NaN/Inf in foreign function call (arg 5)
```

```
## Warning in simpleLoess(y, x, w, span, degree = degree, parametric = parametric, : at -0.005
```

```
## Warning in simpleLoess(y, x, w, span, degree = degree, parametric = parametric, : radius 2.5e-05
```

```
## Warning in simpleLoess(y, x, w, span, degree = degree, parametric = parametric, : all data on boundary of neighborhood. make span bigger
```

```
## Warning in simpleLoess(y, x, w, span, degree = degree, parametric = parametric, : pseudoinverse used at -0.005
```

```
## Warning in simpleLoess(y, x, w, span, degree = degree, parametric = parametric, : neighborhood radius 0.005
```

```
## Warning in simpleLoess(y, x, w, span, degree = degree, parametric = parametric, : reciprocal condition number 1
```

```
## Warning in simpleLoess(y, x, w, span, degree = degree, parametric = parametric, : There are other near singularities as well. 1.01
```

```
## Warning in simpleLoess(y, x, w, span, degree = degree, parametric = parametric, : zero-width neighborhood. make span bigger
```

```
## Warning: Computation failed in `stat_smooth()`:
## NA/NaN/Inf in foreign function call (arg 5)
```

```
## Warning in simpleLoess(y, x, w, span, degree = degree, parametric = parametric, : at -0.005
```

```
## Warning in simpleLoess(y, x, w, span, degree = degree, parametric = parametric, : radius 2.5e-05
```

```
## Warning in simpleLoess(y, x, w, span, degree = degree, parametric = parametric, : all data on boundary of neighborhood. make span bigger
```

```
## Warning in simpleLoess(y, x, w, span, degree = degree, parametric = parametric, : pseudoinverse used at -0.005
```

```
## Warning in simpleLoess(y, x, w, span, degree = degree, parametric = parametric, : neighborhood radius 0.005
```

```
## Warning in simpleLoess(y, x, w, span, degree = degree, parametric = parametric, : reciprocal condition number 1
```

```
## Warning in simpleLoess(y, x, w, span, degree = degree, parametric = parametric, : There are other near singularities as well. 1.01
```

```
## Warning in simpleLoess(y, x, w, span, degree = degree, parametric = parametric, : zero-width neighborhood. make span bigger
```

```
## Warning: Computation failed in `stat_smooth()`:
## NA/NaN/Inf in foreign function call (arg 5)
```

```
## Warning in simpleLoess(y, x, w, span, degree = degree, parametric = parametric, : pseudoinverse used at -0.005
```

```
## Warning in simpleLoess(y, x, w, span, degree = degree, parametric = parametric, : neighborhood radius 1.005
```

```
## Warning in simpleLoess(y, x, w, span, degree = degree, parametric = parametric, : reciprocal condition number 0
```

```
## Warning in simpleLoess(y, x, w, span, degree = degree, parametric = parametric, : There are other near singularities as well. 1.01
```

```
## Warning in predLoess(object$y, object$x, newx = if (is.null(newdata)) object$x else if (is.data.frame(newdata)) as.matrix(model.frame(delete.response(terms(object)), : pseudoinverse used at -0.005
```

```
## Warning in predLoess(object$y, object$x, newx = if (is.null(newdata)) object$x else if (is.data.frame(newdata)) as.matrix(model.frame(delete.response(terms(object)), : neighborhood radius 1.005
```

```
## Warning in predLoess(object$y, object$x, newx = if (is.null(newdata)) object$x else if (is.data.frame(newdata)) as.matrix(model.frame(delete.response(terms(object)), : reciprocal condition number 0
```

```
## Warning in predLoess(object$y, object$x, newx = if (is.null(newdata)) object$x else if (is.data.frame(newdata)) as.matrix(model.frame(delete.response(terms(object)), : There are other near
## singularities as well. 1.01
```

```
## Warning in simpleLoess(y, x, w, span, degree = degree, parametric = parametric, : at -0.005
```

```
## Warning in simpleLoess(y, x, w, span, degree = degree, parametric = parametric, : radius 2.5e-05
```

```
## Warning in simpleLoess(y, x, w, span, degree = degree, parametric = parametric, : all data on boundary of neighborhood. make span bigger
```

```
## Warning in simpleLoess(y, x, w, span, degree = degree, parametric = parametric, : pseudoinverse used at -0.005
```

```
## Warning in simpleLoess(y, x, w, span, degree = degree, parametric = parametric, : neighborhood radius 0.005
```

```
## Warning in simpleLoess(y, x, w, span, degree = degree, parametric = parametric, : reciprocal condition number 1
```

```
## Warning in simpleLoess(y, x, w, span, degree = degree, parametric = parametric, : There are other near singularities as well. 1.01
```

```
## Warning in simpleLoess(y, x, w, span, degree = degree, parametric = parametric, : zero-width neighborhood. make span bigger
```

```
## Warning: Computation failed in `stat_smooth()`:
## NA/NaN/Inf in foreign function call (arg 5)
```

```
## Warning in simpleLoess(y, x, w, span, degree = degree, parametric = parametric, : at -0.005
```

```
## Warning in simpleLoess(y, x, w, span, degree = degree, parametric = parametric, : radius 2.5e-05
```

```
## Warning in simpleLoess(y, x, w, span, degree = degree, parametric = parametric, : all data on boundary of neighborhood. make span bigger
```

```
## Warning in simpleLoess(y, x, w, span, degree = degree, parametric = parametric, : pseudoinverse used at -0.005
```

```
## Warning in simpleLoess(y, x, w, span, degree = degree, parametric = parametric, : neighborhood radius 0.005
```

```
## Warning in simpleLoess(y, x, w, span, degree = degree, parametric = parametric, : reciprocal condition number 1
```

```
## Warning in simpleLoess(y, x, w, span, degree = degree, parametric = parametric, : There are other near singularities as well. 1.01
```

```
## Warning in simpleLoess(y, x, w, span, degree = degree, parametric = parametric, : zero-width neighborhood. make span bigger
```

```
## Warning: Computation failed in `stat_smooth()`:
## NA/NaN/Inf in foreign function call (arg 5)
```

```
## Warning in simpleLoess(y, x, w, span, degree = degree, parametric = parametric, : at -0.005
```

```
## Warning in simpleLoess(y, x, w, span, degree = degree, parametric = parametric, : radius 2.5e-05
```

```
## Warning in simpleLoess(y, x, w, span, degree = degree, parametric = parametric, : all data on boundary of neighborhood. make span bigger
```

```
## Warning in simpleLoess(y, x, w, span, degree = degree, parametric = parametric, : pseudoinverse used at -0.005
```

```
## Warning in simpleLoess(y, x, w, span, degree = degree, parametric = parametric, : neighborhood radius 0.005
```

```
## Warning in simpleLoess(y, x, w, span, degree = degree, parametric = parametric, : reciprocal condition number 1
```

```
## Warning in simpleLoess(y, x, w, span, degree = degree, parametric = parametric, : There are other near singularities as well. 1.01
```

```
## Warning in simpleLoess(y, x, w, span, degree = degree, parametric = parametric, : zero-width neighborhood. make span bigger
```

```
## Warning: Computation failed in `stat_smooth()`:
## NA/NaN/Inf in foreign function call (arg 5)
```

```
## Warning in simpleLoess(y, x, w, span, degree = degree, parametric = parametric, : at -0.005
```

```
## Warning in simpleLoess(y, x, w, span, degree = degree, parametric = parametric, : radius 2.5e-05
```

```
## Warning in simpleLoess(y, x, w, span, degree = degree, parametric = parametric, : all data on boundary of neighborhood. make span bigger
```

```
## Warning in simpleLoess(y, x, w, span, degree = degree, parametric = parametric, : pseudoinverse used at -0.005
```

```
## Warning in simpleLoess(y, x, w, span, degree = degree, parametric = parametric, : neighborhood radius 0.005
```

```
## Warning in simpleLoess(y, x, w, span, degree = degree, parametric = parametric, : reciprocal condition number 1
```

```
## Warning in simpleLoess(y, x, w, span, degree = degree, parametric = parametric, : There are other near singularities as well. 1.01
```

```
## Warning in simpleLoess(y, x, w, span, degree = degree, parametric = parametric, : zero-width neighborhood. make span bigger
```

```
## Warning: Computation failed in `stat_smooth()`:
## NA/NaN/Inf in foreign function call (arg 5)
```

```
## Warning in simpleLoess(y, x, w, span, degree = degree, parametric = parametric, : at -0.005
```

```
## Warning in simpleLoess(y, x, w, span, degree = degree, parametric = parametric, : radius 2.5e-05
```

```
## Warning in simpleLoess(y, x, w, span, degree = degree, parametric = parametric, : all data on boundary of neighborhood. make span bigger
```

```
## Warning in simpleLoess(y, x, w, span, degree = degree, parametric = parametric, : pseudoinverse used at -0.005
```

```
## Warning in simpleLoess(y, x, w, span, degree = degree, parametric = parametric, : neighborhood radius 0.005
```

```
## Warning in simpleLoess(y, x, w, span, degree = degree, parametric = parametric, : reciprocal condition number 1
```

```
## Warning in simpleLoess(y, x, w, span, degree = degree, parametric = parametric, : There are other near singularities as well. 1.01
```

```
## Warning in simpleLoess(y, x, w, span, degree = degree, parametric = parametric, : zero-width neighborhood. make span bigger
```

```
## Warning: Computation failed in `stat_smooth()`:
## NA/NaN/Inf in foreign function call (arg 5)
```

```
## Warning in simpleLoess(y, x, w, span, degree = degree, parametric = parametric, : pseudoinverse used at -0.005
```

```
## Warning in simpleLoess(y, x, w, span, degree = degree, parametric = parametric, : neighborhood radius 1.005
```

```
## Warning in simpleLoess(y, x, w, span, degree = degree, parametric = parametric, : reciprocal condition number 0
```

```
## Warning in simpleLoess(y, x, w, span, degree = degree, parametric = parametric, : There are other near singularities as well. 1.01
```

```
## Warning in predLoess(object$y, object$x, newx = if (is.null(newdata)) object$x else if (is.data.frame(newdata)) as.matrix(model.frame(delete.response(terms(object)), : pseudoinverse used at -0.005
```

```
## Warning in predLoess(object$y, object$x, newx = if (is.null(newdata)) object$x else if (is.data.frame(newdata)) as.matrix(model.frame(delete.response(terms(object)), : neighborhood radius 1.005
```

```
## Warning in predLoess(object$y, object$x, newx = if (is.null(newdata)) object$x else if (is.data.frame(newdata)) as.matrix(model.frame(delete.response(terms(object)), : reciprocal condition number 0
```

```
## Warning in predLoess(object$y, object$x, newx = if (is.null(newdata)) object$x else if (is.data.frame(newdata)) as.matrix(model.frame(delete.response(terms(object)), : There are other near
## singularities as well. 1.01
```

```
## Warning in simpleLoess(y, x, w, span, degree = degree, parametric = parametric, : at -0.005
```

```
## Warning in simpleLoess(y, x, w, span, degree = degree, parametric = parametric, : radius 2.5e-05
```

```
## Warning in simpleLoess(y, x, w, span, degree = degree, parametric = parametric, : all data on boundary of neighborhood. make span bigger
```

```
## Warning in simpleLoess(y, x, w, span, degree = degree, parametric = parametric, : pseudoinverse used at -0.005
```

```
## Warning in simpleLoess(y, x, w, span, degree = degree, parametric = parametric, : neighborhood radius 0.005
```

```
## Warning in simpleLoess(y, x, w, span, degree = degree, parametric = parametric, : reciprocal condition number 1
```

```
## Warning in simpleLoess(y, x, w, span, degree = degree, parametric = parametric, : There are other near singularities as well. 1.01
```

```
## Warning in simpleLoess(y, x, w, span, degree = degree, parametric = parametric, : zero-width neighborhood. make span bigger
```

```
## Warning: Computation failed in `stat_smooth()`:
## NA/NaN/Inf in foreign function call (arg 5)
```

```
## Warning in simpleLoess(y, x, w, span, degree = degree, parametric = parametric, : at -0.005
```

```
## Warning in simpleLoess(y, x, w, span, degree = degree, parametric = parametric, : radius 2.5e-05
```

```
## Warning in simpleLoess(y, x, w, span, degree = degree, parametric = parametric, : all data on boundary of neighborhood. make span bigger
```

```
## Warning in simpleLoess(y, x, w, span, degree = degree, parametric = parametric, : pseudoinverse used at -0.005
```

```
## Warning in simpleLoess(y, x, w, span, degree = degree, parametric = parametric, : neighborhood radius 0.005
```

```
## Warning in simpleLoess(y, x, w, span, degree = degree, parametric = parametric, : reciprocal condition number 1
```

```
## Warning in simpleLoess(y, x, w, span, degree = degree, parametric = parametric, : There are other near singularities as well. 1.01
```

```
## Warning in simpleLoess(y, x, w, span, degree = degree, parametric = parametric, : zero-width neighborhood. make span bigger
```

```
## Warning: Computation failed in `stat_smooth()`:
## NA/NaN/Inf in foreign function call (arg 5)
```

```
## Warning in simpleLoess(y, x, w, span, degree = degree, parametric = parametric, : at -0.005
```

```
## Warning in simpleLoess(y, x, w, span, degree = degree, parametric = parametric, : radius 2.5e-05
```

```
## Warning in simpleLoess(y, x, w, span, degree = degree, parametric = parametric, : all data on boundary of neighborhood. make span bigger
```

```
## Warning in simpleLoess(y, x, w, span, degree = degree, parametric = parametric, : pseudoinverse used at -0.005
```

```
## Warning in simpleLoess(y, x, w, span, degree = degree, parametric = parametric, : neighborhood radius 0.005
```

```
## Warning in simpleLoess(y, x, w, span, degree = degree, parametric = parametric, : reciprocal condition number 1
```

```
## Warning in simpleLoess(y, x, w, span, degree = degree, parametric = parametric, : There are other near singularities as well. 1.01
```

```
## Warning in simpleLoess(y, x, w, span, degree = degree, parametric = parametric, : zero-width neighborhood. make span bigger
```

```
## Warning: Computation failed in `stat_smooth()`:
## NA/NaN/Inf in foreign function call (arg 5)
```

```
## Warning in simpleLoess(y, x, w, span, degree = degree, parametric = parametric, : at -0.005
```

```
## Warning in simpleLoess(y, x, w, span, degree = degree, parametric = parametric, : radius 2.5e-05
```

```
## Warning in simpleLoess(y, x, w, span, degree = degree, parametric = parametric, : all data on boundary of neighborhood. make span bigger
```

```
## Warning in simpleLoess(y, x, w, span, degree = degree, parametric = parametric, : pseudoinverse used at -0.005
```

```
## Warning in simpleLoess(y, x, w, span, degree = degree, parametric = parametric, : neighborhood radius 0.005
```

```
## Warning in simpleLoess(y, x, w, span, degree = degree, parametric = parametric, : reciprocal condition number 1
```

```
## Warning in simpleLoess(y, x, w, span, degree = degree, parametric = parametric, : There are other near singularities as well. 1.01
```

```
## Warning in simpleLoess(y, x, w, span, degree = degree, parametric = parametric, : zero-width neighborhood. make span bigger
```

```
## Warning: Computation failed in `stat_smooth()`:
## NA/NaN/Inf in foreign function call (arg 5)
```

```
## Warning in simpleLoess(y, x, w, span, degree = degree, parametric = parametric, : at -0.005
```

```
## Warning in simpleLoess(y, x, w, span, degree = degree, parametric = parametric, : radius 2.5e-05
```

```
## Warning in simpleLoess(y, x, w, span, degree = degree, parametric = parametric, : all data on boundary of neighborhood. make span bigger
```

```
## Warning in simpleLoess(y, x, w, span, degree = degree, parametric = parametric, : pseudoinverse used at -0.005
```

```
## Warning in simpleLoess(y, x, w, span, degree = degree, parametric = parametric, : neighborhood radius 0.005
```

```
## Warning in simpleLoess(y, x, w, span, degree = degree, parametric = parametric, : reciprocal condition number 1
```

```
## Warning in simpleLoess(y, x, w, span, degree = degree, parametric = parametric, : There are other near singularities as well. 1.01
```

```
## Warning in simpleLoess(y, x, w, span, degree = degree, parametric = parametric, : zero-width neighborhood. make span bigger
```

```
## Warning: Computation failed in `stat_smooth()`:
## NA/NaN/Inf in foreign function call (arg 5)
```

```
## Warning in simpleLoess(y, x, w, span, degree = degree, parametric = parametric, : at -0.005
```

```
## Warning in simpleLoess(y, x, w, span, degree = degree, parametric = parametric, : radius 2.5e-05
```

```
## Warning in simpleLoess(y, x, w, span, degree = degree, parametric = parametric, : all data on boundary of neighborhood. make span bigger
```

```
## Warning in simpleLoess(y, x, w, span, degree = degree, parametric = parametric, : pseudoinverse used at -0.005
```

```
## Warning in simpleLoess(y, x, w, span, degree = degree, parametric = parametric, : neighborhood radius 0.005
```

```
## Warning in simpleLoess(y, x, w, span, degree = degree, parametric = parametric, : reciprocal condition number 1
```

```
## Warning in simpleLoess(y, x, w, span, degree = degree, parametric = parametric, : There are other near singularities as well. 1.01
```

```
## Warning in simpleLoess(y, x, w, span, degree = degree, parametric = parametric, : zero-width neighborhood. make span bigger
```

```
## Warning: Computation failed in `stat_smooth()`:
## NA/NaN/Inf in foreign function call (arg 5)
```

```
## Warning in simpleLoess(y, x, w, span, degree = degree, parametric = parametric, : at -0.005
```

```
## Warning in simpleLoess(y, x, w, span, degree = degree, parametric = parametric, : radius 2.5e-05
```

```
## Warning in simpleLoess(y, x, w, span, degree = degree, parametric = parametric, : all data on boundary of neighborhood. make span bigger
```

```
## Warning in simpleLoess(y, x, w, span, degree = degree, parametric = parametric, : pseudoinverse used at -0.005
```

```
## Warning in simpleLoess(y, x, w, span, degree = degree, parametric = parametric, : neighborhood radius 0.005
```

```
## Warning in simpleLoess(y, x, w, span, degree = degree, parametric = parametric, : reciprocal condition number 1
```

```
## Warning in simpleLoess(y, x, w, span, degree = degree, parametric = parametric, : There are other near singularities as well. 1.01
```

```
## Warning in simpleLoess(y, x, w, span, degree = degree, parametric = parametric, : zero-width neighborhood. make span bigger
```

```
## Warning: Computation failed in `stat_smooth()`:
## NA/NaN/Inf in foreign function call (arg 5)
```

```
## Warning in simpleLoess(y, x, w, span, degree = degree, parametric = parametric, : at -0.005
```

```
## Warning in simpleLoess(y, x, w, span, degree = degree, parametric = parametric, : radius 2.5e-05
```

```
## Warning in simpleLoess(y, x, w, span, degree = degree, parametric = parametric, : all data on boundary of neighborhood. make span bigger
```

```
## Warning in simpleLoess(y, x, w, span, degree = degree, parametric = parametric, : pseudoinverse used at -0.005
```

```
## Warning in simpleLoess(y, x, w, span, degree = degree, parametric = parametric, : neighborhood radius 0.005
```

```
## Warning in simpleLoess(y, x, w, span, degree = degree, parametric = parametric, : reciprocal condition number 1
```

```
## Warning in simpleLoess(y, x, w, span, degree = degree, parametric = parametric, : There are other near singularities as well. 1.01
```

```
## Warning in simpleLoess(y, x, w, span, degree = degree, parametric = parametric, : zero-width neighborhood. make span bigger
```

```
## Warning: Computation failed in `stat_smooth()`:
## NA/NaN/Inf in foreign function call (arg 5)
```

```
## Warning in simpleLoess(y, x, w, span, degree = degree, parametric = parametric, : at -0.005
```

```
## Warning in simpleLoess(y, x, w, span, degree = degree, parametric = parametric, : radius 2.5e-05
```

```
## Warning in simpleLoess(y, x, w, span, degree = degree, parametric = parametric, : all data on boundary of neighborhood. make span bigger
```

```
## Warning in simpleLoess(y, x, w, span, degree = degree, parametric = parametric, : pseudoinverse used at -0.005
```

```
## Warning in simpleLoess(y, x, w, span, degree = degree, parametric = parametric, : neighborhood radius 0.005
```

```
## Warning in simpleLoess(y, x, w, span, degree = degree, parametric = parametric, : reciprocal condition number 1
```

```
## Warning in simpleLoess(y, x, w, span, degree = degree, parametric = parametric, : There are other near singularities as well. 1.01
```

```
## Warning in simpleLoess(y, x, w, span, degree = degree, parametric = parametric, : zero-width neighborhood. make span bigger
```

```
## Warning: Computation failed in `stat_smooth()`:
## NA/NaN/Inf in foreign function call (arg 5)
```

```
## Warning in simpleLoess(y, x, w, span, degree = degree, parametric = parametric, : at -0.005
```

```
## Warning in simpleLoess(y, x, w, span, degree = degree, parametric = parametric, : radius 2.5e-05
```

```
## Warning in simpleLoess(y, x, w, span, degree = degree, parametric = parametric, : all data on boundary of neighborhood. make span bigger
```

```
## Warning in simpleLoess(y, x, w, span, degree = degree, parametric = parametric, : pseudoinverse used at -0.005
```

```
## Warning in simpleLoess(y, x, w, span, degree = degree, parametric = parametric, : neighborhood radius 0.005
```

```
## Warning in simpleLoess(y, x, w, span, degree = degree, parametric = parametric, : reciprocal condition number 1
```

```
## Warning in simpleLoess(y, x, w, span, degree = degree, parametric = parametric, : There are other near singularities as well. 1.01
```

```
## Warning in simpleLoess(y, x, w, span, degree = degree, parametric = parametric, : zero-width neighborhood. make span bigger
```

```
## Warning: Computation failed in `stat_smooth()`:
## NA/NaN/Inf in foreign function call (arg 5)
```

```
## Warning in simpleLoess(y, x, w, span, degree = degree, parametric = parametric, : at -0.005
```

```
## Warning in simpleLoess(y, x, w, span, degree = degree, parametric = parametric, : radius 2.5e-05
```

```
## Warning in simpleLoess(y, x, w, span, degree = degree, parametric = parametric, : all data on boundary of neighborhood. make span bigger
```

```
## Warning in simpleLoess(y, x, w, span, degree = degree, parametric = parametric, : pseudoinverse used at -0.005
```

```
## Warning in simpleLoess(y, x, w, span, degree = degree, parametric = parametric, : neighborhood radius 0.005
```

```
## Warning in simpleLoess(y, x, w, span, degree = degree, parametric = parametric, : reciprocal condition number 1
```

```
## Warning in simpleLoess(y, x, w, span, degree = degree, parametric = parametric, : There are other near singularities as well. 1.01
```

```
## Warning in simpleLoess(y, x, w, span, degree = degree, parametric = parametric, : zero-width neighborhood. make span bigger
```

```
## Warning: Computation failed in `stat_smooth()`:
## NA/NaN/Inf in foreign function call (arg 5)
```

```
## Warning in simpleLoess(y, x, w, span, degree = degree, parametric = parametric, : at -0.005
```

```
## Warning in simpleLoess(y, x, w, span, degree = degree, parametric = parametric, : radius 2.5e-05
```

```
## Warning in simpleLoess(y, x, w, span, degree = degree, parametric = parametric, : all data on boundary of neighborhood. make span bigger
```

```
## Warning in simpleLoess(y, x, w, span, degree = degree, parametric = parametric, : pseudoinverse used at -0.005
```

```
## Warning in simpleLoess(y, x, w, span, degree = degree, parametric = parametric, : neighborhood radius 0.005
```

```
## Warning in simpleLoess(y, x, w, span, degree = degree, parametric = parametric, : reciprocal condition number 1
```

```
## Warning in simpleLoess(y, x, w, span, degree = degree, parametric = parametric, : There are other near singularities as well. 1.01
```

```
## Warning in simpleLoess(y, x, w, span, degree = degree, parametric = parametric, : zero-width neighborhood. make span bigger
```

```
## Warning: Computation failed in `stat_smooth()`:
## NA/NaN/Inf in foreign function call (arg 5)
```

```
## Warning in simpleLoess(y, x, w, span, degree = degree, parametric = parametric, : at -0.005
```

```
## Warning in simpleLoess(y, x, w, span, degree = degree, parametric = parametric, : radius 2.5e-05
```

```
## Warning in simpleLoess(y, x, w, span, degree = degree, parametric = parametric, : all data on boundary of neighborhood. make span bigger
```

```
## Warning in simpleLoess(y, x, w, span, degree = degree, parametric = parametric, : pseudoinverse used at -0.005
```

```
## Warning in simpleLoess(y, x, w, span, degree = degree, parametric = parametric, : neighborhood radius 0.005
```

```
## Warning in simpleLoess(y, x, w, span, degree = degree, parametric = parametric, : reciprocal condition number 1
```

```
## Warning in simpleLoess(y, x, w, span, degree = degree, parametric = parametric, : There are other near singularities as well. 1.01
```

```
## Warning in simpleLoess(y, x, w, span, degree = degree, parametric = parametric, : zero-width neighborhood. make span bigger
```

```
## Warning: Computation failed in `stat_smooth()`:
## NA/NaN/Inf in foreign function call (arg 5)
```

```
## Warning in simpleLoess(y, x, w, span, degree = degree, parametric = parametric, : at -0.005
```

```
## Warning in simpleLoess(y, x, w, span, degree = degree, parametric = parametric, : radius 2.5e-05
```

```
## Warning in simpleLoess(y, x, w, span, degree = degree, parametric = parametric, : all data on boundary of neighborhood. make span bigger
```

```
## Warning in simpleLoess(y, x, w, span, degree = degree, parametric = parametric, : pseudoinverse used at -0.005
```

```
## Warning in simpleLoess(y, x, w, span, degree = degree, parametric = parametric, : neighborhood radius 0.005
```

```
## Warning in simpleLoess(y, x, w, span, degree = degree, parametric = parametric, : reciprocal condition number 1
```

```
## Warning in simpleLoess(y, x, w, span, degree = degree, parametric = parametric, : There are other near singularities as well. 1.01
```

```
## Warning in simpleLoess(y, x, w, span, degree = degree, parametric = parametric, : zero-width neighborhood. make span bigger
```

```
## Warning: Computation failed in `stat_smooth()`:
## NA/NaN/Inf in foreign function call (arg 5)
```

```
## Warning in simpleLoess(y, x, w, span, degree = degree, parametric = parametric, : at -0.005
```

```
## Warning in simpleLoess(y, x, w, span, degree = degree, parametric = parametric, : radius 2.5e-05
```

```
## Warning in simpleLoess(y, x, w, span, degree = degree, parametric = parametric, : all data on boundary of neighborhood. make span bigger
```

```
## Warning in simpleLoess(y, x, w, span, degree = degree, parametric = parametric, : pseudoinverse used at -0.005
```

```
## Warning in simpleLoess(y, x, w, span, degree = degree, parametric = parametric, : neighborhood radius 0.005
```

```
## Warning in simpleLoess(y, x, w, span, degree = degree, parametric = parametric, : reciprocal condition number 1
```

```
## Warning in simpleLoess(y, x, w, span, degree = degree, parametric = parametric, : There are other near singularities as well. 1.01
```

```
## Warning in simpleLoess(y, x, w, span, degree = degree, parametric = parametric, : zero-width neighborhood. make span bigger
```

```
## Warning: Computation failed in `stat_smooth()`:
## NA/NaN/Inf in foreign function call (arg 5)
```

```
## Warning in simpleLoess(y, x, w, span, degree = degree, parametric = parametric, : at -0.005
```

```
## Warning in simpleLoess(y, x, w, span, degree = degree, parametric = parametric, : radius 2.5e-05
```

```
## Warning in simpleLoess(y, x, w, span, degree = degree, parametric = parametric, : all data on boundary of neighborhood. make span bigger
```

```
## Warning in simpleLoess(y, x, w, span, degree = degree, parametric = parametric, : pseudoinverse used at -0.005
```

```
## Warning in simpleLoess(y, x, w, span, degree = degree, parametric = parametric, : neighborhood radius 0.005
```

```
## Warning in simpleLoess(y, x, w, span, degree = degree, parametric = parametric, : reciprocal condition number 1
```

```
## Warning in simpleLoess(y, x, w, span, degree = degree, parametric = parametric, : There are other near singularities as well. 1.01
```

```
## Warning in simpleLoess(y, x, w, span, degree = degree, parametric = parametric, : zero-width neighborhood. make span bigger
```

```
## Warning: Computation failed in `stat_smooth()`:
## NA/NaN/Inf in foreign function call (arg 5)
```

```
## Warning in simpleLoess(y, x, w, span, degree = degree, parametric = parametric, : at -0.005
```

```
## Warning in simpleLoess(y, x, w, span, degree = degree, parametric = parametric, : radius 2.5e-05
```

```
## Warning in simpleLoess(y, x, w, span, degree = degree, parametric = parametric, : all data on boundary of neighborhood. make span bigger
```

```
## Warning in simpleLoess(y, x, w, span, degree = degree, parametric = parametric, : pseudoinverse used at -0.005
```

```
## Warning in simpleLoess(y, x, w, span, degree = degree, parametric = parametric, : neighborhood radius 0.005
```

```
## Warning in simpleLoess(y, x, w, span, degree = degree, parametric = parametric, : reciprocal condition number 1
```

```
## Warning in simpleLoess(y, x, w, span, degree = degree, parametric = parametric, : There are other near singularities as well. 1.01
```

```
## Warning in simpleLoess(y, x, w, span, degree = degree, parametric = parametric, : zero-width neighborhood. make span bigger
```

```
## Warning: Computation failed in `stat_smooth()`:
## NA/NaN/Inf in foreign function call (arg 5)
```

```
## Warning in simpleLoess(y, x, w, span, degree = degree, parametric = parametric, : at -0.005
```

```
## Warning in simpleLoess(y, x, w, span, degree = degree, parametric = parametric, : radius 2.5e-05
```

```
## Warning in simpleLoess(y, x, w, span, degree = degree, parametric = parametric, : all data on boundary of neighborhood. make span bigger
```

```
## Warning in simpleLoess(y, x, w, span, degree = degree, parametric = parametric, : pseudoinverse used at -0.005
```

```
## Warning in simpleLoess(y, x, w, span, degree = degree, parametric = parametric, : neighborhood radius 0.005
```

```
## Warning in simpleLoess(y, x, w, span, degree = degree, parametric = parametric, : reciprocal condition number 1
```

```
## Warning in simpleLoess(y, x, w, span, degree = degree, parametric = parametric, : There are other near singularities as well. 1.01
```

```
## Warning in simpleLoess(y, x, w, span, degree = degree, parametric = parametric, : zero-width neighborhood. make span bigger
```

```
## Warning: Computation failed in `stat_smooth()`:
## NA/NaN/Inf in foreign function call (arg 5)
```

```
## Warning in simpleLoess(y, x, w, span, degree = degree, parametric = parametric, : at -0.005
```

```
## Warning in simpleLoess(y, x, w, span, degree = degree, parametric = parametric, : radius 2.5e-05
```

```
## Warning in simpleLoess(y, x, w, span, degree = degree, parametric = parametric, : all data on boundary of neighborhood. make span bigger
```

```
## Warning in simpleLoess(y, x, w, span, degree = degree, parametric = parametric, : pseudoinverse used at -0.005
```

```
## Warning in simpleLoess(y, x, w, span, degree = degree, parametric = parametric, : neighborhood radius 0.005
```

```
## Warning in simpleLoess(y, x, w, span, degree = degree, parametric = parametric, : reciprocal condition number 1
```

```
## Warning in simpleLoess(y, x, w, span, degree = degree, parametric = parametric, : There are other near singularities as well. 1.01
```

```
## Warning in simpleLoess(y, x, w, span, degree = degree, parametric = parametric, : zero-width neighborhood. make span bigger
```

```
## Warning: Computation failed in `stat_smooth()`:
## NA/NaN/Inf in foreign function call (arg 5)
```

```
## Warning in simpleLoess(y, x, w, span, degree = degree, parametric = parametric, : at -0.005
```

```
## Warning in simpleLoess(y, x, w, span, degree = degree, parametric = parametric, : radius 2.5e-05
```

```
## Warning in simpleLoess(y, x, w, span, degree = degree, parametric = parametric, : all data on boundary of neighborhood. make span bigger
```

```
## Warning in simpleLoess(y, x, w, span, degree = degree, parametric = parametric, : pseudoinverse used at -0.005
```

```
## Warning in simpleLoess(y, x, w, span, degree = degree, parametric = parametric, : neighborhood radius 0.005
```

```
## Warning in simpleLoess(y, x, w, span, degree = degree, parametric = parametric, : reciprocal condition number 1
```

```
## Warning in simpleLoess(y, x, w, span, degree = degree, parametric = parametric, : There are other near singularities as well. 1.01
```

```
## Warning in simpleLoess(y, x, w, span, degree = degree, parametric = parametric, : zero-width neighborhood. make span bigger
```

```
## Warning: Computation failed in `stat_smooth()`:
## NA/NaN/Inf in foreign function call (arg 5)
```

```
## Warning in simpleLoess(y, x, w, span, degree = degree, parametric = parametric, : at -0.005
```

```
## Warning in simpleLoess(y, x, w, span, degree = degree, parametric = parametric, : radius 2.5e-05
```

```
## Warning in simpleLoess(y, x, w, span, degree = degree, parametric = parametric, : all data on boundary of neighborhood. make span bigger
```

```
## Warning in simpleLoess(y, x, w, span, degree = degree, parametric = parametric, : pseudoinverse used at -0.005
```

```
## Warning in simpleLoess(y, x, w, span, degree = degree, parametric = parametric, : neighborhood radius 0.005
```

```
## Warning in simpleLoess(y, x, w, span, degree = degree, parametric = parametric, : reciprocal condition number 1
```

```
## Warning in simpleLoess(y, x, w, span, degree = degree, parametric = parametric, : There are other near singularities as well. 1.01
```

```
## Warning in simpleLoess(y, x, w, span, degree = degree, parametric = parametric, : zero-width neighborhood. make span bigger
```

```
## Warning: Computation failed in `stat_smooth()`:
## NA/NaN/Inf in foreign function call (arg 5)
```

```
## Warning in simpleLoess(y, x, w, span, degree = degree, parametric = parametric, : at -0.005
```

```
## Warning in simpleLoess(y, x, w, span, degree = degree, parametric = parametric, : radius 2.5e-05
```

```
## Warning in simpleLoess(y, x, w, span, degree = degree, parametric = parametric, : all data on boundary of neighborhood. make span bigger
```

```
## Warning in simpleLoess(y, x, w, span, degree = degree, parametric = parametric, : pseudoinverse used at -0.005
```

```
## Warning in simpleLoess(y, x, w, span, degree = degree, parametric = parametric, : neighborhood radius 0.005
```

```
## Warning in simpleLoess(y, x, w, span, degree = degree, parametric = parametric, : reciprocal condition number 1
```

```
## Warning in simpleLoess(y, x, w, span, degree = degree, parametric = parametric, : There are other near singularities as well. 1.01
```

```
## Warning in simpleLoess(y, x, w, span, degree = degree, parametric = parametric, : zero-width neighborhood. make span bigger
```

```
## Warning: Computation failed in `stat_smooth()`:
## NA/NaN/Inf in foreign function call (arg 5)
```

```
## Warning in simpleLoess(y, x, w, span, degree = degree, parametric = parametric, : at -0.005
```

```
## Warning in simpleLoess(y, x, w, span, degree = degree, parametric = parametric, : radius 2.5e-05
```

```
## Warning in simpleLoess(y, x, w, span, degree = degree, parametric = parametric, : all data on boundary of neighborhood. make span bigger
```

```
## Warning in simpleLoess(y, x, w, span, degree = degree, parametric = parametric, : pseudoinverse used at -0.005
```

```
## Warning in simpleLoess(y, x, w, span, degree = degree, parametric = parametric, : neighborhood radius 0.005
```

```
## Warning in simpleLoess(y, x, w, span, degree = degree, parametric = parametric, : reciprocal condition number 1
```

```
## Warning in simpleLoess(y, x, w, span, degree = degree, parametric = parametric, : There are other near singularities as well. 1.01
```

```
## Warning in simpleLoess(y, x, w, span, degree = degree, parametric = parametric, : zero-width neighborhood. make span bigger
```

```
## Warning: Computation failed in `stat_smooth()`:
## NA/NaN/Inf in foreign function call (arg 5)
```

```
## Warning in simpleLoess(y, x, w, span, degree = degree, parametric = parametric, : at -0.005
```

```
## Warning in simpleLoess(y, x, w, span, degree = degree, parametric = parametric, : radius 2.5e-05
```

```
## Warning in simpleLoess(y, x, w, span, degree = degree, parametric = parametric, : all data on boundary of neighborhood. make span bigger
```

```
## Warning in simpleLoess(y, x, w, span, degree = degree, parametric = parametric, : pseudoinverse used at -0.005
```

```
## Warning in simpleLoess(y, x, w, span, degree = degree, parametric = parametric, : neighborhood radius 0.005
```

```
## Warning in simpleLoess(y, x, w, span, degree = degree, parametric = parametric, : reciprocal condition number 1
```

```
## Warning in simpleLoess(y, x, w, span, degree = degree, parametric = parametric, : There are other near singularities as well. 1.01
```

```
## Warning in simpleLoess(y, x, w, span, degree = degree, parametric = parametric, : zero-width neighborhood. make span bigger
```

```
## Warning: Computation failed in `stat_smooth()`:
## NA/NaN/Inf in foreign function call (arg 5)
```

```
## Warning in simpleLoess(y, x, w, span, degree = degree, parametric = parametric, : at -0.005
```

```
## Warning in simpleLoess(y, x, w, span, degree = degree, parametric = parametric, : radius 2.5e-05
```

```
## Warning in simpleLoess(y, x, w, span, degree = degree, parametric = parametric, : all data on boundary of neighborhood. make span bigger
```

```
## Warning in simpleLoess(y, x, w, span, degree = degree, parametric = parametric, : pseudoinverse used at -0.005
```

```
## Warning in simpleLoess(y, x, w, span, degree = degree, parametric = parametric, : neighborhood radius 0.005
```

```
## Warning in simpleLoess(y, x, w, span, degree = degree, parametric = parametric, : reciprocal condition number 1
```

```
## Warning in simpleLoess(y, x, w, span, degree = degree, parametric = parametric, : There are other near singularities as well. 1.01
```

```
## Warning in simpleLoess(y, x, w, span, degree = degree, parametric = parametric, : zero-width neighborhood. make span bigger
```

```
## Warning: Computation failed in `stat_smooth()`:
## NA/NaN/Inf in foreign function call (arg 5)
```

```
## Warning in simpleLoess(y, x, w, span, degree = degree, parametric = parametric, : pseudoinverse used at -0.005
```

```
## Warning in simpleLoess(y, x, w, span, degree = degree, parametric = parametric, : neighborhood radius 1.005
```

```
## Warning in simpleLoess(y, x, w, span, degree = degree, parametric = parametric, : reciprocal condition number 0
```

```
## Warning in simpleLoess(y, x, w, span, degree = degree, parametric = parametric, : There are other near singularities as well. 1.01
```

```
## Warning in predLoess(object$y, object$x, newx = if (is.null(newdata)) object$x else if (is.data.frame(newdata)) as.matrix(model.frame(delete.response(terms(object)), : pseudoinverse used at -0.005
```

```
## Warning in predLoess(object$y, object$x, newx = if (is.null(newdata)) object$x else if (is.data.frame(newdata)) as.matrix(model.frame(delete.response(terms(object)), : neighborhood radius 1.005
```

```
## Warning in predLoess(object$y, object$x, newx = if (is.null(newdata)) object$x else if (is.data.frame(newdata)) as.matrix(model.frame(delete.response(terms(object)), : reciprocal condition number 0
```

```
## Warning in predLoess(object$y, object$x, newx = if (is.null(newdata)) object$x else if (is.data.frame(newdata)) as.matrix(model.frame(delete.response(terms(object)), : There are other near
## singularities as well. 1.01
```

```
## Warning in simpleLoess(y, x, w, span, degree = degree, parametric = parametric, : at -0.005
```

```
## Warning in simpleLoess(y, x, w, span, degree = degree, parametric = parametric, : radius 2.5e-05
```

```
## Warning in simpleLoess(y, x, w, span, degree = degree, parametric = parametric, : all data on boundary of neighborhood. make span bigger
```

```
## Warning in simpleLoess(y, x, w, span, degree = degree, parametric = parametric, : pseudoinverse used at -0.005
```

```
## Warning in simpleLoess(y, x, w, span, degree = degree, parametric = parametric, : neighborhood radius 0.005
```

```
## Warning in simpleLoess(y, x, w, span, degree = degree, parametric = parametric, : reciprocal condition number 1
```

```
## Warning in simpleLoess(y, x, w, span, degree = degree, parametric = parametric, : There are other near singularities as well. 1.01
```

```
## Warning in simpleLoess(y, x, w, span, degree = degree, parametric = parametric, : zero-width neighborhood. make span bigger
```

```
## Warning: Computation failed in `stat_smooth()`:
## NA/NaN/Inf in foreign function call (arg 5)
```

```
## Warning in simpleLoess(y, x, w, span, degree = degree, parametric = parametric, : at -0.005
```

```
## Warning in simpleLoess(y, x, w, span, degree = degree, parametric = parametric, : radius 2.5e-05
```

```
## Warning in simpleLoess(y, x, w, span, degree = degree, parametric = parametric, : all data on boundary of neighborhood. make span bigger
```

```
## Warning in simpleLoess(y, x, w, span, degree = degree, parametric = parametric, : pseudoinverse used at -0.005
```

```
## Warning in simpleLoess(y, x, w, span, degree = degree, parametric = parametric, : neighborhood radius 0.005
```

```
## Warning in simpleLoess(y, x, w, span, degree = degree, parametric = parametric, : reciprocal condition number 1
```

```
## Warning in simpleLoess(y, x, w, span, degree = degree, parametric = parametric, : There are other near singularities as well. 1.01
```

```
## Warning in simpleLoess(y, x, w, span, degree = degree, parametric = parametric, : zero-width neighborhood. make span bigger
```

```
## Warning: Computation failed in `stat_smooth()`:
## NA/NaN/Inf in foreign function call (arg 5)
```

```
## Warning in simpleLoess(y, x, w, span, degree = degree, parametric = parametric, : at -0.005
```

```
## Warning in simpleLoess(y, x, w, span, degree = degree, parametric = parametric, : radius 2.5e-05
```

```
## Warning in simpleLoess(y, x, w, span, degree = degree, parametric = parametric, : all data on boundary of neighborhood. make span bigger
```

```
## Warning in simpleLoess(y, x, w, span, degree = degree, parametric = parametric, : pseudoinverse used at -0.005
```

```
## Warning in simpleLoess(y, x, w, span, degree = degree, parametric = parametric, : neighborhood radius 0.005
```

```
## Warning in simpleLoess(y, x, w, span, degree = degree, parametric = parametric, : reciprocal condition number 1
```

```
## Warning in simpleLoess(y, x, w, span, degree = degree, parametric = parametric, : There are other near singularities as well. 1.01
```

```
## Warning in simpleLoess(y, x, w, span, degree = degree, parametric = parametric, : zero-width neighborhood. make span bigger
```

```
## Warning: Computation failed in `stat_smooth()`:
## NA/NaN/Inf in foreign function call (arg 5)
```

```
## Warning in simpleLoess(y, x, w, span, degree = degree, parametric = parametric, : at -0.005
```

```
## Warning in simpleLoess(y, x, w, span, degree = degree, parametric = parametric, : radius 2.5e-05
```

```
## Warning in simpleLoess(y, x, w, span, degree = degree, parametric = parametric, : all data on boundary of neighborhood. make span bigger
```

```
## Warning in simpleLoess(y, x, w, span, degree = degree, parametric = parametric, : pseudoinverse used at -0.005
```

```
## Warning in simpleLoess(y, x, w, span, degree = degree, parametric = parametric, : neighborhood radius 0.005
```

```
## Warning in simpleLoess(y, x, w, span, degree = degree, parametric = parametric, : reciprocal condition number 1
```

```
## Warning in simpleLoess(y, x, w, span, degree = degree, parametric = parametric, : There are other near singularities as well. 1.01
```

```
## Warning in simpleLoess(y, x, w, span, degree = degree, parametric = parametric, : zero-width neighborhood. make span bigger
```

```
## Warning: Computation failed in `stat_smooth()`:
## NA/NaN/Inf in foreign function call (arg 5)
```

```
## Warning in simpleLoess(y, x, w, span, degree = degree, parametric = parametric, : at -0.005
```

```
## Warning in simpleLoess(y, x, w, span, degree = degree, parametric = parametric, : radius 2.5e-05
```

```
## Warning in simpleLoess(y, x, w, span, degree = degree, parametric = parametric, : all data on boundary of neighborhood. make span bigger
```

```
## Warning in simpleLoess(y, x, w, span, degree = degree, parametric = parametric, : pseudoinverse used at -0.005
```

```
## Warning in simpleLoess(y, x, w, span, degree = degree, parametric = parametric, : neighborhood radius 0.005
```

```
## Warning in simpleLoess(y, x, w, span, degree = degree, parametric = parametric, : reciprocal condition number 1
```

```
## Warning in simpleLoess(y, x, w, span, degree = degree, parametric = parametric, : There are other near singularities as well. 1.01
```

```
## Warning in simpleLoess(y, x, w, span, degree = degree, parametric = parametric, : zero-width neighborhood. make span bigger
```

```
## Warning: Computation failed in `stat_smooth()`:
## NA/NaN/Inf in foreign function call (arg 5)
```

```
## Warning in simpleLoess(y, x, w, span, degree = degree, parametric = parametric, : at -0.005
```

```
## Warning in simpleLoess(y, x, w, span, degree = degree, parametric = parametric, : radius 2.5e-05
```

```
## Warning in simpleLoess(y, x, w, span, degree = degree, parametric = parametric, : all data on boundary of neighborhood. make span bigger
```

```
## Warning in simpleLoess(y, x, w, span, degree = degree, parametric = parametric, : pseudoinverse used at -0.005
```

```
## Warning in simpleLoess(y, x, w, span, degree = degree, parametric = parametric, : neighborhood radius 0.005
```

```
## Warning in simpleLoess(y, x, w, span, degree = degree, parametric = parametric, : reciprocal condition number 1
```

```
## Warning in simpleLoess(y, x, w, span, degree = degree, parametric = parametric, : There are other near singularities as well. 1.01
```

```
## Warning in simpleLoess(y, x, w, span, degree = degree, parametric = parametric, : zero-width neighborhood. make span bigger
```

```
## Warning: Computation failed in `stat_smooth()`:
## NA/NaN/Inf in foreign function call (arg 5)
```

```
## Warning in simpleLoess(y, x, w, span, degree = degree, parametric = parametric, : at -0.005
```

```
## Warning in simpleLoess(y, x, w, span, degree = degree, parametric = parametric, : radius 2.5e-05
```

```
## Warning in simpleLoess(y, x, w, span, degree = degree, parametric = parametric, : all data on boundary of neighborhood. make span bigger
```

```
## Warning in simpleLoess(y, x, w, span, degree = degree, parametric = parametric, : pseudoinverse used at -0.005
```

```
## Warning in simpleLoess(y, x, w, span, degree = degree, parametric = parametric, : neighborhood radius 0.005
```

```
## Warning in simpleLoess(y, x, w, span, degree = degree, parametric = parametric, : reciprocal condition number 1
```

```
## Warning in simpleLoess(y, x, w, span, degree = degree, parametric = parametric, : There are other near singularities as well. 1.01
```

```
## Warning in simpleLoess(y, x, w, span, degree = degree, parametric = parametric, : zero-width neighborhood. make span bigger
```

```
## Warning: Computation failed in `stat_smooth()`:
## NA/NaN/Inf in foreign function call (arg 5)
```

```
## Warning in simpleLoess(y, x, w, span, degree = degree, parametric = parametric, : at -0.005
```

```
## Warning in simpleLoess(y, x, w, span, degree = degree, parametric = parametric, : radius 2.5e-05
```

```
## Warning in simpleLoess(y, x, w, span, degree = degree, parametric = parametric, : all data on boundary of neighborhood. make span bigger
```

```
## Warning in simpleLoess(y, x, w, span, degree = degree, parametric = parametric, : pseudoinverse used at -0.005
```

```
## Warning in simpleLoess(y, x, w, span, degree = degree, parametric = parametric, : neighborhood radius 0.005
```

```
## Warning in simpleLoess(y, x, w, span, degree = degree, parametric = parametric, : reciprocal condition number 1
```

```
## Warning in simpleLoess(y, x, w, span, degree = degree, parametric = parametric, : There are other near singularities as well. 1.01
```

```
## Warning in simpleLoess(y, x, w, span, degree = degree, parametric = parametric, : zero-width neighborhood. make span bigger
```

```
## Warning: Computation failed in `stat_smooth()`:
## NA/NaN/Inf in foreign function call (arg 5)
```

```
## Warning in simpleLoess(y, x, w, span, degree = degree, parametric = parametric, : at -0.005
```

```
## Warning in simpleLoess(y, x, w, span, degree = degree, parametric = parametric, : radius 2.5e-05
```

```
## Warning in simpleLoess(y, x, w, span, degree = degree, parametric = parametric, : all data on boundary of neighborhood. make span bigger
```

```
## Warning in simpleLoess(y, x, w, span, degree = degree, parametric = parametric, : pseudoinverse used at -0.005
```

```
## Warning in simpleLoess(y, x, w, span, degree = degree, parametric = parametric, : neighborhood radius 0.005
```

```
## Warning in simpleLoess(y, x, w, span, degree = degree, parametric = parametric, : reciprocal condition number 1
```

```
## Warning in simpleLoess(y, x, w, span, degree = degree, parametric = parametric, : There are other near singularities as well. 1.01
```

```
## Warning in simpleLoess(y, x, w, span, degree = degree, parametric = parametric, : zero-width neighborhood. make span bigger
```

```
## Warning: Computation failed in `stat_smooth()`:
## NA/NaN/Inf in foreign function call (arg 5)
```

```
## Warning in simpleLoess(y, x, w, span, degree = degree, parametric = parametric, : at -0.005
```

```
## Warning in simpleLoess(y, x, w, span, degree = degree, parametric = parametric, : radius 2.5e-05
```

```
## Warning in simpleLoess(y, x, w, span, degree = degree, parametric = parametric, : all data on boundary of neighborhood. make span bigger
```

```
## Warning in simpleLoess(y, x, w, span, degree = degree, parametric = parametric, : pseudoinverse used at -0.005
```

```
## Warning in simpleLoess(y, x, w, span, degree = degree, parametric = parametric, : neighborhood radius 0.005
```

```
## Warning in simpleLoess(y, x, w, span, degree = degree, parametric = parametric, : reciprocal condition number 1
```

```
## Warning in simpleLoess(y, x, w, span, degree = degree, parametric = parametric, : There are other near singularities as well. 1.01
```

```
## Warning in simpleLoess(y, x, w, span, degree = degree, parametric = parametric, : zero-width neighborhood. make span bigger
```

```
## Warning: Computation failed in `stat_smooth()`:
## NA/NaN/Inf in foreign function call (arg 5)
```

```
## Warning in simpleLoess(y, x, w, span, degree = degree, parametric = parametric, : at -0.005
```

```
## Warning in simpleLoess(y, x, w, span, degree = degree, parametric = parametric, : radius 2.5e-05
```

```
## Warning in simpleLoess(y, x, w, span, degree = degree, parametric = parametric, : all data on boundary of neighborhood. make span bigger
```

```
## Warning in simpleLoess(y, x, w, span, degree = degree, parametric = parametric, : pseudoinverse used at -0.005
```

```
## Warning in simpleLoess(y, x, w, span, degree = degree, parametric = parametric, : neighborhood radius 0.005
```

```
## Warning in simpleLoess(y, x, w, span, degree = degree, parametric = parametric, : reciprocal condition number 1
```

```
## Warning in simpleLoess(y, x, w, span, degree = degree, parametric = parametric, : There are other near singularities as well. 1.01
```

```
## Warning in simpleLoess(y, x, w, span, degree = degree, parametric = parametric, : zero-width neighborhood. make span bigger
```

```
## Warning: Computation failed in `stat_smooth()`:
## NA/NaN/Inf in foreign function call (arg 5)
```

```
## Warning in simpleLoess(y, x, w, span, degree = degree, parametric = parametric, : at -0.005
```

```
## Warning in simpleLoess(y, x, w, span, degree = degree, parametric = parametric, : radius 2.5e-05
```

```
## Warning in simpleLoess(y, x, w, span, degree = degree, parametric = parametric, : all data on boundary of neighborhood. make span bigger
```

```
## Warning in simpleLoess(y, x, w, span, degree = degree, parametric = parametric, : pseudoinverse used at -0.005
```

```
## Warning in simpleLoess(y, x, w, span, degree = degree, parametric = parametric, : neighborhood radius 0.005
```

```
## Warning in simpleLoess(y, x, w, span, degree = degree, parametric = parametric, : reciprocal condition number 1
```

```
## Warning in simpleLoess(y, x, w, span, degree = degree, parametric = parametric, : There are other near singularities as well. 1.01
```

```
## Warning in simpleLoess(y, x, w, span, degree = degree, parametric = parametric, : zero-width neighborhood. make span bigger
```

```
## Warning: Computation failed in `stat_smooth()`:
## NA/NaN/Inf in foreign function call (arg 5)
```

```
## Warning in simpleLoess(y, x, w, span, degree = degree, parametric = parametric, : at -0.005
```

```
## Warning in simpleLoess(y, x, w, span, degree = degree, parametric = parametric, : radius 2.5e-05
```

```
## Warning in simpleLoess(y, x, w, span, degree = degree, parametric = parametric, : all data on boundary of neighborhood. make span bigger
```

```
## Warning in simpleLoess(y, x, w, span, degree = degree, parametric = parametric, : pseudoinverse used at -0.005
```

```
## Warning in simpleLoess(y, x, w, span, degree = degree, parametric = parametric, : neighborhood radius 0.005
```

```
## Warning in simpleLoess(y, x, w, span, degree = degree, parametric = parametric, : reciprocal condition number 1
```

```
## Warning in simpleLoess(y, x, w, span, degree = degree, parametric = parametric, : There are other near singularities as well. 1.01
```

```
## Warning in simpleLoess(y, x, w, span, degree = degree, parametric = parametric, : zero-width neighborhood. make span bigger
```

```
## Warning: Computation failed in `stat_smooth()`:
## NA/NaN/Inf in foreign function call (arg 5)
```

```
## Warning in simpleLoess(y, x, w, span, degree = degree, parametric = parametric, : at -0.005
```

```
## Warning in simpleLoess(y, x, w, span, degree = degree, parametric = parametric, : radius 2.5e-05
```

```
## Warning in simpleLoess(y, x, w, span, degree = degree, parametric = parametric, : all data on boundary of neighborhood. make span bigger
```

```
## Warning in simpleLoess(y, x, w, span, degree = degree, parametric = parametric, : pseudoinverse used at -0.005
```

```
## Warning in simpleLoess(y, x, w, span, degree = degree, parametric = parametric, : neighborhood radius 0.005
```

```
## Warning in simpleLoess(y, x, w, span, degree = degree, parametric = parametric, : reciprocal condition number 1
```

```
## Warning in simpleLoess(y, x, w, span, degree = degree, parametric = parametric, : There are other near singularities as well. 1.01
```

```
## Warning in simpleLoess(y, x, w, span, degree = degree, parametric = parametric, : zero-width neighborhood. make span bigger
```

```
## Warning: Computation failed in `stat_smooth()`:
## NA/NaN/Inf in foreign function call (arg 5)
```

```
## Warning in simpleLoess(y, x, w, span, degree = degree, parametric = parametric, : at -0.005
```

```
## Warning in simpleLoess(y, x, w, span, degree = degree, parametric = parametric, : radius 2.5e-05
```

```
## Warning in simpleLoess(y, x, w, span, degree = degree, parametric = parametric, : all data on boundary of neighborhood. make span bigger
```

```
## Warning in simpleLoess(y, x, w, span, degree = degree, parametric = parametric, : pseudoinverse used at -0.005
```

```
## Warning in simpleLoess(y, x, w, span, degree = degree, parametric = parametric, : neighborhood radius 0.005
```

```
## Warning in simpleLoess(y, x, w, span, degree = degree, parametric = parametric, : reciprocal condition number 1
```

```
## Warning in simpleLoess(y, x, w, span, degree = degree, parametric = parametric, : There are other near singularities as well. 1.01
```

```
## Warning in simpleLoess(y, x, w, span, degree = degree, parametric = parametric, : zero-width neighborhood. make span bigger
```

```
## Warning: Computation failed in `stat_smooth()`:
## NA/NaN/Inf in foreign function call (arg 5)
```

```
## Warning in simpleLoess(y, x, w, span, degree = degree, parametric = parametric, : at -0.005
```

```
## Warning in simpleLoess(y, x, w, span, degree = degree, parametric = parametric, : radius 2.5e-05
```

```
## Warning in simpleLoess(y, x, w, span, degree = degree, parametric = parametric, : all data on boundary of neighborhood. make span bigger
```

```
## Warning in simpleLoess(y, x, w, span, degree = degree, parametric = parametric, : pseudoinverse used at -0.005
```

```
## Warning in simpleLoess(y, x, w, span, degree = degree, parametric = parametric, : neighborhood radius 0.005
```

```
## Warning in simpleLoess(y, x, w, span, degree = degree, parametric = parametric, : reciprocal condition number 1
```

```
## Warning in simpleLoess(y, x, w, span, degree = degree, parametric = parametric, : There are other near singularities as well. 1.01
```

```
## Warning in simpleLoess(y, x, w, span, degree = degree, parametric = parametric, : zero-width neighborhood. make span bigger
```

```
## Warning: Computation failed in `stat_smooth()`:
## NA/NaN/Inf in foreign function call (arg 5)
```

```
## Warning in simpleLoess(y, x, w, span, degree = degree, parametric = parametric, : at -0.005
```

```
## Warning in simpleLoess(y, x, w, span, degree = degree, parametric = parametric, : radius 2.5e-05
```

```
## Warning in simpleLoess(y, x, w, span, degree = degree, parametric = parametric, : all data on boundary of neighborhood. make span bigger
```

```
## Warning in simpleLoess(y, x, w, span, degree = degree, parametric = parametric, : pseudoinverse used at -0.005
```

```
## Warning in simpleLoess(y, x, w, span, degree = degree, parametric = parametric, : neighborhood radius 0.005
```

```
## Warning in simpleLoess(y, x, w, span, degree = degree, parametric = parametric, : reciprocal condition number 1
```

```
## Warning in simpleLoess(y, x, w, span, degree = degree, parametric = parametric, : There are other near singularities as well. 1.01
```

```
## Warning in simpleLoess(y, x, w, span, degree = degree, parametric = parametric, : zero-width neighborhood. make span bigger
```

```
## Warning: Computation failed in `stat_smooth()`:
## NA/NaN/Inf in foreign function call (arg 5)
```

```
## Warning in simpleLoess(y, x, w, span, degree = degree, parametric = parametric, : pseudoinverse used at -0.005
```

```
## Warning in simpleLoess(y, x, w, span, degree = degree, parametric = parametric, : neighborhood radius 1.005
```

```
## Warning in simpleLoess(y, x, w, span, degree = degree, parametric = parametric, : reciprocal condition number 0
```

```
## Warning in simpleLoess(y, x, w, span, degree = degree, parametric = parametric, : There are other near singularities as well. 1.01
```

```
## Warning in predLoess(object$y, object$x, newx = if (is.null(newdata)) object$x else if (is.data.frame(newdata)) as.matrix(model.frame(delete.response(terms(object)), : pseudoinverse used at -0.005
```

```
## Warning in predLoess(object$y, object$x, newx = if (is.null(newdata)) object$x else if (is.data.frame(newdata)) as.matrix(model.frame(delete.response(terms(object)), : neighborhood radius 1.005
```

```
## Warning in predLoess(object$y, object$x, newx = if (is.null(newdata)) object$x else if (is.data.frame(newdata)) as.matrix(model.frame(delete.response(terms(object)), : reciprocal condition number 0
```

```
## Warning in predLoess(object$y, object$x, newx = if (is.null(newdata)) object$x else if (is.data.frame(newdata)) as.matrix(model.frame(delete.response(terms(object)), : There are other near
## singularities as well. 1.01
```

```
## Warning in simpleLoess(y, x, w, span, degree = degree, parametric = parametric, : at -0.005
```

```
## Warning in simpleLoess(y, x, w, span, degree = degree, parametric = parametric, : radius 2.5e-05
```

```
## Warning in simpleLoess(y, x, w, span, degree = degree, parametric = parametric, : all data on boundary of neighborhood. make span bigger
```

```
## Warning in simpleLoess(y, x, w, span, degree = degree, parametric = parametric, : pseudoinverse used at -0.005
```

```
## Warning in simpleLoess(y, x, w, span, degree = degree, parametric = parametric, : neighborhood radius 0.005
```

```
## Warning in simpleLoess(y, x, w, span, degree = degree, parametric = parametric, : reciprocal condition number 1
```

```
## Warning in simpleLoess(y, x, w, span, degree = degree, parametric = parametric, : There are other near singularities as well. 1.01
```

```
## Warning in simpleLoess(y, x, w, span, degree = degree, parametric = parametric, : zero-width neighborhood. make span bigger
```

```
## Warning: Computation failed in `stat_smooth()`:
## NA/NaN/Inf in foreign function call (arg 5)
```

```
## Warning in simpleLoess(y, x, w, span, degree = degree, parametric = parametric, : pseudoinverse used at -0.005
```

```
## Warning in simpleLoess(y, x, w, span, degree = degree, parametric = parametric, : neighborhood radius 1.005
```

```
## Warning in simpleLoess(y, x, w, span, degree = degree, parametric = parametric, : reciprocal condition number 0
```

```
## Warning in simpleLoess(y, x, w, span, degree = degree, parametric = parametric, : There are other near singularities as well. 1.01
```

```
## Warning in predLoess(object$y, object$x, newx = if (is.null(newdata)) object$x else if (is.data.frame(newdata)) as.matrix(model.frame(delete.response(terms(object)), : pseudoinverse used at -0.005
```

```
## Warning in predLoess(object$y, object$x, newx = if (is.null(newdata)) object$x else if (is.data.frame(newdata)) as.matrix(model.frame(delete.response(terms(object)), : neighborhood radius 1.005
```

```
## Warning in predLoess(object$y, object$x, newx = if (is.null(newdata)) object$x else if (is.data.frame(newdata)) as.matrix(model.frame(delete.response(terms(object)), : reciprocal condition number 0
```

```
## Warning in predLoess(object$y, object$x, newx = if (is.null(newdata)) object$x else if (is.data.frame(newdata)) as.matrix(model.frame(delete.response(terms(object)), : There are other near
## singularities as well. 1.01
```

```
## Warning in simpleLoess(y, x, w, span, degree = degree, parametric = parametric, : at -0.005
```

```
## Warning in simpleLoess(y, x, w, span, degree = degree, parametric = parametric, : radius 2.5e-05
```

```
## Warning in simpleLoess(y, x, w, span, degree = degree, parametric = parametric, : all data on boundary of neighborhood. make span bigger
```

```
## Warning in simpleLoess(y, x, w, span, degree = degree, parametric = parametric, : pseudoinverse used at -0.005
```

```
## Warning in simpleLoess(y, x, w, span, degree = degree, parametric = parametric, : neighborhood radius 0.005
```

```
## Warning in simpleLoess(y, x, w, span, degree = degree, parametric = parametric, : reciprocal condition number 1
```

```
## Warning in simpleLoess(y, x, w, span, degree = degree, parametric = parametric, : There are other near singularities as well. 1.01
```

```
## Warning in simpleLoess(y, x, w, span, degree = degree, parametric = parametric, : zero-width neighborhood. make span bigger
```

```
## Warning: Computation failed in `stat_smooth()`:
## NA/NaN/Inf in foreign function call (arg 5)
```

```
## Warning in simpleLoess(y, x, w, span, degree = degree, parametric = parametric, : at -0.005
```

```
## Warning in simpleLoess(y, x, w, span, degree = degree, parametric = parametric, : radius 2.5e-05
```

```
## Warning in simpleLoess(y, x, w, span, degree = degree, parametric = parametric, : all data on boundary of neighborhood. make span bigger
```

```
## Warning in simpleLoess(y, x, w, span, degree = degree, parametric = parametric, : pseudoinverse used at -0.005
```

```
## Warning in simpleLoess(y, x, w, span, degree = degree, parametric = parametric, : neighborhood radius 0.005
```

```
## Warning in simpleLoess(y, x, w, span, degree = degree, parametric = parametric, : reciprocal condition number 1
```

```
## Warning in simpleLoess(y, x, w, span, degree = degree, parametric = parametric, : There are other near singularities as well. 1.01
```

```
## Warning in simpleLoess(y, x, w, span, degree = degree, parametric = parametric, : zero-width neighborhood. make span bigger
```

```
## Warning: Computation failed in `stat_smooth()`:
## NA/NaN/Inf in foreign function call (arg 5)
```

```
## Warning in simpleLoess(y, x, w, span, degree = degree, parametric = parametric, : at -0.005
```

```
## Warning in simpleLoess(y, x, w, span, degree = degree, parametric = parametric, : radius 2.5e-05
```

```
## Warning in simpleLoess(y, x, w, span, degree = degree, parametric = parametric, : all data on boundary of neighborhood. make span bigger
```

```
## Warning in simpleLoess(y, x, w, span, degree = degree, parametric = parametric, : pseudoinverse used at -0.005
```

```
## Warning in simpleLoess(y, x, w, span, degree = degree, parametric = parametric, : neighborhood radius 0.005
```

```
## Warning in simpleLoess(y, x, w, span, degree = degree, parametric = parametric, : reciprocal condition number 1
```

```
## Warning in simpleLoess(y, x, w, span, degree = degree, parametric = parametric, : There are other near singularities as well. 1.01
```

```
## Warning in simpleLoess(y, x, w, span, degree = degree, parametric = parametric, : zero-width neighborhood. make span bigger
```

```
## Warning: Computation failed in `stat_smooth()`:
## NA/NaN/Inf in foreign function call (arg 5)
```

```
## Warning in simpleLoess(y, x, w, span, degree = degree, parametric = parametric, : at -0.005
```

```
## Warning in simpleLoess(y, x, w, span, degree = degree, parametric = parametric, : radius 2.5e-05
```

```
## Warning in simpleLoess(y, x, w, span, degree = degree, parametric = parametric, : all data on boundary of neighborhood. make span bigger
```

```
## Warning in simpleLoess(y, x, w, span, degree = degree, parametric = parametric, : pseudoinverse used at -0.005
```

```
## Warning in simpleLoess(y, x, w, span, degree = degree, parametric = parametric, : neighborhood radius 0.005
```

```
## Warning in simpleLoess(y, x, w, span, degree = degree, parametric = parametric, : reciprocal condition number 1
```

```
## Warning in simpleLoess(y, x, w, span, degree = degree, parametric = parametric, : There are other near singularities as well. 1.01
```

```
## Warning in simpleLoess(y, x, w, span, degree = degree, parametric = parametric, : zero-width neighborhood. make span bigger
```

```
## Warning: Computation failed in `stat_smooth()`:
## NA/NaN/Inf in foreign function call (arg 5)
```

```
## Warning in simpleLoess(y, x, w, span, degree = degree, parametric = parametric, : at -0.005
```

```
## Warning in simpleLoess(y, x, w, span, degree = degree, parametric = parametric, : radius 2.5e-05
```

```
## Warning in simpleLoess(y, x, w, span, degree = degree, parametric = parametric, : all data on boundary of neighborhood. make span bigger
```

```
## Warning in simpleLoess(y, x, w, span, degree = degree, parametric = parametric, : pseudoinverse used at -0.005
```

```
## Warning in simpleLoess(y, x, w, span, degree = degree, parametric = parametric, : neighborhood radius 0.005
```

```
## Warning in simpleLoess(y, x, w, span, degree = degree, parametric = parametric, : reciprocal condition number 1
```

```
## Warning in simpleLoess(y, x, w, span, degree = degree, parametric = parametric, : There are other near singularities as well. 1.01
```

```
## Warning in simpleLoess(y, x, w, span, degree = degree, parametric = parametric, : zero-width neighborhood. make span bigger
```

```
## Warning: Computation failed in `stat_smooth()`:
## NA/NaN/Inf in foreign function call (arg 5)
```

```
## Warning in simpleLoess(y, x, w, span, degree = degree, parametric = parametric, : at -0.005
```

```
## Warning in simpleLoess(y, x, w, span, degree = degree, parametric = parametric, : radius 2.5e-05
```

```
## Warning in simpleLoess(y, x, w, span, degree = degree, parametric = parametric, : all data on boundary of neighborhood. make span bigger
```

```
## Warning in simpleLoess(y, x, w, span, degree = degree, parametric = parametric, : pseudoinverse used at -0.005
```

```
## Warning in simpleLoess(y, x, w, span, degree = degree, parametric = parametric, : neighborhood radius 0.005
```

```
## Warning in simpleLoess(y, x, w, span, degree = degree, parametric = parametric, : reciprocal condition number 1
```

```
## Warning in simpleLoess(y, x, w, span, degree = degree, parametric = parametric, : There are other near singularities as well. 1.01
```

```
## Warning in simpleLoess(y, x, w, span, degree = degree, parametric = parametric, : zero-width neighborhood. make span bigger
```

```
## Warning: Computation failed in `stat_smooth()`:
## NA/NaN/Inf in foreign function call (arg 5)
```

```
## Warning in simpleLoess(y, x, w, span, degree = degree, parametric = parametric, : at -0.005
```

```
## Warning in simpleLoess(y, x, w, span, degree = degree, parametric = parametric, : radius 2.5e-05
```

```
## Warning in simpleLoess(y, x, w, span, degree = degree, parametric = parametric, : all data on boundary of neighborhood. make span bigger
```

```
## Warning in simpleLoess(y, x, w, span, degree = degree, parametric = parametric, : pseudoinverse used at -0.005
```

```
## Warning in simpleLoess(y, x, w, span, degree = degree, parametric = parametric, : neighborhood radius 0.005
```

```
## Warning in simpleLoess(y, x, w, span, degree = degree, parametric = parametric, : reciprocal condition number 1
```

```
## Warning in simpleLoess(y, x, w, span, degree = degree, parametric = parametric, : There are other near singularities as well. 1.01
```

```
## Warning in simpleLoess(y, x, w, span, degree = degree, parametric = parametric, : zero-width neighborhood. make span bigger
```

```
## Warning: Computation failed in `stat_smooth()`:
## NA/NaN/Inf in foreign function call (arg 5)
```

```
## Warning in simpleLoess(y, x, w, span, degree = degree, parametric = parametric, : pseudoinverse used at -0.005
```

```
## Warning in simpleLoess(y, x, w, span, degree = degree, parametric = parametric, : neighborhood radius 1.005
```

```
## Warning in simpleLoess(y, x, w, span, degree = degree, parametric = parametric, : reciprocal condition number 0
```

```
## Warning in simpleLoess(y, x, w, span, degree = degree, parametric = parametric, : There are other near singularities as well. 1.01
```

```
## Warning in predLoess(object$y, object$x, newx = if (is.null(newdata)) object$x else if (is.data.frame(newdata)) as.matrix(model.frame(delete.response(terms(object)), : pseudoinverse used at -0.005
```

```
## Warning in predLoess(object$y, object$x, newx = if (is.null(newdata)) object$x else if (is.data.frame(newdata)) as.matrix(model.frame(delete.response(terms(object)), : neighborhood radius 1.005
```

```
## Warning in predLoess(object$y, object$x, newx = if (is.null(newdata)) object$x else if (is.data.frame(newdata)) as.matrix(model.frame(delete.response(terms(object)), : reciprocal condition number 0
```

```
## Warning in predLoess(object$y, object$x, newx = if (is.null(newdata)) object$x else if (is.data.frame(newdata)) as.matrix(model.frame(delete.response(terms(object)), : There are other near
## singularities as well. 1.01
```

```
## Warning in simpleLoess(y, x, w, span, degree = degree, parametric = parametric, : at -0.005
```

```
## Warning in simpleLoess(y, x, w, span, degree = degree, parametric = parametric, : radius 2.5e-05
```

```
## Warning in simpleLoess(y, x, w, span, degree = degree, parametric = parametric, : all data on boundary of neighborhood. make span bigger
```

```
## Warning in simpleLoess(y, x, w, span, degree = degree, parametric = parametric, : pseudoinverse used at -0.005
```

```
## Warning in simpleLoess(y, x, w, span, degree = degree, parametric = parametric, : neighborhood radius 0.005
```

```
## Warning in simpleLoess(y, x, w, span, degree = degree, parametric = parametric, : reciprocal condition number 1
```

```
## Warning in simpleLoess(y, x, w, span, degree = degree, parametric = parametric, : There are other near singularities as well. 1.01
```

```
## Warning in simpleLoess(y, x, w, span, degree = degree, parametric = parametric, : zero-width neighborhood. make span bigger
```

```
## Warning: Computation failed in `stat_smooth()`:
## NA/NaN/Inf in foreign function call (arg 5)
```

```
## Warning in simpleLoess(y, x, w, span, degree = degree, parametric = parametric, : at -0.005
```

```
## Warning in simpleLoess(y, x, w, span, degree = degree, parametric = parametric, : radius 2.5e-05
```

```
## Warning in simpleLoess(y, x, w, span, degree = degree, parametric = parametric, : all data on boundary of neighborhood. make span bigger
```

```
## Warning in simpleLoess(y, x, w, span, degree = degree, parametric = parametric, : pseudoinverse used at -0.005
```

```
## Warning in simpleLoess(y, x, w, span, degree = degree, parametric = parametric, : neighborhood radius 0.005
```

```
## Warning in simpleLoess(y, x, w, span, degree = degree, parametric = parametric, : reciprocal condition number 1
```

```
## Warning in simpleLoess(y, x, w, span, degree = degree, parametric = parametric, : There are other near singularities as well. 1.01
```

```
## Warning in simpleLoess(y, x, w, span, degree = degree, parametric = parametric, : zero-width neighborhood. make span bigger
```

```
## Warning: Computation failed in `stat_smooth()`:
## NA/NaN/Inf in foreign function call (arg 5)
```

```
## Warning in simpleLoess(y, x, w, span, degree = degree, parametric = parametric, : at -0.005
```

```
## Warning in simpleLoess(y, x, w, span, degree = degree, parametric = parametric, : radius 2.5e-05
```

```
## Warning in simpleLoess(y, x, w, span, degree = degree, parametric = parametric, : all data on boundary of neighborhood. make span bigger
```

```
## Warning in simpleLoess(y, x, w, span, degree = degree, parametric = parametric, : pseudoinverse used at -0.005
```

```
## Warning in simpleLoess(y, x, w, span, degree = degree, parametric = parametric, : neighborhood radius 0.005
```

```
## Warning in simpleLoess(y, x, w, span, degree = degree, parametric = parametric, : reciprocal condition number 1
```

```
## Warning in simpleLoess(y, x, w, span, degree = degree, parametric = parametric, : There are other near singularities as well. 1.01
```

```
## Warning in simpleLoess(y, x, w, span, degree = degree, parametric = parametric, : zero-width neighborhood. make span bigger
```

```
## Warning: Computation failed in `stat_smooth()`:
## NA/NaN/Inf in foreign function call (arg 5)
```

```
## Warning in simpleLoess(y, x, w, span, degree = degree, parametric = parametric, : at -0.005
```

```
## Warning in simpleLoess(y, x, w, span, degree = degree, parametric = parametric, : radius 2.5e-05
```

```
## Warning in simpleLoess(y, x, w, span, degree = degree, parametric = parametric, : all data on boundary of neighborhood. make span bigger
```

```
## Warning in simpleLoess(y, x, w, span, degree = degree, parametric = parametric, : pseudoinverse used at -0.005
```

```
## Warning in simpleLoess(y, x, w, span, degree = degree, parametric = parametric, : neighborhood radius 0.005
```

```
## Warning in simpleLoess(y, x, w, span, degree = degree, parametric = parametric, : reciprocal condition number 1
```

```
## Warning in simpleLoess(y, x, w, span, degree = degree, parametric = parametric, : There are other near singularities as well. 1.01
```

```
## Warning in simpleLoess(y, x, w, span, degree = degree, parametric = parametric, : zero-width neighborhood. make span bigger
```

```
## Warning: Computation failed in `stat_smooth()`:
## NA/NaN/Inf in foreign function call (arg 5)
```

```
## Warning in simpleLoess(y, x, w, span, degree = degree, parametric = parametric, : at -0.005
```

```
## Warning in simpleLoess(y, x, w, span, degree = degree, parametric = parametric, : radius 2.5e-05
```

```
## Warning in simpleLoess(y, x, w, span, degree = degree, parametric = parametric, : all data on boundary of neighborhood. make span bigger
```

```
## Warning in simpleLoess(y, x, w, span, degree = degree, parametric = parametric, : pseudoinverse used at -0.005
```

```
## Warning in simpleLoess(y, x, w, span, degree = degree, parametric = parametric, : neighborhood radius 0.005
```

```
## Warning in simpleLoess(y, x, w, span, degree = degree, parametric = parametric, : reciprocal condition number 1
```

```
## Warning in simpleLoess(y, x, w, span, degree = degree, parametric = parametric, : There are other near singularities as well. 1.01
```

```
## Warning in simpleLoess(y, x, w, span, degree = degree, parametric = parametric, : zero-width neighborhood. make span bigger
```

```
## Warning: Computation failed in `stat_smooth()`:
## NA/NaN/Inf in foreign function call (arg 5)
```

```
## Warning in simpleLoess(y, x, w, span, degree = degree, parametric = parametric, : at -0.005
```

```
## Warning in simpleLoess(y, x, w, span, degree = degree, parametric = parametric, : radius 2.5e-05
```

```
## Warning in simpleLoess(y, x, w, span, degree = degree, parametric = parametric, : all data on boundary of neighborhood. make span bigger
```

```
## Warning in simpleLoess(y, x, w, span, degree = degree, parametric = parametric, : pseudoinverse used at -0.005
```

```
## Warning in simpleLoess(y, x, w, span, degree = degree, parametric = parametric, : neighborhood radius 0.005
```

```
## Warning in simpleLoess(y, x, w, span, degree = degree, parametric = parametric, : reciprocal condition number 1
```

```
## Warning in simpleLoess(y, x, w, span, degree = degree, parametric = parametric, : There are other near singularities as well. 1.01
```

```
## Warning in simpleLoess(y, x, w, span, degree = degree, parametric = parametric, : zero-width neighborhood. make span bigger
```

```
## Warning: Computation failed in `stat_smooth()`:
## NA/NaN/Inf in foreign function call (arg 5)
```

```
## Warning in simpleLoess(y, x, w, span, degree = degree, parametric = parametric, : at -0.005
```

```
## Warning in simpleLoess(y, x, w, span, degree = degree, parametric = parametric, : radius 2.5e-05
```

```
## Warning in simpleLoess(y, x, w, span, degree = degree, parametric = parametric, : all data on boundary of neighborhood. make span bigger
```

```
## Warning in simpleLoess(y, x, w, span, degree = degree, parametric = parametric, : pseudoinverse used at -0.005
```

```
## Warning in simpleLoess(y, x, w, span, degree = degree, parametric = parametric, : neighborhood radius 0.005
```

```
## Warning in simpleLoess(y, x, w, span, degree = degree, parametric = parametric, : reciprocal condition number 1
```

```
## Warning in simpleLoess(y, x, w, span, degree = degree, parametric = parametric, : There are other near singularities as well. 1.01
```

```
## Warning in simpleLoess(y, x, w, span, degree = degree, parametric = parametric, : zero-width neighborhood. make span bigger
```

```
## Warning: Computation failed in `stat_smooth()`:
## NA/NaN/Inf in foreign function call (arg 5)
```

```
## Warning in simpleLoess(y, x, w, span, degree = degree, parametric = parametric, : at -0.005
```

```
## Warning in simpleLoess(y, x, w, span, degree = degree, parametric = parametric, : radius 2.5e-05
```

```
## Warning in simpleLoess(y, x, w, span, degree = degree, parametric = parametric, : all data on boundary of neighborhood. make span bigger
```

```
## Warning in simpleLoess(y, x, w, span, degree = degree, parametric = parametric, : pseudoinverse used at -0.005
```

```
## Warning in simpleLoess(y, x, w, span, degree = degree, parametric = parametric, : neighborhood radius 0.005
```

```
## Warning in simpleLoess(y, x, w, span, degree = degree, parametric = parametric, : reciprocal condition number 1
```

```
## Warning in simpleLoess(y, x, w, span, degree = degree, parametric = parametric, : There are other near singularities as well. 1.01
```

```
## Warning in simpleLoess(y, x, w, span, degree = degree, parametric = parametric, : zero-width neighborhood. make span bigger
```

```
## Warning: Computation failed in `stat_smooth()`:
## NA/NaN/Inf in foreign function call (arg 5)
```

```
## Warning in simpleLoess(y, x, w, span, degree = degree, parametric = parametric, : at -0.005
```

```
## Warning in simpleLoess(y, x, w, span, degree = degree, parametric = parametric, : radius 2.5e-05
```

```
## Warning in simpleLoess(y, x, w, span, degree = degree, parametric = parametric, : all data on boundary of neighborhood. make span bigger
```

```
## Warning in simpleLoess(y, x, w, span, degree = degree, parametric = parametric, : pseudoinverse used at -0.005
```

```
## Warning in simpleLoess(y, x, w, span, degree = degree, parametric = parametric, : neighborhood radius 0.005
```

```
## Warning in simpleLoess(y, x, w, span, degree = degree, parametric = parametric, : reciprocal condition number 1
```

```
## Warning in simpleLoess(y, x, w, span, degree = degree, parametric = parametric, : There are other near singularities as well. 1.01
```

```
## Warning in simpleLoess(y, x, w, span, degree = degree, parametric = parametric, : zero-width neighborhood. make span bigger
```

```
## Warning: Computation failed in `stat_smooth()`:
## NA/NaN/Inf in foreign function call (arg 5)
```

```
## Warning in simpleLoess(y, x, w, span, degree = degree, parametric = parametric, : at -0.005
```

```
## Warning in simpleLoess(y, x, w, span, degree = degree, parametric = parametric, : radius 2.5e-05
```

```
## Warning in simpleLoess(y, x, w, span, degree = degree, parametric = parametric, : all data on boundary of neighborhood. make span bigger
```

```
## Warning in simpleLoess(y, x, w, span, degree = degree, parametric = parametric, : pseudoinverse used at -0.005
```

```
## Warning in simpleLoess(y, x, w, span, degree = degree, parametric = parametric, : neighborhood radius 0.005
```

```
## Warning in simpleLoess(y, x, w, span, degree = degree, parametric = parametric, : reciprocal condition number 1
```

```
## Warning in simpleLoess(y, x, w, span, degree = degree, parametric = parametric, : There are other near singularities as well. 1.01
```

```
## Warning in simpleLoess(y, x, w, span, degree = degree, parametric = parametric, : zero-width neighborhood. make span bigger
```

```
## Warning: Computation failed in `stat_smooth()`:
## NA/NaN/Inf in foreign function call (arg 5)
```

```
## Warning in simpleLoess(y, x, w, span, degree = degree, parametric = parametric, : at -0.005
```

```
## Warning in simpleLoess(y, x, w, span, degree = degree, parametric = parametric, : radius 2.5e-05
```

```
## Warning in simpleLoess(y, x, w, span, degree = degree, parametric = parametric, : all data on boundary of neighborhood. make span bigger
```

```
## Warning in simpleLoess(y, x, w, span, degree = degree, parametric = parametric, : pseudoinverse used at -0.005
```

```
## Warning in simpleLoess(y, x, w, span, degree = degree, parametric = parametric, : neighborhood radius 0.005
```

```
## Warning in simpleLoess(y, x, w, span, degree = degree, parametric = parametric, : reciprocal condition number 1
```

```
## Warning in simpleLoess(y, x, w, span, degree = degree, parametric = parametric, : There are other near singularities as well. 1.01
```

```
## Warning in simpleLoess(y, x, w, span, degree = degree, parametric = parametric, : zero-width neighborhood. make span bigger
```

```
## Warning: Computation failed in `stat_smooth()`:
## NA/NaN/Inf in foreign function call (arg 5)
```

```
## Warning in simpleLoess(y, x, w, span, degree = degree, parametric = parametric, : at -0.005
```

```
## Warning in simpleLoess(y, x, w, span, degree = degree, parametric = parametric, : radius 2.5e-05
```

```
## Warning in simpleLoess(y, x, w, span, degree = degree, parametric = parametric, : all data on boundary of neighborhood. make span bigger
```

```
## Warning in simpleLoess(y, x, w, span, degree = degree, parametric = parametric, : pseudoinverse used at -0.005
```

```
## Warning in simpleLoess(y, x, w, span, degree = degree, parametric = parametric, : neighborhood radius 0.005
```

```
## Warning in simpleLoess(y, x, w, span, degree = degree, parametric = parametric, : reciprocal condition number 1
```

```
## Warning in simpleLoess(y, x, w, span, degree = degree, parametric = parametric, : There are other near singularities as well. 1.01
```

```
## Warning in simpleLoess(y, x, w, span, degree = degree, parametric = parametric, : zero-width neighborhood. make span bigger
```

```
## Warning: Computation failed in `stat_smooth()`:
## NA/NaN/Inf in foreign function call (arg 5)
```

```
## Warning in simpleLoess(y, x, w, span, degree = degree, parametric = parametric, : at -0.005
```

```
## Warning in simpleLoess(y, x, w, span, degree = degree, parametric = parametric, : radius 2.5e-05
```

```
## Warning in simpleLoess(y, x, w, span, degree = degree, parametric = parametric, : all data on boundary of neighborhood. make span bigger
```

```
## Warning in simpleLoess(y, x, w, span, degree = degree, parametric = parametric, : pseudoinverse used at -0.005
```

```
## Warning in simpleLoess(y, x, w, span, degree = degree, parametric = parametric, : neighborhood radius 0.005
```

```
## Warning in simpleLoess(y, x, w, span, degree = degree, parametric = parametric, : reciprocal condition number 1
```

```
## Warning in simpleLoess(y, x, w, span, degree = degree, parametric = parametric, : There are other near singularities as well. 1.01
```

```
## Warning in simpleLoess(y, x, w, span, degree = degree, parametric = parametric, : zero-width neighborhood. make span bigger
```

```
## Warning: Computation failed in `stat_smooth()`:
## NA/NaN/Inf in foreign function call (arg 5)
```

```
## Warning in simpleLoess(y, x, w, span, degree = degree, parametric = parametric, : at -0.005
```

```
## Warning in simpleLoess(y, x, w, span, degree = degree, parametric = parametric, : radius 2.5e-05
```

```
## Warning in simpleLoess(y, x, w, span, degree = degree, parametric = parametric, : all data on boundary of neighborhood. make span bigger
```

```
## Warning in simpleLoess(y, x, w, span, degree = degree, parametric = parametric, : pseudoinverse used at -0.005
```

```
## Warning in simpleLoess(y, x, w, span, degree = degree, parametric = parametric, : neighborhood radius 0.005
```

```
## Warning in simpleLoess(y, x, w, span, degree = degree, parametric = parametric, : reciprocal condition number 1
```

```
## Warning in simpleLoess(y, x, w, span, degree = degree, parametric = parametric, : There are other near singularities as well. 1.01
```

```
## Warning in simpleLoess(y, x, w, span, degree = degree, parametric = parametric, : zero-width neighborhood. make span bigger
```

```
## Warning: Computation failed in `stat_smooth()`:
## NA/NaN/Inf in foreign function call (arg 5)
```

```
## Warning in simpleLoess(y, x, w, span, degree = degree, parametric = parametric, : at -0.005
```

```
## Warning in simpleLoess(y, x, w, span, degree = degree, parametric = parametric, : radius 2.5e-05
```

```
## Warning in simpleLoess(y, x, w, span, degree = degree, parametric = parametric, : all data on boundary of neighborhood. make span bigger
```

```
## Warning in simpleLoess(y, x, w, span, degree = degree, parametric = parametric, : pseudoinverse used at -0.005
```

```
## Warning in simpleLoess(y, x, w, span, degree = degree, parametric = parametric, : neighborhood radius 0.005
```

```
## Warning in simpleLoess(y, x, w, span, degree = degree, parametric = parametric, : reciprocal condition number 1
```

```
## Warning in simpleLoess(y, x, w, span, degree = degree, parametric = parametric, : There are other near singularities as well. 1.01
```

```
## Warning in simpleLoess(y, x, w, span, degree = degree, parametric = parametric, : zero-width neighborhood. make span bigger
```

```
## Warning: Computation failed in `stat_smooth()`:
## NA/NaN/Inf in foreign function call (arg 5)
```

```
## Warning in simpleLoess(y, x, w, span, degree = degree, parametric = parametric, : at -0.005
```

```
## Warning in simpleLoess(y, x, w, span, degree = degree, parametric = parametric, : radius 2.5e-05
```

```
## Warning in simpleLoess(y, x, w, span, degree = degree, parametric = parametric, : all data on boundary of neighborhood. make span bigger
```

```
## Warning in simpleLoess(y, x, w, span, degree = degree, parametric = parametric, : pseudoinverse used at -0.005
```

```
## Warning in simpleLoess(y, x, w, span, degree = degree, parametric = parametric, : neighborhood radius 0.005
```

```
## Warning in simpleLoess(y, x, w, span, degree = degree, parametric = parametric, : reciprocal condition number 1
```

```
## Warning in simpleLoess(y, x, w, span, degree = degree, parametric = parametric, : There are other near singularities as well. 1.01
```

```
## Warning in simpleLoess(y, x, w, span, degree = degree, parametric = parametric, : zero-width neighborhood. make span bigger
```

```
## Warning: Computation failed in `stat_smooth()`:
## NA/NaN/Inf in foreign function call (arg 5)
```

```
## Warning in simpleLoess(y, x, w, span, degree = degree, parametric = parametric, : at -0.005
```

```
## Warning in simpleLoess(y, x, w, span, degree = degree, parametric = parametric, : radius 2.5e-05
```

```
## Warning in simpleLoess(y, x, w, span, degree = degree, parametric = parametric, : all data on boundary of neighborhood. make span bigger
```

```
## Warning in simpleLoess(y, x, w, span, degree = degree, parametric = parametric, : pseudoinverse used at -0.005
```

```
## Warning in simpleLoess(y, x, w, span, degree = degree, parametric = parametric, : neighborhood radius 0.005
```

```
## Warning in simpleLoess(y, x, w, span, degree = degree, parametric = parametric, : reciprocal condition number 1
```

```
## Warning in simpleLoess(y, x, w, span, degree = degree, parametric = parametric, : There are other near singularities as well. 1.01
```

```
## Warning in simpleLoess(y, x, w, span, degree = degree, parametric = parametric, : zero-width neighborhood. make span bigger
```

```
## Warning: Computation failed in `stat_smooth()`:
## NA/NaN/Inf in foreign function call (arg 5)
```

```
## Warning in simpleLoess(y, x, w, span, degree = degree, parametric = parametric, : at -0.005
```

```
## Warning in simpleLoess(y, x, w, span, degree = degree, parametric = parametric, : radius 2.5e-05
```

```
## Warning in simpleLoess(y, x, w, span, degree = degree, parametric = parametric, : all data on boundary of neighborhood. make span bigger
```

```
## Warning in simpleLoess(y, x, w, span, degree = degree, parametric = parametric, : pseudoinverse used at -0.005
```

```
## Warning in simpleLoess(y, x, w, span, degree = degree, parametric = parametric, : neighborhood radius 0.005
```

```
## Warning in simpleLoess(y, x, w, span, degree = degree, parametric = parametric, : reciprocal condition number 1
```

```
## Warning in simpleLoess(y, x, w, span, degree = degree, parametric = parametric, : There are other near singularities as well. 1.01
```

```
## Warning in simpleLoess(y, x, w, span, degree = degree, parametric = parametric, : zero-width neighborhood. make span bigger
```

```
## Warning: Computation failed in `stat_smooth()`:
## NA/NaN/Inf in foreign function call (arg 5)
```

```
## Warning in simpleLoess(y, x, w, span, degree = degree, parametric = parametric, : at -0.005
```

```
## Warning in simpleLoess(y, x, w, span, degree = degree, parametric = parametric, : radius 2.5e-05
```

```
## Warning in simpleLoess(y, x, w, span, degree = degree, parametric = parametric, : all data on boundary of neighborhood. make span bigger
```

```
## Warning in simpleLoess(y, x, w, span, degree = degree, parametric = parametric, : pseudoinverse used at -0.005
```

```
## Warning in simpleLoess(y, x, w, span, degree = degree, parametric = parametric, : neighborhood radius 0.005
```

```
## Warning in simpleLoess(y, x, w, span, degree = degree, parametric = parametric, : reciprocal condition number 1
```

```
## Warning in simpleLoess(y, x, w, span, degree = degree, parametric = parametric, : There are other near singularities as well. 1.01
```

```
## Warning in simpleLoess(y, x, w, span, degree = degree, parametric = parametric, : zero-width neighborhood. make span bigger
```

```
## Warning: Computation failed in `stat_smooth()`:
## NA/NaN/Inf in foreign function call (arg 5)
```

```
## Warning in simpleLoess(y, x, w, span, degree = degree, parametric = parametric, : at -0.005
```

```
## Warning in simpleLoess(y, x, w, span, degree = degree, parametric = parametric, : radius 2.5e-05
```

```
## Warning in simpleLoess(y, x, w, span, degree = degree, parametric = parametric, : all data on boundary of neighborhood. make span bigger
```

```
## Warning in simpleLoess(y, x, w, span, degree = degree, parametric = parametric, : pseudoinverse used at -0.005
```

```
## Warning in simpleLoess(y, x, w, span, degree = degree, parametric = parametric, : neighborhood radius 0.005
```

```
## Warning in simpleLoess(y, x, w, span, degree = degree, parametric = parametric, : reciprocal condition number 1
```

```
## Warning in simpleLoess(y, x, w, span, degree = degree, parametric = parametric, : There are other near singularities as well. 1.01
```

```
## Warning in simpleLoess(y, x, w, span, degree = degree, parametric = parametric, : zero-width neighborhood. make span bigger
```

```
## Warning: Computation failed in `stat_smooth()`:
## NA/NaN/Inf in foreign function call (arg 5)
```

```
## Warning in simpleLoess(y, x, w, span, degree = degree, parametric = parametric, : at -0.005
```

```
## Warning in simpleLoess(y, x, w, span, degree = degree, parametric = parametric, : radius 2.5e-05
```

```
## Warning in simpleLoess(y, x, w, span, degree = degree, parametric = parametric, : all data on boundary of neighborhood. make span bigger
```

```
## Warning in simpleLoess(y, x, w, span, degree = degree, parametric = parametric, : pseudoinverse used at -0.005
```

```
## Warning in simpleLoess(y, x, w, span, degree = degree, parametric = parametric, : neighborhood radius 0.005
```

```
## Warning in simpleLoess(y, x, w, span, degree = degree, parametric = parametric, : reciprocal condition number 1
```

```
## Warning in simpleLoess(y, x, w, span, degree = degree, parametric = parametric, : There are other near singularities as well. 1.01
```

```
## Warning in simpleLoess(y, x, w, span, degree = degree, parametric = parametric, : zero-width neighborhood. make span bigger
```

```
## Warning: Computation failed in `stat_smooth()`:
## NA/NaN/Inf in foreign function call (arg 5)
```

```
## Warning in simpleLoess(y, x, w, span, degree = degree, parametric = parametric, : at -0.005
```

```
## Warning in simpleLoess(y, x, w, span, degree = degree, parametric = parametric, : radius 2.5e-05
```

```
## Warning in simpleLoess(y, x, w, span, degree = degree, parametric = parametric, : all data on boundary of neighborhood. make span bigger
```

```
## Warning in simpleLoess(y, x, w, span, degree = degree, parametric = parametric, : pseudoinverse used at -0.005
```

```
## Warning in simpleLoess(y, x, w, span, degree = degree, parametric = parametric, : neighborhood radius 0.005
```

```
## Warning in simpleLoess(y, x, w, span, degree = degree, parametric = parametric, : reciprocal condition number 1
```

```
## Warning in simpleLoess(y, x, w, span, degree = degree, parametric = parametric, : There are other near singularities as well. 1.01
```

```
## Warning in simpleLoess(y, x, w, span, degree = degree, parametric = parametric, : zero-width neighborhood. make span bigger
```

```
## Warning: Computation failed in `stat_smooth()`:
## NA/NaN/Inf in foreign function call (arg 5)
```

```
## Warning in simpleLoess(y, x, w, span, degree = degree, parametric = parametric, : at -0.005
```

```
## Warning in simpleLoess(y, x, w, span, degree = degree, parametric = parametric, : radius 2.5e-05
```

```
## Warning in simpleLoess(y, x, w, span, degree = degree, parametric = parametric, : all data on boundary of neighborhood. make span bigger
```

```
## Warning in simpleLoess(y, x, w, span, degree = degree, parametric = parametric, : pseudoinverse used at -0.005
```

```
## Warning in simpleLoess(y, x, w, span, degree = degree, parametric = parametric, : neighborhood radius 0.005
```

```
## Warning in simpleLoess(y, x, w, span, degree = degree, parametric = parametric, : reciprocal condition number 1
```

```
## Warning in simpleLoess(y, x, w, span, degree = degree, parametric = parametric, : There are other near singularities as well. 1.01
```

```
## Warning in simpleLoess(y, x, w, span, degree = degree, parametric = parametric, : zero-width neighborhood. make span bigger
```

```
## Warning: Computation failed in `stat_smooth()`:
## NA/NaN/Inf in foreign function call (arg 5)
```

```
## Warning in simpleLoess(y, x, w, span, degree = degree, parametric = parametric, : at -0.005
```

```
## Warning in simpleLoess(y, x, w, span, degree = degree, parametric = parametric, : radius 2.5e-05
```

```
## Warning in simpleLoess(y, x, w, span, degree = degree, parametric = parametric, : all data on boundary of neighborhood. make span bigger
```

```
## Warning in simpleLoess(y, x, w, span, degree = degree, parametric = parametric, : pseudoinverse used at -0.005
```

```
## Warning in simpleLoess(y, x, w, span, degree = degree, parametric = parametric, : neighborhood radius 0.005
```

```
## Warning in simpleLoess(y, x, w, span, degree = degree, parametric = parametric, : reciprocal condition number 1
```

```
## Warning in simpleLoess(y, x, w, span, degree = degree, parametric = parametric, : There are other near singularities as well. 1.01
```

```
## Warning in simpleLoess(y, x, w, span, degree = degree, parametric = parametric, : zero-width neighborhood. make span bigger
```

```
## Warning: Computation failed in `stat_smooth()`:
## NA/NaN/Inf in foreign function call (arg 5)
```

```
## Warning in simpleLoess(y, x, w, span, degree = degree, parametric = parametric, : at -0.005
```

```
## Warning in simpleLoess(y, x, w, span, degree = degree, parametric = parametric, : radius 2.5e-05
```

```
## Warning in simpleLoess(y, x, w, span, degree = degree, parametric = parametric, : all data on boundary of neighborhood. make span bigger
```

```
## Warning in simpleLoess(y, x, w, span, degree = degree, parametric = parametric, : pseudoinverse used at -0.005
```

```
## Warning in simpleLoess(y, x, w, span, degree = degree, parametric = parametric, : neighborhood radius 0.005
```

```
## Warning in simpleLoess(y, x, w, span, degree = degree, parametric = parametric, : reciprocal condition number 1
```

```
## Warning in simpleLoess(y, x, w, span, degree = degree, parametric = parametric, : There are other near singularities as well. 1.01
```

```
## Warning in simpleLoess(y, x, w, span, degree = degree, parametric = parametric, : zero-width neighborhood. make span bigger
```

```
## Warning: Computation failed in `stat_smooth()`:
## NA/NaN/Inf in foreign function call (arg 5)
```

```
## Warning in simpleLoess(y, x, w, span, degree = degree, parametric = parametric, : at -0.005
```

```
## Warning in simpleLoess(y, x, w, span, degree = degree, parametric = parametric, : radius 2.5e-05
```

```
## Warning in simpleLoess(y, x, w, span, degree = degree, parametric = parametric, : all data on boundary of neighborhood. make span bigger
```

```
## Warning in simpleLoess(y, x, w, span, degree = degree, parametric = parametric, : pseudoinverse used at -0.005
```

```
## Warning in simpleLoess(y, x, w, span, degree = degree, parametric = parametric, : neighborhood radius 0.005
```

```
## Warning in simpleLoess(y, x, w, span, degree = degree, parametric = parametric, : reciprocal condition number 1
```

```
## Warning in simpleLoess(y, x, w, span, degree = degree, parametric = parametric, : There are other near singularities as well. 1.01
```

```
## Warning in simpleLoess(y, x, w, span, degree = degree, parametric = parametric, : zero-width neighborhood. make span bigger
```

```
## Warning: Computation failed in `stat_smooth()`:
## NA/NaN/Inf in foreign function call (arg 5)
```

```
## Warning in simpleLoess(y, x, w, span, degree = degree, parametric = parametric, : at -0.005
```

```
## Warning in simpleLoess(y, x, w, span, degree = degree, parametric = parametric, : radius 2.5e-05
```

```
## Warning in simpleLoess(y, x, w, span, degree = degree, parametric = parametric, : all data on boundary of neighborhood. make span bigger
```

```
## Warning in simpleLoess(y, x, w, span, degree = degree, parametric = parametric, : pseudoinverse used at -0.005
```

```
## Warning in simpleLoess(y, x, w, span, degree = degree, parametric = parametric, : neighborhood radius 0.005
```

```
## Warning in simpleLoess(y, x, w, span, degree = degree, parametric = parametric, : reciprocal condition number 1
```

```
## Warning in simpleLoess(y, x, w, span, degree = degree, parametric = parametric, : There are other near singularities as well. 1.01
```

```
## Warning in simpleLoess(y, x, w, span, degree = degree, parametric = parametric, : zero-width neighborhood. make span bigger
```

```
## Warning: Computation failed in `stat_smooth()`:
## NA/NaN/Inf in foreign function call (arg 5)
```

```
## Warning in simpleLoess(y, x, w, span, degree = degree, parametric = parametric, : at -0.005
```

```
## Warning in simpleLoess(y, x, w, span, degree = degree, parametric = parametric, : radius 2.5e-05
```

```
## Warning in simpleLoess(y, x, w, span, degree = degree, parametric = parametric, : all data on boundary of neighborhood. make span bigger
```

```
## Warning in simpleLoess(y, x, w, span, degree = degree, parametric = parametric, : pseudoinverse used at -0.005
```

```
## Warning in simpleLoess(y, x, w, span, degree = degree, parametric = parametric, : neighborhood radius 0.005
```

```
## Warning in simpleLoess(y, x, w, span, degree = degree, parametric = parametric, : reciprocal condition number 1
```

```
## Warning in simpleLoess(y, x, w, span, degree = degree, parametric = parametric, : There are other near singularities as well. 1.01
```

```
## Warning in simpleLoess(y, x, w, span, degree = degree, parametric = parametric, : zero-width neighborhood. make span bigger
```

```
## Warning: Computation failed in `stat_smooth()`:
## NA/NaN/Inf in foreign function call (arg 5)
```

```
## Warning in simpleLoess(y, x, w, span, degree = degree, parametric = parametric, : at -0.005
```

```
## Warning in simpleLoess(y, x, w, span, degree = degree, parametric = parametric, : radius 2.5e-05
```

```
## Warning in simpleLoess(y, x, w, span, degree = degree, parametric = parametric, : all data on boundary of neighborhood. make span bigger
```

```
## Warning in simpleLoess(y, x, w, span, degree = degree, parametric = parametric, : pseudoinverse used at -0.005
```

```
## Warning in simpleLoess(y, x, w, span, degree = degree, parametric = parametric, : neighborhood radius 0.005
```

```
## Warning in simpleLoess(y, x, w, span, degree = degree, parametric = parametric, : reciprocal condition number 1
```

```
## Warning in simpleLoess(y, x, w, span, degree = degree, parametric = parametric, : There are other near singularities as well. 1.01
```

```
## Warning in simpleLoess(y, x, w, span, degree = degree, parametric = parametric, : zero-width neighborhood. make span bigger
```

```
## Warning: Computation failed in `stat_smooth()`:
## NA/NaN/Inf in foreign function call (arg 5)
```

```
## Warning in simpleLoess(y, x, w, span, degree = degree, parametric = parametric, : at -0.005
```

```
## Warning in simpleLoess(y, x, w, span, degree = degree, parametric = parametric, : radius 2.5e-05
```

```
## Warning in simpleLoess(y, x, w, span, degree = degree, parametric = parametric, : all data on boundary of neighborhood. make span bigger
```

```
## Warning in simpleLoess(y, x, w, span, degree = degree, parametric = parametric, : pseudoinverse used at -0.005
```

```
## Warning in simpleLoess(y, x, w, span, degree = degree, parametric = parametric, : neighborhood radius 0.005
```

```
## Warning in simpleLoess(y, x, w, span, degree = degree, parametric = parametric, : reciprocal condition number 1
```

```
## Warning in simpleLoess(y, x, w, span, degree = degree, parametric = parametric, : There are other near singularities as well. 1.01
```

```
## Warning in simpleLoess(y, x, w, span, degree = degree, parametric = parametric, : zero-width neighborhood. make span bigger
```

```
## Warning: Computation failed in `stat_smooth()`:
## NA/NaN/Inf in foreign function call (arg 5)
```

```
## Warning in simpleLoess(y, x, w, span, degree = degree, parametric = parametric, : at -0.005
```

```
## Warning in simpleLoess(y, x, w, span, degree = degree, parametric = parametric, : radius 2.5e-05
```

```
## Warning in simpleLoess(y, x, w, span, degree = degree, parametric = parametric, : all data on boundary of neighborhood. make span bigger
```

```
## Warning in simpleLoess(y, x, w, span, degree = degree, parametric = parametric, : pseudoinverse used at -0.005
```

```
## Warning in simpleLoess(y, x, w, span, degree = degree, parametric = parametric, : neighborhood radius 0.005
```

```
## Warning in simpleLoess(y, x, w, span, degree = degree, parametric = parametric, : reciprocal condition number 1
```

```
## Warning in simpleLoess(y, x, w, span, degree = degree, parametric = parametric, : There are other near singularities as well. 1.01
```

```
## Warning in simpleLoess(y, x, w, span, degree = degree, parametric = parametric, : zero-width neighborhood. make span bigger
```

```
## Warning: Computation failed in `stat_smooth()`:
## NA/NaN/Inf in foreign function call (arg 5)
```

```
## Warning in simpleLoess(y, x, w, span, degree = degree, parametric = parametric, : at -0.005
```

```
## Warning in simpleLoess(y, x, w, span, degree = degree, parametric = parametric, : radius 2.5e-05
```

```
## Warning in simpleLoess(y, x, w, span, degree = degree, parametric = parametric, : all data on boundary of neighborhood. make span bigger
```

```
## Warning in simpleLoess(y, x, w, span, degree = degree, parametric = parametric, : pseudoinverse used at -0.005
```

```
## Warning in simpleLoess(y, x, w, span, degree = degree, parametric = parametric, : neighborhood radius 0.005
```

```
## Warning in simpleLoess(y, x, w, span, degree = degree, parametric = parametric, : reciprocal condition number 1
```

```
## Warning in simpleLoess(y, x, w, span, degree = degree, parametric = parametric, : There are other near singularities as well. 1.01
```

```
## Warning in simpleLoess(y, x, w, span, degree = degree, parametric = parametric, : zero-width neighborhood. make span bigger
```

```
## Warning: Computation failed in `stat_smooth()`:
## NA/NaN/Inf in foreign function call (arg 5)
```

```
## Warning in simpleLoess(y, x, w, span, degree = degree, parametric = parametric, : at -0.005
```

```
## Warning in simpleLoess(y, x, w, span, degree = degree, parametric = parametric, : radius 2.5e-05
```

```
## Warning in simpleLoess(y, x, w, span, degree = degree, parametric = parametric, : all data on boundary of neighborhood. make span bigger
```

```
## Warning in simpleLoess(y, x, w, span, degree = degree, parametric = parametric, : pseudoinverse used at -0.005
```

```
## Warning in simpleLoess(y, x, w, span, degree = degree, parametric = parametric, : neighborhood radius 0.005
```

```
## Warning in simpleLoess(y, x, w, span, degree = degree, parametric = parametric, : reciprocal condition number 1
```

```
## Warning in simpleLoess(y, x, w, span, degree = degree, parametric = parametric, : There are other near singularities as well. 1.01
```

```
## Warning in simpleLoess(y, x, w, span, degree = degree, parametric = parametric, : zero-width neighborhood. make span bigger
```

```
## Warning: Computation failed in `stat_smooth()`:
## NA/NaN/Inf in foreign function call (arg 5)
```

```
## Warning in simpleLoess(y, x, w, span, degree = degree, parametric = parametric, : at -0.005
```

```
## Warning in simpleLoess(y, x, w, span, degree = degree, parametric = parametric, : radius 2.5e-05
```

```
## Warning in simpleLoess(y, x, w, span, degree = degree, parametric = parametric, : all data on boundary of neighborhood. make span bigger
```

```
## Warning in simpleLoess(y, x, w, span, degree = degree, parametric = parametric, : pseudoinverse used at -0.005
```

```
## Warning in simpleLoess(y, x, w, span, degree = degree, parametric = parametric, : neighborhood radius 0.005
```

```
## Warning in simpleLoess(y, x, w, span, degree = degree, parametric = parametric, : reciprocal condition number 1
```

```
## Warning in simpleLoess(y, x, w, span, degree = degree, parametric = parametric, : There are other near singularities as well. 1.01
```

```
## Warning in simpleLoess(y, x, w, span, degree = degree, parametric = parametric, : zero-width neighborhood. make span bigger
```

```
## Warning: Computation failed in `stat_smooth()`:
## NA/NaN/Inf in foreign function call (arg 5)
```

```
## Warning in simpleLoess(y, x, w, span, degree = degree, parametric = parametric, : pseudoinverse used at -0.005
```

```
## Warning in simpleLoess(y, x, w, span, degree = degree, parametric = parametric, : neighborhood radius 1.005
```

```
## Warning in simpleLoess(y, x, w, span, degree = degree, parametric = parametric, : reciprocal condition number 0
```

```
## Warning in simpleLoess(y, x, w, span, degree = degree, parametric = parametric, : There are other near singularities as well. 1.01
```

```
## Warning in predLoess(object$y, object$x, newx = if (is.null(newdata)) object$x else if (is.data.frame(newdata)) as.matrix(model.frame(delete.response(terms(object)), : pseudoinverse used at -0.005
```

```
## Warning in predLoess(object$y, object$x, newx = if (is.null(newdata)) object$x else if (is.data.frame(newdata)) as.matrix(model.frame(delete.response(terms(object)), : neighborhood radius 1.005
```

```
## Warning in predLoess(object$y, object$x, newx = if (is.null(newdata)) object$x else if (is.data.frame(newdata)) as.matrix(model.frame(delete.response(terms(object)), : reciprocal condition number 0
```

```
## Warning in predLoess(object$y, object$x, newx = if (is.null(newdata)) object$x else if (is.data.frame(newdata)) as.matrix(model.frame(delete.response(terms(object)), : There are other near
## singularities as well. 1.01
```

```
## Warning in simpleLoess(y, x, w, span, degree = degree, parametric = parametric, : at -0.005
```

```
## Warning in simpleLoess(y, x, w, span, degree = degree, parametric = parametric, : radius 2.5e-05
```

```
## Warning in simpleLoess(y, x, w, span, degree = degree, parametric = parametric, : all data on boundary of neighborhood. make span bigger
```

```
## Warning in simpleLoess(y, x, w, span, degree = degree, parametric = parametric, : pseudoinverse used at -0.005
```

```
## Warning in simpleLoess(y, x, w, span, degree = degree, parametric = parametric, : neighborhood radius 0.005
```

```
## Warning in simpleLoess(y, x, w, span, degree = degree, parametric = parametric, : reciprocal condition number 1
```

```
## Warning in simpleLoess(y, x, w, span, degree = degree, parametric = parametric, : There are other near singularities as well. 1.01
```

```
## Warning in simpleLoess(y, x, w, span, degree = degree, parametric = parametric, : zero-width neighborhood. make span bigger
```

```
## Warning: Computation failed in `stat_smooth()`:
## NA/NaN/Inf in foreign function call (arg 5)
```

```
## Warning in simpleLoess(y, x, w, span, degree = degree, parametric = parametric, : at -0.005
```

```
## Warning in simpleLoess(y, x, w, span, degree = degree, parametric = parametric, : radius 2.5e-05
```

```
## Warning in simpleLoess(y, x, w, span, degree = degree, parametric = parametric, : all data on boundary of neighborhood. make span bigger
```

```
## Warning in simpleLoess(y, x, w, span, degree = degree, parametric = parametric, : pseudoinverse used at -0.005
```

```
## Warning in simpleLoess(y, x, w, span, degree = degree, parametric = parametric, : neighborhood radius 0.005
```

```
## Warning in simpleLoess(y, x, w, span, degree = degree, parametric = parametric, : reciprocal condition number 1
```

```
## Warning in simpleLoess(y, x, w, span, degree = degree, parametric = parametric, : There are other near singularities as well. 1.01
```

```
## Warning in simpleLoess(y, x, w, span, degree = degree, parametric = parametric, : zero-width neighborhood. make span bigger
```

```
## Warning: Computation failed in `stat_smooth()`:
## NA/NaN/Inf in foreign function call (arg 5)
```

```
## Warning in simpleLoess(y, x, w, span, degree = degree, parametric = parametric, : at -0.005
```

```
## Warning in simpleLoess(y, x, w, span, degree = degree, parametric = parametric, : radius 2.5e-05
```

```
## Warning in simpleLoess(y, x, w, span, degree = degree, parametric = parametric, : all data on boundary of neighborhood. make span bigger
```

```
## Warning in simpleLoess(y, x, w, span, degree = degree, parametric = parametric, : pseudoinverse used at -0.005
```

```
## Warning in simpleLoess(y, x, w, span, degree = degree, parametric = parametric, : neighborhood radius 0.005
```

```
## Warning in simpleLoess(y, x, w, span, degree = degree, parametric = parametric, : reciprocal condition number 1
```

```
## Warning in simpleLoess(y, x, w, span, degree = degree, parametric = parametric, : There are other near singularities as well. 1.01
```

```
## Warning in simpleLoess(y, x, w, span, degree = degree, parametric = parametric, : zero-width neighborhood. make span bigger
```

```
## Warning: Computation failed in `stat_smooth()`:
## NA/NaN/Inf in foreign function call (arg 5)
```

```
## Warning in simpleLoess(y, x, w, span, degree = degree, parametric = parametric, : pseudoinverse used at -0.005
```

```
## Warning in simpleLoess(y, x, w, span, degree = degree, parametric = parametric, : neighborhood radius 1.005
```

```
## Warning in simpleLoess(y, x, w, span, degree = degree, parametric = parametric, : reciprocal condition number 0
```

```
## Warning in simpleLoess(y, x, w, span, degree = degree, parametric = parametric, : There are other near singularities as well. 1.01
```

```
## Warning in predLoess(object$y, object$x, newx = if (is.null(newdata)) object$x else if (is.data.frame(newdata)) as.matrix(model.frame(delete.response(terms(object)), : pseudoinverse used at -0.005
```

```
## Warning in predLoess(object$y, object$x, newx = if (is.null(newdata)) object$x else if (is.data.frame(newdata)) as.matrix(model.frame(delete.response(terms(object)), : neighborhood radius 1.005
```

```
## Warning in predLoess(object$y, object$x, newx = if (is.null(newdata)) object$x else if (is.data.frame(newdata)) as.matrix(model.frame(delete.response(terms(object)), : reciprocal condition number 0
```

```
## Warning in predLoess(object$y, object$x, newx = if (is.null(newdata)) object$x else if (is.data.frame(newdata)) as.matrix(model.frame(delete.response(terms(object)), : There are other near
## singularities as well. 1.01
```

```
## Warning in simpleLoess(y, x, w, span, degree = degree, parametric = parametric, : at -0.005
```

```
## Warning in simpleLoess(y, x, w, span, degree = degree, parametric = parametric, : radius 2.5e-05
```

```
## Warning in simpleLoess(y, x, w, span, degree = degree, parametric = parametric, : all data on boundary of neighborhood. make span bigger
```

```
## Warning in simpleLoess(y, x, w, span, degree = degree, parametric = parametric, : pseudoinverse used at -0.005
```

```
## Warning in simpleLoess(y, x, w, span, degree = degree, parametric = parametric, : neighborhood radius 0.005
```

```
## Warning in simpleLoess(y, x, w, span, degree = degree, parametric = parametric, : reciprocal condition number 1
```

```
## Warning in simpleLoess(y, x, w, span, degree = degree, parametric = parametric, : There are other near singularities as well. 1.01
```

```
## Warning in simpleLoess(y, x, w, span, degree = degree, parametric = parametric, : zero-width neighborhood. make span bigger
```

```
## Warning: Computation failed in `stat_smooth()`:
## NA/NaN/Inf in foreign function call (arg 5)
```

```
## Warning in simpleLoess(y, x, w, span, degree = degree, parametric = parametric, : at -0.005
```

```
## Warning in simpleLoess(y, x, w, span, degree = degree, parametric = parametric, : radius 2.5e-05
```

```
## Warning in simpleLoess(y, x, w, span, degree = degree, parametric = parametric, : all data on boundary of neighborhood. make span bigger
```

```
## Warning in simpleLoess(y, x, w, span, degree = degree, parametric = parametric, : pseudoinverse used at -0.005
```

```
## Warning in simpleLoess(y, x, w, span, degree = degree, parametric = parametric, : neighborhood radius 0.005
```

```
## Warning in simpleLoess(y, x, w, span, degree = degree, parametric = parametric, : reciprocal condition number 1
```

```
## Warning in simpleLoess(y, x, w, span, degree = degree, parametric = parametric, : There are other near singularities as well. 1.01
```

```
## Warning in simpleLoess(y, x, w, span, degree = degree, parametric = parametric, : zero-width neighborhood. make span bigger
```

```
## Warning: Computation failed in `stat_smooth()`:
## NA/NaN/Inf in foreign function call (arg 5)
```

```
## Warning in simpleLoess(y, x, w, span, degree = degree, parametric = parametric, : at -0.005
```

```
## Warning in simpleLoess(y, x, w, span, degree = degree, parametric = parametric, : radius 2.5e-05
```

```
## Warning in simpleLoess(y, x, w, span, degree = degree, parametric = parametric, : all data on boundary of neighborhood. make span bigger
```

```
## Warning in simpleLoess(y, x, w, span, degree = degree, parametric = parametric, : pseudoinverse used at -0.005
```

```
## Warning in simpleLoess(y, x, w, span, degree = degree, parametric = parametric, : neighborhood radius 0.005
```

```
## Warning in simpleLoess(y, x, w, span, degree = degree, parametric = parametric, : reciprocal condition number 1
```

```
## Warning in simpleLoess(y, x, w, span, degree = degree, parametric = parametric, : There are other near singularities as well. 1.01
```

```
## Warning in simpleLoess(y, x, w, span, degree = degree, parametric = parametric, : zero-width neighborhood. make span bigger
```

```
## Warning: Computation failed in `stat_smooth()`:
## NA/NaN/Inf in foreign function call (arg 5)
```

```
## Warning in simpleLoess(y, x, w, span, degree = degree, parametric = parametric, : at -0.005
```

```
## Warning in simpleLoess(y, x, w, span, degree = degree, parametric = parametric, : radius 2.5e-05
```

```
## Warning in simpleLoess(y, x, w, span, degree = degree, parametric = parametric, : all data on boundary of neighborhood. make span bigger
```

```
## Warning in simpleLoess(y, x, w, span, degree = degree, parametric = parametric, : pseudoinverse used at -0.005
```

```
## Warning in simpleLoess(y, x, w, span, degree = degree, parametric = parametric, : neighborhood radius 0.005
```

```
## Warning in simpleLoess(y, x, w, span, degree = degree, parametric = parametric, : reciprocal condition number 1
```

```
## Warning in simpleLoess(y, x, w, span, degree = degree, parametric = parametric, : There are other near singularities as well. 1.01
```

```
## Warning in simpleLoess(y, x, w, span, degree = degree, parametric = parametric, : zero-width neighborhood. make span bigger
```

```
## Warning: Computation failed in `stat_smooth()`:
## NA/NaN/Inf in foreign function call (arg 5)
```

```
## Warning in simpleLoess(y, x, w, span, degree = degree, parametric = parametric, : at -0.005
```

```
## Warning in simpleLoess(y, x, w, span, degree = degree, parametric = parametric, : radius 2.5e-05
```

```
## Warning in simpleLoess(y, x, w, span, degree = degree, parametric = parametric, : all data on boundary of neighborhood. make span bigger
```

```
## Warning in simpleLoess(y, x, w, span, degree = degree, parametric = parametric, : pseudoinverse used at -0.005
```

```
## Warning in simpleLoess(y, x, w, span, degree = degree, parametric = parametric, : neighborhood radius 0.005
```

```
## Warning in simpleLoess(y, x, w, span, degree = degree, parametric = parametric, : reciprocal condition number 1
```

```
## Warning in simpleLoess(y, x, w, span, degree = degree, parametric = parametric, : There are other near singularities as well. 1.01
```

```
## Warning in simpleLoess(y, x, w, span, degree = degree, parametric = parametric, : zero-width neighborhood. make span bigger
```

```
## Warning: Computation failed in `stat_smooth()`:
## NA/NaN/Inf in foreign function call (arg 5)
```

```
## Warning in simpleLoess(y, x, w, span, degree = degree, parametric = parametric, : at -0.005
```

```
## Warning in simpleLoess(y, x, w, span, degree = degree, parametric = parametric, : radius 2.5e-05
```

```
## Warning in simpleLoess(y, x, w, span, degree = degree, parametric = parametric, : all data on boundary of neighborhood. make span bigger
```

```
## Warning in simpleLoess(y, x, w, span, degree = degree, parametric = parametric, : pseudoinverse used at -0.005
```

```
## Warning in simpleLoess(y, x, w, span, degree = degree, parametric = parametric, : neighborhood radius 0.005
```

```
## Warning in simpleLoess(y, x, w, span, degree = degree, parametric = parametric, : reciprocal condition number 1
```

```
## Warning in simpleLoess(y, x, w, span, degree = degree, parametric = parametric, : There are other near singularities as well. 1.01
```

```
## Warning in simpleLoess(y, x, w, span, degree = degree, parametric = parametric, : zero-width neighborhood. make span bigger
```

```
## Warning: Computation failed in `stat_smooth()`:
## NA/NaN/Inf in foreign function call (arg 5)
```

```
## Warning in simpleLoess(y, x, w, span, degree = degree, parametric = parametric, : at -0.005
```

```
## Warning in simpleLoess(y, x, w, span, degree = degree, parametric = parametric, : radius 2.5e-05
```

```
## Warning in simpleLoess(y, x, w, span, degree = degree, parametric = parametric, : all data on boundary of neighborhood. make span bigger
```

```
## Warning in simpleLoess(y, x, w, span, degree = degree, parametric = parametric, : pseudoinverse used at -0.005
```

```
## Warning in simpleLoess(y, x, w, span, degree = degree, parametric = parametric, : neighborhood radius 0.005
```

```
## Warning in simpleLoess(y, x, w, span, degree = degree, parametric = parametric, : reciprocal condition number 1
```

```
## Warning in simpleLoess(y, x, w, span, degree = degree, parametric = parametric, : There are other near singularities as well. 1.01
```

```
## Warning in simpleLoess(y, x, w, span, degree = degree, parametric = parametric, : zero-width neighborhood. make span bigger
```

```
## Warning: Computation failed in `stat_smooth()`:
## NA/NaN/Inf in foreign function call (arg 5)
```

```
## Warning in simpleLoess(y, x, w, span, degree = degree, parametric = parametric, : at -0.005
```

```
## Warning in simpleLoess(y, x, w, span, degree = degree, parametric = parametric, : radius 2.5e-05
```

```
## Warning in simpleLoess(y, x, w, span, degree = degree, parametric = parametric, : all data on boundary of neighborhood. make span bigger
```

```
## Warning in simpleLoess(y, x, w, span, degree = degree, parametric = parametric, : pseudoinverse used at -0.005
```

```
## Warning in simpleLoess(y, x, w, span, degree = degree, parametric = parametric, : neighborhood radius 0.005
```

```
## Warning in simpleLoess(y, x, w, span, degree = degree, parametric = parametric, : reciprocal condition number 1
```

```
## Warning in simpleLoess(y, x, w, span, degree = degree, parametric = parametric, : There are other near singularities as well. 1.01
```

```
## Warning in simpleLoess(y, x, w, span, degree = degree, parametric = parametric, : zero-width neighborhood. make span bigger
```

```
## Warning: Computation failed in `stat_smooth()`:
## NA/NaN/Inf in foreign function call (arg 5)
```

```
## Warning in simpleLoess(y, x, w, span, degree = degree, parametric = parametric, : at -0.005
```

```
## Warning in simpleLoess(y, x, w, span, degree = degree, parametric = parametric, : radius 2.5e-05
```

```
## Warning in simpleLoess(y, x, w, span, degree = degree, parametric = parametric, : all data on boundary of neighborhood. make span bigger
```

```
## Warning in simpleLoess(y, x, w, span, degree = degree, parametric = parametric, : pseudoinverse used at -0.005
```

```
## Warning in simpleLoess(y, x, w, span, degree = degree, parametric = parametric, : neighborhood radius 0.005
```

```
## Warning in simpleLoess(y, x, w, span, degree = degree, parametric = parametric, : reciprocal condition number 1
```

```
## Warning in simpleLoess(y, x, w, span, degree = degree, parametric = parametric, : There are other near singularities as well. 1.01
```

```
## Warning in simpleLoess(y, x, w, span, degree = degree, parametric = parametric, : zero-width neighborhood. make span bigger
```

```
## Warning: Computation failed in `stat_smooth()`:
## NA/NaN/Inf in foreign function call (arg 5)
```

```
## Warning in simpleLoess(y, x, w, span, degree = degree, parametric = parametric, : at -0.005
```

```
## Warning in simpleLoess(y, x, w, span, degree = degree, parametric = parametric, : radius 2.5e-05
```

```
## Warning in simpleLoess(y, x, w, span, degree = degree, parametric = parametric, : all data on boundary of neighborhood. make span bigger
```

```
## Warning in simpleLoess(y, x, w, span, degree = degree, parametric = parametric, : pseudoinverse used at -0.005
```

```
## Warning in simpleLoess(y, x, w, span, degree = degree, parametric = parametric, : neighborhood radius 0.005
```

```
## Warning in simpleLoess(y, x, w, span, degree = degree, parametric = parametric, : reciprocal condition number 1
```

```
## Warning in simpleLoess(y, x, w, span, degree = degree, parametric = parametric, : There are other near singularities as well. 1.01
```

```
## Warning in simpleLoess(y, x, w, span, degree = degree, parametric = parametric, : zero-width neighborhood. make span bigger
```

```
## Warning: Computation failed in `stat_smooth()`:
## NA/NaN/Inf in foreign function call (arg 5)
```

```
## Warning in simpleLoess(y, x, w, span, degree = degree, parametric = parametric, : at -0.005
```

```
## Warning in simpleLoess(y, x, w, span, degree = degree, parametric = parametric, : radius 2.5e-05
```

```
## Warning in simpleLoess(y, x, w, span, degree = degree, parametric = parametric, : all data on boundary of neighborhood. make span bigger
```

```
## Warning in simpleLoess(y, x, w, span, degree = degree, parametric = parametric, : pseudoinverse used at -0.005
```

```
## Warning in simpleLoess(y, x, w, span, degree = degree, parametric = parametric, : neighborhood radius 0.005
```

```
## Warning in simpleLoess(y, x, w, span, degree = degree, parametric = parametric, : reciprocal condition number 1
```

```
## Warning in simpleLoess(y, x, w, span, degree = degree, parametric = parametric, : There are other near singularities as well. 1.01
```

```
## Warning in simpleLoess(y, x, w, span, degree = degree, parametric = parametric, : zero-width neighborhood. make span bigger
```

```
## Warning: Computation failed in `stat_smooth()`:
## NA/NaN/Inf in foreign function call (arg 5)
```

```
## Warning in simpleLoess(y, x, w, span, degree = degree, parametric = parametric, : pseudoinverse used at -0.005
```

```
## Warning in simpleLoess(y, x, w, span, degree = degree, parametric = parametric, : neighborhood radius 1.005
```

```
## Warning in simpleLoess(y, x, w, span, degree = degree, parametric = parametric, : reciprocal condition number 0
```

```
## Warning in simpleLoess(y, x, w, span, degree = degree, parametric = parametric, : There are other near singularities as well. 1.01
```

```
## Warning in predLoess(object$y, object$x, newx = if (is.null(newdata)) object$x else if (is.data.frame(newdata)) as.matrix(model.frame(delete.response(terms(object)), : pseudoinverse used at -0.005
```

```
## Warning in predLoess(object$y, object$x, newx = if (is.null(newdata)) object$x else if (is.data.frame(newdata)) as.matrix(model.frame(delete.response(terms(object)), : neighborhood radius 1.005
```

```
## Warning in predLoess(object$y, object$x, newx = if (is.null(newdata)) object$x else if (is.data.frame(newdata)) as.matrix(model.frame(delete.response(terms(object)), : reciprocal condition number 0
```

```
## Warning in predLoess(object$y, object$x, newx = if (is.null(newdata)) object$x else if (is.data.frame(newdata)) as.matrix(model.frame(delete.response(terms(object)), : There are other near
## singularities as well. 1.01
```

```
## Warning in simpleLoess(y, x, w, span, degree = degree, parametric = parametric, : at -0.005
```

```
## Warning in simpleLoess(y, x, w, span, degree = degree, parametric = parametric, : radius 2.5e-05
```

```
## Warning in simpleLoess(y, x, w, span, degree = degree, parametric = parametric, : all data on boundary of neighborhood. make span bigger
```

```
## Warning in simpleLoess(y, x, w, span, degree = degree, parametric = parametric, : pseudoinverse used at -0.005
```

```
## Warning in simpleLoess(y, x, w, span, degree = degree, parametric = parametric, : neighborhood radius 0.005
```

```
## Warning in simpleLoess(y, x, w, span, degree = degree, parametric = parametric, : reciprocal condition number 1
```

```
## Warning in simpleLoess(y, x, w, span, degree = degree, parametric = parametric, : There are other near singularities as well. 1.01
```

```
## Warning in simpleLoess(y, x, w, span, degree = degree, parametric = parametric, : zero-width neighborhood. make span bigger
```

```
## Warning: Computation failed in `stat_smooth()`:
## NA/NaN/Inf in foreign function call (arg 5)
```

```
## Warning in simpleLoess(y, x, w, span, degree = degree, parametric = parametric, : at -0.005
```

```
## Warning in simpleLoess(y, x, w, span, degree = degree, parametric = parametric, : radius 2.5e-05
```

```
## Warning in simpleLoess(y, x, w, span, degree = degree, parametric = parametric, : all data on boundary of neighborhood. make span bigger
```

```
## Warning in simpleLoess(y, x, w, span, degree = degree, parametric = parametric, : pseudoinverse used at -0.005
```

```
## Warning in simpleLoess(y, x, w, span, degree = degree, parametric = parametric, : neighborhood radius 0.005
```

```
## Warning in simpleLoess(y, x, w, span, degree = degree, parametric = parametric, : reciprocal condition number 1
```

```
## Warning in simpleLoess(y, x, w, span, degree = degree, parametric = parametric, : There are other near singularities as well. 1.01
```

```
## Warning in simpleLoess(y, x, w, span, degree = degree, parametric = parametric, : zero-width neighborhood. make span bigger
```

```
## Warning: Computation failed in `stat_smooth()`:
## NA/NaN/Inf in foreign function call (arg 5)
```

```
## Warning in simpleLoess(y, x, w, span, degree = degree, parametric = parametric, : at -0.005
```

```
## Warning in simpleLoess(y, x, w, span, degree = degree, parametric = parametric, : radius 2.5e-05
```

```
## Warning in simpleLoess(y, x, w, span, degree = degree, parametric = parametric, : all data on boundary of neighborhood. make span bigger
```

```
## Warning in simpleLoess(y, x, w, span, degree = degree, parametric = parametric, : pseudoinverse used at -0.005
```

```
## Warning in simpleLoess(y, x, w, span, degree = degree, parametric = parametric, : neighborhood radius 0.005
```

```
## Warning in simpleLoess(y, x, w, span, degree = degree, parametric = parametric, : reciprocal condition number 1
```

```
## Warning in simpleLoess(y, x, w, span, degree = degree, parametric = parametric, : There are other near singularities as well. 1.01
```

```
## Warning in simpleLoess(y, x, w, span, degree = degree, parametric = parametric, : zero-width neighborhood. make span bigger
```

```
## Warning: Computation failed in `stat_smooth()`:
## NA/NaN/Inf in foreign function call (arg 5)
```

```
## Warning in simpleLoess(y, x, w, span, degree = degree, parametric = parametric, : at -0.005
```

```
## Warning in simpleLoess(y, x, w, span, degree = degree, parametric = parametric, : radius 2.5e-05
```

```
## Warning in simpleLoess(y, x, w, span, degree = degree, parametric = parametric, : all data on boundary of neighborhood. make span bigger
```

```
## Warning in simpleLoess(y, x, w, span, degree = degree, parametric = parametric, : pseudoinverse used at -0.005
```

```
## Warning in simpleLoess(y, x, w, span, degree = degree, parametric = parametric, : neighborhood radius 0.005
```

```
## Warning in simpleLoess(y, x, w, span, degree = degree, parametric = parametric, : reciprocal condition number 1
```

```
## Warning in simpleLoess(y, x, w, span, degree = degree, parametric = parametric, : There are other near singularities as well. 1.01
```

```
## Warning in simpleLoess(y, x, w, span, degree = degree, parametric = parametric, : zero-width neighborhood. make span bigger
```

```
## Warning: Computation failed in `stat_smooth()`:
## NA/NaN/Inf in foreign function call (arg 5)
```

```
## Warning in simpleLoess(y, x, w, span, degree = degree, parametric = parametric, : at -0.005
```

```
## Warning in simpleLoess(y, x, w, span, degree = degree, parametric = parametric, : radius 2.5e-05
```

```
## Warning in simpleLoess(y, x, w, span, degree = degree, parametric = parametric, : all data on boundary of neighborhood. make span bigger
```

```
## Warning in simpleLoess(y, x, w, span, degree = degree, parametric = parametric, : pseudoinverse used at -0.005
```

```
## Warning in simpleLoess(y, x, w, span, degree = degree, parametric = parametric, : neighborhood radius 0.005
```

```
## Warning in simpleLoess(y, x, w, span, degree = degree, parametric = parametric, : reciprocal condition number 1
```

```
## Warning in simpleLoess(y, x, w, span, degree = degree, parametric = parametric, : There are other near singularities as well. 1.01
```

```
## Warning in simpleLoess(y, x, w, span, degree = degree, parametric = parametric, : zero-width neighborhood. make span bigger
```

```
## Warning: Computation failed in `stat_smooth()`:
## NA/NaN/Inf in foreign function call (arg 5)
```

```
## Warning in simpleLoess(y, x, w, span, degree = degree, parametric = parametric, : at -0.005
```

```
## Warning in simpleLoess(y, x, w, span, degree = degree, parametric = parametric, : radius 2.5e-05
```

```
## Warning in simpleLoess(y, x, w, span, degree = degree, parametric = parametric, : all data on boundary of neighborhood. make span bigger
```

```
## Warning in simpleLoess(y, x, w, span, degree = degree, parametric = parametric, : pseudoinverse used at -0.005
```

```
## Warning in simpleLoess(y, x, w, span, degree = degree, parametric = parametric, : neighborhood radius 0.005
```

```
## Warning in simpleLoess(y, x, w, span, degree = degree, parametric = parametric, : reciprocal condition number 1
```

```
## Warning in simpleLoess(y, x, w, span, degree = degree, parametric = parametric, : There are other near singularities as well. 1.01
```

```
## Warning in simpleLoess(y, x, w, span, degree = degree, parametric = parametric, : zero-width neighborhood. make span bigger
```

```
## Warning: Computation failed in `stat_smooth()`:
## NA/NaN/Inf in foreign function call (arg 5)
```

```
## Warning in simpleLoess(y, x, w, span, degree = degree, parametric = parametric, : at -0.005
```

```
## Warning in simpleLoess(y, x, w, span, degree = degree, parametric = parametric, : radius 2.5e-05
```

```
## Warning in simpleLoess(y, x, w, span, degree = degree, parametric = parametric, : all data on boundary of neighborhood. make span bigger
```

```
## Warning in simpleLoess(y, x, w, span, degree = degree, parametric = parametric, : pseudoinverse used at -0.005
```

```
## Warning in simpleLoess(y, x, w, span, degree = degree, parametric = parametric, : neighborhood radius 0.005
```

```
## Warning in simpleLoess(y, x, w, span, degree = degree, parametric = parametric, : reciprocal condition number 1
```

```
## Warning in simpleLoess(y, x, w, span, degree = degree, parametric = parametric, : There are other near singularities as well. 1.01
```

```
## Warning in simpleLoess(y, x, w, span, degree = degree, parametric = parametric, : zero-width neighborhood. make span bigger
```

```
## Warning: Computation failed in `stat_smooth()`:
## NA/NaN/Inf in foreign function call (arg 5)
```

### Perturbations plot

#### PCA of perturbation data

The main variability between the cell lines is due to different receptor expressions (as evidence with the PCA on ligand only stimulations). However, different inhibitors responses also yield an important variability. Overall 3 cell lines (LAN6, SKNAS and CHP212) display similar responses while KELLY, IMR32 and SKNSH each have a unique behaviour.

```
## `stat_bin()` using `bins = 30`. Pick better value with `binwidth`.
## `stat_bin()` using `bins = 30`. Pick better value with `binwidth`.
## `stat_bin()` using `bins = 30`. Pick better value with `binwidth`.
```

| Condition | PC1 |
| --- | --- |
| PDGF+ALKi+IGF1i\_AKT | 0.3090166 |
| EGF+ALKi+IGF1i\_AKT | 0.2710319 |
| PDGF+ALKi+IGF1i\_S6K | 0.2109195 |
| EGF+ALKi+IGF1i\_S6K | 0.2069752 |
| IGF1+RAFi+PDGFi\_AKT | 0.1964089 |
| IGF1+mTORC1i\_AKT | 0.1932274 |
| ALKi+IGF1i\_AKT | 0.1866009 |
| EGF+RAFi+PDGFi\_S6K | 0.1819083 |
| IGF1+RAFi+PDGFi\_S6K | 0.1813362 |
| EGF+AKTi\_S6K | 0.1672964 |
| IGF1+MEKi\_AKT | 0.1609648 |
| EGF+RAFi+PDGFi\_AKT | 0.1572154 |

| Condition | PC2 |
| --- | --- |
| IGF1+MEKi\_MEK | 0.3021081 |
| NGF+MEKi\_MEK | 0.2722556 |
| IGF1+ASK1i\_MEK | 0.2577218 |
| PDGF+MEKi\_MEK | 0.2293835 |
| EGF+MEKi\_MEK | 0.2141076 |
| MEKi\_MEK | 0.2084489 |
| IGF1+PI3Ki\_AKT | 0.1445430 |
| ALKi+IGF1i\_S6K | 0.1362246 |

| Condition | PC3 |
| --- | --- |
| IGF1+ASK1i\_MEK | -0.2349953 |
| IGF1+PI3Ki\_AKT | -0.2125787 |
| IGF1+mTORC1i\_S6K | -0.2103491 |
| PDGF+ASK1i\_S6K | -0.2091652 |
| PDGF+mTORC1i\_S6K | -0.2078444 |
| NGF+mTORC1i\_S6K | -0.2015831 |
| IGF1\_AKT | -0.1998183 |
| EGF+PI3Ki\_S6K | 0.1834519 |
| EGF+AKTi\_S6K | 0.1796845 |
| IGF1+MEKi\_AKT | -0.1791804 |
| IGF1+ASK1i\_AKT | -0.1785039 |
| IGF1+ASK1i\_S6K | -0.1632860 |
| EGF+mTORC1i\_S6K | -0.1629871 |
| ASK1i\_S6K | -0.1536589 |
| IGF1+ASK1i\_cJUN | -0.1440812 |
| IGF1+AKTi\_S6K | 0.1409907 |

```
## 
## ---------------------
## Welcome to dendextend version 1.13.4
## Type citation('dendextend') for how to cite the package.
## 
## Type browseVignettes(package = 'dendextend') for the package vignette.
## The github page is: https://github.com/talgalili/dendextend/
## 
## Suggestions and bug-reports can be submitted at: https://github.com/talgalili/dendextend/issues
## Or contact: <tal.galili@gmail.com>
## 
##  To suppress this message use:  suppressPackageStartupMessages(library(dendextend))
## ---------------------
```

```
## 
## Attaching package: 'dendextend'
```

```
## The following object is masked from 'package:stats':
## 
##     cutree
```

```
## 
## Attaching package: 'ggdendro'
```

```
## The following object is masked from 'package:dendextend':
## 
##     theme_dendro
```

```
## Warning in RColorBrewer::brewer.pal(length(color_range), "Set1"): n too large, allowed maximum for palette Set1 is 9
## Returning the palette you asked for with that many colors
```

```
## Warning: The input is a data frame, convert it to the matrix.
```

#### Overview of perturbation data

```
## `summarise()` ungrouping output (override with `.groups` argument)
```

```
## [1] "DMSO"       "PI3Ki"      "AKTi"       "mTORC1i"    "ALKi+IGF1i" "RAFi+PDGFi" "MEKi"       "ASK1i"
```

### Comparison perturbation/IC50

#### Readouts upon MEKi vs AZD6244 IC50 MEK upon MEKi looks promising but the error bars are too big for some cell lines (only 2 replicates)

```
## function(data, title="") {
##     }
```

#### Readouts vs AZD6244

```
## `summarise()` ungrouping output (override with `.groups` argument)
```

#### Readouts vs other drugs

### Comparison perturbation/expression
