# Supplementary material for: Neuroblastoma signalling models unveil combination therapies targeting feedback-mediated resistance
Source: PLoS Comput Biol. 2021 Nov 4;17(11):e1009515. doi: 10.1371/journal.pcbi.1009515 (PMC8604339; doi:10.1371/journal.pcbi.1009515)

Summary of model extensions

Mathurin Dorel

2021-08-30

Starting from a linear topology plus the well documented ERK->RAF feedback, the models are refined for each cell line using the results from `suggestExtension`.

To remove non identifiability linked to not measuring all inhibited nodes, we fix the inhibitors to concensus values and refit with this constraint.

1 SKNSH fitting summary

1.1 Initial linear model SKNSH

```
## [1] "SKNSH split, residual= 650"
```

adj_idx	from	to	value	residual	df	Res_delta	df_delta	pval	adj_pval
97	IGF1	ALK	-102.5191303	461.2126	34	188.43377	1	0	0
358	p38	TRKA	1.0000000	563.9925	34	85.65393	1	0	0
344	p38	ALK	0.0000399	565.9349	34	83.71152	1	0	0
130	IL1b	TRKA	0.0000077	568.5123	34	81.13410	1	0	0
282	TAK1	TRKA	0.0000036	568.5123	34	81.13407	1	0	0
149	JNK	TRKA	0.0000036	568.5123	34	81.13407	1	0	0
320	cJUN	TRKA	1.0000000	568.5124	34	81.13406	1	0	0
306	cJUN	ALK	0.0000285	570.3533	34	79.29312	1	0	0
116	IL1b	ALK	0.0000564	570.3533	34	79.29307	1	0	0
268	TAK1	ALK	0.0000251	570.3534	34	79.29306	1	0	0

The fit is very bad, 3 features need explaining: the low phosphorylation of AKT and S6K upon IGF1+ALKi, the low phosphorylation of AKT and S6K upon PDGF+RAFi and the high phosphorylation of AKT and S6K upon IL1b stimulation. Both low phosphorylation are likely due to offtarget activity of TAE684 on IGFRs (suggested by the IGF1->ALK extension) and Sorafenib and PDGFRs respectively so we change the experimental annotation to take it into account.

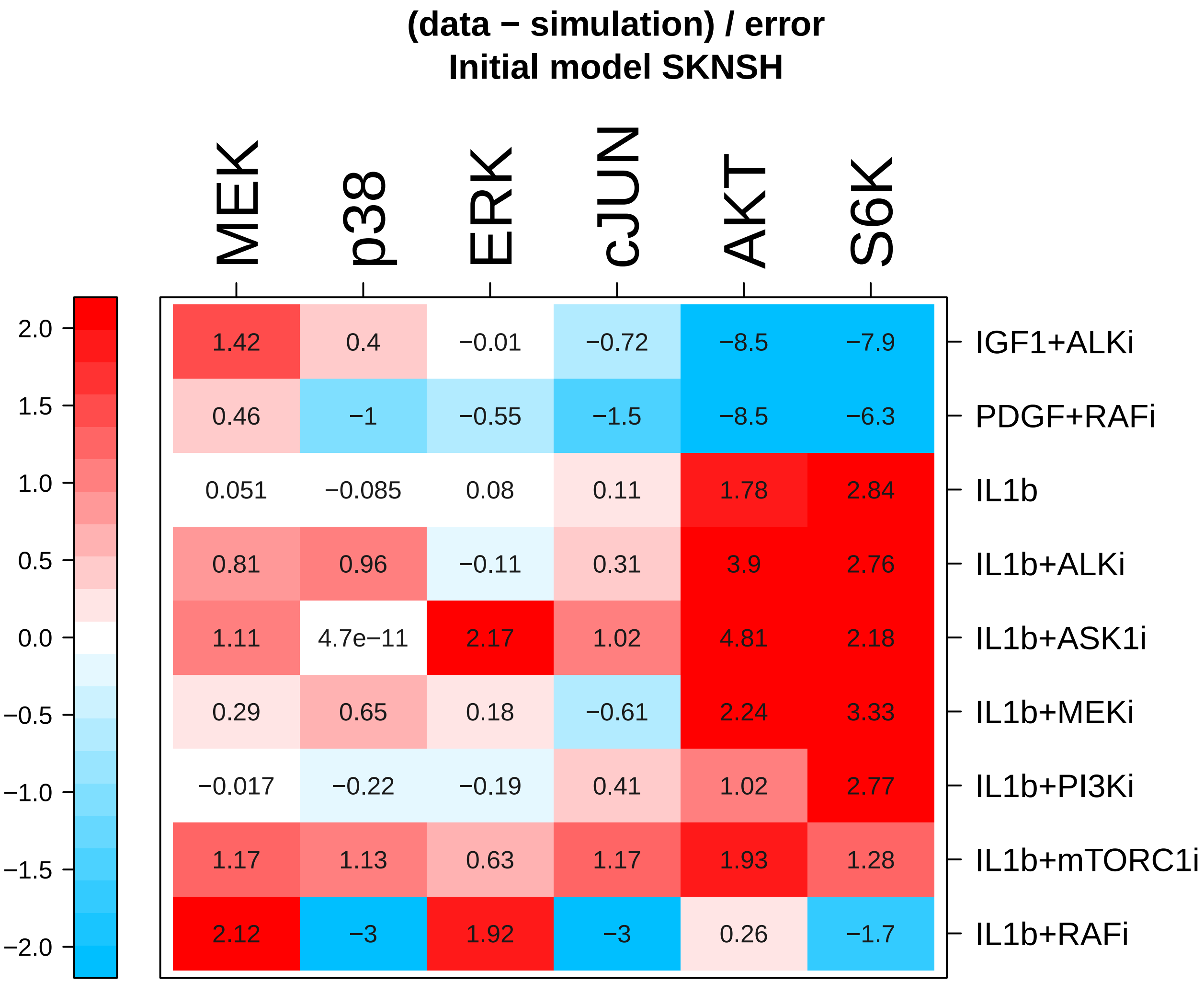

Initial model SKNSH

1.2 Dual inhibitor effect SKNSH

```
## [1] "SKNSH dual, residual= 343"
```

adj_idx	from	to	value	residual	df	Res_delta	df_delta	pval	adj_pval
344	p38	ALK	0.0000847	272.8149	36	70.42725	1	0	0
358	p38	TRKA	1.0000000	273.1494	36	70.09281	1	0	0
116	IL1b	ALK	0.0001702	275.3186	36	67.92359	1	0	0
268	TAK1	ALK	0.0000474	275.3186	36	67.92358	1	0	0
306	cJUN	ALK	0.0000743	275.3186	36	67.92356	1	0	0
135	JNK	ALK	0.0000512	275.3186	36	67.92355	1	0	0
282	TAK1	TRKA	0.0000003	275.5631	36	67.67907	1	0	0
149	JNK	TRKA	0.0000003	275.5631	36	67.67907	1	0	0
130	IL1b	TRKA	0.0000007	275.5631	36	67.67906	1	0	0
320	cJUN	TRKA	1.0000000	275.5633	36	67.67890	1	0	0

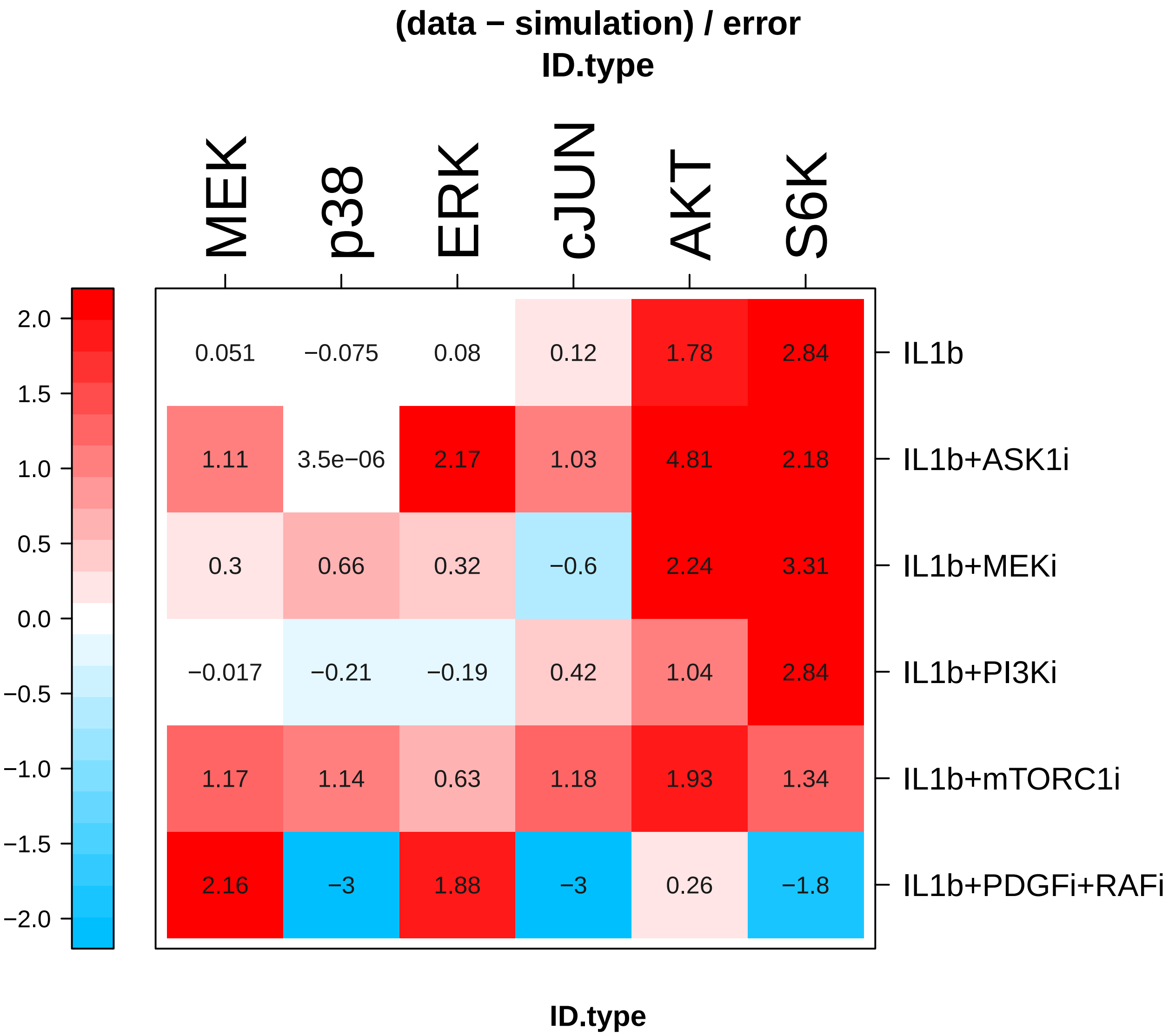

Including the offtarget effects of the inhibitors solved the low phosphorylation problem. To explain the activation of the PI3K pathway by IL1b would require a link from IL1b to this pathway, and IL1b->PI3K was deemed most relevant. The links proposed by the extension would have the similar effect (downstream targets of IL1b activating receptors of the PI3K and MAPK pathways. However the activation of the MAPK1 pathway by IL1b is not supported by the data.

1.3 IL1b->PI3K SKNSH

```
## [1] "SKNSH il1b_pi3k, residual= 313"
```

adj_idx	from	to	value	residual	df	Res_delta	df_delta	pval	adj_pval
84	ERK	JNK	0.3559470	295.8062	37	17.55467	1	0.0000279	0.0061975
160	MEK	JNK	0.3257608	295.8062	37	17.55466	1	0.0000279	0.0061975
93	ERK	cJUN	0.3559364	295.8062	37	17.55466	1	0.0000279	0.0061975
169	MEK	cJUN	0.3257517	295.8062	37	17.55466	1	0.0000279	0.0061975
52	ASK1	S6K	2322.8531923	296.0318	37	17.32908	1	0.0000314	0.0069783
56	ASK1	mTORC1	2529.2115499	297.8346	37	15.52623	1	0.0000814	0.0180638
344	p38	ALK	0.0001190	302.1947	37	11.16617	1	0.0008330	0.1849317

adj_idx	from	to	value	residual	df	Res_delta	df_delta	pval	adj_pval
358	p38	TRKA	1.0000000	302.4250	37	10.93581	1	0.0009432	0.2093979
24	ALK	ERK	96.3758882	303.0658	37	10.29504	1	0.0013339	0.2961223
351	p38	MEK	0.3063381	303.2777	37	10.08315	1	0.0014963	0.3321808

This extension solved all the remaining major problems. A significant MEK/ERK->JNK/S6K extension is suggested to solve cJUN downregulation upon Sorafenib treatment.

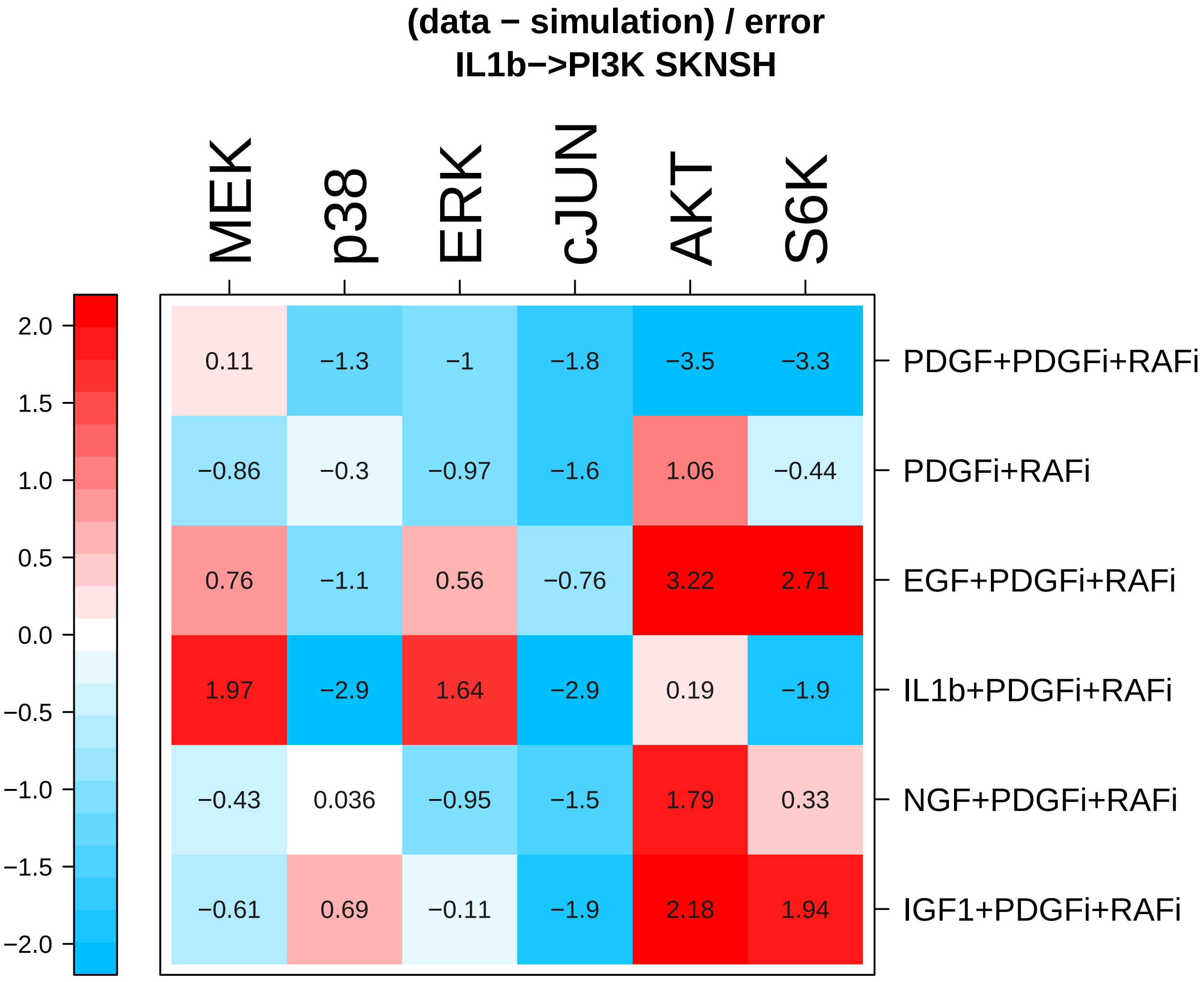

IL1b−>PI3K SKNSH

1.4 ERK->cJUN SKNSH

```
## [1] "SKNSH erk_cjun, resierk_cjun= 267"
```

adj_idx	from	to	value	residual	df	Res_delta	df_delta	pval	adj_pval
230	RAF	ALK	6.8022849	250.0581	38	17.33418	1	0.0000313	0.0069283
358	p38	TRKA	1.0000000	250.8411	38	16.55112	1	0.0000474	0.0104657
344	p38	ALK	0.0001033	251.3339	38	16.05832	1	0.0000614	0.0135741
54	ASK1	TRKA	0.0000018	251.3747	38	16.01752	1	0.0000628	0.0138697

adj_idx	from	to	value	residual	df	Res_delta	df_delta	pval	adj_pval
24	ALK	ERK	0.3699002	252.9018	38	14.49045	1	0.0001409	0.0311328
40	ASK1	ALK	-0.0002199	252.9614	38	14.43089	1	0.0001454	0.0321328
306	cJUN	ALK	0.0000670	252.9800	38	14.41223	1	0.0001468	0.0324528
320	cJUN	TRKA	1.0000000	253.0162	38	14.37601	1	0.0001497	0.0330831
351	p38	MEK	0.3751927	254.2288	38	13.16342	1	0.0002855	0.0630884
355	p38	RAF	0.0000047	254.2289	38	13.16335	1	0.0002855	0.0630905

Those effects are seen in all cell lines, so this topology and the dual inhibition actions of TAE684 and Sorafenib are used in all cell lines However here, Sorafenib inhibits p38 and cJUN activation by IL1b which suggests that Sorafenib also inhibits the IL1b receptor.

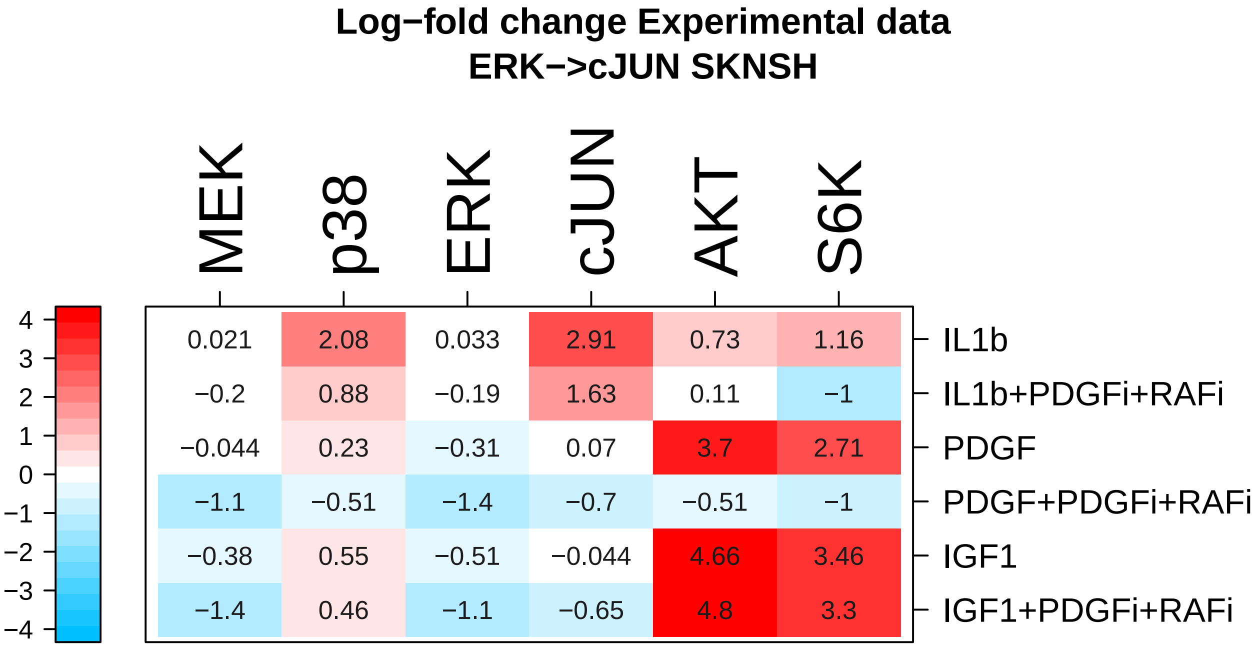

ERK−>cJUN SKNSH

1.5 Sorafenib IL1b offtarget SKNSH

```
## [1] "SKNSH IL1b offtarget model, residual= 241"
```

adj_idx	from	to	value	residual	df	Res_delta	df_delta	pval	adj_pval
52	ASK1	S6K	2952.2799837	221.8276	39	18.79016	1	0.0000146	0.0032248
56	ASK1	mTORC1	1867.7906955	223.6616	39	16.95617	1	0.0000383	0.0084539
24	ALK	ERK	0.5547872	226.4061	39	14.21169	1	0.0001634	0.0361010
147	JNK	S6K	0.0454312	228.4083	39	12.20946	1	0.0004755	0.1050808
280	TAK1	S6K	0.0454344	228.4083	39	12.20946	1	0.0004755	0.1050809
318	cJUN	S6K	0.2053357	228.4083	39	12.20946	1	0.0004755	0.1050809
128	IL1b	S6K	0.4413799	228.5406	39	12.07717	1	0.0005104	0.1128052
356	p38	S6K	0.2877822	228.8501	39	11.76765	1	0.0006027	0.1331942
358	p38	TRKA	1.0000000	229.2391	39	11.37864	1	0.0007429	0.1641885
96	IGF1	AKT	1.1937739	229.4718	39	11.14597	1	0.0008421	0.1861142

SKNSH final network, residual= 241

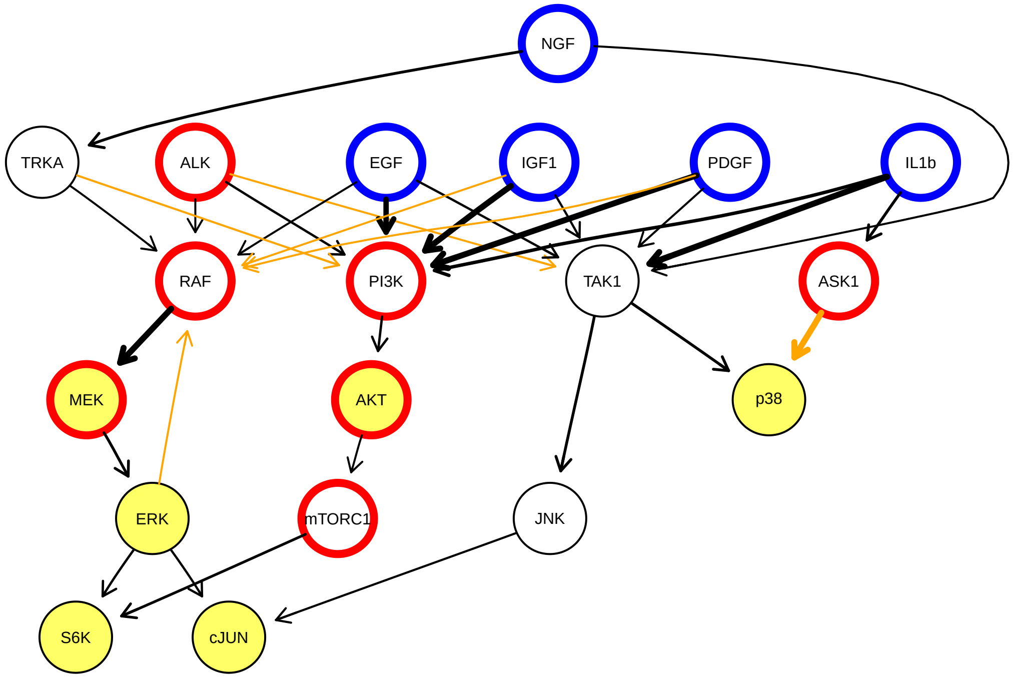

1.6 Final model reduction

Only receptor links would be removed so we keep them for inter-models comparison

```
## Performing model reduction...
## Remove link 4: ALK->TAK1
## New residual =  240.617750053072 , Delta residual =  7.8e-08 ,  p-value =  0.00022
## -----------------------------------------------------------------------------------------
## Remove link 29: TRKA->RAF
## New residual =  240.62185968422 , Delta residual =  0.0041 ,  p-value =  0.051
## -----------------------------------------------------------------------------------------
## Remove link 19: NGF->TAK1
## New residual =  240.747200624224 , Delta residual =  0.13 ,  p-value =  0.28
## -----------------------------------------------------------------------------------------
## Remove link 19: NGF->TRKA
## New residual =  240.940384261203 , Delta residual =  0.19 ,  p-value =  0.34
## --- Other best links ---
## Warning in selectMinimalModel(original_model, accuracy = accuracy): Link TRKA->PI3K belongs to a non-
##     Could remove TRKA->PI3K
##     New residual =  240.940384302818 , Delta residual =  0.19 ,  p-value =  0.34
## -----------------------------------------------------------------------------------------
## Warning in selectMinimalModel(original_model, accuracy = accuracy): Link TRKA->PI3K belongs to a non-
```

```
## Remove link 26: TRKA->PI3K
## New residual =  240.940384261203 , Delta residual =  0 ,  p-value =  0
## ----------------------------------------------------------------------------------------------------
## Remove link 3: ALK->RAF
## New residual =  241.500458287896 , Delta residual =  0.56 ,  p-value =  0.55
## ----------------------------------------------------------------------------------------------------
## Remove link 20: PDGF->TAK1
## New residual =  242.051538896419 , Delta residual =  0.55 ,  p-value =  0.54
## ----------------------------------------------------------------------------------------------------
## Remove link 11: IGF1->RAF
## New residual =  243.900954975041 , Delta residual =  1.85 ,  p-value =  0.83
## ----------------------------------------------------------------------------------------------------
## Reduction complete
## [1] "Best fit: 243.9 , Score= 0.89"
```

1.7 Final SKNSH model with fixed parameters

```
## [1] "SKNSH fixed model, residual= 317"
```

Fixing the inhibitor parameters is necessary to compare the models between cell lines because they create some non identifiability when non measured nodes are inhibited. The likelihood profiles for the final topology show many non identifiable parameters which become identifiable after fixing the inhibitor parameters. The value for the inhibitor parameters have been chosen after a good model for all cell lines was found and set to the majority value.

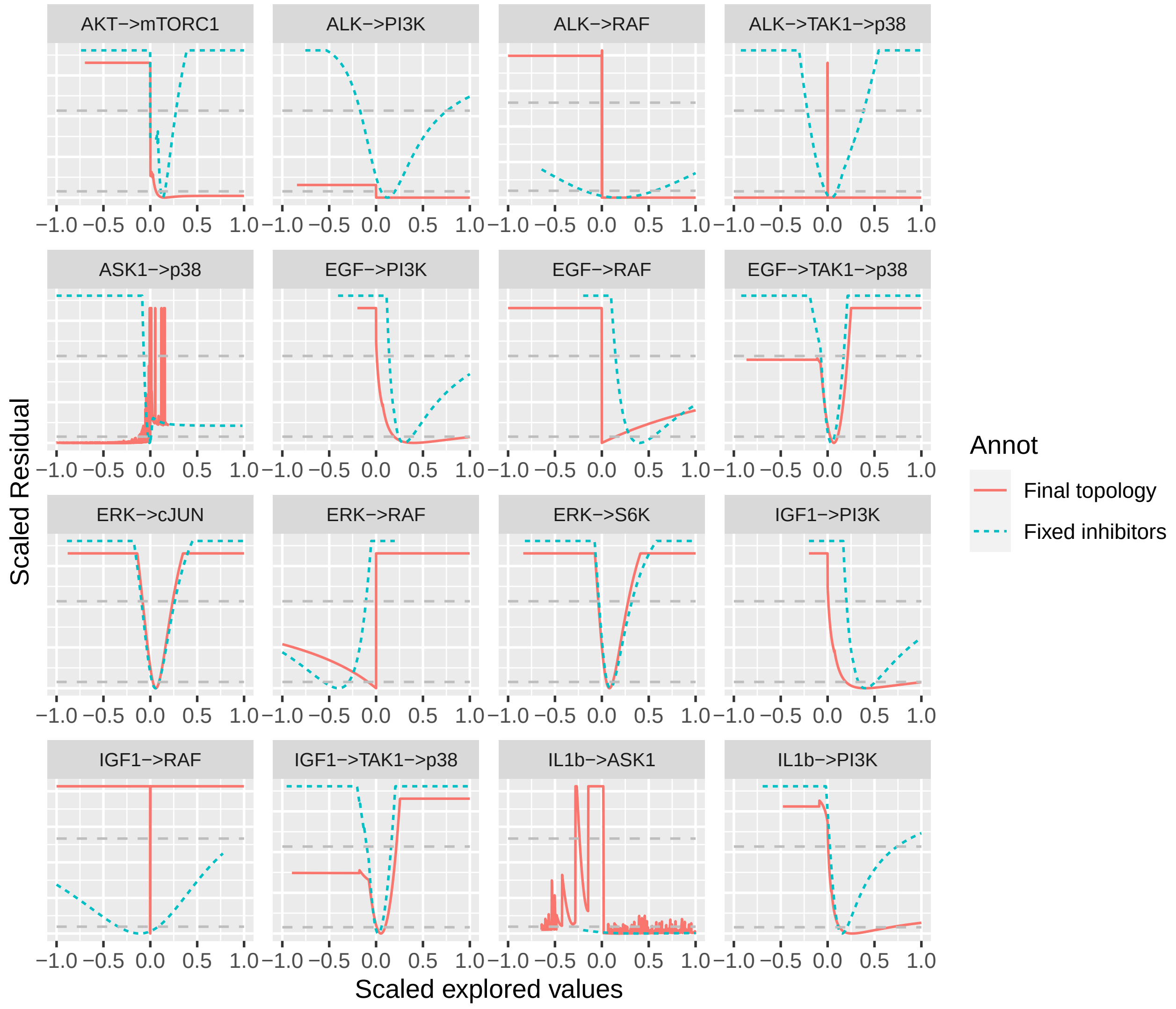
AKT−>mTORC1
ALK−>PI3K
ALK−>RAF
ALK−>TAK1−>p38
ASK1−>p38
EGF−>PI3K
EGF−>RAF
EGF−>TAK1−>p38
ERK−>cJUN
ERK−>RAF
ERK−>S6K
IGF1−>PI3K
IGF1−>RAF
IGF1−>TAK1−>p38
IL1b−>ASK1
IL1b−>PI3K
−1.0 −0.5 0.0 0.5 1.0
Scaled Residual
Scaled explored values
Annot
Final topology
Fixed inhibitors

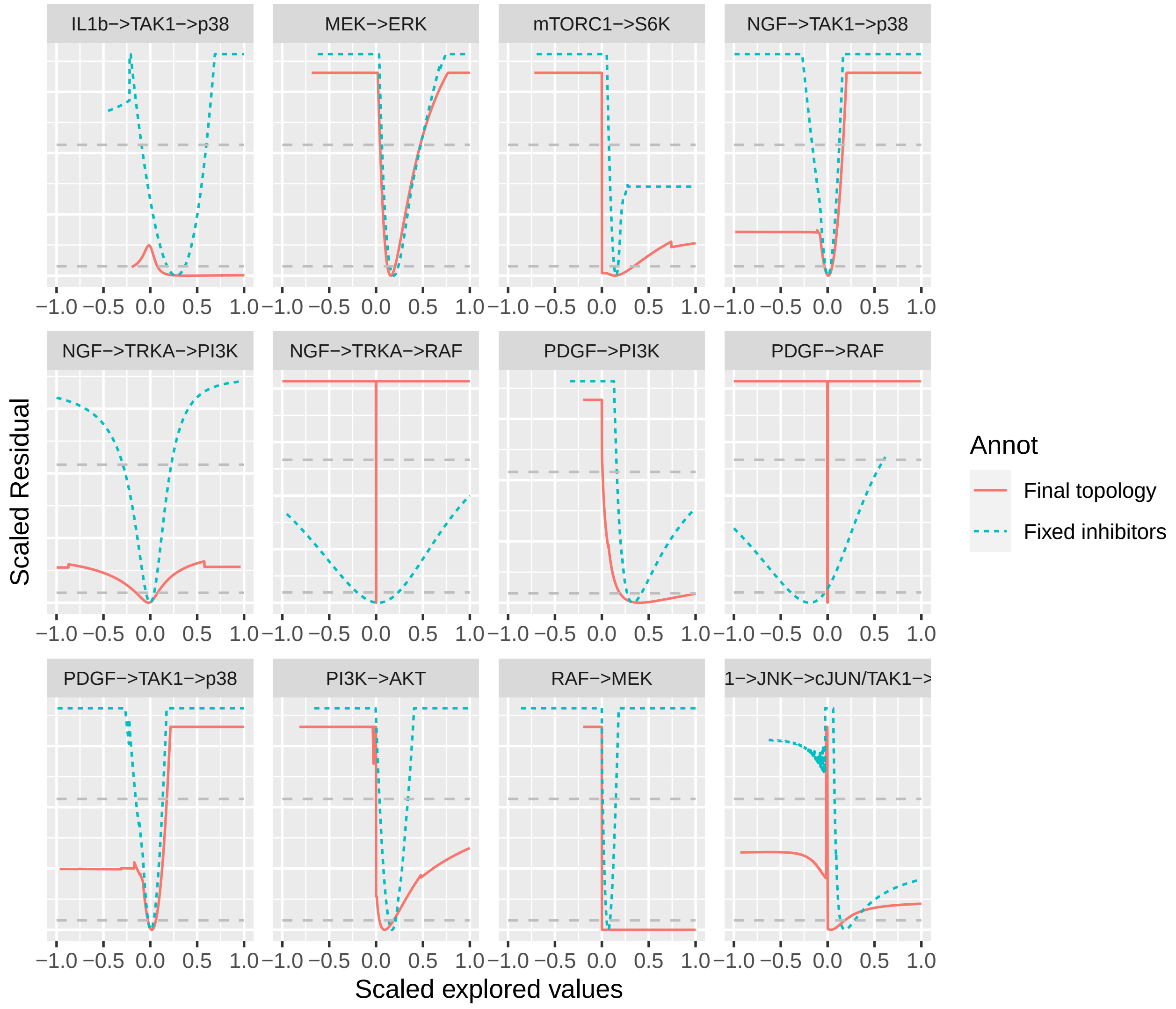

2 CHP212 fitting summary

CHP212 was fitted with a PDGF basal activity

2.1 Initial topology CHP212

```
## [1] "CHP212 initial, residual= 278"
```

adj_idx	from	to	value	residual	df	Res_delta	df_delta	pval	adj_pval
360	p38	mTORC1	-53.37000	253.2	35	24.96	1	6.00e-07	0.0001400
356	p38	S6K	-3.34200	254.2	35	24.00	1	1.00e-06	0.0002297
52	ASK1	S6K	8.80300	258.9	35	19.24	1	1.15e-05	0.0027520
56	ASK1	mTORC1	3.68900	258.9	35	19.24	1	1.15e-05	0.0027520
338	mTORC1	TAK1	1.00000	259.2	35	18.93	1	1.36e-05	0.0032450
262	S6K	TAK1	-0.06643	259.9	35	18.30	1	1.89e-05	0.0045140
260	S6K	RAF	3.55000	261.9	35	16.22	1	5.65e-05	0.0135000
336	mTORC1	RAF	0.13150	262.0	35	16.20	1	5.71e-05	0.0136500
264	S6K	cJUN	0.13560	262.2	35	15.92	1	6.61e-05	0.0157900

adj_idx	from	to	value	residual	df	Res_delta	df_delta	pval	adj_pval
331	mTORC1	JNK	0.00764	262.2	35	15.92	1	6.61e-05	0.0157900

Best extensions are ASK1/p38 -> mTORC1/S6K. p38->S6K was choosen because it was found in other cell lines.

2.2 p38->S6K effect CHP212

```
## [1] "CHP212 p38->S6K, residual= 254"
```

adj_idx	from	to	value	residual	df	Res_delta	df_delta	pval	adj_pval
344	p38	ALK	0.1031	200.9	36	53.14	1	0.00e+00	0.0000000
40	ASK1	ALK	-5.3210	201.0	36	53.04	1	0.00e+00	0.0000000
358	p38	TRKA	1.0000	204.8	36	49.24	1	0.00e+00	0.0000000
343	p38	AKT	4.2090	212.7	36	41.39	1	0.00e+00	0.0000000
50	ASK1	PI3K	-101.1000	212.9	36	41.14	1	0.00e+00	0.0000000
39	ASK1	AKT	-5.8960	212.9	36	41.14	1	0.00e+00	0.0000000
354	p38	PI3K	5.5950	212.9	36	41.14	1	0.00e+00	0.0000000
54	ASK1	TRKA	138.2000	214.0	36	40.10	1	0.00e+00	0.0000001
338	mTORC1	TAK1	1.0000	235.2	36	18.84	1	1.42e-05	0.0033780
336	mTORC1	RAF	4.6610	236.7	36	17.40	1	3.03e-05	0.0072240

ASK1i still had unexplained effects on AKT and MEK. The proposed extensions address these problems. The links to the receptor are unlikely, p38/ASK1->AKT is then the best option.

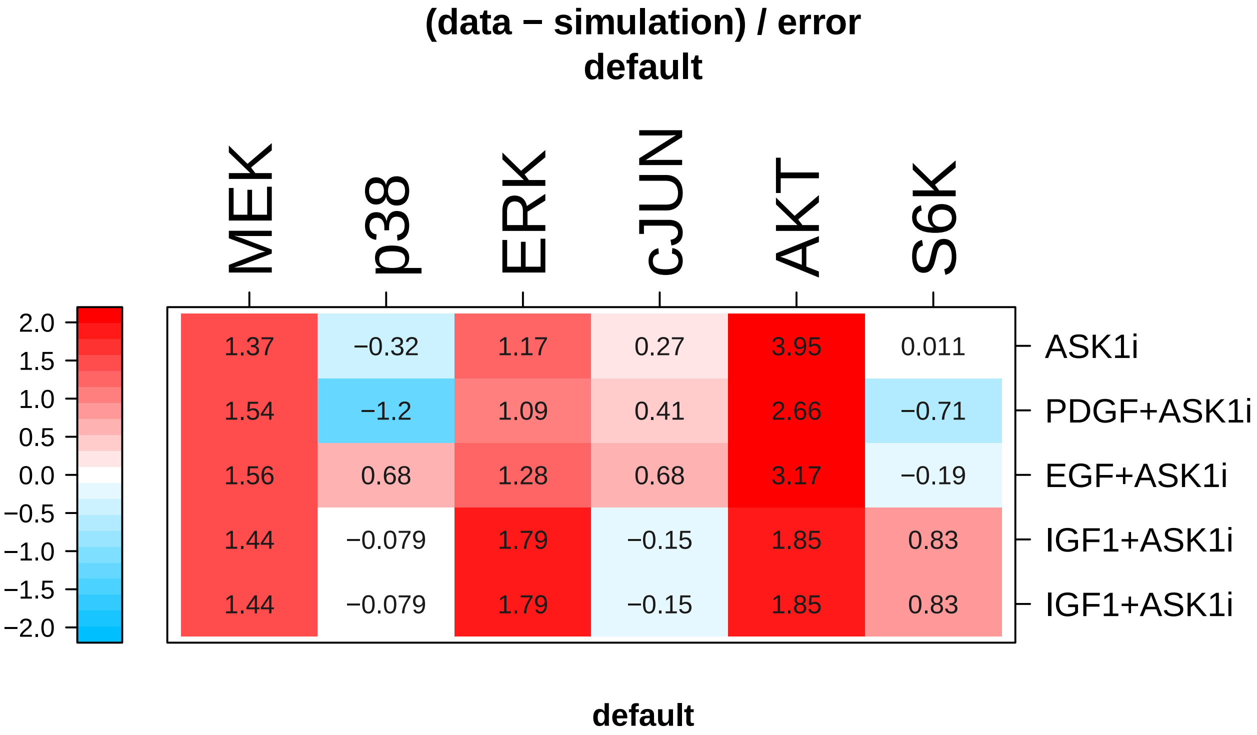

2.3 ASK1->AKT effect CHP212

```
## [1] "CHP212 ASK1->AKT, residual= 214"
```

adj_idx	from	to	value	residual	df	Res_delta	df_delta	pval	adj_pval
339	mTORC1	TRKA	1.00e+00	188	37	25.3	1	5.00e-07	0.000118
336	mTORC1	RAF	5.34e+00	189	37	24.6	1	7.00e-07	0.000169
332	mTORC1	MEK	1.71e-01	191	37	22.8	1	1.80e-06	0.000427
338	mTORC1	TAK1	1.00e+00	192	37	22.1	1	2.60e-06	0.000619
262	S6K	TAK1	-4.67e-02	192	37	21.8	1	3.10e-06	0.000732
2	AKT	ALK	1.45e+00	192	37	21.7	1	3.20e-06	0.000755
263	S6K	TRKA	7.50e+00	196	37	17.7	1	2.53e-05	0.005990
255	S6K	JNK	1.40e-01	196	37	17.5	1	2.82e-05	0.006680
264	S6K	cJUN	1.40e-01	196	37	17.5	1	2.82e-05	0.006680
331	mTORC1	JNK	9.13e-02	197	37	17.0	1	3.81e-05	0.009040
340	mTORC1	cJUN	9.14e-02	197	37	17.0	1	3.81e-05	0.009040
309	cJUN	ERK	5.72e+00	198	37	15.6	1	7.62e-05	0.018100
271	TAK1	ERK	-2.09e+05	198	37	15.5	1	8.28e-05	0.019600
138	JNK	ERK	-1.64e+05	198	37	15.5	1	8.28e-05	0.019600
351	p38	MEK	3.53e+00	199	37	15.0	1	1.05e-04	0.024800
40	ASK1	ALK	-9.98e+01	199	37	15.0	1	1.06e-04	0.025100
344	p38	ALK	6.96e-04	199	37	15.0	1	1.06e-04	0.025100
47	ASK1	MEK	-7.91e+00	199	37	15.0	1	1.07e-04	0.025300
260	S6K	RAF	2.91e+01	199	37	15.0	1	1.07e-04	0.025300
54	ASK1	TRKA	-1.32e+01	199	37	14.9	1	1.11e-04	0.026300

This fit is good. The first extensions suggested would explain the high pMEK upon PI3Ki, AKTi and mTORC1i. However this is likely an overfitting, as the residuals are very low and the signals are not consistent, it would also fail to explain the low pERK upon those same inhibitions. Similarly mTORC1/S6K->TAK1/JNK/cJUN aims at explaining the slight downregulation of cJUN. Overall it appears like the only truly unexplained signal is MEK and ERK upregulation upon ASK1i so ASK1->MEK is selected.

2.4 ASK1->MEK effect CHP212

None of the best extensions significantly improve the model. There is however a strong suggestion of a crosstalk from mTORC1/S6K to JNK/cJUN.

```
## [1] "CHP212 ASK1->MEK, residual= 199"
```

adj_idx	from	to	value	residual	df	Res_delta	df_delta	pval	adj_pval
262	S6K	TAK1	-0.04431	178.4	38	20.99	1	0.0000046	0.001092
347	p38	ERK	-81.59000	178.4	38	20.97	1	0.0000047	0.001098
338	mTORC1	TAK1	1.00000	179.1	38	20.33	1	0.0000065	0.001534
264	S6K	cJUN	0.13950	182.1	38	17.32	1	0.0000316	0.007451
255	S6K	JNK	0.13950	182.1	38	17.32	1	0.0000316	0.007451
263	S6K	TRKA	7.06600	183.3	38	16.16	1	0.0000582	0.013730
340	mTORC1	cJUN	0.08874	184.2	38	15.26	1	0.0000938	0.022130
331	mTORC1	JNK	0.08874	184.2	38	15.26	1	0.0000938	0.022130
309	cJUN	ERK	5.40500	185.7	38	13.72	1	0.0002117	0.049950
271	TAK1	ERK	-276.00000	185.7	38	13.71	1	0.0002137	0.050430

The model is already slightly overfitted, and all new extensions are thus likely overfitting. The 3 exceptionnally badly fitted data points (p38 in PI3Ki and EGF+PDGFi+RAFi, and S6K in IGF1+PDGFi+RAFi) are likely artefacts since similar perturbations do not show the same effect.

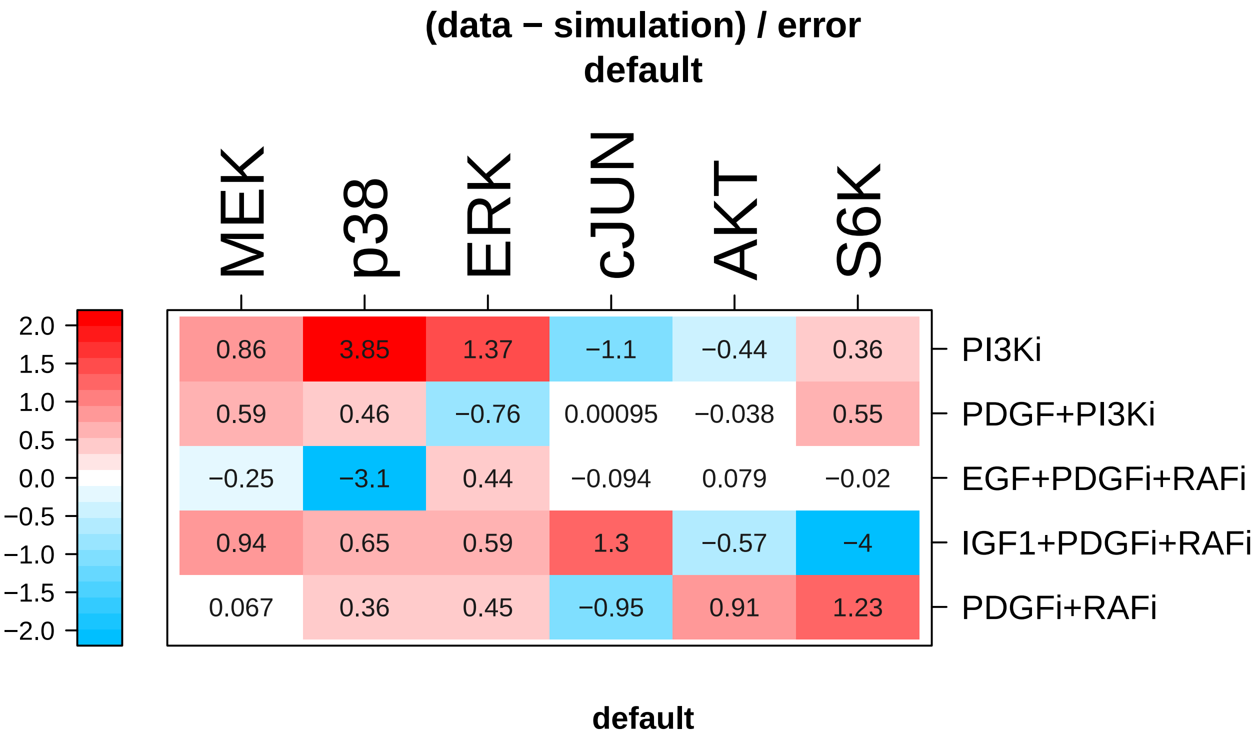

2.5 Final model reduction

Only receptor links would be removed so we keep them for inter-models comparison. IL1b ends up completely disconnected.

```
## Performing model reduction...
## Warning in selectMinimalModel(original_model, accuracy = accuracy): Negative delta residual : -0.785
## Remove link 10: EGF->TAK1
## New residual =  198.63615063827 , Delta residual =  0.79 ,  p-value =  0.62
## -----------------------------------------------------------------------------------------------
## Warning in selectMinimalModel(original_model, accuracy = accuracy): Link IL1b->PI3K belongs to a non
## Remove link 17: IL1b->PI3K
## New residual =  198.63615063827 , Delta residual =  2.8e-14 ,  p-value =  1.3e-07
## --- Other best links ---
## Warning in selectMinimalModel(original_model, accuracy = accuracy): Link IL1b->TAK1 belongs to a non
##     Could remove IL1b->TAK1
##     New residual =  198.63615063827 , Delta residual =  2.8e-14 ,  p-value =  1.3e-07
## Warning in selectMinimalModel(original_model, accuracy = accuracy): Link IL1b->ASK1 belongs to a non
##     Could remove IL1b->ASK1
##     New residual =  198.63615063827 , Delta residual =  5.7e-14 ,  p-value =  1.9e-07
## -----------------------------------------------------------------------------------------------
## Warning in selectMinimalModel(original_model, accuracy = accuracy): Link IL1b->ASK1 belongs to a non
```

```
## Remove link 16: IL1b->ASK1
## New residual =  198.63615063827 , Delta residual =  0 ,  p-value =  0
## --- Other best links ---
## Warning in selectMinimalModel(original_model, accuracy = accuracy): Link IL1b->TAK1 belongs to a non-
##     Could remove IL1b->TAK1
##     New residual =  198.63615063827 , Delta residual =  2.8e-14 ,  p-value =  1.3e-07
## ----------------------------------------------------------------------------------------------------
## Warning in selectMinimalModel(original_model, accuracy = accuracy): Link IL1b->TAK1 belongs to a non-
## Remove link 16: IL1b->TAK1
## New residual =  198.63615063827 , Delta residual =  0 ,  p-value =  0
## ----------------------------------------------------------------------------------------------------
## Remove link 4: ALK->TAK1
## New residual =  198.890265574546 , Delta residual =  0.25 ,  p-value =  0.39
## ----------------------------------------------------------------------------------------------------
## Remove link 20: PDGF->RAF
## New residual =  199.327517856584 , Delta residual =  0.44 ,  p-value =  0.49
## ----------------------------------------------------------------------------------------------------
## Remove link 17: NGF->TAK1
## New residual =  200.270020586738 , Delta residual =  0.94 ,  p-value =  0.67
## ----------------------------------------------------------------------------------------------------
## Remove link 13: IGF1->RAF
## New residual =  201.564649916156 , Delta residual =  1.29 ,  p-value =  0.74
## ----------------------------------------------------------------------------------------------------
## Remove link 8: EGF->RAF
## New residual =  204.951111096433 , Delta residual =  3.39 ,  p-value =  0.93
## ----------------------------------------------------------------------------------------------------
## Reduction complete
## [1] "Best fit: 204.95 , Score= 0.69"
```

2.6 Fixed inhibitor model CHP212

```
## [1] "CHP212 fixed inhibitors, residual= 223"
```

CHP212 fixed_ask1_mek, residual= 223

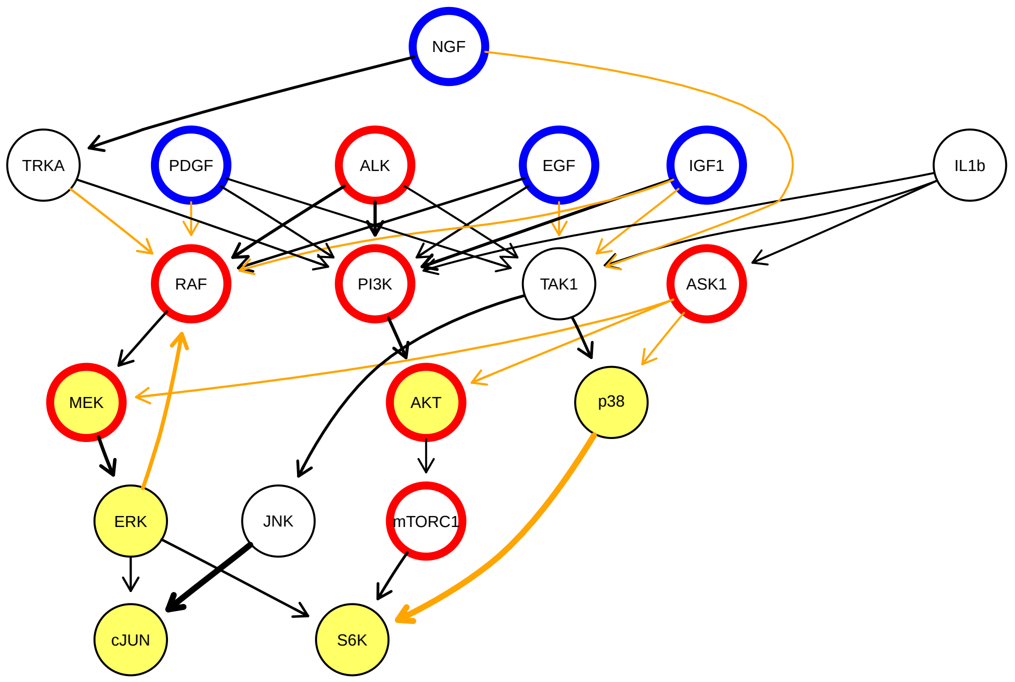

3 SKNAS fitting summary

3.1 Initial topology SKNAS

```
## [1] "SKNAS erk_cjun, residual= 259"
```

adj_idx	from	to	value	residual	df	Res_delta	df_delta	pval	adj_pval
356	p38	S6K	-2.638664	231.0003	35	27.57181	1	2.00e-07	0.0000362
360	p38	mTORC1	-2.459182	231.5457	35	27.02640	1	2.00e-07	0.0000480
318	cJUN	S6K	-1.270095	235.8997	35	22.67240	1	1.90e-06	0.0004591
147	JNK	S6K	-3.999854	235.9740	35	22.59811	1	2.00e-06	0.0004772
280	TAK1	S6K	-3.628994	236.0112	35	22.56087	1	2.00e-06	0.0004866
151	JNK	mTORC1	-4.386238	236.4539	35	22.11817	1	2.60e-06	0.0006127
284	TAK1	mTORC1	-3.340404	236.5791	35	21.99302	1	2.70e-06	0.0006540
322	cJUN	mTORC1	-1.071527	236.7154	35	21.85666	1	2.90e-06	0.0007022
235	RAF	IL1b	1.000000	237.5660	37	21.00610	3	1.05e-04	0.0250877
305	cJUN	AKT	-13.072864	241.0257	35	17.54639	1	2.80e-05	0.0067011

Overall trying to explain the effect of ASK1i on S6K. The best extension p38->S6K makes sense and is used.

3.2 p38->S6K effect SKNAS

```
## [1] "SKNAS p38_s6k, residual= 230"
```

adj_idx	from	to	value	residual	df	Res_delta	df_delta	pval	adj_pval
37	ALK	mTORC1	-5.8836004	207.3902	36	23.06493	1	0.0000016	0.0003728
33	ALK	S6K	-5.4706611	207.4310	36	23.02406	1	0.0000016	0.0003808
7	AKT	IL1b	1.0000000	207.9484	38	22.50672	3	0.0000512	0.0121772

adj_idx	from	to	value	residual	df	Res_delta	df_delta	pval	adj_pval
15	AKT	TAK1	-0.0497459	208.2691	36	22.18605	1	0.0000025	0.0005890
2	AKT	ALK	-0.0710658	209.7469	36	20.70821	1	0.0000053	0.0012730
17	AKT	cJUN	-0.1364757	209.7559	36	20.69919	1	0.0000054	0.0012790
8	AKT	JNK	-0.1317973	209.8701	36	20.58497	1	0.0000057	0.0013576
254	S6K	IL1b	1.0000000	210.8900	38	19.56505	3	0.0002089	0.0497122
330	mTORC1	IL1b	1.0000000	210.8940	38	19.56106	3	0.0002093	0.0498069
22	ALK	ASK1	-99.9804163	211.6246	36	18.83053	1	0.0000143	0.0034001

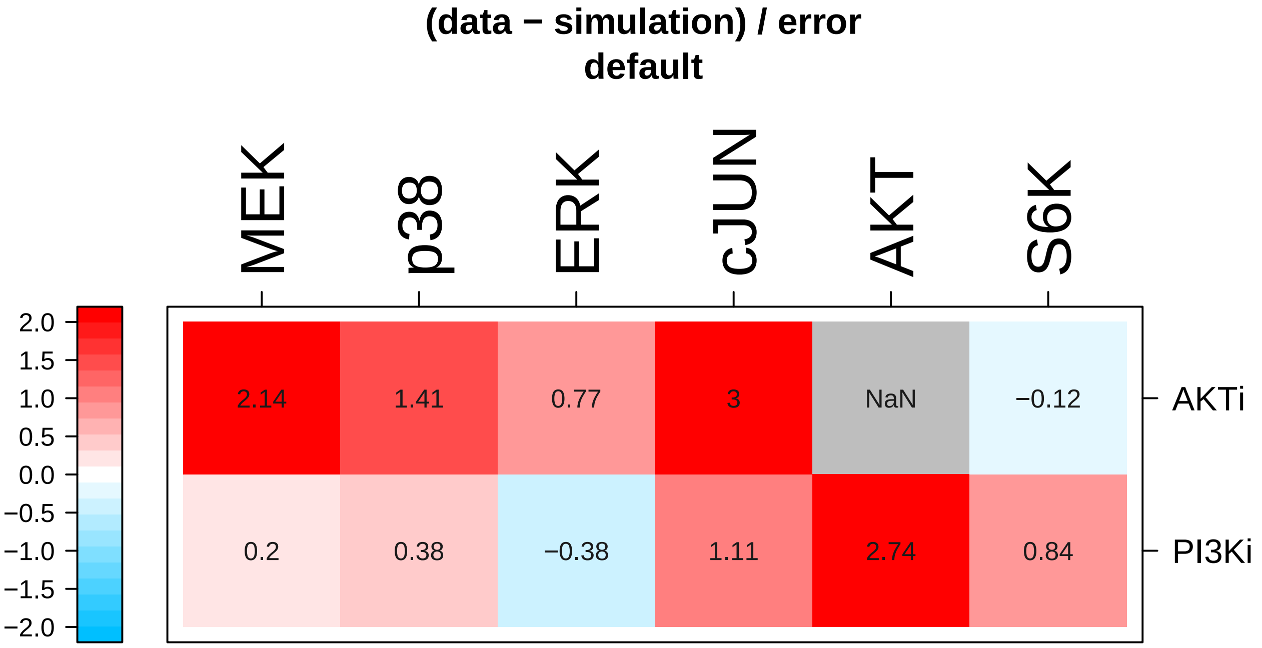

default

The “shortcut” extensions are ignored. The next best extensions correspond to AKT influencing the TAK1/JNK/cJUN axis. AKT->TAK1 has some literature basis and is thus used.

3.3 AKT->TAK1 effect SKNAS

```
## [1] "SKNAS akt_tak1, residual= 208"
```

adj_idx	from	to	value	residual	df	Res_delta	df_delta	pval	adj_pval
235	RAF	IL1b	1.0000000	190.2523	39	18.02943	3	0.0004337	0.1027973
242	RAF	S6K	1.3257914	190.6705	37	17.61128	1	0.0000271	0.0064221
246	RAF	mTORC1	1.7589167	190.7779	37	17.50385	1	0.0000287	0.0067954
243	RAF	TAK1	-1.2678504	191.6396	37	16.64214	1	0.0000451	0.0106975
54	ASK1	TRKA	-0.2352968	191.7310	37	16.55082	1	0.0000474	0.0112252
96	IGF1	AKT	1.2139933	192.0479	37	16.23391	1	0.0000560	0.0132680
358	p38	TRKA	1.0000000	192.3216	37	15.96013	1	0.0000647	0.0153317
33	ALK	S6K	-12.0632868	192.7295	37	15.55232	1	0.0000803	0.0190199
37	ALK	mTORC1	-7.1272104	192.9574	37	15.32440	1	0.0000905	0.0214578
36	ALK	cJUN	-6.4011646	194.6956	37	13.58620	1	0.0002279	0.0540015

The extensions proposed are barely significant and consist in shortcuts. The extension process is stopped at this iteration.

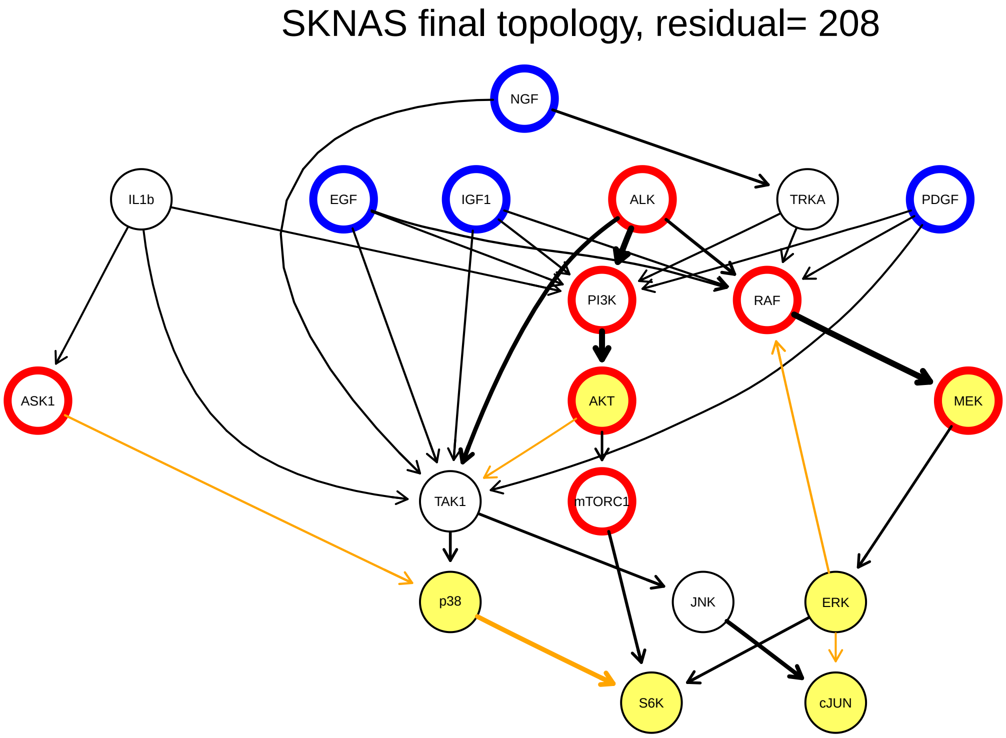

3.4 Final model reduction

All links from the IL1b receptor would be removed so we keep them for inter-models comparison. SKNAS is also the only cell line where cJUN phosphorylation does not seem affected by ERK.

```
## Performing model reduction...

## Warning in selectMinimalModel(original_model, accuracy = accuracy): Link IL1b->ASK1 belongs to a non

## Warning in selectMinimalModel(original_model, accuracy = accuracy): Negative delta residual : -2.842

## Remove link 16: IL1b->ASK1

## New residual =  208.28177608131 , Delta residual =  2.8e-14 ,  p-value =  1.3e-07

## --- Other best links ---

## Warning in selectMinimalModel(original_model, accuracy = accuracy): Link IL1b->PI3K belongs to a non

##     Could remove IL1b->PI3K

##     New residual =  208.28177608131 , Delta residual =  -2.8e-14 ,  p-value =  0

## Warning in selectMinimalModel(original_model, accuracy = accuracy): Link IL1b->TAK1 belongs to a non

##     Could remove IL1b->TAK1

##     New residual =  208.28177608131 , Delta residual =  0 ,  p-value =  0

## ----------------------------------------------------------------------------------------------------

## Warning in selectMinimalModel(original_model, accuracy = accuracy): Link IL1b->TAK1 belongs to a non

## Remove link 17: IL1b->TAK1

## New residual =  208.28177608131 , Delta residual =  0 ,  p-value =  0
```

```
## --- Other best links ---
## Warning in selectMinimalModel(original_model, accuracy = accuracy): Link IL1b->PI3K belongs to a non-
##     Could remove IL1b->PI3K
##     New residual =  208.28177608131 , Delta residual =  2.8e-14 ,  p-value =  1.3e-07
## -----------------------------------------------------------------------------------------------------
## Warning in selectMinimalModel(original_model, accuracy = accuracy): Link IL1b->PI3K belongs to a non-
## Remove link 16: IL1b->PI3K
## New residual =  208.28177608131 , Delta residual =  0 ,  p-value =  0
## -----------------------------------------------------------------------------------------------------
## Remove link 12: ERK->cJUN
## New residual =  208.352925251737 , Delta residual =  0.071 ,  p-value =  0.21
## -----------------------------------------------------------------------------------------------------
## Reduction complete
## [1] "Best fit: 208.35 , Score= 0.65"
```

3.5 Final network with fixed parameters SKNAS

```
## [1] "SKNAS fixed_akt_tak1, residual= 229"
```

4 LAN6 fitting summary

The models without PDGF basal activity are selected for their ERK->RAF negative feedback. The difference in residuals with PDGF basal models is low.

4.1 Initial topology LAN6

```
## [1] "LAN6 erk_cjun, residual= 339"
```

adj_idx	from	to	value	residual	df	Res_delta	df_delta	pval	adj_pval
356	p38	S6K	-4.428000	224.9	38	113.80	1	0	0
360	p38	mTORC1	-0.194900	227.0	38	111.70	1	0	0
280	TAK1	S6K	-4.739000	232.2	38	106.60	1	0	0
147	JNK	S6K	-4.739000	232.2	38	106.60	1	0	0
318	cJUN	S6K	36.310000	232.2	38	106.60	1	0	0
284	TAK1	mTORC1	-0.592900	233.1	38	105.60	1	0	0
151	JNK	mTORC1	-0.592900	233.1	38	105.60	1	0	0
322	cJUN	mTORC1	0.105000	233.5	38	105.20	1	0	0
320	cJUN	TRKA	1.000000	248.4	38	90.35	1	0	0
306	cJUN	ALK	0.001496	249.1	38	89.63	1	0	0

TAK1/JNK/cJUN/p38->mTORC1/S6K extensions greatly improve the model by explaining the effect of ASK1i on S6K.

4.2 p38->S6K effect LAN6

```
## [1] "LAN6 p38_s6k, residual= 243"
```

adj_idx	from	to	value	residual	df	Res_delta	df_delta	pval	adj_pval
319	p38	RAF	14.82000	205.7	35	37.22	1	0.00e+00	0.0000002
49	ASK1	RAF	-99.95000	205.7	35	37.21	1	0.00e+00	0.0000002
45	ASK1	MEK	-99.85000	205.7	35	37.18	1	0.00e+00	0.0000002
38	ASK1	ALK	-0.88970	218.2	35	24.68	1	7.00e-07	0.0001316
308	p38	ALK	0.56890	222.3	35	20.63	1	5.60e-06	0.0010830
221	RAF	ERK	0.43060	222.5	35	20.42	1	6.20e-06	0.0012040
272	cJUN	ALK	0.12640	223.6	35	19.29	1	1.12e-05	0.0021790
74	ERK	ALK	0.10330	223.7	35	19.22	1	1.17e-05	0.0022640
146	MEK	ALK	0.04924	223.7	35	19.16	1	1.20e-05	0.0023360
156	MEK	PI3K	0.12530	223.7	35	19.14	1	1.21e-05	0.0023540

An ASK1/p38->RAF/MEK extension is suggested to explain MEK and ERK up-phosphorylation upon ASK1i. ASK1->MEK is chosen for consistency with other cell lines and biological sense (MAP3K->MAP2K).

4.3 ASK1->MEK effect LAN6

```
## [1] "LAN6 ask1_mek, residual= 206"
```

adj_idx	from	to	value	residual	df	Res_delta	df_delta	pval	adj_pval
74	ERK	ALK	0.05747	185.9	36	19.78	1	0.0000087	0.001680
146	MEK	ALK	0.02556	185.9	36	19.74	1	0.0000089	0.001717
272	cJUN	ALK	0.07479	186.0	36	19.70	1	0.0000090	0.001745
156	MEK	PI3K	0.10680	186.7	36	18.95	1	0.0000134	0.002589
271	cJUN	AKT	1.33700	186.7	36	18.94	1	0.0000135	0.002597
282	cJUN	PI3K	0.27800	186.7	36	18.94	1	0.0000135	0.002600
84	ERK	PI3K	0.23810	186.7	36	18.92	1	0.0000136	0.002624
73	ERK	AKT	1.17900	186.7	36	18.91	1	0.0000137	0.002639
145	MEK	AKT	0.54480	186.8	36	18.85	1	0.0000141	0.002725
36	ALK	p38	3.54200	190.8	36	14.89	1	0.0001142	0.022040

There are signs of overfitting in the qq-plot and the links proposed do not make any biological sense. They likely represent overadjusting of the remaining badly fitted datapoints. We stop the extension here.

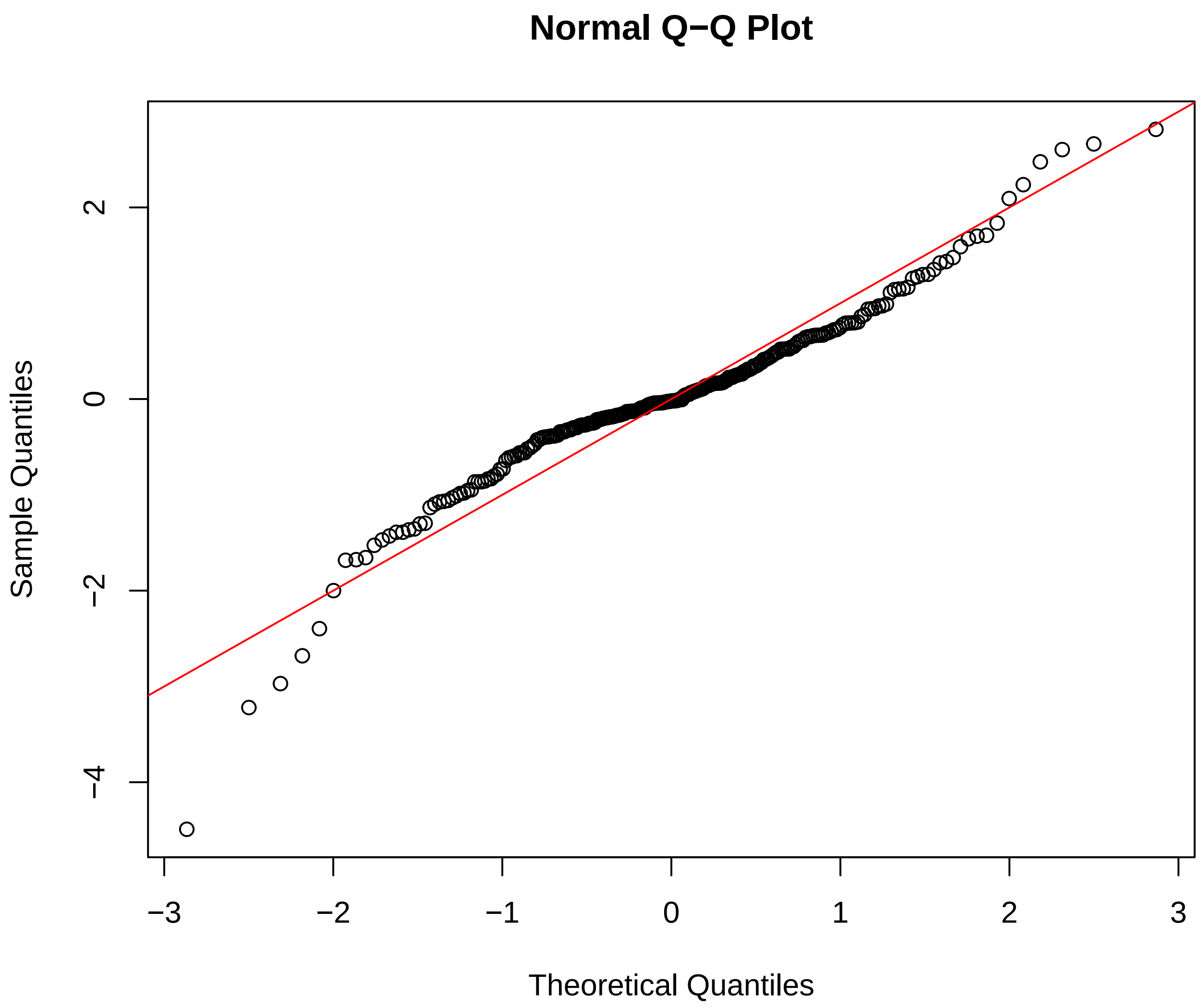
Normal Q−Q Plot
Sample Quantiles
Theoretical Quantiles
−4
−2
0
2
−3
−2
−1
0
1
2
3

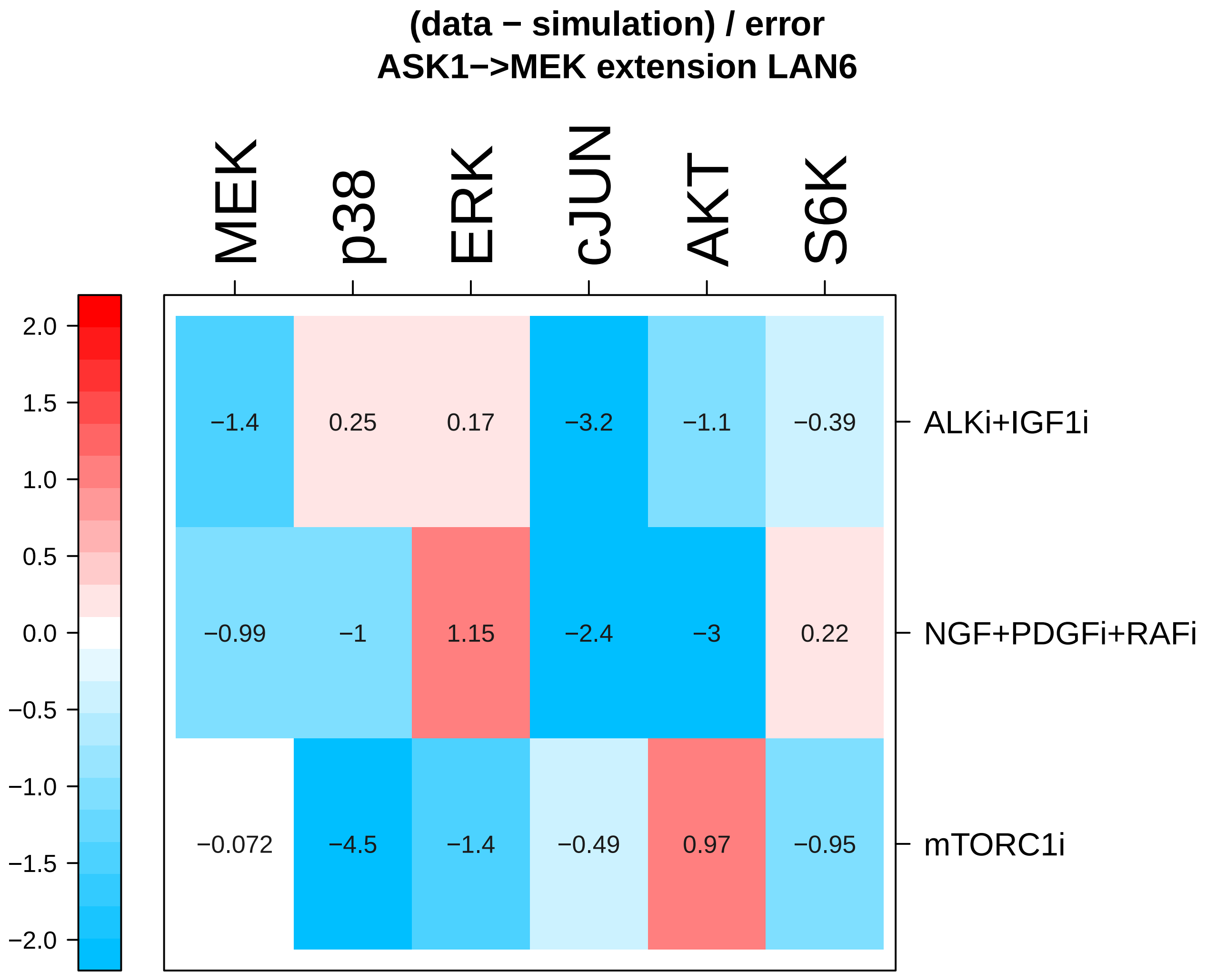

ASK1−>MEK extension LAN6

4.4 Final model reduction

Receptor links to PI3K would be removed but are kept for inter-models comparison. In LAN6 it also seems that cJUN is only regulated by ERK as the TAK1->JNK->cJUN links are not essential.

```
##
## [1] "Best fit: 211.36 , Score= 0.75"
```

4.5 Final network with fixed parameters LAN6

To remove non identifiability linked to not measuring all inhibited nodes, we fix the inhibitors to concensus values and refit with this constraint.

```
## [1] "LAN6 fixed_ask1_mek, residual= 238"
```

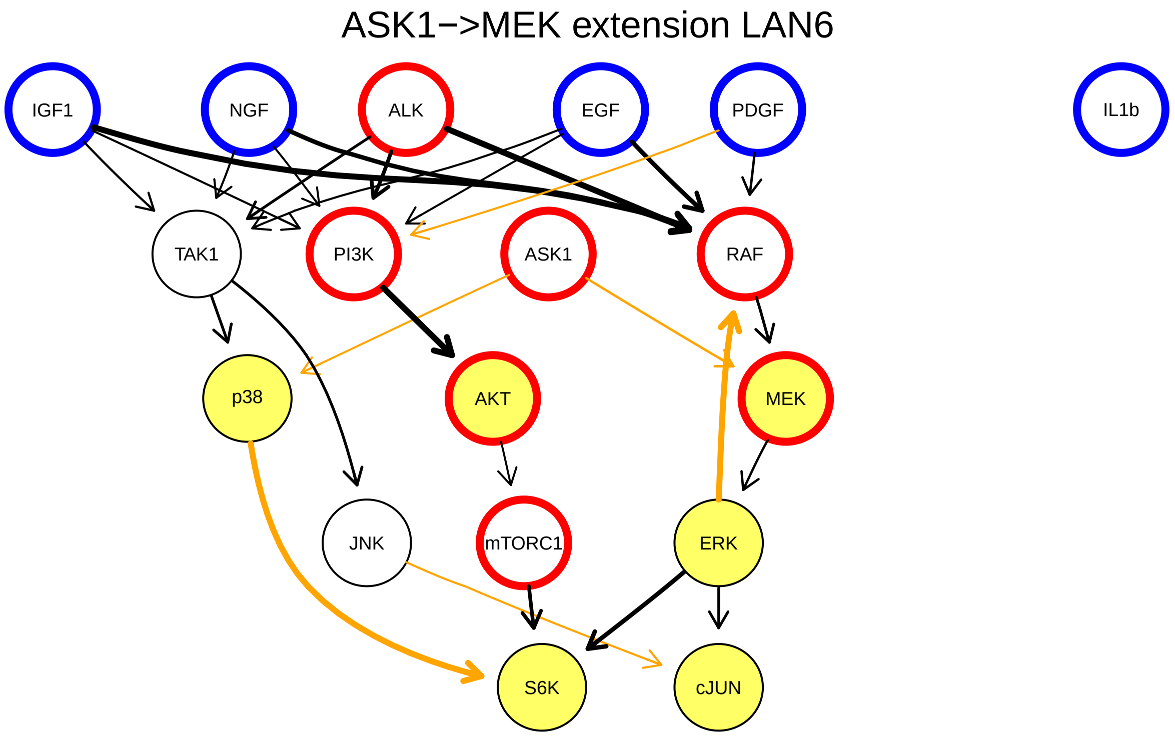

5 IMR32 fitting summary

5.1 Initial topology IMR32

```
## [1] "IMR32 initial, residual= 407"
```

adj_idx	from	to	value	residual	df	Res_delta	df_delta	pval	adj_pval
54	ASK1	TRKA	-29.2992225	320.1884	35	86.31202	1	0e+00	0.00e+00
344	p38	ALK	-32.5523396	333.7740	35	72.72641	1	0e+00	0.00e+00
47	ASK1	MEK	-1.2286086	345.2386	35	61.26179	1	0e+00	0.00e+00
51	ASK1	RAF	-4.9879855	345.2609	35	61.23954	1	0e+00	0.00e+00
40	ASK1	ALK	15.5973073	362.6112	35	43.88924	1	0e+00	0.00e+00
50	ASK1	PI3K	-8.9803434	369.1632	35	37.33715	1	0e+00	2.00e-07
39	ASK1	AKT	-0.3138869	369.2324	35	37.26800	1	0e+00	2.00e-07
354	p38	PI3K	0.1564248	369.7502	35	36.75019	1	0e+00	3.00e-07
343	p38	AKT	-7.6870145	370.6456	35	35.85480	1	0e+00	5.00e-07
2	AKT	ALK	2.0054020	380.8082	35	25.69221	1	4e-07	9.57e-05

The model fits the data for most data, with the notable exception of MEK, ERK, AKT and S6K upon ASK1i treatment. The first extensions suggested ASK1/p38 -> RAF/MEK/PI3K/AKT adress exactly this problem. We chose ASK1->MEK as the best, and more biologically coherent than ASK1->RAF.

5.2 ASK1->MEK effect IMR32

```
## [1] "IMR32 ASK1->MEK, residual= 342"
```

adj_idx	from	to	value	residual	df	Res_delta	df_delta	pval	adj_pval
155	MEK	ASK1	28.521343	249.2412	36	92.71592	1	0e+00	0.00e+00

adj_idx	from	to	value	residual	df	Res_delta	df_delta	pval	adj_pval
79	ERK	ASK1	25.733796	250.4091	36	91.54804	1	0e+00	0.00e+00
269	TAK1	ASK1	-9.176290	275.4155	36	66.54168	1	0e+00	0.00e+00
136	JNK	ASK1	-10.418740	275.4485	36	66.50860	1	0e+00	0.00e+00
307	cJUN	ASK1	19.430463	276.1824	36	65.77474	1	0e+00	0.00e+00
345	p38	ASK1	-100.186168	276.6643	36	65.29284	1	0e+00	0.00e+00
231	RAF	ASK1	1.805141	292.1762	36	49.78099	1	0e+00	0.00e+00
174	NGF	ASK1	78.238801	314.3312	36	27.62597	1	1e-07	3.50e-05
288	TRKA	ASK1	52.046722	314.3312	36	27.62597	1	1e-07	3.50e-05
343	p38	AKT	-9.403166	315.4632	36	26.49393	1	3e-07	6.29e-05

The ASK1i effect on MEK and ERK is stronger with IGF1 and NGF treatments. This would suggest that those ligands, but not EGF and PDGF, activate ASK1. Moreover, ASK1i alone as no strong effect on MEK and ERK (or any other readouts). To reflect this, we added the links IGF1->ASK1 and NGF->ASK1, and removed ASK1 basal activity.

5.3 GS4997 (ASK1i) effect IMR32

```
## [1] "IMR32 GS4997 mechanism, residual= 236"
```

adj_idx	from	to	value	residual	df	Res_delta	df_delta	pval	adj_pval
37	ASK1	AKT	-1.6863093	194.4161	38	41.40809	1	0	0.0e+00
318	p38	PI3K	0.1471028	194.4198	38	41.40438	1	0	0.0e+00
127	JNK	AKT	-0.0082072	194.4399	38	41.38430	1	0	0.0e+00
138	JNK	PI3K	-0.0002225	194.6148	38	41.20938	1	0	0.0e+00
48	ASK1	PI3K	-0.0459175	194.6213	38	41.20286	1	0	0.0e+00
307	p38	AKT	5.7455526	194.8453	38	40.97882	1	0	0.0e+00
308	p38	ALK	-108.7448123	198.5777	38	37.24644	1	0	2.0e-07
282	cJUN	PI3K	-0.1626477	203.2735	38	32.55063	1	0	2.4e-06
271	cJUN	AKT	-5.8302831	203.3721	38	32.45210	1	0	2.5e-06
217	RAF	AKT	-1.7931491	205.8452	38	29.97895	1	0	8.9e-06

This model explains everything except the hyper-activation of AKT by NGF+ASK1i. The proposed extensions ASK1/p38/JNK -> PI3K/AKT would solve this but also overfit the data, with an unexpectedly low residual. Accordingly, we stopped the extensions here.

5.4 Final model reduction

Mostly receptor links would be removed in IMR32 but are kept for inter-models comparison. The ASK1->p38 link is redondant with TAK1->p38 because ASK1 has no basal activity in IMR32.

```
## Performing model reduction...

## Warning in selectMinimalModel(original_model, accuracy = accuracy): Link IL1b->ASK1 belongs to a non-

## Remove link 18: IL1b->ASK1

## New residual =  235.824166020366 , Delta residual =  2.8e-14 ,  p-value =  1.3e-07

## --- Other best links ---

## Warning in selectMinimalModel(original_model, accuracy = accuracy): Link IL1b->PI3K belongs to a non-

##     Could remove IL1b->PI3K
```

```
##     New residual =  235.824166020366 , Delta residual =  2.8e-14 ,  p-value =  1.3e-07
## Warning in selectMinimalModel(original_model, accuracy = accuracy): Link IL1b->TAK1 belongs to a non-
##     Could remove IL1b->TAK1
##     New residual =  235.824166020366 , Delta residual =  2.8e-14 ,  p-value =  1.3e-07
## ----------------------------------------------------------------------------------------------------
## Warning in selectMinimalModel(original_model, accuracy = accuracy): Link IL1b->TAK1 belongs to a non-
## Warning in selectMinimalModel(original_model, accuracy = accuracy): Negative delta residual : -2.842
## Remove link 19: IL1b->TAK1
## New residual =  235.824166020366 , Delta residual =  2.8e-14 ,  p-value =  1.3e-07
## --- Other best links ---
## Warning in selectMinimalModel(original_model, accuracy = accuracy): Link IL1b->PI3K belongs to a non-
##     Could remove IL1b->PI3K
##     New residual =  235.824166020366 , Delta residual =  0 ,  p-value =  0
## ----------------------------------------------------------------------------------------------------
## Warning in selectMinimalModel(original_model, accuracy = accuracy): Link IL1b->PI3K belongs to a non-
## Warning in selectMinimalModel(original_model, accuracy = accuracy): Negative delta residual : -2.842
## Remove link 18: IL1b->PI3K
## New residual =  235.824166020366 , Delta residual =  2.8e-14 ,  p-value =  1.3e-07
## ----------------------------------------------------------------------------------------------------
## Remove link 23: NGF->TAK1
## New residual =  236.062673531463 , Delta residual =  0.24 ,  p-value =  0.37
## ----------------------------------------------------------------------------------------------------
## Remove link 17: IGF1->TAK1
## New residual =  237.184330460005 , Delta residual =  1.12 ,  p-value =  0.71
## ----------------------------------------------------------------------------------------------------
## Remove link 7: ASK1->p38
## New residual =  239.646192151506 , Delta residual =  2.46 ,  p-value =  0.88
## ----------------------------------------------------------------------------------------------------
## Remove link 2: ALK->PI3K
## New residual =  242.597431655414 , Delta residual =  2.95 ,  p-value =  0.91
## ----------------------------------------------------------------------------------------------------
## Remove link 6: EGF->PI3K
## New residual =  245.537380611106 , Delta residual =  2.94 ,  p-value =  0.91
## ----------------------------------------------------------------------------------------------------
## Reduction complete
## [1] "Best fit: 245.54 , Score= 0.66"
```

5.5 Fixed parameters, GS4997 effect IMR32

```
## [1] "IMR32 final, fixed parameters, residual= 340"
```

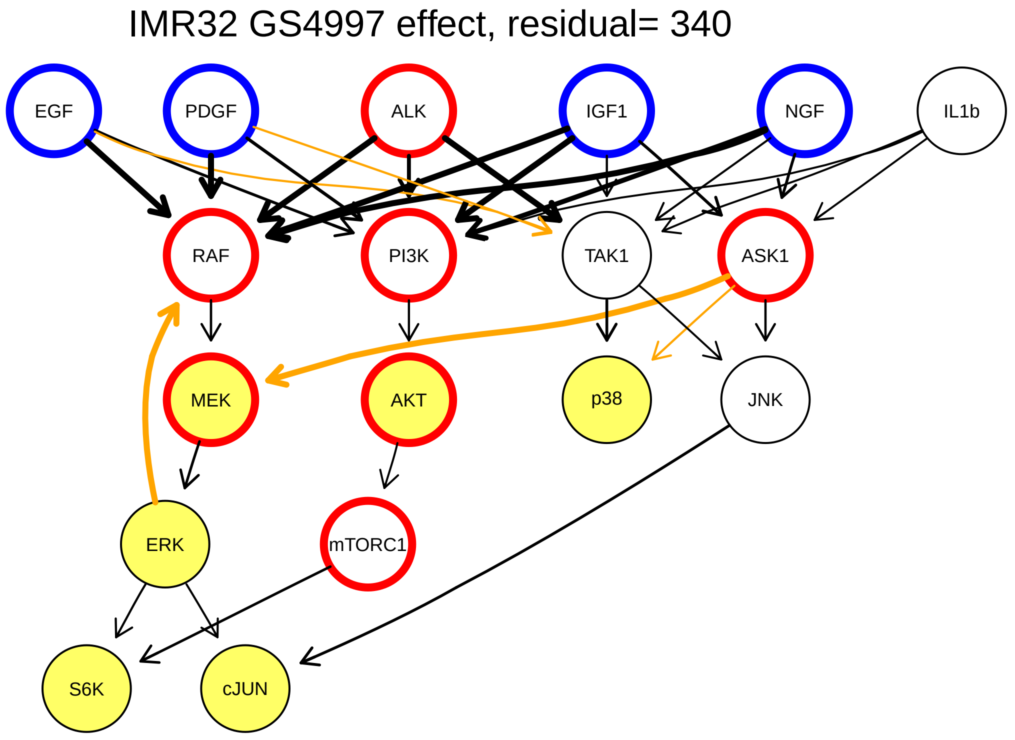

6 KELLY fitting summary

6.1 Initial model KELLY

```
## [1] "KELLY initial (with pdgf basal), residual= 602"
```

adj_idx	from	to	value	residual	df	Res_delta	df_delta	pval	adj_pval
344	p38	ALK	1.801923	524.2083	38	78.08573	1	0	0
360	p38	mTORC1	9.989551	525.4286	38	76.86541	1	0	0
53	ASK1	TAK1	1.949288	526.4027	38	75.89128	1	0	0
46	ASK1	JNK	3.036983	526.8654	38	75.42862	1	0	0
359	p38	cJUN	9.980141	527.0330	38	75.26096	1	0	0
55	ASK1	cJUN	9.953194	527.3230	38	74.97102	1	0	0
350	p38	JNK	5.242374	527.7001	38	74.59386	1	0	0
40	ASK1	ALK	2.324434	529.7311	38	72.56286	1	0	0
357	p38	TAK1	1.000000	531.3149	38	70.97911	1	0	0
51	ASK1	RAF	-10.257071	531.5086	38	70.78535	1	0	0

```
## [1] "KELLY initial, residual= 594"
```

adj_idx	from	to	value	residual	df	Res_delta	df_delta	pval	adj_pval
360	p38	mTORC1	10.673901	497.4614	38	104.83259	1	0	0
344	p38	ALK	1.778739	524.2973	38	77.99671	1	0	0
46	ASK1	JNK	3.233537	525.4429	38	76.85107	1	0	0
55	ASK1	cJUN	2.585803	526.4969	38	75.79710	1	0	0

adj_idx	from	to	value	residual	df	Res_delta	df_delta	pval	adj_pval
53	ASK1	TAK1	9.989546	526.6057	38	75.68832	1	0	0
359	p38	cJUN	9.981842	527.4446	38	74.84935	1	0	0
350	p38	JNK	5.242256	527.6088	38	74.68519	1	0	0
357	p38	TAK1	1.000000	530.4984	38	71.79563	1	0	0
40	ASK1	ALK	2.176704	531.2525	38	71.04147	1	0	0
351	p38	MEK	-99.976378	535.5638	38	66.73023	1	0	0

This initial topology barely explains the behaviour of KELLY and yields a very bad fit. The most striking misfits are the effect of ASK1 on cJUN and S6K. The best extension would be p38->mTORC1, however we chose p38->S6K that makes more sense biologically.

6.2 p38->S6K model KELLY

```
## [1] "KELLY p38->S6K, residual= 484"
```

adj_idx	from	to	value	residual	df	Res_delta	df_delta	pval	adj_pval
55	ASK1	cJUN	6722.186965	381.4888	39	102.33023	1	0	0
359	p38	cJUN	4.462047	381.4888	39	102.33022	1	0	0
350	p38	JNK	4.462048	381.4888	39	102.33022	1	0	0
46	ASK1	JNK	6697.834093	381.4888	39	102.33021	1	0	0
53	ASK1	TAK1	-5423.474295	381.4888	39	102.33019	1	0	0
357	p38	TAK1	1.000000	381.4888	39	102.33018	1	0	0
339	mTORC1	TRKA	1.000000	384.4591	39	99.35993	1	0	0
358	p38	TRKA	1.000000	389.1156	39	94.70336	1	0	0
54	ASK1	TRKA	1636.378801	390.2967	39	93.52230	1	0	0
263	S6K	TRKA	-1.291896	398.8810	39	84.93803	1	0	0

This extension greatly improves the fit. As expected ASK1/p38->JNK/cJUN seems necessary to explain the effect of ASK1 on cJUN.

6.3 p38->cJUN model KELLY

```
## [1] "KELLY p38->cJUN, residual= 411"
```

adj_idx	from	to	value	residual	df	Res_delta	df_delta	pval	adj_pval
259	S6K	PI3K	-1.5814900	331.2781	40	80.14441	1	0	0
248	S6K	AKT	-0.2372540	332.0401	40	79.38243	1	0	0
263	S6K	TRKA	-0.0033137	333.1443	40	78.27822	1	0	0
339	mTORC1	TRKA	1.0000000	347.4404	40	63.98217	1	0	0
324	mTORC1	AKT	-0.0515973	348.2387	40	63.18388	1	0	0
335	mTORC1	PI3K	-0.2939987	348.2413	40	63.18126	1	0	0
24	ALK	ERK	-0.0447852	358.3615	40	53.06102	1	0	0
62	EGF	ERK	0.2768803	359.9729	40	51.44966	1	0	0
271	TAK1	ERK	1.0615881	362.1996	40	49.22295	1	0	0
138	JNK	ERK	1.0662084	362.2010	40	49.22156	1	0	0

S6K->PI3K/AKT would explain AKT phosphorylation upon mTORC1 inhibition as well as from ASK1 inhibition (via p38->S6K) and has literature support via RICTOR.

S6K->TRKA reflects a possible receptor feedback. The link S6K->IRS1 (an IGFR adaptor) is documented in the literature so we tested it (as S6K->IGF1 to take path identifiability into account)

6.4 S6K->IGF1 model KELLY

```
## [1] "KELLY S6K->AKT, residual= 327"
```

adj_idx	from	to	value	residual	df	Res_delta	df_delta	pval	adj_pval
24	ALK	ERK	-0.2566298	301.0397	41	25.86612	1	0.0000004	0.0000801
62	EGF	ERK	0.2784629	303.7743	41	23.13158	1	0.0000015	0.0003313
138	JNK	ERK	1.2068602	306.0927	41	20.81315	1	0.0000051	0.0011089
271	TAK1	ERK	1.2066071	306.0930	41	20.81287	1	0.0000051	0.0011090
345	p38	ASK1	-3312.1010589	311.1293	41	15.77654	1	0.0000713	0.0156105
342	mTORC1	p38	0.0000170	311.9016	41	15.00425	1	0.0001073	0.0234920
326	mTORC1	ASK1	0.0000010	311.9453	41	14.96051	1	0.0001098	0.0240429
266	S6K	p38	0.0389549	312.1051	41	14.80074	1	0.0001195	0.0261681
250	S6K	ASK1	0.0000043	312.1064	41	14.79941	1	0.0001196	0.0261865
136	JNK	ASK1	-0.0095466	312.1079	41	14.79792	1	0.0001197	0.0262072

6.5 S6K->AKT model KELLY

```
## [1] "KELLY S6K->AKT, residual= 332"
```

adj_idx	from	to	value	residual	df	Res_delta	df_delta	pval	adj_pval
24	ALK	ERK	-0.2566298	301.0397	41	25.86612	1	0.0000004	0.0000801
62	EGF	ERK	0.2784629	303.7743	41	23.13158	1	0.0000015	0.0003313
138	JNK	ERK	1.2068602	306.0927	41	20.81315	1	0.0000051	0.0011089
271	TAK1	ERK	1.2066071	306.0930	41	20.81287	1	0.0000051	0.0011090
345	p38	ASK1	-3312.1010589	311.1293	41	15.77654	1	0.0000713	0.0156105
342	mTORC1	p38	0.0000170	311.9016	41	15.00425	1	0.0001073	0.0234920
326	mTORC1	ASK1	0.0000010	311.9453	41	14.96051	1	0.0001098	0.0240429
266	S6K	p38	0.0389549	312.1051	41	14.80074	1	0.0001195	0.0261681
250	S6K	ASK1	0.0000043	312.1064	41	14.79941	1	0.0001196	0.0261865
136	JNK	ASK1	-0.0095466	312.1079	41	14.79792	1	0.0001197	0.0262072

6.6 Final model reduction

```
## Performing model reduction...

## Remove link 15: IL1b->ASK1

## New residual =  332.127005307579 , Delta residual =  0.087 ,  p-value =  0.23

## ------------------------------------------------------------------------------------

## Remove link 15: IL1b->PI3K

## New residual =  332.214820496539 , Delta residual =  0.088 ,  p-value =  0.23

## ------------------------------------------------------------------------------------

## Remove link 13: IGF1->RAF

## New residual =  332.36405170677 , Delta residual =  0.15 ,  p-value =  0.3

## ------------------------------------------------------------------------------------
```

```
## Remove link 14: IL1b->TAK1
## New residual =  333.073023771963 , Delta residual =  0.71 ,  p-value =  0.6
## -----------------------------------------------------------------------------------------
## Remove link 26: TRKA->PI3K
## New residual =  333.814226232691 , Delta residual =  0.74 ,  p-value =  0.61
## -----------------------------------------------------------------------------------------
## Remove link 17: NGF->TRKA
## New residual =  335.727726311905 , Delta residual =  1.91 ,  p-value =  0.83
## --- Other best links ---
## Warning in selectMinimalModel(original_model, accuracy = accuracy): Link TRKA->RAF belongs to a non-
##     Could remove TRKA->RAF
##     New residual =  335.727727688541 , Delta residual =  1.91 ,  p-value =  0.83
## -----------------------------------------------------------------------------------------
## Warning in selectMinimalModel(original_model, accuracy = accuracy): Link TRKA->RAF belongs to a non-
## Remove link 25: TRKA->RAF
## New residual =  335.727726311906 , Delta residual =  1.1e-12 ,  p-value =  8.3e-07
## -----------------------------------------------------------------------------------------
## Remove link 13: IGF1->TAK1
## New residual =  337.891589914914 , Delta residual =  2.16 ,  p-value =  0.86
## -----------------------------------------------------------------------------------------
## Remove link 4: ALK->TAK1
## New residual =  340.217846631212 , Delta residual =  2.33 ,  p-value =  0.87
## -----------------------------------------------------------------------------------------
## Remove link 14: NGF->TAK1
## New residual =  342.634889610926 , Delta residual =  2.42 ,  p-value =  0.88
## -----------------------------------------------------------------------------------------
## Reduction complete
## [1] "Best fit: 342.63 , Score= 0.65"
```

6.7 Fixed inhibitor model KELLY (S6K->IGF1)

The error is dominated by the unexplained but probably false signals of very high ERK with EGF+AKTi (but with no other perturbations with AKTi or EGF) and MEK downregulation with EGF alone (while no such effect is seen with EGF+PI3Ki, EGF+ASK1i or EGF+mTORC1i). Removing those 2 datapoints yield a residual compatible with the number of data points.

```
## [1] "KELLY S6K->AKT without bad points, residual= 288"
```

Log−fold change Experimental data
default

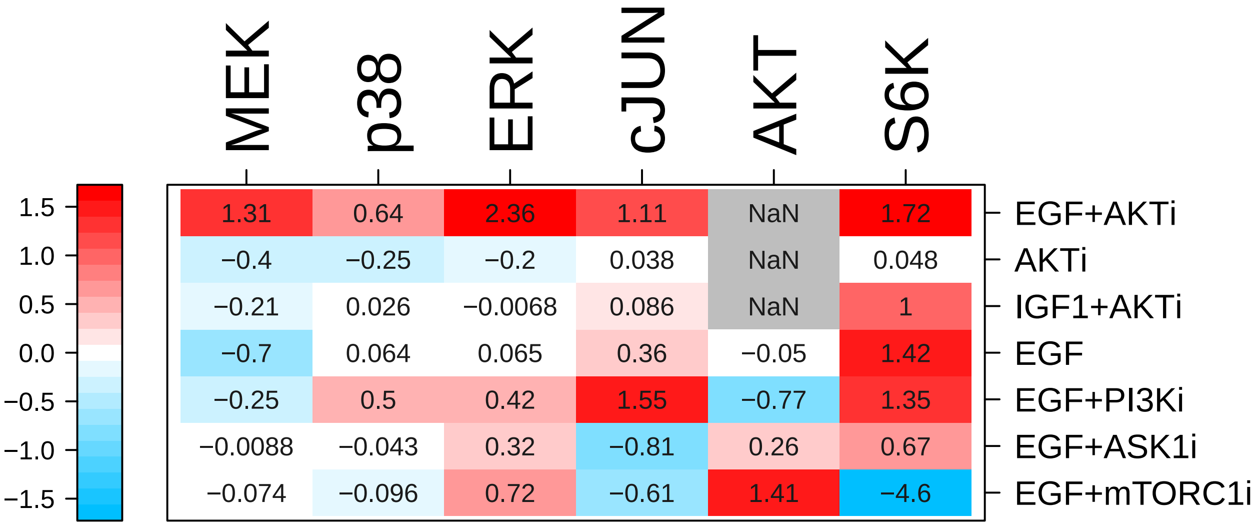

default
KELLY S6K−>AKT, residual= 372

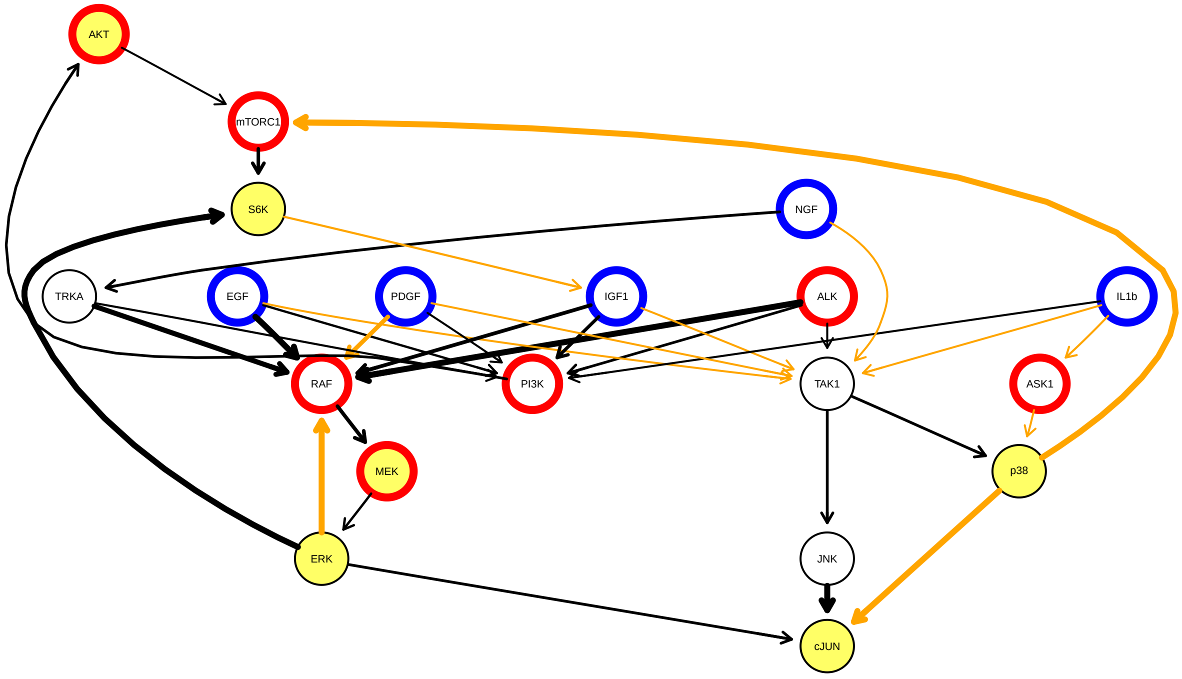

7 Models comparison

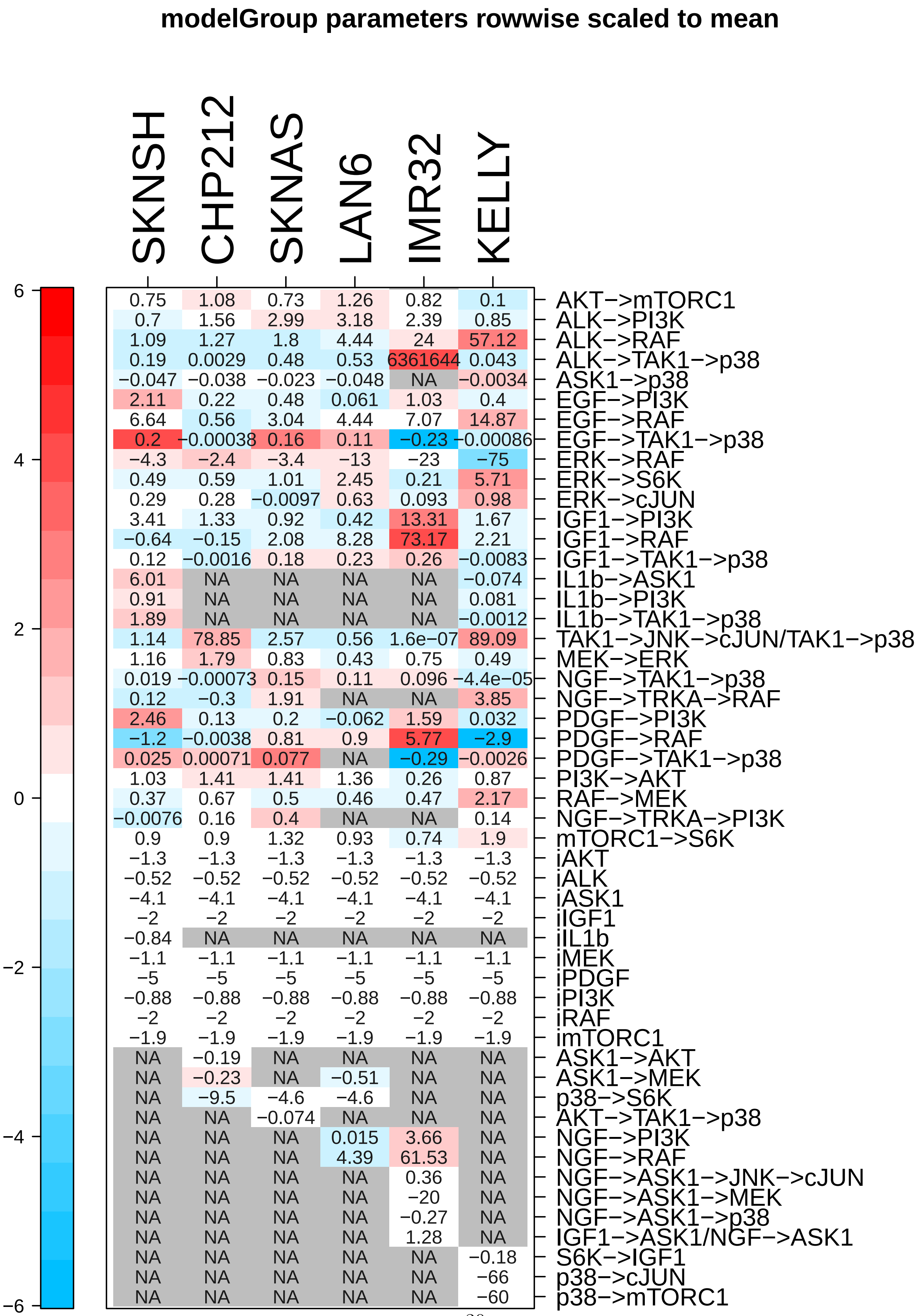

Supplement: S3 File — (ZIP) [file pcbi.1009515.s024.zip › Perturbations_data/summary/all_summaries.pdf]
