# Supplementary material for: Neuroblastoma signalling models unveil combination therapies targeting feedback-mediated resistance
Source: PLoS Comput Biol. 2021 Nov 4;17(11):e1009515. doi: 10.1371/journal.pcbi.1009515 (PMC8604339; doi:10.1371/journal.pcbi.1009515)
Supplement: Attachment — Submitted filename: Reviewer_response-2.pdf [file pcbi.1009515.s025.pdf]

We thank both reviewers and the editor for their helpful and supportive comments. Please see below for a point-to-point response, in which the comments are in black, and our responses are highlighted in red.

Reviewer #1: In this paper the authors investigate mechanisms of drug resistance in a panel of high risk neuroblastoma cells focusing on the ERK pathway that is frequently altered in high risk neuroblastoma. They use a combination of experimental perturbation data and an elegant mathematical method to reconstruct signaling networks in order to decipher molecular mechanisms of drug resistance. Surprisingly, rather that linked to genetic mutations drug resistance seems mediated by differential network topologies in particular different feedback loops. While not entirely new, this finding is an important contribution to the growing case that network adaptations can be responsible for drug resistance in cancer and that we need to look beyond genetic mutations. Overall, the paper is well conceived, and the combination of experimental work and mathematical modelling is convincing. I am happy to recommend publication, but have a few comments that should be addressed.

We thank the reviewer for this supportive comment.

Major comments

As negative feedback loops are ultimately made responsible for the differences in drug resistance, it would strengthen these conclusions by showing more evidence of their direct involvement. For instance, one would expect phosphorylation sites to change that mediate these feedback in a manner that correlates with the feedback strengths. These data seem to be there, but summarizing them in a figure would make the message clearer. Also, mutants that break the feedback could be used to show that is really the feedback that mediates drug resistance.

The reviewer is correct that a key difference between the models is their relative feedback strength. The main effect that the model explains by the differential engagement of feedback is the following effect: In the perturbation data of Fig 4 and Fig. 2, it is apparent that IGF treatment alone does not activate AKT strongly in KELLY and N206, whereas it activates AKT in IMR32 (and other cell lines). Post MEKi treatment, AKT is activated in all cell lines where the model includes a feedback (KELLY and N206). We inspected the phosphor-proteome data if we could directly identify feedback sites, however, these were missing from the phosphoproteome. However, we found that the synergistically regulated sites in N206 contain many known or putative phospho-sites of the AKT/mTOR axis, suggesting that MEK inhibition synergises with IGFR inhibition in regulating AKT signalling. If this can be ultimately attributed to feedback, or if the feedback fitted by the model summarizes a more complex mechanism of synergy remains therefore open. We agree that only additional experiments with mutant feedback sites could resolve this issue, which is however beyond the scope of this work. We however think that the semi-mechanistic modelling conducted in our paper is still a valid tool to quantify signalling differences.

In response to this comment, we changed multiple parts of the text to reflect this (page 11, lines 246-248, page 14 lines 391-395). We also highlight the known and putative AKT/mTOR/P70S6K target sites in Figure 5C.

Fig. 6B. The authors claim a “strong synergistic effect of the combination of MEK and IGFR inhibitions on growth 291 in N206 but little in KELLY or IMR32.” This is not apparent from the figure. A formal measure of synergism should be used, such as the Chou-Talalay method or Loewe isoboles.

We fully agree and provide a quantification of synergy now in Figure 6 using the commonly used Bliss score. This clearly shows synergy in KELLY for MEKi+RAFi and synergy in N206 for MEKi+IGFRi. Because we can reach arbitrary negative growth (depending on the seeding density) and have not reached a plateau in growth inhibition for all drugs, the Loewe isobole model is for our data not robust and subject to many hypotheses. Nevertheless, when we applied Loewe isoboles, we find that there are overlapping regions of synergy for both scores, which would qualify as strong synergy (see Tang et al. 2015 (https://www.frontiersin.org/articles/10.3389/fphar.2015.00181/full), particularly for low, therapeutically relevant, concentration of AZD6244. For reference, we provide the scores by the Loewe method in the supplement.

In response to this comment, we added Figure 6C, supplementary Figure S16 and adopted the text (pages 11-12, lines 292-284)

Fig. 6D. These data are not very convincing. The triple drug combination does not seem to be more potent than the dual combinations. This figure need error bars and a proper statistical comparison.

We agree that the represented data did not clearly show synergy, particularly as we did not have all dual combinations. We therefore repeated the experiment with additional measurements in biological triplicates and lower concentration of each inhibitor to better match physiologically relevant conditions. From this data it is clear that the triple combination is not more potent than all single combinations in all cell lines except for KELLY.

As this was only a minor point of the manuscript, we removed this conclusion from the text, removed the subfigure from the main figure 6, and added the new data to the supplement (supplementary figure 17) and findings to main text (page 13, lines 314-317).

Minor comments

The MYN amplification status of the cell lines should be reported.

We have added this information to figure 1A.

"A strong RAF-mediated feedback is a known resistance mechanism against MEK inhibitors (Friday et al, 2008; Fritsche-Guenther et al, 2011), …" should also cite Sturm et al. 2010.

We apologize for not citing Sturm at this position, that is now corrected.

"… a known nexus between AKT and MAPK signalling (?)." Please supply the citation.

Thank you for spotting this mistake, this is now corrected.

MYCN phosphorylation on S62 has been linked to enhanced degradation using phosphosite mutants (https://doi.org/10.1016/j.ccr.2014.07.015). However, here the authors associate the de-phosphorylation of this residue with lower MYCN protein levels. Would be worth discussing or mitigating this claim as it is only based on a correlation.

Thank you for pointing this out, our point was that lower protein levels would result also in lower phospho-levels, but depending on the kinetics this may not be the case.

We have thus changed this section accordingly (page 11, lines 270-272)

Reviewer #2: The authors have used quantitative perturbation to study the resistance mechanisms in well characterized Neuroblastoma cell lines. They focused on a handful of ligands and signaling genes to build a prior knowledge network. They tested different drugs and combination to fit the network and identify cell-line specific mechanisms like negative feedbacks. They could even pinpoint a vertical inhibition to counter the feedback-mediated resistance in studied cell lines. All together this manuscript represents an important step in the evaluation of resistance mechanisms in cancer cell lines and might pave the way for sensitizing resistant tumors and improve patient outcome.

We thank the reviewer for these supportive comments.

Nevertheless, the author should address the following comments before publication:

- To my understanding the authors have only try to extend the model, by adding edges between genes. Did they also try to remove edges? Although they are well characterized in the literature, I was wondering if they were all essential on if some could be discarded in one or many cell lines. Perhaps it would improve the fit.

This is an important point. The workflow was as follows: We extended the network until no significant link could be added then performed successive rounds of reduction to identify the non-significant links.Most removed links relate to receptor connections. Only three connections not related to receptors were removed during this procedure, each for one cell line only. To facilitate model comparison, these links were ultimately retained in the model, as otherwise the model parameters would not be comparable.

We updated the methods section (page 16 line 481-586) to better describe the model reduction, and updated the supplement (Supplementary model data) to include the output of the reduction.

- IC50 estimation in this study was done by monitoring cell growth with the Incucyte platform. As this instrument generates masks to calculate the confluency of cells, this can be biased by differences in cell morphology or patterns of cell growth. Did the authors consider whether the morphology of the cells was similar before and after drug treatment? It could be of profit to include, as supplementary data, some raw images of the cells at the beginning and end of the recording (72 h), at least for MEKi.

We agree that this is an important point. Inspection of the images shows that cell size did not substantially change. Thus we believe that confluency is a good proxy for all drugs except for rapamycin where we see significant morphological changes (the cells flatten).

As suggested, we provide now raw images of a MEKi-sensitive and MEKi-resistant cell line before and 72 h after MEK inhibition (or DMSO solvent control) in Supplementary Figures 18 and 19, respectively.

- Supplementary Figure2: Which method was used to calculate the correlation? As mutation data are qualitative (or at least Boolean), Pearson's correlation is maybe not the best method to apply here. The authors should add more details about this and perhaps try other methods, like non parametric tests, to infer association between IC50 and mutations.

We fully agree that a statistical test between two groups would be more appropriate for the mutation data and adapted the statistics accordingly.

We updated Supplementary figure 2.

- Line 94-96: "To get insights into the underlying mechanisms of resistance to the MEK inhibitor AZD6244, we selected 6 neuroblastoma cells lines that represented the spectrum of sensitivity to MEK inhibition (sensitive: CHP212, LAN6; resistant: SKNAS, SKNSH, KELLY and IMR32)", the period at the end of the sentence is missing.

Thank you, we corrected this.

- Page 9, line249: What does the (?) stand for?

It should be a reference. We apologize for this mistake caused by an error in our bibtex-file.

- In figure 5 part C legend: there is a typo on phosphopeptides

Thank you, we corrected this mistake.

- Supplementary Figure 3: Is it really adjusted p>0.95? In the text it is written 0.93 and 0.94 for 1000 most variable genes and GO signal transduction genes respectively. Please double check.

Thank you, we corrected this.

- Supplementary Figure 4,5,9: color code is missing

Thank you, we corrected this.

-Supplementary Figure 17: Gene-sets should be re-ordered according to the GeneRatio to better see the ranking.

Thank you, we changed this accordingly (now Supplementary Figure 15)
