# Supplementary material for: Neuroblastoma signalling models unveil combination therapies targeting feedback-mediated resistance
Source: PLoS Comput Biol. 2021 Nov 4;17(11):e1009515. doi: 10.1371/journal.pcbi.1009515 (PMC8604339; doi:10.1371/journal.pcbi.1009515)

Synergies TerminateNB drugs

Mathurin Dorel

2020-07-24

```
# Summary of drug synergies for IMR32, KELLY and N206 for the TNB paper
```

Drug synergies

AZD6244 (MEK) and AEW451 (IGFR)

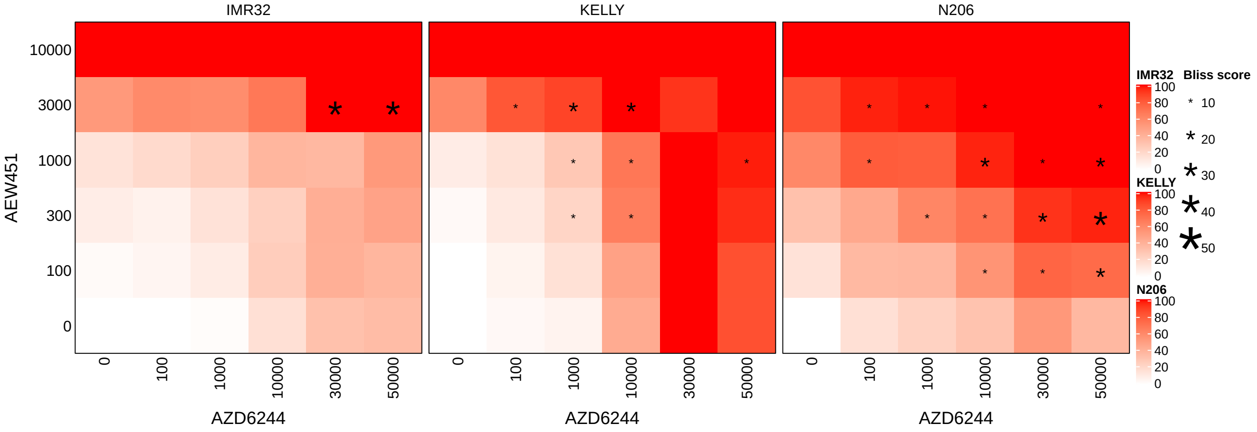

ERK/RAF inhibitors plex

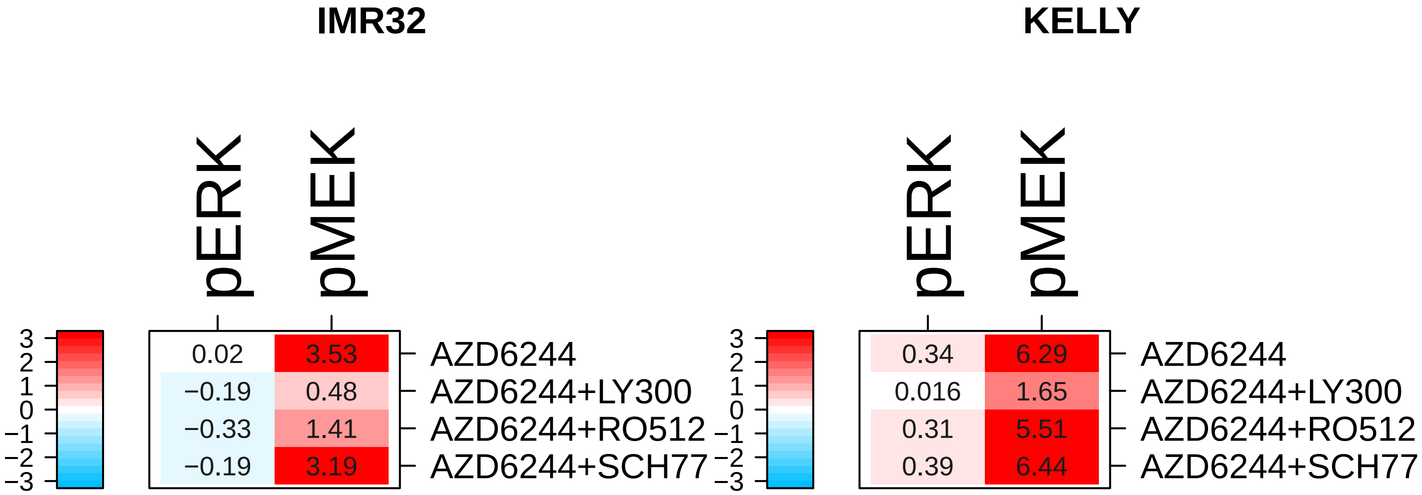

AZD6244 (MEK) and LY300912 (RAF)

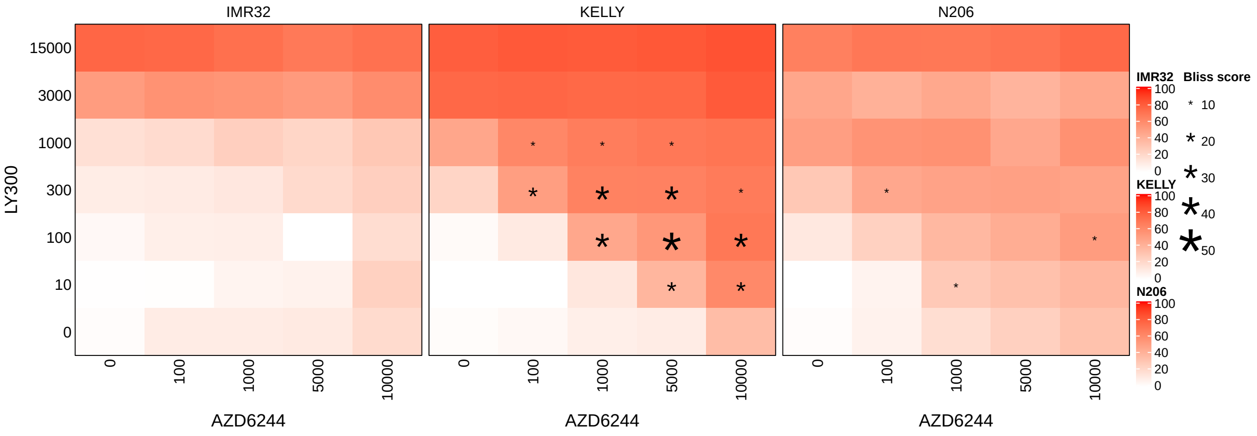

AZD6244 (MEK), AEW451 (IGFR) and LY3009120 (RAF)

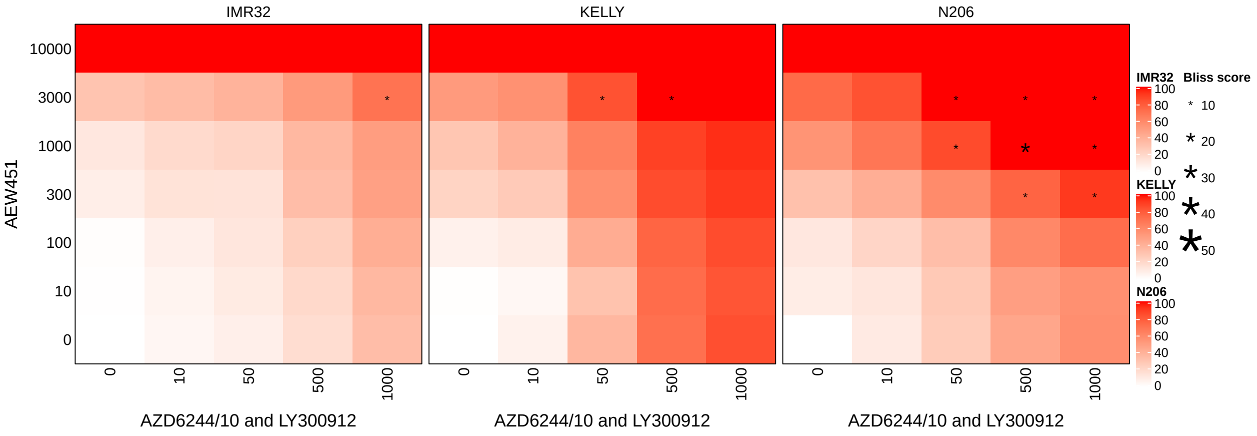

AZD6244 (MEK) and RO5126766 (RAF)

RO5126766 does not seem to inhibit RAF enough to have a distinguishable phenotypic effect.

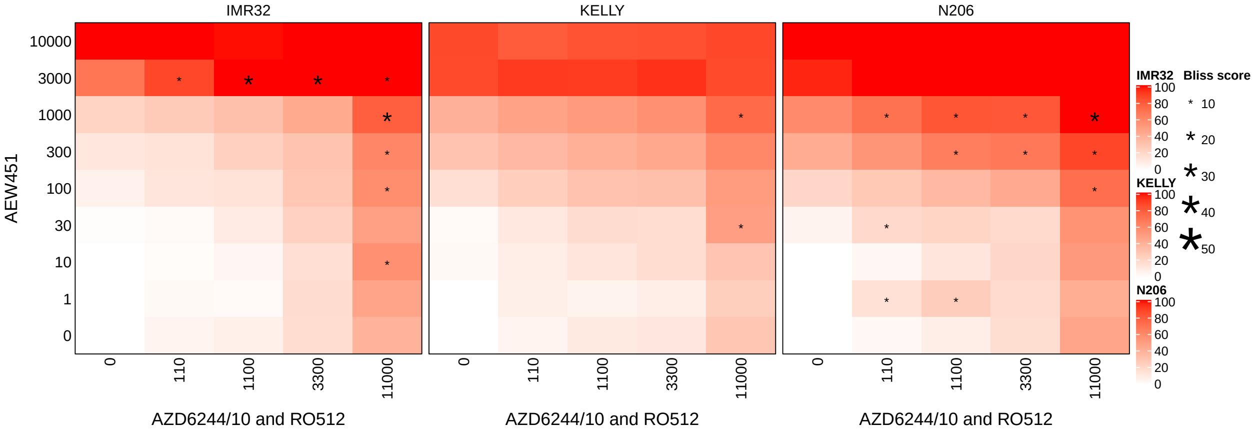

AZD6244 (MEK), RO5126766 (RAF) and AEW451 (IGFR)

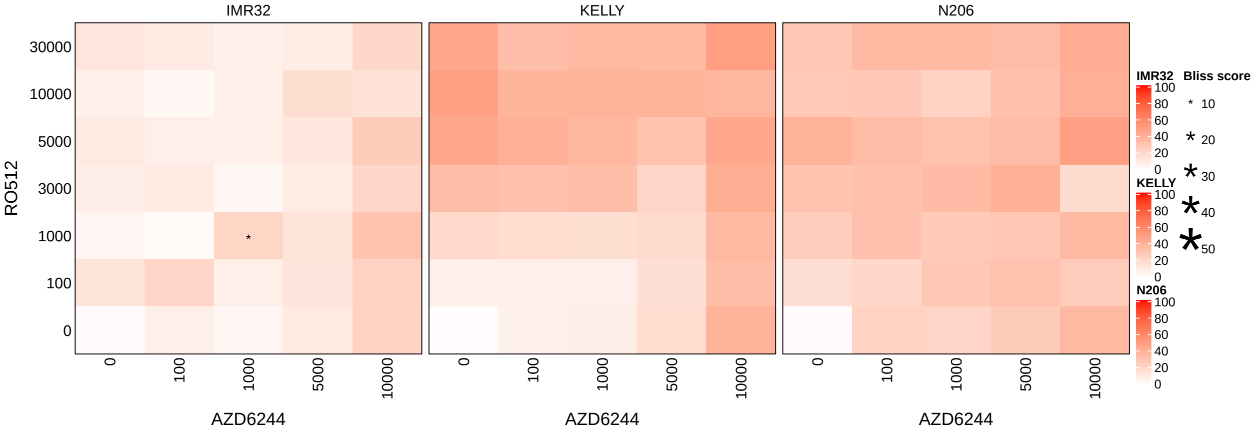

AZD6244 (MEK) and SCH772984 (ERK)

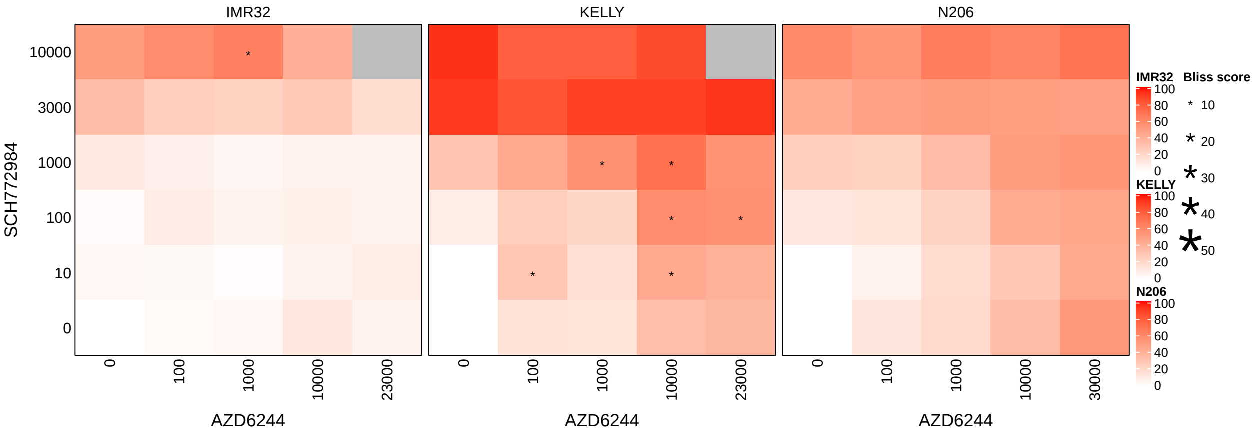

Supplement: S2 File — (ZIP) [file pcbi.1009515.s023.zip › drug_sensitivity/summary_synergies/summary_synergies.pdf]
